# Insights into the architecture of earthworm metallothionein genes, powered by long-read genomics and transcriptomics

**DOI:** 10.1093/nargab/lqaf195

**Published:** 2026-01-08

**Authors:** Maxim A Karpov, Stephen Short, Peter Kille, Carl Hobbs, Amaia Green Etxabe, David J Spurgeon, Stephen R Stürzenbaum

**Affiliations:** Department of Analytical, Environmental and Forensic Sciences, Faculty of Life Sciences and Medicine, King's College London, London, SE1 9NH, United Kingdom; UK Centre for Ecology & Hydrology, Maclean Building, Wallingford, OX10 8BB, United Kingdom; School of Biosciences, Cardiff University, Cardiff, CF10 3AX, United Kingdom; Wolfson Sensory Pain and Regeneration Centre, King’s College London, London, SE1 1YR, United Kingdom; UK Centre for Ecology & Hydrology, Maclean Building, Wallingford, OX10 8BB, United Kingdom; UK Centre for Ecology & Hydrology, Maclean Building, Wallingford, OX10 8BB, United Kingdom; Department of Analytical, Environmental and Forensic Sciences, Faculty of Life Sciences and Medicine, King's College London, London, SE1 9NH, United Kingdom

## Abstract

The metal-handling metallothionein gene family is subjected to a strong selection pressure in earthworms inhabiting metalliferous soils. The resultant single nucleotide polymorphisms and structural variants within metallothionein’s characteristically short exons challenge standard gene prediction and genome assembly tools and complicate phylogenetic analysis of earthworm metallothioneins (wMTs). Here, the genomic origin of wMTs was defined by sequencing four wMT-containing Bacterial Artificial Chromosomes (BACs). This yielded sequence contigs of lengths from 97 to 123 kb and enabled further genetic analyses, supporting the creation of a BLAST-based software for visualizing long-read RNA-seq alignments—lrRNAseq TAST (Total Alignment Search Tool). Phylogenetic analysis of wMT proteins from long-read RNA-seq data grouped wMTs into three distinct clades. Tissue-specific lrRNAseq and metallomic mapping, conducted in context of lead toxicity, revealed enrichment of wMT-1 and wMT-2 in tissues of heavy metal detoxification, and colocalization of wMT with lead in the chloragog. The generation of rare wMT isoforms was attributed to errors in expression, but the erroneous transcripts may possess a degree of order and purpose. A conserved region in the 3′ UTR of wMT transcripts was found completely distinct between wMT homologues, allowing classification without inferring phylogenetic trees.

## Introduction

Earthworms are ubiquitous soil inhabiting invertebrates that live in intimate association with the soil and that exhibit a unique combination of biological characteristics such as hermaphroditism, high regenerative capacity, and resistance to heavy metals in the soil. (Earth)worm metallothionein (wMT) has been a subject of study for decades and is arguably the most well-studied earthworm protein due to its relevance in, notably, cadmium detoxification [1–4]. For example, metallothionein has been documented to be overexpressed several hundred-fold during cadmium toxicosis [2]. The cadmium accumulates/bioconcentrates in sulphur-containing chloragosomes, tethered to sulphur, possibly as part of thiol-containing residues of metallothioneins. The mode of sequestration is metal-specific, and lead (Pb) has a different destiny, being complexed in distinct chloragosomes that are enriched with phosphorus and calcium [5–8]. Despite studies of the protein, uncertainties still exist. For example, the structures of the wMT genes as well as their syntenic relationship with other neighbouring genes remains unclear. Moreover, the role of expression in the toxicosis of other pollutant metals, such as Pb, remains poorly studied compared to, for example, Cd.

To date, a wealth of earthworm bioinformatic data has been forthcoming, including a 17 000-sequence Expressed Sequence Tag dataset [9], the sequencing of multiple wMT loci (totalling 25 kb of sequencing data) [2], the short-read sequencing of tissue-specific transcriptomics in worms challenged with arsenic or silver nanoparticle exposures [10–12], single-cell RNA sequencing in context of regeneration [13], the release of reference quality genome of *Lumbricus rubellus* [14], and restriction-site-associated DNA sequencing of earthworm populations inhabiting metalliferous soils [15]. For short-read datasets, the challenges have consistently manifested as low contiguity due to the presence of repetitive regions. With long-read datasets, the challenges present themselves in high error rate and dependence on the accuracy of the assembly algorithm/pipeline, which often involves approximations/abstractions for the sake of performance, or false sense of contiguity (e.g. filtration or artificial segregation of repetitive regions). This makes Bacterial Artificial Chromosome (BAC) libraries relevant to this day as a source of very long, highly accurate (~0.01% Sanger sequencing error rate) genomic contigs that provide a tool to maintain the authenticity of biological phenomena to support gene prediction.

Four wMT-loci-containing BAC clones were isolated from the two earthworm species, two from *L. rubellus* and two from *Eisenia fetida*, and each was sequenced, annotated, and analysed in terms of sequence repetitiveness and gene structure. Previously investigated *E. fetida* BAC clones of valosine-containing protein (VCP) were also included into the analysis (Supplementary Fig. S1) [16, 17]. In addition, this work incorporated data from the first ever tissue-specific long-read RNA sequencing (lrRNAseq) of 10 earthworm tissues, including tissues thought to be actively involved in metal detoxification (gut, calciferous gland, and nephridia), and other tissues (pharynx, body wall, crop, gizzard, seminal vesicles, clitellum, and nerve cord), from an earthworm native to an abandoned lead mine site.

LrRNAseq is a recently developed sequencing approach enabled by the advances and commercialization of long-read technologies over the past decade. One highly demanded application is the study of gene isoform expression. Following the introduction of lrRNAseq, software applications have been built to handle read mapping, clustering, filtering, polishing, the identification and quantification of transcripts, differential isoform expression, differential splicing analysis, gene-level visualization, and other aims [18].

The obtained long-read dataset was used in the development of a software, lrRNAseq TAST, for visualization of lrRNAseq BLAST alignments to moderately long genomic regions of DNA such as BACs. When aligning full-length transcripts to a genome, using established long-read mapping/alignment software, short and variable exons may struggle to align in cases of genetic heterogeneity. BLAST is a simple to use, well-known, and highly adopted algorithm that can be configured for high sensitivity for detection of short and variable exons. Here, wMT was used as an example gene for demonstration of software capabilities. The gene is known to have short exons (e.g. exon 1 being only ~22 bp in length and exon 2 ~26 bp), posing a challenge for predictive software applications. The discovered wMT transcripts were analysed quantitatively and phylogenetically, focusing on tissue distribution, gene diversity, and expression. Finally, in an attempt to establish a qualitative link between metallothionein and Pb detoxification, metallothionein and Pb were co-visualized in a cross-section of an earthworm gut after acute exposure to Pb.

## Materials and methods

### Earthworm sourcing and maintenance

The *L. rubellus* and *E. fetida* earthworms used for BAC sequencing were sourced from the University of Ohio. The *L. rubellus* earthworm designated for the Pb exposure and metal imaging experiments was sourced from a nature reserve near Glastonbury, UK (OS Grid reference ST4350041640). The *L. rubellus* earthworm used in lrRNAseq was sourced from "Plant" site, at Cwmystwyth, an abandoned Pb mine site (OS Grid reference SN8090074900). The earthworms were stored in perforated plastic boxes containing moistened native soil, inside an incubator (Sanyo MIR-154) at 15°C. The soil was moistened with water once a week during the duration of the earthworm storage. Adult clitellate earthworms were used for all experiments. Prior to processing, the worms were washed with distilled water and placed on a moist filter paper (Whatman) in a petri dish for 48 h to void the gut contents. The filter paper was changed once a day to prevent reingestion of expelled soil.

### BAC library construction

The BAC library was constructed as described before [17, 19]. In brief, earthworm tissues were isolated from several mature *E. fetida* and *L. rubellus*, and trypsinized. The dissociated cells were embedded into agarose, digested with proteinase K, partially digested with HindIII, and the resultant fragments ligated into the pCC1BAC vector (Epicenture, WI, USA), resulting in the average insert size of genomic fragments to be ~100 kb. More than 50 000 clones were individually picked into 530# 96-well plates.

### Polymerase chain reaction screening for *wMT* fragments

The BAC library was divided into 44 super-pools, each containing 12# 96-well plates. This first polymerase chain reaction screen identified the positive super-pool, which was split into respective 12 plate-pools (second screen). The *wMT* containing clones (Lr13H12, Lr6F14, Ef472C1, Ef318A10) were identified from the positive plate in a third screen comprising a column-pool (A–H) and row-pool (1–12).

### Genomic sequencing and analysis

The identified BAC clones were sequenced end-to-end by standard primer walking using the DYE terminator sequencing Kit (GE-Healthcare, NJ, USA) and the ABI PRIZM 310 genetic analyzer (Applied Biosystems, Tokyo, Japan).

### Surgical procedures

The earthworms were anaesthetized by submersion in cold (4°C) carbonated water for ~8 min. Earthworms were dissected under a microscope using fine tweezers, dissecting scissors and 11A Swan Morton scalpel. Following an incision on the dorsal side, all lobes of the seminal vesicle were removed. A section of gut tissue spanning six segments at the midline between the clitellum and the posterior end was dissected out, and any gut contents discarded. The crop, gizzard, and pharynx were then collected and any gut contents discarded. This was followed by removal of all components of the calciferous gland and the entire ventral nerve cord from under the pharynx to the posterior segments of the worm. A section of body wall post clitellum representing six segments was dissected out, followed by the clitellum tissue itself. Finally, nephridia were removed from at least 20 segments throughout the body. To ensure the highest quality RNA, the tissue samples were homogenized in trizol as further tissues were being dissected, and the resulting homogenate was stored at −80°C.

### Long-read RNA sequencing

An *L. rubellus* ‘clade B’ earthworm was sampled from Cwmystwyth, Wales [20, 21]. The worm was dissected live, and tissues immediately added to 500 µl of Tri-reagent (Sigma–Aldrich) and homogenized using a Qiagen Tissue lyser: 2× 1 min 30 s runs at 20 Hz with a 5-mm stainless steel ball. Total RNA was isolated using Tri-reagent (Direct-Zol, Zymo Research, USA) according to the manufacturer’s instructions, which includes a DNAse I treatment. RNA was quantified (Qiagen QIAxpert) and integrity was checked by means of agarose gel electrophoresis. Purified RNA was then stored at −80°C.

The samples were processed at the UCL long-read Sequencing Facility (Institute of Neurology). The SMRTbell Express Template Prep Kit 3.0 was used to prepare the HiFi SMRTbell Library of the RNA with the PacBio barcoding primers. The library sequencing was performed on a PacBio Sequel IIe system (Pacific Biosciences) on a single 8M cell.

Initially, the reads were filtered by quality score (Q30). A custom barcode deconvolution protocol was employed using BLAST to search for barcode sequences within 150 bp of transcript ends. Transcripts without barcodes at both ends were discarded. Transcripts were filtered for the presence of a poly-A tail, and oriented forward based on poly-A location.

### Long-read RNAseq alignment visualization

As input, the software (lrRNAseq TAST—https://github.com/Maxim-Karpov/lrRNAseq-TAST) takes BLAST alignment of lrRNAseq query to genomic target sequence, the FASTA file containing sequences of aligned lrRNAseq reads, and a FASTA file with the genomic sequence. The sensitivity of the BLAST alignment is configurable via word_size (e.g. 4) and e_value (e.g. 1 × 10^–2^). Long introns may be filtered by length, and transcripts may be further filtered by minimal percentage of length covered by the genomic sequence. For each aligned transcript, the local BLAST alignments were linked by a line. If more than a single transcript is plotted in the same row, they are discriminated by different colours. Each aligned region for a particular transcript is drawn as a red rectangle behind the line or yellow, if overlapping the largest open reading frame (ORF). The ORF is decided based on the standard codon table with ATG start codon, and TAG, TAA, TGA stop codons. If the alignment region overlaps any shorter ORF region, it is drawn as a red rectangle with a yellow border. A number is drawn on top of each aligned region based on its order in the transcript, starting from zero. If the neighbouring numbered alignments are sequential or the same (e.g. 1, 2, 3, 3), they are considered to be collinear and the region is highlighted in pink. BAC clones were manually annotated based on visualization of transcript alignments. The transcripts with plausible exon–intron structure were BLASTed against the NCBI nt database for annotation. The software was implemented in Jupyter Notebook, Python, using Matplotlib.

### BAC visualization and repeat analysis

BACs were self-aligned using BLAST with word_size parameter set to 4 and e_value set to 1 × 10^–3^. For each BAC coordinate on the *x*-axis, the alignment depth was calculated and plotted on the *y*-axis. The sequences of repeat monomers were manually extracted from the repeat clusters found in the wMT genes of Lr13H12 and Ef472C1. The repeat monomer sequences were extracted manually by separating the BAC sequence at the troughs of alignment depth at the tandem repeat regions. Decamers were counted in each monomer sequence. The dimensionality of *k*-mer count data was reduced via the principal component analysis (PCA). The monomer sequences were clustered via the DBSCAN algorithm (scikit-learn) using the first two principal components for model fitting. The repeats were visualized based on monomer clusters generated.

### Pairwise BLAST alignments

The region encapsulating MAD-1 and wMT genes was extracted by cutting at coordinates (0–85 000), (7000–68 000), (43 000–98 500) in BACs Lr13H12, Ef472C1, and Ef318A10, respectively. MT sequences were extracted from BACs by cutting at ~2 kb behind the start and ~3 kb from the end of wMT coding region. The sequences were aligned pairwise with BLASTn, word_size = 4, e-value = 1e−2. The overlapping BLAST alignments were merged and plotted on the lane of their respective sequence, in red. The MT exons were drawn in yellow. Lines were drawn between the lanes, connecting the (unmerged) BLAST alignments, with thickness and opaqueness of the line representing the normalized alignment bitscore, and colour of the line representing the directionality of the alignment (red = forward, blue = reverse). For each coordinate, highest percentage identity of collective BLAST alignments was plotted above and below the alignment lanes, in black. The shorter sequence was given borders at its ends. The total percentage identity (*T*) between the sequences was calculated using formula: *T* = *I*/*L*, where: *I* is the sum of the highest identities at each coordinate of both sequences, and *L* is the sum of sequence lengths.

### Phylogenetic analysis

Transcript codings for wMTs were retrieved via BLASTing known wMT exon sequences. Longest ORFs were translated to obtain wMT protein sequences. In certain transcripts, the longest ORF did not code for a canonical MT; here, the true wMT ORF was found based on the high number of cysteine residues. Sequences with <10 cysteines were removed. NCBI wMT sequences [2] were included and aligned using ClustalW (gap opening penalty = 10, gap extension penalty = 0.1) [22]. Redundant protein sequences were removed. Multiple sequence alignment (MSA) and maximum-likelihood phylogenetic tree with adaptive bootstrap were constructed in MEGA12 using default parameters [23–26]. AlphaFold2 was utilized as part of CollabFold [27, 28].

### Earthworm exposures

Air-dried commercial soil (Yorkshire Worms) of high organic content and relatively low density was thoroughly mixed with a lead nitrate (Fisher Scientific) solution up to the desired soil Pb concentration (2500 μg Pb/g soil) [21]. Ten adult earthworms were transferred into 20 cm^3^ perforated plastic box filled with prepared soil and exposed in an incubator for 2 weeks. The healthiest looking earthworm, which retained its mobility after the exposure, was selected for tissue sectioning.

### Tissue sectioning

Adult earthworms were sectioned as previously described [29]. Briefly, the decapitated guts were fixed in 4% buffered formalin (pH 6.9) (Merck) for 48 h. The samples were washed in distilled water and gradually dehydrated in ethanol (70% for 1 h, 90% for 2 h, 100% for 2 h) (Merck). The ethanol was removed by washing in 100% xylene. Tissue was placed in molten paraffin wax for 12 h and the sample was processed in embedding station (Leica EG 1150H). Seven-micrometre-thick sections of the earthworm tissue were cut on a microtome (Reichert-Jung, Mod. 1140/Autocut). The sections were mounted on Superfrost Plus slides (Thermo Scientific) and dried.

### Antibody staining

The sections were deparaffinated by washing in xylene for 5 min twice and rehydrated by washing in dilutions of ethanol (100%, 90%, 80%, 70%, 50%) for 3 min each at room temperature. The slides were immersed in 3% hydrogen peroxide for 10 min. A pressure cooker with 1 mM, pH 6 citric acid was heated in a microwave until boiling. The slides were transferred into the citric acid solution and the pressure cooker heated for further 6 min, then let cool at room temperature until unpressurized. The citric acid was washed-off and the slides were dried. The 2% bovine serum albumin blocking solution was added to the tissue sections and left for 7 min. The primary polyclonal rabbit anti-wMT antibody [1] was added onto the sections and allowed to incubate for 20 h at room temperature. The slides were rinsed in 0.05M Tris-buffered saline (TBS) and then washed for 10 min in 0.05M TBS on an orbital shaker. The slides were then incubated in the goat anti-rabbit (polyclonal) antibody coated with 175Lu (Standard Biotools, previously known as Fluidigm, product ID: 3175002G) secondary antibody for 20 h at room temperature. The slides were rinsed in 0.05M TBS and washed in 0.05M TBS for 10 min on an orbital shaker. These slides dried at room temperature for 1 h and then stored for further procedures.

### Metal staining

Iridium intercalator (Standard Biotools, 201192A) 0.125 µM stock was diluted 1:1000 with 0.05M TBS. The diluted Iridium was pipetted to cover the tissue section encircled by a ReadyProbesTM hydrophobic barrier Pap Pen (Thermo Fisher) (to reduce the volume of reagent required) and incubated at room temperature for 30 min. Iridium was then thoroughly flushed with 0.05M TBS using a pastette. The slide was washed in TBS in a glass trough on an orbital shaker for 5 min and then flushed thoroughly with Milli-Q water. Finally, the slide was washed in Milli-Q water in a glass trough on the orbital mixer for 5 min and dried at room temperature prior to storage.

### Imaging mass cytometry

The tissue sections were imaged on the Hyperion imaging system (Standard Biotools). Regions of interest on the tissue section grey scans were selected using Fiji image processing software for imaging. Metallomic maps were processed and analysed on the CyTOF software (Standard Biotools).

## Results

**Figure 1.**
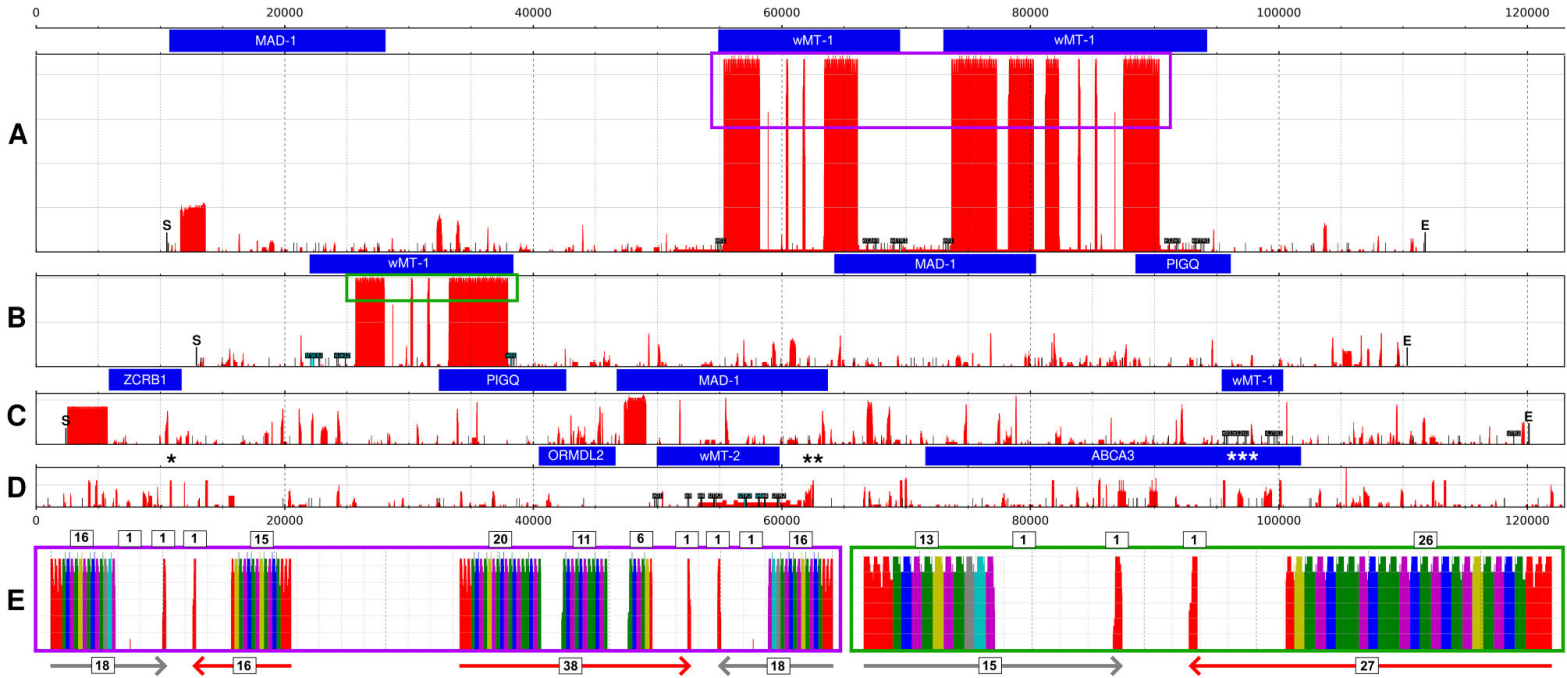
BAC repeat structure and annotation. Visualization of the clones Lr13H12, Ef472C1, Ef318A10, and Lr6F14 (**A–D**, respectively). Repetitive structures were visualized in red. The depth of the self-alignment is qualitatively displayed on the *y*-axis. Gene annotations were displayed above the corresponding repetitiveness plot in blue rectangles. All sequences were centred on the plot, with the starts of the sequences indicated by the letter ‘S’ and the ends by the letter ‘E’. The position of the redacted repeat structures in Lr6F14 clone were indicated by star symbols [* = (TA)n, ** = (TAG)n, *** = poly(G)]. Segments of the wMT gene were aligned, and the alignment regions were indicated with T-shaped flags, with grey and cyan flags representing forward or reverse alignment orientation. The wMT genes were labelled by their respective homologue number of the wMT protein family, as per later discoveries of this investigation. The major tandem repeat structures in clones Lr13H12 and Ef472C1 were boxed in purple and green, to be visualized in detail (**E**). The repeat monomers were clustered using *k*-mer counts. Monomers were assigned a colour based on cluster. The grey and red arrows represent the directionality and type of tandem repeat clusters. The numbers in boxes represent the number of repeat monomers in the cluster (bottom) or in the sequential chunks (top). e1−4: exon 1–4; UTR1-2: untranslated region 1–2.

Four BAC clones containing wMT loci were isolated (Supplementary Sequences)—two, Lr13H12, Lr6F14 (Fig. 1A and D), from *L. rubellus* and two, Ef472C1, Ef318A10 (Fig. 1B and C), from *E. fetida*. Clone lengths were 102 281, 97 604, 117 984, and 122 990 bp, respectively. The BAC sequences were self-aligned using BLAST. The depth of the self-alignment was calculated for each coordinate of the BAC. This technique revealed the positions of tandem repeat structures (e.g. the repeat structures in clones Lr13H12 and Ef472C1, within the first intron of the wMT genes). Simple repeat structures in clone Lr6F14, namely the ~6-kb-long (TA)n at coordinate 10 900, the ~2 kb (TAG)n at coordinate 64 300, and the ~0.5 kb poly-G at coordinate 96 700, were manually trimmed to simplify the representation. The previously published clones Ef20E08 and Ef91F03 of the VCPs loci were also reannotated (Supplementary Fig. S1) [17]. The clones Lr13H12, Ef472C1, and Ef318A10 represent the same wMT loci, collinear with *mad-1, pigq*, and *zcrb1* genes, with Ef318A10 lacking the large repeat structure and clone Ef472C1 being in reverse orientation. Clone Lr6F14 depicted a different wMT loci with *abca3* and *ormdl2* genes in its neighbouring vicinity. Here, the wMT structure contained two duplications of exon 4, one in reverse direction. wMT is duplicated in clone Lr13H12.

The monomers of tandem repeats in the first intron of the wMT gene of clones Lr13H12 and Ef472C1 were analysed via clustering based on 10-mer composition (Fig. 1E). The PCA dimensionality reduction captured ~50% of the variance in the first two principal components for both clones. DBSCAN clustering revealed seven unique monomer clusters in each clone (Supplementary Fig. S2). The minimal lengths of monomers were 25 and 134, maximal were 215 and 216, and the mean monomer length was 178 and 181 for clones Lr13H12 and Ef472C1, respectively. The average GC content of repeat monomers in both clones was 42%. The repeat clusters were composed of two parts that were reflective of one another (Fig. 1E, grey and red arrows). The common structure of each part was a red triplet at the beginning, followed by green, blue purple or green, yellow, purple sequential triplets. The difference between the reflected parts was at the end, where the grey (arrow) cluster ended with grey, cyan, purple triplet, whilst the red (arrow) cluster ended with a red monomer. Based on this, the tandem wMT duplicates in Lr13H12 have experienced inversion of repeats in intron 1. Beyond this, the major red repeat cluster in the downstream wMT has experienced a significant triplet duplication in its core and is split into three chunks and contains two spacers of equal lengths. The number of repeat monomers in the grey repeat region is the same in tandem duplicates. The Ef472C1 repeats exhibited identical repeat microstructure as in the *L. rubellus* clone and were in the same relative orientation as in the downstream wMT gene of Lr13H12. Sometimes, the green monomers are seen as sequential dimers. Two–three reflective monomers are seen in the middle of each tandem repeat region.

**Figure 2.**
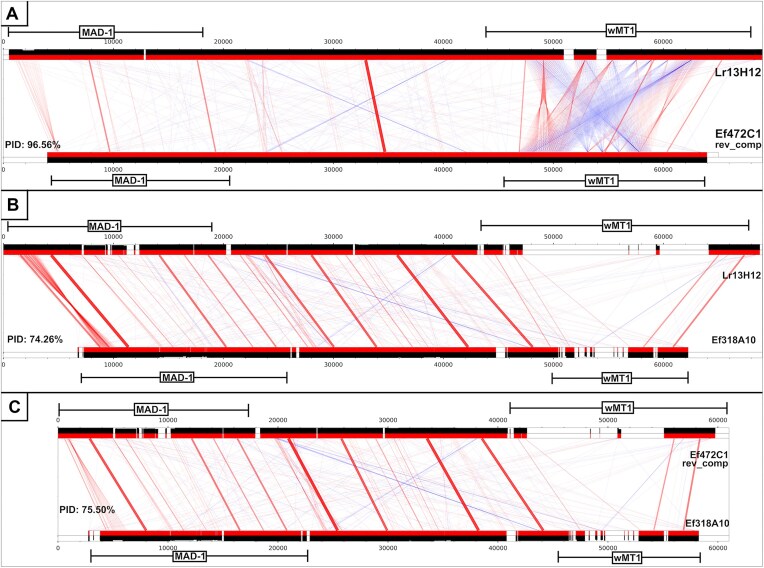
Pairwise comparisons of a locus between and including wMT-1 and MAD-1 genes on BACs. Pairwise BLAST alignments were set up between sections of (**A**) (Lr13H12, Ef472C1), (**B**) (Lr13H12, Ef318A10), (**C**) (Ef472C1, Ef318A10) pairs, to include MAD-1, intergenic region, and wMT into the comparison. Alignment regions were plotted on tracks in red. The paired alignment regions, in forward or reverse alignment orientation, were connected by a red or a blue line, respectively. The line thickness and opacity represent bitscore. The alignment identity is given as a track with an area under curve, in black. Ef472C1 was reverse-complemented. wMT was labelled as the wMT-1 due to the later discoveries of this study. PID: percentage identity.

Lr13H12, Ef472C1, and Ef318A10, being of the same locus, gave the opportunity for further sequence identity comparisons. First, to compare the intergenic regions, the BACs were trimmed between the wMT and the MAD-1 genes. Tandem wMT duplication in Lr13H12, containing large repetitive regions, heavily biases the identity metric towards higher similarity to Ef472C1. So, the upstream wMT was trimmed from the Lr13H12 sequence. BACs were compared using collections of pairwise BLAST alignments (Fig. 2). Identity metrics were collected using a BLAST-based approach in which, the highest alignment identity value at each coordinate was summed and divided by the sum of the sequence lengths. This revealed that the intergenic and intragenic identity was higher between Lr13H12 and Ef472C1, than Ef318A10, as can be seen by the multiple alignment gaps present in the comparisons to Ef318A10. It was noticed that the repetitive region in the MAD-1 gene was present but significantly retracted in Ef472C1.

To gain more insight into the structural differences and the identity of the wMT genes between the BACs, the wMT regions of all the BACs were isolated and compared pairwise (Supplementary Figs S3–S12 and  Supplementary Table S1). Across all comparisons, the exonic regions showed highest levels of conservation and were highly similar (Supplementary Table S2). Exon one was identical between Lr13H12, Ef472C1, and Ef318A10, which had 90% identity with the Lr6F14. Exon two was identical in all BACs. Exons three and four were identical in Lr13H12 and Ef472C1, Ef318A10 had 98% identity to them in both exons, and Lr6F14 was ~81% and 85% identical to the rest of BACs in both exons, respectively. Lowest identity was seen in comparisons of different wMT homologues (i.e. comparisons involving Lr6F14), due to large differences in the intronic and untranslated regions. Excluding the Lr6F14, BACs were highly conserved in the untranslated regions and introns 2 and 3. There were large differences in the first intron due to the presence of large repetitive regions on Lr13H12 and Ef472C1. Lr13H12 contained two unique, equally sized, non-repetitive regions in between the chunks of repeats of the first intron, not found in Ef472C1, the nature of which remains a mystery.

Finally, the wMT gene splice sites were examined. Ten bp regions flanking donor and acceptor splice sites were used for consensus and identity determination (Supplementary Table S3). Splice sites were highly conserved within each intron (Supplementary Fig. S13), forming clear consensus sequences. Regardless of species differences, the Lr6F14 splice sites were most diverged from the rest of the sequences, logically, as they are of a different gene homologue. When splice sites from all introns of all BACs were analysed together, the 5′ splice site, with an average % identity of 96.1%, formed a consensus sequence ‘GTYAGK’ and the 3′ splice site, with identity of 97.9%, formed ‘TTTYAGRNNNNKG’ consensus.

**Figure 3.**
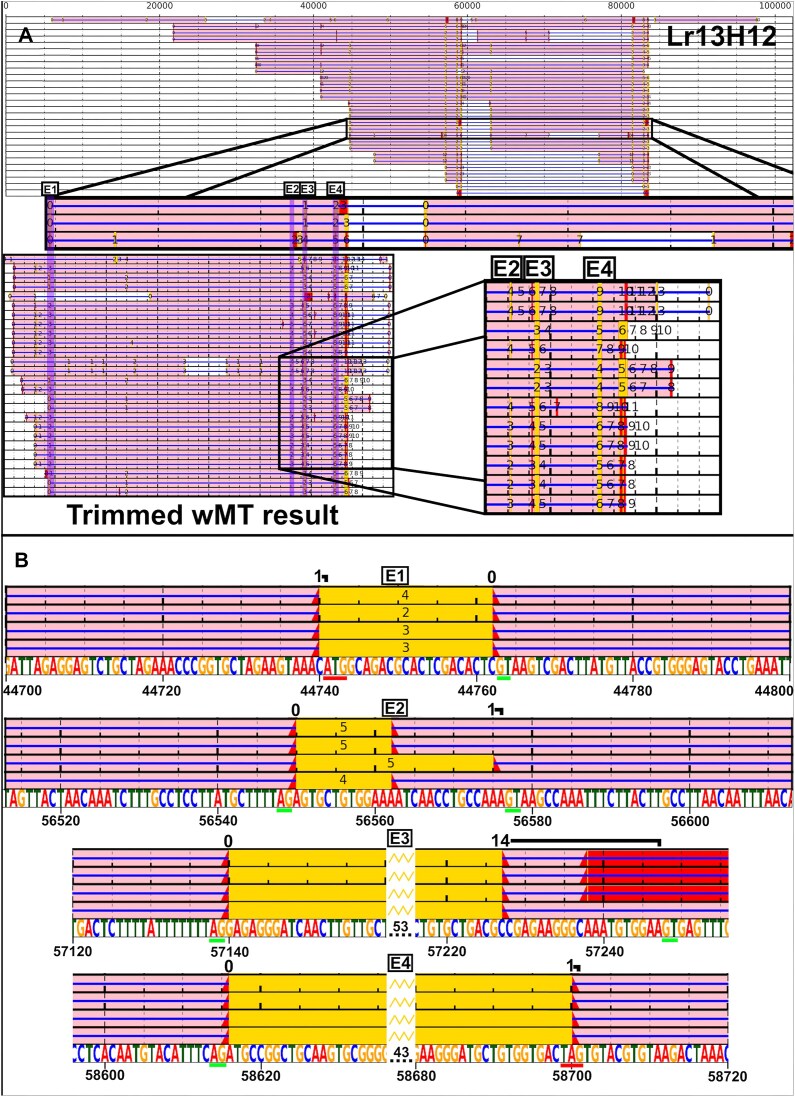
wMT transcript alignments viewed on the Lr13H12 BAC clone. (**A**) wMT transcripts were aligned onto Lr13H12 and visualized with lrRNAseq TAST under default settings (A, top half). Exon 2 was not identified, so lrRNAseq TAST was performed with higher sensitivity on the wMT region (A, bottom half). The locations of the exonic alignments were highlighted in purple columns. Various features were annotated using the lrRNAseq TAST colour coding. (**B**) A close-up of the Lr13H12 wMT gene exonic alignments at the exon–intron junctions. Genomic sequence is shown on a bottom track. The number above the transcript alignments represents the extent of alignment errors. Red alignments: ORFs; yellow alignments: longest ORF; E1/2/3/4: Exon 1/2/3/4; green bar: splice donor and acceptor; red bar: start or stop codon.

The BAC sequences were used for the development and testing of lrRNAseq Total Alignment Search Tool (TAST). Firstly, 10 tissues were isolated from an *L. rubellus* earthworm collected at the Cwmystwyth mine site and sequenced using long-read RNAseq on a PacBio Sequel IIe, generating 3 543 033 full-length RNA sequences, with an N50 of 1877 bp. The sequences underwent barcode deconvolution using a custom BLAST similarity-based protocol, which improved the barcoded sequence yield by ~6% over the standard PacBio lima deconvolution protocol (Table 1). The dataset scored 99.2% in BUSCO completion of the Eukaryota gene set, suggesting it to be highly complete (Supplementary Table S4) [30]. The lrRNAseq dataset was BLAST-queried with known wMT exons to find transcripts encoding wMT. To visualize wMT splicing and test the ability of the software to predict exon positions in a difficult scenario where the gene is tandemly duplicated, contains a large 10–19 kb repetitive region inside its intron, consists of two short and variable exons, and the lrRNAseq dataset is of a mismatched geographical and adaptational origin (albeit of matching species) to the genomic sequence, the wMT transcripts were BLAST aligned to the Lr13H12 clone for visualization using lrRNAseq TAST (Fig. 3). The program running at default settings (word_size = 4, e-value = 1e−2) failed to recognize exon 2. To improve sensitivity, the wMT region was cut out and re-aligned with wMT transcripts (e-value is subject length-dependent), with permissive settings (e-value = 1), which successfully found all four wMT exons, albeit with increased unspecificity. The exonic regions were visualized at the splice junctions to analyse the extent of error in BLAST alignments (Fig. 3B). The other BAC sequences also present some unique difficulties such as reverse directionality (Ef472C1) and tandem exon duplications and inversions (Lr6F14) (Supplementary Figs S14–S19). Species mismatch slightly increased the extent of error in alignments and the presence of a large intronic repetitive region made it more difficult to recognize short exons. Most transcripts aligned without error/minor error of 1 bp. Lr6F14 BAC showed fewest errors. There was a difficulty in correctly predicting the position of the donor site in the third intron of the *E. fetida* BACs. Alternative modes of expression were noticed (e.g. alternative stop codon in Ef318A10).

**Table 1.** Total lrRNAseq and metallothionein (wMT) transcript counts by tissue

Tissue type	Transcripts (count)	Transcripts (%)	wMT transcripts (count)	wMT transcripts (%)	wMT representation (over/under)
**Nephridia**	229 634	6.5	58	11.1	1.7
Gut	296 321	8.4	103	19.8	2.4
Calciferous gland	329 723	9.3	99	19.0	2.0
Pharynx	352 334	9.9	65	12.5	1.3
Body wall	366 811	10.4	50	9.6	0.9
Crop	410 200	11.6	43	8.3	0.7
Gizzard	396 572	11.2	17	3.3	0.3
Nerve cord	274 326	7.7	58	11.1	1.4
Seminal vesicles	439 675	12.4	9	1.7	0.1
Clitellum	447 437	12.6	19	3.6	0.3
Total count	3 543 033	100	521	100	±1

The visualization of all wMT transcripts showed that there were two main transcript types encoding metallothionein—a constitutively expressed, long (~1400 bp) transcript with multiple possible ORF regions, and a short (~700 bp) type predominant in tissues known to play a role in metal detoxification in earthworms (Supplementary Fig. S20). The gut, calciferous gland, and nephridia tissues were found to express half of all wMT transcripts, despite these tissues transcribing only ~25% of all transcripts in total, highlighting their importance in metallothionein-mediated metal homeostasis (Table 1).

**Figure 4.**
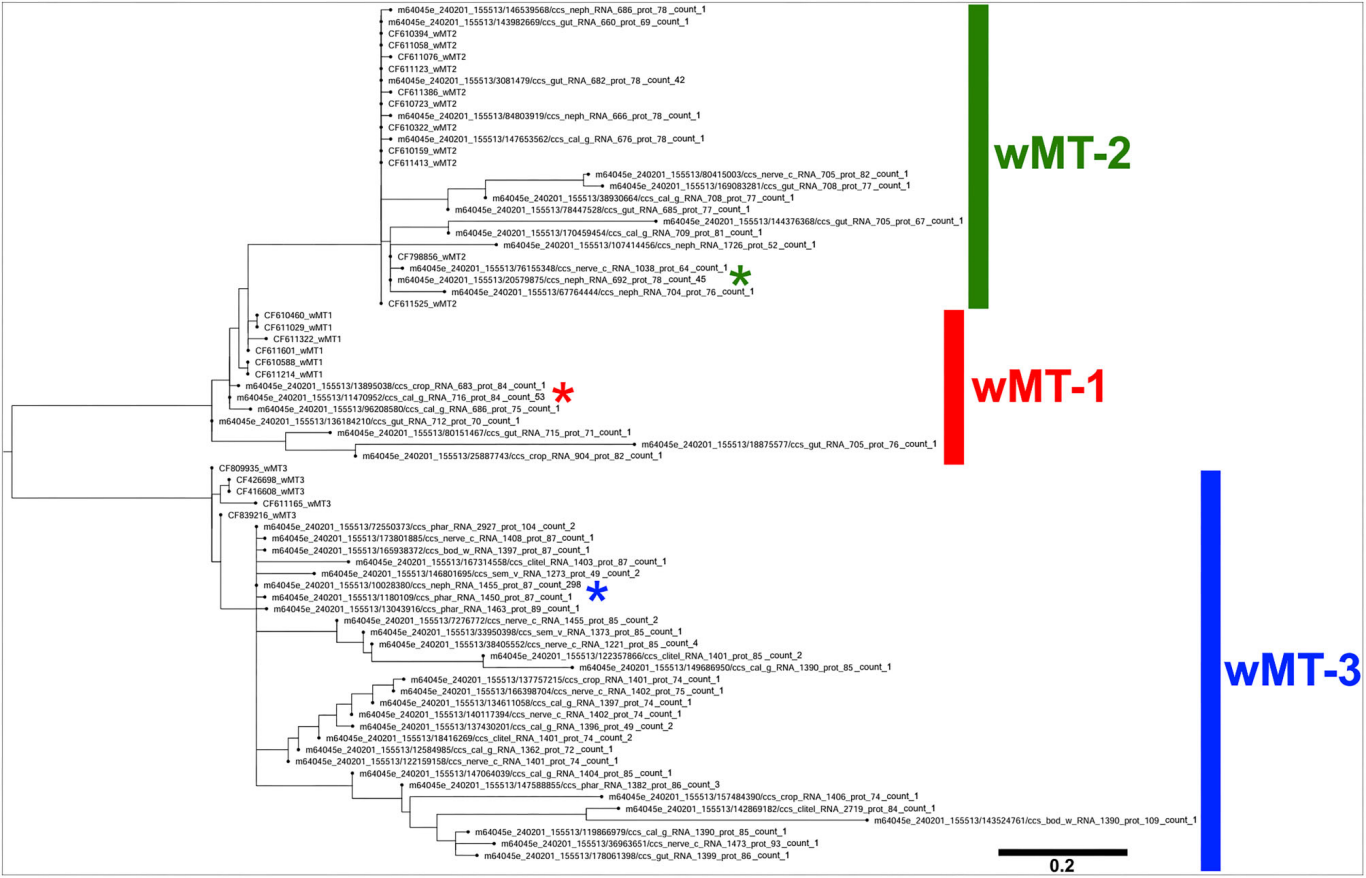
Maximum likelihood phylogenetic tree of wMTs. (**A**) Phylogenetic clustering of previously identified 521 wMT protein sequences (labelled with transcript ID, tissue of origin, RNA length, protein length, and sequence count representing the cluster), and 22 wMT proteins from an expressed sequence tags library for homology reference (labelled with NCBI accession and isoform type) [2]. Clades were labelled with a coloured line on the right. Star represents the cluster from each clade with the highest sequence count.

The ORFs of the data-mined wMT transcripts were translated and aligned using ClustalW, together with previously studied wMT transcripts from NCBI, relating to a previously established wMT classification [2]. Sequence redundancy was removed based on 100% sequence identity; sequences with <10 cysteines in the most cysteine-rich ORF of the transcript were discarded. A phylogenetic tree of this alignment was constructed to study the evolutionary relationship of the sequences (Fig. 4A). Three distinct clades of wMT were proposed—wMT-1, wMT-2, and wMT-3, with 58, 101, and 337 transcripts in each clade, respectively. Transcripts were also counted by tissue, per clade (Supplementary Table S5). wMT-1 was mostly expressed in the gut with some expression in the crop and the calciferous gland. wMT-2 concentrated in the tissues of metal detoxification—nephridia, gut, and calciferous gland. wMT-3 was expressed ubiquitously in all tissues, with heightened proportions in the body wall, pharynx, calciferous gland, nerve cord. Unlike wMT-1 and wMT-2, wMT-3 clade was expressed in the reproductive organs aligning with the previous findings of wMT-3 being enriched in embryonic tissues [2]. wMT-3 was also the predominant clade in the nerve cord. wMTs ranged in lengths from 49 to 109 amino acids. Short (~50 amino acids) wMTs had a single wMT domain, and long (100+ amino acids) had three wMT domains; these variants were rare and possibly erroneous. wMT transcript sequence lengths were measured by clade (Supplementary Fig. S21). wMT-1 and wMT-2 clades had short transcript length [722 and 725 bp mean messenger RNA (mRNA) length, respectively]. wMT-3 clade had long transcript length (1412 bp mean mRNA length) with some rare, extra-long sequences (~2700 bp). The difference in length was mainly in the 3′ UTR (Supplementary Fig. S20).

The MSA of wMTs from each clade revealed the extension of cysteine-containing N-terminal region in wMT-3 (Supplementary Fig. S22). This region may extend the globular MT fold, conferring different metal-binding kinetics, or act as a linker, presenting an area for proteolytic cleavage for production of single domain metallothioneins [31]. It was noted that wMT-1 contained 2–3 N-glycosylation sites, while wMT-2 contained only 1, and wMT-3 did not contain any [32]. For the wMT-2 clade, there were two different, almost equally dominant protein types, with transcript counts of 42 and 45. These ‘isoforms’ differ by a single amino acid at position 21, where an Alanine or a Threonine is found depending on the transcript type. The codon here is ‘GCC’ or ‘ACA’, two single nucleotide polymorphisms (SNPs) difference occurring about 13 base pairs into the third exon. Four other SNPs between the transcripts are silent, and occur in four separate codons of the ORF. This suggests that the transcripts are a product of separate homologous genes (possibly duplicates) rather than alternative splicing.

Exonic wMT sequences were extracted from BACs using BLAST alignments of known wMT exons as a guide, then spliced, and translated (Supplementary Table S2). On a phylogenetic tree, the BAC sequences clustered together but did not cluster into either of wMT clades, lying between wMT-1 and wMT-2 clades (Supplementary Fig. S23). This may be due to the differences in geographical location and/or native soil environment between the US and UK worms in this study. The most diverged part of the wMT transcripts between each clade was the 3′ UTR. This divergence resulted in clade specificity, as revealed by BLAST alignments between 3′ UTRs from randomly selected transcripts (Supplementary Tables S6–S19). The 3′ UTR from wMT-1 transcripts of all possible tissue types solely and specifically aligned to BAC sequences Lr13H12, Ef472C1, and Ef318A10, with alignment e-value and length of ~1e−23 and 205 bp, respectively. Likewise, the wMT-2 transcript 3′ UTR of all tissue types (not seminal vesicles and clitellum) solely and specifically aligned to the Lr6F14 BAC, with e-value of ~1e−20 and an alignment length of ~115 bp. This is strong/conclusive proof for Lr13H12, Ef472C1, and Ef318A10 being part of the wMT-1 locus and Lr6F14 encapsulating the wMT-2 locus. For wMT-1, the alignment e-values were not influenced by species differences nor were they significantly influenced by the tissue of origin of the query 3′ UTR. This suggests that there is a highly conserved region in the 3′ UTR of wMT-1 and wMT-2, not significantly influenced by speciation, geographical distance, or adaptation to different soil conditions (metalliferous soil).

**Figure 5.**
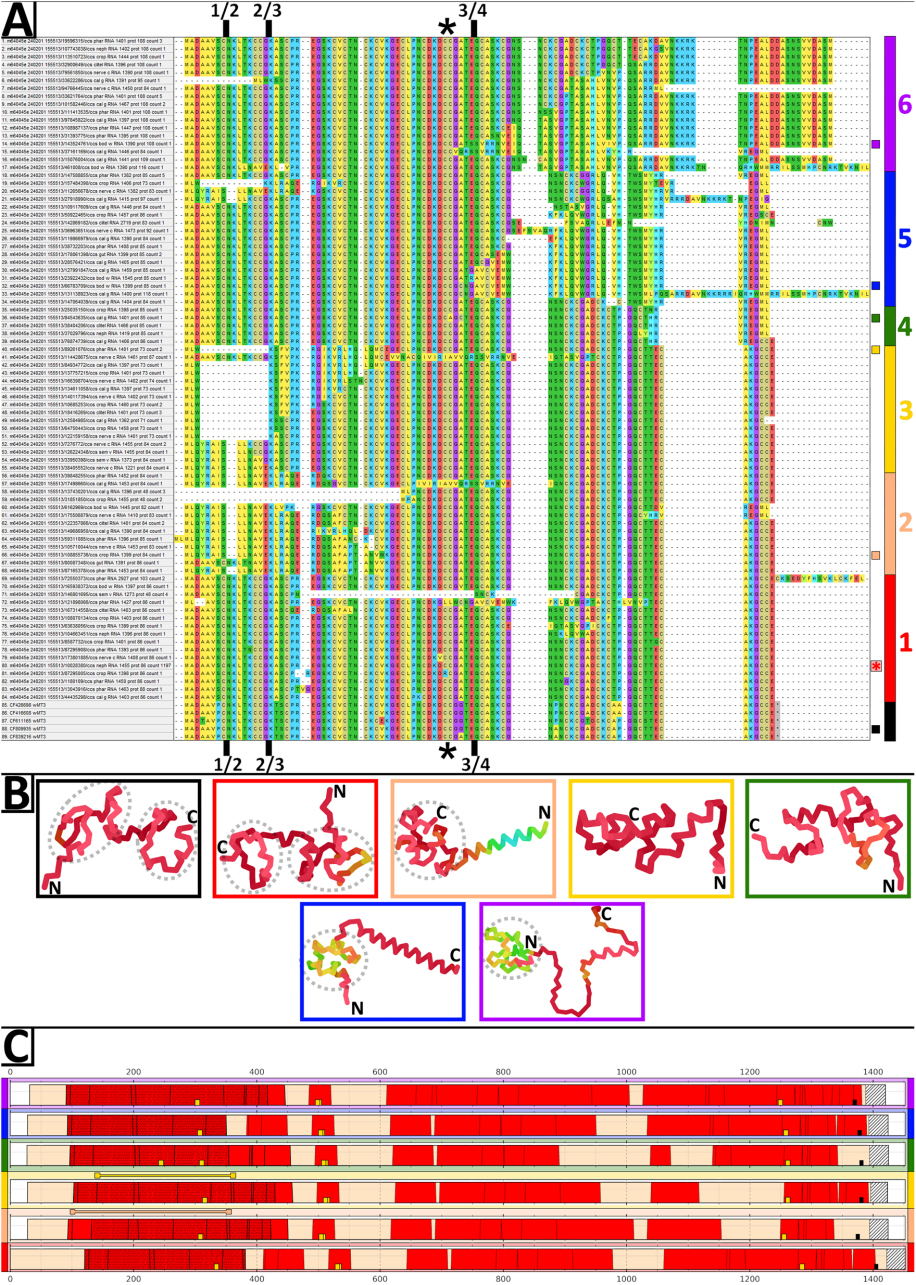
Properties of wMT-3 proteins and transcripts by clade. (**A**) MSA of all discovered wMT-3 protein clusters. Alignment has been arranged in phylogenetic order (Supplementary Fig. S26), with colour bar and numbers representing wMT-3 clades. Black represents NCBI wMT-3 sequences. Coloured squares locate the sequences sampled for structural simulations and transcript visualization. Red star represents the most abundant cluster. Approximate splicing boundaries have been labelled above and below the MSA (Exon 1/2/3/4). Approximate location of the linker region between the globular wMT domains has been labelled with a black star. Transcript ID and properties are specified on the left. (**B**) AlphaFold2 3D structures of proteins from each wMT-3 clade. Picture frames have been colour-coded by clade as in panel (A). Typical wMT cysteine domain structures have been circled in grey. Chain colour represents the predicted local distance test (pLDDT). (**C**) ORF structure of clade-representative wMT-3 transcripts. Red: ORFs; dotted red: longest ORF; yellow: metal responsive elements (MREs); black: poly-A signal; dashed white: poly-A tail; dumbbell: ORF encoding most cysteines; colour bars: wMT-3 clade colour coding.

The 3′ UTR regions of all homologues were BLASTed (e-value = 1, word_size = 4) against the lrRNAseq dataset. The aligned transcripts were filtered by bitscore (Supplementary Fig. S24), merged with the existing list of wMTs, and filtered by cysteine count, yielding 1467 transcripts. High cysteine ORFs were translated and clustered by complete identity, yielding 106 unique clusters. ClustalW MSA was conducted with a representative protein sequence from each cluster to build a maximum likelihood phylogenetic tree (Supplementary Fig. S25). The wMT-1 and wMT-2 clades expanded by a single extra sequence each, compared to the initial tree, while wMT-3 branch grew with 53 new clusters (Supplementary Fig. S26). The ratio of transcripts in predominant cluster compared to the sum of other clusters was about equal in every wMT clade (~8:1). The wMT-3 clade now separated into approximately six sub-clades. Only one transcript cluster was predominant (transcript count = 1197), the rest were in low counts (1–5). The MSA of wMT-3s gave a glimpse of the structural variation in wMTs (Fig. 5A). The most sequence variation was seen in the second wMT domain, or at the N-terminus, with the majority of the first domain being conserved. An extension of the C-terminus was common. Proteins were often seen lacking a particular part of the canonical wMT (e.g. the first domain). Likewise, a canonical part was sometimes replaced with an uncharacterized peptide sequence in a chimeric fashion. In an attempt to resolve structural difference between the wMT-3 proteins of different clades, AlphaFold2 structural predictions were conducted (Fig. 5B and Supplementary Figs S27–S33) [28]. The generated structures were mostly of low confidence, likely due to the heterogenous nature of metallothioneins. AlphaFold2 managed to capture the canonical ‘two domains with cysteine-rich pockets’ structure of metallothionein in NCBI and clade 1 protein predictions. Clade 2 and clade 5 structures showed the replacement of a wMT domain with an alpha helix at N or C terminal, respectively, with very high confidence in clade 2. To pinpoint the cause of these domain substitutions, the ORFs in wMT-3 transcripts were visualized by clade (Fig. 5C). There were many ORFs in each transcript. Generally, the ORF structure remained similar in the wMT coding region; occasionally, certain ORFs were missing (e.g. clade 1, 5). Longest ORF was not always the one coding for the protein with most cysteines (clades 2, 3). Upon closer look at the ORF products of clade 2 wMT-3, it was found that the first ORF in the transcript sequence codes solely for the first wMT domain, and the largest ORF codes for an unknown protein sequence/structure.

**Figure 6.**
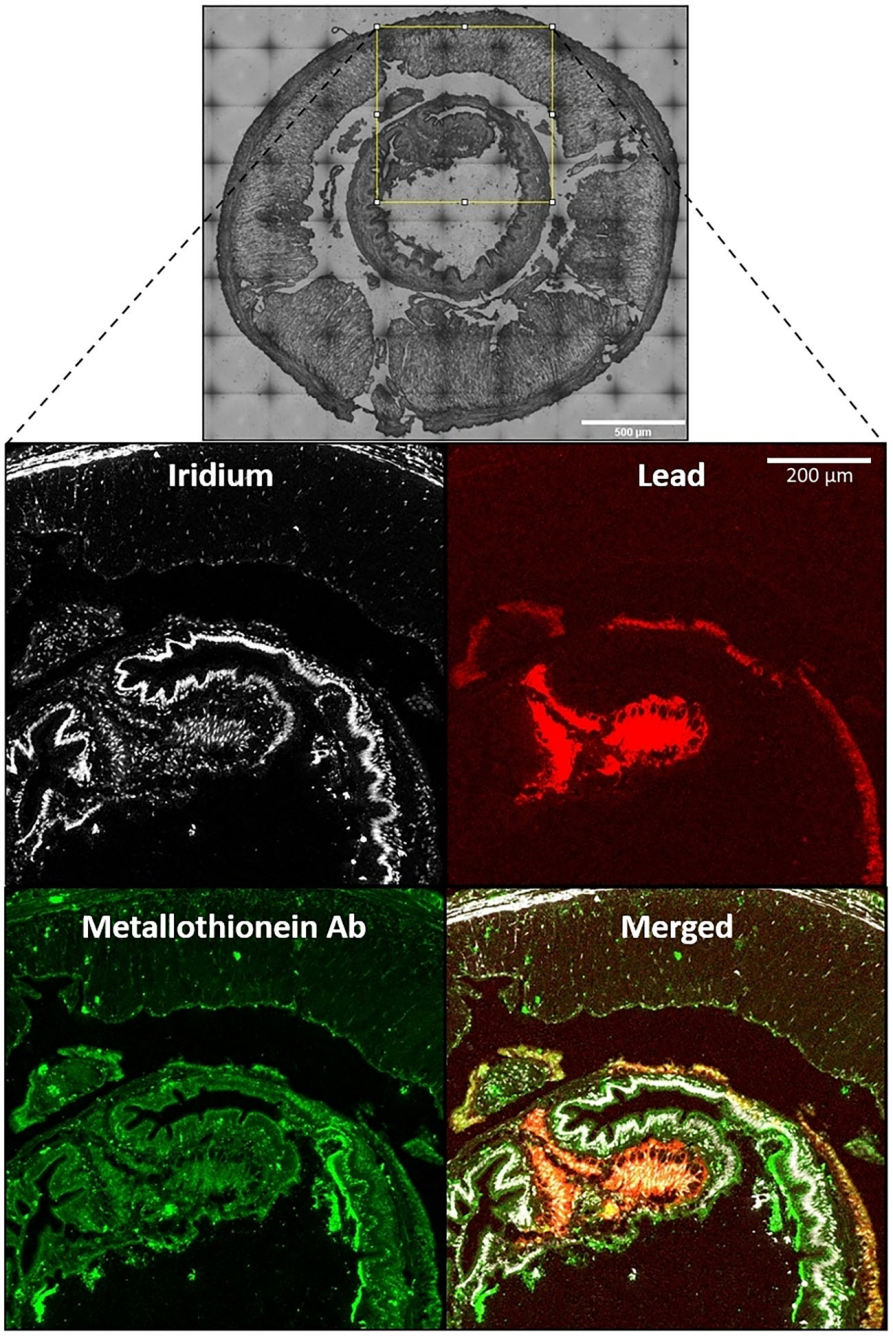
Simultaneous metallomic imaging of DNA, endogenous metals, and proteins.

To visualize the distribution of wMT in the gut tissues of the earthworm during acute Pb toxicosis, an *L. rubellus* earthworm was exposed to 2500 µg Pb/g soil for 2 weeks, which is a concentration well above the national average background and is in line with concentrations of past mining and metal processing sites, such as Cwmystwyth [33]. Imaging mass cytometry allowed simultaneous inclusive imaging of nuclei and Pb at 1-micron resolution (Fig. 6). wMT was visualized using a polyclonal anti-MT earthworm antibody and a metal-coated secondary antibody. As suggested by the lrRNAseq wMT transcript counts, wMT was constitutively expressed in all tissues of the gut region, and elevated in the intestinal walls and chloragogenous tissues, where it co-localized with Pb, implicated in metal homeostasis on an earthworm gut cross-section. FFPE cross-sections of control *L. rubellus* exposed to 2500 µg Pb/g soil Pb for 2 weeks. The gut cross-section was stained with iridium (nucleic acid stain), rabbit anti-wMT polyclonal antibody, and a rare-earth metal-coated anti-rabbit secondary antibody before being imaged via Laser Ablation Inductively Coupled Plasma Mass Spectrometry at 1-micron resolution.

## Discussion

The sequenced BACs served as a valuable resource for studying metallothionein gene architecture and developing the bioinformatic approach. Although the published structures were sequenced in stretches of 2–3 kb, best care was taken to provide the most accurate assembly. Currently, the purpose of the fourth exon duplications, seen in clone Lr6F14, is not known as these duplications are not represented in the final transcript. The nature of the repetitive regions within the wMT gene also remains obscure but could pertain to intron-mediated enhancement or may act as a marker for genetic recombination [34]. From length, the repeat monomers can be classified as minisatellites, implicated in forming hotspots for homologous recombination [35]. Perhaps the reflective shape of the repeats plays an essential role in their ability to recruit meiotic recombination machinery. The tandem wMT duplication observed in clone Lr13H12 may represent a mechanism for coping with an increase in heavy metal burden. The coding regions are 100% identical but the identity across the entire gene is 83% due to differences in the first intron (Supplementary Fig. S3), mainly, the presence of two spacer regions between the repetitive chunks. Besides the conserved cysteine motifs, the MT genes are highly variable and are prone to evolutionary pressure by geographical location and soil conditions. We can hypothesize that because the tandem wMT genes did not diverge in the coding regions, but did in the repetitive intron, the intron has no functional role related to the wMT protein function, further supporting the minisatellite theory. The duplication event could be recent, given extreme instability of certain repetitive regions such as minisatellites. However, we would not see such high conservation of repeats between the different species (Lr13H12 and Ef472C1). Using the repeat structures in Lr13H12 and Ef472C1, it is plausible that the downstream wMT, rather than upstream, is the copied ancestor. Its repeats are more similar to the repeats in Ef472C1. To elaborate, unlike the upstream wMT repeat, the downstream wMT repeat is in the same orientation as Ef472C1 wMT repeat, and its grey repeat cluster differs by the 11-monomer chunk in the middle, an insert. The wMT transcripts could align to the repetitive regions (Supplementary Fig. S14), likely, by chance. The remarkable similarity between the Ef472C1 and Lr13H12 may point towards the issues of cryptic speciation, erroneous initial phylogenetic screening, or even, the emergence of a previously unstudied species.

The lrRNAseq TAST visualizer was designed to examine relatively short (~100 kb), contiguous regions of DNA. The software is capable of comfortably visualizing alignments of heterogenous pools of up to 500 full-length transcripts. LrRNAseq TAST provides options to filter noisy unspecific alignments—minimum transcript coverage and maximal distance threshold between two alignment regions, both of which reduce the number of false transcript alignments. Images of heterogenous transcript alignments may be assessed to pinpoint plausible gene structures (Supplementary Fig. S34). A user could perform a BLAST search to identify candidate loci for extraction and analysis using lrRNAseq TAST. Long-read whole genome assemblers perform poorly when assembling short exons. Long genomic reads themselves should be investigated if the gene of interest is not/partially found in the genome assembly. Several popular software applications that utilize, or process long reads perform poorly—or may fail altogether—when the reference sequence is relatively short (around 100 kb). In contrast, lrRNAseq TAST is optimized for shorter genomic sequences and does not require model training data.

The accuracy of the software ultimately relies on the complementarity in BLAST alignments, which is influenced by the biological noise (e.g. intra-species variation), experimental uncertainty (e.g. sequencing errors), or limitations in experimental design, i.e. mismatched species (*L. rubellus* and *E. fetida*). When sequences are not perfectly complementary, BLAST is still likely to produce an alignment, but the predicted position of exons may not always be accurate, complicating automated prediction of splice junctions. It is recommended to collect all data (e.g. genome and transcriptome) from the same individual organism, if experimentally possible.

LrRNAseq TAST is still in its early stages of development and future updates will bring many new features in all facets of the software including improved visualization features, such as magnification to splice junctions, more sophisticated filtration options. Transcript alignment algorithm will be improved via addition of scoring systems based on sequence heuristics—similarity of splice sites to known splice site consensus sequence, *k*-mer composition, GC%, sequence repetitiveness. External data (protein sequences) could be introduced for statistical and probabilistic methods for enhanced gene detection.

As it stands, manual assessment requires basic knowledge of Python and Pandas dataframes. This is because currently the software is implemented as a sequential Jupyter Notebook, needing minimal Python knowledge to run, but benefitting knowledgeable users with transparency of the code and providing ability for self-directed analysis in the same notebook. Gene splicing is complex, biology is noisy, human reasoning and experience may be required to proceed. LrRNAseq TAST comes with a full documentation, written for the novices, and containing insights for advanced users. Future versions of documentation will explain the inner workings of the algorithms used in the software. Information about the processed transcripts can be retrieved from Python Pandas data structures, which have been described in the user documentation.

A unique method of calculating sequence identity was used in this investigation, involving the collection of BLAST alignments. Repetitive sequences, when present in unequal numbers in the query and the subject, inflate this identity statistic because a single repetitive element on the query can align to multiple places on the subject, and vice versa. This is arguably, rightfully not as stringent as some of the classical methods of calculating percentage identity (e.g. pairwise local/global alignment), because on the *k*-mer composition level, if the *k*-mer length < repeat length, the sequence identity stays the same regardless of the repeat count differences. Secondly, the differences in the order of similar elements between two sequences are not accounted for. Similar substrings will produce a BLAST alignment regardless of shuffled order; element transpositions and inversions will not decrement the identity score. Limitations to the ignorance of collinearity and of alignment multiplicity are partly resolved by visualizing pairwise BLAST alignments, as the lines in the centre of the plot show the extent of genomic rearrangement and repetitiveness. This method also loses local resolution by using the identity statistic over ranges of alignments, without examining exactly where the matches, mismatches, and gaps happen. An elegant solution to this is not clear but may involve some elements of: individually scoring overlapping regions of alignments, producing alignment consensuses, scoring alignment repetitiveness via *k*-mer counting, prioritizing alignments based on e-value, using this technique in low-identity regions of a standard global alignment, using an optimal combination of alignments [36].

The phylogenetic analysis of the non-redundant wMT protein sequences revealed that the identified sequences cluster into three distinct wMT clades. There is, however, significant inter-clade sequence divergence, which may reflect the biological diversity (different genes or alternative splicing), but could be sequencing or biological errors. Equal ratios of predominant to rare transcript clusters between wMT 1, 2, and 3 suggest that these rare wMTs are generated erroneously. Furthermore, the stepwise introduction of the anomalous part of the protein within the sub-clades (2, 3, 5, 6) of wMT-3 (Fig. 5A) suggests a mechanism involving a frameshift. However, the appearance of alpha helices in these structures suggests that there could be order and purpose within these sub-clades. More work is required to understand the function/significance of the alternative wMT ORF products. Filtered transcripts lost during the search for wMT 3′ UTR-containing transcripts may serve auxiliary function in the metallothionein gene network and are worth further exploration. The wMT-3 sequences from NCBI, obtained from early embryo tissues, were an imperfect fit into the wMT-3 cluster. This is likely due to species-specific differences (the earthworm sampling location mismatch). The wMT-3 counts confirm previously observed exclusivity in reproductively linked tissues (Supplementary Table S5). In-depth, population-level, species-specific differences in the wMT genes as well as the differences due to the geographical distance and terrain type are beyond the scope of this investigation.

The distinction between short and long length 3′ UTR in wMT could be in further tissue specificity and additional post-transcriptional control of expression of longer UTRs. 3′ UTR is a region known for its role in transcript localization, stability, export, and translation efficiency via mechanisms such as microRNA silencing or binding of AU-rich element binding proteins [37]. It could be hypothesized that 3′ UTR contains a sequence which stabilizes the transcript in presence of heavy metals, analogous to iron responsive elements which regulate transcript stability based on intracellular iron concentrations [38]. Beyond the promoter region, which is known to contain MREs, and potentially, cAMP responsive elements, MREs were often seen as part of the MT transcript (Supplementary Fig. S20) [21, 39]. Given the existing knowledge of Pb-specific chloragosomes, the findings of this study imply that wMT is not the sole player of the defence against Pb toxicosis, but may provide metal transport and buffering capabilities, reducing oxidative stress and potentially delivering the toxic metals to chloragosomes. There exists knowledge of other, non-metallothionein, metal-binding proteins in earthworms which may be expressed at the same time as metallothionein. An example is the newly discovered, large (150 kDa), protein rich in glutamic acid at the N-terminus, seen to be expressed in chloragogenous tissues during cadmium stress [40]. Furthermore, it was discovered that *L. rubellus* can sequester silver with high tissue specificity, meaning that silver-specific trafficking pathway must exist [21]. LrRNAseq dataset may serve to confirm the existence of or to discover such proteins in the earthworm proteome for procedures of further study. This study establishes a transferable framework for resolving complex genomic loci and analysing transcripts, for accurate curation of gene families with implications for refining genome annotation, understanding gene splicing, and exploration of the stress-responsive pathways across metazoans.

## Supplementary Material

lqaf195_Supplemental_Files

## Data Availability

The data underlying this article will be shared on reasonable request to the corresponding author. The raw and processed long-read sequencing data generated in this study are available through NCBI Gene Expression Omnibus (GEO; https://www.ncbi.nlm.nih.gov/geo/), accession number GSE289972 and the BAC sequences are deposited in Genbank (https://www.ncbi.nlm.nih.gov/nucleotide), accession numbers PV440807–PV440810. The lrRNAseq–TAST project, version 1.0, has been released under the MIT license and is hosted on GitHub (https://github.com/Maxim-Karpov/lrRNAseq-TAST) and Zenodo (https://doi.org/10.5281/zenodo.17593472).
