# Supplementary material for: Insights into the architecture of earthworm metallothionein genes, powered by long-read genomics and transcriptomics
Source: NAR Genom Bioinform. 2026 Jan 8;8(1):lqaf195. doi: 10.1093/nargab/lqaf195 (PMC12783039; doi:10.1093/nargab/lqaf195)
Supplement: lqaf195_Supplemental_Files [file lqaf195_supplemental_files.zip › Figures S1-S34.pdf]

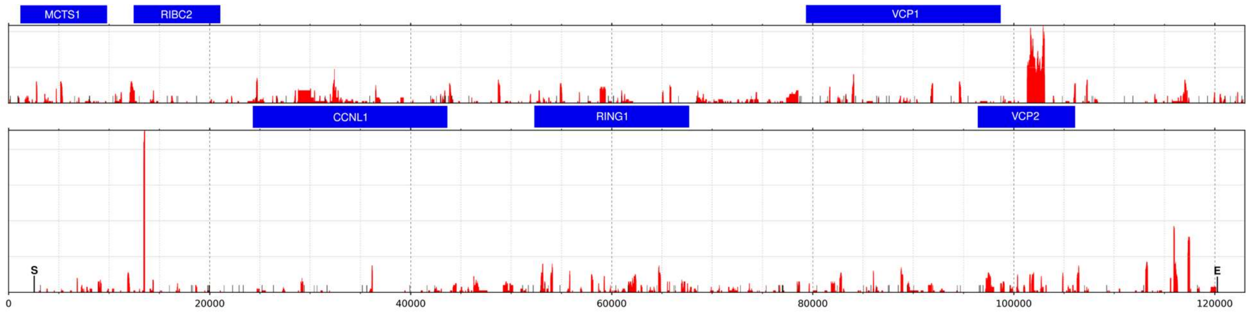

Supplementary Figure 1: Valosine containing BAC repeat structure and annotation. Visualisation of the clones Ef20E08, and Ef91F03 (top and bottom, respectively). Repetitive structures were visualised in red based on BLAST self-alignments. The depth of the self-alignment is qualitatively displayed on y-axis. Gene annotations were displayed above the corresponding repetitiveness plot in blue rectangles. All sequences were centred on the plot, with the starts of the sequences indicated by the letter **S** and the ends by the letter **E**. The ceiling of the plots was scaled to the maximal self-alignment depth of the corresponding clone.

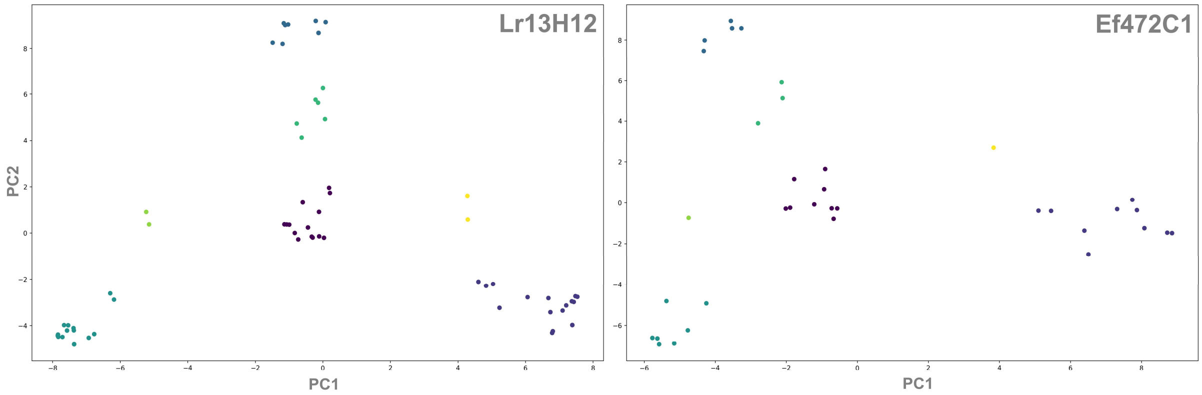

Supplementary Figure 2: DBSCAN clustering of repeat monomer decamer counts principal components. The major tandem repeats of clones Lr13H12 and Ef472C1 were extracted and split into monomers based on BLAST self-alignments. PCA dimensionality reduction was performed on 10-mer counts for each monomer. Monomers were clustered using DBSCAN based on the first two principal components. Each datapoint was coloured based on its cluster.

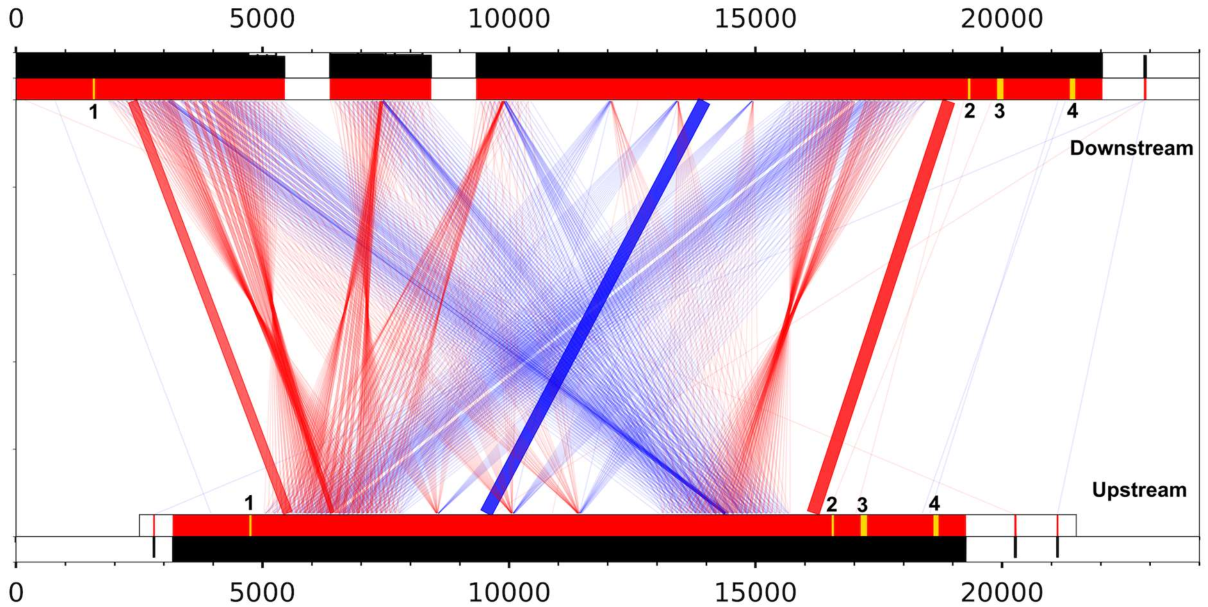

Supplementary Figure 3: Pairwise alignment comparison of the downstream wMT1 gene of Lr13H12 to its upstream tandem duplicate.

Pairwise BLAST alignments were set up between the tandem wMT genes on Lr13H12. Alignment regions were plotted on tracks in red. The alignment paired alignment regions were connected by a red or a blue line depending on alignment orientation (forward, reverse, respectively). The line thickness and opacity is a measure of the alignment bitscore. The alignment identity at each coordinate was quantified and visualised as an area under curve in black, next to the alignment track. PID: percentage identity.

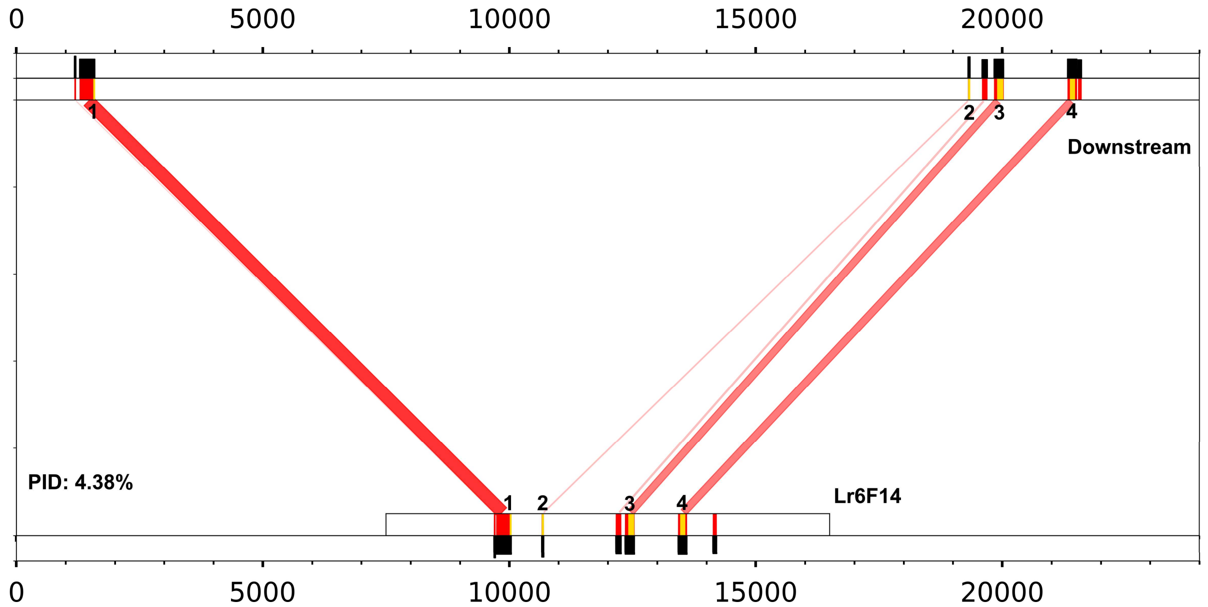

Supplementary Figure 4: Pairwise alignment comparison of the downstream wMT1 gene of Lr13H12 to Lr6F14 wMT.

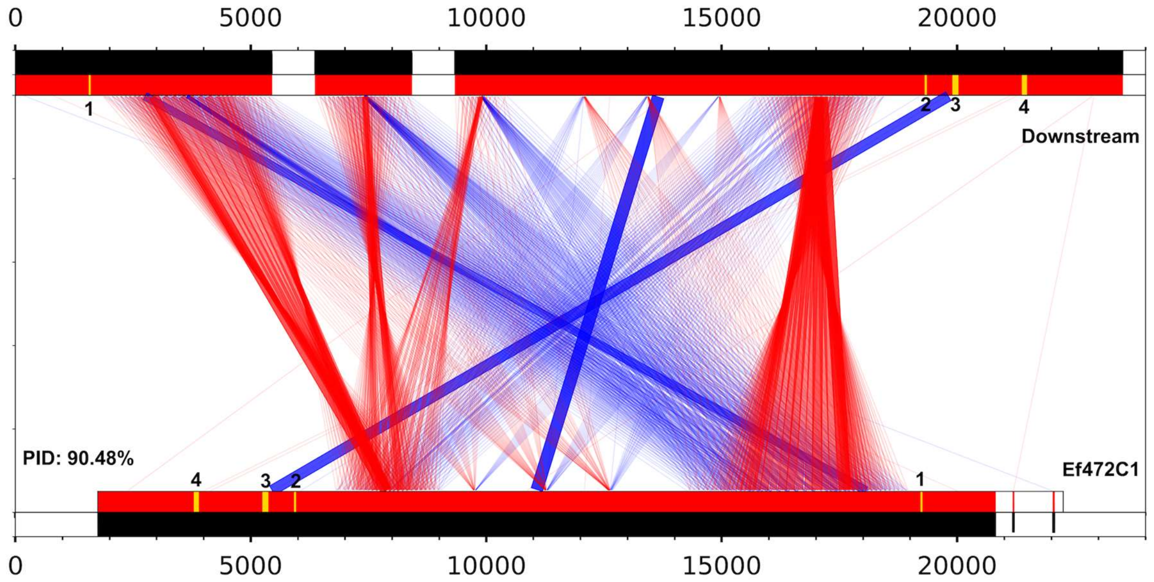

Supplementary Figure 5: Pairwise alignment comparison of the downstream wMT1 gene of Lr13H12 to Ef472C1 wMT. Ef472C1 was not reverse complemented.

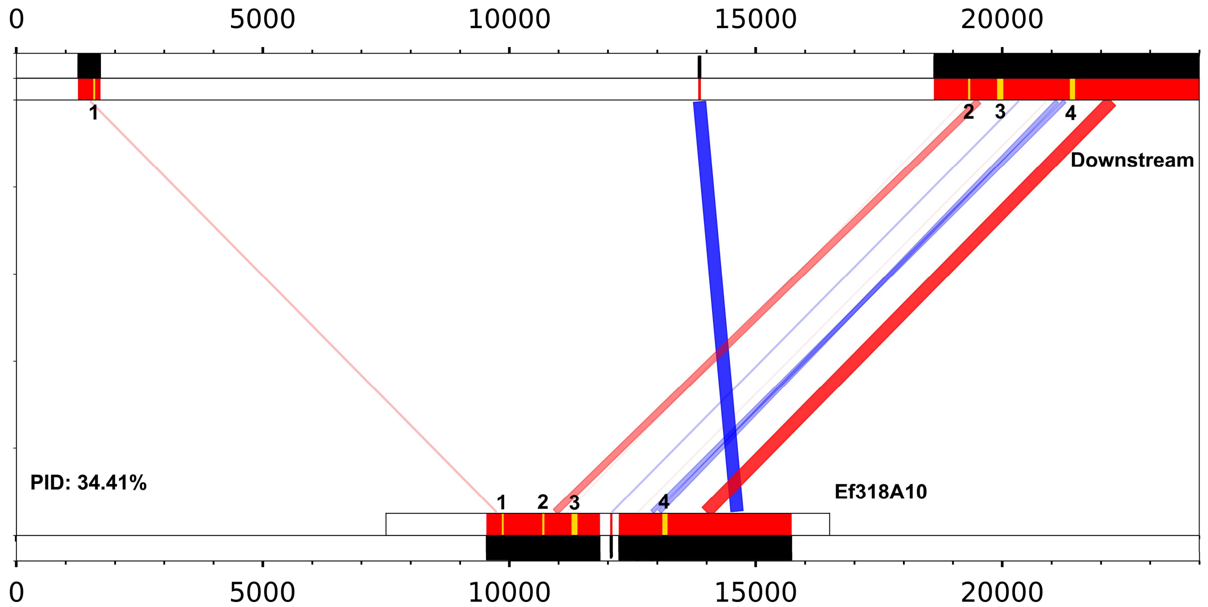

Supplementary Figure 6: Pairwise alignment comparison of the downstream wMT1 gene of Lr13H12 to Ef318A10 wMT.

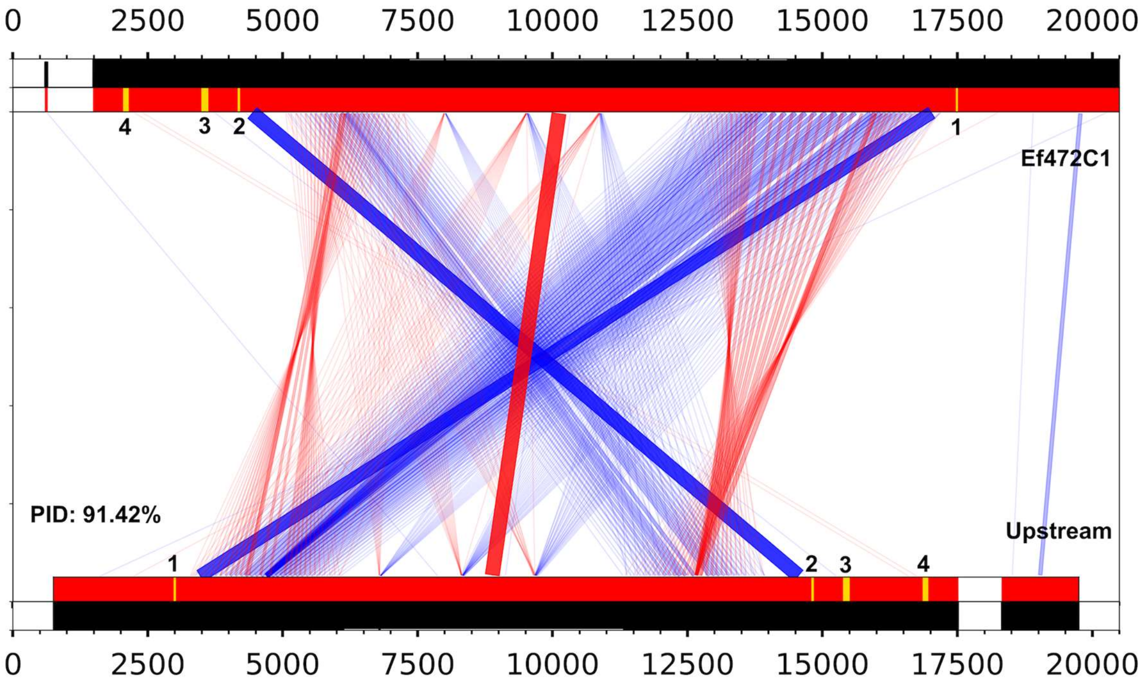

Supplementary Figure 7: Pairwise alignment comparison of the wMT gene between Ef472C1 and the upstream wMT of Lr13H12.

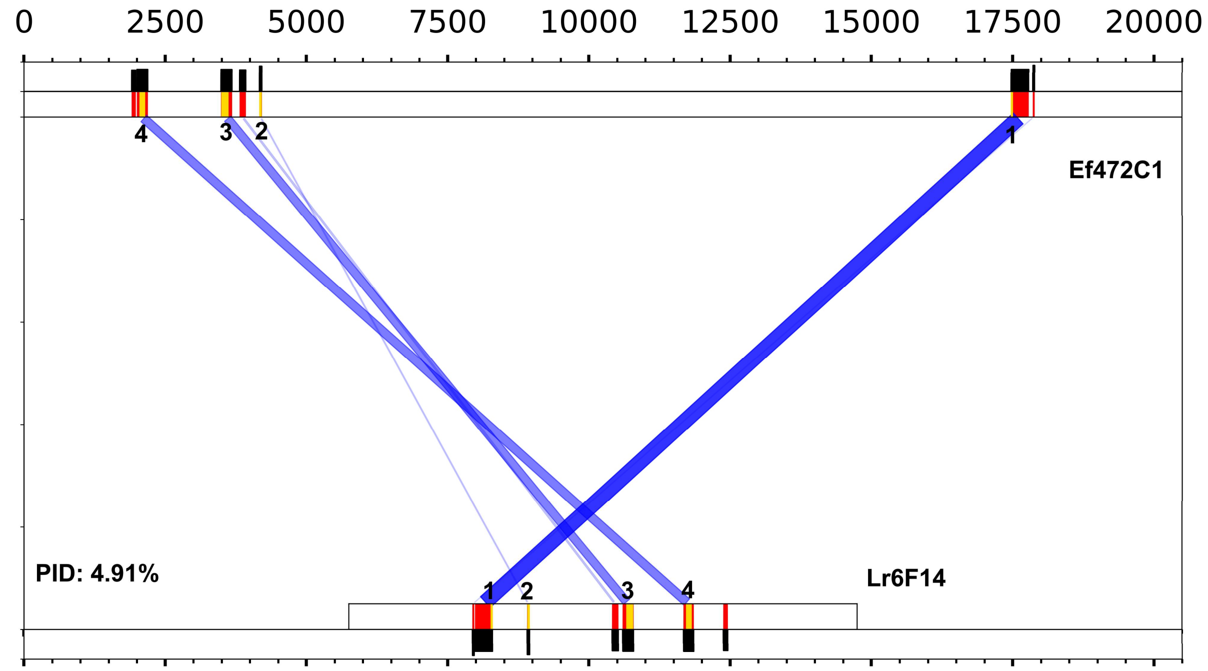

Supplementary Figure 8: Pairwise alignment comparison of the wMT gene between Ef472C1 and Lr6F14.

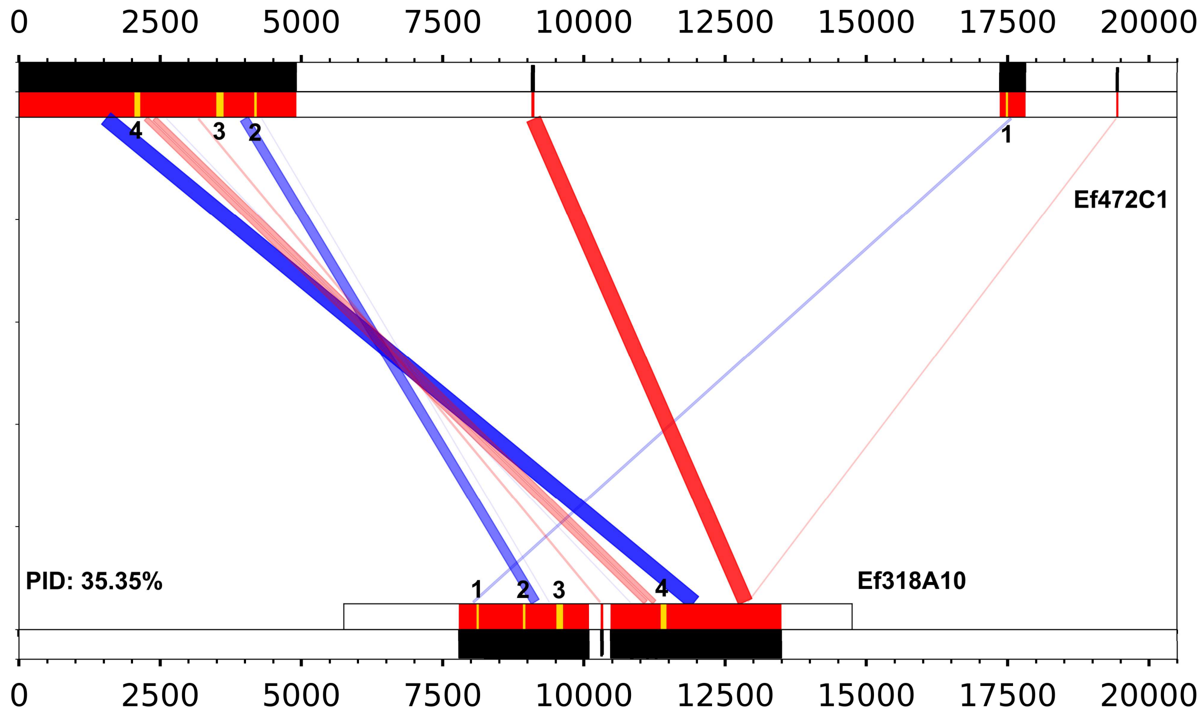

Supplementary Figure 9: Pairwise alignment comparison of the wMT gene between Ef472C1 and Ef318A10.

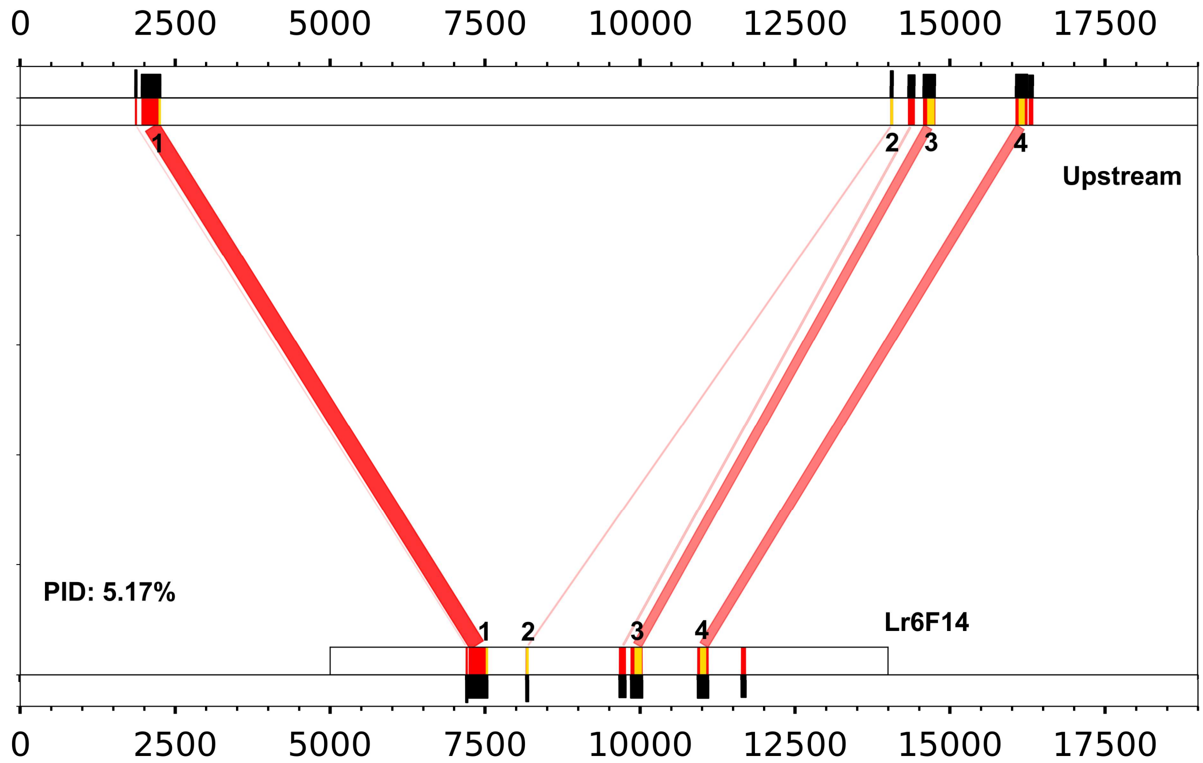

Supplementary Figure 10: Pairwise alignment comparison of the wMT gene between upstream Lr13H12 wMT and Lr6F14.

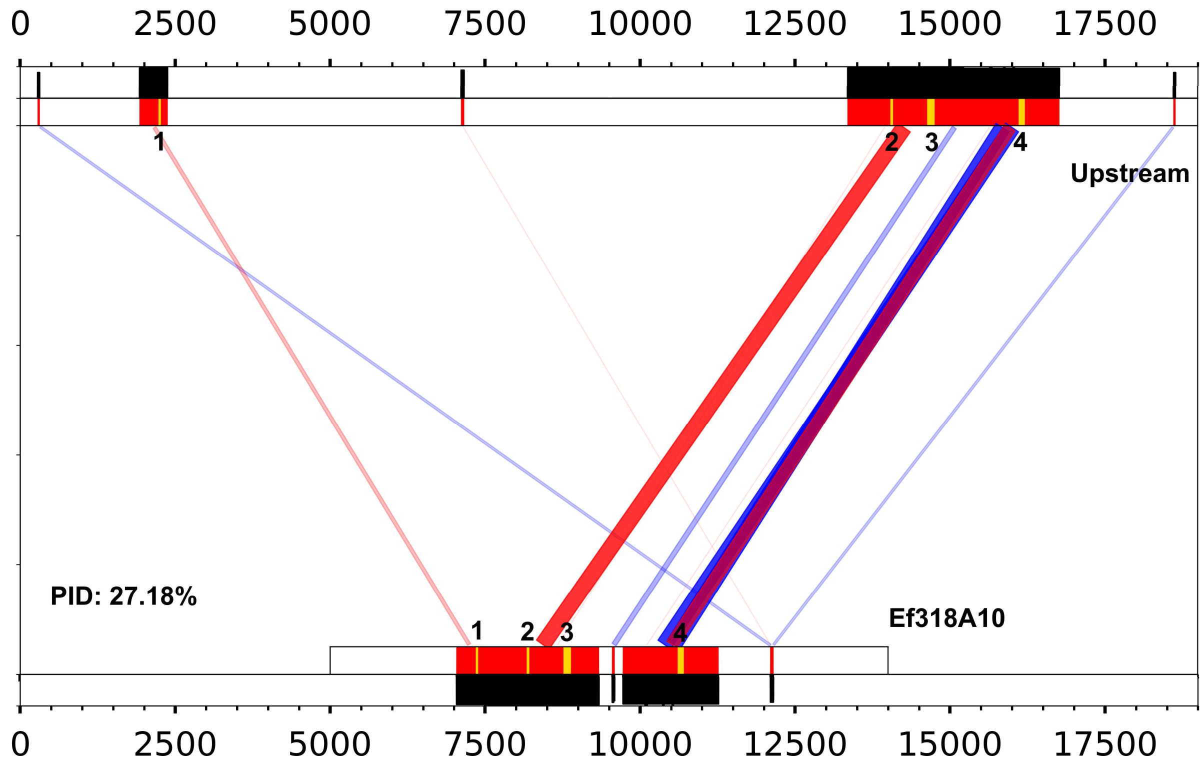

Supplementary Figure 11: Pairwise alignment comparison of the wMT gene between upstream Lr13H12 wMT and Ef318A10.

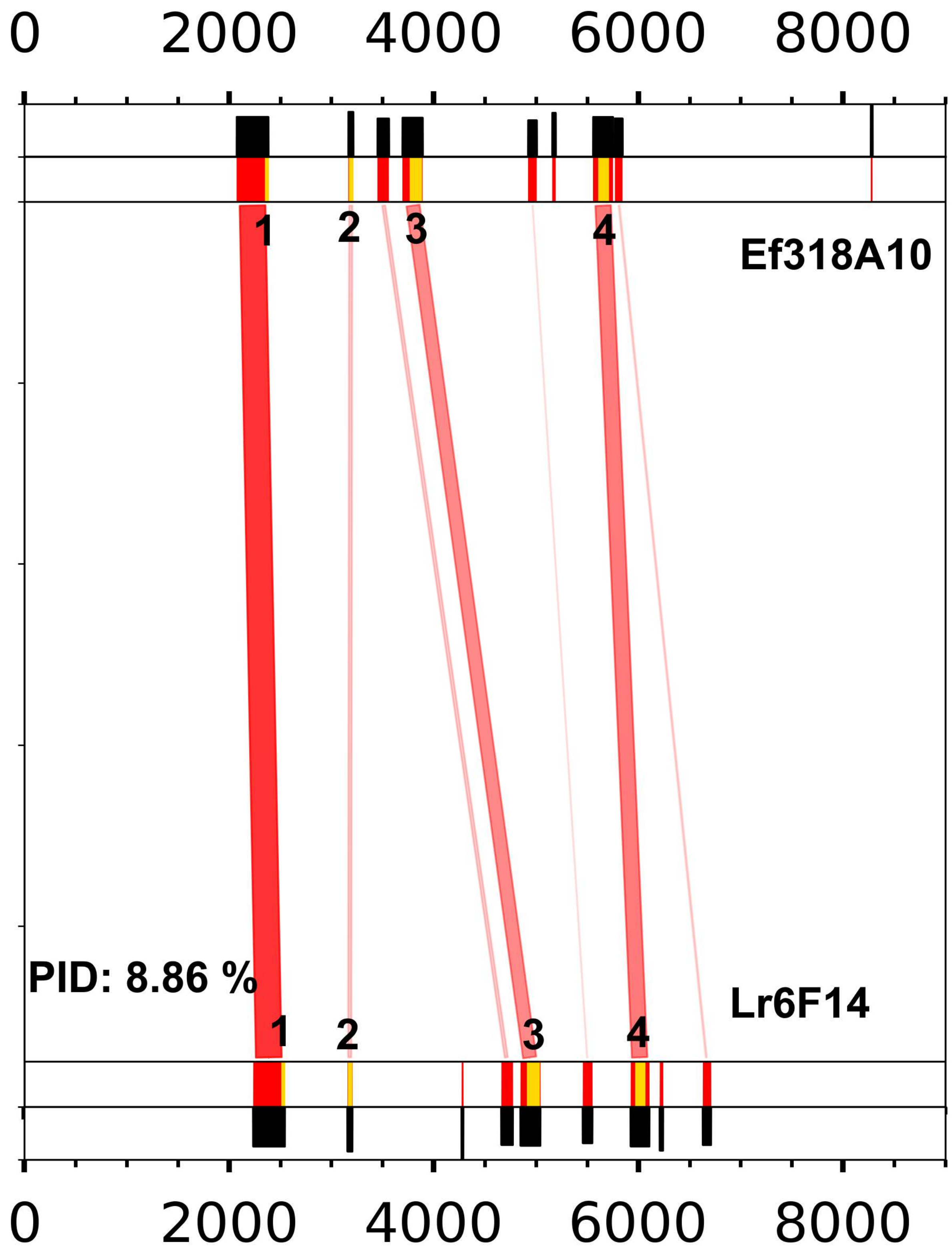

Supplementary Figure 12: Pairwise alignment comparison of the wMT gene between Ef318A10 and Lr6F14.

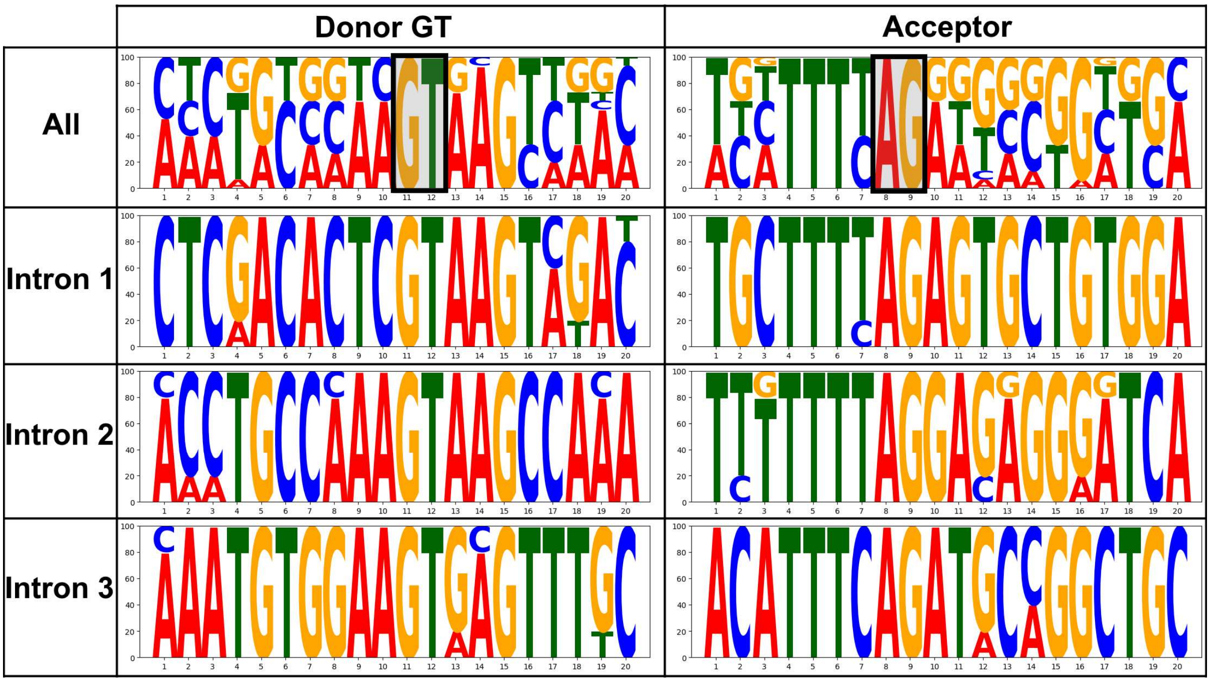

Supplementary Figure 13: Logo plot of donor and acceptor splice-sites of the earthworm MT genes from BACs.

Ten base pair regions flanking donor and acceptor splice-sites were extracted and used for determination of the consensus sequence in a logo plot. The "GT" and "AG" recognition sequences were boxed in the first row for visual assistance.

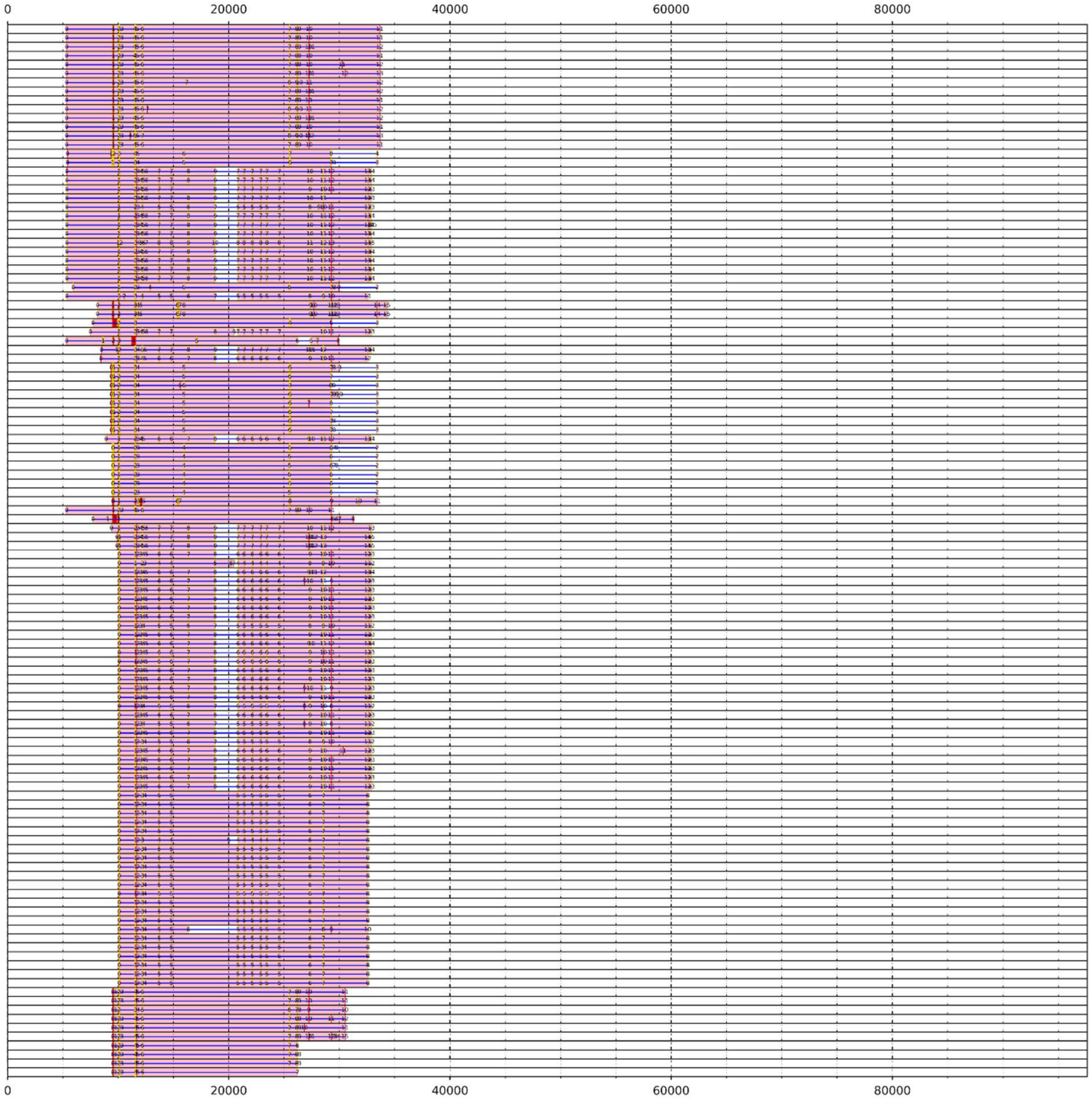

Supplementary Figure 14: wMT transcript alignments viewed on the Ef472C1 BAC clone.

To visualise wMT splicing, wMT transcripts were aligned onto the Ef472C1 BAC clone, and visualised with lrRNAseq GAST under permissive settings (e-value<1) to capture all exons. Various features were annotated using the lrRNAseq-GAST colour coding. Red alignments: open reading frames (ORFs); yellow alignments: longest ORF.

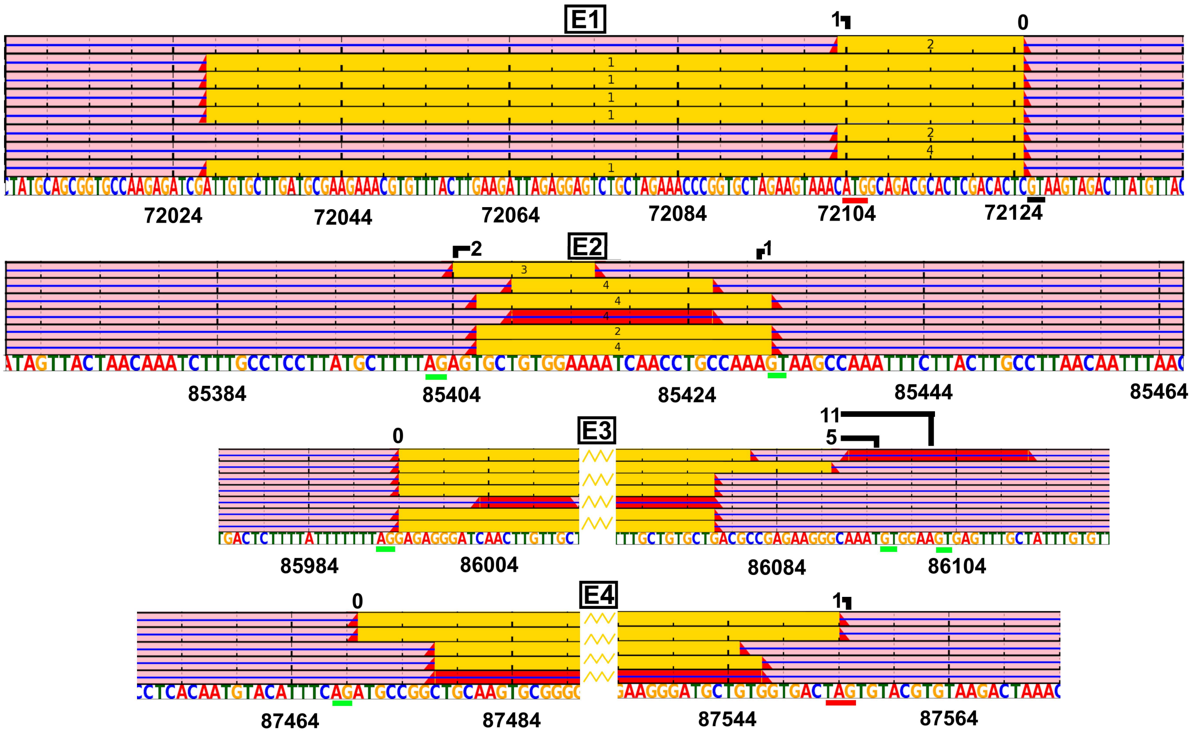

Supplementary Figure 15: A close-up of the lrRNAseq BLAST alignments to the exonic region of the Ef472C1 BAC wMT gene at the exon-intron junctions. Genomic sequence is shown as a track at the bottom. The extent of alignment errors was specified as a number above the transcript alignments. Red alignments: open reading frames (ORFs); yellow alignments: longest ORF; E1/2/3/4: Exon 1/2/3/4; green bar: splice donor and acceptor; red bar: start or stop codon.

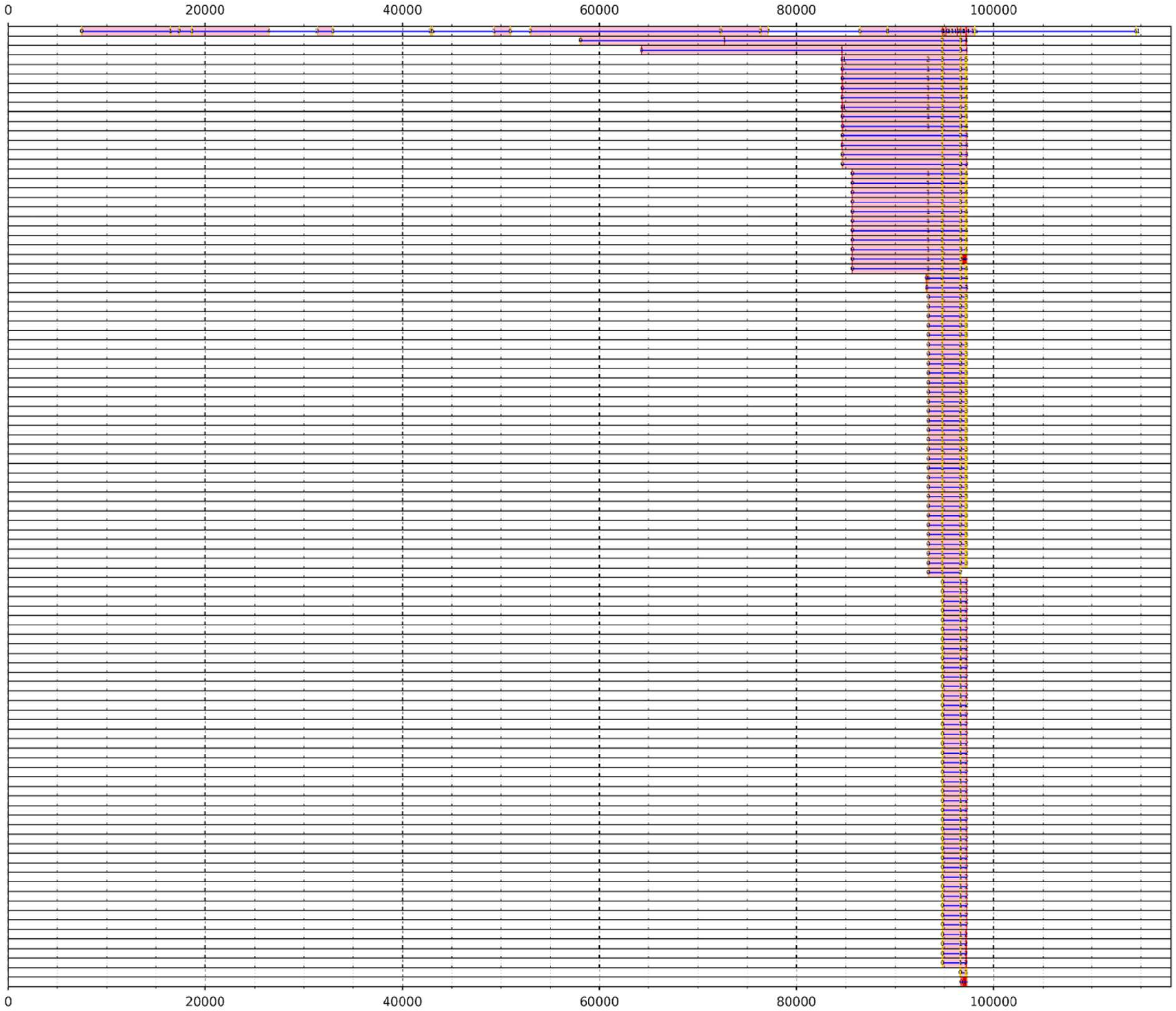

Supplementary Figure 16: wMT transcript alignments viewed on the Ef318A10 BAC clone.

To visualise wMT splicing, wMT transcripts were aligned onto the Ef318A10 BAC clone, and visualised with lrRNAseq GAST under permissive settings (e-value<1) to capture all exons. Various features were annotated using the lrRNAseq-GAST colour coding. Red alignments: open reading frames (ORFs); yellow alignments: longest ORF.

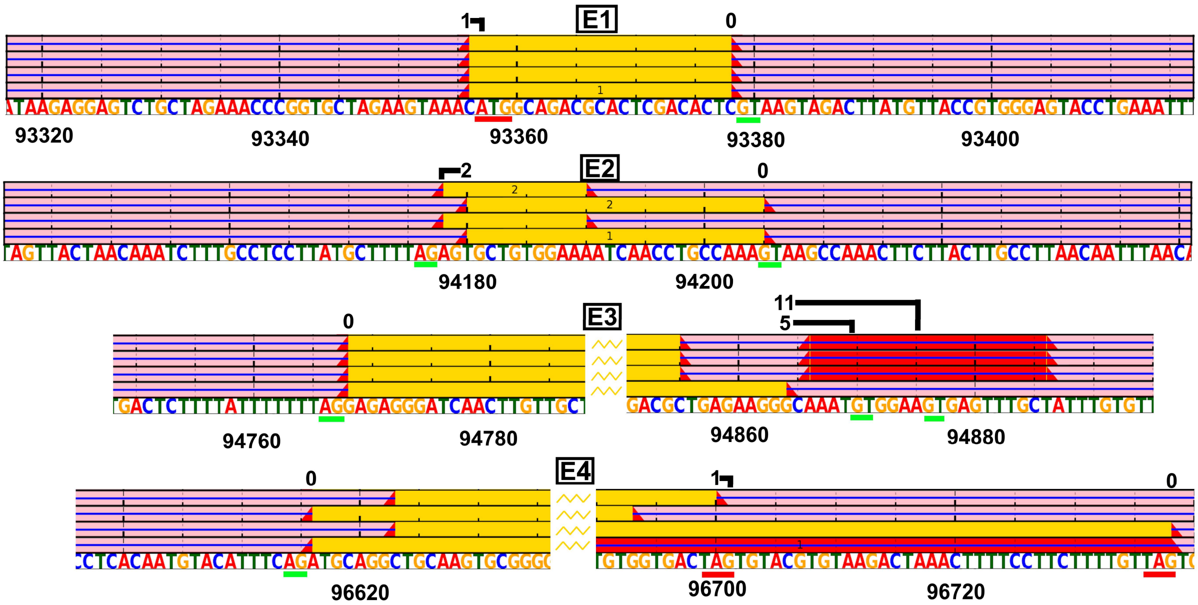

Supplementary Figure 17: A close-up of the lrRNAseq BLAST alignments to the exonic region of the Ef318A10 BAC wMT gene at the exon-intron junctions. Genomic sequence is shown as a track at the bottom. The extent of alignment errors was specified as a number above the transcript alignments. Red alignments: open reading frames (ORFs); yellow alignments: longest ORF; E1/2/3/4: Exon 1/2/3/4; green bar: splice donor and acceptor; red bar: start or stop codon.

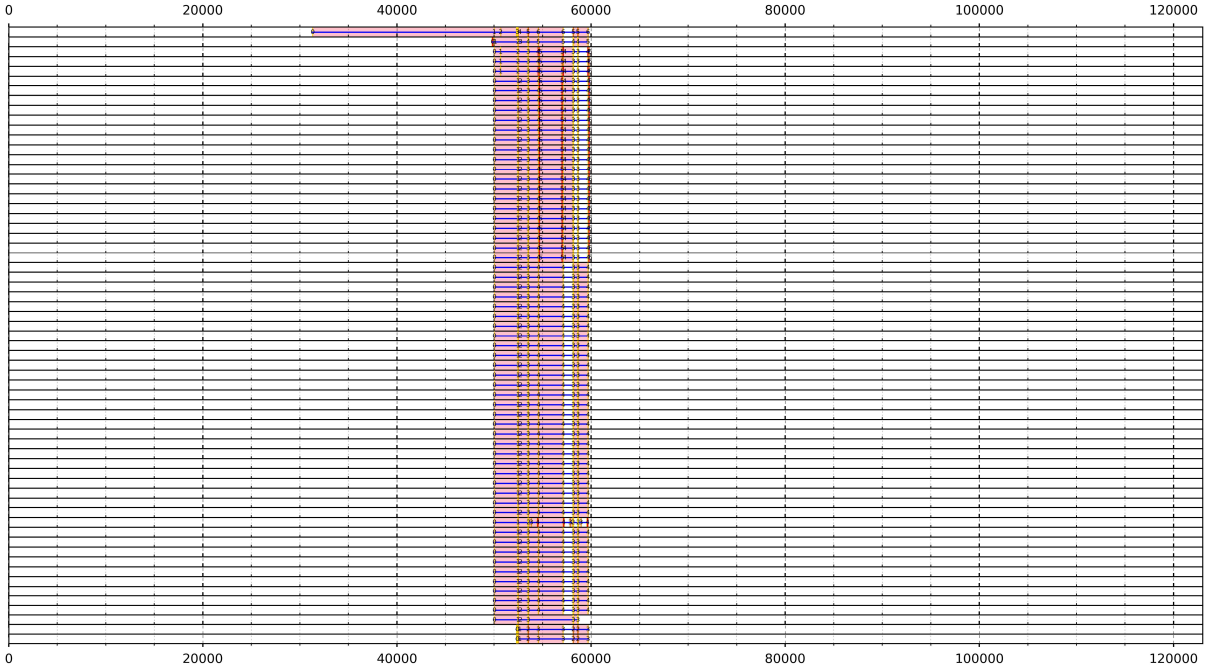

Supplementary Figure 18: wMT transcript alignments viewed on the Lr6F14 BAC clone.

To visualise wMT splicing, wMT transcripts were aligned onto the Lr6F14 BAC clone, and visualised with lrRNAseq GAST under permissive settings (e-value<1) to capture all exons. Various features were annotated using the lrRNAseq-GAST colour coding. Red alignments: open reading frames (ORFs); yellow alignments: longest ORF.

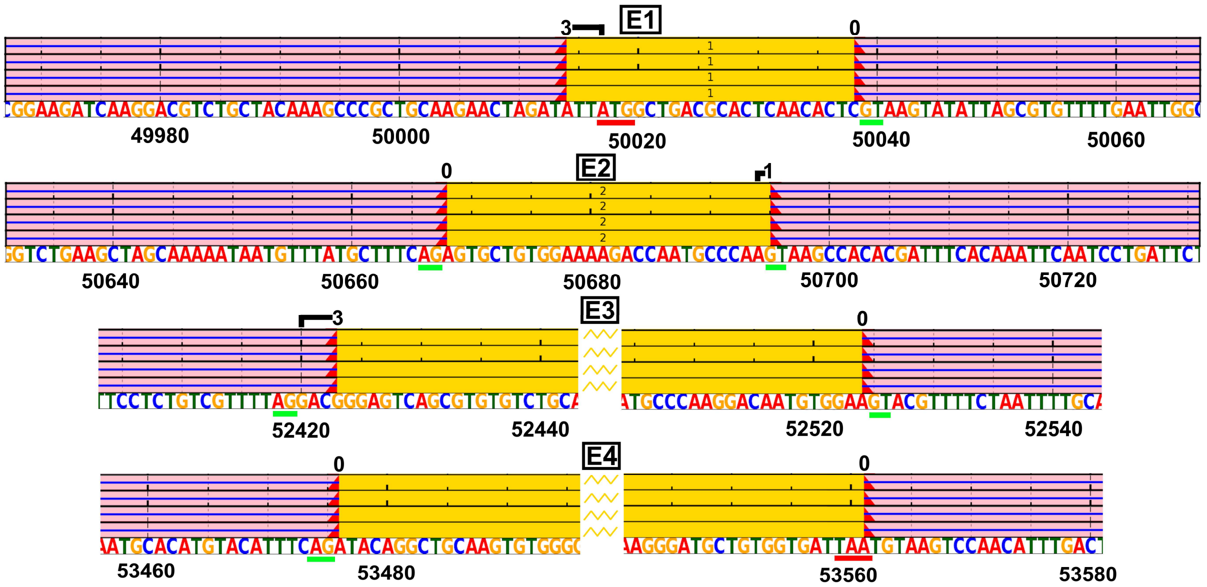

Supplementary Figure 19: A close-up of the lrRNAseq BLAST alignments to the exonic region of the Lr6F14 BAC wMT gene at the exon-intron junctions.
Genomic sequence is shown as a track at the bottom. The extent of alignment errors was specified as a number above the transcript alignments. Red alignments: open reading frames (ORFs); yellow alignments: longest ORF; E1/2/3/4: Exon 1/2/3/4; green bar: splice donor and acceptor; red bar: start or stop codon.

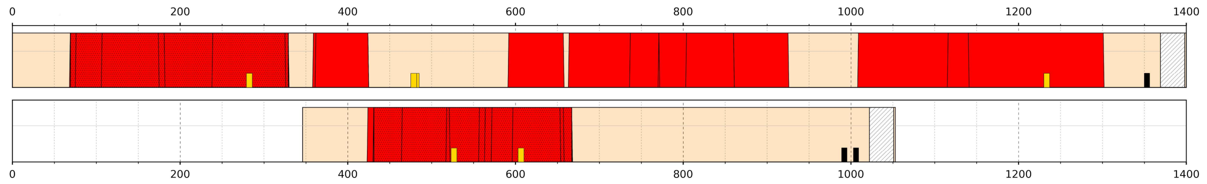

Supplementary Figure 20: Major wMT transcript types.
Visual depiction of the predominant metallothionein transcript types in the lrRNAseq dataset. Red: open reading frames (ORFs); dotted red: longest ORF; yellow: metal responsive elements; black: poly-A signal; dotted white: poly-A tail.

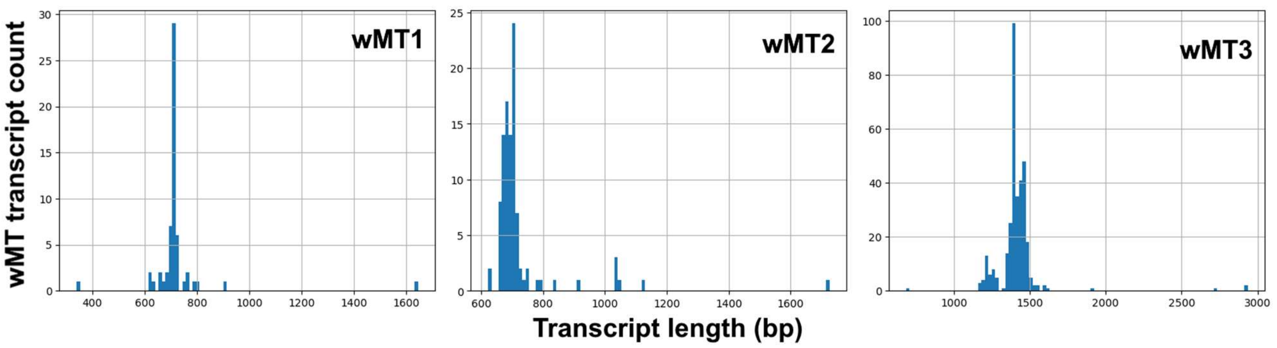

Supplementary Figure 21: Histograms of transcript lengths by wMT clade.

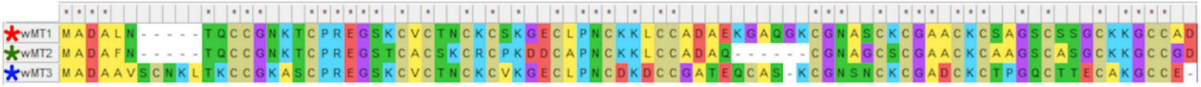

Supplementary Figure 22: ClustalW multiple sequence alignment of wMT protein sequences of highest representation in their respective clade. Coloured stars represent sequences chosen in Figure 4.

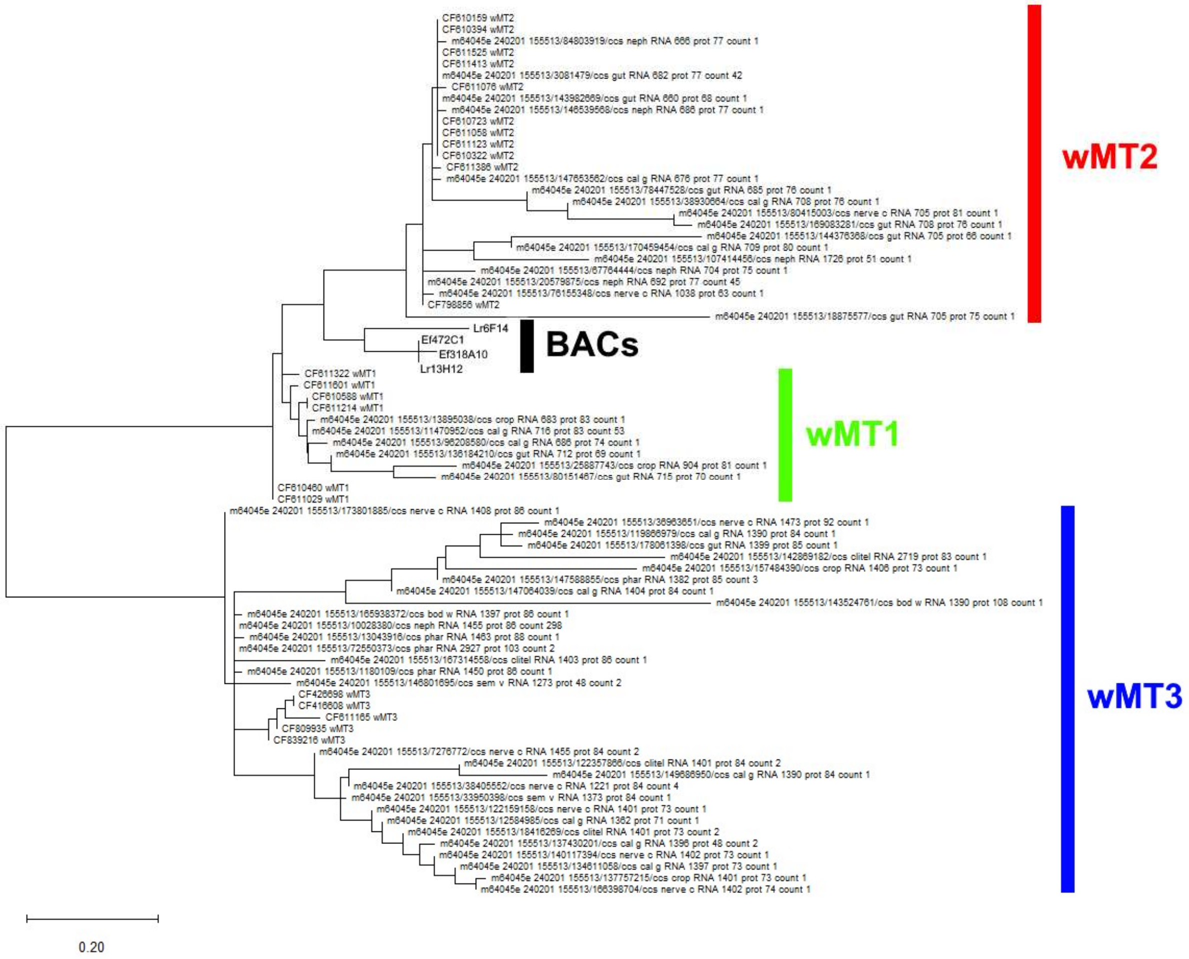

Supplementary Figure 23: Maximum Likelihood phylogenetic tree of wMTs. Phylogenetic clustering of previously identified 521 wMT protein sequences (labelled with transcript ID, tissue of origin, RNA length, protein length, and number of sequences representing the entry) after redundancy reduction, 22 previously studied wMT proteins from an expressed sequence tags library for isoform reference (labelled with NCBI accession and isoform type), and the 4 wMT sequences originating from BACs in this study [2]. The plausible clades were labelled with a coloured line on the right.

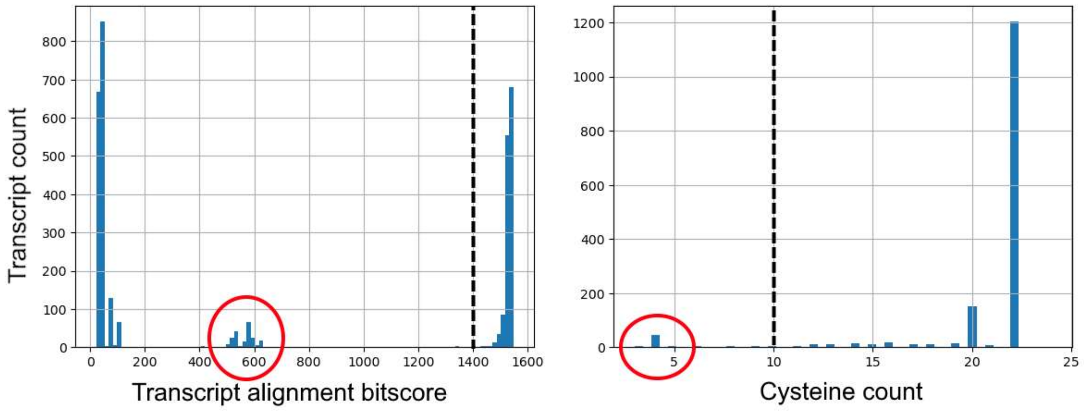

Supplementary Figure 24: Filtering decisions during search for transcripts with wMT 3'UTR.
Filtered transcript populations of potential interest have been encircled. Filtering threshold is displayed as a dashed line.

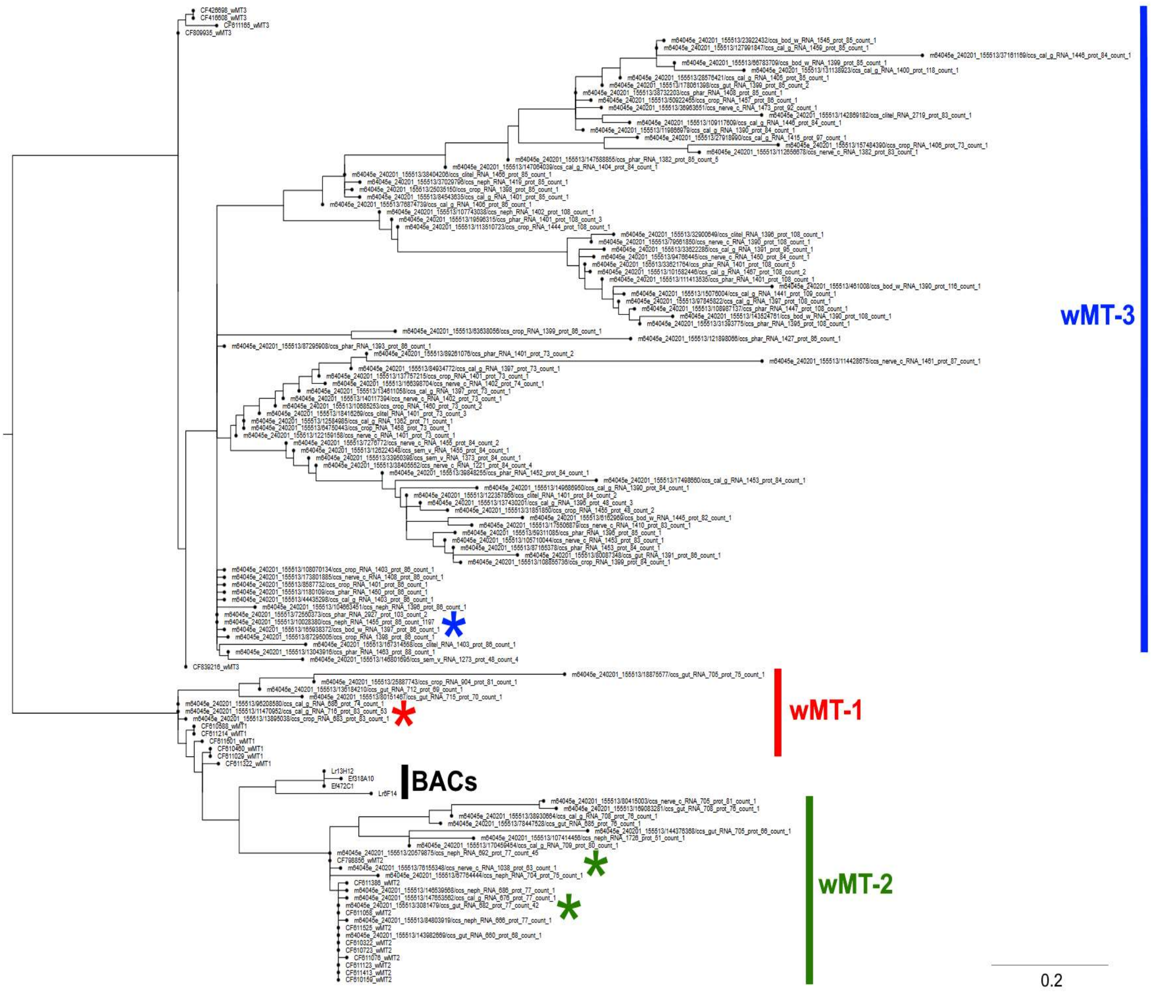

Supplementary Figure 25: Maximum Likelihood phylogenetic tree of all discovered wMTs in the lrRNAseq dataset.

Phylogenetic clustering of previously identified 1467 wMT protein sequences (labelled with transcript ID, tissue of origin, RNA length, protein length, and number of sequences representing the entry) after redundancy reduction, 22 previously studied wMT proteins (labelled with NCBI accession and isoform type), and the 4 wMT sequences originating from BACs in this study [2]. The plausible clades were labelled with a coloured line on the right. Stars indicate the location of predominant (highest count) transcript clusters.

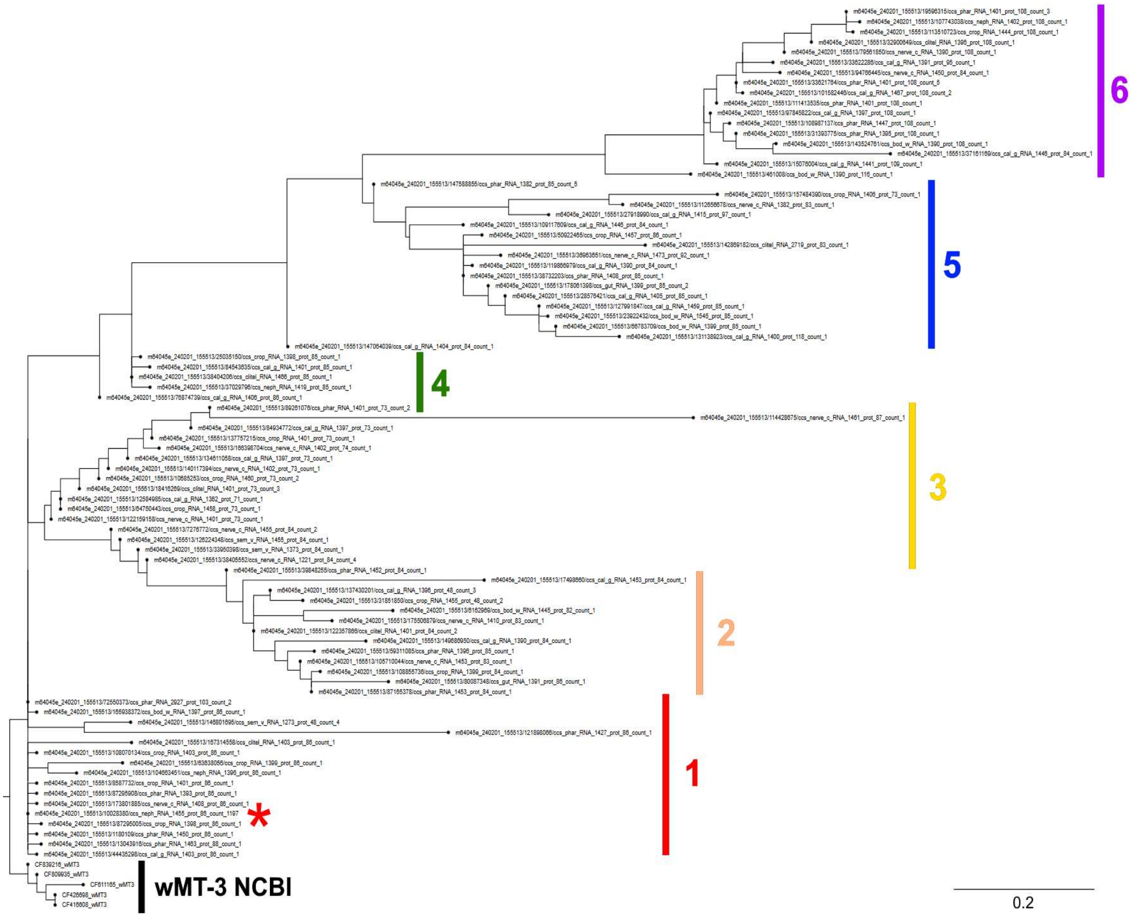

Supplementary Figure 26: Maximum Likelihood phylogenetic tree of wMT-3 clades. Phylogenetic clustering of the wMT-3 clade after coding sequence and 3'UTR data mining. wMT-3 sequences from NCBI have been included as a reference and used to root the tree. The plausible clades were labelled with a coloured line on the right. Star indicates the location of the predominant (highest transcript count) transcript cluster.

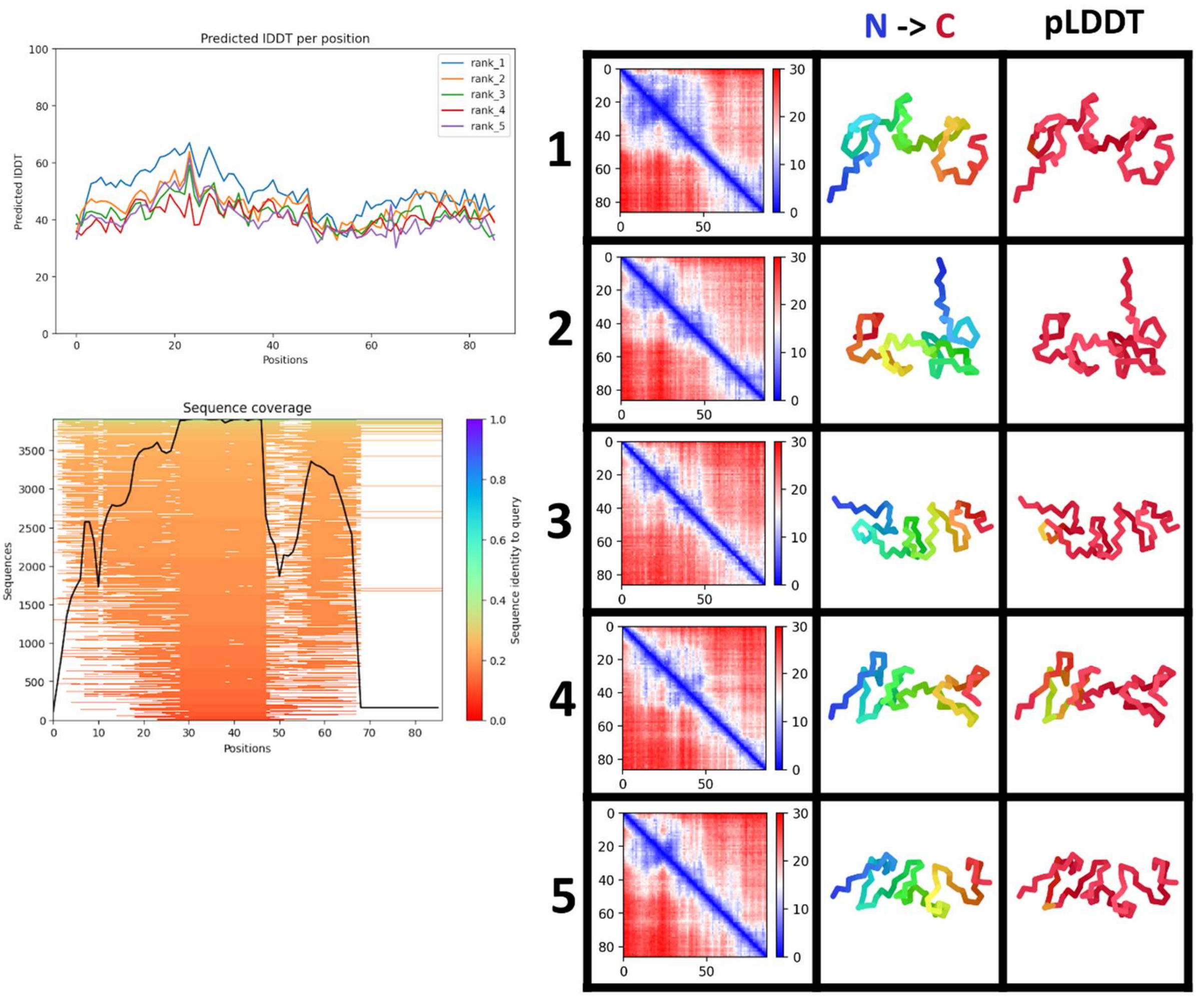

Supplementary Figure 27: AlphaFold2 structural predictions of a wMT-3 from NCBI. (Top left) Predicted local distance difference test (pLDDT) per residue of rank 1-5 structures. (Bottom left) Extent of multiple sequence alignment to the wMT-3 query. Table rows: ranks of structures predicted. Column 1: Predicted aligned error of the structure. Column 2: 3D structure coloured based on position from N (blue) to C (red) terminus. Column 3: 3D structure coloured based on pLDDT.

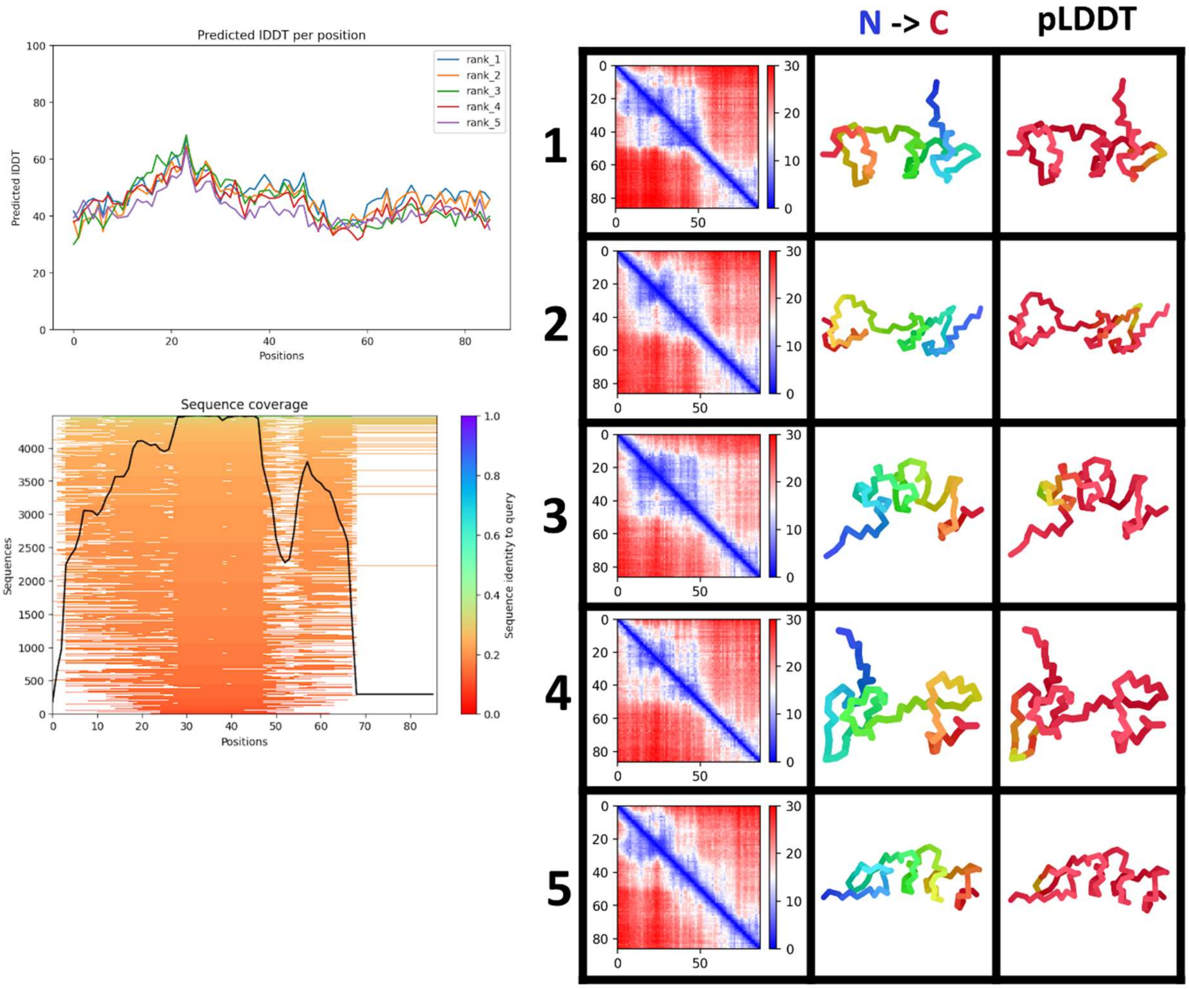

Supplementary Figure 28: AlphaFold2 structural predictions of a clade 1 wMT-3. (Top left) Predicted local distance difference test (pLDDT) per residue of rank 1-5 structures. (Bottom left) Extent of multiple sequence alignment to the wMT-3 query. Table rows: ranks of structures predicted. Column 1: Predicted aligned error of the structure. Column 2: 3D structure coloured based on position from N (blue) to C (red) terminus. Column 3: 3D structure coloured based on pLDDT.

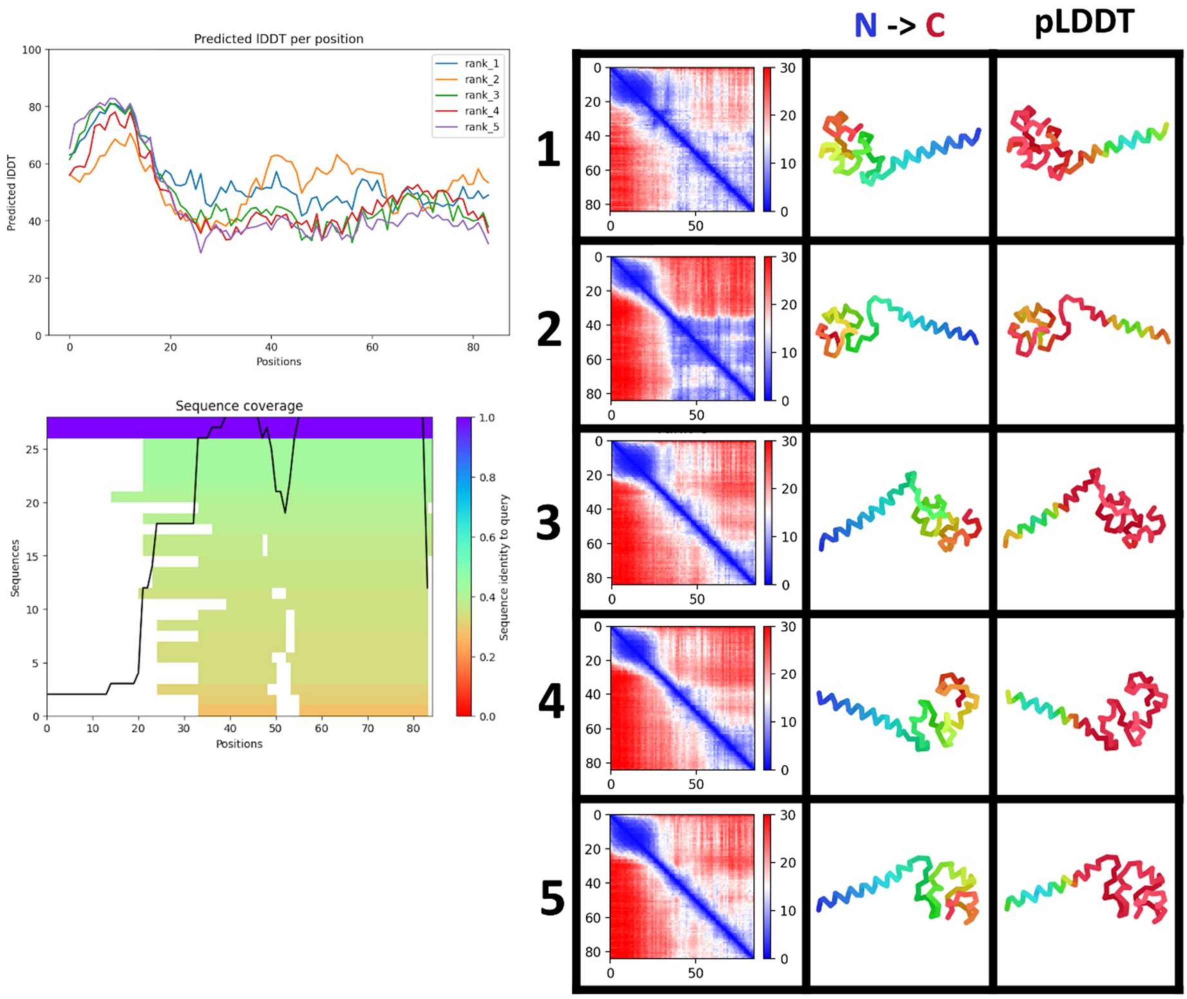

Supplementary Figure 29: AlphaFold2 structural predictions of a clade 2 wMT-3. (Top left) Predicted local distance difference test (pLDDT) per residue of rank 1-5 structures. (Bottom left) Extent of multiple sequence alignment to the wMT-3 query. Table rows: ranks of structures predicted. Column 1: Predicted aligned error of the structure. Column 2: 3D structure coloured based on position from N (blue) to C (red) terminus. Column 3: 3D structure coloured based on pLDDT.

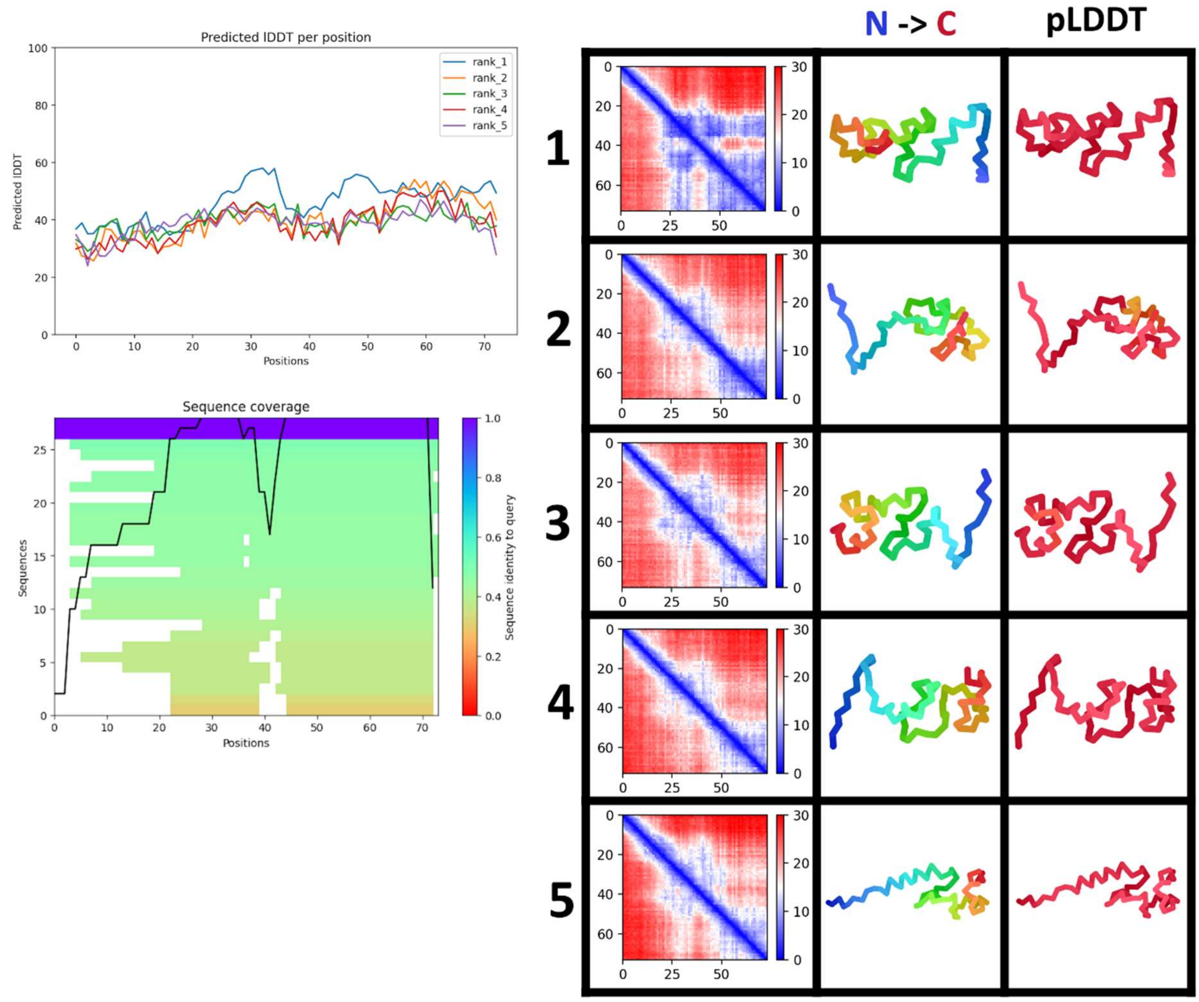

Supplementary Figure 30: AlphaFold2 structural predictions of a clade 3 wMT-3. (Top left) Predicted local distance difference test (pLDDT) per residue of rank 1-5 structures. (Bottom left) Extent of multiple sequence alignment to the wMT-3 query. Table rows: ranks of structures predicted. Column 1: Predicted aligned error of the structure. Column 2: 3D structure coloured based on position from N (blue) to C (red) terminus. Column 3: 3D structure coloured based on pLDDT.

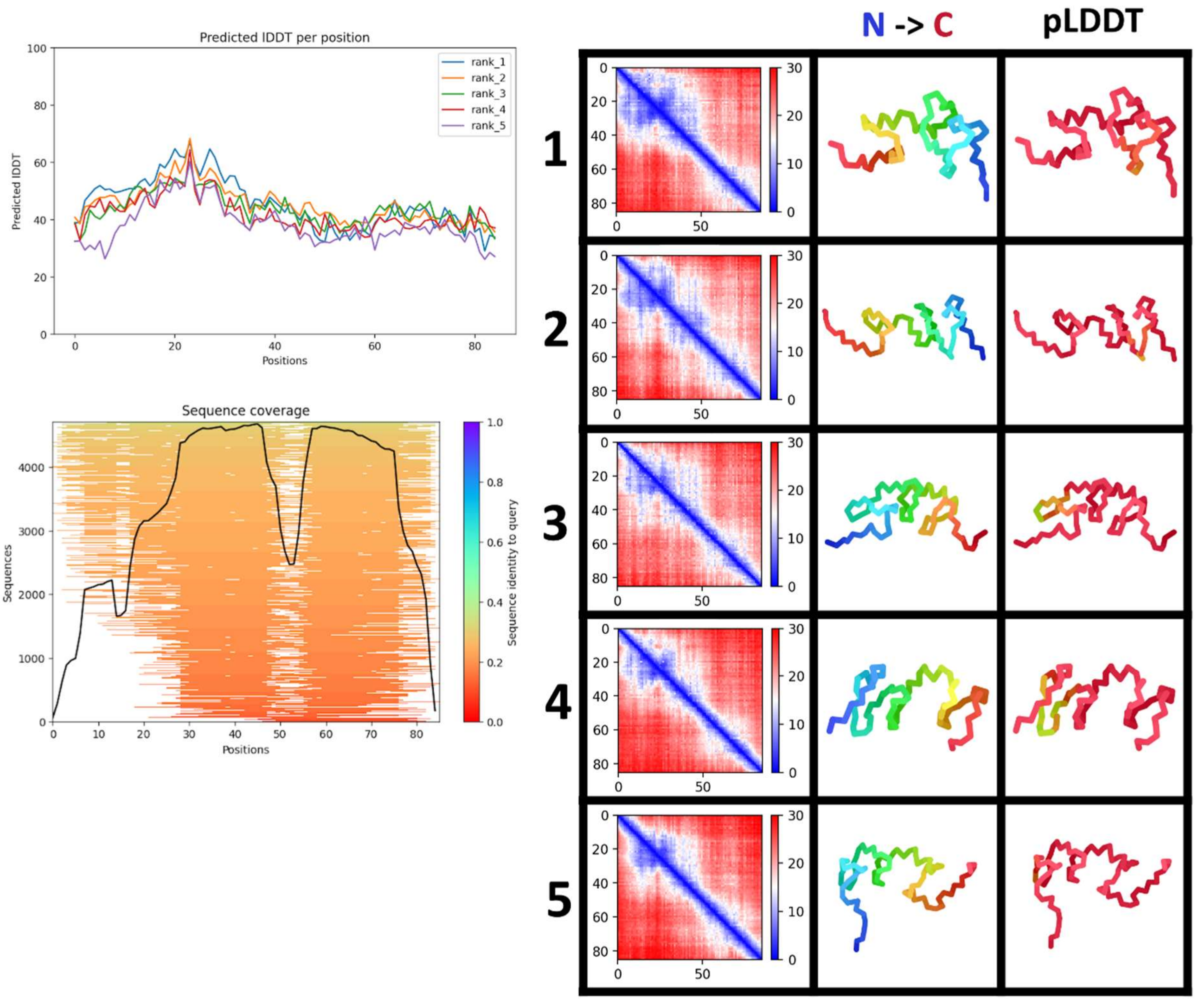

Supplementary Figure 31: AlphaFold2 structural predictions of a clade 4 wMT-3. (Top left) Predicted local distance difference test (pLDDT) per residue of rank 1-5 structures. (Bottom left) Extent of multiple sequence alignment to the wMT-3 query. Table rows: ranks of structures predicted. Column 1: Predicted aligned error of the structure. Column 2: 3D structure coloured based on position from N (blue) to C (red) terminus. Column 3: 3D structure coloured based on pLDDT.

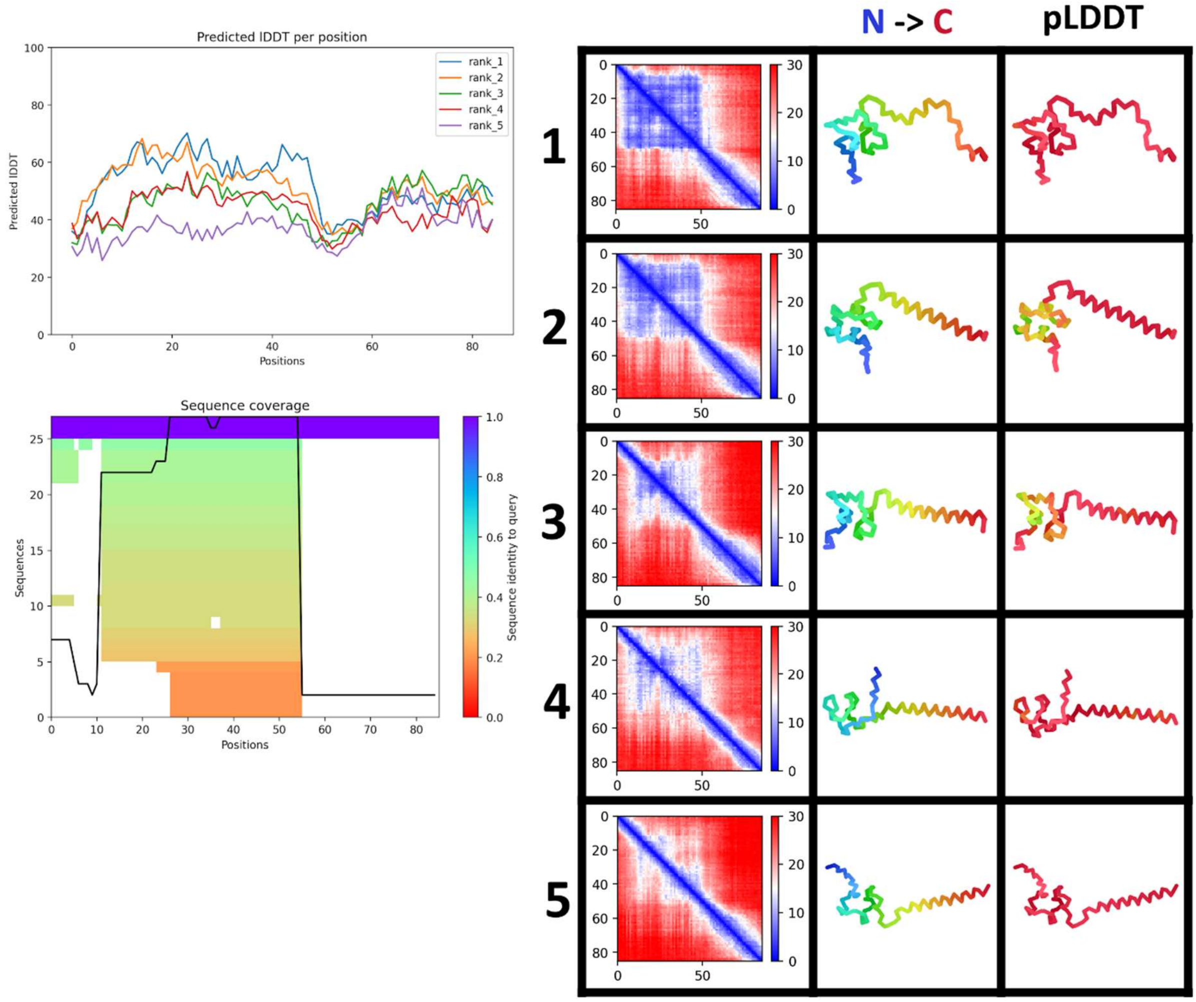

Supplementary Figure 32: AlphaFold2 structural predictions of a clade 5 wMT-3. (Top left) Predicted local distance difference test (pLDDT) per residue of rank 1-5 structures. (Bottom left) Extent of multiple sequence alignment to the wMT-3 query. Table rows: ranks of structures predicted. Column 1: Predicted aligned error of the structure. Column 2: 3D structure coloured based on position from N (blue) to C (red) terminus. Column 3: 3D structure coloured based on pLDDT.

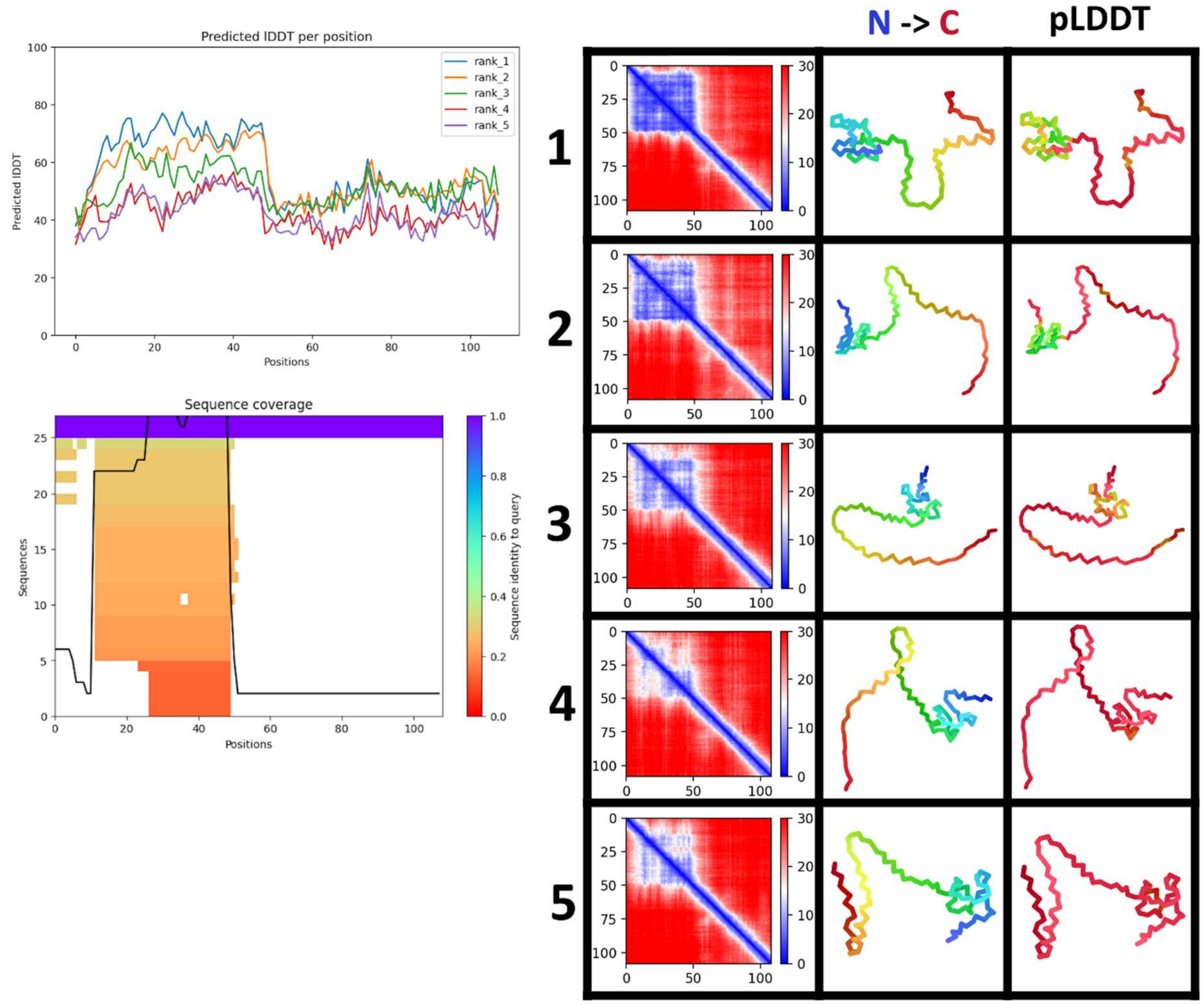

Supplementary Figure 33: AlphaFold2 structural predictions of a clade 6 wMT-3. (Top left) Predicted local distance difference test (pLDDT) per residue of rank 1-5 structures. (Bottom left) Extent of multiple sequence alignment to the wMT-3 query. Table rows: ranks of structures predicted. Column 1: Predicted aligned error of the structure. Column 2: 3D structure coloured based on position from N (blue) to C (red) terminus. Column 3: 3D structure coloured based on pLDDT.

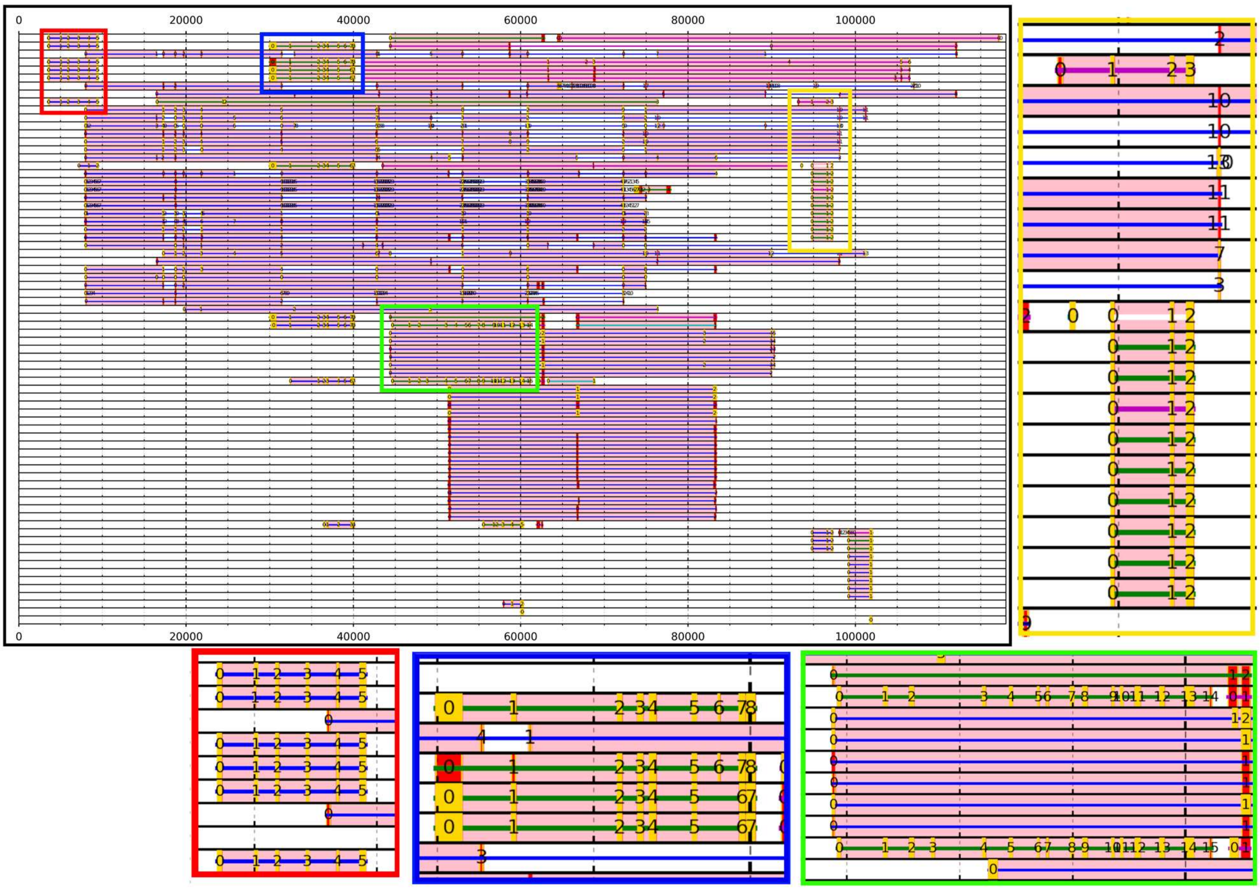

Supplementary Figure 34: Heterogenous pool of transcripts aligned to Ef318A10. Transcript alignments visualisation for clone Ef318A10, highlighting legitimate transcript alignment regions, as characterised by exon-dense regions, with relatively short introns. Transcripts boxed in red: *zcrb1*, blue: *pigq*, green: *mad-1*, yellow: *wMT*.
