# Supplementary material for: Insights into the architecture of earthworm metallothionein genes, powered by long-read genomics and transcriptomics
Source: NAR Genom Bioinform. 2026 Jan 8;8(1):lqaf195. doi: 10.1093/nargab/lqaf195 (PMC12783039; doi:10.1093/nargab/lqaf195)
Supplement: lqaf195_Supplemental_Files [file lqaf195_supplemental_files.zip › Supplementary Sequences.pdf]

>13H12
AAGCTTACATATCAAACCATCTGAATTGCAATGTATTTTGTGCTATATGATGGGTTTATAAAGTG<br>ACCATATAGCCTAAATCGTATTCATTTTGCTGATTTTATCTTCTTGTCTTCTGCTGTGCTTGCAT<br>GCAGTGACGTAACAAGGGGGCAGAGGAGGGTGGTCACCCCAGGGCTTAGGTCAAAAG<br>GGGGCACAAAATGAGGTTTCAGGTAAGTATTTTTACACATCTTGCAGTGTAAATTAATTGCCC<br>AAAGGGGGGTGCAAAATGGAGGTTAAGTGGTAAGTTTTCAACTAGTGGTAAGGGGAGCAA<br>GGGTGCAAAATGCATATACGGCGCTGAATTACTAGAAATTTTTGGTTGACGACTAAAAAGAA<br>AAGGTCATCAAGAATTTGTTATGGAAACATTTTTTTACATCGCTCCCGGGCGCCGACACCC<br>CTAGTTACGCCACTGCTCGCATGCTATTCACTTTCAATAGCCATGCCAGTTTAAGTCTGCCC<br>GTACATAATCATATTGTAATCGTAAACTTGAAATTTCTTCGGCACCCACAAAGGCGAAGTTG<br>CTTGATCCAGCTTATACACAGGCGCTTAATCAAAACAGATCGATTGGCAGAGGGCTAATTT<br>GGTAGGTATACATGTATTTACATTTTAATCGCAATCAAGGAGCAGTTTGGTGGACCGCAGATT<br>ATTAATAGCCTTTCCAGGCTCAAGAATGAGTGACGAGTCAAGGCTTATTTATCTATTTATCTTT<br>ATGTAGACGAAGCCTGTTGAGACTTCAGAACCAAGTTTTGCCGAACTATTGTATTGTATTGCA<br>TTGTATTGTATCTATACATTTATATAGCGCTTCTTGCAGTGCACACCAATCAGAAACGCTTCCA<br>GTGCGAGAGACCCAGAGAGAAGAGAGCAGTCTCGAGAGAACGAAAAGAGGCACTTGGC<br>TCACCAGTTAATAAAGTGGATTAAACATATATTGGATGTGAGTTGAAATTGAAAGCGGAGTTAT<br>TTCGGCTTCTTTGTGTATATTCTGTTAGTTATGTTGCAGGTTAAAGCCATGGACGACACAGAC<br>AACAGCACGGCTGTCATCAGGATCCGTGACAATTTCTATGATTTTATGTCTGGTTGTACTAAA<br>GAGTCAACAGTGAAGCGACTGAAATTTGACACTAGTAGTCTGTGTGAAAGTGATATTGGGAA<br>AGGCAAGTACTGATAATGTTTAATCAGTCGCATTGACAACAAATACATGTGATATTATAACAG<br>GGCTGTAATTAGGTGAACAGAATATCAGTCCCCATTGGGATGGGTTGTAATTAGGTGAACTG<br>TCCCCATTGGGTTGTGTTGTAATTAGGTGAACAGTCCCCATTGGGTTGTGTTGTAATTAGGTG<br>AACAGAATATCTGTCCCCATTAGGTTGTGTTGTAATTAGGTGAATAGAATATCTGTCCTTAGGT<br>TGTGTTGGCTAAGGCTATTTAATTACTTAATTGTTTAGTAATGAGATGCGTTAAGTCATCCTGTT<br>CAAATAAACGAAGATAATGATGATGATGTTGTGATTAGATGAACAGAATGTCGGTCCATTAGG<br>TTGTGTTGTAATTAGGTGAACAGAATATCAGTCCATTAGGTTATGTTGTAATTAGGTGAACAAA<br>ATATCAGTCCCCATTAGGTTGTGTTGTAATTAGGTGAACAAAATATCAGTCCCCATTAGGTTG<br>TGTTGTAATTAGGTGAACAAAATGTCAGTCCCCATTAGGTTGTGTTGTAATTAGGTGAACAGA<br>ATATCTGTCCCCATTAGGTTGTGTTGTAATTAGGTGAACAGAATATCGGTCCATTAGGTTATGT<br>TGTAATTAGGTGAACAAAATGTCAGTCCCCATTAGGTTGTGTTGTAATTAGGTGAACAAAATG<br>TCAGTCCCCATTAGGTTGTGTTGTAATTAGGTGAACAGAATATCGGTCCATTAGGTTATGTTGT<br>AATTAGGTGAACAAAATATCAGTCCCCATTAGGTTGTGTTGTAATTAGGTGAACAGAATATCG<br>GTCCATTAGGTTATGTTGTAATTAGGTGAACAAAATATCAGTCCATTAGGTTATGTTGTAATTAG<br>GTGAACAAAATATCAGTCCCCATTAGGTTGTGTTGTAATTAGGTGAAAAAAATATCAGTCCCC<br>ATTAGGTTGTGTTGTAATTAGGTGAACAAAATATCAGTCCCTATTAGGTTGTGTTGTAATTAGGT<br>GAACAAAATGTCAGTCCCCATTAGGTTGTGTTGTAATTAGGTGAACAGAATATCGGTCCATTA<br>GGTTATGTTGTAATTAGGTGAACAAAATATCTGTCCCCATTAGGTTATGTTGTAATTAGGTGAA<br>CAAAATATCTGTCCCCATTAGGTTATGTTGTAATTAGGTGAACAAAATATCAGTCCCCATTAG<br>GTTGTGTTGTAATTAGGTGAACAAAATATCAGTCTCCATTAGGTTGTGTTGTAATTAGGTGAAC<br>AAAATATCAGTCCCCATTAGGTTATGTTGTAATTAGGTGAACAAAATATCAGTCCCCATTAGG<br>TTGTGTTGTAATTAGGTGAACAAAATATCAGTCCCCATTAGGTTGTGTTGTAATTAGGTGAACA<br>AAATATCAGTCCCCATTAGGTTGTGTTGTAATTAGGTGAACAAAATATCAGTCCCCTTTAGGTT<br>GTGTTGTAATTAGGTGAACAGAATATCGGTCCATTAGGTTATGTTGTAATTAGGTGAACAAAAT<br>ATCAGTCCCCATTAGGTTGTGTTGTAATTAGGTGAACAAAATATCAGTCTCCTTTAGGTTGTGT<br>TGTAATTAGGTGAACAAAATGTCAGTGCCCATTAGGTTGTGTTGTAATTAGGTGAACAGAATAT<br>CGGTCCATTAGGTTATGTTGTAATTAGGTGAACAAAATATCAGTCCCCATTAGGTTGTGTTGTA

ATTAGGTGAACAAAATATCAGTCTCCATTAGGTTATGTTGTAATTAGGTGAACAAAATGTCAGT
CCCCATTAGGTTGTGTTGTAATTAGGTGAACAGAATATCGGTCCATTAGGTTATGTTGTAATTA
GGTGAACAAAATATCAGTCCCCATTAGGTTGTGTTGTAATTAGGTGAACAAAATATCAGTTCC
CATTAGGTTGTGTTGTAATTAGGTGAACAAAATGTCAGTGCCCATTAGGTTGTGTTGTAATTAG
GTGAACAGAATATCGGTCCATTAGGTTATGTTGTAATTAGGTGAACAAAATGTCAGTCCCCAT
TAGGTTGTGTTGTAATTAGGTGAACAAAATGTCAGTCCCCTTTGGGTTGTGTTGCTGCAGAA
GTTTGGATCTAGATGTGTTTCTGTGATCTTTTCACTGTGCTTTAACTTTGAAGACAGAGCCGTA
GCGCACGGCGAAGTGATTGCCGCAAAGTCTGAGGTCACGAGGCTTCAAGCGCAACTGC
AGTCACTCGAAACACATCAGCAGGAACTGATGCTCCAACATGAGAAAGAAATTGACAAAC
TGGTCCAGGAACACCAGGTCATTATATAGCTAGAGATATTTGTCAATTTCTTTCTTCAAATAGT
TTGCTTGTTTTCTTTAATTTGTATCAAACATATAATGGGCATTATTGACAGCCTTGTCGTTTGTTG
GAGTTGTAGTTGTTTTTAAACTGGTGGAATTATTGGGTCGTGAGTTTAGAATGAAGTTGAAAG
GCAAGTGAAACTGCTAGTCATGGTTTGTTGAAGGTCATCCTTATTAAAAAATATCAGTTTCTGT
TTGCTGAAAATTGGCAAGTAAAGGCAACAACGAGAACACGTTGCCGGTGAAATATGTCTAT
CCGGGTGCTTCCGTACTCCATGCAATGTTCAACCAAAGTCTAATCCCTGTCTGCTGTTGTA
GATCACATTGCTCTTGACCTGCAGTGTGGCCTTTCTGCACAAGATCTTCCTAATGACTTTATT
ATCATACCTTGTCTCTTTTCAACTAGACGAACAATTTTTAAAATGAAGAATAGCTATACAAAAC
CACAGTGAGCCTTTGCCACTGTTTGGGTAGTCACATTGTGGATACAGCTGCGCTTGCTAGG
GTCAAGGAATGAGCAAGATAAAAATGGAGCAGTGCTCCCCAGGGACATTGATCTCTAATTT
TAGAGAGTTGGAGAATGTGTATTTTAAGTCTCCAACTGAGAAACATTGTTGGAATTTTCCGCC
ACTATTTTCCACTAAAATCTGCTAAATGGCGGTAAAAACATTTTCCCGTGGGATTGTCATGC
GCTGCGCATGACCGGCCCATGCAACTTCCAAACTAACATGGCGGTCTTACATTGTCCGA
CTCGTCATTTTCGTATCAAATTCGCGTCTTTAAGGTCGCCTAGGGTCAGGTTAAGGGTCGC
GAGGGTAGTTAGGGTTAGTATTAGGGTTAGTGTTGTCATTGGAGCAATATTTCCGCCGCAAT
AGCATATTTTTGGCATTTTAAGTGGCAGAGACTTCTGCAACTTTAGCACCACGCTAGGCTTA
AAGTAACTGCTTTTGTTTTAGAAAGAGATTGCAAAACAAGAGGACGTCAAATATCAACTGCTT
GTCTTTGCAAAGAAGGAAGGCGAAAGGAAGCGGGAGATTCTAGAATTAAAGCAGGAACTC
GAAAAGGCAAAAGAATCACAAAAAATGCTGATGGCTGTTCAGAAGGAGAAAATTAGCCTTG
AAACAGCTTTAGAACAGGTTGTGCTTTAGACTACAATACGTTTGTTTCTGCTTTTTGTCTTTCAT
ATTCTAAATAATTATAAGTTTTCAAGGTGTTCTATTTTAAGTTAGTGCTATTGTTTTGATTTTCAAG
CATTGTTCTGTTTGTGTTTGTTCCTGACACTGCTACTCTGCTCTGTACTAACATTACTATGCTTC
ATTTACACTATTACTCTTTGTCATCATTACTGTGTGTGTGTATATATCTTCGTTTTAATATGAAAAA
ACATCAATAGACAAACCGCTACTCGAATACAAGAGCAATAAGCTTAATACATAAGTCCTATAT
ACTAGTCTTCCTTTACATTGCTACGATGACAGCCACCCTCACCTCTTGGCTACAGACGGCA
TGCGGTTTTCCTGGCGCAGACCTGACCGTGTTTATATCTAGGATGGACTCGATGAGGTCAC
ATGGCTTGCGAACAAAACACCTTTCAAACGTGAAAAATCCCTGGCTAATTCGGCAAAAAC
GTCCGGTACGCTAACCCAGTTCCAAAATCTCCAACATTTATGTAACCACTGCGCAAATTCC
TGCCTGAAGACGAAAATATCCATCGAAACGACACAGCTGTCGACAAGTCGACCAAAATGG
CGGATGACATGCACGGAAATATACAATACAGTTGCTGCTGTTTAGATCATTTAGATATTTATGC
AGGCTTTGCATATGGCTGATTCTGACAATTGCACAAGTTCTTGTTGTTATAAAATGCTTTGATA
CCAGTAGAACATTGGTTGTGGCTCTTGATGTTATATCGATTTCTTTATGACAGACGAAGGAAG
AGATGGCGGATGCGGTCGGCAGAGTGGAGATGGAAAAGAAAAGCCTAAGACAGTCGCT
GGAAGTTGTGCAGAAGCAGTTGAAAGACCAGGAGCAGCTACAGATGGTGATGAGGTACA
GCTGCAGATCAATGTCCTTCTTGATGTACTGATACAGCATCTGCTGATACAATTCAGAAGAA
ATCCTAGTAGGTGGACTCTGAAGACAGCAGAGAAAGTGACTCTGTCATATAATAAGTGGAGA
TTCTCATTTGGAGATTCTCTGACCTACCTGCAAAATCGTTGCTGTCCCACCCTGGGCACCA
GAGGCCGCAGTTCCCTCCGCTCTATGGAACGGGGGTCCTCTTTGGCCATTTCGTCCGTA

CATCCACATGGCAGACTCGTGCATTCTCGGTGGTTGGCCCTTCCCTGTGGAATGGACTTC
CGTTGGCGCAGCGATTGCTCCCCAGGGTTCATTCGGACACATTCTGCTCCAGTCTGAAAA
CTGTTCTTTTTAGCCGTGCAGGGATCGGGAGCGCTTCTGAGTAGTAATCTTGATGAGGCGC
TATATAAACCTCTGTAATGAATGAATGAACAGAATTAGAATAGAGGGATGGAGACAAGAGGG
AAGCTGAGAATGATCTGAATAAACTGGATGTTGAGGATGTGTACATCAAGCTAAAGAAGAGA
ACTGGACAAATGAGAGGAATGGCGACGTTGGACATACAAGACAAGTCTGCCTGATGAGGT
AGGTAACCAAAGAAGAAAATAATAATAAAACGTAGATGTAGTGAATCCAGTGTTTCTGTTAGA
GTCCAAACATAGAAAGGTCTTTTAGGTGCGTCCTGACCTAGAATTTTCGTCATTATGTTCGTG
TGGGGCTGATACATATCCGTTTGTATTAGCCCCTATAGTAGCAGGTATACTTTTAAGACCTGA
GCCATGGCTCCCAATTTAAACCCTTGGCTAGAAATTATCACACTTCTTTACAATTTTGATACA
CTAATGGAATTTGTCATAAACACAAAACGGCTGTGCCCTAGATTGGGCAGTTTCTGCTCTCT
CTTCCCTGGTTGTTGAAGTCTGGTTTTGACTGTGGCGGGTATATGGTTAATAACGACAGAGG
CGAGCCATTTTAATAACTCTCATGAGCAGTGGATGAGCTTGCTTAGATCTGTGTCAACCTTA
GACCACGGTCTCCTTGAAAGCAGTCCTGCTGCAGAGAAGAGAGCTGGTGCTACACAATC
ACGCTATTTGTGTTACTGTCTTGGGATGCGTAATCTATTAAGAGCTTGTGTTTGTGTCTCATGTA
GAACTGGCGGCGAAAGAAACTCTGTTTTGGAACGAGAATCCGAGAACCTTCGCTCGCAG
CTGGCGTCTGCTAACATCCGAATCAAGGTGAGAGACTGGTTTTGTTGGTTCTTGGGGTTTTG
CAACCGGGGTTTCCGGTTGGGATTTGATTCCAGGATTTGCATGAGTTGTACATCTGTGGTTT
ATATCGGGGCGAGTGACCAGGGTATGCATACAGGGTTTTGGATTGGGATGTCCTTCTAGGA
TTTGCACGAGTTATACATCTGTGGTTTTTATTGAGGCAAGTGTCTAAGATTTGTGTCTAGACTTT
ACCTTTGAGATTGGTTGGTGGGGCTTTGTGTCATGGTGGGTGGGGCTTTATGTCGTGGAATT
AGACCGTTTGATTATGGTGAATTATCAAGGTTGTATTATCAGTATCTCTTTAAATATTTATCCGCT
CTTTTACAGCTCTTGTTGTGGTAATGACTTTAAATATTTTTTCAAAATTTAACACTTTAACTGCAA
TGGTGCTTTAATAGTGACAGGAACCAAAGTTTTCTGCTGTAACACGAAGACTGAGTAGTTAA
AGATTTATATTTTGCACTTATTATTTGTATTTTGCACTTTTTATATTTTACACTGACATATTTATATTT
TGCACTTATATAATTATATGCATATAGTTCCCCTTCAAGATAACTTTTCAGAGGCGCTCCCAA
CCCAGGCTCAGCCCCAAAGAGAAATAGCTCAAACTAAGTAGATAATGGTTTGGTTGCAGG
AGTTGGAGGTGACTGTTTCCCGCTTCGAAGAAGATGGAAAGGTTGTACAGATGATGAAATC
ACAGCTTAGTCAGCTGAAGCAGTTTGAAAAAGAAAATAAGACACTCAAGGAAGAAAATCAG
TATCTCAGGTAATAATATTACCTTATGGATGTAACAAAAGGAATCAGCATTGATTGCATGGTAA
ATGTACAGGAGGCTTTCCTTTACAGGAATATGTGGAAGGTAATTCTTCAGTTGATTTTTCTTTA
GTTGTTTGCCGTTGATGGTCAGAATGTTTGGGCTCAAGACTCATCACTTGTCGCTTCCTAGA
TATTGAGCTAGAGGCCCTAATAGGAGAGAGGGGTTTACGAGTTTGCAATTCTTCAATTCAGC
TGGTGTCTGTGAATTTGTCATAAGTTGAAAAGTTGTAACTCGATTTTAATGCGGTTTGTTTTATTT
GATATAGAATGAAGCCCCTTATTGTCAAGAATACTTTCAGAATATTGAAGAACTGCAGAGGTT
TATAAGCACACAAAAAATCTTAAACTTTTTATTCCATTGTTTCACTTTTTTGATTGTTCTAATCAA
ACGAGTATAATTTCGGTTAGAAACAAGATATGTTGATCCTGTAAAAATATTTTGAAGCAGATGC
AGTATAGAATATACGGTATATGATACGGTATAATATTATACAGTATATGATTCAATAAAAAAAGTC
AAAACCGACAAGTGACGGGTTTTGAGCCCTGTGTCACATTTTGTGTCACTGGGTTATGTGG
GGCTTCAGACTAAGAGGCTTTACAACCAGAGCCAATTGCATTTGTATTGAGTGAGTTTCCTC
ATTGCTTTCAGACATGGTCTTTGACATAAAAACTAGCTACTGCTTTTGAATTGAATTTCTGCTTT
GCTGTGCACTTAATCTGAGAACTGACACTCTCTGATTTCATTTTTATTTTTATTTTAAACTTAGTC
ATAGAATTGACACTTTTGAATTTCATTTTTATTTTTACTTTAAACTTAGTCTTAGAACTTGCACGTT
TGAATCTCATTTTTATTTTAAACTCAGTCATAGAATTGACACTTTTGAATTTATATTTTATTTTAAAC
TTTTTATTTTAAACCTAGTCATAGAACTGACACTTTTGAATTTAATTTTCATTTTTACTTTAAACTTA
GTCATAGAACTGACTTTTTAATTGAATTCTGCTTTGCTGTGAACTTAATCTTAAAACTGACTTTT
GTGAATTTAATTTCCATTCTGATTTGCAACTCAATCTTAGAACTGAGACTATTTGTATATGAATTA

AATTTCTGCCGTAGTTGCTGAACTGAATACGTTCCTGTATTGGCAGCCATGGACTTAAATCT
GTGGTCTTTGCGTCTAATTTTAGTGCCATTCAAAAGAACACGCATCTTCTTACCGAAACAAA
CATTGACCTTCGATCGAAACTGGATCAAGCCCAGCAGCAGTGTGCCAAACTGTCAAAGTT
TGAAGTTCTGTGTGAAGTATGTAAGAGTTGAGCAGATTTTAATACTAAGAATGCTGTTGCAGC
ATTTAGTCTTCACAATGGCCTTTCATAGTGTTAGAATTGAGTTGTGCACATGTATCATATGAAC
ATCTGGATGCCAGACATGATACATATACGAACTTACAGACATGCATACAAATGCATACACAT
CACAGAAACATCTAAACGTGATTGTACAGTTTTGTACACATGTTCTTGTTTGCCTTTGATGAAC
TGTTGATGAATTTTCCCTGTTTTCTTATTCATGTGTCATTCGTGATTCATGGATAATTTGTAATTC
ATGGTAATATGCAACTCTTGAGGATGGGCATAGCCAGAAAATGTTCGGTGGTTTTAAGATTT
CATAGGGGTTTTGCATCGAATTTGGTTGTATTTTCCTATAGTTTTAGGCTTTTGCTGCAGTAAG
TCGTTCAATACGGTAGGTTTGAATCCGTAAAACCCCCTAAATATGCCCCTGCTCATGAGTAA
TTTGTAATTCATGGGTCATTGAATTTTTTCTTATGATCCTGTAAACCCCTCTGCGTTTGTGTCAA
AGGTGGTCTGAATAAACGAAGACTTGACTTGATTCATAATTCATTGGTGATTCATCTTGAAGT
GAAATAAGCATGCAGAATGAGACTGGTTTGGAGTTAATGTACTGGTGAATTGGTTATGCCTTG
CAGTGGCTCATTGATCAATTGTTTCATTTGTAACATGGAGTGTGGGCACTGGCGTAGACTTG
TCAAAAATATTGGGTGGGCAAACCAAAATATTGGGGGGCAGAAGGTGGTAAAAAGTGATAA
ATGCATGGGCGTTTCTCAATTATTGGGGGCATGTGCCTGGGCTGCCCCCCAAAGTTTACG
CCTGTCATCATTAAGGGAGATTGAGCTTGTCTTTACTCTGTAGTTGCTACGTAGTAGTGACTA
GAGCCTATACTAGACGGTCCATGTATGCTCTTCTGGTCTTGAGAGCTTGCGGCCTCAGACC
TTGATGATTGCCTGCTAAGTAGCAGTGCAACTAATGTAAAAAGGTCAGTTTGTTTTCCAATTTT
TTGTTTTGAAGGACCTGAAGAAAAGACTATCACAGTGGGAGAGCTTCGACGAAAGCAGCG
GCTTGAAGTCAGTTTCTAACATGTGACTTATATTTAAATACAGAGTCTTAATTCTTTTCAGATTGT
TATTGGAAATATAGAGAATTGATGTTCATGTTGTAGTGAAGATAAGGCTTGAAGTTGAAAATAA
ATGCTGTGGAGAGCCGCTGAAAGTACTATTGTGTTCAGGTGCTTGGATAGTCACTGAAAGT
GCTGTATGGTAAAATTCTTCTTAGAACCTCAAAGTCTATAATGTTCAAAATTCCATTTAAATGG
ATTGTTCAACGAGTGCATGTGATGCAATTTAAGTTATAGGCTTTCTGTAGCAATACATACTGTA
GCATTACATAATACAGTGATTGAGTATGTCTCTTTATGATATCTCAGATCTCCCTCTCAGATCC
GTCGGCACACTGCTGAACTGCAGCGAGACCTTGCGCAGCTGCTGGGGAAAAATGGTCA
GCTACAGGCAAGGTAAGCAAGCATTGTCAAATTACACAAAACTCCAATTGTGCTTCAAGCG
TCTTAGTTAACATGCGCTCTCCTCGCTGTTCCTTGTATGCATACTGGTTACAGCTCAACCCA
GGAGCTTTGCGTATGTTCAACCCACCAGCTGGTATTGTCTTTTTCCGAGAGCTGTGACTCG
AGAGCCTACGTTTGTCCCTTACGCTTCTGACTTGCATCTGAAGTCTGTATCATATCCTGACA
GTGATTTCAGTGTTTCAAATCTATAACACCAATAATGAGTTTTTCCTTGCAGCGGCGCTACAT
AAATGTCCGCTTGCAATTACATGTAGGGTCTTGCAATTACAGTTAATGACGTACAATTAGGTA
GGGTCTTGCAATTACAGTTAATGTTGTACAAGTAGGGTCTTCCATTTAGAGATAGGGTCTTGC
AATGACACGTAGGGTCTTACAATTACATGTAAGGTCTTACAATTACATATAGGGTCATGCAGC
AAACCCTCTTGTAACTCATAGTCAGTTGACAATTTTATTTTGAATTTATTTAATTGCTGTGGTGC
CTGTGTTGTACAACTGTGTCCACATGATTTTGGTCTGTTTAAGTAACTCTTTTTATAGTCGAGC
CTCAAACGTATACTGACCTTCTGAACGGTAGGGCTGTCAACGTTTACACGGTTAACCGACC
GATGGTAGCTAAACGGATTATAAGTATCAAGTACGGTTAGTCGCCAGTTCAATTTCAAAGGC
GGGAATGCCGTACACTAAGCACTAGAACTTGTGTTCTGTTCGTTATAGTTTGGCTCAGCCAT
TGTTTAGTTTCAATTGTCCTATGTCAATTGCAGTGAGTAGGTCTGGAGAGTGGGGTGCACTTT
AGTCTATAGGCCGAGGCCAACATCTTGGCTGAGCCGACCAGATAAATTTGGGAGGGGAA
ATATTATTGTCCAGAGACTGGGTCACGTCGACAAGGCTGACTGTCTGAGTCTGCTTGCATCT
GTCTGCGTCTGCCTGAATATTGTTTGCGCCGGTCTTAATTCTTTTGATCTGCTGTTTTCCAAA
GTGTGGGAACAACTCAAAAGAAGCCCTCCCCCCTCTCTAATAGGACAGTTATCGGAGTCG
GCCTAAAACGGCCTTCTGTAGCGGCTATAGTGTACATATGGAATGACTTCATTCACATAGGG

CCTTTAAATTGTGTCTGGCAAAGGCGACAGGACCTTCCCTGGTCTACATGACTGTAGGCCT
ACGTGTACACGGTTAATCGATCCTGCCTAGAGCGGTTACCCGGGTGGGTCTTTTGGCAAC
TTTTGACAGCCCTACTGAACGGTCCACTGGAGAATTTCATGCTGCTGGGTCTTTTCTCACCT
TAATGTTGTTACCTACAAAGCTCTGATTTTTCTGTTTCTTTCAAACTGCTACTCGAATACATGA
GCAAAAGCTTAATACTAGATAATATGCAATGAACACAAGTTGATGGGAGCCAAGGGCACTA
AATGAAGAGAGAGCGAGATTGAAATAATTGTTGTGGAAAGAACATGAGATTTGGAGAAGCTA
CAGTTTGACTGTTAGGCAGTATTCGTCTATTTCAAAAGAATAAGAGTGCACATTCGTGACTTA
GTTTAAAAATTGACATCATAACAGAAGAAAAACTGGACCCAGCTGGGCTCATCCATAGTATT
TGTGCATACTGAAATAACTTAGAAGCTACCTTGAGCATTATCATAGCAGCAGTCAGTGAGATT
GTGGTTAGGCTAGTTCTTCAGATTATGGTGATGTTTACTGGTAAATGATCATGGCTTATTATAGA
CACACAAACACAGTATATATACAGTCACACATACAGACACACATGCATGCACGCACACATA
TAAACATGCTTGCAAACACATATGCCCATGCCTACTGATTGTTGTGTTTGCTTTGAGTTTTCTA
AACGCTTTGCTGATGCTAATATCTTCAGCAACCACGTTCTGGAGGACACGTGTAGGGCTTC
AGTGGCTGAGCTGCAGAGCACGAAGGCCGAGTTGGCCACAGCTTCTGCCAAACTGAAG
GAATCTGAAGGATTGGTTTCACGACTACGAAAGAAATTGATTTTTGTTAGTAAGGTAAGTTCA
CGTATCTGCTGTTCTGGGCAGTCGCATGTAATTTGGAATTGATTATTGCATTCTGTTGCTTGG
GGTCCCAACAATAGCGTTTTTGGCAAACCGGATTTCAGTTGCTGAAAACCGGTTTGAAACC
TGTTTTACAGGCCGTCATAGAAATTTAAGTTTCAAGCTCAGTTAAACAAAGGGGTTGGGGTA
CCAACCAGACCCCTGTGTTTAACTGAGCTTGAAAATTAAATTTCTATGAAGGCCTGTAAAAC
TGGTTTCGAACAGGTTTTCAGCAACTGAAATCCGGTTTCAAACCGGTTTTCAGGAAATGAA
ATCCGGTTTGCCAAAAACGCTATTAACATACCTAAATCCTACCCTACGTTACCCAATAGATG
TTTCGCAAAGAAGGTTGTTTGTTGTTGTTAGGAGAAAGAACTTTTCAAGAACATCGTCGACG
AGTACAATCGGCACGACGACGACATGACGTCTCGCGTTGACACTCGATCCAGTGCACGG
ATCCAACACTTGGAGACATTGGTGGAAAGGCACCAGAGGCACATCTCCACTCTGGAGTCT
GATATAGAAGAAGCAGCACAGGGGAAGGCACAACTGCGGATGAGAGTCACTCTGGTAGG
TCAGAAATTTATGAGATAACTGGAAAAACGATAGAGGAAGAATTATGAAATGGATATGATGTG
TTAGCGACTTCAGTTTCATAGACACGATATACAAAGGCCGGTTACCAGTCCTCTTCCTAGC
CGAGATGGATTTGTTAATTTTTTAAATTTTACTGAAAACAATAATATTATAATTTATGACATTTAATA
TGGTAATATTTGTCTTCGTTTATTAATTCAAACTGACAGGAGTCAAATTCAACTGTGCAGCAG
CTTTGGCTGAAGCTATCTCTGCTTCCTCTCTCAACTTCCGTTACATGAATGTAGTCTGAAAGG
AATTTGGGGGTTGACATTTTGGGGGATTTTGCATTGAATTTGGTCGTATTTTCCTACAGTTTTG
GGCTTTTATTACAGTAGAGCATTCATTTCAGAGAGGTTTGAACCCATAAAACTGCCCCCTAA
ATATGCCCAATTTTTTGCCCATGCCTCAAACAGTGTACAATATTAGTGCTTTACTGATAAGGA
AGTAACATCGGAGCCGGATTAAGTTTTTAGAGGCCCGTTTCAAAAATAAATTAGTAGGCCTT
TCAAAAGCCTATTTACCACCGAAAATTTGAAAATTTATGAAAATGACCTTTTTTACTTGTCACT
GACAAACAGTTTTTAATCCAAAATCAATCTCAAGGTAGGCCCCTAATTTTATCGACCAAAATT
TCCTATGCCTTTTTACCTTTTTTGTCATTTACTACAAAAAATCTTATACTTTTTCCTCTATTTCTAT
TTTACCTGTTACAAATGCCATAATGACTCTTAAGGTATGTCCCTAAATTGTTAATTTTCACTAGG
CCCGTTTCAATTGAAACATTTGCAACATGATTAATCCGGCCCTGGGTAACATTCATAATCGG
GCCAATTATTACAACAATCGGATCCAGCCCGGTGATGATTTTCAAGATGAGGATACTCACT
GAAATTCGGCATTAAGCAGAGATTCAGACTGCAATAGGTGCTCATCTATTTATTCGTCTAAG
CATTGAGTCTTGACAGACCTAGAATTATACTTGAAGAGTGATGTGTGTGGTGAGTTCTGTTAAT
AATGACTGATTTTTCCCATCAGCTGGAGCAGAGGTTACTGAAGCAGACAGACAAGGAGCC
TGTTGCCGTATCAACAACAAATGCTGCTGACCAGAATATCATTCTTGGACTCAGGTAAGACA
CCCTCTATTCTTGTACATAGGATCCTTTAGGTAAAATACATTCTTGGACTTAGGTACCTGGACT
TGAACACAACTGCGGATGAGGGTCGCTCTGACTGTGGTCTTGGCTATATTCTTGTAAATGG
GTTCCTTTAGATGATATATAATTTGTTATATCGTTGCTTATCTTTGCGTATCATTGAACAGTTACT

GAGGTGAAAATAACAAATCCTGGAGTCTGGTGCTGTCGTTTTGGTGCAAATTTTGCTACAAA
TTTATTTTACTGTTCTCTTTGTTGTTGACCTTGCTAGGCAGAAAGTGGAGGACCTGGAGAAGA
AAGCAGAAGAGCTGAGTGAACAGAAAGAGGAAGCGCTTGCTCAGCTGGAGAGAGTGGAT
CTTCAGGTGGGAAGACTCTGCTTAGATAACTTTAATAAAATCACTTTCCAGCATTTCACTTAT
GTAGTTACGCCTAAAGTGGAGTAATAATTAATAAAAGTAAGTTGTTGAATGGGTGACTAGTGC
AGCAATTAAAGTCAGGGACATAACCATAAAAGATTTGGGGGGCTGGGTTTTGGGAGGCTTT
TCCATTGATTTTGGACGTAATTTTTATGCTTTTATAACAGGAACGGATTCATTTGGGGAGAGG
GTTGAACTCGGAAACCCCGTAAATATGCCCATGGTTAGAGTTAATAATAATTATTAGTTAATTA
AGTGGAGGAAGATAGTTAACTTACTATTCACTATTCAGTCATTTAAACTATTATTAAATAAATGT
CTAACGCGTGCAAGGTCTGTGAATCTGAGCCATGGAACTATTTGCTCTTCTCTTCTTTCGAC
GAAGTTGCATGTTCTTTCTGTTATTCTCTCCAGTTGTGGATTGTTTGTCGAATCTTGAAAGGCA
ATATGATCGGATAGCACGTGCATCACCGATGTTTGTTTTACTAATTAGGGTGGTTACGACCG
ATCAAAGACGAGCGTCTATCACATGCGAATGAATCCGTATTCGATGACCCGTGATCGTCAG
ACGCAGGAGATCGATCGATTGAGAGAGGAAAATGCCCGTCTGAGCGCCAAACTCAATCT
GATGAAAGAATCGGGCGGACCGGTCGAAGGTCTCTCGGTCATCGTCGACGAGAACCTTT
GCCCACCAGGAACTGCGAAAGAACTCGAAGGTTTACAATGGAGCTTGATAAATAAAAATAT
TAATACATTTTTTAGTGGTGTTTTATAATAATCAATTTGTAATACAAAGTATTTTATGAAATGCAAA
AAAGGTTTTCTTTTTTAATGAGAACCTCTCTGCCCACCAGGAACTGCAAAAGAACTTAAAGG
TTTACAACAGACCTTGATAAATAAAAATATTAATATACATTTTAAGATATTATTTTGATAATGTTTAA
TTCTGTAATAGTTAGTTTTTAATATAAGCTATTTTATGAAATGTGAAGATTATTTTTGTCGATAAGA
ATCTCACAGTCCACCAGGAATTGCAAAAGAACTCTAACATAATAATACTGATCATAATAATAC
TGGTGTTGCGCGGTGGCCCAGTGGTTAGCGTGCTGGACCGTCAACCGTGAGGTTCGGG
GTTCAAATCCCGGCCAGGGCAGAAATTTAGTTCGAGATTTCTGCTCCACCTGCGCCCCCT
AGCCAACTCGGCTATGATGAGTTAACTTACCGCACACTGTCAGTGGGAAGATAAGACGGT
GAGGGCGAGGACGCCACTAGCCCTCATATGGCGAGGCTAAGAAAATGAAGTCGCTAACA
CTTCATACCCATGGCTGATCTAGGGCTACCTTAAGGGACTGATCTTCTTCTTCTTCATAATAA
TACTGATCATAATCAGTGACTGATCTGCTTGAGTTTGAGCAATGTGAAACTGTGTCCATTTGA
AACAGAGACAAAGAGCCAACTGGTGAGCGAGCAGCTAAAGAACCAGCGTCTGATGGAG
GCGTTTAAGAAGACGAGCCAAGAAATGCGCGAGATCAACTACAAGGTGCTTGGATATCGC
GTTGACATACCGAACACAAACGAATATCGTCTGATGAGCATGTATGCGGAGTCACCGAATG
ACTTCTTCCTCTTCAAGGTTAGCATTAGAGACTCCGTGCAATTAATTATTATTAATTACTTATGA
TTAACTAACAACTGTTTAGCTCTGTGATGGGCCTCGGTCTGATTCATTAAGCATTTAAGCATA
GGCGGAGACTTGGCCCTGGGTTTGGGGGGCCAAAAAGAATCAAATGGCTAGCGTTTCTA
GATTCAAGATGACCTTTTTTAGTCATGTCATCATTCGAACGGTACTTTGCGGAAATGCAATAA
CTGTCGAAACACTGTGGACGTTTTTGATGATATAAAGTATTTCGGAAATATTGAGGGGATGTA
AGCAAGGACCGTCCCCCCACCTCAAATTTAGCAGGGGACCGTCCCCCTAAGTCTCCAC
CCTGCATTTAAGTGCAGTTGATTGAATTCAATGGCCTCGCCCGCACAGAGCGTTTGTCCAG
TATTTCTTCTAAATGTCTCTTCCGTGCAGGTTCCACTGGATTCTCACTATCATAGTACAATCTT
AGCAGTGACTTACATGTGACTTCTCAAATGATGACTCCTTCCATTCAGCCTTTACTCTGCACT
GCTCAGCTCTCCCGTTCCCTATGCATGTGTGCATTATCAGTAGACACCTGATACGCAAGCA
CGTGGGCGTATTTAAGGGGTCTCCGTGTTCAAACCCCCTCTGAAATGAATTCGTTCCGGTT
AAAAAGGCCTAAAAGTGCATAAAAATGCACCCAAAATCAATGGAGCCCTCAACAAAAATC
CAAACCCCCCAAATTTTTTTCTATCTACGTCCCACGCAAGCATTTTCACTGGATGCTTCCC
CTACATGCGTACAAGCTAAATGGATGGCCCAAGTGTATAGTGGATGGTTCTAAATTTAAGTC
ATCTTATTATTCTACATCCTTGTTTCAAGTAGGTAGTTAGTTACATGAGATCCATCCGCTAGATG
GCATCATAGCGAGTCTCCTGTACATTATGAGCTTGGTTTGATTTTCTCAGTGGTTTCTTGTAGC
AATGTTCGAACGGTGAACTGCAACTGCTTGCCAACAAATATTCCGAGTCATTCGAGAAGAA

CGTTGAAACCTATTTGTCGATGGGCAACAGCTTACCCGCATTTATGAGCAGCGTCACTCTC
GAACTGCACAGTCGTTCTACCATGATGCAGTCATAATGAAGGACTTTACGTTGAGTTCAGTT
TTCTGGCGGTTGCGGACTTGGTTGCTGGGTCCAGACTCGGTCGGAATCTTTTGAAACAGTT
GCCTTCTCCTGGGTCGATTACTCCCAGCTGAAGTGTTTTTGGAACAGTGTGTCTTGTCTGCT
GTTAATTGGTAAATTCTCTGGTAAGGAATGGGAGTGAAACAACCAAGTTGCTTTCTGGTGTTC
TCTGATCTTTTTTGACTTTTACACATGTAGTTGTAGTTTATGGTAGTTTGACTGTTATCAGGGGT
GTGTAGTACGTGTGAAGCGTGATACAAAAGTGCACTATTGTCAAAATAGAGGGAAGCAAAA
GAACATAAATTAGGCAAAAAAATGGGGAAATGTACAAATTTTGTGGAAATAGGGGAAAATTTG
TAAACTTTGTGGAAATAGGGTGGAATATGCAATATGCATCATTGGCCTAGGGGGATGGACA
CTTCTGACAGTTACTTCACTGTCTTAGAGCTGCAATTATGAGCTACTTTGGTTTTCCAGTCGC
TTTTAGTAGACTTGTTATTAAAGTGTCAAAAGTCATACTTTATGTACATACATTCATTATTTCATTC
ATTCCGGCCATTTCTATAGCACCCCTTCAAGTCCTCTACTACTCAGAGGCGCTCCCGAGTA
CAGCACGGATACTGTATCGGAGTTTCGCGCCGAAGCGCACAGGCAACTGCAGGTAAAG
GACTTGTCCAAGGTCCCTACGTGGCGGCTAGAGCGGGAGTCGAACCCACGACCTTCCG
GTTGAAAGTCATCGTCTCAACCAAGGCGCCGCCACGTCCCACACCCCACATTCTAATCAT
TGTACCTTCGCGTGGCTTTGTTTAGTGTATGACATGATGGTGACAGTTCAAAGGGGTATTTTG
GTAGTGGAACAGAAAATAAATTGCTTTTACTGGCATGACCTCTGCATGCTGCGTACTGTATAT
GGTTTTAAAATTATTGGTCGTTGTTACCTCTGCGAATAAACTGATAGTGACAGTAGCTTGTGAA
CATTTGTTATGTCTGGTAAGATATGAACTTGTACAAAGAGACGCAGGCCGGATGTGTTGATC
AGTGCCCTTTGTACTACATGTTGTAGCCATTCGATTTCAATAAAAAATGAAACAAAACAATTGT
GTTCTGTTTACTGGAATCAGTTGTTTGGCTCCTTTTAGTCGTTGTTAAACAGTAACTTCGGCTT
CCTGTCGTCCGTGTCATTTCAACACCCTGCATCCACTCAGCGGCGACCATCCCCCTAAGT
CAACGATGCATATTTCCCCCATTTCCAAAAAATTATAAATTTCCCCCAATTTTTGCAAAATTAA
AAAGTGTCTCCCTATTTTCTTTCAATTTAGGTTTTTCTGCTTCCACCCTGCATGTATTTTGACC
ATGATGCATTTACACGAACTGGACGCCCCTGTATCCACTTGGAATAACCTCCCATCTGATTT
GTGTGTCCTCTTAGCCAGAAACTGTATACACCTTTTACAAGCGTCTGGAAACTGTTATTTCTT
GCTGTAGTTGAGCTGAAAGTGCCTTTGTGTAGGATGGGTTATCTAAGAAGTGTGCTATATAAG
TTCTCTGAATTAACTAATCTTGAGTTGTGCCCTATCCAGGGCAGCGTATTCCACACTGTATAT
AGGGTCATTGAGGAAATTGACTTTTGGGTCCTTCCCTTATCCCACATCTGGTAATCGTAAAC
TCCAGACCTACCAATCTGGCTTCAGAGCAAACCATTCTACCGAAACGGCTCTACTGTCTCT
TCTCTCTGACATTTTCTCTGCTACTGACAAATCGCAGCTGTCCTTATTGGCCCTGTTTGATGT
ATCTGCTGCTTTCAACATGGTCGATCACCAAATCCTTCTTGAGCGTCTTGAGACCTCGTGTG
GAATCTCATCTCTCCCTCTTCTTTGGATTAAATCTTACCTCTCTGACCGTACTCAAATGATTAT
CTCAGATGAATCCAGGACTTCCTGGGTTCCTATATTTTTGGGTGTCCCCCAGGGCTCTGTC
CTGGGTCCTCTTCTGTTCATCTTATATACTGCTGACATTCCCACTCTCTTTTCAAAATATTCGG
CTACTGGTCATCTCTTTGCTGACGACGTTCAGGCATAATGTTCATATGGTCCTCCTTCTAGTC
AACTTCTTCTCGCCAGCAAAATTGAACTACTCTCAAATGATCTTAACTCTTGGATGTCCTCAA
ATAGACTCTCTCTGAACTCCGCTAAAACCCAGCTCATCTGGTTCGGCACACCTCAACAAC
TCCTTAAATTAGATCATGCTTTGCTTTCTGACCGTTTTCCACACTTTACCTTTCATACTACTGTT
CGTGACTTGGATGTCACTCTGGACTCTGCCTTGACCTTTTCGCAGCACATTTCCAACTTAA
CTCGCTCCTCTTACTTCCAACTGAGGCGTCTGAGAACCATTCGCAAAGTTGTATCTGTTCCT
ATTTTCACCTCCATTGTTCACGCATTTGTATGCTCCAGGATTGACTATTGTAATTCTCTACTGAT
CGGTCTTCCTAAGACCCGGCTATCTCCCCTTCAGACTGTACTGAACGCATCTGCACGACT
CATTGCCAGACTTCCCCGTTACTTCCATATCTCCAACTACATCAAGGAACATCTCCTTTGGC
TTCCAATCTCTGCGCGCATTGAATAATTATAAAGTTCTGATTATTGTTCTTAAGGCCCAAATGG
GGGTGGCACCTAAATATCTCCGTGACGCCATCCGACTTCCGACCTCTGCCACATCCCTTC
GTCCTCTACGTTCCATGGACAGGCGGGAGCTTTTTGTCCCTCGGACTAGGACAACCATGG

CCATGTCTAGATCTTTTGCCGTTATTGCCCCTTCTCTTTGGAATCGCCTTCCACCTTCCGCT
CGTGCTTCTCTCCTATCATCCAATCTTTCTATTTCTATTCGTTTATGAATAAACGAATATTGATTG
ATTGATTGATTCTACGTCCTTATCTCTTCTTAAAACTTGTCTCTTTTCTTGGAGCTAATCGAACC
ACAAGCGCCTCTGTTTGCCTATGGCTGTTGAGGGGCGCTATATAAATACTTCAATACAATAC
AATGTATTTCTAGAACACCCACAAAAGCGAAGTCACGGGAACCAGCTTATTCACAGGCGC
TTGACCAGAAAAAAATTGATAGGCAGCGGTCAAAATCCAGAGAGGCAGGGAGACAGACG
GCTATGGAGTTTGGAGTTGAGACGAGGTGGGAGGTACGGGGAAGAGGACGAACCAGGAT
AGGATATGCTAAAGAGCAGTGCGCCCATTTGGCATCAACCTCCCAATTGCTCAGCATTGG
ATTGATTCCTCCGTGTCCTCTATACGCCCTTATACCTTCCTTATCTATACCGATAGGTCACAT
GACAAGTTGAATAGCATGTTATGTAATTATGATATTATGTGATTGATGTTTTGTTAATATCACCAC
AGTGCATTTGGAATAGCCTATTAGTACCAATAAAGTAAGTTATAATAAGCAAAACACATATATC
AAAGAATTCAATTATGAATTGAATTGTGCTTTATTGAAATTTAGATTCCTATGAGCGTTGTCAAT
AATGTCGAATTATTTGTAAAATAGTAAAAAAAGAAAAACTTCTTTATAGCGCACGGTTTTAGTTA
TATGACGTCATCAAGTGTGAATTCATAACATATTATCTTCAGTGTTGTCCATGTTGGCGGGATT
TGTATATTATAATCATATGTCTCGGTTGAATTTTTTGTAAATAAACGAGGTAAACCAAATTTATCA
TTAACAACGCTTATACTTAAATCGGAACTTTCAACATGCACGTGATTTTCGTACCAGCCAGC
CCGCACTTAGAAACCTGTCGTTATTGAGTGCATTACCCCGTACTGGATTCCTGAGGTCGGC
TTTGACAGACTCATAGACCAAAGTAAACACGGGGTAAAGTTGACTAATCCGTGCCCGCCC
TATAAACGGTATGGCCACCCACGAATGCGGTCAAATAGAGCGACGCTCTTAGCTACCATT
CAACTCCCGTCATGCAATCCGGTCAGTAAGTAAGCAAGCAAATAGATATCATATTGTTAGGA
AAAGCAAAAGGTAATGACATATTATTGTTAAATGAAACAACTAAGACAAAAACAAATAAACAA
CATCATCATTAATAGCGCTTATAATAGACTAAGCTAATAGTAAGAACAAATGTCAGTACGAAAT
TTGAAGTGGGATTGATGAAAAATACCGTGTAAGTGGAAGCGGGATTGATGAAAATCACCTTG
AAATTTGAAACGGGATTGATGAAAAAATACCGTGTAAGTGGAAGCGGGATTGATGAAAAATA
CCGTATAAGTGGAAGCGGGATTGAGGAAAATATACTGTGTAAGTGGAAGCGGGATTGATGA
AAGTCACCTTGAAATTTGAAGCGGGATTGATGAAAAAATAATGTGTAAGTGGAAGCGGGATT
GATGAAAATCACCTTGACATTTGAAGCGGGATTAGTGAAAAAATACCGTGTAAGTGGAAGC
GGGATTGATGAAAATCACCTTGACATTTGAAGCGGGATTGATGAAAAAATACCGTGTAAGTG
GAAGCGGGATTGATGAAAATCATCACATCAATTGAAGCGGAACTGATGAAGTTAAGGTATC
CCGCTATTAACGGCCGTAGCGGCTAGCCAATGCCTCCGCTATTAACAGCCTCAGCGGC
CGTCCAGCAAACTATTAATGGCCGGAGCAGCAGGAGCACTGTTTAGTCACTCCCGTATTC
CTGTGCCCAGTGTACCGCGTGCGACTGTGCGTACATTAAAACTTAAAAAAAACCTAAATCT
ATTTTTTAAAAACCTTAGGTTTTTACCAGCCCTGGAATAAACACACATTATTTATCGTCTTTATT
TGCTTACTTATTGACCGTATTGCATGACGGGAGTTAACTGGTAGCTATGAGCGTCGCTCTATT
TGACCGCATTCGCGGGTGGCCATACTGTTTATAGGGCGGGCAAGGATTAGTCAACTTTAC
CAAACACGGAGACACATTGATGTGACTAAACAGTGCTCCGGCTGTACATAGCGGATGCAG
GCCATTTTTGGCCCGTCTAAATGGCCCGTCTGGCTGGGGTCTATACGGGCCATAAGTGGC
CCGCCAAACAGCCAAACAGCAAACAGGCGTCGCTCCGCAGGGCCGCTACATTACCGC
ACATGGCCCGTATATGGTCCGCCTAATTAATGCGGCACGGGCCGTTTTTGGCCCATCTGG
TATTTTCAAATGAGAAGGCCTGAGCGGAGGCATAGGCTAGCGGGACGGCCGCTAACAGC
GGAGACATAGGCTTGCTGTACCGCGTGCACACAGGCTAGCTGGTTTATGATAGGCAGTGG
CTATCGTACGGGCACCCGTACGCTAAAAGCAGTTGCCAGCGTACGGGCACCCATACATT
AAATGAATATAATAACATCTATAGTCTATGAGAGACGCTTTATTTTCATCAATTTCGACTTCTGTT
TAATCGGGCTTCTATTTAAGCAGAATTCTTTTCATTAATCCAGCCTCTACTTAATATTTAGCAAA
TACAATCCCTCATGTATAACTTATGCGATCATCAGTTGGGCACTAACGAGCTAATTAAATGCG
TCACGTGGGCTATGCGTCGTTTAGCATTAGGCCCGTCCACTGGACTTTACGGCCGCAGG
ACTTTGAAACATGCTGCCCGTACGCTAGCAACTTCCTTTTAGCGTACGCTAGTCACTGCTTT

TATGATAGGCCTACCCATGTTATCAAGCGATATCGATGAAAATATTATATTGTCATTGCAATCG
GTATTGATGAAGAAATTACGGTTTAAGTGTGGTTGAGGAAAAAGGCATCGTGTAAATGGAAG
CGAGATTGCTGAAAAATTACCATGTAAGTTGATGCGGGATCGATAAAAAAAATTACCGTCTA
AATGGAAGTGGAATTGATGAAAAATCATCGTTTAAGTTGAAGGGTGATTGATGAAAAATTACA
GTGTAAGTGGAAGCAAGATTGATGAAGTTAAGATCTCCCACTATTAACGGCCGGAGCACTG
TTCAGTCACACCCGATACGCATTGCTTCTGTTTACGGTAAGTTCACTGCGCACCCCATAAC
CCGATTCCCCGAATCTATCAAACATCGAACCCTGAGACTACAGGCAGAAACTATATCCGA
AGAATTTTTGCACAGGTTTAGTCGATTTGAATAGTAATAATATGTAAACATGTAAACATGGCAG
GCAACTAGCTAAGAATGATGTGCCATCATATATCAATGGTGCAATATGTGTTCTTCATGGTAGT
AAGGAACACACATGTGACACAGACCCAAAAGTCAATTTCCTCAACGACCCTATATACAGTG
TGGAATACGCTGCCCTCTATAGGGCACAACTCAAGATAAGTTATAATAAGTTATAATATATCAA
AACAAACTAACTCAAACCCGTCATAATGTTGTTTTTGGCTTTTTTCTTCAGTCATATTCTATTCC
TACTCTGCATCTGCTTCAAAATGCTTTTTACAGAATCATAATATATTGTTTCTAACTGAGGTTATA
CTGGTTTCATTAGAACAATCAAACAACTGAAAAAAATTGAATAAAAGTTTATGATTCTTCGTAT
CATCATAATCCTCTGCAACTTTATTCTTCTGAAATGATTCTGGGCAATAAAGGTATCAAATAAA
CCAAATGGCATTAAAGTCGGGTTACAACTTTGCAACTTATAGCAAATTCACAAACACTGGTC
AGCTGAATTGAAGAATTGCAAACTCGAAAACACCTCTCTCCGATTTGGGCCTCTAGCTTATA
TATCCAGGAAGCGACGAGTTTTAAGCCCTTGCGTCACATATGCAGTATTTTGAACACCAGT
CTTTTAAACGATGTTGCTGCTGTGCGATAGGAATAAACATAAATGGCTTATAAGAAGCTTACA
GTATTTGTTGATAGAAAAATTATAGTTATTCAGTATTGTTTTGACTGTTTCAGTGTTAGAATGTCTT
TGCGTAAGAGTGATACAAAAGAAATCGAAGATATTATTGGATCTATCAAGCAAAACTTTGAGT
GTCCTATTTGGTATGCTCGTCTTTCTTTTTCTGCCTGCTGTGCTCGTTTAATTGTTTTGCATCAA
TTGGCAGATTGCCAATAATTATGACTAATGGAGCTTGCTTTACAAACATGTTTGATTCTGTTGT
TGGCATATTTAAATTTGATTTTTCATGCACGACATTAATATGTCTTCACGTTCTCCAGAAAATGT
ACATGAATACGTATAACTATATACGCAGTGAGACACTTTTACATAGTGCATCGTAAGTACATC
GCAATGCACTGTGTGCATCACTACCTAGTTGATCCCTGTAAATGCATTTTATGATGGTTTAAA
GTAGTTTAAGAACAGAGTCTACCACTGAGGGATTCAAATGCTGTAGTTGTTTCTGTGCTATTA
ATGGGAGAGACCATATTGCATTTTACTCTGTAATGTAATATTGTAATGGATAGATATGCATAAGA
ATTTGGGCTGTCTTGAATAGTTACTTAATAACTAGTGTTTGTTATTGTAATTACATACATTTGATTC
TTTGTCTGCCCACCCCAAAGAGCCAGAAGAGACTTGACGATGAAGGCATGTTTCCATGCA
CTGAACATAGTTATCGTAATCTCAAGATTCATAAAACGCTACTCAAAAGCTAAGTGCACCAG
GGCACCAGATCACGAGCGCTGCGACGAATCAAAAGGGGGTTTCCAAAGGGGGTGGTGG
TTATGCACAGCATGGAAATGAGCCCCAGAGACACACTGTCTATATGGTGTATTAAGATCCTT
ATAAATATTAAATAGCTAATTACTGTTAATTAGCTATTACTTAATTAGCTATTACTGTTAATTAGCT
ATTACTATTAGCTAATTTACGATCAAAATTTGTATTGGATATCGATGAATATCGATATCAGCTCT
CTTATCAGCTCTCTATAGAAATATTGTTTGAACAGTAATGGATCACGCTTTATTTACACTTTTTA
AGAGCCATTCGATATTCGATAATAGAATGAGGCAACACAGACGTACTAATTGTTTTCGACCT
CTGCGAGCTCTGCCACCCTCTCTCCTCATGTGCAGGCCGTCGTACACTCCGGTCCTCTG
CTCACGGTAATTTGGTGGGTGCGAAAGAGCAGACCCGTTCATTTTCTGTGTTTGGTCCAAA
AACCTGGAATGGACTTCCAGTAGATCTAAGGCACCTTCCAAATGCTGCCTGTTCTCAATTCT
ACCACCTTCTAAAGACTGTTCTTTTCCGCTTTGCCTGGGTCGGGACCGCCTCTGAATAGGT
ATCTTGAAGGGGCGCTATATAAATTTTGATTGATTGATTGATTTCAGTGTACTCTATTCGCTTCC
GTTTCTAATTTTTCAGTCTTGAATTGATGCTTAATCCAGTGTCAACAAAATGCGATCACAAATT
CTGTCGGTGAGTTTGAATAACGTTTTTTGCTTAAATTGTACGCTCGGTTTTATGCATAATGATA
GAGCTGAAAGACATTAGGCAGTAGACGTGCAGTAGTGAACCCTAACATTATTTAGGCCTGA
GTAGAAATACCTTGAATTGACTTGACACATCTGCAGACTTCTGGCATTTTTCACAGTTCCTAC
CTAGATTGTGGTGCAGCCACCATGTCTCTAGACATACCATGCTATCCTAGAGGATGCTTGAA

GAACTCATGCTGTTAATGTGCTTACAGTTTAGTTTTATGTGAATTCCTAATTGATCCCAGTATAT
CTTATAATCGTAATCGTAAACTCGATATTTCTACAGCGCCCACAAAAGCAAAGTCGCGGGA
TCCAGCTTATTCACAGGCGCTTGTCCAAAACAAAATCGATAGGCAGCAGGTCAGATCCAG
AGAGTCAGACGGCTATGATGGCTGGTGTTTGGAGTTGAGACGGGGAGGGAAGTAGGAAG
AAGAGGATAAATCAGGATAGGATTTATTGAAGAGAAGTGTTTTCAGTTTGGAGTAAAAGAGCT
GTGGAGAGATAGCAAGGGGTGAGGTCTTGGTGAGTTCGTAGGATGGGTCAGATAGTTGTC
GCAATGCTGGCGGGAGTTTGTTTATAATTGTATACTTAATGTAGTTATAATATTGGTTTGTTTTTC
TTCAGTCACTGCATCTTAAAGGTCATTGGTCGAAAGCCTTCAACTCCGTGTCCTCTATGCAA
GGCTCCTGTTACGAAAAGGTTTGCCACAGCTAGTTATTTCGTTAGGGTCCAAAGAGGACGT
CTATGTAACATACTGGCCTAGAATTTTTGATGTAGCCCATTGCTAACGGCAATGACACTGAA
ACATTTGGACGTTTTGGACGCATTTACAGATGCAAAAACGTCTACTTGGGCTTTAATGGATA
CCCTGTCCGGAGCATGTTTTATTATGGCTTATTTTTATCTGTGTGTGTATGTTGTGCTCCTTTTG
CTTGTCTTAACGGAATTTGTAAGTCTTACCCATGGTTTGCACAGTTTTCAACTTGCTTGTCTAG
GGAATCCGTCTGGATGTAGTGTAGGTTGTCCCTAATTTATTAACCTGTTTAGTCTTGACTTTTTT
TCTACACGTGTATATTTTGTTCTTCTTTTGCTTGTTTTTATGGAAATATGGAAGTATAATGGGCTT
TTAAGATTTACAGCTTGCTTACATCTGTGGCTTTAGTGTAAGTCATCATTAACTTATTCACTGAT
TTGGTTATATTTTAGGAGTCTTCAGGATTTAGTCGAATGGAAGAATTTTGTTGGAAAAGTCAAG
CAGTTGGTTGATGCTTTTAAGCTCGATCTAGCTGAGGAGGAGGAGGTTACTAAAGAGTGCA
GGGAAAGTGCAAAGGAAGCAGGGGTACCTACTCCATCTCCAGGAAAACCTTCGGTAGTTT
ATGTACATCAGGGAGCAACGTCTACCACTCACCAAGTCAGAAAACGAAGAAGCGTTCGAT
TTAGACTAGAGGATGAAGAACTGAACAGTAGACAGGATGAAGAAGATGCGGAAGTTGAGC
AAACAAGCAGTGGCGCTGGCAGCAACAGTACTGGTGTAAAATGTCGGGAGAGTATTGAAA
TACTATCGACCACTAATAAGATGAGTTCAGTTACAAGTGGCGTTTCCAAGCAGACCAGTTGT
CGTAGAAATGACTTTGTTGAAGTTGAGAGCAATGCAAGTGTCAAGTGCAATGCCACTGAAG
TTGAAGCATCAGACGAAGAGGATGATGATAACCGCGGTCTTATAACCGGTGGCTATCAAGT
AGAAGTTGGCGTTGACATGGAAATGAGACTAATTACCGAAAATCGTAAAATCTTCAAGAGC
CGTGCCTTGTCGAAAGAGGTAAGACCAGTCGAAGAGAAATCAAGAGTTGATCAAAGTGTG
GATGATCCCTTCGCTTTCATTGGCAGCCAGCCAACACCGAAAAGAAAGAAGTCTGTGCAG
AAGCAGGTGAAGGATGTAGATGGCATTCACATGTTTGAGGATTTTAATGTGTTACCAAAGAG
AAGAAAGAGAATGTCCCTTCGTGAACGTTCAGAGCTTCGTCTACCTGCCGATAAGAAACAC
GCAGCGGAAGCGAAAGAACCAACGGCCGACGAACATTGTTTCGAAATTCAAGCTATAAGT
GACAAGTTATCAGACGCGGAAAACTATAATCTTGTGCTTAGTCAACAGTTCAGAAGCAGTGA
TGCGGAAAACATGAGGAATCAAAAGCGCATGGAACATAGCAGAGCAAAAAGAAATCTAGT
GTCTGCTGATAGTAAGATTTTGCCGTTAGCATTGGCAGAAAATCGAGATCATATGCAGAAAA
GCGGTTCGATTTTGATGCAGAAGGACGATGAGGAAGTTCTGGTACTTCCATTGACAGCTGG
TGAGACCACGAAAAGCAAAATGGTTCCACACGAGATGACACCAAATGAGACATCACCAAA
TCACACAGCACCAAATCACACAACATCAAATGAGACGTCACCAAGTCACACATCACCAAA
TCACACAGCATTAAATCACACAGCACCAAATGAGACATCACTAAATCACACAGCACCAGA
GAAGGCTATACCAAAGATCAGAGCTCCCAACAGACCGAAGCCAAAGGTAAACGAGATAG
AACAAAGTGAGACTGTACCCAACAAAATACCAAATAGGGCAAAACCAATTAGGGCATCAA
CAAAAGAGGCTTTCAAAATGGAGGCTATACCAAACAAGCTAATGCCAAGTAAGGCAGTTC
CAAGTGAGATGGTTCCAGACGAGACAGCACCAGAGCAGATGGCTCCAAATCCCAACACT
GAAAAAAATATAGGATCTTATTTAGAAAGAACAGGAACTTCCCCAAATACCGAAGTTCTGGT
AAGTCCTTTGATAGCTGGTGAGGTGATGAAAAACAGGATGGTTACACATAAGATGGCACCA
AACGAGTCGTCACCAAATGAGATATCACCAAATCATGCAGCACCAGAGAAGACCGTACCG
AAGGTCACTGCTCCAAACAGACCAACGCCGAAGGTAAACGAGATCGAACCAAGCGAGA
CCATACCCAGCAAAATACGAAATAGGGCAATACCAATTAAGGCATTACCAAAAGAGGTTTC

AACAATGGAGGTCATACCAAACAAGCTAATGCCAAGTAAGGCGATGGTTGCAGACGAGAC
AGCACCCAAAAACACAGCTCCTAGTCCCAATGTTGACAAAAGCATAGAATCTTATTTAGAA
AGAACAAGAACTTCCCCAACTACCGATGTCAGTTTCCGAGCAGATAGCGGTTCAAATTCA
GGGACATCCAAGGTTTGTGAGGCTGACAATTATGGTCATTCAAGTAACCAGACTGGTGTGG
AAGAGAACGCCGTTTGTATGCTTGAACCGGATGGAAATGTCACGGTTCTTTGTTCCGACAA
GCCTTCGAAGCGAAGAAAATGCTCCGAACTAGTTCCAGAAACAAATGACGACAGTTTCGC
TTCAAGAAATCTTCCGAAAACTAGATCGAGTTCCCGCAAAAGGAGTCCTTTTCAAACTGTTG
AGACTAAATGGCAGAAGAAATACCAGAGAAAAGTGTCTGCAAGAGACGAAAGAAAGAAAG
CCATCGAAGAGTTCAATAGACTTCAAGATGTGCCGACATCGAACCGCTGTGCAGCTTCAA
CTTCGGATTCAGGTAAACCCCTTTTTGTGTGTAACAAGTTTCAGAATTATTGTAGTAAGCTAAC
TTCGGGTGTTGTTTAAAGTTAACTCTATGTTTTATTTCTTGTAAGACTTTGTAGCTAAATTTGAAT
TTTCAGTGTGAATTTAAGATAAGATTGCTTGAATTGTTGTTTGTATATATTTGTCTGATGCAGAAA
GAATGGTTGTCAGACCACTACTCGTTTACAAATGAGAATAAATGTCAAAAATACAGTCAAAAA
TTAATGCAACTGTTGAACACAGCTGTGCGTGGCTGATAAGCCGTTGTCAAATTCAGTTGCCT
GATAAACTTGAATGGTGTTGTAAAATCTTGTCTGGGTGCTTGTAGTTGTAATTGAATTTATAAAG
CATCACTCAGAGATTCTGAGGTGAATACAAGTGAAGTTCTTTTTATTATTCTTGTGCCTTTTAAT
TGTTTTGTTATCTATTTTTTAGGTCTGATTCAGAACAAGAGTCCTATGGACAGTGAAAGTATTAC
TTTAATTGATGATGATCAGGTGCAGTGTTTAGAAGAGGAAATATCTGCTGGAGAAAGGGAAG
AACAACCAAGAGGAGAGGAAAGTGGAGAAGGGGAAGAACAACCAAGAGGAGAGGAAA
GTGGAGGAGGGGGAGAACAATCAAGAGGAGAGGAAAGTGGAGGAGGGGGAGAACAAC
CAAGAGGAAAGGAAAGTGCTGGTGGATTGGGAACCCAACCGATGAAAGAGCAGAAGAA
GATTGATAGATTGGAAGAACAATCATCGACAGTTGCCGTAGCTGTTGAATCTTCATCGCAGT
CTTCCATCTCATGTGTTCCAGACTCTGTCGAGAACGAAGTGGAAGGGAACCAGTGCTTTGA
GGAAGTGGGTGAAGCGGTCCAAGGTAAAAGTGAGACATACCCCATGTTACAGACTTTCAC
TGAGAATATAGATGAGACATTTGAATGTAAAATTGTCAAAGAGACTTTGAAGTCTGACCGGCT
GTTCTCGGAAAATCTTGGAAGCGATGTCGTCTACACGGAAAGCGGAGCCACTCCATTGG
GTCTTCCTCAAATGGTGGCAGTGCTGGAAAATTCCGGAGAAGTTGACGGTGTTCCAATACG
AGAGAATGAATGTAAAAATGCAGCGGTTGTGCCAGAGGATGTCGTGCATAAGCCAAAGGA
AAAGAGAAAGACACTTTCACTTGGAGCCAGTCCTTCTCTGGCAGGTGAAAAAATGCTCTG
CGTCAGTGAAAGTACGTTGTCAGAAGGTTCTAGAACAACAGGCAACATTTCTGCAGAGGA
GAGTCAGCCCAGAGATTCAATGGCTGCACCAGCTGACTTTCTGCTTAAGCCAAAAGCCAA
ACGAAAGAGAAAGACGTTTTCACTTGAAGCGAGTCCTCCGAGAACAGAACAGAAGATGAA
CAACTGCAGTAGTTTGCAGACAGATTCAATTGACTTTGAAGCGGTCATTGGCGAGGCGGTT
CTTGGAATGCTCGGCAAAGCAGACGATCGTGCAGACGACGATCTGTCTACACAAGAAATT
GAGATGCACGAGGCTGTTTGGAAATCTGGTCAGACAGCGAAAAGAAATGGTTGTGCTGTC
GGCGTGGCTACCTCTGCGGTTGCTAACAGCCAACGTGTGCCTGCGGTAGTGAAGACTGG
AGCGAAATTGGTACACGAGACTGAAAGTGGAAACACTGGGGTGACAGAAGAGAATGGAAT
CAAGGATAGAGTAACAGTGGAAGCGGTTAATTATGACGAAGTAGTTGCACTTGGTAATGGC
GCAGCACTTCGAGATCGTTGTTACTCTGAGTGCGTTGAGGATGGAAGTGAATGTGCCAAGA
CATTCATCAAATCTAGTTGTGCCAAGGAAGAAACACAATCGGATAGAAAGTCGGAATGCATT
GCGGTTGGAACATATGATTCGTCGTCGTCCGTGCACAGCAATAGTTACAGTCTCCTACAGC
CACCACCACCACCGTTGTGTATGGCTCAGAATGAGAACCGTGCAAATTTGCATCTCAAGG
ACTCTACTGTGACAGTTCAGCATTCTGTCAACAGCCAGCAAAGCGTGTGTCCAAACACTG
GAGGTGTGTGTCTAGACACTGGGCGTGAGTCACTACCAGATGTTGAATCGGAGCATTTCGT
CGAATCAGAAATATTGATAGGTTTAAGATCAGATGAGTTGAAAGTGGATAGCAAGCCAAAAT
CTCAGCTTGATATTTCGTCTAATAACAAAATTGATGGGGTTGACGCAGAAACAGCGAAGGT
GGGAGCGGATTTAGGAAATCGGTCAGAACTACAGAGTGACCTGGTTTTGGTTATGGAATCA

GTTCCAGAGACTGATTCCAGCTTAGGGTTACATTTGATAACAGTAAATGAGCAGAAGAACTT
AGGTGGAGAATCTTCTGATATCCAAAAGTCGCTAGAAGACCCTGCTCATCAACTATCTTCCA
CTTGTGAGCCGACATGTCAGACTGAAGAAATTGATATCCAAAAGTCGCTAGAAGACCCTGC
TCATCAACTATCTTCCACTTGTGAGCCGACATGTCAGACTGAAGAAATTGATGCCCGATCA
CCTAAAGGTGTTATACAAGATTCCAAGAAAATTGAAGTACCAATGGCCTTTGTTAACGGTAA
GATCTTAGGCTGAGGCCAAGCTGTGTACCAGTTCATATTTATAATCATAAGCATAGATTCTTAT
TTATATTTTTATTTATTTTTATTTATTTATTTATATTTATAATCATAAGCATGATTCTTATTTATATTTATTT
ATTTTTATTTATTTATTTATATTTATAATCATAAGCATGATTCTTATTGGCTTTATTGTTACACAGCAT
ATCATAACGGTCGAAGTCAAGACATTTGTTGATTGAAGGAAGCAACGGGTTCGGTGCCTTG
CGTTCGGAAGGTCACAGGTTCGAATCCCACTCTAGCTGCCACTTAGGGACCTTGGGCAA
GTCCTTCACTCGTAGTTGCCTGTAGCGTTTCGGCTTGTTAACTCCGACACAGTAGCAAGGC
TGTAGTCGGGAGTGCCTCTGGGTAGTAGTGAACTTGAAGATGCACTATAGAAATATCCGGA
ATGAATGAATGTAAGTGTTTGTGAAGTTTCCATTTAAATGTAGAGATGCAAGGAGAGTTAGGTA
CGGTTGTTGTGAGATTATTCCAGTTGCACTGTTTATAAACGTCCTGAAACATTTTGCTCTGCTT
TTAATGTGTTTTCGTTTATGTCGATTAAATGCCTTACTTCAGACGTTCGTCTAGCACGGTGTTT
CTATTTTACAAAGTTGATTCTATTTTTGCAGTTGCTGAGAAAAACGAGAGTGATGATAGTGATG
ATGAGGTCATGTCGCCAGTTGGTCGAATAAACCTGACGTCATCAGGCAACTTGTCAAGTGA
AAGTGAAATGCTCACAACTCAAGTATGTGAAAGTGAAATGCAAATAACTCAAGCAAGGTGC
TCCCTTACTGGTAGTCTGTCTATACTCTAGATAGATTGCTTCTGCATCCTTTTATTTATGTTGTTT
ACCTTTATGCTCTACATTGTGTGCTGCATAAGGATGTATTTTTTGACGGAGAAATATTTCGGTA
ATATTTACGTGTTTAATAAGATATTTCAAAAGCGACGCCAGTATGCCCGACTCGTTTCTTTCT
GCCCAAGGGGCCACTGCACATATTTAGTGATGCTAAATACATATAATTATGTGGTGCTAGGA
TGGAACAACACCTCCACTTCTTTCTTCACTCTCCTCAAACCATTACGTTAGGGTTGGTCTAG
TCGACAAAACATATTCATTCATTCATTACGGAGATTTATGTAGCGCCCCTTCAAGGTTACTGC
TCAGAAGCGCTCCCGACCCTTGCACGGCTAAAGAGAAGAGATACGGCAGATATTGTACA
GAAACATGCGGCAGGGAATGGTTACTATACCTAACATACATGTCTGAAATGGCACAAAATA
ATGTTTTCTATACTTGAAATATTTCAAAGTATTTTTTGGCCAATGTAATATTTAAAAAATACCGTC
ATAAAGGATATATTTCATTTGAAATACTTCAGTGCAACATCTCTAGTGCTGCGCTAACTATTTC
CTTTCTTTACAATTTATACTAGTTTAATGGAACCGTTTTCCGCCTGCGATCTATTTAGCGGACC
CAATACACTTCCTTAAATCTAACCTCAAACTTATCTGTTTGTTAAGGCTTATCCCGCTTGCTAG
TTGCTTGTGAGGAGCCTATGAAGTGGTCTGGCTGTATTATGGCACCATATAAATTATCATTATT
AGTATTAACTAGCTAAGCTTCAGCCATTATCTTGCATATAATCTGACCAGCTTTCCAAGATTTA
ATCACTGAAGGAGTGATCCTGCTAGTGGGACATGTGGATTTTTCTCTCCCAATTGTCGTTATT
TCTGTCAAAATTGACAGTTCTTTGATTTTGTTTCTCTTGTGACCTGTGCTTGCTGCCTCGACC
AAACTCACCCGCGTTTTATGATTTCCTCAAACCAGAATTCTTCAGCTGTGGATGGAGTGGG
AGCGCCCTCTGAGTAGGTTTCTTGAAGATGTGATATGTAGATTCTGAGAATGAATGAAAAAAT
TTGATATGTTTGACTTTGGACTTGACCAGAACGTAAACCTGCATCATCTTGACTTGGACTCAA
GGGTTTTGGGTTAACCATAACTCTGTGAACTCTATTAATTTTCCTATTTACGTCTGTAATAACTG
CAAGAAATTAACTTTACGTCCAAAATTCTAGGTCAGGATGCTAGTATGCTTCCTTTTTGGACT
CTAATGGAAACGCTGGACACAGCGCTTGGTTTAAAGCTGTAAGGTTTTTCTGATCTTCTGGT
TTTGTTTTAGCAACGTCACGATCTGGAAGTCGAAGTGAAAACCATGGAGGAAGAGATTGCA
AGACTTCAGGCTGAACTAACCAACGAAGTCAAGACGTTGACCGATAAAATCGACGTTCCT
GTCGTCACCTCGGAAATGAGAGATTCTGAACAAGAAGCAAAGAAGCCCGACCTACCAGC
GTCCCCTGGACAAAATGGACTCAACCCCAATACATGTGACTCCTTTGAAGAAGGTATCATT
GGGGAAAAACCAGAAAGCGATGGTGTAATAGTTGTACTGGATTCTTCTAACGCTGTCAGTA
GTGATCGGACGACAGTAGGGGGCGTCGTCGATGACTTAAAGACGGAACTGCGGCAGTGT
CAGTCTACTGTCGAGGATGTCGACGAGTCGAGCAGTGAAGCGGATCTCTTTGGCAGCCA

GATGTCCGACATTGTGGCTATTCCACAGACGCCGACACCAACTAAGGATAAGACTCCGC
GCACTCGATTTTCTCACCGTGGGCTGGAGAGAGATGTAGAGAGAGAGGAAAACCCACGG
TCTCCCACTCCTCCTCCGCCTCCTGTTGTTACGCGTAAAAAGGAAAAGAATAGTTGTGCTG
ATCTGGAAAGTGCAAGTGAAGCGAAACGATTAAGGTTGAGTCATGATCTAGATAACAGAGA
CGAAAATAATCGAAACGTTAACGAACGCTTTAATCCGAGCAACGGGACTAGTCTAAAATCA
GTTGAGCGACAAGGAAATTGTTCAAGGAAGGTCGTCGAAAATGTGAAACTGGCAGATGAC
GGGATTGAAGCATACCAGAGAACTCCGGCAAACAGAGGAGGTTCCTCGTCTGTTGCTACA
ACGGGTAAAAGAAGTAAAAGCGCCATCTATGCACATTCCGCCGTACTGAGGGAGTGTTCT
TCGCAGCCCTCTAAGGAGAAGAGTCACGGAAGTCCTTTCCCTCAGAAGAAAAGCTGGATT
CTTGTGGCATCTGGCATTAACCGCGCATCTGAAATGGTATTTTGTTCTACACAAATTGAATTA
CAGTGCTTTATGCCTGTGTTACCACTGTCACTCATACGACCTTATCAGCATTAAACATAGGC
CTCCGCGCCTCAGATTCACATTTTGGTGTGACCTTGCGTGCGTTATAAATTTTAATTGTATTGT
ATAGGCGGAATGTATGCATAGACGTGCATCTGAAGTTTTCTCTGTCCAAAGCAGGACGTTTG
GGCGCCGGCACCAGAAACTTTGGCAGTTATCATTCCAGCTTCTGTCTTATCAAAGCATACT
TTAATCAATCCTAATAGTATTTGAGGGGTCATAATTTTCATCAATAGAACACATATCAAGCACA
AATTCATACTCTTTATGAATCCAATTCTACTTCAGTCATTAATAAGAATTCTTTGCATGCATTCC
ACTCCTAGTTCATTTATGCTATGATTTTTTTCAATAATCCCGCTTCGGCTTTGAATATGACGATT
TTAATCAACCTGTCGCCCAAACGGCCAGCGCCCAAAAGTCTTGTACTGGCACCCTTCTCT
GTTGCCTTGCACTTCAGTAGTTGTAAGAATTACTGAAGGGCAAGGCAACAGTAACAAGAAT
TCTTTAGAATTATAAACTACCACTCGTAAGCCAAGTGCTGGGCACCAGCTTGCTCGCAGAA
TATCTTTTCTCAAGTCTCTACATAGACTACTGTGTAGAGACTTTGAGAAATAAAAACAAGCAC
CAACTTAGTGTGATAAAATATTGGTGATTTAAACTTTCAGTTTTAATGATTGTGGTGGAGTGATG
TTCGACAGTGCGTGCTGTGGATGTTTTGTGTGTTGAAGAATGATGTTTATTCCACGAAAGGC
CACTTTAGCGTGTCAATTGTTGTGCATCTTATTGTTCTTTCATTGCAGCCGCTTGTTGAAGAGT
TTGTCCGAAAACTTGGCTGTAGATTTCTCCAGAAGTTTACTAACGGCACAACCCATGTTCTG
GTTAAACCTGGTAAGTGACCTTTAACCTTTGAAGACTGAATTCAAAGGTGGTGCTCTTTTCTT
GGATGGTTACCACACCTTTGTGGATTCTGTTGAAAGCTGATTAAACTTAAGGCATTTGAACTT
ATATTTTTGCTATTTAGTTTAAGGTGGTTTTAATTTTGTTATAAGGGCCATCCATTTATGACGTCC
CAAAAAATCAGGTTTTTGACCCCTCTCCCTCTGTCCACATGAGCCGGACCCCCTCCCCC
TTGTGGACGTCCACATGCGGTCGACATGAAATACACATCGCTCTCCTGAAACAGCTAGTAC
AATGACCTTCCGGATCTAAAGCTGAAATTCGACTATGTGATTGTAATTTATTTAAACTGTACTAT
TAGTAATTTTTGTTACTAATTTATATCGCCGAAAAATTGCCACTCTTTATTCCGTCGAAAGACG
AAATTCTGGTAAAAAAGACACCAACTTCTTTGCAGGAGAAGAAGACAGGATGACGTCAGTG
GACTCTAATTTTAATTTTCTGTGTGGACGTCTACATGGGGCTTGACCTCTATCCCAGTCCACA
TGCGTCCACCTGAGCCTGATCCCCCTCTGCGTGGACGTCATAAATGGATGGCCCCTAATT
CGAACTCTAAATGTTTTGTTTTGTGCATACAAACAGGTGTGCACATATAAATACTTTCACATTT
GGTTCACGGTGCAATATTCCATGTTACACAGGCTTTCCAACATTTTGATATCTGATTGCCTTA
TCCTTGCAGCAAACTGACTGGATCTGAGCATGAGTGTATTTAGGAGGGGTGTGGATCAAAC
TTGTGTTTTTCCTGGATCAAATTTTTGTGTTTTTCTGGATCAAATTGTGGTTTTTCATGGTAAAAA
CTCCATAGAAGAACCCATTCCTGTTATAAAAACCTTAAAATACACAAAAATACACCCCAAGC
CAATGAAAACCCCCAAATCAAAACCCCCAAACACATTTCTGGCTACATCCCTGGATCTGA
AGTAAACATCTCTATGTGGGCATCAGAAACTTTTTCACATTATAATAAAATGATAATTCATTTCC
TTTTCCAGTGAAGGAAGGCAGTCGACGATGTCAGCGAACGTTGAAGTACTTCCAGGGAAT
TGCTCACAGGTCATGGGTCATAAGCATCGAATGGATCCTCGACTCCATCAGGATGGGTTG
CCTACAATCGGAGGTCAGTAGCATCTCTGCCTTTCTTGCTAGTGACTGTCTTTACTGTAGCT
GCCTTTGATCCAAACGTTGGCCACACCATGAACCGTGATCTATCTGTTTAGTTCTTTGTGAG
GCATCCTTGTAGCGGTTTGGCTGTGTTACAGTGCCATATAAATTGTCATCATCATTATTATAGC

GATGTATAGGAGGATAAGATTGTAGATACCCTGGCATTTCCATTTTTCTGCATGGAAGGCTTA
CTCCGGTTGTATTGTAGTGACTGTCTTTCCGGTAGCTGCCCTTACTCCAAATGTTGGCCAC
GCCAGGAACTGCGTCTGTATCAGCTTATACTGACAAGTTGGTGAATAAGATTGTAAATAGTA
GTTACCCTGGTATTTCCATTTTGCTGTAGGGTAGCCTTACTCCAGTTGTATCGTATTATATTCA
AAGAGTTATGAACTGTGTCTCAATATCAGTTTATGGTGATGTTTTGGTGGATAAGATTGTAGATA
GTAGATACCCTGGTATTTCCATATTTCTTCAGGGAAGCCTTACTCCAGTTGTATTATATTCAAA
GAGTTATAAAGCCCTTCTCATAGTGTATCATTCAAGTTTGCTCCCATTGTGAAAGTCCCCGA
GAATAAAGGCAAGTTTTGAGGCTGGAATCGCGGTCTTTTGCCCACTCTGGTAGAAGTGCG
GCCGAGTTCTTGTTGAACATGTCCACCTCGTATGCCACACTAGGTGGTATCATTGTGAAATA
CTTTACTTACAGCTGGCATGGTCTCAGCAAGTGGTGTCCTGCTCAGTCTGGTATTTCCATATT
GCATTGTTGCATTGTGGGTTGTACATTAAGGTGGACCTAATAAATGATAACTATCATGTTGTGC
ATATTTCTGGTAATAATAAACCTGTTAGAATGATTCTTATTTAAATTTTCCTACCTAACAGTCAAT
GATGGTGGATGATGGATCGACTGAAGGTTGGACGGACGGATGAATGGATTGACAAACGAA
TCGGTGAATGAATGATTGAATGTATAGATGGCATCATCATCATCACTGGTTAACTGCAGGGTT
TGATGGGACTATTTATTTTCCAAGGACAAATATGAAATCACTGGAGATGTGGCTGTCAACGA
GGATCACTTCGGTCCGACCAATGCTCGACTTTCAGGTGTTCAGCTACTCCGAAAATTTGAA
ATATGTTGCCTTGGTGCCTTCGACGATTTAACCCGTGGTAAGATTCTTTGCTATGATCGTCAT
AGTTAGCCACCGAAGAATACCACAGGTAGTGCGTAAATGTTTGATGCTTTTCTGACTACTTTT
TGTTGTGTGTGGTAGGTATAGGTGTGGCTTTGGTCTAACTCCTCTGTTTATGTCATGTTCACAC
AATGAATTAGTTGCCATTTTCCTTGCGTTAAAGAAGCAATTTTTACGGAGAAAAAAAAGGCA
GCCACCCATTATTGTTATGTTATATGGCTATTTGTAAAGCGCCTTTCACAGAAGGCTGTTCAG
AGACGCTCTCAGCGTGACAGGCTAGTGAAAATAAATATACAGCAAATTTATATATGTGACGC
AAGGGCCCAAAACCCGTCGTTTGTCAGGAATTATTTACAGTGATACAAGCAAAAATAGGAC
TGAGGGGTTTTCAAGTCTGAAATTCTTCATTTCAGCTGGTGTCGTAAATTTGTTATAATTTGAAA
TGTTGTAACCCGATTTCAATGCGGTTTGTTTTATTTGACACAGAATGAAGCCCTTTATTGCCC
AGAATATTGACGATTGCATAGGATTACGATGACACAAAAATCTGAAACTTTTTATATGATATTTC
TCACTTTTTGATTGTTCTAATCAGGCGAGTATAACTTCAGTTAGAAACAAGACATTTTCATTCT
GAAAAAAGCATTTTGAAGCAGATGCAGTGTAGAAAATGATTCAATAAAAAAGCCAAAAACG
ACAGGTGACGGGTTTTGGACCCCTGTGTCACATATATATTGATACCAATAAGTGAACAGAAA
AACTTAAGAACTTTATATTCATGTAGTGGTTGCATGATTGATTGATTAAGGTATTTAAACTGCATT
TCCTGTTTCTTACTTGTTTATACTTTTTACACGTTTAGCTACATTTTTTTTTCTCGGTTTATAGACT
TTTGTGTGCATGGGAATCTTTCTGTGATTGCATTTCTTTGGTGTTTTTGTTGTTCAGATGAATTAA
GAGGACTTGCTGAAGCGTGTGGTGCAAGCAGTCTTCAGGATCCCGCAATGTTCTCATTTGA
TGTTGACAGAGCTCAGGTCATCATCGTCGAAGGAAGCTCTAGTGATACTGGTCTACAGCAC
TTCAAGAGTAAGGGTGTCATCTCCATCAGGCATATAATTATATCGTTAGGGCGATGTATGTATT
GTAAGCGTTTTAACTGTTTATTTTATCTTTTTCTTCCTCATGTTAGGATGGTTAATCGTCGTTATG
ACCCAAACCGGTTCTAGGACAAAATGGTAGCGGACAAAATGATACGGGTAGAATCCTGAA
TTAATCAAGTAATCCAGCTCCCACTGATAATATGATTTTTTCATTAATCCCGCTTCCATTTACA
CAAACTTAGGCTTATCATTTATTTGTAACTTGTGGTTACTAAATATAAATTCAATTCAATTGAATT
GCTACCATATTGTCCAATATCATTTTGTCCCCCAAACCAGGTCCTTTGTGTCGGTTGGCCC
GTTACCCTGAAATTGCCTCCCTCCTTCTCTTTGCACGTCTATTCTCTCTGCTCCCCTTTCCTT
GTCTCTCTCTGTCTCTGTCTCTCTCTCTCTCTCACCTAAATTCTTACCTTTTTCCTGGAGCTGA
AATTTTGTGAAAGCATTTCTGTTTGGCTTATGCCGTGAGAAACGTTACATAAATATCTACATAC
AATACAATTTGATGTCTTTAGACCAAAGTGCAGTTAGCAATTGGACATTAAAGAATATCTTACT
TACTTATCTAAACTAATCTGTCGATTATGAGGATTATGTGTTTTGACTGAGCCTTGGGTGTGTG
AGCACTCGCCCTACGTGATGTCTCGGTTTCTGTTTCTAGATTTTCCTACTCTTAGAGAAATTT
CAATGGCTTAAGTCCACGAAGCATTTGCCTCAACTTACTTGGAATTTTATTTCTTTTTTTATCTT

TTCCTTTTTTCTTTTTCTTTTTGGATATCCTTAACTTTTTTCTTTTGCAAAGGTTGTGCTCAGTCG
ACTCATTATCATTTATTTACCTCAATGTCCAAAAAGAGGACGTATACTTACATCCTGACCTAGA
ATTTTGGATATTGCTAACAATGATCACATCGAGAAATTTTGACGTTTTGGACATATTTGGGGTC
CGTTTGGGCGTTAACGTTGACCTTGGGCGTTAATCCTGCTCATTTTATAGCCGAATTGACTA
GAGGGCACAGGTGCGGCAGAAATCCCAAACCAGATTTCTGCCCTTGCGGGGAAATGAA
CTGGCGACCTCTGGTTTTGACAGTCTGAGAACTGACCACTAGAAATTGGCTGTTATGCTTG
GAAAAAATAATATGAATACAACTCTAATCAAATTATTATGGTAAATGATCCTTTAAAATTAACAA
GCAACCTTGAGCTTGAGCAATGTAAATATCGTTTTATATTTGTATTTCTTAACCTGCTTCAGAG
CTGTACAAGAAGTTCAGCGTGCCGATTGTTGCAAAGGAGTGGCTCTTGGATTCAATCGGAA
CGTACGATGTCAAGTCTCTCGATGAGTATTCCATTTATCCACCACTGCCTTCATAGGCCTTG
TCTCAACGATGTCAGGGGAACCAGCGTAGGCCTTGTCTCAACGATTTCAAGGGAACCAGT
CGTTTCAAACTTTTTAATCATGAAAGCTGCTTAAGGATTGCTTTGCTGCTGTTTTGTGCCATAT
TGTAATTGCCTCCGATGTCTATAAACACTGTGAACTACGGTCTTGGCTGAGATTTTGCGCTAA
GGGAATTATGTGAAATGGTGGTTAATGGTCTGATGGAATTATGTGAATTATGTGAAACGACGG
CACTGAACTCAGTGATATTTTAGTTTTGAACAGGGCATATTTTAAACAGAGTTTACAACTGCAT
ACAGAAAAAAAAATTTCTGACCTGAAAAAATTTCTCATATCTCATAAAAAATTTCTGACGATCTTTT
TTTTAGTCGTTCGCTAAGAATTTGTAAAAAAGTAAATTTTTGATTCTTTTTTGGTCATTTGACAGA
ATTTTTTAATTTTCGCCTATGCCCAAACGTGTCTCTGTACAAATACAAAAGTACAACTGCAGA
CCTAAATTTCCTTACTTTTTTCGTCATTCCCTCGAAATTTCTCGTTTTTCACCCCCTGTTTTCGA
CCCTCACACTTACAAAGTTACAACTACAACTGCACAATTCACCTTTTACAACTGCAAATTAC
ATTTTACAACTGCAGATATTGTGATCAGTTACACATTAAACTGTGCCCTGGTTTTGAAAATAAT
TTAGTTCCTATTACAGTTCACAAAAATATCACTTGCTGAACTTCCATTAATGGTCCGATATGCT
ATGTGCTATAATAATGTGATGCTGTGAAATGGTGATGATGTGAAATTTCTGTTTGTTTCGCTCTT
GTTAAAACTGGAAACATTGGCGGCACTAGGTCGCTTTTCATGACAGGGCATAGATAGAGAC
AGGTCATTAGTAGCATCAACAAGGGCAGTCAAAGTACCGTTCTACTGTGCTTTTATTATTACC
ACTTTTGACTGCCAGGGTATTTCTACCAGGTCGTTTGACCTGCCACATCCTGGTGTGGCAC
CGCCTTTGGCTGAAAATGTTTTAAGGAGTAATCGTTCTGGGTCTGATGACGTTGTCGTTAGT
GTTCTACTGTTAGGCAGGTTCTTGGCTTCCATATTCCACGTAGCTGTTTTTAAGTATCCGTATA
AGCTTACATAAATTAGGTAATATTATAACTGAATTCATTTATTCTTTCATTCATTTAGAAAATTTAT
GTAGCGTCCTCTCAAGAAATCTATTCAGAATTTCAGAAGTGCTCGATCCCAAGTCCAACCA
CGGTGAAAAAGAAGTTTTAAGCAGCTAGTAGAGCAAAGGCGTGTTCATGTAGCTCTTTGGT
AACGATCAGGGCTGTCGAGACCCATATTAAAAAACCTGGGATTTTAGTTTTTTTTTAAACCTA
AAAAACCTAGGTTTTTTAGTTTTTTTTCATTTTCAATTCAGAATTTTTACTTTTTCATGTCAAACTC
TGTAAATTTATTTGAATTAATTGGAATTGCTATTTCGTAACGTTCAACATAGCAGTGCATTATAAT
CCAGACTGGCAGTCAGGGTCAGTCTCAGACAGTGGTCGAATTACCAAAAAATAAGTAAGG
GTGCCCATACAATTGTTAATGCTAATGAGGCTATGCTCCTGATCACTGGCGGATCCATAACT
TCAGAGTCGGGGGGACAAATTTTTTCTGAAATTTTTTGACGACTTTTTACGGGCGTTTCTGAA
AATCTTTTTGGATTTTTCCCCAAAAAATGTTCATCTCTTCCCCAAAATTTCTGACGACCTTTTT
TTTTTTTAGTCATCGACCTTTTCCAGGTTTTAATACGCAATTTTTTCTTTTCCCTTTTTTAATATGT
ATTTTTCCGTAGGGGGGCCAAATCCGTAGCCGACATCAATAGGGGGACCAAAACCCTTA
CTTTTCCACAAATTCACAGTCGCTGTCATTATTCTTTCTGCCAACTTCGAAGGGGGCCATG
GCCCCCCTGGATCCGCACAAGTAGGGGGGCCGAAGGCGGCCCCCTTTGAACCCCTAG
ATTTTCTTCAAGTAAGGGGGCCGTTCAGAGAAGTAGGGGGGCCGCGGCCTACCCTGGCT
CTATGGTAATTCGAGCACTGGTCTCAGAGGTAGGCCTAGTCCAACTACAATGTAAAACCGA
GCAGCAGTGATCAAGCCGACTAGTAACAAAAGCATGGACTAAGGTAGGTACCGGTACAG
CATATAAGGATGAAATTTATTTCACGTTATGTCTACAACCAATATACTAACTTAATAAATAAGAA
AGTATTAGACGATTTCATCAATTTCTGTCAATTTTCCACTGCTTATTTTTGACAGATAATTCCAC

TTCAATATTTCAATGAAACTTTCAAAAAGTGCCCCTTGAAAGTTTAATAAATTTCGTGAAATTTA
TGAAAATTTCGAAATTGAAAGGATTCATAGCCCATCTCTACCTCTCGTCATTTCAACTTTATTC
ACCGTCGGTTTACATTTGCGGTTTTCAGTATATTTCAGTAGTACGTAATAAGCAATTTGAATAA
GAAAGTAAGTATAGCCTTTATTTTCATCTGACTATCTCAAATGCATTACATTGCGTACATGCGC
AACTAGTAGTAACAGAATATAAAGTCACACATGCGCAACCAATATTAATTTAATTGTCGAGTT
GTTTTATTAGCATTTGCCCAGTATCGGTTCACTACCAGTTCAACCGATCAAACCCTGTTTAC
CATACTTACAACGCTCCTTCTGCACTCTCCGGAAATGACTAGGGTCTATAATTAGCCTATGT
CGCGCAGCGTAAAAATCTAGAGTGTATAAATACTTATACCTATTCTTAGATAAATACTCTCGTT
GTAAACACTTCTATCCTAATGGGTGTTGAACTAGGTCGTTGTGCTGAACCTTTTGCACACGA
CACGTTTCATTTCGCTATTCACATTTCACACGGAATTGTGCAAAACGCGTGCAGACCAATA
GAATGGCTTTATTGAGAGGCGTAATCAGGACAACCAATAACAATGAATTGCGACGGGAATC
ATCTGGCCAATAGGAACGATGCCCAGTTTCTATTTAAACGACTATGCAGCGGTGCCAAGAG
ATCGATTGTGCTTGATGCGAAGAAACGTGTTTACTTGAAGATTAGAGGAGTCTGCTAGAAAC
CCGGTGCTAGAAGTAAACATGGCAGACGCACTCGACACTCGTAAGTCGACTTATGTTACC
GTGGGAGTACCTGAAATTTCATTATTTTATGATGCAGGCCTAGGTGCGATATTGAGGTACTG
CAGAGTAATTTACTTTTCAAGCCCAGATGTGTACAATGTCTGTCAACTTGAGGACCAGATTG
GGATTCGTTTTCTAGCCTCACATTCGCTGGGCGAGTGAGCTCCCCAGTTAACCAAGGGCT
TCTAAGATGGGGGCTGAAATGAAGACCTCAGACCCTAACCATCCAAAATCGCTTTACATGG
TATAGTACACACCCCATGTACTACCCAAAATGATGGGCAGTGTATCGTTTAAGTTGTTAGTGT
CTCGTCCAACCCACTCCAGACTTCGCATATAAATTATAAATGCATGAAAACTGCTCAAAGTT
AGGGTGCTGAGATTAAGAACTCAGACCCTAACCATCAAAAATCACTTTATAGGGTATGTTAC
CCACCCTATGTAGTACCCAAGACAGTTAGCGCTATAACGTTTAAGTTATTAGCGTCTCCGTG
ATACATCATCAAACTTCCCATATGAATGCAAGATAATTGCTCTAAGTTGAGAGGCTGAAATGA
AGAACTCCGACCTTAACCATCTAAAAGCGCTTTATAGGGCATATTACCCAACCCATCTACTA
CCCAAAATGCCGGGCAGTATAGCGTTTAAGTCATTAGGAACTCCGTGATACCTCGTGAAAC
TTCACATATAATTCTCAAGAGCCGAAATTACGTTAAAGCGGGGGGTCTGAAATGAAAAACTC
AGACCCTAACCCTCCGAAAACGCTTTACAGGGTATATTATCCAACCCATCTGCTACCCAAA
ATCATTGGTTGCATAACGTTTAAGTCATTATGGACTCCGTGATACCTCATCAAACTTCACATAT
AATTGCATTGTTCTTTAACAGATGAATATTCCTCGAGTTTAGGGGGCTGAAATGAAGGACTCA
GACCCTAACCATCCGAAAGCGATTTTCAGGGTATATTACCCACCCCATCTACTACCCAAAA
TCTTTGGTTGCATAGCGTTTAAGTCATTAGGGACTCCGTCATACCTCATCAAATTTCACATATA
ATTGCACTGTACTTCAAAGGCTGAAGTTTTCTCGAGTTTGGGGGGCTGAAATGAAGAACTC
AGACCCTAACCATCCAAAAGCGCTTTCCTGGATAAATTACCTACCCCATCGACTAGTCAAA
CTGATTGATAGCATAGCGTTTAAATCAATAGGCACTCCGTGATACCTCATCAAATCTCACATA
TAATTGCACTGTACTTCGAAAGCTGGGTTTCCTCGACTTGGGGGAGGGCTGAAAAAAAGG
ACTCAGACCCTAACCATCCATACGCGCTTTACAGGGTATATTACCCAACCCATCTGCTACC
CAAAATCATTGGTTGCATAACGTTTAGGTCATTAGGGACTCCGTGATACCTCATCAAACTTC
ACATATAATTGCACTGTACTTCAAAAGCTGAAGTTTCCTCGACTTTGGGGGTGCTGAAATAA
AGAACTCAGACCCTAACCATCCGAAAGCGATTTTCAGGGTATATTACCCACCCCATCTACT
ACCCAAAAGCTTTGGTTGCATAGCGTTTAAGTCATTAGGGACTCCGTCATACCTCATCAAAT
TTCACATATAATTGCACTGTACTTCAAAGGCTGAAGTTTTCTCGAGTTTGGGGGGCTGAAAT
GAAGAACTCAGACCCTAACCATCCAAAAGCGCTTTCCTGGATAAATTACCTACCCCATCG
ACTAGCCAAACTGATTGATAGCATAGCGTTTAAATCAATAGGCACTCCGTGATACCTCATCA
AATCTCACATATAATTGCACTGTACTTCGAAAGCTGGGTTTCCTCGACTTGGGGGAGGGCT
GAAAAAAGGACTCAGACCCTAACCATCCATACGCGCTTTACAGGGTATATTACCCAACCC
ATCTGCTACCCAAAATCATTGGTTGCATAACGTTTAAGTCATTAGGGACTCCGTGATACCTC
ATCAAACTTCACATATAATTGCGTTGTTCTTTAACAGATGAATATTCCTCGAGTTTGGGGGGC

TGAAATGAAGGACTCAGACCCTAACCATCCGAAAGCGATTTTCAGGGTATATTACCCACCC
CATCTACTACCCAAAAGCTTTGGTTGCATAGCGTTTAAGTCATTAGGGACTCCGTCATACCT
CATCAAATTTCACATATAATTGCACTGTACTTCAAAGGCTGAAGTTTTCTCGAGTTTGGGGGG
CTGAAATGAAGAACTCAGACCCTAACCATCCAAAAGCGCTTTCCTGGATAAATTACCTACC
CCATCGACTAGCCAAACTGATTGATAGCATAGCGTTTAAATCAATAGGCACTCCGTGATAC
CTCATCAAATCTCACATATAATTGCACTGTACTTCGAAAGCTGGGTTTCCTCGACTTGGGGG
AGGGCTGAAAAAAAGGACTCAGACCCTAACCATCCATACGCGCTTTACAGGGTATATTAC
CCAACCCATCTGCTACCCAAAATCATTGGTTGCATAACGTTTAAGTCATTAGGGACTCCGT
GATACCTCATCAAACTTCACATATAATTGCGTTGTTCTTTAACAGATGAATATTCCTCGAGTTT
GGGGGGCTGAAATGAAGGACTCAGACCCTAACCATCCGAAAGCGCTTTTTTGGGTAAATT
ACCTACCCCATCGACTAGCCAAACTGATTGATAGCATAGCGTTTAAATCAATAAGCACTCC
GTGATACCTCATCAAATCTCACATATAATTGCACTGTACTTCGAAAGCTGGGTTTCCTCGACT
TGGGGGAGGGTTGAAAAAAAGAACTCAGACCCTAACCATCCATACGCGCTTTACAGGGTA
TATTACCCACACCATCTGCCACCCAAAATCTTTGGTTGCATAGCGTTTAAGTCATTAGGGAC
TCCGTCATACCTCATCAAATTTCACATATAATTGCACTGTACTTCAAAAGCTGACGTTTCCTC
GAGTTTGGGGGCTGAAATGAAGAACTCAGACCCTAACCATCCAAAAGCGTTTTCCTGGGT
AAATTACCTACCCCATCGACTAGCCAAACTGATTGATAGCATAGCGTTTAAATCAATAGGCA
TTCCGTGATCATCATCATCATCATCATTAACCAAGGGACCAATGCCCGTAGGTGCCCGATG
CCGTTCTCGTTATTCGACGCCATTTTTGTTTCTCCAAAGCAGCGTGGTATAGTTCTTGTATTG
ATGCACCACACCAGTCTGTTATATTGTCCATCCATTCTGTATGTGGTCGCCCTCTTTTGTTAG
TTCCATCCAATCTTCCGAATACTATTGACTTGATTTTTCTGTCGTTTTCCATCCTACATATATGT
CCAAAGAGTTCTGGTTTTCTGGTTATTATTTTTTGCATAAGGTTTTCCGTGAGGTCAATTCTTCT
GTATAATTCTGCATTTGTTACTTTCTGCATCCAACCAATTCTCAGGATCTTTCTATAACATTTCC
TCTCGAAGGCTAGTATCTTCGCTTCTGCCACTTTAGTTATTGTCCAGGCCTCGCATCCATAC
AGCATGCCGCTGAACACACATGTTTTCAGCACCTCTAGTTTCAGTTTCTTATGAATTGATGTA
CTCTTCCAGATTTTGTCCATCGCACTTAAAGCTGCTGTTGCCTTCGCAATCCTGACCAGAAT
TTCTTTTTTACTGTCTAAGTCGTAGGTATTTAAACAACCTAGGTATGTAAACTTTTCCACATTTT
CTAGTTGGTTTCCATCTATTTACCTCATCTAGATACCTCATCAAATCTCACATATAATCCACTTA
TCTGGCTTACATCCTATCTTTCGGACCGGTCTGCTTCTGTCACCTTTCAATCTTCTCGTTCGT
CTTGGCGCCACACCCCTTATGGCCTACCCCAAGGATCTGTTCTTGTGTTCTTGGGCCTCTT
TTCTATATTCTGTTTACTGCAGATACTGGTCCTCTTCTCGCATTTTGTTTTCTCGCAAGTCATTA
TTATGACGATGACGTTCAATCCTACAAACATTGCGATCAGCATCTGAGGCAGCTACTGCAAT
CCGAACTATGTCCCGGGCCACCGATGCTCTCAATGCCTTGGATGTCATCTAATTGCTTGCT
GCAAATCCGCAAAAAACTCAGTATATTTGGTTGGGCACCCGCCAGCAGCTGGACAGGCT
GGACTCTGAGTCCCTGTCTGCTGAAATCCCTACCTTTCTGTTCTCCACTTCTGTCCGGGAC
CTTGGGGTCATCTTGGACCAAGGACTGTCTTTTACTGAGCACATAATTGCACTGACTCGATC
CTGCTATTACCACTTGCGCCAACTCCGGGGTGTTTCCCGCTCTCTTTCCTCCTCTTCCGCT
TCTACTTTGGCTCATGCCTTCATAGCTCATCGATTACATTATTGCTCTCCTTTCTGCTGTGGA
CTCTCTCAAGTTCGATTGCGGCCTTTGAACGGTGTTCTGCGGGAGGCTGCTCGCATGATT
GGTGGTGTGCCTAAGTTTGGCCATATCTCAGACTACATGTGGGATGTACTCCACAGGCTCC
CAGTTCAGCAGCCCATCCGCTATAGGATCTCATATATTGTCTGGCATTGTGTCCTTGGTAAC
GCGCCTTCTTATCTTCTGGAGCTCTTTATTCTGACTTCGGCCTGCTCTGGTCGCCGATCTCT
GCTCTCGGCCACCAAGGGGGACTTTCATGGTGCCACGTGCTCGCATTGCCACCAGACA
GAAAATGGCTTTCTCGATTGTGGGTCCCTCTGTTTGGAATGATCTTCCCTCTGAACTTCGTT
CTCTGCCGCGGGACCTTTCCAGTTCTTTTTATAAACTCCTCAAGACTCTCCTCCTTGGCCG
AGCCTGGGCTGGGAGCGTCTCTGAGTAGTTACCTTGAAGGGGCGCTATATAAGTTGATATA
TAGATAGATAGCTAGAAAGGATACATAGACGAAGCACAGGAGGAGATAACCTGGTCAAGGT

GAGCACTAATTCTGAGTTCATATCTAATAACCACACCCAGTACCCTCAAAGAACTAACTCTC
TCAGCCACGCTGATGATCGAGGAGGCTTGCGGCAAAACATGCCTAATTCGGTTCTTGAAA
ACAATTAGTTCCTTAGTCTTAGATGGATTCAGTCTCAGATTAGTTTCCCTTGCCCATATAGTTAT
ATGCTTAAACTCCTCTGATGCTTCGGCAATGTGATTCGAGCCTACCAGTAAGTAGGAGTCGT
CTTGCGTTGTACTTCAGGAGCTGAAATTTTCTCGAGTGTGGGGGAGCTGAAAGGAAAAACT
CAGACCCTAACCGTCAGAACGCTCTTCTCAGGGTATATAACCCACCCCATCTACTGCCCA
AAATGATTGACTGCGTAGCGTTTAAGTCATTAGGGACTCTGTGATACCTCATAGTCGTTGCGT
CGGACCTCTAGCCAAAATACGAATGAAACGTATTAGTTAAGTTTGCCGACGACAGGTATCTT
CTTGTCGGCTCTAGACATCTTTCAACAGCTACCGAGGAATTTGAGCACATATCAGGACCAT
GAGAAACAACCTCTGCCTAAATCCCAACAAGACCAGGGAGCCTATTGTCCTCAGAAAGG
GCCAGAAATCAATCATCAGCCCCTCGCTCATCATACCTGGGACCTCACGTGTCAGCAGC
ATCCGAGTACTCGGCGTCACCGTCATCTCTGATCTAGGGATGGAACAGCATCTTGACGAG
GTTCATGCCACCTGCGCATCCTCCACGTATGCGCCCAGAGTTCTTCGTTCGCATGGCCTT
CCTCCTTTTGCCACCCATAAGGTTGCCAGAATGACCACGGTCTCATCTTTAATGTAAGCCT
CCCCGGCATGGTGGGGATTCACTCTGGCGCGCGACAGAGCAAGGGTTTAGCAGCTGCT
GAGGAGGATGAAACGCTGCGGTTTCTCCCACCTACATGCATGTACAGCCCCGACTGCCG
AGCAACTGGCGGCCTCACCCGACTCGCAACTCTTTAAGGCCATCATTCTGAATCCCTACC
CAGTCAGCACTGGAATTGGGCCCCAGTCATTGCCCGAATGGGCGTTTAGGGGAAGACCT
GGACTTTGGCGGATTTGCCTTTGCCCTAAAGTTGCCCTAATTGCTGAGCAGAAATTTGCCA
ACCGTTAGGGCAAAGTTAGGGCAACCATCTTCTTTCCCTAATGGGCCCCAAAATTGGTTTC
CAATAGGGCAATAAATTGGGTTCCCAGCTGGGGCCAATGAGCATAACCAAGCTCTCCCCT
AATTGGGCTTGCCAAATGGGCCCCATTCATTGCCCTAAATATTTGCTGACTGGGTACCATG
TGCTGAACAGTCGCCTTCCGGAGAAACGCAGCACCAATTACAGACTCAGACCTAGGCTC
ATCAATTTAATCTACCACAAAAAGATGAAAACATTTTTTACCACTCCTCCTTTTTACAAAAATTT
ACTAGGCCTAGTAGTGGTCATCCAAACTAAACTCGGTAATGATGCTGTTTCACCGCCTTTAT
TCTCGTCGTTTTGTAATTTCAGTTGCGGCTGTGTCAGCTGGAGTCTTTTTCATTAAGCGAATAT
TGTATTGTATTCATGAAGCTTCACACATAATTGCACAATACTTCAAGAGCTGAAATTTCCTCGA
GTTGGGAGGGGCTGAAGAAAAGGACTCAGACCTTAACCACTCAAAAGCGCTTTACATATT
ATATTACCAACCCCATCTATTACCCAAACTTATGGGCAGCATACCATTTAAGTTGTTATCGTC
TCATTCTTATCCTCCAAGCTTCGCATATATATGTGTGGGAACAGCTCATCGTCAAGGGGAAA
TGTTAGATTGATCAGTATTCCCTGTAGCATAGGTTGTCTAAAAGAGTACAATGCAGAAAGATG
TATGTCTACAGGACCTCGGTCCATGAGCAGTGTTAAAGAGATCAGGACCTCGGTGGTCGC
TATGTAGTATTAATGTAGTGGCTAGCTTCAGCTGCTTGAGAATGATTTTTTGCGTTGTGTGATTG
TGCTTCGGAAATCGTTAGCAGCAATCCACTGATCATTTGTGTGTGGTATAGTGTCGGACCGT
TAAATTACGTCTTTACAATATAGAGGCCCATGAATATGTTAACTCATAGCCGGAGATTGGCG
CCGAGTGTGGGGAGGTGGACAGAAGCAAATTTTTGCGGACTAACAATTTGGAATAACCTTA
TTTAGGAAATAATTTCCATTTTCACGCCGACAATTTCGGATTACCTTTTCTTTCAATCTTAAAAA
TGGGCCCTTATTGACGATAAAAACCTATATTTCTGAACAAATTCATTCATGACACCATTTTTAC
TCAAGTCGTACGTTCGCACACATCCAATCACACTACTTCTCAAAATATTAAGGGGACGAATA
CATGGGCCGTCCCCCCACCTCAAATCTTGGAGGGAACGACCCCTAATTCCCCCTAAGTC
TATGCCCAGGGATGAACTAAGTGAAATCTTCTTCTACTTTTCTCTCTTCCATCAGCGTGCTGA
CAATGAGGCTCTTACAAGATTCCTCCAGACCTTTCTGTTTCTGGTTCCTTCACCAATCCTGG
TGAAGTCGATGTTCCTCTCTTTCAGGTCTTCCCTGACATTGTCCATCCAGGTCTTCCTCTGT
CTCCCTCTGCTTCTCTTTCCCTCTACATGTCCATGTAAAGCTGCATTTGGTAATCTTTTCTCCT
CCATCCTTTCCACGTGTCCACACCACTGTAATCTCCTTTTCTTGATCTTTTGTACCACTGTTTT
TTCAGCCCCAAGCTCTTCTCTTGTTTGTTCATTTCTGACTTTTTCTCTTCTACTTCTTCTTATGAT
TCTCCTCAGCCTAAGTGAACTAAGTGAACTAAGTGAAAAGCAAAGCACCTATTAGGGTGGC

CTACATGTTTAGCATCTACATCATTGTAGATGAAGCACTTACTCACAAATCAGTGTTTATAGGC
AGTGGCATTCAAAATATGCATATGTGAGTACTATACATAACTTTCATTATATCCTATTCCATACT
CGAGGTATGTAGCGTACACTGTATGCAAGTGCCTCACAGGCGTCAAGTACTAAGTATACCC
TATGAAAATTAAAAGCCAACTCCTCTTCAGATAGTAAGAGTAAAAATACGCTGGCAGGTTGT
CGAATTAACACAATAGTGCACAACAAAGCATACTGGAAGCACAGGTTCAAGCCGTATTTCC
ATGAGAGCAAGACATCGACCAGGTCAAGAAGTTTGAAGGGGTATCAAATTTGTACCATTTTT
CATGCATTTTATATGCGAATCTTGGAGGGAAAGGAACAATACGTTAATGAAATAATAACTATG
CTACCCATCATTTTGGATAGTAGATATAGGATCGGTATAATATACCCTGAAAAGCGCTTTGGG
ATGGTTAGGGTCTGAGTTCTTCACTTCAGCTTTCCCACACTCGAGGACATTTCAGCTTTTGA
AGTACAGTGCAATTATATGTGAAATTTGATGAGGTATGACGGAGTCCCTTATGATTTAAACGTT
ATGCAACCAATGGTTTTGAGTAGCAGACGAGATGGGTAATATACCCTTAGAAGCGCTTTCG
GATGGTTAGGGTTTGAGTCCTTCATTTCAGCCCCCCAAACTCGAGGAATATTCATCTGTTAA
AGAACAATGCAATTATATGTGAAGTTTGATGAGGTATCACGGAGTCCATAATGACTTAAACGT
TATGCAACCAATGATTTTGGGTAGCAGATGGGTTGGATAATATACCCTGTAAAGCGCGTATG
GATGGTTAGGGTCTGAGTTCTTTATTTCAACCCTCCCCCAAGTCGAGGAAACCCAGTTTTC
GAAGTACAGTGCAATTATATGTGAGATTTGATGAGGTATCACGGAGTGCCTATTGATTTAAAC
GCTATGCTATCAATCAGTTTGGCTAGGCGATGGGGTAGGTAATTTATCCAGGAAAGCGCTTT
TGGATGGTTAGGGTCTCAGTTCTTCATTTCAGCCCCCCAAACTCGAGGAAACTTCAGCTTT
TGAAGTACAGTGCAATTATATGTGAAATTTGATGAGGTATGACGGAGTCCCTAATGACTTAAA
CGCTATGCAACCAAAGCTTTTGGGTAGTAGATGGGGTGGGTAATATACCCTGAAAATCGCT
TTCGGATGGTTAGGGTCTGAGTTCTTCATTTCAGCCCCCCAAACTCGAGGAATATTCATCTG
TTAAAGAACAATGCAATTATATGTGAAGTTTGATGAGGTATCACGGAGTCCATAATGACTTAAA
CGTTATGCAACCAATGATTTTGGGTAGCAGATGGGTTGGATAATATACCCTGTAAAGCGCGT
ATGGATGGTTAGGGTCTGAGTTCTTTATTTCAACCCTCCCCCAAGTCGAGGAAACCCAGTT
TTCGAAGTACAGTGCAATTATATGTGAGATTTGATGAGGTATCACGGAGTGCCTATTGATTTAA
ACGCTATGCTATCAATCAGTTTGGCTAGGCGATGGGGTAGGTAATTTATCCAGGAAAGCGC
TTTTGGATGGTTAGGGTCTCAGTTCTTCATTTCAGCCCCCCAAACTCGAGGAAACTTCAGC
TTTTGAAGTACAGTGCAATTATATGTGAAATTTGATGAGGTATGACGGAGTCCCTAATGACTTA
AACGCTATGCAACCAAAGCTTTTGGGTAGTAGATGGGGTGGGTAATATACACTGAAAATCG
CTTTCGGATGGTTAGGGTCTGAGTTCTTTATTTCAGCACCCCCAAAGTCGAGGAAACTTCA
GCTTTTGAAGTACAGTGCAATTATATGTGAAGTTTGATGAGGTATCACGGAGTCCCTAATGAC
CTAAACGTTATGCAACCAATGATTTTGGGTAGCGGATGGGTTGGGTAATATACCCTGTAAAG
CGCGTATGGATGGTTAGGGTCTGAGTCCTTTTTTTCAGCCCTCCCCCAAGTCGAGGAAAC
CCAGCTTTCGAAGTACAGTGCAATTATATGTGAGATTTGATGAGGTATCACGGAGTGCCTATT
GATTTAAACGCTATGCTATCAATCAGTTTGGCTAGTCGATGGGGTAGGTAATTTATCCAGGAA
AGCGCTTTTGGATGGTTAGGGTCTGAGTTCTTCATTTCAGCCCCCCAAACTCGAGAAAACT
TCAGCCTTTGAAGTACAGTGCAATTATATGTGAAATTTGATGAGGTATGACGGAGTCCCTAAT
GACTTAAACGCTATGCAACCAAAGATTTTGGGTAGTAGATGGGGTGGGTAATATACCCTGAA
AATCGCTTTCGGATGGTTAGGGTCTGAGTCCTTCATTTCAGCCCCCTAAACTCGAGGAATAT
TCATCTGTTAAAGAACAATGCAATTATATGTGAAGTTTGATGAGGTATCACGGAGTCCATAATG
ACTTAAACGTTATGCAACCAATGATTTTGGGTAGCAGATGGGTTGGATAATATACCCTGTAAA
GCGTTTTCGGAGGGTTAGGGTCTGAGTTTTTCATTTCAGACCCCCCGCTTTAACGTAATTTC
GGCTCTTGAGAATTATATGTGAAGTTTCACGAGGTATCACGGAGTTCCTAATGACTTAAACG
CTATACTGCCCGGCATTTTGGGTAGTAGATGGGTTGGGTAATATGCCCTATAAAGCGCTTTT
AGATGGTTAAGGTCGGAGTTCTTCATTTCAGCCTCTCAACTTAGAGCAATTATCTTGCATTCA
TATGGGAAGTTTGATGATGTATCACGGAGACGCTAATAACTTAAACGTTATAGCGCTAACTGT
CTTGGGTACTACATAGGGTGGGTAACATACCCTATAAAGTGATTTTTGATGGTTAGGGTCTGA

GTTCTTAATCTCAGCACCCTAACTTTGAGCAGTTTTCATGCATTTATAATTTATATGCGAAGTCT
GGAGTGGGTTGGACGAGACACTAACAACTTAAACGATACACTGCCCATCATTTTGGGTAGT
ACATGGGGTGTGTACTATACCATGTAAAGCGATTTTGGATGGTTAGGGTCTGAGGTCTTCATT
TCAGCCCCATCTTAGAAGCCCTTGGTTAACTGGGGAGCTCACTCGCCCAGCGAATGTGA
GGCTAGAAAACGAATCCCAATCTGGTCCTCAAGTTGACAGACATATGTGTACAAGCCATTTT
TAGTTCTGTGATGATGATGATGATGATGATGATGGTGATGATGATGATGATGATGATGATGATG
ACGACGATGACGACGGCGGCGGCGGCGGTGGTGGTGGTGGTGATGATGATGATGATGG
TGGTTGTGATGATGATGATCATGGTGGTGATGATGATGGTGGTGATTATGATGATGATATCTGG
CCAAAACTAATTTATCATTAGTATTCTTTATTTATTTCGTAAGGCAATTAACATAATTACCATCAG
CAAAATTTAGCATTGCTTGAGCAATGTTTTAAATAAGAGGCTCTATGCTACTTATGCAATTGAT
TTCTTATAGAAGATGTAAAACATTTAAATAATAGCAGTTTACATTACCATGCTAAGGGTATAACC
TTGTAATATCAAATATCAGTCACTGAAATCCGATTATGTAAGCTGCTATATTCTGCCTCCTCAC
ACCACCTAAGACCAGCTAAAAAAGTCAGACCAGAAGCAAGGCAACAAGCTCACTTGCCA
AATTGGATAGAGACAAGTTGAGGAGATGCATTAGGCCATTAGCCAGCGTCCAGATGGTACA
GACCTGTACAGGAAGTACCAAGAAACTTATTCTGCCTTGTTGCTAGTTTCAAATTGTTTGCAA
GGTCTTTTTGTAGTGTATAGTTACTAACAAATCTTTGCCTCCTTATGCTTTTAGAGTGCTGTGG
AAAATCAACCTGCCAAAGTAAGCCAAATTTCTTACTTGCCTTAACAATTTAACATGTCTTTGAT
ATCTCTCACAGTTAGTAATTTTATTACCGCGAGTTGGATGTTAGCATTGTTACTTCTTCTCTTTC
ACTGTTACATCCGTGTTATAATAACGACATTTTACCCGTTCTTGTCATCACTACTGGCAATATC
ATCACTATGACTCATACCATGTTAATTTAATAGAATATAATGTGGCAGAACACTTATATAGTGCC
TATCGAGAACCTCCCAAAAGTGCAATTGCTAACACCTCCACCACCTTAAATCTTTCTTTTTG
AATGGAATCATTCCTTTTCTTGTCGCCACCATCAAAATAATGCTCATGGTGTGGTCATTACTA
CCATCATCTATCAGCAGTCATCATCAGTTACCAAACGTGTCACAAGACCTCTGGCGTGTAC
AAGAAGTACTAAGGAAGTTATTCTGTCTTGTTGCTGATTTTAAATTGCTTGCAATTTACATACAT
TTTTATTTTGTAAAGCCGAAAATCTCAGTATATTATTTCAGTCAAAGAGAATGACTCTTTTATTTT
TTTAGGAGAGGGATCAACTTGTTGCTGCACAAACTGCAGATGTTTGAAAAGTGAGTGCTTG
CCAGGCTGCAAAAAGCTTTGCTGTGCTGACGCCGAGAAGGGCAAATGTGGAAGTGAGTT
TGCTATTTGTGTTTTGTTTAAAATTATAGTGCAGTCGAATACTAATTTTAAAGGTTGAACAACTTT
TTTGTCAAAGGTTGAAATGATCTCTCAGGAATAGAAAACATAGTTTCAGCGTAATAATAATTTA
CCTAAACATTTCTGGCCTTGTCCAAATATTCATTTACCATAGAACTTTGTAAAAGCACCAGTTT
CTGATTAAATGCTTGTCTCAAATAGCCCCTTGGGCAAAATGACTTGAGAAAATAAATACAGG
CCTCTCAAATAAAGTTCCCTTATACATGCTGAGCCTTGTTTTGACTCCACTATGAAGATAGCG
ATAACAGGCCAAAAACTCCCCGGCCAATAACCCCCCGACATCGATGAACAAAGTTGCTT
ACATAGAAGCTGGATTGATGAAAGGAGTTTTTATTAACTCGAAGGTAGATTGATGAAAGAATT
CTTCGTTTACGAGAACCTGGACGTACACTGTACAATAATTGTTGGAAATAAAACTATGCTGAA
TCTTGGTCCTCGGGTACTGCTTTCAGCAAAAGGGATAATTATGCGATTTGTACAACTAAACA
ACAGGAATTTGTATATTATTGGTTACTGCATTTTCATAAATTTGATCCCGATGCAGTAGCTGTTC
TGTAATGCTGAGCTACTTGTGTGCAGCCTTGGTTGGAAACAGATAAATTTATTTTATAATGGCT
ATCTTTTCTTCTAAATCCAGTGAAACCTTTCTGACATGTTCTGTTACTGTAATTTCCAATTTATGT
CACCTTATGCACAAGACATAAACCACTGGCACTTAGAAGCCAAATCATAATCAGAATAATAC
CAAGAAGAGCATTCTACAAGGCAGCCTGTGAAGAATTAACAAGTTTATCTGCTTGTCCTTTA
GTTTGTGGCCAGTGTGCAGTTTAGCTCTCTCTCTCTCTCTCCATCATTTTTCCTGCAGCGCA
TCTTGCATGCTTCTTGAACACTTTCCCATTACTCCATTCTGTACTGTGTCACCAGAAGAGATT
TGTCATGAAGTATGATCGCTGATCAGTCCAGTGAATTTGTGTCTTTATGAACTACTTAGTCCTT
TAGTTTCAGAAAAATTTTCAGCTGTGGCTGAACTACAGCAAAACTTCATTATATCGAACCTAA
ATGAACCGGCATTAACGGCTCGATATATCCGAGGTTCGAAATAAGCCATATACATTTATTTG
CATTGATTGTAGCCGAGCCATTTAGCGTTATTAGATAAAACCGGAAGTTCGAAATACCGAAG

TTCGATATAATGAAGTTTTGCTGTATAGAGATCTTTTACACAATAATAGAAATATTTTTACATTGTA
TTTACTACAATGTCCTCACAATGTACATTTCAGATGCCGGCTGCAAGTGCGGGGCAGCTTG
CAAATGCTCGGCGGGTTCGTGCGCCGCAGGATGCAAGAAGGGATGCTGTGGTGACTAGT
GTACGTGTAAGACTAAACTTTTCCTTCTTTTGTTAGTATTACGGTATTGAAGAAAGAACTTGTGT
TGCATGTAAAGAATCACTACTTTGTCTTTTGTGCATTTGTGTGAAGGCTGTCTTAAAGATTGCT
TCTGACTTGTTAAAACAGAGCCAAAATAATTATTTAAAAAAAATTTTTGTCCTAGTTTTACCCC
AGGCGATATGAAGTGATGACCAATCGCGTTAATAAATGGTGCTATAGAAGTGTTTATGTTTGA
TTTGATTAGTAGCTATGATTGTGATATGATTTGTGTCATTTATAAAGTTTTTCAAACTTAATTGTGT
GGAAATTGATGTTATTCATATATTATGTCTATACTGAAATATTGATGCACTGTGTAACTGTTCTTT
GATTTTTGTTTTAGTGAGAGCCAAGGCATTGAAATATGGAACTCTGTCATCAGTTTAATACATA
AATTCTTTTCTGTTACTTATTTGCGATAGAATTATTCTGAAGGACACTGCGTAACTTTTCTCAAG
TAACTTCAGTTATTCAGTATTCCAATTTGATAAATTTGTATTTTGTTTCTTTTTTTATCTTTTCCTTTT
TTCTTTTTCTTTTTGGATATCCTTAACTTTTTTCTTTTGCAAAGGTTGTGCTCAGTCGACTCATTA
TCATTTATTTACCTCAATGTCCAAAAAGAGGACGTATACTTACATCCTGACCTAGAATTTTGGA
TATTGCTAACAATGATCACATCGAGAAATTTTGACGTTTTGGACATATTTGGGGTCCGTTTGG
GCGTTAACGTTGACCTTGGGCGTTAATCCTGCTCATTTTATAGCCGAATTGACTAGAGGGC
ACAGGTGCGGCAGAAATCCCAAACCAGATTTCTGCCCTTGCGGGGAAATGAACTGGCGA
CCTCTGGTTTTGACAGTCTGAGAACTGACCACTAGAAATTGGCTGTTATGCTTGGAAAAAAT
AATATGAATACAACTCTAATCAAATTATTATGGTAAATGATCCTTTAAAATTAACAAGCAACCTT
GAGCTTGAGCAATGTAAATATCGTTTTATATTTGTATTTCTTAACCTGCTTCAGAGCTGTACAA
GAAGTTCAGCGTGCCGATTGTTGCAAAGGAGTGGCTCTTGGATTCAATCGGAACGTACGAT
GTCAAGTCTCTCGATGAGTATTCCATTTATCCACCACTGCCTTCATAGGCCTTGTCTCAACG
ATGTCAGGGGAACCAGCGTAGGCCTTGTCTCAACGATTTCAAGGGAACCAGTCGTTTCAA
ACTTTTTAATCATGAAAGCTGCTTAAGGATTGCTTTGCTGCTGTTTTGTGCCATATTGTAATTG
CCTCCGATGTCTATAAACACTGTGAACTACGGTCTTGGCTGAGATTTTGCGCTAAGGGAATT
ATGTGAAATGGTGGTTAATGGTCTGATGGAATTATGTGAATTATGTGAAACGACGGCACTGAA
CTCAGTGATATTTTAGTTTTGAACAGGGCATATTTTAAACAGAGTTTACAACTGCATACAGAAA
AAAAATTTCTGACCTGAAAAAATTTCTCATATCTCATAAAAATTTCTGACGATCTTTTTTTTAGTC
GTTCGCTAAGAATTTGTAAAAAAGTAAATTTTTGATTCTTTTTTGGTCATTTGACAGAATTTTTTA
ATTTTCGCCTATGCCCAAACGTGTCTCTGTACAAATACAAAAGTACAACTGCAGACCTAAAT
TTCCTTACTTTTTTCGTCATTCCCTCGAAATTTCTCGTTTTTCACCCCTGTTTTCGACCCTCAC
ACTTACAAAGTTACAACTACAACTGCACAATTCACCTTTTACAACTGCAAATTACATTTTACA
ACTGCAGATATTGTGATCAGTTACACATTAAACTGTGCCCTGGTTTTGAAAATAATTTAGTTCC
TATTACAGTTCACAAAAATATCACTTGCTGAACTTCCATTAATGGTCCGATATGCTATGTGCTA
TAATAATGTGATGCTGTGAAATGGTGATGATGTGAAATTTCTGTTTGTTTCGCTCTTGTTAAAAC
TGGAAACATTGGCGGCACTAGGTCGCTTTTCATGACAGGGCATAGATAGAGACAGGTCATT
AGTAGCATCAACAAGGGCAGTCAAAGTACCGTTCTACTGTGCTTTTATTATTACCACTTTTGA
CTGCCAGGGTATTTCTACCAGGTCGTTTGACCTGCCACATCCTGGTGTGGCACCGCCTTT
GGCTGAAAATGTTTTAAGGAGTAATCGTTCTGGGTCTGATGACGTTGTCGTTAGTGTTCTACT
GTTAGGCAGGTTCTTGGCTTCCATATTCCACGTAGCTGTTTTTAAGTATCCGTATAAGCTTAC
ATAAATTAGGTAATATTATAACTGAATTCATTTATTCTTTCATTCATTTAGAAAATTTATGTAGCGT
CCTCTCAAGAAATCTATTCAGAATTTCAGAAGTGCTCGATCCCAAGTCCAACCACGGTGAA
AAAGAAGTTTTAAGCAGCTAGTAGAGCAAAGGCGTGTTCATGTAGCTCTTTGGTAACGATCA
GGGCTGTCGAGACCCATATTAAAAAACCTGGGATTTTAGTTTTTTTTTAAACCTAAAAAACCT
AGGTTTTTTAGTTTTTTTTCATTTTCAATTCAGAATTTTTACTTTTTCATGTCAAACTCTGTAAATTT
ATTTGAATTAATTGGAATTGCTATTTCGTAACGTTCAACATAGCAGTGCATTATAATCCAGACT
GGCAGTCAGGGTCAGTCTCAGACAGTGGTCGAATTACCAAAAAATAAGTAAGGGTGCCCA

TACAATTGTTAATGCTAATGAGGCTATGCTCCTGATCACTGGCGGATCCATAACTTCAGAGT
CGGGGGGACAAATTTTTTCTGAAATTTTTTGACGACTTTTTACGGGCGTTTCTGAAAATCTTT
TTGGATTTTTCCCCAAAAAATGTTCATCTCTTCCCCAAAATTTCTGACGACCTTTTTTTTTTTA
GTCATCGACCTTTTCCAGGTTTTAATACGCAATTTTTTCTTTTCCCTTTTTTAATATGTATTTTTC
CGTAGGGGGGCCAAATCCGTAGCCGACATCAATAGGGGGACCAAAACCCTTACTTTTCC
ACAAATTCACAGTCGCTGTCATTATTCTTTCTGCCAACTTCGAAGGGGGCCATGGCCCCC
CTGGATCCGCACAAGTAGGGGGGCCGAAGGCGGCCCCCTTTGAACCCCTAGATTTTCTT
CAAGTAAGGGGGCCGTTCAGAGAAGTAGGGGGGCCGCGGCCTACCCTGGCTCTATGGT
AATTCGAGCACTGGTCTCAGAGGTAGGCCTAGTCCAACTACAATGTAAAACCGAGCAGCA
GTGATCAAGCCGACTAGTAACAAAAGCATGGACTAAGGTAGGTACCGGTACAGCATATAAG
GATGAAATTTATTTCACGTTATGTCTACAACCAATATACTAACTTAATAAATAAGAAAGTATTAG
ACGATTTCATCAATTTCTGTCAATTTTCCACTGCTTATTTTTGACAGATAATTCCACTTCAATATT
TCAATGAAACTTTCAAAAAGTGCCCCTTGAAAGTTTAATAAATTTCGTGAAATTTATGAAAATTT
CGAAATTGAAAGGATTCATAGCCCATCTCTACCTCTCGTCATTTCAACTTTATTCACCGTCG
GTTTACATTTGCGGTTTTCAGTATATTTCAGTAGTACGTAATAAGCAATTTGAATAAGAAAGTAA
GTATAGCCTTTATTTTCATCTGACTATCTCAAATGCATTACATTGCGTACATGCGCAACTAGTA
GTAACAGAATATAAAGTCACACATGCGCAACCAATATTAATTTAATTGTCGAGTTGTTTTATTA
GCATTTGCCCAGTATCGGTTCACTACCAGTTCAACCGATCAAACCCTGTTTACCATACTTAC
AACGCTCCTTCTGCACTCTCCGGAAATGACTAGGGTCTATAATTAGCCTATGTCGCGCAGC
GTAAAAATCTAGAGTGTATAAATACTTATACCTATTCTTAGATAAATACTCTCGTTGTAAACACTT
CTATCCTAATGGGTGTTGAACTAGGTCGTTGTGCTGAACCTTTTGCACACGACACGTTTCAT
TTCGCTATTCACATTTCACACGGAATTGTGCAAAACGCGTGCAGACCAATAGAATGGCTTTA
TTGAGAGGCGTAATCAGGACAACCAATAACAATGAATTGCGACGGGAATCATCTGGCCAA
TAGGAACGATGCCCAGTTTCTATTTAAACGACTATGCAGCGGTGCCAAGAGATCGATTGTG
CTTGATGCGAAGAAACGTGTTTACTTGAAGATTAGAGGAGTCTGCTAGAAACCCGGTGCTA
GAAGTAAACATGGCAGACGCACTCGACACTCGTAAGTCGACTTATGTTACCGTGGGAGTA
CCTGAAATTTCATTATTTTATGATGCAGGCCTAGGTGCGATATTGAGGTACTGCAGAGTAATT
TACTTTTCAAGCCCAGATGTGTACAATGTCTGTCAACTTGAGGACCAGATTGGGATTCGTTTT
CTAGCCTCACATTCGCTGGGCGAGTGAGCTCCCCAGTTAACCAAGGGCTTCTAAGATGG
GGGCTGAAATGAAGACCTCAGACCCTAACCATCCAAAATCGCTTTACATGGTATAGTACAC
ACCCCATGTACTACCCAAAATGATGGGCAGTGTATCGTTTAAGTTGTTAGTGTCTCGTCCAA
CCCACTCCAGACTTCGCATATAAATTATAAATGCATGAAAACTGCTCAAAGTTAGGGTGCTG
AGATTAAGAACTCAGACCCTAACCATCAAAAATCACTTTATAGGGTATGTTACCCACCCTAT
GTAGTACCCAAGACAGTTAGCGCTATAACGTTTAAGTTATTAGCGTCTCCGTGATACATCATC
AAACTTCCCATATGAATGCAAGATAATTGCTCTAAGTTGAGAGGCTGAAATGAAGAACTCCG
ACCTTAACCATCTAAAAGCGCTTTATAGGGCATATTACCCAACCCATCTACTACCCAAAATG
CCGGGCAGTATAGCGTTTAAGTCATTAGGAACTCCGTGATACCTCGTGAAACTTCACATATA
ATTCTCAAGAGCCGAAATTACGTTAAAGCGGGGGGTCTGAAATGAAAAACTCAGACCCTA
ACCCTCCGAAAACGCTTTACAGGGTATATTATCCAACCCATCTGCTACCCAAAATCATTGGT
TGCATAACGTTTAAGTCATTATGGACTCCGTGATACCTCATCAAACTTCACATATAATTGCATT
GTTCTTTAACAGATGAATATTCCTCGAGTTTAGGGGGCTGAAATGAAGGACTCAGACCCTAA
CCATCCGAAAGCGATTTTCAGGGTATATTACCCACCCCATCTACTACCCAAAATCTTTGGTT
GCATAGCGTTTAAGTCATTAGGGACTCCGTCATACCTCATCAAATTTCACATATAATTGCACT
GTACTTCAAAGGCTGAAGTTTTCTCGAGTTTGGGGGGCTGAAATGAAGAACTCAGACCCTA
ACCATCCAAAAGCGCTTTCCTGGATAAATTACCTACCCCATCGACTAGCCAAACTGATTGA
TAGCATAGCGTTTAAATCAATAGGCACTCCGTGATACCTCATCAAATCTCACATATAATTGCA
CTGTACTTCGAAAGCTGGGTTTCCTCGACTTGGGGGAGGGCTGAAAAAAAGGACTCAGA

CCCTAACCATCCATACGCGCTTTACAGGGTATATTACCCAACCCATCCGCTACCCAAAAT
CATTGGTTGCATAACGTTTAGGTCATTAGGGACTCCGTGATACCTCATCAAACTTCACATATA
ATTGCACTGTACTTCAAAAGCTGAAGTTTCCTCGACTTTGGGGGTGCTGAAATAAAGAACTC
AGACCCTAACCATCCGAAAGCGATTTTCAGTGTATATTACCCACCCCATCTACTACCCAAA
AGCTTTGGTTGCATAGCGTTTAAGTCATTAGGGACTCCGTCATACCTCATCAAATTTCACATA
TAATTGCACTGTACTTCAAAAGCTGAAGTTTCCTCGAGTTTGGGGGGCTGAAATGAAGAACT
GAGACCCTAACCATCCAAAAGCGCTTTCCTGGATAAATTACCTACCCCATCGCCTAGCCA
AACTGATTGATAGCATAGCGTTTAAATCAATAGGCACTCCGTGATACCTCATCAAATCTCACA
TATAATTGCACTGTACTTCGAAAACTGGGTTTCCTCGACTTGGGGGAGGGTTGAAATAAAGA
ACTCAGACCCTAACCATCCATACGCGCTTTACAGGGTATATTATCCAACCCATCTGCTACC
CAAAATCATTGGTTGCATAACGTTTAAGTCATTATGGACTCCGTGATACCTCATCAAACTTCA
CATATAATTGCATTGTTCTTTAACAGATGAATATTCCTCGAGTTTGGGGGGCTGAAATGAAGA
ACTCAGACCCTAACCATCCGAAAGCGATTTTCAGGGTATATTACCCACCCCATCTACTACC
CAAAAGCTTTGGTTGCATAGCGTTTAAGTCATTAGGGACTCCGTCATACCTCATCAAATTTC
ACATATAATTGCACTGTACTTCAAAAGCTGAAGTTTCCTCGAGTTTGGGGGGCTGAAATGAA
GAACTGAGACCCTAACCATCCAAAAGCGCTTTCCTGGATAAATTACCTACCCCATCGCCT
AGCCAAACTGATTGATAGCATAGCGTTTAAATCAATAGGCACTCCGTGATACCTCATCAAAT
CTCACATATAATTGCACTGTACTTCGAAAACTGGGTTTCCTCGACTTGGGGGAGGGTTGAA
ATAAAGAACTCAGACCCTAACCATCCATACGCGCTTTACAGGGTATATTATCCAACCCATCT
GCTACCCAAAATCATTGGTTGCATAACGTTTAAGTCATTATGGACTCCGTGATACCTCATCAA
ACTTCACATATAATTGCATTGTTCTTTAACAGATGAATATTCCTCGAGTTTGGGGGGCTGAAAT
GAAGGACTCAGACCCTAACCATCCGAAAGCGATTTTCAGGGTATATTACCCACCCCATCT
ACTACCCAAAAGCTTTGGTTGCATAGCGTTTAAGTCATTAGGGACTCCGTCATACCTCATCA
CATTTCACATATAATTGCACTGTACTTCAAAAGCTGAAGATTCCTCGAGTTTGGGGGCTGAA
ATGAAGAACTCAGACCCTAACCATCCAAAAGCGCTTTCCTGGGTAAATTACATACCCCATC
GACTAGCCAAACTGATTGATAGCATAGCGTTTAAATCAATAGGCACTCCGTGATACCTCATC
AAATCTCACATATAATTGCACTGTACTTCGAAAGCTGGGTTTCCTCGACTTGGGGGAGGGTT
CAAAAAAAGAACTCAGACCCTAACCATCCATACGCGCTTTACAGGGTATATTATCCAACCT
ATACGGTACCCAAAATCAGTGGTTGCATAACGTTTAATTCATTAGGGACTCCGTGATACCTC
ATCAAATCTCACATATAATTGCACTGTACTTCGAAAGCTGGGTTTCCTCGACTTGGGGGAGG
GTTGAAAAAAATAACTCAGACCCTAACCATCCATACGCCCTTTACAGGGTATATTATCCAAC
CCATCTGCTACCCAAAATCATTGGTCGCATAACGTTTAAGTCATTATGGACTGCGTGATACC
TCATCAAACTTCACATATAATTGCATTGTTCTTTAACAGATGAATATTCCTCGAGTTTGGGGAC
TTGAAATGAAGGACTCAGACCCTAACCATCCGAAAGCGGTTTTCAGGGTATATTACCCACC
CCATCTACTACCCAAAATCTTTGGTTGCATAGCGTTTAAGTCATTAGGGACTCCGTCATACC
TCATCACATTTCACATATAATTGCACTGTACTTCAAAAGCTGAAGTTTCCTCGAGTTTGGGGG
CTGAAATGAAGAACTCAGACCCTAACCATCCAAAAGCGCTTTCCTGGGTAAATTACATACC
CCATCGACTAGCCAAACTGATTGATAGCATAGCGTTTAAATCAATAGGCACTCCGTGATAC
CTCATCAAATCTCACATATAATTGCACTGTACTTCGAAAGCTGGGTTTCCTCGACTTGGGGG
AGGGTTCAAAAAAAGAACTCAGACCCTAACCATCCATACGCGCTTTACAGGGTATATTATC
CAACCTATACGGTACCCAAAATCAGTGGTTGCATAACGTTTAATTCATTAGGGACTCCGATC
ATCTAAATTTCTGGGGGTCATTGTTGATGAACACTTGACTTGGAGGGACCATGTGCTAGCTG
TCTCAAACAAAATAGCCAAAAATATAGGTGTACTCCTTAGAGTCAGACACTGCTTACCCAAA
CACATTTTATTCAATCTATACTACACTCTAATTTTTCCCTATCTCTCTTACTGTAATCTACTGTGG
GGATCGAACTACAAAACCTACCTTAATCATTTATTTAACTTGCAAAAACGTGCTATTAGAATTG
TCTGTAACATTCCCTGGAAGTCGAGCATTGAACCAGTTTTAACTAAACATAACCTGCTCTCG
TTATTTAAAATAAATAAATATCAAGTTTACCTATTTATGTATCGCCTCTATCACAACTTGTTGCCT

ACTTCCTTGTCTTCAAATTTCCAAAGGGGATTTCACATTCATAATTATTTACTCGTTTTTCCCA
TCAGTATAGAAGCCACCCAGCTCGTTTAAAGATAAAACAACTGTCTATCAACTGTTTGGGTC
CAGTTTTATGGAATTCCCTTCCAGAAGTAATTAGAAAGTCACCTTCAATGGGTTCGTTCAAG
CTCTCAGTTAAAAAATTCATCATTTTTGATAAATACTGAATAATTTGTTAATCTGGTCACATACTA
GAAATTCTTTACAATCTACATGTAGTTTGTTTTTTGTTTTGTTTTTTGCATTGTGCATGGTTGTCTT
AATTTAGATGTGGTTTCTCTAGTAATAAGATTGATTGTTTTTTGCTGCCATTTGCTGCATACATG
TCTATTGGGTTACCCATGATAGCGGGATTAGTCCCTTTTGGTTTGCCCTTTACAAACTCCATA
ACTTTCTTGTTAATATGTCTTTTGTGTATCTATTGGTGTTTGCAATAAAAAATGGTATTATTGATTA
TTGATTATTGTGATACCTCATCAAATCTCACATATAATTGCACTGTACTTCGAAAGCTGGGTTT
CCTCGACTTGGGGGAGGGTTGAAAAAAATAACTCAGACCCTAACCATCCATACGCCCTTT
ACAGGGTATATTATCCAACCCATCTGCTACCCAAAATCATTGGTCGCATAACGTTTAAGTCA
TTATGGACTGCGTGATACCTCATCAAACTTCACATATAATTGCATTGTTCTTTAACAGATGAAT
ATTCCTCGAGTTTGGGGACTTGAAATGAAGGACTCAGACCCTAACCATCCGAAAGCGGTT
TTCAGGGTATATTACCCACCCCATCTACTACCCAAAATCTTTGGTTGCATAGCGTTTAAGTCA
TTAGGGACTCCGTCATACCTCATCACATTTCACATATAATTGCACTGTACTTCAAAAGCTGAA
GTTTCCTCGAGTTTGGGGGCTGAAATGAAGAACTCAGACCCTAACCATCCAAAAGCGCTT
TCCTGGATAAATTACCTACCCCATCGCCTAGCCAAACTGATTGATAGCATAGCGTTTAAATC
AATAGGCACTCCGTGATACCTCATCAAATCTCACATATAATTGCACTGTACTTCGAAAACTG
GGTTTCCTCGACTTGGGGGAGGGTTGAAATAAAGAACTCAGACCCTAACCATCCATACGC
GCTTTACAGGGTATATTATCCAACCCATCTGCTACCCAAAATCATTGGTTGCATAACGTTTAA
GTCATTATGGACTCCGTGATACCTCATCAAACTTCACATATAATTGCATTGTTCTTTAACAGAT
GAATATTCCTCGAGTTTGGGGGGCTGAAATGAAGGACTCAGACCCTAACCATCCGAAAGC
GATTTTCAGGGTATATTACCCACCCCATCTACTACCCAAAAGCTTTGGTTGCATAGCGTTTA
AGTCATTAGGGACTCCGTCATACCTCATCACATTTCACATATAATTGCACTGTACTTCAAAAG
CTGAAGATTCCTCGAGTTTGGGGGCTGAAATGAAGAACTCAGACCCTAACCATCCAAAAG
CGCTTTCCTGGGTAAATTACATACCCCATCGACTAGCCAAACTGATTGATAGCATAGCGTTT
AAATCAATAGGCACTCCGTGATACCTCATCAAATCTCACATATAATTGCACTGTACTTCGAAA
GCTGGGTTTCCTCGACTTGGGGGAGGGTTCAAAAAAAGAACTCAGACCCTAACCATCCAT
ACGCGCTTTACAGGGTATATTATCCAACCTATACGGTACCCAAAATCAGTGGTTGCATAACG
TTTAATTCATTAGGGACTCCGTGATACCTCATCAAATCTCACATATAATTGCACTGTACTTCGA
AAGCTGGGTTTCCTCGACTTGGGGGAGGGTTGAAAAAAATAACTCAGACCCTAACCATCC
ATACGCCCTTTACAGGGTATATTATCCAACCCATCTGCTACCCAAAATCATTGGTCGCATAA
CGTTTAAGTCATTATGGACTGCGTGATACCTCATCAAACTTCACATATAATTGCATTGTTCTTT
AACAGATGAATATTCCTCGAGTTTGGGGACTTGAAATGAAGGACTCAGACCCTAACCATCC
GAAAGCGGTTTTCAGGGTATATTACCCACCCCATCTACTACCCAAAATCTTTGGTTGCATAG
CGTTTAAGTCATTAGGGACTCCGTCATACCTCATCACATTTCACATATAATTGCACTGTACTT
CAAAAGCTGAAGTTTCCTCGAGTTTGGGGGCTGAAATGAAGAACTCAGACCCTAACCATC
CAAAAGCGCTTTCCTGGGTAAATTACATACCCCATCGACTAGCCAAACTGATTGATAGCAT
AGCGTTTAAATCAATAGGCACTCCGTGATACCTCATCAAATCTCACATATAATTGCACTGTAC
TTCGAAAGCTGGGTTTCCTCGACTTGGGGGAGGGTTCAAAAAAAGAACTCAGACCCTAAC
CATCCATACGCGCTTTACAGGGTATATTATCCAACCTATACGGTACCCAAAATCAGTGGTTG
CATAACGTTTAATTCATTAGGGACTCCGATCATCTAAATTTCTGGGGGTCATTGTTGATGAAC
ACTTGACTTGGAGGGACCATGTGCTAGCTGTCTCAAACAAAATAGCCAAAAATATAGGTGTA
CTCCTTAGAGTCAGACACTGCTTACCCAAACACATTTTATTCAATCTATACTACACTCTAATTT
TTCCCTATCTCTCTTACTGTAATCTACTGTGGGGATCGAACTACAAAACCTACCTTAATCATTT
ATTTAACTTGCAAAAACGTGCTATTAGAATTGTCTGTAACATTCCCTGGAAGTCGAGCATTGA
ACCAGTTTTAACTAAACATAACCTGCTCTCGTTATTTAAAATAAATAAATATCAAGTTTACCTAT

TTATGTATCGCCTCTATCACAACTTGTTGCCTACTTCCTTGTCTTCAAATTTCCAAAGGGGATT
TCACATTCATAATTATTTTACTCGTTTTTCCCATCAGTATAGAAGCCACCCAGCTCGTTTAAAG
ATAAAACAACTGTCTATCAACTGTTTGGGTCCAGTTTTATGGAATTCCCTTCCAGAAGTAATTA
GAAAGTCACCTTCAATGGGTTCGTTCAAGCTCTCAGTTAAAAAATTCATCATTTTTGATAAATA
CTGAATAATTTGTTAATCTGGTCACATACTAGAAATTCTTTACAATCTACATGTAGTTTGTTTTTT
GTTTTGTTTTTTGCATTGTGCATGGTTGTCTTAATTTAGATGTGGTTTCTCTAGTAATAAGATTGA
TTGTTTTTTGCTGCCATTTGCTGCATACATGTCTATTGGGTTACCCATGATAGCGGGATTAGT
CCCTTTTGGTTTGCCCTTTACAAACTCCATAACTTTCTTGTTAATATGTCTTTTGTGTATCTATTG
GTGTTTGCAATAAAAAATGGTATTATTGATTATTGATTATTGTGATACCTCATCAAATCTCACATA
TAATTGCACTGTACTTCGAAAGCTGGGTTTCCTCGACTTGGGGGAGGGTTGAAAAAAATAA
CTCAGACCCTAACCATCCATACGCCCTTTACAGGGTATATTATCCAACCCATCTGCTACCC
AAAATCATTGGTCGCATAACGTTTAAGTCATTATGGACTGCGTGATACCTCATCAAACTTCAC
ATATAATTGCATTGTTCTTTAACAGATGAATATTCCTCGAGTTTGGGGACTTGAAATGAAGGAC
TCAGACCCTAACCATCCGAAAGCGGTTTTCAGGGTATATTACCCACCCCATCTACTACCCA
AAATCTTTGGTTGCATAGCGTTTAAGTCATTAGGGACTCCGTCATACCTCATCACATTTCACA
TATAATTGCACTGTACTTCAAAAGCTGAAGTTTCCTCGAGTTTGGGGGCTGAAATGAAGAAC
TCAGACCCTAACCATCCAAAAGCGCTTTCCTGGATAAATTACCTACCCCATCGCCTAGCC
AAACTGATTGATAGCATAGCGTTTAAATCAATAGGCACTCCGTGATACCTCATCAAATCTCAC
ATATAATTGCACTGTACTTCGAAAACTGGGTTTCCTCGACTTGGGGGAGGGTTGAAATAAAG
AACTCAGACCCTAACCATCCATACGCGCTTTACAGGGTATATTATCCAACCCATCTGCTAC
CCAAAATCATTGGTTGCATAACGTTTAAGTCATTATGGACTCCGTGATACCTCATCAAACTTC
ACATATAATTGCATTGTTCTTTAACAGATGAATATTCCTCGAGTTTGGGGGGCTGAAATGAAG
GACTCAGACCCTAACCATCCGAAAGCGCTTCTAAGGGTATATTACCCATCTCGTCTGCTAC
TCAAAACCATTGGTTGCATAACGTTTAAATCATAAGGGACTCCGTCATACCTCATCAAATTTC
ACATATAATTGCACTGTACTTCAAAAGCTGAAATGTCCTCGAGTGTGGGAAAGCTGAAGTGA
AGAACTCAGACCCTAACCATCCCAAAGCGCTTTTCAGGGTATATTATACCGATCCTATATCT
ACTATCCAAAATGATGGGTAGCATAGTTATTATTTCATTAACGTATTGTTCCTTTCCCTCCAAG
ATTCGCATATAAAATGCATGAAAAATGGTACAAATTTGATACCCCTTCAAACTTCTTGACCTG
GTCGATGTCTTGCTCTCATGGAAATACGGCTTGAACCTGTGCTTCCAGTATGCTTTGTTGTG
CACTATTGTGTTAATTCGACAACCTGCCAGCGTATTTTTACTCTTACTATCTGAAGAGGAGTT
GGCTTTTAATTTTCATAGGGTATACTTAGTACTTGACGCCTGTGAGGCACTTGCATACAGTGT
ACGCTACATACCTCGAGTATGGAATAGGATATAATGAAAGTTATGTATAGTACTCACATATGCA
TATTTTGAATGCCACTGCCTATAAACACTGATTTGTGAGTAAGTGCTTCATCTACAATGATGTA
GATGCTAAACATGTAGGCCACCCTAATAGGTGCTTTGCTTTTCACTTAGTTCACTTAGTTCAC
TTAGGCTGAGGAGAATCATAAGAAGAAGTAGAAGAGAAAAAGTCAGAAATGAACAAACAAG
AGAAGAGCTTGGGGCTGAAAAAACAGTGGTACAAAAGATCAAGAAAAGGAGATTACAGTG
GTGTGGACACGTGGAAAGGATGGAGGAGAAAAGATTACCAAATGCAGCTTTACATGGACA
TGTAGAGGGAAAGAGAAGCAGAGGGAGACAGAGGAAGACCTGGATGGACAATGTCAGG
GAAGACCTGAAAGAGAGGAACATCGACTTCACCAGGATTGGTGAAGGAACCAGAAACAG
AAAGGTCTGGAGGAATCTTGTAAGAGCCTCATTGTCAGCACGCTGATGGAAGAGAGAAAA
GTAGAAGAAGATTTCACTTAGTTCATCCCTGGGCATAGACTTAGGGGGAATTAGGGGTCGT
TCCCTCCAAGATTTGAGGTGGGGGGACGGCCCATGTATTCGTCCCCTTAATATTTTGAGAA
GTAGTGTGATTGGATGTGTGCGAACGTACGACTTGAGTAAAAATGGTGTCATGAATGAATTTG
TTCAGAAATATAGGTTTTTATCGTCAATAAGGGCCCATTTTTAAGATTGAAAGAAAAGGTAATC
CGAAATTGTCGGCGTGAAAATGGAAATTATTTCCTAAATAAGGTTATTCCAAATTGTTAGTCC
GCAAAAATTTGCTTCTGTCCACCTCCCCACACTCGGCGCCAATCTCCGGCTATGAGTTAA
CATATTCATGGGCCTCTATATTGTAAAGACGTAATTTAACGGTCCGACACTATACCACACAC

AAATGATCAGTGGATTGCTGCTAACGATTTCCGAAGCACAATCACACAACGCAAAAAATCA
TTCTCAAGCAGCTGAAGCTAGCCACTACATTAATACTACATAGCGACCACCGAGGTCCTGA
TCTCTTTAACACTGCTCATGGACCGAGGTCCTGTAGACATACATCTTTCTGCATTGTACTCTT
TTAGACAACCTATGCTACAGGGAATACTGATCAATCTAACATTTCCCCTTGACGATGAGCTG
TTCCCACACATATATATGCGAAGCTTGGAGGATAAGAATGAGACGATAACAACTTAAATGGT
ATGCTGCCCATAAGTTTGGGTAATAGATGGGGTTGGTAATATAATATGTAAAGCGCTTTTGAG
TGGTTAAGGTCTGAGTCCTTTTCTTCAGCCCCTCCCAACTCGAGGAAATTTCAGCTCTTGAA
GTATTGTGCAATTATGTGTGAAGCTTCATGAATACAATACAATATTCGCTTAATGAAAAAGACT
CCAGCTGACACAGCCGCAACTGAAATTACAAAACGACGAGAATAAAGGCGGTGAAACAG
CATCATTACCGAGTTTAGTTTGGATGACCACTACTAGGCCTAGTAAATTTTTGTAAAAAGGAG
GAGTGGTAAAAAATGTTTTCATCTTTTTGTGGTAGATTAAATTGATGAGCCTAGGTCTGAGTCT
GTAATTGGTGCTGCGTTTCTCCGGAAGGCGACTGTTCAGCACATGGTACCCAGTCAGCAA
ATATTTAGGGCAATGAATGGGGCCCATTTGGCAAGCCCAATTAGGGGAGAGCTTGGTTATG
CTCATTGGCCCCAGCTGGGAACCCAATTTATTGCCCTATTGGAAACCAATTTTGGGGCCC
ATTAGGGAAAGAAGATGGTTGCCCTAACTTTGCCCTAACGGTTGGCAAATTTCTGCTCAGC
AATTAGGGCAACTTTAGGGCAAAGGCAAATCCGCCAAAGTCCAGGTCTTCCCCTAAACG
CCCATTCGGGCAATGACTGGGGCCCAATTCCAGTGCTGACTGGGTAGGGATTCAGAATG
ATGGCCTTAAAGAGTTGCGAGTCGGGTGAGGCCGCCAGTTGCTCGGCAGTCGGGGCTG
TACATGCATGTAGGTGGGAGAAACCGCAGCGTTTCATCCTCCTCAGCAGCTGCTAAACCC
TTGCTCTGTCGCGCGCCAGAGTGAATCCCCACCATGCCGGGGAGGCTTACATTAAAGAT
GAGACCGTGGTCATTCTGGCAACCTTATGGGTGGCAAAAGGAGGAAGGCCATGCGAACG
AAGAACTCTGGGCGCATACGTGGAGGATGCGCAGGTGGCATGAACCTCGTCAAGATGCT
GTTCCATCCCTAGATCAGAGATGACGGTGACGCCGAGTACTCGGATGCTGCTGACACGTG
AGGTCCCAGGTATGATGAGCGAGGGGCTGATGATTGATTTCTGGCCCTTTCTGAGGACAAT
AGGCTCCCTGGTCTTGTTGGGATTTAGGCAGAGGTTGTTTCTCATGGTCCTGATATGTGCTC
AAATTCCTCGGTAGCTGTTGAAAGATGTCTAGAGCCGACAAGAAGATACCTGTCGTCGGCA
AACTTAACTAATACGTTTCATTCGTATTTTGGCTAGAGGTCCGACGCAACGACTATGAGGTAT
CACAGAGTCCCTAATGACTTAAACGCTACGCAGTCAATCATTTTGGGCAGTAGATGGGGTG
GGTTATATACCCTGAGAAGAGCGTTCTGACGGTTAGGGTCTGAGTTTTTCCTTTCAGCTCCC
CCACACTCGAGAAAATTTCAGCTCCTGAAGTACAACGCAAGACGACTCCTACTTACTGGTA
GGCTCGAATCACATTGCCGAAGCATCAGAGGAGTTTAAGCATATAACTATATGGGCAAGGG
AAACTAATCTGAGACTGAATCCATCTAAGACTAAGGAACTAATTGTTTTCAAGAACCGAATTA
GGCATGTTTTGCCGCAAGCCTCCTCGATCATCAGCGTGGCTGAGAGAGTTAGTTCTTTGAG
GGTACTGGGTGTGGTTATTAGATATGAACTCAGAATTAGTGCTCACCTTGACCAGGTTATCTC
CTCCTGTGCTTCGTCTATGTATCCTTTCTAGCTATCTATCTATATATCAACTTATATAGCGCCCC
TTCAAGGTAACTACTCAGAGACGCTCCCAGCCCAGGCTCGGCCAAGGAGGAGAGTCTTG
AGGAGTTTATAAAAAGAACTGGAAAGGTCCCGCGGCAGAGAACGAAGTTCAGAGGGAAG
ATCATTCCAAACAGAGGGACCCACAATCGAGAAAGCCATTTTCTGTCTGGTGGCAATGCG
AGCACGTGGCACCATGAAAGTCCCCCTTGGTGGCCGAGAGCAGAGATCGGCGACCAG
AGCAGGCCGAAGTCAGAATAAAGAGCTCCAGAAGATAAGAAGGCGCGTTACCAAGGACA
CAATGCCAGACAATATATGAGATCCTATAGCGGATGGGCTGCTGAACTGGGAGCCTGTGG
AGTACATCCCACATGTAGTCTGAGATATGGCCAAACTTAGGCACACCACCAATCATGCGA
GCAGCCTCCCGCAGAACACCGTTCAAAGGCCGCAATCGAACTTGAGAGAGTCCACAGC
AGAAAGGAGAGCAATAATCTAATCGATGAGCTATGAAGGCATGAGCCAAAGTAGAAGCGG
AAGAGGAGGAAAGAGAGCGGGAAACACCCCGGAGTTGGCGCAAGTGGTAATAGCAGGA
TCGAGTCAGTGCAATTATGTGCTCAGTAAAAGACAGTCCTTGGTCCAAGATGACCCCAAGG
TCCCGGACAGAAGTGGAGAACAGAAAGGTAGGGATTTCAGCAGACAGGGACTCAGAGTC

CAGCCTGTCCAGCTGCTGGCGGGTGCCCAACCAAATATACTGAGTTTTTTGCGGATTTGC
AGCAAGCAATTAGATGACATCCAAGGCATTGAGAGCATCGGTGGCCCGGGACATAGTTC
GGATTGCAGTAGCTGCCTCAGATGCTGATCGCAATGTTTGTAGGATTGAACGTCATCGTCAT
AATAATGACTTGCGAGAAAACAAAATGCGAGAAGAGGACCAGTATCTGCAGTAAACAGAAT
ATAGAAAAGAGGCCCAAGAACACAAGAACAGATCCTTGGGGTAGGCCATAAGGGGTGTG
GCGCCAAGACGAACGAGAAGATTGAAAGGTGACAGAAGCAGACCGGTCCGAAAGATAG
GATGTAAGCCAGATAAGTGGATTATATGTGAGATTTGATGAGGTATCTAGATGAGGTAAATAGA
TGGAAACCAACTAGAAAATGTGGAAAAGTTTACATACCTAGGTTGTTTAAATACCTACGACTT
AGACAGTAAAAAAGAAATTCTGGTCAGGATTGCGAAGGCAACAGCAGCTTTAAGTGCGAT
GGACAAAATCTGGAAGAGTACATCAATTCATAAGAAACTGAAACTAGAGGTGCTGAAAACAT
GTGTGTTCAGCGGCATGCTGTATGGATGCGAGGCCTGGACAATAACTAAAGTGGCAGAAG
CGAAGATACTAGCCTTCGAGAGGAAATGTTATAGAAAGATCCTGAGAATTGGTTGGATGCA
GAAAGTAACAAATGCAGAATTATACAGAAGAATTGACCTCACGGAAAACCTTATGCAAAAAA
TAATAACCAGAAAACCAGAACTCTTTGGACATATATGTAGGATGGAAAACGACAGAAAAATC
AAGTCAATAGTATTCGGAAGATTGGATGGAACTAACAAAAGAGGGCGACCACATACAGAAT
GGATGGACAATATAACAGACTGGTGTGGTGCATCAATACAAGAACTATACCACGCTGCTTT
GGAGAAACAAAAATGGCGTCGAATAACGAGAACGGCATCGGGCACCTACGGGCATTGGT
CCCTTGGTTAATGATGATGATGATGATGATCACGGAATGCCTATTGATTTAAACGCTATGCTAT
CAATCAGTTTGGCTAGTCGATGGGGTAGGTAATTTACCCAGGAAAACGCTTTTGGATGGTTA
GGGTCTGAGTTCTTCATTTCAGCCCCCAAACTCGAGGAAACGTCAGCTTTTGAAGTACAGT
GCAATTATATGTGAAATTTGATGAGGTATGACGGAGTCCCTAATGACTTAAACGCTATGCAAC
CAAAGATTTTGGGTGGCAGATGGTGTGGGTAATATACCCTGTAAAGCGCGTATGGATGGTTA
GGGTCTGAGTTCTTTTTTTCAACCCTCCCCCAAGTCGAGGAAACCCAGCTTTCGAAGTACA
GTGCAATTATATGTGAGATTTGATGAGGTATCACGGAGTGCTTATTGATTTAAACGCTATGCTA
TCAATCAGTTTGGCTAGTCGATGGGGTAGGTAATTTACCCAAAAAAGCGCTTTCGGATGGTT
AGGGTCTGAGTCCTTCATTTCAGCCCCCCAAACTCGAGGAATATTCATCTGTTAAAGAACA
ACGCAATTATATGTGAAGTTTGATGAGGTATCACGGAGTCCCTAATGACTTAAACGTTATGCA
ACCAATGATTTTGGGTAGCAGATGGGTTGGGTAATATACCCTGTAAAGCGCGTATGGATGGT
TAGGGTCTGAGTCCTTTTTTTCAGCCCTCCCCCAAGTCGAGGAAACCCAGCTTTCGAAGT
ACAGTGCAATTATATGTGAGATTTGATGAGGTATCACGGAGTGCCTATTGATTTAAACGCTAT
GCTATCAATCAGTTTGGCTAGTCGATGGGGTAGGTAATTTATCCAGGAAAGCGCTTTTGGAT
GGTTAGGGTCTGAGTTCTTCATTTCAGCCCCCCAAACTCGAGAAAACTTCAGCCTTTGAAG
TACAGTGCAATTATATGTGAAATTTGATGAGGTATGACGGAGTCCCTAATGACTTAAACGCTA
TGCAACCAAAGCTTTTGGGTAGTAGATGGGGTGGGTAATATACCCTGAAAATCGCTTTCGG
ATGGTTAGGGTCTGAGTCCTTCATTTCAGCCCCCCAAACTCGAGGAATATTCATCTGTTAAA
GAACAACGCAATTATATGTGAAGTTTGATGAGGTATCACGGAGTCCCTAATGACTTAAACGT
TATGCAACCAATGATTTTGGGTAGCAGATGGGTTGGGTAATATACCCTGTAAAGCGCGTATG
GATGGTTAGGGTCTGAGTCCTTTTTTCAGCCCTCCCCCAAGTCGAGGAAACCCAGCTTTC
GAAGTACAGTGCAATTATATGTGAGATTTGATGAGGTATCACGGAGTGCCTATTGATTTAAAC
GCTATGCTATCAATCAGTTTGGCTAGTCGATGGGGTAGGTAATTTATCCAGGAAAGCGCTTT
TGGATGGTTAGGGTCTGAGTTCTTCATTTCAGCCCCCCAAACTCGAGAAAACTTCAGCCTT
TGAAGTACAGTGCAATTATATGTGAAATTTGATGAGGTATGACGGAGTCCCTAATGACTTAAA
CGCTATGCAACCAAAGCTTTTGGGTAGTAGATGGGGTGGGTAATATACCCTGAAAATCGCT
TTCGGATGGTTAGGGTCTGAGTTCTTTATTTCAGCACCCCCAAAGTCGAGGAAACTTCAGC
TTTTGAAGTACAGTGCAATTATATGTGAAGTTTGATGAGGTATCACGGAGTCCCTAATGACCT
AAACGTTATGCAACCAATGATTTTGGGTAGCAGATGGGTTGGGTAATATACCCTGTAAAGCG
CGTATGGATGGTTAGGGTCTGAGTCCTTTTTTTCAGCCCTCCCCCAAGTCGAGGAAACCC

AGCTTTCGAAGTACAGTGCAATTATATGTGAGATTTGATGAGGTATCACGGAGTGCCTATTGA
TTTAAACGCTATGCTATCAATCAGTTTGACTAGTCGATGGGGTAGGTAATTTATCCAGGAAAG
CGCTTTTGGATGGTTAGGGTCTGAGTTCTTCATTTCAGCCCCCCAAACTCGAGAAAACTTC
AGCCTTTGAAGTACAGTGCAATTATATGTGAAATTTGATGAGGTATGACGGAGTCCCTAATGA
CTTAAACGCTATGCAACCAAAGATTTTGGGTAGTAGATGGGGTGGGTAATATACCCTGAAAA
TCGCTTTCGGATGGTTAGGGTCTGAGTCCTTCATTTCAGCCCCCTAAACTCGAGGAATATTC
ATCTGTTAAAGAACAATGCAATTATATGTGAAGTTTGATGAGGTATCACGGAGTCCATAATGA
CTTAAACGTTATGCAACCAATGATTTTGGGTAGCAGATGGGTTGGATAATATACCCTGTAAAG
CGTTTTCGGAGGGTTAGGGTCTGAGTTTTTCATTTCAGACCCCCCGCTTTAACGTAATTTCG
GCTCTTGAGAATTATATGTGAAGTTTCACGAGGTATCACGGAGTTCCTAATGACTTAAACGCT
ATACTGCCCGGCATTTTGGGTAGTAGATGGGTTGGGTAATATGCCCTATAAAGCGCTTTTAG
ATGGTTAAGGTCGGAGTTCTTCATTTCAGCCTCTCAACTTAGAGCAATTATCTTGCATTCATAT
GGGAAGTTTGATGATGTATCACGGAGACGCTAATAACTTAAACGTTATAGCGCTAACTGTCTT
GGGTACTACATAGGGTGGGTAACATACCCTATAAAGTGATTTTTGATGGTTAGGGTCTGAGTT
CTTAATCTCAGCACCCTAACTTTGAGCAGTTTTCATGCATTTATAATTTATATGCGAAGTCTGG
AGTGGGTTGGACGAGACACTAACAACTTAAACGATACACTGCCCATCATTTTGGGTAGTAC
ATGGGGTGTGTACTATACCATGTAAAGCGATTTTGGATGGTTAGGGTCTGAGGTCTTCATTTC
AGCCCCATCTTAGAAGCCCTTGGTTAACTGGGGAGCTCACTCGCCCAGCGAATGTGAGG
CTAGAAAACGAATCCCAATCTGGTCCTCAAGTTGACAGACATATGTGTACAAGCCATTTTTA
GTTCTGTGATGATGATGATGATGATGATGATGGTGATGATGATGATGATGATGATGATGATGAC
GACGATGACGACGGCGGCGGCGGCGGTGGTGGTGGTGATGATGATGATGATGGTG
GTTGTGATGATGATGATCATGGTGGTGATGATGATGGTGGTGATTATGATGATGATATCTGGC
CAAAACTAATTTATCATTAGTATTCTTTATTTATTTCGTAAGGCAATTAACATAATTACCATCAGC
AAAATTTAGCATTGCTTGAGCAATGTTTTAAATAAGAGGCTCTATGCTACTTATGCAATTGATTT
CTTATAGAAGATGTAAAACATTTAAATAATAGCAGTTTACATTACCATGCTAAGGGTATAACCT
TGTAATATCAAATATCAGTCACTGAAATCCGATTATGTAAGCTGCTATATTCTGCCTCCTCACA
CCACCTAAGACCAGCTAAAAAAGTCAGACCAGAAGCAAGGCAACAAGCTCACTTGCCAA
ATTGGATAGAGACAAGTTGAGGAGATGCATTAGGCCATTAGCCAGCGTCCAGATGGTACA
GACCTGTACAGGAAGTACCAAGAAACTTATTCTGCCTTGTTGCTAGTTTCAAATTGTTTGCAA
GGTCTTTTTGTAGTGTATAGTTACTAACAAATCTTTGCCTCCTTATGCTTTTAGAGTGCTGTGG
AAAATCAACCTGCCAAAGTAAGCCAAATTTCTTACTTGCCTTAACAATTTAACATGTCTTTGAT
ATCTCTCACAGTTAGTAATTTTATTACCGCGAGTTGGATGTTAGCATTGTTACTTCTTCTCTTTC
ACTGTTACATCCGTGTTATAATAACGACATTTTACCCGTTCTTGTCATCACTACTGGCAATATC
ATCACTATGACTCATACCATGTTAATTTAATAGAATATAATGTGGCAGAACACTTATATAGTGCC
TATCGAGAACCTCCCAAAAGTGCAATTGCTAACACCTCCACCACCTTAAATCTTTCTTTTTG
AATGGAATCATTCCTTTTCTTGTCGCCACCATCAAAATAATGCTCATGGTGTGGTCATTACTA
CCATCATCTATCAGCAGTCATCATCAGTTACCAAACGTGTCACAAGACCTCTGGCGTGTAC
AAGAAGTACTAAGGAAGTTATTCTGTCTTGTTGCTGATTTTAAATTGCTTGCAATTTACATACAT
TTTTATTTTGTAAAGCCGAAAATCTCAGTATATTATTTCAGTCAAAGAGAATGACTCTTTTATTTT
TTTAGGAGAGGGATCAACTTGTTGCTGCACAAACTGCAGATGTTTGAAAAGTGAGTGCTTG
CCAGGCTGCAAAAAGCTTTGCTGTGCTGACGCCGAGAAGGGCAAATGTGGAAGTGAGTT
TGCTATTTGTGTTTTGTTTAAAATTATAGTGCAGTCGAATACTAATTTTAAAGGTTGAACAACTTT
TTTGTCAAAGGTTGAAATGATCTCTCAGGAATAGAAAACATAGTTTCAGCGTAATAATAATTTA
CCTAAACATTTCTGGCCTTGTCCAAATATTCATTTACCATAGAACTTTGTAAAAGCACCAGTTT
CTGATTAAATGCTTGTCTCAAATAGCCCCTTGGGCAAAATGACTTGAGAAAATAAATACAGG
CCTCTCAAATAAAGTTCCCTTATACATGCTGAGCCTTGTTTTGACTCCACTATGAAGATAGCG
ATAACAGGCCAAAAACTCCCCGGCCAATAACCCCCCGACATCGATGAACAAAGTTGCTT

ACATAGAAGCTGGATTGATGAAAGGAGTTTTTATTAACTCGAAGGTAGATTGATGAAAGAATT
CTTCGTTTACGAGAACCTGGACGTACACTGTACAATAATTGTTGGAAATAAAACTATGCTGAA
TCTTGGTCCTCGGGTACTGCTTTCAGCAAAAGGGATAATTATGCGATTTGTACAACTAAACA
ACAGGAATTTGTATATTATTGGTTACTGCATTTTCATAAATTTGATCCCGATGCAGTAGCTGTTC
TGTAATGCTGAGCTACTTGTGTGCAGCCTTGGTTGGAAACAGATAAATTTATTTTATAATGGCT
ATCTTTTCTTCTAAATCCAGTGAAACCTTTCTGACATGTTCTGTTACTGTAATTTCCAATTTATGT
CACCTTATGCACAAGACATAAACCACTGGCACTTAGAAGCCAAATCATAATCAGAATAATAC
CAAGAAGAGCATTCTACAAGGCAGCCTGTGAAGAATTAACAAGTTTATCTGCTTGTCCTTTA
GTTTGTGGCCAGTGTGCAGTTTAGCTCTCTCTCTCTCTCTCCATCATTTTTCCTGCAGCGCA
TCTTGCATGCTTCTTGAACACTTTCCCATTACTCCATTCTGTACTGTGTCACCAGAAGAGATT
TGTCATGAAGTATGATCGCTGATCAGTCCAGTGAATTTGTGTCTTTATGAACTACTTAGTCCTT
TAGTTTCAGAAAAATTTTCAGCTGTGGCTGAACTACAGCAAAACTTCATTATATCGAACCTAA
ATGAACCGGCATTAACGGCTCGATATATCCGAGGTTCGAAATAAGCCATATACATTTATTTG
CATTGATTGTAGCCGAGCCATTTAGCGTTATTAGATAAAACCGGAAGTTCGAAATACCGAAG
TTCGATATAATGAAGTTTTGCTGTATAGAGATCTTTTACACAATAATAGAAATATTTTTACATTGTA
TTTACTACAATGTCCTCACAATGTACATTTCAGATGCCGGCTGCAAGTGCGGGGCAGCTTG
CAAATGCTCGGCGGGTTCGTGCGCCGCAGGATGCAAGAAGGGATGCTGTGGTGACTAGT
GTACGTGTAAGACTAAACTTTTCCTTCTTTTGTTAGTATTACGGTATTGAAGAAAGAACTTGTGT
TGCATGTAAAGAATCACTACTTTGTCTTTTGTGCATTTGTGTGAAGGCTGTCTTAAAGATTGCT
TCTGACTTGTTAAAACAGAGCCAAAATAATTATTTAAAAAAAATTTTTGTCCTAGTTTTACCCC
AGGCGATATGAAGTGATGACCAATCGCGTTAATAAATGGTGCTATAGAAGTGTTTATGTTTGA
TTTGATTAGTAGCTATGATTGTGATATGATTTGTGTCATTTATAAAGTTTTTCAAACTTAATTGTGT
GGAAATTGATGTTATTCATATATTATGTCTATACTGAAATATTGATGCACTGTGTAACTGTTCTTT
GATTTTTGTTTTAGTGAGAGCCAAGGCATTGAAATATGGAACTCTGTCATCAGTTTAATACATA
AATTCTTTTCTGTTACTTATTTGCGATAGAATTATTCTGAAGGACACTGCGTAACTTTTCTCAAG
TAACTTCAGTTATTCAGTATTCCAATTTGATAAATTTGTATTTTGTTTTAAATTGTACATGTTCTATG
ACCGTTTGACTTTGCATAGAAAGATCAAAAGTTATAAACTGCATATTCGAATGTGTCGTTTTCT
TGATGTGATTTTCATAATGGTCATTGAAGTTGATGTAGGTCACACTGTTGAACTGTGCATAACG
TCAGGTAGTTTGGGTAGCACCTGATTAACATATATTTCTACATGCAGTGGCTGATCCAGAGG
GCATGTCCGGCCATGCTCCCCTCCATCCAGATTGGCTATATGCTTTGGCCACCCTTATAC
GAAGAAACAAACATGAGATATTGGGAAACATTAAATATGGCCCTGAACCGAATGTTGAGTCT
CTGGATACGTCACACGATATTCCCCCTAGCCGAATGTCTGGATCCGCAACTGTCTACATGT
ACATGTTACACATTTCTTTAAACAGCATTCCTGATATGTGATCTTGTCTATCTATCTATCTATCTT
GTGGTTGAAGGATATTTGGCTGATGTACAGACAACCATTTCTCTACAGACTTTACGAGCACT
ACTGCTGCTTTTGATACCATCAGTCCTTGCCCGATACATATTTACTTTTTTTAATTAATGACTAT
CTGCCAAGGTATATAATTAAGATCGTAACCAGAATGAACGTATGGTTCCGCTAACAGGGTC
GCCCTAATTACATCAGACATCACAATCAATCAATGCTATTACATGCACTATCTGTATCAATGC
AGACCCGTTCATTTTCTGCGGTTGGTCCAAAAACCTGGAATGGACTTCCAGTAGATCTAAG
ATACCTTCCAAACGGTGCCTGTTCTCAATTCCACCACCTTCTTAAGACTGTTCTTTTCCGCT
TGGCCTGGGTCGGGAGCGCCTCTGAGTAGGTATCTTGAAGGGGCGCTATATAAATTTTGCT
TGATTGATTGATCTGCATAACATAGTACACTGATAGACAATCACATAATTGTTGTTTTGTCCGTA
TTTCAATTCATGGGAAACGTGGATAAAAACACAAAAGAGAACTGCAGTATAAATCGGTTAAC
AAGCAACTGCAAAACAAAATGATACAAGATAAAATAAAAGTCAACTTGTACATTTACACAAG
CGCATTTGAAATAAATAACTTTAGTTTGTATTTAACATTGGTAAATTGTAGATGCAAATACATGTA
GGCCTACACTAGTGAGTTCCAGAGGTAGAGAATTGCAGAGCAATGGTCCACACATTCTTAT
AGAATGCTGTCCTGATGTGGTGCAAACACTTTGTTGATGATATTTATTTGTTGATCTGATAAAAT
AGTCGTGTACTTCTGAATTTTTACAAAACCAATGGCCAAGTAAGGCTTGCTAGAATAAGATTG

GAGTGCAGGTTATAGGCTACATGTACATTCCAATTATAATGGTTGTGTGTTTAGAATGAATTTA
ATCTCTTAGTTAATTCGTAGAAGTCTTAGTTGCCATGGCAGACACTCGCGCTGTGAGACCTG
CAATTCCAGGATTCTGATAGCTTACAAACCTCATTTATGGAATTCCTCAAAGTACAGTGAAAT
GATCAAATTTTAAAATGTATTTTTTGCTCAATACGTAGCCGAAATGATGATTGGAATTTGGATGT
ATCTGTTTTTATTTAGAAGGTCGTTGAAATAAGAGCTTGTGAAAATGGGTAGATGAGTGCGATT
ACTAAACAAGTGATAATAGTCAAAGCGCTACAACATTTTTAACCTTTGACGTTAACGTTATTG
CGTACGTAAAGGCCTTGCGTAAAGGTCGTGCTTGGCCTAGCCTATTTATTAATATTCATAGA
GCGCGATTCTGATTTGCTGCTTATCTTGTTGGTCGACGATGTGGGCGGGGTGCGTGTGTTT
GTGAATATGAATGAAGGACTTTCAACGGCTAACCAACCGTCGCATGTTTAAAAGCATAATAC
GAGAATGGCGCACAATGTTATGAAGTTGTCGTTTGTAGCAGGCTCATTTGGTAACTAAAAAG
TTTATTTGGCACTATAGAAGAACTGTTATAATTTATGGGTTTATTTAAATTTTTTAAAGCAATTCTG
ATCAGTTAAAAGTTAACGTAGTAGTAGTTTAAAATGTGTGATGACATGACATTGACGCGAGTC
GAGAGCGCCTCTGATTTCCAAGTCATAGCAATTGACGCTATATTAGTACTTTATTAAAACGAA
GTGACTCCTGCACCGTCGACGGTGAAGGCATAGTTCCGTGCAAATTCGCAAAATACCGG
CGCACAAAAGCTTTCAAAAGCGTCGTGATGGTCGGCAAGGGGGTTTGGGGTCGCCACTC
CCAGCATCGCTCTGATATATTCCAGCAGAACAGCATCGCACATAGGCCTAATTGTAGGCCT
ATACTCAAGCAAAATAGCATCGTTCCGGTTCGCCCGTATATATTTCGGTAGAACAGCATCG
CGAATATTATATTTCAGCCGAAAAGCAACGCCCGTATATACTCCGGCAGAATGGCATCGTA
TGTTCGTATACGCACCCTGGGAGTGCCGATCCCTGGGAGTGGCGGCCCGTCACCGGCT
AGGGTCAGGGCAAGCGTCATGAGGGTCTTAGGGTTAGTATTAGGGTTAGTGTGGTCATTAG
ACAGAAATTCCACCTCCCGAATCACATCACCAATCTGTAGCCTTCCGCAATCACATGACA
CTCTGGTCCAATAGCATTTCCGCTTGCTGCAACCGTCGACGGTGCTGTAGTCTCGTCCTTA
TTAAAATGTGATTATGACTGTGATACTCCATCTTTACCTTGGCTTAGGACTGGCAGAGTACGG
GCAGTGGTATGATCATCATTGTTACGCGTACATTTTAGGGTTGCCAGGTGTCCGGTATTTAA
CCGGACTGTCCGGTATTTTGGATCTTTGTCCGGTATAATAATGATAGTAATACCGGACAATG
CATGTGCAAATAGCTCAATACTTTGTGCCACAGATACTGACACTGCCAGTGTCCAGTATTTT
GGAGAGCCACCTGGCAACCCTACACACGTACTGTCTGTGTAACTGCGAAGCGTGGTGCT
TCTGCGGCAGAATATTCTGCCAGAAACGCAAGAAAAATTACTTCTGCGGCGGAATATATTTT
TTGCTATGTCTCTAATGACTAAATTAACCCTAGACCTGACCCTAAATGACCGTCACGATGATT
CTATTCTATTCACATATTTATATACGGCGCCACTCGCAGTAAAAACTAATCAGAGGCGCCCA
CCAGTGCGATAAAGCCCAGGAAAAAGAAAGAACTTAAGAAGGCGAGGAGTACATGATGA
GGAGTACATGATGCATATATGTTATAGTGTAAGTAGAAGTCTGATCAAAGAAAATAATGAGAGT
GTAAGCGAAAGTTGGATTGTTTTCCACCACGGAATTGTCATGAAACTTACTCCTGCGCAGA
AGATTTAGCCAACTGCGAATGACGTACGATTGCACTGCTACTTGCACTCAACCAATGAAAT
GAAAATAATTATTGCATTCACTGTAGCTAATGCTCTCAGTGCTCGCAAATCAACTGGAGCTC
AGCACTTTACAAACTTAGAGATTGCGATCATGATTAGCATTAAGGATAAAAAAGTCTGCACTG
TGATAATGTTTGTAAACTTGAGATTTCAAGTTATGCAGATGCAAATAAGTCTCATGTTTTTCGTG
ATTAGGAAGGTTGTCACGGTGTGTTGCCAGCTCTAGTGTCAGACAGTTTTCTGCCACAGCG
TCGGACACAGAAACGTGCAGTGAACGAAAAGTCAAGAAGGCAAAGACACCAATCGGAAA
GTTAGATGATCATTGGGAAGTTGCAGGGGAGGAAATGGCTCCTGTGAAAAATCTCAACAAG
CCGGGTGAAGCTAACGACCCATATGTTCCATTTCCAGATGCCACGAACCCAAAGACGGG
TGAGATCGGAGGACCCCGTGGCCCAGAACCAACTCGCTATGGGGACTGGGAGAGAAAA
GGAAGAGTCATAGACTTCTAATGAAACTATTACGTCTTCTTTCGCTTAACAATGACTTTTGAAA
ACTTGTGTGACCAGTTCAGAATCCACTATTTTAGAGACTGGAAACATTTGATCGTCCAAATTG
AAAAAGTGGTTGAGAACAAATTAAACTGGAAGAAAACTACAACCCCACCAGCTTTGCTGAA
TAATTTATTAGTTGATATTAACGGTTTAGATAATCTTTAGCTGTGATTGTCTCCTAATTACATTAG
CCCCAAAATGGACGTCCACAATGAGCTGGAAGGAGTGTCCTCAAATGTGGACAAGGAGA

GGTAGATTATGACAAGAGTTCTGTTAAATTTGGTACATACAGTATAACCTGTGGCCATAGGCG
TAAATTTGTGCCTTATAAAGCAAGAACTACTAACTTAGTCAGATAGTATTAGTGTAGTCTTTTCT
GTAGGTCTATTACTTTGATTTGTTATGTGCTCTGTCCTGTTGTAGTCTCAGACAGCTCCATGAT
CATTTCCGGATAATATATTAACAATAATAACGTGCTTTCTGATTAAGTTAAAATTCTGTATTTGAA
TACCTCACATTGCAATAGGGCCGCATACTGCCTTGCAGGCATGTTTGAATCTATGTGCAGT
GTTATCTGGATCAACAGCAGTGGCGGTGCCAAGTCCGCTGTTTATGGCAGGCATAGACAG
GTCCATAGTCGCATCAGCAATGGCAGTCAAAATATTTTTATATATAATTTTATTATTACCAATTTT
GACTGCCAGGACAGAGGTATTTCTGCCAGGGCATTTACATAGGCCTACCAATCACTGAAT
GTCGAGTGTCAGTATTTTGCGACTTTATACGAAACTACGTCGGGGTCCAAACTCTGACCTCT
AGTCACTTCGTAATAAATTATCCGAAGACTCAGTTTCAAAAGAGTCACTGTTCATTTAACGAA
AGTATCAATTAATCCACACTGTGCAGCAAGCTGCCTTTCCAAAAGACGATTGTTGGTGCTTC
TGTGCGCCAGATTCAAACTTTTCAGTTTTTGCAATTTGTAAATTTTACCTAACTTACTGACCTAT
TGTGCACTTTGTGTTTAGTATTTAATTCATGGTGGCTATTGACTGAATGACTAATGACTGGCCA
ATTAATCTATTCTAATTGATATAATTGTAGCGGCATGGCTGTGTTACGGCGCCAGTTATAAATT
GTCGATGTTATTATTATTATTATTATTATTAATACAGCTGTGCAACAAGTTACAAGCAGCACAAG
TAGCCTATCTTTTAGAAATGCCAATTAAAGGGTTGTAAACTTGTGTCTCAGAAGACGTATAATA
AATAGCCTGTGTTAAAACTGCTACAAATGAACCTGGGATGCTTACTGGCCCAGTTTCCCAG
GCTTCCTCTTGAAAGCTGTGACTGTCACTGGACAGTGTCCAGGAGTGTGTCCACTGAGAC
GGTACTATCAAAGATCTTTGGTACCATCATGCAACCAATGAACCCATTCCACAAGTCCAATT
CTTGCACTGAAGAATTTTGTCCTCAATCAGTTTTACCTGTTTCCCTGAGAATCCTTTGTTAAG
GGGCCATCCATTTATGACGTCCACACGGAAGGGGAGGGGTCAGGCTCAGGTGGACGCA
TGTGGACGGGGAGAGAGGTCAAGCCCTATGTGGAAGTCCACATAGAAAATTAAAATTAAAG
ATCACTGACGTCATACGGTCTTCTTCTCATGCAAAGAAGTTGGCGTCTTTTTAAAATTAACTTT
AAATTAACTTTATCCTTTATATTAACTTGCCCTGCTTGCTTGATTGATTTATTGAATCCTTGTCGT
CAAGGACATGCGTATAGGCTAAATTAGCCAATTAATCCGCTAGAGGCGTTGTCAACAGAGG
CATCTGGTGCAACCACACACACAGACATTGACCATCTGGCGAAGCCAGATGCAGCCACA
CACACCTTGCCCGTGACCTTGGACATGACCTTGATCCAAGTTTGACCTTGACCCAGAATT
CGACCTTGACCCAGAAGTCGACCTTGACCCAGATTGATTTTGCAGATAAAGGATTTATAATA
GAAACTTGTTATGGGCTTTTCTTGTTACAAGAATTTCATCTTTGGACGGAATAAAAAGTGGAA
ATTTTTCGGTGATATAAATTAGTAATAAAAATTACTAATAGTACAGTTTAAATAAATTACAATCATA
TAGTCGAATTTCAGCTTTAGGTCCGGTATACGTAGGCCATTAGCCGCTTCAAGAGAGCGGT
GTGTATTTCATGTCGACCGCGTGTGGACGTCCACAAGGGGGGACGGGGTCCGGCCCAT
GTGGACAGGGGGAGGGGGTCAAAAACCTGATTTTTTGTGGACGTCATAAATGAATGGCCC
CTAAGTAACCTCTCCCACTGTAGTGCTTCCTTTCACGTAATAATCTTCTAGGCCTCGCTTAA
GATTAAGTAAACTCTGCTCCTCCTCTCCTTCAATATTGTTAGTCCACACCTATTCAACCCTTT
GTAACGTACCGTTTCTTTCGCTTGCTTGAGTGCTTGTGTGAGTATGAGCGTGATGCCTGAGT
GGAGTGAATACGTCTGGTGTCTGTTGCATTGCGAGATGATTAGTATGTGTGCCGTTCGCTTG
AGGAATCGTAATCGTAAACTCCAAACTTCTAAAGCGCCACTCAAAAGCCAAGCGCAGGG
CACCAGCTTATTCACGAGCGCTGCGTCAAATCAGAGGGGTTTTCCAAAGAATAGTCCGTG
GGAGGCTCAGGTTCGTTTTCCAGAGGGTGAGAGAATGCGTCAAATCAGAGGTGTTTTCCA
AAGAATAGTCCGTGGGAAGCTCAGGTCCGGTTGCCAGAAGGTGAGAGGAGGGAGATTAG
GCGTTAAGGCGGGTGTAGTTTAGGAGGGGAGTGGGAAGAAATGGAATCAGGTGAGTCAG
GGAAAGAGAGCTTGAAGAGGTGGGACTTGAGTTTCGAGTGGAAAGCCCGGGGGGTGAC
AGACAGAGGAGCGTGCTGAAGATGATGTTTTATGATTTGGTATGATGATGGTGGAGGTAAAG
AAAAGGTGCGAAGTTCAGGTGGCAGGTCATTCCAGAGACGCGGTGCAGTGACGGATATG
GCTCTTTTGGAGAACGTGAGGTGAGAGGTGACCGGGGGTCGAGAAAGGGTGAGACAGG
AGGAGGATCGGGTAGAGCGGGTTGGCTGAATGGTGAAAAGCTCGCGAAGGTAAGTGGG

CTGGGAGGACTGGAGGGAGTTGTAGGTTAGAGACAGGACTTTGAAGTGGATGCGCTCTG
GGATTTTTAGCCAGTGAAGTGATTTAAGGACAGGAGTGATATGATGATGCCGGGGCGTTCT
GGTGACAGCGCGTGCGAGTGAGTTTTGGATGAGCTGTAGACGCTGTATTTGGGTGGAGTC
GAGGTTGAGAAAGAGGGAGTTGCAGTAGTCTAGTTTTGAGTGGACGATGGAGGTGGCAAT
GGTGGATGCAGTTTTAAAGTCAAGCATGGGTCGGATGCGGCAGAGATCGCGTATGTGCAT
GAAGCAGGAGCTTGAGAGGTTGGAGATGTGGTTGGAGAATGAGAGATGATGTGCAGTTGAT
TGCAATTTGTGCAGTTGTAAAATCGAAATGAGGAAGGAAGAGCAGGAAGATTGAGTGAGAG
GGCAGTATATATATCTTCGTTTATTGTAAATCCGATGACACACCGCATCAGAGAACAAACGA
ACAATCAGACAATTAGACGACGTAAGCACAACATAATAGCCTTAACTATAACAAGAATAACA
GACTAGAAATGTAATCACATATAGTACTTTCATTTGAGTGGGATAAAGGGCAGGTTAGAATCA
GCTAGAAAAGGAGAAATTAGCGTTTAAACAAAATCCTTGAAATGAAGTTAATGTTATCTTTTTC
TGGGAGTGTGAAGTTATGGGCCCTAGGTCTAAGATTATATTGAGTAGCTTGGATGGGAGGAA
AGAGGCACATCAATACGTGGTTATTACAGGTGACAACAGCACGTAGTAGACGTCTATCAGC
TTCATCGGTCATTGTCTCCATGGTTGGTTGGTTGGCAGGGAGGTAACCAAGACGCCTCGAT
TTGTTGATAAAGCGTTCAATCTTTAGACGTTCATCAGCAGATGTCATACCCCACCAGGATGG
AGAGGCATAGAGCAGTCGGGACAATAAAGTGGCTTTGGCCACATTGTGTAGGGCATCCTG
TGGCAGGCCATGAGCTCGCAGAATACGTAGGGCATGCAAGGAGCTAGTGCATCTTCCTA
GGAGGCGGTCTACATGTGCCGATGCCCGAAGGTCGTAACTGATATTGACTCCTAGAACAA
TCATGGAGTCAACTCGTTCAATCCCAGGTAAAGGCGGAGGTAGAATGACAGTTCGAGTTC
GTCTTCTGAACAGCATTTCCTTAGATTTGTTAGGATTAAGCTTGAGGTTATTGAGCAAAGCCC
AACGCTGGATGCCCTCCAGCTCTTTATTGATGGTATCTCTCTTGGAACCAGGCACTATAAG
GTAGGTGTCATCGGCAAATTTAACAATAACATTGTGATCATGGACCGGATGTAAGTCTGATG
CCACGACCACATATGCCGAAGGGCCAAAGCCGGAGCCCTGGACGACACTGGCGTTGA
TGGACGCATCAACCGATTGCTGACCAGCATAACGAGTTCTGTGTTTCCGATCTAGAAGGAA
GTCAACCATCCAATTGTAGATGTTATCTGGAAGGTCGAGTCGAGAAAGTTTTTTTACAAGGG
TCGAGTGAATTATTTCAGTAAACTCCTGAAAGCGAAGTCGCAGGAACCAGCTTATTCACAG
GCGCTTAACCAAAGCAAAATCGATAGGCAGCGGTCAAGATCCAGAGTCCAGACAGGCA
GGCAGACAGTCAGACGGTTTACCGGACCTGAACCTCCCGTCTGGTCAACCAGGGCTATA
ATTATAGGCATTATAGGCCTATAGCAGTGTGCAGTTGATTGCAATTTGTGCAGTTGTAAAATC
GAAATGTGCGGTTGAAAAAGGTAATGTGTGCACTACAGGGCTGGTAAAAGCCTAAGTTTTTA
AAAAGTTTTCAGGTTTTTAGGGTTTTAGATAAATAGGCGACACTTTTTCTTAATTACGGGCTTTT
TACCGCTGATTTAGCTTCAGTTTGGTGATTTTTTCAAAGTTCACGATTCGATAAAATTCCCGG
GAATTCCCGACGGGAATTCAGGGTAGCGCGATTCCCGGGAATTCCCAAATGGGAAAACC
CGGTGGCCGCGGTTGGTTCTTGCGATATAACTTTTGTGGACGCTGGCAAATTCGAAATTGC
TATGACGAGCCGTGAAATAACAAAAATATGATGGTGCTTTATGATAGGCCTAATGTTTTATTTC
AAATTGGAGAACAACAGTATTATGGCAGTGGGCAGTGTCAGTAAGCCTAAAACTAGGACCA
GGAATGTATGGATTATTACCCTGATGGCCTTACTTATTTATCTTATCTCGCGCAAGTGCTATGT
TTACTTGTCCTTTTAAAGTGACGGCGCCTCATGAATTAAATCGAACGCGTTGAGAGTCGCC
ATTTTGTCCAAGCTTCTGTTTTTGTTGCGTGAAATTATAACCAAACTCAGTTTAAGCGGTACTA
TATTGAACATATCGAGCATTAAGCGCCTGTTTTAGTTTAACTGAATTTTGAGTAGAATTTTCATC
GTAAGTTAGAACATATGATATAAATCTGTGTATTTAAAATGACAAACAACCACTCGCCCAATA
CAGCGGGTGTGACTAAACAGCGCCAGGCCATTAATACCGGGAGGCCTTAACTTCATTAAT
CCCGCTTCCACCTACTCTGTAATTTTGCATCAATCCCACTTCAGCTCACACGGTTATTTTTC
ATCAATTCCGCTTCAACTTACACGGTGATTTGTCACCAATTCCGCTTCCATTTACCTGATATT
TTTCCTGCAATCTCGCTTGAAATTACACGGTGATATTTGCATCAGTTCCACTTCCACGGTGAT
TTTTCATCAATAGCGCTTCCAATGACAATGCCACATTTTCATCGATATAGCTTAATAACATGAG
TAGGCCTATCATAAACCACCTTGCCAGCTTGGGGTTGGTCCATAGAGTAGTGGGAACTATC

TGGAACTTTTGTGCTGGCTACCCGAGTTTGCTTCCCGTACGGAATGCTAACTCGGGGAAA
CGTATCCCTTAAGGTTGACTAATCCTCTCACTCGCTAGGCGGGACCCAGTTGTCGAGTAC
CGTAGAGGAAGTCATGCAGGATATGTTTGGTTGCATTACCCATTTCCTCATTTGTGAGGTGC
TCTATCAAAATGAGGATCATGTCGCATTTTTCAGAAAATGAGATTCGTTAACCATATTTTAGAG
CTTGAAATCAGGTTTCTAACAATGCAAAAATTATTAAGATATCTTCAGTAGAAGCTGAGATATG
GGGCCGTCCATAAAGTACGTCATGCTATTTTTTGCCAATTTTGATCCCCCTCCCCCTGTCA
CACTTTGTCACACATCCCAGGACCCCCTCAAAAGTATGTCACACATCTTGAACTCCCCCG
ATTTTTAGTAGGCCTAGTACAAAAAACCCGGACAAAAGCCCCCTGTACAAATTCTCTCTCA
AGTGTTCGCAGGGTTTTTGTCCGGGGGGTTTTGTCTGGAAGGTTTTCTTCGGGGTGGTTTTT
GTCCATTCCCCTTCTATCAGAATACATCTGTTACAACAGAAAGGTAAACATCACTTCAAATTT
TATGCGTCATGTATGATAAAAAAAATTTATTAGCGTGATGTCACATTCTCTTGACCCCCCCATT
ACCTCTGTCACAAACTGTCACACCTTCTCGGACCCCCTCCTCCCTTGAGCGTGACATACT
TTATGGACGGCCCAATGCTGATTTGAAGTGATGTATAATGAGAATATTGAAATCGAGAAAATA
GCATTCATTGCACGCTGCAGAAAGTCCTTGACAATGTACAGGCATATTGAAGCTAGTGTCC
TGAAATTTCTTTTTTCAAAAGTAAGGTTATATTATTAGTAAATCTAAAAGTAGATATTTAGAGCTTT
TCGTACATGTAAAGCATTCACTAGATGGCACTATTAGAAGGCAATATGTTTTGATTTCAATCCT
TTTATTTGCATTTATATTTATTCAGGATCTGCAACAATAATACGATTTAAGTATATTAAAATGCAAT
ATATTTTCATAAAAATGACTTCTTTCGACGAAAATATGACTCGAGCTCAGAACACACCATTTTT
ACCCTGCAGGTCATTTTCGGCTGTTTACTCAAAATTGTATCTTGCGACAAGCGCCTCATTTT
GAAAGAGCACCTCACATTTTATGAAATTTTGTTGTCTGATATTAAACGGCTTTTGTTATTAGATG
GACTACGATAGTTATGAAGTCTACGAGCGAAATTCCCCAGTGGATTGTGGGGAAGAAGCA
GAAATAGTGAGAGAAAGGTTTGTTTAAATTTGTATGATTTATTAACAATATAAGTTAGATGTTGGT
GCTTTTGTATCAAGAACTGGATTCTTAGTTATGAGGCATTTTTGTCACAGAAATAATGGAGCTA
CGAGCTTTCAGATAGCATGAGTAGGGCTGAGGCATTTTGTGTTGCACAACAGAACAGCCA
CAATGTAATGTAATGTACAAATCTTACTTCAAGATCTGTGGTGAGTGCATGGTGGTCTAGTGG
CTAGCGTTCTGGACTGCCAATTGAGAGTTTTGTGATTAAAATCTCAGCCAGGGTAGAAATTT
GTTTCGAGGTTTTTGCACCACCTGCACCCCTGTTCTACGCGGCTATGACGAGTGCACTGA
CCATAAGCTGTCAGCGGGGAGATGAAAGGGCGAGAGAGAGGGCTAGCCACCCTCTGTC
ATGTCATGCAGAGGCTAAGAAAATGAAGTCGCTAGCACTTCTTACTTGCGGCTGCCATAAT
GGGCTGCTCTTCTCTTCTTCTTAGATCTTTCTCGCAAAATAACAAGGCCTTTCTTAAGATGAC
GATAGGGTCGAATAGGTTGAAATAGGCTATTAAGCGTATTAACGCGCACATTGACTATATATA
TTCATGCTCATCTTTCTCTGTGAATCTTGAATGCGTTGTTGACAGTTGGGATACCTCTGGTGTT
ATATCAGAAATTAAACAGATTTTGATCTGATTGATCTTCTTGTGATAATGTTCATTATGAATTTAG
GCTTTGTGCAAATCTGTAAAAACAAGTTTATACTCATTCAGATTTTTGTAATTTCACTGCCTGTA
GTCGGTGCAATTCAACCGCGGTAGTAATGTTTTTCGGGTTATTCTGGTTTCAGAAGCCGTAG
AATAGACCGAGCACGCCGAGAGAGAGAGGATGAGCTGCAGTGGGAACAAGATCGAAGT
AGAGATCGAAATTACGGGTCGAGGTCGTTCGAGGACGAGTACTTCTCCGAAGGTCGTGGA
TACAGGGAAAATAACGCTAGCGGGAAATACGAGGATGCTGGTTTTGGTGGAGGAGATGAC
TTCTTGGATTGCAGTGCTGCAGGGAAGACGTTTGACTATAATCATGGCATGAGTGGTACTTC
ACAGATCAAGGTAGGTTGCTGTGCAAGGTGCCTAGAGCTACGTCTCAGCAGACGGACTTC
TGATAACAGCTGTTGCTGCATATGTCTCATAGGCGTAGACTTTGGGGGCAGTGCGGGCGC
GCTTCCCCAAAATCGGGAAACGCCCATGCATTTATCAGTTTTTACCGCATTTCCCGCAAAA
ATTTGGGTTTTCCCCAATGTTTTTTGCAAGTTCTATGCCAGTACTTCATTGTATATGTTTACTCA
GTGCATTTACGCAGTAACAACTAGCTGTGCAACGTTAGTGAATTAGAAGTAATTGCAAACAA
TACATTGTATGTGGTAAGCTGTTATGTAAATAAACAATGTAATTAACCAAGAATTGAAAAGAGA
AGTTATTGATTATCAGTGGAAGCAGGGGAGGAGGGGTTAAGCAAGACCTTTGTCCCTTAGG
AGCCCTTCTCTATTGGCCTATCGCAATTGAATAGGTAAACGCACAGTTACCAGTGACTCGG

TGTTCCTCTGTGTGCCTCTTGTTTTATTGTACATGTAGCTCGAACTTTTGCCGAATGTTCTACT
GTACTATGCTTTTATTGTTCAATTTAAAATTGTAATAAATTGCTTGATTGGGTGATTGACTGTTGT
AGCAAAAAGCTGAACTTGCGCGACGTGAGGTGAGTCGCCGACTCGAGGACAACGTGAA
CGCTGGGGTCAGTCGAGATACAAGGCCAAGCTTTGCACGTGACATTAAAGATGAACCTTC
GACGTCAATATTTCTTGGTGGAATTCCCAGAACGTTGGACGAACGAGACGTGTGTAGTTTC
ACATTGATTTGAATGTGCTTTCAATGTAGAATCTTAATTCATCACGTTGCACGTGCAGTTTCTA
CTTAAAATTAAATGCAGCTGAAAGGAGTGATTTTCATTTCGATGGTACATGTCATAGAAAGAG
TTGCATCGTTATTAGGGCTATTTTATATAGGGCTATTTTATATAGGGCTATTTTATATAGGCCCAT
TCAAGGCTGCCTATTCAGAAGCACTCCCAGTCAAACAGTGCAGTCCAAAACACTCTGACT
GGGCTGCATCAATATAAACACGCCGGAGCGCCACATAGGACGAGTGAGGGAGGAGCAC
ATTTAGCTAGCCCCATCCTAAGGGGGTATGTGGTTTAGTGATGAATAAACGGATATTCATTGA
TTGATTGTTATAAGCAGCCAGACCTAGTCTTCCTAGGCTCTGTACTTGTGTTTTTTACTGCCG
GATTCTCTCAACGACTGTCTGCAGTCAGTGGAATCATTACCTCTGTTCATTAGACATGCACA
CAAGTGGACCCCATGAGCCTGTAGCTTGAAAAATAACTTCTTCTATGGCAGGGATTTGAGT
GTGTGATCTTCCAATTGCCATTCCAGTGCTCTAACTGCTGGACTCCTGCACCTCTGCCGAT
AATTACATTTGGATTAGACCTTCTTTTGGATCGTTTGTATACTGTGTAATAATTTGTATAAGATGC
ATAACCATTGATGTGATGTAGCGCCCTGGGTGGATGCCTTGCAGTTAAATTAAATTCGGCTC
CTGTAATTATCTGCTATCATTATCTATCTTTCTATCTATCTATCTATCTATGAAATTTATATAGCGC
CCCTTCAAGGTGACTACTTTATATAGCGCCCCTTCAGGTACTACTCAGAAGCGCTCCCAG
GCCCAGGCATACTATATCTGTAATCATTCTGCAGTATGTCAGACCGAATATCAAACGGAAAT
ATTATTATATAACCCTTTACAATGAGTGTGTATTGAAATATATGGGATACCAAAGCGTTTTGCTG
CTAAAGGGTTAAGCTTGAAAATTCGTGTGCCTTACCACTTTATATTTTGGCGATTTGTATTGTA
GTTTAACATAATAATTGTAGAAACAGTTTGAGGGTAATTTCTTTCTTTCAGCTTGAAAACGAATT
GATTCGGTGTGGACTTCGTGCTAGAGATATTCGCATCATACGATCTAAAGAAACAGGTAAAT
GTATTTACGAATTTGTTCTGTTTTTAAGCAAAACGGGGAAGCTGTAGATGTTAGGGTTCTGTG
TGTGATTGGAACGTAATGTTTCTCGTCTTCGTATGTTTGAGAATCTCATGTTGTGTATGACCAA
GTTTTCCAGACTACATTGTTGAGTGTTTTATTTGTTGTTTGAAACTCGCGTTTGTCATTATCTATG
ACAGAGGTGCACAACCTTTTTGGGCCAAGGGACGCAGTTTATTATTTTTAGTGCACTCAAG
GGTCGAAGACAAAATTATGAGCTGAAGTTTTGAGAGTTGAGTATAAAAAAACAGATTTAAAAA
ATTAAACTGGCCTTCTTATTGCTTGTAGTTTGATATTCTTGCCATTTTGGATTGTAAAAAGTTGC
AAAATTGTTAACAGTTCAATGAAGTTTAAATTCATTCGTAAAGTCTTCGTCATTCAGGAGAAGT
TTCTTGTATTTTTTAAATTGCTCCAAAGGCTGGATATAACATGTTCGTGGGCCGGATCTGGCC
TGCGGGCCACGGGTTGTGCATCCCTGATCTATAAGAAGAGTTGTAAGTAAAGAAGTGAATA
CTGCTGAGAGTACAACTACTACCGTACCGTACTTTACTTTAGTCTGACTCTTTGCCAGAATG
ATGTTGTTTGATTGGATGCCTTTTGAGCAGGGTCTTTGTTGTGGATACACCGAGCCATTATGA
TGTGAATTCTGATATATCGATATCAGGAGACAATGATCCCAAATTGATTCAACTTCAGGAAAA
TAGAGCTTGTTGTATAACAACTCCGTTATAATACGGCACTGGATATGGCTCTGAGGGATTGTA
TTGTATTTTATTTAGCCTACCTAATTTTAAAATGGAATTGATTTATTTAATTTTTTAAGCAGAGTTC
TGGAAGTCCTTAAATTCATGCGTCAAATAAAACAAATTGGAAAATTTCTGACTTTGAAAAAGA
AAGTTATTGAATATCAATGGAAGCAGGGGAGGAGGGGTTAAGCAAGACCTTCCCTATTGG
CCTATCAGAATTGAATAGGTAATAGAATAGAGTCTGATTTTGAAGGTCTCATTTGACCTTGGC
CTTTTTATGTATATCTGTCTGTAAGAGCATGACTTTATCTAATGTCGAACCTGATTCTATAAATTT
ATATTCTTTGCGATTTCTGAGAAGTGATGTAAGTTGACCATTTTCCAGTTTGATTTTGAGTGGC
AGTGGTTATAATCTGTGACCTTCTGGATGCAAGGCACTGAACCTACCACATGCAAGTTGTAG
ACACGTAAACCATTCAAAAGGCGGTTAACTTCTTCACTTTGATGCCTTGCTTACATATTTATG
CTGGAAATTCACAACATCACCCAGCAGTCTGAGCTGTTTTGTCTGTACGAAGAAATTTTCCA
ATCAAAATGCCCAGCTTGGCAGAAGACTCTAAGAACAGTGCTGTCCAACCCACGGCTCT

GCAGGCTGCATGCAACCCGCCGGCTGATTTAAAGTAGCCTTTGCCAAACTGGCTGAATTC<br>CTTGCAATATGTTTAAAAATTAAGTAAAAGACAGACACATACCTTGTCTTGATATTTGGCAGTA<br>TCAACACAAATCTAGTACCGTACTCTAAAGTTGTAAACTGCAAATACAGTTTATTAAGTTTAAC<br>AAATTTTCGTGGTTGCTCTTGAGTTCACTGGTGCAGTCTGCAGCTTCATGTGGTTCGTGGGG<br>GTCAAAAAGTTGGACAGCGCTGGTCTAGAAACATTTGAGAATTCTTCAAGTTGAACATTGTT<br>AGGTCGTTCGGTTAGCCATATTAATGTGTCTTTGACAGTTTCCTGAAAGACTTTGTATAAATAC<br>TTGGACACATACATCCATGCAAACATACGATACTGACCGGAGTCTTGACAGAACTGAGGGA<br>TATGGCATCATTACAGTGCCTGTGGGATATGCGAGATGTGCTTTCTAAAGTGTCAGGATATGT<br>TCATTGCAAAATTAGAAAATAGTTAAGAGAAGACTTATACAAAATAAAGTTATTGGTATAACTTT<br>TGGATTTGCGTTTATAACAGCCATGGTCTTTTTGCAAGGCGTGGTAAGGAATTAATTGAAAG<br>CGTGTAAATATCCGGTCCTCGACAATTAGGTCCTGGCGATTAGGTCATGCGACAACTGGGT<br>ACCGTTAATTAGGTCCTGTGACATCTTGGTCCCGGTACAAAATAGGTAAGAGGCGCTTAGA<br>TTCCAAACAAATAAAATAATGCGTGTTTAACCTAAAGAGAAGCTGGTTGAGTAAATTAATATC<br>GATGCTTAAATAGAAGCTGGATTGATGAAAGAATTCTTTAGGGCCTATTAACTGTAGAAACTT<br>GATATTCAGAGGAAAATAATGTTATTTAAATGAAAGCTGGATTGCTGAAAGAATTTTTTTATGAA<br>CTAGAAGCTGGCTAAATGGATGAAAATGCCGTTACTTAAATGGAAGCTGGATTAATGAAAGA<br>ATTCTTTATTAATTAGAAGCCAAATACACTCTAAGCTTTTCGACTGTAAAAACTACGGTCAAAC<br>GACAGGCAGCTATGAACACGGCCAGAATGTAAAAATAGTGTAAATATTACGGGCCTTTATGT<br>AGACCGTTTTCTTGATGGTTATATACCGTACTTTCTACCAGTCAGCAAAACAGGCGTATTTGA<br>GGCTAAATTTGTGTTTAATGACTAAACTAACCCTAATACTAACCCTGAGACCCTCACAACGC<br>TTAACCCGACCCTAGTAGACCTTCCCGACGTGTAAAAAGAAAATTTAAAAAAAATTGGTCG<br>CAGGTGGTTTCGAACCTGGAACCTCCTACACCGAAGGCGAACCCGTTAACCGCGAGCT<br>ACGCATGCTATACGCTGATGACATGCTAAAGCGCCTAGTTGCGCGTAGCCGATGAACTGA<br>CGTCGCTTGCCGTGGGTAGTCACTTCGTATAATTTATCAACGATCTCTAAACTGTTTGATCTT<br>GATTTATTTTAAAAAATAGTTTAAATTGGAGAACTAATTGTTTGAACCAACGCATACGATAGGC<br>CATGTGGAGTGCTAAGAATACATCTCCGTAATAACCAGGTTTTTTGAAAAAACGTTTAGGTTG<br>TCTATGTTTTTTATCTACATAATACTCATACTCATAGTCACAAACACAAACTATGACACGTGAA<br>GAACACCCCATACACCAGGTAGGCTACCTTCCAGAAGTAGCCAAGAATAAATTTCTTCGG<br>GAGAATGCTGTTGTGGGAAGTCTTACAAGCAACAATTTCAATTAATTCTATTATAAACTAGAC<br>CTATTTATCAACCTTTTCATTCTGATTTAGCTTAGATGAAAATAAAAACGAACATTCAAGAGCG<br>CATAACAACAATTAGGCCTACAAACCTCACTTATGTTATGGTGGTCACATTTAAATTTGCTAA<br>GACTTTACCTTGCGACGTCAAATTACTGTCAAATGCCGTATTATTAGGAAACGATGAAATTTA<br>CGGCGAGCCACCCCCAAACGACACTATATTACCGTATTTATTTCCAACGGTCGTATAACGT<br>CACGAAAAACGGTCCATGACTGCTGAAAAAACTGTAGAAGTTTAGAGTGTAGTCCAGGGA<br>CTCGGTTGTCGCGGGACCTAATCGTCCGGTACCTAAACACTGGGACCCAATTGTCCTGCT<br>CCGGTAAATATCTAGGCCTATCTGTTTTATTGATTTTAAGGACCTAATCGAACTAAAGTAATAA<br>TTCAGGTCATGGAAGCAACCAGTTAGCTCCATCTTGGTTATGTAGAACAGGCCAGACATGA<br>TTTATTGTCTGTGAATCAGTTGTGAGGTCCCCCACCCGGTTTCCAGGGGTTCCCCATTCTG<br>CTGTGTTTTGGTATCTCTAACATACGCCACTGTTGAACTTCTTGTCTGACAGGTGCCAACCG<br>CGGCTTTGCATTTGTCGACTTTCATAGCGTGCCTGACGCGCAGAGATGGATGGAGCTGAA<br>CCAGGTTTATGATCGTCTCCTCCTCCTCGTATAAGTTCTCTGCCATTTCAAGTGTGCTGTGG<br>GTATGATCGGATGGGCTATGCAAATCTACAGCAGTGTGTCAACCAGTCTGTTGGTTCAAAGT<br>TTACTAGGCACTAATGTATTACTGCAAAATTTCAGGTTTTTTTGGGTCAGGGAATGCCCAGTA<br>TCAATGGCAGTATCAACAGAAAGTTAATATTAATATCTATTGTCTCTAAACGAGTATTGCCCTT<br>AGCTGATGCCTTTTTGGTGATCAGCTTTTGGAATAATCTCTTGATTTTTGCATTCTCTTATTTAC<br>TTCAATTATTGTCCTTCTAATGCCTAAGCTT
>6F14

AAGCTTAACTTGAAAATTAACAATAAAATCAATAATTATTTGTATTGTAATGAGATTGATGATAAA
ATGGATCAATTTTCATTGTCAAAAATGACTTATAATGACGATCGATTTACATCATTATCAGGATT
GACGTACATGTAGCAATACATGTAGGTACATTCTTCATATTGGTACTTGGGCAACGGATGTTA
TCATTACTGAAAGATAGGCCTACATTGTATAGCATACTCTCAAAATTCACTAAATATAGGACTG
CGATAATTGATTAAATAAATATCATTATGAGTGTTGACGAAACAGAATTTATTATATTTGATCATAT
CATTGGCATATATATTCGAATAATCAGCTTGTAGCCTACATAACAATCTATATTATGTCAAAATC
AAAGTGACGTTAGTCGCGTTACTATAGGCTTATAGGCTACTCTACAATAATTGTTGACTATTAA
TGTTGTCTGAATATATATAGGCCCCTATTCTATCTGAATATTAGATCTGAAGGTTTTTGATGTGA
AAAATTGTAGACATCTTCCAGCTCTTCATGAACAGTTTTACCACTGACGAGTACACATATATA
ATTCAAGCAATCACTAAGTAAAAATTTATATAGCGCCTCTCGAAAATCTCTTATCAGATGTTGT
ATAATATGCAAGTGTAGGCCCTATAAGAAATATAAGCCTATAGCACGCGTAGTGTGGAGCAA
CTGGTAGAAGAAGAAAGACCACAGGAGAGACGAATGCCACAAATCAAGACTCAGATAGAT
GGACGCGCATGCGTGTTAGCGGGTGCGTGCGCGAGGTATCTTTGTCTTTGAATGCTATTC
GTTCCGTTTCATCTCTTGTCGTTCGATCGTCGTTCTCTCGGTTCCTTTTAAATCCGGGGAAAT
CGTCCGAATGATAACAATGATCTGGAATTCCCAGGCGACTTCTATCGTCGGATGATGCGCA
CGACGATAAATGGCGACGTAGGCCTATATGATATAGATGGATGCTAACTGGGATTTGGAATT
GACAGAGAGACTCTAGGACAAGGAAGACGACGAAGAAGGGGAAGAAAGCGGAATATTG
GTAGAGAGAGACATACAGAGACAGAGAGCAAGAGAGAAGGAGAGAGAGAGAGAAAGAAA
GAAAGAAAGAAAGAAAGAGAGAGGGAAAGATACAGAGACAGATACAGAGACAGCGAAGA
GAGAGCGGTGGATATATGAAGGAAGAAAACGGCGAATAGAATTAATAGCAAAGGAGGAAG
AGAGTAAACGAAAGAAGAGAAAAAGACCAAGAAAAATAAATGCCAATTCCTACAAAACGAA
GATGGTGAAGAGACGAAGAGAGGAAAACATAGAAAAGATTAGACGGGGAAATAAAGATAA
CACGAATGATACGACGAAGAAGCTGAAATAAATTGACGATTGAAAAGAAACTAAAATACTTG
AAATGAATTTGAGAAATGAAGCAAAGATAATGAAACTGATTGCGGAATTGAATCGAACAACA
GAAGACAATGATTATGAAGAAAATTTTATAAAAAGATTAAGAAAATAGGGTATGAAAAGGGAC
AGAAGTTTACACCTTACAAGGCGGAATAGGCAGCTATCTATAGGAGATCAAGCGAATGCGT
ATTCGTCGGTGGTGGAAACATTGCAAACTATGAGTAACGGAAAATATTAACCTTACAAGGAA
CTTCGGAGACGATAATGAGAAGAAAGAGCAGAGGGAAAAAGTGCAAAGATAGGCTGAGG
AGTGGGATGAAAAGCCTTTGGAAGAAGATGTAACAAAGAGAATGGCATGCCAGACGATGG
TTCAGAAAATGCATATGCGATGGTATAGATAAAAGTGGAGAAGTGATTCTTCCTAAATTAGTAT
CTATGTTCCGCGTTCATTTCCTATGCAGTCGAGGGACAACCTTTCACGGTCCATATTATGAC
GCCTATGATGTTCATGCCATCCCACGCAGCCGTAAATATGATGAATACAAGTTGACTCATGC
ATAAATGCATCCATGGGTTCCATCAATGAACGAGTATGATGTGTTCTCGACGTGGGAATACA
ATCTAAGCACTCAGCCATATTGGGAGCCATAATAATTGTTCACTGTTGAACATAAAATTATATA
CATGTACTGCATGAGCCGCCTTTGAACACACACTTGCCTGCATGCAAGTCCAAAATGTTCA
CGTATTCATGCGCCAATACAATATATATATATATATATATATATATATATATATATAATATATATATA
TATATATTATATATATTATATATAGGCCTATATATATGCCCACACGATGCATTATATGGAATATTGC
ACTGTATATTAAGATTTATATAGCGCCTCTGCCAAAGTCTGCCGTTCAGAGGCGTTCAAAATA
ATTATACATGTACTGTATACATAGGTATTACATGCATGTACATACATGCGAAAATTTAATTATTGT
AAGATATGTAAGGAGAAATAATATCGGCATGCGTGCCTGTCTACACATATAAAACTATATAAA
ATTTACCAAATTGGCAATAACATGACTGTCAGACTTTCAAATAAAAACATTTTTGGGGGTTCA
TAGAGGGGGAGATCGGGGCGAGGTCTCCTCTTTGAATCTGCCACTGCTATCAAGCAGAG
AATGTCAGACGTAGCCTATCATACAAAGTTAAGACTGTCTATGTATAGGCCTAAATAATGACA
ATCAATCATGGGGGCCCGCCGACTTTTCTCAGAAGGGGCAAAACCTAACCTTCTTATCCT
TCATTCATTCATTCATTCAGAGATTTATATAGCGCCCCTTCAAGCGGACTACTCAGAGGTGC
TCCCAACTCCGACCCGACCAAAAAGAAAAGACTTCTATATCCACTTGTGCTGAAGGTTTTT
CGGTGTAATATGACACATTCACGTGTAGTTGGGCATTGTTGTATATAACGCATACTTATTATCA

TCATTAGTATAGAAATGACATGGGTACAGTACACTGCAGATAAAATTAGAGTATAACCATTTGA
TACGTGTACTTGAAGTTACAGTTTTCAGTTTCGTGTTGTGAATCTGAATTATTCGTATCATTATTA
CTGCGGTGCGAGGAACTATTTGAAAATCAAAAGCTCTGCCTGCAAAGGCAGATGATTATTT
TCCAGAAGGGGCAAATGCCCCCTCTTGCCCCTACGTGTGGTCGCCCATGCAATCAATCT
AATTATATCCCGATCATGTGGAGCGATCATTCTCGTCATCGATACAAGTATCGTTGTGTCGTC
GTCATCAAAACGTCATGATTTTTATTGTTGAAAGGACTCTGATCGCATCTGACGAAACCCAT
CGCTCTTGCTCATCACAACGGCAGCCAACCATCTGCATCGCTATCTTAATGATAATCTTCGT
TAGAATTCAACTCAGTAGCCACGCGTTGTCGTTCTCAATCCGCTAAGTCCCCTCTCCCGCT
GGGCCACTCTCCCCTCCCGTAGCAACACGCCGACCGACCGCCGCTAGCGTTATCAGC
CGCAGTCAGACTTGGACGCCGAGCATGGACGTGCAAAGCGAGTGGAAGAAGTAATGGA
GAAGCGGAGAGGAAACTGGAAAAGACCGAGTCTCCCAATGCTTGACCGAGGTCATCGTC
GGATTGGAAGAGACTAGAGCCGTGGACTATACATGTATGCAACGGGCGACATCTGCGAAG
GACGAAGGACGTGGAATATTAATTATTTCGGTTCCACAGCATAGCTGCGCTCTCTCATCCG
GGTCGTTTCGGATTATAAATAATGCGGGAGACGCATAAGTGGCTGAGCACGCGTAGAGAA
AATGGAGGGAATTATCGTTCCGCTAAATTCCCTCAGCTATCCGTCCAGCTTTGAGCGTCTC
CCCGTTAATGGCAAATCTCGTTTTCAATTAGAAAGGATTTGGAGCAGTTGTTTTCCATAAATA
AGACGCATAACCGTTGTTCCGGAGGCAGTTCAGCGGAGTCATTGCGCATGACGCACAGA
ATCAGAATCGTTCCTGGTTCGTTTTCATTTCTGTATTCGCATAAATTCAATACACGACAGGTTT
ATCCTTAGCATGATTCCTCTCTATAGTGGACTATTGAAAACGAATATTTTTTAATAAAACGAGA
GTAAAATGATTCTAGCGTCTGTTGAAGTTCGGCTCAATGGACTTTATAATAGAGAAATTGAGA
TTTTACCATGTCCCGCGTAGGCCTATAATCAGGCTGTAATCTAGTCCCCCAAGTCTGGGGG
GGAATCAAGGTTTCTGGGGGGGGAATTACTATGGTGGATAGTAAAAAAGTCCTAAAAAAAAT
TTTTCTGGGGAAAACACAAATTTTTTGGGGTGAAACTTCCCCCCCCCCCCCAGAGACGTC
TAGATTAATCACTGCATGCAAATCACTGCATGGTGCGTCGCCGGACTACCTTGGAACTTGC
TGTGTGAATGCGTCTTCATCAGTAAGCGGTTTTGCGCTGAGGTCGGCTGTGAAACGTGACC
TATAAAATTATGTAGGCCTACGAAAGTCGAAAACGCGCTTCCGAGATCGCGCTTTCTCGAC
TACTGGTCCCGCTGTTGGAACAACTTGCCTGCGACGCTCTGCTCAACAGAATCGGTCGA
GTTATTCAAGTCTAGACTAAAGACATAAGTTCACATAACTGTTTTCTAAAGCGTATCCGGAAC
TCGCTACGCTTTGATGATTCTGACGGCGCCTTGTAACAGCGCATGTGCTGTGTTGTGGTGC
CGTAGAAATCATCGCTATTATAATTATTATTAGTAGTAGTCAGTGAGTAATCTGGACGTCTCCC
GGGGAAATGTTCCCCTCCCAGGATTTGTGTCCCCCTCCCAAATCCGAAAAATCTGGCCTT
TATTACTATTCACCATAGTAATTTCCCTCCCAGAAACCTCGATTCCTCCCAGACTTGGGGGG
ACTAGATTTACACACTGAGTAGTAGTAGTAGTTATTTGTTATATAGTAGGCCTACCTGATTGGC
TATTATCCGTCTGTTCTTGCATAAACACGTCAGAGAACATTTCGTCTGTTTTCCACTCGATCTT
GATTCTTAGATCCGGCCACGGAGCGATTTTTCTCTCGGATCGGAGACGGATCTTGTAGGTG
ATCGTCTGCGGAAGGCTTTCCGTCGAATAATCCTCATCGACAAAAACGATTGCGCCAAAA
ACATCTTCGTCTGTCAACGCGTGGTTTTCTAGCGCAGTTTCGGAGGCAAAACCCAGATCTA
AGACAGACGAATATCGTGCGTCATGTTTGAATTTTCTGTCTGCTGTTAAAACATCCCTCCCTG
CCTGAAACTCAATTTGCATCACATTTATAGTTAGTAACCCTCGGGCTACCGGGGTGGTCGA
AACGACCCCCCTCCCTACATGTATGTTTTTTGGGATCTAGGACTTTTTGTAAATAAAGTCTGA
TTTTGACGTTTTAAGGATAGCTATAGCCAGATCTTTTCCGCACAGTTTGAGATATAGATCATG
CTGATAGCATAAAGGGCCTATCATATCAAAGTTATAGGGCTATACCCTGTGAGTTATCTGCAT
CTTGGCTCAGCGAATGTGTTAACGACCGGTATGAAATTTTTAAACGCAGAGGGTAAAAAGA
CGAGTGCACGAGGTAGTAGCGATTAATGATACTGTACCTGTAGATGTGTTTGCGTGTTGATTC
TGTTGAATGATCGTGCGCTTTGCAGCAGAGTAGGCCCCTATCCTTGGCGAAAAGATGACTA
AAGTGGTAAAAGAAAGTGCTGCGTTTCTAGACGCCGGTATATGTAAGATGCACGAAGGATA
CGCTAAAGAATCCTGGAAATGTCATTGAGGTTGTGGATAGGGTCTGAAGTTAGAAAGGTCTC

TCGTTGCTATTTAACAGTAGGCCTAAATACAAGACTATAGCCTACATGTATATCAGCTCAGCT
ATGACAGAAAACACTACAGCAAAAGACAGGTTAGTAAATTACATGTAGGTGTCTAGAAAGTT
AGAAAAAACGTCGGAAGAAGAAAAGCAACCGGTGTAATTACAATAATTACAATGCCCACTC
GGATGAGTTCCCAGAATAGCAACAGACGTGTGAACTTTGAATAAGAATGAATGAAGATCCC
AATTAACGCAGTCTGTCACATATATATATATTATATATATATATATATATTATATATATATATATAT
ATATATATATATATATATATATATATATATGTATGTGTGTGTGTGTGTGTGTGTGTGTGTGTGT
GCACATGTGTGACATGAATAGCCAAACCATCGAAGAATCAAAATGTAGCATTCGTATTTAGT
GGCAAACAGAATAATGATTCAGTTCCATAGGCCGAATTCACTTCTACATTCTATCAACAGGC
TTAGGCTTTAATAACGAGAATTTCTGCGGAAGCCAATACAATGACGTAATTGGACAGCACG
GCCAGTGTTCATTAACCCTCGGGCTACCAAGGGGGGGGGGGTCGAAACGACCCTTCCC
CTATTTGTTTTGGATATGGCACATTTTATACATGAAATATAATTTTGACGCTTTAGGTTGCTTTAG
CTACAATCGAGATCGGTTGTGGAATAGTGCTGTTTCAATTGACTGCGTAACTTTTCCGAGGC
TCCAGCTAAGCTTCGAGACTAAACATACGACAAACACAGAATTTCTATTTTGGTTAAGTAGC
AGGAGTACAGAGCAGTAATGTAGATCTATTCCGCACAGTTTGATATATTGTTGCGAAAATCGA
ATTCTCGCTACAAAAATATATATATTAGTTCAATTAATGATTATTATCATTTACTGTCGTTGGCCC
CGGAAATTTTCCAGCGATGACTCATAGCGATGAATCATATTTGTCGTTGCCTGATTTTCGGCT
GCCTTACGCGTGATCTCCACGTGATAGGAACAGGTGCTTGCGGCTGTCGCCTGTAGGGG
GCGTGGGAGGTCGCGCGTGATGGAAAACTTCTTTTAGTCACCCGTTCAGGCGAAATACC
CTGGATTGGCCGACTCCATATAAGGCCCACACTCTTTTGCCACGTCAGTTCCTCCAAAGA
CGACAGCCAGTGAAGACGTGGAGTTCCGATGAAGACGCCAGCCAGTGAAGACGACGAC
GCCCGCCCAGAGAAGAAATGTCCCTTCGAGTCCAGTTTCCCCGAATGAGCCTCCACTTC
GCCCGCCCACTTGCTGAAAGGATATCAGCGAGTGTGGCAATGTTCCAGTTCGATTCCGAA
TGCCCCTTTCTGCGTCTTCAATGCTGACGGATATCAGCTGTGTCTATTTTCTATTCGTCGATT
GCGGTGTATCAATTCGAATCCTACTGTTCGGCAGGAGCCGTTATCGTTCTCGTTATTTTTATC
GCTGAGTTCAGTGCGAATTAGTTCGATTCCCTAGATTGAATTCCGGCAGGAGCCGTTATCG
TTCTCGTTATTCGTATTGCTGAGTTCAGTGCAAATTAGTTCGATTCCTTAGATGGAATTCCGG
CAGGAGCCGTTATCGTTCTCGTTATTCGTATCGCTGAGTTCAGTGCGAATTAGTTCGATTCCT
TAGATTAAATTCTGGCTGGAGCCGATTACGTTCGAGGTATTATGGAAACGGTGTCATAAGCG
AATGCGTAACGCTGCGGGCTGTCCTTGGGCCCCACGCACACTTCGGATTAACCATCGCT
TCTATCTTCGCTGATTCGTCGAATGGATGTCGTCGCCTGCTCCTCCACGCACGCTTGACC
CAGAACCGTGACCCAGAACCGCCAAGATCTTTCTTTTAGAACGAGGTTTAATTTCATATATT
CATTTCTTAAGAATTCAACTTGCCTAGTCCCGATAACAATAATGATAAGCATCGGTGTCTGAT
CTACAGTCAATATAATAGAGAGAAACGTAAATGATTACGATGTCTTTGTCTAATTTGAGCTTGG
CTGAAGAAGTGGAGGATTACTCGGATATGCAGCCTGAACAAGTAAAGCTATCTTTTGCCCC
TGTTTCGTCAGCTCTTAGTCAGGTGATTGATCGACCAGGTTCTGGAATGAGTGAAAGTCAGT
TAGAAAGGATGTTAGAAAGAATGTCTGAAATGAATCAACAGATGTTTGAAGGAATGAATCGA
CAATTAGAGAGGATGTCTGAAGAGACGAATCGGCAGATCTCTGAACGAATATACGAGAACC
AGGCCGAAACGACTCGCCAGATAGCCAATGTGTCTAAAATCCACGAAGAACTCGTGAAG
CGGCAGTCCTTACTGGAAGATTCCCTCCTCAGCGTGAAAGCTTCTAGCCCGGATGTGGTT
GATCCCGCTTCGAATCCTGGTTGCAGTATGATTACTGCGGCTGCCGTCCGACAGATGGCC
GCGTCAGAAGCCCTCGGTCGACCGGAACTTCCGGTTGGGGGAACTCACCTCAAAGCGC
CCCCTACTGTTCCTCGAAGAAGTGAGCGCCTAGCGACTAAACCACGACTGGACTATAGG
GAAGCTCTATATAAATTTCCTGACCACGACTACACTAAAGCCAGGTTGGGCCAGCCTTTTC
CTCATCCGTCCGACAAAGGTCCGCTGGAAGATGGGGGTTGGCAATTAGCAACTGCTGAC
GTCAGAGGTGACGTCATGGACCCCCGTTTCCGGTACAATATCATGCTGATTGCCCATGCC
ATACCAAAGTTATACCCGATATAGTCAAGGCATAGTGGCATACTATCATAAAATCTCACACAC
TGTACAATTAGCAGTTATCTGAACTGGACGGGTGCCCTTGGTATATCTTGCATAACTTTCGTA

TACATTCACGTATTCGTAATAGGAATACATCACTTTTGTGCAGAAATGAATGCTGAATCGAATT
ATTATTAGTCGATATGACATGTGGAGCCCCCCCCCCCCCCCCCGGTAGTCAATGTCGCAAAA
AAGCTTGGTAGCCCCAGGGTTAAATAAAATGAATTCAAAATCACTTTATTGCAACTCAAATAC
ATAACAGTTGCCGCATAGGTATATTAACCCTCGGACTACAATTAGTATATGAAATATAGGCCT
AATTGTGACGTTTTGGGTAGCTGTAAGGAAATCAATTCCACGCACTTTGATAACCCATAGCC
TACTACATGTATACAAAAGTTATATCCGATATAGTCGAGGTTTATAATAAAAAATTCTCAGCAAA
CAGTACAATCAGCAGTGTTTTCTTAAATGGACCGGAGCCCTCGTTATACATTCATGTATTCTG
AAACGGAACACAACCTTTTGTACAGAAATAAATGCTGAATGAAATGCTGTAGTACTCAATATA
TGTGGAGCCAGACCAAAAATCAAGAGGGTCGAACGACCCCCCGGTAGCGAATGTCAAA
AAGAGCTTGGTAGCCTGAGGGTTAATAACGTGAATGAATATAGCAAGTGTTGAAACACAAAT
TCATAATTTGCGTAGTTATCAAGTGTTACTCGAGCATACGGCAGAGGTGGGCAAACTCTTTT
AGCTGGGCCACATAAAGTTCATCATTGGAGGGCAGAGCGAGCAATTCAGACACTTTTCTTT
ATTATAATGGTCAAACAATAAGATATTCTACATAATATAGGGGCTTAACTAATTAGAATAAAACT
CAAATACTATCAATGATGATATTCAACTCCCTGTGGGATCCTGTTTACATAACTAAGCCAATC
AGTAAATGTAAAAAGCTTTATTGCTGGGGGTTTCTCGCGGGCCGGATAAAACTTGTGTGCG
GTCCAAATACGGCCCGCGGACCGTAGTTTGTCCAGTTTGCCACCTCTGGCATACGGCAT
ATTCAAAAGTGTATGTTATTCAGGAATATATATATATATATATATATATATATATATATATATATAT
AATTTATATATATATATTAGCTGTACCTGGTAAAATGTTGATGCCCATGAGAGTCGCCATCCTAT
TCACAATTCTCGCCACGGGGGGTGTGTTGGGGGCGTAGGCAAGTTTCTTTGGTCCAACTT
CCACGAATTTAGACAAATTCGTGGTAACCAAGAAGCTATCGAAGGTCTCTCCTTTTGGCATT
GATTCAATTGGCTTTGAACTTCTAATCCAGGGGAGAAATAGAGATAGGACGATCGGCAGTA
GAATGATGAAGAACGTGCGCTTGGGTTGATGCTTCTGGAGCAACCAGTTCTTCCAAAGCA
GCAGCAAAAACTGTCTCCAGCTCCTGCAGCACAATTCACCATCATCTGCACTTTTAGGAG
GCAGCTGCCTATCGCGCTCAATAACATCAGTATAGGCCTAATGTCTAAATTATCGCTTCCGA
TTCGGTCATCCAAAGAAAACATTCATTTGAAGCATCACGTACGATCTATACTTTACTATCGGT
GTTATAGGGGGGTATATGCAAATTATACATGTGTGGTCTACTCCATTCATGCTCAAGAGCTCT
CGGCTGTGAACACGCAAGACATCAATGTATACATACGCCCCTAAGCTGGGCGACTTCCAA
CAATGACGTATACCATAGAGTCATAGACTCCCATAGTAATCACAAGTTGAGGAATTTCAGTAA
ATTATTCAACTGTGAACATTAAACACCAACTCAATGATCATAATCATCACAACCACTACCGCA
ACATATACCTACCAGTGTAGGCCTACACATCACGGCATTATTATCACGGTCGCGTCCTCTAT
AGAGCGTATACGCCCTATATCTTCTCCTCTGCAAGGCATTATTTATATGACCTCGACTTGAA
GTCAAGGTCGTATTACTTGCATAAGCTTAGGCCTAATTGTATGCGTTTCCATTTCTACAAAAC
TATTATGCTTCATGCTGAAGTATGCCCATACATGAACCCACCCAGGCTGTAATCTAGTTCCC
CAAGTCTGGAGGGGGAGGGGAAATCGAGGTTTCTGGGCGGGGGGATTACTATGGTGGAT
AGTAATAAAGTCCTAAAAAAAATTCTGGGGGGAACACAAATCCTGGGGGGAACCCCCCnn
nnnnnnnnTATATATATATATATATATATATATATATATATATATATATATATATATATATATATATATA
TATATATATATATATATATATATATATATATATATATATATATATATATATATATATATATATATATATATATAT
ATATATATATATATATATATATATATATATATATATATATATATATATATATATATATATATATATATATATAT
ATATATATATATATATATATATATATATATATATATATATATATATATATATATATATATATATATATATATAT
ATATATATATATATATATATATATATATATATATATATATATATATATATATATATATATATATATATATATAT
ATATATATATATATATATATATATATATATATATATATATATATATATATATATATATATATATATATATATAT
ATATATATATATATATATATATATATATATATATATATATATATATATATATATATATATATATATATATATAT
ATATATATATATATATATATATATATATATATATATATATATATATATATATATATATATATATATATATATAT
ATATATATATATATATATATATATATATATATATATATATATATATATATATATATATATATATATATATATAT
ATATATATATATATATATATATATATATATATATATATATATATATATATATATATATATATATATATATATAT
ATATATATATATATATATATATATATATATATATATATATATATATATATATATATATATATATATATATATAT
ATATATATATATATATATATATATATATATATATATATATATATATATATATATATATATATATATATATATAT

ATATATATATATATATATATATATATATATATATATATATATATATATATATATATATATATATATATAT
ATATATATATATATATATATATATATATATATATATATATATATCCCCCCCCCCCAGAGACGTCTA
GATTAATCACTGCATGAACCACGTTTCATGAAAAGCATTCCGATTTGACGAGGCTCTATGTG
CATGAGTCTATGACAATAATGACAAGAGAGGCTGTTGCCCTCGACAGAAATTGGTCTTTTCA
TACTACAGCCGTGATTCCGAGAGGAATATTCCCCCACGAGTTGTGTTCCCAAAAAATGCAA
AAAACAGGTGCTCATTAGGTCTACTATCCCCCGTTATAATCTCCCAGAAACCTTGATCCCG
AGACGCGGGGACTAGCTCTATATTACACGCTGAAGCGCTGCCTAATAATTTAGAATATTATA
GCCACATACACATGGCTCCAACGAATAAACAATTTAAGTGAAATCGTAAAACAAGTGGAAA
AGGAGAATCCGAGTGGCTGACCCCTACGCAGAGAAGGATTAATCCAAGCCTGAAGGAGA
AGAAGAATTTAAGTTAAATCTTAACGATGTGAATGTGAGAAAAGCATACGATCGTCCTGCGTT
GTACATGTAGGTCTGTTCAGTTGGCTGGACCATTACGGCGACGTAGTTAGGTCTATAGCTGT
CGAAGAAGATCCGCCAGAATCCAGAAAGTGTGACCTAATAGTGACAGTTCGGATAACAGA
TTGGTTTATGTAACTTCGCAAGATAGCGTTGAAATTATGTTACGACTGCTTGAATGTAAGCCTT
GATGAATCAAATTAAAATCAAATGCAATGCTTTCTAATCAATACTTCAACGCCGAAATACGAC
ATAGCGTGTAAATTCTTTAAATTTGGCTGCAATTTATTAGTAAATTAGTCAACACAAAATTAATA
ATAACGGATGCGGTTTGTTTATGACGTCATCGTTTATTTTCGGATGTGTGCGAATAATGCAAG
GACGCGTGTTACACCTACTCTCCGCGTTAGAATTGGTAGGCCTATTTATCGTCTATTCAAAC
CTTTTTCATCAGTTTTTATCGTGCTTCTGCAAAACATAATAATTTTTCACCAAGTTTTATCTCAC
CTACTCAAAATGATAGACTTCGGCGACTCTTCGCGGCTTATGGTGATAATGGTGGCCAGTC
GAGAGTCCTAGGAAAAGCATTAAAAATGATTAGGCCTAAACCTTTAACGCCGAGAGTGTAG
TAAAACCTATGCATTTTACAACACTCTTAGCGTTAAAGGGTTAAGGACATATGGGCGTCCGC
AGCGGGGCGCAAGGGGACGCATGTGCGCCCCTAGATTATATTTGCAGAGAAAGTTTGATT
ACTTGGTTGTAATGATTATTACAACACTATTTTTAACGTTATTTTCGGAAAATACCAAAAATCAA
TCCAGAAAGTGAAATCAGTAATTAGGTAGCAGCTAGGGTATAGGCCTACCGACCATTAAAA
CACGGGCGATTTACAAGTTGGACCTATCTTTGTGGATTCGTGTGGAAACTATTTAAAATTTGG
GCCCCCCTAAAAATTTTCTGAGGACGCCCATGGTTATAAGGAGGTTCAAAAAGACGCTGA
AACGGCCTATGTTCTACAGAAACCACGAGTTAAGAGAACGATTGTTTTTGTTCAGATAGATC
GATTGATTGAAGGCCAGAGCAGACCTAGACCTAGATTGGTGACATTGGTCTGTCTGAGGAT
TTCAGACTCGATGACTCGCAAATTCTACACGATTGAAAAAGGGTTAAAGTGCAACTCGATC
ACCAATCCAACCACCAGTGTGTAATCTAGTCCCCCAAGTCTGGGGGGGGGATCGAGGTTTC
TGGGGGGGGGGGGGGAATTACTATGAAGGATAGTAATAAAGTCCCCCCCCCCCAATTTCT
GGGGGAAACACAAATCCTGGGGGGGGAACTTCCCCTCCCCCCCCCCCCCAGAGACGTCT
AGATTAATCACTGTCCAACCACAACCTTGTCAGAACACGAATAAATAATTATTACAGGGTTTA
TATGACGCATGGCGCGTTTAAAAAAAATAGCGTCATTCAATTTATTGTTTCAACTTTCAACAAT
TTACACGGTAGTTTCACGACCCAGAAGTAAGAATTATCAAAACGTGCTGAAGTAGACGCGC
AGATCGAGTAGCTTCATAGCCCTAATTATTCGTTTTCAAACACAAACCCATCGTAAGCAAAA
TGGACACGGTTAAAAAATTAGAACATTTGACAAAACTCCTTCTGATAGAACTAGCATATGACA
CAATCTCTCAGCCACAATTAAATTAGACAAAATTAAGTGGTCAAATATACCACAGCAGTAATA
AGTTCACACAAACGCATACTGTAGAACGATAGATTCTCGCTTCAACTTCCAGGTTAAATATAT
GAGTTGTAGAATAATATTTCAGATAGTCTCATCATAATCATCATTCATTTATTCATACAGCACTC
AATGAAGAAACCACTTCATAATGTTTCTGGTCCATACACAACTATAACAAGACAAAGGATTTG
TAACACCTATATCAGAGCTGTCCAACTTCCAACCTCATGCGGCCCGCGGATGATTTAAAGT
GGCCCGCCCAAGCACCAGGAATTTTCAATTTTGTAAAAAATATTTAGATATCAAATATAATGT
TATTAAAATAGTTTTATTTGTGTCAAAATTGGGTTTTCTCTGGCCCACTTCTGCAGATAAGTAC
CCATAACGTGGCTAATCATTTGAAAACGCGAGCATAAAATTCAGGTCCGATGACGCATAAC
TTTGTTGACCATGGGTTTAATAGTGCGGCCCTTGGCCCCAAGTATAAGTTCTTTGGACGCC
CGCGAGGTTGGAAAAGTTGGGCAACACTGACCTAGATAATATTATCTTCAGCTTTGGAGAC

ACAGAGAATCTGATTTTGTTTCTTGGAAATTGTTGGGGTCATTCAGCATTTATTCTTCCATCAC
AATTGTATCCAGCCCTGCAAACTCCATCAAAGTGTTTGCTGCTTCAAGACCACTTTTCTCTC
AGGGTCTCTTGCACTGGAACTGAGTGGATTGATGAGCATGGTTGAGAAGTTCTTTACAAATC
TATACATATAATATAATACAATACAATACAAGTATTAGGGTGGTCTGTTTACTGGGCAATACAGT
ATCATACAAATCCAAGATATTAATGTCATACTCCCAATTTACATCTGACTGACTCACTCTCTGT
CCTTTTTGTAACTGCATCTAAAATCTGTCTATTGTATCTCTACCTCCTAAAATCTCTAGAAGTAT
ACTTTACGTCCATGTTCTACTCTCGGTCTTTCTTGACAAAAGTACATAATTTTCATTGTTGCCTA
TCCCTTTCATCCTCATCATTGTCTAGGATTCTTTTTAGGTGCTCTTGTAGCAGCATCGCCGAG
TTTTCGCACCCAAAAAATTGTTTACATTATTATTATTCTTATTATTCATAGACAGCATAGGTTAAG
GGATGATAGAATCATTCTCAGGAGTAGTTTTAGGTTAAAATTCTGATGCTTGCTCATAAAAATC
TCCATTGTTTATGGATGAAGATAAAAACAGCAGAAAGATCATCTCAGGTTAAAAACTGGCAA
TGACCCATCAGGATCAGACACTGATCTGTTCCGTATTAAACGATGAACAAAGAAGCAGTTA
CGATATCAGCAATTCCCGACCTTACAAGAAGAGCAATTATTAGATTAACCTTACAAGAAGGG
TAATTGAAAGATTGACCTTACAAGAAGGGCAATCAAAAGATTGACCTTACAAGAAGGGCAA
TCAAAAGATTGACCTTACAAGAAGGGCAATTATTAGATTGACCTTACAAGAAGGGTAATTGA
AAGATTGACCTTACAAGAAGGGCAATCAAAAGATTGACCTTACAAGAAGGGCAATTAAAAG
ATTGACCCTACAAGAAGGGCAATTAAAAGATTGACCTTATAGAAAGGGCAATCAAAAGATTG
ACCTTACAAGAAGGGCAATTAAAAGATTGACCTTACAAGAAGGGCAATTAAAAGATTGACC
TTACAAGAAGGGCAATTAAAAGATTGACCTTATAGGAAGGGCAATTAAAAAGATTGACCTTA
CAAGAAGGGCAATTAAAAGATTCATGATGATGAGCATAAGATAAAGTAAATATTGGTGATTAA
AGGACCAGCGCAACGTCCATCAGATCCAAATCGTCTGTCACCAAATCCTCCGAGTCATCT
TCGCTGAAAGAAACACCGGCCAAAGTCATCTGGTTGTCGCCAGATTTCGACAGAGAAGA
GAACAAAGATGGTGGCTTTGCAGATGAAGATGGGCTTGAACCATCCTTTGGACTCGCCAT
CAGCATTTCAACTTGCGCTTCTTGACCCGTCGACTTCTCTGCATCGTTGCCACCGCACTCT
CCCAGTTCCGCCCCTTGCCGTCTCTCTTCCACGTGTTCCAGCAGCTCAGAGCCATTATTG
CACTTGTCGTCGGAGTCTATTTCGTGCTCCAAAAGGTTAATGTCTTCTCTATAGTTATCGCTT
CGAGGACTTGAGGACATGTCGAAATGACTGACTTCTGGATAAAGACCTTTGTCGTTTTCCTT
CAAACTGTTTGGCAGATTCTGACTTTTGACGGTCGGAGACAACACAGGCCTGCTGATGCA
TGAATCAGTTTCGGCGTCCTTAACGGCATGAAGGTTCTCCGATAACCAAGACCAAGCCCG
TTCTGCGACAGGGTCGTCATCTATGGACTCGTTAAAAGACTCATCCGCTTCACAGCTTGCA
GCTGCAACGACTGCGTCGCAAGCACTCTTCAGTGAACTTCTTTCCACTTCAGTCACTCGG
TCTCTCGATTCTGAATCATCAACCGTTAGAGGATTGTCCTTGCTGAAGTCTACTATCCTGAGC
AACGCAGGTTCCGAGGGGCCTGAACTGAGAGGGCAAAGACCAGATGATCCTAACGTCG
GGACAGCACAGTCGTCTCCAAGAGGCGAAGGAGATCTCGGTGGAGAAAGCAAGCGCTC
CACGGCACCGTCCTCCTCCCCGGAAGACTGCGGCTTCGTTTCGACAACCGCATCCGTG
AAATGGCGATCCGGAGCATCACCCGGTTTGGAATGATGGCTGGAGGCGTCGTCTGCTGC
CACTCCGCAGGAGAAGGAAGACTCCGATTTCTCCCTTTGTTCCGTATCGACCTCTTTACCG
GGCGACGGACTGGAGATGGCAGAATGAGAGGAAAGACGACTAGGAGGGCCTCTGCTCG
GCGTCGGAGTCGTCGGAGAAGACGACAGAGAGGATGACGAGATGCTGAGGACCTCGTC
TGGTGCAGGATCCGGCATTTGACTAGAACTATACGTATCACAAGACTTAGCGCTGGCGTTG
ACTAGAGCGCTTGAAGGTCTCGGTTTATCGACTGCGGCCGCCAGTGGCACTGAATGCCC
AGACAGATCGTTCGTGAACAGCGGCTCAATAGAGACGCGTGGCTGACTTATGACCAACGA
ACCACTTAGATCGGTCCGAGCCTTCATTCCCGATGCTGGCCAGTGTCCAGCGGAGACTTT
AGTACCTTGCATAGAGGCAGAACTTCGGACCGGCAGTCCGTTACCCGACGATTTGGCTTG
CGATGGATTCGCTTTCCCGGCTACCGATGTCGCGGTCGATGCCTGCGTTGTTACCATGAC
AACCTTTCCCTCGGAAGAGGTTCCTCTGCTGGCATCGCCTCTTCCTGGTTCCCCCTTTTTC
TGCAACTGCTGCTGCCCTGCGTTCTTCGCGACGCCGGCATTTTCTCGCGTCGGCGTGCT

CGGCTTATTGCCCTCGCGTATGATTTCCAATCGAATGGCATCGTACAGTTCCTTCTGGCCA
ACAGCAGCACTGCTGCCACCATCGCCAGTTCCACCTCCACCTGGAGTGGTGCAGGTCG
TATGCTGCTTCTTCATCAGATTATCAATGACGACGAGAAGGGCTCCTTCTTTACTTCCGAGA
CGATTTCGGCCGCCGTGGCTCAAAGTCGTCGCTCCTGGACTTGGTTTCGTCAGCTGTGTT
GACGACGAGGCGACTGTATTTGCAGTCGAACCACTCGGAGCTGCGGAAGTCACAACGA
CGTGCTTCAACTTAACCGTCGGAGTTCGACTCAACTTCTGATCGACGACTTTCGAACCACT
CAGAGCTCTCCGCCCGGTGAAGTCCGACTTCATTCGACTCATTCCACGAATCGAAAGATT
GCCCGGTCGGACGGATTTCGGGAGTAGCACGGATGATGGTGGGGGCTTGCTGCCGCG
GGGAGCAATGGAGTTGACGTGAGATGAAGAGCCGCTGGGAATGGCTTTGACCCCGGTC
GACTTATGCGTCAGGATGGTCGAGCTGCTCTTCACCGGATTATTCTGAATCGTCAGCTTGC
CTCCGTAGTTGGAGATCTTGATGGTGTTGATCTTTGCCGTGGATGGAGGCGTCGCCCGATC
CACCAGCGACGATGACGGAAGGCTGTTTGGAGGACGTTTGACAACCGCTTTCTTCCCAG
ATTCGTCTCCTTTCTTTGGCTTCTTCTCCATGATATCCAAGCCTTCCTTTTCCAGCCTCGGC
CGCTTCTCCGCTCCCGCAGGTCGCATGAGATCCACCCTCTGTTGTCCGGAGGACGGTG
CCTTGATGAGCCTAGGCGAAGACGAACATCGGCCAGACGGAGGAAGTCCCATGACCTTT
GACCCCACGATGACCCCAGAGGTCAGGCCGTCCACTTCGGAGTCGGCCGAGAGGTCG
GAGGTGATGTCTGAGAGACTGAGGCTGAGCTTGGAGACTCCGCCGCCGTGCAGTCTCCT
ATCTTCCTTCGTTCTCTCCTTCCATATTCCGTCCGGGGAATTGCTCGGCACGTCGAGCGAG
GGATCTCGGAGAATCGTTCGCCGACTCAGCTTCACCCGGATTGGAGGCTTACTTCCAAC
GGACTCTCCTAAAACAGACGAGGAACCAGCAGCAGATTCCCTGATTTGCTGTGCGACAA
CAGCACCCACATTATCAGGGCTCTCGTTCTTCGACTTGCTCCTGGCAACCTTGAAACAGA
CTGGTCCCGGCTTATTCTCGAGACTAACATTACTGACCTGGTCCTTGAATTCGACCTCTTCG
GAGGCAACGTTCTGCCTCAGTTGTCCATTGCTTAGCGATTGCATTTCTCTGTTTACTTCAGA
GTCGGTCTGTAAAGACACCTGCCTCCTTGGACTGTTGTTCCTTGCATTACTAAAAGGTGTCT
TATTGGAAGTTGCGTTTAACAGGAAGACGGTCGATTCTTGTTTAACGTGCATCCCTTCGCTC
GTCTCTAAGGCCAGAACGCTAGAAGAGGAGGAGGAGAGAGAGGAGGATGAAGAGGAGG
AAGAGGAATACGAGTTGAAGGAGGATGCTGACGAGATAATTGGAGATGCATTCGTTTTGCG
AGCGGCTGGGCTTCTGCGTTCCTTACCGAGTGAACCGTACTCGGACTCTTTTGATGTCTGA
GAGGGTTTTGCTGTCGAGGCTTTTGGTGTCGGTGAATCTGAGAGGATGCTTGTCAGCTCTG
GGTTACTTATCATCTGCTCGCTTATCCCCTTAGATTTGAGATTCTGCGCAAGGGTCGTGATT
GCTAGTGACCTTTCGGTAGACGATGTCCACTGGCTGATGTCTGGCTTAGCAGGAAGAACT
GGCATGGCCGAACGACTGCAGTTGTTTGGACTCTGAATATATCCGCCGATTCTTGGTAGGT
CGGCGCAAGAATTTACGGGCGAAGGAGTGTTGAGTACGACCCTGTCACTTTTGACAACTG
TGGGTTTCATCAGGTGGGCCGTCGAAGGTAATTCAGCCCGAAGTGGACGAGATCCAATTC
TGGCGGCACCTCCCAGTCTGTCCTGCTTTGCGGCCTGAGAGACAGGAGTGACGAGCAA
CGCCGGGTTTATTGGAAGACGAGAGGGACAACTCGACAGGATGTTGTATTGCTGGTCTTC
CTTTGGCTTTCGCTTGGGGCTGTGACCTACGCTGATGGGGCTCTCAGACTGACGTTTTCGT
GCCTGCTTCGCCGGTCCTCCTCCGGTTCCTCCCTTTTCGCCCCCTCTCCCGGGCGCAC
CCGGTCCCGAGAAGGACGCGGAAGATGAGCCGAGACTGAAGTTGAAGTCCCCGCAGA
CGGCAGATTGGTCGAGCAGACTGGCCAGCATCGGATTGCAGAGCACCTGGTCGTTTCTG
ATCAACTCTGCGTCGCAGCTCCTGGAAGCGTTCGTCTCCCTCTGAAAGGCCAACGACTG
CTGAGACTTCTTGGACTGCGACTGCCACTCGGCCATCTTCTTCCTGGCCAGCTGCCTCC
ATCCCGGCAGTTGGCTCATCAGCGGCGTTCTCAGGTGGAGAGCCGGAGAACGACATTTC
CGTTGCAACTCCGGAGATGCCTTGCGCCAGATTGTCCTCAGCAATATCGGCAGAGACAG
CGACCTGGCAGATGCAAAACACAACAGATAAGCACAGATTCACATACTGGTATATTGTGGG
GTGCGGTGGCACAGTGGTGAGAGTTACAGCCTTGCGATTCCCAGTCCAGCCGCCATGTA
TGAACCTTGAGCAAGTCCTTCACCTACAATTGCTCACAAGGGTGGGGTGTTAACTCCGACT

CAGCATCAGCACTGTAGTCGGGAGCGCCTTTGAACAGTTCATACTATGAGAGGCGCTATAT
AAACACACAAATACAATACAATATTCGCATACACAATTGGCTATTCCAGAGGAGAACAAACT
TTTTATTACCTGGGCCACTTGAAGCTCGCCAAACCTTTGGGGGTCCGGAAGACAAATTTCA
TGATGGTCAAAAATGAAGATATCCTACATACTATAAATAATTAACTAATTAGAGTAAAACTCAAA
TACTATCAAAGATGATATTCAAGTCCCTGTGGAATCTTGTATATACAAAAATAAGCCAATCAGA
AAACGTAAAATGCTTTATTGCTGGGAATTTTTGCGGGTCTGATGCTTATACCGTTTGCTGATA
ATGTAATAAAGAATATTCTTTCTTACTAACTTAGCTACTTACTCACTTACTTACTAACTACAGAA
GAGACATTCATCAATATCTCTGTGTTCCTGTTATGACTAGGTAAGAGAGGGACAAGTGTCTC
CTTCACCTCTGAAGCACCCTGGTTGCAAATTCATCGGGACAGATGATCCCATCATCAGGC
ACAATCATTCGACACTTGACGCACGTGATGTCTGTCAGGTCGACATCCACTGAAAAACAAA
ATAAAAATAACAATGACAACAATTTATATGGTGTCGTAATACAGTCATACTATTACAAGGACAC
TTGACACATCCAACATCAAAAGATGTGTGGGAAAATGGCCTACAACAGTGCTTCTCAAACT
TTCTGATGTTGAGGACCGGTGAACGTGTTAACGAAATTACTGCGGACCGGTATATTTTAAAC
TGCTCTAACAGCCTTACGGTTTCATGCTTTTTCAATAAAATTGTATAACGCAATATCTTAATTTA
ACTTTTCGCTACTATTACCGTTATTCAGAATGATGCAAAATTAAAATTTAATTCAATTAGCACGA
TAATTCTCCAATTTTTAATTAACACTGCTTAGTCCCGTTCGTTTCAGTGGAATTTTATTTGCAAC
ACGCGGCCCGTTGACGAAACCTCCGCGGCCCGTTGCTGGGCCGCGGCCCATAGTTTG
AGTAGCACTGGTCTACAACACCTGCTGCAAAATTTGAAAGCACTGGGTGGTGGTGGAATTG
TCAGGCTCCTTTAGATTGGTCAATCAATTCAGCCAATTAAATGCAAGCATCTCAAGATCAATT
ACTCAGTTCAGCCAATCACTCAGCACAGCCAATCACATCAGAGCTTGTGCAGGCCAATCA
CATCACAGCTCTTGCAGGCCAATCAGTCAGCACAGCCAATCACATAAAAGCTCCTTTAGAT
GGATCACTCAGTCCAGCCAATAAAACAGAGGTTTCCTTAGACCAATGCTTACCTGTCACAA
GAGAATCCTGAAATGGGTGTTCGAATGTGGTGGTCACATTTTGCAGTGACAGAGGAGTCAC
TTCAAATGTCAATGAATCGTCTGACAACACACAAGAATAAAACTCCACATTTAAAAATAAGTC
CAAGAAAATCCAACCAAAAAGAATAAATTCAATCCGTTACTACCATGCACTCCAATTCTGAC
AGAACTGTATAAAAGGTGAATGAACTATGCAGACTTGAAAATTTCAAATGCACTTAAAAGTTTT
GGGCAGTTGGCTCATTTGAATGAAGATGTCAAAGTAGACGATGAAAAAGATGACAGAATATC
TTCCAGGATGCCAACGATTGTTACAGGAACAACGAAGATGACATCCAAGCTGTTAATTCTAT
GATGCAGCAAGAGTGCAATGTGCTCTGTAGCATAACACAACACAAGAGGATCAGTATCGG
CAGAGCAGACTAAATGTGATACTACAGGTGATATTCCAGAAACAAATCTAGAAATAAATAAC
AAATGAATATAATAAAAGCGTAAAGAAATATTATTCATAAATGCACATAGAAACAAACTTACAA
AGCAATAAATACTGCACTAAGAAAACTAAGTAATTGAATAACTGCAAAATGCCGCTGATGTT
GGAGATGGGGAAGGTATCCTGCCTTTTGTTGCTGATACTGCTGTAGTTGGTCTTCGAATGCA
ACTGAGCATGATCGTATTGAAGAGCACCTGCTGACGAAGCAGAATAATGATGGATGGAAC
GTTGGTAGGATGACTGAACGGAATCCTGGAGATGAGCGCTCCTTGCAACGGAAAACCTC
GGCACCCATCGAAGAAATACCTATGGCTCTGGTCTGGAAGTTTCTAGAGAAGGCAAAAAC
ATGGAAAAAGAAAACAATAAGCCAGAAAGAACCCCGGTAGCTGTCTTCAATATGGGGATTT
TGAATATGTATCAACTGAATTTGAATTTCTTTTATTCAATGCCCACATGAGGGACACTCAATGA
ATATCTTTCTCACTCATCTTGTATGTGAGCGGTACAAAACTTAACAATTTTAGGCAATAAATGC
ACTCAACAATAATAAGACAAATAGGAAGTATCACATACATTTTTAGACCATTTATACAAAGTTC
CTAAAATGCATCATATACATGAGGTTTTAAAATTTGCAACTATAGCAATGTTCAAATATTTGCAA
TATAAATAAGGTTTTTAAAACTCGACAATATAAATTGTTTTCAAAATCTTCAATATAAATGTGATTT
TAAAAATCTACAATATAAATGAGGCTTTAAGATATAGATATTTCAATGCCCTGATAGATAAATGC
AAGTGTAATGACTGACTACAAGAGAGTGCCATCTTGTGTTCAGCTGCTCTTCAGTCCTTCGC
TCATTTCTGGCTCCATAGCTCGTTTGTCTGAGGAGGAGAGAAAGAATCGACTGATATTCTGT
TGCCGGAGCAAACTCAATCCCTTCAACAAAACAAAAGACATGCCTCATTCATTATTCATGAT
GCCAATTGTATTCATTCATCATTATCCTCAAAATAGACGTCATTACATCAGTACATACCATCGC

ATCATAACCATACCATTAACTAAAGTCCTTTCAATAACTGCCTTCATTAACAACACTGCATCAT
CAACATCATATATCATCTTCATCATCAAGATTCCTAGCAAATGACCACAGAGAGACATACATA
AGATGCAATTATACATATAATAGGCATACTGAATCAACCAGTTTTTATGAGCAGTCGAGACCA
TAACCAGCTTTAGAAGTGAATATGTGCAAAATGCAAAACACACTGGTCAATGTTCAAAAATAT
TTCAACAATTTTCTCAAATTCCAATCAGAAAACAAACTGCCAATAACTCGTATTTGATGTTGC
CAGTAGACATCCTGGTAAAGTCCCATTCTACTCTCTGGTGGAGGGAGTGGTTAGAGCTGGA
TTGTCAATCAAAGTCAGAGGTCCCAATCTTTACCAAGGCAAAAATTGAATTCAAGATTTTTGC
TCTACCGAATCAACTCATCGTATATCTATAGCTCAGCTATAACGAGTACAATGACAGTGGGA
ACATATACAGAAGGAGAGGTCTAGACACAAGCTATCATATGCCAAGGCAGAGGAAATGAA
GTCGTTAAAACTTCATACACACAACCGTCCTAGCTCAAAGACTCCTCCGTTTCTTCATCTGC
TCAAATCTCAGTTAGGTAGTAGTTGATGCTGTTACCTGTCAGCTGTTTGATGCTGCTGATGAC
CGTGGCTGACAACACGAGTGGACTTTTCAGCTGAAGCACAAACTGGGCTGAAAGGTGAG
CACTGTTGTAGTTCGTCAGTCCACCAAATGAAGGAAGACTGGACACAGGCAAGACCAATA
TAAGCACAATATCAATGACCATCACAACAACAACGTGGTGGTGTAGCGGTGAGAGTCGCA
GCCTTGTGACCGATAGGTCGCTGTTGCGAATCCACCTCCAGCCACTGTGTAGAGACCTTG
AGCAAGTCCTTCACCTGCAATTGCTCACAAGGCGCCAGTGGCATGTCAAATCCGACTCA
GGATTAGCACTGTAGTCGGGAGCACCTCTGAGTAGATTCCTTGAAGTGGCGCTATATAAATT
CTCAACATGAATGACAAGTAGTAGCCAAAGTATGTATATAATCTGAAGCAACTGTGGTATGAT
GACTTGGCAGTTGATTGTGGGGCAAGGAGAGTGAATATTTATATTATTCACATTTATGATTACT
AATATTCTGAGAGCCCTATTTATGTAAAGACTCTAGTCTCCCATGAGATGGTTACAGTGTAGT
GACTAGTGAGCACGTCCTCCTTTGCCCATAATTCCTCAGAGCCAGCAATTGGCGCTTAGC
CAGACAGCAGTACTAAATAAATCGTTCAATAATGACAATAATAATATAAAAACTCCTCTATAAG
CTAAAGACAGAAGAAATACAATGATTTAGAAACACCATTCGAAATTTCTGAATTTATATTTGCA
GGTTTTCAGAAGTATGAAGACACAAAATACAGAAGAATTACATCTAATCAAACTTCTTAAACG
CCATGTACTCAAGCAGCGGAACAGAATTCTCCTAAGTCTCTCAAATTCTAAGTCTCTCAATT
GCTCAACCTTTTTCCCTCAAGATTCTTTGTCGTCGTGAGAAGCGGCGTTATCTGAAGACGA
CGAAGAGTGGATGAGGATTCGATTCCAACCGTCAGACTAAAACCAACGTCACTGTCGAGA
ATTGCTACAGAGCCACAGACCACAAGATTATACAAAGACTTAAAACATAAATATTATTCATGC
CAGAATTACAAAATAAACAGTTTCTATATTAGCAATATACATGTTAATATTATCACTGTTAAGAAT
ACGAACATTATAACTGATATACCAGTAAATGCAAAAAGCTGACCATATTCATGATCAGAATAT
GAATATAAATTTAACTGCAAAGATAAATAGTAACCATGGTACAAATATATATTTGATACGAAGAA
TATTTATATTATTCACACTTATGATTACTAATATCAATATCATCTCTTTCAAAATGTGATAATCACA
TATTAATAATGAATACCATATATTATTTCTGTTAAGAGTATTATAGCCAGGAGAAATAGATGCGG
TACTTAGGTACATAGTTGCAAAATGCAAATTACAATCACATCAGATGACTGGAATTTGGAACA
TCAAGAATAGGGGCATCAAGACATAAAAAAATCAAGACTTAAAGACATCAAAACTTAGAACA
ACAACAGTAAGGAAATCAAGACTTTAGAACAGCAACAGTAAGGAAATCAAGACTTTAGAAC
AGCAACAGTAAGGAAATCAAGACTTTAGAACAGCAACAGTAAAGACATCTGACTTATGATAT
CAACAATTAATTAAACCATCAAAAGTTAAAACAATGACACAAAAGACATCAACAAATAAGAC
ATCGAGACTGAGGGCATCGAGACATAGAACATCAACAGCTAAGATATCAATGTTTAAGACAT
CAGGACTAAAGACTTAGGACAATAAAGACATAGGACATGCATTTCTTGTGCTGAGATAGTCT
CAAAGTATGGAGGAAGCGCTAAACTAATCAACTAATCAAGTAAAACAATCTAATCAAAAGCT
GGCATTGTACGGATGTATTTCCAAATTACTTCACCCACGCTGTACAGTGTACACCGCAGTC
ACTATTTGTCACCTACCCTTACCTTCAATGGTGAGTGGAGTCATAGAGCGAGTTTTGTGGTT
GAGCAAATCAAATGGTCCAACAAAGTACGTCAACTTCATTGGCAAACCTAAAAGACACGAT
AGCATCAATGTTTAGCATTTCAACAATGCCAGTAGAACAGGCAATATCATCCCATATTAAATT
AACACTTCATTCTACCTCAACGAAATAAAGACAGCCAACTTTCTGCCCTGGTCAGAGATGG
GAAAATTACAATTTCTTTCAGAATTTTGCAAGAAAATACAAATAATGTAAATCAACTACAATAAT

AACATCAAATATAATAATAAATAATAACAAATTAACATCAATTAAAAATTATGTTCACAAATGAGA
TAAAGGGACTCTACAATTAAATTACTAGCTACCCTAGAGCAATGGTTTCCAACCTTTCTCATC
ATGTGACACCTTTACTAACACAACGTAAGACCCAAGGCACCCATGGCCATAATTTCATCTC
CCTTGCAGCTTCTCCAAGTTAGTTCCATGTTTTAAAGACAAGCAGTGTTTCTTGTATTAAAAAA
AGGGCCTGCAACTATTTCTACACTACAAGTTTTTAAATAAAACCAGGCTAATTTTAGTAGTTA
GCTCCAGTAATTTTCGCATTTTCTACAGAATGAGCTGCACAAATGTATGGTTCTGCCAATCC
CCAAGTGATGCAGCCTTGTAAAAGTTGACAACACCCTTGCGTGGTGCGACACCCAGATTG
GGAAGCATGGCTCCAGTGACAAAGAATTTCATTATACAAAGTTGTAAAGATAAAACGTGAAA
ACACGAACCCAGGTTTCTGGAAGTGACAACTCCAAGTGGCGACTTATGAATGTAGCTCTG
GACACTTGCAATAGTACTAGAACACAATCACACCAAGAGCACCTAAATAATCAGTAGACAC
ACAAGGATTCCGACCCAAGACTAAACAGGGCCCAGTATAATAAAATAATATACGAAGCAAC
TGTATTTAATGTTCCCCTACATCCAAAGTCCAACCATACGGCAAAACATCAATGACCTATGTT
GCATAATTTAGTTATTTAAGATCCTTAGACACAGTGAATGCCTCAGTGGACGCTGACACCTG
TTGCACTGCAGAGACCGAGTGCTCTGTTAACTTGGATGCCATTACCTGAACACAAGGGAC
ATGCATGTAGAGACTGAAGCATTCTAAACGACTCACTGAAACTAACCAATGTAAAAAGCCA
CTGCAGCAAATATAATACTACTGCAATATTCTACATAAACCAATTCCACTGTCAATTTATCTAA
ATCCTTTACCCACATAATTTAATTCTCACCGAACCATGCAGAACTGCGATAAATGTCACCAC
ATTAATATCTCCTTAGTTTTCTATTTAATAGTTATATCCACAAAGTCTTCTCTCCAAGTTGTAAAC
AAGGTTTAACTAAATTAAATTGCTCAGTGGATATATTTTTGCATCAAGACTCGCATATAACATC
GTCCTCAGAATCTAAATGTCAAGTAGTCATGTAAGCCATATGATGCAAACTGCAGGTCCCTC
AAAATGACACATGTCTAAGAACTGTACAACATGTACGGTTTCCATCAGGCAATACCTATTTTC
CTGCCTACCTTCAAGGCCCTCATCTCTTCCTCCTCCATCATATACTCCTCTTTCCTTCATGT
CTTTCTTATCTTCCCTCTACCCACCTCATTCTATCCTTTCTTCTCTTCATCCCTATGTTTCCATC
TCATCCTCCTTTTCCACTCCACTGCAGATCGATATAATGAATCATCATAATGTAGAAATTAAGA
GAATCACAAAACAGATTCTGCCAACCTTTGAAACTTAGAGAGCATGTTGAGATCAGACTCA
AGAGACTGCAGAGCCTGGATGGCCTTTGCTTTTGTCTTCCTAATATAAAATTCCATTATTCAG
AATAAAACACCAATTTTAATTAACCTAAAATACAGAAATGAGTAAAAAAATAGCAGGCAATATT
GGGCAGCAATAGGAATAGGATCCAAAGGGATATGGGTTCAACTCAAGAGCCACCTATGAG
ACAGTATAAAATATTTGGTGATGCAGTGTACAACACAAATTCTTAACTAATCAACTAATACAGT
TTAACTATCAGACCACTATTTGAACAAAAAGAAAATTTTTGAAAAGTCTCGTAAAGTAGGAGG
TATGTCATTCCAAATCCGAGGGCCAGCACAGTTAATTGGGATAAACGGGAATTAGTCCATG
GATAAGTTGCTGTGGCATTGGAAGCAGAACGTACCGAATAAGGGCTACAAGAAAATTACAA
AACTCAGAAAAAACAGCTGTGAGAAACTGCAGAAAGATCACTTTTACAACGAAGACCAAAA
ATTGAGTACTGCAGACTCCTACTTCTTTTGCCAGGTAGCATGGTAAAATAAGGCGTGTTAATT
CCAACTCAGCATCACTGCTGCATTTGGAAATGCCTCTGAGAAGGCTTTTTGAAAGGTTGCT
ATACAAATGTTCAGAATGCACGAAAGTTTCACAGCAAATAACAGGAAGAAAGAGATTTTAAC
CATACATGAAGTCTAAGGTTAATGACTGGAATAAATGTAGGGCTGAACCGAGTCAAAAAAAA
TAACCGAGTACCGAGTATTAAGAGGTCAGTTTTGCCGATACCGAGTACCGATACCGAGTAT
TATACAACAATGTGAAGTTGTTTGAATTGTGTTAATTAAATACTGTTAGGTCTAAAAAACTCTAA
ATGGTAGCTCTTGAAGTGTGTCTTAGTTGCTACTAACCTACGACTGCACAGAATTTGAATACT
TCAATGTAATAATTCCTTAATTTAAATTAATATGAATAATTTATTAATATATATATATATATATATATA
TATATATCATGTTGCTGACCATACTATGCTTTGGGAAGGCCTAGTATATTCAGCTGAGCTGTG
CAGAGTGATATAACAGGTCCTACAATTGAAACTGGAACCTCAGTCATAGAATCCCTAAGATC
AGACAGAATTGCACGTGGCCTAGCCTCAGTACCCTGACACGTTGCTTTGGCTATACATAG
CAGGCAGGCAGAGGTTTCTCAGCGACGCCCACAAGAGCAGGGCCCTGAGGACCAAAC
ATTGGGCTTTAAAGACCGAGCTCGGACGTCTTTGGTTTACAGTCCAAACCAAGATGATTGA
CAGAGAATTGTTGACCAATGAGTATTAATAATAATAATAATAATGAGACGGCTTATATAGCGCC

GTAACTCGGCCGAGGCCGAAGAAAAGGGCGCTTTACATATTTCACAATGTTAGAGAAGGC
AGTATGATTGCTTAAACAGGTGGGTTTTTAGGTGCGCTTTGAATGCCTCGATTGTTGGCGCA
TTTTTCACGTAGTTTGGCAGACTATTCCATGCCAGTATTGGCTGGCATTTGGATAGGGGATG
TTGTTGATATGCATAACTATTACTAATGTCCGTTGTTGATAACCGTCATTCTCTCTCGCTCTCTC
TCTCTCTCCTTCCGAGCCAGTAGGAGAGCGAGAGAGAGAGAGAGCGAGAGAGAGAGAG
AGAGAGAGAGAGAGAGAGAGAAAGAGAGATAGAGAGAGCGAGAGAGAGGGGATGTTGAGA
GAATGACGGTTAGCAACAACGGTTACAACGAACAAGTTGTGCCCTCGGCCGTCTTAATTG
ATGCACAGGACCGCTTGTTTGTTTACACAGCCAGGCTGTATGAATAGAGGGGTCCCAGTAT
GACCTGCAGAACCAGCTGCCACCTGTGTGATCAGTACAGCTTTTCTGATAATAATCTCTAG
GGTAGTTATTCATGACATCCAGACGATGTCTGAGGCCTGTCGGCATCGTAATTTCCAATTAA
CACTCAACTGCTCAGCCAAATAGGACAAGTTGCGTAAGTACTGCATGTCGTCTGGCACAA
CCATTCTAACCTAAAATTAACTCTTGCAACGAAAGAATGCTGGAAGCAACGAATGAACTGTA
AATGGTGCAGTTTCAAGGTGCATTATGGCCTGCCATGTGACTGGATGTATAGCTTCAGGCT
GACCAAATGAAGACTGCTTGCCGACTGTCTTCACGAACCTGGGTATGAGTAGTTTCAATCA
GCTGAGCAGCACAGGGTGAAGTCATACAGCTCGTATTACCTGAGCCCAGCCTTTAACATG
TCACCATGATATATATAGAGCGATCCCAAAGGTAAAAGGGTCACTCCCGGCAAGCTCATTA
CTCGCCAACTCCTACACGGGTAGGGGTCGCACGATGGTACACCAACATGCCATCTCCG
CCTGGGCCAGAGTTAGTCACAGCCCTGAGAATATCTGGGCCAAAGTAAAAAAAAAAATTTT
TTTTTTTAACTACTCGGAATCGGTTCCATTCTAACGACCGAATACCGAGTAACGCCGAGTAG
CGGATATTCGGCCGAACCGAGTAGCCGAACCGATTTTCGGTTCAGCCCTAAATAAATGGC
ATCACATAGTGAGTAGTGCAAGAGCAATGCTCATCTGAGTATGCTTCTGGAAACAAATGCAC
AAAAGCTAAAGAGATAGTTGAACTGAAGACAAACAGACTGAGCGTGGAAAAGAATCAGAA
AACAGAAGTCAGACAGAGAGGTACACAGACAACAGACAGAGAAAGCGTGTGAAACAAGG
ACACAAAAGGCAGACAATGGAGAATTCATAATATATAGCATAAATAATGTATTTCTTCTCATGT
GCAATCCTTTATGTATGTAATCTCGCATACTATAATCCTGTATGAATTCCTGTATATATGCCTAT
GCAATGTGCATTCCTGTATTTATCTCTGCATGAATTCACTAAACCATTCATTCAATCATTAATTC
ATTCTGGTCATTCAGATACCACCCTTTCAAGAACCTACTCAAGGGCACTCGATGGCCCCA
CTATGACCAAATAAGAAAGACTTAGCGACTTCAGAAATCACACCATAAGACCCAAAGGGG
GAAGGTCAGCAAGAATATAATAGTGGTTAATCAAACTGGAAAAGAAGGCACTCGATGGCC
CGACTACAACCAAGAAAGACTTAAAACAAAGACTTAAGACAAGGACCATGGTCGTGTCAT
GACTCGGGAAAGAGGGATCCATGAGAAATGACCCAAAATTGTCAAAGCTAAAGCTGATAG
TAATAGCTTTTAGCAAATAGCTTAACCCTTTGAACCCGAAAACCCGGTTTTAGGTCCGTTAT
CCATAGCGTGACGGAAGCCCAGGGTTCAAAGGGTTAACATGCTGAAAGGGAACAAACTC
GCAATCAACCGTAACTCCCAAATCTCGGACAGTACCAGAAAAACTTATATGAGGAAAGAGT
TAAGTGTTTGTGAATGAATGTAACTATGCATGCATCTATTCCAATTCATTTCAGCATGTATATAA
GAGGTAAAAGAACAAATACATTGTCATCTGAAAAGACCAATCAGAAACAATGAGTTTTGCGA
ATAGATTAGGCTCAAATTCTCACTTGTCCCCTTCGATGTCATAAGAGGAGACTAGACCCTTC
AGGTGGCGCTGCAGTTCTTCATAATCTCCTCCTTTCAAAGACTTCATCAGCTCAGGACATG
GCTACAAACAGCATTTGCACATAACCGCAATCATCACTACCACCATCACATTCACTTCCAC
CGTTTGCAGTGGCAACGGTGGAAGTGAAAGCAGGAAAAACCAGATGGTTTCCCCACATTT
ACTTCCACCTCCACTCAGGTCCAATGCAACAACCACCATGACCAGTGACCACCAACACC
ACTACGGCGACCATCACCAAGACTGCCATTACTACGATCACCATCACTACGACAATGATC
ACCATCAAAACATGACTCATAAAAGAGGGATGAGGCAACAAATAAGGAAATGAGGGAATAA
TAAGGCATGTCAGATACCCTTGAAAGCAATGACCACTGCAATTGCAACAAACAGGTACAG
CTGAACTGAAAGACATGCCACACTTCAACAGAGCATCCAGTCACATTGAAATGCACAATAA
CATAATTCTCTTCACAACCTAAACACAGAATCTCCCGCATAGCTTATCATAGAAAACATGTAA
ACGCATTAGATTGTAAACATACAACATCACCATTAAGTACAAACATCATGGATAATAAATAAG

CTCACCATAGTAAAATATATGCCTTTGGGTAAACAGTACACAGGTTCAAACCAAACATAAAG
AAGTCACACATTTGTAGAGAAACTGGACAAAAACCAAATAATAGAATTCAGAGCATGCCAG
CTTAGTCTTCATACAACTGGTCAAGGGCCTCAATTAACCCTTTAATGCCGAGATCGTAGTCA
ACTGCATGCATCTCACTACCATCTCGGCATTAAAGGGTTAAGAGCAGCAGACAGCAACAA
AATTCAGAGCAGTAGAACTAGAAGCACATGCACCCTGCAACTGAAATGGAGCAGAATATC
CTTGACACATGCCAGCTTAGTCTTCGTACAACTGGTCAAGGGCCTCAATTAAGAGCAGCA
GACGGCAACAAAGTTCAGAGCAGTAGAACTAGAAGCACATGCACCCTGCAGCATCACTC
CAACTGAAATGGAGCAGAATATCCTTGACACAGAACTCTCAGAGAAAGGAGGAAACCAGA
TGACAAAGTCTGCAAAAGATGACAGGATAACTGACAAGATCAATGCCAGACGATGATGGC
GTTGGCATGTCCAAATAATAACAGCCCTAATAAGTAACAGCTGGACTGAGCTGGTTCAACC
ACCTGGTTTTTCCTACATCTCAAATCTGCCACCTTCTTACATCTTTCACTAGAAACTGAATTAG
CTTACAAGTGCACCATGATGGTTCAAAATATTTCGCTGTTAGGTGGAAGTTCAAAGTGCACC
CTAGACTTGTTTGTCTCAGAAGTTTACACTCACTCTAGTCACATATTCAGTCAACCTGTAATAC
TGGTGGTCACAAAGTACACTTACACCTTTACCTTCATGCAGGACTCTCTTCACTGGCACCC
CATAGCCCAGCGTATTCAGTTCAAAATTCTAACCTTTACATGCAATTGCCTCATTGGTTCATC
TTCTGCTTACCTTAAGGCTTACTGTCCTTCCATTTCCATACTCCCAGGTAACTTCTCAGTTTG
TCCCTTCCACAGTTTTGCAAACGTCTTAAGACCCATGTTTTTGGTCTTGGTGGAGCACCTC
CAAGTCCGTCCGCTTTCCACTCAAAACTGAGATCTCACCTTTTTCAAGAACTCATAAACTG
CATCTGATCTTCTGCCTTCCAACATCCGTCTTAACAGATGTTCGGTCGCCTCTGAACTTATG
GAAATCAGACTCAGACACCATTGATAACCCTCTTAATACCCCTTTGACTCAAGGCAGTGCT
TGTGAATAAGCTGGTACCATGCACTTGGCTTTAGAGCGGAGCACAAGACATCTTAAGATCA
CAATTACAATTCTGCTGATGTTACTAGCACATGGTCCATGATACGCTATGTACTTCACACCAT
CTGAATAAAGTCTGCTCTCATATGACAATATCATCAAATCCAGCTGTAGTATACCGCAGTTGT
AATTAGTATAACGCACAAGGCAGATTTTGCACAAAATTCAAACCATAATAATCAGGATCAATA
TCCATAAAAAAACATAGATGTAAACGAGACATAAACAAATTATAAGAAATCAACGGCTAGAC
ACTGACCTTGTTCTCCGAGTTCTGGCAGAAGACAACATTCGTAATGCTGATGTTGTCTTCTG
CCAGAGACAGTCGTAGACAGAAGGACTCGCCGCATGTGAAGCTCAGTTCCCTTTTCGTCT
CATCTGCCACCAACTTGACTTTCGTCTGTCGCGCAATTGACTCCAGACGATTAAAAGCATT
TGCTCGATTGACAACTAACAACAGAAACAAATGAAACATAGACAACGAGTGTGTGAGCATT
TTGCTTCTGCTTACAGCTTGAATTTGTATGAGGGGCAGGGAATCAGTCTATGGCGGGACAG
TAGAAAAAAACACAACGAGAAATATGGCGTGAAGTGTGATTAAAGATTATTATTAATTACAAAA
CACTAAACTTTTGTGTTGGACTTTAATCAACTTCATGACAGAGTGAATCATGAATGCAACGGC
AATTCACAATAGAGACGATGAGACTACAACAGGAAGACAATTGGTCAAAACACTGACCTTG
TCGAACTTTCTTTATAGCACCCCTTCAAGAAATCGACTCAAAAGACGCTCCTGACTACAGC
GCTGATGCTGAGCTGAAGTTTACACACCAAAGTGTCTAGGAACAAGCGTGTGTGAAGGATT
TGCTCAGGGTTCCTAGACAGCGGCTTGAGTGGGATTCAACGAGTGACCCTCCGATCAGA
AGGCAACGAACATAACACTAGGCCATCATGTCTCACATGGAAAGTACTTTCTTATTCTGTCT
AGCATCCTGACCTTAGATACACAAGAGAGCTTTGCTTTCTCAATAACTCTTTCAATCAGGCC
TCCTCCCTTCTGATGGCTGTGTTAAACAATACCATCACGATTGCCATTGATTGCTGCATGGT
CATTAGACAAAGGCAGTTCATAAATGCAAACAATTAATAATGACAAATATGAGGCAAATGCC
CAACAGATAAAGTACATAAAGCATACAGGCAGAGGCCAAAGCAAGGACATAAATGTACAA
ACTGACAGAGGGCACAGCAAGAGGACAGATACAGATGTAGAAAGGCCAGACACAAGAA
GTGCCAAGACCAAGGCCACTCAGACCTGTACACCCTAATCACCATGCCATATATCAAACA
TGTGATATGTATAACGACAACAAAAAATCCCAAAGCCAGACATACAATATTTTGCAGATTATG
CACATACAGCAAATGATTTAATTGATTGATTGATTAATTGATTGAAACAGACCAAGAACATGAT
GAGACATGCAACCAGAGATTGAATTGCCATGAGATTGGGGGGTTGATCCCCCACCCACA
GTTCACTGCTCAACCCCCCTCCCCCCCAGTTAAAATATTTGACAATTCAATCACTGCATGC

AACACACATACAGACCAGAGACAAGCATCGATAAAGAACACAACACATGTCAAAGCAATC
AACCAATCACGGTGCGTATACGTTAAGTGATAGCAAGTGTAGAGACGGGGTAGGAAGCTG
TTCAGTACCTGCACTGTATCTCTCCAGACAGTCCATGTGTTTATGTAGAGCGAGTATTTCATG
TCGTTCCGGTTTCTTCTTATCCTTAACACACAACATGTGCAGCAAATTATAAATGCAAAACTT
CTCACATTATTCCTTCAGTCGCATGAAAACACAATTGTCTGGTGAATCAACTAAACTCTTGCA
GTCTAAGCTGTCATTTGTACATTCTTGATCCAAGCATCTAGTCATCCAACCATAATCCACACA
TCCAATCCATCCAGCCATTCAGTCAACGACCCAATCTATCCATCAATCAGTCTATTCATCCA
TTTAAACTTCCTGCCTGCTTTGCTTTTATTGCTAAATGTATTTTTTTGTTCTACATGCCTCCCTC
CCTTTATTGCTTCCTTCCTTGCCATCCACTCATTCCCTAAACCTTTTCATTTAGGTTGATTACC
TAATTCATTCACTTGCTTCGAGACAAACTATAACTCAGAGACACAAGTCAGAGATTGGCAGG
AAGTTAGAGTTATGTAGAGTCACAAATATGACACTGTAAAGAAAATGACAAGGTAAGTACTGT
AAACAAATAATGTGCTACTGTAAAGAATATTAACATGATACCGTAAAGAATATAACATGACACT
GTAAAGAACATAACACAAAACACTAAAAACCTAACATGATGCTGTAAACCAGTGCTTCTCAA
ACTTTCTGATGTTGAGGATCGGTGAAAGTGTTAACGAAATTACTGCGGACCGGTATATTTCAA
GCTGCTCTAAAGGTGACAAAGGTCTAACGATTTTAATGCTTATTCGATGAAATTGTATAGCGA
AATTTTTATTTAACTTTTCGTTACTATTACCCTTATTCATACTGATTCAAAATTAATATTTGATTCAA
TTAGTACGATAATTCTCCAAATTTTAATTAACACTGCTTAGGCCTAGTCCAGCTCATTTACGTG
GAATTTTATTTGCAACACGCGGCCCGATGACCAAACCTCCGCGGCCCGGTGCTGGGCC
GTGGCCCATAGTTTGAGTAGCACTGCTGTAAACAACAAAACACGATACTATAAAGAGCATA
ACAAACTGTAAAGAGAAAAAGGTGATACTGTAAAGAGCATAACATGATAGTATACTGTTAGTG
CAAAAGTGAACTTTCAATGTTTAAGTGTCTCTGCTTTAGCCAACGCACAAGGCAGCCGTTAT
TCAAGAGTTAGACAAGATGTCCCAAAAGTAGTGAAAAGGAAGTCCAAATCATCCCGACTAC
CCATGAGAGGGGCAGAGTAGATGCAAAGACAGGTGCCACTTTGGTCGAGAACTGAAAGG
TGCAGGCTAACGGTGGCGATAAATAATTCATAATAAAGGTAACGTTATCGCATAGACTCACA
TCTGAAGGCAAATTAAATGTTTTAATTGTGTCGACTGAAGTCAGTGGACATCGACTTTTGATC
CTCAGTCGATCCATTAATCGATCCATCAGTGTCTCGAGCTCACTGCTCTTCAAATCATCTAC
ACAAGAACAATTATAGATGCCGAATATTAATAAAGTATTTCTAAGAAATACCAATTATAATAATA
ACGTTATATAGTGGTTCAACAAGACCATGTCATAATTGCATTGTATTGTATTTGTGTATTTGTATA
CGCCTCTTGTAGTATGAACTGTTCAGATGCGCTCCCGACTACAGAGTTGATGCTGAGTCAG
TGTTAACACGCCACTGGCGCCTTGTGAGCAATTGTAGGTGAAGGACTTGCTCAAGGAAGG
TCCAAACGCAGCGGCTGGACAGGAAATCAAACCCATGACCTATCGGTCACAAAGCTGCA
ACTCTCACCAGCGCGCCACCATGCCCCACAATAATAACAGTATCTCACAGAGTAGTAGGC
CTTTGGAAAACATATGTCCTGCATTTCAATTATAAAGACTCCACTGAATCAGCTACTTCAACA
GTGGAGGGAAACCATTCCAGCATGCTGGACCTCCAGCAGTAAACGCAGTCACCGAACC
AATAAGCGTTTCCCTTATTGAATGAATGCAATCGAATTCAGCGGCTGATGATAAATTCTTATGT
GTAGCAAAATGAGGAAGTATAGATTCTTTATTTTCATGAACCAATTTTATTAACCATTCATGATT
GCACTTTAAATGCTTGGTGCATTGGGTTGTCGCCTGCGGAAGATTATAAAGTCAGGAACAA
AGCATGGTGGGAGTAGAGGGTGGTTCTGTTTGGAGGGGTTGAATGGTTGATGTATGTAAACT
CTATATGGATAGATGAGAAAGTAGGGAGGCTAAGGCAAAGAGTTTTGGGGCTCCAGATTCA
ACTGAAAGCTCTAGATACTTCTTCTATTACATGACTAAGAAGTCAACCATTTTCCAGCAGTGA
AATATCATCATCACCACAGGGCAATCTGCTCCCAACAGTTAGTCAGTTGTCTCTATTAACAG
TTTGACCACCCGGTATGAGTTAACTTGTGCTCCAGATATACAGGTCTGCTATAGTCCATTGG
GTATCATGACATCAATGCCATTTCTGGTATTGATTGCTCAGATTTCAAGTCTCACCTTGGCTG
ATTGAGGGGTTTGATCGCCTGAAGCAAGTAAGATTACTGTAAGGCTGCCGCTCGTCCAGC
TATACAATGGGCATATTCGAGCTTGTCATCATTGCTACCTCTGGTATTGATGACTCACATTTC
GAGTCTCGCCTTCACTCATCAATGCTTTTCGGTAGGGCTCCAGAACAGCAGTCAAAGTGA
AAAGGATCAAGCGTATCAACTTAACGACAGAACTGCCGAATTTCAAATTGTCAGCAGGACC

ATCGCTAGCTGGAGATAAAAAGTTGTTCTGCTGAATAAAGGTCATTTAAAAACTTACAATGCC
CCTCTGCTTTTAAAACAGTGTCTAGGCTATGCAACATAAGCAATGGCCATGAAACATAAATTT
CAGTTTCACTTTCTTGACATAACCAACCACAAAAGCAACCACAATGCCACAACTCCAAACA
ATTTTGTCTGATCGAGATTTTGGGGAAAGGCAATTATTTCTCAAGAAGCCAATTACACCACAT
GCTTTTTTCAATTTTAACCACAACAGATTCTGGAAAATTAAAACTAAATATTGAACGAATGTAAT
CAGACTTTTAATACTCACTACTACTTTTGCACATTTTCAAAGGTTTATAAACACATGTGTCCATT
GCTATTCTATTTCCATAGTTCAACCTTTGGTAATTTCACAACTTAGCATCATTATTAAGCACTTA
CTCATCAGCTTATGGTATAATTTCTAACCCATATTCCAGTTTTCTTTAACTATTAATTTCTTCAGT
CACAATCCCACTTCTTATTTACTCTGAAACATTTCCATCTGTCTCATCTTTATCCGTCATCGCA
CTTCTTTAACTATAAACTATAAACAACCTCGCTCCTCATTCAACTTCTGTTTTACAGCCAATGT
TTCCATCTCTCGTTCCAATTATTGTGTTATCTATTCTGTGGTTATACGTACATACTAAATAGCGC
CAAACTGGAAGATAATCCAATTTTTCTTAATACCATTTAAAACTGTCATGTCCACCATCTCTAG
ATGTAAGGCAAAGTAATGCACTCAGAGAACAGTTCAGCATTTGCCCTATACCTAAATCAGC
ACGAAAGTTAATCAATTGATCGCTTTGATCAACGAAGAGTCAAACAGGCGTCGCATAAATA
GACCGACCATCTAACAATCTCTGCCAATAACGGGACCTTAGTAAACAACTGTCTCGTCGTA
TTAAAAACGGATATTCCACATTAAATACAACATCCAATTAGGGTGCAAATGTAAGCGATGAA
CTTCGAAACCGTCGTGACAATTATTTTTAGTAAAATTAGTCTGAGTAGCAGTAATTAATGAAAC
ACTGACCCATGTTTTCATTTTCATGCGATGACAACAATCGACCACCGTCAACAACAGCCG
CCATCTTGTTCTTAGTGGCGCCGCCTGCCGTCAGCCACAGATGCCACCAGCACTCTGCC
TAAGCCTCCCTACTCTCCCATCAATCCAACAATATATTATGGAGTCTAGAGTCTAGACGATA
CATCAGTCCTGACAGTCCTTGTAATAGGCGCAGTATCTGCCACTCTCGTTCTGTCTTTTCGA
TTTCTGAATGAATAAGTAATGTAAAAAGATTTCTATTAGGCCCATAAAAGCCAATGCACTGTTA
GAAACGTGTATTTCTCGAACAAAACGAATTATAGGCCTACAATTGCAGCGTGTTTGCATGGA
TAATTTATCAGGTTCATATATTGCTCTCCCTTATATTTATATAATTTATAGCTGGGAAGCGTGTTA
TATATTATAATTTAATTAGGTTATGCTCGAGTCGTAGTTATCTTTTCTATGACCCTCAGTGATTGA
ATTGCCGGCCGTGATCGACCCCCAGTTCACTGCTCAACCCCCCTAGTTATTTTTTTAAATACT
TTGAGTGACAACAAATGCATATTAAAAAAATTGTCAACCCACCCACTTGAAATTTGACAATTC
AATCACTGATGATGCTCAAACCAACGATCACAGTAGGCCTATATACGATCGAATGATTGCAA
TGTTAATTAGGCATAAATCCGCATAATGTACAACGAGGCAATTGATGTACCGATTTCAGGGA
AGTAGGCCCTAATTAGTTTTAAGTAACACAAACGAAAATTCGTAGTTACGTTCCTGACAAATT
TAGCAGAAGTCGAATTTTTTATATGACTTCTTAATTCGAAAGATGCGGGCCGGACGCGCGC
GGAACTCTATTTCGATACCGCTTCATTGCTCTTCACGTAAAGCCATTTTACCGCTGACTGCC
CTACACCTCCCACCCTCCCCCGCCGGAGTTAATTGGGTATTTGGGATCGAATCATACTCG
GGTCCCGTTCTCTTCGTCCCAGTTGTCAGTTCCAGAGGACTCCGATCAACGACGCGAGG
CGATGTTATTGTGCTTCGCACAAACCGGAAGTTTGGAGGCAGACAGGCAGTATATCCGGA
ACTAATATTACAAGTAGGCCTACTTGCTTTCTCAGTTGCAGGACCTTTGGAATGGAACAGTC
TTCCAAAAAGTTCCAACTACAGTCCGCATTGTTACATCTGTTGCTCAGTTTAAATCGAGAATA
AAAATTTATCTGCTTTCGTTGTAAGCCTAGCTACTTATACCAAAGATGGTAGAGTCGTAGACT
CTTTGCTTATACTATGACTAGATCGTAGTGCTATACGCGCTTTCGTGCAAGACATGTCTTGTG
AAAGCGCGCTACAAATGTTTATATTGTATTGCAATCCTAAAGAAACCTCTGTGTAGCGTCCAT
GAGCACAGATTTTGAGCTTTAACACATTAATAAAATTAAATATAGAACATAGAATTGATGCAGC
GTGCGGCGTTTGCCAGTAAAGTGAGGCGCTAGTTGAGTTTTAGCAATTGCAATCTTGAGCG
TATAGCAATGAATGATAATAGACCCAAACAGAGCCTTAATTCTATATCTATGGCTCTGGGCC
CAAAGATCTATACCTCGTTGTGCAAGCAATGAGATAATATGCAAGACATGGCTGATTATACGT
CGATGTGTGAAACCACGGTGCAAGACCGGAAGTGACTGAAGCGCCGCACTGCTGCTGA
CGCATTGACAGATACAGAATCAAGGAATCCAGCAAAGACAAGAGATCGAGACTCGCCAG
CCATCAGCTGAATCAGCTGCCAACGTTCAGCGACAAATAACAATCGTGAATCCGCATTTTC

CCATCTGCAATTCATCGTATCAGTTTGTGAGTATCTCATTGTATCTTGCGACGTTAGCGTTGA
CCTTTTCTTGTCCATATGCTGGTGTATTTCGTCATTGTTCTCTTTCTGCGTTGTACTGTTATCGC
CAATCGCAGTTGGTCTTTGTTTTGATGCGCTATTCTTTAACGATAGTTTTTTTTAGCGCACTCG
TTATTAGACACGTCACTTTTGCCAGTATGTAGCATAATAATTATGGTAATCCCGTAGTTTAGGT
CGACAAACGATCATCATGCTCTTCCACGCTTCCAAGTGACTTTGTACAGAGATGAAAGTCC
TAGTTTGAAAACAGAAGTGGGATGAAAGACGAAAATGTTGATTCTGAACTAAGAGTTGAGAT
GAGGAAGTGACTTTTGCATAGAGATGAAAGTATTATTTTAAAAATAGAAGTCGGATGAGAAAC
CAAACTGTTGATTGTGAAATAAGAAGCGGGATGATTTATAAACATGTTGATAATAAAGACGCA
ATTACTTACGGATGGAAATCTGATTTAGAACAAGAAGTTGGCATACACATCAGAATTTCGAAG
CCTTTGACACAGATTCCGATAGTTTGTCGACCTAAACCACGGGATAACCATAATTATTATTTG
AACGTTGTCCGTATAATTGGCGCTAAGATGCGTTGCTGGTTGTTTTATCAATCACGTTTCAAC
CACTCACATTTTTGCAATACTCCAAATACTTTGAAAGTTGTACCGAATGGTAAATTTTCAATTG
TAATAACGTGAGTCCCATTTATAATTTTAATTTACGAAATTATACATATAACACGAAATATTGTGC
AGTTTGTCAAACCATTCAGAATGATTTACTTTTCTCACTTACCGCTTTATTATGACTGTTCCTTG
ACCCAGTAAAAGGAATCTGAATCATTCTGCTAATTTTGCTTTAGTATTAAGAAGCATGACATTG
TTCTTTATTACATGAATCAGTTAGAATTTATAAAGCACCCCGGCAAAGTCTATACTGAGAGTAA
ATGCCTCCTTGTCTATGTGCAACTTAGGCGCTTTACATAAATTTTTTTGCCCTCATGTTGCAGT
TTAGTATACCTATTTTTTAGTTGATGCAGCTCTGTTGTCTCTTGGTAATGTGCATAATATTAGATC
AGGGGTGGCCAACCCGCGGGCCGCATGCGGCCCGCGGGCTACCAAATGGCGGCCC
GCACCAGATTCCTAGAAATATTTTTAAAATTAAAGAAATCATACAAATTTCTGAAAATAATTGTA
AAGTTAAAGAAATCGTACAAATTTTACACTTTCTTAACTCCAGCCAAGTATCCATTATTGCGG
AAGAATGCATTGAAGCTAATGAGTTTGTTTGGAAGTACATTCCTCTGTGAGCAGGCGTTTTC
CAGAACAAAGTGCAATACGACGGTCCACAAGGGAAGACTTTCTGACAATCATTTGCGGGA
CATCTTGCGCATTTCAGTGTCTGCATTTCATGCTAATAATTGTAGAAGAACTGTCGTCTGGCA
AAGAAGCTCAAATTTCGCACTGAAAGACTCCCGAACTCTTTCAATGCTTTTGCTGAAATTTG
CGAAGATTCTTTTAGAAAGCTTAGTTTTTAACAGCTTTTAATATATTTTTAAATTGAACAAAATTA
AAAAGTCCATTATTTTAACTTAGGCCTATACCATCACGATCTACTGTTTAGGCTTACTAAAGAA
ACTTAATAATATCTTAATCTAGTGACTCCAGTAACTAGTTAACCCAAAATTTACTTTAATAATGTT
AATTTTTTATAAATATTATGTCTCTTCTATTAAAACTTAAATTTGGAAAGAAATTGTTGTAAATTTAA
GCTGTTTCGCCATGGAATGTTTTGATGATTTTTGGACATTTTGTTGAGCCCTGGGCTGCGATT
AAATAGTGCGGCCCGCATCATGCTTACTCAGCACTGAAACGGCCCTCGTAACAAAAAGGT
TGGCCACCCCTGTATTAGATCATGCAGTTTTGAACTCAAATCGCTGAAAAAACACTGAGCA
AGTTGGTTGACCATATGTGTGTATGTATTGTATGTGTGTTTTTCACTAAATAGTAAGCCTATGAA
CTTCTCTGAGTAGTTTTCTTTCAGAGGCGCTCTAAAGATGTTCTGAATGAATTGGGTACCAGT
ATAAGCCGGGAAGCTATGACAAGTCTTGAGATTTACAGGATGTGTTCAAGACCATGGGATC
TTTCTGTTGCTGGCTCAAGAGCCACATAAAGAAAATGAGTGCCTCACCTATGTACTACATTG
GATGTGGGCTTTACATAAAAATCCCATGTGTAATATGTTCATTTAATGTAACTGATTACATTGAT
TTCTGATTAGTTTACTTCACAAGTACATCAGCAAAAGACGCCACGATGAACGTCGGTGTCG
CTCACAGCGATCCAAACCCCAATTCAACATACTTCAGCAGCAAGGGAATGTGGGTGACTT
ACATCATTGTCGTTGCTTTCATACACTACGTTATTCTCAGCCTGCCGTTTCTGTCTGTCGCTAT
GGCCTGGACTTTGACAAACGTCATTCACAACACGGTTGGTTGGATTCCCTTCTTTTCATAGA
GAATCATTTCTTCAGATAACTGAATCAGCTCTTCTGCTGGGTTAAGAAATTCTCTTATAGGGA
ACAATTCTTTATCAGTGAAGGTATACTGCAGCATGTCGAATATTAATCCTCTTGTAATTTAAGG
ATTCAGGATATTTGTCATTGTGTGAGTTGCTTCTGAGTAAAAGATGTTTGGTATGCAAGAACC
CTAATCATAGCATACAAGGCCTTTTCAGTGGTTCAGAAGACTTGTAGTCTGTCTTAAAATCAC
TCCGAAGTGACCTGAAATGGGACGTCGACCTGGTCAGCCTAATTTATAGTCAAATATAGTCA
ATTGTTGTAGATATGTTATCCTTGTAATAAAACACTGAAAAAATACAAAAAGAAGTCACTGGTT

CAATCAGAAAAAAACAGTAGAAACTGTGGAACGCTACAAAGAGTGGCTCTCATGATCAGTT
GAGGAGCGACAATTTAGTCTTTAAAAACACTTCATGGGTGAACAACTATCATCTGGTATATTG
TAAATTTCCTAAATGTCGCATTGCCACTGAAAAGCCACATCCGGGAGACCAACTCGCCAG
CACTTTATCGAATTAAGTCTTCTGTAACCTGATTGCATAATGTGTTCCGGTGCCAGGTCAGAA
GCTAGATGCTGTAAATAGCGTGCGCCGTGCACTCTCGCTTGGAGCTGATAACACCGACCA
AATCCAGAGCCATGTCAGTGGTCGGGACCACATGATACTAAGGAATGAGTGATTGTATTGT
GTTCAACAGGATTTAATTTCCATAAATTTAGAAACAGTAAACACAAGCAGTATGTATGTCAAG
GCATCATGAGTGAGATCATTTATATTTCAACATGATGGATTGTGTAAGTCAGACGGAATGCAT
TACCCCTACTCCAGCCATAGCCTATTGCAGTGTGTCCCAAAATGGTAGTCTGTGGACCAGT
ATAAGTCCGCAAGTTGCCGGTTTTACAAGCGCAAGAAACTTACATAAAATCGTAGCAAACC
ACTGGTAAGGCTATCATTTTAAACGGCAATAGAGTCATAAAATAGGCTCAGCATGCATTTTAA
CCAATTTCAGTACATACTTGAGCTGGAGACCGATAATTCTTCAACTAAACAATGAAACAAATT
AAAATAATTAATAAACTAGTCAGCGGTCCTTCTGCAATGGGTAAATTCCTATTCCTGCCATTC
TGTCCTACTGAACATGTTACTGCTGATCCACAATTCTTAAAAGGTTTTGAAGAAATGGTTTATT
GCCAATCAATAAGTGATAATTTCAATGTTGTTAGCTGATGTTCATCATACTACACATCGAGAAG
GGAACTCCATTTGAGACAGCCGATCAGGCCAAGTTCCGCTATCGCACCGTCTGGGAACA
GCTCGACTATGGAGTCCAGTTCTCACCATCACGAAAGTTTCTTACAGTTGTACCAGTTGTGC
TGTAAGTTAACATGTGTGTCTGTTCTTTTCCGTTCTTAGTCATTTTTTGACTGTGTGATATTGGTA
TTTTTGGCACAAGGTTTTCATAAATTAAAGTAAAAGGCAGTCAAGAACTACGAGAATAACCT
GTTTATGTTTATTGGGGGAAGAATTGTCCACCTACAGTCCTACACCTGATGTAGTTCATAAAT
ATTAAAGGAAAAAGGCTGCTTTTATTCAGGAATTCTATTTGTTAGCAAGGACAATTTCTTAATT
CTTTGGCTAACGAGTTGCATTGTTTTTTGATGAACTTGGCCATTATTAGTTGATATAAATACAAT
ATTGAAATGTTGTAATATTGACAGTATTCTGCCCTGCACAAGTTGATCAGATTAAGATGTTTGC
CACAAAATTAACTTCTGAATGTAGCCACTTGTATGGCAAACATGTTGCCTTGGTTGAAATCC
ATTAAATTAGGCAATATAGTTAAAAATATAAGCCTTTGCTACTCTATGATGATTTTGAGACTCAT
TGAATGTCTCGATTATCGGCTTTCAATCGCAGAAAATGGGCTTTGTCCGTTGTATTTCTTCTA
GAAAAGAGCCTTTACAACATTACCTGACCAATATAATTTAATTTTGGACTTGACTAACAACTA
GAAAGCATTAGACTACAATGAAGCATTCAACACGAGAGTGTTGTTTTTCCTTTTAAAAATTAA
AATCGTCTCTTATAGTATGGCATCAAATCCTGAAACCATACATATTTGCATGAGAGCCTGTAC
AGCATTTGCTGAACAGAGAATTTACTTAATTTTTTACATGGCTGATGAATAAAAAATATCATGA
CACAGCCTTCAATCTGTAATTTTTTCTTTTAATACTTTTAGATTCTTTCTTGCCAGTTTTTACACA
AAGTACGATCCATATCATTTCGTTGTCAACGCTGTCTCTTTGATATCTGTTCTGATTCCGAAAC
TTCCTCAACTTGACGGAGTTCGACTTTTCGGAATCAATGCCTACTAAAGAAGTGACGTTGTT
GAACTGAGGCGGCGACATGTGAAGACGAGTAAACTATGCGTTTGCATAATGTACAGATAGA
TAGCGGGACAAAGCAGAACTAGCTATCATTCTGCCCTTTAGTGGTTTTTTATTAATTATTCGTA
ATCTCGGTTGGTTTTTGGTTCATTTTCTGCATTGGTTGCTTCGTTTGTTGTGCATTCCTAAGGTT
AATAGCAAAATCAAATAGATTTCAGACTTACAATGCATAGATGAATACGGCCTGGTTTGTTCT
CTGAGAAAAGTTTATGCTGCAAGAAAATGGACTGATATTATTGTATTTGTTTAGTTTGTTGTAGT
TATTATTCTGCCCAGTTTTTAAGGGTTTAACCTTTGCAGGGCTTCTGTATATACTTGCTGCTCT
AGAGGAGCATATATGATACTGATTTTGTTGCAAATGAGATTGTTTAGTCTGTAAATAAATTACAT
TTTGTTGCCTTCCGATGCATATACGAAAGCTGCAGTAGAACCAGTTAGATATCTTGTTCTTTT
CATTAGATTAGTTCTTTCGTCTTTGGAATTTCACAGGATGTTCAGACGTCCTAAACAATGTGTC
TGAGGATACTTATTGGAAGTATTATCTTAAGTTTGTTGCATTCTGTGGATTTTGGGCCATTATTT
AGTCACATATCATTACAGGCCAACTGCCAATTTAAAGGCTGCATTTAAGCAAGCGCTTTTAG
ATGCAGTAAACATCGTTGGCGTGGCTATGGAGTTGACTGTGAAAGTGTTACTACCATAAGTT
AGCGACTTCAGAATGCTTGTCCAACTTAGTAAATTTAATATATCGGTGGCCTGATTGTTGGAT
GTTTCGTGTATCACCTGCCTGACAGGATTGCTTGAAAAGGAACTTTGAATATGTTGTCCTCA

CTGATTGTGACTCTACTCTGTTGGTTGAGATGCATTGATAATAAGCCCAAAAGCAGTGTATAT
TCACTTACATGAGCTGTGTAAAATGTTACAAATTAATTAATTACTTACTGTTAATTAACTTTACTT
GTGCTTTTGTGAGGTTCCTATAAAGGTGCTATATTGAGTATCTGAGTACTTACAATTATTCAAAA
TCTTTTATAAGGAAGAAATTAGGCTTGCTAATTTGTGGGTCAGTGTAGTTGAAATTTATCAAGA
AGATCATTCACAAAGGGCATCTGCAAAGAATGGTCTTCCTTGTACCTCTGACCTTGGTTTAG
TACTTCTCTAAAACTTTGAAGACATCTACACTCAAACACAGTACCTGGTACTATGGCTATCCT
ATTGATTTTATAGGGCTAACTGTGCTTAAGGATGTGGTCCCATCTCCAGCACAGGACACTTT
AATTCAGTGCAGTAAAAACTTTGTTCTTTCTCAATAAAATTGGCAATCAAGTCGAAATCTGGA
AACAACTGTTCAGATACCTTCAACCTTTTGAATGACTGTTCCATTCACTTACCACTGACTTGT
CCACATCCAAGAGCTAAGTAAAGGATACCGGTATAATCACAATGGCTGCATCAGTTTGTAA
CTGAAATTGCAAATTATGCTTACAAATTAGTCATCTATATAACGACTCCAATCCGGTAGCAAA
CTACGTATCAATGGGAAACATCCTTGCAAAGGTCGAACTTGGAAACTAAACTGTAAGGCTC
GCAAAAAAACAAGTTAGGTTCATTTGAGCAAAGAATGACATGTATCATGAAAAATAATAAAAA
ATAACATGAACACCATTCATTACATGGATTTATATACCCTTCCAAACTACTGAGAAGCACTCT
AAACACGGCTATGCAGAAATAGTTTTGAGGTGAGAATAAAAGTAACTGGGTGGACCCTGTT
ACAAGCAAAGTGCTAAAGAGAGCACCTTCAAAGTGAGAAGCCAAGAATTAAACAATAAAAA
TAAACTTTGAGACTTCAAATAAGACTTAATATCTCAATAACAAATCATAAACAGAATTAGGCTA
AAGAGAAGAACTGTAACAGATTGGGTACACATTCTCAGTTAAATTTGGCAACAGTTTGTAATT
GTATCTCCATAACTGTTATTACTGTAATTTTACATGTAAAGTACTTTGTGAATATACTCCTCAGTG
CTTTTGTTGGCCTGACCCTCTGAGATTGCTGTAAAAATACATATTAAAAGGGCATGCTAAAC
CATGAGTAGCTTCAGCCACATTTCGATTCCAGCTGCTAATAAACCTTAGTTTCAAGTATCAG
GGTTGCAGTTATAGGTCTATTTTAATTGATTTAAATGAGAGTGGAAGTATGACAATGTACGTTC
AGTGAAGCCGTTTGCAACATAGAAAATCAGTAAACATAAAACAAGGTGACATCAGTCCTAG
GTATGAGAAATAATTGGAAAACATAGGTATTGACAGTTTCAGTATCTACTGAAGACATTTCAAA
TTGAATCACATCATATATGGCTTTTAAAACCTTTTTTAAAAAATCTCTGGAAGACGTCCAATAT
GTAGATTAATGTACCAAACAAGAAAGGAAACCCTACGAAAAACTGTTTCATTGCAATTCTTC
CTATAGAGACGAAATCAAGCCGTAGGGCATACATAATAAAAATTATTGAGCAGTAGGCCTG
CATTTTACATGCCTCTCCGAATCTGCAGGCCTACCAAGAAATCTGTGAAGCAACAATTTTG
CTAAGGGCTGGAAATGTACGCTGACAGTCTGCGGGTTTGGTTAGAAAATTTATCCTAGATAG
TGATCTATAGCAGTTACTTATCGCAAAAAATGTGACTGTCAATTATCGACACTTATAATTTTATT
CAATATTCATGGACTAACCCCAGATAAAATGTGAAGCACGAAAATAAGTGTTCAATGACTTC
ACGTAATTTCGAGTAGGCCACCAACTGATAACCGAACAAAAGAATTGCAATCTATAATCATA
TCATATGGTCGTGGCTGGGCTTTCACGGAAAATTTGCAACCTAAAATTAAGGCCTACGGTC
AAAATCCGTGTTTACACTTCAGCGCGAAGATAATTTATTTAATATTACTAGAATTTTCAGTTGC
CAGCTGCTTGCGACACGCTTCCCTTTCCGGAAGTAGCTTTCTGATTGGATCAACATATGAA
ATTTTTTTACCACAACGGCGGCTTCAGCGGAGTGTGGATAATATAATGCAGAATGTCTAAAC
ACGTAGAAATTATTTTTTCGTAGAGTCGAGATCTGAGACTCCTGTGGTCACCACTAGCCCGA
GATTTAAATCATTGAAGCCCCTAAACCAACTGACTGATATAATAGGCCTACGTTTATTTTACAA
CGCCTTCATATTGAAGATGATTGGCGAATCGCAATTATTTTATATGCTCTGGCAGAGGGGCA
TGCGACGCGACCTAAGCCCTTGTCCATACAGGGTAGCAATAACTGGGCCAGAGGCCAGT
TATGCCATTCAGGCTAGATATTCTGCTTTTAACGCAGCAATCGTGAGTAGGAGAAGCCTGGT
ATTCTGCGCCTAACTATCCAGTGGTGCTTGTCATGTAAATTTAAATCCTCAAAATTTCTCAGC
ACCACCTGAAAGATGCGTCAGAGATATACCATGCAGTTTATTCAATCAAAGTTGCTTCCGC
GTTTAAGATCATAGGCCTACGATATACCACAAATCCAATTCTCTTTGTCCAGTCTCTGAACCT
ATTACATCGCTCAAAGCTAGGCCCTATAGACTCTTTGAACCCTTTTTCGGAATGTTTCGATTT
TCAGAAATCAATTTCAAGTGGACAGCTATTGCTAATTTGTTGAGCATCGGTCGAAATGACCG
ATTAATAGTTTTCACATCTTACAACAATACGTGCAATTTATCTAAACACTCCGGATGTAGCCAA

AGGTTGCGCATAGCAAAAATCTAGAGTGTATAAATACATTTATCGCAAAGTGTGTTGAACTTTT
GCACACGGCACGCTACATTTCGCTGTTCACACGAAGTTGTGCAAAAACGCGTGCAGACC
AATCGGATGGCTTTATTCGTAGGTGTAATCAGGACAGCCAATGAAATGAATCGCGATGAGA
ATCAACTGACCAATAGAAACGACGCCGTTTTGCTATATAACTGAACCTACGTCCCAGCTAG
ACCATAACGCTTAATGTGAAGCAAATGTGAATTGTTGCGGAAGATCAAGGACGTCTGCTAC
AAAGCCCGCTGCAAGAACTAGATATTATGGCTGACGCACTCAACACTCGTAAGTATATTAG
CGTGTTTTGAATTGGCTTTGCAGTAGGCCCTATTATAAATGCAGAGTATGTTGTATGTTTTTTAT
GTGTACAACAGCCATAGCTTTGTTTAAATACACATACTGGACGATTAGTTTTGCGATCTCATT
GAAAACTGATGAATAACCCTGTTCTATGGTATGTTTTATTTAATCGTAATAATAAACTCAGGATT
TTTACAGCACCCACGAAATCAATGCAAGAATGAAAGAATGCAATACATGCATGGTAAGTCG
CCGGAGCCAGCTTATTATTCACAGGCGCAATGACACATTTCAGCACAAAATGAAGCAAGG
CTTATACATATATGTGTGTGTGCGTGTATATAGGTGTGTGTGCGTGTGCGTGTGTGTATGTGTG
TGTGTTTGTGGTTGGGTTCTTCAAATTTTGTATGAAAGAATGCAATACATTTCAAATTGCAGTA
CATTACCACACTAAAAACTATGTGGGAAAATATTGTATAAAAACTTACTAAAACCTGGTTATAC
AATATATAAGCTATAGTATTCTGTCTCATTGTTGTTATTAGATTGTGAGTTTGAGCCTAGTTCCTT
AATATATGGTCTGAAGCTAGCAAAAATAATGTTTATGCTTTCAGAGTGCTGTGGAAAAGACCA
ATGCCCAAGTAAGCCACACGATTTCACAAATTCAATCCTGATTCTGTCTGGCGACATGTATG
TCATGTTAATGAAACTCATACCTGTTATTGTCCTGGTATCACTATGATCTGCTCCATCTGAATTT
AATAACTTTTGTGTATGTAGCTCCTGTTTAGCACCCAGTGTTATTTTAGTTCCTGAGTATAGAAT
AGAAATAATAATTATTTTAAATTAGTTTCAAGTAAAGACCGACCGAGACTCGGTCTCGGTTTC
GGCTTCGGCGCCGTGAGTGTCCAAAATGCCAGGTTCGGCCAAGTCTCGGTTTCGGCCG
AACTTCTTCTCAAGGTTCGGCTGTTGGCCGAACCTACCTGTTGAATGACCAGACGGTTCGT
GGCTCACGTAACGGTTACTTATTTTGGTCTGTTATTCCTGCTTCTCATAAGACGTCCAAACG
GAGGGAGGGGATAGGTGGCAATGGACGTTAAGCAGTAATCACTTTATTCAAAGTTTATTTGG
CGGTACTGAGTTTGTGGGTCGTAGTACACCGCTTGTTAGGCTTGTTTAAAATACACAGTTTTA
CTTCAATAGAAATAAAATGATAGACGATGTCTAAGAACGTAATTTGACAATTCTTATCGGCAC
CTAAAATAAGTGTGGCGAACGCACAGCTATTCAGTTCGAAAAGACAGTTATATGTCGATTGC
CAGAGCAATTTGCATGGCGAAAATGCAGACAAACTGTTGTTCCTTTCGTACAACATGGCCT
GTTTGGATTTAACTATTAGGCCTTTTTGTTTGTTAGCTATTATTTTCATTATTCGCTAGTATAATCA
AATTTAAATGTAACACGTTTATGTGCATTTCTAGGGGCCTGATATATTTCTTCACAGATTTTATA
GGGTTCGGTCTCGGTTTCGGTTTTGGCCGAACTTGTCAAAGGAGGTTCGGCCAAGGTTCG
GTCTCGGCCGAACCTCAAAAAGTCACTCTCGGTCGGTCTCTAGTTTCAAGGAACAAAATCT
GGAATAAAAAATAAGTCAGGAACGAGAATAGAATAGGAATATTAATTTAAATATTTTTCTAGAA
CAAGAACGGAATAGAAATGAATTATTTAATTTTTTTAAATAGTTATGTAGTCTGACACAGGCTTC
TGATTTTAAGTGAAGGTCTTGCGCTACAAAAGAATGAAGCAGTAGCATTCACATTGTCCAAA
CTATGCTAATCTAATAAAATGATCAAGCGACTGTTTCGTTTGACATTTTCATAGACATAAATCAT
TCATGTTACACATTTTTTTTAAGACGTGGAAGTGGAACTATAAATAAAACTTTTCAGGAACTGT
AACTAAAATTATTTCTAAATCGGAAGTGGTACTAGAACTAGAATAAAATTTATTTATACAGAAGA
ACTAAAAGTAGAATAGAAATGATGACAGCTAGTGGAATAGAATAAGAATAGGAATAAAAAGTA
ATCCCAGAACTTAAGTAACCCTGGTAGCACCTCTCAAAAGTGCCACTGCTAATTGCCTCTA
CCACTTCATAACTTATTCTTTAAACTGCCAGCATTAATTTTCTTGTATTCACTTTCAAAATATGG
TTGTGGTGTGGTCTTGCATCATCAGCCCAGGATCAGAAAGTGTCACACGAGCTTTGTTTTG
GTGGACAATGGCGGTACTACAAAATATTTTTATAGGAAAGCCAAAGATTTCAATAAACTATTA
CTCACTCAAAATGAAACATTCCTCTGTCGTTTTAGGACGGGAGTCAGCGTGTGTCTGCAAC
AATTGCAGATGTCTGAAAAGTGAATGCTTGCCAAACTGCAAGAAGCTGTGCTGTGCTGATG
CCCAAGGACAATGTGGAAGTACGTTTTCTAATTTTGCATATTATGGCAGTAATACATAATGGG
GTCAAATGATACATAAGGAAATAAATACCAGAACAAATTACCTTCATAACTGTGATACACTGTT

TTGACTTCTGTATGAAGGCATTAGTATCAGTATGTGCTATGACTGCATCTCACAAACATGACA
GGCAAAGCTGAAACACGTACAGTAAAATGGTAAAACAGTACATACAACAATAATTATTGTTC
AATGGATCTACAGTATTATAAGAAATGAGACTGACCTCTGCCAAGCATCAATCTCCATGTTTT
TTTCTGGCAGGAGACTAGATAAAAGCAATTTGTACAGCATTGGAATTTATATTCTATATGGTTTT
TACATATTTTTAATGTTAGATACCTTCAGTAGTTTTTTGATTATGCTTATCTAATTATACACCATCT
TCTGGAATCAGTTATTGTCATTTTACTGTAGTTATCTATTCTTAGGATGGCCTACAAATCCAGTA
AGTCACTTTGATATGTTCTGTAGCTGTAATTTAAGACTTTCATCACACTGCAGTGGTGTACAC
GTATAATGCATCGTAAACAGTCCATAGTCTGACTGATCTCTAGAAGATCTGTCCATAAAACAG
AGAATCAGCCTACGTGTAAAAAGTCTCTTTACCTGTCTTGTTGTTTATCAGTCTTGACTCCTTG
CGTAGCTTCCATATTTGCAGCCTATGCTGCGTTTTTCCTACACATTTTCATTCAATCTGCAAT
GTATTGCCAGCAGTGTTCTTGGATGAAAATTATCGTAAAGCAAAACCTGTCCAGCTGATTTGT
CACTTTAATAAAAAAATTTCCATCATGATGCTTTTTCAGCTGCTGAAAAATTTTCAAGTGTAGAT
GAGTATTAGTGAAAATCTTATGTGATGATAAAAATATTTGTCAAAGTCTGCTTTACAATGCACAT
GTACATTTCAGATACAGGCTGCAAGTGTGGGGCTGCCTGCAAGTGTTCAGCTGGTTCGTGT
GCAACAGGCTGCAAGAAGGGATGCTGTGGTGATTAATGTAAGTCCAACATTTGACTGCTTTT
GTTATTATAAAGATATTTAAAATACAGATTTAAGAAGGATCAATGTAATACAGCTGCCTTATAAA
ATAGGGACTCTCCATAAACAATATCTGCAAAAAAGAGAGTACAGACAAGAGACAGAGGAAA
AAGAGAGAGAAAGAGACAGTGAGAGAGATCCAAGCAAGGACAAGTTATTTTCAAATGTGTT
TTAACACATATTCATGGCTACCAGAGCCGTTGGTTGACAAGGAGAGGGGCTATGCTTAGTG
GGGACATATTATTGGAAATTGGTGCAACTATAGTTATTCTGCTACATTCCTGCCTTGAACACC
AAACCATATTTAGATACCTGAGAATATGTGGATCTACTGGACTGTGGCAGTGTTAACAAGGTT
GTCTACTTTAGACCTTAGAACTTTGGGGGAGTGTGGACGTTAGTGCTTGGGAGAAGAATGC
CAAATGAGGCAATGATAGAACAAAGTTGATTACTATTTTAGAGTTGCTCCACGGATTATCCAA
GCAGGTGATGACTACAATATAACAAATTCTCCTGCTAGAATTGTGTAAAAGGAATCGGTACTT
TTACTTTGTGAACCTATGTAAAGGCCGTCTTTGAGTTAAACATGACTTCTAACTTGTTAAAAGC
TTACATTTGATCTTAGTGCTGCTCTCAGTGATGCTGTATGAATGATTTTATGCATCGTCGTAATG
GCTTTGAAGAAGTCTTTCGATTGGACTCGATTGCTATCTATTGTATATTGATAAGACTAACAGT
TTTTGATAACCTAATAGCATTGAAAGGGTCGCTATTCATCGCTATTAGGCCTATATTCAGAAAT
TTTGCACAAAGTTCTGTAATGTGGTCTTTTGTTTCTCTGTAGAGAGTGTGAGAGAAAATTACAA
TCACCGGTGTTGAGATGCGGATCCAATCTGTCGACAGTCACTTTCTTAATTGATTTTTATTGC
TTAGTTGAGAAACATTTTGTAACATTCTTGAATAGGTTCAGTTAATCAAAATTCCATTTTATGTAT
TCAGGCTAAATTCAGATGATGAACTTATCTTTGAAAAATATGTTGAGTTGTTAAATTGTTTTGGA
GAAATTGTTGCCTAAATCAATTGTCTAATAAAAGCTACTTAAGTGAATTATTTGCTTAGAGAAGT
TAACAATTTACACATTAATCAATATGCAACAGTTCATAAATTTGATGGACGAAGTTTTAGCATT
GAGCCTTGCCCTTAATATATTATGGGTACGAGGATATTGGATATACGTTCAGATTATTACATTG
ATCTGTACTGGTTCCCCATTCATAAGTACTTATTAGTACATTCTCATCATTGAAGTGTGTTAAGC
CACATCTCAACAGTTGACCTAATGATGTTTCACTCATATATACCTACACGTAGGCCACTATAT
TACCTATTCATCAGCAGATGTTTGTTTAGAAGGACCAATTATGTTAAGTGCCCGAGTTGAGGA
GAGTGGGTGCTTATTTCGAGCTTTATCCCCATCTCGACACTAATAACCTTCGCCTTGTCACA
TGGAAACACCATCTTTTGTAACCCTGTCATTGTGTGTATAACAATTGAAAATTAACTGGACCC
AATTCTTACCTTACCTACCACCAATCGACTTTGACTAAGCGTCTGTGAGTATAGCCTACATGT
ACAGGGTGGGCCAAAATAAATGGCAACACCTCAGATTATTGCTAATATCTCGGTCAAATATA
AGAATTTTAAAACACAATTTTACAGGCTTATAGCCAAGATATATTTACACATTTTGGCCAAATAT
CATCAAAATATTTCAAACAGAACAAAGGTTATATAACTTTAAGTGAGACTAAATCCTGAATTTT
GATCGTTCAAAAACATATTTGCACTAAACTGTGATTATATAAAGTTGATTAAGTCATATCTCAAA
GAACTTTTTTCAAACAAACATTGCAATAAAGCTGCACAACATAACACATGAAGAGAAACAG
GGTACCAGCGTATCTGCTCGTCACTCTCAATCCAAAAATCAGCAACCAAAACAGTCGTGTT

TGGCCTAGCGGCAAGAAATCAGATGTCAGACCTGATCGTTTGATTGTTGAGTGTGAAAAGTT
CGCTCAAGATGTTATGGTATCGGCTGGTGTTTGTTTTGGTGGCAAGGGTCGACTGCATTTTG
TTGATGAAAAGGCGAAGGTCAATGCTGCATATTATGTTGGTCGCCTGCTACCAGAACTGATT
TCAGATTGTAAACGTCTGCTACCAGATGGATTTATTTTTCAGCAAAAAATACCAGCGCATACT
GCTCCTTTGGCGCAAGGTTGGCTCACAGACAATTGCCCTGATTTTATTGAGAAGGACCAG
AGGCCCAAAAACTCTCAGGACTTGAACCCGCTGGATTACTACGTTTGGGGAGCTATATGTT
GGAGAAATACCACAAACTGCGACCAACACCAAGAACGGTAAGCGAATTGAAGGCAGTGC
TGAATTTGTTATGGAAAGGCCTGTCCCACTGTCAACAAGGCCATCAAAAACTTTAGAAAGC
GGCTCAGAAAATGCGTCGGTGCTGGTGGTGGACACATTGAACATAAGTTGTGAAGCAATAT
AACATTTGTTTAACAAAAGTTCTTTGAGATGTGACTTAATCAACTTTATATAATCACAGTTTAGT
GCAAATATGTTTTTGAACGATCAAAATTCAGGATTTAGTCTCACTTAAAGTTATATAACCTTTGT
TCTGTTTGAAATATTTTGATGATATTTGGCCAAAATGTGTAAATATATCTTGGCTATAAGCCTGTA
AAATTGTGTTTTAAAATTCTTATATTTGACCGAGATATTAGCAATAATCTGAGGTGTTGCCATTT
ATTTTGGCCCACCCTGTACATGTAGGCTATACTCACAGACGCTTAGTCAAAGTCGATTGGTG
GTAGGTAAGGTAAGAATTGGGTCCAGTTAATTTTCAATTGTTATACACACAATGACAGGGTTA
CAAAAGATGGTGTTTCCATGTGACAAGGCGAAGGTTATTAGTGTCGAGATGGGGATAAAGC
TCGAAATAAGCACCCACTCTCCTCAACTCGGGCACTTAACATAATTGGTCCTTCTAAACAA
ACATCTGCTGATGAATAGGTAATATAGTGGCCTACGTGTAGGTATATATGAGTGAAACATCATT
AGGTCAACTGTTGAGATGTGGCTTAACACACTTCAATGATGAGAATGTACTAATAAGTACTTAT
GAATGGGGAACCAGTACAGATCAATGTAATAATCTGAACGTATATCCAATATCCTCGTACCC
ATAATATATTAAGGGCAAGGCTCAATGCTAAAACTTCGTCCATCAAATTTATGAACTGTTGCAT
ATTGATTAATGTGTAAATTGTTAACTTCTCTAAGCAAATAATTCACTTAAGTAGCTTTTATTAGAC
AATTGATTTAGGCAACAATTTCTCCAAAACAATTTAACAACTCAACATATTTTTCAAAGATAAG
TTCATCATCTGAATTTAGCCTGAATACATAAAATGGAATTTTGATTAACTGAACCTATTCAAGAA
TGTTACAAAATGTTTCTCAACTAAGCAATAAAAATCAATTAAGAAAGTGACTGTCGACAGATT
GGATCCGCATCTCAACACCGGTGATTGTAATTTTCTCTCACACTCTCTACAGAGAAACAAAA
GACCACATTACAGAACTTTGTGCAAAATTTCTGAATATAGGCCTAATAGCGATGAATAGCGA
CCCTTTCAATGCTATTAGGTTATCAAAAACTGTTAGTCTTATCAATATACAATAGATAGCAATC
GAGTCCAATCGAAAGACTTCTTCAAAGCCATTACGACGATGCATAAAATCATTCATACAGCA
TCACTGAGAGCAGCACTAAGATCAAATGTAAGCTTTTAACAAGTTAGAAGTCATGTTTAACTC
AAAGACGGCCTTTACATAGGTTCACAAAGTAAAAGTACCGATTCCTTTTACACAATTCTAGC
AGGAGAATTTGTTATATTGTAGTCATCACCTGCTTGGATAATCCGTGGAGCAACTCTAAAATA
GTAATCAACTTTGTTCTATCATTGCCTCATTTGGCATTCTTCTCCCAAGCACTAACGTCCACA
CTCCCCCAAAGTTCTAAGGTCTAAAGTAGACAACCTTGTTAACACTGCCACAGTCCAGTAG
ATCCACATATTCTCAGGTATCTAAATATGGTTTGGTGTTCAAGGCAGGAATGTAGCAGAATAA
CTATAGTTGCACCAATTTCCAATAATATGTCCCCACTAAGCATAGCCCCTCTCCTTGTCAAC
CAACGGCTCTGGTAGCCATGAATATGTGTTAAAACACATTTGAAAATAACTTGTCCTTGCTTG
GATCTCTCTCACTGTCTCTTTCTCTCTCTTTTTCCTCTGTCTCTTGTCTGTACTCTCTTTTTTGC
AGATATTGTTTATGGAGAGTCCCTATTTTATAAGGCAGCTGTATTACATTGATCCTTCTTAAATC
TGTATTTTAAATATCTTTATAATAACAAAAGCAGTCAAATGTTGGACTTACATTAATCACCACAG
CATCCCTTCTTGCAGCCTGTTGCACACGAACCAGCTGAACACTTGCAGGCAGCCCCACA
CTTGCAGCCTGTATCTGAAATGTACATGTGCATTGTAAAGCAGACTTTGACAAATATTTTTATC
ATCACATAAGATTTTCACTAATACTCATCTACACTTGAAAATTTTTCAGCAGCTGAAAAAGCAT
CATGATGGAAATTTTTTTATTAAAGTGACAAATCAGCTGGACAGGTTTTTCCTACACATTTTCA
TTCAATCTGCAATGTATTGCCAGCAGTGTTCTTGGATGAAAATTATCGTAAAGCAAAACCTGT
CCAGCTGATTTGTCACTTTAATAAAAAAAATTTCCATCATGATGCTTTTTCAGCTGCTGAAAAAT
TTTCAAGTGTAGATGAGTATTAGTGAAAATCTTATGTGATGATAAAAATATTTGTCAAAGTCTGC

TTTACAATGCACATGTACATTTCAGATACAGGCTGCAAGTGTGGGGCTGCCTGCAAGTGTT
CAGCTGGTTCGTGTGCAACAGGCTGCAAGAAGGGATGCTGTGGTGATTAATGTAAGTCCA
ACATTTGACTGCTTTTGTTATTATAAAGATATTTAAAATACAGATTTAAGAAGGATCAATGTAATA
CAGCTGCCTTATAAAATAGGGACTCTCCATAAACAATATCTGCAAAAAAGAGAGTACAGAC
AAGAGACAGAGGAAAAAGAGAGAGAAAGAGACAGTGAGAGAGATCCAAGCAAGGACAA
GTTATTTTCAAATGTGTTTTAACACATATTCATGGCTACCAGAGCCGTTGGTTGACAAGGAGA
GGGGCTATGCTTAGTGGGGACATATTATTGGAAATTGGTGCAACTATAGTTATTCTGCTACAT
TCCTGCCTTGAACACCAAACCATATTTAGATACCTGAGAATATGTGGATCTACTGGACTGTG
GCAGTGTTAACAAGGTTGTCTACTTTAGACCTTAGAACTTTGGGGGAGTGTGGACGTTAGTG
CTTGGGAGAAGAATGCCAAATGAGGCAATGATAGAACAAAGTTGATTACTATTTTAGAGTTG
CTCCACGGATTATCCAAGCAGGTGATGACTACAATATAACAAATTCTCCTGCTAGAATTGTGT
AAAAGGAATCGGTACTTTTACTTTGTGAACCTATGTAAAGGCCGTCTTTGAGTTAAACATGAC
TTCTAACTTGTTAAAAGCTTACATTTGATCTTAGTGCTGCTCTCAGTGATGCTGTATGAATGATT
TTATGCATCGTCGTAATGGCTTTGAAGAAGTCTTTCGATTGGACTCGATTGCTATCTATTGTAT
ATTGATAAGACTAACAGTTTTTGATAACCTAATAGCATTGAAAGGGTCGCTATTCATCGCTATT
AGGCCTATATTCAGAAATTTTGCACAAAGTTCTGTAATGTGGTCTTTTGTTTCTCTGTAGAGAG
TGTGAGAGAAAATTACAATCACCGGTGTTGAGATGCGGATCCAATCTGTCGACAGTCACTTT
CTTAATTGATTTTTATTGCTTAGTTGAGAAACATTTTGTAACATTCTTGAATAGGTTCAGTTAATC
AAAATTCCATTTTATGTATTCAGGCTAAATTCAGATGATGAACTTATCTTTGAAAAATATGTTGA
GTTGTTAAATTGTTTTGGAGAAATTGTTGCCTAAATCAATTGTCTAATAAAAGCTACTTAAGTGA
ATTATTTGCTTAGAGAAGTTAACAATTTACACATTAATCAATATGCAACAGTTCATAAATTTGAT
GGACGAAGTTTTAGCATTGAGCCTTGCCCTTAATATATTATGGGTACGAGGATATTGGATATA
CGTTCAGATTATTACATTGATCTGTACTGGTTCCCCATTCATAAGTACTTATTAGTACATTCTCA
TCATTGAAGTGTGTTAAGCCACATCTCAACAGTTGACCTAATGATGTTTCACTCATATATACCT
ACACGTAGGCCACTATATTACCTATTCATCAGCAGATGTTTGTTTAGAAGGACCAATTATGTTA
AGTGCCCGAGTTGAGGAGAGTGGGTGCTTATTTCGAGCTTTATCCCCATCTCGACACTAAT
AACCTTCGCCTTGTCACATGGAAACACCATCTTTTGTAACCCTGTCATTGTGTGTATAACAAT
TGAAAATTAACTGGACCCAATTCTTACCTTACCTACCACCAATCGACTTTGACTAAGCGTCT
GTGAGTATAGCCTACATGTACAGGGTGGGCCAAAATAAATGGCAACACCTCAGATTATTGC
TAATATCTCGGTCAAATATAAGAATTTTAAAACACAATTTTACAGGCTTATAGCCAAGATATATT
TACACATTTTGGCCAAATATCATCAAAATATTTCAAACAGAACAAAGGTTATATAACTTTAAGT
GAGACTAAATCCTGAATTTTGATCGTTCAAAAACATATTTGCACTAAACTGTGATTATATAAAGT
TGATTAAGTCATATCTCAAAGAACTTTTTTCAAACAAACATTGCAATAAAGCTGCACAACATA
ACACATGAAGAGAAACAGGGTACCAGCGTATCTGCTCGTCACTCTCAATCCAAAAATCAG
CAACCAAAACAGTCGTGTTTGGCCTAGCGGCAAGAAATCAGATGTCAGACCTGATCGTTT
GATTGTTGAGTGTGAAAAGTTCGCTCAAGATGTTATGGTATCGGCTGGTGTTTGTTTTGGTGG
CAAGGGTCGACTGCATTTTGTTGATGAAAAGGCGAAGGTCAATGCTGCATATTATGTTGGTC
GCCTGCTACCAGAACTGATTTCAGATTGTAAACGTCTGCTACCAGATGGATTTATTTTTCAGC
AAAAAATACCAGCGCATACTGCTCCTTTGGCGCAAGGTTGGCTCACAGACAATTGCCCTG
ATTTTATTGAGAAGGACCAGAGGCCCAAAAACTCTCAGGACTTGAACCCGCTGGATTACTA
CGTTTGGGGAGCTATATGTTGGAGAAATACCACAAACTGCGACCAACACCAAGAACGGTA
AGCGAATTGAAGGCAGTGCTGAATTTGTTATGGAAAGGCCTGTCCCACTGTCAACAAGGC
CATCAAAAACTTTAGAAAGCGGCTCAGAAAATGCGTCGGTGCTGGTGGTGGACACATTGA
ACATAAGTTGTGAAGCAATATAACATTTGTTTAACAAAAGTTCTTTGAGATGTGACTTAATCAA
CTTTATATAATCACAGTTTAGTGCAAATATGTTTCTGAACGATCAAAAATCAAGATTTAGTCTCA
CTTAAAGTTATATAACTTTTGTTCTGTTTGAAATATTTTGATGACATTTGGTCAAAATGTGTAAATA
TATCTTGGCTATAAGCCTGTAAAATTATGTTTTAAAATTCTTATATTTGACCGAGATATTAGCAAT

AATCTGAGGTGTTCCCATTTATTTTGGCCCACCCTGTAGTTTCCACCACTAGCTGCGACTAA
ATTGTTTAGGCTACAGGCTATAGAGGTAAATTTGCGCATTACGATTGTAAATGTAACTATACC
CGATTAATCCCAAAGTTTTTGGAATACAAAATAGCAGCATATGCTGTTCCATGTCTAAAAGCA
CAGAACAGAGATAAAGTGCTAAACGACTTTGCACAGGGATGAAACTATTGCTTTAAATTAGA
AGTCGTAGCCTATAAAATTCGAAAATTTCGATTCCGAAATAAGAAAAGCGATGACGCATGAA
CATGTTGATAATAAAGAAATAAAATAGTGCGTCTCGAATGAAAACGTTGCTTTAACCCTCGG
GCTACCAAGCTTTTTTTGTGACATTGACTACCAAGGGGGGGGTTCGAACGACCCCCCCC
TGGATTTTGGGTCTGGCACCACATATCATATCGCGTAATACATCGTTCGATTCAGCATTCATT
TCTGCACAAGACGGTATATTTCTTTTAGAAATACATAAATGTATACCGAAGTTATGCAAGATAT
ACCAAGGGCACCCGTCCAGTTCAGTCAGCTGCTGTTTGTACTGTGTGTGAGAAATTCTGAT
TGTATACCTCGACTACATCGGGTATAACTTTGGTATGGTATGGGCTATCAGCATGATATATAC
CTCAAACTGTGCGGAAAAGATCTAGCTATAGCTACCTGAAACGTCAAAATCATATTTCATAC
ACAAAAGGCGCGAGATCCCAAAAACCTAGGGGGGGGGGTGGTTTCAACCCCCCCCCT
TGGTAGCCCGAGGGTTAAAACAACAGGTTGGCATAAGCCCTATAGGGCGTTAGAATTAGC
GTGATGAGCGTCCGCGGGAATTCACCTATAGGGCCCATTAGGGCGCAAACAGTGATTAAT
CTAGACGTTTCTGGGGGGAGTTCCCCCCCAGGAAAATTTTTGTAGGACTTTATTACTATCCA
CCATAGTAATTCCCCCCCCCCCCCAGAAACCTTGATTCCCCCCCAGAGTTGGGGACTAA
TAAATGGGTTTAAACACACAGGTATTAGTTTGTAGTCCAAAATTTCGGAATTAGTTTATCACCA
TTCCAAAAAGTGCATCTAGTTCAAAACTTCAAATGGCAGACAATTGAACCAAGGACATTCA
GACAGCGAGCGAACTTAACACAATCGATCAGCAGCCGAGGAGAATGGATTTCCGGCGAT
GACCAGTTGAGACAGTGACGTCGATCGCTAGGTGTCGCCAGCCGCGCTGACTCTGTCGT
CGTCGTCGTCGTCTGGTCCTGCGAGCAAACAGTCATTTCCATAACTGCGTTAGACGACGT
CCTACGCAAACAGCGATCTCATGATTTTCGACAGGAATAAAGAAACTCAGACTCAATATCTT
AACATATCTGACTGTGCGTTATGATCTGGCGGTCAATCCGTCTGTCTGCCTCACTGTTTGTGT
GTGACTGTGTAGACTCTTTGCATGTCTGCATGTGAGAAAAGAATAAGGCACGAGTAGAGATT
AAGCGTGTTTATGAAGAGACAGATTAAGAGACAGACAGACAGACAGACTGATAGATAGATA
GATGGATGGATGGATGGATGGATGGATGGATCGATAGATAGATAGATAGATAGATAGATAGAT
AGATAGATAGATAGATAGATAGATAGATAGATAGATAGATAGATAGATAGATAGATAGATAGATA
GATAGATAGATAGATAGATAGATAGATAGATAGATAGATGATAGATAGATAGATAGATAGATAGATAGA
TAGATAGATCTGATAAATAGATAGATAGAGACAGACAAAAAGACAGACAGACAGAGACAGAT
AGACAGAGACAAATTACATGTATACATGTATGTACCGTATAAACAGAAACGGATTATTTATTGT
CATGCAGTGATTCCATGCAACTTTGACCACAAACGAATAATATGGCTGCAAACAATCTTAAA
TGAAATGAAGTTTCTGGGATTCGGTGACAGAAAGTCAACTTGTAGCCTAACTGAACTCATAA
CCTGGCCTAATATCATACAGTAGGCCCAATTTCTCACTGATAAAGTTGTGTTCAGCATTCCG
AATTTCACTTGGAGTATGGACTGTAGGTCTATGCATGAGATGTAATGTAATCTAAAGTGAAAAG
TCGTAGATGTAGGCAAATTTCTGCTTGTAATATGATATGCATATGTTTAATAAGTAAATAAGTAA
GCGAGCGATCTTTATTAGTACGAGACAAGCGCTAATTCGTCACAGCAGCTGCTGCATGTG
CAGACTGACTGTGGCCAACTACATGTAACTGACAGAAATGGATTCGTCTGCACCGCAGGA
AGAGGTCACGCAGCAATATATTATCTGCGGTCGAGATGATCGAGAGTCGACGCCAGTCGT
CAGCAGCCGATATATTTTAGGAGCTTTCCATTGATGAGACTCCGAATCCATCATCTGAGTGG
AAAAGCGTGTGCAGGCGGAAGTGTGGAGGAACGAAAATCGATGGATGGCGGTGCTTCTG
CCGCTGCTTCTGAGGCTGCTGGCCCTATGACCACTGAGGCGTTTTTCCTTGGGGGAAGA
AACGATGGAGAGCAGTGAAGGCCTCGGCTTTATGGCGGTCAATGTGGTCAAAGAACAAA
GACACAGGGGACACTAACATCAGGGAGGAGTGACCCATAGGTGGACAAGACTCTTTGTC
GGGATGAACGATACAACAACCGAGGAACAAAGGAACGGTAAACCGACAGTAGTCCCAAC
AGTCTAAAGGCTTAACnnnnnnnnnnAGATAGATAGATAGATAGATAGATAGATAGATAGATAG
ATAGATAGATAGATAGATAGATAGATAGATAGATAGATAGATAGATAGATAGATAGATAGATAGA

TAGATAGATAGATAGATAGATAGATAGATAGATAGATAGATAGATAGATAGATAGATAGATAGATAGAT
AGATAGATAGATAGATAGATAGATAGATAGATAGATAGATAGATAGATAGATAGATAGATAGATAGATA
GATAGATAGATAGATAGATAGATAGATAGATAGATAGATAGATAGATAGATAGATAGATAGATAGATAG
ATAGATAGATAGATAGATAGATAGATAGATAGATAGATAGATAGATAGATAGATAGATAGATAGATAGA
TAGATAGATAGATAGATAGATAGATAGATAGATAGATAGATAGATAGATAGATAGATAGATAGATAGAT
AGAAGCCGAGCCAGAGACCGAAAGACAGACAGAGAGGCAACGAGACATAGAGACAGA
GAGGCAGAGAAGGAGAGAGTACTAAAGGCCAACGAAAGCAGTGAACCGTACGAGGGAG
ACATTAGTCGGACCGGGTGACAAGACAGCCACGCCGACCGCTCACCGGATAAGACTCG
GCCGCACCACTGCCACAGCCGCCGTTGCCACCGGACCTGCTCTCCCTCACGGTCGGT
CGGTCGGTCGGTCGGTCGGTCGGTCGGTCGGTCGGTCGGTCGGTCGGTCGGTCGGTCGGTCGGTC
GGTCGGTCGGTCGGTCGGTCGGGCAGGGAAGAGTGAAGAAGGAGGGAGAGGCGAGC
GGACCCTCTATGCATCGATGGATCGAACGTACACGGCCTTCATTTAGGGCTTAAGCGGTC
TTGGCAGCGTCGGTGACCGCGCGGGCGGGCGGGCGAGCGGGCGGGCGGGCGGGC
GGCAGGCATCGAAGTGGTACACAGGCCGCGCTATAGGTGCGTCGTATAGCGGCTTTCCA
GGCGTCGTCGGATATCGATGGAAGTGGAGCTCGAGGGACGACGATGGAACAACAGCGA
CACCGTCGACCGTCTCTGCAGTCGATCGCTATGAACAGCCGTAGACGAAGTCATCGACC
GATAGATGGCGCTTCTGCTCCAAGGGATCGGGTTTAACGACGACGACGAAGATAAGAGC
CAACAACAACAGCCACCGGTTGGTTATCAATCAAAGCCCCGTCTATGTATGCTATATTGTAG
TGTATTGTATTGTTATGATGTGCTGTTCTGTTTGAAAACACTGCGATTTTCTGTCGAAGTCATTT
ACAATTAATCATAATTAGGCGTCTTTATATTGCGCAAACAGACTTATCATAACTATTATGGCGA
TACATGAAATTAAAAAATAGAGACTTAACTATCATAAGCTATCAGAAAAGTACTCTGATCAGAA
TAAGCGATGAGAGCCGTCGGTGGAGTGCCCTCTCAAACTCGTGTAGCTCAACACTGATTC
CTATTACGCAATAATATGTCTTCAACAGAGGCATTAAGAAATGATGAAGCATTGTAGGGAAAA
TTGGCATAGTCATTATTGTTAGATGAGACAAATTTGTTGATAGTCTGAGGACAGCATAATATATA
GGAGTATATATAGGGGCCTAGTTAAAGGGACCCATGGAGTTCTAACGGTAACTAGTTGTAAC
TTGCACGGAACGCCTTTGTGTAAGGGAGCAGGCTATATTATAAGTGATGTCTTGAGGGTTTT
CAAGCTTTTAATGAAGTCCGCCATGATATGAGGGACATTTAAAGTAGCCAAACAGAATTTGC
TGTGCTGGAATTCATGCAATTGTAGGGGTAGAGGTGAAGCGAGACTGATTGGAAATTTATTC
GGCTGCTATAGGCCTACTGTGAATTTTAATGACTTTTGAACACGATTGGGCTTAGTCTGAAAA
CGGTAAATTCAAACATTTCTTAGGATTTAAGGCCAATGTTGCAAAGTCCTTACGTGATCTACA
TTTCTCCCGCGAAGCGAAAACTCATTTGCGGCGCTTTAATTAGTTTCTTGATATAGCGTTTC
CATGGTAACAGCACACTTGCTAACGACGTCCACAGAGGTTTTTGTTCTGTTACCATGGTATC
CGTAAGAGCAGACGCCATCGAATGTCTTTGTTATTTCAAGAATCGTTTTCTTGGAAGCGCTG
AGCGACACCTGTCCTGCGTCTGCTGCGAATTATGTCCGACGGATTCCTCTTCCGGTTTGC
CTTCGTAGCCTCAGCCTTCCGCTGCACAGAACAACTCCTTCATTCCGGTCAGTACCCAGA
ATGCACGCTGCTTCCGGTTATTCTATTCAAGGCCATCTTAACAATATATAAATTTATATATATAT
ATGTATATATATATACATATATATATATATATATATATATATATATATATATATATATATATATATATATGGTA
CGCCTAGGTATGGATATGAAGAGTTAACGACTTTACTTTCTTAGCTTCGGCATATGAGGATGA
TATGAGTGGTCAAGGCTTTCCTTGCCGTATAGGTATTGTTTATATTATACTGCATACTGTCTGG
ATTTTGTACTATCCTTCGACCAACTGTCAAGCGAAGGATTTTCTCGCGTCCGCTTATTAGGC
CACAAACCTACAATGAAAAACAGTTGAGTTCCAAACGCGGTTGAACCTGACTTTACCTGAG
GGTTGATGGGTTGAAACCGCGGGTTCAACCGCGGTTGGGACTCAAAGGGTTAACCCTCG
GGCTACAAGGGGGGGGTGCAAACTCCCTATCTTTTCTGGGTCTCCCACTTTTGTATATATGA
AATTTGATTTTGACGTTTTAGATAGCTGTAGGCCTATAGTGCATGTATACGAGAATCAATTCCG
CACAGTTTGAGGTATATAGGCCTATAGATCATGCTGATAGCCTATACCTAAAAGCTTACCTCT
TCTCCAAAGCATACTCTGAACTCACTGTGCGTTTACTTATGTGAAGGCGCCCTTGTAACGG
AATGTGCTGTGCTACGGCGCCGTAGAAATTGTCGCTATTATAGGAGTGAACCGGGTATCAA

TTTCCTTCCGGGGACCGGGTATTACGACTGTGTAGTGCCGGGTCCGAGTCCGGGTCCGG
GTATTTAGAAATGGAAAAAAAATTATATAGAAATTTCTTAAATTATATAGAAACGTCTCATTTTTA
AAGGTAATAAAATTCAGCGATAATTTGGCGGAAGATGTAGACTGTTTCCCCGAACTTCCAAT
AGTCTGCTTCACCGACCACCATAGCGGCGTAGCCTGTTACAGTAGTATAGGGCTCTCTCA
CGTATATGTCATGATCAGTTGATTTGTATACGCAAACTCGATAGTTTAGCGCTAGGTGGCCAG
CCTCAACGGACGCCATCTTTGTGTCCAACGAGGCTACAGAAGGACTTTCCGGTCCCAAA
AAACGGTATATGGAAATTTTTTAAATTATAAAAACGTTTCATTTTTAAATTGTAATATGATTCAAC
GATAATTTGGCGCTAGATTTAGACTGTTTCCCCGAACTTTCAATAGTCTGCTTCACCGACCG
CCATAGGGGCGCAGCCTGTAGTATAGAGCTCTCTCACGTATAATGTCATGATCAGTGGATTT
GTATGCGCAAACTCGACATTTTAGCGCCAGTTGGCCAGCCTCCACGGACGCCATCTTTGT
GTCCAACGATCGAGGCTACAGAAGGACTTCCCGGTCCTCTGTATAATAGGTAGGGACAGA
CGGAAAACACGATTACACGGAATCCGTGTTTTACGTTTTTTTTTATTTGCCGTGTTTTCCGTTT
TTTTTGTCATTTACAATCAAGGACCTATTATTGAGATGTCCTTGGTTGATAGGTTGCGTAGGTA
TGATAGGTTGCGTTCGGAAATGCTCGCACGCGCTAGTTATTTTAATATTTATTTGAGTTGGAG
CACCAAAGACCAAAGTCACCAAACGAACTAGTCTTAACTCAAAGGAGCTTTGTACTACTAC
TTTTGTATGATATTATTCGGTAATAATTATTTTTATTAATATTGTATTAAAAAATAATCAGTACCCGG
CCACATTATTTCTCAGAGTCCGGGTAATACCGGGTATTAAGATACCCGGCAGGGTCCGGG
TACCGAGCCCAGACTCGGTTCACCCCTTGTTATTATTATTATTATTATTATTATTATTTATTAT
TATTATTATTATTATTATTATGGTTGTAGCGGTTGTGACGTTGGAAATGGAAGTAGTAGTAGCAG
CTGTAATAATGATGAGAATAATAATGTTAATAATAATAATAGCATTAATAATTTATTAATAAGTATCT
GACACCTGAGGGATCCTAAATACTTTGGGTAAAAAATAATAATAATAATAAAAATAATAATTATT
TTTATTATTATTATTATACCATACCAAAGTTACACTCGATATAGTCGAGGTTTATAGGCAGAATTT
ATCGGCACATCAATCAGAATGCAGGTGTTTGTACTGGATGGGTGCCCTTGGTATAGCTTGC
ATAACTTTGGCATACATTCATGTATTCTTAAATAGAATATAACGTCGCAAAAAAGGTAGCCCG
AGGGCTAAGGACAAGATTAGGACAGCGATTAGGAAGCTGTGCACTTGTCATTGGCTGATG
ACACTCATGTGGCTTAGGACTCGGGAGCCCGGATGAGCTGAGGCTGATCGAACACCTGT
TCCTCGCCCACGGATACAATCCACTGATTCGCCCTGTGAGGAATCTCTCCGACACCGTTC
TCGTCAAGTTCGGAATGGCCATGATACAGCTCATCAATGTCGTAAGTATACACTCCAGACAT
GTCACTCTTATTTACACTGATATGTATTATGTGCATGTGACAGGATGTGGAATGAAAATGTTAAT
AGTTAAACAAGAAGTCTATAGAATGAGAGATGAAAATGTTGATGCTTACATAAGAAGTAGAAT
GAGGAATGAAAAGTTGATAGTGAAACAGAAAGTGGGATGATGGATTAAAGAGGTAAATGAG
AGATGAATACGTTGAGAGTAAAACAAAAAGTTGGATGATGCTTGAAACTGCTGGTACTAAAG
CAAGATTATTCGGAGCAACTTCTGTTCTGTTTAAAGTCTGGCATGTCTGGCTGGTTGGCTCG
GTCATTTACCTGCTTCGAATTATCCGACTCCGAACGTTACTTCCCGGGTTCCCTCTTCTGTC
TTGATCTTCTCATGTTCGCTGCGTATTACAAATTAACAAATGTAACGCAATGTTAGCACACATT
CAAAAGCAGTGATTAATCTAGACGTCTCGGGGGGGGGGGGGAAGCCCCCCCCCAGGATT
TGTGTTCCCCCTAGAAATGGAAACATTTAGGACTTTATTACTATCCAGCATAGTAATTCCCCC
CCCCCCCCAGAAACCTCGATTCCCCCTCCAGACTTGGAGGACTAGATTAAAGCCTGATTT
GCATGCCAGCAATGAGAGCTTGCACTCAATGCTTTAACCCTCAGCACGTCCTGCAGTAAT
GGCGTCACAATGCCAGCCGATTACACACGAGCCTCGCCCGCTGCATTGAGCACTGACT
GAAGTCTGTCAAGTGTAGGCCTAAGTAACCAGGCGCTCCGGAAAGTATTCTTTTGCACCTG
TTGACCTGCGAGACAACGAAGCAGACAACAGCCGTTTTTGCAGTGATTAATCTAGACGTCT
CTGGGCGGAGTTCCCCCCAGGATTTGTGTCCCCCCCAGAAATGGAAAAATTTAGGACTTT
ATTACTATCCACCATAGTAATCCCCAGAAACCTCGATCCCCCCAGACTTGGGGGGAATTAA
ACATTAAAGCCTGCCGTTTTTGCAGCCTCGAAGGAAAGGAACCTCACGCATGTCTTGATTA
GCCGTTGCTAACGGAAACACCTGCCAACTCTCTGTCTCACCTGGTGCGACATGGACAATA
ATATTATTGGACATGTCTGACTCAAACAACACACCAAGATCCCTCGCAGCTGTCGAAGATG

TCCGCCGCACATTACATGCAAAACGAGAGAAGAAGCAGCACATCGAAGTTGCACATCCA
GCAGTCACAAACGGCGCCGTATACTTGTATACATGCAGGTCTCAAGGAACACGTCTGAATT
TAAATAGTTAAACTATACTTGTATAAACATACATAATAATAATAATAATAATAATTTTAATAATAATG
ATAATAATTATAATTGCAGCTCTTTAGTGGAGCCAGGTCTCAAGGAACACGTCTGAATTTAAA
TAGTTAAACCATTCTTGTATAATTGTAAAAAAATAAAAATAATAATAATAATATTAATGTTACCTCG
GGCAATTTTTAAAATAATAATAATAATAATAATAATAATAATAAATAGTCGTATCGTTCGAGATCA
GCTGAGATCGCGGGCGCTGCTGCAGAAATAGCAGCAGAGCGAAAATCCTCAAAATATAC
CGAAATACTTCCTGCATACTGTTTTGTGCCCCTGGCCTTCGAAACTCTAGGCCCAATCAAT
CACGAAGGCCTGGCTTTTTTTAACAATCTTGGTCGCCGTCTTGCTCAGGCATCTGGAGAC
GCCAGGGAGACCACATTCCTCTATCAGCGCTTATCTGTTGCCATCCAACGCTTCAACGCA
ATTACTTTTCACGGCACCTTTGGCACACAGCACGACATCGACGAAGGCTAGCCATCCCA
GAAGAAGAAAGCTATCCCTAATTTTTGAAATCCTGAGGGATTATTGATACCTCGGGTAAAAA
AATAATAATAATAATAATAATAATAATAATAATAATAATAATAATAATAATAATAATAATAATAATAAG
GTGAAAACATAATAATAATAACAATATTTGGGAGTTTAATAATAATAATTATTATATGTAAAACGA
GAGAACAAGCAGCACATCGAAGTTGCACATGCAGCAGCCACAAACGACAGAGTGTGGG
CGGGTGTTTCCGTAATAATAGTAATAAATAATAATATTTCGGGCGCGGCAGCGGAGATTGCG
TCGGAAAGGAAAAATGCCAAATAAAGTGCACTGTTGAACACACACGTTTTCGTGCCGCTG
GCTTTAGAGACTCTAGGGTCTAGGGCCCATGAACACCACTGGATTTAGCTTTCCATCAGAT
CTCGGGCGTAACTTGACACTGATTACAGGTGATGATAGGGAAACTAGTTTTTTGCTGCAGC
GTCTGTCCATCACTATCCAACGGTTCAACGCGGTGGCATGTCGCAGCTCTTTAGCGGAAC
AAGGACACATGGGAGACGGCTGAGTAGCTAATTTTTTAAAGTTTATATTTGCATGTAAATACTA
TGATTTGGAAAAATCAAGTTATAATATAATAATAGTAATAATAACGACAAAAATAATAATAATAGT
AGTAATAATACAGCCCTCTTGTACAAACTTGATTACAACTAGGCCTATGTCCGGTTGTTGTTT
GAGACTCGATCACTGCCCAAGTAACAGCATTGGGCTAATTTTCATCTCAGAAATTGGAGAG
CGCCTGTCTCCCTCGAATTACTGGAGCTACTGCAGAAACCACTATATTTTTCAACGATTCTC
CAGCGTGATCTAACGCTGATTTCTAAGACACCTTTTCCGAACCAGACCTATGATGTTTACGG
TTTCTAGCGCTTCCAAATTGTAATTTCTATTTCTCCGGAACATTTTAAAATTCTCATTTGATAAAT
GATTATTTCACCATTTAGGAATATTTTCACATACATGTATGTTCACACTAGTTAGATTGTCAAAAT
ATACGTATTTAAGTTGCTGATTCAATTGCTATGAATGATGCTAAAAATGGTAAATATCCTGTAAC
CAAATACCTAATCAATCTTTTTTGTGTGAAGTAGAACTAGAAAGCAACGTCAATCACCGTTGC
TCAATCTAATTTGTTACTTGGAAAATAGGTGACCATAGCTTAGGGGACTTCCATCTCTTCAGG
GTCTCCTTGTTACCCAACCATAAAAATTGTAGGACCTACCTATCTGTAAATGTAAAATATTATAT
AGAAAGTTGATTAGCAATAAACGATCGATTGAGTTATTGTGAATTCATTGATACCTAAGGTGAA
ACTGATAATAATGGTTCCTCGTCATTCAATAAGATGACGATAATATTAATGACAATGATATGAC
ACCATAAGCAGTTGCACTAGCTTTGGAAATGTTGAGAGCTGTAAGTAGCAGCTCAGTCAAT
CAATCAATCAAAATTTATCGTCCCTTCAGGTATCTACTCAGAAGCGCTGTGGTAAAAGTTGTA
CCTGCAACTTCAGTAGTATAGTCATGACCGTGCTTATTTTGTAGTGTATGAAAAGGAAGGTTT
TCTGGCGATTCAGAAGGCCGTTGACATGAGTCTAATTGCCGAAGTGAACCGAAGTTTCGA
CCCAATGACGACGAACGTAACGACTCAGCTAAAGAAATATCCGTTTCCTCCATACGTCAAA
GACGGCATGATTAGTCAAATTAGACAATTGATACCACTAATCATTATACTCAGTTTCATTGTCA
TTGTTCCGTCAATCTGTCATGAGATTGTCCTCGAGAAAGAAAACAAATTCAAGGTACAGCCA
CCGAAGTAAATAATTTTCAGCTATACTGTTACGTTTGCTCTAGGATGAGAACCACTTCTATAG
ATGTGTCATCATCATCATCATCTTCATCATCATCATCATCACATCACCATCATCATCATCATCA
CCACCACCACCACCACCACCACCACCATCATCATCATTATCATCATCATCATCACCATCA
CCACCATCATCATCAATCATCATCATCATCATGGTACAAGAAATGAGAGTCCATAGGTGTCC
AATGTAAATTTTATAATTAGTTTTCAGAGTTCCCGATCCTGTGCACATAGGCCTACACAAAGC
TCGAGGGTACGCGTACCCTAAAATAGAATACGTTAAAATACGTACGAACCTTTCAAAAAATA

CAATACGCGTATGGTACGTTTCAGACACGACAATTTTATTTAGGGTTTAAGTTACTCGGCGA
CCCACATAACTTGATATCAACCGTATTTCATTGAAAACGGTACTTTTAGTAGCGTGGCAGTAG
GCCTACATTTAATTTGATAAATTCTTCAGTTATAGGCCTAACTATTTTAGTGATTTTTTGCTTAAA
TTAAATTGAACATTATTATAATGAGCAAGGGCTGGTTAACGTATTTTTGTATTATTTACGTACCC
TAAAATAGGGTACCGGTACGTTAAAATACGTACGTACCTTTCACAAAATAGAATACGTATGGT
ACGTACCCTCGAGCCTGCCTTGGGCCTACACTCAAACTGTGTAACCTAAATCTTGCTTAGA
CCATTCCCTATCCCTGATGTCATCCGTCCACCCTATTATCTCCTATACGCGCGATTAAGAAG
ACATTAATAATTATGAAAGTAGCTTGATAATCATTTATAATCTCTTGATTGCTGCATAAACGTAAT
TTTAGGTAAAACGCATACCATTTAATAAGTGATGACGAATTACTAGAAGAAGTCATTGCAAAT
GTCTTGCATGAATCACGCGCTTTGATGACGTCATTGATGATTAGGGCCTAAGCAATCGTAG
GCATAGACCACACGGCGACTCGAGCAGACCATGGTTTCCACTCGAATGCCCGACCACTT
CGTATATGGACAATATAAAATATTGTTTCCCAATGAGGCTATGTGATGCAGCAGACTTGCCTT
CATCATGGAACGTGGCGTGGGGTATCTACGGAAAGGAAGGCCACACGGTGTGTTTGGAA
GAAGAACCTACCGGTACCCTCTAAATTTATTATTGGTGATTAGTGGGAAACCCATTTATAGTA
ATTATACTAGACTATGCCCGGTCCTAACACGGGAATGCGCATATACTGTATCTATATAAATAA
AAATGCAAATCCTTATTTTCTACACACGAAGACATGGCCGAAAGTTCTTCACCAATTCCTTT
CAAACTTTACCAAAACCTTCCACTCCAATCAGCGGATGTTCCTTTACTTACTATGTTTTAATGT
AAGTCTTCCTTTCTTCAAAATCTTAAATCTAAAAACAAACTTAAACATTCCTCTAAAATAGCAA
ACTTCCATTGCGATACAATATCGCCATCTGGTGAATATCAGAGCAGCACACGCTAACGGGA
ACCTCTGACGTAAAACATTTCCTTTCCATAACACTTCGCCCATATCCTGAACTTGAAAGCAA
CACACGCTAACGAGATCACGGACATGCTTCTTTATAGCAGGCCTGTACCTAAAATGTATTC
CTTAGTATACCTTATTTCCTTCTCTTTCATTTATCTCACTCCTGTTCTTATTCTCAATTTTCGGAG
CAATTGACCTCATTCATCTGCGTTTTAATTTGCGGCCTAAATATTTCCTTACCATTCTCTATTTA
TATTTCTCTTCTCTTTGCTTCTTACTTCCTCTTCTTTCCTTTCTAATGTTCTGCAATTTCGTAGGT
TAGGCTATTCTTATCCGCATAACTTTCTTTCTGCCACCGCACGTACTGCATTTGAAAATAGG
CGTGCCAATTATCATGTGTGAGAGGTTCGACTGACCCCATTGCACGAGGGGGTAACGAGC
ATGATTGACTCTGGGGGTAAACTTGGAGTCGCTCTGCCACTGACTTGCACAACATCAGTGC
CCGTCCCAAGCCGGGAAAAACTGAGAGGGCGACGCTAAATCATCTTCGATATGGCACAG
CGTCGTTAAACACCTACTCTCAATTATCATGTTGAAAATATCAACCAGCCAAAGCTTTGCAA
CGGCACGCGACTTGCAGTAAAAAAATTACTGAGCGACGTCGTTGAAGCCACAATCTTGAC
AGGACCTTTCAAAGCTGAAGATGTGCTCATTCCTCGCATTCCTATGATTCCAACGGATATGC
CATTTCAATTTAAGAGATTGCAATTCCCAATTCGATTGGCGTTTGCAATCACCATAAACAATA
AACAAGGCTCAGGGACAATCTTTAGAATTGTGCGGTTTAGATCTTCACACGGATGGCTTCT
CACATGGACAACTATATGTTGCGTGTTCTAGAGTCGGCAAACCAGACAAACTCTATATATAC
ACAGACAATGGAACAACGAAAAATATTGTATACCAAAAAGCATTGCGAAATTAAATATTTTAC
AAACGTGCGCGCTCTCTTTTCTTTCTTTCTATTTAACCAGATTGAGCCACAGCAACGCGTG
GCCGGGTACAGCTAGTCATTAATAAATTGTAAGGTAAAAATTGTCTCTTACAAAGCGCCTAC
GGCGCTCAGGGCCATCAACTGAAACTCCAGACCACATAATCTCTATAATCACTGGGCGTC
CTTCCTATCCGGTAGATTCTCCACGCCCCGTTCATTGCATCCATAAATTCACGTCTGGTGC
ATCACGTTACCACACTGGGAAACGCTATTATTCTCCTTATAATTAGCTACACCGAACAGTCA
CATGATTAATAAATAGGCCTAAGCAATTTCTATCGAGTTTCGTCTACAAATTTTCGTATTGGCC
AGGCGCGATTCCATAATCTGAACCAACCAGGAAGAAGCTAAAGGCATAGAGGGGCAGTC
ATCATCCAAACGCCTGCGCAGAACAGTAGGCCTAACCGCTTTACTTCGTGTCTATAATGGA
TGGGCTCTGGAAGCTAAGCGAACAGATCGGAAATGATTTATCTACTATATATAGAAGAGTCT
CAAAAGTTGTTTGCCTTTGGGTAAGTCCGCTATCGAGAGGCTGCGTTGAATTGGTAGCCAG
CCATATATAGGGACATCATTTTCCCCATATTACGGGTCTACAGGTAATTTGTCACTAGGCATA
ATTGACTTGATAGGAAGAGAACTCAAGGGTCAATCCAAAAAGGTGAGAAATGCCAACAATT

AAGTTAACCGATCCATGCGCGTGCACTATCTTTGTGAAGAAATAATTACTGACCCTCTAGCT
TCAACCTTGACGAGACCAGCCGAAGATTCGCATCGAATATTCCCAGATAATAATTCTGTAGA
ATACATATTATCAACGTAGGCCTACTTAGGCATGATAGTTATGATCTCCCTATAGATATTCGTAT
GATAAATTTCCATATCATTAATTTGTAATTGATTCGGGCGAAAGCTTGGAGGTTTAGCAAATGT
TCAGATATATTTAGGCGTGTTCTCGTTTGTAAAACTTGTGAAGGAATCGATGAAGCTGATGGG
ACTGCGTACGTGGGTGTATTGGGCATCGTGGTTTGTCAAGGGCCTGCTGTTCATGCTTACC
TCTGTGATTCTGATGACGCTCCTAATCCACGGTTCGATGACGTCCAGTGGACCAGTGTTGG
CCAACAGCTCATCTACCGTCACATTCGTCTTCCTTCTACTCTACTCCCTCTCGCTCATCAC
CTTCTGCTTCGCCATTTCCACTCTTGTTTCCAAAGGTTAGTCTATATAATAATTAGTGTCTACTC
AGAGCCTCAGACAGATTCACACGCATTGTCGGCAAACATTGGCGAGATAATGCGATATGA
GGCTAAATGTTAAGACATAATAATTATAGGACCTCATATGTTACAGGGAATGCGTATAATCCC
TGAAATTCCTGGGAATTCGTTGGTCTATATCTTACCTGAAGATATTAAAGCATCCGCAAATTT
GAAGCGGACTGGTTTCACACGCATCTGCAGAAAATTTAATCGAACTTTAATATTAGGCGTTT
TATGCAACATGCGTAGGCCTACATAATACATATTGAATTGCGTTATATCTTATGCATATATAATAT
AATATAATATAATTCGTATACATATTATAGGCCTTTATAGGGCCTATCGTCGTCCAATTTGGATT
CTAGGACGATTGGTCACAGAACGTTTGGTCAAATGGCCTATTGGTCATATGGACTTATTGGG
ACTATTGGTAATAATTTTATAGGAAATATTGTTCTACAGCCTATTGTCATTCCGGCCAATAGGG
TATTGGGTATACGGTCAATCGTTCATAAAGGAAATGTAAATTATACAGGCTTACATGTATTTATT
TCCGTGCATTGAAACAATGTCGGAGGTCCGAAAGCACAAAGTAATATCGCTCGTATAAAAG
GTGCAACTAATAGCACAGAATCATTATCTCGTACGCCTACATAATTTCCAAACAGAGTATGTT
TACAGAAGTAGCCTACATCAAATCATCAAAACATCCTGTTATCAAAGCTGTTTGAAAGAAATA
AGCCTATAAGCGATAGCTCACAGGCAAGTCAGAATATCGGTTTGCCCATATGTAACCACTG
CCAAAACGCGCGACACGATCTTTAAGTTGGCCCTAAACGATATCGCTATACGCTTGGTTG
CTCAATACCACCGCGCCTTGGAAGAAGGATGCTTTCCGTTTCGTACACTCCTTCTGTAGTC
CAGAGAGGAACTTCCACATCGTGGATTGACTGCATTTTAAACAATGTCTGAAGTCCGTGGT
GCCATCCTCTTACGGGTAATCCCGTAGTTTAGGTCAACAAACGATTATCATGCTGTTCCACG
TTTCCATTTGACTTTGTACAGAGATGAAAGTATTAGTTTGAAAATAGAAGTGGGATGAAAGAC
GAAAATGTTGATTCTGAACCAAGAAGCGGGATTTGGAAGTGACTTTGCACATAGATGAAAAT
ATTATTTTTTAAATAGAAATCGGATGAGAAACCAAAATGTTGATTGGGAAATAAGAAGCGGGA
TGATTTATGAACATGTTGATAATACAGACGCAATTACTTACGGATGGAAATGTTGATTTAGAAC
AAGAAGTCGGCATGCATATCAGAATTTCGAAGCCTTTGCCACAGACTCCATTACTTTGTTGA
CCTAAACTACGGGATTACCCTCTTACGTACATGTAGGCCTACGTTGTTAGTCGTTCACGCTA
TTCAGCCGAGTATCGCTGGTTCCATGGTGCGATGTCGAACAGTACTGTTCCGTAATTTGTG
CCGCTGCCTCTCAGACGATGACCTCTTATGTAGGTGCGCTCGAAGTAGGCTATGAGATCA
GTTTTTTTACTCTATACCACAGACTGCTGGCATAGATTAAATGTGACTGTGTTGTGTCTCTATCT
ATTGAACATTTACTTCTAAGTATCTCCTGTTTTAGTTTAAGTCGGTGCATTGGGCGCTTTGATC
GTTTTCATACTGATGTTGGAGCTGAACACACTTTGCTTAGACGTTCGGAAATCTGCTACAATA
AACACACCATGCTGTAGCACAATATTTCCTATAAAAATTATGACTAATAGTCCGTATGACGAA
TAGTCCGCATGACCAATACAATCCCTATGACCATTAGCCCATATTATGATCAATAGTTGTGTAT
TCGTTCATGTCTTTAATATTTATTTATCTGCAGCTTTCTTTGGCCTAATCATCGGTTTAGTTGCTT
ACAATCTGTTGGATTTGCCTTTTAACTACCTGAACGAGGTCTATGACACTATGACACTTGGAC
AGAGGTTGGGAGCATCGCTCGACTTCAACTTAGCCCTCCATTTCGGATGGATCGTGATATC
TCAATACGAAGCCATCGGTACGTTACTGATGACTACTGATTATTGTGTACAGCTGTGCATGAA
ATTTATGAATCAGCAATTTACTATCACCCTACATTACTTTCTTCTGTGGGTTTGGTTTGCGAGT
CAGTAAGTTTGGGACTTTTGGCATATAATCCACAGTGTGTGACGTTGGCCTAACCCAGTGCT
GTCCAACCTTTCTGACCTCGCGGGCCGAAAAACAACTTATTCTTGGGGTCGCGGACCGA
ATCCATAAAAGACAAGGTCAACAACGTACGTTATATACGTCGTCGGAACAGAAGTTTATGCT

CACGTTTAAAAATGATGATAGCCACTCTATGTGTGCTTATAATACCTGAACAACCAATTTTAAC
ACACACAACATATTTTCATAACATCATATTTTATATTTTAGGCTTACATAATTCTTACACAAAATC
GAAATTTCCGGTTGTTTCGGCGGGCCGCACTAAATCACCCGGCGGGCCGCGTGTTGGA
CAGCACTGGCCTAACCGAATACTTCTAAAGGGCGCTGGCAATTAAGGAGGCTTAAGGTGA
AATTAGGATCACAATTAATGTGCTTTAAACCACACAACCTTTATCTGAGCTTCATCGACTTCAT
CTTTATATTAAATTTTGTTTTACAGCTGTGCTTTCTCAGTGCAGGACCTTTGGAATGGAACAAT
GTTCCAGCTACAGTCCGCAATGCTACATCTGTTGTTCAGTTTAAATCGAGACTAAAAACTTGT
CTATTTTCGTTGTACCATGACTAGAAATTATTATTATTATATTCGTTTATTGTAATCAGAAAGCAG
ATTGACAAAGCGCACTCTGGTACAATGACAGGTTAATAAAACAACTGAAAACAAAATGACA
AATTGACCAAAAAACATAGTTAAACAACATAGATGAGATGGAGGTGGAAGTAAGGAAATCA
GTAACTGGCATTAAACAAGACCCGAGGGATGAAATTACTGTCATCCTTAATGGGAAGTTCA
AAGTCGTGGGACCGTGGTCGGAGGCTATAGGAACATGATCGGGTGGGAGGAAAAAGTGG
GCGAAGCACGTGGGAATCGTTAGTAGCCACAGCACGTTTATGTAGAAGCTGACAGTCTGC
AGACTCAGCAAGATCAGCAACGCTGGGGCCCGTCCGAGGCAGTGCTATAAGCGCTCTC
GTGCAAGACATGTCTTGTGAAAGCGAGCTACAATGTTTAGCTGTAGCTTATTGCATTGTATTG
TAATTTATGCTACACAACTGACGAGGAGGCGATGCGGTTAATTGTTTTGAATTTACTATAGGC
ACAGGCGTTAATTGGAGCAACATTTGGTCTCCTGCGAAAGTAGGAGACGGCCTAACGATG
GGTCACATTCTGATTATGCTTTTGGTCGATACGATCATTCACTCGGTGCTTACCTGGTATTTG
GATGCTGTTATACCCGGCGACTTCGGAACTCCGCAGCCGTTCTATTTTCTCTTCACGGTTA
GTATTGGTACTTTATTAGAGGCTGAACTGGATAGATATATCTCTGCTCTCGGGTAGGCTATTG
GATGAAAAGCTTTCACGGTTAAGCTGATTATACGTTGTGAGTTTGTCGCTTTAAATAGATCGTT
CTCTACCAGTTTTCTGAAAACAATTATTGAGAGAGTGAACTTTTAGTAGGGCCTATTTGGAGA
AAAATATTTGGAAATGGTAATTACCGAACGCTGGAGATAAATGAGTGGTAATGAGAGTGTAGT
AGGGCCTATATTGACCACCAGACCTAGAGACAAAGAAGCCAACTTCCGTTTAAGTTAATTC
AGTCAGTCCAGAGCCATAGATAGTGCTCTGAGTCAGTCTAGTGAGCAAGCCTCAAGTTGTG
TTACAATAAAATAATTGTTCTATAAATTCTATTAAAGTAAACATTACAGGGAGCATTATCATTTAC
TGTGGAAATTGCACGTTTGCAGAAGAATTACTGGATGGGAAGGAAAAACGATAAGATAAGC
ATTTCGGACGAAGATTTCATGACTCTGGTTCCAGAAGAGAGAAGACCATATTTTGAAACTCC
AGCGACGAATGCTAAAGCTGGGATTCAAATCAGAGGATTGACGAAGGTTCGACCCCTTTA
GACGCAATAGCTGTCTGTTTAAAATCTCAATTTCTATTGTTTCTGTAACTCTTGCGAAAACATG
TTGTCCTGGAAACAATCATGTTGCATGCAATACAAAATGAGGATGTCTGAGGCTAAATTATCA
TCATGCTGAGAGTGTCATGAAAACTAAAGGCATAAAGTGACATTAGTGTCCATGTGTATAATT
CTAATTTTAAATTTATGTTACGACACTTTTTGAGAAGTCACGGGAATTCTTCTCTAACCCACGT
TAACTTTAGGCCTAATGATGCAATTTATGATGTCGAAATTAAAATTAAAAACAGTAAGATCAAT
GAGAAGCAACCTGCATACTAGTTCAAAAGACACACGGAACGCAAACAACGCGCGGATTC
TTTTTCTAGAGCTCACTGATCTATCATTATGCACACTTCTTAGATTAGTTTGCTTGACAATGTAG
AGAAATGACTATCAACAGTGAGATGAAATTTGCCGACTTCAGACGAATTGCAGAGTTCCGTA
CGTGGTTTGACAGCTAAAACGGTTGAGCAATGGATGAGTCGTTAGAGGGAGAGTTGGACA
TCATCTGAATTTTAAGATGGTGCGTGAGCAGACTGGGAAGTTGGAGAAGAGTCGTTTGGAA
TCTGGTGAGCCTACGACACAGTGTTAGGGGTTGCGAGGAGCGATTGGAGACTTTAACAAA
GTTTAACAAAGGATTAGCGGGAAGACATGATTATTAGTCTATAGATGACTGGAACCACAGCA
GAGCAGTGCCAGAGACGCAAAAACAAGTGGACCACGAAATTAGAGGTTTACACAGATAAA
TATTATTTCGCAAGTTGTACATTACAGCCAGACGATGGCTGTAGATAGAGAAATTAATTTTCC
GCTGGAGCGAATGTTGATGACCAGGCTGTAATCTAGTCCCCCAAGTCTGGGGGGAGGGG
GAATCGAGGTTTCTGGGGGAATTACTATGGTGGATAGTAATAAAGTCCTAAAAAAATTTTCTC
GGGGGAAACACAAATCCTGGGGGGGGGAACTTTTCCCCCCAGAGACGTCTAGATTAATC
ACTGGTTGATGACCTGAAATCGTCGATATAGAACTGATCAAACTGCGTAATATCCACTTTTGG

TGTAAACCTACTATTATTATGCATATTAGGGCCCACTAAGTAAGTTTTCTGTTTCTGTAAACTTT
TATTTGAAAACTGACTATTGTGACATTGTTTGAGAATAATTTATGCACGCTGTGGAAGTAGAAA
ATACTAGCAGACAAGATTAGAAAAAGACACGGAGACGTTTTTAGCCTGCTCTGTCAGGTTA
CCATCAACGATATCATATAGTGATCACTTTGAGAAAACGCAGAGTCTTAATTAAGGAAGACA
ATAACTTATTAAAATGCTAAACTCTGATATGAGGGCGACCATGGTCAGCGTCAGAGACAAAA
TGGAAGAAACATCTTTAGTCGACTCACTTCTACAGGGATAAAAGAGTTAACAGAAGAAGAA
GAAGAAGAAGAAGAAGAAGAAAAAGAAGGAGAGAGAGAGAGAGAGAGAGAGAGAGAGA
GAGAGAGAGAGTGGCGGTCTGGCCGGGGAAAAGATTTCGTCGACCCGAGCGAATAAATT
GCCCCATACAGTAAATACAGTAAATAGATCTTTCTTGTAGGATTTCGGTACCAAGGCAAATC
CAGCAGTTAATAACCTTAACATCGATGTCTATGAGGGAAATATAACCGCTCTATTGGGTCAC
AACGGAGCGGGAAAAACCACGACCATGTTCATGCTAACAGGTATACGCAATACGCTGTAT
TATTCTTCTATTTACCTAACAAATATTTCTTTTCACTTGTAGGGCTACATAATTGTTTCCTCTTTTT
CGTATTTTTCGTCTTCTGTTGTTCTTCGTCTTCAACTTCCTCTTCTTGTTTTCTCTGTATAGGCTA
TTCTTCTAGTTTATTTTATACTTTGGTATAAGTGTACATGTATTTCATCCCCTCTGTTAAAATTTTC
TGTTGTTTCTTTACATCTTTTGTTCCGTTATCTTATTCTCCTGTCATTAATTATTACTATCTTCTGTC
ATCGTCTATTTGTTTTTTTATCTTTGCGCTGTTTCATTTGTCTTGACGCGCGTCAACAGCAGAG
CCATGCATGAACATCGTCACCTCGATTCTTTTTCGCTATCGCTTAATTAGTTGTCGATTATAAG
CCCTATTCAGTGCTGACAGAAGAATTCGGTGACTACTGTATAAGAATTGTAAGCCCATTCTT
GACGCAACTCTTAGTGACTCCAGCCCAATTAGACTCTACAATCTACATTAAGCCCTAAATG
CATCGTATAGGGTAAAGCTTATACGCATCGTTACGAACGATAGTTTTTCGTTCAGCATTTGGC
TTTGCAGGATTTTTCCCTCCGTCGAAAGGCACAGCCTACATAAACGGTTTCGATATAACGA
GGGAAATGGATAAAATCCGGCAGAGCCTCGGTCTCTGTCCACAGCACAACATACTCTTTG
ACACCCTGACTGTCAGAGAGCACCTTCTCTTCTTTGCCGTTGTTAGTAGCCATGTGCTAAC
ATCATATCGTCACAACGTCGCAAATTCACCATACAAAATCATTCTCTTATATAGGCCTAAATG
AGAAAGGGTTTGAGTTTCAAAGTTATTGCTATAATTTTTACTGTTAATATGATAATAGGCTAATG
CTTCGCATTAAATATTTTTACTGCCATTATTGTAAATTATTATTATTGCCTATACATGTAAAATTATT
TATTTTACTACTAGTAATAGTATTGTAATTACTATCATGTGCATTAGTAGGCCTATATTTTTAATTTA
TAGTTTATTATGTTATTTTGTATTGAATTGTATTTGTGTATTTATATAGCGCCTCTCGTAGTATGAA
CTGTTCAGAGGCGCTCCCGACTACAGCGCTGATGCTGAGTCGGAGTTAACACGCCTTGT
GAGCAATTGTAGGTGAAGGACTTGCTCAAGGTCCATACGCAGCGGCTGGACTGGGAATC
GAACACGCGACCTATCGGTCACAAAGCTGTAACTCTCACCATTAGGCCTACCCCACTGC
GCTCCACGTATTTTCTTTGTTTATTGCCGTTACTATAATTATTATTAGGCCTACTATTATTAATATA
GATTTATTTTCAGAACATATTAAGATTCTTTCTAGAACTCAAGCTAGGCCCTACTATTTGTTCAA
TTGCCTGAGGTAGGCCTATACATGATATTTATAATGATTACTATTATTATTTATTATTGTTATTATAA
TTAGGCCTATTACTACTACTACTAATGATAATGCTGTTGTTAATATTGTTTACTGTAAAATGATGC
TTTCGTTGGGATCTTTTCCAACCTTGGATATTGCATGACCTTGACAATAAAGGACTGGAAAAT
TTGTACAGTGAGTTTCCTTCAATTGGAGTAACTCTAAGTGAGTTTATGCATAAAGCAAAAGAA
GCTGCTTCACATTTGGTTCAGAAATGACCATATTACGCCGCTTCATGTTCTATTCTGATCTTA
CCTTTCACACGACAGTGCAATCAAAATTACCACAATCGGCTGAATAGTCAATTCTTAGACG
CCACGGCCGTTGAAATCACTCGATAAATGTTTGACGTGTAGCGGTAATTACATAGAATGTTT
CGTAAACTCTGGGTTAAACGGATTATCGTTTAATGGACCGCCAAGTCTGGGGTTGAATTTAG
CTAAAGGGAAGCTCATTTGAAAAGGCTTCATTGGACGTGAACGAAATGATCAAAGTCTTGC
ACATGAAAGAGAAGATCAACGCACAAGCAAAATTTCTTTCCGGCGGACAGAAGAGAAAAC
TCAGTGTTGGAATTGCTCTCATCTCCGGTTCTAAGGTACGAAAAGCGTCCATAACTCATTGC
TACTAACGTTGATTTAAAATGCGCATTTAGAAACACGTAAGATAGCCTGGTGAAGTTGATTTG
ACTGTGAATTTAGTCTCCACTCACTTTCCATACACCAAATAATGACTGCATCCGGCTATAGTG
CTTGACCCACCCAGTACGATTTCCACCTGTCCATGAAACCGGAGGCAATGCCTCCATCA

CATCCGCGATCTCCAAATGAAGGCAGCAATATTGCACCAGCTCTCTGGAATAGTCTCCAGT
TTTCATCATTTGCAACAATAGCTTGTAGATGCACCAATCTGCCGTCAGTGCTCATCATGTCG
ACACTTATCCCTCGGCTACCAAGGGGGGCCCGGGGAGTTGACGCGACCCCCACCGTAT
GTTTTTGGGATCTCGCACTTGGTAGCCCGAGTGTAAACTTCTATTTTAACAACTTGTATCCTAT
CTGCTCTAGTTTTCAACTTAGACCTCACAAATTAAGACATACCATGGCAGTATGCAGCAGAA
CATAACCAAAAATCTGCTTCTGTTGCCGCTTTTCAGTCTGTTTATATTAAAGCCGTGATCAAG
ATGCTTTGTATAATATGTACCGATAGGGGGCCTACTGGATTTCTTAATCAGCTACGCCTTCTT
GGCTTTCTCGTAAACTAGGATTTTACCTAAACATAAGATGTCACACGACTTGCTTTCCTGTCC
TGCGCGGGCTTCTACCTTGTTTCAGCCAGCTAACGACGTTCTGCATTGAGGTGCAGTGTGT
AATCTATAGTCCCCCAAGTCCTGGGGGAGCGGGGGAATCGAGGTTTCTGGGGGGAAATT
ACTATGGTGGATAGTACTAAAGTCCTAAAAAATTTCTGGGGGAAACACAAATCCTGGGGGG
AACTTCCCCCCAAGAGACGTCTAGATTAATCACTGATTGAGGTGCCCATGACAGTTTTCTAC
CCTATGTAGATAGATTGGCACGTAAGACGTTTATGTGATGTACATGTAGGCTACTCTTGTTAAA
TCATAATGTACTCCGACATTGGGTTATGAGTTGGTTCGGTAAGAACATAGCTACTAACTTATGT
TATCCTGCGAGAAATGCTGTGCAAGAAGCTGGATTTGCAAATGTGAACCCTATTTCGGATC
ATATTAAGAGGAAAGGCGCCGTAATGCACACAGTGACTCCGAAATTCACCATATCGAAACT
TCAAAAGCCCATATCGGAATTTAACAGAATTTAAATGACCTATGTATGACGCTGAACATAGTT
GGGCTACCTAGCTGTGGGAGAACTTTTTTAAATTAGCCACATCGCTGTGGAGTTTTCTTTTC
ACGAGCCTCTGTTGAGACAGACAAGCTGCGGACCTGCCTACAAAATGTACAAAAAGTTCG
GAATATATATATATATATATATATATATATATATATATATATATATATATAGCATAGAGGTTAATTATATT
AATACAATTATTGCAGTTTGTCATACTGGACGAACCGACTTCTGGTATGGACCCAGAGACAC
GCAGACAGACTTGGGACATTCTGCAGAACCAGAGAAAAGACCGAACGATGCTTCTATCAA
CACACTACATGGATGAGGCAGATCTTCTGGGAGATCGTATAGCCATCTTGTCCAAAGGGG
AGATTGAGTGCTATGGAACGCCCATGTTCCTTAAAAACAAATATGGTAAGAAATTATCAGCT
GCCGTTAGTCATTGGCGATTAAAACTATGTTGTTGTCGATCAAACGGTCAACATCCACCAC
CCAGCGACCTCCCTCCCCACTTAGTTTCGTTGCTACATAATTCGCGTAATGTATGAACGCA
TTCTTTGTCTCATGGATGCATAGGCCCTAGCATACATGTACTTAAGTGCATAGGCGCCGACT
TTCGCTTTTGTCTATGGGTGCCACCCAGCTGCCAGCTGGATGCAAACTCCAGTGGGTACA
ACCAAAGACTGGTTATTTGTGGACGGGTGAAAACAAGGAATTTACAGTAGCTGAATGGAAG
CTCATTTCAAAATGCTTAATTGGACGTGTTAATATTGACAATAATATTGAAATTGTAGAGGTCCA
TGATGGTGAGAATGAGATCGTGCAATGTTGGGTAGGCCTAAATGTGTGGCTGTAAATCAATT
GTCAATTTTTATAATGGGATTTTGCAACTTAAGCCTATGTGTAAAATTGGAGTGTCAGGTAAGA
ATCCTAATGGCACCCACTGAACTTTCTTGTATTCGCACTCAAAGCCCCAAGACGTCGGCG
CCAATGATGTAGTGTTTTTCAGATGGATCAACATTTCTACTAGAATTTCACGTTTAGTAGGGC
GGATAAGCAAAACCATTGTGCACTTTGTGATAAAAGCATGAAATTTGGCACAGAGGTAGAC
CGATGTAATATGAACAAATTTGGATGTGGAGCCATCGGCCAACTGCCCTCTGGCGGCCTT
GGCGGCCATTTTCCAAAATGGCCGCCATTGGAAACTGAAAGACAACAGAAGTTAGATAAA
TTTTGTGATAAAAGCATGAAATTTGGCACAGAGGTAGACCGATGTAATATGAACAAATTTGGA
TGTGGAGCCATCGGCCAACTGCCCTTCGGCGGCCTTGGCGGCCATTTTCCAAAATGGC
CGCCATTGGAAACTGAAAGACAACAGAAGTTAGATAAATTTTGTGATAAAAGCATGAAATTT
GGCACAGAGGTAGACCGATATAATATGAACAAATTTGGATGTGGAGCCATCGGCCAACCG
CCCCCTGGAGGTCTTGGCGGCCATTTTTCAAAATGGCCGCCATTAGAAAACTGAAAGACA
ACAGAAGTTAGATAAACTTTGTGATAAAAGCATGAAATTTGGCACAGAGGTAGACCGATGTA
ATATGAACAAATTTGGATGTGGAGCCACCGGCCAACTGCCCCCTTGCGGCCATTTTCCAA
AATGCCCGCGAACACCCAGTGGACACTCGAAAGACAACAGAAGTTAGATAACGTTTACTG
CTGAAGGTTTTGGTTAATGTTTATTGAATGGTTTGAATGAAAACATGCCAAATAGTCTGTATTTT
AAATATATTTATTTAACAATTTTATGTTTCACATTTTAAATTATATTTCAATTATTTAAAAAAATACAA

CAATTGAGAGAGAAGAAATTAAAAATTTGTGTCAGTAGTTTTAAGAATGCGATTTCAGTTCTCA
GAGCACTCTCCGTCGCAAGCACATAGTGGTGTGCATTCCAGACCGGCCTTCTTGCACTTT
TTTATACAGTCTTTGCATTTGCAAGAGAGGAGTTCCCTGCAGATCTTGGAAGCTTCGGGTAG
CGTTGTCCAGAGAGGTTCATACACGCCGCTGGTCTTGATCCAACCCCAGTCGGTTGGTGA
TGGCAGTGTTGGTGCAGGAACCAGGGCCTGACCCCAGACATGTCTGCCCTGGTATACTG
CTCTTCGGACGTGCTGCTCCAGGGCTGCCCTTGTTGGTGGGATCAGCTGGACGTTGCTTT
TCTCTGCAAACAGTTTGCGACGTGCCTTGTCGATATCGGTGTCTGTGCTTGTTCTATCACAA
AGCAGTGTGATGAACCTTTCAATGGTGTGCATAGCGTCTTCATTAATACGATCTGGTGCAGT
GGACAGAAGAGTGATTGTGTGGGTCAGGTCAGGTAGCAATGCCCACACTGCCCACGCTG
TCCTCTTTCCACGTCCGGAGAAGCAGGAGACCGTGTCGCAGCCCGTCAAAGCATGAAAC
ATCGGTAGTGCCTGAGCTTTTTCAGGACCCAGAGCCCGAAAAATCTCATGGGCTGCCAA
GAAGCGGAACGTCTTCCCCGATCCGAAGGACACCCACACTTCTGCTGTCACTTGATGCG
CTCTCCTGGTTCGAGTGACATGTGATGTCCGCAGGAAGGAATCCGCAGTCCCGGCACTG
GTGATCTCTGCCTCAACCAGTGCTTGCACCCATCCGCTCCCTTGCATCAAGTCACCAATG
GTCTTCAGTGCTGCAATCTCGATATGAAGACCTCTGAAGAGGATGACGAACTTGTCTTCATC
GTACCTCTGTGGCCTCTTCCAATGGATTTGCTTTGCCATGGCAATTGCAGCGCAAATAGCG
GTTGGTCGAATGTGACCACTGGAATCTGGCCAGGGTTAAGATGTTCCACTGCATTCCTGAC
AACATCCATGGAGTGCTTGATCATAGCGACGTACTGTATGGGCACTCTCCAGTAAGAGTGA
GAGTAGTGCTTTTGGACAGACTCCCTGCTGGCGGTTTTGGACTGCAGACTTGCGTGGTATG
CAGCCCATGAAATGCTTTCTTATGGAGAACTTTCTTACGGCTAATCGGGTGTGTTGTAGCCA
GAGATACTCTGTTTCAATATGTTGCTTGAGGCCGATGTTGCCGCTCAGGGACGTCAGGTTG
GTAACCGGTACCTGTGTGTTCTTGATGTTGCTGTTGACGGATGGGACATCTGTATAGTACTGT
GGCAAACGGTCAACTGCTTTGAGACCTCTTTCGTTCAACCCTCCGAGGATAATGATGCAG
CGGTCAACACCGGCCCCATGGAGCGCAGAATGCTGCACCAGCGAAATAGCAGTCCCGT
GAAATGACTGTTTTGAGGTCGTTGAACTTGGATTATGGTATGTTGTCTACCTCGGCTGTGGTG
AAGACCATGCCTCTCATCTTGGGAGGGCAGACTACTTGTTCCTGGTGGTAGAGGCGGCA
GGCACTGTTCCCAACTGTGCGGAGAGGGACAGGACTCTTCTCTCTCAATTGTTGTATTTCTT
TAAATAATTGAAATATAATTTTAAATGTGAAATATGAAATTTTTAAATAAATATATTTAAAATACAGA
CTATCTGGCATGTTTTCAATCAAACCATTGAATAAACATTAACTAAAACCTTCAGCAGCAAAC
TTTATCTAACTTCTGTTGTCTTTCGGGTGTCCACTGGCGGCCATTTTGGTAAATGGCCGCCA
GGGGGCAGTTGGCCGATGGCTCCACATCCAAATTTGTTCATATTACATCGGTCTACCTCTTT
GTCAAATTTCATGCTTTTATCACAAAGTTTATCTAACTTCTGTTGTCTTTCAGTTTTCTAATGGC
GGCCATTTTGGAAAATGGCCGCCAAGACCTCCAGGGGGCGGTTGGCCGATGGCTTCAC
ATCCAAATTTGTTCATATTACATCGGTCTACCTCTGTGCCAAATTTCATGCTTTTATCACAAAG
TTTATCTAACTTCAGTTGTCTTTAAGTTTCCAATGGCGGCCATTTTGGAAAATGGCCTCCAAG
GCCGCCAGAGGGCAGTTGGCCGATGGCTCCACATCCAAATTTGTTCATTTTACATCGGTC
TACCTCTGTGCCAAATTTCATGCTTTTATCACAAAGTTTATCTAACTTCAGTTGTCTTTCAGTTT
CCACTGGCGGCCATTTTGGAAAATGGCCGCCAAGGCCGCCAGGGGGCAGTTGGCCGA
TGGCTCCACATCCAAATTTGTTCATCATTATTACATCGGTCTACCTCTGTTCCAAATTTCATGC
TTTTATCACAAAGTGCACAATATGTTGAAATATCCTACCTAAACTGCTGCACTAGTTGAACAA
CGTCTATGAGTTGTCTAAATACTTCGACGGAAAGGCATATGTTCATTTATACGTAGGCCCTAC
TATAAGGCTGGTGAGTGAAGGATTTGCTTTTCACTGAAACACTGTAATGCAAGTTGCGCATG
ACTAATGTACATGTATTATAAAGCATGCGAAACGACCAGATCGAGAGTGTTGTGGATGACGT
TCCAGTGATGTTTCTGCTATAGACTCCTCATATTAAATTATCCCACCATCGCGTGATTTCTACT
GCACGATGGGGAACACGTGGCATAAGAGATCGAGCAGAAACCACGAACGCTCGTATTCA
CCTACAATGTACGACAGTCTAAGTGTGTGATTTGACAAAGCATAGTCGTTTATGCTGCCATC
GTGAAATCGTGATACGATTGAAGACTGATTCCAGATTTTAAGTAATGTATTTGGTCAGATTTAA

GTGCATAGCCTATTTGACAATGAGTCCAAAATAGAATGAAATTCTTAGTGATTTGTACAGAGAT
AATAATCAGATAGCGTGATGTATTTGTGATAAGAAAACGGCCCGATGCCTCTGTCCGCCTCT
CTTAAGTTGAACGACGAGTCGAAAATTAAGACGTGGATTTAAGGCGTACTGTGTTGATACGA
AGACGGAAAGTTAGAATTTCAGTAATATTTGCTTTGTATGACGGATGGAAATATTGATATTGAA
ACAGAATTTAGAATACGTCCAAGATGGAGATACTGATTGCAGAACAAAAAATGAGATAGCAG
TAAAGCATGATATCGAGTAAATATTGATTGCTAACGGTAATGAATATACAAATAACTTACAAAAT
TAAATATATAAAATATTTTGGTGTTAAGATATTATTATGTCTGAAATACCATGACTAGGCCTGCAT
AAAATAAAGGCAGCAGCATTTAAGGACTAAAAATACATTTTCCCATGAATTATCAGCAATCGT
TGTCAATCCATTATTGTAGATTGACTAAGTTCTAAAGTACTTCTAATGCACCTTTTTAAAAGGC
GTTGGGTATCATCTGACGATAGTGAAGGCGTCAACTTGTGATGTTGATAAACTGCGTGGAGT
TATAAAGTCTCAAGTACCCAATTCCCACATGGTCAGCAATGAGGCTGAGGAGGTTACGTTC
CTTCTGCCGAGTCAAGAATCCTCAACATTCGAGGCACTCTTCGAGACGCTGGAAACTGAT
GGCAACACTCTAGGTATCAAGAGTTTTGGAGTCTCGGTCACAACAATGGAAGAAGTCTTTAT
CAGGTCAGTCTTTCTAGACCTTGCATCAAACACCTATGCGAAGCAGCAACTTGGTCAAATT
GGAGCTTTCTATATTCGGGTCGTTTTCAATAATTGTTAGAGACAACAAAATCTCACGACCTGT
TCTCATCTGACGCAAAGTTTAGTTTAATGCGTGCATATATATATATATATTATATATATATATATATA
TATATATATATATATATATGTATATATATATATATATACACTGTGTCCCAATTGAACTGACACCCTTT
TTGTTTTTGCGATAAATCTGTCAATTCTAAACATAATTCAATATAATTTCACAGATTGAAAGATG
GATAAATTTGCAAGGTATTCCGAAAATTTTATCAAATTCAATCAACAAAGTCCAAAGTTATGC
GCATTCAAATTTGACAAACAAAAATTTGCACTTTCCAACATGAGAAATTTGACAATATGCGTA
TTTATAAAAGCAACTTTACATGAAGATATCAGCATTTAAAATTACTTTGAATTAAATTTCATTCTT
TAAAGCTGATGTTTGAAATGACTGCCATTAGCTCGAATATATGCTCTAAGGCGGCGTCGCC
ATTCATTGATTGCACAATCAACAATTCCTTGATCAAAACGATTCCATTCCTTCACAACTCGCT
GTTTGAGATGGTCAACGTCGTGTATACGGGTCTGATAGACACGGTGTTGCAGAACGCTCCA
TATCCTGTAGTCGAGCGGATTGAGATCAGGGCTATTAGGAGGCCAAATAGTCGATGAAATA
AAATCTGGAGTTTTTCTTGAAAGCATATCCACAGTTTCTCTTGCTCGGTGAGCGGGAGCGC
TGTGTGGCTGGAATATGAAAAATTCACTGCCCGACAATTCCTTGATAGCAGGCAGTAAGTGT
TCACCGACGAGTAAAACCTTTTTGTACCGTAATGTTTTCTTGTCTTGGAATTCTAATCAAACTC
AAGTCATCTTTAGTGAACACCATTTTTTTTTATTATAATGGACGGCATAAATGAAACTGGGTCA
TTACGAGTTTACGACGTCATATTGTTTATAGGTAAGGTATATGCAGTTTGCAAAAATTGTAAATT
TTCCCATGTTGGGAAGTGCGAATTTCTGTTTGTCAAATTTGAATGCGCATAACTTTGGACTTT
GTCGATGGAATTTTATGAAATTTTTGGAAATCCTTGCAAATTTATCCCTCTTTCAATGTGTGAAA
TTATAGCGAATTATATATTTAGAAATGACAGATTTATCGCGAAAACAAAAAGGGTGTCAGTTCA
ATTGGGACACAGTGTATATGCTTTTCTGTCAGAATCCTGTTGTTAAATGCGACACTTAGGCG
CATTTCGTTAGGCTGTCGAGTAAGAACTAACTGAAACAACAGAGCACTGTATGACAGACGC
AGCCCATGTAGCAAAGGTTAGATATCTGAATCTAACCTGAGTTTATCTTGTCCATTCCATGTTA
TGAAATGACTGCTAGAATGTCATTATCGTCAGGTCTAATAAATCAATTTCTGTTCGCTTCCAGT
TTCATTTAATAACCTGACACTAGAGAAATCTTGGTTTATAAGCCTAGGCCTACATGTACCATG
AAACAAGGGAAAGACCTAGACAAAAGCTACTGAACTGGTTGACGACGATGGATTATGGAA
CACTACAGACAGAAGCCCAGCAACGAGAAGAGTGGCAACGTCAGAAGTTCGAACCTATC
CCCGGGCCGAGAACCTAAAAAGAAGTTTTATACTAAAGTCGGATAAAAGACGAAAGCATT
GATTGTTAAATAAGATTATAAGAAGCTGGGTGACGAATTATAAAAGTTGATAATTAACATGAAG
TGGGTTGCTGGTTGAAATGTTGATTTTGCCTGACAGAAGAAGTTGGGCTACATGTTAGAGTC
TCGAAGCCTAATGTACCGGATTGAAAAAATGCCAAAAATGCGAATTACGGTACCTCATTTTC
ATGACAATATGATTAAATTCAGCATTGTAACCATTCTCTGTACCGTAAAGACAAAGTTCAAGTT
CATTATAACTCATGCGACGGTCGCCTAAAAGTTCCATTTAATTGGTTGTAGTCGTAATCGCAG
GTGCAGTGTGAGCTACGGCTCAGTCAGTAGTGTTAGGGCTTCTTTACGGCGTCCACAAAA

CAAGAATGTCTCCTCCGGTTTAAATGTGTACATTAAAAAGCATTCTGTATGCAGTATGCCAAC
GAACATTCAATTGGCGCTGTTAGCCTCCATTGCTAGTCGTAGAAGATAATACAGCTGCTGTT
AGCTACGTGAATGTGTTGGTTGCTTGAACGCAGAGTGAATGAGTCGTCTGTTCAACAAGGC
GAACCAAGGATGAGCGTTGCCACCGATGCTGCAAGGCTCAGAAGGACGTCACGTTAAGT
TCCATCTTTTATATTTTCTTAGTAGGTGCTAAACCATTAGTTATGATTATGTTAGTTAATCAGCCG
CAAGAGAATCTGCTACCCATTCGTTGTCGCGATTGGTTTAGTTAACGGAAGCTATAGTTTGC
AGGCGGCGGCAGATCTCCTCTTCTAGGTGGCGGTTCTCGTATTTTAGGTGACCGATCTCTA
ATTTTAGGTGGCGGATCTCAAATCTAACGTGTCGAATCTGAGATCCGGCTAAATTTAACCCC
TGGTCGTACCATAATCTGATTCCAGTCAGTGTCCCCGTACTGGCCTAGCTTAATTGACAGGT
CCGAGGGGACCAGCTTTGACAGGTCCTTGTGGATCCGTCACGATAAAGACCATTTCTTAC
GTCTAGTCCCTTATCAGACTAATACACTTGAAGTGCAATTCAGTGAATTTAGTTTGGGTTTGA
GATTTCCAAACCACAATTCATGTTGTGGTTGACCGTCAACGTCAGATTCATTCACCAGTCTA
TGTAATTTGTATATATCACATGACACGACCTCCATCATCTTTTAGGTAACAATCTCGTAATCAT
GCCTTACATGGATTTCAGATGCTGGTGTGCAATTTCGTCAGGAAACTGGTTGGAGAAGGCA
GAAACTCCAGCTATATGCCATGTTCTACAAGCGAACAATCCATTCCATTCGAAACTATATACT
TACATTAGCCCAGATTATTACTCCAGTTCTGTGCACCATACTCGCCTGCTGCATGCTTTTGC
AAACGCCAGAAAACAAACCTTTTCCGTCTCTGACTCTTAACCTGACCCACTTCAAGGATCC
CATCACTCCGTACGACGTTCGACATTCAACTACAAGCGCCACACAATTGTCAACTTGTTAT
GCAACCAGCGTTTCAAAGCAGAGCAGACCAGTCTTCATTAGCAATGAGCTGCCTAACTCC
ACAATGGACGACTACTTGATTATGGTCGGCAAAGATCGCAGAGACCAGTACGATTGGAACT
ACCAGATCGGCGCTATCATTGAGGAAGTGCCAGATGGAAAAGTGAACATCATCGGTCTTTT
CAACAGTCACGCTTACCACACGATCGCCATCTCCTTGTCGTACGTAGGAAATACTCTGATG
GAATGTTTTGGCAATCCCGAGAAACAAATCGAAACAATCAATCACCCTCTTCCCTTGAACT
CTACTAAGCTACTGCAAGCCCTTCTACAAGAAGAAATAAGCGGATTTGGATTCTCCTACTG
GATATCTCTCGGAATGGCTTTCGTCTTCGCCACTTTCGTTGTGTTTCTGGTAAAAGAGAGAAT
GTCCGGAGCCAAGCACATCCAACTCGTAAGCGGAGTCAGTCTGCGTAATTTCTGGCTGAC
AACATTCATCTGGGATTACGTCATCTACCTCATTATCTGTATCTTGATCATCGTCGTCATACTG
TGCTTTCAATCCGACGGATACTGGCAGAAGTCTTGGTGGGTGCAACATGTCTAGGTCACAC
TAGGTGTAGCCTTCCAACGTTCGTTCAGTTTGATCACCATTAACAAAGGTGGACGGGAAAG
AGAGAAACACCGTTAAGAATATAAGAGACGAGTATAGTGGTGAGATCGGTGAAGGCCAGT
GATTAATCTAGACGTCTCTGGGGGGGGGGGAAGTTTCCCCCACCCAGGATTTGTGTTCCC
CCAGAAATTGAAAAATGTAGGACTTTAGTACTATCCACCATAGTAATTCCCCCTCCCCGAAA
TCTCGATTCCCCCAGACTTGGGGGACTAGATTAAAGCCTGGGTGAAGGCAGGATAGGAA
CATCATCGATCAATCAATCAATCAATCAATGTATTGTTTTCCATAGAATATTGTTAAAAAATAATT
TCACTCAAAAACATAGACAATTATAATTTTGCTCCGTGGAAACAAGAGGAAACCCGTAAAAA
TTGACCATAGTCTCGATGGATAGGGCTACCTACATGTATGCACTGTGCCAGCGATTAAAATG
ACTCAAGTCTGATTCAAAACCCTGAGTTCTCCATACTATGTCACAAATAATTAGCTTTCCCAG
ATAGTTGAAAGGCACCTATCCACCATCACCTGAATTCCGAAGCTGAAGTTCCTATATCATAC
TTTGAGACTCGACTAAGCTTAGACAAAAGCACGCATGCGTAGGCCTATATAGCCTATTCATA
AACTAGCTACCGATGGGCTATCATATGGCTAATGTAAATTAAGCATCGGTCTTATTATTCAGTT
AACACAAGAAAGAAGCAAAGATAACATCGTTTGAAATTCGGTAATGTTTATCACAAGAAAGC
ATTTCAGACGTTATCTTAATACAGCTCTACAATTCATATCGATTAATAGCAAAACAAGTAGGCC
TAGAGTTGTTCAAGTGATTTGAAGTAAACTTGAGTAGACTTAAACAGAAGAAAAAGAGAGGA
CTATGAAACAGTAGAAGAAGAATGAAGACAGCAAGAAGCACGGGAGAATACTATTAATAAT
GTAGCATCAAAAACGAAAGAGGGTAAAGTACCCTAAGAAAAATGAACAGAAGGTAGTGAG
AAGGATGGCAAATATAATTATGAGGGATACTAAGAGGACAGAATGTAATTTAACAGAAAGAA
AACGGCGTTGGGTGAAGGAAATTAGTTATTAGAGACTGTGATGCTGACCAATTCCATTTTCC

CCTTGCCGGATAACATATAAATCCTTTATCATCCTTCTCTTGCGATAAGTATTTGATTTGATATT
TTAGGTAATTAGACAATTTGTTTAAATGTCTGATTACTTAACCCTCGGGCTACCAAGGGGGG
GGGGGnnnnnnnnnTGATAGCCCTATGTTTTTTTGACATATTGAAATATTATTTGAAATATTATTT
TGACGTTTTAGATTGCTATAGCCAGATCCTTTCGCACAGTTTGAGGTATAGATCATGCTGATA
GCCATACCATACCAAAGTTATAGGCCTACCCGATACAGTCGAGCTATATAGGGCAGAATTC
TCAACACACAGTACAATGAGCAGTTGTCTGAAATGAACGGTGCCCTTGGTGTATCTTGCAC
AACTTCGGTATACATTCATGTATTCTTAAACGGGATACACCGTTCTGTGCAGAAATGAATGAT
GAATCGAACGGTGTATTACTAGATATGATATGTGATGCCAGACCCAAAACCCAGAGGGGTC
TCGTTCAAACCCCCCTTTGGTAGCCAGTGTCACAAAAAAGCTGGGTAGTGGGTATACATGTA
GCCCGAGGGTTAACTGGGTCACAGATGCTTTAGCAGTCAGTCACAGCAGCACTAAATCAC
TGTGCTTTATACCTTTTGTAGGGTTCATTGCGGCCTTAGTAATGCTTGTTCTACAAATACTGCT
AAAGCCGTCAACTGTGGAGATCGTGGAATGGATATTCTACGTATTCTTTCAAAATTACTGCTT
TATGGCGGTTCTTGAGAACATCTACAGAAACTTCAACAAACATAACGATTGTATCCAATTGG
CTCCACAAGTGAAACTCACCGATTTCTGCGCTTCTTTGAGAGGGCTTGGCCAAACCCATC
CCTGTTGTCCAGGTAAATGATTCATGTATAGAATGTTTCGGAACACCTATATCTTAGTTGATTA
CAAGATGCTCTTGGTTATTGCTACTGAATGTCTATAAATGTTGTAAGTGCACGATACGCATGT
GTGCATCAATGTAATATATGTTTAGTGTTGGTACATGGCATGCATGCACGAATGTGCACGAAT
ACGTGCTATAACTTTTTACTTTCTGGAAAATAATATTTCCTGTATCGTAAATCGATGCACTTTTG
CTTAAAAATATTGGAAATTTTATGAAAATGTCACCTCTCCTCCAATAAAAGTTTGATTCGGCAT
GTAGGCCCAACGCTACTGGCACTCACATAATGCTCCATTTAATTGACTTTCTTATAGTCATAG
GAACCGTATGGTTACATGTATGCGTGTGCTTGTTTGTGTTTGTGCGGCCATATGTGGTAAGCA
AGAAAACGCCCACAGCACACATTATGCTAATTAAACCAATGGCGTCACTTCCGCTTTATTA
CACTCAGTTACTTACGTTCTTGTATTCAAGAAGGCTACATGAAGCGGCTTATACTTAATTCAC
GTGAGAACTGTTGTTGTATTTAAGGCAACTGCGGCCCCGGCGGCTGCATAATCGCCGACA
ACTATCTGTCTTGGGCGTTGCCTGGGGTTGGGCGCCCACTGACCATCATGACTCTTCAAG
GCATCGTTTTCTTCCTCATTCTCTACGCCATCGAATCCGATTTCGTCGTGCGCCTACTCAAG
TTGAAGGCCCCGAACCGAGTGCAACAGACGATGCTGACGGAGATGAATGCCGACGCTG
CTGAACAGACGAGAGAAGACTCGGATGTGGCGAACGAGAGACAGCGCATCCAATCGAC
GCCGATAGAGGAACTCGTTAAGACGGATGCTGTCTTCACACAGGATCTAACGAAGCTCTA
CGGAAGGTTTGTGGCCGTTAACAAGTTGAGCATCGGAGTCTCTCAGATGGAATGCTTCGGT
CTTGTGGGCGTCAACGGAGCCGGAAAGACAACCATTTTTAGGATGCTGACCGGAGAAAG
CCGACCATCTTTCGGAAACGCCTATGTTGCAGGGATAAACGTCCAAGAAGATATTGCAAA
GGTGCATCAGAAGATCGGTTACTGTGCTCAGTTTGACTCCATCATCGACCAAATGACTGTTC
ATGAGACGCTCTGGATGTACGCATGCGCACACGGAATTCCTGCTGATGCCATGGAAATGG
TCATCGATCGCCTCATAGAACAGCTAACTCTGAAGCCATACACTAACACACGAGCCGGGG
AACTCAGGTGAATCAGATTATACCGCGTGAATCCCAGGAAGTTTGATTGTGGAGCGTTGTG
ATGTCGTGGTGATGTTCGGCCCTTGGGACCAAAGGGTTGCATGTTAGATTCCACCTCAAG
CCGCTGCGTAGGGACCTTGAGAAAATCCGTCACCTGCAATTGCTCACTGGCGCCATCAG
CGCGTCAACTCCGACTCAGCATCAGCGCTGTAGACTGGATCGCCTTTGAGTAGATTCCTT
AACCCTCGGGCTACCAAGCTTTTTTGTGATACTGACTACCAAAGGGGGGAGGGTTCAAAG
AACCCCAATGGATTTTGGGCCTGGCACCACATATCATATCGAATTATACAACATTCGATTCA
GCATTCATTTCTGCATAAAACGGTGTATTCCTTTTAGGAATACATGAATGTTTACCGAAGGTAT
GCAAGATATACGAAGAGCACCCGTCCAGTTCAGACAACAGCTAAATGCACTGTGTGTGAG
AAATTCTGATTGTATACCTCGACTATATCGGGTATAACTTTGGTATGGTATGGGCTATCAGCAT
GATTTATACCTCACAATGTGCGGAAAAGATCTAGATAAAGCTACCTAAAACGTCAAAATCATA
TTTCATATACAAAAGGCGCGAGATCCCAAAAATATAGGGGGTCGTTTCAACCCCCCCCCC
CCCTTCCTCTTGGTAGCCCGAGGGTTAAAGTGGCGCTACAAAAATTCTCGAATTTCATTGG

TGATGATGGTTTTCAATTGAAATAATTATGTGATAGAAGCAAGGACATGTATATAGACAATCAG
TCATGGTGACAATCGTATTATGTTAAACGCTGAAGGCTTCACAGGTCTACGTACGTACGTTC
CGCATATTTAGCTTTGAAACTGATTTACTTTTCATATCTTACCTTAATTGTTGTATCCGATGTTTG
TCATCGTTCCTCTTCCTTTGTTATCTTTCTTAAACCAGGTCGTTATAATTTATTATTTCATGCAAT
ATCTTGTTGGTAGCGGTGGCAACAAGCGTAAGTTGTGTGTGGCCGTTGCTTTGATTGGAAAT
CCGACGGTCGTCTTCCTCGATGAACCGACAACGGGCATCGATCCAGAAGCCAGAAGAC
ACGTCTGGAATACTCTGGCCGAAGCACGATACGCCGGCTTCACTATCGTACTCACGTCGC
ACAGGTACATCAACGTCCACCAGTACTGCTTAGTAAAGTGCAAACAATATAATCTAACATTC
GTGTCCTGCTTTTACCTACATTGTATACGGTGGTCTCATTGTGTGATATCTCTTCCGAAATCAC
ACAGACTTATGCGCCAGTGATTAATCTAGACGTCTCTGGGGGAAGGTCCCCCCAGGATTT
GTGTTTCCCCCCAGGAAAAATTTTTTAGGACTTTTTTACTATCCACCATAGTAATTCCCCCTC
AGAAACCTCGATTCGCCCCCCTCCCAACTTGGGGGACTAGATTACAGCCTGCAAGTTATG
CGCAAATCAGCTTCATCAGACTGGAGCAGTTTCCTGTGGTTATCCACCACTCAACGAGTGT
CTAATCAATGAAATGTCTAATTCAGTCCGTCCCGAAGCCCAACAGTGAATATCCATTAATGAT
GTAGGCCTATAATATAGGCCTACATCATTAATCACATCATATATTATATTCTATAATCTTTATAGA
CAGCATGACATGTCGTATTATGTTTGTACAGGTTAGAGTAAACACGTAAGAACTAGAACAGA
CAGATTATATTAAGTAAAATTAACAGATTACATTTACAAAAAAGACTACATGGACAAGCATATT
AGCAACCAAAACACATTATTATAAGTATTAAACAGTAGCAGAAGACAACTTACACACAAGTG
TAACATAGAGTAAGTCATATCATTTATACATAATGTGATCAATGTTTAAGGTTAACATGTAATTGA
GGGATTTGATTAATATGCTTATATAAAACCCGATTAATAAAATGAGCGTCATCTTCATTTGGGA
GGCAAAAATTGTGAGATATTGGTCGCAGGTTGTAGGAGAGGTGTCGTGTTGGAGGTATAAG
TTTATCTATCATATGATAAGAGGAGGAGCAGATAGTCTTAAATAAGCTATCATCAACTCGCTCA
GTTAGGATTTGTGGTGAGGGAAAAGTGAGTGAAAAATGCTCCTACATGTATCTCTCTCGCCA
CTGCCTTAAATAAGCACCGGAAAGTATGACCAGAAGCAACAGTTACTGACTGATCACAGC
GTCACACAAGTTTACTACAGTGTAAGCTTCTCCCATACTTTTCATCTCTCGGGGTGTGGGGT
GGGGGTCATCGAACTAAGCGCGCCTGTGATTTGGCCAGCAAAATGTGCTGTAAAATCCCT
AAATACAAGAGACAATCTCGTCCCCACCCCGTTTAGCGATAAGCCAGTTTTCCCCGCATG
TATCACCTATTGCTCATTGAAACCCTCAGCATTTATTCCAATGCCAAGATGTCTAGATGACCA
TAGGCTCATCTTTTATCAAATTGGACGGATCAATTATGCCTCAAGGATTTTGTATGATTATGTC
CTTGAATCCACAGCGCGAGGTTTATCTTTCAGTAAAGCAGACAAGTTTTGATCTATCATTGCT
TTATTTACTTTGAGAAACCAGAACGTACGAGATTATATGGTGAAAAATTTACTACTTTTACCGC
GGTATCTGGCTGCTTATAAAGTGTCTTTAAAATTTCTCATGAGCTGACAACTATGAGAAAATG
CATGAAACCAATGACAACATTGTACATGTGTTTTGTTTTGCAAGCATGGAAGAATGCGAAGC
CATTTGTACTCGGATGGGAATCCTCGTCAACGGTCGATTTCTCTGCCTCGGGACCCTACA
GCACCTCAAGGATAAGTTCAGCGATGGATACACGCTCATCGTCAAGCTCCAGCGGTTAGT
CACCACAGACAAGCTCGGGGAACTACAGGAACGGATCGAGAAACTGAAGACTTTCGTCA
AGCATACGTTCCCAGAGAGCGTCCTCAAGGACCATCACGAGAACCTTCTTACCTACCGG
ATTAAGACGCGGACTTTCAAGTGGTCTGAAATCTTTGGGATAATAGAGAGGGCAAAGGTGA
AGTACGAGATCGAGGACTATTCCGTGGGACAAACGACTCTGGAGCAGCTGTTCATCCAAT
TTGCCCGTCTGCAGCAAGGTCCGAGAGAAATCTCAAGAAAATATGCTCTGGACTCTGCTAA
AAAGCATATTACATAATTATGTATGTATCGCAATACAGTTAACCATGCCCTATTTCGCCATCCA
TTGCTGTAAAGTAGCACCGTGAACCTTAGATGCGTTAGGGCCTACTGTATAGCTGTACCGTA
GATAATCCATAATTTAATCACGTAAATGTTTGATTAATTATTATTTGTTTTGATTATTTTCCGAAATA
TTACTTTTACGTGATTCAAAACATATAGGATACATTCAAGCGACGTTTAGATCATTCTTAAGAC
TAAAGAGTATCTATAGAAATCAAGCTTCTTGTCAATCCGCTAAAGACATGCGCGATGCAAGA
ATTTCAATCGTTTGAACCCTATTGTCTTTAAAATGGGTTTTCGATCGCGTCAATTATACTGGAA
CAAAATTCATTACACTGCAGTATTACGCAGCTCCAAAATTGAGCCGTAATCCGTGGAATTGG

GTAGGTAAGCAAGTAAGCCATTCATTGACAAGGCACAAACTCCGCGACTGCGATGCGTG
CAGAATCGTATGAAAACACATCCAGAAATGACGCCAGTGTCAACAATTTATCCAAAAGAGT
CATTTGAGACAGATTGACATTATTCCTGGTATAGGCTACTTTTGGTAGCAACAGTGGCGGCG
CGTGCATTTTTCAAAGGGGGTGCAAGCCAACAATGTGCCGCCCCTCCCCCCCGCCCCC
GCTAGACGGCAGAACTGTATATTTACTGTATTTTACAAAATTATGACCGTTTTCTGTCTGATAG
TAATATCATTAGTATATCATTAGCAAAATTAGCCTAACACATGATGTATGCATATAGTGTCATTG
CGTACTGTGCAGTCATAGTATTGTTATAGCATTATAGCAAGTTACGTATTTGTAGAAAAACTTG
AAAGTTGAAAGAAATTTATATAAAAGAAAATTAAATTGAAGTTGAAGAAAGTATATCAGCTGAA
TCAGTTTTTTCTAATCTATCCGCAATTATTACGCAATTAGAACACACACGGAAATAAGCTTCA
GCAGTGACTGAATTGCCGGAGAATGGGTTCGGGGTTGACCCCCTCAGTTCACTGCTCAA
CACTCCTAGTTATTTCACAAAAATTACCTTGGGTGACAACAAAAGCATAGTGAAAATATTTCC
AACCCCCCAGTTAAAATTTGACAATTCAATCACTGAGCTTCAGCAATTTGTAGCCTCAAGAA
TCAAGCATAAAGGCATCAAAAATAAATTTAAAAATTTAGCAATTTCCACGAGTCTATTAACCC
TCGGGCTACCAAGGAGAAATCGAAACTACCACCCCGCCCCTGTGTTTTTCGGATATCGC
GCGTTTTGTATATGAAATACGATTTTGATGTTTTAAGTAGCTGTAGGGGAATCAATTCTGCACA
GTTTGAGGTTTGATAGCCCATACCATACCAAAGTTATACCCGATATAGTGGAGTTATATATCG
CTGCTATTATCGCAGACTCGCAGTGTCGCGCACGTTGGACTCTCAGCCTGCAACTGTCTTT
CTCTCAAGCTTCTCTACCTTTCTCCCTCTGGGTTTCGCCGGCGTCGTGTAAAAAGTCTCC
CCCATCAAAGAGTCTCTCCCAGCTGCAAACAAGTCTACGGGGCTCAAGTTTTTTGCCGCA
AATAAGCAACCCATTTTAGCATAAGTTGCCCCTTCACGCAAAATGTCACCCCACAGCAAAA
TTTAGTTCTAGCATACATGGAAAAGATTTGTAAACTCAAAAATGTAGGTATGTAATAGGTCTATA
ATTATATGTAAAAAATAAATAAATATTAATATATGTACAATTTAATTGCTGGCTGAATGGATATATC
CAGATAAATCAATGAATTATTGAACAAGTCAATCCATTCTTTCATTCATATAATTCATTTGTTACG
GGAAAACACTCGCCGGGGCGAATTTTTTACGTACAAAATAATTCGCCCTCCGGCGACTTA
TTTTTTTGGGGGTGGGGCGACTTTTTTCACGGCACACCGGCCCATGAAGACTGAAATTTTT
GCCAATGCCTATACCTGATCGGGTCGGAAGAACCCTGTTGAGTTTGCTTTTATAGGGGCTT
TATAAATGTAAGTTGCAATTACGAGTCTATTACAATTCTCACTTTTCGGTCATTATAATAGTCTG
AGTACAGCATGACATATAATGAATTACACTTATGCTGATATGAATAGTGATTTTATTCATGATAAT
TATCTTAGCTTCATTGAGCTTTATTGCAAAGGCTTAACACACCGGCAGTGAGTCCATGCATA
TGACTTACTCGCCGAAAAAATATCACACGGAGCCATATTGCCGCCCCCCTCGTGTGGCC
GCCCGGTGCGGCCGCACCCCTTTGCCCTCCTACGCGCCGCCACTGGGTAGCAATACG
GTCCAACAAAAAACTAAATGTATTCTTTGAATGCATCAAACACAACTGTGACTTACATGTACT
AAACTTGACTTATAGCCTATATAGAAACTAAACACTCCCATCATTAACGCTTGTTGACTCGTG
CCGTTCTTCAAAGACAAAAGACAAAGACTGGATCATGTAATGGGGCGTACAATGTACATGT
ACATGTATAGTAAGCTGATTCTTCCAATTCCTCCTCGTTCCTCCAACCTCTGCTGTGGCTTTG
CTTAGCAGCAAAACAAATAAAGGCAGCAGATGACGTCACCACTGATGAGTCCTAAACAGA
GTTTAAAGTGGAGTGGGAACTGTGGGAACGGCGTTCCCACTGCTCTGGAAATCCTTCTCTT
GCGTTCTCACTGCGCACATGCAACAGAACCATAAAATGCATGCAAAATGATGCAAATCAG
ACTTTACGTTCCCACTGCTCAACTTTTGCGTTCCCACTGCTCAACCAGACCACTTTAACCT
CTGGTCCTAAATGGTGTACAACGAGAAAAATAATTCAGGTACAATGGGGGAATCAAATCCAT
GAGCGTCTTATATATTCTATCTCGTCGTGACATAATTATATACACGGATATCTATGATGCGAAC
AGTCGTTTAGATTTCTAATATATAGTGGATTTTGGATGGTGTAAGAAAGCTCTCTTAGAATGTTT
ACTTAAAGCATAGTTGCAACAAGCGGTACAAAGGGAATGAAAAGGCAGAAGGCCTCGTTT
TTCAAAAATAATAAAAACGACCAAAATCGACAAAATGAAATTGTTTGACCTCCATGCATCTTG
TTGCGCATGTAGGCTCTGCATGACATTGTCATAAAGGTATATGCATGCCTGTATGCCCTCGC
ATGATAATTTGCACCATAAACAGTGACGTCACAATATACCAAGTCTCTAGTTCAAGAATGAAA
TAATATGGTAATAGATGTGACTTCACAGAGGAAACTCAAGTTTCATCCAAAAAATGTTTGCAG

CTGCTATTGTTATTTTTGTTGAACTGTCACGTTGACGTCACAATTGGCCAGCCTCTCTTCCCA
AAACGAAATAATATTAAATGGATATATATATGTATATATATATTATATATATATATATATATATCACGA
CCCAATTGCAGTGACGTCACAGCTAACGTAATCTTTATTTTCTAAATGAAATAATTATTGTAATT
GTATTTCAGTGCAATATGGTCACAGTTACGTCACAATAGGTAAATCGCAAGATGAACTAACA
CCGTAAGGTATTTGCATTACAATCACAGTGGTATGACAAGTAACGAGTCTCTATTCTCAGATG
AATTTCTATTGCAATACAACTGTTTGAATCAAAGTTACGATGTCGTCAAATATGGTCCGATTAG
CTATTAGCAGAATAAACCAATCTTGCCTTATACTTGCATTATAATCATCACAGTGACGTCACA
ATATAGCTTTTTCTGTTATCAAAATTAGCTTCTATACGTCACAATAGAATAGTCTCTTTTCTGAAA
GTGAACTCCCATTGCCGTGTATTTGTGTCATAATAACAGTGACGTCACAATGTGCCCATATCT
CTATTCTTAAAATGAACTTAATATGTGACAACATATATGTATCATTATCACAGTGACGTCACAAC
AAACCATGTCTCTGTTCTAAAACTGAACTCATACTGCATTGTATTTATATAATTCTCACAGTATT
ATCACAGTATTCTTATTCTCAGTTCTCAAAGTGAACTGCAATCATTTGCAAAAGACAGAATTAA
CCCCATTCTCATTCGCTTAACTATTATTGTATCACAGTATTCCTAGTCTCAGTTCTCAAAATAA
GCTATAAATTCTATCTCGTCGTGACATAATTACACGGATATCTATAATGCGAACAGTCGTTTAG
ATGTCTAATATACATGTATACTGGATTTTGGATGGTCTAAGAAAGCTCTCTTATGGCCATGACG
GAATCAAGTGACAATGCACGGACGTATGGAAAACATTCGTATGCCTTTATCACGTGATCAC
GTATTTAATTTAGTGTTTATGTAAAATGTAGGCCCTACTGGTACTCACCGTCTGCGATGGGTA
CCCTTTCCATTCTGCCCAATTGGCAACAGAGGAAATACAAGCGCAATACTGAGCGCTCAT
TAAAAGTGTCCTACAATCAATCAATCAATCAATCAACATAAATTTATATAGCGCCCCTTCAAG
ATCTCTACTCAGAAGCGCTCAAATGTATATGATTCGCCTATGGTATTGCTAAAGGTTTTCTCAT
TCATTCTTTCTGGACTTTTATATAGCGCCCTTCACGAAACTACTCAGATTATATGGTTAGCTTG
GTACTGTTGTCTGACATTTACCAAGGAAAGTCGTAGGCCTACTGGCGATATGTTTGTTTCTG
CAAAAAATCAGCCTATTGAATATTTGACCTATAACGAGCAGAATTAGTCAGTGTTCGACATCA
CAGTTGAAGTGGTCAATTAAACTAAAGTGGCATATGTTAACCCTCGGGCTACCAAGGGGG
GGGTTGAAACGACCCCCCTCTAGGTTTTTGGGATCTCGCGCCTTTTGTGTATGAAATATGATT
TTGACGTTTTAGTTAGCTATAGCTAGATCTTATCCGCACAGTTTGAGGTATATATCATGCTGATA
GCCCATACCATACAAAAGTTATACCCGATATAGTCGAGATACATTATACAATCAGAATTTCTC
ACACACAGTACAATCAGCAGCTGACTGAACTGGACGGGTGCCCTTGGTATATCTTGCATAA
CTCCGGTATACATTTATGTATTTCTAAACAGAATACACCTTCTTGTGCAGAAATGAATGTTGAA
TCGAACGATGTATTACTTGATATGATATGTGGTGCCAGACCCAAGATCCAAGGGGGTCTTTC
GAACCTAGCCCCCCCTTGGAAGTCAATGTCACAACACAGCTTGGTAGCCCGAGGGTTAAA
CAACGCGAACAGTTTGTGTATTTTCTACTTTTATGACTAACAAGTGAGGAATGCATTAAAATG
GTAGACTTACTGTTGGCGCATACATGTACATTGCACATACCACCATATGAAGCAGGCTAAA
GGCATAGACCGTAATGTTCTAGCAAAGGACTTCTATTATTTCCGGTTTCCTATATTATGAAGTC
GCATGACCGTCATATGAACGATCTCCTGTGATCGATCGACGTTACACCGCTGGACATATTG
TGCTAACCCTGGAATTTGGCTTGCGGTTTCAATTAATGTAGCCATCCACTGATGTCTGAGTAT
AATTCTCTGCACCAGCAGCACTTTTGATGTATTGGATAAGACTTTGTTTTTACCGACTCAATAT
ACATGTAGTACATACTTACATACCTACTTATTTAGGCCCTATTGTGAACGCCACTGAAAATGTT
CCGTTGAATATAGGGTAGGTTAGGCCGTATGCAGAGGGGCATGGTTAGGCCAAGGCCAG
TTAAGAACAAGCAGCAATAAAACAGTGGTTATGAGATCGAGTAAGCTAAGTAATTGCATTTG
CTGATGGCGTAAGTCCCAAGCATATTATAGATTCAAGAAAGCTATGACGTCATCAACAATAC
GTGGAATAAGCCTGTACATCACACACTATAATCGGCATTTGACAGGGATTAATCGCCTTTCA
CTTTAAATTAGCTGATGTTTAGTAGAGAAATATATTTTATCTCGTCGTGACATAATTTCAGCGCA
TTCTTAACAGAAGTTACACGGATATCTGTCATGCGAACATTTGTTTAGACTCCGAATAGATGTT
GGATTTGGGATAGTCTTAAGAACGTTCTCCTATGGCCATGGCCGAACCAAGTGAAAATGAA
CGGAAATATGAAAAACATTGGTATGCCTTTATGACTTGATCATGTATTTAATTTAGTGTTTATTTA
AAATTTAGGGCCTACTGTTACTCACCGTCTGCGACGGGTAGGCCTACCCTTTCCATTCTCC

CCAATAGGTCTACAAGCGCAACCGTCCTACTGAGGGGGTCTACCCGAATTGCCGAAACC
CGAATCGCCGAAAGGGTAGAAACTGTCAAATTGCCCGTATTGCCGAAAGGAAAATTCTGC
CAAGCGCCCGATTCGCCGAAAAGTAATTTCGATCCAAATCGAAACGACCAGCGATGAAA
ATGTTGATTGTGAAATACGAAGTCGGAATATACGTTTTAAATTATATAGTTGGCTAGTAAATACA
GAATAAATAGCGCTAAACTGGTAAAATTACCCGACCAGCCTTTTCGGCAATGCGGTCGCTA
TCTATAAAATCATTTCGGCAAAACGGCCCTTCTACCCTTTCGGCGATTCGGGTTTCGGCAA
TTCGGTACGGCACCCTACTGAGCGCTCATTAAGTGTCCTAGAATGTTGATGATTAGCCTATA
GTATTGCTAAAGGTTTTCTCATTCATTCATTCATGTCCAGTCGAGTACAAGAAAAAAGATTTAA
TAGATCTGTTGAAGCCAGCAGTTGACCAGCAGTCTATGTAAAATGTAAAAGGTAAGCCCTA
CTGTTACTCACCGTCAGCGACGGGTATGCTTTCCATTCTGCGCAATAAGCGAAGGTCATGT
GGCAGACATGCTTCCACGTTATCAACTATTTCACTATCAAAATACTGTCTTCAAACTTGGCA
GTTCAATTTTCTCTCAAGCGCAATGTGTCTTCTAATGGGCTGCCGATTCCAACCTTTCGCCG
ACCATCTCATAAACGATAGAGATAGTCGCACATACATGTACCGACATGAGAGAGGCACGC
GCAAGTGCATTTGCAGTGACAGAACAAAATTACAAAAAGAGAGAAAACTAATAGAGAGCAG
AGAGAGATGTATCCCAGTAGACATATTTGACATCATTGTATCAATAAGTCTGTCGACGAATCG
ATGATTCAGCCAATCGGGAAATCCGATCAAGGCAACGTGTCCCCTTTCGGTTGCAGAGGC
AGACCTAGTCTCTTCGAAAGGGCCTTCCATTGCCGGTGTCACAAAATAAAGTCTTCGCCG
AAATATATCCTTCCCTTTACAATCAAGGATAGGCGCGCTTACATTTTACCGTTTTGACAAGCT
AAAACCAGTGTAACCATGACTCCATGAGAATAACATTTCTCAAGTATTTCAAAAGAGAAGTC
AGAGCAGAGTTGGCTGTGAACATTATGCGTTCACATGCCTGCCGATCTCACGCTGAAATAT
TAGTCTTCCTCGGGAAGAGTTCACTTCGGCGGGGATGAAATATTCTCTCTCCCACGGATAC
GATTTTAGTCTGAAACAGTTTCGCCTGAGGAGAATCTACATGTATTTCGAAATAGAATAGTCT
CCGGGGTAGACTTTTAGGAGGAGACTCTGTCACGCGACAGCGGCAGACCAAATGATTGA
ACTGCATAGTCTCACTTCCACGCGATCATTATATAGGACCATACATGACATCATAGATGACCT
TAGCTCTAAGCGGTGGTGATGACGTGGGCGTCTGAATCATTCATTCATTCTGAGAATTTGTAT
AGCGCTTCCGAAGAAATCTACTCTGAGACCCTCCGGACTACAGCGCTGATGCAGAGTCG
GAGTTACCACGCCACTGGCGCCTTGTGAGCAATTGCAGGTGAAGGATTTGCACAAGGTTC
CTACGCAGCAGCTGCGGTGGATTAGAACATGCGACCTATCGGTCACAAGGCTGCGACTC
TCACCACTACACCACCGCGCCCAACAAATGATGTTTGTGGAGCGCGGTGCTGATGTTTCC
AGATAACTTAATATGTTGACGCCGTCAAAGTGTCAAGTGCTGCAACAGATAAATCCTGATATC
TACGTATTGTAGAAGAACTGGTTTCTCCTGTCGTGGGAGGTCATTGTGACCATATTTATAGTT
GTATTTCAGATATACTTGATATTCTGCCGATACTTTTCGGTTGAATGTAACATTCGAATATATAC
AGGTTATTGTCCTAAATCTGTTCATAACGCTAATGAACACGGATTCTAACTTTGCAGAACCAC
ATAAAAACTAGCATCACCCATTACTATCGTAGCGTGATGCTGGTTCGAGGAGCCTCAGTTTT
ACGTCTTCTTATTTTCCTGTGGAACGGACAAAAGAGCTTAGGCCTACCAATCGGTAACTTTA
ATGATACGGCGCTAAGTTTGACTTCCAGCCACGACTGTTGCGTGGCGCTATGTAATTAATTT
CATATGGCAGCACATTAAATATTATGATATCACATTTCGTAATATCACAAGCAGGCATACAAC
TGTTTTGAGTAACTGCAGTTGATATGGTAGTATAAAGGAGGTTGAGTTATGATAATGATTGTGG
TGAATGATTTACAGGAGCTGGATACAGTTGTGGGTGCTGCTCTGGAAGAACTGGTTGCTCC
AGAAAGAGAAGCTCTGTCATACGATTATGATTCTTATTGTATTGCCAATCTGCTTCATCATAAC
AATGGCATCGTTGGAAACACCGGGCAACCCTAAATTCGTATCACAACCAACAACCTACGA
CAGCTTCCCCGTTACCCCAAACTTGACCGACAACCTGAAATTTCCACCAGTAAATTTAATC
CTCGTCTACTTTCCAAACACGGAACTCGTTACGAGGATTGTGAACAGAACAAGCAACATCA
TGAGCGTCAAAAATCAAACAAGTACGTCCATTATATATATATATATATATATATATATATATATATAT
ATATATATTATATATCTAATGTGTATATATATTGAATTATATATATATATATTGAATTATATATATATATAT
TGAATTATATATATATATATATATATATATATATATAGCATAGGACGAGCATAGGACTTATATATATATA
TATATATATATATATATATATATATATATATATATATAATGTATATATATTGAATTATTATGTTCGTTATGT

TCCTATTATACTTCATATAATATATATATATTTCATTTCATTCATTTCATTTCATTTTTCTTTTATTTCA
GTAACCACAACAAACAGAATCGACAGAAACAAATATAAGTACAAACAAGTGGTTACCCAGG
AGACCCAAAAGGGGCCAATGCTCCCGCAAGCCAGGGCCACCTGGGTGATGTCTTGTGC
GTAGAATAGGGATTTACATTTTTATTTATATAGATTATTATAGATGACAACATGGAAAATGGCTC
CTGTCCTGTGTTTATAAAAAAAAACTGAATTATGTTTGTTATGTTCCTATTATACTTCATATTGTTG
TGAATAAGCTGATGTTTCTGGCTTATGTGCATTGTGCAACGTTGCGAATATTGAATAATTAGTT
GTAACTTTTAGTAAGCCTACGTCGTGTCATTGCAATAAGGTATCAATACGCATCAAGGATGC
CTTCTTTTCTATTCCACGTTCGTTGAAGCCAATGACTAACTGCGACTTATGTATGTCCAACCT
AAAATGCAGAAATGCGATGCAGAAAGTGGGCTCTAAGGTCACACTGATAGTTCGTGCCAA
GACTAGCCACACTGTCACACATTTATAGGAGCTCGTCACTGTTCTGTGATACTTCTGAAGATT
GCCTTCATATAAAGTCTAATTAGACTAAATGCAAGTTGACTTCAGTTATCTATATTTGTGTAGAT
CTTGGTTTTGCATCCGAGACAGCTGTGGAGGACTACGTGGCGATGCATGAAAATGTTTTTG
GGGCAATAATTTTCATCGGTTTCGAAGGCATCGTCAGTCTTCCGAAGAACATCAACTACAA
GGTCCGTCTGCGTTCAGACGAAGTTGGGTGGAGAACAAAGGACACGTTCTACGAGCAAA
TTAACATCACGCCGAGAGATAGGAAAGAGCGATTTGGTGGGGAGAAGCCAGGTAAGCCC
TTTCTCTATTTCAAGATCAACCGATCGTAACTTAGTTTTTTTAATACATGTAAAGAAGAAACACA
AACTCCTCCGGAGAAGACCAGAGGATAAATCAGCGAAGTAGGCTAGTAATATACATGTAGC
CTAAGTGGCAGATGCAATGGTAAACGCATAGGACCTTACTCTGATTGTGGTAAGACTCAGT
GATTAATCTAGACGTCTCCCGGGGGGGGGAATGTTCCCCTTCCAGGATTTGTGTTTCTCCC
CAGAAATCAGAAAAATCTGACTTTTATTACTATTCACCATAGTAATTCCCCTCCCAGAAACCT
CGATTCCCCTCCCAGACTTGGAGGACTAGATTACACACTGGGTAAGACTAAACTAAGCAG
CAAGAGTAGTCATAAGAATTAAATTAATGTAGTCCTTCGGGGATGACGATAATTCATTCACTC
ATTGATTCATTCATGTCGAGCATTTGAATAGCGCCTCTTCAAGAAACCTTCTCAGAGGTGCC
CCGGCCCGGCCACGGTCAAACAAGTAGGTTTTAAGTATCCTTTAAAACGTGGATGAAATG
CTCTGAGGAAGTAGTTTCAAATGCATCCGAAGTCCATTCTAGAACGCGAGCGGAGGACAC
ACAGCTCAACCTTGGCCTAGGAACGGAGAGCTCGACGGCCCACAACTTGTGAAACTGC
AATGGAAAGATCGGGTATAGGTAATGTAATGTAATGTAAATCCTCTAAATTTCTCAGCGCCAC
ACGAAAGCTGCGTCAGGGAGACCAGCTTATTCACCGGCGCTAAAAGGTATGAGGGGGC
GTTGCCAAGACGGCATTGTCAGACAAGCGCAGCCACCCGATATTGAAGTGATAGTTCATA
CGTTGCAGGGTATTACAAGGAGGGCTTCCTGGCTATCCAGCATGCCATCGATCTGAGTCT
AATCGCCGAAGTCAACAGGAGTTTCGACCCAACGGAGACGACGAACGTAACGACTCAG
CTGAAGCGATATCCATATCCACCATACTACTATGTTGTATTCAATTATAGGATCAGATTCGCA
GTGCCTCTCTACGTTTTCCTCTGTAACTTATGCGGCGCTTCATTATGCTATCATAATATGGTG
CTGGAGAAGGAAAGCAAGATCAAGGTACAGTAACTGAACTTCAGGCTGTAATCTAGTCCC
CCAAGTCTGGGGGGAATCGAGGTTTCTGGGGGGGAATTACTATAGTGGATAGTAAAAAAGT
GTTAAAAAATTTTTTTTCTGGGGGGAAACACAAATCCTGGGGGGAACTTTCCCCCCCCCC
CAGAGACGTCTAGATTAATCACTGACTGAACTTGCAATTTGAGCCACAAGTAAATTATGGTA
GGCCTATGCGTATACGATGTATATATCTATAGCCAATAATTTGTTTCGATCTAAAACAGAGGTT
ATGGACGATGTAAACCGTGGAAAAGATGGGTTATAGGCCTACTGAACGAGAGACGTGCAA
ATGCCATGATAGGAATTTAAAGCGAGTTGTTATTGTAGACTGAAATGGCTGAATTTTGACAGG
TACGCATTTCGTGATCTGCAAGCGAGGATCGCAGCAGCTAAAATCACATAAGGACCTGCT
GCCAAATGCTCCAGTAACGAGCAGCTGTTAAGCAAAGTATTTCCATTAATAGGCCTACATTC
ATGTCAATGTGTGACATCACGTGTCATCATGTAACACGTATACGAACCTCAGAATGAATGGC
TCCGGTGGAAACAATCGCCTCGTCAGTATTTACATGAACTTATTGACTTCGATCAATTTTTTG
CACAGGTCGCACTGTTCCATTTGGTTACATCGCTAGGAATTAACTGCATAAAATTATTTAAAG
TCTACTGGCATATCAACTACAAATTGCATGTAGCATGTATACGGACTTCAGGTTCATATGATTT
GTGATCGGTACATGTATATCCAGGGTTTAAATTGGTGTGGGAACTGTGGGAACGGCGTTCC

CACTGCTCTAGAAATCCTTGGCTTGCGTTCCCATTGTTCACATGCAACGGAACCATAAAAT
GCATGCAAACTGATGCAAATCATAATTTGCGTTCCCACTGCTCAACTTTTGCGTTTCCACTG
CTCAAACAGACCACTTCAACCTCTGGGTATATCTATAGGTATATAGAATTGTTCAACCCGCC
GCCCCATTTAAGCTTTGACAATTCAATTGCAGTGTTATAACTATTAATGTTCAGATGCACTTAA
GTGAGTTTGTGAACACCGTACAGGAGACGATGAAGATGATGGGGTTGCGTACGTCGGTCT
ACTGGGCTTCGTGGTTCCTCAGTTGCCTGCCGATCGCACTCTTACATGTCATCATTTTTGTC
ATTGTTCTCCACGTAGGCCTACCACTCGCTTCCAAAGGTCCATTTTTTGCCCACAGTTCGG
TGGTCGTCACTTTCGTCTTTCTCCTGCTCTACTCTCTATCCCATATCACCCTCAGCTTCGCC
GTTTCTACTTTTATTTTCAAAGGTAAGGATGTATTCTTTTTACAGGCAGGTGTGTCGCGATGTA
GAGTGTCCCCTAAAATGATTTCCCCCTAACGAAATAAGTTATCTGGGAGAAAGAATTCACAC
GCAAAGAAGACAGAAGTGTCCCAACTTGTGCAAATCTGTGGGATAGTTTCACAAGATGGGA
ATTTAAAAGTTTGTCCTCCTGATATTCTATACCGGTATATTTTATTCCAGAGAATATATTCATTTA
GCCCACATCCAGTAGTTGGAAAAAATAGGCTTAATTTGTTCAATGCGACATTAATGACTAATC
AGATAATTATCACCTGGCATTATTTTAATTTAGACAATCGGTAGTATTCATTCAGAAAACTGATT
TCAGGACAATTGGCCATAGGTCGATTTTTTTTCATGGTAAATCCTTATTAATATAATATTATTAG
GACTATAAATATTATTAGGACAACTACATGTCCAGTTTTAAACCTCATCAACAATATACACTTC
TTCATTAAAACTTGTCATTTTTTATACCTCTTTGTCCATGATATTTGGTCTGATGACCTGTTGTCC
GTAACACGTCCATGACATTTTGTAAAACCACCTTTTTCCTTTTCACACAATCTGGGTTTTAAAT
GAGAATTTCTCTAATTCCTTGGGGTAAACTCATGAGTTTCTAGAAGATTTCAAAAGTTCCTTCT
CCACAGGTCTGTAAGAGCAGTAAACTTTCAGAGATGCGTTCAATAAATATTCCTCAACCCCT
CAGCATCGGTTTATTTTGTTTAAATGTCATTATGCCTTTGTATTTCTTGACAGCAAACTCTTCC
GGCGTAATCACGATACTAGTCTTACTTTTGTACGTTCCTTATTTCTATCTGGAGAAGATCTATG
ACGATATGACTTTCGCGCAGAAGTTAGCAGCATCGTTCGACTTCAACTTGGCCATGGGATT
CGGATGCATCCAGATATATCAGAGTGAAGCACTCGGTACGTCACTTATGACGATTTAGTGCT
GATTATTATGATCGCGAAATCGATGCGTCGACAATCTCTCTTCTCTCTCATTTCCTCTCACTC
ACTCACTCACTCACTCACTCACTCACTCACTCACTCACTCACTCACTCACTCACTCACTCA
CTCACTCACTCACTCACTCACTCACTCACTCACTCACTCACTCACTCACTCACTCACTCAC
TCACTCACTCACTCACTCTTTGTCTCTCTCTCTGTCTCATGCTCTCTCTCTGTCTATCTCTATG
TCTCTGTCTCTGTCTCTGTCTTTCTGTTTGTCTGCCTGTCTGTCTCTCTCTCGCTCTCTGAGTG
AGAGCGTGAATGAATGATAGCGAGCGATTCACTTGATGATTACAACCGTAGGCCTACACTT
GATCCTGATTAAAATGTGGCTGGTTGAATGTGACCAGGAATTGGTGTACAGTGGAGCAACC
TTTGGTCTTCACCATATGCAAATGATGATTTTACGATGGGTCACGTTATGATCATGCTTGTGGT
CGATACAATTATTTACTCGGTCCTTACTTGGTATTTGGATGCTGTTATACCCGGCGACATCGG
AGCTCCTCAACCGCTCAATTTTCTCTTCACGGTTAGTGGATTCGTGCATTTCTAAAGACAGA
GGTCGATAGTCCGCTATAGCCCTGAATACTGTATAACTTTCGTCATTGAGGTCAGATCTATCT
CTACGGTTCATAGAGTGACGCTAGAACATGAGGTATTTATGAGAAAATAACAATCTGCCGTA
ATAATAATCGGGTTGCGGATAGAAGTATAACGAATAGAGCATCAATAGCGTGTGAGTTGTAG
GCTATAATGACGAGTTCTTTGCCTGAAAGGCAGGCCCATTTTGCTTTTGCTACATGTACACG
TTCCTATGATAGATGAATTAGGTGGTTTCCCCATTGCTTTCCTCATAGCATTAATATGGATGAA
CAACTTGTCTAAGTCCGTCACGCAGCGGTCTATAGGTGGATTTGAACCCACAACGGGCAC
GACCATCCGGTCTCAAGGAGTCGAACACCACCACTAGGCTTACACCACAGCGTTCCACC
GCTAGCCTATATATACAAAAGCCTACAGTAATAGGGAGTGATCAATTAAGGGACAAAGAATT
AGAGAAATAATGGAGACAGGATGGAGAGGATTTAATTTGAATGGATGGAAACCGGAGGGA
GACAGAGAAAATATTGAGACTCACTGTCAGGAATTTGGTAAAAAAAACTTTGACCAAACTAC
ATGATCTACATGTAGTGTAGACCTAATGACGTTATAATAGGCCTACTAGTATTAATTCAATTTTA
CTTAGAGATGTTTGCTGTGCCAGTTCCTATTTTCGGGACTCCTTTGTGACATTCTATCGTTGA
ATGTCATATTCTAGGTGTATTTTCTTAACTCCTATAGCTCCAAAGCCTACTTGCGTGTGCCGG

ATGTTCATTTACAGAAGGAGTACTGGTTGGGTAGAAAAGGAAATATGATGAGAATATCGGAC
GAAGACTTCATGACGCTGATTCCGGATGATAAAAAACCGTACTTTGAAACTCCATTACAGGA
GGCTATAGCTGGGATTCAGATCAGAGGACTGACTAAGGTCTGACCGCCAATCATTCAACAT
TTGTCTCTTACAATTTCACAAAACTTTTCCGTTTTACAGAGAGCGTGCGTGTAGTGTCAAGCA
ACATCCTTGAGCAGAATAAGGCATTTCAAAACAGTGTTAGCCTAGGACTTACACTGATTATA
CAGGTATGTTTTAAGGAAATTACTTCGTTGGCAGTTGACCGATGATACATGTAGATGTAATGTA
ATGTAATGTATACCCTCTAAATTTCTCATCGCCACAGGAAAGCTGCGTCAGAGAGACCAGC
TTATTCACCGGCGCTATATCAAATCAAGTCCGCATTAGAAAGGGTCAGAATCCGGGATGTC
AAGCCGAAGGGACAACAAGATGCTAAGATGGCAACCGATGATGATGGTGATTGGATGATG
ACACTGGAGAAGAATCACGATATAACAGTTTGAAGAGTTCCGTTTTCAGTCGTGAGTGGAAA
GCGACAGAGGATATAGAGCGAGCGGTTGGAGAGGTGGAAAAGACAGGCGAAATTCTAGG
GTGTGCGAACTGTCGGAGATTTTTCGAGAGTTTATTTCAAAGAGCTGCAGCAGCGTAGACT
ATAAGGAGCGGTTGCAGAATTTCAGAGAAGAGGAGAATGGAGGCAGTGAAAGGCGAGAT
GAAGAGCGTGTAGACCTGAGCGGCTGAATGGTGATAAGCTGACGGAAGAGAGATCTGTAG
AGCGCTGTATGTAAGAGATACAATCTTGTAGCGAATGCGTTGAGGACTTTCAGCCAATGAA
GTAATTGCAGAACAAGAGTGTTGTGATGATGCTTGGGAGTTTTCTTGACTGCACAGACGAGA
GCGTTCTGGATTGTCTGTAGGCGTTTCATCTGAGTTGAGTCAATGTTGTGGAAAGGTTAGTTT
CAATAATCGAGTTTGGAATGAACAATCGATGTGGCTATTTTGCACGCGGTTTTGAAGTCAAAT
ATTGGGCGCAGTTGACGTAAGTCACGAATGTGCATAGGCCTATAACAGGTCCGAGATAGC
TGGGTGAGTTTTTTTATAAAAGATGATTCGAAGGTTGCGGACAGGAGAGGAATGAGGCGC
ATTCGAAGTTAGAGCTGTTGAAGAACGGTCGGATGAATATGTTAATAATTATGTTTAATTCCTC
ATTTAGGCGGTGGATGAGAAATCCTGCTTTTGAAGGGTTTAGTAGATTTGCTGTCATCCACG
CTGATACATGTATATGGTCAGGTTGCATGTGTTTGGAAAAGCGTGAGAAGAAAAATATGAAC
ATTTGGGTATCGCCAGCAAAGAGATGATGAACGACAGAAGATGAGGAAATGAGTTTGTTGA
GCGGGGAAGTGTGGAATAGGAACAAAAGAGGATCAAGAACAGAGCCCTGAGAAACACC
GCAGGTGAGTGTGTGCTGATTTAAGTGAGTGACCAGCGGCATTAACAGTGAAAGAACGTAA
GGAAAGGTACATGTATGACTTTATCCACGAAAGGGTATTGCCAGAGATTCCAAATGCAGTA
GAAAGGCGTTTGATGGGAATCCCGTGGTCAATTATTGTATGATAAGCGGCGCAGATATCCA
GGAGACACAGGCACGAGACTTTTCCACGACCAATCGCTAGGTCAAGATTGTTGTATAGTGA
AGTGAGGACTGTTTCAGTAGAGTAGTATTTAGTGTAGCTAGATTGATTTGGATTCAGAAGTTTG
TTCGAAATTAGATAGCTGTTTAGACGAGAGAGGACAACCCGCTCAGTGAGTTTCCCTAAGA
ATGAGAGATTTATTTGACTTTGGACGGTAGTTTGAGAAGAGTTTCTTGTCCAGATATGGTTTTT
CAGAGGAGATTCCACTATATCGAGACAGGTTGATATTTGTTATTGTAGGAGTGAGAAACATGT
TTAAAGATGGAGGCGGGACAGTAGCCAACGAAGCGGGAGATCTGGCGGATTAGGGTCAA
TGTTCTTGAAGGGGTCAGACTTCACTTTGGAGTTCAATGCGTGGGTGATTAGTCTGAAGACG
GTGAGAAAGAAACGGAATTGAGAACCGGACACGGAATAATGAAGAGATAACAATTGAGGT
GTCGAAAGTGGCGTTTTCCCTGAATACTCTTTTAGCCAAGCAAGAGGATTGTTATCCAACGT
TGACTGTTGGTATTATCTCAAGGATACTTCAATCGATTTTCAGGATGTGCTGAACAATTAAAAT
CGGTAATGCATTGTGCGCTTGTACATTGACACAATAACCTCCCAATGTTGGTTATTCACAAA
GCCGACAGGCACTCGATCGCCGTCTGTTATTTGACGCGTTGAAGAGCCACAAATAACTCT
AACAGTCGCTGTCACCGGGGAATGACCTGAATTTCTTCTCCTACAAAAACTAATCGACTGC
GAATCTATTACATCGGACAAATGATACGAAATGCAATTGGAAAGCAGGAAGCTCAACTCCC
CCGACATTTGTACCCAGATAATTTGAGACGCAGAAACGAGAAACACTTAACTCAAACGCAA
GAGCTAATTAGCCAACTTCTGTATTGCGAAAAATACGCTAGTGTTTTTCAGTGGACTTAAATAT
GACCCACTAACGCTGTGGATTCTTTTAGTAAGATTTATTTTTTGGGACAATACAATAAAATTAA
GTGGATGAAAAGGGCGATTTAGTTGCAGTTCAATGGTTTCAAATTTTTTCCATTAGAAATTCC
GCTCCAATGCGAAGCCAGCAGTTGACAACCTTAACATTGACATCTACGAAGGCAATATAAC

AGTACTACTGGGACATAATGGAGCTGGTAAAACAACGGTCATGTTCATGTTAACCGGTTAGT
AACATTACCCATGCATGACTAATTAATTTCATAAACATACAGGTAGGGGCCTACCTCTCCTC
CTCGAACTGATTTCGTAGTTGTTTTATTTATACTGTTTACTTTAGTATTTCTAATTTTAGTATTTTCT
CTTCCTAATTCATTCTTTCTTTCGTTCGTTCCTTCTTTTCCTTTGTTAATTCAGTTCTCCATGTTTT
CCAACTTGAATTCAAATTTTCTCATTATTTTTTATATTTCTACGCAGTCGTCAATAATGTTATCTC
GACCATCGTCATCGCCTGATTCGCATTACCATCGATATAGGCCTAGGTTTAAACGTTTTGTA
ATGATAGGAATAACCTGATAGTAATAATCATGGCGTACCACTTTATTTTTCTATCTCGCGTGTC
TTAGCCACTTCGTAATAGGCTAGTAGTCATTAATGGTGTAATGCCTTATTTTTCTTTCTCACGT
GTCTTAGTCACTGCAATCCGCAAGAACTAGATCTCGCATTTTGTAAAATCACTGTAATAACTG
GTCTAAAGAATAATTTACTTGACGTAAACTTCGCGATTTCTGAGGTCCAATGTCTAGCCTGAT
CTGTTGGTATTACATGTGTTGCAGGGTTTCTACCTCCGTCGAAAGGAACAGCCTTCATAAAT
GGCTTCGACATAAGACGAGGAATGGATGATATCCGACAAAACCTTGGCATATGTCCCCAG
CATAACGTACTTTTTGACGCCCTAACCGTCGAAGAACATCTCATCCTCTTTGCCATTGTTAG
TACTATTGTCAAAGTAGGGGAGACCGGGGCATTACGGGATACTTAAGCATATTTTGTCTTTGT
CCCACAAGTGGTAATGTGCTCAACGATGGCCAGTTTGCACACGTGTAGTACTCAACCTGC
TCTATGGAAACCACATAGCATTCAGCTTTGCAATTGACAAGAATGAACAGAAATTTGTTATAA
ACAATCATCATGAATATCCCTATGCCCGTACTATGGGGTACAGTGGGACATACCTTCTTCTTT
ATTGGCGAGCACAGAATTTGACTTGGGGTTCTCTATGAGGCACGCGTGTCGATTTTCCATA
GAAGGACGATGCTTGAATGGTGCTGTTATCTGTCAGTGCCATGGTCACAGCAAATGGTGTT
GCAAACAACATAGTACCAACACTTATCCTTCTCCCGTTATGCCCCAAGCTGTCCTACCCC
CATATGCCCTATTAGCTGGCTCATTGTTTTGCAGCCATAGTGAAATTACCGAGAACATATGC
GGATTATAAATACAACATTATTGCCTGTATTTAAAAAGCACACTAGCCTATATCCAAGATTGCC
GTACTCTACTGAGCTCAATCTGCGATAAACGAAATATGTACCTTAGATCACATACAGTAAGAG
GAAAAAACCAAAACGTACACAACGTTAACAACATACAACTTTTACGCAAGGTTTTTCGGCA
TTGGCCTTTCGTTTCCTTGTTGGATGTTACCGTAAACATTTGCATTGATGTGCATTGCCGTAA
ACCCTTGAAATCGCAACTTCCCTTTATGCCCCGGTATCCCGTAATGCCCCGGTCTCCCCA
AAGTAATGGATATAACACTTTAAAAGAGAGTAAAGGACCAGAGTTCAATTTTTCCAAAAGACA
CCTTATCCTTCGTTTGCTAGAAGGCGTCGGAATATTGAGCAGTTGAAACTGATAAGGGCCTT
AATTTGTATGGCTGTCCATAAACAAGTTAAATTGAACTACTGTTAAACTGTTATCTGCATGTTG
CTGCACTGAACATGATGTGTATTGTAAACATGTTCAGTTGTTGGTTTGAAATCATTAAAGTAAA
GATTGCTATTGTTATTATAGTTGGTATTTTTATTTTTATTTATTTATCGCATTTCTATAGCGCCCCTT
CAAGCAATCTACTCAGAGGCGCTCCCGACCACAGCGCAGGCACTGTGTCGGAGTTTCA
CGCCACTGGCGCCTTGAGAGCAATGGTAGGTGAAGAACTTGCTCAAGATCAAAACGCAG
CGGCTGGACTGGGAATTGAACCCGCGACCTATCGGTCGCAAGGCCGCAACTCTCACCA
TTATGCCACCGCGCCCCACAATAGTTGGTAGTGAAAGTAGTAGTAGTGGAAGGTGTGGTAC
TAGTAGAAGTTTACAACGTCTCAATTGGTAGATGACAGAAGGTTCAGAGGTCCTAAAGTATA
GTAATATAGTATACATGTAATATAGTAGTTATAGTACAATATAGTAGTTACCTTCAGCTGGATATG
ACAACTGGCGTCCTTCCAAGACTTCATAGCTTAGGCCTACACTTGTGTAGCACACAACGAA
TAGTTGTGTGCTTAGTCAAGTAGTCCAAGATTAGTCAAGTAGTCCAAGACTCCCAAGTTCTTG
TGTTCTTTAATTGGCTTTGAAATACCGTCACACAAATGTGTTTTTATGACGAAAATCGACCAC
ATGCGCCTTACTTCAGGCATCTTATGCATGGAAGAATGGCAAATGGACCAACACAATGCAA
TCATCCTTTTAATCAGATTGACATGCCGTTATCATATAAACAATGGATGATGTGTGAGTGTAAT
CTTTTGAAGAAGATGCATTGATGAATGAAATCTTGTTGTTGATATTTAGCTAAAGGGAAGTTCA
TTTAGCGAAGCTCCATTGGAAATGAATAAAATGATTGAGGTCTTGCACCTGAAACAGATGAT
CAACACAAAGGCGAAAATTCTCTCAGGCGGACAGAAAAGGAAGCTCAGTGTCGGAATTG
CACTAATATCAGGATCTAAGGTACCGGTAACGATTATAGATACATGCAATTAGGAGACATAAA
AATTTGTTTTTCATTTTAACCATTTTCATTTTCATTTTAACCATTAAAATGTGTTTGTGTAGGCCTA

TCCTACCGTGCATTTCATGTATCGTTTTCGCGTGGCGGTATGCAATACTTACCCAGAGCATA<br>TTGCATAAAAAAGTGTTAAAATTGTAACCCTAGGGCTACCAAGCTT
>472C1
AAGCTTGGACAAAATGGCGACTCTCAACGCGTTCGATTTAATTCATGAGGCGCCGTCACTT<br>TAAAAGGACAAGTAAACATAGCACTTGCGCGAGATAAGATAAATAAGTAAGGCCATCAGGG<br>TAATAATCCATACATTCCTGGTCCTAGTTTTAGGCTTACTGACACTGCCCACTGCCATAATAC<br>TGTTGTTCTCCAATTTGAAATAAAACATTAGGCCTATCATAAAGCACCATCATATTTTTGTTATT<br>TCACGGCTCGTCATAGCAATTTCGAATTTGCCAGCGTCCACAAAAGTTATATCGCAAGAAC<br>CAACCGCGGCCACCGGGTTTTCCCATTTGGGAATTCCCGGGAATCGCGCTACCCTGAAT<br>TCCCGTCGGGAATTCCCGGGAATTTTATCGAATCGTGAACTTTGAAAAAATCACCAAACTG<br>AAGCTAAATCAGCGGTAAAAAGCCCGTAATTAAGAAAAAGTGTCGCCTATTTATCTAAAACC<br>CTAAAAACCTGAAAACTTTTTAAAAACTTAGGCTTTTACCAGCCCTGTAGTGCACACATTAC<br>CTTTTTCAACCGCACATTTCGATTTTACAACTGCACAAATTGCAATCAACTGCACACTGCTAT<br>AGGCCTATAATGCCTATAATTATAGCCCTGGTTGACCAGACGGGAGGTTCAGGTCCGGTAA<br>ACCGTCTGACTGTCTGCCTGCCTGTCTGGACTCTGGATCTTGACCGCTGCCTATCGATTTT<br>GCTTTGGTTAAGCGCCTGTGAATAAGCTGGTTCCTGCGACTTCGCTTTCAGGAGTTTACTG<br>AAATAATTCACTCGACCCTTGTAAAAAAACTTTCTCGACTCGACCTTCCAGATAACATCTAC<br>AATTGGATGGTTGACTTCCTTCTAGATCGGAAACACAGAACTCGTTATGCTGGTCAGCAATC<br>GGTTGATGCGTCCATCAACGCCAGTGTCGTCCAGGGCTCCGGCTTTGGCCCTTCGGCAT<br>ATGTGGTCGTGGCATCAGACTTACATCCGGTCCATGATCACAATGTTATTGTTAAATTTGCCG<br>ATGACACCTACCTTATAGTGCCTGGTTCCAAGAGAGATACCATCAATAAAGAGCTGGAGGG<br>CATCCAGCGTTGGGCTTTGCTCAATAACCTCAAGCTTAATCCTAACAAATCTAAGGAAATG<br>CTGTTCAGAAGACGAACTCGAACTGTCATTCTACCTCCGCCTTTACCTGGGATTGAACGAG<br>TTGACTCCATGATTGTTCTAGGAGTCAATATCAGTTACGACCTTCGGGCATCGGCACATGTA<br>GACCGCCTCCTAGGAAGATGCACTAGCTCCTTGCATGCCCTACGTATTCTGCGAGCTCAT<br>GGCCTGCCACAGGATGCCCTACACAATGTGGCCAAAGCCACTTTATTGTCCCGACTGCT<br>CTATGCCTCTCCATCCTGGTGGGGTATGACATCTGCTGATGAACGTCTAAAGATTGAACGC<br>TTTATCAACAAATCGAGGCGTCTTGGTTACCTCCCTGCCAACCAACCAACCATGGAGACA<br>ATGACCGATGAAGCTGATAGACGTCTACTACGTGCTGTTGTCACCTGTAATAACCACGTATT<br>GATGTGCCTCTTTCCTCCCATCCAAGCTACTCAATATAATCTTAGACCTAGGGCCCATAACT<br>TCACACTCCCAGAAAAAGATAACATTAACTTCATTTCAAGGATTTTGTTTAAACGCTAATTTCT<br>CCTTTTCTAGCTGATTCTAACCTGCCCTTTATCCCACTCAAATGAAAGTACTATATGTGATTAC<br>ATTTCTAGTCTGTTATTCTTGTTATAGTTAAGGCTATTATGTTGTGCTTACGTCGTCTAATTGTCT<br>GATTGTTCGTTTGTTCTCTGATGCGGTGTGTCATCGGATTTACAATAAACGAAGATATATATAC<br>TGCCCTCTCACTCAATCTTCCTGCTCTTCCTTCCTCATTTCGATTTTACAACTGCACAAATTG<br>CAATCAACTGCACATCATCTCTCATTCTCCAACCACATCTCCAACCTCTCAAGCTCCTGCT<br>TCATGCACATACGCGATCTCTGCCGCATCCGACCCATGCTTGACTTTAAAACTGCATCCAC<br>CATTGCCACCTCCATCGTCCACTCAAAACTAGACTACTGCAACTCCCTCTTTCTCAACCTC<br>GACTCCACCCAAATACAGCGTCTACAGCTCATCCAAAACTCACTCGCACGCGCTGTCAC<br>CAGAACGCCCCGGCATCATCATATCACTCCTGTCCTTAAATCACTTCACTGGCTAAAAATC<br>CCAGAGCGCATCCACTTCAAAGTCCTGTCTCTAACCTACAACTCCCTCCAGTCCTCCCAG<br>CCCACTTACCTTCGCGAGCTTTTCACCATTCAGCCAACCCGCTCTACCCGATCCTCCTCC<br>TGTCTCACCCTTTCTCGACCCCCGGTCACCTCTCACCTCACGTTCTCCAAAAGAGCCATA<br>TCCGTCACTGCACCGCGTCTCTGGAATGACCTGCCACCTGAACTTCGCACCTTTTCTTTAC<br>CTCCACCATCATCATACCAAATCATAAAACATCATCTTCAGCACGCTCCTCTGTCTGTCACC<br>CCCCGGGCTTTCCACTCGAAACTCAAGTCCCACCTCTTCAAGCTCTCTTTCCCTGACTCA<br>CCTGATTCCATTTCTTCCCACTCCCCTCCTAAACTACACCCGCCTTAACGCCTAATCTCCC

TCCTCTCACCTTCTGGCAACCGGACCTGAGCTTCCCACGGACTATTCTTTGGAAAACACC
TCTGATTTGACGCATTCTCTCACCCTCTGGAAAACGAACCTGAGCCTCCCACGGACTATTC
TTTGGAAAACCCCTCTGATTTGACGCAGCGCTCGTGAATAAGCTGGTGCCCTGCGCTTGG
CTTTTGAGTGGCGCTTTAGAAGTTTGGAGTTTACGATTACGATTCCTCAAGCGAACGGCAC
ACATACTAATCATCTCGCAATGCAACAGACACCAGACGTATTCACTCCACTCAGGCATCAC
GCTCATACTCACACAAGCACTCAAGCAAGCGAAAGAAACGGTACGTTACAAAGGGTTGAA
TAGGTGTGGACTAACAATATTGAAGGAGAGGAGGAGCAGAGTTTACTTAATCTTAAGCGAG
GCCTAGAAGATTATTACGTGAAAGGAAGCACTACAGTGGGAGAGGTTACTTAGGGGCCATT
CATTTATGACGTCCACAAAAAATCAGGTTTTTGACCCCCTCCCCCTGTCCACATGGGCCG
GACCCCGTCCCCCCTTGTGGACGTCCACACGCGGTCGACATGAAATACACACCGCTCT
CTTGAAGCGGCTAATGGCCTACGTATACCGGACCTAAAGCTGAAATTCGACTATATGATTGT
AATTTATTTAAACTGTACTATTAGTAATTTTTATTACTAATTTATATCACCGAAAAATTTCCACTTTT
TATTCCGTCCAAAGATGAAATTCTTGTAACAAGAAAAGCCCATAACAAGTTTCTATTATAAATC
CTTTATCTGCAAAATCAATCTGGGTCAAGGTCGACTTCTGGGTCAAGGTCGAATTCTGGGT
CAAGGTCAAACTTGGATCAAGGTCATGTCCAAGGTCACGGGCAAGGTGTGTGTGGCTGC
ATCTGGCTTCGCCAGATGGTCAATGTCTGTGTGTGTGGTTGCACCAGATGCCTCTGTTGAC
AGCGCCTCTAGCGGATTAATTGGCTAATTTAGCCTATACGCATGTCCTTGACGACAAGGATT
CAATAAATCAATCAAGCAAGCAGGGCAAGTTAATATAAAGGATAAAGTTAATTTAAAGTTAATT
TTAAAAAGACGCCAACTTCTTTGCATGAGAAGAAGACCGTATGACGTCAGTGATCTTTAATTT
TAATTTTCTATGTGGACTTCCACATAGGGCTTGACCTCTCTCCCCGTCCACATGCGTCCAC
CTGAGCCTGACCCCTCCCCTTCCGTGTGGACGTCATAAATGGATGGCCCCTTAACAAAG
GATTCTCAGGGAAACAGGTAAAACTGATTGAGGACAAAATTCTTCAGTGCAAGAATTGGAC
TTGTGGAATGGGTTCATTGGTTGCATGATGGTACCAAAGATCTTTGATAGTACCGTCTCAGTG
GACACACTCCTGGACACTGTCCAGTGACAGTCACAGCTTTCAAGAGGAAGCCTGGGAAA
CTGGGCCAGTAAGCATCCCAGGTTCATTTGTAGCAGTTTTAACACAGGCTATTTATTATACGT
CTTCTGAGACACAAGTTTACAACCCTTTAATTGGCATTTCTAAAAGATAGGCTACTTGTGCTG
CTTGTAACTTGTTGCACAGCTGTATTAATAATAATAATAATAATAATAACATCGACAATTTATAAC
TGGCGCCGTAACACAGCCATGCCGCTACAATTATATCAATTAGAATAGATTAATTGGCCAGT
CATTAGTCATTCAGTCAATAGCCACCATGAATTAAATACTAAACACAAAGTGCACAATAGGT
CAGTAAGTTAGGTAAAATTTACAAATTGCAAAAACTGAAAAGTTTGAATCTGGCGCACAGAA
GCACCAACAATCGTCTTTTGGAAAGGCAGCTTGCTGCACAGTGTGGATTAATTGATACTTTC
GTTAAATGAACAGTGACTCTTTTGAAACTGAGTCTTCGGATAATTTATTACGAAGTGACTAGA
GGTCAGAGTTCGGACCCCGACGTAGTTTCGTATAAAGTCGCAAAATACTGACACTCGACAT
TCAGTGATTGGTAGGCCTATGTAAATGCCCTGGCAGAAATACCTCTGTCCTGGCAGTCAAA
ATTGGTAATAATAAAATTATATATAAAAATATTTTGACTGCCATTGCTGATGCGACTATGGACCT
GTCTATGCCTGCCATAAACAGCGGACTTGGCACCGCCACTGCTGTTGATCCAGATAACAC
TGCACATAGATTCAAACATGCCTGCAAGGCAGTATGCGGCCCTATTGCAATGTGAGGTATT
CAAATACAGAATTTTAACTTAATCAGAAAGCACGTTATTATTGTTAATATATTATCCGGAAATGA
TCATGGAGCTGTCTGAGACTACAACAGGACAGAGCACATAACAAATCAAAGTAATAGACCT
ACAGAAAAGACTACACTAATACTATCTGACTAAGTTAGTAGTTCTTGCTTTATAAGGCACAAAT
TTACGCCTATGGCCACAGGTTATACTGTATGTACCAAATTTAACAGAACTCTTGTCATAATCTA
CCTCTCCTTGTCCACATTTGAGGACACTCCTTCCAGCTCATTGTGGACGTCCATTTTGGGG
CTAATATAATTAGGAGACAATCACAGCTAAAGATTATCTAAACCGTTAATATCAACTAATAAATT
ATTCAGCAAAGCTGGTGGGGTTGTAGTTTTCTTCCAGTTTAATTTGTTCTCAACCACTTTTTCA
ATTTGGACGATCAAATGTTTCCAGTCTCTAAAATAGTGGATTCTGAACTGGTCACACAAGTTT
TCAAAAGTCATTGTTAAGCGAAAGAAGACGTAATAGTTTCATTAGAAGTCTATGACTCTTCCTT
TTCTCTCCCAGTCCCCATAGCGAGTTGGTTCTGGGCCACGGGGTCCTCCGATCTCACCC

GTCTTTGGGTTCGTGGCATCTGGAAATGGAACATATGGGTCGTTAGCTTCACCCGGCTTGT
TGAGATTTTTCACAGGAGCCATTTCCTCCCCTGCAACTTCCCAATGATCATCTAACTTTCCG
ATTGGTGTCTTTGCCTTCTTGACTTTTCGTTCACTGCACGTTTCTGTGTCCGACGCTGTGGC
AGAAAACTGTCTGACGCTAGAGCTGGCAACACACCGTGACAACCTTCCTAATCACGAAAA
ACATGAGACTTATTTGCATCTGCATAACTTGAAATCTCAAGTTTACAAACATTATCACAGTGCA
GACTTTTTTATCCTTAATGCTAATCATGATCGCAATCTCTAAGTTTGTAAAGTGCTGAGCTCCA
GTTGATTTGCGAGCACTGAGAGCATTAGCTACAGTGAATGCAATAATTATTTTCATTTCATTGG
TTGAGTGCAAGTAGCAGTGCAATCGTACGTCATTCGCAGTTGGCTAAATCTTCTGCGCAGG
AGTAAGTTTCATGACAATTCCGTGGTGGAAAACAATCCAACTTTCGCTTACACTCTCATTATT
TTCTTTGATCAGACTTCTACTTACACTATAACATATATGCATCATGTACTCCTCATCATGTACTC
CTCGCCTTCTTAAGTTCTTTCTTTTTCCTGGGCTTTATCGCACTGGTGGGCGCCTCTGATTA
GTTTTTACTGCGAGTGGCGCCGTATATAAATATGTGAATAGAATAGAATCATCGTGACGGTCA
TTTAGGGTCAGGTCTAGGGTTAATTTAGTCATTAGAGACATAGCAAAAAATATATTCCGCCGC
AGAAGTAATTTTTCTTGCGTTTCTGGCAGAATATTCTGCCGCAGAAGCACCACGCTTCGCA
GTTACACAGACAGTACGTGTGTAGGGTTGCCAGGTGGCTCTCCAAAATACTGGACACTGG
CAGTGTCAGTATCTGTGGCACAAAGTATTGAGCTATTTGCACATGCATTGTCCGGTATTACTA
TCATTATTATACCGGACAAAGATCCAAAATACCGGACAGTCCGGTTAAATACCGGACACCT
GGCAACCCTAAAATGTACGCGTAACAATGATGATCATACCACTGCCCGTACTCTGCCAGT
CCTAAGCCAAGGTAAAGATGGAGTATCACAGTCATAATCACATTTTAATAAGGACGAGACTA
CAGCACCGTCGACGGTTGCAGCAAGCGGAAATGCTATTGGACCAGAGTGTCATGTGATTG
CGGAAGGCTACAGATTGGTGATGTGATTCGGGAGGTGGAATTTCTGTCTAATGACCACACT
AACCCTAATACTAACCCTAAGACCCTCATGACGCTTGCCCTGACCCTAGCCGGTGACGG
GCCGCCACTCCCAGGGATCGGCACTCCCAGGGTGCGTATACGAACATACGATGCCATT
CTGCCGGAGTATATACGGGCGTTGCTTTTCGGCTGAAATATAGTATTCGCGATGCTGTTCTA
CCGAAATATATACGGGCGAACCGGAACGATGCTATTTTGCTTGAGTATAGGCCTACAATTA
GGCCTATGTGCGATGCTGTTCTGCTGGAATATATCAGAGCGATGCTGGGAGTGGCGACCC
CAAACCCCCTTGCCGACCATCACGACGCTTTTGAAAGCTTTTGTGCGCCGGTATTTTGCG
AATTTGCACGGAACTATGCCTTCACCGTCGACGGTGCAGGAGTCACTTCGTTTTAATAAAG
TACTAATATAGCGTCAATTGCTATGACTTGGAAATCAGAGGCGCTCTCGACTCGCGTCAATG
TCATGTCATCACACATTTTAAACTACTACTACGTTAACTTTTAACTGATCAGAATTGCTTTAAAA
AATTTAAATAAACCCATAAATTATAACAGTTCTTCTATAGTGCCAAATAAACTTTTTAGTTACCA
AATGAGCCTGCTACAAACGACAACTTCATAACATTGTGCGCCATTCTCGTATTATGCTTTTAA
ACATGCGACGGTTGGTTAGCCGTTGAAAGTCCTTCATTCATATTCACAAACACACGCACCC
CGCCCACATCGTCGACCAACAAGATAAGCAGCAAATCAGAATCGCGCTCTATGAATATTAA
TAAATAGGCTAGGCCAAGCACGACCTTTACGCAAGGCCTTTACGTACGCAATAACGTTAA
CGTCAAAGGTTAAAAATGTTGTAGCGCTTTGACTATTATCACTTGTTTAGTAATCGCACTCATC
TACCCATTTTCACAAGCTCTTATTTCAACGACCTTCTAAATAAAAACAGATACATCCAAATTC
CAATCATCATTTCGGCTACGTATTGAGCAAAAAATACATTTTAAAATTTGATCATTTCACTGTA
CTTTGAGGAATTCCATAAATGAGGTTTGTAAGCTATCAGAATCCTGGAATTGCAGGTCTCAC
AGCGCGAGTGTCTGCCATGGCAACTAAGACTTCTACGAATTAACTAAGAGATTAAATTCATT
CTAAACACACAACCATTATAATTGGAATGTACATGTAGCCTATAACCTGCACTCCAATCTTAT
TCTAGCAAGCCTTACTTGGCCATTGGTTTTGTAAAAATTCAGAAGTACACGACTATTTTATCA
GATCAACAAATAAATATCATCAACAAAGTGTTTGCACCACATCAGGACAGCATTCTATAAGA
ATGTGTGGACCATTGCTCTGCAATTCTCTACCTCTGGAACTCACTAGTGTAGGCCTACATGT
ATTTGCATCTACAATTTACCAATGTTAAATACAAACTAAAGTTATTTATTTCAAATGCGCTTGTG
TAAATGTACAAGTTGACTTTTATTTTATCTTGTATCATTTTGTTTTGCAGTTGCTTGTTAACCGATT
TATACTGCAGTTCTCTTTTGTGTTTTTATCCACGTTTCCCATGAATTGAAATACGGACAAAACA

ACAATTATGTGATTGTCTATCAGTGTACTATGTTATGCAGATCAATCAATCAAGCAAAATTTATA
TAGCGCCCCTTCAAGATACCTACTCAGAGGCGCTCCCGACCCAGGCCAAGCGGAAAAG
AACAGTCTTAAGAAGGTGGTGGAATTGAGAACAGGCACCGTTTGGAAGGTATCTTAGATCT
ACTGGAAGTCCATTCCAGGTTTTTGGACCAACCGCAGAAAATGAACGGGTCTGCATTGATA
CAGATAGTGCATGTAATAGCATTGATTGATTGTGATGTCTGATGTAATTAGGGCGACCCTGTT
AGCGGAACCATACGTTCATTCTGGTTACGATCTTAATTATATACCTTGGCAGATAGTCATTAAT
TAAAAAAAGTAAATATGTATCGGGCAAGGACTGATGGTATCAAAAGCAGCAGTAGTGCTCGT
AAAGTCTGTAGAGAAATGGTTGTCTGTACATCAGCCAAATATCCTTCAACCACAAGATAGAT
AGATAGATAGACAAGATCACATATCAGGAATGCTGTTTAAAGAAATGTGTAACATGTACATGT
AGACAGTTGCGGATCCAGACATTCGGCTAGGGGGAATATCGTGTGACGTATCCAGAGACT
CAACATTCGGTTCAGGGCCATATTTAATGTTTCCCAATATCTCATGTTTGTTTCTTCGTATAAG
GGTGGCCAAAGCATATAGCCAATCTGGATGGAGGGGAGCATGGCCGGACATGCCCTCT
GGATCAGCCACTGCATGTAGAAATATATGTTAATCAGGTGCTACCCAAACTACCTGACGTTA
TGCACAGTTCAACAGTGTGACCTACATCAACTTCAATGACCATTATGAAAATCACATCAAGA
AAACGACACATTCGAATATGCAGTTTATAACTTTTGATCTTTCTATGCAAAGTCAAACGGTCAT
AGAACATGTACAATTTAAAACAAAATACAAATTTATCAAATTGGAATACTGAATAACTGAAGTT
ACTTGAGAAAAGTTACGCAGTGTCCTTCAGAATAATTCTATCGCAAATAAGTAACAGAAAAG
AATTTATGTATTAAACTGATGACAGAGTTCCATATTTCAATGCCTTGGCTCTCACTAAAACAAA
AATCAAAGAACAGTTACACAGTGCATCAATATTTCAGTATAGACATAATATATGAATAACATCA
ATTTCCACACAATTAAGTTTGAAAAACTTTATAAATGACACAAATCATATCACAATCATAGCTA
CTAATCAAATCAAACATAAACACTTCTATAGCACCATTTATTAACGCGATTGGTCATCACTTC
ATATCGCCTGGGGTAAAACTAGGACAAAAATTTTTTTAAATAATTATTTTGGCTCTGTTTTAAC
AAGTCAGAAGCAATCTTTAAGACAGCCTTCACACAAATGCACAAAAGACAAAGTAGTGATT
CTTTACATGCAACACAAGTTCTTTCTTCAATACCGTAATACTAACAAAAGAAGGAAAAGTTTA
GTCTTACACGTACACTAGTCACCACAGCATCCCTTCTTGCATCCTGCGGCGCACGAACC
CGCCGAGCATTTGCAAGCTGCCCCGCACTTGCAGCCGGCATCTGAAATGTACATTGTGA
GGACATTGTAGTAAATACAATGTAAAAATATTTCTATTATTGTGTAAAAGATCTCTATACAGCAA
AACTTCATTATATCGAACTTCGGTATTTCGAACTTCCGGTTTTATCTAATAACGCTAAATGGCT
CGGCTACAATCAATGCAAATAAATGTATATGGCTTATTTCGAACCTCGGATATATCGAGCCGT
TAATGCCGGTTCATTTAGGTTCGATATAATGAAGTTTTGCTGTAGTTCAGCCACAGCTGAAAA
TTTTTCTGAAACTAAAGGACTAAGTAGTTCATAAAGACACAAATTCACTGGACTGATCAGCG
ATCATACTTCATGACAAATCTCTTCTGGTGACACAGTACAGAATGGAGTAATGGGAAAGTGTT
CAAGAAGCATGCAAGATGCGCTGCAGGAAAAATGATGGAGAGAGAGAGAGAGAGCTAAA
CTGCACACTGGCCACAAACTAAAGGACAAGCAGATAAACTTGTTAATTCTTCACAGGCTGC
CTTGTAGAATGCTCTTCTTGGTATTATTCTGATTATGATTTGGCTTCTAAGTGCCAGTGGTTTAT
GTCTTGTGCATAAGGTGACATAAATTGGAAATTACAGTAACAGAACATGTCAGAAAGGTTTCA
CTGGATTTAGAAGAAAAGATAGCCATTATAAAATAAATTTATCTGTTTCCAACCAAGGCTGCA
CACAAGTAGCTCAGCATTACAGAACAGCTACTGCATCGGGATCAAATTTATGAAAATGCAG
TAACCAATAATATACAAATTCCTGTTGTTTAGTTGTACAAATCGCATAATTATCCCTTTTGCTGA
AAGCAGTACCCGAGGACCAAGATTCAGCATAGTTTTATTTCCAACAATTATTGTACAGTGTA
CGTCCAGGTTCTCGTAAACGAAGAATTCTTTCATCAATCTACCTTCGAGTTAATAAAAACTCC
TTTCATCAATCCAGCTTCTATGTAAGCAACTTTGTTCATCGATGTCGGGGGGTTATTGGCCG
GGGAGTTTTTGGCCTGTTATCGCTATCTTCATAGTGGAGTCAAAACAAGGCTCAGCATGTAT
AAGGGAACTTTATTTGAGAGGCCTGTATTTATTTTCTCAAGTCATTTTGCCCAAGGGGCTATT
TGAGACAAGCATTTAATCAGAAACTGGTGCTTTTACAAAGTTCTATGGTAAATGAATATTTGGA
CAAGGCCAGAAATGTTTAGGTAAATTATTATTACGCTGAAACTATGTTTTCTATTCCTGAGAGA
TCATTTCAACCTTTGACAAAAAAGTTGTTCAACCTTTAAAATTAGTATTCGACTGCACTATAATT

TTAAACAAAACACAAATAGCAAACTCACTTCCACATTTGCCCTTCTCGGCGTCAGCACAG
CAAAGCTTTTTGCAGCCTGGCAAGCACTCACTTTTCAAACATCTGCAGTTTGTGCAGCAAC
AAGTTGATCCCTCTCCTAAAAAAATAAAAGAGTCATTCTCTTTGACTGAAATAATATACTGAGA
TTTTCGGCTTTACAAAATAAAAATGTATGTAAATTGCAAGCAATTTAAAATCAGCAACAAGAC
AGAATAACTTCCTTAGTACTTCTTGTACACGCCAGAGGTCTTGTGACACGTTTGGTAACTGAT
GATGACTGCTGATAGATGATGGTAGTAATGACCACACCATGAGCATTATTTTGATGGTGGCG
ACAAGAAAAGGAATGATTCCATTCAAAAAGAAAGATTTAAGGTGGTGGAGGTGTTAGCAATT
GCACTTTTGGGAGGTTCTCGATAGGCACTATATAAGTGTTCTGCCACATTATATTCTATTAAAT
TAACATGGTATGAGTCATAGTGATGATATTGCCAGTAGTGATGACAAGAACGGGTAAAATGTC
GTTATTATAACACGGATGTAACAGTGAAAGAGAAGAAGTAACAATGCTAACATCCAACTCGC
GGTAATAAAATTACTAACTGTGAGAGATATCAAAGACATGTTAAATTGTTAAGGCAAGTAAGA
AATTTGGCTTACTTTGGCAGGTTGATTTCCACAGCACTCTAAAAGCATAAGGAGGCAAAG
ATTTGTTAGTAACTATACACTACAAAAAGACCTTGCAAACAATTTGAAACTAGCAACAAGGC
AGAATAAGTTTCTTGGTACTTCCTGTACAGGTCTGTACCATCTGGACGCTGGCTAATGGCCT
AATGCATCTCCTCAACTTGTCTCTATCCAATTTGGCAAGTGAGCTTGTTGCCTTGCTTCTGGT
CTGACTTTTTTAGCTGGTCTTAGGTGGTGTGAGGAGGCAGAATATAGCAGCTTACATAATCG
GATTTCAGTGACTGATATTTGATATTACAAGGTTATACCCTTAGCATGGTAATGTAAACTGCTAT
TATTTAAATGTTTTACATCTTCTATAAGAAATCAATTGCATAAGTAGCATAGAGCCTCTTATTTAA
AACATTGCTCAAGCAATGCTAAATTTTGCTGATGGTAATTATGTTAATTGCCTTACGAAATAAA
TAAAGAATACTAATGATAAATTAGTTTTGGCCAGATATCATCATCATAATCACCACCATCATCA
TCACCACCATGATCATCATCATCACAACCACCATCATCATCATCATCACCACCACCACCAC
CGCCGCCGCCGCCGTCGTCATCGTCGTCATCATCATCATCATCATCATCATCATCATCATC
ATCATCATCATCATCATCATCATCATCACAGAACTAAAAATGGCTTGTACACATATGTCTGTCA
ACTTGAGGACCAGATTGGGATTCGTTTTCTAGCCTCACATTCGCTGGGCGAGTGAGCTCC
CCAGTTAACCAAGGGCTTCTAAGATGGGGGCTGAAATGAAGACCTCAGACCCTAACCATC
CAAAATCGCTTTACATGGTATAGTACACACCCCATGTACTACCCAAAATGATGGGCAGTGTA
TCGTTTAAGTTGTTAGTGTCTCGTCCAACCCACTCCAGACTTCGCATATAAATGCATGAAAA
CTGCTCAAAGTTAGGGTGCTGAGATTAAGAACTCAGACCCTAACCATCAAAAATCACTTTAT
AGGGTATGTTACCCACCCTATGTAGTACCCAAGACAGTTAGCGCTATAACGTTTAAGTTATTA
GCGTCTCCGTGATACATCATCAAACTTCCCATATGAATGCAAGATAATTGCTCTAAGTTGAGA
GGCTGAAATGAAGAACTCCGACCTTAACCATCTAAAAGCGCTTTATAGGGCATATTACCCA
ACCCATCTACTACCCAAAATGCCGGGCAGTATAGCGTTTAAGTCATTAGGAACTCCGTGAT
ACCTCGTGAAACTTCACATATAATTCTCAAGAGCCGAAATTACGTTAAAGCGGGGGGTCTG
AAATGAAAAACTCAGACCCTAACCCTCCGAAAACGCTTTACAGGGTATATTATCCAACCCA
TCTGCTACCCAAAATCATTGGTTGCATAACGTTTAAGTCATTATGGACTCCGTGATACCTCAT
CAAACTTCACATATAATTGCATTGTTCTTTAACAGATGAATATTCCTCGAGTTTAGGGGGCTG
AAATGAAGGACTCAGACCCTAACCATCCGAAAGCGATTTTCAGGGTATATTACCCACCCC
ATCTACTACCCAAAATCTTTGGTTGCATAGCGTTTAAGTCATTAGGGACTCCGTCATACCTCA
TCAAATTTCACATATAATTGCACTGTACTTCAAAGGCTGAAGTTTTCTCGAGTTTGGGGGGCT
GAAATGAAGAACTCAGACCCTAACCATCCAAAAGCGCTTTCCTGGATAAATTACCTACCCC
ATCGACTAGCCAAACTGATTGATAGCATAGCGTTTAAATCAATAGGCACTCCGTGATACCTC
ATCAAATCTCACATATAATTGCACTGTACTTCGAAAGCTGGGTTTCCTCGACTTGGGGGAGG
GTTGAAAAAAAGGACTCAGACCCTAACCATCCATACGCGCTTTACAGGGTATATTACCCAA
CCCATCTGCTACCCAAAATCATTGGTTGCATAACGTTTAGGTCATTAGGGACTCCGTGATAC
CTCATCAAACTTCACATATAATTGCACTGTACTTCAAAAGCTGAAGTTTCCTCGACTTTGGGG
GTGCTGAAATAAAGAACTCAGACCCTAACCATCCGAAAGCGATTTTCAGGGTATATTACCC
ACCCCATCTACTACCCAAAAGCTTTGGTTGCATAGCGTTTAAGTCATTAGGGACTCCGTCAT

ACCTCATCAAATTTCACATATAATTGCACTGTACTTCAAAGGCTGAAGTTTTCTCGAGTTTGG
GGGGCTGAAATGAAGAACTCAGACCCTAACCATCCAAAAGCGCTTTCCTGGATAAATTAC
CTACCCCATCGACTAGCCAAACTGATTGATAGCATAGCGTTTAAATCAATAGGCACTCCGT
GATACCTCATCAAATCTCACATATAATTGCACTGTACTTCGAAAGCTGGGTTTCCTCGACTTG
GGGGAGGGCTGAAAAAAAGGACTCAGACCCTAACCATCCATACGCGCTTTACAGGGTAT
ATTACCCAACCCATCTGCTACCCAAAATCATTGGTTGCATAACGTTTAAGTCATTAGGGACT
CCGTGATACCTCATCAAACTTCACATATAATTGCGTTGTTCTTTAACAGATGAATATTCCTCGA
GTTTGGGGGGCTGAAATGAAGGACTCAGACCCTAACCATCCGAAAGCGCTTTTTTGGGTA
AATTACCTACCTCATCGACTAGCCAAACTGATTGATAGCATAGCGTTTAAATCAATAAGCACT
CCGTGATACCTCATCAAATCTCACATATAATTGCACTGTACTTCGAAAGCTGGGTTTCCTCG
ACTTGGGGGAGGGTTGAAAAAAAGAACTCAGACCCTAACCATCCATACGCGCTTTACAGG
GTATATTACCCACACCATCTGCCACCCAAAATCTTTGGTTGCATAGCGTTTAAGTCATTAGG
GACTCCGTCATACCTCATCAAATTTCACATATAATTGCACTGTACTTCAAAAGCTGACGTTTC
CTCGAGTTTGGGGGCTGAAATGAAGAACTCAGACCCTAACCATCCAAAAGCGTTTTCCTG
GGTAAATTACCTACCCCATCGACTAGCCAAACTGATTGATAGCATAGCGTTTAAATCAATAG
GCATTCCGTGATCATCATCATCATCATCATTAACCAAGGGACCAATGCCCGTAGGTGCCC
GATGCCGTTCTCGTTATTCGACGCCATTTTTGTTTCTCCAAAGCAGCGTGGTATAGTTCTTGT
ATTGATGCACCACACCAGTCTGTTATATTGTCCATCCATTCTGTATGTGGTCGCCCTCTTTTG
TTAGTTCCATCCAATCTTCCGAATACTATTGACTTGATTTTTCTGTCGTTTTCCATCCTACATAT
ATGTCCAAAGAGTTCTGGTTTTCTGGTTATTATTTTTTGCATAAGGTTTTCCGTGAGGTCAATTC
TTCTGTATAATTCTGCATTTGTTACTTTCTGCATCCAACCAATTCTCAGGATCTTTCTATAACAT
TTCCTCTCGAAGGCTAGTATCTTCGCTTCTGCCACTTTAGTTATTGTCCAGGCCTCGCATCC
ATACAGCATGCCGCTGAACACACATGTTTTCAGCACCTCTAGTTTCAGTTTCTTATGAATTGA
TGTACTCTTCCAGATTTTGTCCATCGCACTTAAAGCTGCTATTGCCTTCGCAATCCTGACCA
GAATTTCTTTTTTACTGTCTAAGTCGTAGGTATTTAAACAACCTAGGTATGTAAACTTTTCCACA
TTTTCTAGTTGGTTTCCATCTATTTACCTCATCTAGATACCTCATCAAATCTCACATATAATCCA
CTTATCTGGCTTACATCCTATCTTTCGGACCGGTCTGCTTCTGTCACCTTTCAATCTTCTCGT
TCGTCTTGGCGCCACACCCCTTATGGCCTACCCCAAGGATCTGTTCTTGTGTTCTTGGGC
CTCTTTTCTATATTCTGTTTACTGCAGATACTGGTCCTCTTCTCGCATTTTGTTTTCTCGCAAGT
CATTATTATGACGATGACGTTCAATCCTACAAACATTGCGATCAGCATCTGAGGCAGCTACT
GCAATCCGAACTATGTCCCGGGCCACCGATGCTCTCAATGCCTTGGATGTCATCTAATTGC
TTGCTGCAAATCCGCAAAAAACTCAGTATATTTGGTTGGGCACCCGCCAGCAGCTGGACA
GGCTGGACTCTGAGTCCCTGTCTGCTGAAATCCCTACCTTTCTGTTCTCCACTTCTGTCCG
GGACCTTGGGGTCATCTTGGACCAAGGACTGTCTTTTACTGAGCACATAATTGCACTGACT
CGATCCTGCTATTACCACTTGCGCCAACTCCGGGGTGTTTCCCGCTCTCTTTCCTCCTCTT
CCGCTTCTACTTTGGCTCATGCCTTCATAGCTCATCGATTAGATTATTGCTCTCCTTTCTGCT
GTGGACTCTCTCAAGTTCGATTGCGGCCTTTGAACGGTGTTCTGCGGGAGGCTGCTCGCA
TGATTGGTGGTGTGCCTAAGTTTGGCCATATCTCAGACTACATGTGGGATGTACTCCACAGG
CTCCCAGTTCAGCAGCCCATCCGCTATAGGATCTCATATATTGTCTGGCATTGTGTCCTTGG
TAACGCGCCTTCTTATCTTCTGGAGCTCTTTATTCTGACTTCGGCCTGCTCTGGTCGCCGAT
CTCTGCTCTCGGCCACCAAGGGGGACTTTCATGGTGCCACGTGCTCGCATTGCCACCAG
ACAGAAAATGGCTTTCTCGATTGTGGGTCCCTCTGTTTGGAATGATCTTCCCTCTGAACTTC
GTTCTCTGCCGCGGGACCTTTCCAGTTCTTTTTATAAACTCCTCAAGACTCTCCTCCTTGGC
CGAGCCTGGGCTGGGAGCGTCTCTGAGTAGTTACCTTGAAGGGGCGCTATATAAGTTGAT
ATATAGATAGATAGCTAGAAAGGATACATAGACGAAGCACAGGAGGAGATAACCTGGTCAA
GGTGAGCACTAATTCTGAGTTCATATCTAATAACCACACCCAGTACCCTCAAAGAACTAACT
CTCTCAGCCACGCTGATGATCGAGGAGGCTTGCGGCAAAACATGCCTAATTCGGTTCTTG

AAAACAATTAGTTCCTTAGTCTTAGATGGATTCAGTCTCAGATTAGTTTCCCTTGCCCATATAG
TTATATGCTTAAACTCCTCTGATGCTTCGGCAATGTGATTCGAGCCTACCAGTAAGTAGGAG
TCGTCTTGCGTTGTACTTCAGGAGCTGAAATTTTCTCGAGTGTGGGGGAGCTGAAAGGAAA
AACTCAGACCCTAACCGTCAGAACGCTCTTCTCAGGGTATATAACCCACCCCATCTACTG
CCCAAAATGATTGACTGCGTAGCGTTTAAGTCATTAGGGACTCTGTGATACCTCATAGTCGT
TGCGTCGGACCTCTAGCCAAAATACGAATGAAACGTATTAGTTAAGTTTGCCGACGACAGG
TATCTTCTTGTCGGCTCTAGACATCTTTCAACAGCTACCGAGGAATTTGAGCACATATCAGG
ACCATGAGAAACAACCTCTGCCTAAATCCCAACAAGACCAGGGAGCCTATTGTCGTCAGA
AAGGGCCAGAAATCAATCATCAGCCCCTCGCTCATCATACCTGGGACCTCACGTGTCAG
CAGCATCCGAGTACTCGGCGTCACCGTCATCTCTGATCTAGGGATGGAACAGCATCTTGA
CGAGGTTCATGCCACCTGCGCATCCTCCACGTATGCGCTCAGAGTTCTTCGTTCGCATGG
CCTTCCTCCTTTTGCCACCCATAAGGTTGCCAGAATGACCACGGTCTCATCTTTAATGTAAG
CCTCCCCGGCATGGTGGGGATTCACTCTGGCGCGCGACAGAGCAAGGGTTTAGCAGCT
GCTGAGGAGGATGAAACGCTGCGGTTTCTCCCACCTACATGCATGTACAGCCCCGACTG
CCGAGCAACTGGCGGCCTCACCCGACTCGCAACTCTTTAAGGCCATCATTCTGAATCCC
TACCCAGTCAGCACTGGAATTGGGCCCCAGTCATTGCCCGAATGGGCGTTTAGGGGAAG
ACCTGGACTTTGGCGGATTTGCCTTTGCCCTAAAGTTGCCCTAATTGCTGAGCAGAAATTT
GCCAACCATTAGGGCAAAGTTAGGGCAACCATCTTCTTTCCCTAATGGGCCCCAAAATTG
GTTTCCAATAGGGCAATAAATTGGGTTCCCAGCTGGGGCCAATGAGCATAACCAAGCTCT
CCCCTAATTGGGCTTGCCAAATGGGCCCCATTCATTGCCCTAAATATTTGCTGACTGGGTA
CCATGTGCTGAACAGTCGCCTTCCGGAGAAACGCAGCACCAATTACAGACTCAGACCTA
GGCTCATCAATTTAATCTACCACAAAAAGATGAAAACATTTTTTACCACTCCTCCTTTTTACAA
AAATTTACTAGGCCTAGTAGTGGTCATCCAAACTAAACTCGGTAATGATGCTGTTTCACCGC
CTTTATTCTCGTCGTTTTGTAATTTCAGTTGCGGCTGTGTCAGCTGGAGTCTTTTTCATTAAGC
GAATATTGTATTGTATTCATGAAGCTTCACACATAATTGCACAATACTTCAAGAGCTGAAATTT
CCTCGAGTTGGGAGGGGCTGAAGAAAAGGACTCAGACCTTAACCACTCAAAAGCGCTTT
ACATATTATATTACCAACCCCATCTATTACCCAAACTTATGGGCAGCATACCATTTAAGTTGTT
ATCGTCTCATTCTTATCCTCCAAGCTTCGCATATATATGTGTGGGAACAGCTCATCGTCAAG
GGGAAATGTTAGATTGATCAGTATTCCCTGTAGCATAGGTTGTCTAAAAGAGTACAATGCAGA
AAGATGTATGTCTACAGGACCTCGGTCCATGAGCAGTGTTAAAGAGATCAGGACCTCGGTG
GTCGCTATGTAGTATTAATGTAGTGGCTAGCTTCAGCTGCTTGAGAATGATTTTTTGCGTTGTG
TGATTGTGCTTCGGAAATCGTTAGCAGCAATCCACTGATCATTTGTGTGTGGTATAGTGTCGG
ACCGTTAAATTACGTCTTTACAATATAGAGGCCCATGAATATGTTAACTCATAGCCGGAGATT
GGCGCCGAGTGTGGGGAGGTGGACAGAAGCAAATTTTTGCGGACTAACAATTTGGAATAA
CCTTATTTAGGAAATAATTTCCATTTTCACGCCGACAATTTCGGATTACCTTTTCTTTCAATCTT
AAAAATGGGCCCTTATTGACGATAAAAACCTATATTTCTGAACAAATTCATTCATGACACCAT
TTTTACTCAAGTCGTACGTTCGCACACATCCAATCACACTACTTCTCAAAATATTAAGGGGA
CGAATACATGGGCCGTCCCCCCACCTCAAATCTTGGAGGGAACGACCCCTAATTCCCCC
TAAGTCTATGCCCAGGGATGAACTAAGTGAAATCTTCTTCTACTTTTCTCTCTTCCATCAGCG
TGCTGACAATGAGGCTCTTACAAGATTCCTCCAGACCTTTCTGTTTCTGGTTCCTTCACCAA
TCCTGGTGAAGTCGATGTTCCTCTCTTTCAGGTCTTCCCTGACATTGTCCATCCAGGTCTTC
CTCTGTCTCCCTCTGCTTCTCTTTCCCTCTACATGTCCATGTAAAGCTGCATTTGGTAATCTTT
TCTCCTCCATCCTTTCCACGTGTCCACACCACTGTAATTTCCTTTTCTTGATCTTTTGTACCA
CTGTTTTTTCAGCCCCAAGCTCTTCTCTTGTTTGTTCATTTCTGACTTTTTCTCTTCTACTTCTT
CTTATGATTCTCCTCAGCCTAAGTGAACTAAGTGAACTAAGTGAAAAGCAAAGCACCTATTA
GGGTGGCCTACATGTTTAGCATCTACATCATTGTAGATGAAGCACTTACTCACAAATCAGTG
TTTATAGGCAGTGGCATTCAAAATATGCATATGTGAGTACTATACATAACTTTCATTATATCCTA

TTCCATACTCGAGGTATGTAGCGTACACTGTATGCAAGTGCCTCACAGGCGTCAAGTACTA
AGTATACCCTATGAAAATTAAAAGCCAACTCCTCTTCAGATAGTAAGAGTAAAAATACGCTG
GCAGGTTGTCGAATTAACACAATAGTGCACAACAAAGCATACTGGAAGCACAGGTTCAAG
CCGTATTTCCATGAGAGCAAGACATCGACCAGGTCAAGAAGTTTGAAGGGGTATCAAATTT
GTACCATTTTTCATGCATTTTATATGCGAATCTTGGAGGGAAAGGAACAATACGTTAATGAAA
TAATAACTATGCTACCCATCATTTTGGATAGTAGATATAGGATCGGTATAATATACCCTGAAAA
GCGCTTTGGGATGGTTAGGGTCTGAGTTCTTCACTTCAGCTTTCCCACACTCGAGGACATT
TCAGCTTTTGAAGTACAGTGCAATTATATGTGAAATTTGATGAGGTATGACGGAGTCCCTTAT
GATTTAAACGTTATGCAACCAATGGTTTTGAGTAGCAGACGAGATGGGTAATATACCCTTAG
AAGCGCTTTCGGATGGTTAGGGTCTGAGTCCTTCATTTCAGCCCCCCAAACTCGAGGAAT
ATTCATCTGTTAAAGAACAATGCAATTATATGTGAAGTTTGATGAGGTATCACGGAGTCCATAA
TGACTTAAACGTTATGCAACCAATGATTTTGGGTAGCAGATGGGTTGGATAATATACCCTGTA
AAGCGCGTATGGATGGTTAGGGTCTGAGTTCTTTATTTCAACCCTCCCCCAAGTCGAGGAA
ACCCAGTTTTCGAAGTACAGTGCAATTATATGTGAGATTTGATGAGGTATCACGGAGTGCCT
ATTGATTTAAACGCTATGCTATCAATCAGTTTGGCTAGGCGATGGGGTAGGTAATTTATCCAG
GAAAGCGCTTTTGGATGGTTAGGGTCTGAGTTCTTCATTTCAGCCCCCAAACTCGAGGAAA
CTTCAGCTTTTGAAGTACAGTGCAATTATATGTGAAATGTGATGAGGTATGACGGAGTCCCTA
ATGACTTAAACGCTATGCAACCAAAGATTTTGGGTAGTAGATGGGGTGGGTAATATACCCTG
AAAATCGCTTTCGGATGGTTAGGGTCTGAGTCCTTCATTTCAAGTCCCCAAACTCGAGGAA
TATTCATCTGTTAAAGAACAATGCAATTATATGTGAAGTTTGATGAGGTATCACGCAGTCCATA
ATGACTTAAACGTTATGCGACCAATGATTTTGGGTAGCAGATGGGTTGGATAATATACCCTGT
AAAGGGCGTATGGATGGTTAGGGTCTGAGTTATTTTTTTCAACCCTCCCCCAAGTCGAGGA
AACCCAGCTTTCGAAGTACAGTGCAATTATATGTGAGATTTGATGAGGTATCACGGAGTCCC
TAATGAATTAAACGTTATGCAACCACTGATTTTGGGTACCGTATAGGTTGGATAATATACCCT
GTAAAGCGCGTATGGATGGTTAGGGTCTGAGTTCTTTTTTTGAACCCTCCCCCAAGTCGAG
GAAACCCAGCTTTCGAAGTACAGTGCAATTATATGTGAGATTTGATGAGGTATCACGGAGTG
CCTATTGATTTAAACGCTATGCTATCAATCAGTTTGGCTAGTCGATGGGGTATGTAATTTACC
CAGGAAAGCGCTTTTGGATGGTTAGGGTCTGAGTTCTTCATTTCAGCCCCCAAACTCGAG
GAAACTTCAGCTTTTGAAGTACAGTGCAATTATATGTGAAATGTGATGAGGTATGACGGAGTC
CCTAATGACTTAAACGCTATGCAACCAAAGATTTTGGGTAGTAGATGGGGTGGGTAATATAC
CCTGAAAATCGCTTTCGGATGGTTAGGGTCTGAGTCCTTCATTTCAAGTCCCCAAACTCGA
GGAATATTCATCTGTTAAAGAACAATGCAATTATATGTGAAGTTTGATGAGGTATCACGCAGT
CCATAATGACTTAAACGTTATGCGACCAATGATTTTGGGTAGCAGATGGGTTGGATAATATAC
CCTGTAAAGGGCGTATGGATGGTTAGGGTCTGAGTTATTTTTTTCAACCCTCCCCCAAGTC
GAGGAAACCCAGCTTTCGAAGTACAGTGCAATTATATGTGAGATTTGATGAGGTATCACGGA
GTCCCTAATGAATTAAACGTTATGCAACCACTGATTTTGGGTACCGTATAGGTTGGATAATATA
CCCTGTAAAGCGCGTATGGATGGTTAGGGTCTGAGTTCTTTTTTTGAACCCTCCCCCAAGT
CGAGGAAACCCAGCTTTCGAAGTACAGTGCAATTATATGTGAGATTTGATGAGGTATCACG
GAGTGCCTATTGATTTAAACGCTATGCTATCAATCAGTTTGGCTAGTCGATGGGGTATGTAAT
TTACCCAGGAAAGCGCTTTTGGATGGTTAGGGTCTGAGTTCTTCATTTCAGCCCCCAAACT
CGAGGAATCTTCAGCTTTTGAAGTACAGTGCAATTATATGTGAAATGTGATGAGGTATGACGG
AGTCCCTAATGACTTAAACGCTATGCAACCAAAGCTTTTGGGTAGTAGATGGGGTGGGTAA
TATACCCTGAAAATCGCTTTCGGATGGTTAGGGTCTGAGTCCTTCATTTCAGCCCCCCAAA
CTCGAGGAATATTCATCTGTTAAAGAACAATGCAATTATATGTGAAGTTTGATGAGGTATCAC
GGAGTCCATAATGACTTAAACGTTATGCAACCAATGATTTTGGGTAGCAGATGGGTTGGATA
ATATACCCTGTAAAGCGCGTATGGATGGTTAGGGTCTGAGTTCTTTATTTCAACCCTCCCCC
AAGTCGAGGAAACCCAGTTTTCGAAGTACAGTGCAATTATATGTGAGATTTGATGAGGTATC

ACGGAGTGCCTATTGATTTAAACGCTATGCTATCAATCAGTTTGGCTAGGCGATGGGGTAG
GTAATTTATCCAGGAAAGCGCTTTTGGATGGTTAGGGTCTCAGTTCTTCATTTCAGCCCCCC
AAACTCGAGGAAACTTCAGCTTTTGAAGTACAGTGCAATTATATGTGAAATTTGATGAGGTAT
GACGGAGTCCCTAATGACTTAAACGCTATGCAACCAAAGCTTTTGGGTAGTAGATGGGGTG
GGTAATATACCCTGAAAATCGCTTTCGGATGGTTAGGGTCTGAGTTCTTCATTTCAGCCCCC
CAAACTCGAGGAATATTCATCTGTTAAAGAACAATGCAATTATATGTGAAGTTTGATGAGGTAT
CACGGAGTCCATAATGACTTAAACGTTATGCAACCAATGATTTTGGGTAGCAGATGGGTTG
GATAATATACCCTGTAAAGCGCGTATGGATGGTTAGGGTCTGAGTTCTTTATTTCAACCCTCC
CCCAAGTCGAGGAAACCCAGTGTTCGAAGTACAGTGCAATTATATGTGAGATTTGATGAGG
TATCACGGAGTGCCTATTGATTTAAACGCTATGCTATCAATCAGTTTGGCTAGGCGATGGGG
TAGGTAATTTATCCAGGAAAGCGCTTTTGGATGGTTAGGGTCTCAGTTCTTCATTTCAGCCC
CCCAAACTCGAGGAAACTTCAGCTTTTGAAGTACAGTGCAATTATATGTGAAATTTGATGAG
GTATGACGGAGTCCCTAATGACTTAAACGCTATGCAACCAAAGCTTTTGGGTAGTAGATGG
GGTGGGTAATATACACTGAAAATCGCTTTCGGATGGTTAGGGTCTGAGTTCTTTATTTCAGCA
CCCCCAAAGTCGAGGAAACTTCAGCTTTTGAAGTACAGTGCAATTATATGTGAAGTTTGATG
AGGTATCACGGAGTCCCTAATGACCTAAACGTTATGCAACCAATGATTTTGGGTAGCGGAT
GGGTTGGGTAATATACCCTGTAAAGCGCGTATGGATGGTTAGGGTCTGAGTCCTTTTTTTCA
GCCCTCCCCCAAGTCGAGGAAACCCAGCTTTCGAAGTACAGTGCAATTATATGTGAGATTT
GATGAGGTATCACGGAGTGCCTATTGATTTAAACGCTATGCTATCAATCAGTTTGGCTAGTC
GATGGGGTAGGTAATTTATCCAGGAAAGCGCTTTTGGATGGTTAGGGTCTGAGTTCTTCATT
TCAGCCCCCCAAACTCGAGAAAACTTCAGCCTTTGAAGTACAGTGCAATTATATGTGAAATT
TGATGAGGTATGACGGAGTCCCTAATGACTTAAACGCTATGCAACCAAAGATTTTGGGTAGT
AGATGGGGTGGGTAATATACCCTGAAAATCGCTTTCGGATGGTTAGGGTCTGAGTCCTTCAT
TTCAGCCCCCTAAACTCGAGGAATATTCATCTGTTAAAGAACAATGCAATTATATGTGAAGTT
TGATGAGGTATCACGGAGTCCATAATGACTTAAACGTTATGCAACCAATGATTTTGGGTAGC
AGATGGGTTGGATAATATACCCTGTAAAGCGTTTTCGGAGGGTTAGGGTCTGAGTTTTTCATT
TCAGACCCCCCGCTTTAACGTAATTTCGGCTCTTGAGAATTATATGTGAAGTTTCACGAGGT
ATCACGGAGTTCCTAATGACTTAAACGCTATACTGCCCGGCATTTTGGGTAGTAGATGGGTT
GGGTAATATGCCCTATAAAGCGCTTTTAGATGGTTAAGGTCGGAGTTCTTCATTTCAGCCTCT
CAACTTAGAGCAATTATCTTGCATTCATATGGGAAGTTTGATGATGTATCACGGAGACGCTAA
TAACTTAAACGTTATAGCGCTAACTGTCTTGGGTACTACATAGGGTGGGTAACATACCCTATA
AAGTGATTTTTGATGGTTAGGGTCTGAGTTCTTAATCTCAGCACCCTAACTTTGAGCAGTTTT
CATGCATTTATATGCGAAGTCTGGAGTGGGTTGGACGAGACACTAACAACTTAAACGATAC
ACTGCCCATCATTTTGGGTAGTACATGGGGTGTGTACTATACCATGTAAAGCGATTTTGGATG
GTTAGGGTCTGAGGTCTTCATTTCAGCCCCCATCTTAGAAGCCCTTGGTTAACTGGGGAGC
TCACTCGCCCAGCGAATGTGAGGCTAGAAAACGAATCCCAATCTGGTCCTCAAGTTGACA
GACATTGTACACATCTGGGCTTGAAAAGTAAATTACTCTGCAGTACCTCAATATCGCACCTA
GGCCTGCATCATAAAATAATGAAATTTCAGGTACTCCCACGGTAACATAAGTCTACTTACGA
GTGTCGAGTGCGTCTGCCATGTTTACTTCTAGCACCGGGTTTCTAGCAGACTCCTCTAATCT
TCAAGTAAACACGTTTCTTCGCATCAAGCACAATCGATCTCTTGGCACCGCTGCATAGTCG
TTTAAATAGAAACTGGGCATCGTTCCTATTGGCCAGATGATTCCCGTCGCAATTCATTGTTAT
TGGTTGTCCTGATTACGCCTCTCAATAAAGCCATTCTATTGGTCTGCACGCGTTTTGCACAA
TTCCGTGTGAAATGTGAATAGCGAAATGAAACGTGTCGTGTGCAAAAGGTTCAGCACAACG
ACCTAGTTCAACACCCATTAGGATAGAAGTGTTTACAACGAGAGTATTTATCTAAGAATAGGT
ATAAGTATTTATACACTCTAGATTTTTACGCTGCGCGACATAGGCTAATTATAGACCCTAGTCA
TTTCCGGAGAGTGCAGAAGGAGCGTTGTAAGTATGGTAAACAGGGTTTGATCGGTTGAACT
GGTAGTGAACCGATACTGGGCAAATGCTAATAAAACAACTCGACAATTAAATTAATATTGGTT

GCGCATGTGTGACTTTATATTCTGTTACTACTAGTTGCGCATGTACGCAATGTAATGCATTTGA
GATAGTCAGATGAAAATAAAGGCTATACTTACTTTCTTATTCAAATTGCTTATTACGTACTACTG
AAATATACTGAAAACCGCAAATGTAAACCGACGGTGAATAAAGTTGAAATGACGAGAGGTA
GAGATGGGCTATGAATCCTTTCAATTTCGAAATTTTCATAAATTTCACGAAATTTATTAAACTTT
CAAGGGGCACTTTTTGAAAGTTTCATTGAAATATTGAAGTGGAATTATCTGTCAAAAATAAGC
AGTGGAAAATTGACAGAAATTGATGAAATCGTCTAATACTTTCTTATTTATTAAGTTAGTATATTG
GTTGTAGACATAACGTGAAATAAATTTCATCCTTATATGCTGTACCGGTACCTACCTTAGTCC
ATGCTTTTGTTACTAGTCGGCTTGATCACTGCTGCTCGGTTTTACATTGTAGTTGGACTAGGC
CTACCTCTGAGACCAGTGCTCGAATTACCATAGAGCCAGGGTAGGCCGCGGCCCCCCT
ACTTCTCTGAACGGCCCCCTTACTTGAAGAAAATCTAGGGGTTCAAAGGGGGCCGCCTTC
GGCCCCCCTACTTGTGCGGATCCAGGGGGGCCATGGCCCCCTTCGAAGTTGGCAGAA
AGAATAATGACAGCGACTGTGAATTTGTGGAAAAGTAAGGGTTTTGGTCCCCCTATTGATGT
CGGCTACGGATTTGGCCCCCCTACGGAAAAATACATATTAAAAAAGGGAAAAGAAAAAATT
GCGTATTAAAACCTGGAAAAGGTCGATGACTAAAAAAAAAAAGGTCGTCAGAAATTTTGGG
GAAGAGATGAACATTTTTTGGGGAAAAATCCAAAAAGATTTTCAGAAACGCCCGTAAAAAG
TCGTCAAAAAATTTCAGAAAAAATTTGTCCCCCCGACTCTGAAGTTATGGATCCGCCAGTG
ATCAGGAGCATAGCCTCATTAGCATTAACAATTGTATGGGCACCCTTACTTATTTTTTGGTAAT
TCGACCACTGTCTGAGACTGACCCTGACTGCCAGTCTGGATTATAATGCACTGCTATGTTGA
ACGTTACGAAATAGCAATTCCAATTAATTCAAATAAATTTACAGAGTTTGACATGAAAAAGTAA
AAATTCTGAATTGAAAATGAAAAAAAACTAAAAAACCTAGGTTTTTTAGGTTTAAAAAAAAACT
AAAATCCCAGGTTTTTTAATATGGGTCTCGACAGCCCTGATCGTTACCAAAGAGCTACATGA
ACACGCCTTTGCTCTACTAGCTGCTTAAAACTTCTTTTTCACCGTGGTTGGACTTGGGATCG
AGCACTTCTGAAATTCTGAATAGATTTCTTGAGAGGACGCTACATAAATTTTCTAAATGAATGA
AAGAATAAATGAATTCAGTTATAATATTACCTAATTTATGTAAGCTTATACGGATACTTAAAAACA
GCTACGTGGAATATGGAAGCCAAGAACCTGCCTAACAGTAGAACACTAACGACAACGTCA
TCAGACCCAGAACGATTACTCCTTAAAACATTTTCAGCCAAAGGCGGTGCCACACCAGGA
TGTGGCAGGTCAAACGACCTGGTAGAAATACCCTGGCAGTCAAAAGTGGTAATAATAAAAG
CACAGTAGAACGGTACTTTGACTGCCCTTGTTGATGCTACTAATGACCTGTCTCTATCTATGC
CCTGTCATGAAAAGCGACCTAGTGCCGCCAATGTTTCCAGTTTTAACAAGAGCGAAACAA
ACAGAAATTTCACATCATCACCATTTCACAGCATCACATTATTATAGCACATAGCATATCGGA
CCATTAATGGAAGTTCAGCAAGTGATATTTTTGTGAACTGTAATAGGAACTAAATTATTTTCAAA
ACCAGGGCACAGTTTAATGTGTAACTGATCACAATATCTGCAGTTGTAAAATGTAATTTGCAG
TTGTAAAAGGTGAATTGTGCAGTTGTAGTTGTAACTTTGTAAGTGTGAGGGTCGAAAACAGG
GGTGAAAAACGAGAAATTTCGAGGGAATGACGAAAAAAGTAAGGAAATTTAGGTCTGCAGT
TGTACTTTTGTATTTGTACAGAGACACGTTTGGGCATAGGCGAAAATTAAAAAATTCTGTCAA
ATGACCAAAAAAGAATCAAAAATTTACTTTTTTACAAATTCTTAGCGAACGACTAAAAAAAAG
ATCGTCAGAAATTTTTATGAGATATGAGAAATTTTTTCAGGTCAGAAATTTTTTTTCTGTATGCA
GTTGTAAACTCTGTTTAAAATATGCCCTGTTCAAAACTAAAATATCACTGAGTTCAGTGCCGT
CGTTTCACATAATTCACATAATTCCATCAGACCATTAACCACCATTTCACAAAATTCCCTTAG
CGCAAAATCTCAGCCAAGACCGTAGTTCACAGTGTTTATAGACATCGGAGGCAATTACAAT
ATGGCACAAAACAGCAGCAAAGCAATCCTTAAGCAGCTTTCATGATTAAAAAGTTTGAAAC
GACTGGTTCCCTTGAAATCGTTGAGACAAGGCCTACGCTGGTTCCTCTGACATCGTTGAGA
CAAGGCCTATGAAGGCAGTGGTGGATAAATGGAATACTCATCGAGAGACTTGACATCGTAC
GTTCCGATTGAATCCAAGAGCCACTCCTTTGCAACAATCGGCACGCTGAACTTCTTGTACA
GCTCTGAAGCAGGTTAAGAAATACAAATATAAAACGATATTTACATTGCTCAAGCTCAAGGTT
GCTTGTTAATTTTAAAGGATCATTTACCATAATAATTTGATTAGAGTTGTATTCATATTATTTTTTC
CAAGCATAACAGCCAATTTCTAGTGGTCAGTTCTCAGACTGTCAAAACCAGAGGTCGCCA

GTTCATTTCCCCGCAAGGGCAGAAATCTGGTTTGGGATTTCTGCCGCACCTGTGCCCTCT
AGTCAATTCGGCTATAAAATGAGCAGGATTAACGCCCAAGGTCAACGTTAACGCCCAAAC
GGACCCCAAATATGTCCAAAACGTCAAAATTTCTCGATGTGATCATTGTTAGCAATATCCAA
AATTCTAGGTCAGGATGTAAGTATACGTCCTCTTTTTGGACATTGAGGTAAATAAATGATAATG
AGTCGACTGAGCACAACCTTTGCAAAAGAAAAAAGTTAAGGATATCCAAAAAGAAAAAGAA
AAAAGGAAAAGATAAAAAAAGAAATAAAATTCCAAGTAAGTTGAGGCAAATGCTTCGTGGA
CTTAAGCCATTGAAATTTCTCTAAGAGTAGGAAAATCTAGAAACAGAAACCGAGACATCAC
GTAGGGCGAGTGCTCACACACCCAAGGCTCAGTCAAAACACATAATCCTCATAATCGACA
GATTAGTTTAGATAAGTAAGTAAGATATTCTTTAATGTCCAATTGCTAACTGCACTTTGGTCTAA
AGACATCAAATTGTATTGTATGTAGATATTTATGTAACGTTTCTCACGGCATAAGCCAAACAGA
AATGCTTTCACAAAATTTCAGCTCCAGGAAAAAGGTAAGAATTTAGGTGAGAGAGAGAGAG
AGACAGAGACAGAGAGAGACAAGGAAAGGGGAGCAGAGAGAATAGACGTGCAAAGAGA
AGGAGGGAGGCAATTTCAGGGTAACGGGCCAACCGACACAAAGGACCTGGTTTGGGGG
ACAAAATGATATTGGACAATATGGTAGCAATTCAATTGAATTGAATTTATATTTAGTAACCACAA
GTTACAAATAAATGATAAGCCTAAGTTTGTGTAAATGGAAGCGGGATTAATGAAAAAATCATAT
TATCAGTGGGAGCTGGATTACTTGATTAATTCAGGATTCTACCCGTATCATTTTGTCCGCTAC
CATTTTGTCCTAGAACCGGTTTGGGTCATAACGACGATTAACCATCCTAACATGAGGAAGAA
AAAGATAAAATAAACAGTTAAAACGCTTACAATACATACATCGCCCTAACGATATAATTATATG
CCTGATGGAGATGACACCCTTACTCTTGAAGTGCTGTAGACCAGTATCACTAGAGCTTCCTT
CGACGATGATGACCTGAGCTCTGTCAACATCAAATGAGAACATTGCGGGATCCTGAAGAC
TGCTTGCACCACACGCTTCAGCAAGTCCTCTTAATTCATCTGAACAACAAAAACACCAAAG
AAATGCAATCACAGAAAGATTCCCATGCACACAAAAGTCTATAAACCGAGAAAAAAAAATG
TAGCTAAACGTGTAAAAAGTATAAACAAGTAAGAAACAGGAAATGCAGTTTAAATACCTTAAT
CAATCAATCATGCAACCACTACATGAATATAAAGTTCTTAAGTTTTTCTGTTCACTTATTGGTAT
CAATATATATGTGACACAGGGGTCCAAAACCCGTCACCTGTCGTTTTTGGCTTTTTTATTGAA
TCATTTTCTACACTGCATCTGCTTCAAAATGCTTTTTTCAGAATGAAAATGTCTTGTTTCTAACT
GAAGTTATACTCGCCTGATTAGAACAATCAAAAAGTGAGAAATATCATATAAAAAGTTTCAGAT
TTTTGTGTCATCGTAATCCTATGCAATCGTCAATATTCTGGGCAATAAAGGGCTTCATTCTGTG
TCAAATAAAACAAACCGCATTGAAATCGGGTTACAACATTTCAAATTATAACAAATTTACGAC
ACCAGCTGAAATGAAGAATTTCAGACTTGAAAACCCCTCAGTCCTATTTTTGCTTGTATCACT
GTAAATAATTCCTGACAAACGACGGGTTTTGGGCCCTTGCGTCACATATATAAATTTGCTGTA
TATTTATTTTCACTAGCCTGTCACGCTGAGAGCGTCTCTGAACAGCCTTCTGTGAAAGGCG
CTTTACAAATAGCCATATAACATAACAATAATGGGTGGCTGCCTTTTTTTTCTCCGTAAAAATT
GCTTCTTTAACGCAAGGAAAATGGCAACTAATTCATTGTGTGAACATGACATAAACAGAGGA
GTTAGACCAAAGCCACACCTATACCTACCACACACAACAAAAAGTAGTCAGAAAAGCATC
AAACATTTACGCACTACCTGTGGTATTCTTCGGTGGCTAACTATGACGATCATAGCAAAGAA
TCTTACCACGGGTTAAATCGTCGAAGGCACCAAGGCAACATATTTCAAATTTTCGGAGTAG
CTGAACACCTGAAAGTCGAGCATTGGTCGGACCGAAGTGATCCTCGTTGACAGCCACATC
TCCAGTGATTTCATATTTGTCCTTGGAAAATAAATAGTCCCATCAAACCCTGCAGTTAACCAG
TGATGATGATGATGCCATCTATACATTCAATCATTCATTCACCGATTCGTTTGTCAATCCATTC
ATCCGTCCGTCCAACCTTCAGTCGATCCATCATCCACCATCATTGACTGTTAGGTAGGAAA
ATTTAAATAAGAATCATTCTAACAGGTTTATTATTACCAGAAATATGCACAACATGATAGTTATC
ATTTATTAGGTCCACCTTAATGTACAACCCACAATGCAACAATGCAATATGGAAATACCAGA
CTGAGCAGGACACCACTTGCTGAGACCATGCCAGCTGTAAGTAAAGTATTTCACAATGATA
CCACCTAGTGTGGCATACGAGGTGGACATGTTCAACAAGAACTCGGCCGCACTTCTACCA
GAGTGGGCAAAAGACCGCGATTCCAGCCTCAAAACTTGCCTTTATTCTCGGGGACTTTCA
CAATGGGAGCAAACTTGAATGATACACTATGAGAAGGGCTTTATAACTCTTTGAATATAATAC

AACTGGAGTAAGGCTTCCCTGAAGAAATATGGAAATACCAGGGTATCTACTATCTACAATCT
TATCCACCAAAACATCACCATAAACTGATATTGAGACACAGTTCATAACTCTTTGAATATAATA
CGATACAACTGGAGTAAGGCTACCCTACAGCAAAATGGAAATACCAGGGTAACTACTATTT
ACAATCTTATTCACCAACTTGTCAGTATAAGCTGATACAGACGCAGTTCCTGGCGTGGCCA
ACATTTGGAGTAAGGGCAGCTACCGGAAAGACAGTCACTACAATACAACCGGAGTAAGC
CTTCCATGCAGAAAAATGGAAATGCCAGGGTATCTACAATCTTATCCTCCTATACATCGCTAT
AATAATGATGATGACAATTTATATGGCACTGTAACACAGCCAAACCGCTACAAGGATGCCTC
ACAAAGAACTAAACAGATAGATCACGGTTCATGGTGTGGCCAACGTTTGGATCAAAGGCA
GCTACAGTAAAGACAGTCACTAGCAAGAAAGGCAGAGATGCTACTAACCTCCGATTGTAG
GCAACCCATCCTGATGGAGTCGAGGATCCATTCGATGCTTATGACCCATGACCTGTGAGC
AATTCCCTGGAAGTACTTCAACGTTCGCTGACATCGTCGACTGCCTTCCTTCACTGGAAAA
GGAAATGAATTATCATTTTATTATAATGTGAAAAAGTTTCTGATGCCCACATAGAGATGTTTACT
TCAGATCCAGGGATGTAGCCAGAAATGTGTTTGGGGGTTTTGATTTGGGGGTTTTCATTGGC
TTGGGGTGTATTTTTGTGTATTTTAAGGTTTTTATAACAGGAATGAGTTCTTCTATGGAGTTTTTA
CCATGAAAAACCACAATTTGATCCAGAAAAACACAAAAATTTGATCCAGGAAAAACACAAG
TTTGATCCACACCCCTCCTAAATACACTCATGCTCAGATCCAGTCAGTTTGCTGCAAGGATA
AGGCAATCAGATATCAAAATGTTGGAAAGCCTGTGTAACATGGAATATTGCACCGTGAACC
AAATGTGAAAGTATTTATATGTGCACACCTGTTTGTATGCACAAAACAAAACATTTAGAGTTCG
AATTAGGGGCCATCCATTTATGACGTCCACGCAGAGGGGGATCAGGCTCAGGTGGACGC
ATGTGGACTGGGATAGAGGTCAAGCCCCATGTAGACGTCCACACAGAAAATTAAAATTAGA
GTCCACTGACGTCATCCTGTCTTCTTCTCCTGCAAAGAAGTTGGTGTCTTTTTTACCAGAATT
TCGTCTTTCGACGGAATAAAGAGTGGCAATTTTTCGGCGATATAAATTAGTAACAAAAATTAC
TAATAGTACAGTTTAAATAAATTACAATCACATAGTCGAATTTCAGCTTTAGATCCGGAAGGTC
ATTGTACTAGCTGTTTCAGGAGAGCGATGTGTATTTCATGTCGACCGCATGTGGACGTCCAC
AAGGGGGAGGGGGTCCGGCTCATGTGGACAGAGGGAGAGGGGTCAAAAACCTGATTTT
TTGGGACGTCATAAATGGATGGCCCTTATAACAAAATTAAAACCACCTTAAACTAAATAGCA
AAAATATAAGTTCAAATGCCTTAAGTTTAATCAGCTTTCAACAGAATCCACAAAGGTGTGGTA
ACCATCCAAGAAAAGAGCACCACCTTTGAATTCAGTCTTCAAAGGTTAAAGGTCACTTACC
AGGTTTAACCAGAACATGGGTTGTGCCGTTAGTAAACTTCTGGAGAAATCTACAGCCAAGTT
TTCGGACAAACTCTTCAACAAGCGGCTGCAATGAAAGAACAATAAGATGCACAACAATTGA
CACGCTAAAGTGGCCTTTCGTGGAATAAACATCATTCTTCAACACACAAAACATCCACAGC
ACGCACTGTCGAACATCACTCCACCACAATCATTAAAACTGAAAGTTTAAATCACCAATATT
TTATCACACTAAGTTGGTGCTTGTTTTTATTTCTCAAAGTCTCTACACAGTAGTCTATGTAGAGA
CTTGAGAAAAGATATTCTGCGAGCAAGCTGGTGCCCAGCACTTGGCTTACGAGTGGTAGTT
TATAATTCTAAAGAATTCTTGTTACTGTTGCCTTGCCCTTCAGTAATTCTTACAACTACTGAAGT
GCAAGGCAACAGAGAAGGGTGCCAGTACAAGACTTTTGGGCGCTGGCCGTTTGGGCGA
CAGGTTGATTAAAATCGTCATATTCAAAGCCGAAGCGGGATTATTGAAAAAAATCATAGCATA
AATGAACTAGGAGTGGAATGCATGCAAAGAATTCTTATTAATGACTGAAGTAGAATTGGATTC
ATAAAGAGTATGAATTTGTGCTTGATATGTGTTCTATTGATGAAAATTATGACCCCTCAAATACT
ATTAGGATTGATTAAAGTATGCTTTGATAAGACAGAAGCTGGAATGATAACTGCCAAAGTTTC
TGGTGCCGGCGCCCAAACGTCCTGCTTTGGACAGAGAAAACTTCAGATGCACGTCTATG
CATACATTCCGCCTATACAATACAATTAAAATTTATAACGCACGCAAGGTCACACCAAAATG
TGAATCTGAGGCGCGGAGGCCTATGTTTAATGCTGATAAGGTCGTATGAGTGACAGTGGTA
ACACAGGCATAAAGCACTGTAATTCAATTTGTGTAGAACAAAATACCATTTCAGATGCGCGG
TTAATGCCAGATGCCACAAGAATCCAGCTTTTCTTCTGAGGGAAAGGACTTCCGTGACTCT
TCTCCTTAGAGGGCTGCGAAGAACACTCCCTCAGTACGGCGGAATGTGCATAGATGGCG
CTTTTACTTCTTTTACCCGTTGTAGCAACAGACGAGGAACCTCCTCTGTTTGCCGGAGTTCT

CTGGTATGCTTCAATCCCGTCATCTGCCAGTTTCACATTTTCGACGACCTTCCTTGAACAAT
TTCCTTGTCGCTCAACTGATTTTAGACTAGTCCCGTTGCTCGGATTAAAGCGTTCGTTAACG
TTTCGATTATTTTCGTCTCTGTTATCTAGATCATGACTCAACCTTAATCGTTTCGCTTCACTTGC
ACTTTCCAGATCAGCACAACTATTCTTTTCCTTTTTACGCGTAACAACAGGAGGCGGAGGA
GGAGTGGGAGACCGTGGGTTTTCCTCTCTCTCTACATCTCTCTCCAGCCCACGGTGAGAA
AATCGAGTGCGCGGAGTCTTATCCTTAGTTGGTGTCGGCGTCTGTGGAATAGCCACAATGT
CGGACATCTGGCTGCCAAAGAGATCCGCTTCACTGCTCGACTCGTCGACATCCTCGACA
GTAGACTGACACTGCCGCAGTTCCGTCTTTAAGTCATCGACGACGCCCCCTACTGTCGTC
CGATCACTACTGACAGCGTTAGAAGAATCCAGTACAACTATTACACCATCGCTTTCTGGTTT
TTCCCCAATGATACCTTCTTCAAAGGAGTCACATGTATTGGGGTTGAGTCCATTTTGTCCAG
GGGACGCTGGTAGGTCGGGCTTCTTTGCTTCTTGTTCAGAATCTCTCATTTCCGAGGTGAC
GACAGGAACGTCGATTTTATCGGTCAACGTCTTGACTTCGTTGGTTAGTTCAGCCTGAAGTC
TTGCAATCTCTTCCTCCATTGTTTTCACTTCGACTTCCAGATCGTGACGTTGCTAAAACAAAA
CCAGAAGGTCAGAAAAACCTTACAGCTTTAAACTAAGCGCTGTGTCCAGCGTTTCCATTGG
AGTCCAAAAAGGAAGCATACTAGCATCCTGACCTAGAATTTCGGACGTAAAGTTAATTGCTT
GCAGTGATTACAGACGTAAATAGGAAAATTAATAGAGTTCACAGAGTTATGGTTAACCCAAA
ACCCTTGAGTCCAAGTCAAGATGATGCAGGTTTACGTTCTGGTCAAGTCCAAAGTCAAACA
TATCAAATTTTTTCATTCATTCTCAGAATCTACATATCACATCTTCAAGAAACCTACTCAGAGG
GCGCTCCCACTCCATCCACAGCTGAAGAATTCTGGTTTGAGGAAATCATAAAACGCGGGT
GAGTTTGGTCGAGGCAGCAAGCACAGGTCACAAGAGAAACAAAATCAAAGAACTGTCAAT
TTTGACAGAAATAACGACAATTGGGAGAGAAAAATCCACATGTCCCACTAGCAGGATCACT
CCTTCAGTGATTAAATCTTGGAAAGCTGGTCAGATTATATGCAAGATAATGGCTGAAGCTTAG
CTAGTTAATACTAATAATGATAATTTATATGGTGCCATAATACAGCCAGACCACTTCATAGGCT
CCTCACAAGCAACTAGCAAGCGGGATAAGCCTTAACAAACAGATAAGTTTGAGGTTAGATT
TAAGGAAGTGTATTGGGTCCGCTAAATAGATCGCAGGCGGAAAACGGTTCCATTAAACTAG
TATAAATTGTAAAGAAAGGAAATAGTTAGCGCAGCACTAGAGATGTTGCACTGAAGTATTTCA
AATGAAATATATCCTTTATGACGGTATTTTTTAAATATTACATTGGCCAAAAAATACTTTGAAATA
TTTCAAGTATAGAAAACATTATTTTGTGCCATTTCAGACATGTATGTTAGGTATAGTAACCATTC
CCTGCCGCATGTTTCTGTACAATATCTGCCGTATCTCTTCTCTTTAGCCGTGCAAGGGTCGG
GAGCGCTTCTGAGCAGTAACCTTGAAGGGGCGCTACATAAATCTCCGTAATGAATGAATGA
ATATGTTTTGTCGACTAGACCAACCCTAACGTAATGGTTTGAGGAGAGTGAAGAAAGAAGTG
GAGGTGTTGTTCCATCCTAGCACCACATAATTATATGTATTTAGCATCACTAAATATGTGCAGT
GGCCCCTTGGGCAGAAAGAAACGAGTCGGGCATACTGGCGTCGCTTTTGAAATATCTTAT
TAAACACGTAAATATTACCGAAATATTTCTCCGTCAAAAAATACATCCTTATGCAGCACACAA
TGTAGAGCATAAAGGTAAACAACATAAATAAAAGGATGCAGAAGCAATCTATCTAGAGTATA
GACAGACTACCAGTAAGGGAGCACCTTGCTTGAGTTATTTGCATTTCACTTTCACATACTTG
AGTTGTGAGCATTTCACTTTCACTTGACAAGTTGCCTGATGACGTCAGGTTTATTCGACCAA
CTGGCGACATGACCTCATCATCACTATCATCACTCTCGTTTTTCTCAGCAACTGCAAAAATA
GAATCAACTTTGTAAAATAGAAACACCGTGCTAGACGAACGTCTGAAGTAAGGCATTTAATC
GACATAAACGAAAACACATTAAAAGCAGAGCAAAATGTTTCAGGACGTTTATAAACAGTGC
AACTGGAATAATCTCACAACAACCGTACCTAACTCTCCTTGCATCTCTACATTTAAATGGAAA
CTTCACAAACACTTACATTCATTCATTCCGGATATTTCTATAGTGCATCTTCAAGTTCACTACT
ACCCAGAGGCACTCCCGACTACAGCCTTGCTACTGTGTCGGAGTTAACAAGCCGAAACG
CTACAGGCAACTACGAGTGAAGGACTTGCCCAAGGTCCCTAAGTGGCAGCTAGAGTGGG
ATTCGAACCTGTGACCTTCCGAACGCAAGGCACTGAACCCGTTGCTTCCTTCAATCAACA
AATGTCTTGACTTCGACCGTTATGATATGCTGTGTAACAATAAAGCCAATAAGAATCATGCTTA
TGATTATAAATATAAATAAATAAATAAAAATAAATAAATATAAATAAGAATCATGCTTATGATTATAA

ATATAAATAAATAAATAAAAATAAATAAAAATATAAATAAGAATCTATGCTTATGATTATAAATATG
AACTGGTACACAGCTTGGCCTCAGCCTAAGATCTTACCGTTAACAAAGGCCATTGGTACTT
CAATTTTCTTGGAATCTTGTATAACACCTTTAGGTGATCGGGCATCAATTTCTTCAGTCTGACA
TGTCGGCTCACAAGTGGAAGATAGTTGATGAGCAGGGTCTTCTAGCGACTTTTGGATATCAA
TTTCTTCAGTCTGACATGTCGGCTCACAAGTGGAAGATAGTTGATGAGCAGGGTCTTCTAGC
GACTTTTGGATATCAGAAGATTCTCCACCTAAGTTCTTCTGCTCATTTACTGTTATCAAATGTA
ACCCTAAGCTGGAATCAGTCTCTGGAACTGATTCCATAACCAAAACCAGGTCACTCTGTAG
TTCTGACCGATTTCCTAAATCCGCTCCCACCTTCGCTGTTTCTGCGTCAACCCCATCAATTT
TGTTATTAGACGAAATATCAAGCTGAGATTTTGGCTTGCTATCCACTTTCAACTCATCTGATCT
TAAACCTATCAATATTTCTGATTCGACGAAATGCTCCGATTCAACATCTGGTAGTGACTCACG
CCCAGTGTCTAGACACACACCTCCAGTGTTTGGACACACGCTTTGCTGGCTGTTGACAGA
ATGCTGAACTGTCACAGTAGAGTCCTTGAGATGCAAATTTGCACGGTTCTCATTCTGAGCCA
TACACAACGGTGGTGGTGGTGGCTGTAGGAGACTGTAACTATTGCTGTGCACGGACGACG
ACGAATCATATGTTCCAACCGCAATGCATTCCGACTTTCTATCCGATTGTGTTTCTTCCTTGG
CACAACTAGATTTGATGAATGTCTTGGCACATTCACTTCCATCCTCAACGCACTCAGAGTAA
CAACGATCTCGAAGTGCTGCGCCATTACCAAGTGCAACTACTTCGTCATAATTAACCGCTT
CCACTGTTACTCTATCCTTGATTCCATTCTCTTCTGTCACCCCAGTGTTTCCACTTTCAGTCT
CGTGTACCAATTTCGCTCCAGTCTTCACTACCGCAGGCACACGTTGGCTGTTAGCAACCG
CAGAGGTAGCCACGCCGACAGCACAACCATTTCTTTTCGCTGTCTGACCAGATTTCCAAA
CAGCCTCGTGCATCTCAATTTCTTGTGTAGACAGATCGTCGTCTGCACGATCGTCTGCTTTG
CCGAGCATTCCAAGAACCGCCTCGCCAATGACCGCTTCAAAGTCAATTGAATCTGTCTGC
AAACTACTGCAGTTGTTCATCTTCTGTTCTGTTCTCGGAGGACTCGCTTCAAGTGAAAACGT
CTTTCTCTTTCGTTTGGCTTTTGGCTTAAGCAGAAAGTCAGCTGGTGCAGCCATTGAATCTC
TGGGCTGACTCTCCTCTGCAGAAATGTTGCCTGTTGTTCTAGAACCTTCTGACAACGTACTT
TCACTGACGCAGAGCATTTTTTCACCTGCCAGAGAAGGACTGGCTCCAAGTGAAAGTGTC
TTTCTCTTTTCCTTTGGCTTATGCACGACATCCTCTGGCACAACCGCTGCATTTTTACATTCA
TTCTCTCGTATTGGAACACCGTCAACTTCTCCGGAATTTTCCAGCACTGCCACCATTTGAG
GAAGACCCAATGGAGTGGCTCCGCTTTCCGTGTAGACGACATCGCTTCCAAGATTTTCCG
AGAACAGCCGGTCAGACTTCAAAGTCTCTTTGACAATTTTACATTCAAATGTCTCATCTATATT
CTCAGTGAAAGTCTGTAACATGGGGTATGTCTCACTTTTACCTTGGACCGCTTCACCCACTT
CCTCAAAGCACTGGTTCCCTTCCACTTCGTTCTCGACAGAGTCTGGAACACATGAGATGG
AAGACTGCGATGAAGATTCAACAGCTACGGCAACTGTCGATGATTGTTCTTCCAATCTATCA
ATCTTCTTCTGCTCTTTCATCGGTTGGGTTCCCAATCCACCAGCACTTTCCTTTCCTCTTGGT
TGTTCTCCCCCTCCTCCACTTTCCTCTCCTCTTGATTGTTCTCCCCCTCCTCCACTTTCCTC
TCCTCTTGGTTGTTCTTCCCCTTCTCCACTTTCCTCTCCTCTTGGTTGTTCTTCCCTTTCTCCA
GCAGATATTTCCTCTTCTAAACACTGCACCTGATCATCATCAATTAAAGTAATACTTTCACTGT
CCATAGGACTCTTGTTCTGAATCAGACCTAAAAAATAGATAACAAAACAATTAAAAGGCACA
AGAATAATAAAAAGAACTTCACTTGTATTCACCTCAGAATCTCTGAGTGATGCTTTATAAATTC
AATTACAACTACAAGCACCCAGACAAGATTTTACAACACCATTCAAGTTTATCAGGCAACTG
AATTTGACAACGGCTTATCAGCCACGCACAGCTGTGTTCAACAGTTGCATTAATTTTTGACT
GTATTTTTGACATTTATTCTCATTTGTAAACGAGTAGTGGTCTGACAACCATTCTTTCTGCATCA
GACAAATATATACAAACAACAATTCAAGCAATCTTATCTTAAATTCACACTGAAAATTCAAATT
TAGCTACAAAGTCTTACAAGAAATAAAACATAGAGTTAACTTTAAACAACACCCGAAGTTAG
CTTACTACAATAATTCTGAAACTTGTTACACACAAAAAGGGGTTTACCTGAATCCGAAGTTGA
AGCTGCACAGCGGTTCGATGTCGGCACATCTTGAAGTCTATTGAACTCTTCGATGGCTTTCT
TTCTTTCGTCTCTTGCAGACACTTTTCTCTGGTATTTCTTCTGCCATTTAGTCTCAACAGTTTG
AAAAGGACTCCTTTTGCGGGAACTCGATCTAGTTTTCGGAAGATTTCTTGAAGCGAAACTGT

CGTCATTTGTTTCTGGAACTAGTTCGGAGCATTTTCTTCGCTTCGAAGGCTTGTCGGAACAA
AGAACCGTGACATTTCCATCCGGTTCAAGCATACAAACGGCGTTCTCTTCCACACCAGTC
TGGTTACTTGAATGACCATAATTGTCAGCCTCACAAACCTTGGATGTCCCTGAATTTGAACC
GCTATCTGCTCGGAAACTGACATCGGTAGTTGGGGAAGTTCTTGTTCTTTCTAAATAAGATTC
TATGCTTTTGTCAACATTGGGACTAGGAGCTGTGTTTTTGGGTGCTGTCTCGTCTGCAACCA
TCGCCTTACTTGGCATTAGCTTGTTTGGTATGACCTCCATTGTTGAAACCTCTTTTGGTAATG
CCTTAATTGGTATTGCCCTATTTCGTATTTTGCTGGGTATGGTCTCGCTTGGTTCGATCTCGTT
TACCTTCGGCGTTGGTCTGTTTGGAGCAGTGACCTTCGGTACGGTCTTCTCTGGTGCTGCA
TGATTTGGTGATATCTCATTTGGTGACGACTCGTTTGGTGCCATCTTATGTGTAACCATCCTGT
TTTTCATCACCTCACCAGCTATCAAAGGACTTACCAGAACTTCGGTATTTGGGGAAGTTCCT
GTTCTTTCTAAATAAGATCCTATATTTTTTTCAGTGTTGGGATTTGGAGCCATCTGCTCTGGTG
CTGTCTCGTCTGGAACCATCTCACTTGGAACTGCCTTACTTGGCATTAGCTTGTTTGGTATA
GCCTCCATTTTGAAAGCCTCTTTTGTTGATGCCCTAATTGGTTTTGCCCTATTTGGTATTTTGT
TGGGTACAGTCTCACTTTGTTCTATCTCGTTTACCTTTGGCTTCGGTCTGTTGGGAGCTCTGA
TCTTTGGTATAGCCTTCTCTGGTGCTGTGTGATTTAGTGATGTCTCATTTGGTGCTGTGTGATTT
AATGCTGTGTGATTTGGTGATGTGTGACTTGGTGACGTCTCATTTGATGTTGTGTGATTTGGTG
CTGTGTGATTTGGTGATGTCTCATTTGGTGTCATCTCGTGTGGAACCATTTTGCTTTTCGTGGT
CTCACCAGCTGTCAATGGAAGTACCAGAACTTCCTCATCGTCCTTCTGCATCAAAATCGAA
CCGCTTTTCTGCATATGATCTCGATTTTCTGCCAATGCTAACGGCAAAATCTTACTATCAGCA
GACACTAGATTTCTTTTTGCTCTGCTATGTTCCATGCGCTTTTGATTCCTCATGTTTTCCGCAT
CACTGCTTCTGAACTGTTGACTAAGCACAAGATTATAGTTTTCCGCGTCTAATAACTTGTCAC
TTATAGCTTGAATTTCGAAACAATGTTCGTCGGCCGTTGGTTCTTTCGCTTCCGCTGCGTGT
TTCTTATCGGCAGGTAGACGAAGCTCTGAACGTTCACGAAGGGACATTCTCTTTCTTCTCTT
TGGTAACACATTAAAATCCTCAAACATGTGAATGCCATCTACATCCTTCACCTGCTTCTGCA
CAGACTTCTTTCTTTTCGGTGTTGGCTGGCTGCCAATGAAAGCGAAGGGATCATCCACACT
TTGATCAACTCTTGATTTCTCTTCGACTGGTCTTACCTCTTTCGACAAGGCACGGCTCTTGA
AGATTTTACGATTTTCGGTAATTAGTCTCATTTCCATGTCAACGCCAACTTCTACTTGATAGCC
ACCGGTTATAAGACCGCGGTTATCATCATCCTCTTCGTCTGATGCTTCAACTTCAGTGGCAT
TGCACTTGACACTTGCATTGCTCTCAACTTCAACAAAGTCATTTCTACGACAACTGGTCTGC
TTGGAAACGCCACTTGTAACTGAACTCATCTTATTAGTGGTCGATAGTATTTCAATACTCTCC
CGACATTTTACACCAGCACTGTTGCTGCCAGCGCCACTGCTTGTTTGCTCAACTTCCGCAT
CTTCTTCATCCTGTCTACTGTTCAGTTCTTCATCCTCTAGTCTAAATCGAACGCTTCTTCGTTT
TCTGACTTGGTGAGTGGTAGACGTTGCTCCCTGATGTACATAAACTACCGAAGGTTTTCCTG
GAGATGGAGTAGGTACCCCTGCTTCCTTTGCACTTTCCCTGCACTCTTTAGTAACCTCCTC
CTCCTCAGCTAGATCGAGCTTAAAAGCATCAACCAACTGCTTGACTTTTCCAACAAAATTCT
TCCATTCGACTAAATCCTGAAGACTCCTAAAATATAACCAAATCAGTGAATAAGTTAATGATG
ACTTACACTAAAGCCACAGATGTAAGCAAGCTGTAAATCTTAAAAGCCCATTATACTTCCATA
TTTCCATAAAAACAAGCAAAAGAAGAACAAAATATACACGTGTAGAAAAAAAGTCAAGACTA
AACAGGTTAATAAATTAGGGACAACCTACACTACATCCAGACGGATTCCCTAGACAAGCAA
GTTGAAAACTGTGCAAACCATGGGTAAGACTTACAAATTCCGTTAAGACAAGCAAAAGGAG
CACAACATACACACACAGATAAAAATAAGCCATAATAAAACATGCTCCGGACAGGGTATCC
ATTAAAGCCCAAGTAGACGTTTTTGCATCTGTAAATGCGTCCAAAACGTCCAAATGTTTCAG
TGTCATTGCCGTTAGCAATGGGCTACATCAAAAATTCTAGGCCAGTATGTTACATAGACGTC
CTCTTTGGACCCTAACGAAATAACTAGCTGTGGCAAACCTTTTCGTAACAGGAGCCTTGCA
TAGAGGACACGGAGTTGAAGGCTTTCGACCAATGACCTTTAAGATGCAGTGACTGAAGAA
AAACAAACCAATATTATAACTACATTAAGTATACAATTATAAACAAACTCCCGCCAGCATTGC
GACAACTATCTGACCCATCCTACGAACTCACCAAGACCTCACCCCTTGCTATCTCTCCAC

AGCTCTTTTACTCCAAACTGAAAACACTTCTCTTCAATAAATCCTATCCTGATTTATCCTCTTC
TTCCTACTTCCCTCCCCGTCTCAACTCCAAACACCAGCCATCATAGCCGTCTGACTCTCT
GGATCTGACCTGCTGCCTATCGATTTTGTTTTGGACAAGCGCCTGTGAATAAGCTGGATCC
CGCGACTTTGCTTTTGTGGGCGCTGTAGAAATATCGAGTTTACGATTACGATTATAAGATATA
CTGGGATCAATTAGGAATTCACATAAAACTAAACTGTAAGCACATTAACAGCATGAGTTCTTC
AAGCATCCTCTAGGATAGCATGGTATGTCTAGAGACATGGTGGCTGCACCACAATCTAGGT
AGGAACTGTGAAAAATGCCAGAAGTCTGCAGATGTGTCAAGTCAATTCAAGGTATTTCTACT
CAGGCCTAAATAATGTTAGGGTTCACTACTGCACGTCTACTGCCTAATGTCTTTCAGCTCTAT
CATTATGCATAAAACCGAGCGTACAATTTAAGCAAAAAACGTTATTCAAACTCACCGACAGA
ATTTGTGATCGCATTTTGTTGACACTGGATTAAGCATCAATTCAAGACTGAAAAATTAGAAAC
GGAAGCGAATAGAGTACACTGAAATCAATCAATCAATCAAAATTTATATAGCGCCCCTTCAA
GATACCTATTCAGAGGCGGTCCCGACCCAGGCAAAGCGGAAAAGAACAGTCTTTAGAAG
GTGGTAGAATTGAGAACAGGCAGCATTTGGAAGGTGCCTTAGATCTACTGGAAGTCCATTC
CAGGTTTTTGGACCAAACACAGAAAATGAACGGGTCTGCTCTTTCGCACCCACCAAATTA
CCGTGAGCAGAGGACCGGAGTGTACGACGGCCTGCACATGAGGAGAGAGGGTGGCAG
AGCTCGCAGAGGTCGAAAACAATTAGTACGTCTGTGTTGCCTCATTCTATTATCGAATATCG
AATGGCTCTTAAAAAGTGTAAATAAAGCGTGATCCATTACTGTTCAAACAATATTTCTATAGAG
AGCTGATAAGAGAGCTGATATCGATATTCATCGATATCCAATACAAATTTTGATCGTAAATTAG
CTAATAGTAATAGCTAATTAACAGTAATAGCTAATTAAGTAATAGCTAATTAACAGTAATTAGCT
ATTTAATATTTATAAGGATCTTAATACACCATATAGACAGTGTGTCTCTGGGGCTCATTTCCAT
GCTGTGCATAACCACCACCCCCTTTGGAAACCCCCTTTTGATTCGTCGCAGCGCTCGTGA
TCTGGTGCCCTGGTGCGCTTAGCTTTTGAGTAGCGTTTTATGAATCTTGAGATTACGATAACT
ATGTTCAGTGCATGGAAACATGCCTTCATCGTCAAGTCTCTTCTGGCTCTTTGGGGTGGGC
AGACAAAGAATCAAATGTATGTAATTACAATAACAAACACTAGTTATTAAGTAACTATTCAAGA
CAGCCCAAATTCTTATGCATATCTATCCATTACAATATTACATTACAGAGTAAAATGCAATATG
GTCTCTCCCATTAATAGCACAGAAACAACTACAGCATTTGAATCCCTCAGTGGTAGACTCTG
TTCTTAAACTACTTTAAACCATCATAAAATGCATTTACAGGGATCAACTAGGTAGTGATGCAC
ACAGTGCATTGCGATGTACTTACGATGCACTATGTAAAAGTGTCTCACTGCGTATATAGTTATA
CGTATTCATGTACATTTTCTGGAGAACGTGAAGACATATTAATGTCGTGCATGAAAAATCAAAT
TTAAATATGCCAACAACAGAATCAAACATGTTTGTAAAGCAAGCTCCATTAGTCATAATTATTG
GCAATCTGCCAATTGATGCAAAACAATTAAACGAGCACAGCAGGCAGAAAAAGAAAGAC
GAGCATACCAAATAGGACACTCAAAGTTTTGCTTGATAGATCCAATAATATCTTCGATTTCTTT
TGTATCACTCTTACGCAAAGACATTCTAACACTGAAACAGTCAAAACAATACTGAATAACTAT
AATTTTTCTATCAACAAATACTGTAAGCTTCTTATAAGCCATTTATGTTTATTCCTATCGCACAG
CAGCAACATCGTTTAAAAGACTGGTGTTCAAAATACTGCATATGTGACGCAAGGGCTTAAAA
CTCGTCGCTTCCTGGATATATAAGCTAGAGGCCCAAATCGGAGAGAGGTGTTTTCGAGTTT
GCAATTCTTCAATTCAGCTGACCAGTGTTTGTGAATTTGCTATAAGTTGCAAAGTTGTAACCC
GACTTTAATGCCATTTGGTTTATTTGATACCTTTATTGCCCAGAATCATTTCAGAAGAATAAAGT
TGCAGAGGATTATGATGATACGAAGAATCATAAACTTTTATTCAATTTTTTTCAGTTGTTTGATT
GTTCTAATGAAACCAGTATAACCTCAGTTAGAAACAATATATTATGATTCTGTAAAAAGCATTTT
GAAGCAGATGCAGAGTAGGAATAGAATATGACTGAAGAAAAAAGCCAAAAACAACATTATG
ACGGGTTTGAGTTAGTTTGTTTTGATATATTATAACTTATTATAACTTATCTTGAGTTGTGCCCTAT
AGAGGGCAGCGTATTCCACACTGTATATAGGGTCGTTGAGGAAATTGACTTTTGGGTCTGTG
TCACATGTGTGTTCCTTACTACCATGAAGAACACATATTGCACCATTGATATATGATGGCACA
TCATTCTTAGCTAGTTGCCTGCCATGTTTACATGTTTACATATTATTACTATTCAAATCGACTAA
ACCTGTGCAAAAATTCTTCGGATATAGTTTCTGCCTGTAGTCTCAGGGTTCGATGTTTGATAG
ATTCGGGGAATCGGGTTATGGGGTGCGCAGTGAACTTACCGTAAACAGAAGCAATGCGTA

TCGGGTGTGACTGAACAGTGCTCCGGCCGTTAATAGTGGGAGATCTTAACTTCATCAATCTT
GCTTCCACTTACACTGTAATTTTTCATCAATCACCCTTCAACTTAAACGATGATTTTTCATCAA
TTCCACTTCCATTTAGACGGTAATTTTTTTTATCGATCCCGCATCAACTTACATGGTAATTTTTC
AGCAATCTCGCTTCCATTTACACGATGCCTTTTTCCTCAACCACACTTAAACCGTAATTTCTT
CATCAATACCGATTGCAATGACAATATAATATTTTCATCGATATCGCTTGATAACATGGGTAGG
CCTATCATAAAAGCAGTGACTAGCGTACGCTAAAAGGAAGTTGCTAGCGTACGGGCAGCA
TGTTTCAAAGTCCTGCGGCCGTAAAGTCCAGTGGACGGGCCTAATGCTAAACGACGCATA
GCCCACGTGACGCATTTAATTAGCTCGTTAGTGCCCAACTGATGATCGCATAAGTTATACAT
GAGGGATTGTATTTGCTAAATATTAAGTAGAGGCTGGATTAATGAAAAGAATTCTGCTTAAATA
GAAGCCCGATTAAACAGAAGTCGAAATTGATGAAAATAAAGCGTCTCTCATAGACTATAGAT
GTTATTATATTCATTTAATGTATGGGTGCCCGTACGCTGGCAACTGCTTTTAGCGTACGGGTG
CCCGTACGATAGCCACTGCCTATCATAAACCAGCTAGCCTGTGTGCACGCGGTACAGCA
AGCCTATGTCTCCGCTGTTAGCGGCCGTCCCGCTAGCCTATGCCTCCGCTCAGGCCTTC
TCATTTGAAAATACCAGATGGGCCAAAAACGGCCCGTGCCGCATTAATTAGGCGGACCAT
ATACGGGCCATGTGCGGTAATGTAGCGGCCCTGCGGAGCGACGCCTGTTTGCTGTTTGG
CTGTTTGGCGGGCCACTTATGGCCCGTATAGACCCCAGCCAGACGGGCCATTTAGACGG
GCCAAAAATGGCCTGCATCCGCTATGTACAGCCGGAGCACTGTTTAGTCACATCAATGTGT
CTCCGTGTTTGGTAAAGTTGACTAATCCTTGCCCGCCCTATAAACAGTATGGCCACCCGC
GAATGCGGTCAAATAGAGCGACGCTCATAGCTACCAGTTAACTCCCGTCATGCAATACGG
TCAATAAGTAAGCAAATAAAGACGATAAATAATGTGTGTTTATTCCAGGGCTGGTAAAAACCT
AAGGTTTTTAAAAAATAGATTTAGGTTTTTTTTAAGTTTTAATGTACGCACAGTCGCACGCGGT
ACACTGGGCACAGGAATACGGGAGTGACTAAACAGTGCTCCTGCTGCTCCGGCCATTAA
TAGTTTGCTGGACGGCCGCTGAGGCTGTTAATAGCGGAGGCATTGGCTAGCCGCTACGG
CCGTTAATAGCGGGATACCTTAACTTCATCAGTTCCGCTTCAATTGATGTGATGATTTTCATC
AATCCCGCTTCCACTTACACGGTATTTTTTCATCAATCCCGCTTCAAATGTCAAGGTGATTTT
CATCAATCCCGCTTCCACTTACACGGTATTTTTTCATCAATCCCGCTTCAAATGTCAAGGTG
ATTTTCATCAATCCCGCTTCCACTTACACGGTATTTTTTCACTAATCCCGCTTCAAATGTCAA
GGTGATTTTCATCAATCCCGCTTCCACTTACACATTATTTTTTCATCAATCCCGCTTCAAATTT
CAAGGTGACTTTCATCAATCCCGCTTCCACTTACACAGTATATTTTCCTCAATCCCGCTTCC
ACTTATACGGTATTTTTCATCAATCCCGCTTCCACTTACACGGTATTTTTTCATCAATCCCGTT
TCAAATTTCAAGGTGATTTTCATCAATCCCGCTTCCACTTACACGGTATTTTTCATCAATCCC
ACTTCAAATTTCGTACTGACATTTGTTCTTACTATTAGCTTAGTCTATTATAAGCGCTATTAATGA
TGATGTTGTTTATTTGTTTTTGTCTTAGTTGTTTCATTTAACAATAATATGTCATTACCTTTTGCTTT
TCCTAACAATATGATATCTATTTGCTTGCTTACTTACTGACCGGATTGCATGACGGGAGTTGA
ATGGTAGCTAAGAGCGTCGCTCTATTTGACCGCATTCGTGGGTGGCCATACCGTTTATAGG
GCGGGCACGGATTAGTCAACTTTACCCCGTGTTTACTTTGGTCTATGAGTCTGTCAAAGCC
GACCTCAGGAATCCAGTACGGGGTAATGCACTCAATAACGACAGGTTTCTAAGTGCGGGC
TGGCTGGTACGAAAATCACGTGCATGTTGAAAGTTCCGATTTAAGTATAAGCGTTGTTAATGA
TAAATTTGGTTTACCTCGTTTATTTACAAAAAATTCAACCGAGACATATGATTATAATATACAAAT
CCCGCCAACATGGACAACACTGAAGATAATATGTTATGAATTCACACTTGATGACGTCATAT
AACTAAAACCGTGCGCTATAAAGAAGTTTTTCTTTTTTTACTATTTTACAAATAATTCGACATTAT
TGACAACGCTCATAGGAATCTAAATTTCAATAAAGCACAATTCAATTCATAATTGAATTCTTTG
ATATATGTGTTTTGCTTATTATAACTTACTTTATTGGTACTAATAGGCTATTCCAAATGCACTGTG
GTGATATTAACAAAACATCAATCACATAATATCATAATTACATAACATGCTATTCAACTTGTCAT
GTGACCTATCGGTATAGATAAGGAAGGTATAAGGGCGTATAGAGGACACGGAGGAATCAAT
CCAATGCTGAGCAATTGGGAGGTTGATGCCAAATGGGCGCACTGCTCTTTAGCATATCCTA
TCCTGGTTCGTCCTCTTCCCCGTACCTCCCACCTCGTCTCAACTCCAAACTCCATAGCCG

TCTGTCTCCCTGCCTCTCTGGATTTTGACCGCTGCCTATCAATTTTTTTCTGGTCAAGCGCC
TGTGAATAAGCTGGTTCCCGTGACTTCGCTTTTGTGGGTGTTCTAGAAATACATTGTATTGTAT
TGAAGTATTTATATAGCGCCCCTCAACAGCCATAGGCAAACAGAGGCGCTTGTGGTTCGAT
TAGCTCCAAGAAAAGAGACAAGTTTTAAGAAGAGATAAGGACGTAGAATCAATCAATCAATC
AATATTCGTTTATTCATAAACGAATAGAAATAGAAAGATTGGATGATAGGAGAGAAGCACGAG
CGGAAGGTGGAAGGCGATTCCAAAGAGAAGGGGCAATAACGGCAAAAGATCTAGACATG
GCCATGGTTGTCCTAGTCCGAGGGACAAAAAGCTCCCGCCTGTCCATGGAACGTAGAGG
ACGAAGGGATGTGGCAGAGGTCGGAAGTCGGATGGCGTCACGGAGATATTTAGGTGCCA
CCCCCATTTGGGCCTTAAGAACAATAATCAGAACTTTATAATTATTCAATGCGCGCAGAGAT
TGGAAGCCAAAGGAGATGTTCCTTGATGTAGTTGGAGATATGGAAGTAACGGGGAAGTCTG
GCAATGAGTCGTGCAGATGCGTTCAGTACAGTCTGAAGGGGAGATAGCCGGGTCTTAGGA
AGACCGATCAGTAGAGAATTACAATAGTCAATCCTGGAGCATACAAATGCGTGAACAATGG
AGGTGAAAATAGGAACAGATACAACTTTGCGAATGGTTCTCAGACGCCTCAGTTGGAAGTA
AGAGGAGCGAGTTAAGTTGGAAATGTGCTGCGAAAAGGTCAAGGCAGAGTCCAGAGTGA
CATCCAAGTCACGAACAGTAGTATGAAAGGTAAAGTGTGGAAAACGGTCAGAAAGCAAAG
CATGATCTAATTTAAGGAGTTGTTGAGGTGTGCCGAACCAGATGAGCTGGGTTTTAGCGGA
GTTCAGAGAGAGTCTATTTGAGGACATCCAAGAGTTAAGATCATTTGAGAGTAGTTCAATTTT
GCTGGCGAGAAGAAGTTGACTAGAAGGAGGACCATATGAACATTATGCCTGAACGTCGTC
AGCAAAGAGATGACCAGTAGCCGAATATTTTGAAAAGAGAGTGGGAATGTCAGCAGTATAT
AAGATGAACAGAAGAGGACCCAGGACAGAGCCCTGGGGGACACCCAAAAATATAGGAA
CCCAGGAAGTCCTGGATTCATCTGAGATAATCATTTGAGTACGGTCAGAGAGGTAAGATTTA
ATCCAAAGAAGAGGGAGAGATGAGATTCCACACGAGGTCTCAAGACGCTCAAGAAGGATT
TGGTGATCGACCATGTTGAAAGCAGCAGATACATCAAACAGGGCCAATAAGGACAGCTGC
GATTTGTCAGTAGCAGAGAAAATGTCAGAGAGAAGAGACAGTAGAGCCGTTTCGGTAGAAT
GGTTTGCTCTGAAGCCAGATTGGTAGGTCTGGAGTTTACGATTACCAGATGTGGGATAAGG
GAAGGACCCAAAAGTCAATTTCCTCAATGACCCTATATACAGTGTGGAATACACTGCCCTG
GATAGGGCACAACTCAAGATTAGTTAATTCAGAGAACTTATATAGCACACTTCTTAGATAACC
CATCCTACACAAAGGCACTTTCAGCTCAACTACAGCAAGAAATAACAGTTTCCAGACGCTT
GTAAAAGGTGTATACAGTTTCTGGCTAAGAGGACACACAAATCAGATGGGAGGTTATTCCAA
GTGGATACAGGGGCGTCCAGTTCGTGTAAATGCATCATGGTCAAAATACATGCAGGGTGG
AAGCAGAAAAACCTAAATTGAAAGAAAATAGGGAGACACTTTTTAATTTTGCAAAAATTGGG
GGAAATTTATAATTTTTTGGAAATGGGGGAAATATGCATCGTTGACTTAGGGGGATGGTCGC
CGCTGAGTGGATGCAGGGTGTTGAAATGACACGGACGACAGGAAGCCGAAGTTACTGTTT
AACAACGACTAAAAGGAGCCAAATAACTGATTCCAGTAAACAGAACACAATTGTTTTGTTTC
ATTTTTTATTGAAATCGAATGGCTACAACATGTAGTACAAAGGGCACTGATCAACACATCCG
GCCTGCGTCTCTTTGTACAAGTTCATATCTTACCAGACATAACAAATGTTCACAAGCTACTGT
CACTATCAGTTTATTCGCAGAGGTAACAACGACCAATAATTTTAAAACCATATACAGTACGCA
GCATGCAGAGGTCATGCCAGTAAAAGCAATTTATTTTCTGTTCCACTACCAAAATACCCCTT
TGAACTGTCACCATCATGTCATACACTAAACAAAGCCACGCGAAGGTACAATGATTAGAAT
GTGGGGTGTGGGACGTGGCGGCGCCTTGGTTGAGACGATGACTTTCAACCGGAAGGTC
GTGGGTTCGACTCCCGCTCTAGCCGCCACGTAGGGACCTTGGACAAGTCCTTTACCTGC
AGTTGCCTGTGCGCTTCGGCGCGAAACTCCGATACAGTATCCGTGCTGTACTCGGGAGC
GCCTCTGAGTAGTAGAGGACTTGAAGGGGTGCTATAGAAATGGCCGGAATGAATGAAATAA
TGAATGTATGTACATAAAGTATGACTTTTGACACTTTAATAACAAGTCTACTAAAAGCGACTGG
AAAACCAAAGTAGCTCATAATTGCAGCTCTAAGACAGTGAAGTAACTGTCAGAAGTGTCCAT
CCCCCTAGGCCAATGATGCATATTGCATATTCCACCCTATTTCCACAAAGTTTACAAATTTTC
CCCTATTTCCACAAAATTTGTACATTTCCCCATTTTTTGCCTAATTTATGTTCTTTTGCTTCCCT

CTATTTTGACAATAGTGCACTTTTGTATCACGCTTCACACGTACTACACACCCCTGATAACA
GTCAAACTACCATAAACTACAACTACATGTGTAAAAGTCAAAAAAGATCAGAGAACACCAG
AAAGCAACTTGGTTGTTTCACTCCCATTCCTTACCAGAGAATTTACCAATTAACAGCAGACA
AGACACACTGTTCCAAAAACACTTCAGCTGGGAGTAATCGACCCAGGAGAAGGCAACTGT
TTCAAAAGATTCCGACCGAGTCTGGACCCAGCAACCAAGTCCGCAACCGCCAGAAAACT
GAACTCAACGTAAAGTCCTTCATTATGACTGCATCATGGTAGAACGACTGTGCAGTTCGAGA
GTGACGCTGCTCATAAATGCGGGTAAGCTGTTGCCCATCGACAAATAGGTTTCAACGTTCT
TCTCGAATGACTCGGAATATTTGTTGGCAAGCAGTTGCAGTTCACCGTTCGAACATTGCTAC
AAGAAACCACTGAGAAAATCAAACCAAGCTCATAATGTACAGGAGACTCGCTATGATGCCA
TCTAGCGGATGGATCTCATGTAACTAACTACCTACTTGAAACAAGGATGTAGAATAATAAGAT
GACTTAAATTTAGAACCATCCACTATACACTTGGGCCATCCATTTAGCTTGTACGCATGTAG
GGGAAGCATCCAGTGAAAATGCTTGCGTGGGACGTAGATAGAAAAAAATTTGGGGGGTTT
GGATTTTTGTTGAGGGCTCCATTGATTTTGGGTGCATTTTTATGCACTTTTAGGCCTTTTTAAC
CGGAACGAATTCATTTCAGAGGGGGTTTGAACACGGAGACCCCTTAAATACGCCCACGT
GCTTGCGTATCAGGTGTCTACTGATAATGCACACATGCATAGGGAACGGGAGAGCTGAGC
AGTGCAGAGTAAAGGCTGAATGGAAGGAGTCATCATTTGAGAAGTCACATGTAAGTCACTG
CTAAGATTGTACTATGATAGTGAGAATCCAGTGGAACCTGCACGGAAGAGACATTTAGAAGA
AATACTGGACAAACGCTCTGTGCGGGCGAGGCCATTGAATTCAATCAACTGCACTTAAATG
CAGGGTGGAGACTTAGGGGGACGGTCCCCTGCTAAATTTGAGGTGGGGGGACGGTCCT
TGCTTACATCCCCTCAATATTTCCGAAATACTTTATATCATCAAAAACGTCCACAGTGTTTCG
ACAGTTATTGCATTTCCGCAAAGTACCGTTCGAATGATGACATGACTAAAAAAGGTCATCTT
GAATCTAGAAACGCTAGCCATTTGATTCTTTTTGGCCCCCCAAACCCAGGGCCAAGTCTC
CGCCTATGCTTAAATGCTTAATGAATCAGACCGAGGCCCATCACAGAGCTAAACAGTTGTT
AGTTAATCATAAGTAATTAATAATAATTAATTGCACGGAGTCTCTAATGCTAACCTTGAAGAGG
AAGAAGTCATTCGGTGACTCCGCATACATGCTCATCAGACGATATTCGTTTGTGTTCGGTAT
GTCAACGCGATATCCAAGCACCTTGTAGTTGATCTCGCGCATTTCTTGGCTCGTCTTCTTAA
ACGCCTCCATCAGACGCTGGTTCTTTAGCTGCTCGCTCACCAGTTGGCTCTTTGTCTCTGT
TTCAAATGGACACAGTTTCACATTGCTCAAACTCAAGCAGATCAGTCACTGATTATGATCAG
TATTATTATGAAGAAGAAGAAGATCAGTCCCTTAAGGTAGCCCTAGATCAGCCATGGGTATG
AAGTGTTAGCGACTTCATTTTCTTAGCCTCGCCATATGAGGGCTAGTGGCGTCCTCGCCCT
CACCGTCTTATCTTCCCACTGACAGTGTGCGGTAAGTTAACTCATCATAGCCGAGTTGGCT
AGGGGGCGCAGGTGGAGCAGAAATCTCGAACTAAATTTCTGCCCTGGCCGGGATTTGAA
CCCCGAACCTCACGGTTGACGGTCCAGCACGCTAACCACTGGGCCACCGCGCAACAC
CAGTATTATTATGATCAGTATTATTATGTTAGAGTTCTTTTGCAATTCCTGGTGGACTGTGAGATT
CTTATCGACAAAAATAATCTTCACATTTCATAAAATAGCTTATATTAAAAACTAACTATTACAGA
ATTAAACATTATCAAAATAATATCTTAAAATGTATATTAATATTTTTATTTATCAAGGTCTGTTGTAA
ACCTTTAAGTTCTTTTGCAGTTCCTGGTGGGCAGAGAGGTTCTCATTAAAAAAGAAAACCTT
TTTTGCATTTCATAAAATACTTTGTATTACAAATTGATTATTATAAAACACCACTAAAAAATGTATT
AATATTTTTATTTATCAAGCTCCATTGTAAACCTTCGAGTTCTTTCGCAGTTCCTGGTGGGCAA
AGGTTCTCGTCGACGATGACCGAGAGACCTTCGACCGGTCCGCCCGATTCTTTCATCAGA
TTGAGTTTGGCGCTCAGACGGGCATTTTCCTCTCTCAATCGATCGATCTCCTGCGTCTGAC
GATCACGGGTCATCGAATACGGATTCATTCGCATGTGATAGACGCTCGTCTTTGATCGGTC
GTAACCACCCTAATTAGTAAAACAAACATCGGTGATGCACGTGCTATCCGATCATATTGCCT
TTCAAGATTCGACAAACAATCCACAACTGGAGAGAATAACAGAAAGAACATGCAACTTCGT
CGAAAGAAGAGAAGAGCAAATAGTTCCATGGCTCAGATTCACAGACCTTGCACGCGTTAG
ACATTTATTTAATAATAGTTTAAATGACTGAATAGTGAATAGTAAGTTAACTATCTTCCTCCACTT
AATTAACTAATAATTATTATTAACTCTAACCATGGGCATATTTACGGGGTTTCCGAGTTCAACC

CTCTCCCCAAATGAATCCGTTCCTGTTATAAAAGCATAAAAATTACGTCCAAAATCAATGGA
AAAGCCTCCCAAAACCCAGCCCCCCAAATCTTTTATGGTTATGTCCCTGACTTTAATTGCT
GCACTAGTCACCCATTCAACAACTTACTTTTATTAATTATTACTCCACTTTAGGCGTAACTACA
TAAGTGAAATGCTGGAAAGTGATTTTATTAAAGTTATCTAAGCAGAGTCTTCCCACCTGAAGA
TCCACTCTCTCCAGCTGAGCAAGCGCTTCCTCTTTCTGTTCACTCAGCTCTTCTGCTTTCTT
CTCCAGGTCCTCCACTTTCTGCCTAGCAAGGTCAACAACAAAGAGAACAGTAAAATAAATT
TGTAGCAAAATTTGCACCAAAACGACAGCACCAGACTCCAGGATTTGTTATTTTCACCTCA
GTAACTGTTCAATGATACGCAAAGATAAGCAACGATATAACAAATTATATATCATCTAAAGGAA
CCCATTTACAAGAATATAGCCAAGACCACAGTCAGAGCGACCCTCATCCGCAGTTGTGTT
CAAGTCCAGGTACCTAAGTCCAAGAATGTATTTTACCTAAAGGATCCTATGTACAAGAATAG
AGGGTGTCTTACCTGAGTCCAAGAATGATATTCTGGTCAGCAGCATTTGTTGTTGATACGGC
AACAGGCTCCTTGTCTGTCTGCTTCAGTAACCTCTGCTCCAGCTGATGGGAAAAATCAGTC
ATTATTAACAGAACTCACCACACACATCACTCTTCAAGTATAATTCTAGGTCTGTCAAGACTC
AATGCTTAGACGAATAAATAGATGAGCACCTATTGCAGTCTGAATCTCTGCTTAATGCCGAAT
TTCAGTGAGTATCCTCATCTTGAAAATCATCACCGGGCTGGATCCGATTGTTGTAATAATTGG
CCCGATTATGAATGTTACCCAGGGCCGGATTAATCATGTTGCAAATGTTTCAATTGAAACGG
GCCTAGTGAAAATTAACAATTTAGGGACATACCTTAAGAGTCATTATGGCATTTGTAACAGGT
AAAATAGAAATAGAGGAAAAAGTATAAGATTTTTTGTAGTAAATGACAAAAAAGGTAAAAAGG
CATAGGAAATTTTGGTCGATAAAATTAGGGGCCTACCTTGAGATTGATTTTGGATTAAAAACT
GTTTGTCAGTGACAAGTAAAAAAGGTCATTTTCATAAATTTTCAAATTTTCGGTGGTAAATAGG
CTTTTGAAAGGCCTACTAATTTATTTTTGAAACGGGCCTCTAAAAACTTAATCCGGCTCCGAT
GTTACTTCCTTATCAGTAAAGCACTAATATTGTACACTGTTTGAGGCATGGGCAAAAAATTGG
GCATATTTAGGGGGCAGTTTTATGGGTTCAAACCTCTCTGAAATGAATGCTCTACTGTAATAA
AAGCCCAAAACTGTAGGAAAATACGACCAAATTCAATGCAAAATCCCCCAAAATGTCAAC
CCCCAAATTCCTTTCAGACTACATTCATGTAACGGAAGTTGAGAGAGGAAGCAGAGATAGC
TTCAGCCAAAGCTGCTGCACAGTTGAATTTGACTCCTGTCAGTTTGAATTAATAAACGAAGA
CAAATATTACCATATTAAATGTCATAAATTATAATATTATTGTTTTCAGTAAAATTTAAAAAATTAAC
AAATCCATCTCGGCTAGGAAGAGGACTGGTAACCGGCCTTTGTATATCGTGTCTATGAAACT
GAAGTCGCTAACACATCATATCCATTTCATAATTCTTCCTCTATCGTTTTTCCAGTTATCTCATA
AATTTCTGACCTACCAGAGTGACTCTCATCCGCAGTTGTGCCTTCCCCTGTGCTGCTTCTTC
TATATCAGACTCCAGAGTGGAGATGTGCCTCTGGTGCCTTTCCACCAATGTCTCCAAGTGTT
GGATCCGTGCACTGGATCGAGTGTCAACGCGAGACGTCATGTCGTCGTCGTGCCGATTGT
ACTCGTCGACGATGTTCTTGAAAAGTTCTTTCTCCTAACAACAACAAACAACCTTCTTTGCG
AAACATCTATTGGGTAACGTAGGGTAGGATTTAGGTATGTTAATAGCGTTTTTGGCAAACCG
GATTTCATTTCCTGAAATCCGGTTTGCCAAAAACGCTATTGTTGGGACCCCAAGCAACAGA
ATGCAATAATCAATTCCAAATTACATGCGACTGCCCAGAACAGCAGATACGTGAACTTACCT
TACTAACAAAAATCAATTTCTTTCGTAGTCGTGAAACCAATCCTTCAGATTCCTTCAGTTTGG
CAGAAGCTGTGGCCAACTCGGCCTTCGTGCTCTGCAGCTCAGCCACTGAAGCCCTACA
CGTGTCCTCCAGAACGTGGTTGCTGAAGATATTAGCATCAGCAAAGCGTTTAGAAAACTCA
AAGCAAACACAACAATCAGTAGGCATGGGCATATGTGTTTGCAAGCATGTTTATATGTGTGC
GTGCATGCATGTGTGTCTGTATGTGTGACTGTATATATACTGTGTTTGTGTGTCTATAATAAGCC
ATGATCATTTACCAGTAAACATCACCATAATCTGAAGAACTAGCCTAACCACAATCTCACTG
ACTGCTGCTATGATAATGCTCAAGGTAGCTTCTAAGTTATTTCAGTATGCACAAATACTATGGA
TGAGCCCAGCTGGGTCCAGTTTTTCTTCTGTTATGATGTCAATTTTTAAACTAAGTCACGAAT
GTGCACTCTTATTCTTTTGAAATAGACGAATACTGCCTAACAGTCAAACTGTAGCTTCTCCAA
ATCTCATGTTCTTTCCACAACAATTATTTCAATCTCGCTCTCTCTTCATTTAGTGCCCTTGGCT
CCCATCAACTTGTGTTCATTGCATATTATCTAGTATTAAGCTTTTGCTCATGTATTCGAGTAGCA

GTTTGAAAGAAACAGAAAAATCAGAGCTTTGTAGGTAACAACATTAAGGTGAGAAAAGACC
CAGCAGCATGAAATTCTCCAGTGGACCGTTCAGTAGGGCTGTCAAAAGTTGCCAAAAGAC
CCACCCGGGTAACCGCTCTAGGCAGGATCGATTAACCGTGTACACGTAGGCCTACAGTC
ATGTAGACCAGGGAAGGTCCTGTCGCCTTTGCCAGACACAATTTAAAGGCCCTATGTGAAT
GAAGTCATTCCATATGTACACTATAGCCGCTACAGAAGGCCGTTTTAGGCCGACTCCGATA
ACTGTCCTATTAGAGAGGGGGGAGGGCTTCTTTTGAGTTGTTCCCACACTTTGGAAAACAG
CAGATCAAAAGAATTAAGACCGGCGCAAACAATATTCAGGCAGACGCAGACAGATGCAA
GCAGACTCAGACAGTCAGCCTTGTCGACGTGACCCAGTCTCTGGACAATAATATTTCCCCT
CCCAAATTTATCTGGTCGGCTCAGCCAAGATGTTGGCCTCGGCCTATAGACTAAAGTGCA
CCCCACTCTCCAGACCTACTCACTGCAATTGACATAGGACAATTGAAACTAAACAATGGCT
GAGCCAAACTATAACGAACAGAACACAAGTTCTAGTGCTTAGTGTACGGCATTCCCGCCTT
TGAAATTGAACTGGCGACTAACCGTACTTGATACTTATAATCCGTTTAGCTACCATCGGTCG
GTTAACCGTGTAAACGTTGACAGCCCTACCGTTCAGAAGGTCAGTATACGTTTGAGGCTCG
ACTATAAAAAGAGTTACTTAAACAGACCAAAATCATGTGGACACAGTTGTACAACACAGGCA
CCACAGCAATTAAATAAATTCAAAATAAAATTGTCAACTGACTATGAGTTACAAGAGGGTTTG
CTGCATGACCCTATATGTAATTGTAAGACCTTACATGTAATTGTAAGACCCTACGTGTCATTG
CAAGACCCTATCTCTAAATGGAAGACCCTACTTGTACAACATTAACTGTAATTGCAAGACCC
TACCTAATTGTACGTCATTAACTGTAATTGCAAGACCCTACATGTAATTGCAAGCGGACATTT
ATGTAGCGCCGCTGCAAGGAAAAACTCATTATTGGTGTTATAGATTTGAAACACTGAAATCA
CTGTCAGGATATGATACAGACTTCAGATGCAAGTCAGAAGCGTAAGGGACAAACGTAGGC
TCTCGAGTCACAGCTCTCGGAAAAAGACAATACCAGCTGGTGGGTTGAACATACGCAAAG
CTCCTGGGTTGAGCTGTAACCAGTATGCATACAAGGAACAGCGAGGAGAGCGCATGTTAA
CTAAGACGCTTGAAGCACAATTGGAGTTTTGTGTAATTTGACAATGCTTGCTTACCTTGCCTG
TAGCTGACCATTTTTCCCCAGCAGCTGCGCAAGGTCTCGCTGCAGTTCAGCAGTGTGCC
GACGGATCTGAGAGGGAGATCTGAGATATCATAAAGAGACATACTCAATCACTGTATTATGTA
ATGCTACAGTATGTATTGCTACAGAAAGCCTATAACTTAAATTGCATCACATGCACTCGTTGA
ACAATCCATTTAAATGGAATTTTGAACATTATAGACTTTGAGGTTCTAAGAAGAATTTTACCATA
CAGCACTTTCAGTGACTATCCAAGCACCTGAACACAATAGTACTTTCAGCGGCTCTCCACA
GCATTTATTTTCAACTTCAAGCCTTATCTTCACTACAACATGAACATCAATTCTCTATATTTCCA
ATAACAATCTGAAAAGAATTAAGACTCTGTATTTAAATATAAGTCACATGTTAGAAACTGACTTC
AAGCCGCTGCTTTCGTCGAAGCTCTCCCACTGTGATAGTCTTTTCTTCAGGTCCTTCAAAA
CAAAAAATTGGAAAACAAACTGACCTTTTTACATTAGTTGCACTGCTACTTAGCAGGCAATC
ATCAAGGTCTGAGGCCGCAAGCTCTCAAGACCAGAAGAGCATACATGGACCGTCTAGTAT
AGGCTCTAGTCACTACTACGTAGCAACTACAGAGTAAAGACAAGCTCAATCTCCCTTAATG
ATGACAGGCGTAAACTTTGGGGGGCAGCCCAGGCACATGCCCCCAATAATTGAGAAACG
CCCATGCATTTATCACTTTTTACCACCTTCTGCCCCCCAATATTTTGGTTTGCCCACCCAAT
ATTTTTGACAAGTCTACGCCAGTGCCCACACTCCATGTTACAAATGAAACAATTGATCAATG
AGCCACTGCAAGGCATAACCAATTCACCAGTACATTAACTCCAAACCAGTCTCATTCTGCA
TGCTTATTTCACTTCAAGATGAATCACCAATGAATTATGAATCAAGTCAAGTCTTCGTTTATTCA
GACCACCTTTGACACAAACGCAGAGGGGTTTACAGGATCATAAGAAAAAATTCAATGACC
CATGAATTACAAATTACTCATGAGCAGGGGCATATTTAGGGGGTTTTACGGATTCAAACCTA
CCGTATTGAACGACTTACTGCAGCAAAAGCCTAAAACTATAGGAAAATACAACCAAATTCG
ATGCAAAACCCCTATGAAATCTTAAAACCACCGAACATTTTCTGGCTATGCCCATCCTCAA
GAGTTGCATATTACCATGAATTACAAATTATCCATGAATCACGAATGACACATGAATAAGAAA
ACAGGGAAAATTCATCAACAGTTCATCAAAGGCAAACAAGAACATGTGTACAAAACTGTAC
AATCACGTTTAGATGTTTCTGTGATGTGTATGCATTTGTATGCATGTCTGTAAGTTCGTATATGTA
TCATGTCTGGCATCCAGATGTTCATATGATACATGTGCACAACTCAATTCTAACACTATGAAA

GGCCATTGTGAAGACTAAATGCTGCAACAGCATTCTTAGTATTAAAATCTGCTCAACTCTTAC
ATACTTCACACAGAACTTCAAACTTTGACAGTTTGGCACACTGCTGCTGGGCTTGATCCAG
TTTCGATCGAAGGTCAATGTTTGTTTCGGTAAGAAGATGCGTGTTCTTTTGAATGGCACTAAA
ATTAGACGCAAAGACCACAGATTTAAGTCCATGGCTGCCAATACAGGAACGTATTCAGTTC
AGCAACTACGGCAGAAATTTAATTCATATACAAATAGTCTCAGTTCTAAGATTGAGTTGCAAA
TCAGAATGGAAATTAAATTCACAAAAGTCAGTTTTAAGATTAAGTTCACTGCAAAGCAGAATT
CAATTAAAAAGTCAGTTCTATGACTAAGTTTAAAGTAAAAATGAAAATTAAATTCAAAAGTGTC
AGTTCTATGACTAGGTTTAAAATAAAAAGTTTAAAATAAAATATAAATTCAAAAGTGTCAATTCTA
TGACTGAGTTTAAAATAAAAATGAGATTCAAACGTGCAAGTTCTAAGACTAAGTTTAAAGTAAA
AATAAAAATGAAATTCAAAAGTGTCAATTCTATGACTAAGTTTAAAATAAAAATAAAAATGAAAT
CAGAGAGTGTCAGTTCTCAGATTAAGTGCACAGCAAAGCAGAAATTCAATTCAAAAGCAGT
AGCTAGTTTTTATGTCAAAGACCATGTCTGAAAGCAATGAGGAAACTCACTCAATACAAATG
CAATTGGCTCTGGTTGTAAAGCCTCTTAGTCTGAAGCCCCACATAACCCAGTGACACAAAA
TGTGACACAGGGCTCAAAACCCGTCACTTGTCGGTTTTGACTTTTTTTATTGAATCATATACT
GTATAATATTATACCGTATCATATACCGTATATTCTATACTGCATCTGCTTCAAAATATTTTTACA
GGATCAACATATCTTGTTTCTAACCGAAATTATACTCGTTTGATTAGAACAATCAAAAAAGTGA
AACAATGGAATAAAAAGTTTAAGATTTTTTGTGTGATTATAAACCTCTGCAGTTCTTCAATATTC
TGAAAGTATTCTTGACAATAAGGGGCTTCATTCTATATCAAATAAAACAAACCGCATTAAAAT
CGAGTTACAACTTTTCAACTTATGACAAATTCACAGACACCAGCTGAATTGAAGAATTGCAA
ACTCGTAAACCCCTCTCTCCTATTTGGGCCTCTAGCTCAATATCTAGGAAGCGACAAGTGA
TGAGTCTTGAGCCCAAACATTCTGACCATCAACGGCAAACAACTAAAGAAAAATCAACTGA
AGAATTACGTTCCACATATTCCTGTAAAGGAAAGCCTCCTGTACATTTACCATGCAATCAATG
CTGATTCCTTTTGTTACATCCATAAGGTAATATTATTACCTGAGATACTGATTTTCTTCCTTGAGT
GTCTTATTTTCTTTTTCAAACTGCTTCAGCTGACTAAGCTGTGATTTCATCATCTGTACAACCTT
TCCATCTTCTTCGAAGCGGGAAACAGTCACCTCCAACTCCTGCAACCAAACCATTATCTAC
TTAGTTTGAGCTATTTCTCTTTGGGGCTGAGCCTGGGTTGGGAGCGCCTCTGAAAAGTTATC
TTGAAGGGGAACTATATGCATATAATTATATAAGTGCAAAATATAAATATGTCAGTGTAAAATAT
AAAAAGTGCAAAATACAAATAATAAGTGCAAAATATAAATCTTTAACTACTCAGTCTTCGTGTT
ACAGCAGAAAACTTTGGTTCCTGTCACTATTAAAGCACCATTGCAGTTAAAGTGTTAAATTTT
GAAAAAATATTTAAAGTCATTACCACAACAAGAGCTGTAAAAGAGCGGATAAATATTTAAAGA
GATACTGATAATACAACCTTGATAATTCACCATAATCAAACGGTCTAATTCCACGACATAAAG
CCCCACCCACCATGACACAAAGCCCCACCAACCAATCTCAAAGGTAAAGTCTAGACACA
AATCTTAGACACTTGCCTCAATAAAAACCACAGATGTATAACTCGTGCAAATCCTAGAAGGA
CATCCCAATCCAAAACCCTGTATGCATACCCTGGTCACTCGCCCCGATATAAACCACAGA
TGTACAACTCATGCAAATCCTGGAATCAAATCCCAACCGGAAACCCCGGTTGCAAAACCC
CAAGAACCAACAAAACCAGTCTCTCACCTTGATTCGGATGTTAGCAGACGCCAGCTGCGA
GCGAAGGTTCTCGGATTCTCGTTCCAAAACAGAGTTTCTTTCGCCGCCAGTTCTACATGAG
ACACAAACACAAGCTCTTAATAGATTACGCATCCCAAGACAGTAACACAAATAGCGTGATT
GTGTAGCACCAGCTCTCTTCTCTGCAGCAGGACTGCTTTCAAGGAGACCGTGGTCTAAGG
TTGACACAGATCTAAGCAAGCTCATCCACTGCTCATGAGAGTTATTAAAATGGCTCGCCTCT
GTCGTTATTAACCATATACCCGCCACAGTCAAAACCAGACTTCAACAACCAGGGAAGAGA
GAGCAGAAACTGCCCAATCTAGGGCACAGCCGTTTTGTGTTTATGACAAATTCCATTAGTGT
ATCAAAATTGTAAAGAAGTGTGATAATTTCTAGCCAAGGGTTTAAATTGGGAGCCATGGCTC
AGGTCTTAAAAGTATACCTGCTACTATAGGGGCTAATACAAACGGATATGTATCAGCCCCAC
ACGAACATAATGACGAAAATTCTAGGTCAGGACGCACCTAAAAGACCTTTCTATGTTTGGAC
TCTAACAGAAACACTGGATTCACTACATCTACGTTTTATTATTATTTTCTTCTTTGGTTACCTAC
CTCATCAGGCAGACTTGTCTTGTATGTCCAACGTCGCCATTCCTCTCATTTGTCCAGTTCTC

TTCTTTAGCTTGATGTACACATCCTCAACATCCAGTTTATTCAGATCATTCTCAGCTTCCCTCT
TGTCTCCATCCCTCTATTCTAATTCTGTTCATTCATTCATTACAGAGGTTTATATAGCGCCTCAT
CAAGATTACTACTCAGAAGCGCTCCCGATCCCTGCACGGCTAAAAAGAACAGTTTTCAGA
CTGGAGCAGAATGTGTCCGAATGAACCCTGGGGAGCAATCGCTGCGCCAACGGAAGTC
CATTCCACAGGGAAGGGCCAACCACCGAGAATGCACGAGTCTGCCATGTGGATGTACG
GACGAAATGGCCAAAGAGGACCCCCGTTCCATAGAGCGGAGGGAACTGCGGCCTCTG
GTGCCCAGGGTGGGACAGCAACGATTTTGCAGGTAGGTCAGAGAATCTCCAAATGAGAAT
CTCCACTTATTATATGACAGAGTCACTTTCTCTGCTGTCTTCAGAGTCCACCTACTAGGATTT
CTTCTGAATTGTATCAGCAGATGCTGTATCAGTACATCAAGAAGGACATTGATCTGCAGCTGT
ACCTCATCACCATCTGTAGCTGCTCCTGGTCTTTCAACTGCTTCTGCACAACTTCCAGCGA
CTGTCTTAGGCTTTTCTTTTCCATCTCCACTCTGCCGACCGCATCCGCCATCTCTTCCTTCG
TCTGTCATAAAGAAATCGATATAACATCAAGAGCCACAACCAATGTTCTACTGGTATCAAAG
CATTTTATAACAACAAGAACTTGTGCAATTGTCAGAATCAGCCATATGCAAAGCCTGCATAA
ATATCTAAATGATCTAAACAGCAGCAACTGTATTGTATATTTCCGTGCATGTCATCCGCCATTT
TGGTCGACTTGTCGACAGCTGTGTCGTTTCGATGGATATTTTCGTCTTCAGGCAGGAATTTG
CGCAGTGGTTACATAAATGTTGGAGATTTTGGAACTGGGTTAGCGTACCGGACGTTTTTGC
CGAATTAGCCAGGGATTTTTCACGTTTGAAAGGTGTTTTGTTCGCAAGCCATGTGACCTCAT
CGAGTCCATCCTAGATATAAACACGGTCAGGTCTGCGCCAGGAAAACCGCATGCCGTCT
GTAGCCAAGAGGTGAGGGTGGCTGTCATCGTAGCAATGTAAAGGAAGACTAGTATATAGGA
CTTATGTATTAAGCTTATTGCTCTTGTATTCGAGTAGCGGTTTGTCTATTGATGTTTTTCATATTA
AAACGAAGATATATACACACACACAGTAATGATGACAAAGAGTAATAGTGTAAATGAAGCATA
GTAATGTTAGTACAGAGCAGAGTAGCAGTGTCAGGAACAAACACAAACAGAACAATGCTTG
AAAATCAAAACAATAGCACTAACTTAAAATAGAACACCTTGAAAACTTATAATTATTTAGAATAT
GAAAGACAAAAAGCAGAAACAAACGTATTGTAGTCTAAAGCACAACCTGTTCTAAAGCTGTT
TCAAGGCTAATTTTCTCCTTCTGAACAGCCATCAGCATTTTTTGTGATTCTTTTGCCTTTTCGA
GTTCCTGCTTTAATTCTAGAATCTCCCGCTTCCTTTCGCCTTCCTTCTTTGCAAAGACAAGC
AGTTGATATTTGACGTCCTCTTGTTTTGCAATCTCTTTCTAAAACAAAAGCAGTTACTTTAAGC
CTAGCGTGGTGCTAAAGTTGCAGAAGTCTCTGCCACTTAAAATGCCAAAAATATGCTATTGC
GGCGGAAATATTGCTCCAATGACAACACTAACCCTAATACTAACCCTAACTACCCTCGCGA
CCCTTAACCTGACCCTAGGCGACCTTAAAGACGCGAATTTGATACGAAAATGACGAGTCG
GACAATGTAAGACCGCCATGTTAGTTTGGAAGTTGCATGGGCCGGTCATGCGCAGCGCAT
GACAATCCCACGGGAAAATGTTTTTACCGCCATTTAGCAGATTTTAGTGGAAAATAGTGGC
GGAAAATTCCAACAATGTTTCTCAGTTGGAGACTTAAAATACACATTCTCCAACTCTCTAAAA
TTAGAGATCAATGTCCCTGGGGAGCACTGCTCCATTTTTATCTTGCTCATTCCTTGACCCTA
GCAAGCGCAGCTGTATCCACAATGTGACTACCCAAACAGTGGCAAAGGCTCACTGTGGTT
TTGTATAGCTATTCTTCATTTTAAAAATTGTTCGTCTAGTTGAAAAGAGACAAGGTATGATAATA
AAGTCATTAGGAAGATCTTGTGCAGAAAGGCCACACTGCAGGTCAAGAGCAATGTGATCTA
CAACAGCAGACAGGGATTAGACTTTGGTTGAACATTGCATGGAGAACGGAAGCACCCGG
ATAGACATATTTCACCGGCAACGTGTTCTCGTTGTTGCCTTTACTTGCCAATTTTCAGCAAAC
AGAAACTGATATTTTTTAATAAGGATGACCTTCAACAAACCATGACTAGCAGTTTCACTTGCC
TTTCAACTTCATTCTAAACTCACGACCCAATAATTCCACCAGTTTAAAAACAACTACAACTCC
AACAAACGACAAGGCTGTCAATAATGCCCATTATATGTTTGATACAAATTAAAGAAAACAAG
CAAACTATTTGAAGAAAGAAATTGACAAATATCTCTAGCTATATAATGACCTGGTGTTCCTGG
ACCAGTTTGTCAATTTCTTTCTCATGTTGGAGCATCAGTTCCTGCTGATGTGTTTCGAGTGAC
TGCAGTTGCGCTTGAAGCCTCGTGACCTCAGACTTTGCGGCAATCACTTCGCCGTGCGC
TACGGCTCTGTCTTCAAAGTTAAAGCACAGTGAAAAGATCACAGAAACACATCTAGATCCA
AACTTCTGCAGCAACACAACCTAATGGGGACTGATATTTTGTTCACCTAATTACAACACAAC

CTAATGGGGACTGATATTTTGTTCACCTAATTACAACATAACCTAATGGACTGATATTCTGTTC
ACCTAATTACAACACAACCTAATGGACCGACATTCTGTTCATCTAATCACAACATCATCATCA
TTATCTTCGTTTATTTGAACAGGATGACTTAACGCATCTCATTACTAAACAATTAAGTAATTAAA
TAGCCTTAGCCAACACAACCTAAGGACAGATATTCTATTCACCTAATTACAACACAACCTAA
TGGGGACAGATATTCTGTTCACCTAATTACAACACAACCCAATGGGGACTGTTCACCTAATT
ACAACACAACCCAATGGGGACAGTTCACCTAATTACAACCCATCCCAATGGGGACTGATA
TTCTGTTCACCTAATTACAGCCCTGTTATAATATCACATGTATTTGTTGTCAATGCGACTGATTA
AACATTATCAGTACTTGCCTTTCCCAATATCACTTTCACACAGACTACTAGTGTCAAATTTCA
GTCGCTTCACTGTTGACTCTTTAGTACAACCAGACATAAAATCATAGAAATTGTCACGGATC
CTGATGACAGCCGTGCTGTTGTCTGTGTCGTCCATGGCTTTAACCTGCAACATAACTAACA
GAATATACACAAAGAAGCCGAAATAACTCCGCTTTCAATTTCAACTCACATCCAATATATGTT
TAATCCACTTTATTAACTGGTGAGCCAAGTGCCTCTTTTCGTTCTCTCGAGACTGCTCTCTTC
TCTCTGGGTCTCTCGCACTGGAAGCGTTTCTGATTGGTGTGCACTGCAAGAAGCGCTATAT
AAATGTATAGATACAATACAATGCAATACAATACAATAGTTCGGCAAAACTTGGTTCTGAAGT
CTCAACAGGCTTCGTCTACATAAAGATAAATAGATAAATAAGCCTTGACTCGTCACTCATTCT
TGAGCCTGGAAAGGCTATTAATAATCTGCGGTCCACCAAACTGCTCCTTGATTGCGATTAAA
ATGTAAATACATGTATACCTACCAAATTAGCCCTCTGCCAATCGATCTGTTTTGATTAAGCGC
CTGTGTATAAGCTGGATCAAGCAACTTCGCCTTTGTGGGTGCCGAAGAAATTTCAAGTTTAC
GATTACAATATGATTATGTACGGGCAGACTTAAACTGGCATGGCTATTGAAAGTGAATAGCAT
GCGAGCAGTGGCGTAACTAGGGGTGTCGGCGCCCGGGAGCGATGTAAAAAAATGTTTCC
ATAACAAATTCTTGATGACCTTTTCTTTTTAGTCGTCAACCAAAAATTTCTAGTAATTCAGCGC
CGTATATGCATTTTGCACCCTTGCTCCCCTTACCACTAGTTGAAAACTTACCACTTAACCTC
CATTTTGCACCCCCCTTTGGGCAATTAATTTACACTGCAAGATGTGTAAAAATACTTACCTGA
AACCTCATTTTGTGCCCCCTTTTGACCTAAGCCCTGGGGTGACCACCCTCCTCTGCCCC
CTTGTTACGTCACTGCATGCAAGCACAGCAGAAGACAAGAAGATAAAATCAGCAAAATGAA
TACGATTTAGGCTATATGGTCACTTTATAAACCCATAATATAGCACAAAATACATTGCAATTCA
GATGGTTTGATATGTAAGCTTCGGCACCTTAGTAATATCATGTAATATAAATCTTTGAAAGCTTC
TCAGTGCCCACAAATGCCACATCAGGGAGACCAGCTTATTCACAGGTGCCAATACCAAAT
GCCGAAGATGAGGAAACTGATGGAGGCCTTTTGGGAGTCCGTTTCAAAGAGCTGATGCAA
CATAGACTAAAGAGCGGTTGCATCACTTCAGAGAGAATGAGAATGGAGGCCGAGATAGAG
ATGATAATGAACGAGTAGACCAAGGCGTTTGGATGGTGAGTAATTGGCGAATGCAGAAAGG
TTGAGAGGTTTGAAGGAGGGTGAAATTAAGATATACTATTTTGTAATAGAAGCGTTGAGGGG
CTTTTAGCTAATGAAGTGATTTGAGCACAGAAGTTATGTGGTAATGCTTTCGTGACATCGCGG
GCAAAAGCTTTCTGGATAGTTTCCTTCACTTCTTTTTATAAACTCCTCAAAACTCTCATCTTTG
GCCGAGCCTGGGCTGGGAGCGCCTCTGAGTAGTTACCTTGAAGGGGCGCTATATAAGTT
GATATATAGATAGATAGTTTCCATGCTTTTAATTTGAGAAGAGTTGGTGCTGTATAAAAAGGAAT
TGCTTGGAATGAATAACTGAGGTGGCTATTGCGCTCACAGTCTTTTTGATTCATTCATTCATTC
CAGCCATTTCTATAGCGCCCCTTCAAGTCCTCTACTACTCAGAGGCGCTCCCGACTACAG
CACGGATACTGTATCGGAGTTTCACGCCGAAGCGCACAGGTAACTGCAGGTAAAGGACTT
GCCCAAGGTCCCTACATGGCGGCTAGAGCGGGAGTCGAACCCACGACCCTCCGGTTG
AGAGTAATCGCCTCAACCAATGCGCCACCATGTCCCACAAATTAAAAATACGTCTAGTTAA
CGACGCATCCATTTGATGCCATGCGCTACACGACGTGAAGATTAAAAGGTGTATCTATAATC
CAGAAGTCATTTTAGTAAACAAGCATGTTCAAATAAAACAATTAAAAGCAAAATTAATTACATA
CGAAGTTCAAAACCCTAGCGATAAAACAGGAAGTAATCTTTTTAACTTCCCCAGTGGCCCA
GCGCAGCGTTTTATAGCGCAGCTATAGTTGATTGAGAATTATTATGAAACGTAATCGTAATCT
CAAGATTCTAAAGCGCTACTCATAAGCTAAGCGCACCATGGCACCAACTTATTCACGAGC
GCTGCGACGAATCAAAGGGGTTTCCAAAAGGGGTCAAGAGAAGTTCAGGTCCGATTTCC

AGAGTACCAGGAGGGGACAGAGTAGCTGTTAGGGTGGATGTTGTTGAGATGGGGAGGGG
AATGATCAGATGGGTCAGGGTAGGAGTGTTTGAAGAGACGAGATTTTAATTTTGAGTGGAAG
GCCCTGGGGGCAGGGTTAGATTTGACGGGGGGTTGGGGGGGTTGAACCCCCTAGCTCA
CAATGCCAACCCTCCCAGCTTATTGGAAATTTCGACCCCGGGGGGGTCGGATAATTCAG
GCTTTTAACCCCCCCGTTCAGTATTTTCAACACACTTTGATCTCTATAGTTTTGTTGATGTAGG
CCTACTGTAGTCTGTAATGCGGTGTGTCATTGTATGTAACCAAATCTTGTAATATTAATATACAT
CAATGTATATAGCAGTGATATCGTGTCAGCGACTTATATACCTCGATGCTGAGTATCTTCTTTT
CACAGTGGCGTAGCCAAAGGCGGGGTGCTAAACCCTCCCATTGGCTCTTCGGAAAAAAT
ATTTGATGTCCATTGTAAAAAATAAGTAATCACTTAATGAATAATCACTTAAATATGTTGCCGAA
ACAGGCCAAATTTGATCGCAAATGCTCTAAAATGCGTTGGCGGCTGGGGCTCCGCCCCA
GACCCCGCTGGGGAACTTACAACGTCCCCCCCAAATCCCCTAATCGTGAGAGAAAACC
CCTCCCATCATAAATTCCTGGCTACGCCACTGAGTGGCATAGCCAAGAATTAATGATGGGA
GGGGTTTGTCCCTCACGATTAGGGGTTTTTGGGAGTGTCCTAAGCTCCAAAGTGGGAGCG
GGCACACATTTTTGAGCATTTGAGGTCAAATTTAGCCTATTTCGGCAACATATTTAAGTGATTA
TTCATTAAGTGATTATTTATTTTTTACAATGTACATAAAGAAATTTTTCCCAAGAGCCAGTGGGG
GGTTGTAACCCCCAAATCTCCTTTGGCTACGCCACTGTTCTTTCAAGTTCTTTGTGATCAAA
CCTTTTAGTTTTACTTCCGTTCACTGTTTTCCTAATCGGCGTGACAGTGAATTATTGTGGTGAC
AATTATAGCTGGTTGGATGTGCAATTGTGGCCAGAGGATGAGGGTGAGCGGCTATTATATG
GTGATAAAGAAGTTGCATTTTTTGCTGAGTTAACTGGTTTAAATACAGTTGATGTTTTAATTGATT
TCAGAGATTATAAATTGACCAAAAGAAGCATTTTACTTTTGATAAAAAGTTTCTTATGTAGACTT
TCAGTCATACCTATTTCATCAGCTGCCTGTGAACGTGGATTTAGTGCTATGAATGCTCAGAA
GTCACCCACCAGGAATCGGTTGCTGATGAAGAATCTTGATGGCATATTTATTATTTGTTTCTG
TTAATGATATGCCATTGAATTGCTGGGATCCAAAACCGTATATGTCATGACTTATAAGTGTTCA
CTTGCGCGGGCCGTCACCAGAACACCCAGGCATCATCACATTACTCCTGTTCTTAAATCA
CTTCACTGGCTAAAAATCCCAGAGCGCATCCACTTCAAAGTCTTGTCCCTAACCTACAATT
CCCTGCAGTCTTCCCAGCCCACATACCTTCGTGAACTCTTCACCATCCAGCCAACACGC
TCTACTCGATCATCATCCTGTCTCACCCTTTCTCGACCCCCGGTCACTTCTCATCTCATGTT
TTCCAACCGAGCCATATCAATCACTGCGCCACGTCTTTGGAATGACCTACCACCTGAACT
CCGCACCATTTCTTTGCCTCCACCGCCGTCATTGCCAATCACAAGACATCATCTTCACCT
GCCTCCTCTATCCGTCACCCCCAGGACCTTCCACTCAAAATTAAAATCTCATCTCTTCAAT
CTCTCCTACCCTGACCCTTCTGATCATTCCCCCTCCCCATCTCAACAACACCCACCCTAA
CAGCTACTCTGTCCCCTCCTGGTACTCTGGAATTCGGACCTGAGCTTCTCTTGACCCCCC
TTTGGAAACCCCCCTTTGATTCGTCGCAGCGCTCGTGAATAAGCTGGTGCCCTGGTGCG
CTTGGCTTTTGAGTAGCGCTTTAGAAGTTTTGAGATTACGATTACGATTACGATTACGATTAC
GATTACGAATCTGTCACTCTAATAATCTTCAAAATGTGCGTCTCTCTTGTATAATTATGTTGTTG
AGCTTATTTTGTAAGCTGTTCCACAATTTGCCCATAATGGCGCATAGTTCAGTTGTGCTACTG
ATATTAAATAGGCATAAGCTGTAATTAGTGACACAATCATTTCTAAATGTTTTGATAAATTTCTGT
GAATGAATAATCACTTAAATATATTGCCGAAACAGGCCAAATGTGATCTCAAATGATCTAAAA
TGCGCTGGGGGAGCTTACGACGCTCCCCCAGACCCCCTAATCGTGAGCGGCTTCGCG
CCGAAGGCGCTCGCACCTCGGCCCCTGCGGCGCCTCAAAGCTGACCCCCCTAGCTTT
TTTGGGACAAATCTAACCCTGCCTGGGGGTGACGGATAGAAAAACACATTGATTTTCCTTC
CAAGCAAACTACATCTAATTTATTCTGTCTTTTAAATTAAAAAGTAGACAAACAATAAAATGTGA
CTAGAGTCATCAGAATTATTTGAAAATAAAAGAAAAATAGCACTCGCAATTTAATTCTATTGCA
GCCTACTGCTGAACAAGTCGTAGACTAGGTTGTAAACAGGCTGTTCTAATCGAATTTTATGTT
TTAGTATTTTTAGACCTAAAATACCTGAAAAGTGACAATTTTAGATCCTTTACTTTTGAAGTCAA
AATTAAGTTTCATGTTTATTGTAATTGCCATTATTTATAACGTTCATCACAGTAACGACTGTGCA
TACGTTTAAACCCATAAAAGTGGAAAAGTCTTTTTTTAAAACCTTCAAAACTTAAAAATCTCTT

TTAAAAAGCCCCAGTTTTTAACAGCCCTGATTGCAAGCGTCGTCCATCACTTCATTAAGACA
CTTTGACACTATAGGGGCCATCCATTTATGACTAGAGGCCGACCGAAACCAATTTATGCAC
ATTCGGCGTAGCCGTAACCCGGCCGAAACTCAAATTTAAGATACGGCCGTAACCGTAAC
CGGGCCGAAAGTCAAAATTTCGGTTACGGCCGTAACCGTAACCAGGCCGAAACTCATATT
TCAGTTACGGCCGTAACCGTAACTAGGCCGAAACTGGTATTCTAAAATATGATACGTTTACG
GCTAGACAGAAATAAGCGAATTAACCTAAGAAAAGCTTAGAATGAATATTGAAAATGGATAG
GTCGCTACAAAGTACAGACTCTGGCAGCTACGGCTGCTGTTCATCTGTCGTTCTTAGAACT
GATTGTATAAATAATGACACTGATAGGCCTAGTGACAACTTCTCAGCTACTCATCGCTGGCG
AGCGACGAGCCCTATGTGGTCGCCAACACCTAATCCTATGTCTCGCCCCGAGGGCCGA
AAGTTCAAATAAGATAACGAGTGTCTACTGTCTAACATAAATTCCCACTGTGTCCCTGCTAGC
GGGGTCACCACCTTGGCCCTTCTAGCGGGGTCACGCAACACCACTGCCACTAGAAGCC
AGCTTAGCATACGGGCAGGCAGAGACTGCGTTAGGGAAAGGCTGTAATATGATTCCGTGT
TTGCCGAACTCAATGTCTGAGTTTCGGCTGGCCGTAACTATGCCGAAACTAAATTTCGAGTT
ACGGCCGTAACCGAAACCAGGCCGAAACCAAAATGCGAATTACGGCCGTAACCGAAAC
CAGGCCGATACCAAGAAATTTGGCAGTTTCGGCGCCGAAACCGTAACCGAAACCGAGTT
TCGGTCGGACTCTATTTATGACGTCCATAAAAAATTCGATTTCCACATGCGTCCACATGAGC
TGGACCCCCTTGTGGACGTCCACATGCGGTCGACATGAAATACACATCGCTCTCTTGAAA
CGGCTAGTACCTAAAGCTGAAATTCGACTAGACTTATATGATTGTAATTTATTTAAAACAGTAC
TTACTATTATATTACTAATTTATATCGCCGAAAATTTTCCACTTTTAATTCTGTCGAAAGACAAAA
TTCTGGTGAAAAAAACGCCAACTTCTTTGCATGAGAAGAAGACAGGATGAAATCAGTGGAC
TCTATTTTTAATTTTCTGTGTGGAGGTCCACATGGGGCTTGACCCCCCTCCGTGTGGACGT
CATAAATGGATGGCCCTTGGAACGTATCATCGCCAGCCTCCCAGGTGCTCAACTGCTCCT
ATCCTGAACCTTGATCGGCAACATATGCAACCATCGTAATCGTAAACTCCTGATTTCTAGAG
CGCCCACAAAAGCGAAGTCGCAGGAACCAGCTTATTCACAGGCGCTTAACCAAAACAAA
ATCGATAGGCAGCGGTCAAGATCCAGAGAGTCAGGCAGGCAGGCAGACAGTTAGACGG
CTATGGTGGATGGTGTTTGGAGTTGAGACGGGGAGGTAGGTATGGGGATGACGATGACCA
TGTTGCTCCACAGCCCTTCTCAGCCCTGCGCTTGCGCGGGTCCTATTACAAGTAAGCCC
AATAGAACTGTAATCACCAGAACACTGGAATTTTCAAAGGACGATAATTCCAAGAAAATGTC
TTTCTGGGGCAATCTTCAAAAAATATTACTGCCAGATCCTTAATTAATTAAGATAGTCAGGTTT
CAATGTCAGTGAAGTACTCAAAGGTTATTAAATTCATTCATTCATATTGAACATTTATATAGTGC
CGCTTGAGAAAACACTACCCAGGAGCGCTTCCAACACCAGCACGCTTAAAACGTAGTCT
TAAGGTGAGAAAAAACGTAGGTGAATGGGGACATTGAGAAGTGATCAATGCATGTTAGACA
TAGGTATAGACTTTGGAACAGCAGCCTGGGCGCATGCCCATCCAATAATTTCATCAGCTAT
TGCCACCACTTGCACTCCCAATATTCCTGGCAAATCTACACCAGTGAAAATAAAAACCTCT
ATTTAAATATCATCAATCAACTTCACTGCAACTTTTGAACTATATCTACATTAATGGGAAGTGG
GATGTGGTGGCGCATTGGTTGAGTCGATAGCTTTCAACCAGAGGGTCGTGGGTTCGGCTC
CCGCTCTAGCCGCCACGTAGGGACCTTGGGCAAGTTCTTAACTCACAGTCGCCTGTGGC
GCTTCGGCGTGAAACTTCGACACAGTATCCGCGCTTCTGAGTAGAAATGTACTTCAAGAG
GCACTATAGAAATGGTCTGAATGAATTAATTACTTAATTAATAACGGAATGAATGCTCCCTTTA
TTAAATCTAATGAGCTGACACAAATGCAAATTAGACAGACTGCAAGATGTTACACAGAGGGT
AATTTATGCAGTGTCAATAGAGCACACTGGAAATGTAATCACAATCGTCTTCCTCCAGTAGA
GTACAGCTACGTGGTCCTGCTCACTCATGTTTGCTGTTGTGGAGGTGGGAGCAAGTTATAA
GACACATCAATCAATCAATCAATCAATCAATCAATCAATCAATCAATCAATCAATCAATCAAT
CAATCAAATCTTCTTTAAAACATGAAATGGGTCTGTGGTGATTGCATGTAATTCTTATTCAAAAT
GCTTAAAGTACTTTAAAAATAAATTTTAAATTAAACAATAATATTTGTGAAATAGTCACATCATCT
TCAAAGAAACAATCAGAAGAAGATATCGGATCTGTTGTACTTTTTATCCAAAAAGTACAAGAA
ATGACCACATTTATAAATCCTGACATCTCTGATATTCAGTCAACAAGATAGCTTTTACAAAAT

CATCTGGCTTTAAACAGATGACGATCGTTCTATCCACGGATAAATCAGATGACAAAAATCAT
CTGGCTTTAAACAGATGACGATCGTTCTATCCACGGATAAATCAGATGACAAAAATCATCTG
GCTTTAAACAGATGACGATCGTTCTATCCACGGATAAATCAGATTACAAAAATCATCTGGCTT
TAAACAGATGACGATCGTTCTATCCACGGATAGATCAGATGACCACGAAGCAATTTCTTTCC
GAGACAAAACCAAGTGTTGTTGGGAGTTACGTCTGGTGACACCTGGCCAGACATTTTAACC
AGCTCCAGGGCAGACAGAGGTCTCAGCTGTAATTGAAAACATACAAAATCATAGACTCCA
CATTTCTGAAACTCACCTTGAAAGCCAAGCAGGGGTGTCCATTCCCTCTAAGCCAATGATG
CATATTATTTTTTCCCCTCCTATTTCTCCAAAATTTATAAATCTTCCCCCTATTTTCGTAAAATTT
ACGTTTTTTGCTTAATTTACGTTTCTTGCCTCCCCCTATTTTGACCATGATGCATTTATGCAAC
ATGCTTTACACGTATTGGACGCCCCTGAAGCCAAGCCCAGAGCACCAGCTTATTCATCAA
GTCAGACCAGAGGCTGTCACATGGAGTTACAAGTAGGGTTACCAGAGATGCCCAGGAGA
GGGGAAGGAGAGCCTTTGCCATCAACCAAACTAGAAACTGGAAAATCTTAAACTTTAGCC
ATAAAAAGGCATCGTTATTTTTAATAATCTGACAGAATATGGCACAGTAAAAATCATGCTACAC
GAATTTGATTCAGACTCACAACACACAATTTTTGCCCTGCATGTCATTTTTTACCAGTTTCTC
GAAACTGCATCTTGCGACATGCGCCTGGTTTTGTTTAGAGCACCTCACCACGGATACAAAA
TGCCAGACAAGAAAAGACATCTGATATAGAAGCAACCGCAACAGATCAGGAAAATCGAAC
CTTGAGATGGACACACAGCGGGCTCTGCTGCACTATGGAGATATGAAGATGACCTACAAA
CAAACAATACACAAAAGACATAAACATAACTAGCAGCTAACATGCACATAAGTAATATACTCT
GATCACATTCAAGTCTGAGAAGCATGTACTCAAGATCAAGATGAATTTCGTTTATTGGTTGGC
AATAAGAAAATCTAGGGCCCCAGCTGACACAGCCGCAACTTGGGACCATTACACAGGGC
AATCAAATTAAATGAATCAAAATACAATCAATAAATGTCAGAGTATAAAACGTGGGACATGAA
GCTACGATTGTCCTTCTTGGGAAGCTCATATACATGGGCGCGTGGGTGCAGGTTGTACCC
AGTGGACTTGACCCTTGGAAGGAAGCGGCTGAGAACACGACCCGGGTAAGATGTAATTG
ACCGGAAAAGGCTCTTGTCAGCCTTCGAGGCCATTTCGGCAAATGAGGGGGCGCTCTCT
GGTAGGTACCCACACCGTCGCAATCTCCCTACCATACGTTCCAATCTGCCAGCACATAAA
TGAGCGGCTAGGCTTATATGACACCCTATTACATTAAATTACTTAGCACAGTCTGAACTTGGT
TGCAAATTCAAATACAATTAAATAATTAATATGACAATAGCATGTAGATGATGACCTGCAAGAT
GGAAAACAACAAGCTTATGGAAAACATAACTGTGTTATGCAAATGATGACAGATGCCAGAA
AAATAGCGATGACGTCGTTCTACAAACCAGTTACGGCCTCCGGATGGACCATTCGAAGAA
ACAACCAATACACTGGTACCCTGTTCATCAGTGACACCAGAGTGATCAGTACGCCTTGCA
GCGACAACACCATCAGACGCAACTAAACACAAAACCAAGTACAAAAACAAGAACACAAA
GCATCCATAAATTACAAGAAATTGGTGTTTCCCTTACAGTATGTCACAATGAGCGGTATTGTC
CACCAAAGAGGGTCACTGGTTGATCACCCTACTATGTGCTCAAAAACGAAGTCTACACAC
ATGTTCTATGAGGACTCAGAAGTAACATGGCAATGTAAGAAAACTGACTGGTGAATAGCAAG
TAACTGATTTCTTCAAAATGCCAGGTTGCCAAGCCAATTAAATTAAATTAATGAAATTTGAAAA
GAGGTCTTTTAGGTATATTGTTTACACAAGCACTCCCCTTGTTACAATGTGACCAAGAAATCT
ATAGGGAGGAACTGACGTGCGAAAGCGTAGAAACCGTGGAAATACCGGAACCCTGATTG
TGGTTACGCAAACCATTCTCCTCAGATTAGTATAACACAGACGACAGGTACATAATACTTTA
GAGCATAAAGTATAACAATATTAATAATACTGTGGTATATGGCAGCCAATTGAGGGAATAGAG
ACCAATCAGAGCATAGTAGACGATTGGGACATAGTAATAGCCAATTGAGATATAGTACCAAC
TGGGGCATAGTAGCCAATTGGGGAGCAGAGTAGCCAATTGGAGGTGGTTGCTGTGTAGG
GGACCCTGAGCAGGTCCTTTGTTGTTTCATGCCTTTGATTAAGTTTAAAGACCTTGTGCACA
AAGCAAAATCAAAACAATAAGAAAATTAACAAATGTCTGGTGGATGGTAAACTGCTGCTGCT
TTAGATTCAGATAGTATCTCTGAGAAGAAGAACCCACACAATAAATAAAAAAATTCCAAACG
AAAATGAATTAAATGAATACTCGTCTTTCTCTAAAACAACCTTGTTTTATTCTGTTTTACATCTAG
GAGAGATCATGGAAATACTTTATAGTGCTACATCATAAAAATATTCTATAGTTTATGTCATGAAG
ATGTTTCACAGTGTTACATCATGGAGATATTTCACAGTGTTACATCACATAGATATTTCAAAGT

GTTACATCATGAAGATATTCCAGTGTTACATCATAGAAATATTTCACAATGATCTTTACAACAA
CTTGGGAAATTATCCTTAAAACTCAAAATTGCAAAATAAAGCCAGAGGAAACTGACGAGTG
CAAAGACGACGTAGAAAAGTGCGGTCGTCGGAAGCGTGAAGAGAAGAATTGTGAACAAG
AGCGTTCCTGCGAACAATTGGTCGATGTCGTACGACGCCGAGTCGACGCGCCTCCGTAG
GACGTTCCACTTCTTGCCACAGAAGAGTCGCCAGAGGGAAGACAGCGCCAGAATCTGG
AGACTGTAAAGCCTACCGACCAAAACAGAAAATATATTTAACATAATATTAATAACGGCAAGA
TCAAAACAGATCAGTTGCTAGACAGTAAATTCTATAACATTGGACGGTATTTAAAAAGCGCTG
TATTAAGTATCTCATACATACACGCAAAGCAATGAAAACATCAAATGGACTGCACATTGACA
AAGGGAAGACAAAGCAGGAAAATAAAAGACTGGTTTATATGAGAATTCTTACCTTGCAGCAT
AGACATAAAAGCAGTAGATGTGAACAGTCATCATCGAGATAAGATCCTGTAGAAGAGACAGT
TGAACTGTCAGACCCAGACATCCAGAAAGACAGCTGTAGTAGCAGATGTCAGACCAGAAC
GGTCGCAGTGTTCGTAGATAGGCTACAGAAAAAAACCAAAGCATCCAGTGAAATCTAATTC
TTCTGTACGCCAGATTTTCTTTCGCCTCAAATCCAACAACAGCTTAGGTAAAACTCCTACTG
GGTTTAACAGTCTATTGCAACACATTAGCATTATTAAAAAAAATGTAGATTTAAAAAAATTTATG
TTGCTATACCTGTTAGAATTGACCATTGGGGTTTGCCTTTCTACTGTGTAATTTTTTTATCATGA
TGTGGATCTTGTACTGTTACTTATAGAATTACTATTAGGGTGGCCTTACTCAGCGGGATTTTTC
TTATCCCTTCGGGGTTTCCTTTGCCAGCCAAAGTTGTACCCCTATTGTATAAGTTGATGTTAC
TATTAATTTGGTTGCAATAAAAGAAGATTTGATTTGATAACACACCATCGCACAGCAGGGTG
GAAAGACGCAATAAAATTTAGCAAGCATACCACTGACGTCACTGGTTGGAATGCCAAATCC
TTGTTAAATATCATATACACTCACCTTTCCACAGAAAAATATGGTATAGAAAGAAGTTTCCAAG
GGAGTAAGCAAACGGTTCGTTGAGTTTTAGTCCAGCAGGCGCTCCCATCAGCCAATTCAA
CAGTGACTCAAACTCCACTGCAACCACCTAATGCAAACAGGAATGCACTAGACAAATTACT
TGCTTCACTGTCTAAAGGTCAGTTTTCAACTAATATGTGACCCATCCATACGCTTAGACTTTG
GGGATGGCCCTGGCACGTGCCACCCTCAATAATTGAGAAATGCCCATACTTTCATGCTTTA
AAACCAAAAATCTGAGGATTGTACAACACATGAACCAATATGGCATGTTCAGTCAAAAGCTT
TGGGTGACATAATGCCACTTGACATTATCCAGAATGCAATATAAACCAGGAAGAATTAAATG
CTTTCAAGCTGTATACACATTCAAAATTAATTAGATATGAATTTTTATACTGCAGATCATATAAAA
TAGTAATTTTAGAAGTTTACCTGTATACACATTCATAATTAATTAATTGGATGACCTTTTCTATTG
CAGATCAGTCAAATACTAATTTCTTATGTCCAAATTGTATACACGTACATAATTAATTTGATATAA
ATTTTTCTGTTTCAGATCATATAAAATACTAAATTTATCAGTCAAAACTGTATACACATTTATAGC
TAATTTTATAAGAGTTTTTATACTGTAGAATATATAAAATACTAATTATATAAGTGCATAGACTTTC
ATGACTTTCAAGTTTAGACATTCATGATGATTTTATAAGAAATTTTCCATGGCACAAGAGACAT
CGACGCTAATTCACCTCGGCCCAGGTGATGAAGCGGTCAGCATAAATTCCGATGACGTTG
CTAGACCACAACCAGCTCATCAAACAGACACCAAGAAAGACGTCCAACAGAAGACAATA
CTTGTTGTTCTCGAACCTTAACACAAAACATTAATAGTTGGCGGTAAACACAATAAAAAATTA
GCACCAACAAATGGAATCATGATTTTAATCCTAATGCTGTGCACTCAAATGGCAAACTAAGA
ACATGTAGGCATAGTTTTTTGCATACGTGAATGTCTAATGTGCATATGCCAGCAACCAGATAT
ATGCAAGTACATACCAAGATATTCTGACACAGGATTTTAAGAGCCTTTCACAAGTGCAAAGT
AAACTACTGCAAATTTGTGCCAAAGATTTAAACAACCAGTTTATGACTTTCTATCTGAGATAAA
TTCAGGTGCACAGAGGTCTAGTGGTTAGCGTGCTGGCTTGCAGATCGAAACGCCAGGGTT
AAAATCTCCGCGAGCGCATAATTTTTTTTACAGATTTCTGTTCCACTTGCACCCCATAACCA
ACCCAGCTAAGTTGAGTACACTGACAGTGGGAAATATGCAGAGGCCAAGAAAATACTGTC
ACTAGTATTTCATACCCATGGATGCCTTAGGGACAGCTTAAGGGACTGATCTTCTAAATGGG
AAGCATTATACCTGACGTTGGCTGGTGGAAGAGGTCGAACGCCGCTTCGGGACAGAGCG
GCAGCGTCGGCCAAGTTGGACAGAAATCGACGTTTAATCTGCGTCAGAGTGGCAGGCAT
TTGTAAGACTGCCCTCACTATCGACAATCTAAAAATCCTGCCAGGAAAGAGAAGAAATTTTC
AATTTTACAATCAAATTCAGGAAGCACTAATATGCCTACTAACAGACAAAACAGAACCATTTA

GTCATTACACTGATTTGAAACACTTCATACAATTTTTCATCTAGGAAAATTGAAACAGAAATTTT
AATGCATTAAGTCAATAAAATTCCCCCAAGCTAATACATATCTGAATGTATTACACATTTCATTT
AGTAACATCACAACGTTATTACCTTACAAGCAATCAGGGCTATTTTGTAAAGGAACTCGCTG
GTACGGCATACTGGCAAATAAAAAACAATGTTATGAAATGTTTGAAATTTCAGTGTAAATGACT
AAAAAAGGTCATTTCACTAGTGTTAGCACTGGCAAATATTTTTCAGTACTGGCAAATATTTTTT
TTACAAATAAAACACTGCTAACAATAATCCTTAGAGTTAAAGCCATATGGCAAAGGATCACC
TAATTAACGATGCTAATTTTTGAATTATAATTTTTGAGTATGAACTATCAGTCATATTATGACCAT
GATCAGGTTAAATCGTAGTCTGTGCGCTTCGGCGTGAAACTCCGATGCAGTATCCGTGCTG
TAGTCGGGAGCGGATCTGAGTAGTAGAGGACTTGAAGGGGTGCTATAGAAATGGCCGAAT
GAATGAAATCACAGTTTCATCTATTGATTGATTGATATTTTGCCAAATGTCTAACATGACAATAC
TACACAATGGCTTTTTTCAGAAGAGTCGTACGGCTTCACACAGGACTTTCCATGTCAAGCTA
TATGAGCTCTGTGTGTCATGTCCAAATGCCAATTAGTTATGACAGAGAGGGACCCATGGCTT
GACACAGGATAGTGAATTAGGTAGGAAACACTGGAGTCACAGTTTAAAACAAGATTGGCAC
TTAGGCATGAAACACTGGGAGCCATACTGTTTGACACAGGATAGTCAATTAGAGATGACAC
TCTGGAGGTAACATAGTTCGACACAAGTTTGCCAAAATGGGTCAAGCCACATGACCCCCT
TGTATCATACCTAATATGCAACTAGGCAAGAAACACTGGGATCTAAATCCAGGAAGCAGAG
GAAATAGATCTTACGAATCAACTACAAAGCAACTAACTGGAACATGGCAGACATTTTCGCTT
TACACAGTATTAAAGGAAATGCAAGCACCTCCTTTTCATTGTATTTCAAGCCGAACATTCAC
AAACCTTAAATTTGATAGCCACAGTGATATCTTCAAAATTATACAAACTATCACATAGTTTGGC
AACATGAGAACAACATCTGCAACAAAATGAAGAATTGTTTTCCTTCGAGGCTCTGCAGGAT
CATAGAATTCGGTTTGTTTTTCGTATCCAACACTGCTGAAAAACTGTTGCTGTTTTGCAAGAG
ATCCTAAAATGACTGCTGCATTCGAAGAATCACATTCAACTGCAGAATTGTTTTAATCATCAT
CTCATTCAGCGTTGTAGAGGCAAGAAATGACGCCGACTCATACAGTATTAGAACAAACTCA
AAGGAACAATCTATGATTTGGTAACTGGTGCTAGGATGCCTTCGAACTGATAAGCAGTCTTT
CTTTTTCTCGATATATAACAATGCATTATTCGTCGAACAATTACTGCTTTGTATTTCCTCGGGA
CCGCCAGATTCGCTTAGGACTGCTTCGGGCTCATCTGTGTTCGCCCAAACAGCAACTACA
CGCACATCGCCTGCAGTATTCAGTGAAGAACGATCGGATGAGCGGATGAGGCCAGTAAC
GCAACAGGATCGGTTCACTTGATCGAGGCAACCGATGGCACGAGAACTTTTCTCATGAAG
GCACTCGGTAGGAAAGTAAACACGAAATACGTTCCTGTTGCCTTCCATAGTGACGCGAAG
CCGAGAGTCATAAGACCATATTATCGCAATGCCACAATAGTTAGAATTTAGAAAGCTATCTCA
CGGCTGGTCATGAGAAGGATTAGCTGGCTTATAATGGCGCTCTTGATGCACATGTTCAGCA
AACCAATTATTATTATTCACTGAAAATCGACATCGCTAACATAACATAACTGGGGGCTGCCAT
ACTGGGTGCGTCCACCGCTCGCTCAGCGTACGCTCCGAGTAAGCTCTTGATTTATTTTATT
AGAGCTGTGTTCGCTTCAAGTTGAACACACTCGATGCGTGTTCGACAGGGCTGGTAAAAA
CTGAAGGTTTTTAAATTTTTTAGTTTTTAGGTTTTAATCAGGGCCAATGGGATATCCCATTCGG
GATATTCCGTGATATCCCGGGAATCGAGCAACCCCGAAAATCCGTTGGGATATCCCGGGA
TTTTCGTGAATCATAGTATTTTTCATAAATTTCGCGAAAAGTAAGCTAAAAATGTCCTATATCTC
AGTGCGAATGACTAAAGAAGGTCATGAAAATGTTTGTGCGAATAAAAAAAGGTCATGGAAAA
ATTTTCCTGGACAAATAGAAATTTCTCTTATAATAAGTGTACTTTTCTGTAATGGGACTTTTCGG
GATTTTTTTTAAAAGTCGACCCATTTCGCGGTTATTTATAATAAATTATTAGTATTAGTTGTCAATT
TTTTGTGCGAATGACTACCCAGTCAGCACTGGAATTTTGCCCCATTCATTGCCCGAATGGG
CATTTAGGGCAAGACCTGGGCTTTGGCGGATTTGCCTTTGCCCAAAAGTTGCCCGAATTG
CTCAACACAAATTTGGTAACTGTTAGGACAAAGTTAGGGCAAAGATCTTCTTTCCCTAACGG
GTCCCAAATTTTATGCACAGTAGGGTAATGGCCCCGTTTTATTTGTGAGCAGACTTAAACAC
TGGTCTAAAGTGTTTAAGTCGTTGATGATGGATGGCCCCTTACAATGCATTAGGCTACACTAA
TAACAATGTCTTAGGGCAAATAATGGGGCCCATTTGGCAAACCCAATTAGGGGAGAGCTTA
ATGCTCATTGGCCCCAGCTGGATTCCCAACATTTAATGCACCTATGATTAAGGTAGGACCTA

ATGGTCGCAGGTGTGAACCTCTGCTGAGTGGAGGGTTTTGCGTACCATACTTGGCCCAGT
TACCTCATACCCAAAGTAATGCCAATGTAAAAGTGCAGTTGACATTTCTCAACTGAGAAAGC
TGCATGCCATTGGTCTGTTCAAATCCCTCCAATTCTATCTTAGCCAAAACACCTTTCCCTCA
CATTGGAGAAAAGTGCTCTTTGCATGATGACAGCTATTCGAATAACTACCACACACCTCGG
CCCCGAGTCATGTACATATAATTATGTATGGAAGCAAGCATGTACAATGTACATACATTGTGTA
CCTAGCCATTGCAAAGCACCCTTCAGGAATGTATGTATGTCTGTATGGATGTAAGCATGTATA
AGCAAGCACGCTTTGGCATTTAAACGGCGACACAGTGTCAATTGCTGTGTTGGGAGCGGT
TATGAAAGGCTCATGCTGTGAGAAGTGCCATAGAAATCGATAAATACAATTATAATAAATATTG
TGTACCTGGCCATTGACTTCAGGAACCCTAACAGGTGACCCAACACTTGCTGATCCTAGG
CAATGGTATTACTTCTAATAACATTTTTAACTGCCAAGAGAACGTCACTTTCTATCTGGTATCTT
GCCAGGTGCGTATGCATGAAAGCCAGAACTCTATATATGTAACTATGTATGTATGTAAGTATGT
ATTATCAGGACTTAACTTAAAGAGAACGATGTGTATTGCATGTCGGCCGCAAGTGGACGTC
CACAAGGGGGAAGGGAGTCCGGCTCATGTGGACGCATGTGGACAGGGGAGAGGGGGG
TTGAAAAAATCTGATATTTTTGTGGACATCATAAATGGATGGCCCCTAAAGCAAATTAAACATT
ACAATACATTCAGAAGTACAGATTATTTCTGAACTTCTGTAACCTATAAAAGACAAGCACACA
CACGAATTCTGCATGGCACATTGTAACTATACATGTAATTTCTTGAATTCAACCACAGCACCT
CCAATATTACCATTCATTTTATTTGATACCCAGAATCAAACTTAACGTCTACACTTAATGGCAA
TGTAAGAAAATGCTACCATTGAGAACACAAAACATCTAAATTTTATAGCTCTACTGACTTGGTA
ATTACACAAGATAATATGACATGTAAAAGTCATACCCGCAACTATTTTTCTAGCCAATTTAATAT
AGGCTGTCTTTTAATGTGCAAAGTTCAAAAGAAGCCGATGCCTCGACTACAAACCACACGA
AATATCAAGCATACCCGAGATAATCGTACATGGCTTTATATAAACTTCTGAACATCAGCCAAA
CATCCACACTCTCAACATTAGTGTCTCAGAGCAGGAGACACGGATGTGATGAGAAATAATG
TGACCAGAACACTCACAGATAAAACTCAGTGCCATCTAGATATACTGTGAAAGAAGAGGTA
AAGTCACTTGATCTGGCTCCGGAGAGGCAGCCAATTTTTTGAGTGTTCTAGCCACGAAAAC
GTCAGCCATTAAAAATCGTATGACCACATGCACATGCTTTCGTATACTCTAATAAGCATAAGC
CAAACATATGTTGAAGCACCAGTAATAAAAAAGTTGAAAATACTGTTGTAACATTTACAAGTAA
AGCAATTGTTTAAAACTGTACACTTTTTTCCAGTACTGGTTTACGCTATCAACACAGAATAGA
AAACTCATAAATGTCTAAAAAATTGTTTCTACATCAAGTAATGGTAAACTGAAAACAGTCTGCT
GGTCTAAGAACAATCCTATGTAAGAACAGACAACAAGCACGGAAAATAAAATGTGAAAATTA
GCCTTAAAATTTCTCAGTTTCTCTTCAACGGTTCAGAAATAAGGAGTATTTCTTTACAAAGTGT
AACACCCTTATATACCACCCCAGCAAGTATCCCTTTATAATACATATCATATATACAAACAGA
ACTCATTATTTTAAAGACTCTAATTATTGTGCGGTTTAATTATATTTTGTGCGATATTCAAGAACT
CTTACATGTAATTGCCCCTATACAACATTACAAAATGTTAATTAATTATTACAAGAATGTCAATT
AAAAATTAATTAATTAAAAGAATCAACTTACAAAGATTGTATTATAGAAATGTTAGACAGCATAG
GCCTACATGAAGCGATAGAGTCAAAAACATTTACAATTGGAAAAACATTTGACAGATGCTGA
ATACACTTAACAATAGCATGCATATTATAATTACTTTTCTAAAAAACTTCTTGGAAGAAAGAATG
CATAGAAGTGATATTTACATATAAAAATGTAACAAACAAAAGCAAATGTACAAAGATATATTTAG
GTTAGGTCAGGTCCTGACCTTTAAAGAAACCACATCAAAGTCAACAGAAGCAGAACTACAT
TAATTAACACATTACCACAAAGATCTGCACTGGCTTCCCAATCACGATACGCTCATCAGCA
GCGTGAATCTGGTTTGAAAACTGCAGGGGTCGCTGGAAAGGCCGTCGACCAAATTTCTGA
ATGACCTTTTTTTACTCATTTACACCGAAATTTCTATTTCTCCGTCCAAAAAAATCTGTCTATTT
CTCCAAAGTCAGCACTCTGGAAAGTTTTCTCATCTCCTAACCACAAAATCTCTCCCAACCC
CAGGATTGACGCCCTATGACGCTCATCATTAAAACTTGTCAAATAACTGAAGTTCTTTTAAAG
TTTGCTTTCAAACATCATTTAGTTTAATGGCTTCATTTTTTCTGAAAACAAAATCTTATTTGCCAT
TTGCTTACTCTTATTCTTCAATCCAATTTCTATGAGATCTTTTCTTCCTATTGGTTCCATATCTTT
GCTTTTTTTTCTTCCGCTCTATTAAGTTAATGGACAACTGTTCGTTGTGTCATTCTTTGAGAAG
CTTTCACTCTTCTATCCCAGATGGAATCCAAATCAGCAGCTCCCGTGCCTCCTTGTACATAA

AATAGTAAAAAATAAGAATGTATTTTGATAGTGACCCCAGGGGCGTCCATCCCCTCAATGAT
GCATATTGCATATTCCCCCTATTTCTTTAAAATTTATAAATTTCCCCCCATTCTGGCAACATTTA
TAAATTTCTCCCTATTTTCATTCAATTTAGGTTTTTTGCTAAATCTACGTTTTTTGCTTCCCCACT
GAGTGTCCCCACCAAAACTTCGGAGGGGGGGCATCGATTGTGCAGAGCAACTGAGAGT
GAAGGACTTGCTCAAGGTCCAGACACAGTTACCATCTCATTAGAAATTCAAATCCTCATTGA
GCACATTACCCATCAGCGTTTAGTTATTAAGTCTAGATTCACCTAAGTGCATCTCCCGATAC
CAAATTGTTCGGTGCGAATATGTTACTCATTGCTTAAAGATTAAGAATCATAACCATCATCAAA
CCAACAACCTGGACAAGACAGCTTTTGGAGCCACACTTTTCAACATGGCAACAGCCTTG
CACTTCAATGGAATTCAATACTTCCATATTATTCAACACTTGGTAATTGTTGAAGGAATAAAAA
ACGAATAAAATCTAACAATTAAGAGCAAGCCATAATCCTTCAACAATTCGGCCAAGAGTTTG
AAAACTAACAACATTGACAGAGGTGGTGTTCTCTTTTTGAAGTTACAGCAAAAATGTTCTCTG
CAGTGTACGCACAATTAATTGCCAACTCACAATGAATTTCTCTTGAGTGTTAGCACGTACTCA
CACCACCATCTGGCTCATGCATGACTTAAGATTCACATCAACACAATCAATCAGTCAATCAA
TCAGTCAATAAATCAGTCAATCAAAAATGAGAAGTTCTTCCAAGCATGACCTATTTGACCTAC
GCCAAGGAAAATCATACATCAGTTGGCTTCGAAATCATACATCAGTTGGCTTCGTGACTCCT
GGTTTCGATAAATAACCTAACACCCGAATTAGTTGAGTGTTATCAGAGAAGCAGCAGTCATC
ACTGGTACATGTATTTCTCTTGAGTGTTAGCACGTACATCACCATCTGGCTCATGCATGACTT
AAGATTCACGTCAACACAATCAATCAATCAATCAATCAATCAGTCAATAAATCAGTCAATCAA
CCAACCAACCAATCAATCAATCAATCAATCACTCAATCAATCAATCAATATCGTTTCTACATG
ACTTCCTAAAACTACAGCGGAGTAAACCTAGCTTGGTTGGCCCAAAATAATTAACCAAAGA
TAGCAACAACAAACACAATAACTGTAGGAATGGTCACTAACAGCAACTGCCATAGCTGCTA
AGATAACGCAGAGGTCAAGTATGTTGTCTTACACCAGGGTTAAAAGGCAAGACTGAAATAAT
AAACATCGATGAGTATTAAACAGATGATAGCAATCGTAAATGGTATGATGCCAATACCAATGA
CCATTGCTGAATAATAAAAATAATACTGGGCCAAGTTTTATTGAGGAGCTTCAGCTCTCTCTA
ACACATACACCTGCATTATTTAGTGTTAGCACGTACTCACACCAACACTTCCGGTTTGTCGG
CTTAAATATCTAGACAGGGTCCTGTGAATGGCAGCCTGCCTTGTGGGCCACATTCCTAGGT
TTGGCCGGGTCTCTGGGCATGTCCGGGATGTCCTTCACAGGCTTCCATACTCACAGCGC
ATTGTCTACCATGTATCTGTGCTGGTTCGGCGCTGCATAGAGGGTCTAGCATCACCCTATCT
GTGGGAACTCAGCTGCCCCCATATGTCTCATTGCGCTCTTCTGCACAAGCGGAGTTACTG
GTCCCTCGCACGCGGACAGTTATCCGACAGCACCACACCTTCTCTGTGGCTGGTCCGAG
GTCCTGGAATGGTCTCCCGGTTGCGCTATGTCTGACGCCTGTTCTATATTTCTCTGGCCTTT
AGATCATATAGTTTGACTGAGGTAGGGCTGGGAGGGCTCCTGAATAGAATATACCTTAAAGG
GGCACTATATAAAACTACTGTGATAATAATAATCTGGAGATATAAGGAAAACATAGCATCTAC
CAGCATCTATGTCAATCGTGATATAAAACTTTTAATGCCATCACCTTCCGTACCACTTCTCCC
TTCCCAGAACTTGACATGGTTTAGCCAACCTGGACAATTTATTTCTTCCGAGCTTTTCAAATT
CTCAAAGAATAACATAAACGTTGGAATAAAGTAATAAAATATGTCATCACAGATAGCTGGGCA
CTACCTTAAGGTATAACTGGCAAAACAATAGAAAAACAAAGATTTCCTACCACAATGTAGGC
CTAATGGCAATCTAGTGCTCTGACGTCATTCTTTTTCCGCAAACAGCTTTCCTAAGTCAGAT
CTGAATAAATTCCAGCTGTTCCTAAATCTCATTTCTCCAAAGTTTTAATCCTGGAGATCGGTG
CTTCCATAGGTCGCAAATAATAGAAAGAATAAGCAAGTCCTTGTAATAATTATGCCTAATCCT
TTGGATTTTACCAATTGGCTATGAAATCAAATCACACACTAGTTGTGAAAGAAACACAAATCT
TCAGACCTACATTTGCAAGCAATAAATACTCGCCTGCACTTGCTTTGGCTCCATAGAAAATA
TAGGCATACCAAATACAAACGAATCAAATACTAAGAATTAGGGATGTACCGGTTATCCATTC
GGTACTCAGTATCTGCCGAGTATCTCACTACTCAGTATTACTCGGACTCTGCAAAATAATTAG
CCAGAGTAAACGAAATTTTTTCAATGTAAAATATTGAACAGAATCGTAGATAAAAATATTTTGTG
CCGAATTAATAACATACAAAGTTACAAACTTGTGTTGTGTGTTCAGTCTTCTTCTTCTTTCTTCT
CTTCCGTCGGCTGCGCCGACGATGTGGCTCTCACAAGACTCCTCCAGACCTCTTTTTTTG

TCTTTTTGGTCGATTTACCAGTCCTGATAGTTTTCGGATATTTTTCTCCTTCTGGTCCTCCCTG
ACATTGCCCCTCCAGATTCATCATGTAAGAGTTTCTTGGTCTCTTGCCAGGCAGTGACCAAT
CCTCTCCTGGTAGGGTAAGGAGGCCGGTGGGTCTTAAAAGACCTCTTTCGCCCTAAGGTG
TCCTTCCAACACCTGCCGTCTGCATTAGCCAGCTTTTCAGGGTTCCTGGTGAGCCTTTCCA
CCTCGTGTAGCGCCCTAGGAGGTTCTTCAGCCAATGGCCTTCAACCCGATGGCATAGCC
CCACCAGGCATTCCCCTACTTGGCTGTTCACCCCACACCACGATGAGGTACAGAACGG
GGGGGCTGTGTTCAGTATTTTCTTATATTTACTGCTTCTCGTTTGCCTGAATAGGCCGTGCAT
TTCCGCTGAAAGCCACTTAATTTTGCTAATTTGTTAAATACTCGGACTTGGCAATAAACCACC
TTAGAACTCAGTACCCGGTACTCGGTAAAAAAATTGACCTCGGTACATCCCTACTAAGAATA
TTTACTCAACAACTGAATAGTGCTCATATCTTTCAGTGGTAATACAAAAAGCACAATAAACAC
ATACAAAATACACACATGCACAAGCGGCTAGCAAACACAGTTAGCGAACACAGTTTAATTC
TTAAATTTTCTTAAACTCCATTACAATTACTGAAAGACAGATAACTTTCAAATTATTAAAGACAT
CAGTAATGGAATTCTAACGAGGTTGCCGTTTCCGAAGTAGACAGTAGCTTACAATACAAAAA
TTCTTTGACTAGATGAAGTTCGTAAACAAATCAAAGACGAGATATATAAAAAATAATGCACAGT
AGAATTTCAGTGGCAAAATGGTTACTTGTGCAGGCCAAGGACAATCTTGGTATGTATGTCCG
AACAGGCAATACAGCTACTTGAGCAGGCCGAGGGCTTTCTTCGTGTGCTCATAGACCACG
TAGCTAATGCTGACGGCAGGTAGCACCTTTATAAAATTCGGGACAAGGCCTCGGTACAAG
CCTCGAAGTCCCTCCCTCTTTATAATGTTTCGCATTAAGGTCACAAATCCCTCTCGTGTGTC
GTCGGTTGCTGAAAACAATTATTCATAACCATGATAATATCAGCACAATATGCAGTTCTCATAA
TGAGCTATATAAATCTTAACACCTTATCCTTATCTTAACACCTTATTAAGCCTGAGGCTTAACG
CAGTGCTAAGTTAGGAATCCATGCAGCCAAAAAATTGAGCCCAATCATAACAACCTCACGA
AGATAACCATTATGATGATGATGGTGATCAATATGCCTGATAGCTGCCTGTGGCGCTTCCGT
GTGAAATTCCGGCACAGTAGCCGTGCTGTGTCGGGAGCGCCTCTGAGTAGTAGTGGACTT
GAAGAGGCACTATAGAAACAGCCTGAATGAACGAATGAAAGTATAGATCAAGGATGTAGCC
AGAAAAAATTTAATTTCTTTTGAACATTTCGGTGGGGGTATGCATTGGAGTTTTGTTGTATCAT
GATATAGTTTTAGGCTTTTCTAAGATTAAAGCATTCATTTTGGGGTGGGGGGTTTAACCCGTA
AAACCACACCCATGATACAGATGCCTTGCTGATGGACATGCTTGGTGACAATGATAATGAT
GACGATGATGACAAAACTTGCTGCTGCTGCTGAGGATGATGATGTTGGAGTAAGGCAGAC
GAATAATACTTGATGAACCGTGTCGAACCTTGAGCCTGAAGCCTGGTTCTGACGAGAGCC
AGAGGATAGCTGGCCAGTTGGCCGCAGGTGCTGCTGATCGACCCACAAGCCAATAGAA
CGGCAACACCTGGATTGTTTTTGTTCGGATGACGTCGAAGGTACATCAGCTTCAGAGTCTT
CAAAACATAAATCAATTCATGCATGACATGCTTCCAATCCAGTAAATCATAAATAGAAACCCA
AATATCTGGAGCGCTACCCTAAAGCCAGGCACAGAGCAACACCTTATTCAGGAGCAACA
CAGTGGTCTATAAGCAACAAGCATACTCAATGCTCATGCTTGGATACAAGTGAGTTCAACAG
TGAGTAATGAATCCAGGCAAATTGTTTACCCACATATGCTTCTGTCAGAAAGCAGGCTAATG
TTAAATGTAAAAGGGAAGACATGCAATGCACTAGCACCTGTCCAGGACCTTCAAATTTGTC
CATTGCCAGCTCAAGGGGTGAAGGGATTAAGTTCAGTCCATTGCCGGCATTGGTTTCAATT
GCCAGCTAAAGGGGTGAAGGGATTTGGTTCAGTCCATTGGCAACTCAAGGGATTTGGTTC
ATGCCAATTGACAACTCAAGGGGGAAGGGATTTGGTTCAGTCCATTGGCAACTCAAGGGG
TGAAGGGATTGGGATTCATCCATTGGCAGCTCAAAGGGTGTAGGGATTCAGTCCATTGGC
AACTCAAGGGGGAAGGGATTTGGTTCAGTCCATTGGCAACTCAAGGGGGGAAGGGATTT
GGTTCATGCCAATTGACAACTCAAGGGGGAAGGGATTTGGTTCAGTCCATTGGCAACTCA
AGGGGTGAAGGGATTGGGATTCATCCATTGGCAGCTCAAAGGGTGTAGGGATTCAGTCCA
TTGGCGGCTCAAGAGGTGAAGAGATTGGGTTCAGTCCATTGCCGACTAAAGGGATGAAGG
GATTTGGTTCAGTCCATTGGCAACTCAAGGGGGAAGGGATTTGGTTCAGTCCATTGGCAA
CTCAAGGGGTGAAGGGATTGGGATTCATCCATTGGCAACTCAAAGGGTGTAGGGATTCAG
TCCATTGGCGACTCAAGAGGTGAAGAGAATGGGTTCAGTCCATTGCCGGCTAAAGGGGT

GAAGGGATTTGGTTCAGTCCATTGCCGGCAAAAGGGGTGAGGGCATTGGAATTCATCCAT
TGCTCGCTCAATGGCAAAGTAAAGGAAATGAACTTATCCTCAAAGTCCCACATGGGATATA
GGAGTTTCGCTTACTCCACTCAGTCAGGGCGGTTGTCTGAGTCCAGGCAAACTTCGTGTA
ACTGAGTCTGTCCAAGCATTTAAAAGTATATGATCACTGTGCATATTTACATATAGACCAGGTG
TGCCGTAGCTGCTATTATCAATTGCGCCAGCTGAGGGTCATTGCTCGCTCTTTGACCTTTAA
TGCTGCTGTCTCCCTTGTTCATGCTTTTGTGTTCAGCCGCCTTGATTATTGCAGTTCCATCTT
CGTTGGTCTCCCGGGGGTTCGGATGGAGAAGTTGAGGCGGGTCCACCGGGCTGCGGC
TCGACTTATTGGCGGGTTCAGGAAGTTTGACCACATATCCCACTATATGCGGAATGTGTTGC
ACTGGCTTCCATTCCCACAACACATCTTTTACAGGATCGCGTCCTTGGTGTGGCGGTGCTT
GTCAGGCTGGGCGCCCTCCTATCTGCGCGAGCTCTGCCGTCCTCTCTCCTCATGTTCAG
GCCGTCGGGCACTCCGGTCCTCTGCTCACGGTAATTTGGTGGTCCCATTTGCCCGCTCT
GCGACAATGCAAACCTGTTCATTTTCTGTGGTTGGTCCAAAAACCTGGAATGGACTTCCAG
CAGATCTAAGGCACCTTCCAAACGGTGCCTGTTCTCAATTCCACCACCTTCTAAAGACTGT
TCTTTTCCGCTTGGCCTGGGTCGGGAGCGCCTCTGAGTAGGTATCTTGAAGGGGCGCTAT
ATAAATTTTGATTGATTGATTGATTGACACTATGAGCATGCCATGTGCGTGCTTGCAAAGAAT
CTGGCAGCCTCTGAGGGACAGTTAGTACCCAAAATTAATGGGGTGTAAGCGTTGTCTAAAA
AGCCAACCTCGTAGATGGTGAGGTCGATGCCAGCGTAGGGTATGATGCCGATGAGATTTG
GAAGGTAGCCCCTGAAGAAAGCACGAACGCCCTCTCTCTTGAAGATCTTAACAGTGCAGT
CAACGAGGCCTTTGTATTGACCCGTTTTGGACACGGCCAGACGCGTCTTAACAACCTACA
AACACACAGAATGGTATAGAGTTAACAACAGTCAACTTACAGATAACCACAAGTAAATAAGT
AAGTAAATACTATGATTTGTATAGCATCGTAACCGTTAGTAGCAAGCCACTTCTAGGATGCTT
TACAGGACTGTCCACTGTCAGCACAATATCCAACTCATTCATTTATTCAATCAAGCTATTTCTA
TAGTGCCTCTTCAAGTCCACTACTATTCAAATGCGATCCCAACTACAGCATTGATACTGTGT
CAGAGTTAACACGCCGAAGCGCTACAGGCAACTGCGAGTGAAGGACTTGCCCAAGGTC
ACTACATGGCGAATGGAGTTGGATTAATGCTCGAGATTATACAAAAAGCGCAGTTTCAACTG
GGCTTTGAGAGCAAGAAGGGTTATGGTTTTCTCCGTCAGAATTGAAAATGATTAATTAAGTG
GACGACAATAACAAAGAAAATAAAGATGATGATTACGACAATGACAACACGGACTAGCAGA
AGAAAGATGCATATCTTTGTTGTGACCTCCATTCTTACCACAAAGAAGAGAACAGTGACAAT
TACCAGTTTGACCATATAATCCTACCATTTAATCGACAATGATAAAATTATCAATCAGGCGGTA
TTTGTTGTACCAATGACAGTTATGCTGTAGCTATCTCAATTAGAACAGATTACAGCAAGACGT
GAAGTCTCTCCGATTAAAGACAGAGCATACTGCTGATCGAAAGAAGCGGAGAGGAAGGAT
CCGAGTGGCTGACCCCTCCCCTCTGAGGTATTAATTCAAGCCGGAAGGAGATTATTATATC
TCAATTAGAACAGATAACAATGACAATTGTTAGAATGATTATGCCGCCTCCATTAAAAAAGAT
AATGATCACCAATACAATGATGTTACTACCTCCATTGGGAAGATTGCTGTCTGTGCGAACGC
CCCAGCCATGGACCCGGCTGCAAACCGTTCGATGATTGTAATATTAAGCACATCTTTACCT
TTGAGCAACTTCTTTATCTGTGACAGCAGACAACCAAACAGACAACAATAATATCATCCATAT
GACACAGCTTCAAACGTCTCGGTTACATGTGCATGCGTGAAAATCAAAGCAGAAAACAATT
GAGGGACATGGTGGCGCCTTGGTTGAGTCGATAGCTTTCAACCGGAGGATCGTGGGTTC
GACTCCCGCTCTAGCCGCCATGTAGGGACCTTAGGCAAGTCCTTTACCTACAGTTGCCTG
TGTGCTTTGGCGTGAAACTCCGATACAGTATCCGTGCTGTAGTCGGGAGCGCCTCTGAGT
AGTAGAGGACTTGAAGGGGCGCTATAGAATGAATGAGGACACGAATTCGAAAACCTGCTA
AGTAGAAATTCTTCAGAAATGAGCTAATGTGGAAATTTGCTATCGAAGGTACGAGCTCCTAG
CAAATCGTGATCTAAACTTTACACCCCAATCGCTTTACAATGCACATTTATGCAAACAGTGG
TCTCCACTGGCATCTTAGTAACATTGACTATAATTATTCTGAACAATTGTCTTAGCATCTACATA
GACAATTTACTACCTAATTTTAGCTTTAATTTCTATTTCTCTAATTCAATTGTTTGTAATTTTGTCA
GCCGGGATACACTGATCTATTAATCAGCCATAAAACGGAAATTGATTCTGATTAGTATTAAGC
AAAGGTCAGTACCACTGCTGTCATCATCACCGTGACACCAATCACCATAGCAACAATAAC

CAACATGACCACAAACAAAACCACCACCACAACCACCACCACTATTATCATGAATATGCT<br>GATAAGATATTGGTAATTATCATGACATAATATCATTTAAGTGAGCTAAAAAGAAGCCACAAAT<br>TCTGTCTCATTACGCATACCTCTTCATATGCTCCAAACTTGAGCGCTGACTCTGGGGCTATT<br>TTGAGCACGCTCATCCCGTTCCCTCGCCACAGAGATCGCACTCCACCTTCCTTAATCATAT<br>TCTTGAATGCATCCCAGATCCTCATTTCTGTCTTTGACCCGTAGACCTGAAGCATGATCTTG<br>AGGCGATCGAGAGGCGCAGTGGCTGTTCGGGAGACAGCTCCTGCAACGCCACCGGCC<br>ACAAGGTGTCGCCACGATATTCCTTTTGGCTGGAATTCTGGAGGAATGGTCAGCTGCTCG<br>CCTATATCAATCATCTCCACAAAAGTACAATTACATTCATTATCACTAATAAATTACATTTGTAA<br>ATTTAATCAAAGAAGCAGTCAACCATCTGGGGTAGTGAGAATTGTACCCAGCTTTTCTGCG<br>CTACACCAACAAAAGCAAAAATTTCCTTCAACGTTTATCTCTGATTCCTCGCCAACTCATCT<br>ATAATACGTACAATGACAGTTGACGGTTGGTGGAAAGATTCGACAGCAGGGCAGAGGGCT<br>GAGCACTCACATTCTCATATGCTGAGGCTAAGAAAATGTAGGTGCCATCACTTCATGTCCAT<br>GGCTGCCTACCAGGCTAGTTTAAAAGACTGCTCATCATTTTCTTCTTCGTTCACTACATCAG<br>GAGCACTTCTGAGTAGTTTTCTTGCATGTAACTTATTTTTATTTATTTTTTCCGGATATTTCTATAG<br>CGCCTCTTCAAGTCAACTACTAACTCAGAGGTGCACCCGACTACAGCACTGATACTAAGT<br>CGGGGTTAATACGCAGAAGCGCTACAGGCAACTGTGAGTGAAGGACTTGCCCAAGGTCC<br>CTATGTGGCGGCTAGAGTGAGATTCGAACGTGCGACCCTCTGGACGCAAGGCACCAAG<br>CTTACCACCGAGCCACCACACCCCATAATTGTCTGATTAGTGATTACAATACAACGCTCAA<br>CTTAACAACAGACGACAGTTTTGAGGATGAAATACCAGTCGACTCCTCCAGATGTCGATCA<br>TATCCTCCAAGGAATCAGCCGGATAGATTCCGAAATAATCTCTCCACTCGTGCCACTCAAT<br>CTCAAGATCATTGTTCTTATCCATTCTACAAAAACAATGCTACACATTTAAGACAACTGTCAA<br>GAAAACATGTGACCTCGACAGAAAAAGGGACTGCTTCACCCGTAAAACTGACCTTTTGGT<br>AATACAGTAAATTCTGATTTTTGCCAATATTAGTACGTATACAGATAAGACTCTCTCTGGCTATA<br>AAAATGATCAATGAACTGAATTGAACTGGTGAACTCTTTAATCCCACATGATACATTAAGGCC<br>AAAATTCGCTCTTAATGCACTGGTTAGAAATAGTTTGAAAATTTATAGGTGATTTCCTGCCAAA<br>TGACACTTCACATTTCCATTAAATATGGCATAGATTTTTGGCCAAAACATCCTGAAATGTTTAA<br>CCTAATAATAAGCTT
>318A10
AAGCTTCATTGGTATCACGGAGTCCCTAATGACTTAAACGCTATGCAGCCAATGGCTTGGG<br>TAGTAGATGGGGTAGGTAATATACCCTGAAAAGCGCGTTTGGATGGTTTGGGTCGGAGTTC<br>TTCATTTCAGCCCCCAAACTCGAGAAAATTTCAGCTCTTGAAGTACAGTGCAATTATAAGTG<br>AAATTTGATGAGGTATCACAGAGTCCTTAATGACTTAAACGGTATGCAGCCAATCATTTTGGG<br>TAGTAGATGGGGGTGGGTTTATACCCTGAGAAGCGTTTTCGGAGGGTTAGGGTCTGAGTTT<br>TTCATTTCAGATCCCCCGCTTTAACGTAATTTCGGCTCTTGAGAATTATATGTGAAGTTTCAC<br>GAGGTATCACGGAGTTCCTAATGACTTAAACGCTATACTGCCCGGCATTTGGGGTAGTAGA<br>TGGGTTGGGTAATATGCCCTGAAAAGCGCTTTTAGATGGTTAAGGTCTGAGTTCTTCATTTCA<br>GCCTCTCAACTTAGAGATATTATCTTGCATTCATATGGGAAGTTTGATGATGTTTCACGGAGA<br>CGCTAATAACTTAAACGTTATACCGCTAACTGTCTTGGGTACTACATAGGGTGGGTAACATA<br>CCATATTAAGTGATTTTTGATGGTTAGGGTCTGAGTTCTTAATCTCAGCACCCTAACTTTGAG<br>CAGTTTTCATGCATTTATATGCGAAGTCTGGAGGGGGTTGGACGAGACACTAACAACTTAA<br>GCGATACACTGCCCATCATTTTGGGTAGTACATGGGGTGTGTACTATACCATGTAAAGCGAT<br>TTTGGATGGTTAGGGTCTGAGGTCTTCATTTCAGCCCCCCCACTTCAACGAAATTTCAGTTC<br>TTGAAGTACAGTACAATTATGTGTGAAGCTTCATTGGTATCACGGAGTCCCTAATGACTTAAA<br>CGCTATGCAACCAAAGATTTTGGGTAGTAGATGGGGTAGGTAATACACCCTGAAAATCGCT<br>TTCGGATGGTTAGGGTCTGAGTCCTTCATTTCAGCCCCCCAAAGTCGAGAAAACTTCAGCT<br>TTTGAAGTACAGTGCAATTATATGTGAAGTTTGATGAGGTATGACGGAGTCCCTAATGACTTA<br>AACGTTATGCAACCAATGATTTTGGGTAGCAGATGCGTTGGATAATATACCCTGTAAAGCGC

GTATGGATGGTTAGGGTCTGAGTTCTTTATTTCAACCCTCCCCCAAGTCGAGGAAACCCAG
CTTTCGAAGTACAGTGCAATTATATGTGAGATTTGATGAGGTATCACGGAGTGCCTATTGATTT
AAACGCTATGCTATCAATCAGTTTGGCTAGTAGATGGGGTAGGTAATTTACCCAGGAAAGC
GCTTTTGGATGGTTAGGGTCTGAGTTCTTCATTTCAGCCCCCCAAACTCGAGGAAACTTCA
GCTTTTGAAGTACAGTGCAATTATATGTGAAATTTGATGAGGTATGAGGGAGTCCCTAATGAC
TTAAACGCTATGCAACCAAAGATTTTGGGTAGTAGATCGGGTGGGTAATATACCCTGAAAAT
CGCTTTCGGATGGTTAGGGTCTGAGTCCTTCATTTCAGCCCCCCAAAGTCGAGGAATATTC
ATCTGTTAAAGAACAATGCAATTATATGTGAAGTTTGATGAGGTATGACGGAGTCCCTAATGA
ATTAAACGCTATGCAACCAAAGAATTTGGGTAGTAGATGGGGTGGGTAATATACCCTGAAAA
TCGCTTTCGGATGGTTGGGGTCTGAGTCCTTCATTTCAGCCCCCAAACTCGAGGAATATTC
ATCTGTTAAAGAACAATGCAATTATATGTGAAGTTTGATGAGGTATGACGGAGTCCCTAATGA
CTTAAACGTTATGCAACCAATGATTTTGGGTAGAAGATGGGTTGGATAATATACCCTGTAAAG
CGCGTATGGATGGTTAGGGTCTGAGTTCTTTATTTCAACCCTCCCCCAAGTCGAGGAAACA
CAGCTTTCGAAGTACAGTGCAATTATATGTGAGATTTGATGAGGTATCACGGAGTGCCTATTG
ATTTAAACGCTATGCTATCAATCAGTTTGGCTAGTCGATGGGGTAGGTAATTTACCCAGGAA
AGCGCCTTTGGATGGTTAGGGTCTGAGTTCTTCATTTCAGCCCCCCAAACTCGAGGAAAC
TTCAGCTTTTGAAGTACAGTGCAATTATATGTGAAATTTGATGAGGTATGACGGAGTCCCTAAT
GACTTAAACGTTATGCAACAAATGATTTTGGGTAGTAGATGGGGTGGGTAATATAACCTGAC
AATCGCTTTCGGATGGGTAGGGTCTGAGTTCTTCATTTCAGACCTCCCACTTCAACGAAATT
TCAGTTCTTGAAGTACAGTACAATTATGTGTGAAGCTTCATTGGTATCACGGAGTCCCTAATG
ACTTAAACGCTATGCAGCCAATGGCTTGGGTAGTAGATGGGGTAGGTAATATACCCTGAAA
AGCGCGTTTGGATGGTTTGGGTCGGAGTTCTTCATTTCAGCCCCCAAACTCGAGAAAATTT
CAGCTCTTGAAGTACAGTGCAATTATAAGTGAAATTTGATGAGGTATCACAGAGTCCTTAATG
ACTTAAACGGTATGCAGCCAATCATTTTGGGTAGTAGATGGGGGTGGGTTTATACCCTGAG
AAGCGTTTTCGGAGGGTTAGGGTCTGAGTTTTTCATTTCAGATCCCCCGCTTTAACGTAATTT
CGGCTCTTGAGAATTATATGTGAAGTTTCACGAGGTATCACGGAGTTCCTAATGACTTAAAC
GCTATACTGCCCGGCATTTGGGGTAGTAGATGGGTTGGGTAATATGCCCTGAAAAGCGCTT
TTAGATGGTTAAGGTCTGAGTTCTTCATTTCAGCCTCTCAACTTAGAGCAATTATCTTGCATTC
ATATGGGAAGTTTGATGATGTTTCACGGAGACGCTAATAACTTAAACGTTATACCGCTAACTG
TCTTGGGTACTACATAGGGTGGGTAACATACCATATTAAGTGATTTTTGATGGTTAGGGTCTG
AGTTCTTAATCTCAGCACCCTAACTTTGAGCAGTTTTCATGCATTTATATGCGAAGTCTGGAG
GGGGTTGGACGAGACACTAACAACTTAAGCGATACACTGCCCATCATTTTGGGTAGTACAT
GGGGTGTGTACTATACCATGTAAAGCGATTTTGGATGGTTAGGGTCTGAGGTCTTCATTTCA
GCCCCCATCTTAGAAGCCCTTGGTTAACTGGGGAGCTCACTCGCCCAGCGAATGTGAGG
CTAGAAAACGAATCCCAATCTGGTCCTCAAGTTGACAGACATCATGTACTTTCCATTTCCGT
TTATCTAAAGATAGCTAGTTGTGTTTACTATTTTGTCGAGTTGCATCAAACTCTTATTTTCGCGA
GGGGCTTTGTGAGCCAACGATGTTATGTTGCAGTCGTAATTCTAGTGTCCGCGAATCACTTT
TTCCAGTAGTAGTTTAGCGATATTCTCCTTACTTTTGTGGCCCGAGAAAGCGTCGGTCAATC
ATGAGTGGTGGATTAGCCCCCAGTAAAAGCACCGTTTACGTGTCTAATCTACCTTTCTCGCT
AACGAACAATGATGTTCACCAGTTATTTGCAAAATACGGACGTGTTGCTAAGTATGAACTCT
CAGTCTCATTATTCATATTAACAAGCTTAAGCGCAGTGGTTTTCTTCCAATGTCTGCTCCCTC
TCCCAATGAAGAACTTCAGAGCTGACAAAATCGCAACAATGATTACATAAAGGTTACATGTA
AATCGATTACATTTGCCCTACTACTAATCCTAAGACCCTCGCGATAAGACTTTAACGATTCAT
AAAAAAGTAAAATTATAAAAGTTGTGTGCGAGTGGGATTTGAACCTGGAATGTCAACGTGGG
AGGCGAGATGATAGATTAACTGTCTGCCTTCGGGGAAGCCAGACGGTTAATCTATCATCTC
CGCCCTAGGGCCTATGGGTTCCAGCTCCCTACTAAGGACGACAGAAACTTTATTCCTCGC
CTCCTATACAAAGACATATACTAATCTCTGCTTTGACTGCTTGGTGACAATGTTTTCCCCTTGT

ATTCTAAGACAATGTAATCGATTTACATGTAACCTTTCTGTAAACATTGTTGCGTTTTTGTCAGC
TCCGAAGTTCTTCATTAAACGATTATTGATTGATTATCGCGGCTGTACTTTTGTCTTTCAAGCG
TATGAAATCTGCCTGTGTGTTATCCTCGTGAAACGACCGATAGTCATAATTATAAACAACGAA
ATGTCTAGGTGTCCTAAAGTTGGACTCCCAGTATCAGGAAATCTAGGAGTCCAAATGTCTCA
TGGGAAGTCGTGTCAAGTAGGCTTAGTTATTACATCCAAATTGTATAGTGCAGCGGTAGCTC
AGTCATTAAGGCATTCGCCTTCCACGTTGCACCTTCCAGGTTCAAATCCCACTCGCGCAC
AACTTTTATAATTTTACTTTTTTTATGAAGCGTTAGGTCTTAGGGTTAGTAGTAGGGCAAGTTTA
GTCATTACAGACATTCATGACTTTACATAAGTTTATTTTTTAGCATCTTGACGGTCGGTTAAGG
GTTGTTTAGGGTCAGTTTGAGGGTCGTTTAAGGCCAGGTTAAGCGTCATGAGGGTCTTAGG
GTTAGTATTAGGGCTAGTTTAGTCATTAGACACAAATTTCAGCTTAGATATGCCTGTTATTCAC
AAATCATATGAAATTCAAACTCCTAGTTTTTCTCATATTGGGAGTCCGACATTTGGACTCCTG
GGCACAGTCTATAAACAATAGGCCTACTACGAAAATTCAAAAAAACGACTTTTCGGTCAGTA
TCCACCAAATGAGTATGTTTACCATATATTCTTGTATATCTTGAAAGTTAGATTTCCACAAATAA
TTTTTCTTTTGCTTAACATGTAGGCCTACTCATAATTATGCTGACTGGACTTCTGAATTCTTGTG
AATATGTTGATTACAGGGTTACGATTGCCAAAGACAGAGATACGAGACAGAGCAGGGGAG
TGGCATTTGTGCTATTTGTGGAAAAAGAGGCGGCTCAGAAAGCAATTCAGGATATGAATAAT
ACCCAGGTTGACCCTTTGTCTTAAAATTGCAAATAAGATATGTACTGTTCATCAAAGGTGCTT
ATAATGGCTTAAAAACATAAAACCCTAGGATATCTTGGTTTTTGTTTAGTAGCATTTTAGTGCA
CAAATTATATACATAACAAACAGACAACACAGATGCATGTTGTATACCTAACGATGTGCACAT
TTTTTCGGTTTTTGTTTGTTACATTGTATGCCCACATACATAAAATGCTCAACACATGAATGTTG
TACCTGGATTCTAAGGCTATCTTGAAGCCAAGTTATGCTGTAGAAATGTGTCGATATTGTTTTT
TGCCAATTATTCCGATATAGCCGATAATTGAAACAGCCTATACAGATTATGCGGCCGATTATG
ACACCGTTTATAAAAGGCCGATTATGACCGATATTTTCAGCGAAAATTCGAGATTTCCCTATC
AAAATTTCCGACGACCTTCTTTTTAGTCATTGACTACAAATAATGACCGTTTTCAGTTTTCTAC
ATGTAACTTTCAGTTTTCTAATGACTTTTTAGTCCTCGACTAAGAGAAGTTCCCAAAAATAAAA
ATTCTTGAAATGATGATATGCAGTATTGGCATGATATGCAGTATTGGCGGTGCAATTATTCATA
CCGATTACAACCGATTGTTTTAATAATCTGCCCGATTATGCATATGGCCGATGTATCAGTACA
GCTCTATTACACTGTGCTGTTAAATTAAGTTATTGATTGTCTTGGTTGAACTTTGATGCAGCTG
GGAAGTCGAACAATCAAGTGCAGCATCGCAAAAGACAACGGCCGAACGACTGAGTTCAT
TAAACGTAAAATCTACAAAGACAAATCACGATGCTATGAATGCGGTGTAGGTATAGTTGATAT
GTTTCGTCACAGGCTCAATGGCATTGTTTATATATTTATTTTTCATGTCTTTCTTCAGAGTAATTA
TTACACGTGCATAGTTAGTAAATCAGAATTCAGTTCAGTTTAGTGATCCCAAAATGACATGAC
ATAATAATCAAATAATACAATGCATATTGAGGGGATACAACGAGTGACCCAGAAGGGACCA
CGGTCCCGCTAATTTAGGGTCATCCATATACCATAGACAGATAAAAATGACAACAGGTAGA
ACAATATACTTTGAACAAAGTAAGTAATTAGCTTAGTTTAACAACAAAAACCTAATCTAGTCAA
ACAAACAAAAAACATGTGTATTTATGACTTGGATATGTAGAAAAAATGATAAATGTAAGTTACTA
TAAGTTTATAGAGGTTGTGTGTTCTGAATTGCTCCTCAAAATCTAATTCCTTCTGTGTTCCTTCA
GTTCATTGAAATCTAAGCTTATATAGTGTTACCTGTCTTTTCTTCAACTATTTTCAAACCAAGGA
TCAGCTGCACCAAATAACAATCTTGCTCCAACTACTTGTTGTCTCTGGTTTACCTTGTGAACA
TGTGCACAGCGCCATTTCCTTCGGCCTTGGCTGAGCTATGGTGCTATACAAATCGGGCCA
ATCGGCTTATCCTATCCATGACTTCTTCAGTCTGTGTCATATTACATCTTCCTTGGGCTCTTTT
ACCAGCAACTGTGTCTTCTATTATCTCTCCAGGCTGTCTCCACTCTTCCATATCACTTGCAC
GAAATACGAAAGCTTTTGTATTTCTTTCAAATTTCTGATATTATCAGCACGTTAAGTTGGTTCTA
TTGAGACTGCAAGACGGGTTTGAATGTTTTGGGGTTTAGTTTAATGCTGGATTCTGAGTACAT
TTGGTCTTATCTTGAATTTGATTTGTTGCTTCCATGGGGAAACTAATAAAATTCACAAATTTTAA
ATATTTGGTCAACTTTATCCAAATGTAATTTGTGAAGTTAAGAATAACGCTTTATGTGGAAATAT
GGACATGTATTTTGGAGAAGGTAACTACTAAACCTTCTTTGAATAAATGTAAATTTTGGTAATTG

TTGTGTTTGTCTATGTTACTATTCTATAGGAGGAGGGACACTTAAGTTATGCCTGCACAAAGA
ATGTTCTTGGCCCAAGGGAGCCTCCACCAAAGAAGAAGAAGAAGCAGAAGTCAGAGGG
AAATGACAGGTGCTTAAAATCTCTTAGCTGGGGAATTTGTATGAAATAAGTCTGTTTTTGGATG
AGTATTCTCATCCTGCCCAATTAAGAGATTAAATAATTTCTTAGGTTGGATCCTGTGCCCCCTTT
TTTATACCATTTACACTAATTTTTAGATCTTTTATTTGTTCAAGAATAATTACTTTCATCTTGTTAAT
GAAGTCTGTATTAGGGTTTTAAGTATAAGCTTCTTTCATTCATTCGTTATGAGGATTTATATAGC
ACACCTTCAAAACCACTGCTCAAGGGTGCTTTCCCAACCCCAGCACGACTGAAAAGGAC
AATTTTGCAATGCTTGGTATGCTGATTAGTTGAAATGGGTGACCTTGGGGTGTGATGTGTTTA
GATCGCTGTGATCAAATCTTTTCTTTTCTAAGTGCCACCGATTAATACTGGTTACCTAGGTGAT
GTAGTGTTGCTATATAAATAAACGAGAAATCTAACAGTGAAATCTAACCGTGTATTGTCTACAA
ATTAAACAGAATGAATTTTACGTTCTCGGTTATCTTTAATGTCCTGAGTGATCATCAGAAGTCA
TTTGAAGGGCCTTTGTAGCCCAACTATCTTATTTGCTTAGTTACAGTAACATTCATTTAATAATA
ATTCAAACTGACAAATATGCAGTAAGATTACACAGTACAGTGTGGGACATGGTGGCGCATT
GGTTGAGTCGATAGCTTTCAACCAGAGGGTCGTGGGTTCGACTCCCGCTCTAGCCGCCA
TGTAGGGACCTTGGGCAAGTCCTTTACCTACAGTTGCCTGTGAGCTTCGGCGTGAAACTC
CGATACAGTATCCGTGCTGTAGTCGGGAGCGCCTCTGAGTAGCAGAGGACTTGAAGGGG
CGCTGTAGAAATGGCCGAATGAATGAATGAATGAAAGTACAGTAGTTACAGATAATAACAAG
TTTAAGGTGCAGTCACCCCTTGAGAGAGGTTGGCTTGGTCAGCCTAAAAGAGCTAGTGTG
CCACCATGTTTCCATTTGACTACCTCTCTGCCCCTTATATAGTTTACCTGGTGCTTAGTTGGT
TATTCACATCTGGTGTCTAGCTGTACTTACTTATGGCTGCACATGCTGTTTCAATGCATTTCAT
TTGAATTTGTTCAATATTTGATTTGCAGAGAGGAAGAAAATTCCGATGAAGATGGTCCTCCAG
GACAACCTGAGGACGACACTTTTAAGGCCGCCATTTTATATCAGGTTTGCAAAATTGATATT
AATTGCGTACGTAAAATTTATGGGCTATTACTTCATGTACTTTCTTCTTCTCTTAAAAATAATAAG
GAGACTGAAATTTCATGAGATTTTGTGACTGTGTTCATGTAAATACATATTTATAAAATAATCTGA
TATATAAATATTCTTGCTAGATTTCTCTCTCTCTCTCTCTCTCTCTCTCTCTCTCTCTCTCTCT
CTCTCTCTCTGCCTTTGTGCATGTCTCAATCTGTCTCTCTGTATATCTCTTGGTATTTCAGGATA
CATTTAAAAAATCACCTGGAATGGTTGAATATATGTTTTATGTGCTTTGGTTTCTTTACAGCCAA
ATTTCGGAGAAATGTTTTGGTCATTCCCAGGGTGTCCATCCCCCCTGAGGCAATGATGCAT
TTTCCCTCTGTTTCAGATTCCCCCTATTTCCGAAAAAATTTTCAGACTCCATGGAAAATATTC
CCAATTTTTCCTTTTCCCAAACAATTTCCTGATTTTCATCTGCCAAAATTTCTGATGACCTTTTT
TAGTCATTCACTGCAGATTTTAAATCCCCCTATTTCCGCTGTTTCGGTACATTTTCACCCGGG
ACCCCTATTTCGGGAAGATTATTATTATTCCCCCCTACTTTTGCAAATTTCCCCCCTGATTTC
GTAAAATTGACATGTTTTTTTACATACTTTGTGTTTTTTGTTTCCCCCTAGTTTGACCATGATGC
ATTTATACCTCACACAATGCACGTACTGGATGCCCCTGGTTATTCCTAACATTATCCATGCGT
TTCTTTTTTTAATTTATCCATCAATGAATTGGAATGTTTTGCCATTTATTACATTGTCTTTACTTTTC
TGCTCTTTTGCAGCAAAGCAAGGAATCAGAACTGGCCGCATTTCACTACGACTATTCAGCT
GAAGAGTCAATGAAACCAAAACAGAAAAAATACAAAAAGGACAGCTACTTTAGTGACGAAG
AAGAAGAAGAATTAGTTTAAATAAATATTTATCAAGTATGCCTGTTCTATTAAATCCTTTAATATA
AGACTGTTGTAATAACTAGTAATTTGTGGGCACTTGTATTGGTAGAAATCTGAAGTATGACATT
AGGAGAGGCGTCCAGTACGTGTAAAGCATGATGCACAAATACATTATGGTCAAAATAAGGG
GAAGCTAATAAAACGCAAATTAAGCCAAAAAACGTAAAAAGAAAACATGTGGAAAAATTTGT
GAAAATATGGGGATATGTATGAATTTTGTGGAAATATGGGGAAATTAATTTTCTGGAAATAGGG
AGGAATATGCAACATGCATCATTGGCTTAGGGGGGATGGACGCCCCTGGACATTAGTGAC
CCATTACTTAGACTTGTGGGCATCACCCTGGTTCTTATGATGTCCACCCAGATTCTTAGGAT
GTCCACCCAGATTCTTATGATGTCCACTTGTATTCTAGTGATGTCCACCCAAGAGGTGGAG
GACGAGTAGCAGATGGAGATGAGAAGGAAGAGTATCAGGTGGTGAAAGAAGAAGTGGAG
GAAGAGTATCAGGCGGTGAAGGAACAAGAAGTAGAGGAAGAGTATCAGGTGAAAAAGAA

GAAATAGATTAAGAGGGCCATAAGAAGCTAAAGTGCACCCTATACAAGATGCTAAGCTAAA
GCAGTGCTATGTACAAATATTCACGCACATTCGAATAATCTCATTTTATAGTCTTTTCACCGTC
TTAGGGCTGACTAACATATAGTAATCAATATTAATCATACTGTAGCAGCCTGTTAGGTTGTGTC
TGTATAACTTGACTTCTTTTAAGGCCCAACTTGTTCACTTTTACCATTTGAATACGCTACATTG
AACGCCAAGTCAAGCACGGTTCCTCTGAATTTCTTCATGTTTGTCTGTCCGATACCTGGGG
ACAGGGCCTTTTTTGTAAAATAACATTTCCCGGTACGTCAAATTTTTGATGACCTTTTCAGAC
ATTTACCCACAAAAAGGTTTCTCCACCCGAAATTTTCCCATTCTCCACCCGAAATTTTCCGA
TGATCTTTTTTAAGTCATTTACCGCAAAAATTACTCCATCCAAGCCATTTTAAAGAGAAATTTC
CACCCGAAATTTTCCAATGACTTTTTAGTCATTACTTACCAAAGAAAATTTTTTCCACCCAAC
TTTTTTGCAAAATGTTATGGTCTCTTGGATTTTTATACATTTTTACTGTTTCTCTCGAAAAGGTCC
CAGAAGGTCCCGGTACACCATGATTTATTATATGATACTGCAATGCTGACATACAAAATATAC
ATTCATTTTCATTTCCCGGTATGCCGTACCGGCGCGTACCTTCACAAAAAAGCCCTGCCTG
GGGACCAATAAAAGCCACCCACCGACGAAATTCCTAATGACTGATGTTGTTACGCTGCGC
CTATCCCATTTGGTGAGGAATCGCTAATGTAGGGTCCTAGGGGAACACAATCACCTTAATG
CTCTTAAGAAACCTTTCGTGTTCTGACTTCCTTCCTGTTGACTTTCCATCTTTTGAAGAACGC
TTCTCTAGAACTTTGCGCTAAGTCCTGCTAACCCTATGTCCATGAGCGTACCCATAAAGAAT
ATGGGGGGAGTTGGATTTGGTCGCATTTTCCAACAGTTTCATTTTCAGTAACAAATTCATTTG
GGGGTTTAAACCAGTAACCCCACTAAAAATGCCCATGTCACATCCTTTGTGACCTGACCC
CTACAACATGCGCTCCAGGCGACTACATGACCTGGCTCAGGGTCTACTCTATCTAGAGCT
GATACCATTGCCAAGAGGACTTGTTATGCGCTTTGTTTAAATTGTTGCTATTATTTATATTTATGT
GTGAGTTTATTAGCAATCTAATTAAAACTATTCTTACTTAATTACTTGTGTATGATTATACTCATGA
ATAAATAAGTATAAGATCAGAATAACAGAAATAATAATAGTCTTTATTAACAATTTAAGCTTACAA
TAATAATGCAGTATGATCACAAGTATCGTATGAGCAGATTATATCAAGTTGTGACAGATGTTTA
ATTTAACTGTTAAGTAAGTAATGTTATGCAAAACGCCCATCCTTCATATTATCAATGCCACCC
AGCTAAAGAAAAACTAGTCAACCCTATGGAGGTAGTGAGAATTGATCTTTGATCATCTGTGCT
TCATGTCAACGTGTCACACAGTTGTCTACGCCCCAAACTTTTCAACAACAATTAACTTTCCA
GTGGTGTAATTTCATGTAAAGCACAATCTGCCCATAGTCAGATCATCAAATTAGTCAGAGACT
CAAAGGAATAAAATAATATATAATTGCATCATTCTGCAATTTCTTTCTTTCTCGCTGCTTCCTAC
GTATTGCATGCAGGGCTGTCCAGACCCATTTAAAAAACCTAGGTTTCTTTGGATTTTTTTAAA
AACCTAAAAACCCTGAAAATTTTTGTTTTCAGGTTTTTTATTTTTTTTTTATTTTCGTGTCAAACT
GTGTAAATTTCTACATGAGTTAATCTTTGTGCTTTCTTCTTGATCGTATTCTTGTGTCTAGTGTAC
CGCGTGCGACTGTGTGTACATTAAAACCTAAAAACCTTTAAATGCCTAAAAAGTCCTAGAAA
CCTAACAAATCTAGGTTTTTAGGTTTTTACCAGGTCTGGTTGCATAATAACATGCAGCGAACA
AGTTATTGCGCCATATAAACGAAGGAATCATTGCGAAGAGCAGAGATTCAGATGAAAAGCC
AGCTGGGTAGTAAACAGACTATTATGTTTTGGTGACTTTTATTTACTATTTTTGCATATAATGTCC
GACAAAAAGCCTTGTATGCGGGAGGAAGCCGACACTCTTTCTGAAGATGACGAGAGGCG
ATTTAAAGAGCTTTTCAATAAACTAGATGCAAATAAAGACGGATCGATTGATATAAAAGAGCTT
ACATCTGCCCTTCACGGAGTGGCTATAGGAGACGCTGCAGGACGGGCAAAGGTAAAGC
ATAAATCAATCATTCATGCGTGTCGTTGCTGTGAATGGCTGGACAAAATGTTTGGCGAAAGG
GAAGATTTATATAAATTTATGCACCGAATTCTGCATGTCATATGAAATTGTGAATGCGATCCTT
GGGGAAAACGATTAGATTAGAACCAAACACAGCGTCTCCAATAATTATAGACAAAGAAGGT
TATATTTAGGCCTACATATAATCTCCTGAACACCAGCAATATAGTATTCTTTTTTCTGCAGTAAA
AAGGATTTTTCAGTATGCATGGTCTCGTTTATTCAATTATGTCAAAGGTTACCCACTCAAATGA
CCATGTCTAGTGTAAGTTAGAGAATGTAAATGTAATGTTTAAATGTTTTGAAAACTATGTGGATT
ATGGGCACAGGGTATTCCTTGTTTTGACATCGTATCAATTCCGACTAGGTCCAATGTGATTC
AAGTTGTGGCTATGACAGCTTGAATTACCTATTTGGTAGGGCCTGGCTGGAAACCTCTTTAG
ACATTCGCCCCCGCTGGATATTGAGTTTTAAGGAACTTTTTGCCTTGGAAAATAATACCCAT

GCAAATATCTGTGTCTATCTGTCTTTGATGAGACCAGGCTATTACCTTGTTGGACTAACAATC
ATTATCACATGCCTTGAACATGGTAATTAACCCTTATAAATTGGCCCTCCTTCTGACAGCCTC
TAGAGACAAGGCATTTCTTGTCAAATCAATTCAGGCCTATTCAAGACACTGTACACTGAAGA
CTGCAGTCTGTTGTCTATACGAGGTTGACGGATGCCTATAGAAATGTATAAATGTTGGCACC
CAATAATCCATTCGGCATGCCATAACTAATTTGTTACATGTAAACAAATAAATCTCGGTTCGTA
CCCGGATTCGGCAAATAGTACATCCAAATACTCAGATTTACTACCCGATTGAAAATGTCTAC
CCGGCCAGACCCTACTATTTGGTGAATTAAATGAATATTGTATTATATAAAAACTCTACAAAAA
CATTAATGTACTAATAATGGGCATATATTGCGAGCAGTGTTTTTGTTAAGCGTCCAGAAAGAG
GGTTGCTATTCAACCGATAAGACTAGCCGTTTGGACGTTAATGAAAGCACTGATTACGAAC
GTTGTAAAGTTGGTTTTTTTGTTTAATGAATTGCAATATGAAACGTAACAGTGCTTCATTCATTA
GAAGCCCAGGAGTCCATGCTGCTTGTCTGGTGACCGACAACTAGTCTTGTTAGTTGCCACT
CTGTGTGCTGTGTTAATGTCTATAAATTGTTTTCCAGGAAATGCTGAAAACTGCAGATACAGA
CAAGGATGACGTGATAAAGTTTAGCGAATTTGTGCGGTATATGCAGCAGCATGAACACAAG
CTTCGCGTGGCATTCTCAAGCCTAGACCGGGATCAGAATGGTGAGATTTTGAACAGTGCT
CATCAGATAAACTGATGTTCTCGCTTAATCTAACTTACAAGTACTTTATTACACACGAACGCA
GAACAAGAAAAAGCATAAATTTAATCAAGCACACCTGGGCATATTTTAAGCAGAGTTTACAA
CTGCACAGAGAGCAAAAGACATTTTCTTGAAAAAATTTCTCATCTCATCTGTTAAAAATTAAAT
TTCTGACTACCTTATTTTTAGTAATTCGCCTGAATTTCTGAATTTTCGCGCCCGTGCCCAAAC
GCACTTTTGTTACAAATGTACAACTGCACACCAAAATTTCTTATGACCTTTTCGTCATTCCCT
CTAAGTTTCTCGTTTTTCACCCCTGTTTTCGAGCCTCACTCTTACAAAGTTACAACTACAACT
GCACAATACACCTTTTACAACTGCAAATTAGATTTTACAACTCTAGAAATTGTGATCAGTTACA
CATTAAAATATGCCCTGAAGCACGTAAAATGTAAAAATGTTCGTGTCTTTTAAGCGTCTATGTT
TGCCATTCGTGACGAAGAGTTCAGCATCATACAGAAAGTTTTTTTTGAAGCAACACCATAAG
CTATCGTGGAGAAAGTTAGATGTCAAGTGAGCTAGTCACCCCATAGAGTGTTTCTTAAGAAT
GTGATCCAAATCCAATCTGCCAAGGTTGCATATGAAGCCTTATTTTTTTATTGTTATCAGAGAC
GTAAATCTGGTTTGAAAACTTGGGAGTCGTGGGCCCTAAAAGTTCTACAGACAGAGACATG
TAGCACATGATTGAGGGTATCATCCCCAGAATTTTGATTTATTGTACACAAATCTTTCTATTTCT
GAAAAGTCACCACTTTGAAAAGTGTTCTCATATCATATCCTGTACATTATTGGATATAATAAAAT
TTCATGGAGACCCACGACCCCCATACCAAAAATTAGGGGGTTGTGACCCCCAACGCTCC
CAAAATTGATGCCTGTGATTGACTCTTATAGAGAAAAAATTCCGCAGAGGCTCTGCCGACC
TAAGAGCACCAACAACAATAGAAAAGAATGGGTTGCTGAAGATGGGCAAAGAAAGATACA
TGTAGTTCATTAACGAGGTTGCTAGGCAACGCATTCCGGGAAAATGGACGAAGGATAGCA
ATGCCTTTATAGAGCACTTTGTAAGTTCAAGCCATAGCGACAACCAGGATATCTATGATGTG
GTTTTTGATAGAATTTGTTTAAGAAAAGGAAAGGAACATTAGGGCAGACATGCTTTCGTGCA
AGAGTTGTAGACCTACGAAATGAGTTGTATGATAGTACTTTTTAAGTGGATAATGTCACAGCTT
TTAAGAGGATGCTAGGAAAATTGGGATATTAAGCATTCCATGGCAATGTGTAGTTGGTGTAAC
ACAGACTACAAGTCTTCTGAAACACTTAAAACCCTTGGTATGTTATGTTAGAAGGTGATTTCTA
GTTTTATTCTATTTTACAATGTTTTGTGTAATTTTATTTTAATGCAGGATTTCTGTGACTCTGATGG
TTGCTATTGCAATCTACATGTAATGTAATTTACTTGCAATTATCTTGCCCTAGTCCTTCCCATG
CAAAGTTGCACAATGGGCAATGCGTTTTCCTCCAACCATCTCTGTCCTACTTACAATTGATC
ACATCAAATCTAATGTTGTTGTTGTCTCTCAGTAACCAAATCTAATGTAATCATACCAGTACAT
CTAATCAAAGTAATTTACCAAGAATTGTATTTACATCTATGTTACCGTTTTCATGATGCATCAATT
GATGTGAGACCACTGGATATAGTTGTGTCTTATAGTTTCATGCATGCAATTAGACTAGCTAAGT
CAAACTGTAACGCTTGGGCTGACTTTACGTCGAATGTGATGATTAGTGCTCATGTCCCATGT
CTGTAGAACGTAGCATTCTGACGGGTGGCTGTGGGCGTGGCCTAATCACTAATTGCGCCC
AATGGCAACGTACGTGGAGCTACAGTCAGTAAAACTTGACCCAATCGAAATCGTGTAATTC
AGGGAAAGCAGAAATCAGACTCGTGAATAGTTCTTGAATTGATTCGTGAATGGTTCTTGAAT

CAGATCATGAATGGTTTTTGAATTGATTGATGAATGGTTCTTGAATTGATTTGTGAATGGTTCTT
GAATCAGATCATGAATGGTTCTTGAATTGATTGATGAATGGTTCTTGAAGTGATTCATGAATGG
TTCTTGAATCAGATCATGAATGGTTTTTGAATTGATTGATGAATGGTTCTTGAATTGATTAATGAA
TGGTTCTTGAATTGATTGATGAATGGTTCTTGAATTGATTGATGAATGGTTCTTATAAGTACACA
TACAAGACTTATTTTACTCTTTTCAGGGAAAATCGAGCCATCTGAAGTGAAAGCAGCATTTGA
AAAATTGAACGTACACATCACTCTCGAAGAAGCGGATCATCTCACTAGGAAGTAGGAGCTC
ATATTAATCAGCCAGATACTTTTTGGGCTTTTTATTCGCTTGAATAAACTCACAAATCATTTCTC
CAGTGCTTTGTAAAATTTTAACCAAACATTGTTGCTGTATATGTGGATTTGCTTAATTCTAATTCT
AATTACAGTAGTTTTATATAGCGCCTCTTCAAGATTGCCTACTCAGAAGCGCTCCCAGCCC
AGTCAAATATAACGGTATTGAGGGCAGAGAGGAAGGAGATGGAGTGATGAACAGGTATTTG
GCGAAGAGTGACAGGAAGCTTATGCGTATATATGTATGGGTGTGTAATAAGCTTATTATCAGG
TTAAACATATCAGGATGTTTTGACCAAAAATCTATGCCATATTTAATGGAAATGTGAAGAAATG
TGTCATTTGGCAGGAAATCACCTATAAATTTTCAAACTGTTTCTACCCAGTGCATTAAGAGCG
GATTTTGGCCTTAATGCATCATGTGGGATTAAAGAGTTCGCCAGAGAGAGTCTTATCTGTATA
CATACTAATATTGGCAAAAATCAGAATTTACTGTATTACGAAAAAGTCAGTTTTCCGGGTTATT
TAGTAAGGAGTAAGCAGTCCCTTTTTTTGCCAAAGTCGCATGTTTTCTTGACAGTTGTCTGAA
ATGTGAAGCATTGTTTTTATAGAATGGATAAGAACAATGATCTTGAGATCGAGTGGCACGAGT
GGAGAGATTATTTCGGAATCTATCCGGCTGATTCCTTGGAGGATATGATCGACATCTGGAGG
AGTCGACTGGTATTTCATCCTCAAAACTGTCTGTTGTTAAGCTCAGCATTGTATTGTAATCACT
AATCAGACAGTTATGGGGCGTGGTGACTCGGTGGGAAGCTTGGTGCCTTGCGTCCAGAG
GGTCGCAGGTTCAAATCTCACTCTAGCTGCCACATAGGGACCTTGGGCAAGTCCTTCACT
CACAGTTGACTGTAGCGCTTCTGCGTTTTAACCCCGACTCAGTATCAATGCTGTAGTCGGG
AGCACCTCTGAGTTAGTAGTGGACTTGAAGAGGCGCTATAGAAATATCCGGAAAAAATAAAT
AAAAAATAAGTTACATGCAAGAAAACTACTCAGAAGTGCTCCTGATGTAGTGAACGAAGAAG
AAAATGATGAGCAGTCTTTTAAACTAGCCTGGTAGGCTGCCATGGACATGAAGTGTTGACG
CCTACATTTCCTTAGCCTCAGCATATGAGAATGTGAGTGCTCAGCCCTCTACCCTGCTGTC
GAATCTTCCCACCAACCGTCAACTGTCATTGTACATATTATAGATGAGTTGGCGAGGAATCA
GAGATAAAGCTGAAACGTTGAAGGAAATTTTTGCTTTGGTTGGGGTAGCGCAGAAGGGCT
GGGTACAATTCTCAGTACCCCAGATGGTTGATTGCTCTTTGGTTAAATTTACAAATTTAATTTAT
TAGTGGTAACGAATGTAATTGTACTTTTCTGCAGATGATTGATATAGGAGAGCAGCTGACCAT
TCCTCCAGAATTCCAGCCAAAAGGAATATCGTGGCGACACCTTGTGGCCGGTGGCGTTG
CAGGAGCTGTCTCCCGAACAGCCACTGCGCCCCTCGATCGCCTCAAGATCATGCTTCAG
GTCTACGGGTCAAAGACAGAAATGAGGATCTGGGATGCATTCAAGAATATGATTAAGGAAG
GTGGAGTGCGATCTCTGTGGCGAGGGAACGGGATGAGCGTGCTCAAAATAGCCCCAGA
GTCAGCGCTCAAGTTTGGAGCATATGAAGAGGTATGCATAATGAGACAGAATTTATGGCTTC
CTTTTAGCTCACTTAAATGATATTATGTCATGATAATTACCAATATCTTATCAGCATATTCATGAT
AATAGTGGTGGTGGTTGTGGTGGTGGTTTTGTTTGTGGTCATGTTGGTTATTGTTGCTATGGTG
ATTGGTGTCACGGTGATGATGACAGCAGTGGTACTGACCTTTGCTTAATACTAATCAGAATC
AATTTCCGTTTTATGGCTGATTAATAGATCAGTGTATCCCGGCTGACAAAACTACAAACAATT
GAATTAGAGAAATAGAAATTAAAGCTAAAATTAGGTAGTAAATTGTCTATGTAGCTGATAAGAC
AATTGTTCAGAATAATTATAGTCAATGTTACTAAGATGCCAGTGGAGACCACTGTTTGCATAAA
TGTGCATTGTAAAGTGATTGGGGTGTAAAGTTTAGATCACGATTCGCAAGGAGCTCGTACCT
TCGATAGCAAATTTCCACATTAGCTCATTTCTGAAGAATTTCTACTTGGCAGGTTTTCGAATTC
GTGTCCTCATTCATTCTATAGCGCCCCTTCAAGTCCTCTACTACTCAGAGGCGCTCCCGAC
TACAGCACGGATACTGTATCAGAGTTTCACGCCAAAGCACACAGGCAACTGTAGGTAAAG
GACTTGCCCAAGGTCCCTACATGGCGGCTAGAGCGGGAGTCGAACCCACGACCCTCC
GGTTGAAAGCTATCGACTCAACCAAGGCGCCACCATGTCCCTCAATTGTTTTCTGCTTTGA

TTTTCACGCATGCACATGTAACCGAGACGTTTGAAGCTGTGTCATACGGATGATATTATTGTT
GTCTGTTTGGTTGTCTGCTGTCACAGATAAAGAAGTTGCTCAAAGGTAAAGATGTGCTTAATA
TTACAATCGTCGAACGGTTTGCAGCCGGGTCCATGGCTGGGGCGTTCGCACAGACGGC
AATCTTCCCAATGGAGGTAGTAACATCATTGTGTTGGTGATCATTATCTTTTTTAATGGAGGCA
GCATAATCATTCTAACAATTGTCATTGTTATCTGTTCTAATTGAGATATAATAATCTCCTTCCGG
CTTGAATTAATACCTCAGAGGGGAGGGGTCAGCCACTCGGATCCTTCCTCTCCGCTTCTTT
CGATCAGCAGTATGCTCTGTCTTTAATCGGAGAGACTTCACGTCTTGCTGTAATCTGTTCTAA
TTGAGATAGCTACAGCATAACTGTCATTGGTACAACAAATACCGTCTGATTGATAATTTTATCA
TTGTCGATTAAATGGTAGGATTATATGGTCAAACTGGTAATTGTCACTGTTCTCTTCTTTGTGGT
AAGAATGGAGGTCACAACAAAGATATGCATCTTTCTTCTGCTAGTCCGTGTTGTCATTGTCGT
AATCATCATCTTTATTTTCTTTGTTGTTGTCGTCCACTTAATTAATCATTTTCAATTCTGACGGAA
AAAACCATAACCCTTCTTGCTTTCAAAGCCCAGTTGAAACTGCGCTTTTTGTATAATCTCAAG
CATTAATTCAACTCCATTCGCCATGTAAGGACCTTGGGCAAGTCCTTCACTCGCAGTTGCC
TGTAGCGCTTTGGCGTGTTAACTCCGACACAGTATCAGTGCTGTAGTCGGGATCGCATTTG
AATAGTAGTGGACTTGAAGAGTCACTATAGAAATAGCCTGAATGAATGAATGAATGAATTGGA
TATTGTGCTGACAATGGACAGTCCTGTAAAGCATCCTAGAAGTGGCTTGCTGCTAACGGTTA
CGATGCTATAGAAATCATAGTATTTGCTTACTTACTTACTTGTGGTTATCAGTAAGTTGACTGTT
GTTAACTTTATACCATTCTGTGTGTATGTAGGTTGTTAAGACGCGTCTGGCCGTGTCCAAAAC
GGGTCAATACAAAGGCCTCGTTGACTGCACTGTTAAGATCTTCAAGAGAGAGGGCGTTCG
TGCTTTCTTCAGGGGCTACCTTCCAAATCTCATCGGCATCATACCCTACGCTGGCATCGAC
CTCACCATCTACGAGGTTGGCTTTTTAGACAACGCTTACACCCCATTAATTTTGGGTACTAA
CTGTCCCTCAGAGGCTGCCAGATTCTTTGCAAGCACGCACATGGCATGCTCATAGTGTATA
ATGTAAATATGCACAGTGATCATATACTTTTAAATGCTTGGACAGACTCAGTTACACGAATTTT
GCCTGGACTCAGACAACCGCTCTGACTGAGTGGAGTAAGCAAAACTCCTATATCCCATGT
GGGACTTTGAGGATAAGTTCATTTCCTTTACTTTGCCATTGAGCGAGCAATGGATGAATTCC
AATGCCCTCACCCCTTTTGCCAGCAATGGACTGAACCAAATCCCTTCACCCCTTTAGCCG
GACTGAACCCAATCTATTCACCCCTTGAGTCGCCAATGGACTGAACCAAATCCCTTCACC
CCTTTAGCCGGCAATGGACTGAACCCAATCTCTTCACCTCTTGAGTCGCCAATGGACTGA
ATCCCTACACCCTTTGAGCTGCCAATGGACTGAACCAAATCCCTTCACCCCTTTAGCCGG
CAATGGACTGAACCCAATCTCTTCACCTCTTGAGTTGCCAATGGACTGAACCAAATCCCTT
CACCCTTTTAGCCAGCAATGGACTGAACCCAATCACTTCACCTCTTGAGTCGCCAATGGA
CTGAACCCCTACACCCCTTGAGCCGGCAATTGAACTGAACTTAATCCCTTCACCCCTTGA
GCTGGCAATATACGAATCCCAGTCCCTTCACCCCTTGAGTTGCCAATGGACTGAACCCAA
TCTATTCACTCCTTGAGCTGGCAATGGACAAATTTGAAGGTCCTGGACAGGTGCCAGTGCA
TTGCATGTCTTCCCTTTTACATCTCACATTAGCCTGCTTTCTGACAGCGGAATATGTGGGTAA
ACAATTTGCCCGGATTCATTATGCGCTGTTGAGCCCACTTGTATCCAAGCATGAGCATTGA
GCATGCTTATAGATCACTGTGTTGCTCCTGAATAAAGTGTTGCCCTGTGCCTGGCTTTAGGG
TAGCGCTCCAGATATTTATGATTTACTGGATTGTCATGCATGAATTGATTTACGTTTTGAAGACT
CTGAAGCTGATGTACCTTCGACGTCATCCGAACAAAAACAATCCAGGTGTTGCCGTTCTAT
TGGCGTGTGGGTCGATCAGCAGCACCTGCGGCCAACTGGCCAGCTATCCTCTGGCTCT
CGTCAGAACCAGGCTTCAGGCTCAAGGTTTGACACGGTTCATCAAGTATTATTCGTCTGCC
TTACTCCATCATCATTCTCAGCAGCAGCAGCAAGTTTTGTCATCATCGTCATCATTATCATCA
CCTTACGCTTCTGTATAAATTCCATAGATAAATGGATAATTATCATCGTCACCAAGCATATCCA
TCAGCAAGGCTTGTGATCATCATGACGTTGTAGTGCACCGTGAAGCATGTGTATCATGGAC
GTATTCAATGGGAAGTTTTTTGGGTTCAAACCTCACCCCGAAACGAATGCTTTGCTCTTATA
AAAGCCTAAAACTATAGGATGATACAACAACTCCAATGCATACCCCAACCGAAATGTTCAA
AAGAAATAAAAAAATTTCTGGCTACATCTATCTTTCATTCATTCGTTCAGGCTGTTTCTATAGC

ACCTCTTTAAGTCCACTACTACTCAGAGGCGCTCCTGACACAGCACGGCGCCACTACTGT
GCCGGAATTTCACACGGAAGCGCCACAGGCAACTGTGAGTGAAGGACTTGCCCAAGGC
TAGAGCGGGATTCGAACCCATGACCCTTCGGACGAAAGGCGTCGACTCTACCAATGCGC
CACACATGCCCCACAAATAATATAATATCATCAGCAGGCATTTTGATCACCATCAAACATTAT
CATCATCATGGTCCCAATTGGTCCCATGGTCATTATGAGCCCAATCATAACAACCTCCGTG
AGGTTGTTATGATTGGGCTCAATTTTTTGGCTACATGGATTCCTAACTTGGCATTGTATTAAGC
CTCAGGTGTTAAGATTTATATAGCTCGTTATGAGAACTGGATATTGTGCTGTTATTATCATGGTT
ATGAATAATTGTTTTCAGCGACCGACGACACACGAGAGGGATTTGTGACCTTAATGCGGAA
CATTATAAAGAGGGAGGGACTTCGAGGCTTGTACCGAGGCCTTGTCCCGAATTTCATAAAG
GTGCTACCCGCCGTCAGCATTAGCTACGTGGTCTATGAGCACACGAAGAAAGCCCTCGG
CCTGCTCAAGTAGCTGTATTGCCTGTTCGGACATGCATACCAACATTGTCCTTGGCCTGCA
CAAGTAACCATTTTGCCACTGAAATTCTACTGTGCATTATTTTTTATATATCTCGTCTTTGATTTG
TTTACGAACTTCATCTAGTCAAAGAATTTTTGTATTGTAAGCTACTGTCTACTTCGGAAACGGC
AACGTCGTTAGAATTCCATTACTGATGTCTTTAATAATTTGAAAGTTATCTGTCTTTCAGTAATTG
TAATGGAGTTTAAGAAAATTTAAGAATTAAACTGTGTTTGCTAACTGTGTTTGCTAGCTGCTTGT
GCATGTGTGTATTTTGTATGTGTTTATTGTGCTTTTTGTATTACCACTGAAAGATATGAGCACTAT
TCAGTTGTTGAGTAAATATTCTTAGTAGGGATGTACCGAGGTCAATTTTTTTACCGAGTACCG
GGTACTGAGTTCTAAGGTGGTTTATTGCCAAGTCCGAGTATTTAACAAATTAGCAAAATTAAG
TGGCTTTCAGCGGAAATACACGGCCTATTCAGACAAACGAGAAGCAGTAAATATAAGAAAA
TACTGAACACAGCCCCCCCCGTTCTGTACCTCATCGTGGTGTGGGGTGAACAGCCAAGT
AGGGGAATGCCTGGTGGGGCTATGCCATCGGGTTGAAGGCCATTGGCTGAAGAACCTCC
TAGGGCGCTACACGAGGTGGAAAGGCTCACCAGGAACCCTGAAAAGCTGGCTAATGCA
GACGGCAGGTGTTGGAAGGACACCTTAGGGCGAAAGAGGTCTTTTAAGACCCACCGGC
CTCCTTACCCTACCAGGAGAGGATTGGTCACTGCCTGGCAAGAGACCAAGAAACTCTTAC
ATGATGAATCTGGAGGGGCAATGTCAGGGAGGACCAGAAGGAGAAAAATATCCGAAAACT
ATCAGGACTGGTAAATCGACCAAAAAGACAAAAAAAGAGGTCTGGAGGAGTCTTGTGAGA
GCCACATCGTCGGCGCAGCCGACGGAAGAGAAGAAAGAAGAAGAAGACTGAACACACA
ACACAAGTTTGTAACTTTGTATGTTATTAATTCGGCACAAAATATTTTTATCTACGATTCTGTTCA
ATATTTTACATTGAAAAAATTTCGTTTACTCTGGCTAATTATTTTGCAGAGTCCGAGTAATACTG
AGTAGTGAGATACTCGGCAGATACTGAGTACCGAATGGATAACCGGTACATCCCTAATTCTT
AGTATTTGATTCGTTTGTATTTGGTATGCCTATATTTTCTATGGAGCCAAAGCAAGTGCAGGC
GAGTATTTATTGCTTGCAAATGTAGGTCTGAAGATTTGTGTTTCTTTCACAACTAGTGTGTGATT
TGATTTCATAGCCAATTGGTAAAATCCAAAGGATTAGGCATAATTATTACAAGGACTTGCTTAT
TCTTTCTATTATTTGCGACCTATGGAAGCACCGATCTCCAGGATTAAAACTTTGGAGAAATGA
GATTTAGGAACAGCTGGAATTTATTCAGATCTGACTTAGGAAAGCTGTTTGCGGAAAAAGAA
TGACGTCAGAGCACTAGATTGCCATTAGGCCTACATTGTGGTAGGAAATCTTTGTTTTTCTAT
TGTTTTGCCAGTTATACCTTAAGGTAGTGCCCAGCTATCTGTGATGACATATTTTATTACTTTAT
TCCAACGTTTATGTTATTCTTTGAGAATTTGAAAAGCTCGGAAGAAATAAATTGTCCAGGTTG
GCTAAACCATGTCAAGTTCTGGGAAGGGAGAAGTGGTACGGAAGGTGATGGCATTAAAAG
TTTTATATCACGATTGACATAGATGCTGGTAGATGCTATGTTTTCCTTATATCTCCAGATTATTAT
TATCACAGTAGTTTTATATAGTGCCCCTTTAAGGTATATTCTATTCAGGAGCCCTCCCAGCCC
TACCTCAGTCAAACTATATGATCTAAAGGCCAGAGAAATATAGAACAGGCGTCAAACATAG
CGCAACCGGGAGACCATTCCAGGACCTCGGACCAGCCACAGAGAAGGTGTGGTGCTGT
CGGATAACTGTCCGCATGCGAGGGACCAGTAACTCCGCTTGTGCAGAAGAGCGCAATGA
GACATATGGGGTGATGCTAGACCCTCTATGCAGCACCGAACCAGCACAGATACATGGTAG
ACAATGCGCTGTGAGTATGGAAGCCTGTGAAGGACATCCCGGACATGCCCAGAGAGCCG
GCCAAACCTAGGAATGTGGCCCACAAGGCCGGCTGCCATTCACAAGACCCTGTCTAGAT

ATTTAAGCCGACAAGCCGGAAGTGTTGGTGTGAGTACGTGCTAACACTAAATAATGCAGAG
AGAGCTGAAGCCCCCTCAATAAAACTTAGCCCAGTGTTATTTTTATTATTCAGCAATATTATG
GTCATTGGTATTGGCATCATACCATTTACGATTGCTATCATCTGTTTAATACTCATTGATGTTTAT
TATTTCAGTCTTGCCTTTTAACCCTGGTGTAAGACAACATACTTGACCTCTGCGTTATCTTAG
CAGCTATGGCAGTTGCTGTTAGTGACCATGCATGAGCCAGATGGTGGTCTGAGTACGTGCT
AACACTCAAGAGAAATACATGTACCAGTGATGACTGCTGCTTCTCTGATAACACTCAACTAA
TTCGGGTGTTAGGTTATTTATCGAAACCAGGAGTCACGAAGCCAACTGATGTATGATTTCGA
AGCCAACTGATGTATGATTTTCCTTGGCGTAGGTCAAATAGGTCATGCTTGGAAGAACTTCT
CATTTTTGATTGACTGATTTATTGACTGATTGATTGACTGATTGATTGTGTTGATGTGAATCTTAA
GTCATGCATGAGCCAGATGGTGGTGTGAGTACGTGCTAACACTCAAGAGAAATTCATTGTG
AGTTGGCAATTAATTGTGTGTACACTGCAGAGAACATTTTTGCTGTAACTTCAAAAAGAGAAC
ACCACCTCTGTCAATGTTGTTAGTTTTCAAACTCTTGGCCGAATTGTTGAAGGATTATGGCTT
GCTTTTAATTGTTAGATTTTATTCGTTTTTTATTCCTTCAGCCTGTAAATACCAAGTGTTGAATAA
TATGGAAGTATTGAATTCCATTGAAGTGCAAGGCTGTTGCCATGTTGAAAAGTGTGGCTCCA
AAAGCTGTCTTGTCCAGGTTGTTGGTTTGATGATGGTTATGATTCTTAATCTTTAAGCACTGAG
TAACATATTCGCACCGAACAATTTGGTATCGGGAGATGCACTTAGGTGAATCTAGACTTAATA
ACTAAATGCTGATGGGTAATGTGCTCAACTGGTGATGCAGAATGAGGATTTGAATTTCTATTG
AGGCGGTAACTGTGTCTGGACCTTGAGCAAGTCCTTCACTCTCAGTTGCTCTGCACAATCG
ATGCCCCCCCCCCCCCCGAAGTTTTGGTGGGGAAGCAAAAAACGTAAATTTAGGAAAAA
ACCTAAATTGAACGAAAATAGGGAGAAATTTATAAATGTTGCCAGAATGGGGGGAAATTTATA
AATTTTAAAGAAATAGGGGAAATATGCAATATGCATCATTGAGGGGATGGACGCCCCTGGG
GACACTATCAAAATACAGTCTTATTTTTTACTATTTTATGTACAAGGAGGCACGGGAGCTGCT
GATTTGGATTCCATCTGGGATAGAAGAGTGAAAGCTTCTCAAAGAATGACACAACGAACAG
TTGTCCATTAACTTAATAGAGCGGAGGAAAAGAAAGATAAGTTATGGAACCAATAGGAAGAA
AAGATCTCATAGAAATGGGATTGAAGAATAAGAGTAAGCAAATGGCAAATAAGATTTTGTTTT
CAGAAAAACTGAAGCCATTAAACTAAATGATGTTTGAATGAAAACTTAGAAAGAACTTCAGTT
AATTGATACTTTAATTATGAGCGTCATAGGGCGTCAATCCTGGGGATTGGGAGAGATTTTCTG
GTGGGGAGATGAGAAAACTTTCCAGAGTGCTGACTTTGGAGAAATAGACAGATTTTTGGAC
GGAGAAATAGAAATTTAGGTGTAAATGAGTAAAAAAAAGGTCATTCAGAAATTTGGTCGACG
GCCTTTCCAGCGACCCCTGCAGTTTTCAAACCAGATTCACCCTGCTGATGAGCATATTGTG
ATTGGGAAGCCAGTGCAGATCTTTGTGGTAATGTGTTAATTAATGTAGTTCTGCTTCTGTTGAC
TTTGATGTGGTTTCTTTAAAGGTCAGGACCTGACCTAACCTAAATATATCATTGTACATTTGCT
TTTGTTTGTTACATTTTTATATGTAAATACCACTTCTATGCATTCTTTCTTCCAAGAAGTTTTTTAG
AAAAGTAATTATAATATGCATGCTATTGTTAAGTGTATTCAGCATCTGTCAAATGTTTTTCCAATT
GTAAATGTTTTTGACTCTATCGCTTCATGTAGGCCTATGCTGTCTAAGATTTCTATAATACAATC
TTTGTAAGTTGATTCTTTTAATTAATTAATTTTTAATTGACATTCTTGTAATAATTAATTAACATTTTG
TCATGTTGTATATGGGCAATTACATGTAAGAGTTCTTGAATATCGCACAAAATATAATTAAACC
GCACAATAATTAGAGTCTTTAAAATAATGAGTTCTGTTTGTATATATGATATGTATTATAAAGGGA
TACTTGCTGGGGTGGTATATAAGGGTGTTACACTTTGTAAAGAAATACTCCTTATTTCTGAATC
GTTGAAGAGAAACTGAGAAATTTTAAGGCTAATTTTCACATTTTATTTTCCGTGCTTGTTGTCT
GTTCTTACATAGGATTGTTCTTAGACCAGCAGACTGTTTTCAGTTTACCATTACTTGATGTAGA
AACAATTTTTTAGACATTTATGAGTTTTCTATTCTGTGTCGATAGCGTAAACCAGTACTGGAAA
AAAGTGTACAGTTTTTAACAATTGCTTTACTTGTAAATGTTACAACAGTATTTTCAACTTTTTTATT
ACTGGTGCTTCAACATATGTTTGGCTTATGCTTATTAGAGTATACGAAAGCATGTGCATGTGG
TCGTATGATTCTTAATGGCTGACGTTTTCGTGGCGAGAACACTCAAAAAATTGGCTGCCTCT
CCAGAGCCAGATCAAGTGACTTTACCTCTTCTTTCACAATATATCTAGATAGCACTGAGTTTT
ATCTGTGAGTGTTCTGGTCACATTATTTCTCATCACATCCGTGTCTCCTGCTCTGAGACACTA

ATGTTGAGAGTGTGGATGTTTGGCTGATGTTCAGAAGTTTATATAAAGCCATGTACGATTATCT
CGGATGGTATGCTTGATATTTCGTGTGGTTTGTAGTCTAGGCATCGGCTTCTTTTGAACTTTG
CACATTAAAAGACAGCCTATGTTGAATTGGCTAGAAAAATAGTTGCGGGTATGACTTTTACAT
GTCATATTATCTTGTGTAATTACCAAGTCAGTAGAGCTATAAAATTTAGATGTTTTGTGTTCTCAA
CGGTAGCATTTTCTTACATTGCCATTAAGTGTAGACGTTAAGTTTGATTCTGGGTATCAAATAA
AATGAATGGTAGGTAATATTGGAGGTGCTGTGGTTGAATTCAAGAAATTACATGTATAGTTACA
ATGTGCCATGCAGAATTCGTGTATGTGCTTGTTTTTTTATAGGTTACAGAAGTTCAGAAATAAT
CTGTACTTCTGAATGTATTGTAATGTTTAATTTGCTTTAGGGGCCATCCATTTATGATGTCCACA
AAAATATCAGATTTTTTCAACCCCCCTCTCCCCTGTCCACATGCGTCCACATGAGCCGGA
CCCCCTTCCCCCTTGTGGACGTCCACTTGCGGTCGACATGCAATACACATCGTTCTCTTTA
AGTTAAGTCCTGATAATACATACTTACATACATACATAGTTACATATATAGAGTTCTGGCTTTCA
TGCATACGCACCTGGCAAGATACCAGATAGAAAGTGACGTTCTCTTGGCAGTTAAAAATGTT
ATTAGAAGTAATACCATTGCCTAGGATCAACAAGTGTTGGGTCACCTGTTAGGGTTCCTGAA
GTCAATGGCCAGGTACACAATATTTATTATATTGTATTTATCGATTTCTATGGCGCTTCTCACA
GCATGAGCCTTTCATAACCGCTCCCAACACAGCAATTGACACTGTGTCGGCGTTTAAATG
CCAAAGCGTGCTTGCTTATACATGCTTACATCCATACAGACATACATACATTCCTGAAGGGT
GCTTTGCAATGGCTAGGTACACAATGTATGTACATTGTATATGCTTGCTTCCATACATATATGT
ACATGACTCGGGGCCGAGGTGTGTGGTAGTTATTCGAATAGCTGTCATCATGCAAAGAGCA
CTTTTCTCCAATGTGAGGGAAAGGTGTTTTGGCTAAGATAGAATTGGAGGGATTTGAACAGA
CCAATGGCATGCAGCTTTCTCAGTTGAGAAATGTCAACTGCACTTTTACATTGGCATTACTTT
TGGGTATGAGGTAACTGGGCCAAGTATGGTACGCAAAACCCTCCACTCAGCAGAGGTTCA
CACCTGCGACCATTAGGTCCTACCTTAATCATAGGTGCATTAAATGTTGGGAATCCAGCTG
GGGCCAATGAGCATTAAGCTCTCCCCTAATTGGGTTTGCCAAATGGGCCCCATTATTTGC
CCTAAGACATTGTTATTAGTGTAGCCTAATGCATTGTAAGGGGCCATCCATCATCAACGACT
TAAACACTTTGGACCAGTGTTTAAGTCTGCTCACAAATAAAACGGGGCTATTACCCTACTGT
GCATCAAATTTGGGGCCCGTTAGGAAAAGAAGATCTTTGCCCTAACTTTGTCCTAACAGTTA
CCAAATTTGTGTTGAGCAATTCGGGCAACTTTTGGGCAAAGGCAAATCCGCCAAAGCCCA
GGTCTTGCCCTAAATGCCCATTCGGGCAATGAATGGGGCAAAATTCCAATGCTGACTGGG
TAGTCATTCGCACAAAAAAATTGACGACTAATAATTTATTATAAATAGCCGCGAAATGGGTCG
ACTTTTAAAAAAAATCCCGAAAAGTCCCATTACAGAAAAGTACACTTATTATAAGAGAAATTT
CTATTTGTCCAGGAATATTTTTCCATGACCTTTTTTATTCGCACAAACATTTTCATGACCTTCTT
TAGTCATTCGCACTGAGATATAGGACATTTTTAGCTTACTTTTTGCGAAATTTATGAAAAATACT
ATGATTCACGAAAATCCCGGGATATCCCAACGGATTTTCGGGGTTGCTCGATTCCCGGGA
TATCACGGAATATCCCGAATGGGATATCCCATTGGCCCTGATTAAAAACTAAAAAACTAAAA
AATTTAAAAACCTTCAGTTTTTACCAGCCCTGTCGAACACGCATCGAGTGTGTTCAACTTGA
AGCGAACACAGCTCTAATAAAATAAATCAAGAGCTTACTCGGAGCGTACGCTGAGCGAGC
GGTGGACGCACCCAGTATGGCAGCCCCCAGCTACACTGTTATGTTATGTTAGCGATGTCG
ATTTTCAGTGAATAATAATAATTGGTTTGCTGAACATGTGCATCAAGAGCGCCATTATAAGCC
AGCTAATCCTTCTCATGACCAGCCGTGAGATAGCTTTCTAAATTCTAACTATTGTGGCATTGC
GATAATATGGTCTTATGACTCTCGGCTTCGCGTCACTATGGAAGGCAACAGGAACGTATTTC
GTGTTTACTTTCCTACCGAGTGCCTTCATGAGAAAAGTTCTCGTGCCATCGGTTGCCTCGAT
CAAGTGAACCGATCCTGTTGCGTTACTGGCCTCATCCGCTCATCCAATCGTCCTTCACTGA
ATACTGCAGGCGATGTGCGTGTAGTTGCTGTTTGGGCGAACACAGATGAGCCCGAAGCA
GTCCTAAGCGAATCTGGCGGTCCCGAGGAAATACAAAGCAGTAATTGTTCGACGAATAATG
CATTGTTATATATCGAGAAAAAGAAAGACTGCTTATCAGTTCGAAGGCATCCTAGCACCAGT
TACCAAATCATAGATTGTTCCTTTGAGTTTGTTCTAATACTGTATGAGTCGGCGTCATTTCTTG
CCTCTACAACGCTGAATGAGATGATGATTAAAAACAATTCTGCAGTTGAATGTGATTCTTCGA

ATGCAGCAGTCATTTTAGGATCTCTTGCAAAACAGCAACAGTTTTTCAGCAGTGTTGGATAC
GAAAAACAAACCGAATTCTATGATCCTGCAGAGCCTCGAAGGAAAACAATTCTTCATTTTGT
TGCAGATGTTGTTCTCATGTTGCCAAACTATGTGATAGTTTGTATAATTTTGAAGATATCACTGT
GGCTATCAAATTTAAGGTTTGTGAATGTTCGGCTTGAAATACAATGAAAAGGAGGTGCTTGC
ATTTCCTTTAATACTGTGTAAAGCGAAAATGTCTGCCATGTTCCAGTTAGTTGCTTTGTAGTTG
CTTCGTAAGATCTATTTCCTCTGCTTCCTGGATTTAGATCCCAGTGTTGCTTGCCTAGTTGCA
TATTAGGTATGATACAAGGGGGTCATGTGGCTTGACCCATTTTGGCAAACTTGTGTCGAACT
ATGTTACCTCCAGAGTGTCATCTCTAATTGACCATCCTGTGTCAAACAGTATGGCTCCCAGT
GTTTCATGCCTAAGTGCCAATCTTGTGTTAAACTGTGACTCCAGTGTTTCCTACCTAATTCAC
TATCCTGTGTCAAGCCATGGGTCCCTCTCTGTCATAACTAATTGGCATTTGGACATGACACA
CAGAGCTCATATAGCTTGACATGGAAAGTCCTGTGTGAAGCCGTATGACTCTTCTGAAAAAA
GCCATTCTCTAGTATTGTCATGTTAGACATTTGGCAAAATATCAATCAATCAATAGATGAAATTA
TCACAGGCGCAACTGTGATTTCATTCATTTATTCATTCCGGTCATTTCTATAGCGCCCCTTCA
AGTCCTCTACTACTCAGATCCGCTCCCGACTACAGCACGGATACTGCATCGGAGTTTCAC
GCCGAAGCGCACAGGCAACGATTTAACCTGATCATGGTCATAATATGACTGATAGTTCATA
CTCAAAAATTATAATCCAAAAATTGGCATCGTAAATTAGGTGATAATTAGGTGATCCTTTGCCA
CATGGCTTTAACTCTCAGGATTATTGTTAGCAGTGCTTTTTTGAAAAAAATATTTGCCAGTACT
ATATTTGCCGGTACTAACACTAGCGAAATGACCTTTTTTAGTCATTTACACTGAAATTCCAAA
CATTTCATAACATTGTTTTTTATTTGCCGGTACGCCATACCAGCAAGTTCCGTTACAAAAAAG
CCCTGATTGCTTGTAAGGTAATAATGCTGTTATGTTAGTAAATGAAATGTGTAATACATTCAGAT
ATGTATTAGCTTGGGGGAATTTTATTGACAATGCATTAAAATTTCTGTTTCAATTTTCCTAGATG
AGAAATTGTGTGTAGTGTTTGAAATCAGTTTAATGACTAAATGGTTCTGTTTCGTCTGTTAGTAG
TCATATTAGTGCTTCCTGAATGCTGAATTCAATTGTAAAACTGAAAATTTCTTCTCTTTCCTGG
CAGGATTTTTAGATTGTCGATAGTGAGGGCAGTCTTACAAATGCCTGCCACTCTGATGCAGA
TTAAACGTCGATTTCTGTCCAACTTGGCCGACGCTGCCGCTCTGTCCCGAAGCGGCGTT
CGACCTCTTCCACCAGCCAACGTCAGGTATAATGCTATGTCAGAATATCTCGGTATGTACTT
GCATATATCTGGTTACTTGCATATGCACATTAGACATTCACGTATGCAAAAACTATGCCTAAAT
GTTTTTAGTTTGCCATTTGAGTGCACAGGGTTAGGATTAAAATCATGATTCCATTTGTTGGTGC
TAATTTTTTATTGTGTTTACCGCCAACTATTAATGTTTTGTGTTAAGGTTCGAGAACAACAAGTA
TTGTCTTCTGTTGGACGTCGTTCTTGGTGTCTGTTTGATGAGCTGGTTGTGGTCTAGCAACGT
CATCGGAATTTATGCTGACCGCTTCATCACCTGGGCTGAGGTGAATTAGTGTGCCATGGAA
AATTTCTTATAAAATCATCATAAATGTCTAAACTTCAAAATCATGAAAGTCTATGCACTTATATAA
TTAGTATTTTATATATTCTACAGTATAAAAACTCTTATAAAATTAGCTATAAATGTGTATACAGTTTT
GACTGATAAACTTAGTATTTTGTATGATCTGAAACAGAAAAATTTATATCAAATTAATTATGTACA
TGTATACAATTTTGACATATAAAATTAGTATTTGACTGATCTGCAATAGAAAAAGTCATTCAATTA
ATTATGAATGTGTATACAGGTAAACTTCTAAAATTAATATTTTATATGATCTGCAGTATAAAAATTC
ATATCTAATTAACGTTGAATGTGTATACAGCTTTATATTGCATTCTGGATAATGTCAAGTGGCAT
TATGTTCATTAATTCATTCATTCTGGAGACTTATATGGCGCCTCTTCAAGAGACTACTACTCAG
AGGCGCTCCCAGCCCAGTCATGGCCAAAAAAGGACTTCAGAGAGATGTAAAATTTGGAA
GGGTGGGCCATCAGCAAGGAACGCAGCTCAAAGGGGAGATCATTCCATGCTGATGGAC
CCACAACCAAAAAGGCCCTTCGCTGCATAATAGCCAAACCCAAAGCTTTTGACTGAACAG
GCCATATTGGTTCATGTGTTGCACAAAAACTGTAGTGTAATGCTTTATAACTTTTTCAGCATTA
ACCCCTGCCTGATATTGTCACAAGTCGTAGGACTAGGCTTCCTTGTTCCCAACGACTTCCC
TTTAAGTCTCCCCCTCTTATATGTGTTATTTGAATGGTTGAAGAACTTCTCAGAGCTCCTGCC
ATCTAATAGATCTGAGAAGGTGTTTCTTAATATGTGACCCATCTGCAAAAAACCAGTCGTAAA
TCGGAAAATCAAGGTATTGAGCAAAAACTTGATGAAAGGCGTTTTTCTCAATTTTTTCATTGAT
ACAGATTATAATTTTAATATGTTTATTTTTTTTAAAAGATGCAAAAGTTTGAACGAAAAAAACCTG

TGTATGATTTTGTGCACGCTATGATTTTTTATGCATTATTGTCACAAATAGGGCTGTCAAAATTT
GCCAAAAGACCCATTCGTTTAATCGATCCATGCCGCAGCGATTAATCGTTTAATCGCTCAG
GTCCGAAAACTAATTTTTAATGAAACAAATTCACGAACCTGTTTTCATTGCAGTATTACATGGT
CGCGAAGACTCGCGATAGTGAGTGATTACTTCAACAATAAAGACCAAAGAAGACAGAGCA
CTACAATACCGTTGACCGCGGAATTCTTACTATGTTTTTCTTTATATTATTTGTTAATAGACCTA
ATAAAAGCTACCAACACTCGCACCTGCGTTTAGTTTCATTGTTACATTTCGGCGGTAAAAAT
AAATAGCCGCGAATGCTAAACGTTAATGGCGCATGCCTAGAAAGTAAACAAACAGTCAATG
CGCTAAAGATACTGAAATAAAAAAGCGCTGCCACCCAAAGATTTTCAGATGGGTGCTTGTG
CCAATTAAATAATAATTGCAACGAACCCAAAGTAGGCCTAGTCCTTGTTTCACTTCAGTTAAG
GTTTCGTTGAACGTTGGTCGTTATCTGTCAATCTTTCACAGAGGTCGCCATATTAAGGCACTA
ACGCTATTTAAGGCCAACGAAAAAGGTTTTTACCTCGGCACCATGCCAAAACATGAGTCA
CGCGACCCTTCTAAGTTCTAATCAGCCACAAACTTTGTAAATTGATTCCCGCAAACGGAAG
TAGCACGCCATCTTGCGTAGGGTAGGACGGCTATCAATCATTCGAAGAAAGACTATCCAA
CCAAGGCCTCTGTTGTTTCAATGGCCACCTCTCGTCATAGTCGTCAAACATCAATCGTTGG
CCGTATTGCCATCCTGGCCTACTGGGCGGAGTCGAGTGACAATAAGGCAGGCGCTCAGA
AAATTTAACTCCAGCCAGAAGTCTTAACAGCCACCTGCCGGCTGCCGTATCCCATATTCC
CATAATGCAATTGGCTCCATAGGCAAATAGCGAACACCAACATTAAGGTAATGACTCGTCC
CTTTGCCCTGGGGCGGATTTATTCAAGCTGGAAGGAGATATACAGGATAATAGGATTATAGG
AACTGGGCCTATTCACTAAAGTGTTCACTCTGAGAGGAGGCCAGCATACTGGCAGTCTGC
GACCCTGCTGGTGAAAGGGCCTGCTGACTCGCTCAGTCTCGCTGACACTGGGCCTTACC
TGTTAGGCTGCGACAATAATATGCGCTAGGCGACAATGTACAGCAGAACGATAAGTCGATG
ACCGAATTCTCATCGTCTCTTAGTCAAAGGCTCACTGGCTGCGGCCCACGGCCACGAGT
GCCTGGGGAATAACCGTCAACTGAATAACACTGATCACTCAGCGCATACAACGATACGAA
CACACACACACACACACAACGCCAAACAACAGCTGAACGCCTTGAATGAGTCACTCAGG
CAGCTGTGCATCGTATTCCTCAGAAATTAGACTGAACGGTCAAATCAATCAGAAATTACACA
CTGGTTAAATCAGAAAATAAACTACCGCGATTAAACGACTATGGCAGTTAATAAACGTTTAC
CAATGAACGGTCGTTTATACGGGGGAACGTTTAACCGATGCCAGCCCTAGTCACAAACTA
GTTCCACTCTTGCAATTAGTTGTTTATCCATAAATTCAGTTTGGATTGAGATAGAATTATTATTTT
GGTGTCATTGTAAAGAGAACATGTTTCTCTTTCAAGTGGTCTTTGATACTCCAAAAATCAAAA
ACTGCATGTGTGACTTAAGACTGGGTCTGTGCAGGTGGGTCCACTGGCGTAGACTTGTTAA
AAATATTGGGAGGCAAGCCAAAATATTTGGGGGAAAAGTTGGTAATAACTCATGTCTTATAGT
ATGTCATGTCTGTCATTATCTTCGTTTATTGGAGAATATAACATGGGACAACAGTACTCGTTGC
CGCCTATGAACAGCAGACGTTAAAAATGAGATTAAGTTAGTACGTTATATAGGTTACAAAAG
GAAATACTCAGGCTACAATCCTCAGATTTTTGGTTTTAAAGCATGAAAGTATGAGCATTTCTC
AATTATTGAGGGTGGCACGTGCTAGGGCCACCCCCAAAGTCTACACCTATGGATGGGTCA
CATATTAGTTGAAAACTGACAGTTAAACAGTGAAGCAATTAATTTGTCTAGTAGTGTATTCCTG
TTTGCATTAGGTGGTTGCAGTGGAGTTTGAGTCTCTGTTGAACTGGCTGATGGGAGCGCCT
GCTGGACTGAAACTCAACGAACCGTTTGCTTACTCCCTTGGAAACTTCTTTCTATACCATAT
CTTTCTGTGGAAAGGTGAGTGTATATGATATTTTAACAATGATTTGGCATTCCAACCAGTGTGA
CGTCAGTGGTATGCTTGCTGAATTTTATTCCGTCTTTCCACCCTGCTGTGCGATGGCGTGTT
ATCAAATCAAATCTTCTTTTATTGCAACCAAATTAATAGTAACATCAACTTATACAATAGGGGTA
CAACTTTGGCTGGCAAAGGAAACCCCGAAGGGATCAGAAAAATCCCGCTGAGTAAGGC
CACCCTAATAGTAATTCTATAAGTAGCAGTACAAGATCCACATCATGATAAAAAAATTACACA
GTAGAAAGGCAAACGCCAATGGTCAATTCTAACAGGTATAGCAACATAAAAAAATTTAAATC
TACATTTTTTATAATAATGCTGATGTGCTGCAATAGACTGTTAAACCCAGTAGGAGTTTTGCCT
AAGCTGTTGTTGGATTTGAGGCAAAAGAACATCTGGCATACAGAGGAATTAGATCTCACTG
GATGCAGCGGCTTTGGTTTTTTTCTGTAGCCTATCTGCGAACACTGCGACCGTTCTGGACT

GACATCTGCTACTACAGCTGTCTTTCTGGATGTCTGGGTCTGACAGTTCAACTGTCTCTTCTA
CAGGATCTTATCTCGATGATGACTGTTCACATCTACTGCTTTTATGTCTATGCTGCAAGGTAA
GAATTCTCATATAAACCAGTCTTTTATTTTCGTGCCTTGTCTTCCCTTTGTCAATGTGCAGTCC
ATTTGATGTTTTCATTGCTTTGCGTGTATGTATGAGATACTTAATCCAGTGCTTTTTAAATACCGT
CCAAAGTTATAGAGTTTACTGTTTAGCAACTGATCTATTTTGATCTTGTCCTTTGTGATATTATGT
TAAATGTGTTTTCCGTTTTGGTCGGTAGGCTTTACAGTCTCCAGGTTCTGGCACTGTCTTCC
CTCTGGCGACTCTTCTGTGGCAAGAAGTGGAACGTCCTGCGGAGGCGCGTCGACTCGG
CGTCGTACGACATCGACCAGTTGTTCGCAGGAACGCTCTTGTTCACAATTCTTCTCTTCAC
ACTTCCGACGACCGCACTTTTCTACGTCGTCTTTGCACTCGTCAGTTTCCTCTGGCTTTATT
TTGCAATTTTGAGTTTTAAGGATAATTTCCCAAGTTGTTGTATAGATCATTGTGAAATATTTCTAT
GATGTAACACTGGAATATCTTCATGATGTAACACTTTGAAATATCTATGTGATGTAACACTGTG
AAATATCTCCATGATGTAACACTGTGAAACATCTTCATGACATAAACTATAGAATATTTTTATGA
TGTAGCACTATAAAGTATTTCCATGATCTCTCCTAGATGTAAAACAGAATAAAACAAGGTCGTT
TTAGAGAAAGACGAGTATTCATTTAATTCATTTTCGTTTGGAATTTTTTTATTTATTGTGTGGGTT
CTTCTTCTCAGAGATACTATCTGAATCTAAAGCAGCAGCAGTTTACCATCCACCAGACATTT
GTTAATTTTCTTATTGTTTTGCTTTTGTTTTGTGCATGAGGTCTTTAAACTTAATCAAAGGCATGA
AACAACAAAGGACCTGCTCAGGGTCCCCTATACAGCAACCACCTCCAATTGGCTAGTTTG
CTCCCCAATTGGCTACTCTGCTCCCCAATTGGCTACTATGCCCCAGTTGGTACTATATCTCA
ATTGGCTATTACTATGTCCCAATCGTCTACTATGCTCCGATTAGTCTCTATTCCCTCAATTGGC
TGCCATACACCACAGTATTATTAATATTGTTATACTTTATGCTCTAAAGTATTATGTACCTGTCGT
CTGTGTTATACTAATCTGAGGAGAATGGTTTGCGTAACCACAATCAGGGTTCCGGTATTTCC
ACGGTTTCTACGCTTTCGCACGTCAGTTCCTCCCTATAGATTTCTTGGTCACATTGTAACAA
GGGGAGTGCTTGTGTAAACAACATACCTAAAAGACCTCTTTTCAAATTTCATTAATTTAATTTA
ATTGGCTTGGCAACCTGGCATTTTGAAGAAATCAGTTACTTGCTATTCACCAGTCAGTTTTCT
TACATTGCCATGTTACTTCTGAGTCCTCATAGAACATGTGTGTAGACTTCGTTTTTGAGCACAT
AGTAGGGTGATCAACCAGTGACCCTCTTTGGTGGACAATTCCGCTCATTGTGACATACTGTA
AGGAAAACACCAATTTCTAGTAATTTATGGATGCTTTGTGTTCTTGTTTTTGTACTTGGTTTTGT
GTTTAGTTGCGTCTGATGGTGTTGTTGCTGCAAGGCGTACTGATCACTCTGGTGTCACTGAT
GAACAGGGTACCAGTGTATTGGTTGTTTCTTCGAATGGTCCATCCGGAGGCCGTAACTGGT
TTGTAGAACGACGTCATCGCTATTTTTCTGGCATCTGTCATCATTTGCATAACACAGTTATGTT
TTCCATAAGCTTGTTGTTTTCCATCTTGCAGGTCATCATCTACATGCTATTGTCATATTAATTATT
TAATTGTTTTTGAATTTGCAACCAAGTTCAGACTGTGCTAAGTAATTTAATGTAATAGGGTGTCA
TATAAGCCTAGCCGCTCATTTATGTGCTGGCAGATTGGAACGTATGGTAGGGAGATTGCGA
CGGTGTGGGTACCTACCAGAGGGCGCCCCCTCATTTGCCGAAATGGTCTCGAAGGCTGA
CAAGAGCCTTTTCCGGTCAATTACATCTTACCCGGGTTGTGTTCTCAGCCGCTTCCTTCCA
AGGGTCAAGTCCACTGGGTACAACCTGCACCCACGCGCCCATGTATATGAGCTTCCCAA
GAAGGACAATCGTAACTTCATGTCCCACGTTTTATACTCTGACATTTATTGATTGTATTTTGATT
CATTTAATTTAATTGCCCTGTGTAATGGTCCCAAGTTGCGGCTGTGTCAGCTGGGGCCCTA
GATTTTCTTATTGCCAACCAATAAACGAAATTGATCTTGATCTTGAGTACATGCTTCTCAGACT
TGAATGTGTACAGAGTATATTACTTATGTGCATGTTAGCAGCTAGTTACGTTTATGTCTTTTGTG
TATTGTTTGTTTGTAGGTCATCTTCATATCTCCATAGTGCAGCAGAGCCCGCTGTGTGTCCAT
CTCAAGGTTCGATTTTCCTGATCTGTTGCGGTTGCTTCTATATCAGATGTCTTCTCTTGTCTGG
CATTTTGTATCCGTGGTGAGGTGCTCTAAACAAAACCAGGCGCATGTCGCAAGATGCAGTT
TCGAGAAACTGGTAAAAAATGACATGCAGGGCAAAAATTGTGTGTTGTGAGTCTGAATCAAA
TTCGTGTAGCATGATTTTTAATGTGCCATATTCTGTCAGATTATTAAAAATAACGATGCCTTTTTA
TGGCTAAAGTTTAAGATTTTCCAGTTTCTAGTTTGGTTGATGGCAAAGGCTCCTTCCCCTCTC
CTGGGCATCTCTGGTAACCCTACTTGTAACTCCATGTGACAGCCTCTGGTCTGACTTGATG

CGGTGTTCGTGAATAAGCTGGTGCTCTGGGCTTGGCTTCAGAGGCGTCCAATACATGTAAA
GCATGATGCATAAATGCATCATGGTCAAAATAGGGGGAGGCAAGAAACGTAAATTTTACGA
AAATAGGGGGAAGATTTATAAATTTTGGAGAAATAGGAGGGGAAAAAGTAATATGCATCATTG
GCTTAGAGGGAATGGACACCCCTGCTTGGCTTTCAAGGTGAGTTTCAGAAATGTGGAGTCT
ATGATTTTGTATGTTTTCAATTACAGCTGAGACCTCTGTCTGCCCTGGAGCTGGTTAAAATGT
CTGGCCAGGTGTCACCAGACGTAACTCCCAACAACACTTGGTTTTGTCTCGGAAAGAAAT
TGCTTCGTGGTCATCTGATCTATCCGTGGATAGAACGATCGTCATCTGTTTAAAGCCAGATG
ATTTTTGTCATCTGATTTATCCGTGGATAGAACGATCGTCATCTGTTTAAAGCCAGATGATTTTT
GTCATCTGATTTATCCGTGGATAGAACGATCGTCATCTGTTTAAAGCCAGATGATTTTTGTAAA
AGCTATCTTGTTGACTGAATATCAGAGATGTCATGATTTATAAATGTGGTCATTTCTTGTACTTTT
TGGATAAAAAGTACAACAGATCCGATATCTTCTTCTGATTGTTTCTTTGAAGATGATGTGACTA
TTTCACAAATATTATTGTTTAATTTAAAATTTATTTTTAAAGTACTTTAAGCATTTTGAATAAGAATT
ACATGCAATCACCACAGACCCATTTCATGTTTTAAAGAAGATTTTATTGATTGATTGATTGATT
GATTGATTGATTGATTGATTGATTGATTGATTGATTGATTGATGTGTCTTATAACTTGCTCCCGC
CTCCACAACAGCAAACATGAGTGAGCAGGACCACGTAGCTGTACTCTACTGGAGGAAGA
CGATTGTGATTACATTTCCAGTGTGCTCTATTGACACTGCATAGATTACCCTCTGTGTAACATC
TTGCAGTCTGTCTAATTTGCATTTGTGTCAGCTCATTAGATTTAATAAAGGGAGCATTCATTCC
GTTATTAATTAAGTAATTAATTCATTCAGACCATTTCTATAGTGCCTCTTGAAGTACATTTCTACT
CAGAAGCGCGAAATTATTGGATGGGCATGCGCCCAGGCTGCTGTTCCAAAGTCTATACCT
ATATGTCTAACATGCATTGATCACTTCTCAATGTCCCCATTCACCTACGTTTTTTCTCACCTTA
AGCCTGCTCGTTTTAAGCGTGCTGGAGTTGGAAGCGCTCCTGGGTAGTGTTTTCTCAAGC
GGCACTATATAAATGTTCAATATGAATGAATGAATTTAATAACCTTTGAGTACTTCACTGACATT
GAAACCTGACTATCTTAATTAATTAAGGATCTGGCAGTAATATTTTTTGAAGATTGCCCCAGAA
AGACATTTTCTTGGAATTATCGTCCTTTGAAAATCCCAGTGTTCTGGTGATTGCAGTTCTATTG
GGCTTACTTGTAATAGGACCCGCGCAAGCGCAGGGCTGAGAAGGGCTGTGGAGCAACA
TGGTCATCGTCATCCCCATACCTACCTCCCCGTCTCAACTCCAAACACCATCCACCATAG
CCGTCTAACTGTCTGCCTGCCTGACTCTCTGGATCTTGACCGCTGCCTATCGATTTTGTTTT
GGTTAAGCGCCTGTGAATAAGCTGGTTCCTGCGACTTCGCTTTTGTGGGCGCTCTAGAAAT
CAGGAGTTTACGATTACGATGGTTGCATATGTTGCCGATCAAGGTTCAGGATAGGAGCAGTT
GAGCACCTGGGAGGCTGGCGATGATACGTTCCAAGGGCCATCCATTTATGACGTCCACA
CGGAGGGGGGTCAGGTGGGAGGGGAGGGGTCAAGCCCCATGTGGACTTCCACACAGA
AAATTAAAAATAGAGTCCACTGATTTCATCCTGTCTTCTTCTCATGCAAAGAAGTTGGCGTTTT
TTTCACCAGAATTTTGTCTTTCGACAGAATTAAAAGTGGAAAATTTTCGGCGATATAAATTAGT
AATATAATAGTAAGTACTGTTTTAAATAAATTACAATCATATAAGCCTAGTCGAATTTCAGCTTTA
GGTACTATAGCCGTTTCAAGAGAGCGATGTGTATTTCATGTCGACCGCATGTGGACGTCCA
CAAGGGGACGCATGTGGAAATCGAATTTTTTGTGGATGTCATAAATGGATGGCCCCTATAGT
GTCAAAGTGTGTCTTAATGAAGTGATGGACGACGCTTGCAATCAGGGCTGTTAAAAACTGG
GGCTTTTTAAAAGAGATTTTTAAGTTTTGAAGGTTTTAAAAAAAGACTTCCACTTTTTATGGATT
TAAACGTATGCACAGTCGTTACTGTGATGAACGTTATAAATAATGGCAATTACAATAAACATGA
AACTTCATTTTGACTTCAAAAGTAAAGGATCTAAAATTGTCACTTTTCAGGTATTTTAGGTCTAA
AAATACTAAAACATAAAATTCGATTAGAACAGCCTGTTTACAACCTAGTCTACGACTTGTTCA
ACAGTAGGCTGCAATAGAATTAAATTGCGAGTACTATTTTTTTTTTATTTTCAAATAATTCTGATG
ACTCTAGTCACACATTTTATTGTTTGTCTACTTTTTAATTTAAAAGACAGAATAAATTAGATGTAG
TTTGCTTGGAAGGAAAATTAATGTGTTTTTCTATCTGTCACCCCCAGGGCCTTCCACTCAAA
ATTAAAATCTCGTCTCTTCAAACACTTATACCCTGACCCATCTGATCATTCCCCTCCCCATCT
CAACAACATCCACCCTAACAGCTACTCTGTCCCCTCCTGGTACTCTGGAAATCGGACCTG
AACTTCTCTTGACCCCCTTTGGAAACCCCCTTTGATTCGTCGCAGCGCTCGTGAATAAGTT

GGTGCCATGGTGCGCTTAGCTTATGAGTAGCGCTTTAGAATCTTGAGATTACGATTACGTTT
CATAATAATTCTCAATTAACTATAGCTGCGCTATAAAACGCTGCGCTGGGCCACTGGGGAA
GTTAAAAAGATTACTTCCTGTTTTATCGCTTGGGTTTTGAACTTCGTATGTAATTAATTTTGCTTT
TAATTGTTTTATTTGAACATGGTTGTTTACTAAAATGACTTCTGGATTATAGATACACCTTTTAAT
CTTCACGTCGTGTAGCGCATGGCATCAAATGGATGCGTCGTTAACTATACGTATTTTTAATTT
GTGGGACATGGTGGCGCATTGGTTGAGGCGATTACTCTCAACCGGAGGGTCGTGGGTTC
GACTCCCGCTCTAGCCGCCATGTAGGGACCTTGGGCAAGTCCTTTACCTGCAGTTGCCT
GTGGGCTTCGGCGTGAAACTCCGATACAGTATCCGTGCTGTAGTCGGGAGCTCCTCTGAG
TAGTAGAGGACTTGAAGGGGCGCTATAGAAATGGCTGGAATGAATGAATGAATCAAAAAGA
CTGTGAGCGCAATAGCCACCTCAGTTATTCATTCCAAGCAATTCCTTTTTATACAGCACCAA
CTCTTCTCAAATTAAACGCATGGAAACTATCTATCTATATATCAACTTATATAGCGCCCCTTCA
AGGTAACTACTCAGAGGCGCTCCCAGCCCAGGCTCGGCCAAAGATGAGAGTCTTGAGG
AGTTTATAAAAAGAAGTGAAGGAAACTATCCAGAAAGCTTTTGCCCGCGATGTCATGAAAG
CATTACCACATAACTTCTGTGCTCAAATCACTTCATTAGCTAAAAGCCCCTCAACGCTTCTAT
TACAAAATAGTATATCTTAATTTCACCCTCCTTCAAACCTCTCAACCTTTCTGCATTCGCCAAT
TGCTCACCATCCAAACGCCTTGGTCTACTCGTTCATTATCATCTCTATCTCGGCCTCCAGTC
TCATTCTCTCTGAAGTGCTGCAACCGCTCTTTAGTCTATGTTGCATCAGCTCTTTGAAACGG
ACTCCCAAAAGGCCTCCATCAGTTGCCTCATCCTCGGCATTTGGTATTGGCACCTGTGAAT
AAGCTGGTCTCCCTGATGTGGCATTTGTGGGCACTGAGAAGCTTTCAAAGATTTATATTACAT
GATATTACTAAGGTGCCGAAGCTTACATATCAAACCATCTGAATTGCAATGTATTTTGTGCTAT
ATGATGGGTTTATAAAGGGACCATATAGCCTAAATCGTATTCATTTTGCTGATTTTATCTTCTTG
TCTTCTGCTCTGCTTGCATGCAGTGGCATAACAAGGGGGCAGAGGAGGGTGGCCACCC
CAGGGCTTAGGTCAAAAGGGGGCACAAAATGAGGTTACAGGTAAGTATTTTTACACATCTT
GCAGTGTAAATTAATTGCCCAAAGGGGGGTGCAAAATGGAGGTTAAGTGGTAAGTTTTCAA
CTAGTGGTAAGGGGAGCAAGGGCGGAAAATGCATATATGGCGCTGAATTACTAGAAATTTT
TGGTTGACGACTAAAAAGAAAAGGTCATCAAGAATTTGTTATGGAAACATTTTTTTACATCGC
TCCCGGGCGCCGACACCCCTAGTTACGCCACTGCTCACATGCTATTCACTTTCAATAGCC
ATGCCAGTTTAAGTCTGCCCGTACATAATCATATTGTAATCGTAAACTTGAAATTTCTTCGGCA
CCCACAAAGGCGAAGTCGCTTGATCCAACTTATACACAGGCGCTTAATCAAAACAGATCG
ATCGGCAGAGGGCTAATTTGGTAGGTATACATGTATTTACATTTTAATCGCAATCAAGGAGCA
GTTTGGTGGACCGCAGATTATTAATAGCCTTTCCAGGCTCAAGAATGAGTGACGAGTCAAG
GCTTATTTATCTATTTATCTTTATGTAGACGAAGCCTGTTGAGACTTCAGAACCAAGTTTTGCC
GAACTATTGTATTGTATTGCATTGTATTGTATCTATACATTTATATAGCGCTTCTTGCAGTGCACA
CCAATCAGAAACGCTTCCAGTGCGAGAGACCCAGAGAGAAGAGAGCAGTCTCGAGAGA
ACGAAAAGAGGCACTTGGCTCACCAGTTAATAAAGTGGATTAAACATATATTGGATGTGAGT
TGAAATTGAAAGCGGAGTTATTTCGGCTTCTTTGTTTATATTCTGTTAGTTATGTTGCAGGTTAA
AGCCATGGACGACACAGACAACAGCACGGCTGTCATCAGGATCCGTGACAATTTCTATGA
TTTTATGTCTGGTTGTACTAAAGAGTCAACAGTGAAGCGACTGAAATTTGACACTAGTAGTCT
GTGTGAAAGTGATATTGGGAAAGGCAAGTACTGATAATGTTTAATCAGTCGCATTGACAACA
AATACATGTGATATTATAACAGGGCTGTAATTAGGTGAACAGAATATCAGTCCCCATTGGGAT
GGGTTGTAATTAGGTGAACAGTCCCCATTGGGTTGTGTTGTAATTAGGTGAACAGTCTCCATT
GGGTTGTGTTGTAATTAGGTGAACAGAATATCTGTCCCCATTAGGTTGTGTTGTAATTAGGTGA
ATAGAATATCTGTCCTTAGGTTGTGTTGGCTAAGGCTATTTAATTACTTAATTGTTTAGTAATGAG
ATGCGTTAAGTCATCCTGTTCAAATAAACGAAGATAATGATGATGATGTTGTGATTAGATGAAC
AGAATGTCGGTCCATTAGGTTGTGTTGTAATTAGGTGAACAAAATATCAGTCCCCATTAGGTT
GTGTTGTAATTAGGTGAACAAAATATCAGTCCCCATTAGGTTGTGTTGTAATTAGGTGAACAG
AATATCTGTCCCCATTAGGTTGTGTTGTAATTAGGTGAACAGAATATCGGTCCATTAGATTATG

TTGTAATTAGGTGAACAAAATATCAGTCCCCATTAGGTTGTGTTGTAATTAAGTGAACAAAATA
TCAGTCCATTAGGTTATGTTGTAATTAGGTGAACAAAATATCAGTCCCCATTAGGTTGTGTTGT
AATTAGGTGAAAAAAATATCAGTCCCCATTAGGTTGTGTTGTAATTAGGTGAAAAAAATATCG
GTCCCCATTAGGTTGTGTTGTAATTAGGTGAACAAAATATCAGTCCCCATTAGGTTGTGTTGT
AATTAGGTGAACAAAATGTCAGTCCCCATTAGGTTGTGTTGTAATTAGGTGAACAAAATATCA
GTCCCCATTAGGTTGTGTTGTAATTAGGTGAACAGAATATCTGTCCCCATTAGGTTGTGTTGT
AATTAGGTGAACAGAATATCGGTCCATTAGATTATGTTGTAATTAGGTGAACAAAATATCAGTC
CCCATTAGGTTGTGTTGTAATTAAGTGAACAAAATATCAGTCCATTAGGTTATGTTGTAATTAG
GTGAACAAAATATCAGTCCCCATTAGGTTGTGTTGTAATTAGGTGAAAAAAATATCAGTCCCC
ATTAGGTTGTGTTGTAATTAGGTGAAAAAAATATCAGTCCCCATTAGGTTGTGTTGTAATTAGG
TGAACAAAATATCAGTCCCCATTAGGTTGTGTTGTAATTAGGTGAACAAAATGTCAGTCCCC
ATTAGGTTGTGTTGTAATTAGGTGAACAGAATATCGGTCCATTAGGTTATGTTGTAACTAGGTG
AACAAAATATCAGTCCCCATTAGGTTGTGTTGTAATTAGGTGAACAGAATATCGGTCCATTAG
GTTATGTTGTAATTAGGTGAACAAAATATCAGTCCCCATTAGGTTGTGTTGTAATTAGGTGAAC
AAAATATCAGTCCCCTTTAGGTTGTGTTGTAATTAGGTGAACAGAATATCGGTCCATTAGGTTA
TGTTGTAATTAGGTGAACAAAATATCAGTCCCCATTAGGTTGTGTTGTAATTAGGTGAACAAAA
TATCAGTCCCCATTAGGTTGTGTTGTAATTAGGTGAACAAAATGTCAGTGCCCATTAGGTTGT
GTTGTAATTAGGTGAACAGAATATCGGTCCATTAGGTTATGTTGTAATTAGGTGAACAAAATGT
CAGTCCCCATTAGGTTGTGTTGTAATTAGGTGAACAAAATGTCAGTCCCCATTAGGTTGTGTT
GTAATTAGGTGAACAAAATGTCAGTCCCCATTAGGTTGTGTTGTAATTAGGTGAACAAAATGT
CAGTCCCCTTTGGGTTGTGTTGCTGCAGAAGTTTGGATCTAGATGTGTTTCTGTGATCTTTTC
ACTGTGCTTTAACTTTGAAGACAGAGCCGTAGCGCACGGCGAAGTGATTGCCGCAAAGTC
TGAGGTCACGAGGCTTCAAGCGCAACTGCAGTCACTCGAAACACATCAGCAGGAACTGA
AGCTCCAACATGAGAAAGAAATTGACAAACTGGTCCAGGAACACCAGGTCATTATATAGCT
AGAGATATTTGTCAATTTCTTTCTTCAAATAGTTTGCTTGTTTTCTTTAATTTGTATCAAACATATA
ATGGGCATTATTGACAGCCTTGTCGTTTTTTGGAGTTGTAGTTGTTTTTAAACTGGTGGAATTA
TTGGGTCGTGAGTTTAGAATGAAGTTGAAAGGCAAGTGAAACTGCTAGTCATGGTTTGTTGA
AGGTCATCCTTATTAAAAAATATCAGTTTCTGTTTGCTGAAAATTGGCAAGTAAAGGCAACAA
CGAGAACACGTTGCCGGTGAAATATGTCTATCCTGGTGCTTCTGTACTCCATGCAATGTTCA
ACCAAAGTCTAATCCCTGTCTGCTGTTGTAGATCACATTGCTCTTGACCTGCAGTGTGGCCT
TTCTGCACAAGATCTTCCTAATGACTTTATTACCATACCTTGTCTCTTTTCAACTAGACGAACA
ATTTTTAAAATGAAGAATAGCCATACAAAACCACAGTGAGCCTTTGCCACTGTTTGGGTAGT
CACATTGTGGATACAGCTGCGCTTGCTAGGGTCAAGGAATGAGCAAGATAAAAATGGAGC
AGCGCTCCCCAGGGACATTGATCTCTAATTTTAGAGAGTTGGAGAATGTGCATTTTAAGTCT
CCAACTGAGAAACATTGTTGGAATTTTCTGCCACTATTTTCCACTAAAATCTGCTAAATGGCG
GTAAAAACATTTTTCCGTGGGATTGTCATGCGCTGCGCATGACCGGCCCATGCAACTTCC
AAACTAACATGGCGGTCTTACATTGTCCGACTCGTCATTTTCGTATCAAATTTGCGTCTTTAA
GGTCGCCTGGGGCCAGGTTAAGGGTTGTGAGGGTAGTTAGGGTTAGTATTAGGGTTAGTGT
TGTCATTGGGGCAATATTTCCGCCGCAATAGCATATTTTTGGCGTTTTAAGTGGCAGAGACT
TCTGCCACTTTAGCACCACGCTAGGCTTAAAGTAACTGCTTTTGTTTTAGAAAGAGATTGCA
AAACAAGAGGACGTCAAATATCAACTGCTTGTCTTTTCAAAGAAGGAAGGCGAAAGGAAG
CGGGAGATTCTAGAATTAAAGCAGGAACTCGAAAAGGCAAAAGAATCACAAAAAATGCTG
ATGGCTGTTCAGAAGGAGAAAATTAGCCTTGAAGCAGCTTTAGAACAGGTTGTGCTTTAGA
CTACAATACGTTTGTTTCTGCTTTTTGTCTTTCATATTCTAAATAATTCTAAGTTTTCAAGGTGTT
CTATTTTAAGTTAGTGCTATTGTTTTGATTTTCAAGCATTGTTCTGTTTGTGTTTGTTCCTGACAC
TGCTACTCTGCTCTGTACTAACATTACTATGCTTCATTTACACTATTACTCTTTGTCATCATTACT
GTGTGTGTGTATATATCTTCGTTTTAATATGAAAATCATCAATAGACAAACCGCTACTCGAATA

CAAGAGCAATAAGCTTAATACATAAGTCCTATATACTAGTCTTCCTTTACATTGCTACGATGAC
AGCCACCCTCTTGGCTACAGGCGGCATGCGGTTTTCCTGGCGCAGACCTGACCGTGTTT
ATATCTAGGATGGATTCGATGAGGTAACATGGCTTGCGAACAAAACACCTTTCAAACGTGAA
AAATCCCTGGCTAATTCGGCAAAAACGTTCGGTACGCTAACCCAGTTCCAAAATCTCCAA
CATTTATGTAACCACTGCACAAATTCCCGCCTGAAGACGAAAATATCCATCGAAACGACAC
AGCTGTCGACAAGTCGACCAAAATGGCGGATGACATGCACGGAAATATACAATACAATACA
GTTGCTGCTGTTTAGATCATTTAGATATTTATGTAGGCTTTGCATATGGCTGATTCTGACAATTG
CACAAGTTCTTGTTGTTATAAAATGCTTTGATACCAGTAGAACATTGGTTGTGGCTCTTGATGT
TATATCGATTTCTTTATGACAGACGAAGGAAGAGATGGCGGATGCGGTCGGCAGAGTGGA
GATGGAAAAGAAAAGCCTAAGACAGTCGCTGGAAGTTGTGCAGAAGCAGTTGAAAGACCA
GGAGCAGCTACAGATGGTGATGAGGTAGAGCTGCAGATCAATGTCCTTCTTGATGTACTGA
TACAGCATCTGCTGATACAATTCAGAAGAAATCCTAATAGAAGGACTCTGAAGACAGCAGA
GAAAGTGACTCTGTCATATAATAAGTGGAGATTCTCATTTGGAGATTCTCTGACCTACCTGCA
AAATCTTTGCTGCCCCACCCTGGGCACCAGAGGCCGCAGTTCCCTCCGCTCTATGGAAC
GAGGGTCCTCTTTGGCCATTTCGCCCGTACATCCACATGGCAGACTCGTGCATTCTCGGT
GGTTGGCCCTTCCCTGTGGAATGGACTTCCGTTGGCGCAGCGATTGCTCCCCAGGGTTC
ATTCGGACACATTCTGCTCCAGTCTGAAAACTGTTCTTTTTAGCCGTGCAGGGATCGGGAG
CGCTTCTGAGTAGTAATCTTGATGAGGCGCTATATAAACCTCTGTAATGAATGAATGAACAGA
ATTAGAATAGAGGGATGGAGACAAGAGGGAAGCTGAGAATGATCTGAATAGACTGGATGTT
GAGGATATGTACATCAAGCTAAAGAAGAGAACTGGACAAATGAGAGGAATGGCGACGTTG
GACATACAAGACAAGTCTGCCTGATGAGGTAGGTAATCAAAGAAGAAAATAATAATAAAACG
TAGATGTAGTGAATCCAGTGTTTCTGTTAGAGTCCAAACATAGAAAGGTCTTTTAGGTGCGTC
CTGACCTATAATTTTTGTCATTATGTTCGTGTGGGGCTGATACATATCCGTTTGTATTAGCCCC
TATAGTAGCAGGTATACTTTTAAGACCTGAGCCATGGCTCCCAATTTAAACCCTTGGCGAGA
AATTATCACACTTCTTTACAATTTTGATACACTAATGGAATTTGTCATAAACACAAAACGGCTG
TGCCCTAGATTGGGCAGTTTCTGCTCTCTCTTCCCTGGTTGTTGAAGTCTGGTTTTGACTGT
GATCAGGCAGCCAACCCGTGGCTGGTATATGGTTAATAACGACAGAGGCGAGCCATTTTA
ATAACTCTCATGAGCAGTGGATGAGCTTGCTTAGATCTGTGTCAATCTTAGACCACGGTCTC
CTTGAAAGCAGTCCTGCTGCAGAGAAGAGAACTGGTGCTACACAATCACGCTATTTGTGTT
ACTGTCTTGGGATGCGTAATCTATTAAGAGCTTGTGTTTGTTTGTCTCTTGTGTAGAACTGGC
GGCGAAAGAAACTCTGTTTTGGAACGAGAATCCGAGAACCTCCGCTCGCAGCTGGCGTC
TGCTAACATCCGAATTAAGGTGAGAGTCGGGTTTTGTCGGTTCTTTGGGTTTTGTAACCGGG
GTTTTCGGTTGGGATTTGATTCCAGGATTTGCATGAGTTGTACATCTGTGGTTTATATCGGGG
CGAGTGTCCAGGGTATACATACAGGGTTTGGGGTTGGGATTTCCTTCTAAGATTTGCACGA
GTTATACATCTGTGGTTTTTATTGAGGAAAGTGTCTAGGATTTGTGTCTAGACTTTACCTTTGAG
ATTGGTGGGTGGGGCTTTGTGTCGTGGTGGGTGGGGCTGTTTGTTGTGGTGGGTGGGGCT
TTGTGTCGTGGTGGGTGGGGCTGTGTGTTGTGGTGGTTGGGGCTTTGTGTCGTGGTGGGTG
GGGCTGTGTGTTGTGGTGGGTGGGGCTTTGTGTTGTGGTGGGTGGGGCTGTGTGTTGTGGT
GGGTGGGGCTTTGTGTCGTGGTGGGTGGGGCTGTGTGTTGTGGTGGGTGGGGCTTTGTGT
CGTGGTGGGTGGGGCTGTGTGTTGTGGTGGGTGGGGCTTTGTGTCATGGTGGGTGGGGC
TGTGTGTTGTGGTGGGTGGGGCTTTGTGTTGTGGTGGGTGGGGCTGTGTGTTGTGGTGGGT
GGGGCTTTGTGTCGTGGTGGGTGGGGCTGTGTGTTGTGGTGGGTGGGGCTTTGTGTCATG
GTGGGTGGGACTGAGTAGTTACAGATTTATATTTTGCACTTATTATTTGTATTTTGCACTTACAT
ATTTATTTATATTTTGCACTTATACACTTATAGTATAGTTCCCCTTCAAGATAACTTTTCAGAGGT
GCTTTCAACCCAGGCTCAGCCCCAAAGAGAAATAGCTCAAACTAAGTAGATAATGGTTTGG
TTGCAGGAGTTGGAGGTGACCGTTTCCCGCTTCGAAGAAGATGGAAAGGTTGTACAGATG
ATGAAATCGCAGCTTAGTCAGCTGAAGCAGTTTGAAAAAGAAAATAAGACACTCAAGGAGG

AAAATCAGTATCTAAGGTAATAATATTATCTTATGGATGTAGCAAAAAGAATCGGCATGGATTG
CATGGTAAATGTACAGAGGCTTACCTTTACAGGAATGTGTGGAATGTAATTTTTCAGTGTATTT
TTCTTTGGTTGTTTGCTGTTGATGGGCTCAAAACTCATCACTTGTCGCTTTCTAGATATTGAGC
TAGAGGCCCAAACAGGGGTTTACGAGTTTGCAATTCTTCAATTCAGCTGGTGTCTGTGAATT
TGTCATAAGTGGAAAAGTTGTAACTCGATTTTAATGCGGTTTGTTTTATTTGATATAGAATGAAG
CCCTTTATTGCCAAAAATACTTTCAGAATAATGAAGAACTGCAGAGGTTTATAATCACACGAA
AAATCTTAAGCTTTTTATTCGATTTTTTCACTTTTTTGATTGTTCTAATCAAACGAGTATAATTATTT
CGGTTAGAAACAAGATATGTTGATCCTGTAAAAAGTATTTTGAAGCAGATGCAGTATAGATTAT
GATTCAATAAAAAAAAGCCAAAACAAGTTACATACATTTTGTGTCACTGAGTTATGTGGGGCT
TCAGACTAAAAGGCTTTACAACCAGAGCCATTTGCATTGAGTGAGTTTCTTCATTGCTTTCA
GATATGGTCTTTGACATCAACACTAGTTGCTGCTTTTGAATTGAATTTTTGCTCTCCTGTGCAC
TTAATCTGAGAACTGACCCTCTCTGATTTCACTTTCATTTTTATTTTAAACTTGGTCATAGAACT
GACACTTTTGAATTTAAATTTTATTTTAAACTTTTTATTTTAAACTTAGTCATAGAATTGACACTTTT
GAATTTCATTTTCATTTTTACTTTAAATCTATTCTTAGAACTGACTTTTGAATTGAATTCTGCTTTG
CCGTGAACTTAATTTTAGAACTGACTTTTTTGAATTTAATTTCCCTTTTTTATTTGTAACTCAATC
TTATAACTGAGGCTATATGTATTTGAATTAAATTTCTGCCGTAGTTGCTGAACTGAATACGTTC
CTGTATTGGCAGCCATGGACTTAAATCTGTGGTCTTTGTGTCTAATTTTAGTGCCATTCAAAA
GAACACGCACCTTCTTACTGAAACAAACATTGACCTTCGATCGAAACTGGAACAAGCCCA
GCAGCAGTGTGCCAAACTTTCAAAGTTTGAAGTTCTGTGTGAAGTATGTAAGAGTTGAGCAG
ATTTTATTAGTAGAAATGCTGTTGCAGCATTTAGTCTTCACAATGGCCTTTCATATTGTTAGAAT
TGAATAATGCACATGTATCATATGAATATCTGGATGCCAGACACAATACACATACGAACTTAC
AGACATGCATAGAAATTCTTACAAATTACAGAAACATTTAAACGTGCTTGTACAGTTTTGTACA
CATGTTCTCATTTGCTATTGATGAACTGTTGAGGCGCTCCCAGCCTAGGCCCGGGTGAAAA
TAAAAGTTTAAGAAGCTTGTAAAACGAGCTGGACAAATCCCGTGGAAGAGAGCGGATTTCA
GATGGGAGACTGTTCCAAGCAGAGAGACCCACAATCGAGAATGCCCTATGTTGTTTAATGG
GTGCGAGCATGAGGCACCACAAAATCACCTCTGGAGGCAGAGCGAAGAGATTGTCGGA
CAGTGCTGGACGAAGTCAGGATAAAGAGTTCCATTAAGTAAGTAGGCGCAATGCCAAGGA
CACAACGCCAAACAATGGTGGTAATATGTAACTCTTGAGGATGGGCATAGCCAGAAAATGT
TCGGTGGTTTTAAGATTTCATAGGGGTTTTGCATCGAATTTCATTAATTCATTCTGGCTATTTCT
ATAGCGCCCCTTCAAGTCCACTACTACTCAGAGGCGCTCCCGACTACAGCACGGATACT
GCATCGGAGTTTCACGCCGAAGCGCAGAGGCAACTGTAAGTAAAGGACTTGCTCAAAGT
CCCTACGTGGCGGCTAGAGCGAACCCACGACCCTCTGGTTGGAAGTCATCGACTCAAC
CAATGTGCCACCACGTCCCACACAATTTGGTTGTATTTTCCTATAGTTTTAGGCTTTTGCTAC
AGTAAGTCATTCATTACGGAAGGTTTGAATCTGTAATCCCTTAAAATTGTCATCATTAAGGGA
GATTGAGCTTGTCTCTACTCTGTAATTGCTATGTAGCAGTGACTGGAACCTATACTAGGCGGT
CCATATATGCCCTTCTAGTCTAGAGGGCATGCGACCTCAGATCTTGATGATTGCCTGCTAAG
TAGCAGTGCAACTAATGTAAAAAGGTAAGTTTGTTTTTCAATTTTTGTTTTGAAGGACCTGAAG
AAAAGACTATCACAGTGGGAGAGCTTCGACGAAAGCAGCGGCTTGAAGTCAGTTTCTAAC
ATGTGACTTATATTTAAATACAGAGTCTTAATTCTTTTAAGATTGTTATTGGAAATATAGAGAATTG
ATGTTCATGTTGCAGTGAAGATAAGGCTTGAAGTTGAAAATAAATGCTGCGGAGAGCCGCT
GAAAGTACTATATGTTCAGATACTTGGATAGTCGCTGAAAGTGCTATATGTTAACATTCTCCTG
AGAACTTTAAAGTTCGTAATGTTCTAAATTCCATTTCAGAATAATGCATGTATGTGATGATTTTTT
TAATCGGATGTCACTACTGTAGCAAGACATACATAATACAATGAAAATTTGTCTCTTTACAATAT
CTCAGATCTCCCTCTCAGATTCGTCGGCACACTGCTGAACTGCAGCGTGACCTTGCGCA
GTTGCTGGGGAAGAATGGTCAGCTACAGGCAAGGTAAGCAAGCGTTCTCAAAATCCACAA
ACCTCCAGTTGTGCTTCAAGCTTCTTTTTTAACATTCGGGCTCCAGACTGTCCCTCGTAGG
CATACTGGCTCAACCCAGAAGGTTTGCCTATGTTCAACCCACCGGTTGAAATTTTCTTTCTC

AGAGAGCTGTGACTCGAGAGCCTGTGTTTGTCCCTTATGCCCGCATGCTCTGGCATGCAT
CTGAAGGCTTTATTATATCATGATTTCAATGGTTCAAATCTGGAACACCACTAATGAGTTTTTC
CTTGCAGCGGTGCTACATAAATGTCCGCTTGCAATTACAGGTAGGGTCTTACAATTACATGT
AAGGTCTTACAATTACATGTAGGGTCTTACAATTACAGGCAGGGTCATGCAGCAAACCCTC
TTGTAACTTGTAGTCAGTTGACAATTTATTAATTTAATTGCTGTGTTGTACAGCTGTTTTCACTTT
ATTGTGGTGTGTTTAAGTAACTTTACTGGCTTTTATTATATTGTATGTAACCTTAAACATATACTG
ACGTTCTGAACGGTCCGTGGGCAAATTTTCATGCTGCTAGGTCATTTCACACCTTAATGTTAT
TACCTACAAAGCTCTGCTTTTTCTTACGTTTCTTTCTATTCAAACATACACATGCAAACACATA
CACCCATACAAACACACTTGCAAACACATACACCCATACAAACATACATGCAAACACATAC
ATCCATACAAACACACATGCAAACACATACACCCATACAAACACACTTGCAAACACATACA
CCCATACAAACATACATGCAAACACATACATCCATACAAACACACATGCAAACACATACAC
CCATACAAACACACATGCAAACACATACACCCATACAAACACACATGCAAACACATACAC
CCATGCCTACTGATTGTTGTGTTTGCTTTGAGTTTTCTAAACACTGTGCTGATGCTAATATCTT
CAGCAACCATGTTCTGGAGGACACATGTAGGGCTTCAATGGCTGAGCTGCAGAGCACGA
AGGCCGAGTTGGCCACAGCATCTGCCAAATTAAAGGAATCTGAAGGATTGGTTTCACGAC
TACGAAAGAAATTGATTTTTGTTAGTAAGGTAAGTTCACGCATCTGCCGCATGTTATTTGGAAT
TGATTATTGCATTCTGTTGGTTGGTGTACCAATAATAGCATTTTTGGCAAACCGGATTTCAGTT
GCTGAAAACCGGTTTGAAACCTGTTTTACAGGCCTTCATAGAAATTTAATTTCGAGCTCAGTT
AAACAAAGGGGTTGGGGTACCAACCAGACCCCTGTGTTTAACTGAGCTTGAAAATTAAATT
TCTATGAAGGCCTGTAAAACTGGTTTCGAACAGGTTTTCAGCAACTGAAATCCGGTTTCAAA
CCAGTTTTCAGGAACTGAAATCCGGTTTGCCAAAAACGCTATTAACATACCTAAATCCTACC
CTACGTTACCCAATAGACGTTTCGCAAAGAAGGTTGTTTGTTGTTGTTGTTAGGAGAAAGAA
CTTTTCAAGAACATCGTCGACGAGTACAATCGGCACGACGACGACATGACGTCTCGCGTT
GACACTCGATCCAGTGCACGGATCCAACACTTGGAGACATTGGTGGAAAGGCACCAGAG
GCACATCTCCACTCTGGAGTCTGATATAGAAGAAGCAGCACGGGAGAAGGCACAACTGC
GGATGAGAGTCACTCTGGTAGGTCAGAAATTTATGAGATAACTGGAAAAACGACAGAGGAA
GAAGTATGAAATGGATATGAAGTGTTAGCGACTTCAGTTTCATTGTCACGATATACAAAGGCC
GGTTACCAGTCCTCTTCTTAGCCGAGACGGATTTGTTAATTTTTTTAATTTGACTGAAAACAAT
AATATTATAATTTATGACATTTAATATGGTAATATTTGTCTTTGTTTATTAATTCAAACTGACAGGA
GTCGAATTCAACTGTGCAGCAGCTTTGGCTGAAGCTATCTCTGCTTCCTCTCTCCACTTCC
GCTACAGGAATGTGCCCTCAAAGGAATTTGGGGGTTGACATTTTGGGGCATTTTGCATTGA
ATTTGGTCGTATTTTTCTACAGTGTTGGGCTTTTATGACAGTAGAGCATTCATTTCAGAGAGGT
TTGAACCCATAAAACTGCCCCCTAAATATGCCCATGCCTCAAACAGTGTACAATATTAGTGC
TTTACTGATAAGGAAGTAACATCGGAGCCGGATTAAGTTTTTAGAGGCCCGTTTCAAAAATA
AATTAGTAGGCCTTTCAAAAGCCTATTTACCACCGAAAATTTGAAAATTTATGAAAATGACCT
TTTTTACTTGTCACTGACAAAAAGTTTTTAATCCAAAATCAATCTCAAGGTAGGCCCCTAAATT
TCTAATTTTATCGACCAAAATTTCCTATGCCTTTTTACCTTTTTTGTCATTTACTACAAAAAATCT
TATAGTTTTTCCTCTATTTCTATTTTACCTGTTACAAATGCCAAAATGACTCTTAAAGTATGTCC
CCAAATTGTTAATTTTCACTAGGCCCGTTTCAATTGAAACATTTGCAACATGGTTAATCCGGC
CCTGGGTAACATTGATATTATTACAACAATCGGATCCAGCCCGGTGATGATTTTCAGGATGA
GGATACTCACTGAAATTCGGCATTAAGCAGAGATTCAGACTGCAATAGGTGCTCATCTATTT
ATTCGTCTAAGCATTGAGTCTTGACAGACCTAGAATTACACTTGAAGAGTGATGTGTTTGGTG
AGTTCTGTTAACAATGACTGATTTTTCCCATCAGCTGGAGCAGAAGTTACTGAAGCAGACAG
ACAAGGAGCCTGTTGCCGTATCAACAACAAATGCTGCTGACCAGAATATCATTCTTGGACT
CAGGTAAGACACCCTCTATTCTTGTACATTGGATCCTTTAGGTAAAATACATTCTTGGACTTAA
GTACTTGGACTTGAACACAACTGCGGATGAGGGTCGCTCTGACTGTGGTCTTGGCTATATT
CTTGTAAATGGGTTCCTTTAGATGATATATAATTTGTTGTATCGTTGCTTATCTTTGCGTATCATT

GAACAGTTACTGAGGTGAAAATAACAAATCCTGGAGTCTGGTGCTGTCGTTTTGGTGCAAAT
TTTGCTACAAATTTATTTTACTGTTCTTTTTGTTGTTGACCTTGCTAGGCAGAAAGTGGAGGAC
CTGGAGAAGAAAGCAGAAGAGCTGAGTGAACAGAAAGAGGAAGCGCTTGCTCAGCTGG
AGAGAGTGGATCTTCAGGTGGGAAGACTCTGCTTAGATAACTTTAATAAAATCACTTTCCAG
CATTTCACTTATGTAGTTATGCCTAAAGTGGAGTAATAATTAATAAAAGTAAGTTGTTGAATGGG
TGACTAGTGCAGCAATTAAAGTCAGGGACATAACCATAAAAGATTTGGGGGGCTGGGTTTT
GGGAGGCTTTTCCATTGATTTTGGACGTAATTTTTATGCTTTTATAACAGGAACGGATTCATTT
GGGGAGAGGGTTGAACTCGGAAACCCCGTAAATATGCCCATGGTTAGAGTTAATAATAATT
ATTAGTTAATTAAGTGGAGGAAGATAGTTAACTTACTATTCACTATTCAGTCATTTAAACTATTAT
TAAATAAATGTCTAACGCGTGCAAGGTCTGTGAATCTGAGCCATGGAACTATTTGCTCTTCTC
TTCTTTCGACGAAGTTGCATGTTTTTTCTGTTATTCTCTCCAGTTGTGGATTGTTTGTCGAATCT
TGAAAGGCAATATGATCGGATAGCACGTGCATCACCGATGTTTGTTTTACTAATTAGGGTGG
TTACGACCGATCAAAGACGAGCGTCTATCACATGCGAATGAATCCGTATTCGATGACCCGT
GATCGTCAGACGCAGGAGATCGATCGATTGAGAGAGGAAAATGCCCGTCTGAGCGCCAA
ACTCAATCTGATGAAAGAATCGGGCGGACCGGTCGAAGGTCTCTCGGTCATCATCGACGA
GAACCTTTGCCCACCAGGAACTGCGAAAGAACTCGAAGGTTTACAATGGAGCTTGATAAA
TAAAAATATTAATACATTTTTCAGTAGTGTTTTATAATAATCAATTTGTAATACAAAGTATTTTATGA
AATGCGAAAAAAGGTTTTCTTTTTTAATGAGAACCTCTCTGCCCACCAGGAACTGCGAAAG
AACTCGAAGGTTTTCACTGGAGCTTGATAAATAAAAATATTAATACATTTTTAGTGGTGTTTTAT
AATAATCAATTTGTAATACAAATTTTTTATGAAATGTAGAAAAGGTTCTTTTTTTGATGAGAACCT
CTGCCCACCAGGAACTGCGAAAGAACTTAAAGGTTTACAACAGACCTTGATAAATAAAAAT
ATCAATATACATTTTAAGATATTATTTTGATAATGTTTAATTCTGTAATAGTTAGTTGTTAATATAAG
CTATTTTATGAAATGTGAAGATTATTTTTGTCGATAAGAATCTCACAGTCCACCAGGAATTGCA
AAAGAACTCTAACATAATAATACTGATCATAATAATACTGGTGTTGCGCGGTGGCCCAGTGG
TTAGCGTGCTGGACCGTCAACCGTGAGGTTCGGGGTTCAAATCCCGGCCAGGGCAGAA
ATTTAGTTCGAGATTTCTGCTCCACCTGCGCCCCCTAGCCAACTCGGCTATGATGAGTTAA
CTTACCGCACACTGTCAGTGGGAAGATAAGACGGTGAGGGCGAGGACGCCACTAGCCC
TCATATGGCGAGGCTAAGAAAATGAAGTCGCTAACACTTCATACCCATGGCTGATCTAGGG
CTACCTTAAGGGACTGATCTTCTTCTTCTTCATAATAATACTGATCATAATCAGTGACTGATCT
GCTTGAGTTTGAGCAATGTGAAACTGTGTCCATTTGAAACAGAGACAAAGAGCCAACTGGT
GAGCGAGCAGCTAAAGAACCAGCGTCTGATGGAGGCGTTTAAGAAGACGAGCCAAGAAA
TGCGCGAGATCAACTACAAGGTGCTTGGATATCGCGTTGACATACCGAACACAAACGAAT
ATCGTCTGATGAGCATGTATGCGGAGTCACCGAATGACTTCTTCCTCTTCAAGGTTAGCATT
AGAGACTCCGTGCAATTAATTATTATTAATTACTTATGATTAACTAACAACTGTTTAGCTCTGTG
ATGGGCCTCGGTCTGATTCATTAAGCATTTAAGCATAGGCGGAGACTTGGCCCTGGGTTTG
GGGGGCCAAAAAGAATCAAATGGCTAGCGTTTCTAGATTCCAGATGACCTTTTTTAGTCATG
TCATCATTCGAATGGTACTTTGCGGAAATGCAATCACTGTCGAAACACTGTGGACGTTTTTG
ATGATATAAAGTATTTCGGAAATATTGAGGGGATGTAAGCAAGGACCGTCCCCCCACCTCA
AATTTAGCAGGGGACCGTCCCCCTATGTCTCCACCCTGCATTTAAGTGCAGTTGATTGAATT
CAGTGGCCTCGCCCGCACAGAGCGTTTGTCCAGTATTTCTTCTAAATGTCTCTTCCGTGCA
GGTTCCACTGGATTCTCACTATCATAGTACAATCTTAGCAGTGACTTACATGTGACTTCTCAA
ATGATGACTCCTTCCATTCAGCCTTTACTCTGCACTGCTCAGCTCTCCCGTTCCCTATGCAT
GTGTGCATTATCAGTAGACACCTGATACGCAAGCACGTGGGCGTATTTAAGGGGTCTCCGT
GTTCAAACCCCCCTCTGAAATGAATTCGTTCCGGTTAAAAAGGCCTAAAAGTGCATAAAAAT
GCACCCAAAATCAATGGAGCTCTCAACAAAAATCCAAACCCCCCAAATTGGATGGCCCA
AGTGTATAGTGGATGGTTCTAAATTTAAGTCATCTTGTTATTCTACATCCTTGTTTCAAGTAGGT
AGTTAGTTACATGAGATCCATCCGCTAGATGGCATCATAGCGAGTCTCCTGTACATTATGAG

CTTGGTTTGATTTTCTCAGTGGTTTCTTGTAGCAATGTTCGAACGGTGAACTGCAACTGCTTG
CCAACAAATATTCCGAGTCATTCGAGAAGAACGTTGAAACCTATTTGTCGATGGGCAACAG
CTTGCCCGCATTTATGAGCAGCGTCACTCTCGAACTGCACAGTCGTTCTACCATGATGCA
GTCATAATGAAGGACTTTACGTTGAGTTCAGTTTTCTGGCGGTTGCGGACTTGGTTGCTGGG
TCCAGACTCGGTCGGAATCTTTTCAAACAGTTGCCTTCTCCTGCAGGGTTGATTACTCCCA
GCTGAAGTGTTTTTGGAACAGTGTGTCTTGTCTGCTGTTAATTGGTAAATTCTCTGGTAAGGA
ATGGGAGTGAAACAACCAAGTTGCTTTCTGGTGTTCTCTGATCTTTTTCGACTTTTTCGACTTT
TACACATGTAGTTGTAGTTTATGGTAGTTTGACTGTTATCGGGGGTGTGTCGTACGTGTGAAG
CGTAATGCAAAAGTGCACTATTGTCAAAATAGAGGGAAGCAAAAGAACCTAAATTAGGCAA
AAAATGTAAATTGAACAGGGGGAAATGTACAAATTTTGTGGAAATAGGGGAAAATTTGTAAAC
TTTGTGGAAATAGGGTGGAATATGCAATATGCATCATTGGCCTAGGGGGATGGACACTCCT
GACAGTTACTTCACTGTCTTAGAGCTGCAATTATGAGCTACTTTGGTTTTCCAGTCGCTTTTA
GTAGACTTGTTATTAAAGTGTCAAAAGTCATACTTTATGTACATACATTCATTATTTCATTCATTC
CGGCCATTTCTATAGCACCCCTTCAAGTCCTCTACTACACAGAGGCGCTCCCGATTACAG
CACGGATACTGTATCGGAGTTTCACGCCGAAGCGCACAGGCAACTGCAGGTAAAGGACT
TGTCCAAGGTCCCTACGTGGCGGCTAGAGCGGGAGTCGAACCCACGACCTTCCGGTTG
AAAGTCATCGTCTCAACCAAGGCGCCGCCACGTCCCACACCCCACATTCTAATCATTGTA
CCTTTGCGTGGCTTTGTTTAGTGTATGACGTGATGGTGACAGTTCAAAGGGGTATTTTGGTAG
TGGAACAGAAAATAAATTGCTTTTACTGGCATGATCTCTGCATGCTGCGTACTGTATATGGTT
TTAAAATTATTGGTCGTTGTTACCTCCGCGAATAAACTGATAGTGACAGTACCTTGTGAACATT
TGTTATGTCTGGTAAGATATGAACTTGTACAAAGAGACGCAGGCCGGATGTGTTGATCAGTG
CCCTTCGTACTATATGTTGTAGCCATTCGATTTCAATAAAAAATTAAACAAAACAATTGTGCTC
TGCTTACTGGAATCAGTTATTTGGCTCCTTTTAGTCGTTGTTAAACAGTAACTTCGGCTTCCT
GTCGTCCGTGTCATTTCAACACCCTGCATCCACTCAGCGGCGACCATCCTCCTAAGTCAA
CGATGCATATTTCCCCCATTTCCAAAAAATTATAAATTTCCCCCTATTTTTGCAAAATTAAAAA
GTGTCTCCCTATTTTCTTTCAATTTAGGTTTTTCTGCTTCCACCCTGCATGTATTTTGACCATGA
TGCATTTACACGAACTGGATGCCTCTGTATCCACTTGGAATAACCTCCCATCTGATTTGTGT
GTCCTCTTAGCCAGAAACTGTATACACCTTTTACAAGCGTCTGGAAACTGTTATTTCTTGCTG
TAGTTGAGCTGAAAGTGCCTTTGTGTAGGATGGGTTATCTAAGAAGTGTGCTATATAAGTTCT
CTGAATTAACTAATCTTGAGTTGTGCCCTATCCAGGGCAGCGTATTCCACACTGTATATAGG
GTCATTGAGGAAATTGACTTTTGGGTCCTTCCTTTATCCCACATCTGGTAATCGTAATCGTAA
ACTCCAGACCTACCAATCTGGCTTCAGAGCAAACCATTCTACCGAAACGGCTCTACTGTC
TCTTCTCTCTGACATTTTCTCTGCTACTGACAAATCGCAGCTGTCCTTATTGGCCCGGTTTGA
TGTATCTGCTGCTTTCGACATGGTCGATCACCAAATCCTTCTTGAGCGTCTTGAGACCTCGT
GTGGAATCTCATCTCTCCCTCTTCTTTGGATTAAATCTTACCTCTCTGACCATACTCAAATGAT
TGTCTCAGATGAATCCAGGATTTCCTGGGTTCCTATATTTTTGGGTGTCCCCCAGGGCTCTG
TGCTGGGTCCTCTTCTGTTCATCTTATATACTGCTGACATTCCCACTCTCTTTTCAAAATATTC
GGCTACTGGTCATCTCTTTGCTGACGACGTTCAGGCATATGTTCATGGTCCTCCTTCTAGTC
AACTTCTTCTCACCAGCAAAATTGAACTACTCTCAAATGTCAAGTCAAGTCAAATATCGTTTAT
TGCATGGAATATTAGAAAAGATTGGGGAGGCCTCAGCTGACACAGCCGCAACTGAGACCT
ATTACATGAAGGGGTATTATAATTTGGAAGAAGAAACGATTAAAGTTAACGATCTTAACTCTTG
GATGTCCTCAAATAGACTCTCTCTGAACTCCGCTAAAACCCAGCTCATCTGGTTAGGCACA
CCTCAACAACTCCTTAAATTAGATCCTGCTTTGATTTCTGACCGTTTTCCACACTTTACCTTTC
ATACTACTGTTCGTGACTTGGGTGTCACTCTGGACTCTGCCTTGACCTTTTCGCAGCACATT
TCCAACTTAACTCGCTCCTCTTACGTCCAACTGAGGCGTCTGAGAACCATTCGCAAAGCT
GTATCTGTTCCTATTTTCACCTCCATTGTTCATGCATTTGTATGCTCCAGGATTGACTATTGTAA
TTCTCTACTGATCGGTCTTCCTAAGACCCGGCTATCTCCCCTTCAGACTGTACTGAACGCA

GCTGGGTTTCCAAAGGGGGGGGGGTCACGAGAAGCTCAGGTCCGAATTCCAGAGTACC
AGGAGGGGACAGAGTAGCTGTTAGGGTGGGTGTTGTTGAGATGGGGAGGGGGAATGATC
AGAAGGGTCAGGGTAGGAGAGATTGAAGAGATGAGATTTTAATTTAGAGTGGAAGGTCCTG
GAAGTGACGGATAGAGGAGGCAGGTGAAGATGATGTCTTGTGATTGGCAATGACGGCGGT
GGAGGCGAAGAAATGGTGCGGAGTTCAGGTGATAGTCGAACCACAAGCGCCTCTGTTTG
CCTATGGCTGTTGAGGGGCGCTATATAAATACTTCAATACAATACAATAATTTCTAGAACACC
CACAAAAGCGAAGTCGCGGGAACCAGCTTATTCACAGGCGCTTAACCAGAAAAAAATCG
ACAGGCAGCGGTCAAAATCCAGAGAGGCAGGCAGACAGACGGCTATGGAGTTTGGAGTT
GAGACGAGGAGGGAGGTACGGGAAGAGGACGAACCAGGATAGGATATGCTAAATATGCT
AAAGAGCAGTGTGCCCATTTGGCATCAACCTCCCAATTGCTCAGCATTTGATTGATTCCTC
CGTGTCCTCTATACGCCCTTATACCTTCCTTATCTATACCGATAGGTCTCATGACAAGTTGAA
TAGCATGGTATGTAATTATGATATTATGTGATTGATGTTTTGTTAATATCATCACAGTGCATTTGG
AACAGCCTATTAGTACCAATAAAGTAAGTTATAATAAGCAAAACACATATATCAAAGAATTCAA
TTATGAATTGAATTGTGCTTTATTGAAATTTAGATTCCTATGAGCGTTGTCAATAATGTCGAATTA
TTTGTAAAATAGTAAAAAAGAAAAACTTTATAGCGCACGGTTTTAGTTATATGACGTCATCAAG
TGTGAATTCATAATATATTATCTTCAGTGTTGTTCATGTTGGCGGGATTTGTATATTATAATCATAT
GTCTCGGTTGAATTTTTTGTAAATAAACGAGGTAAACCAAATTTATCATTAACAACGCTTATAC
TTAAATCGGAACTTTCAACATGCACGTGATTTTCGTACCAGCCAGCCCGCACTTAGAAACC
TGTCGTTATTGAGTGCATTACCCCGTACTGGATTCCTGAGGTCGGCCTGGACAGACTCATA
GACCAAAGTAAACACGGGGTAAAGTTGACTAATCCGTGCCCGCCCTATAAACGGTATGGC
CACCCACGAATGCGGTCAAATAGAGCGACGCTCTTAGCTACCATTCAACTCCCGTCATGC
AATCCGGTCAGTAAGTAAGCGAGCAAATAGATATCATATTGTTAGGAAAAGCAAAAGGTAAT
GACATATTATTGTTAAATGAAACAACTAAGACAAAAACAAATAAACAACATCATCATTAATAGC
GCTTATAATAGACTAAGCTAATAGTAAGAACAAATGTCAGTACGAAATTTGAAGTGGGATTGA
TGAAAAATACCGTGTAAGTGGAAGCGGGATTGATGAAAATCACCTTGAAATTTGAAACGGG
ATTGATGAAAAAATACCGTGTAAGTGGAAGCGGGATTGATGAAAAATACCGTATAAGTGGAA
GCGGGATTGAGGAAAATATACTGTGTAAGTGGAAGCGAGATTGATGAAAGTCACCTTGAAA
TTTGAAGCGGGATTGATGAAAAATACTGTGTAAGTGGAAGCGGGATTGATGAAAATCACCTT
GAAATTTGAAGCGGGATTGATGAAAAAATAATGTGTAAGTGGAAGCGGGATTGATGAAAATC
ACCTTGACATTTGAAGCGGGATTAGTGAAAAAATACCGTGTAAGTGGAAGCGGGATTGATG
AAAATCACCTTGACATTTGAAGCAGGATTGATGAAAAAATACCGTGTAAGTGGAAGCGGGA
TTGATGAAAATCATCACATCAATTGAAGCGGAACTGATGAAGTTAAGGTATCCCACTATTAAC
GGCCGTAGCGGCTAGCCAATGCCTCTGCTATTAACAGCCTCAGCGGCCGTCCAGCAAA
CTATTAATGGCCGGAGCGGCAGGAGCACTGTTTAGTCACTCCCGTATTCCTGTGCCCAGT
GTACCGCGTGCGACTGTGCGTACATTAAAACTTAAAAAAACCTAAATCTATTTTTCAAAAAC
CTTAGGTTTTTACCAGCCCTGGAATAAACACACATTATTTATCGTCTTTATTTGCTTACTTATTG
ACCGTATTGCATGACGGGAGTTGACTGGTAGCTATGAGCGTCGCTCTATTTGACCGCATTC
GCGGGTGGCCATACTGTTTATAGGGCGGGCACGGATTAGTCAACTTTACCAAACACGGA
GACACATCGATGTGACTAAACAGTGCTCCGGCTGTACATAGCGGATGCAGGCCATTTTTG
GCCCGTCTAAATGGCCCGTCTGGCTGGGGTCTATACGGGCCATAAGTGGCCCGCCAAA
CAGCAAACAGGCGTCGCTCCACAGGGCCGCTACATTACCGCACATGGCCCGTATATGGT
CCGCCTAATTAATGCGGCACGGGCCGTTTTTGGCCCATCTGGTATTTTCAAATGAGAAGG
CCTGAGCGGAGGCATAGGCTAGCGGGACGGCCGCTAACAGCGGAGACATAGGCTTGC
TGTACCGCGTGCACACATGCTAGCTGGTTTATGATAGGCAGTGGCTAGCGTACGGGCACC
CGTACGCTAAAAGCAGTTGCCAGCGTACGGGCACCCATACATTAAATGAATATAATAACATT
GATAGTCTATGAGAGACGCTTTATTTTCATCAATTTCGACTTCTATTTAATTGGGCTTCTATTTAA
GCAGAATTCTTTTCATTAATCCAGCCTCTACTTAATATTTAGCAAATACAATCCCTCATGTATAA

CTTATGCGAGCATCAGTTGGGCACTAACGAGCTAATTAAATGCGTCACGTGGGCTATGCGT
CGTTTAGCATTAGGCCCGTCCACTGGACTTTACGGCCGCAGGACTTTGAAACATGCTGCC
CGTACGCTAGCAACTTCCTTTTAGCGTACGGGTGCCCGTACGCTAGTCACTGCTTTTATGA
TAGGCCTACCCATGTTATCAAGCGATATCGATGAAAATATTATATTGTCATTGCAAGCGGTATT
GATGAAGAAATTACCGTTTAAGTGTGGTTGAGGAAAAAGGCATCGTGTAAATGGAAGCGAG
ATTGCTGAAAAATTACCATGTAAGTTGATGCGGGATCGATAAAAAAATTACCGTCTAAATGGA
AGTGGAATTGATGAAAAATCATCGTTTAAGTTGAAGCGGGATTGATGAAAAATTACAGTGTAA
GTGGAAGCAAGATTGATGAAGTTAAGATCTCCCACTATTAACGGCCGGAGCACTGTTCAGT
CACACCCGATACTCATTGCTTCTGTTTACGGTAAGTTTACTGCGCACCCCATAACCCGATT
CCCTGAATCTATCAAACATCGAACCCTGAGACTACAGGCAGAAACTATATCCGAAGAATTTT
TGCACAGGTTTAGTCGATTTGAATAGTAATAATATGTAAACATGTAAACATGGCAGGCAACTA
GCTAAGAATGATGTGCCATCATATATCAATGGTGCAATATGTGTTCTTCATGGTAGTAAGGAA
CACACATGTGACACAGACCCAAAAGTCAATTTCCTCAACGACCCTATATACAGTGTGGAAT
ACGCTGCCCTGGATAGGGCACAACTCAAGATAAGTTATAATATATCAAAACAAACTAACTCA
AACCCGTCATAATGTTGTTTTTGGCTTTTTTCTTCAGTCATATTCTATTCCTACTCTGCATCTGC
TTCAAAATGCTTTTTACAGAATCATAATATATTGTTTCTAACTGAGGTTATACTGGTTTCATTAGA
ACAATCAAACAACTGAAAAAAATTGAATAAAAGTTTATGATTCTTCATATCATCATAATCCTCTG
CAAATTTATTCTTCTGAATTGATTCTGGGCAATAAAGGTATCAAATAAACCAAATGGCATTAAA
GTCGGGTTACAACTTTGCAACTTATAGCAAATTCACAAACACTGGTCAGCTGAATTGAAGAA
TTGCAAACTCGAAAACACCTCTCTCCGATTTGGGCCTCTAGCTTATATATCCAGGAAGCGA
CGAGTTTTAAGCCCTTGCGTCACATATGCAGCATTTTGAACACCAGTCTTTTAAACGATGTT
GCTGCTGTGCGATAGGAATAAACATAAATGGCTTATAAGAAGCTTACAGTATTTGTTGATAGA
AAATTATAGTTATTCAGTATTGTTTTGACTGTTTCAGTGTTAGAATGTCTTTGCGTAAGAGTGATA
CAAAAGAAATCGAAGATATTATTGGATCTATCAAGCAAAACTTTGAGTGTCCTATTTGGTATG
CTCGTCTTTCTTTTTCTGCATGCTGTGCTCGTTTAATTGTTTTGCATTGGCAGATTGCCAATAA
TTATGACTAATGGGGCTTGCTTTACAAACATGTTTGATTCTGTTGTTGGCATATTTAAATTTGAT
TTTTCATGCACGACATTAATATGTCTTCACATTCTCCAGAAAATGTACATGAATACGTATAACT
ATATACGCAGTGAGACACTTTTACATAGTGCATCGTAAGTACATCGCAATGCACTGTGTGCA
TCACTACCTAGTTGATCCCTGTAAATGCATTTTATGATGGTTTAAAGTAGTTTAAGAACAGAGT
CTACCGCTGAGGGATTCAAATGCTGTAGTTGTTTCTGTGCTATTAATGGGAGATACCATATTG
CATTTTACTCTGTAATGTAATATTGTAATGGATAGATATGCATAAGAGTTTGGGCTGTCTTGAAT
AGTTACTTAATAACTAGTGTTTGTTATTGTAATTACATACATTTGATTCTTTGTCTGCCTACCCCA
TAGAGCCAGAAGAGACTTGACGATGAAGGCATGTTTCCATGCACTGAACGTATCGTAATCT
CAAGATTTCTAAAGCGCTACTCAAAAGCTAAGCACACCAGGGCACCAGCTTATTCACGAG
TGCTGCGACGAATCAAAGATGGGTTCCCAAAGGGGGGTTGGTTATGCACAGCATGGAAAT
GAGCCCCAGAGACACACTGCCTACATGGTGTATTAAGATCCTTATAAATACTATTAGCTAATT
ACTGTTAATTAGCTATTACTTAATTAGCTATTACTGTTAATTAGCTATTACTGTTAGCTAATTTACG
ATCAAAATTTGTATTGGATGTCGATGATTAATAAATCTTATCAGCTCTCTATACAAATATTGTTTG
AACAGTAATGGATCATGCTTTATTTACACCCTTTAAGAGCCATTCGATATTCGATAATAGAATG
AGACAACACAGACATACTAATTGATTTCAGTGTACTCTATTCTCTTCCGTTTCTAATTTTTCAGT
CTTGAATTGATGCTTAATCCAGTGTCAACAAAATGCGATCACAAATTCTGTCGGTGAGTTTGA
ATAACGTTTTTTGCTTAAATTGTACGCTCGGTTTTATGCATAATGATAGAGCTGAAAGACATTA
GGCAGTAGACGTGCAGTAGTGAACCCTAATATTATTTAGGCCTGAGTAGAAATACCTTGAAT
TGACTTGACACATCTGCAGACTTCTGGCATTTTTCATAGTTTCTACCTAGATTGTGGTGCAGC
CACCATGTCTCTAGACATACCATGCTATCCTAGAGGATGCTTGAAGAACTCATGCTGTTAAT
GTGCTTACAGTTTTATGTGAATTCCTAATTGATCCCAGTATATCTTATAATCGTAATCGTAAACT
CGGTATTTCTACAGCACCCACAAAAGCAAAGTCGCGGGAACCAGCTTATTCACAGGCGC

TTGTCCAAAACAAAATCTATAGGCAGCAGGTCAGATCCAGAGAGTCAGACGGCTATGATG
GCTGGTGTTTGGAGTTGAGACGGGGAGGGAAGTAGGAAGAAGAGGATAAATCAGGATAG
GATTTATTGAAGAGAAGTGTTTTCAGTTTGGAGTGAAAGAGCTGTGGAGAGATAGCAAGGG
GTGAGGTCTTGGTGAGTTCGTAGGATGGGTCAGATAGTTGTCGCAATGCTGGCGGGAGTTT
GTTTATAATTGTATACTTAATGTAGTTATAATATTGGTTTGTTTTTCTTCAGTCACTGCATCTTAAA
GGTCATTGGTCGAAAGCCTTCAACTCCGTGTCCTCTATGCAAGGCTCCTGTTACGAAAAG
GTTTGCCACAGCTAGTTATTCCGTTAGGGTCCAAAGAGGACGTCTATGTAACATACTGGCC
TAGAATTTTTGATGTAGCCCATTGCTAACGGCAATGACACTGAAACATTTGGACGTTTTGGA
CGCATTTACAGATGCAAAAACGTCTAATTGGGCTTTAATGGATACCCTGTCCGGAGCATGTT
TTATTATGGCTTATTTTTATCTGTGTGTGTATGTTGTGCTCCTTTTGCTTGTCTTAACGGAATTTG
TAAGTCTTACCCGTGGTTTGCACAGTTGTCTAGGGAATCCGTCTGGATGTAGTGTAGGTTGT
CCCTAATTTATTAACCTGTTTAGTCTTGACTTTTTTTCTACACGTGTATATTTTGTTCTTCTTTTGC
TTGTTTTTATGGAAATATGGAAGTATAATGGGCTTTTAAGATTTACAGCTTGCTTACATCTGTGG
CTTTAGTGTAAGTCATCATTAACTTATTCACTGATTTGGTTATATTTTAGGAGTCTTCAGGATTTA
GTCGAATGGAAGAATTTTGTTGGAAAAGTCAAGCAGTTGGTTGATGCTTTTAAGCTCGATCTA
GCTAAGGAGGAGGAGGTGACTAAAGAGTGCAGGGAAAGTGCAAAGGAAGCAGGGGTAC
CTACTCCATCTCCAGGAAAACCTTCGGTAGTTTATGTACATCAGGGAGCAACGTCTACCAC
TCACCAAGTCAGAAAACGAAGAAGCGTTCGATTTAGACTAGAGGATGAAGAATTGAACAGT
AGACAGGATGAAGAAGATGCGGAAGTTGAGCAAACAAGCAGTGGCGCTGGCAGCAACA
GTACTGGTGTAAAATGTCGGGAGAGTATTGAAATACTATCGACCACTAATAAGATGAGTTCAG
TTACAAGTGGCGTTTCCAAGCAGACCAGTTGTCGTAGAAATGACTTTGTTGAAGTTGAGAG
CAATGCAAGTGTTAAGTGCAATGCCACTGAAGTTGAAGCATCAGACGAAGAGGATGATGAT
AACCGCGGTCTTATAACCGGTGGCTATCAAGTAGAAGTTGGCGTTGACATGGAAATGAGAC
TAGTTACCGAAAATCGTAAAATCTTCAAGAGCCGTGCCTTGTCGAAAGAGGTAAGACCAGT
CGAAGAGAAATCAAGAGTTGATCAAAGTGTGGATGATCCCTTCGCTTTCGTTGGCAGCCAA
CCGACACCGAAAAGAAAGAAGTCTGTGCAGAAGCAGCTGAAGGATGTAGATGGCATTCA
CATGTTTGAGGATTTTAATGTGTTACCAAAGAGAAGAAAGAGAATGTCCCTTCGTGAACGTT
CAGAGCTTCATCTACCTGCCGATAAGAAACACGCAGCGGAAGCGAAAGAACCAACGGC
CGACGAACATTGTTACGAAATTCAAGCTATAAGTGACAAGTTATCAGACGCGGAAAACTATA
ATCTTGTTCTTAGCCAGCAGTTCAGAAACAGTGATGCAGAAAACATTATGAGTCAAAAGCG
CATGGAACATAGCAGAGCAAGAAGAAATCTAGTGTCTGCTGGTAGTAAGATTTTGCCGTTAG
CATTGGCAGAAAATCGAGATCAGATGCAGAAAAGCTGTGCGATTTTGATGCAGAAGGACG
ATGAGGAAGTTCTGGTAATTCCAATGACAGCTGGTGAGACCACGAAAAGCAAAATGGTTCC
ACACGAGATGACACCAAATGAGACATCACCAAATCACACAGCACCAAATCACACAACAC
CAAATGAGACGTCACCAAGTCACACATCATCAAATCACACAGCATTAAATCACACAGCAC
CAAATGAGACATCACCAAATCACACAGCACCAGAGAAGGCTATACCAAAGATCAGAGCTC
CCAACAGACCGAAGCCAAAGGTAAACGAGATAGAACAAAGTGAGACTGTACCCAACAAA
ATACCAAATAGGGCAAAACCAATTAGGGCATCAACAAAAGAGGCTTTTAAAATGGAGGCTA
TACCAAACAAGCTAATGCCAAGTAAGGCAGTTCCAAGTGAGATGGTTCCAGACAAGACAG
CACCAGAGCAGATGGCTCCAAATCCCAACACTGAAAAAAACATAGGATCTTATTTAGAAAG
AACAGGAACTTCCCCAAATACCGAAGTTCTGGTAAGTCCTTTGATAGCTGGTGAGGTGATG
AAAAATAGGATGGTTACACATAAGATGGTACCAAATGAGACATCACCAAACGAGTCGTCAC
CAAATGAGATATCACCAAATCACACAGCACCAGAGAAGGCCGTACCGAAGGTCACTGCT
CCAAACAGACCAACGCCAAAGGTAAACGAGATAGAACCAAGCGAGACCGTATCCAACAA
AATACGAAATAGGGCAATACCAATTAAGGCATTACCAAAAGAGGTTTCAACAATGGAGGTC
ATACCAAATAAGCTAATGCCAAGTAAGGCGATGGTTCCAGACAAGACAGCACCAAAAAAC
ACAGCTAGTCCCAATGTTGACAAAAGCATAGGATCTTATTTAGAAAGAACAAGAACTTCCCC

AACTACCGATGTCAGTTTCCGAGCGGATAGCGGTTCAAATTCAGGGACATCCAAGGTTTGT
GAGGCTGACAATTATGGTCATTCAAGTAACCAGACTGGTGTGGAAGAGAACGCCGTTTGTA
TGCTCGAACTGGATGGAGATGTCGCGGTTCTTTGTTCCGACAAGCCTCCAAAGCGAAGAA
AATGCTCCGAACTAGTTCCAGAAACAAATGACGACAGTTTGGCTTCAAGAAATCTTCCGAA
AACTAGATCGAGTTCCCGCAAAAGGAGTCCTTTTCAAACTGTTGAGACTAAATGGCAGAAG
AAATACCAGAGAAAAGTGTCTGCAAGAGACGAAAGAAAGAAAGCCATCGAAGAGTTCAAT
AGACTTCAAGATGTGCCGACATCGAACCGCTGTGCAGCTTCAACTTCGGATTCAGGTAAA
CCCTTTTGTGTGTGACAAGTTTCAGAATTATTGTAGTAAGTTAACTTCGGGTGTTGTTAAAGTT
AACTCGATGTTTTATTTATCGTAAGACTTAGTACTTAAATTTGAATTTTCAGTGTGAATTTAAGAT
AATATGGGACATGGTGGCGCCTTGGTTGAGTTGATAGCTTTCAACCGGAGGGTCGTGGGT
TCGACTCCGCTCTAGCCGCCATGTAGGGACCTTGGGCAAGTCCTTTACCTAGAGTTGCCT
GTGCGCTTCGGCGTGAAACTCCGATACAGTATCCGTGCTGTAGTCGGGAGCGCCTCTGA
GTAGTAGAGGACTTGAAGGGGCACTATAGAAATGGCCAGAATGAATGAATAAGATTGCTTG
AATTGTTGTTTGTATACATTTGTCTGATGCAGAAAGAATGGTTGTCGGACCACAACTCGTTTA
CAAACGAGAATAAATGTCAAAAATATAGTAAAAAATTAATGCAACTGTTGAATACAGCTCTGC
GTGGCTGATAAGCCATTGTCAAATTCAGTTGCTTGATAAACTTGAATGGTGTTGTAAAAGCTT
GTCTGGGTGCTTGTAGTTATAATTGAATTTATAAAGCATCACTCAGAGATTCTGAGGTGAATAC
AAGTGAAGTTCTTTTTATTATTCTTGTGCCTTTTAATTGTTTTGTTATCTATTTTTTAGGTCTGATTC
AGAACAAGAGTCCTATGGACAGTGAAAGTATTACTTTAATTGATGATGATCAGGTGCAGTGTT
TAGAAGAGGAAATATCTGCTGGAGAAAGGGAAGAACAACCAAGAGGAGAGGAAAGTGGA
GAAGGGGAAGAACAACCAAGAGGAAAGGAAAGTGCTGGTAGATTGGGAACCCAACCGA
TGAAAGAGCAGAAGAAGATTGATAGATTGGAAGAACAGTCATCAACAGTTGCCGTAGCTGT
TGAATCCTCATCGCAGTCTTCCATCTCATGTGTTCCAGACTCTGTCGAGAACGAAGTGGAA
GGGAACCAGTGCTTTGAGGAAGTGGGTGAAGCGGTCCGAGGTAAAAGTGAGACATACCC
CATGTTGCAGACTTTCACTGAGAATATAGATGAGACATTTGAATGTAAAATTGTCAAAGAGAC
TTTGAAGTCTGACCGGCTGTTCTCGGAAAATCTTGGAAGCGATGTCGTCTACACGGAAAGC
GGAGCCACTCCATTGGGTCTTCCTCAAATGGTGGCAGTGCTTGAAAACTCTGGAGAAGTTA
ACGGTGTTCCAATACGAGAGAATGAATGTAAAAATGCAGCGGTTGTGCCAGAGGATGTCGT
GCATAAGCCAAAGGAAAAGAGAAAGACACTTTCACTTGGAGCCAGTCCTTCTCTGACAGG
TGAAAAAATGCTCTGTGTCAGTGAAAGTATGTTGTCAGAAGGTTCTAGAACAACAGGCAACA
TTTCTGCAGAGGAGAGTCAGCCCAGAGATTCAATGGCTGCACCAGCTGACTTTCTGCTTAA
GCCAAAAGCTAAACGAAAGAGAAAGACGTTTTCACTTGAAGCCAGTCCTCCGAGAACAG
AACAGAAGATGAACAATTGCAGTAGTTTGCAGACAGATTCGATTGACTTTGAAGCGGTCATT
GGCGAGGCAGTTCTTGGAATGCTCGGCAAAGCAGACGCTCGCGCAGACGACGATCTGT
CTACACAGGAAATTGAGATGCACGAGGCTGTTTGGAAATCTGGTCAGACAGCGAAAAGAA
ATGGTTGTGCTGTCGGCGTGGCTACCTCTGCGGTTGCTAACAGCCAACGTGTGCCTGCG
GTAGTGAAGACTGGAGCGAAATTGGTACACGAGACTGAAAGTGGAAACACTGGGGTGACA
GAAGAGAACGGAATCGAGGATAGAGTAACAGCGGAAGCGGTTAATTATGACGAAGTAGTT
GCACTTGGTAACGGCGCAGCACTTCGAGATCGTTGTTACTCTGAGTGCGTTGAGGACGGA
AGTGAATGCGCCGAGACATTCATCAAATCTAGTTGTGCCAAGGAAGAAACACAATCGGATA
GAAAGTCGGAATGCATTGCGGTTGGAACATACGATTCGTCGTCGTCCGTGCACAGTAATAG
TTACAGTCTCCTACAGCCACCACCACCACCGTTGTGTATGATAGGTTTAAGATCAGATGAGT
TGAAAGTGGATAGCGAGCCAAAATCTCAGCTTGATATTTTGTCTAATAACAAAATTGATGGG
GTTGACGCAGAAACAGCGAAGGTGGGAGCGGATTTAGGAAATCGGTCGGAACTACAGAG
TGACCTGGTTTTGGTTATGGAATCAGTTCCAGAGACTGATTCCAGCTTAGGGTTAGATTTGAT
AACAGTAAATGAGCAGAAGAACTTAGGTGGAGAATCTTCTGATATCCAAAAGTCGCTAGAA
GACCCTGCTCATCAACTATCTTCCACTTGTGAGCCGACATGTCAGACTGAAGAAATTGATG

CCCGATCACCTGAGGGTGTTATACAAGATTCCAAGAAAATTGAAGTACCAATGGCCTTTGTT
AACGGTAAGATCTTAGGCCGAGGCCAAGCTGTGTACCAGTTCATATTTATAATCATAAGCAT
AGATTCTTATTTATATTTATTTATATTTATAATCATAAGCATGATTCTTATTTATATTTATTTATTTATTT
ATATTTATTTATTTATATTTATAATCATAAGCATGATTCTTATTTATTTTATTTATATTTATAATCATAAG
CATGATTCTTATTGGCTTTACCGGAGGGCAAGATACTGGCAGCCGTCGTCACAGCATATCA
TAACGGTCGAAGTCAAGACATTTGTTGATTGAAGGAAGCAAAGGGTTTGGTGCCTTGCGTT
CGGAAGGTCACAGGTTCGAATCCCACTCTGGCTGCCACTTAGGGACCTTGGACAAGTCC
TTCACTCGTAGTTGCCTGTAACGTTTCGGCTTGTTAACTCCGACACAGTAGCAAGGCTGTA
GTCGGGAGTGCCTCTGGGTAGTAGTGAACTTGAAGATGCACTATAGAAATATCCGGAATGA
ATGAATGTAAGTGTTTGTGAAGTTTCCATTTAAATGTAGAGATGCAAGGAGAATTAGGTACGG
TTGTTGTGAGATTATTCCAGTTGCACTGTTTATAAACGTCCTGAAACATTTTGCTCTGCTTTTAA
TGTGTTTTCGTTTATGTCAATTAAATGCCTTACTTCAGACGTTCGTCTAGCACGGTGTTTCTATT
TTACAAAGTTGATTCTATTTTTGCAGTTGCTGAGAAAAACGAGAGTGATGATAGTGATGATGA
GGTCATGTCGCCAGTTGGTCGAATAAACCTGACGTCATCAGGCAACTTGTCAAGTGAAAGT
GAAATGCTCACAACTCAAGTATGTGAAAGTGAAATGCTAATAACTCAAGTATGTGAAAGTGAA
ATGCTAATAACTCAAGTATGTGAAAGTGAAATGCTCACAACTCAAGTATGTGAAAGTGAAATG
CTCACAACTCAAGTATGTGAAAGTGAAATGCTAATAACTCGAGCAAGGTGCTCTCTTAGGG
GTCATCCATTTATGATGTCCACAAAAACATCAGGTTTTTGACCCCCCTGTCCACATGGGCC
GGACGGACCCTCCCCTTGTGGTCGTCCACACACGGTCGACATGAAATACACACCGCTCT
CTTGAAATGGCTAGTACAATGACCTACTGGACCTAAAGCTGAAATTCGACTATATGATTCTAA
TTTATTTAAACTGTACTATTAGTAATTTATATCACTAATTTATATCGCAGAAAAATTTCCACTTTTTT
CTGTCCAAAGACAAAATTCTGGTAAAAAAGACGCTAACTTCTTTGCATGAGAAGTAGACAGT
ATGACGTCAGTGGACTCTAATTTTAATTTTCTGTGTGGACGTCCACATGGGGCTGGACCCC
CCCGTCCACATGCGTCCACCTGAGCCTAACCCCCCTCCCCCCTCCATGTGGACGTCAT
AAATGGATGGCCCTTACTGGTAGTCTGTCTAGATAGATTGCTTCTGCATCCTTTTATTTATGTT
GTTTACCTTTATGCTCTACATTAGACTTGTGTGCTGCATGGAGATGTATTTTTTGACGGAGAAA
TATTTCTGCAAATATTTACGTGTTTAATGAAATATTTCAAAAACAATGCCATTATGCCGACTTGT
TTCTCTCTGCCCAAGGGGCCACAGCACATATTTAGTGATGCTAAATACATACAATTATGTGG
TGCTAGGATGGAACACCACCTCCACTTCTTTCATCACTCTCCTCAAACCATTATGTTAGGGT
TGGTCTAGGCGACAAAACATATTCATTCATTCATTACGGAGATTTATGTAGCGCCCCTTCAA
GGTTACTACTCAGAAGCGCTCCCGACCCTTGCACGGCTATAGAGAAGAGATATGGCAGA
CATTGTACAGAAACATGCGGCAGATATTGCACAGAAAAGGGAAGGGTTACTATACCTAACA
TACATGTCTGAAATGGCACAAAATAATGTTTTCTATACTTGAAATATTTCAAAGTGTTTTTTGGC
CAATGTAATATTTAAAAAATACCGTCATAAAGGATATATTTCATTTGAAATACTTCAGTGCAACA
TCTCTAGTGCTGCGCTAACTATTTCCTTTCTTTACAATTTATACTAGTTTAATGGAACCGTTTTC
CGCCTGCGATCTATTTAGCGGACCCAATACACTTCCTTAAATCTAAGCTCAAACTTATCTGTT
TGTTAAGGCTTATCCCGCTTGTTAGTTGCTTGTGAGGAGCCTATGTAGTGGTCTGGCTGTGTT
ACGGCGCCATATAAATTATCATTATTAGTGTTAACTAGCTAAGCTTCAGCCATTATTTTGCATAT
AACCTGACCAGATTTCCAAGATTTAATCACTGAAGGAGTGATCCTGCTAGTGGGACTACTC
CCTCTTTGTGGATTTTTCTCTCCCAATTGTCGTCATTTCTGTCAAAACTGACAGTTCTTTGATC
TTGTTTCTCTTGTGACCTGTGCTTGCTGCCTCGACCAAACTCACCCGCGTTTTATGATTTCC
TCAAACCAGAATTCTTCAGCTGTGGATGGAGTGGGAGCGCCCTCTGAGTAGGTTTCTTGAA
GATGTGATATGTAGATTCTGAGAATGAATGAAAAAATTAGATATGTTTGACTTTGGACTTGACC
AGAACGTGAGCTTGCATCATCTTGACTTGGACTCAAGGGTTTTGGGTTAACCATAACTCGTT
GAACTCTATTAATTGTCCTATTTACGTCTGTAATCACTGCAAGCAATTAACTTTACGTCCAAAA
TTCTAGGTCAGGATGCTAGTATGCTTCCTTTTTGGACTCCAATGGAAACGCTGGACACAGC
GCTTAGTTTAAAGCTGTAAGGTTTTTCTGACCTTCTGGTTTTGTCTTAGCAACGTCACGATCT

GGAAGTCGAAGTGAAAACAATGGAGGAAGAGATTGCAAGACTTCAGGCTGAACTAACCAA
CGAAGTCAAGACGTTGACCGATAAAATCGACGTTCCTGTTGTCGCCTCGGAAATGAGCGT
TTCTGAACGAGAAGCAAAGAAGCCCGACCTACCAGCGTCCCCTGGACAAAATGGACTCA
ACCCCAATACATGTGACTCCTTTGAAGAAGGTATCATTGGGGAAAAACCAGAAAGCGATG
GTGTAATAGTCGTACTGGATTCTTCTAACGCTGTCAGTAGTGATCGGACGACGGTAGGGGG
CGTCGTCAATGACTTAAAGACGGAACTGCGGCAGTGTCAGTCTACTGTCGAGGATGTCGA
CGAGTCGAGCAGTGAAGCGGATCTCTTTGGCAGCCAGATGTCCGACATTGTGGCTATTCC
ACAGACGCCGACACCAACTAAGAATAAGACGCCGCGCACTCGATTTTCTCACCGTGGGC
TGGAGAGAGATGTAGAGAGAGAGGAAAACCCACGGTCTCCCACTCCTCCGCCTCCTGTT
GTTACGCGTAAAAAGGAAAAGAATAGTTGCGCTGATCTGGAAAGTGAAGCGAAACGATTAA
GGTTGAGTCATGATCTAGATAACAGAGACGAAAATAATCGAAAAGTTAACGAACGCTTTAAT
CCGAGCAACGAGACCAGTCTAAAATCAATTGAGCGACAAGGAAATTGTTCAAGGAAGGTC
GTAGAAAATGTGAAACTAGCAGATGACGGGGTTGAAGCATACCAGAGAACTCCGGCAAAC
AGAGGAGGTTTCTCGTCTGTTGCTACAACGGGTAAAAGAAGTAAAAGCGCCATCTATGCAC
ATTCCGCCGTACTGAGGGAGTGTTCTTCGCAGCCCTCTAAGGAGAAGAGTCACGGAAGT
CCTTTCCCTCAGAAGAAAAACTGGATTCTTGTGGCATCTGGCATTAACCGCGCATCTGAAA
TGGTATTTTGTTCTACACAAATTGAATTACAGTACTTTATGCCTGTGTTACCACTGTTACTCATC
TGACCTTATCAGCATTAAACATAGGCCTCCGCGCCTCAGATTCACATTTTGGTGTGACCTTG
CGTGCGTTATAAATTTGAATTGTATTGTATAGGCGGAATGTATGCATAGACGTGCATCTGAAGT
TTTCTCTGTCCGAAGCAGGACGTTTGGGCGCCGGCACCAGAAACTTTGGCAGTTATCATT
CCAGCTTCTGTTTATCAAAGCATACTTTAATCAATCCTAATAGTATTTGAGGGGTCATAATTTTT
CACCAATAGAACACATATCAAGCAAGAATTCATACTCTTTATGAATCCACTTCTACTTCAGTC
ATTAATAAGAATTGTTTGCATGCATCCCGCTCCTAGTTCATTTATGCTATGATTTTTTTCAATAA
TCCTGCTTCGACTTTGAATATGGCGATTTTAATCAGTCTGTCGCCCAAACAGCCGGCACCC
AAAGTCATGTACTGGCACCCTTCTCTGTTGTCTTGCACTTCAGTAGTTGTAAGAATTACTGAA
GTGCAAGGCAACAGTAACAAGAATTCTTTAGAATTATAAACTACCACTCGTAAGCCAAGTGC
TGGGCACCAGCTTGCTCGCAGAATATCTTTTCTCAAGTCTCTACATAGACTACTGTGTAGAG
ACTTTGAGAAATAAAAACAAGCACCAACTTAGTGTGATAAAATATTGGTGATTTAAACCTTCA
GTTTTAATGATTGTGGTGGAGTGATGTTCGACAGTGCGTGCTGTGGATATTTTGTGTGTTGAA
GAATGATGTTTATTCCACGAAAGGCCACTTTAGCGTGTCAATTGTTGTGCATCTTATTCTTTCA
TTGCAGCCGCTTGTTGAAGAGTTTGTCCGAAAACTTGGCTGTAGATTTCTCCAGAAGTTTAC
TAACGGCACAACCCATGTTCTGGTTAAACCTGGTAGGTGACTTTTAACCTTTGAAGACTGAA
TTCAAAGGTGGTGCTCTTTTCTTGGATGGTTACCACACCTTTGTGGATTCTGTTGAAAGCTGA
TTAAACTTAAGGCATTTGAACTTATATTTTTGCTATTTAGTTTAAGGTGGTTTTAATTTTGTTATAA
GAGCCATCCATTTATGACGTCCCAAAAAATCAGGTTTTTGACCCCTCTCCCTCTGTCCACA
TGAGCCGGACCCCCTCCCCCTTGTGGACGTCCACATGCGGTCGACATGAAATACACATC
GCTCTCCTGAAACAGCTAGTACAATGACCTTCTGGATCTAAAGCTGAAATTCGACTATGTGA
TTGTAATTTATTTAAACTGTACTATTAGTAATTTTTGTTACTAATTTATATCGCCGAAAAATTGCCA
CTCTTTATTCCGTCGAAAGACGAAATTCTGGTAAAAAAGACACCAACTTCTTTGCAGGAGAA
GAAGACAGGATGACGTCAGTGGACTCTAATTTTAATTTCTGTGTGGACGTCTACATGGGGC
TTGACCTCTATCCCAGTCCACATGCGTCCACCTGAGCCTGATCCCCCTCCGCGTGGACG
TCATAAATGGATGGCCCCTAATTCGAACTCTAAATGTTTTGTTTTGTGCATACAAACACATTTG
GTACACGATTCCATGTTACACAGGCTTTCCAACATTTTGATATCTGATTGCCTTATCCTTGCA
GCAAACTGACTGGATCTGAGCATGAGTGTATTTAGGAGGGGTGTGGATCAAACTTGTGTTTT
TCCTGGATCAAATTTTTGTGTTTTTCTGGATCAAATTGTGGTTTTTCATGGTAAAAACTCCATAG
AAGAACCCATTCCTGTTATAAAAACCTTAAAATACACAAAAATACACCCCAAGCCAATGAAA
ACCCCCAATCAAAACCCCCAATCAAAACACCCAAAATCAAAACCCCCAAACACATTT

CTGGCTACATCCCTGGATCTGAGGTAAACATCTCTATGTGGACATCAGAAACTTTTTCACATT
ATAATAAAATGATAATTCATTTCTTTTTCCAGTGAAGGAAGGCAGTCGACGATGTCAGCGAAC
GTTGAAGTACTTCCAGGGAATTGCCCACAGGTCATGGGTCATAAGCATTGAATGGATCCTC
GACTCCATCAGGATGGGTTGCCTACAATCGGAGGTTAGTAGCGTCTCTGCCTTTCTTGCTA
GTGACTGTCTTTACTGTAGCTGCCTTTGATCCAAACGTTGGCCACACCGTGAACTGTGGTC
TATCTGTTTAGTTCTTTGTGAGACATCCTTGTAGCGGTTTGGCTGTGCTACGGTGCCATATAA
ATTGTCATCATCATTATTATAGCGATGTATAGGAGGATAAGATTGTAGATACCCTGGCATTTCC
ATTTTTCTGCATGGAAGGCTTACTCCGGTTGTATTGTAGTGACTGTCTTTCCGGTAGCTGCC
CTTACTCCAAATGTTGGCCACGCCAGGAACTGCGTCTGTATCAGCTTATACTGACAAGTTG
GTGAATAAGATTGTAAATAGTAGTTACTCTGGTATTTCCATTTTGCTGTAGGGTAGCCTTACTC
CAGTTGTATCGTATTATATTCAAAGAGTTATGAACTGTGTCTCAATATCAGTTTATGGCGATGTT
TTGGTGGATAAGATTGTAGATAGGGCTGTGAACGAATATTCGGATATTCGAATATCCGACGG
TAGTGAGATATTCGGTTTTAAAAAGGAGAAGACGAATATCCGGTCACAAAAATAATTTGTCCA
CTAACACTATCAAAATACGCTGGCTGCTAGCCACCATAGGCACAGTACCATAGACAAGCT
CTAGCTGGGTAGCTACCTGCAAGATTGAGCCACTGGTGAACGGCTGCAGTATAATGGTCT
GTCAGCTCCCCCTATGTAACCGAGGGCTTATCTAGGCACTAGACAGACAGGGATGCAGG
GTTGAGCCAGCACTGGGTGGCATCATTCATTAGTATGCACAGGTCTGCATTAGGGGCAATT
AGAGGTCCTTGGCAGCAAACAAGTAACCACTAGTCCCTTTAGTAATCACTAGCCAGCGTG
GCCGCTGATCCTCCACCGGCTGCATCAGTAGCTGCTATACCGGCAGCTGCAGTTCAAAC
AACACAAGATTATTGGCCAATAATTTATTTACCGCGCCTGGCCAATTATTGGGCCCTCAGTC
AAGCCAACATGACCGCTGATCCTCCTCCGGTTGCATCAGTAGCTGCTATATTGGCAGCTG
CCTTCAAACAACACTAGATGTTTGGCCTATTGCCGCGCCTGACCAATAATTGGGCCCACA
GTCAAAGTGTTAAACTTCTTGCCTGGGTAGCAAGCAGTCTACAATCTAGAGAAACAAATGTA
GCAGATTTTGCGTGCGTTTTGGACCGGAATTTTTCGACGAAAATCAAGGCAAAGATTTCTAA
CGGATATTCGTTAAACGACGCAACGAATATTCGGTTGAGGTTTTATGCAGTTTTTCACAGCC
CTAATAGTAGATACCCTGGTATTTCCATATTTCTTCAGGGAAGCCTTACTCCAGTTGTATTATAT
TCAAAGAGTTATAAAGCCCTTCTCATAGTGTATCATTCAGGTTTGCTCCCATTGTGAAAGTCC
CCAAGAATAAAGGCAAGTTTTGAGGCTGGAGCACCGGATAGTATTGTGGTACACCAGATAA
ACTATCCATCTTCACAAACTGACCGCTGCTGAATCGTGATCTTTTGCCCACTCCGGTAGAA
GTACGACCGAGTTCTTGTTGAACATGTCCACCTCGTATGCCACACTAGGTGGTATCACTGT
GAAATACTTTACTTACAGCTGGCATGGTCTCAGCAAGTGGTGTCCTGCTCAGCCTGGTATTT
CCATATTGCATTGTTGCATTGTGGGTTGTACATTAAGGTGGACCTAATAAATGATAACTATCAT
GTTGTGCATATTTCTGGTAATAATAAACCTGTTAGAATGATTCTTATTTGAATTTTCCTACCTAAC
AGTTAATGATGGTGGATGATGGATCGACTGAAGGTTGGACAGACGGATGAATAGATTGACA
AACGAATCGGTGAATGAATGATTAAACGAATGGATAGATGGCATCATCATCATCACTAGTTAA
CTGCAGGGTTTGATGGGACTATTTGTTTTCCAAGGACAAATATGAAATCACTGGAGATGTGG
CTGTCAACGAGGATCACTTTGGTCCGACCAATGCTCGACTTTCAGGTGTTCAGCTACTCCG
AAAATTTGAAATATGTTGCCTTGGTGCCTTCGACGATTTAACCCGTGGTAAGATTCTTTGCTA
TGATTGTCATAGTTAGCCACCGAAGAATACCACAGGTAGTGCGTAAATGTTTGATGCTTTTCT
GACTACTTTTTGTTGTGTGTGGTAGGTATAGTCGTGGTTTTGGTTTAATTCTTCTGTTTATGTCAT
GTTCACACAATGAATTAGTGGCCATTTTCCTTGCATTACAGAAGCAATTTTTACTGATTAAAAA
AAGGCAGCCACCCATTATTGTTATGTTATATGGCTATTTGTAAAACGCCTTTCACAGAAGGCT
ATTCAGAGACGCTCTCAGCATGACAGGCTGGTGAAGATAAATATATCCAGCAAATTTATATAT
GTGACGCAAGGGCCCAAAATCCGTCGGTTGTCGGGAATTATTTACAGAGATACAAGTAAAA
ATAGGAGAGAGGGGTTTTCGAGTCTGAAATTCTTCATTTCAGCTGGTGTCCGTAAATGTTTTA
TAATTTGAAAAGTTGTAACCCGATTTCAATGCGGTTTGTTTTATTTGACACAGAATGAAGCCC
TTTACTACCCAGAATAAGTTTCAGAATAATGAAGTGACGATTGCATGGGATTACGATTGCACA

AAAATCTTAAACTTTTTATATGATATTTCTCACTTTTTGATTGTTCTAATCAGGCGAGTATAACTT
CAGTTAGAAACAAGACATTTTCATTCTGAAAAAAGCATTTTGAAGCAGATGCAGTGTAGAAA
ATGATTCAATAAAAAAGCCAAAAACGACAGGTGACGGGTTTTGGGCCCCTGTGTCACATAT
ATATTGATACCAATAAGTGAACAGAAAAACTTAAGAACTTTATATTCATGTAGTGGTTGCATGA
TTGATTGATTAAGGTATTTAAACTGCATTTCCTGTATCTTACTTGTTTAGCTACATTTTATTTTTTC
TCGGTTTATAGACTTTGTGTGCATGGGAATCTTTCTGTGATTGCATTTCTTTGGTGTTTTTGTTG
TTCAGATGAATTAAGAGGACTTGCTGAAGCGTGTGGTGCAAGCAGTCTTCAGGATCCCGC
AATGTTCTCATTTGATGTTGACAGAGCTCAGGTCATCATCGTCGAAGGAAGCTCTAGTGATA
CTGGTCTACAGCACTTCAAGAGTAAGGGTGTCATCTCCATCAGGCATATAATTATATCGTTAG
GGCGATGTATGTATTGTAAGCGTTTTAACTGTTTATTTTATCTTTTTCTTCCTCATGTTAGGACG
GTTAATCGTCGTTATGACCCAAACCGGTTCTAGGACAAAATGGTAGCGGACAAAATGGTAC
GGGTAGAATCATGAATTAATCAAGTAATCCAGCTCCCACTGACAATATGATTTTTTCATTAATC
CCGCTTCCATTTACACCAACTTAGGCTTATCGTTTATGTGTAACTATACTTGTGGTTACTAAAT
ATAAATTCAATTCAATAGAATTGAAATCTAGAAATTGCTACCATTTTGTCCGTATATTGTCCAAT
ACCATTTTGTCACCCAAACCAGGTCCTTTGTGTCGGTTGGCCCATTACCCTGAAATCGCCT
CCCTCCTTCTCTTCACACGTCTATTCTCTCTGCTCCCCTTTCCTTGTCTCTCTCTCTCTCTCA
CCTAAATTCTTACCTTTTTCCTGGAGCCGAAATTCCGTGAAAGCATTTCTGTTTGGCTTATGC
CGTGAGAAACGTTACATAAATATCTACATACAATACAATTTGATGTCTGTAGACCAAAGTGCA
GTTAGCAATTGGTCATTAAAGAATATCTTACTTACTCATCTAAACTAATCTGTCGATTATGAGGA
TTATGTGTTTTGACTGAGCCTTGAGTGTGTGAGCACTCGCCCTACTTGATGTCTCGGTTTCTG
TTTCTAGATTGTCCTACTCTTAGAGAAATTTCAATGGCTTAAGTCCACAAAGCATTTGCCTCA
ACTTACTTGGATTATTTTGTTTTCCTTTTTTTATCTTTTAACTTTTTTCTTTTTGGATATCTTTAACTT
TTTTCTTTTGCAAAGGTTGTGCTCAGTCGACTCATTATCATTTACCTCAATGTCCAAAAAGAG
GACGTATACTTACATCCTGATCTAGAATTTTGGATATTGCTAACGGTGATCACATCGAGAAATT
TTGACGTTTTGGACATATTTGGGGGACGTAAATAGGTCCGTTTGGGCGTTAACGTTAACCTT
GGGCGTTAGTCCTGCTCATTTTATAGCCGAGTTGACTAGAGGGCACAGGTGCGGCAGAAA
TCCCGAACCAGATTTCTGCCCTTGCGGGGAAATGAACTGACGACCTCTGGTTTTGACAGT
CTGACATGCTGACCACTAGAAATTGGCTGTTATGCTTGGAAAAATAGTATGAATACAACTCTA
ATCAAATTATTATGGTAAATGATCCTTTAAAATTAACAAGCAACCTTGAGCTTGAGCAATGTAA
ATATCGTTTTATATTTGTATTTCTTAACCTGCTTCAGAGCTGTACAAGAAGTTCAGCGTGCCGA
TTGTTGCAAAGGAGTGGCTCTTGGATTCAATCGGAACGTACGATGTCAAGTCTCTCGATGA
GTATTCCATTTATCCACCGCTGCCTTCATAGGCCTCGTCTCAAAGATGTCAAGGGAACCAG
CGTAGGCCTTGTCTCAACGATGTCAAGGGAACCAGTCGTTTCAAACTTTTTAATCATGAAAG
CTGCTTAAAGATTGCTTTGCTGCTGTTTTGTGCCATATTGTAATTGCCTCCGATGTCTATAAAC
ACTGATTTTGTGCTAAGGGAATTATGTGAAATGGTGCTAAACTTGCATTAATGGTCTGATGGA
ATTATGTGAAACGACAGCACTGAACTCAGTGATATTTTAGTTTTGAACAGGGCATATTTTAAAC
AGAGTTTACAACTGCATACAGAAAAAAATTCTGACCTGAAAAAAATTTCTCATCTCATAAAAA
TTTCTGACGATCGTTTTTTTAGTCGTTCGCTAAGAATTTGTAAAAAAGTAAATTTTTTATTCTTTTT
TGGTCATTTGACAGAATTTTTTAATTTTCGCCTGTGCCCAAACGTGTCTCTGTACAAATACAA
AAGTACAACTGCAGACCTAAATTTCCTGACTTTTTTCGTCATTCCCTCAAAATTTCTCGTTTTT
CACCCCTATTTTCGACCCTCACACTTACAAAGTTACAACTACAACTGCACAATTCGCCTATT
CCAACTGCAAATTACATTTTACAACTGCAGAAATTGTGATCAGTTACACGTTAAACTGTGCC
CTGGTTTTGAAAATAATTTAGTTCCTATTACAGTTCACAAAAATATCACTTGCTGAACTTCCATT
AATGGTCCGATATGCTATGTGCTATAATAATGTGATGCTGTGAAATGGTGATGATGTGAAATTT
CTGTTTGTTTCGCTCTTGTTAAAACTGGAAACATTGGCGGCACTAGGTCGGTTTTCATGACA
GGGCATAGATAGAGACAGGTCATTAGTAGCATCAACAAAGGCGGTCAAAGTACCGTTCTA
CTGTGCTTTTATTATTACCACTTTTGACTGCCAGGGTATTTCTAACAGGTCGTTTGACCTGCC

ACATCCTGGTGTGGCACCGCCTTTGGCTGAAAATGTTTTAAGGAGTAATCGTTCTGGGTCT
GATGACGTTGTCGTTAGTGTTCTACTGTTAGGCAGGTTCTTGGCTTCCATATTCCACGTAGCT
GTTTTTAAGTATCCGTATAAGCTTACATAATTAGGTAATATTATAACTGAATTCATTTATTCTTTCA
TTCATTTAGAAAATTTATGTAGCGTCCTCTCAAGAAATCTATTCAGAATTTCTGAAGTGCTCGA
TCCCAAGTCCAACCACGGTGAAAAAGATCAGTTTTAAGCAGCTAGTAGAGCAAAGGCGTG
TTCATGTAGATCAGGGCTGTCGAGACCCATTTTAAAAAACCTGGGATTTTATTTTTTTTTTAAA
ACCTAAAAAACCTAGGTTTTTTTATTTTCAATTCAGAATTTCTACTTTTTCATGTCAAACTCTGTA
AATTTATTTGAATTAATTGGAATTGCTATTTCGCATATTGAATTGCATTACTTAGTCCTCATGTAT
AGGCCTTCTTCTTGATCGTATTTCTGTGTCCAGAGTACCGCGTGCGACTGTGCGTACATTGA
AACCTAAAAATCCTAAAAACCTAATTTTAAAAGAAACCTTAGGTTTTTACCAGCCCTGGTAAC
AATCGAATTTGCGAGGGAAGTGAATTTCAGATAGAAGGAGCAACAACAGTTACTGAGAATG
CACGGCGCTGCATAGAGCCTATTGGAGGTGTTTACATGGGGAACTAAGCACCGGCGTAG
ACTTGTCATAAATATTGGGGGGCAAAAATTTGGGTCACAAGAAAAATACAAAGAAAAGGGT
GTACATTTTGTAAAAAAGTTGTTGAAGACTAAAAAAGAGGTCGTCAGAAATTTCAGAGAGAAT
AGAAGGGAACTTTTCAAGATTTTTTGTCTGAAAATATATTTTCCCAAAAATTTTTGCCCCCCC
AATACTTCTGGATTCTGGATTAGCCACTAGTGATCTTATTATTATAAGTAGTAGTAGCCCTCGA
TGTTATAAGATTTACTGGTTTATAACATTAAAAGACCTATTCGTATCACGTTAAATAACTTCGTTT
CTTAAAAATTATGGGATGTCCTTTTTGGGGGGGAGGGGGTCTGGCTGAAAGGGACAGAAA
AGGACATGGGGGAGGGGGGTCAAAAATGTCAATTTTTTAGGGGACGCCCTTAATGGATGC
ACCCTATTCAGACTGGCAGTCAGGGTCAGTCTCAGAGGTAGGCCTAGTCCAACTACAATG
TAAAACCGAGCAGCAGTGATCAAGCCGACTAGTAACAAAAGCATGGACTAAGGTAGGTAC
CGGTACAGCATATAAGGATGAAATTTATTTCACGTTATGCCTACAACCAATATACTAACTTAAT
AAATAAGAAAGTATTAGACGATTTCATCAATTTCTGTCAATTTTCCAGTGCTTATTTTTGACATA
ATTCCACTTCAATATTTCAATTAAACTTTCAAAAAGTGCCCCTTGAAAGTTTAATAAATTTCGTG
AAATTTATGAAAATTTCAAAATTGAAAGGATTCATAGCCCATCTCTACCTCTCGTCATTTCAAC
TTTATTCACCGTCGGTTTACATTTGCGGTTTTCAGTATATTTCAGTTGTACGTAATAAGCAATTT
CAATAAGAAAGTAAGTATAACCTTTATTTTCATCTGACTATCTCAAATGCATTACATTGCGTACA
TGCGCAACTAGTAGTAACAGAATATAAAGTCACACATGCGCAACCAATATTAATTTAATTGTC
GAGTTGTTTTATTAGCATTTGCCCAGTATCGGTTCACTACCAGTTCAACCGATCAAACCCTG
TTTACCATACTTGCAACGCTCCTTCTGGACTCTCAGGAAATGACTAGGGTCTAGGCTATATG
TCGCGCAGCGTAAAAATCTAGAGTGTATAAATACTCTCGTTGTAAACACTTCTATCACAGATA
GTAGATTTAGACAGATCGGCAGCTCCGCCCACTTCCCATGCAAACTGCTGTAATCAATGTA
AACGGCCTGCCACCTACCGACGCTGGGGTTAGAAAGACAAGTTAACTATGGCGTCTAATC
ACAAGGGCTGCGCTGTTGTTTACTGCAGCAATACTTACTACAACGTGAAAGAAAAGAAGAA
GTTTAGCTTTTTCGGATTTCCAAAAGATCCAGAGAGGTATGACCTACTGCATTTTCAAATCAA
CGAGATGTGTCCATGCGAAGCGGTGACAGATCAGTTAATCGACCGGCTACATGTACTTCC
CAATGTTGTATTTTGCATGGAGCTTGCGTGGCGGTAAGTTCCGCCACGAAAAATAGGAAAA
ATACGCTATTGCGGTGGAAATGTTGCTCTAATGACGACACTAACCCTAATATTAACCCTTTGT
AATTTACATCGAGCCGATTAGCCCACTGGCGTGCAGAGGATCTTGGATACCCGTGGCCAA
CGAGGTTCTTGGATGCCCACAAATACAAGTTTTCAATATTTCCCTGTCATCTAAGAAAATATTT
TTACATTCCTGTCCAAAAAAATTCTGATGACCTTTTTTTAGTCGTTTTACATTCCATCTCCAAAAA
ATTCACTGTTTTCACACCAAAAAAAAATTCACTGTTTTCACATGTGTATGACATGTGAAGTTACT
GCAATAGGAAAAGAGTTAGGCCTATTTGTAAACTGTAAAACATGTTGTGTGATATTGTAATCTC
CAATCCTCTAGAGCAGTGATGTCCAACCTTTTGACTCTGTAGGCCATATAAAGAAATAATCT
GGAGCCACAGGCTACAATGACCTCTGCTGCCCTGCAGTGTCGGTTTTGGCAGCACATCA
GGTGCTGCGTTCTGCTGCGAGGGGTGAGCTTTTGGGCCCATTTATCTACTGTGCAGCGAA
GGGCATTCTCGGTTGTGGGCCTATCAGTACGGAATGATGTCCCCGTGAGCTGCGTTCCTT

GCTGATGACCTGCCCTTCCAAATTTTACATTAACCTCAAGTCTTTCTAATTTGGCCATGACTG
GGCTGGGAGCGCCTCTGAGTAGTTGTCTTGAAAAGCCGCTATATCAGTCTCCAGAATTAAT
TAATGAATAGTAAACTCAAATGTGGCCACACAAAATGTAGCCGGATAAAATGTCGTTGGCAA
AATGGTACGATTGATATATCGCAAGTATTTTCAACCGGTTCTTCTTCCACTATTTATTACAATAC
TATAGTTTTCAACCGGTTCTTATTCCACTATTTATTACAATACTATAGTTTACACCTATATCATGG
TCACACCAGTCTAAACCAATCGTCAGTCAGTTAGGGTCAGAGTTAAGGTTAGTGTCGCTGTT
ATAAAATGCCCACTACACTTTCGCAACTTAGCCAAACTTAGCCAAAACTACTTTTCTCAGTC
CAGTTTCTAGTTAACAAAGAATGCTTTCATCAGTCCAGCTTCTAAATGACAAAGAATTCTTTC
ATCAATACAGCTTTTCTTTAAGCAGCATCCTTTTTATTTATCCAGCTTCTACTTAAGGAATAATT
CTTTCATCAATCCAGGTTCTATTTGAACAGCATTCTTTTCATTCATCTATATTCTACTGAAACAA
GAATTCCTTCCTCTATCCACCTTCTACTAGCTTCTGCAAGAATTTTCTTTAGCAAGTCCAGCT
TCTAGTTAACAGAATTTTCTCATCAATTTTGCTTTTGCTTATTCACGAACGGTGTGTTGTATTGT
ATTGTATCTATACATTTATATAGCGCTTCTTTCAGTGCACATCAATCAGATGTGCTTCCAGTGTA
AGAGACCCAGAGAGCAGTTTGAGAGAACAAAGTTAATAAAGTGTAACGTGTAGAAGGAAG
GAGTTGGTTCCAATGATTGCAAAGGCTCATGTCAAGTGAGCGTAGCAGACGAAGAGAGAT
GGCAGAATGATGTTGACTTGGAGGGTTGTCCAAAGGATGGCCCAATGGAGGTTCAGGTCC
GTTTGCTCTGTCGATTATAATAGCTAGGGTGACAGTGGAAAGGCAGAGGCTCAAAGCCCAT
AGTTGTCCAACGAAAATTTCACCGTTTGTTTCCACAATCCTCGGTCTTTTGCAGATTGTATTG
TATTTAAGAATTTATAAGGCACTTTCAGTGGGGTGAACCATTCAGAAGTGCTACCAGTTCATG
AAGGCAAGTGTATTGATAGTATTGTTGGGAGTTGTAGGTTTGTGACAGAATTTGGAATTGGATT
CGCTCTGAGATTTTTAGTCTGTGGATTGACATGAAGATGGGAGTGATATGATGATGTCTTGGC
ATTCTGCTGATGCTCACATGAAAACAATAACAAGGACATAATATTATAATAAGACCATCCATA
GGTGTAAGCTGTGAGTGTTGACTGTATTTTTAGATCAAACCAGTGGGTTGTCAACTGTCGCA
CAAGAAACCTTGACAAAAAGACAGCGGCTGAGTTACACAAATACTACTTGGTCTCAATGGT
CTGTGGAGAACACTTTGAAGACCGCATGTTCATGAACACAACGAGCAGAAACTCATTGGT
CGTCCCTCTTGTTATAGCCAATAGGTTCAAGTGGTCCTTGGATTAGAAACTATTGAATGGGT
GAAATAATTGAAACAGCTTGAATTCTGTAAAAAGTTTAACGTTTATGTCTCTTTTATTGCTTCTG
CTTACGGTATATTTTGTCACAAAATATGTTCCCGATGGTGTTAAATGGAGATTGTTCGAATCTG
TTGCTTTACCTGGGTTAGCTTATTCACTAAAGACTGTAAACTTGTCGTCAGCTCACATGCATC
AATGGGAATCTGTTAAAGACATTCTATTCTTTTCATAATAGTCCGCAACGATTACTCCTTGTAC
ATATTCTGAAATAAAGCTTTTTTGTCAATAATATTATTATTATTATATGGAACTTACTTGCATGCAG
GGGCGGACTGGTAGGGTGGAAGGTCATCGGGAAATTGACCGGGCCGGTTTTTAATCCCA
GCCCGCCCCTGCTTGTATAAACAGTGTTCTCAGAATTTCTGGCAATGGATATAGCTACAGTA
TGGAACATTGGGTATGGAAAAATAGTAAATAGGAAGAGGGTAATTAGGAAAAGAGAAATGAA
GAGAAAGGATGGGACTAAGAGGAGAAAGGGGATAAATGAAGGGAGGCAGAATGTAAGAA
GGGAGAGCAATAGGAGGATGAACATTAGAGAGGAGAATTAAGGAGACTGTAGCAAAGGAT
CGAGGAGGGATTGAAAAGAAGTTAGGCTTAGATGAGGTAGGGGCGTCCAGTACGTGCATT
GTGTGATGCATATATGCATCATGGTCAAAGTAGGGGGGAAACGAAAAACACAGAAAGTGTG
TAAGAAAAACGTGAATATGACGAAATCAGATTGAAATTTGCAAAAGTAGTAGAAACAATAATT
TTCCCGAAAGTTGCAGTGAAGAGAAAAAAGGTCAGAAATTTTGGTGGATGAAAATCGGAAA
ATTTTCAGGGAAAAGGTAAAGTTCGGAAAATTTTCCATGGAGTAAGAAATTTTTTTCGGAAAT
AGGGGGAAATCTGAAACAAGGGGAAAAGCATCATTGCCTCAGGGGGATGGACGCCCCT
GAGAGGAGGGCGTGGAAACAGAAGGAAAAGGCTCAGAGCACGATGTGGGAATGGAAGA
GAAGTAAAAGGATGTAGAAGAATAGGAGATGACGATTATGATGAAGCAAGAGGAGGCCTAA
ATGTTGAGGAAGGGAAGAAATCAAAGGAAGGAATGGAGAGGAAAGCAGAGGCTGTGAAG
AAGGAGAGGAGAAACAAAGCAAGAATAAAATAAAAGAAGGAATAGAAATAGGAGGATGAG
CATTATATAGGAGGAAGCTAATATGTGCAGGATGTAATGTAATGTAAAATTCCGAAAACTTCTC

AGCGCCCACAAAAGCCACGTCAGGGAGACCAGCTTATTCACCGGTGCTATACCAAATCC
AGTCCGCACTAGCCAAACTTCTCGTTTCGTTTCGGTGCCAGGTCAAAATCCGGGAAGGTC
AAGCCGGGGATGAGCCAGGATGAGGGAGGGAGTAAGAAGAGGAAAGAATGAGTAGGGA
GATTGGGAAAGAGGAGGGAGTAGGCATCGAGGGAGAAAGAGGAGTACAGAATGATGAGA
CATGAAGAGAATTCAGAAGCAGCTGTAGAAGGGAAGTAGGATAAACCCAATATGTACAAGA
GGGAATAGGAAGAAGAGGCCATAGTAGGAGGTGGAGGAGGAGTAGCAGGGGAATAGGA
GGAAGAGGGAGATGAGGATAAAGGGCATATAGGGGGAGGAGGTGTAGGGAAATAGGAG
GAAGAGGGAGATGAAGATAAAGGGTATAGGTGGAGGCACAGGCATAGACTTTGGGGCAG
CCTGGCGCCCATGCATTTATCAGTTTTTACCACATTTCCCCCCTCAAATTTGGGTTTCCCCA
ATATTTTTACAAGTCTACGCCAGTTGGAGGAGGAGGAGGACTAGGTAGGGAATTGGAGATG
AGGACTAGGATGAAGAGGAAGAACTAGGATGAGGAGAAAAGAAAGGAAGGCTAAGAATC
ACAAAGTGCAGGAGGAGGGAATAGGATGAAAGGGAAGCGGAATGGGTGGAGAATACGG
ATGAGGATAAAGTGACCATTACGTTAACGAGGATGGTATTGGAAGAAGAGGAGGAAGGAG
AAGGCATAGGATTAGATAACTGTCTTGAAATGTGACTAAGGAATATAAACGGACACGTCTTA
GCGATAGCATTTCTACTGTTAATGCTATCGGCGCGATTTTTCTAACCCCATGGCTGCCAGGT
GGCGCTTTTTGACCTAATTACACGAAAATGGGAGGAGCTTCCTATCTGGCTAAATCTACTAT
CTGTGCTTCTATCATAATGGGTGCCTTGTGTTGAACTAGGTCGTTGTGTTGAACCTTTTGCAC
ACGACACGTTTCATTTCGCTATTCACATTTCACACGGAATTGTGCAAAACGCGTGCAGACC
AATAGGATGGCTTTATTGAGAGGCGTAATCAGGACAACCAATAACAATGAATTGCGACGGG
AATCATCTGACCAATAGGAACGATGCCCAGTTTCTATTTAAACGACTATGCAGCGGTGCCA
AGAGATCGATTGTGATGCGAAGAAACCTGTCTACTTGAAGATAAGAGGAGTCTGCTAGAAA
CCCGGTGCTAGAAGTAAACATGGCAGACGCACTCGACACTCGTAAGTAGACTTATGTTAC
CGTGGGAGTACCTGAAATTTCATTATTTTATGATGCAGGCCTAGGTGCGATATTGAGGTACTG
CAGAGTAATTTACTTTTCAAGCCCAGATGTGTACAAGCCATTTTTAGTTCTGTGATGATGATGA
TGATGATGATGATGATGATGATGACGATGACGACGGCGGCGGCGGCGGCGGTGGTGGTG
GTGATGATGATGATGATGATGATGATGATGGTGGTTGTGATGATGATGATTATGGTGGTGATGA
TGATGGTGGTGATTATGATGATGATATCTGGCCAAAACTAATTTTTCATTAGTATTCTTTATTTATT
TCGTAAGGCAATTAACATAATTACCATCAGCAAAATTTAGCATTGCTTGAGCAATGTTTTAAAT
AAGAGGCTCTATGCTACTTATGCAATTGATTTCTCATAGAAGATGTAAAACATTTAAATAATAG
CAGTTTACATTACCATGCTAAGGGTATAAACTTTGTAATATCAAATATCAGTCACTGAAATCCG
ATTATGTAAGCTGCTATATTCTGCCTCCTCACACCACCTAAGACCAGCTAAAAAAAGTCAGA
CCAGAAGCAAGGCAACAAGCTCACTTGCCAAATTGGATAGAGACAAGTTGAGGAGATGC
ATTAGGCCATTAGCCAACGTCCAGATGGTACAGACCTGTACAGGAAGTACCAAGAAACTTA
TTCTGCCTTGTTGCTAGTTTCAAATTGTTTGCAAGGTCTTTTTTGTAGTGTATAGTTACTAACAA
ATCTTTGCCTCCTTATGCTTTTAGAGTGCTGTGGAAAATCAACCTGCCAAAGTAAGCCAAAC
TTCTTACTTGCCTTAACAATTTAACATGTCTTTGATATCTCTCACAGTTAGTAATTTTATTACCGC
GAGTTGGATGTTAGCATTGTTACTTCTTCTCTTTCACTGTTACATCCGTGTTATAATAACGACAT
TTTACCCGTTCTTGTCATCACTACTGGCAATATCATCACTGTGACTCATACCATGTTAATTTAA
TAGAATATAATGTGGCAGAACACTTATATAGTGCCTATCGAGAACCTCCCAAAAGTGCAATT
GCTAACACCTCCACCACCTTAAATCTTTCTTTTCGAATGGAATCATTCCTTTTCTTGTCGCCA
CCATCAAAATAATGCTCATGGTGTGGTCATTACTACCATCATCTATCAGCAGTCATCATCAGT
TACCAAACGTGTCACAAGACCTCTTGCGTGTACAGGAAGTACTAAGGAAGTTATTCTGTCTT
GTTGCTGATTTTAAATTGCTTGCAATTTACATACATTTTTATTTTGTAAAGCCGAAAATCTCAGT
ATATTATTTCAGTCAAGGAGAATGACTCTTTTATTTTTTTAGGAGAGGGATCAACTTGTTGCTG
CACAAACTGCAGATGTTTGAAAAGTGATTGCTTGCCAGGCTGCAAAAAGCTTTGCTGTGCT
GACGCTGAGAAGGGCAAATGTGGAAGTGAGTTTGCTATTTGTGTTTTGTTTAAAATTATAGTG
CAGTCGAATACTAATTTTAAAGGTTGAACAACTTTTTTGTCAAAGGTTGAAATGATCTCTCAGG

AATAGAAAACATAGTTTCAGCATAATAACAATTTATCTAAACATTTCTGGCCTATATATTCATTTA
CCATAGAACTTTGTAAAAGCACCAGTTTCTGATTAAATGCTTGTCTCAAATAGCCCCTTGGG
CAAAATGACTTGAGAAAATAAATACAGGCCTCTCAAATAAAGTTCCCTTATACATGCTGAGC
CTTGTTTTGACTCCACTATGAAGATAGCGATAACAGGCCAAAAACTCCCCGGCCAATAACC
CCCCGACATCGATGAACAAAGTTGCTTACATAGAAGCTGGATTGATGAAAGGAGTTTTTATT
AACTCGAAGGTAGATTGATGAAAGAATTCTTCGTTTACGAGAACCTGGACTGATGAAAAAAG
TCTTGTATAGGTAGCGAAAACGTCATGCACACTGTAAAATAACGACATTAACCAGAACCCTG
ACCCTCACATTTGATAAAATACGTTCTCTTTTTGAATGGCGTTTGAAATTGATGAAGGTAATGA
ATTGCCTAAATAGAAGTTGGATTCGTGACAGTTATCACCACCTACCGAATGAATAATTGATCA
ACTTTCGGGGGTTATTGGCCGGTGGGTTATTGGCCAGGGGGTTATGGGCCTAGAACCGA
AGATGATAATGCCAGAGGTCCAGAGCTAGAAGAGTCATACAGCCATAGATGAGCAGTACA
GTTGGAACTTGGCAGTATTCATGGTGAAGAGAAACACAATTTAAGCAATAATTTATCGGCTGT
ACACTGTACAATAATTGTTGGAAATAAAACTATGCTGAATCTTGGTCCTCGGGTACTGCTTTC
AGCAAAAGGGATAATTATGCGATTTGTACAACTAAACAACAGGAATTTGTATATTATTGGTTAC
TGCATTTTCATAAATTTGATCCCGATGCAGTAGCTGTTCTGTAATGCTGAGCTACTTTTGTGCA
GCCTTGGTTGGAAACAGATAAATTTATTTTACAATGGCTATCTCTTCTTCTAAATCCAGTGAAA
CCTTTCTGACATGTTCTGTTACTGTAATTTCCAATTTATGTCACCTTATGCACAAGACATAAAC
CACTGGCACTTGAAGCCAAATCATAATCAGAATTAGTTTGTGGCCAGTGTGCAGTTTATCTC
TCTCTCTCTCTCTCTCTCTCTCTCTCTCTCTCTCTCTCTCTCTCTCTCTCTCTCTCTCTCTCT
CTCTCTCTCTCCATCATTTTTCCTGCAGCACATCTTGCATGCTTCTTAAACACTTTCCCATTA
CTCCATTCTGTACTGTGTCACCAGAAGAGATTTGTCGTGAAGTATGATCGCTGATCAGTCCA
GTGAATTTGTGTCTTTATGAACTACTTAGTCCTTTAGTTTCAGAAAAAGTTTCAGCTGTGGCTG
AACTACAGCAAAACTTCATTATATCGAACCTAAATGAACCGGCATTAACGGCTCGATATATC
CGAGGTTCGAAATAAGCCATATACATTTATTTGCATTGATTGTAGCCGAGCCATTTAGCGTTA
TTAGATAAAACCGGAAGTTCGAAATACCGAAGTTCGATATAATGAAGTTTTGCTGTATAGAGA
TCTTTTACACAATAATAGAAATATTTTTACATTGTATTTACTACAATGTCCTCACAATGTACATTT
CAGATGCAGGCTGCAAGTGCGGGGCAGCTTGCAAATGCTCGGCGGGTTCGTGCGCCG
CAGGATGCAAGAAGGGATGCTGTGGTGACTAGTGTACGTGTAAGACTAAACTTTTCCTTCTT
TTGTTAGTGTTACGGTATTGAAGAAAGAACTTGTGTTGCATGTAAAGAATCACTACTTTGTCTTT
TGTGTATTTGTGTGAAGGCTGTCTTAAAGATTGCTTCTGACTTGTTAAAACAGAGCCAAAATA
ATTATTTGAAAAAAAAATTTTGTCCTAGTTTTACCCCAGGCGATATGAAGTGATGACCAATCGC
GTTAATAAATGGTGCTATAGAAGTGTTTATGTTTGATTTGATTAGTAGCTATGATTGTGATATGAT
TTGTGTCATTTATAAAGTTTTTCAAACTTAATTGTGTGGAAATTGATGTTATTCATATATTATGTCTA
TACTGAAATATTGATCCACTGTGTAACTGTTCTTTGATTTTTGTTTTAGTGAGAGCCAAGGCATT
GAAATACGGAACTCTGTCATCAGTTTAATACATAAATTCTTTTCTGTTACTTATTTGCGATAGAA
TTATTCTGAAGGACACTGCGTAACTTTTCTCAAGTAACTTCAGTTATTCAGTATTCCAATTTGAT
AAATTTGTATTTTGTTTTAAATTGTACATGTTCTATGACCGTTTGACTTTGCATAGAAAGATCAAA
AGTTATAAACTGCATATTCGAATGTGTCGTTTTCTTGATGTGATTTTCATAATGGTCATTGAAGC
TGATGTAGGTCACACTGTTGAACTGTGCATAACGTCAGGTAGTTTGGGTAGCACCTGATTAA
CATATATTTCTACATGCAGTGGCTGATCCAGGGGGCATGTCCGGCCATGCTCCCCTCCAT
CCAGATTGGCTATATGCTTTGGCCACCCTCCTACGAAGAAACAAACATGAGATATTGGGAA
ACATTAAATATGGCCCCGAACCGAATGTTGAGTCTCTGGATACGTCACACAATATTCCCCC
TAGCCGAATGTCTGGATCCGCAACTGTCTACATGTACATGTTACACATTTCTTTAAACAGCAT
TCCTTAGATGTGATCTTGTCTATCTATCTATCTTGTGGTTGAAGGATATTTGGCTGATGTACAGA
CAACCATTTCTCTACAGACTTTACGAGCACTGCTGCTGCTTTTGATACCATCAGTCCTTGCC
CGATACATATTTACTTTTTTTAATTAATGACTATCTGCCAAGGTATATAATTAAGATCATAACCAG
AATGAACGTATGGTTCCGCTAACAGGGTCGCCCTAATTACATCAGACATCACAATCAATCA

ATGCTATTACATGCACTATCTGTATCAATGCAGACCCGTTCATTTTCTGCGGTTGGTCCAAAA
ACCTGGAATGGACCTCCAGTAGATCTAAGGCACCTTCCAAACGGTGCCTGTTCTCAATTC
CACCACCTTCTAAAGACTGTTCTTTTCCGCTTGGCCTGGGTCGGGAGCGCCTCTGAGTAG
GTATCTTGAAGGGGCGCTATATAAATTTTGCTTGATTGATTGATCTGCATAACATAGTACACTG
ATAGACAATCACATAATTGTTGTTTTGTCCGTATTTCAATTCATGGGAAACGTGGATAAAAAAC
ACAAAAGAGCACTGCAGTATAAACCGGTTAACAAGCAACTGCAAAACAAAATGATACAAGA
TAAAATAGAAGTCAACTTGTACATTTACACAAGCGCATTTGAAATAAATAACCTTAGTTTGTATT
TAACATTGGTAAATTGTAGATGCAAATACATGTAGGCCTACACTAGTGAGTTCCAGAGGTAG
AGAACTGCAGAGCAATGGTCCACACATTCTTATAGAATGCTGTCCTGAGGTGGTGCAAACA
CTTTGTTGATGATATTTATTTGTTGATCTGATAAAATAGTCATGTACTTCTGAATTTTTACAAAAC
CAATGGCCAAGTAAGGCTCGCTAGAATAAGATTGGAGTGCAGGTTATAGGCTACATGTACA
TTCCAATTATAATGGTTGTGTGTTTAGAATGAATTTAATCTCTTAGTTAATTCTTAGAAGTCTTAGT
TGCCATGACAGACACTCGCGCTGTGAGACCTGCAATTCCAGGATTCTGATAGCTTACAAA
CCTCATTTATGGAATTCCTCAAAGTACAGTGAAATGATCAAATTTTAAAATGTATTTTTTGCTCA
ATAGGTAGCCGAAATGATGATTGGAATTTAGATGTATCTGTTTTTATTTAGAAGGTCGTTGAAAT
AAGGGCTTGTGAAAATGGGTAGATGAGTGCGATTACTAAACAAGTGATAATAGTCAAAGCG
CTACAACATTTTTAACCTTTGACGTTAACGTTATTGCGTACGTAAAGGCCTTGCGTAAAGGTC
GTGCTTGGCCTAGCCTATTTATTAATATTCATAGAGCGCGATTCTGATTTGCTGCTTATCTTGT
TGGTCGACGATGTGGGCGGGGTGCGTGTGTTTGTGAATATGAATGAAGGACTTTCAACGGT
TAACCAACCGTCGCATGTTTAAAAGCATAATACGAGAATGGCGCACAATGTTATGAAGTTGT
CGGTTGTAGCAGGCTCATTTGGTAACTAAAAAGTTTATTTGGCACTTTAGAAGAACTGTTATA
ATTTATGGGTTTATTTAATTTTTTTAAAGCAATTCTTATCAGTTAAAAGTTAACGTAGTAGTAGTTT
AAAATGTGTGATGACATGCAGGGTTGTTAATAGCGATAGCGCCGCGCTACCGCTACTGTAG
CGGCGCTACTTTTATAGTAGCGGTAGCGGTAGCGCCGCTACTTTTGGCTGAAAGTAGCGG
TGATAGTAGCGCCGCTACTTTTTTTAGCTCCTTTTTAGCGCTATTCGCTACCGCTACTTTTAA
AAAAGAAAACTAATTTTTTCCGCTGCTCATTTTTCTGAGAGCGTTTTCTCATCAATCCATCAA
CTTTTCACGTCGTTGATATGCTTTTCTTGACGTTTGCTCCATCGCCCTCCAATCTATACTCCA
ATCAGAGGGCCGCATTACATTTTGCAGAGCGGCATTTTCCCGGATCCCAGACCATTCATC
AAGTAGGCGAAGTTATGTGCTGAAATCCCTAAATCTGATTGGCCCACGGCCTAATAGATGTT
TTTATCGTAGAAAATAACAGGTTTCAGTAACCTTGGATTGATGAAATAATATGAAGGGCCTATA
GTACGTGTAAGTAGAAGCTCGATTGACAAAAATATCGTAGTGTAAGCTTAAGTTAAGTTGGAT
TGATGAGAAAATAATAGTAATTTTAACTCCAAGCGAATGGTCACATAACTGCGCCTACTTGAT
GTTCTGGTCTGGGATAGAAAACTCAGATTGAGTCCTGTTGCATAGGCTACTTTGTTGTAACTA
TTCCTCGGCAGAGTAGCGGTAGCGCTAGTAGCGGTAGCGCCGCTACTTTTAAATATTGGTA
GCGGTAGCGCCGATACTTTTAAATCTTAGTAGTGGTAGCGGTAGCGCCGCTACTTTTTCAA
AGTAGCGTTAACAACCCTGATGACATGACATTGACGCGAGTCGAGAGCGCCTCTGATTTC
CAAGTCATAGCAATTGACGCTATATTAGTACTTTATTAAAACGAAGTGACTACTGCACCGTCG
ACGGTGAAGGAATAGTTCCGTGCAAATTCGCAAAATACCGGCGCACAAAAGCTTTCAAAA
GCGTCGTGATGGTCGGCTAGGGGGTTTGGGGTCGCCACTCCCAGCATCGCTCTGATATAT
TCCAGCAGAACAGCATCGCACATAATTGTAGGCCTATACTCAAGCAAAATAGCATCGTCCC
GGTTCGCCCGTATATATTTCGGTAGAACAGCATCGCGAATATTATATTTCAGCCGAACAGCA
ACGCCCGTATATACTCCGGCAGAATGGCATCGTATGTTCGTATACGCACCCTGGGAGTGC
CGATCCCTGGGAGTGGCGGCCCGTCACCGGCTAGGGTCAGGGCAAGCGTCGTGAGGG
TCTTAGGGTTAGTATTAGGGTTAGTGTGGTCATCAGACAGAAATTCCACCTCCCGAATCACA
TCACCAATCTGTAGCCTTCCGCAATCACATGACGCTCTGGTCCAATAGCATTTCCGCTTGC
TGCAACCGTCGACGGTGCTGTAGTCTCGTCCTTATTAAAATGTGATTATGACTGTGATACTCC
ATCTTTACCTTGGCTTAGGACTGGCAGAGTACGGGCAGTGGTATGATCATCATTGTTACGC

GTACATTTTAGGGTTGCCAGGTGTCCGGTATTTAACCGGACTGTCCGGTATTTTGGATCTTT
GTCCGGTATAATAATGATAGTAATACCGGACAATGCATGTGCAAATAGCTCAATACTTTGTGC
CACAGATACTGACACTGCCAGTGTCCAGTATTTTGGAGAGCCACCTGGCAACCCTACACA
CGTACCGGTACTGTCTGTGTAACTGCGAAGCGTGGTGCTTCTGCGGCAGAATATTCTGCC
AGAAACGCAAGAAAAATTACTTCTGCGGCGGAATATATTTTTTGCTATGTCTCTAATGACTAA
ATTAACCCTAGACCTGACCTATTCTATTCACATATTTATATACGGCGCCACTCGCAGTAAAAA
CTAATCAGAGGCGCCCACCAGTGCGATAAGGCCCAGGAAAAAGAAAGAACTTAAGAAGG
CGAGGAGTACATGATGAGGAGTACATGATGCGTATATGTTATAGTGTAAGTAGAAGTCTGATC
AAAGAAAATAATGAGAGTGTAAGCAAAAGTTGGATTGTTTTCCACCACGGAATTGTCACGAA
ACTTACTCCTGCGCAGAAGATTTAGCCAACTGCGAATGACGTACGATTGCACTGCTACTTG
CACTCAACCAATTAAATGAAAATAATTATTGCATTCACTGTAGCTAATGCTTTCAGTGCTCGC
AAATCAACTGGAGCTCAGCACTTTACAAACTTAGAGATTGCGATCATGATTAAGCATTAAGG
ATAAAAGGTCTGCACTGTGATAATGTTTGTAAACTTGAGATTTCAAGTTATGCAGATGCAAATA
AGTCTCATGTTTTTCGTGATTAGGAAGGTTGTCACGGTGTGTTGCCAGCTCTAGCGTCAGAC
AGTTTTCTGCCACAGCGTCGGACACAGAAACGTGCAGTGAACGAAAAGTCAAGAAGGCA
AAGACACCAATCGGAAAGTTAGATGATCATTGGGAAGTTGCAGGGGAGGAAATGGCTCCT
GTGAAAAATCTCAACAAGCCGGGTGAAGCTAACGACCCATATGTTCCATTTCCAGATGCC
ACGAACCCAAAGACGGGTGAGATCGGAGGACCCCGTGGCCCAGAACCAACTCGCTATG
GGGACTGGGAGAGAAAAGGAAGAGTCATAGACTTCTGATGAAACTATTACGTCTTCTTTCG
CTTAACAATGACTTTTGAAAACTTGTGTGACCAGTTCAGAATCCACTATTTTAGAGACTGGAA
ACATTTGATCGTCCAAATTGAAAAAGTGGTTGAGAACAAATTAAACTGGAAGAAAACTACAA
CCCCACCAGCTTTGCTGAATAATTTATTAGTTGATATTAACGGTTTAGATAATCTTTAGCTGTG
ATTGTCTCCTAATTACATTAGCCCCAAAATGAACGTCCACAATGAGGTGGAAGGAGTGTCC
TCAAATGTGGACAAGGAGAGGTAGATTATGACAAGAGTTCTGTTAAATTTGGTACATACAGTA
TAACCTGTGGCCATAGGCGTAAATTTGTGCCTTATAAAGCAAGAACTACTAACTTGGTCAGA
TAGTATTAATGTAGTCTTTTCTGTAGGTCTATTACTTTGATTTGTTATGTGCTCTGTCCTGTTGTAG
TCTCAGACAGCTCCATGATCATTTCCGGATAATATATTAACAATAATAACATGCTTTCTGATTA
AGTTAAAATTCTGTATTTGAATACCTCACATTGCAATAGGGCTGCATATTGCCTTGCAGGCAT
GTTTGAATCTATGTGCAGTGTTATCTGGATCAACAGCAGTGGCGGTGCCAAGTCCGCTGTTT
ATGGCAGGCATAGACAGGTCCATAGTCGCATCAGCAATGGCAGTCAAAATATTTTTATATAT
AATTTTATTATTACCAATTTTGACTGCCAGGACACAGGTATTTCTGCCAGGGCATTTACATAG
GCCTACCAATCACTGAATGTCGAGTGTCAGTATTTTGCGACTTTATACGAAACTACGTCGGG
GTCCGAACTCTGACCTCTAGTCACTTCGTAATAAATTATCCGAAGACTCAGTTTCAAAAGAG
TCACTGTTCATTTAACGAAAGTATCAATTAATCCACACTGTGCATCAAGCTGCCTTTCCAAAA
GACGATTGTTGGTGCTTCTGTGCGCCAGATTCAAACTTTTCAGTTTTTGCAATTTGTAAATTTT
ACCTAACTTACTTACCTATTGTGCACTTTGTGTTTAGTATTTAATTCATGGTGGCTATTGACTGA
CTGACTAATGACTGGCCAATTACCAATCAATCTATTCTAATTGATATAATAATACAGCTGTGCA
CACAACAAGTTACAAGCAGCACAAGTAGCCTATCTTTTAGAAATGCCAATTAAAGGGTTGTA
AACTTGTGTCTCAGAAGACGTATAGCGTGTGTTAAGACTGCTACAAATGAACCTGGGATGCT
TACTGGCCCAATTTCCCAGGCTTCCTCTTGAAAGCTGTGACTGTCACTGGACAGTGTCCAG
GAGTGTGTCCACTGAGACAGTACTATCAAAGATCTTTGGTACCATCATGCAACCAATGAAC
CCATTCCACAAGTCCAATTCTTGCACTGAAGAATTTTGTCCTCAATCTGTTTTACCTGTTTCC
CTGAGAAGCCTTGTTAAGGGGCCATCCATTTATGACGTCCACACGGAGGGGGAGGGGTC
AGGCTCAGGTGGACGGGGGGGAGAGGTCAAGCCCCATGTGGACGTCCACATAGAAAAT
TAAAATTAAAGATCACTGACGTCATACTGTCTTCTTCTCATGCAAAGAAGTTGGCGTCTTTTTA
AAATTAACTTTATCCTTTATATTAACTTGCCCTGCTTGCTTGATTGATTGATTGAATTCTTGTCGT
CAAAGACATGCGTATAGGCTAAATTAGCCAATTAATCCGCTAGAGGCGCTGTCAACAGAG

GCATCTGGTGCAACCACACACACAGACATTGACCATCTGGCGAAGCCAGATGCAGCCAC
ACACACCGTGACCTTGGACATGACCTTGATCCAAGTTTGACCTTGACCCAGAATTCGACCT
TGACCCAGATTGATTTTGCAGATAAAGGATTTATAATAGAAACTTGTTATGGGCTTTTCTTGTTA
CCAGAATTTCATCTTTGGACGGAATAAAAAGTGGAAATTTTTCGGCGATATAAATTAGTAATAA
AAATTACTAAGAGTACAGTTTAAATAAATTACAATCATATAGTCGAATTTCAGCTTTAGGTCCG
GTAGGCCATTAGCCGCTTCAAGAAAGCGGTGTGTATTTCATGTCGACCGCGTGTGGACGT
CCACAAGGGGGGACGGGGTCCGGCCCATGTGGACAGGGGAGGGGGTCAAAAACCTG
ATTTTTTGTGGACGTCATAAATGAATGGCCCCTAAGTAACCTCACCCACTGTAGTGCTTCCT
TTCACGTAATAATCTTCTAGGCCTCCCTTAAGATTAAGTAAACTCTGCCCCTCCTCTCCTTCA
ATATTGTTAGTCCACACCTATTCAACCCTTTGTAACGTACCGTTTCCTTCGCTTGCTTGAGTG
CTTGTGTGAGTATAAGCGTGATGCCTGAGTGGAGTGAATACATCTGCTGGTGTCTATTGCATA
GCGAGCTGATTAGTAGACCCGTAAGCCGGCCGCCGGACAATTTCTCGGCCGGCTTAATG
CAAATTGCGGCTTAAATTCGGCTTAGCCTCAATTTTCTCGGCCGGCTGTCGGCTTAATGGT
TTTCCGGCTTAAAATCGGCTTATTTTCCGGCTTATTTGTATGTTTTTGCTTTTACACTTTCCTTA
CTCGTACTTAATCATACATGTACTTTTATTCAAAACTCACCTCTTGCCAGTTTCTCATATCTCTC
TTGCACCATTCCACATCTCTTTCACTCCAAACTGAAAACACTGCTCTTAGCAAATCCTATCC
TGATTCATTCTCTTCCCATACCTCCCTCCCCCCACTGAACTCCAAACACCATCCACCATAG
CCGTCTCACTGTGTGAATGCATGCAGCGGCGTCAATCTGGTTTGAAATCTGGTGGGTCGTA
GATCCGGGTCAATAAAATTTCAATTTTTGGGGGCAAATTTAAGTGAAGTTTCGATTTTTTTCAG
GCAATTTCACAAAAAAATTCGATTTTTCCAGGCAATTTTTTTTAAATTTTGATTTTTCCAGGCAA
ATTTGTGCAAAAATTTCAATTTTTTCAGGCAAATTTGTGACAAATTTCGGTTTTTCCAGGCAAA
TTTTTGAAAGATTTCGATTTTTCAAGGCAAATTTTAAAAAAATTTCAATTTTCTCAGGGAAATTTT
CGAAAAATTTTGATTTTTTCAGGCAATTTAAAAAAAAATCGATTTTCCAGGCAAAAATTGCTCA
TTTACAGCGACTTTTGAATTATTCTATTTCTCTTCAAAAGTCTCCACTTTTGAACATGCTTCCTG
TACATGATAAGATATAATTATATTTCACGACCCGATCACGACTTCCTGCGACCCCCCCCCC
CACAACTCCCCTGCCCAAAATCTGGGGGTGGCGAACTCGCGACCCCCCAACCCACCC
AGGATTGACGCCCCTGAATGCATGACTTTCTGGATCTTGAGCCGCTGTCTATCGATATGTTC
TGGTTAAGTGTCTGTGATTAAGCTGGTTCCCGCGACTTCGGTTTTTGTGGACGCTGTAGAAA
TCTAGAGTTTACGATTACGACTGTAGACTTCATTAATTAATTAATTTACTTGCATGAATACGCGT
TTATGACATTTCTGCCTTTGCGTTAAATGAACACTGATAAGTTGATCACATCATTATTCCTTACA
AAGAAAATAGTAGGCCTATGCTACAAAATTAAATAAGGAATTAAAAAGTGTTAGTCAATCTTG
CAAAAGATCCAATCAGCTCTAATTTCTCAGGTGATGAGTGCATATTATACCGTAAAGAACTGT
TTCACAGGTGTCAGTGTGTCACTATCACTCGGTCACAAGCTCGTGACCTTACTCGTCGCAA
ACTGACAGTATGTAGTCCAAGTTATCATGGAAACAACTGATCTTGTTAAACTTTGATAGGCAG
ATGTTTGATCGCCTGCTGTTAAAAATTAGACCGGTAGCTGAAAATAGACACTGATCCCGAC
GACATGCCAAGAAACAGTCCAGCAAGTTGCTGTAACATTGTGTAATTGACAGCATTATCTTC
CCAAAATAAAAGTGAATTGGCAGAATAGTTCGCTATTAAACTCGGTTCAAAGACTCGTTGAG
ACAAATAGGCCTATCCATCAGTCTCTATCCTATACACAACAGCGCCGACGAGGGATGGCG
AAGCAGGCCATGGCGATTAACTTGAGGATGCTGAAAACTTTAGTATTTGATTTTTAGTATTTAA
GCCGCAATTTTGTCGGCGGCTTAGCCGAGTATTTTTCTTGGCCAGCTCGCGGCTAAGCC
GCAATTTTCGCGGCCGGCTAGCCGGCTTAGCACAAATCGCGGCGGCCGGCCGGCTAA
GTCGGCGGCTTAGGGGACTACTGATTAGTATGTGTGCCGTTCGCTCGAGGAAGGAATTAA
GAGCAGGAAGATTGAGTGAGAGGGCAGTAAACTCCTGAAAGCGAGTCGCAGGAACCAG
CTTATTCACAGGCGCTTAACCAAAACAAAATCGATAGGCAGCGGTCAAGATCCAGAGTCC
AGTCAGGTAGGCAGACAGTCAGACGGTTTACCGGACCTGAACCTCCCGTCTGGTCAACC
AGGGCTATAATTACAGGGATGTTAACAAGGACCTGACCCCCAAGGACCAGGACAAGGAC
AAGGACCTGACCCCCAAGGACAAGGACCTGACCCCCAAGGACCAGGACAAGGACAAG

GACCTGACCCCCAAGGACAAGGATAAGGACAAGGACCTGACCCCCAAGGTCAAGGACT
TGAAATATGTCCTTAAGGACAAGGACGAGGACAAGGACCACAGCTAGACAAATAATACTAA
TTGCACATATTAACTTATCAAACGTTCAATGGTGAACGTGCCTAAGTTTCATCGCCTAGACAT
AATTTTCATGGGTCTAGGACATTTCCCCCGGACATTTCCCCCCGGAGAAAAATGTTAATAA
CTTTTCTTAAATAGAAACTGGAATGATGAAACAATATTCTTCAAGTAGAAGCTGGATTGATGAA
ACAATTCCTTGATAACTCGAAGCTGGAGTGATGAAAGAAATCTTTGCTGTGCAGCGAAAAC
GTTAAAAATTGACGAAATTAGCCCAAAAATCGATATAAACGACCCTTACATTTGTTGAAAAAC
GTTCTTTGTTGAAATGGAAGTGGGATTGACGGACATAGTTCGGTCTGTAAATAGAAGTTGGAT
TTATGACACTTAGTTCAGCTTATATAGAGCTTTTCATCCGGGGGGAAAAGTCCGGGGGAAAT
GTCCGGGGGGGAAATGTCCGGATACCAATTTTCATCAATATTTCTTACCCATATTTTTTATCTT
CTTATTTCTTCCTCATTTACACGTATGTTAACTTATTCAAAATTCAAATACATAATAAATTATTGCT
CTTTCATTGACGTGTGACCTACCTAAGTACGCGTGTCATTATATGATTATAAACTGTATTTTTCT
GAAAAAATCGGCGGAACAATGGAATCTACGCGTTACTCATGAAAAATGCAAATACTCTGTTA
GCAATTACTAATTTATTTTCAATAATGACTAGTGACTATTGACTAATGGTTAAAACTAAGTCACT
TAGTGACACTTACTTTGTGAAAGCACTTGTGACGTTGACTTTGTCCTGAATACGCTTGTTGCT
GCTCCTACCCGCTGCATGCCACCGCTGACATTTCACTGTAAATTCTTTATATCCGAACTTTA
TAACACGTGGTCCAGTGACTCCAGTCGGCTCTACTGTTACTGATGCACTATTACTGTCTTGT
GACGCTTGTACTTCATCCCTATCACTCTCATCTGAGTCACTTGACTCCATAATTAATAATTATTT
CAAATTTTCATTAGAAGAAAGCGGAAAGACCATAGATATGATATTTATAGACGCAAAGCAAG
ATGGCGGCAGAAAAGACATAAGTAAGTATAATTAAAACTAATTGCATGCGTCGATAGTACAAT
ATAATATATTATGCGAATAAATCGGAATAATGGAATAATGGAAACAAGTCCTTGTAAGGACTAA
AGACAAGGACAAGGACTAGACCACAAGGACCAGGATAAGGACAAGGACCTGACCCCCA
AGGACCAGGACAAGGACAAGGACCTCCTGGACCCGAACCCCAAGGACAAGAATAAGG
ACAAGGACCTGACCCCCAAGGACCAGGACAAGGACAAGAAAAAAGTCCTTAAGGACAA
GGATTAACATCCCTGTATAATTATAGGCATTATAGGCCTATAGCAGTGTGCAGTTGATTGCAA
TTTGTGCAGTTGTAAAATCGAAATGTGCGGTTGAAAAAGGTAATGTGTGCACTACAGGGGTG
GTAAAAGCCTAAGTTTTTAAAAAGTTTTCAGGTTTTTAGGGTTTTAGATAAATAGGCGACACTT
TTTCTTGATTACTGGCTTTTTACCGCTGATTTAGCATCAGTTTGGTGATTTTTTCAAAGTTCACG
ATTCGATAAAAATTCCCGGGAATTCCCGACGGGAATTCAGGGTAGCACGATTCCCGGGAA
TTCCCGAATGGGAAAACCCGGTGACCGTGGTTGGTTCTTGCGATTTAACTTTTGTGGACGC
AGGCAAATTCGAAATTGCTATGACGAGCCGTGAAATAACAAAATATGATGGTGCTTTTGATG
ATAGGCCTAATGTTTTATTTCAAATTGGAGAACAACAGTATTATGGCAGTGGACAGTGTCAGT
AAGCCTAAAACTAGGACCAGGAATATATGGATTATTACCCTGATGGCCTTACTTATTTATCTTA
TCTCGCGCAAGTGCTATGTTTACTTGTCCTTTTAAAGTGACGGCGCCTCATGAATTAAATGG
AATGCGTTGAGAGTCGCCATTTTGTCCAAGCTTCTGTTTTTGTTGCGTGAAATTATAACCAAA
CTCAGTTTAAGCGGTACTATATTGAACATATCGAGCATTAAGCGCGTGTTTTAGTTTAACTGA
ATTTTTAGTAGAATTTTCATCGTAAGTTAGAACATATGATATAAATCTGTGTATATAAAATGACAA
ACAACCACTCGCCCAATACAGCGGGTGTGACTAAACAGCGCCAGGCCATTAATACCGG
GAGGCCTTAACTTCATTAATCCCGCTTCCACCTACGCTGTAATTTTGCATCAATCCCGCTTC
AGCTCACACGGTTATTTTTCATCAATTCCGCTTCAACTTACACGGTGATTTGTCACCAATTCC
GCTTCCATTTACCTGATATTTTTCCTGCAATCTCGCTTGAAATTACACGGTGATATTTTCATCA
GTTCCACTTACACGGTGATTTTTCGGGCAGGGGTAAAACGCTGATCTGCTGATCTGTGGAC
CTGCTGATTTGGTGAGGGTAAAACGCTGATTCGAAACTGCTGATTGGTCATGCTGATTTGGG
GGCTACAGGGGGTAAAATGCTGATTAGAAAATGCTAATTTTAACAGCCGACAGGTTGGTTA
AAAATTGGGATTATGTAAAAAAACAGGTAAGCTTGAGAGGGAAACTGACCAGCCACCTGG
GTGCTTGAATTCACTGGACAATAGATAGGCCTATTCATATCAGCAGCACCAGCTGTGAGGC
ACTTTTCAAGCAAGTGCTGATAAAGATTGGCAAGCAACTCTCTATAGGCCACGCCTTTCTGT

TTCTCGGCCTTTAAAATGAAGTCTGTGGTCTGCCTTATATTTGCTAAGTGTATAATCTTCTGTTT
TCACTGGCTAACAAGCCTCATATTTACTAATTAAAGCAAATTATAAGACAATGAGGTATGTGG
TCTGCCTTGGTGACTGCCTCATATTTGCTAAGTGTATTATCTTCTGTGTTCACTAGCTAACAAG
CCTCATATATACTAATTAAAGCAAATTATAAGACAATGAAGTCTGGGATCTGCCTTGGTGACT
GCCTCATATGTGCTAAGTGTATTATCTTCTGTGTTCACTGGCTAACAAGCCTCATATTTACTAA
TTAAAGCAAATTCTAAGACAATGAATTGTGTGGTCTGCCTTGGTGACTGCCTCATATTTGCTA
AGTGTATTATCTTCTGTGTTCACTGGCTAACAAGCCTCATATTTACTAATTAAAACAAATTATAA
GACAATGAATTGTGTGGTCTGCTTTGGTGACTTTCTAATTTTCTAATCAGCATTTTACCCCCTG
GCTTGCTGATTTGGCTAGAGGGTAAAACGCTGACCAATCAGCAGTTTCAAATCAGCGTTTT
ACCCTCACCAATCAGCAGTTCAGCAGATCAGCGTTTTACTCCTGCCCTGATTTTTTATCAAT
AGCGCTTCCAATGACAATGCCACATTTTCATCGATATAGTTTAATAACATGAGTAGGCCTATC
ATAAACCACCTTGCCAGCTTGGGGTGGGTCCATAGAGTAGTTGGAACTATCTGGAACTTTT
GTGCTGGCTACCCGAGTTTGCTTCCCGTACGGAATGCTAACTCGGGGAAACGTATCTCTTA
AGGTTGACTAATCCTCTCACTCGCTAGGCGGGACCCAGTTGTCGAGTACCGTAGAGGAAG
TCATGCAGGATATGTTTGGTTGCATTACCTATTTCCTCATTTGTGAGGTGCTCTATCAAAATGA
GGATCATGTCGCATTTTTCAGAAAATGAGATTCGTTAACCATATTTTAGAGCTTGAAATCAGG
TTTCTAACAATGCAAAAATTATTAAGATATCTTCAGTAGAAGCTGAGATATGGGGCCGTCCAT
AAAGTACGTCATGCTATTTTTGGCCAATTTTGACCCCCCTCCCCCTGTCACACTTTGTCACA
CATCCCAGGACCCCCTCAAAAGTATGTCACACATCTTGAACTCCCCCGATTTTTAGTAGGC
CTAGTACAAAAAACCCGGACAAAAGCCCCCTGTACAAATTCTCTCTCAATTGTTCGCGGG
GTTTTTGTCCGGGGGGTTTTGTCTGGAAGGTTTTTTTCGGGGTGGTTTTTGTCCATTCCCCC
TTCTATCAGAATACATCTGTTACAACAGAAAGGTAAACATCACTTTAAATTTTATGCGTCATGT
ATGATAAAAAAAATTTATTAGCGTGATGTCACATTCTCTTGACCCCCCCCTCATCACCTCTGT
CACAAACTGTCACACCTTCTCGGACTCCCTTGAGCGTGACATACTTTATGGACGGCCCAA
TGCTGATTTGAAGTGATGTATAATGAGAATATTGAAATCGAGAAAATGGCATGCATTGCACGC
TGCAGAAAGTCCTTGACAATGTACAGGCATATTGAAGCTAGTGTCCTGAAATTTCTTTTTTCA
AAAGTAAGGTTATATTATTAGTAAATCTAAAAGTAGATATTTAGAGCTTTTCGTACATGTAAAGC
ATTCACTAGATGGCACTATTAGAAGGCAATATGTTTTGATTTCAATCCTTTTATTTGCATTTATAT
TTATTCAGGATCTGCAACAATAATACGATTTAAGTATATTAAAATGCAATATATTTTCATAAAAAT
GACTTCTTTCGACGAAGTAGTTATTCTGTTACACAAATATGACTCAAACTCAGAACACACCTT
TTTTACCCTGCAGGTCATTTTCGGCTGTTACTCAAAATTGTATCTTGCGACAAGCGCCTCAC
TTTGAAAGAGCACCTCACATTTTATGAAATTTTGTTGTCTGATATTAAACGGCTTTTGTTATTAG
ATGGACTACGATAGTTATGAAGTCTACGAGCGAAATTCCCCAGTGGACTGTGGGGAAGAA
GCAGAAATAGTGAGAGAAAGGTTTGTTTAAATTTTTATGATTTATTAACAATATAAGTTAGATGTT
GGTGCTTTTGTATCAAGAACAGGATTCTTAGTTATAAGGCAATTTTGTCACAGAAATAATGGA
GCTACGAGCTTTCAGATAGCATGAGTAGGGCTGGGGCATTTTGTGTTGCACAACAGAACA
GCCACAATGTAATGTAATGTACAAATCTTACTTCAAGATCTGTGGTGAGTGCATGGTGGTCTA
GTGGCTAGCGTTCTGGACTGCCAATTGAGAGTTTTGTGATTAAAATCTCAGCCAGGGTAGA
AATTTGTTTCGAGGTTTTTGCACCACCTGCACCCCTGTTCTACGCGGCTATGACGAGTGCA
CTGACCATAAGCTGTCAGCGGGGAGATGAAAGGGCGAGAGAGAGGGCTAGCCACCCTC
CGTCATGTCATGCAGAGGCTAAGAAAATGAAGTCGCTAGCACATCATGGCTGCCATAATGG
GCTGCTCTTCTCTTCTTCTTAGACCTTTCTTGCAAAATAACAAGGCCTTTCTTAAGATGACGA
TAGGGTCGAATAGGTTGAAATAGGCTATTAAGCGTATTAACGCGCACATTGACTATATATATT
CATGCTCATCTTTCTCTGTGAATCTTGAATGCGTTGTTGACAGTTGGGATACCTCTGGTGTTAT
ATCAGAAATTAAACAGATTTTGATCTGATTCTTGTGATAATGTTCATTATGAATTTAGGCTTTGTG
CAAATCTGTAAAAACAAGTTTATTCTCATTCAGATTTTTGTAATTTCACTGCCTGTAGTCGGTG
CAATTCAATCGCGGTAGTAACGTTTTTCGGGTTATTCTGGTTTCAGAAGCCGTAGAATAGAC

CGAGCACGCCGAGAGAGAGAGGATGAGCTGCAGTGGGAACAAGATCGAAGTAGAGATC
GAAATTACGGGTCGAGGTCGTTCGAGGACGAGTACTTCTCCGAAGGTCGTGGATACAGG
GAAAACAATGCTAGCGGGAAATACGAGGATGCTGGTTTTGGTGGAGGAGATGACTTCTTG
GATTGCAGTGCTGCAGGGAAGACGTTTGACTATAATCATGGCATGAGTGGCACTTCACAGA
TCAAGGTAGGTTGCTGTGCAGGGTGCCTAGAGCTATGTCTCAGCAGACCGACTTCTGATAA
CAGCTGTTGCTGCATATTTCTCATAGGCGTAGACTTTGGGGGCAGCGCGGGTGCGCTTCC
CAAAAATCGGGAAACGCCCATGCGTTTATCAGTTTTTACCACATTTCCCGCAAAAATTTGG
GTTTTCCCCAATTTTTTTGCAAGTTTATGCCAGTACTTCATTGTATATGTTTACTCAGTGCATTTA
CACATTAACAACTAGCTCTGCAGCGTTAGTGAGTTAGAAGTAATTACAAACAAGTGCCCTAC
ATTGTATGTTGTAAGCTGTTATGTAAATAAACAACATAATTAGACAAGAATTGAAAAAGAGAAG
TTATTGAATATCAGTGGAAGCAGGGGAGGAGGGGTTAAGCAAGACCTTTGTCCCTAAGGA
GCCCTTCTACATTGGCCCATCGCAATTGAATAGGTAAACGCATAGTGACCAGTGACTCGGT
GTTCCTCTGTGTGCCTCTTGTTTGGTATACATGTAGCTCGGACTTTTGCTGAATGTTCTGCTGT
ACTATGCTTTTATTTTTCAATTTAAAATTGTAATAAATTGTTTGATTGGGTGATTGACTGTTGTAGC
AAAAAGCTGAACTTGCGCGACGTGAGATGAGTCGCCGACTTGAGGACAACGTGAGCGCT
GGGGTCAGTCGAGATCCAAGGCCAAGCTTTGCACGTGACATTAAAGATGAACCTTCGAC
GTCAATATTTCTTGGCGGAATTCCCAGAACGTTGGACGAACGAGACGTGTGTAGTTTCACA
TTGATTTGAATGTGCTTTCAATGTAGAATCTTAATTCATCACGTTGCACGTGCAATTTCTACTTA
AAATTAAATGCAGCTGAAAGGAGTGCATCATAACCTTCTGATTTTCGTTTCGATAGTACATGT
CATAGAAAGAGTTGCATCGTTTTTAGGGCTGTTTTATATAGGGCTATTTTATATAGGCCCATTC
AAGGCTGCCTATTCAGAAGCACTCCCAGTCAAACAGTGCAGTCCAAAATACTCTGACTGG
GCTGCATCAATATAAACACGCCGGAGCGCCACATAGGACGAGTGAGGGAGGAGCTCATT
TAGCTAGCCCCATCCTAAGGGGGTATGTGGTTTAGTGATGAATAAACGGATATTCATTGATTG
ATTGTTATAAGCAGCCAGACCTAGTCTTCCGAGGCTCTGTACTTGTGTTTTTTACTGCCGGAT
TCTCTCAACGACTGTCTGCAGTCAGTGGAATCATTACCTCTGTTCATTAGACACACACACAA
GTGGACCCCATGAGCCTGTAGCTTGAAAAATAACTTCTTCTATGGCAGGGATTTGAGTGTGT
GATCTTCCAATTGCCATTCCAGTGCTCTAACTGCTGGACTCCTGCACCTCTGCCGATAATTA
CATTTGGATTAGACCTTCTTTTGGATCGTTTGTATACTGTGTAATAATTTGTATAAGATGCATAAC
CATTGATGTGATGTAGCGCCCTGCGTGGATGCCTTGCAGTTAAATTAAATTCGGCTCCTGTA
ATTATCTGCTATCATTATCTATCTATCTATCTTTCTATCTATCTATGAAATTTATATAGCGCCCCTT
CAAGGTGACTACTTTATATAGCGCCCCTTCAGGTACTACTCAGAAGCGCGCCCAGGCATA
CTATATCTGTAATCATTCTGCAGTATGTCAGACTGAATATCAAACGGAAATATTATTATATAACC
CTTTACAATGAGTTTGTATTGAAATATATGGGATACCAAAGCGTTTTGCTGCTAAAGGGTTAA
GCTTGAAAATTCGTGTGCCTTACCACTTTATATTTTTGGCGATTTGTATTGTAGTTTAATATAATA
ATTGTAGAAACAGTTTGAGGGTAATTTCTTTCTTTCAGCTTGAAAACGAATTGATTCGGTGTG
GACTTCGTGCTAGAGATATTCGCATCATACGATCTAAAGAAACAGGTAAATGTATTTACGAAT
TTGTTCTGTTTTTAAGCAAAACGGGGAAGCTGTAGATGTTAGGGTTCTGTGTGTGATTGGAAC
GTAATGTTTCTCGTCTTCGTATGTTTGAGAATCTCATGTTGTGTATGACCAAGTTTTCCAGACT
ACATTGTTGAGTGTTTTATTTGTTGTTTGAAACTCGCGTTTGTCATTATCTATGACAGAGGTGCA
CAACCTTTTTGGGCCAAGGGACGCAGTTTATTATTTTTAGTGCACTCAAGGGTCGAAGACA
AAATTATGAGCTGAAGTTTTGAGAGTTGAGTATAAAAAAACAGATTTAAAAAATTAAACTGGC
CTTCTTATTGCTTGTAGTTTGATATTCTTGCCATTTTGGATTGTAAAAAGTTGCAAAATTGTTAAC
AGTTCAATGAAGTTTAAATTCATTCGTAAAGTCTTCGTCATTCAGGAGAAGTTTCTTGTATTTTT
TAAATTGCTCCGAAGGCTGGATATAACATGTTCGTGGGCCGGATCTGGCCTGCGGGCCA
CGGGTTGTGCATCCCTGATCTATAAGAAGAGTTGTAAGTAAAGAAGTGAATACTGCTGAGAG
TACAACTCCTACCGTACCGTACTTTACTTTTGTCTGACTCTTTGCCAGAATGATGTTGTTTGAT
TGGATGCCTTTTGAGCAGGGTCTTTGTTGTGGATACACCGAGCCATTATGATGTGAATTCTG

ATATATCGATATCAGGAGACAATGATCCCAAATTGATTCAACTTCAGGAAAATAGAGCTTGTT<br>GTATAACAACTCCGTTATAATATGGCACTGGATATGGCTCTGAGGGATTGTATTGTATTTTATTT<br>AGCTGACCTAATTTTAAAATGGAATTGATTTATTTAATTTTTTAAGCAGAGTTCTGGAAGTCCTT<br>AAATTCATGCGTCAAATAAAACAAATTGGAAAATTTCTGACTTTGAAAAAGAAAAGTTATTGAA<br>TATCAATGGAAGCAGGGGAGGAGGGGTTAAGCAAGACCTTCCCTATTGGCCTATCACAAT<br>TGAATAGGTAATAGAATAGAGTCTGATTTTGAAGGTCTCATTTGACCTTGGCCTTTTTATGTATA<br>TCTGTCTGTAAGAGCATGACTTTATCTAATGTCGAACCTGATTCTATAAATTTATATTCTTTGCG<br>ATTTCTGAGAAGTGATGTAAGTTGACCATTTTCCAGTTTGATTTTGAGTGGCAGTGGTTATAAT<br>CTGTGACCTTCTGGATGCAAGGCACTGAACCTACCACATGCAAGTTGTAGACACGTAAAC<br>CATTCAAAAGGCGGTTAACTTCTTCACTTTGATGCCTTGCTTACATATTTATGCTGGAAATTCA<br>CAACATCACCCAGCAGTCTGAACTGTTTTGTCTGTACGAAGAAATTTTCCAATCAAAATGCC<br>AAGCTTGGCAGAAGACTCTAAGAACAGTGCTGTCCAACCCACGGCTCCGCAGGCCGCA<br>TGCAACCCGCCGGCTGATTTAAAGTAGCCTTTGCCAAACTGGCTGAATTCCTTGCAATATG<br>TTTAAAAATTAAGTAAAAGACAGACACATACCTTGTCTTGACATTTGGCAGTATCAACACAAA<br>TCTAGTACCGTACTCTAAAGTTATAAACTGCAAATACAGTTTATTAAGTTTAACAAATTTTCGTG<br>GTTGCTCTTGAGTTCACTGGTGCAGTCTGCGGCTTCATGTGGTTCGTGGGGGTCAAAAAGT<br>TGGACAGCACTGGTCTAGAAACATTTGAGAATTCTTCAAGTTGAACATTGTTAGGTCGTTCG<br>GTTAGCCATATTAATGTGTCTTTGACAGTTTCCTGAAAGTCTTTGTATAAATACTTGGACACATA<br>CATCCATGCAAACATACGATACTGACCGGAGTCTTGACAGAACTGAGGGATATGGCATCAT<br>TACAGTGCCTGTGGGATATGCGAGATGTGCTTTCTAAAGTGTCAGGATATGTTCATTGCAAAA<br>TTAGAAAATAGTTAAGAGAAGACTTATACAACATAAAGTTATTGGTATAACTTTCGGATTTGCG<br>TTTATAACAGCCATGGTCTTTTTGCAAGGCGTGGTAAGGAATTAATTGAAAGCGTGTAAATAT<br>CCGGTCCTCGACAATTAGGTCCTGGCGATTAGGTCACGCGACAACTGGGTACCGTTAATT<br>AGGTCCTGTGACATCTTGGTCCCGGTACAAAATAGGTAAGAGGCGCTTAGATTCCATACAA<br>ATAAAATAATGCGTGTTTAACCTAAAGAGAAGCTGGTTGAGTAAACTAATATCGATGCTTAAAT<br>AGAAGCTGGATTGATGAAAGAATTCTTTAGGGCCTATTAACTGTAGAAACTTGATATTCAGAG<br>GAAAATAATGTTATTTAAATGAAAGCTGGATTGCTGAAAGAATTTTTTATGAACTAGAAGCTGG<br>CTAAATGGATGAAAATGCCGTTACTTAAATGGAAGCTGGATTAATGAAAGAATTCTTTATTAAT<br>TAGAAGCCAAATAGTCCAGGGACTCGGTTGTCGCGGGATCTAATCGTCCAGTACCTAAAC<br>ACTTGGACCCAATTGTCCTGCTCCGGTAAATATCTAGGCCTATCTGTTTTATTGATTTTAAGG<br>ACCTAATCGAACTAAAGTAATAATTCAGGTCATGGCAGCAACCAGTTAGCTCCATCTTGGTT<br>ATGTAGAACAGGCCAGACATGATTTATTGTCTGTGAATCAGTTGTGAGGTCCCCCACCCGG<br>TTTCCAGGGGTTCCCCATTCTGCTGTGTTTTGGTATCTCTAACATACGCCACTGTTGAACTTC<br>TTGTCTGACAGGTGCCAACCGCGGCTTTGCATTTGTCGACTTTCATAGCGTGCCTGACGC<br>GCAGAGATGGATGGAGCTGAACCAGGTTTATGATCGTCTCCTCCTCCTCGTATAAGTTCTCT<br>GCCATTTCAAGTGTGCTGTGGGTATGATCGGATGGGCTATGCAAATCTACAGCAGTGTTTCA<br>ACCAGTCTGTTGGTTCAAAGTTTACTAGGCACTAATGTATTACTGCAAAATTTCAGTTTTTTTTG<br>GGTCAGGGAATGCCCAGTATCAATGGCAGTATCAACAGAAAGTTAATATTAATCTATTGTCTC<br>TAAACGAGTATTGCCCTTAGCTGATGCCTTTTTGGTGATCAGCTTTTGGAATAATCTCTTGATT<br>TTTGCATTCTCTTATTTACTTCAATTGTCCTTCTAATGCCTAAGCTT
>91F03
aagcttgTCATTAAAGTGCTGAAACAAAGTTGTACGAAGACGAAGACCAGAATAGGGATCAC<br>CTGGAAATCCCCTTGGACATTCGTTTGGGCTGAATAGACTGTTTTTACCACGATTTGGAGAG<br>TATTCGCCTCGGTAAATATTACACACTCACATACAAATAGATTAAAATTCTTTTACAGAAATATT<br>ATTGTTCATTACTCTTTGTCTGTTAAACTTTGCTCGTTTGATTTTAAATCACGAGAATAACAACTT<br>CAAAGTTCACAGCGGAAATCAAGTCCATTTCAATGATTCCAAAAGCGAGAAATGTATCAATT<br>TATTTAGATAATTTTTTATTACATGAGAGTGCGGAGACTAACGAAAGTCGGATCCAAGTCAGC

ATCCGGCAACTAGTACATCCAAACACTCGGAGGAAAAAGGCATACCCGGCTGACCCCTA
TTCCTGAGAAAGCCCATATGTTCGTAAGTAGATACGTGTATGAGTGCGTGTGTATTAGCTTGC
ATGTAAATAGATATGTGTGTGCATGCATGTGTGCCTGCACATAAAGACATATCGGCAACTATA
ATCAGTGCATCAACATCAACAAATAGAGACACACATAGACATATCATACCACTGAGTAGCTG
GAGGTACTAGATAAATTCCCTCGTTGTGCATCTGTGTTTATGTAGCCCCTTTTGAAAACCTCT
CGGATTACAAACACTTTCCAAAGACATCACAACAATTCAGAAGGCTAACGTATTCTTGTCAT
TCAAGTTGTCTACAAATATTCATTCCTGTTAGTGCCATTGCTTAGTGCTCTATAGAAAGACAGA
TACCATAATGATACCTCGTGCTATAACTAGACACATTTGTCTGGCTGTGTACTGCTGTAACTT
CTAGAACCCACAGACACTTATTTAGAGTAACTAAAAACATAAAGACGATGGTGATGGAGCAT
ATTTGTTGAGGCTTAAGCCAGACATATATACATATACACACACACATATATATTTCTATATCCAT
TATAGGATCTCCTCTATCGTCTGGAACTGCATCCTATGAAATGCGCCTGCCTATCTTCTGGA
GCTCTTTATCTTGACTTTGGCTTGCTCTGGTCGCCAATCTCTCCACTCGGCCTCCAGAGGT
GACTTCTTGGAGCCATTTGCTCATACTGCCACCAGACAGAAAGGTGCTTTCTCGATTGTGG
GCCCCTCTGTTTGGAATAGTCTCCCCTCTGACCTCTGCTCTTTTTAATTCAAAGCTTACTAAG
TCAGACTACATCTCTGAGGTGCTGAGCACCTGTTCCTCCTCGATATTCGCCTTACGACTTAT
CCGGAACCACGGTCTCAGGAGCAAATAAGCTTCACCTGGGGGCGCGAGCAACTACTATT
GCATCCATCCTGTATGCCACCCCAGCGTGGTGGGGGTTTGTGGGCGATGGGGACCGCC
AGCGCCTGTAACAGCTGGTGGCCAGATTGCGGCGTATGGGTTACCTGCCAGCGGATTTT
CCGAGCGTCGAGACTCTCGCTGAGGAGGCTGACAGAAACCTATTCAAGTCAATTTCACGT
CTTTCTCACGTCCTACGGCATCTACTTACCGACAAGCCCACATCTTCGCGATCCCTCCGT
GTTAAGGCGCATAACTTCCTGTTGCCTCCTAAAGACAACAGGAATTTTCTTTCTAGGGCACT
ATATAATGCCATCTGTCCTCCTATAGGTCATGCCTAGAAGGTGTTGAACTGTCGTCTAGGGA
CCTTCTGACAGGCTGCGGTAACATCTCATGTCCGTTTTTTTATATTTTGGCTTTTGTTTTTCTTG
CTATATTAATGTTTTTCTTTCTTTTTATCATCGACATTTATAAACCCCTCTGCGTCTATGTCAAAG
GTGTTCAATTAAACGAAGATTTGATGATGATGATGATGATGATGATGATGATGATGATGATGATA
ATGATTATGATGATGATGATGATGTTCTCAGCCGCAGAACCATTCTAGTTCTTTTTATAGACTC
CTCAAAATTCTCCTCTTTGACCAAGCCTCTAAACGTATCAAGATCTTCTCTAAACCATCAAGA
TCTTTTCTAAAGCACCAAAATCTGTTTTCAGAGCATCGAGGTCTAGTCCACAGGTCCATGAA
GGTCCCTCTAAATGAGAGACCTTACTTACCAGTGAGCTGCAAAAGGTCGACAGAGGTCGA
GATGAAACGGGAAAGACTTGAGATCCCTGAGTCGAACTCCAACCTCGATGTAGAGAAAAA
GCAACCAGAGGAGGACCGAAGGAAGACAAATACCACGCTCTACAACAGCATCATCAACA
TTTATGTAGCTGCTCAGCTACCTACATCACCAATCATGCATAATATCATAAAAATCAGAATTAT
CATTCTGAGTAACTGAGTTAAAACTGACAGGCAATCAGTGACACAATTGTATACCTCTATCAG
TTGACAGGTATGTTATGGTATGACACTCATTGATACAAACCTCGTTTAATTCCTCAGATGCGG
CATCTTGAGTTTAATGTAAAATACAAAATCCTACTAAAATATTTGTAGTGGTAGAAAAAATGCA
GGCCTATATTTCAACATATAGACAATCGGCAACATAAAGAAGACAAAGATTATGACAACAGC
CGTTTGGGACAGCTTTGACACAGGCGGAGAGGAAACAGAAGTAGTAACAAGCTTTACGTT
TCTGGGATCAGAAGTGGAAAAAGATGGAAGGTGTGAAATAAAACGAAGGGTGGCTATAGG
AAAAGCTACAATGATTGGACTAGAAAAGTTATGGCAGGACAAACACACGACATAAAAAAAG
GATAGTGAGGTCACTGATTTTCTCGACAGTTCTGTATTAGAGCTTGACCGATGTTACATTTAG
CCGATTGTTCCGATGGCCGATTGTTCCGATGTTCAGAAAAGCCGATGTTGAGGCCGATGTT
GAGTGTTGCCGATGTTGAGTGTTGCCGATTGTCTACTGAATAAGCCAAATGTGGCGATTTAA
TTATGAACACTACTATAGCAATATCAGTGTATCACACCAGCGTTGCATAAATACATGTGTCTTC
AATATGCAAGCATCGTCCATCAAGACTGAATGCATAATGTGTGTCAATTGATTTTGTTATTATGT
GAATTATAAATTTGTTATTATGTGAATTATAATTTTCAAATATGCAGTGTCTTTAATTTCTATTTAAA
ATGTTAAAACTGAAGCCTATTTTGTCAAGTTATTCAACTTAGTTCAACGTCAAGTCATAGAATA
GTAAAGTGAAGTCTTCATTAATGTTTCTTTTTATTAAATTATTTATTCAAATTTAAATTTATAACCG

GAAACTGGAGTCAGGTAGCAAGTAGCAAATTATTTAGTACAAACTCTCAAAATTTTTCATATA
CTTCGGCTAGAATTATTTCAATAATACAGCTCCTATTTAAGGAGAAAATAGGAAGGATGGACA
CTCGGAATAAGCGGCATTAAGAATTTCCCTCCGATTGATAATCACATCTCTGCTTTAAATCTA
CCAATCACAGCACACTGCATGATCGTTCGCCCCCACTGCTTAAGCGTCCGGCTATTGTTTT
ATGGCTGACGGTGCGCCAGCGTTGATTTAAATCTACCAATCACGGCACGGCATGATCGTT
CGCCCAGCTACTGGTCTATGGCCGCCGGGAAATCAGTCTCGGTTAGGGCAGATAGGCTC
TGTTTTACGTAAGATTTACCTTTAGGAATCCTGGGAATTTTCCCAAGTAAACCTGCATTTGCC
CTAACTAGGCGATAGTCAGAACTTTCGTTCACGCCAATCAACAACCCATGACACCGAATTC
GGGAAAGAACGTCATCGTGAAACATCGGCACGAAATGGTTACCGATTGTCATCGTTTGCC
GATGGTTCCGATGGTGCCGATTGTCCTAACATCGGCACCGATGGTGAGTGTTGCCGATGT
ATCGGTCAAGCTCTATTCTGTATGGCTGTTTGACGTGGACAATAACAAAGAAAATGGAAAAA
AGATCAATGCATGTGAGATGTGAATATGGAGGAAGATGCAAAGAATTTCATGGACGGAGAA
AAAGACTAATGAAAGTATAGTATGAAGATTGGAGTAGAAGACGATGAAACGCTACAGCAAA
CTGCGGTAAGAAGGAAGCTGGGTTTTCTGGGACATGTTATGTGATCATTCATTCATTCATTCA
TTCTGGAGACTTATATAGCTCCTCTTCAAGACACTACTACTCAGATGGATCAGATGGATTGGA
AAAAGGAATGATGCTGGCATACCGAGATGGAAGGGGGAGGAGAGGGCGACCAAGGAGA
AGATGGATGGATGAGATACATGAAGTAATTGGAATGAAGCTGGCAGAACTAAGAGACGTGA
CGAAAAAAAGGAAACAATGAAGAAGGCTTGCCATGATGGTCGCTAGAGTTCCAGGAACC
GAGAGCACAATGTGACAAGGTGAGAGAAAAACTATAGGTCATATAAAAGCACTAATCTTAGC
AGTAACTGAAAAATGGAAGGGCATGGTATTGTAAGATAAGCAACAACAAGTGTAAATAAATTA
GTCTTCAGAAGCCTCATTATCTGACAGCCAAGGGCCCTTAGGCTGTCAGATTGCACCTTTG
GCCACATTGTTTGACCGAGGTTGGGCTGGGACCGCTCCTGAGTAGACTTCCTTGAAGGG
GCACTTTATAAAACTGCAGTAATTAGAATTAGAATTGGATGTCAGATTTGGACCTTTGGCTGT
CAGATTGGACCTTTGGCTGTCAGATCGGACCCTTCGCTGTCAGATCGGACCCTTCGCTGT
CAGATTGGAACTTTGCATGTCAGATTGGACCTTAGGCTGCCAGATTGGACCTTTGGCTGTC
AGACTGGACCTTACTTAGGCTGCCAGATAACGTGACTGCTGCAGATAAATGAAAAGTCCAA
CCTTTCCGTACAAAGTTACTTGCTTGGGTAACGCATTGACAAAGCCAAACAACAAACTGGA
ATTCAAACCTCAGATACAGGGGTGAAAAGCAACTTATGTGCACTTCACAAGCTGCCCCTC
GACATTTAGCATCGTAAAGGCTAGTGCATAGAGCACCTCTGAGTATATATTTTTAAGGGATGC
AATAAAAATGTTGCACTACATTACATTACGTATATATACGTATATATATATACGTATAGACATATAC
ATATATATACATATATATGTATATGTATATATATATGTATGTATATATATATACATATATGTGTATATGTA
TATATACGTATATATACATTTATATACGTATATATACATATATATACATATATGTATGTATGTTTGTATG
TATTTATGCTGGGGAGCGCCGGGATGCAGCTAAATTCGTATGGGGCGAAAGAATTAAGGT
GTGGTATATAAATGTTCAATTACATTGCATTACTGTGACATGCATTATGATCTTTTCCATATATGC
AGAAATATAGAAAGGGTTTTCATAAAAAGATTAAAATAAATAATGAGAGCAATTCTCCCAGGA
TTTCATAAAAATATCAACTTAAAAAAACTAACTATAATTCTCCCAAGGTTCTCATAAAAACATTA
ACATAAACAAAAAGTTAAAAATTTCCAGGGTTCTCCTGAATAGATTAACATAAATAATATGAGA
GCAATTCTCCCAGGGTGCTCATGAAAAGATCAGCATGCTAATCCTCCCAGAGTTCTATTTCT
AATTTAAATGAACTTGGGTGACAGACCGGTGCATCCAAGGCACAGTCTAACATTCTACTCA
GAACAAAGACAGCAGGAAACAGCCTGAGGACAGCCATTGCATCATGAATGCCTGCCGGT
CCCCTTACCTGTCTGCCAGACATACTGGACCCAGATGTAGGTGCTTGCAGTGAACATCAG
CCACTGAACGGGAATGAACAGGTAGCAGGCAACGTCTGACGTGATCGTAATGCAGACAA
AGAATATGGAAAACGCCTGTCAATGAAAATCCATCAACATAATTAAATCAGAATTCCCTTAGC
ACCTGTCAGGCACATAAGGCAGGCTCAGATGGAAGACGACAGCAGTGCAGCGAAAACA
CAATAAGTGCCTACAGAAACCGTATTAGGTAAAATTCAGCACTGAAGGCTTAAGGCAGCAA
TGGGGAAACATCAACTACGGCAGGTCATACAAAAATCAATATATCACCAACACCCAAGTAT
CAGCAAAAACCAATAAAAATATCACCCAAAGCAAAGTATTAGCCGAATCAAACTGAATTACT

AAGAAAACTAAACAAATCAAAGCCAGGGGCGGAGACCTAGGGGAGACTGGGGGGACG
GTCCCCCCAAAATTTGAGGTGGGGGGACGGCCCATGCATCCGATCCCCCAATATTTTGA
AAAGTAGTGTTGTCGGATGCACGCGGAAGTATGAACAGAGTAAAAAAGTAATTTTTCTTGTG
AGGAAAGGGTCATATACGACATTTAACATAGTAAAGATGCGGAAAATCCGAGAAACCTGGT
CGATGACTAAAAAAAGTCATAAGAAATTTTTGCCGTGAAAATGGACATTTTTTTCGAAAAAAA
CGTCATTCAGAAATATTGGTCCTCAAAAAAATGTTCCGTCCCCCAAATTCGTCGCCAGGTC
TCCGCCACTGATCAAAGCACCAACAAAATCAAAATATCACCAAAAACAAAGCCAGTGTAG
CATAATCACTCAACCTCTAACTGAATCTACACTGAATCTACACTAAACTGACACCAGCACAA
AATTCACATCATAATTAACTTTTATTATCACAGCCAACTCCACAGTAAGAAAACCACACAGC
AGAAGGAGCCATGTGCTCAAAAGCCACAGAATCTGCTGCCAGATGCGATTTAATAATCAG
CTGTAAGAAACGTCAGATTCCGGAGCAAACAAGAGGCATAGTAACCACCAGACAACCTTC
ATCAATCCACACAGGAATTTCTGCTCAATGCATAGAGATGATCCATCAAATGCCAACTAAAT
CCAGTCTGCCACTCTCCACAGATCCAGGCGGGTTGAACGGAGCCACACTGCTAATGTGT
TTGCCGTCTGACTCTGCATCCAGGGTTTGAATCCAGGTACATCATCATTGTAGTCATCGTCA
TCATCATCATCAATACAAGTTTAAAAGTGAGCGGTCCCACAGACATGCAAACTCTGAAATGT
CATCAACCCGGTGACAGTGTTTCCGTTACAGTCCAAAAATAAGACGTTTACTCACATTCTGA
CCTAAAATTTTGGACGTATATTCATTGCTAACGACTATTACACCGACAATTTTGGACGTTTTG
GGCCTATTTTTCAGACGTAAAAACGTCCGTTTGGGCGTTAACGGAAACCCTGCCCGGTGA
TAATTCAGTCGAAGATATCCAGATCTATGTTTGACAATCGGAAAGAAGTAATGGGTTTGGAG
GCATTTCTTGTTGGAGGAAATCTCAGCAACAGGGAAACACAGTGGCTGTCAGTTCCATTTC
CTTAAGAAAGAGTTCTACGTGCAATGTTTTATATCCGATGACAGAATAAAATCTGTGTTGGAG
CTACACAACTAGAGTCGTTACAAATATCACAGCATGCATATAGAAATATAACAGAGGGGAGT
TATTGTTACCACTACCATTATTATAAGAAGATTTATATAGCACCGTCTTTTAGGATAAAATAACTA
AATACAAATCAGACAGATAACAATATTATAAAGGCATTAATTCTGGGGGATTGGGTGGTCGT
GACCCCAGATTTTGGGCTGGGGGTCGTGGGGGTTGCAGGGGGGTCGTGGACAGGTCTG
GGAAAATAGTATAGCCTATTTTGCACAGAAAGTATGTTGGAAGGTGGTTTTTTCACGAAAAAG
AGATGAATTAGCCAAGAATTCACTTTTTTTGAGAAACGATAGTTTTTTAGTTGACGACTAAAAA
AGGTAATTGGAACTCTTTGCCCGGAAAATTGGAAACCAAGATTGATGAGCACTGCCGGATA
AGAATAGGTTGGCCTGGTCAAAGTTGTAGCAAAACTGTTAAGTTGCAGGAAGAGGTTGAAG
GATATTGATCAAGGAAATTGAAGTGAACGTCTGTAAGTACTAATACCTATAACACACTCCTCA
ACCTCTGAAGACTACTTTGGTGCGAGTTGCATGGTCTCCATATGAAATGGCTGAGTACATCT
ATAGAGGTTATGCAACGCAAAGCATGGCTAGTATTTCAATAGTGTATCGACTATAACATGGTT
CTCAAACGTGTTTAAATGTGGACCCACCCATCTGAAACACAGCAACTTTGCGACCCAAGA
CAAAGTCATGTTTTAAATTTGCATTTCTTACAGCTACAGCTTAAAACATTTCTTATTTATTGTATG
CGTTGTGTCTCTGATTTCAGTTAGAAAATAATGCATCTATTTTGAACAAATCGCTAGTTAGCGC
CGGAAAAATAATCATATAACGGCCCCGTTGTATTTGTGGGCAGACTAACACTGGTCAATGG
TCATGACCGTAACAATACTCTTTCAAACACCATTCTCTCAACCCACGTGGGATAATTTCGGG
GCCCACTTGCGCGTCGCAACCCACAGTTCAAGAAGCCGTGGACTATAAGATGGCACTCG
AAGTGAAGTGAGAGGCCATGCACTGTTTGCTGATGCAAGCTATGCTGATTTCACATCATAAG
TCTGGGATTCGAACACAGACGCGCAGGTAAAAAAAATAACGTTTCAGCCACTCAACCAAT
ATGTGGCCTTGAATATCTGCAAGGATAGCGATGCACAGAATTCATGGACAAACTCAGTTCC
TCCATTCCAGGGTCAACAAAATAAGGACAGACATAAGCAGGGAAGTTAAAATGTAAGCCAA
TGCTCACCAGTATGAGCAAGCTTGCATGCATAGATCTTTTTACAAAATGTACAGGTACACTG
CAAACTTGTTGTATCATGCATTCTGACATAACATAGTAAATACGTATTTATGCATAAAAGCATAC
ATTTATATAGCACCTTAGAAACCACTGCTATTATCCCTTTTGTGCCTCAGTCAACATGTGGAG
ACTTTTACTAAAGGGCTTTGCTTGTCAGAAGTTTCTGTAACTGCAGGTAAACAAGTGGGTTG
CCTTTTTTACAACTATTGACAGGGGCAGATGATGAATTAAATCAATTAATTATTGAATAGACCA

AACCATGCTCTGTAGCAGGGTGCTTTAACTAAAATATCACATACATAGATGTGATTGTGGGGT
GTGGTGGCACATTGGTAGAGTCGACGCCGTTGGACCGGAGGGTCGTGGGTTCGAATCC
CGCTCTAGCCGCCACGTAGGGACCTTGGGCAAGTCCTTCACTTGCAGTTGCCTTTAGCG
CTTCGGCGTGTAAACTCTGACACAGTGTCAATTGCTGTGGTCAGGAGCGCTTCCGAAAGG
CTCATGCTGCGAGAAGCACTATAGAAATGGATAAATACAATACAATACAATACAATAGATACT
TCTCCAGTTACCAGTTGACTATACATTAGCTGACCGCAAGCCAAAGTTCCTTCCAAGACCA
TGCTTAAAGATGGTCTCAACACTCTGGGTACCAAGACCATGCTTAAAGATGGTCTCATGGTA
CCATTTGTCACAGACAGCAGACACCCTGACCATAAGAACAGTCCTCTATGAAAGTCTTCAG
GTAAATGCTTGCCCGAGATAACTACCCAAACCGAACCAATGCCCAAGACAGACCGCCTC
TCCCAACACCTTGAAAACTACTACCCTCTGCTCCACTGGCAACTTAAACATGGTAACCTAC
CTCCAATCGCTGTCTTACATAATTGCTGCCAGCCATATATGGCTAATACAGAGAGTTTGATG
GCAGTCAAAACCTTAAACAGGTAAAAGCAACACCTTAAACAGATAAAAGTCTCGAATGTTG
GCAAACAAAAAGCACCTATGAGGCTAAAATTCTATACAGTTTGATCTGTTTATACTAAAAAAG
GCCACAGCACTGAATGGTTATAGATATCACACTGACCCTGCTTTATTACCATACAACTCATAT
TTATAGCAGTATCACGACAATATCCGGTGTCTGCTATTTCCATTCAACATGACTCCTACACAG
TGTGAAAGGTGGTTATGGTAGCAATGTAACACCAGACAGCAAGTTCACTGATTGATTTGCAC
AGCTTCATCCCGAAGACAACGTGATATGCAATAAATCAAATAATGACATCGGTAAACATAAA
TAAAAGCCATCCAAATGCACAAAACCCACAGAACTCTGCAATTTCTATTTTAAAGGACGCA
CTGTCGACGGTCGCAGCTAGCTGAAATGCTATTGGACAAGAGCGTCATGTGATTGCGGAA
GGCAACCAATTGGTGATGTGATTCGGGAGGTGAAATTTCTGTCTAATTGTCTAATAATTACCC
TAACCTTAATACTAGCCCTAAGATCCTCACGACGCGTAGCCTGACCCTAACCGACCCTCA
CGGCGCTTTTGAAAGCTTTTGCGTGCCGGCATTTTGTGACTTTGTGCGAAACTATTCCTGC
ACCGTCGACAGAGCAGCAGTCACTTCGTATTTTGAAAGTAGACTTCAGGTGCATTGCATGG
ACACAGTCACCAATTGGTTAGAGAATTTGTCTTGTAACATGGAGAACACAGTTTTGGCCCAC
CCGCCGACCTACTGACCGATGTTTGGTCGTTAGACAGTACGCTTTAAAATACGCTTTTTAAA
GTAATAATAATAAACAGGTAAAACCAAGACCTTAAACAGATAAAAGTCTCTAATGTTGGCACA
CAAAAAGCACCTATGAGGCTAACATTCTAGACCTAGACAATTTGATCTGTTATACTAAAAAAG
GCCACAGTATTGAATGATAATAATAATAATAATAATAATAATAATAATAATAATAATAATAATAATA
AAAATAATAATAATAATAATAATAATAAAAATAATAATAATAATAATAATAATAATAGTAATGCATAT
GCATATTATACACACAACACTAAGAACTTTTCTGTACTACTTTGGGCAACCTCTACAAAGGC
TGAAGAGTTCAAACTTTAATAGCTCATCATTATCATACTTACACAATGTATCAGTCAATAGTTAA
TCTAAACGAAACCACGGTCACAATCATAATCTGTTGGAGAAGCAGCCTGGTGGAAATCTG
GAGGATCAGAAGAACCAGAGATAAAACTGGAGATGGAGAGCGATTGCTGCTATCCAACAA
AATCTCGAAATGCTGCTACCTTAAGACTTGCACAGTCATGAGCTATACAAAGTGTGAAAGGT
CTACGTAAACAATCCTTTGAATTTAGGTTGTTAAAGTCCGACGTTAGAGGCCGAAAACCGTC
TATCAATAAAGAACATTTTGTAAAAAGGTGAGTTTATATATTCAACCAAAAACTATATGTTCAGA
GGTGAGAAAAAGGGCAAACTGTCCAGCGAATGGTAGTCAGGTTAAAGGGTGACCGAACA
AAATGGTAGGTAGTGGGCAAAATGGTACGGACAAAACGGTATGGACAAAACGGTATGGAC
AAAATGGTATGCGGACAAAAATGGATGGACAAAACGGCACAATTTTATATTTTGTGTACATTT
TAATTCAGTTAAATTCAACATATATTTAGTAACCAAATGTCCCAAATAAATGATAAGCACATAGA
GGAAAACTAAAGGCATCAAAGTAGAAGCGGGATTGATGAAAAAGTCATATTGTCAATGGAA
GCGGGATTGATCGATGACTTTATCCGTAATATTCTGTCTGTACCATTTTGTCCATACCATTTTG
TCCAATATTTACCATTTTGTCCGTATACCATTTTGTCCGTACCATTTTGTCCTAGAACCAATTAA
AGCCTCAACAGCACACACATTATTGCTCATTGCCAGTGATCGCTGCTTCTTTATTAGATACTA
TTCACACTAGTTAAATAAACATGGAAGTAGCAGTGCTATGGTACTCACCAGACCCTGAAACT
TAAACGAGTCATACACACTCCGAAGCATGAGCCAGAACGGCCAAAGATACTCAAAACGA
AAATCCAGAATGAAGTCAGCCAGTAGCACAAGCGCCCAGACGAGGAAGAATTTCAGGTA

CAAGAAAATGCTGTCAACAAAACAGACGATGAAAACCATCAAAACGCAGATCAAGATACAT
ATGCATGCAGATACGTATTGTATGCAAGCTGTTCTTCTGGCATATGCCATTCATGCCCAGCT
AGTTGCAAGCATCGTTATTAGCCAGGTACAGAGCCAATCACTAAATGTATTATTTGTTCAGCA
TATCTTTAGGCGCTTACTTTAAATAGTTTAGGCAGCTAAATAATTAAAATGTAAGCACACAAAG
GCATGCTGAGCAAAGGAATGTATAACTTTAGAAATATCTAGAATTATAAAATTTGCCCATGCTT
ATCATTGTCAGCTTCTGTAATCGTAATTACACATTTTTGTATTCCAATAGAAACAAATCAAAAGT
GCCCAGGACACGATGTGAAACTACAATAAGTTAAAATAGCTGTTGAAAAAGAAAGGCTGAG
GAGGTCAAGCCTTACTGTATTTTAACTGCTATCAATTAAAATGGTTGAAGAAAGAGAAAGGTC
AATAAGATCAAACCTTACTGTAAAACTACAATCAATTAAAATGGTAGCTGAAAAAGAAAGGTC
AACAAGATTTACTGTAATCTACAACTGTCATACAAACATTTAAATCCCAGCCTAACATTGCAG
CTCATGTAACGTTCAAGTCTTTCACTCAAAACTGAAAGCACGCTTCTTCCAAAAATTCTATCC
GGATTCCTCTTTATCTCCCTCGCTGCTTCAAATCTAGACAACCACGGCTTTTTTGTAAAATAA
ATTTCCACGTACGCCAAATTCCCGATGACCCTTTATTTAGATATTTACCCAAAAACTTGCAAT
TTTCTACCTGAAATTTCCCATTTTCAAGCCGAATGATTGAACTTTGAACTGAACTTTATTGTTT
GTTTCCACAGCAACAGCACATAAAATTCAGAACTATTACAATTGGTGATTCTAACTAAATCTT
AACTGCATCGTTGCTGCCAAACAATGGGCAGTCCTGAAGGGACCTTAATTGGTCCCACTA
GGTAGGGCTCCCTAGTTACACACAGTATTTTAAGCGGCACACTGATATAACAGTTATAGTTA
AAAGACACGATAGTTTATGGCTAACAACGACAATGATTATAGTCATAATTCAAGCAGTTTTAG
CAGTTTGCTGCAACCGTTCAGTAGCATAAATGAGATCATATATAAACAGACATAGCAGCTCT
TTTGTAGATAATTATAAAACTGAGGAAAAAAAGCTAAACATTACTTAACTAGACCTAACTAAAA
AAAAGAAGAAAACTCGACCAAAAGAATCAAACAAAAGATTCCGATAGGGTAGACAGCCAA
GGACAGTAATTTACATTTATTGTGTGCACTTTATAAGCCATGAATTTAGTTTGGTCTTAAATAGA
TTGACCTTTTTTTAGTCATTCGCCACAAAACAATCTCTCTCTAGCCAATTTTTGTGCGGATTTT
ATGCATTCTTGTTTCTCTCGAAAAGTTGCCGGTATGCCATGATTAATTTATCATACTAGTACAAT
ACTGACATACAAACTATACTTTCGTCATTTTCATTTCTAGGTACGCCGTACCTGCGAGTACC
CACACAAAAAAGCCCTGCAGACACCATTCCCAGCTCAGCCGTGTGATTATCTGTCTGCCC
CACCACCTCTGGAATTTGACCTGAACCCTGACCTACTGATTTTGCTCTGACTGACAGGATG
CCAATAAGCCGGTTCCTCTGACTAAATGTTTCAATTCTTAAGATAATAATGCTTATGATGTAGT
AAATCACATTGGTGGTTGCCAAACTGCAGTTTTAATCTCAATTTATATAGCTCCTATTCAATAG
AAATGCCCCTTTGAGTGCTTTCTGCCTTTATAACAGAAAACAAAAACTTAACAGCAATTTTTC
TCGTTAACGGCTGCTTTGAGGACATTTACAGCTCATTTCTCAGCTAGCCGTAATAGCACAAA
AACTCCACGTTTTGGCTATAAGCGAACAGGGGCGTCCATCCCCCCTGAGACCATGATGC
ATTTTCCCCCCTGTTTCAGATTTCCCCCTATTTTCGAAAAATTTTCGGACTCTGAAGAAAATG
TTCACAATTTTACTTTTTCCAGAAAAATTTCTTGATTTTCATCCGCCGAAATTTCTAATGACCTT
TTTTTTAGTCATCGACCACAAAATTTCCCCCTATTTTCCCTGTTTCAATACATTTCCCCCCCC
TGTTTCACGAAAATTATTCTTTCCCCCCTACTTTGACAAATTTTCCCCCCTGTTTTAGACGAAT
TCACCTGCTTTTTACATGCTTTACGTGTATTTCATTTCCCCCCTAGTTTGACCATGATGCATTT
ATGCATCACCCAATGCACGTACTGGACGCCCCTGTAAGCGAACCCTGCCAATAAACTCT
GGCTTGAAAAATAGTTTTTTTCAAAAAGTGTTTGGAATATTAGAGTTAAATTAAGCCAATTAATT
AGACATTAGAACAACAGATGGCCATTTGAGAGAGGAGCGCCTAACAGGCTGCTAGTCGAA
TTTGTTTATTCCAACTTATTGTTAAATTCGATCTCTCCAGCCGCTAGGAATGTTCGGTTTTTGTT
GACCTGTCTCTGGATATACCCACCGGGAACAACACTATCTAACCTGTGCTACGTCTGTTGC
CTGTTTTTAACAAAAAAATCAACATTTTAAGCTACATGTTGTGGACTTGGCGAAAGATTTCGT
CTCGCTCGAGTGAACACTAAACACCAAACAGTTGTTTCATCTCGGCTGTGGGCGGTGTAC
TTTTACTCCCATCAAACTGCCTACTGTGGCCATTAAACAACTCGTATTGTCTATCAGTCAAGG
CCTCACTCTCGACATGAACACCCGAGTCGAGTTTGTCCCAACTGGCTGCTCAACTACGCT
ATTCGTTTGCCTTCAACGCACATATGGGAGAGAAAAGCCACCCATATACTCAAAGACACCT

CACAGACTGAGAGTAAAGCGGGGCGGAATCCGCAAGTAACGGTCGATCTTCGTCGTCAC
CTCGTGCGGGAACCACCCGCTGCCCGGATGAAATGGAAACGGATAGAAGAGAGAGAGG
GAGACAAATTGTCCCACCTTGAACACCTTCGCGGCCTAAATCAACAACCGAAAGCAGCG
CCACCCTCTGGATCAGCATTACGCCTAATCCATCCGTATGTCCTCCATCACCTTGTTCACG
TTTGTCCTTCCTCTACTGACTCAGTCAATAACAACAACGACAACCGACACTTCCTTTTGCTT
TGGCTTCGGGCAGTGGATGGTGGGCCGAAACAAGATAGCGTTGTAACCTGGTTGGAACAT
TATGTCAATCCATGAATAGGCCTAACTGTTGTCAAAACCTAATTCCTAGGGCAAGTATTTTGT
ATCATACCCCTGTTGCGGCTGTGCCAGCAGAGGTATATTTGTATATTCACCAATAAAACGAT
ATTGATTGATTGATTGATGTTGCTGACATCAGCACTCACCATTCATTGTGTACCAAGCACAAA
CCTGCAAGCGAGCATGCAAACTTTATAAGCACAACTGTGTGCACATTCTAGCTGAACTTTTT
CTGTTGGCTAATCCAAAAAATTTCTGGCAGCTTATCAGCTTGCATAAAATGTCACCTCAGCA
GCTAACATCGGCGGCTTAGCTTAGGGGTATGCACGCACTGCAAATGAACAGAATCAGTTT
GAAGTCCATAGAGAGAAGAGGTAAAGAAATTCCAACCAGTTCAAGTCACAGGTTGCTAGG
CAACCGATTTCAGAAGTTTACACGGAGTATGCAAGGAAGTAATATGTCGTTGATCTCTGCAT
GTGTGACTAGCGTATACAGTATGTAGTTCTGTTTGCATCGAGTGTCAAATATATAAGCAATTCC
CGAAATTGGAAAGCTAAAACTTAATAATTAATTATCCTCATTGCATTACAATGATATCGTAATC
GTAATCATAATCTCAAAACTTCTAAAGCGCTACTCAAAAGCTAAGCGCACCAGGGCACCA
GCTTATTCACGAGCGCTGCGACGAATCAAAGGGGGTTTCCAAAGCGGGGGGTCAGAAG
AAGCTCAAGTCCGATTTCCAGAGTACCAGGAAACAACACTTCTAGCGCAATAAACATTTAG
CACTTTAAATAATGGCACAATGCAGTCTAGGACATGCTGTTGCAGAGCACAAAATTACCTGA
TGCGAACAGTTGCATGCTGCTAGAGAGTGCCTAGAGCTTTAGCATAGCATCGCCATATTGA
AATCTAGTACTGGACAGCCGGCGTCACTGTGCTATGCTATCCGAGTGCTACAATTGCGCTG
CAGCTGAGCAGCTCCGCCATTGCAAATGCGGAACGGAAGTTCAACGAAATGTATTCAAAG
GGTTGCGAATGTAAAAATCAGGGCAAGACGGAAGATTACCTCGCGTAAATGCCGTCGGAT
ATCTTGCCTCGCTTCAACGGGCGCCTTGGAGGTGGCTTGCCACAGTCGGCGGCGGCAT
TCCGTCTCTTCATATTCCACAGAATTCCTGGGTAAAATATGGCGATGGTTGAAAAAGAGGG
GAAATTAACAAAACAACGCAGCAGAGATCGACTATAAAGCGGACGACGAGGCTAAGATGT
TGGCTTTCTTTGTGCTTCCTTCTTCCGTCTCTGTCTCTGTCCGTCTTTCTGCGTCCGTGTCGC
TGTATCACTGCTCTTCTGTCTCTGTTGCTGCTCTGTTTTCGTGCAGCACAGACTCGAAAACA
GTAGAGGGCGGAGCGTAGGTCGGGGCCGAGCGGCGGTTTAAGCTACATGTATGTCCTC
GCATCTGTGTCTACGCTTTCTGGAACCAGTTACAGCAGTGGTGCACAACGTCCGGCCCG
CGGGCCACATCCGGTCGCGACGAGTCCTGATGTGGCCCGCGACGTCCAACGAGAAAA
GCGACTATTTTCAGAGCAGACACTGCCGGGCCCCTGATGGATGTTGCTTTTCAGTTTTTGC
AGAAAACTGTTTATTCACATAGTGTGACGCACCGTAAGACTCCTTTCTTTCTACACAGTGAAA
TATGACAGCCGGTGTTATTGTTCCACAACTATAGGCTATAGAAACAAGATTATTATGCCCCTC
AACCCTTGGCCAGTGAGCTGGGTTTGCACCAATGTTCCCTCTAAGCTGTGCGCGTGTGCG
TGCGCGCACAAATCGTGAGATGAGCACACACAAGAAAACTTGGAGCGCACATAAATTTTC
CTCTTAAAATGCTTTTGCACATATTTCATATATGTCGTTTTAAATATAGGCTATATGAGTGCTCGT
AAAATGTTGCACACAAGAAATTTAGTGCGCACTCAGACTTTAAAAAAATTAGAGGGACCATT
GGTTTGCACCCCCCTGAAAAAATCCTGCGGGCGCCCATGAATAGGCGTATACAGTTTAATG
GAAAACTTTAGACATTTTCAAGAAATAATTGTAGGACGGGTATAAGGCAGGAAAAAGCGGT
CAACTGTAGAGATATGGGAAATTAAATGAAAATATAGAAACCCTGGACACTTTCAGCTAGGA
TAAAGTTACACCCAACTATGGAGGTGTATACGGACCTCCTGTTAGTCTGTTTCTGCTACTAAC
CTAGCCTACATGTATTTGTCCTACATCAACTTTGAAAACTTTAGGCCGATTTACCTATCCACTT
GATGAAAGACTGTCGTAACTTTCCGCAGCTTGGCATCGATCGTTCTTCTTTCTTTCGCATTCT
GGGCTTCTTGTGGATGCACCACTTGGGTATGAACGCGACATTATTTAATTCTATTTTATTCAAT
TAAACGACGAATTTTTATTGAACATTTAACTCGCCAAACTGTAAATAATAAAAGCCTTAAATAA

CGCTAGAATGCAAACGAAAGTTGAAACAGAACGTCGCGAAGGTCAACATCGACTACCGA
CTCCAGCCCTGCACTGTTGTGTGTAAATTATACCTAAGGTGACCATCCGTCCCGGATTAGC
CGGGACAGTCCCGATTTTCAACGGGGTGTCCCGGGAAAATTGAGACCTCTCCCGGGAC
GCTCAATTGTCCCGATTTTGACTGGCTGTCCCGGATTTGTCCCGCATTTGCGTTTGAACAC
ACTGCTTCGATTAAATATCTCAGAAAATAAAAGAGCTGTAATATGCCAAAAACAAATTTCTGT
CGAGGGCGACGGCCATTGTTGCCATAGGTCCTCGTCAAATTGGGAAACCTAAGAGTAAG
CGAGCGAGCAACCGTAAACCAATGCCGATCAATGTTTATTTTTAAGACTGTTGCGAATGAC
GTCATGCCACTGAATCATGTGACAACGCAAATAGCGGGTTTTTGTTTACGCCAGTGGTAAC
TTAAATTTAAACTAATAACAGTAGGCCTAATCTTAGTAGGCCTACTGTAGTACAGCGTTTCCC
AAACTGTGGGTCGCGACCCAAAAGTGGGTCGCGAAGTTCTGTCAAGTGGGTCGTCAAAG
TTTTTCTTTAAATATTTTTTATTTATTTGTAAAATGCATAATTTGTTACCTCTTACCACTGTATTCGG
AGTTCTCCATTGACCTGCAAAGCTCATCAAAATTTTGACGACACGTTGTCATGTCACCCCA
CCCTTCGTGAACTGCACTGACTTCCCATGTAAGCCAGCGTAAATGCCAAAATGTGCCTCT
CAATGTTCCGAATCCACACAAACTCATCTCCCTCCTATGTTTCCTAACTTGTTACATCTTGTTT
ATCTCTCCAATTCAGACGAACTCTACAATCTTCATCTCAAGCTGATTTTGTTGCCACGCGAT
CACTTAGAACATTTTGAAACCGTGCATTCGCATTAGCTGCACTCGGCTGAGCGAAACAAG
CTGCCTAACTTCATTCACAAATCTGCATCATGATCGCTTTGTAAGACTAACCTTCAAGACATA
CTTGCTCAAAATTTAATTTGACTTGACTTTCTTCTCTTACGCGCTTTGTGCAAGATACTTTTTAT
TTGGGTGCGCTATAAATGACAGCTTAATAAAAATTCTAAAAGTGCATGAGCAACAGCATTAAT
TTCCCAGATTTTACACAATGCTTCGTAAGACAGCATATTTCCATGACGATTTTCTAAGCAAATT
TTAGTACACGCCATTCAATTTTATTCAGCAGGTCCCGAGATTCCCACGATATTTACAAGTGG
GTCGCGACCCAGAGAAGTTTGGGAACCACTGCTGCAGTACATCGACTGGACTGTGGACA
TTACCTTGGAAATGCCACCGAAACGAAAAGGTACATTTAATCTTAAATGCAAAAGAAGAAAT
CCTCGTTGATTGGGAGGTTAGATGCAACAAATGTACGTCAACGCTCAGCTTAGCTCACGG
CGGTAAGTCGGATATAACCGTTCATTTGAAGCAAGACAAGCACAAACGTGCTGATTTGGCA
GCTGTTTCCACCACCTCAGCTACCCATTTTTTTCGTTCTGCCACAGTGGGTGACTCCGAAC
AAAAGTTGGCAGATGCAGAAGGCACATGGGCTTATCACACTGTGCAACATAATCAGTCATT
TCGTTCTACTGATTGTACTTCAAAACTAGTGAAAAGTGTTTCGAACCAAAGTTCAGTACTGCA
AGAACAAAAACTGATGCAATTTTTTGCAACGTCCTTGCACCATATGCCATGGAAGAACTGG
AGTGTGATCTAGAGTGCGCAAATTTCGTGTAAATTTTTACCGGCTCTTCCAACCACAACAGC
ATTAAGATGTTTCCTGTGCTTGTAAGGTACTTCAAGCCCACATCGGGTGTTCACGTTAAAATA
CTGGAAATGACAAATGATGTTATTGTTAGGCCTATATTACATAGGCTATATAACGTTTACTCTAA
ATTACTGGAATTATTAATGATCATTGGGGAATACAATCAGTACTCACAGTCATTTGTTGCAAAA
TGCTGTTGTATTTAAGCAATTTAGGCTTTTGCGGGATGTTATGTGCAGAGATGAACCACAAAA
TTTGTGTTAGAGATCTATCCCGGATTTGGGGCAAAGCATTATGGTCACCTTAATTATACCATG
ATTAGGTCAGAGTTCAATCAAACTCCCATGTGTGTGTGTGGTTGTGTGTACATGCACATTTCT
GCCACTGAATTCCGTTTCCAAAGCCTGATTAGTTTAGCTGCACATCGGATGAGACATTAATC
TTAGGGATGTCAGAACGAGAACCCGGTATAAGGGCGTACCAAACTATATAGGCTTCTATGG
CCAGACTGTTTCTAATCAACGGGCATAAACTCAGCACCGCTCATTGACCTAACCGACCTAT
GACCAGTTGGACATTGGGAACCTGAGCGCATGTATGAGGAAATGTTGTTGGAATGAAGGAA
GGCTCTGTACATATATATATATATATTTATTTATTTATTTGAAGGACAAACTTTTGGAATAAACTTT
AAAGCCAAATTAAACCGAGAAAGCCAATCAATAGTTTGACCGTAAAATTCAACAATAAAAAA
ATTACCGCTGCCTGCAGCTCGGGGCCCCGGGCCAGTGGCCTGTTTGGCCCGTGGTTAA
GACGGCACTGGTTTCGGAGCGGTATGACTTGTAATAATACTTTCTTATCGGAATAAACAACC
GCAGGCAGAGCTGTCCATCATCTGTTATAATATATTAAATATTAAGGATATGCGACACGTGGT
GTCCAGTAAGCGTTAGCTCCGCCCCGGACCGTATGATCCCATCAATTGGAAAGATGCGC
CCCGGGCGCATATTCCCGAATCCCACCGCTTTCAGCACTGCGTAAATTATTCATACACTTA

TTCTCACCGCCATTGGCTGACGCCTTGGAAATTACGTAGGCTTTTAACTCGGGGTGGGGA
GTTTCTGACGTCACTGGATCAAACTGAGATCGCTTCGTTCAGAGCAGTACTAGATTTGTACG
CCCTAAATTGTTTACGTAGGATAAACCAACTTCGCTAATAGAAGATAGTTTTAATTAGGTTATA
AATTAATTTTGGCGGATTCTCAAAGGGACTTTGTCATTCAATAGCTGGTCCAAAATCTACCAC
CAACTGATTGATGCACATGAGCAAGGTCATACCATGACCTTCAACTATGCAGTCGCTAACG
ATAAAAAACAAAGGGATTAATATATACAAACAGTTTTATTTGACCTGCAGACAACTTAAGATAC
TGTACAAATCAAGAACTTTTGCCAGCATTTTTTGTACTTTTGTACATTTTTTGTACTTTTTGTTTTT
TTTGGTACTTTTTTTAACTTTTGTAGCATCTGCACTAGATCCAACAAAAGCAAATTAAGCCCAT
TTCAGAACAAGCAGAAATCAGAACTTGGCACAGTTCTTCAGTTTTCTATACAATGTACAAAAT
GCTAAAACAGTTTAAACATTATGCAATCGTCATCTGCTGCTTCAGTAAAATGAGGAAAATTAA
ACAAAAATATACCAACTCGTGCAGTCTCAACGCAACCACAGCATTGTCTTGAACACATATC
GAAGGCCTCCGGTCTTTTTACATCAATCCAGTAATTTTACATCAATTCGTGACCTTCGTAAAC
ATAATTACCATCCCTACCAACAGAAAGGACTTGGGCTTACAGCAAGCCGTCACTATATTCA
ATTGCTCATGATGATAAACCCACAAATTTACAGTTAAATCATTCCAACAGACACAAGGGTCC
TCATCAACCACAATTTTTTTTTCTTGTCCTCCAGCTTCCCCTACCACTGATCAGAACTTCAT
CTCTGAACATGATGGTGGTTGGAATGACCGACTCGACTCTGTCCATTGCGCCTTTTTGAATC
AGTCCTTTCGTCCTGCAATGACGTTTTGCTCTTGTGCTTGTTCTTCTTCTTTGCGGCAGTCGA
TCTGTCAGGGGAGCTGCTGTAAGCACGAGAATTTGGCGGTGAAGCATCTTCCCTCACGTA
CTTTGTGCGCTTCTCGTACTTATTGTCGTTTTTCGTATCGTATTTCGGATGCCTGTCCTTTTTG
CCGTCGTGTGCGGGGTATTTAGAATATTTCGTTGACAGTTCATTGATAGTCGTTGTTTCTGATC
GACTGTTATTGTTGCTGCTAGACTTTCGTTTCTTCCAGTCGCGATTGCTGCTGCTGTTGCAAT
TGCTGCAGCTGTACTTTTCTTCATACTTCACTGGCTTCTGACGCGAGTAGGTGCTGCTGCTG
TACTTTTCTTCATACTTCACTGACTTCTGACGCGAGTAGGTGCTGCTGTCACGATTGCTGCT
GCTGTACTTTTCTTCATACTTCACTGACTTCTGACGCGAGTAGGTGCTGCTGTCGCGATTAC
TGCTGCTGTACTTTTCTTCATACTTCACTGGCTTCTGACGCGAGTAGGACAAGTAGTCATCTT
TCTTTGACGTATGACAACTGTGTTGCTGCTGCTGCCTGCCTCTTTTTGACCGTGGACTGCG
ACTTTGGCTCCGACTGCTGCTGTGGTACGACCTGTGACACGCAGACAAAGAATGTTATGTT
ATATGGTTATTTGTATATCGCCTCTCACACGAGGCTATTAAGGGGTACCCTCAGTGTGACAG
GCAGGTGAAAAGAAGTCTCCAGACTACAGTGATATCCCCTGAAGTGCAGGAGGAGCGTC
ATTTCAGAGTGCAGGACCCACTGCCAAAAAAGACCCGGTTCTGGGATAGATAAGTACGGG
ACCAAGGTACAAGAAGATCAAAGCAATCGGCAGCGTAGTGGACGAGAGGAGCGTAGTGG
ACGAGAGGAGCGAGCAGTCTAGACATGAGCTCACAGAGATATTTCAGTGCTAGACCGGTT
GGGACTTAGCAACTAGGAACAGGACCTTGTATTTAATATCGGCCCAGAATGGGAGCAAGA
GTAGATAATTAGTCGTGTAGATAGAGATGTGGGAATAAGGCACCAGAAGGAGCAATCATCTT
GGCTGCAGCATTTAAGACAGACTGCATGGGAGCAAGCCGAATCTTTGGGAGCCCAATCT
GCATACTTTTTGCTTGTTCATGGTGGCGAGTATAAACAATTTCACAAAATTCCTGAACTGCAA
ATAGCACAAACTCCACATTAAAAAGGATTTAATTTTTTTAGATATAAACTATTTCATTTATTTAAG
AATACTTTGAAAGTATAGAATGCGCCAGAGTATATGACTTGAAAAATCATTCACGACACTTTG
CATGACTGCCTAACATTTGTAGTGCAGTAAAGCACTCACTTTGTCAAACGAGACATAATTTTT
TAGTAAATCTAAACCTGAAGACGATTTTTGCTTTTGTTTCGTGTTCATTATTTTAGACTTCACCT
CAACGTGAACCTTACATTTTAAATTAGCCTGAAACAAGAGACTTCGACTTATATTGAAACTGA
AATGTACTCGAAGATATACGTTAATAATCAGTTAAAGCAAAATGGTAAATGTAGAATCATAACC
CTTTACCCACCGCAAAATTTTTTCAACCTACCCCGTCCCGACCGCACATTTTGGCTTAAAT
GAGTGAGAAATAAAAAATTTTGAACTTGCAATGTCTCTGTTAGTTTTTGACCTGTAGAAATAAC
CAAAATACCATTCTTTAGGATTTTATCTCCTCTTTCCATCAGTCCGTCCAGGTTTTTGTAAAAT
ATTTTTTGACATACCCAAAATGACCCCAAACCGTACACCCCCCAAATTTCTTACAACTTTTC
AGGTTTAAGACAGTCTACAACAAAATTTTCAGTGACAACATATGTCTTATGAACCCAAGAAAT

GTGTAGAAAATGTTTGCACACAGCATGAAAGAAACCGTTTTCAAATATTGTAAGAGCTGAAG
GAGATACGCATTTTTTATTTAGACCCACCAGCACCTCTTCGGCAAGGATGCAGGCCTATGT
CGGATGCCATCGTCATCGAAGTTCGTTTCCATGACCGACTCTTCATTCGCTTGAATAAGTTC
ATAACCTAACTCCTACACTCCGTCGGACAAATTTCATTCTTCAGAAATTTCAGAAGTTTCATA
GTATGATAATTTATCTAACATTCGCCGAATTTCGTCCACGCGGAGAGATCGCTCACCATGTT
GTCAAATGTAAATAGCCATTTATAGCCACGCATCAGGTCATTCAAATGCTCATTTTGATTCATT
GTTTTAATATTTAGAGGTATTTTCCGGAGGTTTCCAATACGTCATTTGGCTTGAAATTATACGTA
GGATCTAATGTTCATTAGTATTCATAAACCCTACGAGCTGTGAAACAACACGAGGCTACGAT
ATGACATGACGCGTCTACTACGGGTTACTACCGTGCGATTTTTGGACGTACTACGGGTTACT
ACCGTGCGGTCGGTAATGGGTTAAATAGCTTATAGTCTTAACAGCAAACAGGAGTTTAAGTA
TGTATAACATTAATCCTTTGGATATACACACAACAGATATCCAAAGCAAAAGAAGCAACTTCA
CACTTACTGAGACTTATGTTCGCCTGAGTCATCTGATCGCAATTTCTTGACAGCACTGCGAC
TTCGACGCCTAGAGCGACTTCTCTTGCGCCTTGTCCCCCGACTCTGGTCTTTACGACTAC
AAAGTATAAATATCATTGCAAATTTACCATCAAGACCACATGCAAATTTTATGCAAGAATAGAT
GACCAAAAGTTATGTAAATATCGACATCAGTTTCTAGGGCCGAAGACAAAATTATGAGGTGT
ACTTTCAAGAGTCAAGTATAAAAAATCTAATTTGTTTTATTTAACAAGACTTCTTATTGCTTGTG
GTTTGATATTATTGCCATCAGAGGTGTTTCAGATTGTAATAAGTTGCAAAATTTTTAATAGGTCA
ATCAAGTTTAAATTAATTCATAAAATTTCCGTCATTCAGGTGAAATTTCCTTTATTTTTTTAACTG
CTCCGAGGGCCGGATAAAACATGCTCGGATCCGGCCCGCGGGTTGTGCATCCCTGGAC
TATATCATTGGGACTACAATCCTCTGGTCAACAAACAGTGGCTCACTAGAAAAGGACTTATC
TAGAAAGAATCAGAAACCACCATTTAATTTTATTACAGTTTCCGAATAAAACAGCTGTGTTGA
GCTAAGACACCGAGCTAAATGCTCACTTCGTTATGGAGTCCTGCTTCTCATGGTCAGCGCT
CTTCTTTCCACAACTCAGAGATAGGCCAAACTCGTCTGGTGTACTGGGTGTCGATGTGCTG
TTTGGAGTTGCAGTATGAGCAGCAGCCGCCAGCTCCTTTGCCTTAAACTTTGCTTCGATAT
GTTGTTTCTTGATCTCGCCAACAATTCTTTCAAGTTCATTGACATTTGGCTGCCACAGAAAGA
AAATACAAAGTTATCTAATGCATATAATATCATAATTCACATAGAAAGCACTGCATTAAGAATTT
TGAATCATACTAAAGATGTCATAGCGTTGTCAAAAGCATACTAAAGATGTCATAGCGTTGTCC
ACATAAGCCTTAGCAGAAGCCCGCTTCATAAAGAGGAGGCATTATGTTATTATTATAGAAAAT
TTTCAAAGTGCAAGTAAATAAACTTTAAGTACACCACAAATGCGGTAAACATGACTAAGTCA
ATGTTTCTACAACGGCATTATAGCAGATTCCCTGCCACAAGAAGTGGCTCTGGTGTACGGA
CTGAAAACCAACAACTGGAAAGGCAAAAATCCAAATGTAAAGCTTCCAAGTTTTGTTTGAA
CCCCGGGGGAAAAGCGGAAGAAATCCATACATTGACAAAAGTAACTGACCAAGGAAACA
ATGGAATCGATGACATCGGATTCTAGTTAGCAGTCTTTCAGGAATGGTACATTAGCATATCAT
CAAACAAACAATGACAAATCTCGACTCTGCCGACAAGGACAGGCTGAAACCACGTCATTA
AGAAACATCTTGCGGGAACAGCATTAACAAGGCAATGCAATGAACAACAAACAGATGGTG
CTCACCTTGCTGCGCTTGTAGAGTTGCAAGATGGACAGACAGATCCTGCGAATGTCTTGCT
CCTTAACACCAAACAACAGGAACCAGTGCGGATTGTTCGGCAAAGGAATCTACAATTACA
ACATCACATGAACATTTACACTTGTTATTATTAAAAGCGCTTCGGTAAGGCTTGAGAAATGCT
ACATAAATGTTCTAATACAATTGCAAGGCTTCTAAGCAAAACATCCTTTGGACAAGGCCAGA
CGAAACTGGACACAGCAGACTAAACTGGAACACAACAAACCAGGAAAGGAAGTCACAGT
GAGCTATCTATAAATCAATCTATATATGGAATTTATATAGCGCAACTTCAAGGTTACATAGTAAA
ACATTCGTCATCACCTGAAGCTGTCTTGCGGCCAAATAAATGCAGGCACTGGCGATCGTC
TCTGGATTGAAACGGACGAAGACGTTCGTTCGGAAGCTGTCGTTCATGTAGTTCCTAAAGG
CAAAAGACAGATAAAACAATGCTCATCACAACTTGCAACAGAAGAAATCTTGTCAATAACAA
GGCACAAATGGCCATGGGCGGAGACTTGGTGCAGAGTTTGGGGGACGGGAAAAAAGTTT
TCGCGGATCAAATTTTCAAATTACCTTGTTTTTGGAAAAAAATTCTATTTTAACGCCGAAAATTT
CTGATGAATTTTTTCGTACTTTGCCATGCTTCCAATAACACTACTTCTCGAAATATTAGGGGA

CTGATGCATGGGCCGCCCCCACCTCAAATTTTTGGGAGACCATCCTCCCAGTCCCCCTC
TTAGTGTCCGCCCATGCAAATTTCCACAACATAGCTTCGTGCTTAAGTATGTTCCGATAAGC
ATTGCTTCATCATTTACTCCGAACTCTTAAAGAACTACTGCGCACGGTGACGTGTTTGATGAT
TAATGATTTTCAACGGTTACAGGCTGATAGGGGCACTTTTAATTTCAATCAATCAATCAAACT
ATTGGTTTAATGTGCAAACTTTCATCCGTTAGGATGACTCTGATGCACAGAAAAAACACATTA
GCCACCGAAATGCAGCCGCAAAAAGTTCAGTCTTAAGCAGTTGTCAAACATTCTTGCGAT
GGGCGACACGTAGCTCGAAAACAGCAACGGTCCCAACAAACTCCCTTGTGGCACGCCC
TCATGGCAGATCCAGGTGTCCGATCGGGAGTTACCGACTGCCACGTAGCATAGACGATC
GGTCAGGTAGAAACAGAGCCATGCTGACGCAACGCGTCTGATGCCGAAGCCCAAGTGA
AGTCTTGACAAGAGTGTGTCAATGTCCAGCATATCGAATGCCGCTGAGATGTCAAGAGACA
GCAACAGACTGCACGACTTGTTGTCCATGTTAGAGTTGAGATCATCCGTGACTTTGAGTGTA
GCAGTCTCCGTGGAGTGACCCGATCGATAAGCGGATTGATACGAACTGAAGTTGGATGAA
CCGTGCAGGTGTGGCCGCAGGCGAGTGAGGGCCAGCTTCTCGAATACCTTAGGGATGTA
CCGGGTACAATTTTTTACTGGGTACCGAGTACCGGGTATTAAGACGGTTTTTTACCGGGTC
CGGGTACCGAGTACCAAGTATTTAACAAATTAGAAAAAGTGGAAGAAAAGTAAGCCTAGTAT
TTGTCTTGTTTTTCTTATTTAGGCAGTTATCTATGCAACATGAAAGAGTTCGAGAAACAGATTTT
AATGAATGACATGATGAACAAGAAACAGTAAAATACTGAACACAGAACACAAGTTTGTAACT
TTGTATGTTTTTAATTCGGCACAAAATGTTTTTATCTACTATTCCGTACGATTTTTTACTTACGGT
AATAAAAAGTAAAATCATCATGCCATAAATGACATCATTTGGCGTTCACTGCAGAGTGCAGA
TGTTCCAAGCATGAAGGAACCATCTGGTCTTCTGCAAACTGATGGCAAGCGGCCCGGTG
GTGCCACACTCATTCCCTGGTCTGCTGGAAGATACATGGCCTGGGATGCCACAGTGGTC
CACACTTGTGCTGCATCATACTTGTCGCAGACAGCAATCTCGGCAGGTTCTGCAGCTGAG
CAGGCTGCTGTTCACAAGACTGCCAAATATGCATTGTTGCCTGCAACCCATGTGTTTGTCC
CCATTGCCTTCGAAACTCTTTCGCCTCTAAATGCTGAGGGTGCAGAGTTTCTCTCAGAGTT
GGGACGTCGCATTTAATCTGTCTCCGGATATCAACGCGAAAGAAATTTCCTCCTGCAGCG
CCTTTCCATCTGTATGCAGCGCCACAATGCCATCGCATTCAGGGGTACATTTCAGGATGGT
CGTGAAGCCTGGGACGAGGCCTGATTTCCTAGAGAACATTTTTACTCCTAACATAATTTTTG
TCTTACTGAGAGATCATTAATACCTCGGGTAAAAAAAAGTAAAAAATTATTACTCGGCCCCAT
TATTTTACCGGGTCCGGTTAATACCGAATAGTGAGATATCCGGCACTTACCGGGTACCGGA
TGGATATCCGGTACATCCCTAATGCTCACGTGTTTGTCTTGCTATAACTTTTCTAATCCAATCA
TTGTAGCTTTTCCTATCGCCACCCTTCGTTTTATTTCCTTATCACACCTTTCCTCTTTTTCCAC
TTCTGATCCCATAAACATAAAGCTTGATGCTACTTCTATTTCCTCTCCGTCTATCTCAAAGCTG
TTCCAGTTGGCTGTTGTCTAGGGCTGTCAAAAGTTGCCAAAAGACCCACCCGTGTAACCG
CTCTAGGCAGCATCGATTAACCATTTACACGTAGGCCTACAGTCATGTAGAGCAGGCAAG
GTCCTGTCACCTTTGCCAGACACAATTTAAAGGCCCAGGGTATTACCTGTGTGAATGAAGT
CATTCCATATGTACACTATAGCCGCTACAGGAGGCCGTTTTAGGCCGACTCCGATAACTGT
CCTATTAGAGAGTGGAGAGGGCTTCTTTGGAGTCGTTCCCACACTTTGTAAAACAGCAGAT
CAAAAGCAGAAAGACCGGCGCAAGCAATATTCAGGCAGACGCAGACAGATGCAAGCAG
ACTCAGACAGTCAGCCTTGTCGACGTGACCCAGTCTCTGGACAATAATATTTCCCCTCCCA
AATTGATCTGGTCGGCTCAGCCAAGATGTTGGCCACAGCCTATAGACTAAAGTGCACCCC
ACTCTCCAGACCTACTCACTGCAATTGACATAGGACAATTGAAACTAAACAATGGCTGAAC
CAAACTATAACGAACAGAACACAAGTTCTAGTGCTTAGTGTGCGGCATTCCCGCCTTTGAA
ATTGAACTGGCGACTAACCGTACTTGATACTTATAATCCGTTTAGCTACCATCGGTCGGTTAA
CCGTGTAAACGGATAAGCGTTGACAGCCCTACTGTTGTCATAATCTTTGTCTTCTTATGTTGA
AATATAGGCCCGCCTTTTCACTTTCTCTCTTTACCCTTCTTATCAGTTTTGCCAGATCTGCTTT
TTTCCTGCCATCAATGTTGTGTCATCTGCATATCTCAAATTGTTCAGTGTCCTTCCTGTTATTTT
CACTCCTTCTTTTGCCTCTTCCATTTCTGCTTTTCTCATTATCCTCTCTGCGTACAGGTTGAAT

AAAAATGGGGATAAGCCGTACTCCTTTTTTGATATCTATCGGTGTCTCCAAATTCTGTTCTTAT
CACCCCTGATTGGTCTTCATACAATCCCTTAAGTAGTTGTATCAGATGTTTTGGTATTCCCATC
TCTAGTAAAGTCTTCCACAACAGTTCATGATTGATGCAATCAAATGCCTTGCTGTAATCAATG
AAGCATATGTGGACTTCTTTTCCGTATTTCAATTGTCTCTCCATTATCCATCTCGTGTTAGCAAT
GTGATCTCGTGTTCCTCATGCTTTCCTGAATCCTGCCTGATTGTCTGGAAGTTCTCTCTCAAT
TGTGCTCTCCATTCTCTTGTGTATTATTTTAAGCAGTATCTTGCTAGTGTGTGATATTAAATCTAT
TGTCCTGTAGTTATCGCAACTTCTTGCATCTCCTTTTTTAAATATTGGTATAAAGGTTGATCCCT
TCCAATATTCTGGCCATTTTCCAGTTATTGCATATTTTCTTTACTATCTTTATTGTCGCATCTTCC
CCAGCTTTTATCAGTTTTATGGGTATCCCGTCCTGTCCTGGAGCTTTGTTGTCTTTCAGTTGTT
TAATTGCCCATTCTACCTCTGCTTCCATGACCTCTGGTTCCATTTCGAAATCTGTCGTATCAG
TTTGTTCTTTCTCTATTCTTTTGTCCTCACTGTACAGTTCTTCCGAGTATTCTTTCCATCTGTTTT
TCATCCCCTCTTCATCTCCAATTACCTTCCCATCTCTTGATTTCAGGGAGCCCATTCTTGGT
GTGAATTTTCTCGTTATTTCTTTCACTTTTGAGAACATTTCTCTAGTTCTTCCCTTCTTTCCTTCC
TCTTCCATCACTTTACACATGTCATTCAGATGTTTTTCTTTGTCTGCTCGTGCCTGTTTCTGGA
ATGCTCTGTTCAATGATCTTACTTTATCTTTGTCTCCTACTCTTTTTGCATCTTTGCTTTCATCTG
CTAAACTGTTGGCTTCTTTTGAAAGCCATGGAGTTGTTTTGCCTTTTTTCTTCTTTGGGATGTG
TTTCTCTGCAGTCTCTAATATCGTTCTTTTTGTCTTTTCCCATAATTCATTTGTATATTTCAAGTTG
TATAGATCAATTATATCCGATAAGTGTTCATCAATTTCACAACTCGATTTACCTACACAGTTGA
AATCACCGATGTAAATCATGTTTCCTGGCATCATGTTGATTTCACTGAACAGACTTCCAAGCT
CGGTGAAAAACTTCTACTTGCAGCCCTAGATCCAGGTCTGTATATACCAACGATGTTTGTAA
CGCCAGTCCGAGATAAGATTTTAATTCCGAGCAGTTCAAGAGACTTCCAGCCTCAGTTTTC
TAACCTTCAGTTTCAAGCTGTAGACGATCGACACGCCTCCAGCAATGGCCATCTCGGAAG
AAGTGTTCTACACCGAAGCCGCCAGGGGCTATACTGTCCGTTATCGCGGGCGACATATC
GCTGTTGAACCAGGACTCTGTGATGACAAGGGCGTCCAGTACATGTTCCTCAATGATATCC
CAAATTAACGGGCCCTTGTTGACAGCCGACCATGCATTGAGTGTACCAACCCAGATTGAA
GACTTTTGTTTATATGGCGCTGGTCCTGGGTTTGGTTCGATGCCTGTGAGTAGCAGCAGTG
CGATCATAAATACAGACCGTCGCTGTGTACAGGTGGTTGATGCAAAGCTGCACTGTTTGAA
CTGTACTTTGCAGTAGGTTGGGCCAGGGTTGACTTCGACATCACCCGAGAGTAGCAGGAG
AAATGCAACTACTGCTGATGAAAGAGACCGGCAGCGACGGGGACGAAAAGGCCGAGCA
GTACGTGACCATAACAAAATCCATGTGATGCAACGTAGCGCTGGGTTGATGCGTCCAGTG
AACGCTTGCACAGGTCGATTTTCCGACAACTGAAGACAGCGAACGTTCATTGCAGGAAAA
GACTGCAGGGCAGATGAACTATCACATTGCTGGACTGATGAGTGTTGTTTGTCTAGAAATGT
TACTAAGATGGCGATGTCAACAAACAGAGATCCATTCTTACTTGCAGTGCAACGATCGCCT
AACGGCTGCTGCAGACGATAACGGTTTCTAACATTTAAATTTTCGTTCTCGGGTAAGTGCAA
ATCAGTTAAATTTACCGAGTGAGGGACCGATATCGAGATGGTCCATATTATGACAACGAAAT
AAAACAGATAAATCGAAAACATGATGGAAAAAACGAGTCGAAGATCCGTGAAAAACTTTTTG
GCCTCCATCTTGGCCACCTGGCGCAATTTATGCAACATTAAAAGACAGTTTGCTTGTCATTA
CCTGGCTCATGCAAAGTACTACTCGCACGACAGCAATGAAGTGGAAACCGCCCGTTGGC
TCCATCATTCGACTGACTTGACAAACACCGTTCCCTTAAATAGAAATTACATCCTATAAAGAA
CAGCTGGTGTAGATGCATATCACTCACAACCACTGTTTCAATTTCTTGTCTCTGGATATTTTTT
TATGACTCCGACCCACTCCCAATTCAGCGATTTCGAGGCAAAGAAAAATGATGGGCATATC
TTTACAGTCGAATCTACATCAACTATTAACACCATCACCAACAGCAACCAGTAAACTCTGTG
CAGGCCCGCACATGTTACATAACTGTAAGTAAACGAAAGAACGTGAAAGTCTTTACGTTTAA
TACAAAGTATGTTTAACGCAAAATCAAATGAATCATTTCGTATTTACATTTGCCTTCCGTCAGC
AACTGTGCGATGAAGCTCGACCGCTCGACCAAATGCAACTCATCCGTGATGTTTCAAGAC
AGTACATGATGTTCTGCAACAGTGTCTGACTTTATAGTTCCACAGAAACATGACAGTTACTTA
GCAGAAAGTTTCTTTTACAAAAATCTACCTAGATCATTCTGACCAAGAAAGTTCAAAGCTCG

CAGTTCGAGCAGGCATGATCTCTGCTGACTTAGCTAGCAACTGTCACTCTATTAGTCTGAAG
AAGTTTGTAACAATGTTCTGAATAAAATGTTCCATATGCATTAACATTTTGAACTGTGTGCATAC
TATTTAATAGTCAATTTCAGATGAGCTGACAACAATATCTTTCTCAGAACTCAGAGTACAATGT
GATGCATGTGCAAAACCAAAACCTTTCCTTTGCAAAGCTAGTATTTCTCATTTGCCAAAAAG
CTAAAATGACCATTATTAATCGATTTCAATCATATGGTTGGGTGAGTGAGTTATGTTACCAATC
AGCCTCTCAAATAGAAAACTGGTTTGATTTACAGATCCAGTTTAAACAGAGCACAGAAAGAT
TTTCAAGAAATTACTCCTATTACTACCCATCCACAAGCCAAATGATAACGGTGATGTAGTCAT
CTGCGACTTCGTCAATCCATTGTCTGTAATTGAATAGCGTCCAATATGAATTCACATCACCAC
ATCTCCTCGGCCAAGCCTTCCAAGAAGCCTGCCTCGGTGTTGTGTGCAATCTGCAACTTC
CACCCTTGTGCACGCCTTCGTTGCAAACAGGCTGGACTACTGTTGCTCGCTCTACGCTGG
CCTCCCTGCATGTCGACTGGGGTCCTTGGATCGGGTCCTGCGTTCGCTGCCCGCCTAAT
TGGTGGCATACCTAAATTTGGCCATGTCTCTCAATACATGCTTGATGTTCTTCACTGGCTCC
CTGCTGAGCAGCGGATTTCATATAGGATCGCTTCCTTGGTCTGGCGCTGCTTAGTTGGCCT
TGCTCCTGTCTACCTAGGTCCTACGTGAACTCTGTTGTCCTCCTCTTAGTGCTATGAGCTCG
CGATCACTCCACTCATCCCAACAAGGTCTCCTTCTTGTCCCTTTTGCCCATACCTCCACTA
AACAGATCCGTGCTTTCTCCGTGGTGGGCCCCTCAACCTGGAATGGCCTCCCTTCTGAA
CTTCGCATTTTTAACAGAACCACTTCACCTGCGTTTTTTTCTCACCTTAAGACTGCTTTGTTTG
ACCGTGCTGGTGTTGGGAGCGCTTCTGAGTAGTTTTCTTGAAGAGGCGCTATATAAATGTTC
AATATGAATGAATGAATGAAATGTCTTGCACATGTTGTTTTTATGAGAAGTCTATAAAGAACAC
ATACACCTGGCAGTCATGTCAGTATTTAACCAGTCCATAAAACAACCACTTTAAGTTAAATGT
TAGTAGATGTTTTCACCCCGAAATGCCACAAATCTTGATACAACAGAAGCACAAGAATCAG
CATACGACATCATGCGTTGTTACTAGATTAACGATGGAGTATGACAGTGAAAGGAGAGCGA
ACAACACGTTGAAAATCATCATCACACTACCCACAGCCACTTTCGCAACAGTAATTATTTAA
GTTTCACTTGTCAGAAACAAAAAAAATTAGTAAATTTAGATTACCTTTAACATACAGTCAGTTC
TTGTCAAATATTTCAAAATCTAAGCCTTTCCTTCAATTATAAAAATTTCAAAGGCACTGCAACA
TGAGAAAAATGACCAGAGTCTGATCTGCTCTGATCATTCTTCACGATCCACAGCAACCTATT
AAGACAAGAGAAGCCAACTTACCAGGCACACTGCAGTAATCTCTGGTCGCCCTCAGCTT
CGAGAACTTGGAGATACATCACAATGATCTGTAAAACAAGTTCACTCAAATATGAAATTCATG
AACAGATTCAACACACTAATTAAACTAGTAACATCCAACACCCTCTGCAGAATGTAGATGGG
TGTTCTTACAGACCTCCATGTTTTGGGTAATCAGTGAACAAAACCCAAGGCACAGGACATT
GGGACAGTGCATCACAGGACTTGCCATTTACTCCCCAGCTCACAAGAGTAGGAAATTACA
CTCTATGGTGACAGAGGCACACTTACAAACACACACAGCTTTCAGAAAGTCTACACAACA
CACAAGAACCAGCAACCTATGTGTAGAGTCCCCGCACACTATCTAAACACACCACAAATT
ACAAACTAATACCATATCATATTAAAAACCAAAACTGCGGAATTTATTAGCTATAGTAATCAAA
ATTTTTCAAAGCATTTAAACTTATATTCAAATGTGAATTTAATAAATATCACAATTTTCTATATATAT
GTTTGCTTGTCTATTGATTAATTTTCAGTCAAGTAGACTACTCGCATCTTCAGTGGAAACGCTT
GCCTGAAAAAAGAATCATGGAAACAGTCAAGACTGGTTAATTTCTCAGAATTCTAGGATAAA
CGAATAAAACAGTTTCGTACCTTATGAGGATGCTTCATGTAGACACAGAAACCGAGTTCCTT
CAGGACTTGTCTTTCAGACTTTATCACTTGATTCTTCAGTTTGATACAGTTCTGATCGAGTATC
AAAGGCGAGAGCGTCCTAACGCAAAGCAACCACAAAACAACACTTGCACATGAATTGTC
AAAATATAAGAGGACTTCATTTACAGATTATGCTCTATTCTTTATTGTTATTAACGAGATTTAAAA
AGTGCATGTCACAATAACTAAAGACACCACAAATGTGCTTGTGAACACATTTTAGTTGTACC
CTAGTGACTGAGCCCCTGTGAGCTAGAGACAGACAGACAGAAAACAACAGAGAGAGAGA
GACAGACAGACAGAAAACAACAGAGAGAGAGAGAGAGGGGGGTATGTCGGGCAATTGA
CCGATTTCACGCGCCAGCCGCAAGTGAATTTACATCGAAATATGAATAGTGGTTAGTGTGT
GTTGAACTGATATATGCTGAATATAGAAAACGTGATTTGATGCGATCGCTTGATTAGTTGATAA
CTGTAATTAGAAATTAAAGAAACATTATGTGCAATCATTGCTAGATAGTAGAGTAAGGTATTTTA

TCTAGATAAGGAAAGTCAGTAAGAAGAAGTGAGTAAGGTGATGGTGAGATTAGGCGAGAAA
TCGCAGACATCTACCTCTGGTGGCAGCCTACCGAGGGGAACATTTGTCAAGTGGGAATGT
TGTACTGTCAGCAGAAGTAGAGCGTTGGCAGTGCTAGTAGTAGTTCAGCTTCATTGGATGAT
GCAAACACGAATAAATCCAATAAATGTATATTTCACATACTATACCATCTGTCTAAATTGATTCA
TGTATGATAAATTAGACCAGCCTAAGAATAAGATTGGCAAAACATGACAACGATAATAAAAA
GCAGAAGAAAACAGGCACTTACTTTTTTTGCTTAAATTGCTTGATATGATGGAAAACATTAAC
TACATCTTGAATTCCTCGCGGAGCTTCTTCAATCTTGGACGCCAGGTTAATGCAAGCCATC
GCAACCACCTAGCAACAAGTTTTCATTATATCAGATTAATTTTTACAATATAACAGGCAGGAT
GAAATAGTAGTAATATTGCTTGTTACACCTTTTAGGAACCCTATAACATTAAATGCAAACATGA
TCATTTCATATAAACTGGTGTTGCCAAAGGCTTATTGACAATTAGTGACCAAATTATGCCATAT
GTACATTGTATAAATATCATGAAAATCATTAATAAAACCTCTTTTATGTCAAGAAATATGAAGCA
AATAATCCCATGTAGAACAAATATCCCAAAGAGAAACTTAATCTCTGTCCTAGATGAACGTTT
GTTAAAATATACAGACATACACATCTGGACATAAATGTGACAATACAGATGAAAGCATTTGAA
GAGAATGAACAGCATTTACCTCCATTCCGTGCTTCACGAAAGACTTTGAATAATAGAATCGT
TGAAAGAGAACTTGCCCAGTTGCCATCGCCACCTATTCGCATTCCACAAAACAAAAGTTTA
TTACCAGGTCCATCTGTAAGAACATTAATTGCTCTACCTTCACAGTCAGTCATCCCTTCTCAT
CTTTGCTAGCCAGCACCAGCTTAACAATAGTGACAGAAGCATGATTAAAACCGCTGGAGTA
GCCATAAGTTGGACACCGAGATCAGTTACTGACTAGGGCATGATTGGAAACTGCTCCGGA
CATTTGCTTCGGCCGGTTATTGACATCCGGTACTAACCGGATTCGGCCAAATATTGACATAA
GATAATTACCGGATCCGAATATCAATCAAAAGTTCCTTTCTTGATCCATTCTGTGTGATAAGAA
TTTAACCAAACACAGCTCTACCTTAGCTTAACAGCTTAGCTTAAATGCAAGACAGAGGAAG
GCAGATGCCCCTAAAACAGATGCCCTGGAGGCAGGTCGTCCAGCAAGCATCAATTAAGC
TCCTTGTTCTCACCACCGACCTCCCGTGGGACAGGCTGCCAGGGTTTCCGTTAACGCCC
AAATGGACGTTTTTACGTCCGTGAAAATTCTCCAAAGCGTCCAAAACTTGTGGGCTGGGC
GTGAGTAAAATTGCAGTTATGAGTTGCTTACGTCCATTTGTTTCTGTTCGGACGTATGTGCAC
CAAGCACTAGGGCTAATTAAATATTATTTCGTCCCTATGGAGATTGGTCTACCGGGTATACC
CGTTGAAGCTGTAGGCCTACTGTGAATAGCACAAGATACCAATGTTAACGTAGATAAGGTCA
GAACGAATTATATATACATATATGGATAATCAAAGACACCTTAATAAATATATATAATATAGTCAA
TTAACCAGCCACAGTAACAGAAGAGCAGATGGACAGAGGCAGACACACCGACGGAAAG
AGACGACAGACGAGCGTAGAGAGTCGGGGGTTAAATGGGAGACCTTTATAGCGAGCAAT
GTAACAGAGAAGTTCTGTAAAGAAGAGCGGATTACGAGAGAGGAGTTTGACAAGGCGATG
ATAGGCTGCAAGAGTGCAGAAAGAGACGACTTTTGAACTCGAACGGACTGAAGACGAGAT
TTGAGAGAGATTACGATGATGATGAACGGAGAATAGTTAAGAAGGAAATAGGCCTACGAAT
CACTGATCAAGAAGGAGGGAAGAGACCGAGGAACCGTTCAGGAGAGATGGACTTTGATT
GTGAGGAACAGAGAGATATAACAGAACAGGCGGCGGAATGAAATATTAATTCGAGCCGAT
GGAGTAAGCCTAAGACTGAAAAGTTGCAGCTCTGAGCAGTGCGGTAAGACTTTGTAATGAT
CGAGGGACTTTCAAAACGTGACTGTGTTCATAAGTAGGACGGCTGAGTAGGCCGAATCAA
ATGAAACACTTGTATGCTTCAACTAGTCTGTAGAGTGTAGACATCATCGTGAACATCTATTGG
CTTTCATACTCGTCAGCCACTGTACCAAACTAATTTAGGATTTAGCTTAAACCACAGAATCCT
TATCTCACTGACTCATAATTATGACAGACCAGTGACCACGGCTTCGGCTCAGTAGGAATTA
GGAGGCACTAGTAAGAATGTTGAAGTTCGTCGTGCATTCTGGTGGAAACAATAGCACCGA
GGAGAAACTTGTAAACGTTAAGAAAGATATCGTTGTGCCAATCATAGATGACATACACCGTG
ACGTCGTAAAAAGAAATATTGCCATTAAAGCAGCATCGACAAAGACCGGGCCAAAATACA
ACAGCAACTGGGAGAAGACGTATTCTTAGCAATTTGTTTACAAATAAGAAACAAAATGTGTA
CGTCCTCTTTTTTCACTCTAACGGAAACGCTGCAGGCTGCCATCACATTCCCTCCGCCTTA
ATCAACGACCCTTATGAAGTGCTGGACACCTTCAATATTACCCTTGGCTGGCTGCATGATT
GTCCCACTGTATCAGATGATCAGGGCACCTCACCTGCTGACGAAAGTCCACATCTGGCCA

TTGCAGAGATGTTCAGTTAGGCCTGCACATTTCATTACTTAAAAGAATTAGTTAGGTTAGGCC
AACGCTGGACAGCGATTGATTAACTGACTTTAAAGCCAGAGGCATGTAACGGGACTGGCT
CCACAACAAGTGAGGCTACCAGCTAATACATATGGGCACTGACTGAAGGCTAGACTGACG
CGTGTTGGCCACACCCACTAGAGTGACCGACCGATGGTCGAGATACCCACGCTACCAG
ATATGTAGGGGCAATAAGTGAGGGCATGACTGACAGGTGTTGGCCACGTCCACTAAAATG
CAGTCAAGTGGCAATATTCGGTACCGGAACCGGATTTGCCAAATATTGAGGCCAAATATCC
GGATCCGGTGGAAAATGTCTATCTGGTCATGCCCTATTACTGACCTAGATACACAAGACATT
ACAAACCCCAGCTGAACCAGCATCAGTTCTACAGCAAGATGAGAAATGCAACAATCACAC
ATTAACAACAGCCCATATGTTCTTTCCATGTATACCAGAAGGTCGCAACAATGCTGTCTTTG
GGAGCAATCTAAAGAAACCTGTGTTACAATTAGGGATGTTAATTTCAAATTTTTGGGCAAAAC
GATTATAGGTTTTTTGCCACAACAGTTATCCGTTTACAAAAACCGGTTTTAAACCAGTTATCA
GTTTTACTGGCCTCCATAAAAATATAGTACTATATAAAGCAAATAACCTTATGTAACGCATTGT
AACTTGCACACGCACAACAGAAAATAAACACCAAATTTCAAGACTTAGCCTAAGTGAAGTT
TATGAAGACAATTTATATTAGTATAAATGCAGCAGAGCGCGTTTGTCGAGTCCATATCTTTCTA
TATATTATCTAGATGTCAGCACAGTCATAAGTAGCCTAATTAAACCGGCAACATCAAAGGCG
TGAAAATCAATTACTCTTAATCGGTCCTAGGACCTGTTCAGAGTCAACGATTCTCATGCCTC
ATATACCTTTTGATGAAGCCGGCTTAATTAGGCCACGAAGATCGCTGTAAATACAACCGTAC
CAACTAAGCAAATGCATGAAATAATTGTTTCTGGCATTTTTTATCATCATAATGCAATTTTCTTC
AGGCAAAATGATAAAAGAAATAAATTCTATTATTATTTGCCAAAGAAATATAACCGGTTATGGG
TTTTAAAACAGATAAACCGATAGCCGGTTATCAGTTAATATCCCAGGTTATAATGACAATCAAT
ACATATACATGTATATATGTGACCCACCTACCCTGTATTTAGTAGGGCCGACATTGCTAGGCT
AAATATTCTGCGTTGGAAAATTCCGCGGCAGAAGTAAGTTTTGCGACAATTCCGTGGCTTA
CATTATCATTTTCAATTTTTCATCAATCGAATCGTTACTTACACTACATTTACGCATCATCGTGA
CAGTCGTTTATTGTTTGCTGTTGCGGCAGTCCCAAGCCTGGAAACTCACTATTAGAAAGTGA
AGGGTCGATTGAATGGGTGCAGATTTGTCGGTCGTAAAAGAAGAAACACGAATTAAATCCA
ATGGCAGGTGAAGAGTGAATAGAAAAACACTGAGATATGGAAGGCAAAACAGGAAAAGGT
GATGGTCGCCAACACGGTCGGAATCCCAGAATACCTGAGCCAGTTCCAATAGCCTGGAA
ACTCACCTAGTGAGGAAGTGAGGGGACATTGGACACCAACGGGCTGTGGGCCCAAGAC
TATACAATATATTGTATTGTGAAATATCTGCGTACATCAGATGGAAAACGATGTGTGTTACGAC
ATCTGGCAATTAATTCGCGGTGTTCAGGGATAAGTGCACAGAACAGGGGAAAAGCAAGAT
TGTTATTTTTCTATCGCTGTTAATGAACATGGAAGAATTAATAATAACAAATGTGAAGATATTACT
TTTAATATATATTATATATATATCCTATCAAATTTCGAACATGTGGGTCAGTACAATTAGGTGTTGT
GAGGGTCTGTTAGGGTCATGTTAAGCGTCGTGAGGGGGTTGGGTTAGGTCAAAATTACGGT
CAGTGTTGTCTCTAGGGTTTAGGGCCGCCATTCCCAGCATCCGTTACTTCAGGATAACCAA
CAACAACCACCCATCGCCAAAGAATTCTTCCATAGATCCATATTCTAGCTAATAAAGAGTTA
TTTCATCAATCCAACTTCTATCTAATCAACGTCATATTTGGCTAGTAATTGTTTCATCAACCCA
ACTTCTTGTTAATAAGGACTTCTTTCGTATGGAGTAGCATAAGCATTATGAATATAACGACGGA
CGCTGGGAATGGCGGCCTGATACCGTCTTTAGACAATCTGCCGAAAGCCTTCCGCCGCA
AAAGCATATATTTCCAGAATTTCGGGCTGAATTTTCCGCCGCAGAAGCACCACGCAAACA
AAAACGCCTCATAAAAAATGATACGTTTTTAAATGGTTTCACAGTAGTGATTGTCTGAATAATTT
AACAGGCAAAAAGCGAATGATTATCACCAAAATGATATAAACCAACCTGTGGAAGTTTGAG
CAGAATGCCAGCAGTCTGAATCAATTCGCAACCCAGGATCCGCAGATCCATCTCCGTTTC
TTCGGTCAGTCCATCAATCATGGACGGTGTCGGCGACAGGTTTTCGTCGGGGATGACGGT
ATTATCTAAGGTCATGATAACTCGGCTGAAATCTCGAACGAACTTTGACTTTTCTTTGTCACC
TGCTGGTGGCATGGTGTGTGTTTTATCCGTGTCCGACATTGATTTGATTCACTCGATTCAGAC
TTCGTAGTCTCTAAATTTTTCTCTTACACGAAGATAATGTTGTTCGTCAAACAATCCGATAACC
TACAAATACCTTAACTTATAAATTCAATTTCTGACGATAAATTCTACAAAAATATTTTGAACACA

ACTTCGACAATATGGCGACCAATGAGTTGACAATGACAACAACTTCTTGTGCAAGACTCTA
CCTCTAAAATCATTGACTTCAACTCAATGATTGACAATGGATCTATAGGTATATTTATAGTAATG
GATACGCGCGAAATATTTGAGTACATCCGGTGGAATACGACGAGCGTCGCGACATCTGGC
AATTGATTTACGTTTTTCGGGATAATTGCACGGAACAGAGGAAAACCCGATTGTTATTCTATC
GTTGTTAATGAACACGAAAGAATTAATAATAACAAATGTGCAGATATTATTTTTAATAACCTTCA
ATTAATTAATCTATAGGCATACTGCGGTAAAATGCTGTGGGCATTATTAACATCTTCACGCTG
ACAATCTAGGCTGACGCAGGGCTGTCGCGACCCATTTTAAAAAACCTAGGTTTTTAGGTTTT
TAAAAATCCTACAAAACCTAAAAAGTTAGGTTTTTAGGTTTTTTAGGTTTTTTATTTTCAAATCAG
AATTTTTACGTGTTCACGTCAAACTCTGTAAGTTTATTAGAATTAATTAATTAATTGCTGTTATTTC
GTAACGTTAAACATAGAAGTGCATTAGTTAATCTTCATGCATAGCAGTTTTTCTTGATCATATTC
CTGTGTCCAGTGTACCGCGAGCGACTGTGCGTACATTAAAACCTAAAAAACCGAAAAGAC
CGAAAAACCTAAATCTCTTTTTAAAAACCTTAGGTTTTTACCAGCCCTGGGCTGACGCCATT
TGCAGGATCACGTACGACTTTCCAGTCTATTGTATAATAGATCCCAAGGAATCTATATTACAG
GAATGGTTACTCAAGCTAAAACTGTGCAGCGATTTTACACTTAAACATAGATGGCGCCCCA
AAATTAAAAAAATGTTTTTACATTAATTATGGTGTCTTGAATTTCCGTAGGCAATTCTTTACTTCG
CTAACATAATTGTTCGTCGATTAGCGTTCATAAACATAATAACTTGAATGCATTTGTTATGTAAA
AGAGATTAGAAATTTTCCGGAACCTTGATTATCGCCCAGAATTTAGGATGACAAACTTATACA
TTAAGTTTCCAGTGTGGTATGAAAACCTGTCCGGCGTGGTCAGCTGTCCGGAAAGTATCTA
ATATAAATATCGATGGTTCGGATGAGTGCTGTAATTGGTTCTCGTGACCAGGGTTGTTAACGC
TACTTTGAAAAAGTAGCGGCGCTACCGCTACCGCTACTAAGATTTAAAAGTAGCGGCGCTA
CCGCTACCGCTACTAAAATTTAAAAGTAGCGGCGCTACCGCTACTAGCGCTACCGCTACT
CTGACAAGGAATAGCTACAACAAAGTAGCCTATGCAACAGGACTCAATCTGCCTTTTCTATC
CCAGACCAGTACATCAAGTAGGCGCAGTTAAATGAAACATTCGCTTCGAGTTAAAATTACTA
TTATTTTCTCATCAATCCAACTTAACTTAAGCTTACACTACGATATTTTCGTCAATCCAGCTTCT
ACTTACACGTAGGCTATTGTAGGCCCTTCATATTATTTCATCAATTCAAGGTTACTGAAACTAG
TTATTTTTTGCGATAAAAACATCTATTAGACTATTAGGCCGTGGGCCAATCAGATTTAGGGATT
TCAGCACATAACTTCGCCTACTTGATGTAATGGTCTGGGATCCGAAAAAATGCCGCCCGG
CAAAATGTAATGCGGCCCTCTGATTAGCTAAATTTCAATGGAAGACGGGAGTATAGATTGGA
GGGCGATGGAACGTACGTCACGAAAAGCATATCAACGACGTGAGAAGTTGATGGATTGGC
GAGAAAACGCTCTCAGAAAAATGAGCAGCGGAAAAAATTGGTTTTCTTTTTTAAAAGTAGCG
GTAGCGAATAGCGCTACAAAAACAGCTAAAAAAGTAGCGGCGCTACTATCACCGCTACTT
TCAGCCAAAAGTAGCGGCGCTACCGCTACCGCTACTGTAAAAGTAGCGCCGCTACAGTA
GCGGTAGCGCGGCGCTACCGCTATTAACAACCCTGCTCGTGACTAATGACGTCAGGCG
GCATCATTTTCATAAACAGAAAATCTAATTACTAAGAATTTCTTCCGTTGATTTAGCCTCATTTC
CGTTAGGAAATGATTATTTTCAACAAAACAACATAGATCTACATGTAGAAATAATTCATTGCAT
CCAGGCTATATGACGTCATTGGTCACGAGAACCAATCACAACACTATCCAGACCATCGTAT
TTATGTTAGAGTTTTCCGGACAGCTGACCACGCCGGACAGATTTTCATACCACACCGGTC
CTGTGCATACAGGTCCTTGAATAGATCCATTGATTTCAGTCAAGTCAAGTCAAGTCAACGGT
AGTTTATTGTTCGCCAAATTTACAAAACGAGTTTGCAGTATACGTACAAAGTAGGGACAAAA
GGGAAAACCCCAGGGCTGTCGAGACCCATTTTAAAAAACCTGAAAAGTTAGGTTTTTAGGA
TTTTTAGGTTTTTTGATTTTCAAGTCAGATTTTTCACTTTTTCATGTCAAATTCTGTAAATTTCTAT
GAGTTCATTGGATTTATAACGTTCAACATAGAAGTGCATGAGCTATAGTCTACATGTATAGCTT
TTTATTTGATCGCATTCTTGTGTCCAATGTTCCGCGTGCGACTGTGCGTACACATTAAAACCT
AAAAACCTAAAAACCTAATTTTTTAAAAAACCTTAGGTTTTTACCAGCCCTGGAATCGTAATC
GTAATCGTAAACTCCAAACTTCTAAAGCGCCACTCAAAAGCCAAGCGCAGGGCACCAGC
TTATTCACGAGCGCTGCGTCAAATCAGAGGGGTTTTCCAAAGAATAGTCCGTGGGAGGCT
CAGGTCCGTTTTCCAGAGGGTGAGAGAATGCGTCAAATCAGAGGTGTTTTCCAAAGAATAG

TCCGTGGGAAGCTCGACTCAGGTCCGGTTGCCAGAAGGTGAGAGAAAGCAGATTAGGC
GTTAAGGCGGGTGTAGTTTAGGAGACGAGTGGGAAGAAATAGAATCAGGTGAGTCAGGGA
AAGAGAGCTTGAAGAGGTGGGACTTGAGTTTCAAGTGGAAAGCCCGGGGGGTGACAGAC
AGAGGAGCGTGCTGAAGATGATGTTTTATGATTTGGAGTGATGATGGTGGAGGTAAAGAAAA
GGTGCGGAGTTCAGGTGGCAGGTCATTCCAGAGACGCGGTGCAGTGACGGATATGGCTC
TTTTGGAGAACGTGAGGTGAGAGGTGACCGGGGGTCGAGAAAGGGTGAGACAGGAGGA
TGATCGGGTAGAGCGGGTTGGCTGAATGGTGAAAAGCTCGCGAAGGTAAGTGGGCTGGG
AGGACTGGAGGGAGTTGTAGGTTAGAGACAGGACTTTGAAGTGGATGCTCTCTGGGATTTT
TAGCCAGTGAAGTGATTTAAGGACAGGAGTGATATGATGATGCCGGGGCGTTCTGGTGACA
GCGCGTGCGAGTGAGTTTTGGATGAGCTGTAGACGCTGTATTTGGGTGGAGTCGAGGTTG
AGGAAGAGGTTGTTGCAGTAGTCTAGTTTTGAGTGGACGATGGAGGTGGCAATGGTGGATG
CAATTTTAAAGTCAAGCATGGGTCGGATGCGTCGGAGATCGCGTATGTGCATGAAGCAGG
AGCGGGAGAGGTTGGAGATGTGGTTGGAGAATGAGAGATGAGGATCAAAAGTAACACCAA
GATCGCGGACAGGAGCATCAGAGATGAAAGTAGTGGAAGAGGAGTTGTTGGACAATCGTA
TGGAAGAGTCGGGGATTTTCTTGATTTGGGCAGGTAGGCCGATTATAATGAATTCTGTTTTG
GAAGGGTTGAGGCAGAGGTTGGATGACATCCATTGAGAGATTTGGTTTACAACCGAGAGTA
GGTGGGCAATTGAGGTGGAAAAAGAGGCCGGGGAGAATGATATGAAGAGTTGAGTATCGT
CAGCATAGAGATGGTGGTCAACAGAAGAGGTCTCTATTAAATGACTTAACGGTGTGGTGTAT
AGAATGAAGAGGAGCGGACCCAAGACGGAGCATTTAGGGACCCCACAGGAGAGTGGGA
GGGGTTGTGAGGTGTGGTTAGAGGCTTTGACAGAGAATATAGAAGATAGATATGATTTGAAC
CAGAGTAGAGCAGTTCCAGAGATGCCAAACCAAGAAGAAAGTCGATGAAGAAGGAGATTG
TGGTCTATAGTGTCGAATGCAGCGGAGATGTCAAGAAGACAGAGGCATATATGATATACTCT
CTGATGGCTGACGGCAACGACCAGCTTATTGTACAGAGAGACAAGCAGAGTTTCTGTGGA
GTGGTGTTTAGTGAAGCCAGATTGGTGAGGATTGAGAAGTGAATTTGAGGAGAGGTAGTCAT
TTAGACGAGCAAGGACAATTCGTTCGGTTAGCTTAGATAGAAATGATAGATTATAGATGGGG
CGGTAGTTTGATGGGTTTTCCTTGTCAAGATTAGTCTTTTTGAGAAGAGGAGTGACTAGAGAG
TGCTTGAAGACCATTGGGAAGTCTCCAGAAGAAAGTGAGAGGTTGATAATTTTGGTAATTAC
AGGAACGAGGACAGAGGCACAGTCTTTTAGAAGAGAGGTGGGGATAGGATCAAGTTCAC
ATTGCTTATTGGGAAGAGAATTTAAGAGGAGTGACACTTCTGCTTCAGAGGCGGGAAAGAG
AATTGAGAGTGATGAGTGGGTAATAGGAGGAGTAAGGCCTGCAGGAGAGGGAAGTGAGTC
AGTGCTGGCTGCATGCGAATCTAGAAGAGACTGTAGTGTGAAACGAAGGGATGAGATTTTG
TCCGAGAAGAATGAGCAGAAGGTATCAGCAATGGAAGGCGATGGAATGGAGCTAGGGAA
AACCCTAAGGAACGAACCGTTCCGCTGATAGGCTCACCTAGTTTCACAATCAGTTTAGGCT
GCATAACCAATAAAGTGGTATTTGTGAATACCTAACTAAAAAACAGTATCAAAACCAATGAAA
AGGTAGTAGATAATAAGAGTCATTTGGCTATAACACAGTTACAATCAGTCCAATTTATGATTTTA
TAAGCGTTAACTAACGCCTATATTTTGTCATTAAATATAACAGAACAGTAACACCATAAGCCA
TATTTTTACATGTATATGAACAGTAGTAACATAAACCATGTTTTACATGCCTATAGCCTACATTA
CGTTCTCATGATCATTCAGTTAGCGCAGTTAGCACATGCATTAAACCTAAATAACAGTATGTA
GTACCAAAAGCCATGTGAGGCTATTACTTTTATACATTAAAAACCAGTGCAGTGCAAAAAAA
CAATGCAAGACAAAAATAAAACCAGTCAAATGACAACGTTGGTTGAACGTAGTGCCTAGGC
CTACTATTTGACTTGGGTTCAACGCAGGGCTGTCGAGACCCATTTTTAAAAACCTAGGTTTT
AAAACCTAAAAAGTTAGGTTTGAGGTTTTTTAGGTCTTTTGATTTTCAAGTCAGAATGCACATG
TTTACCTTTTCTTGTCAAACTCTGTAAATTTATTTGAATTAATTGGAGTTGCTATTATTTCGTAAC
GCTAAACATATCAGTGCATTAGTTAGGCTAATCTTCATAGGCCTTCTTCTTGATCGTATTCCTG
TGTCCAGTGTTCCGCGTGCGACTATGCGTACATTAAAACCTAAAAACCTAAAACACCAAAA
AACCTAAATCTCTTTTTTTAAACCTTAGGTTTTTGCCAGTCCTGGTTCAACGACTAAACTAATG
ACGTATTTCTAGTGTTCCGCGTGCGACTGTGCGTACATTAAAACCTAAAACACCTAAAAACC

TAAATATTTTTCTTTAAAAAACCTTAGGTTTTTACCAGCCCTGGTTAAGCTCAGCTCGTTTTGTT
TTGGTATAGGCCTAAAGCCTATCGAGATGTGCTTTAAAAGGCAAAATATAGTCTTAATGTTTG
CCGTAGCTATATCCTAGAAATTCTTTGCATAAACGACGGCATGATTGACACACTCGGCCTGT
AAGCCTACCTTGGTGAGTAGGGATACGGGTCCAAGACTACATACCCAGGGCTGGTAAAAA
CCTAAGGTTTTTAAAAAAGTTTGTAGGTTTTTTAGGTTTTTAGGTTTTAATGTCTATGCACAGTC
GCACGCGGAACATTGGACACAAGAATACAATCAACTAAAAAGCTATACACGTCGACTAACT
TATGCACTTCTATGTTGAACGTTATAAATCCAATGAATTCATATAAATTTACAGAGTTTGACATG
AAAAAGTAAAAAAATCTGACTTGAAATCAAAAAACCTAAAAAACCTAAAAACCTAACTTTTCA
GAATTTTTAGGTGTTTTAAAAAAACCTAAAAAACCTAGGTTTTTTAAAAGGGGTCTCGACAGC
CCTGCTACATACTGGCAATTCAGGCTTAGGATCTGGTAGACCTACATGTAATTAAGGGACTT
AATTGTGCACTTTTCAGGCCTTTCACTCATAATGATTTAGCAAATTATTGGGTTATTCGTCTTAT
GTATGTGATAAACCTTCGATTGCGTGACAATTAAGCAAAAATACAAACGAAAGCTAACAGTC
CGTATAATTTTAATAGAACGACTCGAATAGCATTCATTATTTTGGCGACGCACTCACTGACTG
AACTACTAAAGCTGAAAACTTTGACAAGTATCATCGAGTCAGAGACTCTTTCCACGGATATC
CACGAGACTCGGAAATGTAAACAAGCGTGCTTCTTGCCAGTGTTTGCAGACTCAGATAATA
TTTGAGATTTCTGAATGCAGCTGTGTTAATCTATTATTTGAGATGAAATACACCAAGACGAATA
ATCATTATTAAGTAGGCGTACAAGATGGCAGCTAGGGAGATGCATTACAACATTAGCCAATC
AACTCACCAAGCGTTTAGTGAATTGACAAAGTTTTTGGCCAATTATAGCTCCATCTATAACTT
CAAAGTTACAGAATTCTTCCGCCAGCGGATTTGGGAGAAAATTCCAAATGAGGTAAACCTT
TCTTCATACAGGTACGTTTTTCATTCATTCATTCATTCATTCATTCATATTTCATTTTTGGCCAAA
ATATTATCAGATATTAAAATTCTTGTCTAACCAGAAGCTGGATTAGATGATAATAACGTCGCCC
ATAGGCGTCAATCCTTGGGGGTCGGGGAGATCGTGACCCCCAGATTTTGGAGTGGATGG
GTCGTGGGGCCTACATGAAATATTATTATATCCTATAATGTAAGGAATATGAGAACACTATAAA
AATAGAAACTGGACTGATGAAGGAAGTCTTCATTAACTAGAAGCTGAGAAGATGCTGAAAAT
AATGTGTATTTTTAATTTCAGTGGGTGCAGCTGTTGACTGAGTTATCCTCTGATGAGTTGAATG
CTATACCTTATGGTTTTGTTAAGGTATTGCTTCTTATCTCGAACTTATATTTTTTCTTAGCCTTAGT
GTATGAGGGTGAGGGACTAGCACTCTCCCTCACTCGACTAATCATTCATCTTCAGGGGTAC
ACTGAGTATATTCATTTACTGATTTTGCTAGGGGAACAAGTGGAACGGAAATCTCGAACATCT
TTCTGTTCTGACTTGTATTTGAACCAGGGTTGGCACGATTTAAATCAAACGATTTAAATCATTG
ATTTAAATCACGATTTAAATCAACAATAAAAATCATGATTTAAATCAATGAAGGAAAATTTCTATT
ATTATATATAACTTGATGATCACTTTTTTGTCAATTCTATTAGAATGTTTCCATTTCATTCGCATAA
CTTATTGTTCATTCACTAGCTGGTACATATGAAGTTAACAAATGCTATTAGATACATGTACAATT
GTACATCTTTAGTTTAATTTAGTTGTGCTACTCTTACAGTTTTTGGTATGTATGGCTGGTGTACA
GTGTTGCGTGCACTTGTCAGATGTAAATAAAGGCCACTCATTTATTGTCTGAATAAAATCATA
CGAAATATGTTGAAGATGTGTGATTGTTTGGGCTAAATTTAAAATAATTCATAACCTCAGTGGC
TTTTAACAGGAAGTTGTGCAGGCGATAATGATGAGACTTAGTCGAACTGCAGAACTAAAGC
ATTTCTTAAACTTTAATCGTTTACGACTTTTAAATAAACAATTCAAAATTAAAGTCTAGCTTGATT
GCAGACCTGTTCTGCAAGATTGTTTACATAATTATGTCAATTATAGCTGCAGTAACCTAGAATA
AAATTGAAAATTCTGATGTAAATCATGATCTGATTTAAATTTAAAAAATCTGATTTAAATCAAAGA
AATCCGATTTTTTTATTCTTTTTTAAAAATCGTGATTTTTGCCAACCCTGATTTGAACCTAAACC
TGTGCATTGATAGATAGTCCAACATGCTAACCAGTAGACTATTGCACACCCCTGTAGACTGT
TGCTATATAAATTTGATTAGGTGTCGCTGTTGGCAGTGAAGAAATCCCCAAGTTCTTAAGGAC
CAGTTTATTGTGGTACTGTCTGTTTTGCCTCTATGATTAAATTCTTATAAGTGCTGCTTTGGAAG
TTAACTTGAAGTTTTATGATGCACTGGACACATTGAAGTTAGTCCGTTGTCAAAGAGTTGAAA
AGAAGTGCCCATTATGACATTGGAAGACTTCATACCAAATTCTGTTACTTAATTTATTGCAAGT
GATTACAATTAATCACTCCATTGTTTTAGTTGCAACCCCCAGCCCCAGATGAATGAAAACCC
CTACCTCTAGACTTTTTTATGTTTGTCAAGGAAACTTGGCCGTCGTCATTCTCGTTGTTTCTG

GATGAAGCGCTGAGACTTTCTTTGCTTCATTTGCCCCGGAAGGTCATGGCTGAGCCGGTC
GAAGTGGCGAACGGTTGTCAGTTTGGCATGTCTCCAAAGAAATTTCATGAAGTGCGCCATC
TGGTGGCTGCAATTGATGCGCTGTGCCGCAGATCAGGCTGCAAATTCATTGTGGATATCG
GCTCTGGCCTGGTAAGTTTCAAATGTTATGCACTTTTGTCATTTTGTTGCTGTGTTTCAAGAAT
TCAAAGTCTAGATGACTTGCCTCGGATTTGCACATTTAATCTTCATAGGTCCACAAACTTTTT
GGGCCAAGGGTCGCAGTGTACTTTATTTTCAGTGCACTCGAGGGCCGAAGTGGGACATG
GTGGCGCATTGGTTGAGGCGATTACTCTCAACCGGAGGGTCGTGGGTTCGACTCCCGCT
CTAGCCGCCATGTAGGGACCTTGGGCAAGTCCTTTACCTGCAGTTGCCTGTGCGCTTCG
GCGTGAAACTCCGATACAGTATCTGTGCTGTAGTCGGGAGCGCCTCTGAGTGGTAGAGGA
CTTGAAGGGGCGCTATAGAAATGGCCGGAATGAATGAATGAATGAATGAAGTCAAAATCAT
GAAACAATATTTCTTTAACTGGCCTTCTTTTTGTTGCGTGTGGTTTGATATTGTTGCCAATATAG
GGACGTGGTTGGTTGAGTGGTGGTGGTCGGTGCCTTCGGTGAGGAAGGTCGCTAGTTCAA
CTCCACCTCTAGCCACCACTTAGAGACCTTGGACATTGGACAAGTCCTTCACTTGTAATTG
ACTGTATGACATGATGTGGCTGCCTTGCAGCTCAGCTCTAAATTAGACTCCAGTGATAGCCT
GCTATCATTCATTCATTCTTTACTTGTAAACATTCTGCGATGTGTCAGAATGTATATTAAAAATG
AAAATATTATTATTATCAGAGCTGTTGTGGATTGTAATAAGTTTTAAAATTGTTAATAGTGCAATG
AAGTTTCAATTCATTCATAAAATCGCAGTCATTCACGAGATGTTTATTATGCATGTTAACCTACT
GCGAGGGCTGGATATGACACATTCGGGGGCCGGATCCGGCCCGCGGGTGGTGGGTGA
GGTGTGCATCCCTATCCTAGGTAACTTATGATCCTAGTTTGTGTGGTTTTACTGTTATACACTG
AAGAAAGGTATGTAAGTTATATAACGTAGTGTTCAAGTGAGTACTTAACGGTCTTTACCAGTTG
GAGACAGACGCCACAGAATAGTAAATAAGAAGCAAACACTCAATAATTAAAGCTTCACAAT
GCAAGGGAACAACACGAACATGGGATGTGGTGGCGCATTGGTTGAGGCGATCACCTTCA
ACCGGAGGGTCGTGGGTTCGACTCCCGCTCTAGCCGCCATGTAGGGACCTTGGGCAAG
TCCTGTACCTACAGTTGCCTGTGCGCTTCGGCATGAAACTCCGATACAGTATCCGTGCTGT
GGTCGGGAGCGCCTCTGAGTAGAGGACTTGAAGGGGTGCTTTAGAAATGTCCAGAATGAA
TGAATGAATGAACATGCAAACATGATTGATACATGTAGAAGGACTGTTCTAATAATGTTTCTCT
TTCAAGATTTGTTCCACTTTAACTTGCTGAAATGCTGTTTGCATTTCCAGTTTCCAGGGTTTTT
GTATTTTTGGATGACTTCAGAGCTATTTACCTGTTGTGTTAGTTTCAGAGCATATTGCATGTTCT
TCTGTTGTTGCCAGGGCTACCTGGATTTGGTTCTGAGTAGACAATTGGGTTATGAGGTTCTT
GGTCTGGAGAACCAGTCTGGTCGAGCATCTGGTGCTGGAAAAAGAGCCGACACTGCCTC
TGAGAAGAAAGGCACAGGTTTCTTGTCTATTGTTTACGTTTCTGTAATACGGGTATAGCCACT
ATACAGGCCATGTGTTATATTATGCTATGTCTTCACTATCTATTAAATTATTTCGAGCCAATTATG
TGAGTAGCATACGCTTGGTAGTTCAGCAGGACTTCCTTTACTGACAAATCTGCTGCTCTATG
TGTGATGTATGCATGTATGTATGCGTATGTATGTATGCATATGTATGTGTATGCATTTATTATTGTA
TGTGCAGTACCGTTTGCAAACCTCACAGAAGTTATGAACATGGCCGTCAACCTAATAAGAG
AGATGCAATGTTATGTGCTTGGGTTCAAATTCAATTCAGTTTCTTTCAGCTTTTCTGCTTCATCT
GCATCCCTCGCTGACTCGACCACAATGATGCACTCCGGATACTAAGGTCCCATGGTCTTC
CACCAAAGGCTCTTCATGAAGTTGCAAGATCCACCACCCTGAGCCGCCTCATGTACGCC
GCCCCTGCGTGGTGGGGTTTCGCCAGCGCAGCCGACAGGGAGCGCGTTGATCGCTTC
ATCTGAAGAACAATCAGAATGGGTTACCTACCACCCCACACCATCGATGCTTCAGCCATG
GTTGCCGACGCGGAGGACCGTTTGCTAGCTGCCATCTCCTCCTGCTCAAACCATGTTCTC
CGGCCAGTCTTTCCTCCACAGATTGAGCGCCGTCCTGGGCTCAGGCCCAGACCGCACA
ACTTCACACTTCCTGATAAAGACGACACTAATTTCATTCCTCGCTTATTATACAGAGTGCTCA
CTCACTAAACTTTTTCTGAACTCTCGTAGCTTACATTATTATTTTTTATATCCATTTTACATTTCAT
TAACATTTACTGTAGTCGAGTTGCGGTATGTCGACCGATTGTTTTGTCAATAAAACGAATATAT
CAAGATGAGTATATGCTGACTGATGCTGTGGTTGGATGCGAAGATAAGACAGCTAAGCAAAT
TGAAGTCGATTATCCTTCATGTCTTCATATTGGCGCAAGTGACTAAACCTTCGAGTTGTGTTT

CCGATCTTTCTATGCCCTTTGTATCATTAAGATGAATATACACACACACTGCCTACCTTACTG
ACCTTTGTCGCCCTTCCCTTAGCGCTCGGAGCACCCGTCACCTTCGCTCGCTGAGCAGG
GCCTCCTTCATGTCCCATTTGCTCGCACCTCCACCATGCAGAGCCGGGCCTTCTCCGTG
GTTGGCCCTTCGGTATGGAATGGTCTCCCACTGGCTCCCCGGTCACTTCCAAGAGTATTC
TCCTAGAAATTCCTTCAGCAACTTAAAACAACATTATTCGGCTGTGCTGGGGTTAGGAGCG
CTTCTGAGTAGCCCCACTTGAAGAGGCGCTATATAAATGTCTGCAATGAATGAATGAACATT
AAACAATCCTAGTGAGGTAGCAGAGATGACCCTTTATACCTGTGCAATTGTGCATTATTTTGT
TCACGGAATGCATAGGTTCTCAGGGTTCTTAAAAGCCGGCCATGCGTCAGATCGTTAAGTC
ACTTGGTAGTCAACTAGCGCTTGTTTACAAACGGACTAGGTAAGCAGATCACTACAGACTA
GGGAATGACCGGGTAGACATTTTCTACAGGGTAGCAAATCCGAGTACTTGGATGTACTATTT
GCCGGATCCGAGTACGAACCGAGTATTATTTGTTTACATGTAAAAAAATTAGTTATGGCATGT
GTGCCTGGATAATTGGGTGCCGATATTTCTATTAGGCAGCCGTCAGCCTCGTATATATACAG
ACAATAGACTTCAGTGTACAGTGTCTACAATAGGCCTGAATGGATTTGACAAGAAATGCCTT
GTCCCTAGAGGCTGTCAGAAGGAGGGCCAATTTATAAGGGTTAATTCCCATGTTTAAGGCG
TGGGATAATGATTGTTAGTCCAGACAGATAGGCACAGGTATTTGCCTCTGTATTTGCTTGGAT
ATTTGCTGAGGCGAATAGTTCAAAAAAACACGACATCCGGCCGGAGCAAATGTCCAGATT
GATTTCCGGTCATTCCCTACTACAGACCTCGAACCACTTTTCAGGTCGTCTGTTTTATTCTAC
GATCTGAGATCGTGTGTGTTGCAGATTCAGATTCCGGTCGCTTCATTTCGAGACACTTTGAG
CTGACGAACAGCGAAGACAGCATCACGTGTCTCAAGAGCCTCATCAATGAAACATGGCC
GACGTCAGGTTCGGTTGTTTCCGGTTCTCTGTGACATCTCTCTATGGTCCGTACTCAGCATG
CATTCAACACATGCGTGGATGCTGCTCTCAAACAGAAGTCTGTTGTAATTGGACATTTATGTA
GCCCCGCTCTGGGATTCAGTACCAATGATGGTGCTGTTGATGTAGGGGTGCTTAGTGTGCT
GGACTGTAAACTAAGAGGGGGCAGTCAGGGACGTCCAGTGCGTGTAAAGCATAAATGCAT
CATGGTCAAAGTAGGGGAGAAGCAAAAAAACGTAAATTGAACAAAGATATTGGGGGAAATT
TATAAATTTGTTGGAAATAGGGGGGAATTTATAAGTTTTGTAGAAATAAGGGGGAATATACAAT
ATGCATATTTGACTTAAAGGCCAAGATCGGGGAGCTAAATTCTTCTGGCGGAACAGAATAAT
AGGCGTGACTAATAAAATCTGCTGTCCAATGAATACCCTGCTTCATGTCTTGATGACCGATG
ACTTGAATAGATGGTCTCCAGAGACGTGCTGACGTGCCAGAGGCGTTGTAGATTTAGAGAT
TGGGCAAACAAACAGGTAAGCTTCAGAGGGAAACTGACCAGCGACCTGCGTGATTGAATT
CACTGGACAATAGCCCTGTGCCTATTCATACCAGCCGCCCCAGCTGTGAGGCACTCTTC
GAGCAGGTGCTGATAAAGATTGACAAGCAATAGATGGTATCCAGACAAGCAATCCTTATTAG
CCACGCCTTGCGTTCTAGGCCTTTAAGGGGGATGGACGCCCCTGAGGGCAGTGGTTCA
AGTCCCCACCAGGACAGAATGATATCCTCCACATTGTCCCCAATACCACAATAGGACCTT
GACTATAAAATGAGTGTTAGTCTATGTAATTAGTGCAGAATACTGGTAGAAAATTTCACTTCCG
GCTACCAGTCTGAAATTTTGCATGCAGGTTGTATATCTAGTAAGCTTCGAAATTCCACTGGG
ATGCAACCTGTAAACCCTCAAATGAGGGCGGGGCGCTCAATAGCCCCCCCACTGGCCA
ATGCTGGTTAGCTGCTTGATGTATGTGGCTGCCTTTTGGAGATATTGTCATAGAAATGCTCTT
CTACAGTTTCTTCTGTAATATAAGTCTTGCACATATAACATATATCTGGCAGTCTGAGCATTTG
CTGGGAATTTTATTTATTTATTTATTATTCATCTAAAATACTTGTTACACGTGCAAAGTTGATGTTT
TATTCAATTTTTAAGTAAGCATGCTGTAACAACGAACATGATTGTGAGTCTTTTGGTAGATTCAT
CACCTTTCGTCCGAAGGGTCATGGGTTCGAATCCCGCTCTGGCCGTCACATTGGGACGTT
GGGCAAGTCTTTTACTCGCAGTTGCCTGTGACGCTTTGGCGTGAAACTCTGACACGGTATT
CGTGCTTTATCGGGACGCATCTAAGTAGTAGTGAACTTGCGCTATTGGAATAGCCTGCATG
CATGAATGAGTGAATGATTAATTGCCAGCTGTTCTTTTCGCTAATTCTATCATTATGAATTTAATT
GTACTTTCATATTGGCAATAACTGAGAACGATACAAATTAAACTGACACACTGACCCAAATAA
TTGCATGGCATTGTTGATATATCATAAAATTTATTGTATTTTATTGATTTTATTGTGTTTCATGTATTT
CGGAAGATGATCGATATACGTAATGTATAGCTGTACTTTTATTGACATCTTCTTTGTGTGAATTA

TTTCTGTTCTTATTTTTTGTCAGATCCAGACGCAGGCAAATGTGACATCTGTTTGATTGGTTTA
CATTGCTGCGGTGACCTAACGCCCGCCACCCTCAGGGCATTCACTCACCTACCTCGTGT
GAAATGTGTTGTCTGCCTTGGCTGTTGTTATCACAAAATGCAACCGTCAGGTACTGTGATCA
TTGCGGTGTATGTAAACTGCCATGTTTTGAATTATTGTTCTCGGCTGTTATGATGTGAACAATC
TATGTGTTTGTGCACTTGATTTTGTATTGTATTGTATTGTATTCAAGTATTTATATAGCGCCCTTCA
ACAGCCATAGGCAAATAGAGGCGCTTTTGGTTCGATTAGCTCCAAGAAAAGAGACAAGTTT
TAAGAAGTGATAAGGACGTAGAAAGATTTTGTGATTGTAGCACTTCTGCAATTCTTGTTTCTGT
TTATGCCATCAAGCATAACTAAATAATAATAATAATAATAATAATATACGTTTACTCAGAACATAT
AATAGGGCTGACGCATCACAACCTTTTACAAACATGCAAGTTTAGATATATCTGTCAATATCT
TGTAACCTAGCATTAGTTTTGATGCATCAGTCACTTATGTAAATGTGTTGAGTATGCTTGCCAG
TATTCTTTTGATGGAGCTATATGGAGGTGTTTTGCTTTCACACAATTGTAACTGATGATGAAAAT
CATGATGTTGGCAACATGATAACAGTGTGGTAACAGGTAGTGAGGATGATGGTGCTTATCAT
GTTAGGAAATAAAAAAATAAGTAATAATAAGTAATAAGTCCAGAGACTTACCAATCAATCAATC
AATCAGTCAAAATTTATATAGCGCCCTTTCATGATACCTACTCAGAGGCGCTCCCGACCCA
GGCCAAGCGGAAAAGAACAGTGATATACATTGAAATACCTAATATCTTAGCATCCACAAACT
GTTTACATCTTTCAACTACAAGTAATAATGCATTAGCACAGCATTTAAACCAGTGCTTTACATG
AAGGTTGTTATCGTGTAAATATGTATTTAAACACAATATACACATATTAAGGCACACATGCGCA
TTGTCTGGTATTTTGCAGGTGAAGGATTTTTGCATTTTCCGTTGAGTACGGAACTGAGGATGG
CTATGAGGGAAGCAAAGATTGTGGATGAAGCATGGCATCTGACGAAGTACGGCCTTCGCT
TGGCTGCTCAAGGAACAAGGTACAACTCCCAGCCTTGGGGCTTCCAACTAATTCGTTTGTT
TAAATCAGGGCTCGAAATGGAATGGAACGTCATTCCGGTTCATCAAGTGCATCAGTATCGT
CGTTCCGTTTCATTAAGATATACCATTACTGGGTCTAGGACATTTCCACCCCCCCCGGATAT
TTCCCCCGGACTTTTCCCCCCGGAGAAAAATGTAAATAACTTTTCTTAAATAGAAGCTGGAA
TGATGAAACAATATCCATCAAGTAGAAGCTGGATTAATGAAACAATTCGTTATTAACTAGAAG
CTGGAATGGTGAAAGAAATTATGCTGTGTAGTGAAAATGTTAAAAATTGACGAAATTAGCCCA
AAGATCGATCTAAACGAACCTTACATTTGATGAAAAACGTTCTTTGTTGAAATGGAAGTTGGA
TTGATGGAAATAGTTTGGTCCGTAAATAGAAGTTGGAATTATGACACTTGGTTCAGCTTATATA
GAGCTTTTCATCCAGGGGGAAAAGTCCGGGGGGTAATGTCCGGATACCACCATTACCATA
ATCATATAAATTTCTCAGAATTTTTCTCTGAATGCTCCAGTTTTTTGACTAGCATTCCGTTTTGTA
AAATATTCCATTTCGAACACTGGTTTAAATGCTGTGCTGATCGCATTATTACTTGTAGTTGAAA
GATGTAAACAATTTGTGGATGTTAAGATATTAGGTATTTCAATGTATATCTTAAAGAGCGTACTG
GTTTGACCTCATCTTGTGCTTGCAATGACCTCATCAGTGGTGTGCAGAGGGGGGGGGGTG
CGGACGGTGCGGTTGCCCATGGCATCCAGCATGGGGCATCCAACGGCCCAGTTTTCGT
AAAAAATTTAGGTAAAAGGGTTGTCAGAAATTTAGGGAGGCGAATCAAAAAATGTTTCTTTAA
AAATCGGAATTTTTTTCGGAAAAAGGCAGATTTTCTTGAGGACAAAAGAAAATGTAGGGATTT
GCCCTGCCCCGGGCATCCAAGAACCTCGTTGGCCCTGGGCATCCAAGTACCTCGTTGG
CCCGGGGCATCCAAGAACCTCTGCATGCCACTGGACCTCATTGAAGGTGATTCATGTGAG
AGAACTTGAGAAATGACTGAGTAGACCCCTTGCAATAATACTTGTCATTTAAAGACCTGTCTG
TCACCAAGATGGAGATGTTTGTGAAGATTTAAGTTAGGAGGTAAGTGGGAAGTCCTGCAATG
TGCTGGCTAAGACCTAAGGCTAAGGTCCTTTTACATGTTTAAGGGCAAAAGGAAGGAAACA
AGCGTCTCCACTATATCTCCTTGTTTTATATATTAGGCCTACTAGTAACATTTGCATACAGGCA
TTTACATTCTAAGAACCTCTAAAGCTGGAATGGACTATCAGCGGACTGGATCCTGCCTGCA
AATGTGTTATATCCGGCCCTTGCAACAAGTTAAAACGTGCAAGAAACTTGTTGGGAATTATTA
TGGCAATTTTATGAAGGAATTTAAACTTCATTGCGCTATGAACAGTTTTGCAACTTATTACAAT
CCGAAAGAGCTCTTGTGTATTCGCTAAATGGCAACAATATCAAACTACAATGAACAAAAAG
GCTAGTTATAAAAATTTTGTTTCCGGTTTTTGATACTTGACTTTCGAAAGTTCATATCATAATTTT
GTCTTCAGCCCTCAAGTGCACTAAAGATAATACACTGCGACCCTCATCCCAAAAACGTTGT

GCACCTCTGCGCTAAAGGGAACAACGATGAAACTGCAGTTACATATATTTCTGTTTTTTTAG
GTGCTGTGTTTAATCTACGAATGTTTTGTTTTACGAAAATCAGGTCACATTGGAGAAAGCAGA
CGGATGACGATCACAAGAATCACGTCCGAAATGTTGCTTATCGAGCTCTTTTTGAATTGCAT
GGTACCTTCTTTCCCACTGATTGATAATGAAGCTAGATAGTTGTTACAGTGGTTATATTAGCG
GTTGTTGTCATGGCGACTATGATCATGGGCGCAGGGGCGGAGACCTAGGGGGGACGGT
CTCCCCCAAAATTTGAGGTGGGGGGGACGGCCCATGCATTGGTCCCCCCAATATTTAGA
GAAGTAGTGTTGTCGAATGCGCACGAAAGTACAAACAGAGTAAAAAAATGGTCTTATTAAGG
AATTCTTTTCTGAAATAGTGTTTTTTCTCCGCCACTGCATGGGCGGAGACTTAGGGGGGACT
GTCCCTCAAAATTTCAGGTGGGGGACGACCCGTGCATTCGTCAATATTTTGAGAAGTAGTG
TTATTGAATTCATGTGAAAGCACGAAACTGAGTAAAAAGTGTCGTAAAGGATTTTTTTGCTCTG
AAATGGAGGTTTTTCGTCAAGAAAAGGGCCATATATGTTATATATCAGATTTAAGACAGTAAA
GACAGGTAAAAAAACAGGCAAAATACGGTCGATGACTAAAAAAAGATCATCAGAAATTTATG
GCGTTGAAATGGAAATTTTTCCTGAAAAAGGTCATTCGAAAATTTGGTCCGCGAAAGTTTTTT
TCCATCACCCCAAACTTGACGCCAAGTGTCTGCCTATGGCTATGATGATGATAATTCATTAAT
TTAGGCTATTTCTATAGCGCTTCTTTGAGTTCACTACTACTCGGAGGCTCTCTCGACACTGC
ATGGACCCTGTGTCGAAGTTTCACGCCGAAGCGTCACAGGCAACTGCGAGTGAATCACTT
GCTCAAGTTTCCTATATGGCAGCTAGAGTGGGATTCGAACCCTTGACCCTTCGGACGAAA
GATGACGAATCTACCAATAGGCCAACATGCCCCATATGTGACTTTGTTGATGACTGTGATGA
CAGAAAGGATCGTTCGCTTGCTTCGGGCAGTGGTGGCATCTGATGCCCACGTCCTCCGT
CAGTTTTCCCGTCTGTTGCAAAAAGGACTTGTAATCTGCGTCCTAGAAAGCATCCATTAAAG
GAAGATATATAAATATATATTATGGTATCAGTGACTATAATGGAAATTTTGATGATAACGTTAATAT
CTGTAAGATGCAATTGAGATTCAATTGGTTTATGATCTCCTCATGAATATTCATTACTTCCCTTT
CAAGTAAAGACCGGTAGATCAATGATGATATTGCTTTTGCAATTTGACAAAATAATGATAATGA
TTATTGTACAGATCCCAAACCATTTATCTCCATATAAATACTCCAATGAGCTGCTCCAAGGAA
TTTTGGCTGACTACAGCTTCTTTTGCAGTGACCTTCAATTGATAACCTATGCATTAATATAATC
GTAATCTCAAGGTTTCTAAAGCGCTACTCAAAAGCCAAGCACACCAGGGCACCAGCTTAT
TCATGAGCGCTGCGACGAATCAAAGGGGGGTTTTCCAAATGGGGGTCAAGAGAAGCTCT
GGTCCGATTTCCAGAATACCAGGAAGGGACAGAGCAGCTGTTAAGGTGGGTGTCATTCAG
GTGGAGAAGGTGAATGATCAGGTGGGTAAGAAAATAATATAGATAATAAAATAATATAAAGTC
ATTCTCTGAGTCAATAGATTGTATTAGTCTGTGACGACTGTCCTACCTGGATGTCAGCATATTC
TTCGTTTCAGGTGATCTGTCTTTCAAAAAGCTCCGACGAAAGCTGGTGACCAAGAACGGTC
ATGCGGACTTTGAGAGCTATGTGAACGAAATTGTGAAAAGAACGACTCTTACAGGCAAGCT
GCATTGCTTCGTGAATGAATCGAATATGGATTTATTTTGTTATTTGTTTGTTCCATGCATCAATT
CCTACCTATGACAATAGGTCCAGCTATCCATGCATTCGTACTTTAATCTCCGTCTATTCATCC
ATACTTCCATCTACGATTCATTCTTACATCCATCTATTATATTTATTATTATACTTGTAAAGTGTGC
ACGGTCACGCCAAAATGTGAATCTGAGGCAACCAGGAGAAGATGTGTCGTTAGATATCCAT
GGACTCGAAAGTCATGAAGAAATAGTGAATTTTAAACTGCTTTTTAACGGGAAACAATCAAA
AAGTTAAATTGGAAGAGCAAGTAACAGTCGATTCCATTCCTTTGGACAGGGGCATTCGCAG
GGGCGTAGACTTGTCATAAATATTGGGGGGGCAAAAATTTGGGTCACAAACATTGGGGGG
GGCAAAAATTTTTGGAAAGTATATTTTCAGAAAAAAATCTTTAAAAGTTCCCTTCAATTCTCCA
AAAAATTCTGACGACCTTTTTTTTTTAGTCATCGACAACTTTTTTCTAAAAATGTACACTTTTAAT
TGAAAATGTACAGATTTACTTCCTTTTCATTGTATTTATTTTTCTTTCTCTCTGTTTCTGCTTTCTTT
CATGTCTATTTTTTTACAAATAAATTTTTTCTTAAAATTCTCATCTGATTATTGGGGGGGCAAAA
AAGGGGTTTTGCCCCCATCCTAATTATTGGGGGGCGCGTGCCCGGGCTGCCCCCACAG
AGTCTATGCCTATGGGCATTCGTACATCCATTCGTACATCCATCCATTTCTACATCCGTTCTT
CTTTCCGTCCATGCTGATTCGTGTAACTAAGCATTGTATTGTATTCAGGTATTTATATAGCGCC
CCTCAACAGCCATAGGCAAACAGAGGCGCTTTTGGTTCGATTAGCTCCAAGAAAAGAGAC

TACCGTAGTTTTAAGAAGTGATAAGGACGTAGAAAGATTGGATGATAGGAGAGAAGCACGA
GCTGAAGGTGGAAGGCGATTCCAATTATTATGGTGATTGTGGCGATTCCATTGCCAATACTC
CATTCATCCTTATATAACTATGTTGTTAAACATTGCCGAGCAGCTTCTAATTTATTTCTGTGTTC
ATATATTATTGCACTTTTATAATGCACTGTATTCACCAGACAATTATGCACACACAACTGTCCA
AGATCATCATTGCGTGGAGTCTGTGGAGCCAACTCGAACCACGATCATCCAGTTACAGAC
CTAACAAGCCATCAAACACACCACCATATATCTGAGGAGCCATTTACCAGAGTTCATTCATT
CATTCATTCGGCCATTTCTATAGCGCCCCTTCAAGTCCTCTACTACTAAGAGGCGCTCCCG
ACTACAGCCCGGATACTGTATCGGAGTTTCACGCCGAAGCGCACAGGCAACTGCAGGTA
AAGGACTTGCCCAAGGTCCCTACATGGCGGCTAGAGCGGGAGTCGAACCCATGACCCT
CCGGTTGAAAGCTATCGACTCAACCAAGGCACCACCATGTCCCATCGACATGGTGGCGC
CTTGGGAAACATCATCTGTTTCCCCTTTCCTTTGTTCACTCGACTGGTCATACAGTAAATCAA
TTATGCAACTAATTACCGCTGTTTTTAATGATTGACTTAACCAGTGTGCTAACGAGAAACAAG
AGAAGAGATAAAATGAGAAAATCATACGTGATGTGGAAGAGTATTCGAACTATGTTTGTGCTT
ATAAACATATAATTTTAGGTTCAGGAATGTTTCTATAGTCATGTTGTTGGTTGACATTGTGGTTTG
ATAATAAAATCTCTGCCATAAATGCGTGAACACTATATCTAGATTAATCATGCGTTGTTTGTTCA
CAACAGACTCCGATGATGTAAAAGATCGTCTGCTCAGTCTTTACAGAGATTATGAACCGTAT
TTTGTTTACATCGAACCAATTACTGTAAGTTCTGCTCCCTAGAAAGTGCATCATTTAGCCATTT
TCGACTCTTGCATCCTGTTGATTTATGTAACACTGATGTTGTTTCAAGTAATGCTGACGTTTAA
GTATTTCCGATCGTAGTGGTACTGTGTATAGAATGTGCTGCTGATAGTACGAATAATGACATTA
ATTTAAGTTTTTAGATATGGTTTTATGTGCCGTATACATAGTAGGTTAACACCTAGATTGGTTCTA
GGACAAAATGGTAGGGGACAAAATGGTACGGACAAAATGGCACCGGTAGAATCATCAATT
AATCAAGCAATCCAGCTTCCATTGACAATATGATTTTCTTATTAATCCTGCTTCCACTTAGACC
CATTTAGGCTTATCACTTATTTGTGACTTGTGATTACTGAATAAAAATTCAATTCAGTTGAATTG
GAATCTATAAAGGTTTCTACCATTTTGTCCGTACCATTTTGTCCGGTCACCCCTAGATTTAGC
AAGAAGGATAAAAGCCTCTTTCATAACTCTTAACTGATGGTAGAATTGATTTATCATGATTTTTA
AAGTGTCCACGAGTAATTACTAATTCGCCTGGAGTGGTCAATAAATATTTTGATTCTTATATTAT
ATGATTTAATTTGTTAAGATCTTAAAGAGTCTTATTATTTGGATCTGTGTAGATGCTTCAAGTGAT
ATTGCAGCCAGTTCTGGAGGCCCCTTGTGGCGATGGATAGAGTCCAGTTCCTCAAAGAAT
CCGGATTCCGTTCAGAGGTTCTGGCAATGTTTGACGACAGCATTTCCCCGCGAAACTCTG
CCATCGTGTCTTCCAGAGACTCTTCCACCATCGACTGTCTCTTCCCTCCCCAAGTGACTTC
CTGACCTTGCCCAATATTTGTTGGGTCAGTGCGCTGCATGCGTGTGCATCTCTGCTAATCAT
GGCCTGAAGACACGCCCAATTGAAATGCTACTGGAGATATGAATGCTCATGCATATTCATG
AGGTGTCAGTATTGGTCTCTTTGTTAGCGGATTGGACTGTTAGATGCACTCACTTGAGTTGCA
ATTGGTTGATGATCTCTTCATGAATATTCATAATTTTTCCTGTGCAGTGAATAGATCAATCAGTG
TTGAATAGCTTGGTATGGAAAATAAAACTGCAGTGGTGTATTTTGGCAGGTTGGCCAGCATT
CAGTGAACTCTGACAGAGGAGTTTCTTTATTGGGAATACTGGGTATTTCTAGGACTGGTTTAT
CAGGAGTGTCTTGCTCCTTTTTGAATAAGCTTACAGTTTCTCAACTTATGCATAACTCGGAGT
ATTGGAGGATGGTGCGAGAAGGCATGTCGTCATGGAAACTGGATGGCAAATTGTCGTTGA
GTCCAGTTTCCGAGTCCGTTTCCAAGAGAAGCCGACTGCCACTCGCGGTTACCATGGCA
AAGGAAGGTACGGGATGCTTTGGTCTGCCCATGCGATATCCATCAACAGAGTCGTCGGCC
GTCATTCGTGCTGGCGGATTCGGTCCATCGGTCGTCGAAACCGGTCAGTTCAACCGGAA
CATGTTCACAGTCTGCGACTTCGGTTCCAGGACCAATAGTCCAGGTAGGCCTGAGATGGA
TTGAAGTGTTGATTGAGTCATTGCCATAGGCGGAGACTTGGCGCCGAGTTTGGGGGGACG
GAGAAAAAAATTTCGCAGACCAAATTTTAGAAAAAAAATTCCATTTTAACGCTGAAAATTTCTGA
TGACCATTTTTAGTCATCGACCATTTTGGCTGTTTTTTGCCTGTCTTTACTGTCTGATGAAATCT
CATATACAACATATATGGCCCTTTTCTTGACGAAAAACCTCTATTTCAGAAAAAAAATTCCTTC
GTGAATCGTGACACCTCTTTTCAACTCGGTTCGTGCTTTCACACGCATCCAATAAAACAACT

TCTCGAAATATTGGGGGGACGGATGCATGGGCCGTCCCTCCACCTCAAATTTTGGGGCG
ACCGTCCCCCCAGTTTCCCCCTAAGTCTACGCCCATGGTCATCGGTGACGTATAAACTTAT
AGCAAAGGAATTGGCGAGTAAAGGCACAGAGGGACTATTATAAGTAATGAGAGGTTTTCAC
TTAATAGCTTAATGAGCTCATTAGAGTTTTTACCAGGATGAGGACAAACTTTTAATTATTCCAT
GGACCCTAGTCACGCCATTGTACTGGAGTCTTAGGACTCGTTGAGTCTCGAAAAATCAGTC
AAAATTTATATAGCCCCCCTTCAAGATACCCACCCAGAGGCGCTCCCGACCCAGGCCAA
GCGGAAAAGAACAGTCTTGAGAAGGTGGTGGAATTGAGAACAGGCACCGTTTGGGAGGT
GCCTTAGAGTCTAAAGTCTTTTTGAAAGGTGTCTGATTGAGCTTCCCTCAGGCTTACTTCCC
TGCATAAGAGCTGCTTATCCATTGAGGATGGAGGCAGGGGCGTCCATCCCCCTATGTCAA
TGATGCATATTGTATTTTGCCCCTTTTTAAAAAAATAATTATAAATTCCCCCTATTTCCACAACA
TTTATAAATTGTCCCCCTATTTTCGTTAAATTTACTTTTTTTGGCTTAATTTACTTTTTTTCTTCCTC
CTATTTTGACCATGATGCATTTATGCATCATGCTTTACACGTACTGGACGCGTCTGGTTGGAG
GTTACCTTGCAGGCTAATTCAAACCCTCTTATAAGTGCAGTTAAAGGCAATATGATATCCGC
AATTCTGTTCTGGCCTGAACCGTTGTCTGCATCGTTGGATGATGAAGTGTTATCTTTTGATTC
GTGGTAACCGAACCCCTTGGTCACAAAGGTAGGCTAAAGGCAGCTAACGTGTTTGGGGT
CAACTCTAAGCATATACGTGTAAAGATCGTATATAGGCCTACCGATAATACATTGTCAGAAAG
ATCATCATCATCATCATCATCATCATCATCATCATCATCATCATCATCATCATCATCATCATCAT
CATCATCATCATCATCATCATCACTGATGGTTATGGATTATGATGATTATTTATAATTATGATTCAT
CATTATCATCATCATCGTTTAACAGGGACGCCGCCAGAAATGTTGTTGAGAGGGGGGGCA
GATGCGAGGGTTTCCAATGAATTTTTTCGTATTTTCATACATTCTTAGGCTGTTATAACAGTGTT
AATGGATATATTTCGAGGGGGCTGAACTTGTAAGACTCAACTGAATATGCCCTTTATTGAAAC
TACTAGATCAGCGGTTCCCAACTTTTTCTTTTGTTGTACCCACAAAATAAAATGTACTTGTGC
GCATACCCCATTAGAAAAACGTTTCATAAACGTTCTATTTAGTAATTTAGTAACCTTTTATTAAT
TGGTTATATGCTAAATAATATCAATTTTTATAAATTTATCATATTACAATGATTATTGTCGCATATAA
ATAAAATCAGTTTAATTATTGTGTTACTCTATTTTGAATATTTTCGAATAACGTGAAGATAGGTTAT
TAGCCTCTATATAATTAATCAGCCAAAATTATTTTGTGAAAAACACAGAATAGCTGAATAGCAG
AGTAACTAATAGCAAGAAAGTATTTATGTTATCTGTTTAAAAAAAATTACAATATTTTTTCCTGCG
TACCCTTTGGCAACAGCTGATGTACCCCTAGGGGTACGCGTACCACCGGTTGAAACCGC
TGTACTAGATAGCGTGAAGCAGTAGCTTTATAAGAGTTGAAACACGCATGGTACCGGTATAA
AATTTGCTGAGTTTTATCAGAAAGGCTACAGTTACCAGATGTCTCTCTCTCTGAGCAAACAC
GCGTGATTAAATGTTGGTAAATCGGTGAAAAGTGTGTTTCAAATAAACGTCTATCGTGTCTTG
GAATTTTTTGGAGGTGCAGGTGGTGGTCAAATTCAGCTGTGGCAGTTCCTCCTCGAACTTC
TTTCGGACGTGTCAAACGCGTCGTGCATCGCCTGGGAGCCGGAACCGCCCCTCGAGAC
AGCAGTGGGTAGGTACCTTGGAGGCGCTTCCACTGCCGGCGAATTCAAGCTCATCGACC
CGGACGAGGTGGCTCAGCGCTGGGGCCGACGAAAGGGCAAGCCGAACATGAACTACG
ACAAGCTGAGTCGAGCCCTGCGGTACTACTACGACAAGAACATCCTGACAAAGGTCCAC
GGCAAGAGGCACGTCTACCGGTTCCACGACTTCCCTTCTGAGCACACCGTCCTGCTGCA
GCCAACCACGCGGGGCTTCGGGTCCGGTCCATCGGCGCCCACCAAAGCCCAGGTGC
ACAGAGGGGCAGAAACGGAGACCGATGACGAGTGTTGTCGTCATCTGCGTCGACGTCAT
CATCTAATGGAGACGACGTCATCGGTTACGAAATTTGTCTCTGGATACGGAAGCGTAGCCG
ATGACGTCACACCTTCGTCATCGTCTTTTTTCCAGTGTAGTTCTATGGCGTTCAGAGGTGGTT
GCTATGGACACCGTCGCAGTGACGTATGGTTACCATGACAAATGTACAGCCGTGTGCGCG
TAGGCAGTCAAAACCAATGATTTTGGAGGTAGGCTACCTCTAAAATTATTGGTCAAAACCAG
ATAGGCTTCCACTGTTAGGGGTCATCCATTTATGACGTCCACAAGAAAATCCGGGCTTTGA
CCCTCCCTCTGTCTAAATGCGTCCACATGAGATCGACCCCCCACCCCCATTGTAGACGTC
CACATGTCGTCGACATGAAATACACAGCACTCTCTTGAAGCAGCTAGTACAATGACCTTCC
GGGCATAAAGCTGAAATTGGACTATAATAATCATTGTAATTTATTTAAAACTGTACTGTTAGTAG

GCCTAATTTCTTCTTATGGTTCTCTGCCTACTAGGCAGGTTCAAATGTTTGACGTCGCCATTC
CTCTTGTTGTTGAGCTTGTTCTTTCAGCTTTCTATATCCTTCCACCATCATCCAGTCCAGCAT
CATATGTCTTGGTCTTCCTCTTGATCTTTTTCCTAGCATTTTCCCTTCGATTACCGTTTTTAACA
GTGATTCTCCTCTTAGAGTGTGTCCGATCCACTTCTTTTGTCTGGTTTTTATTGTGCGTATGAG
GGATCTTTCCTCTCCAATCGTTTCCAATATTTCTTCATTTGTCTTATGTTCTGTCCAGCTGACTT
TCTCCATTCTCCATTCTTCTCCACACAGAGCAGATAAGAGGCCTAATTTCCAGGCCTAATTT
ATATTACTAAAATATATTAAAGAAAAGTGTAAACTTTTCATTTCGACTAAAGACGAAATTCTGGT
CCAAAAAAGCCAACTTCTTTGCATTAGAAGAAGACAGTGGGCTATAATTTAAATATTTTCTGT
GTGGACGTTCACATGGAGCTCACCCCTCCCCCGTCCGCATACGTCCACCTTAGCCTGAC
ACCCCTCCCCTTCCATGTGGACGTCATAAATGGATGGCCCCTTATAGCAGATGTGGGCCA
GACGTACGAGTACCCTATACTTGGGCGACCAATCATGCATTTGATAAGACGTACATGTAGAA
GTTAGCATAAGTTTGTATACTTACAGGCAAAGAGACCTGATTTTGGCAAAACACATTCTGGC
ATACATGTATTTAGGGGTTGTCCATAAAGTATGTCGCATTATTTTTGACCAATTTCCCCCCTTA
CACTGTCACAAACTGTCAAATCTCGGACCCCTCCACTAATCACACACATTTGAAAAAAAAA
ACAACTAACAATTAATAGGGGGCCATAAGTCTAATTTCATTTTAAAAATTGATCTATACGATTTA
TTTGTATCTTTGAATGCTCAAAAATTGTTTAAATTAATATAATTACAGATATTTTGCTGATAACAC
CTTTAAAACACTACCGACATGAACGACAGCTAATAAAGATGCCAAATGCCACATAGCATATA
TTACAGAAGTGTGATCTGCCGAATACCTGTGTCAAAATAGGAAGTTAAACATCACAACATAA
AGTTAAATTTCAGCTGTTTGTATGCATGATATAAAATGTGTGTCGTCACAAAAATGTGTGTCGT
CACATATGGTCTTTACCCCTAACCCAACACACACAAACTGTCACTTTCTTAGAGCCCTCCC
TCCTCTGGAGCACGAGGAGGTACTTTATGAACCTCCCTTGCCCCGGTTTGCTGGCACCTG
TGTATAGGCCGATTATAATAATTGTGTGTTTGTGTGTGCCCCGACTTTTAGTGTAGAATTGTTG
CTGCTATTTCCGGTGGTGCATTATAGGCCTAGGTAGCAGGGTTGACATACCGTCACGAAAA
GCGCGAGTTCGGATACTCTTCGGCGCCTTTGTGACTGAGGTGCCGTCCATAAAGTACGTC
ACGCTATTTTTGGCCAATTTTACCCCCTCCCCCTGTCACACATTGTCACACATCCCGGGAC
CCCCCCTAAAAGTATGTCGCACATCTCGGACTCCTCCCCCGATTTTTAGTAAGCCTAGTAC
AAAAAACCCGGACAAAATTAAGCCCCATGTACGAATTCTCTCTCAATTGTTTGTGGGATTTTT
TGTCCGGGGGGGTTTTGTCTTGGGGTCTTTTGTCTGGAAGGTTTTGTCCGGGGTGGTTTTTG
TCCGTTCCCCCTTCTGTCAGAATTTCTGTCACAACAGAAAGTTCAACACATCACTTTAAATTT
CATGTTTAATATGTATGATAAGAAAATTTATAAGTGTGACGTCACATGCTCTTGAACCCCCTCC
CTCTCTGTCACAAACTGTCACACCTTCTCGGACCCCCTCGAGCGTGACGTACTTTATGGA
CGGTCCCCGATGAGAATTTCTAGTATGTTAGGGGCCATCCATTTATGACGTCCACAAAAAA
ATCACGTTTTTGACCCCCCTCCCCCCTGTCCACATGCGTCCACATGGGCCGGACCCCC
CTCCCCCTTGTGGACGTCCACACACGGTCGACATGAAATACACACCGCTCTCTTGAAATG
GCTAGTACGATGACCTACCACACCTAAAGCTGAAATTCGACTATATGATTCTAATTTATTTAAA
CTATACTTTTAGTAATTTATATCACTTATTTATATCGCAGAAAAATTTTCACTTTTTTTTCTGTCCG
AAGACGAAATTCTGGTAAAAAAGACGCCAACTTCTTTACATGAGAAGAAGACAGTATGATGT
CAGTGGACTCTAATTTTAATTTTCTGTGTGGACGTCCACATGGGGCTGGACCTAGCTCCCT
CCCCCCGTCCACATGAGCCTGACCCCCTCCGTGTGGACGTCATAAATGGATGGCCCCTT
ATAGGCTATTTGGTCTGCTTTCGACCGCTGTGGGTTATTTGGTTTCAGTCTTTTGTGGCGTTAT
GATTAAATAGGCCTACACGTTCTTCTATGGACCAGAGATGCAAATCGCTGTTTTAAGTAGGT
GGGGCTATCGACAGAATGCTATCTATAAGGGTTATTTAAGGGGAGGGAGGCTATTTCATGAT
TTTCTGGGGCTTATGCAATATTTAGCCCCTCCTGATAAATTCTTGGAGGGGCTATAGCCCAG
GTAGGCCCCCGGGATTTGCACCCACGCTGTGGACTAGTCTATGGTAAGCACTAATCAGTT
TACCATGCTTGAGACTTCATGTCGCTGTTTATCTCTTAACGGGGTTACTTTTGACGTGTTTTG
GCGAACAAGCACTTCCGAAATATGGCAACCATGATAGATAGCACACTCTGTGGATAGACTG
ATGTAGACCCTACTTTATTCGAACCGGGAAAACGCGAAAACCAATAAACAGGCTGTTTAGG

TTTAAAAGCGGAGGGAAGTAATCGCCGAATAAGTTTCCGATAAGAGTCGTCGCGAAATTGA
CGCTGAAGCGGCGGACAAAAGAGCGCGAGAACGCAGACGACAGCGTTATCCATCGTTT
GACGCCATTGTTATTCAATTTCCTTGGTCGTCTGCTGTTCGGCGACGGAGCAGACGATCAA
GATAAATAGTGCGGATGATGAACGAGAGGTCGCTTCGATATTCCTGCTCGACTGAGGTCCA
GGGTTGATCGGGTACGCCCATTAAGCTCTCGGTTTTTCGATGGGGTGTTGATAGCGGCCG
TGTCGATTTTCGTATTGTTCTGATCACAAAGTTGACTCTGTTCAGCCATTTTGAATTTAGATTAA
TTTGATTCCGCTTATTTAACGCCGCCGCATTGAATGCGATGGTGAGATTGGCGTCTTTTGTA
ATTGTGTACTGAATTTTGAAGGTCTTTACATGCATCCTTAACATTTAACGAGAGTGACAATACA
AAGCTGTTTACAGCGGCGTAGACTTGTCATAAATATTGGGGGGGGCAAACATTTGGCTCAC
AAATATTGGGGGGGCAAAAATTTAGGGAAAATATATTTTCAGACAACGTTCTCAAAATTTAAA
AAAAAAATATCCTTCTATTCTCAAAAATTTCTGATGACTTTTTTAGTCATCGACAACTTTTTTCAA
AAATGTACTCCCTTCATTCAAAATTTACACTCTTTTCTTTGTATTTTCTTTCTTTGTCTCTGTTTCT
GCTTTCTTTCATGTTTTTTTTTACAAAAATAAAAAAATAAAAAATTCTCCTCTGATTATTGGGGG
GGAAAAGGGGTTTTGACCCCATCTTAATTATTGGGGGTGCGTGCCCGGGCTGCCCCCCA
GAGTCTACGCCTATGGCTGTTTAACTACACTGTGCCATAACGAAGATGACTAGTGTCGCCC
TCCTCCTTTATCCAGGCTTGGGACTTGCATTGTCAGAGTGGCTGAGTTGGGAGAGAAGGC
TTGGCACTGGTGCTGGCAGAGTACGAACAGTGAGTGGCGGACGCCAAAGAATCAAGGGT
GTTGAACATGGTGTTAATTTGGACCAATAAGGCCAGCATTACCATGAGGCCAGAAATCTTTG
GATACTGAGATAACTGTAACGTAAGTTGAAATGCTATCACTAAGCGATGCAATCATACAGAC
AGATCATTTACTGCTGTTATTGAGCTACATCCATTCTTGAACACAAACATCAAAGAATTCCGT
GCAAGTAAACAAACTTTGTAAATGACAAAGAGGCATACGGATTAAAGTAGCTTAATACTTATT
ATAGTCATAGAAATGTTCAGTTTTATTACTTTCATTCAATCTAACTAATATGCTATCACATGTTAA
GTAACATGTTAATTTTGTGTTTTCCTTTATTAAGCACCTGATGGTGCCTCTAATCATGATAGAAT
TAATAATCGTAAATACCTGTTTGCGAACAGATGAATTTACATTTACAAAATAACTGTACATTTAC
AAAATAAGGTAAACTTTTTAATACACCTCTATAGAAATCTGGTGTGAACGGATAATCTAGCCA
AGAGAGGCAAGCATAAAGCCATTAAATGACGAGCCTTGTTTGTTTAAACATGTCTCCGGGC
TGCCCCCCCCAAAGTCTACGCCTATTGTAGCTTGTATAACTATGATAGTTACTTTTGATCAAA
ACAAACGCACAGCGATAAATGAATACTTTTAAGAGGTGACATAGCATAGAAAATGTAGTTGC
CGTACTTTGCATTACTTTCAAATCAACCTAATACATGCACCTGTGTTCGAGTTGTCTAGTTCC
ATTATGCTGCTATAAAATTATTAAAGGTGTTTTAACATCAACCTGCAAATTCTTTGGTTCACATG
CATCAAAGTTACCTAGACATTTTACATTTGTGCTCCCGTCTTTCGTCAATTTCAAAGTCTAATT
TCATCATGGAGGGAGTTCAAGATTCAAGCTAACATTACCACTTACCGTAACTGAAAACACTC
ACTTTTCAAATGAACTGTAATCATTTACTACTTACTTTTTGCTAAAGAAACTGTAGGTAACGGG
CATATTTAGGGGGGATTGACAGGTTCAAACCCCCCCAAATGAATAACATACTGTTAAAGAA
GAATAAAACTGTAGGAAAATAATGATCACATTCACTGCAAACCTCTGAAATCATAAAACCTCA
AACTGTGTTTCTGCCATGTTCCAGTAGGTAACTCCTGCCATTACAAGACTCCGATCAATAGA
ATGAAATTCATCTGTTTGCAAACAGGTGCACAATAAAGAAAAGCTTGAGTTACTAGAAGCAT
GTGGCATCCATAATAAAGATTCCAGGGTGCATTGTGGAAGTATGGGACAGAGGAACAACC
AGACCATGTATGCTATGTTGGATACAACAATAAATACATGTAATATAAATAATTAAAAATAATAA
CTTTTTTACATCTCTTTCCGCTTGGGATAAAATCCTTTGTGTTTAAAACAAAAAATATACTTCAT
GTAAGTCTTTTATTCACACTCAACATAATTTCTGCATTTTGTTAACAAGTGTACAGTAAAAATAC
TAACATCGGTTCTACACCGAGTAAATCATAAATAAGCGACAAAATACCAACCAACAAACTTA
TTCAAACAGCAGATAATTCAATCACAAAACAATGCAACCATTGATTTAGAAATAAACAAATCT
AATGAATGTGCTTAAGTTACAATTAAAAAAATTCAGATTCAAATTTGCGGAGAGCCTAGCGC
AAGAAGGAATTGATTGAGATGCAAGATCACACAATGCTAAACTTTACTGAAACAATGCTTTA
CACAGTTATTAAACGTTTATAATGTTTAAGCAGATTACAGATATGTACACAAATTGGGAATCAC
CTCGCTCATTCATCATTTAATCTATATATAGATTTAGATTATATATAATCTAATATAATCTATATATAA

TATATAGATTATGCCTGTGTTACGCCAGACGTGCTTAGTTATCTTTAGAAAGTGACGATCATCA
AACAATAGCAATACACTTAATCAGGCCTTCTCATTTGAGACGGGCCATTTTTGGCCCGTCTA
AATGGCCCGTCTAGCGGGGGTCTGTAAGGGCCATAAGTGGCCCGTCTAGCCGCCAAAA
AGCAAACAGGCGTCGTCTCGCTGGGCCGTTACATTACTGCACATGGGCCGTTACATTACC
GCACATGGCCGTCTACTTAATGCGGCCATTTTTGGCCCGCCTGGTATTTTCAAATGAGAAG
GCCTGCACTTAATCAGAAAAGATATTGCAATACAAATTTCATTGGTACTAAGAAACACAGTAC
AAGTAATAGCAATACTTATAGCATGAGTAACAATAGTTCCAAGTCAGATGTATTGCACGTGAT
GAACCATAAACTGTTCATAGTCCAGCGGTTCTCAACCGTTTAAGACTCAAGGACCGCTTAA
AGGAATGTGGTCTCTCGGTCGCGGAACCACCACTGAAAATTGTGCCATGGATGATTGCAA
AATTTCGTCTGTTTATATCCTTAAAAAATAAAAAAATAGCCTTTGCTCGCGGACCACCATGCA
GCTACTGTCTGCGCACCACAGGTTGAGAACCGCTGGTTTAGACAGGCACACATACACCTT
TTGTCCAGGTTTGAGCCGGGAGTGCTACAGAGTAGTTCCCTAGAACGGGCGCTATATAAAT
TTCGAATGAGTGAATGACGAACGTTGTGATTCCAGGTGTCATTTCTAAGTTTTATTGGAAAAAT
TTTTAAACGTGAGGATCCTTCTTTCTACCACGAGTCGATCTACAACCTCTGCATTAAGACAA
ATTCTGACCATGAATTCTCAAATTGAAGCCGTATTAGAACATATAGTGTTAGCTTACGTTGCTA
AGCATACGTTGTTTAAGCTAAAGTTCCATCACAGGCAATGTTCCCTCTAAGCTGCACGCGT
GCACACAGGTCGTGAGACGAGCGCACAGAAGAAATCTTGGAGCGCACATAAAATTCTCT
CCAAAAATCATTCTTGCACGCATTTCATACTCGTCATTCCAAATTCAGATGAGTTCAGGTATA
TGTTGTATGAAAGAAATTTTACGTGCATACAGACTTTAAAAAATTAGAGGGAACATTGATCAC
AGGATGCAATTTGGGCAGTGTAAACCCAACCCACCCAGACTCAAACCCACTTTAACCAGT
CTGTGCATACACGTATTATGTTACACTGGTGCAGTTCACTTGCGTCTCTTCAGTTCCTCAAAT
CTTCTTGCGAGGTCATCAAAGTCCACATCATCGCCGCCGACAGAGGCGCCACCTATGCT
GTTTGATGGGACACTTGGAAGTCCAGGCACATCAGGAAAGGACGGGATGTCGGGAAAAC
GGTTGATTTCCGTTCCATCTGCCCCAGAAGACGGTTGTCTCTGTCCTGGTTGAACTGTAGG
GTCTGAAAGAAGAAATTTCCATCACTTGCCAGCTCAATGAAGTTCAGTTAACTGCAGGAAC
AATAGTGCCTTAATAATTATTGACCACTAGGCTCAAATGAAACACATATAGTGTGAGGGGGC
TCGGTGGAGTAGTGGGATTAACGCTATCTACCGTGCAGCGACCTTGGACAAGCCGTTAAC
CTCTCATTGTCTGTAGCTTAGACAAGCGCCTGTCCGGCTGCTAGCGGTTAATTCGACTCCT
GTAAAACCTGCATAAATGTCTAAGTCTACTGTGTCTAGAAGGTCATGTTAGGGCGGACTTAG
GGCACACATTGTAAATCAGGGTGCGGTTTGACACCCTGATATTTTAAAAAAAGTGTGGGCT
CATTAGTGCAAAAGACGGATATAAGTACATTGTATCCTGTCGATATTGAATGTATATAAATGTAT
ATAATTGTAATCAAGTATTTTTAAATACACTAAGTACCTTAAGTACTAAGTAAGTAAGTACTAAGT
ACACTTAACTCAGAGGTGACCTGGGCCAGTGTCGTCACTGACAAAGATGGAGGTTTTCAG
GCACTAATATATAGCCCTTTGATGCTTAACAGATTATATGGTTTAAAAACCCGCTAATGTTAAT
ACTATATTTGAAGACAGAATTATAAATGAGCGACAAATGTTACGTACACTGATTAGGTGAACT
GACGACCTGTTCGTACGGAGGTAGGCTCTGCAAAATATAGAGCATCATAAACTGTAATAAA
GGTTATACTTATTCTAATTTAATCTAATGACTTTTACAGCACTGTTATCATAATTAATGCACAAAG
CATTAATTGATGATCATTTCAAGGAAAACAACACTAAGGCCAGGTAGGTCGAATCCAATAGG
ACTCTGAGAAACAAGAAGTTAAGCTATGGCGGCTATTAAGAAACTTGAAACAGACAAATCTT
AACTTTAAACCTGACCTACACTTAAGAATAGATCAGTGGCGGAGACCTGGCGCCGAGTTT
GGGGGTGAAAATTTTTTCGCGGACCAAGATTTCTGAATGACGTTTTTATCGTCAAAAATTCC
GTATCTTTACTATGTTAAATGTCGTATATGACCCTTTCCTCACAAGAAAAACACCTTTTTTTACT
CTGTTCATACTTTCGCGCGCATCCGACAACACTACTTCTCAAAATATTGGGGGGACGGATG
CATGACCCGTCCCCTCACCTCAAATTTTGGGGGGACCGTCCCCCCAATACCCCCTAGGT
CTTCGACCCTGGAATAGACAGAATGTAAAGAATTGTTACTATACAGTCTATACGTATTAATATTT
CTCCGTGTATCCGATACATTTTATAACTTATGCAACGACAAACGAACTTTACTTTGAAGCCTT
GTTCAAGTGAGACACAGCTTTCGTCTGAGGAAGCAAACCCAGCAATGATGTCATTGCATCT

GTGAATGATGTGAATGTTACTAAATACATATGTTGTAATATAGTCTTTCAAAGGTGCTATATAAAT
GTGAAGAGATAAACACAGATAGCTGTTTACAGAAAACATGGGTTCAAGTTCAGGGTTGTGC
AGCTTTTCAACAGAAGTGTGTGTTAGGAATTAAAACAGATACAGCATACTAGATGTACAAATT
GTGTTCTGGTTGTATAAAGTATGGAAAGTACACTGTGAGACTTAAACAAACATTCGAGCTCAC
AAAGCAGCATGCCATGCTCAAATTGAGACATATATCAGTGCTACAGTACAGAGTAATAATGC
CATTAGTAAGCAAGAAGTATGCAAGTAGTTACAGGCAAGTAGTCATCGTTAATCCATGACAT
CTGTGGATTCATCGGAGCTCGGCCAGCCGTTCCTGGTCCAGCTCTTGGTACGTCGACTG
GGTTGGCATCAGCTGAATGACTGGGCTGTGATGATGATGGAATAGCACCAGATGGTGGCA
GGTACCCATCAGGAATTGGAGGCGGGAACTGTCCCTGGAAGCGGAAAACCACATTAACT
ACTGGCAATGCCTGTGCTACAGCATATTTCTCGTGCAACTCTATCTCATCCACTGACTTTATA
AGGCATTTGTTGAAATTCAACAGATCGAAGAAGCCAAAACCCTGGCTAAGGAAATTGAAG
GAGAAATGTCTTCAGTTGAGACCTGATTAACATCAAACGCCTCTGTTTGCCCATGGCTGTTG
AGGGGCGCTATATAAATACTTAAATACAATACAATACAATACAAACATATGACTGAACAACTG
AACCATGTGGCGAATTTCAAACAGTACGGTGTTGGACAGAACAATTGCATAAACTTGTAAGA
GGTTGATCAAGTGGCAAAGGGTCGTACACCATCATCACATTACAATGTCAAACCATAAACA
GAAGCTTTCATTCCAGGTTCAACAGAGTCCCCAAGGTGTTGACTGGTTTTGTGGGCTAACC
ATGGATATCTTCTAGTACCTTTCTTTTCCACATCAACAAAAGAAAACAATGCTCAAAGCTGAG
ATAAACACACACACAAACACACACATTTAGAAGGTCGGTGCCAACTAAGGTTTTCTTGACC
ATTGTTCATGCGTTCGTCTGTTCACGCTTTGACTATTGTAACTCACTTTTAATTGGGCTCCCAA
AGACTCGGCTTGCCTCTGTGCAGTCTGTCTTAAATGCTGCAGCCAGGATGATTGCTTGTCT
GCCGCCTTATTCCCACATCTCTGACTACATCATTAAGGATCTGCACAGGTTCGGTTCCCAA
TTTTGGCTCGTGTTAGATACAAGATGCTGCTCCTAGTTGCTAAGTCCCAGCAGGGTCTAGC
ACCGAAATATCTATGTGAGCTCATGCCTAAACCGCTCGCTCCTCTCGTCCGCTGTGCTCTA
CTGATCGCTGTGATCTTCTTGTACCTTGGTCCCATACTTCTCTATCCCGGAACTGGGCCTTT
GCTGTAGTGGGTCTTGCACTCTGGAACGACACTCCTCAGCATATAAAGAAGTCTTGCATAC
ATACATTTCAGTTAACTTAAATCTGTCCCTATAAACACTAACTCCAACAAGCAACAGAAAATA
ATAAAAATATCATACATAGGCATATGGAAATTTTGACGGCGAGGAAATTTTTTACTTAAGACC
GAAAAATATCCAAAATAACTTGCATTTGTTCCACTTTTACTGGTGCCTATTCACCCAGAAAAC
AGGAATGACAAAAACTTGGAAGCAAGAACAATTTTCTCCCTATGCTCCTGCCTCCTATGCTT
ATGTCATCAGACAACCAAATCAAAAATAGTTGTGTTATCCATAATAAAAAGAAAAACTGTTATT
TGTACAATAAATCCTGATAACAGTAACACTGAACTCTTTAATAATTATTATCGTCAAAATTAAAG
AAAACCAAGTTGAATATTCACCTCTGATGGCAAGACTGGCACTCCACCAATCAGAGCTGC
ACTGTAGTCTGCATTGTTGCCTCCCGGCGCTTTGGCATTACCTCCAGCAGATCCTACTCCA
CCTGGAAAACCATTGATGCCGCCACCACCACCAGCTGCTGCATCTCCTCTACCAATATTC
TCATACAAAGGATTACCACCATTACTATTACTGTCACCACCTGTCGCTCCAAACGCAGCAC
TGCCCGCCACACCCTGACCGCTGTTGACATAACCTGCTGTACCACTGCTTACACCACCA
GCCGTTCCAAACACACCACTTGCCCCAACGCTGGTACCACCTGATGAACTATATCCAAAT
CCAGCCCCACTGGTAGTACCACCACCACCCGGCTGAGAGTAACCATATCCTCCTCCAC
CACCACCAGCTCCGGTAGTACCACCACCAGGCTGAGAGTAACCATATCCTCCTCCACCA
CCACCAGCGCCGGTAGTACCACCACCAGGCTGAGAGTGACCATATCCTCCTCCACCAC
CAGCTCCAGGAAATCCATTATTTGTACCACCTGCATTGTACCCATTGCCGCCGCCACCAC
CACCAGAGCCATACCCCGTTCCATAGCTGCCAATTGATCCAGGGATGGAAGGCAGCTGC
ATCTGACCACCTCCAGGTCCTCCTCCTCCCATCACCTGACCCGCTTCCATGGCTTTCAGT
TGAGCGGACGTTGATCCACCACTGCTGGTCCCAATGCCTGATGCATTCGGAGACAGGGC
GTCCACTCCTAGGTCAATCAGATTGCTGGCCTCTGGCGGCTGACCCTGGAAGTAACATGT
ACGTACGCATGCACTACGCAGTGAGCATACATGCATAAAAAAATTTTATACGATAGTATTGC
AGCTGTCAAAACACAGCTAAGTGCGTAAACTCAGGCAAGTCTCTAATGTAGTTACCGGTAA

ATATCCTGCACAAGAAACAACTGAAAACTCATGAAATAGTTTAGATTCCTAACCCCAAAGTC
TTTTCTATTATCCTAACCCCAAAGTCTTTTCTATTATCCTAACCCCAAAGTCTTTTCTATTATCT
GAACAGTTTTTCTATTATTCTAACCCCAATGTCTCTTTTATTATCCTAACCCCAATGTCTTTTCT
ATTATCCTAACCCCAATGTCTTTTCTATTATCCTAACCCCAATGTCTTTTCTATTATCCTAACCC
CAATGTCTCTTCTATTATCTGAACAGTTTTTCTATTATTCTAACCCCAATGTCTCTTTTATTATCC
TAACCCCAATGTCTTTTCTATTATCCTAACCCAAATGTCTTTTCTATTATCCTAACCCCAATGT
CTCTTCTATTATCCTAACCCCAATGTCCTTTCTATTATATAATGGAGGAGGTCAGAAGAAGAG
CAAGCTTCGGGTTGAACCCTTACAACTCAAAAACGTAATGTAATGTAAATGTAATGTAAAACT
CCGAAAACTTCTGAGTGCCCACAAAAGCCACATAAACGCTTGTAGCTCTTCTCCTTGACCT
GCAAGAAGTGCTTCTTTTTCTCAAGAGTTCAATCTATTAAATGCAAGGCACACTGCTAGCTTA
ACTGTACCGGATAACCCTCTGCACTCCGGGCCAAACCAGGGAGTGAGACAACTACATCA
ACTGTGGAATGTAAACATGGTGGAACCAAGCAAGCATAGTCTCGCACAAATTGAACTTTTCT
GTGCGGGAAAGGTCATTGTCAGCATGTAGCTCGAAACAGACAACTGCCAAGTGGGATCA
GGCACAGGCTAACTGGTTATAGCAAGGTTATATTATAATGCTTTTATAAAGAGATTGTATTGTAT
TGTATAGTATTTATCCATTTCTATAGCGCTTCTCACAGCCTGAACCTTTCAGAAGCGCTCCC
GACCACAGCAATTGACACTGTGTCGGAGTTTACACGCCGAAGTGCTACAGGCAATTGCAG
GTAAAGGACTTGCCCAAGGTCCCTACATGGCGGCTAGAGCGGGATTCGAACCCACGAC
CCTCCGGTCGAACGGCGTCATCTCAACCAATGCGCCACCATGTCCCACAATTAGGGATG
TTATAGATTCAGATATAACAAGATTACTTCTTACAGCCACACTGAACTCGTATTCCTGACCATT
TTCAACTAAGGCACCATTTCCTGTGTTTCATTATAAATCCATTGTACAGCAGGACTTATATCAG
GACTTATAAATTCCCCCTATTTTCGCAAAATTCTTTAATTTTCCCCCTATTTTTGTTCAATTTACA
TTTTTTGGCTAATTGATGATTTCTTGGCTTCCCCTATTTACGCATCATGCTTTACACGTACTGG
ACCTCTGAGTAGCAGTTAACAATGCACTACTTTGCATTTTTCTGTGCTACTAAGATAGCAAAG
ATTAGACATCCAGATGGTTTTACAGTAGGAACTATGGCTTTTATATTTTGCTTTTTAACCCATAA
CTCCAGGTCTTCCTTGCTCTTCATCGTCAACACTGGCGAAACATTTTACGCACATTATGAAC
TCAAAATTATCTTATGATTCCCTCTAACATTACTTACCAGCATGACTAAGGGATCTGGCTCAA
ACGGCACTTTGTACGTTCGTGCGATCTCTATCATGTAATGCTCCACAAGGATTTTCGGTGGT
GCTGCCAGACTCATCTTATGCACAACCTGAAAGAATTATCGAAAGAAAGTATTGATAATACTA
TTATTAGAAATCAACCATTACATCAGGATAAGAATCGATTTTCTAAACGTTTCTATTGTGCTTCT
AGTAAACAATTTAAATCACAAAAACTCAATACATAGAAACGAGAAAGCCCACAATGTTTGTG
TATGGTGTTGGATGCAAAGAGTTCGCTTCGTTGAATGGATTCATGGAAGAGTTGAATGGATT
CAATGTCAAACCAGATGCCAACACATTGAATGGATTCGCTAATTACTAATTAATTACCAATTA
ATGGATTCACTAATTAAAAAATTTACGAGGTACAATCGCACAATGCATATGTATAGGACTGATT
GTTGCGTGGAATCTCTTGTCTTCACTGCTTATATTTGCTACAATGTATGTTCAACTTGAGGTAA
AAGATCATAACGTTCAAAAGTAGTTCTTAGTAGTAGGCAAACGTTAACACTAACCTTTTCGTTT
ACACTTTCAAAGGTATTGCGTCTACACATCTCACCAAACTCCTTTCCATATTTGATCGTCAGC
TGGTGAGCGACCTTAAAGATAAGTATGAAATGATTGTTGTAGTTCACTCAAACCAAAGCTTC
CACTCAAATTCAGTTTTGCATATTCTCAGTATTATTTAAGGCAAAATAAAGGACAGAATAGACA
GCTCCTACTGTCACAGTGTGGCTGACTGGCTCTATGGTGAACCTCTAACAGAGCAGCATC
AGTTCAAGCCTCTCCCTGTCTGTTGCTTCTACTTTGAGAAGCTCAAACGTTTGCAGCTCTCT
TTCTTGCATTTATTTTCCTTCATGCACCGGCATAATTGTATCATTATGTAATTTATTATTCTATATA
CGTTGCTATCAATAAACCATTGATTGATTGATTGATTGATTTGGATACACAAGCCGCAAGATTT
AATGTAACATGATCGATATTACATGTTATAGTAAGTCAACACTGAACTTTCTCATCTAAGTATAA
TATAATCTGAAGTAGGTGAATTCCCCCGGTGATTTCCCCCCGGTAAACTCCCCCCGGTAAT
TTCCCCCCGGTAAATATCCCCCGGGTCAGGTTAAGTGTCGTGAGGGTCTTGTAGAGTTAGT
ATTAGGGTAGTCATTAGACCCTGTAGTCAACTGACGGGTCACTGGCTTGGCGAAAACTAAT
CTCTTCATCCGTTGTAGGAGGCTCTCAACGTCAGGGGGAAATCACCAGGGGGAAATCTCT

GGGGAGATCACCGGGGGAAGTTCACAGGGGCAAATCACCTAGACCCATATAATCAATCA
CTATCATCAAGATCAAGCCATAGTTAATCCCTGTATCTATCGTCTGTCGCTGTTGAGAGTCAA
TTATCTTAAGACTCAAGTGTCTGGTTGTCCATCTAACGTCAATCAAGTGGGCGTGGCTAACG
CCTGTCAATCCACAGCATTCAGCCAATTACGTTACACTTGTTCCTGCCTCTGAGGATATCGT
CTACATGCTTGTACAAAGTTATCCAATTGTGGCAGAAAGAAAGAGTAAGATATCTTTCTGCTA
TTGCACTCGTGCAATCAAATGATTTCTGCTACACCGTTATCAAAACAGCCAGGTAAATAAGA
AAACAACAAGAAGCTGAATTAAGTAAAAGCAAAATGTTTTAGAAGAAGATCAAAGAAGAAAT
ACTACTTCACGCATTTCTTGAACATCGCCCGAGAGACGAGGGGCAACCCAGATAAGCGTT
GCCACGGCTTCTTCGATCCCAGGATCCAATTCCCTGACAATTCAAACAGAACAGAATCAC
GGCTCAATCTAATCTGGAAACTTCTACAGCACTGTTATCACAACACACGACTGTTAAGGGT
GCTATATAAATACTCGAATACAATACATACAATACACATAGCATAACGTAATGGCTGCCTTAA
GGCAAAATACATTCCAACATCTCAATCAATTGACCAACTGTGCCATGCAGTGAAGACCCAA
GAAGCCTCTACATAAATGAACTACAGGCAGGGTTTCAGTTAATGCCAAATTGACAGTTTTTAT
GAGTGTAACAAATGTCCGAAAGCTAAAAATTTTCAGTGTGATTGTCATTGGAAATGAGTTACA
TCCAAAATAATTGGTCAGAACCCAAGTATCCGACCTATGTTCGGACTCTAAGAGAAACAGT
GGCTGCATATTAAGCAATTTTAAAAAAACATGCTTCTACTTCTGACTTAAATATTATCACAGAA
ATAGGAAGTGATGAGTGACAAAGAAGGACCCAGTTCTGGTTCGGTCTTGGACGCAAAAAG
TGCCCGAGCAACCAATGAGCGAGTGCAGCAGTTGTGAACTTCAGGTTAAGCAATCAAGTA
CAGTACTACAGTAGCTCAGAACAACATTTCTCTAAGTGGTGACAGCGACAAAATTTTGACG
CTGGATTTATATCTCTGACGAGTGAAACTGGACTTCCATCTATCTATTTATCTATCTATCTATCTA
TCTATCTATCTATCTATCTATCTATCTATCTATCTATCTATCTATCTATGGAATTTATATAGCGCCA
CTTCAAGGTAACTACTCAGAGGCGCTCCCAGCCCAGGCCCGGGCGAAAGTAAAAGTTTT
AAGGAGCTTGTAAAACGAGCTGGACAAATCCCGTGGAAGAGAGCGGATTTCCATGGCAG
AGTTCGCATCGGTGGAAGAAGAGAGTATGCGTCGAATTCAAGATGTGGGGTTGCGCAGTG
GCCTAGTGGTTAGCGTGATGGACCGCCAACCGTGAGGTCCAGGGTTCAAATCCCGGCC
AGGGCAGAAATTTGGTTCGAGATTTCTGCTCCACCTGCGCCCCCTAGCCAACTCAGCTAT
GATGAGTACACTGACCGCACACTGTCAGTGGGAAGATGAGACGGTGAGGGAGAGGACTG
GCCATCCGCCCTCATATGCCATGGCTAGGAAAATGAAGTCGCTAACACTTCTTACCCATG
GCTGCCCTTGGGCTAGCTTAAGGGACTGATCTTCTTCAAGATTATATATATATATATATTGCTTA
ATTGCTTAATATATATATATATATTGCTTAATTGCTTTATATATATATATATATATATATATGAACCTACG
ACATCTTTTCAATGAGTCCATATCTTGCAAGCAGTAGATCGCACAACATCTCAACGATCTCC
ATCGCCTCTACGAGATAATCCTCACGTATTATGTGCTCAACCTAGCAGATTGAAACGCCAA
AATCAAACCTCAACATTTCAACGTCATTAAATTCAATATAAGAGTAAATCTGGAGGCTGCATA
TGAAAAGGAATCACAAGGTACATTTTGGAGTTATTCATACCACCATGAACAACAAAAGAATTT
TAAAAAAATTATGTATGGTAAACCGTTAGGGACATAGCCATGAAAGATGTTGGTGGGGGGTT
ATATTTCCGGGGTTCTCCATTGAATTTTTTTGTATTTTTCTACGTTTTCAGGCTTTTATAACAGC
GACGGATCTATTTGGGGTTCTGAACCCATAAGTCCCCCATTAACTACGCCCATGATTGGTG
TATTATGACATTTTGGTAGAATTTAGAACATGCCGTACATGAAAGATGAGATTTTCAGTAAAAC
TCATTTAAAGTTAAGTACCAAATTGCTCACATTTGACAATGTATACAATTTCACTTTGCAAAAGT
AAAGACAAAATAATACTCCCATTGTAAAACATTAAACATCATTGTCTTCAGAATTTGTTGCTGA
TCATAGCTAGTAGTATGAGTAACTAGCTATGATAGTACTATTTTCAGGTTGATACCTCAAGAATA
GTGATGACGCCCTGTGGACTATCAAACATGAAATTATTTGAAAATTTTAAAAAACTTGAACTG
CTCTATTTGCACAAAGTACTGTGCTAAATTTATAATGCCAGTGAAAACTTAATTTCAGTACTTT
GATGGGTGCCCATGATCAAAGGAATGTAAAAAAATCATCCCATGTTGTTTTCCTTCTCATATG
AATCATCACAACTATTCATACACATACCATTACGGATGTGTGTGCATATGTATGTGTCTATGTAT
GTATGGTTGTATGTACATGTGTGCGTATGTATATAAGTGTGTATGTATATGCGTGCATGTATACAT
AAGTGTGCATGTATGTATGTGTAAGTGTATATATAAAAGCCCGAGACAGATGATGGGTGACC

GACAGTAACTGAGAATATGCATATCTGCCAGGAAGTGCTCCCATCCCAGACTCAACAGAA
GACCGTCACTAAGTGTCAATAGGAGTACATGTAATGTAAATCTCTAGAACTTATCGGCTTCAA
GGAGATGCCTATTTGCTGGTGTATCCATATTGTTATCCAGCCTCAATCTCTTCGGATCCTGAC
CAGGCACTGAGAAAGACCGAAGCAATTGGACATTTGATTTGATAAGTGGGTAAGATGGCTA
ACGTGGTCTCCTCTACGTCATTTCTTACGTGGCTCTGAACAGTTCGGGAGATTCACTTATAC
TCATAAGATGATGCTACAAAGGAACTGCCCCGAAGAGGAGTACGCTCAAAAGGACCAAC
CCATATACATAGCATGTCATAGCTGTTATACACAGTAGGTTTTCAATACTGTAAAGAAACAAAA
AAACTTAAGAAAGCATTTGAGAAGACTTACTCGTATTCTCGCCCGATCATCCTTGCCTGCCA
GGATGTAGTCGGCAATTTCTCGTCTGGCTTTTAGGGCAAGTTCAGCTACAGAAGCAGATCA
AAACATAGTTTCAGTAAACAATGCAACAGATTACAAAAAGGAATTACAACAGTTGTAGTAACT
AGTAAGAAACAATAATTTATGTAGTGCTGTGTCAAGAAGGAATCCCCATCGGTGTTCATATTA
ATCACCATATATATCAGGGCATATTTTAAGAAAGGTTTACAACTGCAGAAAACGGAAAAAATT
TCCGACCTCCAAACATTTGAAAAAAAGTTTTTCATTTCCACAAAAATTTTTGACGATAGCACA
GCTTAGAGGGAGCATTGATCATGAGTGGAATGTAGCAGAGGAGAGTGCAATGTGAGGAAG
TGTGAAATTAAGAGGTGGTTATGGGTATATAAGCAATATAGAGATCACTATAAAGACTCGTGG
ATCGAAAAATACAAAGAAATATTGCCAAACTGACTTACTCTTTTTCTTCTCAAGCAGTTTCAGT
CGATTGATGCACAGACGCAGATTTGTCCGCAACTTCAAGTAGTTTGGTTTACTAGCGAACAT
CTTGCCTCAGTCTCTAGAATGTTATCACATCGCCAGGATTGTATTCATTCATTCATTCAGGCT
ATTTCTATAGCACCTCTTCAAGTCCACTACTACTCAGAGGCACTCCCGACACAGCACGGAT
ACTGTGTCGGAGTTTAACGCCAAAGCGCCACAGGCAACTGAGAGCGAAGGACTTGCCTA
AGCTACCTACGTGGCGGCTACAGCGGGAGTCGAACCCGCGACCCTTCGGACGATAGG
CGCAGACTCGACCAATGAGCCACCACACCCCACCGATATGCTGTTGCAAATTTATATTATA
CCACATTGATTAAAGATAAATAATAATCTGACTAATTATGAAAATTCATAGACGATCTAAAATTT
CATTTCATCTCATTAGGTGAGTTTGAACAGAAAGAGTCCATGAATCTAATTTAAAATTTCCATT
ATCACAGTTTCATTGCATATTTTTCAATGGAATTAAGTATCTTCATCTCAGTGCATTTTGCTGTG
AATTGACTGCCTACATTCAATGAATAAATATGCTTCAATGAATTCAATGAATAAACATGCATATT
TATCACATTGTAACAATCTAAAATAAATTTAACTTGAGTTTCCGTGAGTTATGATTCTGGTCGTA
TTCCTATGGCCTCAATCCTCTGCTGCTGGAAGCTTTTCGTATTTCCTCAGGACTGTAGCACT
GAGTGCACCCCTGAATAGCTTCACTCTAACATCGTAATCGTAAACTCCAAACTTCTAAAGC
GCCACTCAAAAGTCAAGCGCAGGGCACCAGCTTATTCACGAGCGCTGCGTCAAATCAGA
GAGGTTTTCCAAAGAATAGTCCGTGGGAGGCTCAGGTCCGTTTTCCAGAGGGTGAGAGAA
TGCGTCAAATCAGAGGTGTTTTCCAAAGAATAGTCCGTGGGAAGCTCAGGTCCGGTTTCC
GGAAGGTGAGAGGAGGCAGATTACGCGTTAAGGCGGGTGTAGTTTAGGAGAGGAGTGGG
AAGAAATAGAATCAGGTGAGTCAGGGAAAGAGAGCTTGAAGAGGTGGAACTTCAGTTTGG
AGTGGAAAGCCCGGGGGGTGACAGACAGAGGAGCGTGCTGAAGATGACGTTTTATGATTT
GGAGTGATGATGATGGTGGAGGTAAAGAAAAGGTGCGGATTTCAGGTGGCAGGTCATTCC
AGAGACGCGATATAGCTCTTTTGGAGAACGTGAGGTGAGAGGTGACCGGGGGTCGAGAA
AGGGTGAGACAGGAGAATGATCGGGTAGAGTGGGTTGGCTGAATGGTGAAAAGCTCGCA
AAGGTAAGTGGGCTGGGAGGACTGGAGGGAGTTGTAGGTTAGAGACAGGACTTTGAAGTG
GATGCACTCTGGGATTTTTAGCCAGTGAAGTGATTTAAGGACAGGAGTGATATGATGATGCC
GGGCGTTCTGGTGACAGCACGTGTGAGTGAGTTTTGGATGAGCTGTAGACGCTGTATTTGG
GTGGAGTCGAGGTTGAGAAAGAGGGAGTTGCAGTAGTCTAGTTTTGAGTGGACGATGGAG
GTGGCAATGGTGGATGCAGTTTTAAAGTCAAGCATGGGTCGGATACGGCGGAGATCGCGT
ATGTGCATGAAGCAGGAGCGGGAGAGGTTGGAGATGTGGTTGGAGAATGAGAGATGAGG
ATCAAAAGTAAGATCAAGATTGTGGACAGGAGCATCAGAGATGAAAGTAGTGGAAGAGGA
GTTATTGGACAATCGTATGGAAGAGTCGGGGATTTTCTTGATTTGGGCAGGTAGGCCGATTA
TGATGGCACTTTATAAATTCCCAGCTACAATACGAGTTTCCCAATGCTGTAGCTTTGCTGTTG

TAAATGCTACCTAGTATTCACAATGTCTTTGGGTTTAGCGAAAACTAACAACATTAAGGCAGT
GGATTCTGTGGACAGGGATGAGATTTCTTGGGATTTCAATCTCAATAGAAGGTTTGAAGTGT
GGTTAGATAGTTATGCTCACGTTACCTGACACTGCATAGAAAACAGGTGATGGTGAAACGG
AGATTTTCCTAAACTAGCAATACGGATCGGTAAGAGAGCCAGGCCTTCTCATTTGAGATGG
GCCATTTATGGCCCGGCTAAATGGCCCGTCTAGCGCGGGTCTGTACGGGCTATAAGTGG
CCCGCCTAGTCGCCAAACAGCAAACAGGCGTCGCCCTGTTTTTCCGATACATTACTGCAA
AAGGCACGTCTACGGCCCGCCAAAGTAATGCGACACGGGCCATTTCTGACCCGCCTGG
TATTTGCAAATGAGAAGGCTTGAAGAGAGCTGTTGGTGACTGAAAAATTTAAAGCTGAATGT
GCTACGAGATTTCCGCCTGACATGCATGCGATTTTAGAAAAGGTAAGCCTATTTCCGAGTTT
CAAGCTTGTAAATAATTATATTCCACCTTTGTCGCTCAAATCACCGACCCGACTTCTACTGAA
CAGGACCCATTGGTTGGTTGGTCACAAAAAATAGAAACTGCGATGGATGATGATTAACTTAC
TCAGAATATCGGAATACGTTTTACTCAATCCAAGTAAAAATTACAACATTGTCCAGTATCCTG
CGCAACCGTAGAGAGCTGATTCAGTGATTAATAACTTAGTAACATAAAAATATACTAAATGTTG
ACAAACATCCACGTGACTGTATCTATTCGCTAGATGGCAGTAGCATTATCACATTCACATCG
AAAGGATGTTCGACAACGTAAACGTTGCGTAGAGCCGAAGGCCTAGCCTACAAGGCCTT
GGGCCTACACTGTGCACTGTGCAGGGCTGTCGAGACCCATTTTAAAAAACCTAGGTTTTTT
AGGTTTTTAAAAAACCTAAAAAACCTGAAAAGTTAGGTTTTTTAGGTTTTTTGATTTTCAAGTCA
AATTTTCTACTTTTTCATGTCAAACTCTGTAAATTTCTATGAATTCATTGGATTTAATTATAACGTT
CAACATAGAAGTGCATGAGTTAGTCTACGTGTATAGCTTTTTAGTTGATCGTATTCTTGTGTCC
AAAGTTCCGCGTGCGACTGTATGTAGACATTAAAACCTAAATACCTAAAGAAACCTAAAAAC
CTAAAAAACCTAAAAATCTAAAAACTTTTTTTAAAAAAACCTTAGGTTTTTACCAGCCCTGAC
ACTGTACCTGTCAGCTTAGTTTATAAACCAAATCTGATTGATCACGTTCTTAAGTTTTTCATTTA
CCTAAATTCATGGGCGGAGACCTGGCGCCGAATTTAGGGGACGGAAAAGCGACCTTTTT
ACCAAAAATGTGTATTTATCCACCCAAATTTCCGAGTGACCTTTTTTTGTCACTGCACAAACA
GGCTTTCATCATTGCACATTTCGATTCACCACTGCACATTTTGCGCATCACTTCACGTTAAA
ACAAGCCCTGATAGTTGCTTTTAGGCCCACCGATTGGTCTCCCGAGTAACTGTTAATAACT
GGTAGTGGTAGGGGCCTAGGACTCAGAACTAAACAGCAGCAGCTCCTCAAAAATTTGACC
ACCCACTCCTGACGTCAGTGTATCCACATTTGACTTATTCATCTAGGCCTAGGCCTACCTAG
TCCTAGTGTGCGTGACTTGAGTGTGGCCCTTGATGGCACGCTGTAGTTCTTCTTGTCGGCC
TATCTCTCGCAGTTGTCGAGCTCATGTTACTAGCCTATTATGGATCTAAAATCGAGAGGTGCA
CCGACTAAACGGCCATAGTATCGTGCAATCGGGTCGATTAGCCGTTAGCCTAGCGACTTA
AAAAATAATAAGCGGCACCTATGTCAAAAAGATTTTTCCTTAGTTTTGGCGTTTTGCCACTCG
CCAGATGGCAGATGATTTAAATCGCAACACCAAAAGAAAGATGATCAACGATGCGTAGAA
GCACCAGGAGGAATCTAGAATTCTAGATGGATTGATGGAGCCAAACAGGCCCTACGTCAA
CAGCAGGCCGTCCATAAAGTAGGCCTACGTCGCGCTATTTTGGACCAATTTTGACCCCTT
CCCTCCCCCTATCACACTTCGTCACACATCTCGGGACCCCCATGAAGTACGTCACACATA
TCAGACCCCAATTTTTAGTAGGCCTAGTAAAAAAATCCGGACAAAACCCCCTGTAAAAAAC
CTATCTGAATTGTTCGCGGGGGCTTTTGTCCGGGGGTTTTGTCCGAAGTTTTTTGTTTGGTAA
GTTTTGTCCATGGTGGTTTTGTCTGTTCCCTACTGTCAGAAAACACCCGTTGCGTTACTACA
AGAAAGATAAACATCACCTTCAATTTCAGGTTTCATATGTATAGGCCTATGATAAAAAAATTTA
ACGGGTGACGTAACATGCTCTTGACCCCTCCCCCATTCACAAACTGTCTCACCTTCTCTCT
CCCCTCGAGTGTGACGTACTTTATCGGTACAGCTCTACTGGCACTGTTGCCTGAATAGAGT
AGGCCTAGGCCTACAATTCGTTCATGAACGCCGGAAGTCCGGAACCGCCCGAGGAACG
AAAGGGGTGGAGACACAACCTGCGCCTATAGTACCCCTCACTAGGGCTGGGAAAGGCT
GAAGCACCCCCGTCACTCCTCTTTATCCAGGAACAGATTGACCGGAATTCAGAGTGAATG
AAATCCAAATAACTTATGTTCTTTGAAAATGACAACCATTTTGGACGCCATACTGGATTTCTAA
AAGTGTTCCAGATGGATTATCGGGTACTTTTAGTTTGCTACTCTCCTTAGTCAATTCTTCATAAT

CTGCATTAGTATGACCGTTGATTTACATGTAACTTTCTTCCTGTTTTTCAAGCTATGCAACCTA
CTCATAGGCGTCTGTTTTAGTTAGACTACTCCAGTAGGACGCTCTATACCGCCTCAGAAAC
ATGATCTGCCCACAACGTCGTAGGCCTACGAGATCGCGGGACCAGCAGATCCCCATTGC
CTGACAATAAGCTTACATAAGAACAAATTCTTAAACTGCGGGTGAATAAAACTTCTTTATTCTG
ATCTACTGACCCTGAATTAAGATGTTATTAATTTACAAACTGAATGATTTGTCAACAGAATATTG
ATGTCCGAGCGAGAGAATGAGGGCGAGGGAGATCGAGAGAGACTATGACGTTAGAAGAA
CCAATTCTTCTTTCAACTCCTCCGGTCGAATTCGTCATCTTAGCCATCTGAGGGTGCGGAG
CTGAGCACAGGAACAAAACCACAGAGGGAAATATTACCTAAACACACGTAATTCAAACTC
GTAGGCTAATATATTATAGTCTATCTTCATTTCATCTTATCCTAAGCAATTAATCTAAACCTAACT
TAGGCTGATCTAACTATGCAAGCCATAGCTTAGGCCTCTATATCACAATTTAAATCAAATCTA
ACGAAACCTAGCCAAAAATAACGTGACACAACTTAACGTAAATTGACGTAACGTAGGCTAA
AACATAGCCTACCGTAATTCGTCCTAAGCCTGACCTTATAGTCCATTCTAAGTTATTCAGTCA
TTATAGCGCCACTCACTCGTGTAATAGGCCTACTCTTAGCTATCAATATATTTGGAGAGAGGA
TATATTCGAAGCAAACTCGAGTGGACAGCGGCGGACAATGAGATATTAATGGCTCTATTTC
CTTGTCCCGGCGTATGGGAGCAAGTGGTCACTCTCTCCTCACAGTCTGATCTTCGCACATA
CAGTGTGCAGTAGATGTATTCATTGTACAAGTGACAGAACTAGAGGTCCAGTCTAACTATCC
ATTTTCAGGCTGTTTCGATCTGGTAACTGTAGGCTTACTCGAGTTTTCCTTTTAGGCCTACCA
TCTAAGGACATTATCATGCAACGCTTACGATAATAAGCTGGTTCTATGCGGATAGCGTAGGA
GTAGCGCTATATAAAATCGCAATGTACTATTATGAATACGGCCTGGTGTGAAGTAATCCTTCT
AACCTGCCCTTTTTAGCTGCGAAACGACGGTAGACTTCGATGTCCTCGTCTGTTACGGATC
TTCGAGCGCATTTCATGGCTTCGTCGAAGTGACGCTTCGATATGAAAGGCACAGGGTCAC
AGTTGACATCTCGCTGAAAGGCACAATCAGAGATAAACAGTGACTGCCACAGCGTTCTTCA
CAGGCCATGAATGAGCTACATAAATCAACCGTCATTTAAACGAACCAGGATTTTTAAATATAT
TGCCACACAGACAACAGGTTCATAGAACTAGAGCCCTGCGCCAACAAATTGTCGGCGGC
GGCGGCCGGCTCTACATAGAACTGCTGCATATGATATGGTTTGTTGAACACATTTCTCCTCA
AAACAAAAGCAGTCAACAAGTGATGTGTTATTTGTTGAATACAATCTGTTAAATACGTTGCATC
ATTTCTTCAGAGACGAGTATCATACTATAATCTTTCCACATAAAGATTCGCCCGCCTGACATA
ATTTCTTCTTGACCTAATCTAATGTTTCCGCAAAGCAGCCTATCATAAGCTCTACGCGTACTG
TAACATCATTCCCGCTATACACGTACACACTAAACGCAACCCATACCAAGTGGTAAGCTTA
GGCTTACTTCGGCTTGTCCATCAGGTGGATGGTTTAAGCTAAGCGATAATAATAGTAACCTTT
GAAGCTTGCTATGAAGCAAGCTATCTAGGGTTACTACCAGAGCTGCCATATGGCTCTCTCC
ACAATGCCGGACACTGGCAGTGTCCGGGTCTGCGGCACAAAATATTGAGCTATTTCGTGC
AATATCCGGTATTAACATCATTTTTATACAGGACAAAGAGCCAAAATACCGGACAGTCCGGT
TGAATACCGGACACCTGGCAACCCTGTATACACGGAGGGATACATGCAGTATCGATTCTG
CAGAACATTGCACGATTTCAGCCTAACACTTTGGGTCCCTTTACGAGATGGTTTCTGCTGC
ATATGAGCACATAGACTATAATAGTTATTATAATAATATATAATTATATACATATGCATGTAAGAGC
TTATTTCAACTACTTATAGTGATATGTTTCCATTGCCATTCGAATAATACAAGGTCTATTGGCAA
AGACATCAACTCTTAGTTTGGGCGACGTCTGATGTCGCGTGATGCAAGGGATGATCTTACG
AGGAAGGATGTGAAAATGTGATATTGACGTAATAAGCCTTAGTCAAAAGGAGCAAATTCAGC
TCTAGAGCAGTGGTAGCAGGAGGGAGGGTAGAACGCAGGACATATCTTAATGTGCAGTTG
ATCACAATTTGTGCAGTTATAAAATCTAAATGTGCAGTGTAAAACGTGCAGTTGTTGCATTGTC
GATTGGCCGATTGCCAGGCTTTTTGCAGTTGTAAATCCTGATTGAAACATGCCCTGGTAGA
AGGCATGTACTTGGGTCGCCTAACGACCATTTATCTGGACATAAAATGGTTTATTTTAAGTGA
ATGTCCAGTACATATAGCATAAGCTGTCAATACACGAGCAGCAATTAAATGCCTTCGGCATA
GAGTCAACTTAGGAAGACGATTATCGTCTCATTTTGATGCAAGGCTGGAGATGGCTAAGCC
AACGTTTGTGGGTTCGTTTCCAGAGCCTTGCTTTAATGGTATGCTGCGTGTTTAGGGGAGAT
GGCATTTAGCAAGGATTAGTTTAGATGATGTTCATATGGGGAAGACTGATAGAATATGCATGT

GTTGGTTGTGCGTTCCGATTACGGCCCGCACTCGGGAGGAAGGTGGTTTATTTACGAGAC
TTCAAATCTCGAGGTGGCACGGTCCCACCCTGAGACCTTAAACGGAGCAGCACCAAATG
TAACTGGGGAACGGCGGTGATGGACCTCACCTGAGTAGGGTAGAGGTCATAGGGTCGTA
GTCCCAAACGTGTGCAACTGTGCATTAAACATGAAGATGCACCACTTGGATCTTTCCTACC
ATTTCGGAGTCTCCGTCTTCCGGGTCCAGGTTCTTCCTGTCCAGCTCGCACTGGATGCAC
TCCCTTATCGCCGCCTTGCACGCTCTCTGGCAGATCTCTTTTATATCGGCTCCACTGAACC
CTTCTGTATCAATTGCCATACGCCCCAGCTCAACATCCTTATGTATAACACACACATACAGA
AACATGTAGGCCTATGCATACATACATACATACATACATACATACATACATACATACATA
CATACATACATGCATGCATGCATGCATACATACATACATACATACATACATACATACATA
CATACATACATACATGCACATAGTGCACACGCATACATACATACATACATACATACATA
CATACATACATACATACATACATACATACATACATACATACAGCAAGTATACATCATAAAT
CGATACACAGACATAAAGGGCAAATAAGAAAAATTAATATTTTACCGGATTTAATAATAATTAT
CAGTAAATATAAAGAAACATATGATCACAGCTGGCACCATTCAAGTCTTCCGACTTCTAATGT
TAGTAGCAACAGTAGAAGTCAGCGGCGTCAATCTGGTTTGAAATCTGGGGGGGTCGTGGA
CCCAGGTAAAAAAAATTCGATTTTCTAGAAAAATTTCCGAAAAATTTCGATTCCTTCAGGCAA
TTTCACAAACAAAAAATCGACTTTTCAGGGCAAATTTCCGAAAAGTTTGGTTTTTTCAGGTAA
TTTCACCAAAAATTCCGATTTTTCAAGTTAAATTTCCGAAGAATTTCGATTTCTTTAGGCAATTT
CCCAAAATATTTCGATTTTCCTAGGGAAATTTCTGAAAAAATTCGATTTTTCAGGCAATTTCAC
AAAAAAATCGATTTTTCAAGGCAAATTTAAGAAGAATTTCGATTTTTAGGCAATTTCACAAAAA
AAATCGATTTTCTAGGAAAATTTCCCAAAAATTTCGATTTTCTTTCAGGCAATTTCACAAAAAA
GTCGATTTTTCAGGGCAAAGTTGCGAAAAATTTCGATTTTTGTCAGGTAATGTTATACAAAATT
CCGATTTTTCATGGCAAATTTCCGAAAAATATCGATTTTCCAGGCAAAAATTGGATATTTACA
GCTATTTCTGGGCAAATTATTCTATTTCTCATCAAAAGTCACCACTTTCGAACATACTTCCTGT
ACATGATAAGATAATAATAATATTTTACGACCTGTCCACGACCCCCCTGCGACCCCCCTGA
CCAAAATCTGGAGGGTCGCGACTCCCCAACCCCCCAGGATTGACGCCTATGGTAGAAGT
AGCAGAAGCAATATTAGTAGTGTTAGTATTAATAGCAGTAGAAGTAATGTAATATAAATAGTAAT
ATTTATAACAATGATTTTAATAATTTATAGTAATAATAATGACATCGAAAGGGTGAAAATATTACG
ATGTACCATTCGACATACACTATATAATTAATATTTTAGTCCCTGAGGTTATATATAGGCCTAAT
AATAATAATAATAATAATAATAATAATAATAATAATAATAATAATAATAATAATAATAATAATAATA
ATAATAATAATAATAATAATAATAATAATAATAATAATAATAATAATAATAATAATAATAATAATTA
TAATTTAATACTAATAGCAATAATAAGTTCATGTTACTTTATCGACGGGTGACTTTCGAAGAGTT
GCCCGAAATATCATTAGTCGAGATTTCAGATCCGGAAGGGGGATGTATACCATCTGATCCA
AGCGCCCAGGGCGAAGAATTGCTGAATCGATGACGTCCGGCCTAGAAAATGATCACGTG
GGTTCAAGTAGCCAGAGGATTTAGGGTAAGATAAACAAGGTGGGTCAAATTACTCCTCGAT
AGCCAAGGCAATAGAAGGTGAGTACAGATAGCTAAGGTTAAACCTATGGGCAAGCTCAGG
TGAGTAAGAGTAACAACATAATCTTAATACAAATTTATTTGTCGCTTATTGTAACTGAACTCCTC
TGTTATTAAAAAACAAGATTACAATTAGACGCGTGTAATGTAGCATAGGCGGAGCCTTGGCG
CCGAGTTAGGGGGGACTAAAAAATTTTTCGCGGACCAAATTTTCGAATGACTTTTTTAGAAC
AAAATTTACATTTTAACGCTCAAAATTTCTTATGACCTTTTTTTTAGTCGTCGACCGTCTTTTGTT
TGTCTCTACTGTCTGAAATCTGATACCATATATCCATATTATAGGCTATTATATGACCCTTTTCTT
AAGAAAAAGCCTCTAGTTCACAAAAAATCCTTCATCTCACCATTTTTTAGTCACTTCGTACTT
GTCACGCGCGTTCCTTAACACTATCTCTTCTCCAAATATATTGGGGGGGGGCGGATACATG
TGTCGTCCCCCACCTCAAATTTTGGGGATCGTTCGTCCCCCAATCCCCTGTAAGTCTCCG
CCCATGAATGTATGACATAATATATCATTAATTATTGTGATATACGAAATGTTGTGTATATAATGTA
AAGTAATATAATACGCCTAATATTATATGCAGTGCAATGGTGGCAGAGTAATGGTCGACGCAT
AGGTCAATAGACTGTACGTAATGTAATGCTGGTAATAATGCTGCATGCATAACGTAGTGTAAT
ACACGCAATGTAGTAAAGCAATGAGGTGCAATGCACATAATGTAATGCGCGTAATGCCATT

CGATGCTACATGTACATGTACATACGTGATGTAATGCAATGACATACAATATAATGTAGGCTTA
AGTAATGCAACGCAAGTCAATTAGGCTAAGTAATGTAATGCAGTTTCGGGATTAGAATTGTCA
TGCGGTATCCTCCTGCATGTCGTAAGAGGCGACTAAAAGGAGGGGGTGCGTGGTAGTCTA
GTGGTAACGTTCGCGGCCGCTAGATGCCAAGGTCCGAGGTTCTACCCCGGCCGCTCTT
GAGAGGTAAGCTTACTTAAAATTTGGATCGAGATTTCTGCTCCATGCGCACCACTGTTCCG
CCTCTGGGACCACAACATCGGGTACCAGAGCCAGTCCCAAACCTGGAAACTCACCTACA
TGTAGAAGGTGAGTAAGTGAAGGGTCGACCGAATGGGTGCCGATACGTCGAAAGAAGAAA
TACGAATGAAATCCAATGGCAGGCGAAGAACAGTGGGTGGACTAACACCGAGATGTGGA
GGGCAAAGGGAAGAGCGATGGACACCAACACGAGCGAAAGCCCAGGATACCAGAGCC
AGTCCTAATAGTCTGGAAACTCACCTAGTGAGGAAGTGAGGGGACATGGGACACCTAATG
TGCGTAATGCAATGTAACGTACGCAATGTATTGTGCGTAATGCAATGTAACGTACGCAATGTA
TTGTGCGTAATGTGATTACGACGTATGTCGGCAAGGTTGTACAGAGACTGTTGAATGAGGAC
TCATACTGTATACTTAATTTGATCTATGTCTTGTTAGAAGATGAGAAACACACACCTGTTCGTG
GCGCCAATAACGAAGACGTTTTTCGATGGGCTGACTCCATCCATTTCTGTCAGCAGCTGGT
TGATGACGCGATCAGCCGCAGGACCTTCATACCACAACAGATGACTCACAGCTTAATAATT
TAGATGCTCGGGTTTCAGTGACGTGCAGAGGGGGTGCGTACAGGAAGTATGTTCGAAAGT
GGTGATTTTTCAAGGAAAATAGAATAATTTGCCCAGAAGAAGCTGTAAATGGGCAAGTTTTG
CCTGGAAAATCGATTTTTTGTGAAATTGCCTGGAAAAATCGAATTATTTTTGACCCGGATCCA
CGACCCCATCAGATTTCAAACCAGATTGACGCCGCTGTTTGGTATGAAGTGTTGGCGACTT
CATTTTCTTAACCTCTGCATATGAGGGTAGGTGTCCAGTCTTCTCTATTGTAGTCTCTGTATAT
GAACGAACAGTGAGTACTGAGCTTTGCAACCTGCTGATTCCTGTGACTTCTCGCCTCTGGA
CACAGTGTAAACCTGGAGGAAACGACGATTATTATTACGACTAATAAAATCATTATAATTGTTG
TTGTTGTCTTCGTAATTGTTGTTATTGTGGTCTTTGTCAAAAAGCTGATAAATCGGTTAAAGCAT
AGGTCAGAAGACATTTCTCCTGTATGCTGAGTAGAACGTTTATGGCTGGTGTGTATGAGAGA
GTGTGTGCGTGGGTGTAGAACTGGCTTGAAGTAGGCATGTATATACCTACATGTCAATTTGTT
TCCGCGCTTCATTGAAATGGAGTCCAGTTCGTCGAAGAAGAGTACGCAAGGGGAGGCAG
ACCGTGCCTATAGTAACAGCAGATGCCACACTGTACATAACTATTAGCAAACACACACACA
CACACAGACAAACACACAGACAAACACACAGACAAACACACACACAAACACACAGACA
AACACACAGACAAACACACAGACAAACACACAGACAAACACACAGACAAACACACAGA
CAAACACACAGACAAACACACAGAATCTAGCGCACAACGGAATCTACCAGAGTAACCAG
AGCATTCAATGTGCCTCCAGCATGCACATCAAGTCCTGACGCGTAACACCTAAGCCACCT
GAGAGGTAGCACAGCCATGTGGTCGGGTCAACTCCTTCATACCTCCAGACCCTATGCACT
TGTGTCTCTTCATTTACCGTGGCTTTGTCCCTCCACTTGGACTTGCCTGCCTTTCTTCCACG
GCTAAGCTCCTGGCTGGGATTTCAACACATCCTCTCACACTCTCAAGACATTGATCCCAAC
ACTTCCGCTAGGAAAGCACCTCTTAGTAGTCTATACTGATAGATGCGCTATATAACTTCATTTA
CGATAGTACGACGACGATAACGTTTGGTGTTGTTAATGATGATGATAATGGCGTCGTTGATTT
TGGTCTTTTCATTAACTAATTAATCAGACAATGTGGGGCGGAGTGGCTCATTGGAAGATTCG
TCGCCTTTCGTCCGAAGGGTCGTGCATTAGAGTTCCGCTCTAGCCGCAACTAAAGACTTT
GGATTAGTCCTTCACTCGCAGTGGCGCTTCGACGTGGTAACTCCTTACGGTAGGCGAACT
TGGTTCGCCAGCACAAACGCACAAGAATGTTTCCCACTCTCTACGGCTCCCATGAATATAT
CATTGTGGGACGTGGCGGCGCACTGGTAGAGTCAAAGCCTTTCGACCGCAGGGTTCGA
GTCCCGTTCTCTAGCCGCCACGTTGGGACCTTGGGCAAGTCTTTAAGTCGCAGTTGACTG
TGGCGTGGTAACTCCGACACAGTATCCGTGCTGTTTCGGGAGCGCCTCTAAGTAGTAGTG
GACTTGAAGACAATGAAGAGGCGCTATAGAAATAGCCTGAATGAATGAATGAATGAATAATC
GATCGGACCATATACAGGGTGGGCCAAAAAAGTAAAAACGTTTTAAATAAAATGTTACCTAC
TTAAAGGTAATTCATATCATAATACGAAAGTATGAATTACCTTTAAGTATGTAAAATTTCATTTAA
AACTTTTTACGGTTTGTTGAAATATTGGCAAAATATCCTTAGGGGTTACATTTTTATGGCCCAC

CCTGTGGAAGAGAAGAGATGCCGAGAGATGGCGATGGCTGACCTTGGCGAAGATATCTCT
GACGTTCGACTCCGATTCTCCAAACCACATCGATAACAGTTCCGGTCCCTTGATGGAAATG
AAGTTGGCCTGGCACTGGGTGGCAATGGCCTTCGCCAGCAGGGTCTTGCCGCACCCG
GGGGGACCGTAGAAAAGCACGCCCTTCGACGGAGGCAAGCCATATTTGGTGAACAGAT
CAGGATGTTCGACGGGGTACTGAACAATCTCCAGTAGTTCTTTCTTCACTCCCTCGAGGCC
CCCGATGTCGTCCCAGGTGACGTTCGGTATCTCGACGATCGTCTCACGGAGAGTGCTCG
GGTGGAGCTCAGTCAGAGCAGCCTGTATCAGACACGCACACGCAGACGAGCGTCTTCA
AGTACGCAAGAAAGAAACAGAACATTGTCCTAAGCGACAGCGGCGTCAATCTGGTTTAAA
ATCTGGGAGGGGCGTGGCTTCGGGTAAAACAAATTCAATTTTTCGAGGCAAATTGCCGAA
AAAAATTCGATTTTTTCAGGCAATTTAAAAAACTTTCAATATTCCAGGCAGAAATTACATTTAC
AGCTACTTCTGGCCAAATTATTCTCTTCAAAAGTCACGACTTACGAACACACTTCCCGTACA
TGATAATATATAATAATATTTCACGACCCCCACTCCCCCCTTCCCAAAATCTGCGGGGGGT
CCGCGACCCCCCAAACCCCTAGGATTACGCCTCTGCTAAGCGAGATCTTAGAAGCAGC
CACGGGTGTGAGTCATTTGCCAGTTCATTCTTCATATCATCAAGATAATGCTTTTGTGGCCAA
TCCCCTCCCTAAATTAGGTGCCCAGTCCTCTAAAGGGTGGTCAGTCCCCTCCCTAGATATA
GTGGCCAGTCCCCTACATAAAGTGGGGGCCAGTCATTTCGTCTAAGTCAATGGCAAAATTT
CCCAACAGATTTCGCTCGTGAATAAGCTGGTGCCCTGGTGCGCTTAGCTTTTAAGTAGCG
CTTTAGAAATCTTGAGATTACGATTACGATTACGATTACGATTACGATTACAGGTTCTCTGCAG
AGGCGCAATATAAACCTTCGACAACAGGAGCACTCCTACTGACCATGAAGTTGCTCTGCG
TGACGGCCAGTGACGCGAGGAACTTGTCATCGATCGTGTCATCGTCCAGATCGAGGCCTT
TTATCTTCTCCTCGATCTGCTTATGAACAGCCTTCGAACACAACGATGCCAGGTCGGCTCC
CACGAAACCATGCGTCTCGTTAGAAATCTGCAGCAGAAGCCATCCATAATGCTTTATACATT
GGCTAGTGGACTCCGCGAAGAAGAATACGAAGAAGAAGAATGCGAAGAAGATGAATACG
AAGAAGAAGAATGCGAAGAAGAATGCGAAGAACAATGCGAAGAACAATGCGAAGAAGAA
AATGCTAAGAAGAAGAATGCGAAGAAGAAGAATGCGAAGAAGATGAATGCGAAGAAGATG
AATGCGAAGAAGAAGAACGCGAAGAACAATGCGAAGAACAATGCGAAGAACAATGCGAA
GAAGAAAAATGCTAAGAAGAAGAATGCGAAGAAGAAGAATGCGAAGAAGATGAATGCGAA
GAAGATGAATGCGAAGAAGAAGAATGCGAAGAAGAATGCGAAGAAGAATGCGAAGAAGA
AGAAGAAGACGAAGAAGAAGAAGAAGAAGAAGAAGAAGAAGAAGAAGAAGAAGAAG
AAGAAGAAGAAGAAGAAGAAGAAGAAGAAGAAGAAGTAGAAGAAGAAGAAGAAGAAGAA
GAAGAAGAAGAAGAAGAAGAAGAAGAAGAAGAAGAAGAAGAAGAAGAAGAAGAAGAAGA
AGAAGAAGAAGAAGAAGAAGAAGAAGAAGAAGAAGAAGAAGAAGAAGAAGTAGAAGAAG
AGTGGATGGTGGATTGATGGATAGATACATGGCTCTGAATGAATGGACTGAACAATGTTTGG
CAGAATGGTCGGAAGAAAATAAATGAAGGATGAATGTATAAAAAGATGAATGGTTTAATTGAC
AGATTATTGTAATTTACTTATTCACTGATTTACTGAATTAGAGATCGAACCTTTGTTTAATTAATTT
ATGGATTATTGAAACTGGAAATAAATCGTAATCGTGTTTACTGTCTGTAGGTTGATATCAGAGG
ACAGTCGCATGTTTCGGGTGTGAATCCGAAGGATCGCCAGTCTCCCGTTTGTATCGGGTA
CCCCAATGTCGACTTCACGGTCGAAACGACCTTTTGCAAAACGCAGAATCATATTTTAAGA
ACACTCATTGACTCAGATAAATGAAAAAGACAAAGAAGAGAATTAATAGGATATATATTAAATA
GAGAGTATGTTTCCGTTTTTCGTCTTCTTCATCTTCTTCTTTTATTTTTCATCTTCCATTTCTTCTT
TATCTGCTTCTACTTTCTCTTACTCTTCTTCTACTTCTACTTAGTCTTATACTTCCTCTTCCTCTT
CTATTTTCTCTTCTTGTACTTCCTCTTCTTGAACTTCCTCTTCTTCTTCTTCTTCTTCTTCTTCTTC
TTCTTCTTCTTCTTCTTCTTCTTCTTCTTCTTCTTCTTCTTCTTCTTCTTCTTGAAGAAACCCAAC
ATCATTATTTAGCCGCGCTGGGGTTGGGAGCGCTTCTGAGTAGTCCCACTTGAAGATTCGC
TATATAAATATCTGCAATGAATGAATGAACCAATATCATCACCACGATCTCCATAATCGTCGTT
CGTCATCGTCACCGTGGGCTGCCCATACACCTGGGTCCGTATCGAGGCACTTACCACAT
CTTCTGAGGGCTTCATCGATGCTATTCGGTCTATTCGTGGCAGCCATAACAACCACCTGTG

ATGTTTGCTTCATTCCATCCATCAGTGTCAGCAGCTGCGACACAATGTGCCTGTCCGCCC
CGGAACACTGCATCATAACAAACTCACAGTACGTTGACAACAGTGCTTACATAGGCAGAC
GATACATGTATACTAACCAACGGCGTCTCTAATCTACCTGTGATAGTGCCAGTCCCAAGGC
TAGAGAAAGGAGGCGGGTGAGCCGCCAATCATCTTTCCTGTGACACCGTGTCGTGGAGT
AGCAAGATACCATCCAAATTGAAGACATAATCGAATTAGGGCACACACATACATGCATGAC
GTCAGCATGTATTGTAATGTAAATCTCCGAAAACTTCTCAGCGGCCACAAAAACCACGCCA
GGGAGACCAGCTTATTCACCGGCGCTACACCAAATCAAGTCCGCAATAGCCAAACTCTC
ATTTCGGTCTGTCGAATAATAATAAATGGCAATGTCTGTTTGTACGTCTGTCTACCTGTTTGTCT
ATCTATGTGTGTCCATAAGTCTTTGATTCTGTTCATCCGTCTGTCTTTCTATCTGTCTGTCCGTC
TGTCTGTCTGTCTGTTTGTCCTCTTGTTGGTCCGTGGCTGCACCACCCTATGACACGGACC
AAACACCCTGTCACTCCAAATTGACCTAACCCAATGGACATACACACACGTACCTACACA
GATAGCTACACAAACAGACAGGCGAACAAATGGTTAGAACAGACGACCACCAGAGACAT
AGACAGACAGACCGACGAATAATAAATGGCCATACTGAAATACACACACACACATACACA
CGGAAACAAATAGACACAGGGAAACGAACAGACAGACTGAGGTAAACAAACAAACAAGC
AGTCAGACAGGCAGACCTTATCGCGTTTCGGTGCTATGGCATCCATTTCATCTATGAAGATG
ATAGACGGGGAGTTAGCTTCGGCGGTCTCAAAGATCTTTCGGAGGTTGGCCTCCGAATCG
CCGAACCACTTGCTCATGATATCCGGACCATTGATGACGATGAAGTAGACCCCTGTTTCAT
TTGCAACAGCCCTCGCTATCAGCGTCTTACCAGTGCCGGGCGGACCGTACATCAGAACG
CCCTTTGGTGGCTGAAACACCGACAGATTGTCGTTAGAAGAAAAACGATTGGCGTCACAC
AAATGGTTAGTCAGTTGCAGTACTCTATATTCACACAATCATAATTTCTGTTGTGGGGCGTTGT
GGCTCAGTGGATGAGTTCGGTGCCTTCCATCAGGAGGGTCGCAGATTTGAATCGCACTCT
AGCCGCCACGTAAGGACCTTGGGCAAGTCCTTCACTAGCAGTTGCCTGTAGCGCTCCCG
CGTGTCAGCTCCGACACAGTATCAATGCTGTTATCGGGAGCGCCTCTGAGTAGTAGTGAA
CTTGAAAAGGCGCTATAGAAATATCCGGAATGAATGAATGTATAAATCCAAGTTACATGTTAA
CAATTAAGAAGGATCGTTGGGAGCACATATCGGCTGATGTCAGCTTAATTTCAGAAAATAAA
TGATTGATTGCATGGTGCTGGGTAGGCCTGCTTAAGCTCAGTGATTTTACAACAGAGCAGTA
GACCTACAGATTGTAATTGGCGTATCACGCTTTCAACAGGGAAGCAACAAGTTTAGTGCTC
AGGGTGTTAAATTAATGAGAAACTATATCATGTAGGAACTAGGTTGGCGTATTTGTTATGGGG
CATTTATGTATACATTTTCATGTACATGAGTTTTAAAGTTTCCTTGTATAGTCCCGTTTCTGTACC
ATAAGCAAGACACTAGGAGAGGAGCTGGATCTGCTCAATATTCAACCCCAATGGCTTTTAA
TCACAGCCATTGTACCTTATATAATGTGGATGACTCGATAAGTAGAATCAGTTACTAAGAAAC
GAGGCCATAAAAGCCAGGTGACCCTGTAGTTATACCTGTCCTTCTCTGAGCTCACCTTTCA
GTGGCGTGCAGAGGAGGTGCGAACGGTGCGACGGCCCCGGGCATCCACCCTGGGGG
GCATCCAAGAGTCAGTTTTCGTTAAAAAATGTAGGTAAATGACTAGAAAAGAGAAAAATGTC
GTTATCAGGCACGATGCAGCACGGGGGGCATCCAAGGAGCGAATTTTCATGAAATTTGAA
AACTGATGCGAAAAGGCGAAAAATTTGGGTAAAGACTAAAAAAGGTCGTCAGAAATTTTTG
GAGACGAATTGGAAAAGAAAAATTTTGGAATTTTGGTGAACGACTAAAAAATGGTCGTCAGA
AGTTTTGCGAGGAATAGAAAATATTTGTTTAGGAACTTGTTAGAAAAAAATTCGAGGGCATCC
AAGAACCTTGTTGGCTCCGGGCATCCAACAGCCTCTGCATGCCACTGTCACTTTTCAATG
CCAACTGATATTTCAGTTGCGTGGCTGTAAGATCGATTGTCAATTACGGTAATGCATAAACAA
AGATCGAAAGAATCGGGAGGTTTAACCTTGACGCCCATCGTTCTGTAGACCTGTGGGTAG
CGAAGAGGCAGTTCCACGATCTCTTTGATTACTGCCAGTGCCTTATCGCAACCCCCGATG
TCTTCGTATCCCACTTCATTCAGGTACGATTCCTCCGTCTAAACGCAATTACAATTGCAATTC
TAATTAGTAGGCTACTTTCGACGCCGTCAGTTCCTATTATTATTCGCAGTCATATCTGGCCAC
TTCTAATATGGGGCGTGCTAAGCACCTCACAAGGGCATTGTTGCACGGAAATAAAATTTAAT
CTAGAAACGCTCGTCTCAAAAAAATTTCTTACCAATTTATGGCGGTGAAGTTTAAATTGCATG
TGGAAAACGCCAGAAGGAACGATGTGAGCGGGAGCTTCATGGGAGCACAGAACCGACA

GATCCCCATTTGCAGTAGAGAGCAGTGCCCGCTGCTCTGACGGCAATATAGGGCTCCAT
GGCGTAGCTTATTCTGGGATCAGACACACCACCGACGCTCGGAGGAGAGCCTTGGATAT
AACTGGATCCTCAACTGGAAGCCAGTGCAATATCTCTTGCAGATTGCTTGAAACGTGCGAG
AATTAAGTCAAGTCAATTTCCGTTTAATGGCTGAATAAAACATCAGTGTACCCCGGCTGACA
AAACACTGCATTCGAGATTTACAAGCAATTAAGTTAGAGAATAAAAATTTAGGCTTATCGAAG
ATTGTCCACGACCGGGGCAGCGCAGTTAAGGACGAACTTAAGCCATGGAATGCTTTACCT
TCTACAGGTCTTAAAGTACAGCGTTGCAATGGTCCGAGAGAGTCATGATGAGGACCCGAA
CATGGGTGGCAGCGGTATCAAGCGTTATGTTCCGTGCAAAGGCCTTCATCTGGCACAGTT
AGTGAAAGCAGGGCCAGAAAGAGAGCTGTCGGAGAATATTATACATACTGCAAGGCATCG
AAATCAGGCACGCTATAAACCGAGCCGTAATCAGACCGATATAAAGCATACATGTATCACA
CGAGGTGGATAAGCGATTTGAAAATTGAAACAAGTAATGAATGTATACAGGCTATGCAACAT
TTCGGAAGCTAAATTAGAGACGGCAACAATTCTAAGTAATACTGACTCTCGTTGGTTTCATTG
AGGCCATACTGAAGCATCTTTGGCCACTTTTACTTTTAGTGAAAAGCGGCCAAAGAACAAC
AAATTCGCTACATTTAGCTTACTTTGGGCTATATTATTTACTGTACATGTATATTTAAGTAGTCTT
ACTCACTACTAATATTACTAGCGGCAAAGTCGCGTCAGTGAGACCAACTTAAATGTGGTCTC
GACAGTGGCGTAGGCAGAAATTCGTGATGGGGGGTTTTTTAGTCATGTCCAATTTTTAATCA
TGTCCAAAAAATGTCCAAAATTTTATTTTCTTATGATCTTGTTTTTTAGTCATGTCCAAAAAATCAC
AAAAAATTCTCAAAATTTTACATCGATACATCGATTTTACCTTCCTTCTTCAACAATATCTCCGT
ATTTTAACTCATCCTATTATTCATTTCTTACATTTCTGACACTGTATCCAATTTTTTTCAATATAATT
ATCTCGAAAATTTTTTCTCCTACGAGCCAATGGGGGCAGGTTTTAACCCCCCAAACCCCC
ATTTCCTAAGCCTCTGGGTCTCGAATTGCAAGGTTGTTCGGTCCGCTTGGGTGCAGGATCA
GCGTATCATAGAGGTCAAGCAGAGGAGACTGCACATCATGTTACCAAGTGATGATGGTTAG
TGGTGATGGTGGTGATCTCTCCTTTCCTCGTGTCTTCTCTTCCCACCAACTGATGCAAGTCT
GTCTACTCATTATAGCTGAGTCGGCTAGGGGCACATAATATAGGCTAGTAAGTCTTCCCGCT
TGAGGATTTCCAGCCTTGAATCTGACCTGTCGCCAGAATTGACCTCAACGACGTTGTTCTAT
TATAAATTATTGATGTAGCGCCGGTCCATAATCTGGTCTCTCTAACGCGATTTTGTCTTATGCC
GCGAAAGGTCTGTGTTTTAAACCAAGTTTTAGTGTAAAAACACTGACAGCGATTAACTAGTAA
GTCTGCTCTAGAAGTTGCGCTACATATAATTCAATTACAGTTACTGATCGAAGCGGGGCATA
CATGTACCTGGCGCGCTATGGCCCTTCCTTGCCAAAAGACCGTCGTCTGAGGTCCGACG
ATGGATGAGGGCGCCGGGTCGACGTGTATAACCTTGAAATCTATATGGCCTGCCGTTACA
CCCGTTACTCCCTTCACTGCGAAGATGTCGCCCTTCGTCAGAGGGCGGTAGGCGTTTAG
GAAGTAGGGTTTGAGACAGAGATCAAACAGATTTCTACAATGGTTTAACGCAGGCATACTCT
TAGATCAACGATTGTAACCCACATTACTAACTAATTAGTAGTGGGAAACTACGCAAGTTTGTC
GTTCGAAACCTTCCTCACAGCAGGGCACCTGTTACTAAAAATCATTCTGTCAAATGCTCATG
GCCTCAATTTAAATTAGTTCCCTTCTATTTGGCCCATTCTCCTAATGACGAGCAACGTTCTGT
GACGTCACCTGAAATAGCTCTGCCCTATTTGCAGAGCGTGGGCCAGAGACTCTGAAAAGA
CATGTCATCTGTCAACGGTAATGCTTTGGTTGGTTCCATGTGTGTACGCGGCTCCAGAGCC
ACAGGTACTGTGCATGGTTTTGCTAGCTGATGCAATACTGCCGTTCGTAATGACATTTAGCA
CGGTTTAAAATCTAAAATCGAAGCTCATCTGACGAATCTTAGCCGCATGAAACTTAAGAATA
ACATATTCAATGTGCTTAACAGGACGATTATCGTTACAAAACCAAGCACTTAGGCGACGCAT
CTCATGGTCTTCCAAGTCCCGGTTCGGCGTAATTTAACCACTGCTGATACTTATGTGTGTGT
GCGTGTGTGTGTGTGTGTGTGTGTGTGGGTGAGTGTGTGTAGGTGTAGGTGTGTATGTATGTG
TATGTGTAGGTGTAGGTGTGTGTGCGTGTATTCGTTTTAATATGAAAAACTTCGGTAGACAAAC
CGCTACTCGAACACAAAAGATAATAGCGAATAGATGATACCGATACTCAATCAGACACCGA
GTACTAGCAAAGTGTCCCATAGTGTAAAGATGTTACTCGATATATAAGGCAGCTGAAGTTGG
CCACTTACCCTGTGAAGTTTCCGAGAGTATCTGCGATCGGAAGGACTTGAACATTGACTGC
ATACTTAATGCCACGATAGATTCTCACACTGCAGACATTTCACATAATATCAGAATTTTGTTAA

TTTTCTCCAACGTGTCTTCGTTTCTTCACTGTTATGGGTCCAACACTTATAGCCAGGTCCTCT
ACACATTTATCTATATCGACGTTTGAGCATCGAAATGCGCTGGTATAATCGAACTGTTGGCG
CCTTTAAAACAATTTTGGTAGTGAGATAAATGACATATTTTTTCACGCTAGCCTAATTACTTGAA
ACTCTAATTATATTTAATATTTATGTAACAGCGTCAGTAAGAAATTACTTCAATGATGTTTTACAC
AGCTTGTTGTAGCTCGCTCAGCTTCGTTTGTTTGTTCTAATAAATATATATATAGGCCCTATTTA
TATTATAATAATAATATTATAGCTACCAGTGGAAATGCCAAGATGTACAATAATTATGGCAAAGA
CTATAATTTTAAACTGCTGTTTAAAAATACCTCGGCTACTGTCAAACGGAATTTGCAAGTTTTA
CCTCGTATACAAATGCTCTTTCTTCTTCTGTCTCCACATTATCTTTCTCAACAGTACTGTTGTCT
TACAAGTCGCAGTTCCTTGATTTTTTTAGTCTGTCAAAGCCCTGCTCTAACAAAACTGGCAA
CGTTCCCTTGTACCATCTTCGAGCGTCTCGTCTTCCGTAGTTGTGTCTCGCATTGTATTGTCA
TTCGATTGTCTTTTATTTCATTTTTACACTGTAAATTCTTCTGGTCATCTCTGGCTCGATTTGCC
CTATCACATACTTTTCCATAGTAGGGCCCTCAAAACATGGAATCGCATAAAATTGCATGTATC
TTATCAAGCTTTCTGACATCTGGTCCTCCTTGCAAGATCTAGCCACAATCGCCCTAAGTAAC
CTTAATTCCCCATAACTTGTCTTCCGTTACCTGGCTCGTGAGTCGTTAACTTACCTCGACTTG
CCTAACCTCATGTCGCCTCACCTGACTCAAGTCGGCCTGGACTGACCCAACCGCACCAT
GCCCTGTCCCTCCTCTCCTGATCTGACATGCCCTGACCGGTCACCTTAGTACTTGTCTCCA
TTATATTCACCATTCACATAAACGGCCTGACATCCATTTGGATCCTGACACTAAACCTATTATT
CATGCTTTCTCCGTGTAACCATTTTAAAAACTAAGCTCATGTATATTTAGCTGGTTACCGTAAC
TTCGCTGTATAAACCAGAGCATGTTTTAAGTAAATTTCATCACAGCACAATTGAGCGCGAAA
TTTCTGAATGATCTTACCTACAACAAAATTTCTATTTCTCCAGCCAAATTTCCAAATGACCTTT
TTTAATTACTGCACAAACAGCCTTTCATCACTTTACATTTCAAGTCATCACTGCACATTTTGTG
CATCACTGCAGGCCAAAACAAGCCCTGGTATAAACCCCGGCATTAGAATACGTGAGCAG
AGGGACAGTAAGCTACAGCAGCAGCTGACCTGATGACATCTCCTAATTTGACGCGCAGGT
TTCCGCGTGTGTGCTTGCTGATGCGGATATCTCCATCGCTCTTCAACGAATCGTCTGGCAG
CAGTATGCAGACGGTCTGATGACGGCGTTTCCCACAAAGTTGTACGTAGTCTCCCTTTCCC
ATCTTAAGCTCATTCATCTTTTTCTGCATCAAGCACACAAAGCAACACAATACAACATAATAC
AACACAATGCAACGCAGTGCAACAAGATACAACAAAATACAACACACTACAACACAATGC
ACCACAATACAACATAATGCAAACAGTGCAACACAATGCAACGAAATAAAACGCAATACAA
CACAATACAACAAAATGCAACACAATGCAACACGATGCACCACAATAAAACATAATACAAT
ACAACACAACACAAAACAACACAATACAAAACAATGCAACACAATACAAAATAATACAACA
CAATACAACACAATGCAAACAATACAATACAACACAATACAACACAATACGATACAATACAA
CACAATACAACACAATTCAATACAATTCAACACAATGCAACACAATACAAAATAATACAACA
CAATACAACACGATAGAACACAGAGGGAGTCGATTCTGCCAATCCTTCCAAAGTTCAAAA
GTGCACACGGCAATGTAACATCGATGGCGCCGATAAATAGTGAATGAAGGCCCCTGTACT
CTTGCTGCCTAGGTCGAGGATCGAACTCCTGCTCTTCGCGATGCAGGTCGGCCGCTATAA
CCAATCAGACACCGTGCAACTTCATGGCTGACGTCCTTTAATCAGCTTGGCACTAGAGCTA
CATCATTAGAGTAATAATAATAATAATAATAATAATAATAATAATAATAATAATAATAATAAT
AATAATAATAATAATAATAATAATAATAATAATAATAATAATAATAATAATAATAATAATTAAT
AATAATAATAATAATAATAATAATAATAATAATAATAATAATAATAATAATAATAATAATAATA
ATAATAATAATAATATCTATTTGACAAAAATTAACCTCTTAACTGGGCAGGACACGTATACTCG
TCCAAAACCAGGCTCATTCCATATAAATTCCCCAGGTAAGGTGATAGGAGACATTGAGGAA
TTTTTGCGGAATGTATGTATATAATGTATGACAGACAGACAGGTAGACTGATAAAGAAGCAGA
TCCGCTGGCACCAAACCCCCCACCAAGGGCGTTCATCCCTTGGGGGAATGAAGCAGAA
ATATTCAATAGCCACTTTACAGGGGGAAGTAATTTTTCGGCATTTTATGGAGGCGAAAGAAA
TTTCAGTACATGCATCGGTCACGTTTTAAAGGGGGGAACTGTTAATTTTGTAGTAAATAAAAG
AAAGATAGGGTGAAGTTTATAGGGGGGAAATTTTGGCGGACGAAAAGACATAAAAAGGGA
CATCACGAATTTTTGCGGAATTTTTTTGTGACAGTCTACAAAAATTTGAAAAAAGGGAAATGC

TTCATTGTGTTAGGGGTGATGGACGCCCTTCCACCACCCTTCCCACACACACACGAACAT
CCACACTAACACACACACTGGCTCGCTTAAGAGTACAACTGAAAATTCTTTATCGGGACTT
TAAAAATATTCGATTACAATCACATATATACATGTAGGCAAAACGACCCACTAATATGGACTA
GCATGTATGGACACAAAAGGAGGACAGACACGCAACCATATGCAGCCCTATATATATATATA
TATTATACAGTATTCACGGTATGAAACAGACCCAGTAGACAAGCGGATCCGATGAAATGAA
CACAATGATGATGGTCGATGCTTAAAATAGCTTTGACCTCCAGCTGACCTTTGTTAAGTTGA
CGATGGAACAATCGTCGTCGGAAGTCGATGGTTCGACGAAAAGGCGGTTCGGTCTCTTAT
CCTTTTCCCTGAATCAGGAATTACGCCAACAATCATGATTGGTTCATAGTAGGCTTCTTTTAG
AAGCGGTATGTAAATGTGAGAAATGAATGACTGACTGAATGAATGAATAAATGATTGAATGAA
TGAATAAATTAATGAATGAGTGAAGAAAGGAATGAATGAATATTAAATAATCGTGGGATAGGCT
AATTAATCGCACGTGCTCAGTAGTGGTTGCACATCATGACTGGTACGAGTAACTATTCCCGA
TTGGAGCTAATGCACTTAACTTTTTTAATTAATTTTTTAAATCACATGGAATACATGAAAAAATC
CATGGAATCTTAGCGGCCGTTGATATATACCGGAAGCGCGTCCGCATACGTCATGCGTCA
TCGACTTCCGGAATATGTAAACAACCGCTGAGATTCCATTAATTTTTTCCTGCGTTTCATGTTA
TTTAAAAAATTAATAAAAAGTTAGGTGCATAACCGTTCATTAGCTCCAATCGGGAATAGTTAC
GCTAGACTGGGTTCATACCCAGCTATCACCAGGTAAGTTTTCATTACATTTGAAGCTCACCC
ACCAAACTTACTCGTACTGGTGTGTGGTAGCTTCCACGTCTATTCGTATCCTTAAGACGATTT
TGCCCCCCGGATGGTCTCTACACTGACAGTGCCTTGCCTTTAAATAGCCCATGCACTTTAT
AAATAGTCAGATGCGGTACGATAGGATAATCAATTATTTCTGCCTACCCAGAGTCAGCCATG
GTTCTTCTAAACCGTTGTAGTTTCACGCAGGTAAAGAGCTCTCGACCCTGTGTAATATCTGA
ATAATTACGTATTTGTACGGTCACTGTCGCAGTTGGATAGAAAAAAACTGTTGCGGATGATC
CTGGCGCTTAGCTGTAAATTCACCGAAACTTGATTTAAACCTGGGTGTAAATTCGGTTAATTA
AATCGCCTTGCTGTTATTATCTGGCTCTTAAGACAAGGGAAGAAAATGTTGGCGGTGTATGA
CGACATAAGGCTATTTTAGAGGCCGTGACAATCAATATGACGTAACAATGTAGCAGCTTTCT
TTCTTTGGCGCAAGTGTTAATAGTCATTCAATTCTTAATACAAATTCAATCAACTGTCGTTTATT
AAAACAAGCGAACAAGTGCATAACTGTTACATAATATACATCTATTTTGATGAATTGAAGATTA
GATTAACGCAATATTAGCAATAACGCAATCCTGCTGTCCAATTTAAGTCTAAATTAAAAACCC
AACTATTCTCACTGAATGTAGCTTAGCGCGCTCTCGTGCAAGACTTGCTTTTGTTAAGGCGC
GATATAAATGCTTAGTTGTAATAATAATAATAATAATAATAATAATAATAATAATAATAATAATAA
TAATAATAATAATAATAATAATAATAATAATGTCCCAGTAGCCATCGAAACACTAGGTCCAATC
AATGACAAAGGTCTTGAGTTTATTGCAGACTTAGGCCGTCATCTTACCCAGGCAACAGGCG
AACCTAGAGAGTCCAGCTTCTTTTTTCAGCGTCTCTCCATAACAATACAACGCTTCAACGCT
GTGGCTTTCAGTGGTTCTTTCGTGAAACCCGCAATTGACACGGACGAGGGGTAGCCGGG
AAATGTACTGACTTTCGAAATTTTATGCATAATGTTTGTTTTTGTAATAAAAGGATTTATAATAATA
ATAATAATAATAATAATAATAATAATAATAATAATAATAATAATAATAACAATAATAATAATAATA
ATAATATTTACATTTTCATTCATTTCTTGTTAACGTTGTTTTTGTTATAATAATAATAATAATAATAAT
AATAATAATAATAATAATAATAATAATAATAATAATAATAATAATAATAATAATAATAATAATAATA
ATAATAATAATAATAATAATAATAGTAGCAGTAGTAGTAGTAGTAGTAATAATAATAATAATAATAA
TAATAATAATAATAATAATAATAATAATAATAATAATAATAATAATAATAATAATAATAATAATAAT
AATAATAATAATAATAATAATAATAATAATAATAATAATAACTTGCTGTCAAAAGACTTGAAAATAT
CGCCAAACACGACGCGTTAGTTTTCTATTAAGAAACGCGCTATGCTCCCAGAAAATACTAC
ACACTTTGAGATGCTTTCCATGCTTTGAACATCCGCTCCTCGAGAAATATGATGACACACAA
AGAAGAGGCCTTTCCACAATTTTAAATATCAACCTGAATGACAATCAATGGTGGACTAGGTA
TACGTCTGGCTGCTTCGCTTGCGCTTCCCGCCTTTCTGGCTTCCGCTGCGAGTACCCTCG
TCCTCCAGTCTTCCATGCTTGGCCACGACCACGCAGATATAGACACAGATGAGCAGGAA
GTTCTCCTAATCCAACGATGGAACGAAATAACTGGAACAACTCATCCAGACAACCAAGCA
GCATGTAAACAAGCTCACTGGTGAATAAAGTGCACGAATCACTGATTGAATCATGTCAGGAT

GCATACCATCAAGCAAGACTTAAAGCGACAGCAGCCCCTCACGCTGGTGACTGGCTGTT
TGCACTTCCTATCTCATCATGTGGGCTGCGAATGGATGACGAAACGATCCGAGTAGTGGTT
GGTTTCCGACTGGGAATGACGATATGTGAACCCCATACCTGCCGCTGCGGAACGATGGT
GACTGCAAACGGCTCGCACGGCCTATCATTCATGTTGCCTGGTCCCGGGCCGAATAGCC
AGACATGCGACCATAAATGACATCATAAGCAGAGGTCTAACACAAGCCGGAATGCCTAAC
ATTAAAGAACTCCCGGACTTTCCCGAACGGACGGGAAAAGACCCGACGGTTTACGTTGAT
CCCGTGGCGAGGGGGTCGCAGCCTAGTATGGGACGCCACCATAATAGACACGGTCGCT
CCATCATACCTACATGCGGCGTCCGGGATAGCAGAGACCTTTCAGAACCCCTCATTATGT
GAGTACATGTCCATGTACAGGCCTATAGGCGTTAGACGACATCCAAAGACACGCTCAGAA
AAGACTATTTTAACTTTTCTTGACCTGGATAATAATAATAATAATAATAATAATACTAATACTAATA
ATAATAATAATAATAATAATAATAATAATAATAATAATAATAATAATAATAATAATAATAATAATAA
TAATAATAATAATAATAATAATAATAATAATAATAATAATAATAATAATAATAATAATAATAATAAT
AATAGTAGACTGTTAACTTCAGCTCATTGTTTGGATGAGCATGTCTCAGCGTTTAGTTTTTAAA
GCAATATGTGATTACGATGATGACGATAACGATCATGATCATGACGATACATATCACATGGCA
CTCAAGACAAGTTTCACCACCGATTTTAAAGTAAACACATGTAGGTCTTTCAATCAACAAGC
CGCTGCGTACCATACGAAAGATCGTAGTTCGTTCTTTGATGTAGGTACAGTTCTTACCCAATT
ATAATAAAATAACACAAAACAACATTTGTTACAAGAAACCAAGAAAACAAATTGTAGCAAAA
GAGAATGTTTAGTTCCTGCAAGGAAAGCCTTGTAAAATTGTTCGGGTTCTTTACTACTTTTAAA
TTGTTTCAGCAATGTTCCATTTAAGTAGGGTTTATGTTGAAAAAGAAACTAAATCAAGCTGATA
ATCTCGATGAAAATGAAATTGCATAAAATCCTTTGGTCCACTAGTCTATACGCTGTACTGAAAT
ATGGTGTGCTATAGTGGGACAATCGAAGACACACGTTAGCTTCGAAATATGTCATAGATAATT
TAGCTATAATTACAGTCCTACGAGTTACAACTAAAATATTTCTCAATGAAAACTTTCACTGACT
CTGCTACAGGCAGAGTTCCTGATGTATCTAACCTTTCACCAACCAGTAAAGGTTTTGGAGG
GTTAAGAAATGCTGCCAGGCGACTGAAACTTTTAACAGACCTCTGAATCATTGTAAGTGATG
GGTCGACTGAACGGGTGCAGATACGTCAGTCGTAAAGAAGAAACACGAATGATATCCTATG
GCAGGGGAAGAAGAGTGAATGGAAAAACACCAAGATATGGAAGGCAAAGGGAAAACGC
GATGGACACCAACACTGGCGGAAGCCCAGGATACCAGAGCCAGTCCCTATAGCCTGGA
AACTCACCTAGTGAGGAAGTGAAGAGACATTGGACACCAACGGGCTGTGGGCCCAAGG
ATTTATCACATCATATACTAAATCATTGAAACGTGTACTGACAATTTTCAGAATATCGTATTGAC
GCGCTACTTTTACGGTTCTCCTTTCAATATTAAACAAATTGAAATATATTATCATCAAAAGAAC
ACCAGGGCGACAGAACGTGGAATGTTAAAACCAAAAAGAACATGCCAGCTGCATAAGATA
GTTCAAATCTGCAATTATATGGAATGCAATATAGAAGTACGCTTAACGAACATTGCAGGACGT
CCACATAACGATGGTTACTCGAGAGCAAAACGTGATAAAACAGGGACTGAATTAATGAGCA
ACAGTTCTCGGTGTACGCTGATTTGTGTATACGCAAATGACGTTTCAAATCGAGTCTATACAA
CCGAGTCAAAGCCAAACATGGCGTGGTCAACATATCCCCAGTTCGACAACGGTGACTGC
TTAGGAGAATCTGCTCTGCCTGTTCTCCATTCCAGGTCATCACACGATAAATAAGGATAGTC
TATGTTCGCATGTTTGGTATAGCCACCAAACTGTGCACCGTTTTCTCCAGGTGTCGGTGTTG
GTGGCGGCGGCAGTGGTAACCCTGGCTCATTGTTTTCACTATGGGTCGTAGGCTCTTCTAT
TTGAAGGTCGTTCTTCAGAGCAGGATCGGTACTTAAAGTCTCTAGCAGCTGGTCGCTCGTT
GCCCTATGCACTAAATTGGATCGCTCGTACCGTACCTCGGCTTGATTCGAATAAAGTAACT
GATGTACTTTGGAGTCTTGGGATCCTTCGTTTACCCCATCAGTACTTGTCGGCTGGTTGATC
TGGACATAGAGATTTTGATGAAGGTTATCGATCTTTGCGTAGGCAATGTCCGCTGCATTGTTA
CTTGATGTGTCAGCTGCAGCTGCTGACGGGTTAGAAAAACTAAACCCCGTGCAGTAATCG
ACCTCGCTTCTTTCACTACACGGATGATTCCTCGTCAACATTCCAAGCCGTTCCATCTGCT
CTGGTAAAATATATTGCGAGTGGCGTATTATTTCTCCAGTTGACACAACACTCGATATCGTAG
GATCACCCATTGTTACAGCATTGTTGGTCACAGATGGCTCTCCGGTTGGGATATCGTTGGA
CACAATCGTATATGCGAGGACGCATTCATGTGTACCTTCACCTTCGTCTACATCTTTCTTGTC

CTTCAGTCCGCTGGAGTCTGCTACCGTGAAGATGTCCAGCACAGCTGGTTCCCCTCTTGG
AGCGTCACCGGGTACAATCGTATATGAGAGGACGCACCCATGTGTACCTTCACCATCGTC
TTCATCTTTCTTGTCCCCCGATCCGCTGAAGTCTGCTGTCGCGAAGTTGTCCGGCACAGC
TGGTTCCCCTCTTTGAGCGTCACCGGGTACAATTGTATAAGATAGGACGCACCCCTGTGC
ACTTTCGTCGCCGTCTTCATCTTTTATGTCCTCCGGTTCGCTGGAGTCTGCTGTCGCGAAGT
TGTCCGGCACAGCTGGTTCCCCTTTTGGGGCGTCACCGGATACAATCGTATATGACAGGA
CGCCATCATGCGTACTTTCACCATCGTCTTCATAGTTCATGTCTTTCGATGCGCTGAAGTTTG
CTGCCACGGAGCTGTCCAGGTGTTCATATCCATTGTCAACTCTGAGAGAAGCGGTGACG
TGCCTTGGGAAGCTGGAGAGAGGCTGCAGCGCCCGCTGTCGCCTGAAACGGTGCTCAC
TTGACGGTTGAAATCAAACTAAAAACATAATGTTTTTAAACGCCCTTTATTTCAGTTGAATAAG
CTTTTAGTTTATGGAAAGCCTAGATAAAACAAAAATTATAATGCAGTATTTATAAACAGAgcaaa
gctt

>20E08

aagcttGGTTATTGTGGACTTGATTTGGTATAGCACCGGCAATGTTCCCTCTAATTTTTTAACGT
CTGTGTGCACACAAAATGTGACCGGCTCCGGCAAAAGCAGGCTTTTGGGGAAAAAATTAG
TTTTGCAGATTTTTCACTAGAGTTAATAAATAATAATTTCTAATTAAAATGTTGTAAAAATTATTATT
CTATCTTTATCGGTTCCAGAGATATGAATTTTTAAATGTTATCCTACTCGAAATTACAATTTCGA
GAAAAACGCATGCAAAGTTGATTGATGCAAATAAAAATCCTTAGCAGTGCATGTTTAGTCTA
CCTTGAATTTGTGAGAACTACTAAGGCAAAACGTCCAAATGAAGTCATTTTTAGGAAGCTGA
ATGCATAGGCATTCCAATGACGTATTGCACATGTGGATGCCAAACAGAATGACGGCCAAA
CAAATCTTTTAACTTGTCGGACAATAATTTTAAATTTCCATATAATTTTTATCTAATCTCAGAAAC
TAATTAAATTTCTTGTGATATAGATGAAATATTGTTATATAAATACATTTTCCATGTAGTACAACAT
CTTATTTAATAATATATGCCAAAAAACGAAAATTGAAAATTTTCCACCTGAATGGCTTTTCAGC
TAATTTCTGCTGTTTTATCATTGGCGACTTAAGCCCGCTTTTGCCAGAGCCGGTAACAAATTT
CTTTTGTGCAACATTTTACCAAGACTCGTATGTATATGCGACAATGTACATGAAATATGGGCA
AACACATTTTTTTAAGAAATATATTTTGTTCACTCCTAGTTTTCTTGTGTGCACTTGTCTCACAA
CCTGTGTGCATGCGCACATGCACACAGCTTAGAGGGAACATTGTGCTGCGGTAAATAAGC
TGGTCTCTCTGACGTGGCTTTAAGTGGGTGCTGAGAAGTTATCAAAGATTTACGTTACATTAC
ATCAACGTCTGGTGAATGTTAGCCAGTTATTCGCACATACCAACTGTTGTTGAAGAGCATCT
AAAGGCAAACCATTCCTCACTGAACACAATGCAGAACAAGTAAACCTCGAGTACTTTCATT
GATGGAGCAAAGCATAAAAGGCAATCACAGAATCTGAAGAGAGATAGACAGCAGGATACA
CACAATACGTGAGTCAATCTCAAGCATGTTCTGGCCAATAGCAAAACCTATCTTCTATCGCA
CTGGGGCCTGCTCAGCATTTCCTAAACAGCTTCAAATAAATATCAAAACGTTTCCCATCCC
AATGAAAACATGGCAGTTGTGTAAACCAAACATTGTGAACATGGCTAAGTGCTACACATCC
CGAGGTAGCGATTGCGTTTTGTGTTAAGCCCCCGCTTGTCAGATGGAGAGTCCCTCGATT
GCATCATTTCATTGGCTTCATATGCCACAGTCCATCGTTTAGATAGTGAATAGTTTCAATCGC
TATTCCTTTGTTGACTTCTAATCTGTAAACATTTAACACAAGTCTCGAGTTGCAATACGACTTC
ATCATATGATTATTTTCATCAACACAACACAGTTCACTTAACAAGAAATAATTGCATGGATGCA
TTCAGCTTCTAGTTCAATGAAAGAAACATTATTTTCATCAACCCAACATTCAATAAGCAAAAAT
TCATTGCCTGTATTCAGCCAGCTTCCATTTCATTCAAGGAAGGATTATTTTCATCAACCCAAC
ACATACTTAAGTAGAAATCATTGCATGCATCCAGCTGCTAGTTCAGCTAATTAAACATTCTTTT
CATTAACACAACTAGAATATTTCTGTACATTCATACATACATGCATGCATACACAGATACATGC
ATACCTATGTACATATGTCTACATATATTCAACCTTACGTACACACGGAAGGAAATCTCCTAGT
GGGGAAATGTCCAAGACCCACTTCTAATGCGATTTTAGGCTTTCATAACAGGACCGGATTC
ATTCGGGGGTCTGAACCCGGAAACTCCCCTAAATACGCCCATGACGTTATCCCTTCGGTC
ATTGCTTTTCCACAATCTTTTATGTCATAAGGCTCAGCCAACACTGAAAGGTTCAGCTGATAC
AGAATCTCAGCATTTCAATAATGTCGGGTAATTACAAAGTTTGCCTGTAAACCATGGTTTCTAT

TCCAACTGATTTCATACAGAGTGTTGTGCCTGTTCTCAGTCTCACCAACAATGCTGAAGACT
GTTCCTTTCCCAGTTTGGCCTGGCTTGGGAACGTCTCTGCATAGGAGGAATCTTGAAGGG
CGCTATATAAATTTTGATTGACAGTTACGATGCCACTAGAAAAACCAATGAAAGTGAAATGTA
CGAATTAAGACGACAAATTCAGAAGGGAAACAATACATTGATTATGAACATACATCGCTTGT
GTTGACATCTTCGTAAGTCCAACTGCCAGCGCATGGTCTTTGCCCTCAGCCATAACAGCC
TTCAGAAAAATTAAGGAAACACGCCAGAAAATTCAAACAACTTCACAACTCTTTTACAGATT
CACGCATTCTCTTCCGCTCCCATGCGCAGACGCAAAAATCAGCTACCATTACGTAAAAGC
AGAATATACTTAAGCTACATCAACATATCTGCCACATTTAAGTATATGACTAACTTGATTCGTAT
AATCAATATTTCATTCATTCATTCAGACTATTTTATAGCGCCTCCTCAAGTCCACTACTACTCA
GAAGCGATCCCGACACAGCACGGATACTGTGCCAGAGTTTCACACAGAGTTTCTTTGTGT
ACATAATATATTGAAAGGCCCATACCCAGTCCAACTGCCTAGTTGGGAGGCCAACATAGAA
TGAACAGCAATAATCTAGGCGGGTCATGATGAAGTGGATTAGCGTAGCGGTGGAGTCTGG
AGAGAAACAGAGCGGAACTGACACAACTGGTAGTAAGAATCACGGCAGAGAATGGGGCA
AACAAGACATTTTAACTTTTACATGGCTGGTGTAGCACTTCAGAAATTCTGTATGTAACTTCAA
AATATACACAGTATATGCAATCGAGGCATGATTTTGATCCAAGAATTGCAAAGATGGGGCTG
CTCGATGATGTCCAACTGACATAACGGGGTCAATCAGCCACTTGAATAACAATATACCACC
ACACCAGTTGAATTCTTCTGAATTTTTTTACATAACTACATGCTAAATTAGGGCAAACAGCGA
AAATGTTAGCAGAATAGCTCAAATTTGACTGAGATATTAATTGTTGAAGTTAATAGAAAAAAGC
TGATTATGAGAAAAACACCCAGAAAATGAAGGATTGGAATCAGTTTGCTCTTTCTAAATAAGC
AAGAATCTGGGTGGTGTAACTTGTGGTTTAATCTACAAATTTGTAAATAAATTGGGCAGATTTT
GTTCGAGTTTCGGACATTATATCAGTAGGCAGGAATGGTATAAAGCAGGGATGCACAACCT
GCAGGCCAATTCTAGCCTTTCGTGCAGGGGCGTCCAGTACGTGCATTGTGTGATGCATAA
ATGCATCATCGTCAAAGTAGGAGGGGAAACGAAGAACACAGAAAGTATATAAAAAAACAC
GTAAAATTTACGAAATCAGGGGGAAAATTTTTTAAAGTAGGGGGAAATAATAATTGTTCTGAA
ATAGGGGGAAATGTACTGAAACAGCGAAAATAGGGGGGAAATTTCAAATTTGCAGTCGATG
ACTAAAAAAAGTTCATAAGAAATTTTGGCTGATGAAAATCGGAAAAGGTAAAATTTTTAAAAGT
TTCGACGGGTTAGAAAAATGTCTTTGGAAATAGGGGGGAATCTGAAACGGTGGGAAAATG
CATCATTGCCTGAGGGGGGATGGACGCCCCTGCTTTCGTGAATTATGGAGAGTTTATGAAA
GCATTTAAACAACATTGAACTATTCGAAATTTTGTAACTTATTACAATCCCAAACAACTCTGAT
GTATTCGATATATGGCAACAATATAAAATTTAAAGCAACAAAAAGGCTAGTTAGAAAAGTTTTG
TTTTCAGTTTTTGATACTTGACTTTCAAAAATTATTCAGATTATAATTTTTGTCTGTCAAGTGCTAA
AAAAAATAATACACTGCAGCCCTTGGCCCAAAAAGGTTGTGCACCTCTGATATACAGTAAC
TAGATGTTGGCCCGTCTTATAGGTATACGAGTGCTCATGAGACATATTGTCAGTGACTAATATT
ACAATAAGCAGGGCTTGTTTTAACGTGCAGTGATGCAAAATGTGCAGTGATGAACGAAAAT
GTGCAGTGATGAAAGGCTCTTTGTGCAGTGATGAATTGAAGAAATTACAAAATCGAAGCGAT
AGAAACAGTGAAAAGTTGAAAGTTAGGGTGTAAAACAAGGAAAAGAAGGTTATGTGCAGTG
AAGAATTTTTCTTAAAACATGCTCTGACAATAAGCAAACATTTCAACTAATATCTAAATTTTTATA
AGTTGTTTCGTAATTCAAAATGATAAGTACTCAGAGATTACATGATCGAGTAGTAATAGGCGAT
AAGAAGTTGAAGAGGAGTTGAATTTAATGTATTCAACTACTTCACTCAACAGCTGCCTCATAC
AACATCATCTGGCATCTTGCAGTTGAATATCTATCTATCTATCTATCTATATATCAATTTATATAG
CGCCCCTTCAAGGTAACTACTCAGAGGCGCTCCCAGCCCAGGCTCGGCCAAACAGGA
GAGTCTTGAGGAGTTTATAAAAAGAACTGGAAAGGTCAGGCAGCAGAGAAATACACAACA
CATTAACGACTGAAAGGATACAACAATAGTTTCTGCCTCGACTTTAGCCATTTTGGCCCCA
GGCGATGTCAGACCAGGACACATGATGTTCGCACCACTCAGTATGAACTTGATGGCACCT
TTATCGACCTGCATGTGTGGCATCATAAAAGGATCTAAAAGCAGAAGACAGAAAACTATATC
AAGAGAAATGAAGGAACAAACAAAACAGAAATCTATGTTAATTAAGTAAACAATGCAATGAA
CAACAATTAACAGAAAGTTAAACAAACAAACAAAGAAATCCACCTGAAATTCATTCATTCATT

CATTCATTCCGGCCATTTCTATAGCGCCCCTTCAAGTCCTCTACCACTCACAAGCGCTCC
CGACTACAGCACGGATACTGTATCGGAGTTTCACGCCGAAGCGCACAGGCAACTGCAG
GCAAAGGACTTGCCCAAGGTCCCTACGTGGCAGCTAGAGCGGGAGTCGAACCCACAAC
CCTCCGGTTGAAAGTAATCGTCTCAACCAAGGCGCCACCACGTCCCACAAAATACACTC
CATACACTTTTTCTGACGAGCACACATAAACAATTTCAACAGAGAAATAATGACACAGTCAT
GTCAGCTGTGATACTTACATTTATGCAAAAGTTTAAGAGTCGGATAGAAGTTGCCATCTCTTT
GTCGATAGAAGAGAAGCTCTCCGTCTTTGCCGACGACAATTTCGATGTGGTCCTGGCTGG
AAAAGAAACAGTCATCACCATCATCAGTTCCTGACACCAGCCATTTGGCGGCAATGGGTA
CGAATGAATGACCTCCACTGTCCTTGGTCCTTCATCAAGTCCATTGCTACCCTCAATGTCA
GTTCAATTGACTTTATGTCCTCGTTGATATTGTCTAGTGAGAAGCCGTAAAACAGGTATCGTC
AGTGCTTCCGCTATGGAGTCCAGAAAGAGGACTTACATCCTTGCCTAGGATTTTAGACGTA
GTCCATTGCTGACGGCAATCACACTGAGAAATTTGGAAATTTTTGAATATTTCACGGATGCA
AAAGTGTCATTTGGGCGTCATTTGGATATCATATTTTAAAATTGAAACATCCTAGAAATTACTA
GAAAGCATACGACTTGGTTACCAATGCTGAGCTGAACCGCAAAATAAATACAATTGCTGAA
CAGTGCATTGGAAGAAAAACTACCTTTGTAATGTAGCTCAGCGCTGTCCAACCTGTGGCAC
ATGGGCCACACGCAGCCCTCAGGCTGATTTCAAGTGGCCCACCCGAGCTCATAAAATCT
TTCCAATTTGTGTAAAAAATGAAGCAACAGAAAAAACTTGGCTTAACATTTGGTAATATGAAC
AAAGATGCAGTTCCAAAGATGCATTAAACTTGTCTACATTTTTCTCGACCCACAACAGGCAG
CAGTTGAGTTTACTGGTGCAGCTCATGGCTTCAAAATTAATTTCTTATGTGACCTGCAGTGTC
AAAAACGTTGGACGGCACTGTAATATATGATCAAGACTCAAGACAAAAATTCCATGGCAAA
ATTTCAAAGCAGTGCAACACAAATGAACCTTACCCAATCAAAAAAAACAACAAGAACAACT
GGAGCAATAAGCATATTTATAGTAAGACGCGCTGTAGACTGAAGCATAGATTTACAGTGAAC
CAAACAAGGCATAAACATGCCTAACAAGAGCCACACTGTTCAACCTAGCACCATTGCGC
CCAAATGAAAATCACGAATAAAGTGTGCGAAGTGACAGTAAGCCAGCTAAGTATGACTAGA
TCAATGGTTCTCAACCTGTGGTCAGCGGATATCTGCCTTGGTTTTCCGTGAGAAAAGGCTAT
TTTTTTTCTACGAATATAAACACACGAACAGACCAATTTTGGCAATTTTCCATGGCAGAATTTT
CAGTGGTGGTCCGCGAGTGAGAGACAAAACTTGTAAGTGGTCCTCGAGTTTAAAAAGGCT
GAGAACCACTGGTCTATATCAAATAAACATGTCAGGCAAGATTACAGTGAAGACCTCACAC
TCAAGCAGTATAAAAAGCATACAGCCAACCAGTCTAGCTAAGATTTCAAATACAAGTCTCTG
CCACCATGATACTAATATTATAATAATATATTTTTGTTTAATATTCAAACTGGCAGACCCCAGAA
TGATTACAGGCACAGCATGAACAGGTGATCACTGAATTGTATCTGTAATAAAGAATAGAGTTC
GATTTTTTGTTTCTAGAGAAGTCTTTCCCCTGGCACTTCCTGGAACTACAAGGTCTAATTTTC
AAGCATAATCCCAAACCTCTTTCGTAAATTGTACAGTGGAAACTGACATTAAACAATGAAAA
AAATTACCATTTTGCCACTCGCAGAGGATCCTTTTTTGGCAGTATCTGGTCCAACGACTCTT
CGAGGTATGGAAACTGCTCCAACAACTTTGATCGAATCGCCTTCTGAACCGATGACTTCAC
ATTGTTCACTCCTGTCAAATTCTCTCTCTCGCTAAATCTAAAACATCAGTATTGAAGCTATGCA
AACCTATCCTAAAATATAGCAGAACATGTTGTTTATATGCCATAGCTCTGCTCTCATTTATAAA
ATGAAATACTTGTAGGAATCGAGCATTATATTTACTGGAGCAGTGCTACATTGCCTCTAAAGA
CTTTGTAAGAAATATTCTGCAGCAAATGCAATTAATACCAGTGATGAAATTTTGACAAATGAAC
AGCTCAAGTGATCATAGGTTCATCCATATCTGGCATATTGTAATGCAAGCTGGGCTTGCGAA
CGGGTACCCGTGGAAACGCTGTCGCAACGTTTATATACCAACTGGTGCACTGCACAGAG
CATGCAGAGGGGTGATACAAGGGAAAATCACAGAGGAAGAGCAAGCAAACCATGCAAGA
GATGAGAGGCAACCCAGGCGAAAGAGCCTAACACTAGTACTGCTTCGCGGTTGACCAAA
AACGGATCGACTAAGCGCACTTCCCTTAGGGTAGCACTCGCACAGCGGCAGATCCAGAT
ATTCGGCTAGAGGGCACATCATGTGGCATATCGAGACATTTGGCTGGGGAGGGACAATTT
AATATTTTTCCCAGTATCTCACATTTATTTCTTCTTTGGAAGGGGCCAAAGTCTGTAGCCAAAT
TGGATGGGGAGGTCATGTCCGGATTTTCCCCCTGGATACGCCACTGCACTCGCATGATTG

CATCCCATGCGAAATAGAGCGCATGCATAATGGTTAGGTAGTCTTTGTGTAGAGATCGCCAT
GGATAAGATAATGGCATGTATTGAGCGGGTGACTGAGCACCCATTCAAAATTTGGACTGGT
ACCTGGGCAGCTTTTTTTAAAATCGACAGTCCTACCCAATGGTTCCCATCTGGTGAAACAC
TAGCCCTTTTGGATCTCCCATTGTTTCCCATCTATATATAAGCATCTAATCTGAATAAGTAACTT
GGCAAAGTTTTAGAAGTGAGGATGCATATATACAGTATCGTTTGGCTAATGGGGTAAATGATA
TTGGTGTGCTGTGACTGGTTGAGTTGAGGTATGCCATATTTTCTGCTTTTAGGATATTTTCTAAT
GATGATGCTAACCCTGATACTGACCCCTAAACGACCCCCAAACGCCGGTGTACATAAACA
CTTCTCTGACCTTCTTCAACACCAGATAAGTATCAGGTAAGTGTATGTAAATGCTGCTCAGG
CCTTGTTCGGTGCCGGAGAAGTATCAGATAGCTGTAAGGGTAGACCTATGTAAATACTGCC
TAAGTAGAAGCTGGATAGATGAAAAGAAAGTTGCTTAAATAAAAGCTGGACTAATGAAAGAA
TCCTTCCCTAAGTACCTGGATAGATGAAAAGACGTTAAGTAGAAGCTGGATACTTATCCGAC
CCTGATGAAGGCCAGAGTAGCATTTACATACACTTATTCGGCGCCGGAGAAGTATTTATATT
AACTTCTCCAGCCCCGGAGAAGCCGGAGAAGTTGGAGAAGTACTTATGTACACACCATGA
CGCTTAACCTGAATCTCACCGACCCTCAAACTGACCATAGGCAACCCTCAACACGCCTA
ACCTTACCCTAACTGACACCTCACGATATAAAATGTATACCCAAATAATGAACTGAATAATGT
CACAGTATGCGCATACAAGAAGCATGCCTCTGCATATTTATAGTTCTTGCATATTAAATATTCA
TAGTAAACGATCACTATCTTATAGTGTCTGCATCCCCACTTCTATATTATTTCCCAGTCTAGCA
TTTACTTTCCTTTATTGACGAGAGGTGCGATGAAATACAATAAAGAACTATGTACTACAGTCA
GATGTCAAAACCTAGTTACTGGCCGTCATCTAAAATATTGTCGAATTTGGAAAATAGAATGGA
CTAGAAATGGGTTTTCATTGGGACCTGGCAAGCACAAGAATGCCGAAATCCCTTCGTGAC
ACACATCCACATAAACGCGCTAAAGGTCCGCATAGATTCCGTTTATTCTGCTACGCATACG
ACTGATAAAGCGCCCCTACCAACAAACACAGGAATTGGGGATTCTCTTGAACTACAATCTG
TGTGAGTGACGAGACACGTGTGAGGTATAAAACATCGCTTTGTTCTCCTATAGCTCAACTGA
AAGCTTCATTCAAGTATTAAATCAGACAGCTACATATTTCATTATGCTTCGGCTTTAAAACAGC
CTAGTTTAACTCTTACTTTTTAAACATCGTGAATGTGTTTCGCAACACAATCTATTACAGCACG
ATCCGCGTTTTTGGGTTATACGAAAAGCAAAATACCTAAGCTACATCAACACATGTGCACGT
AAGTAAGTAAACGACTAACTTGGTTCGTATAACTGTAAATAATAATTATTTATAAATTGCGACGT
ATTGACAAAGATGCAATAACTTGCATTTCTCTAAAGACTCTGCAGACTCTGTTTAACGATACT
GCGCCATAACGAAGATGATTCGTGTCGCCCTTCTGGTCTCCTCCTTTTACCAGAGTTGGGA
CTGGCAGTCTGGCACTGCCGTATATATCACTAACCATAGGCGAAACTTTGTCAAAGCACTT
GGTGACCTATTGCCTTCTCCTCCTATTACCTTCCCCTCAGCTCCCGAGTTCTCTTATATGCA
TAGGCGTAATTACTTGTCGAAGATATAGGGGGCCAATTGCCTTCCCATCCAGAAGCGGATC
TAGCGATGGGTCGGGGGTCAGCTGACCCCCTGGCCCGATTTGGTGAATAAAGTGGCCTG
TTTTTAGGACTAAATGTAATTTTTAAAACTTGAAAAAAGAGGATTTAGGCCTACTATATTGGAGT
TTAAATCTTTTTGTCAATTGTAGGCTACAAGCAGATGTTGTAAGATGCTGTAATTTATGAAGTTC
ATAAAATCTGTGATGTTTTACATTTCTTATAAATTCGTGAAATGAGCTGGATTTTTTCAAACCCG
GCCAACCTTTATAGAATATAACGTTTTGCCTGTCAGCAGTGCATCTGCTGAACAAAGTTTTTA
ATTTAAATAAAAAGCTGCACACGCTCAACAAACGATTAGATCCGATTGTCGGACTTGTCTGT
GCTAAATATTGAACAGAAATTTACTGAAAATTTAGATTCCTACAGTGTAGTTGACACTTTTGCT
AAAATGAAGAATAGACGTAAATAATAGTAGGCCTACTAGAACTATATATGTATAATGTAGGTCT
ATATAGGCCCTGGGGTGCGCCGTATAGTCGAGTAGTAACGTTCGCGGCCATCGGACTGC
ATGCAAGGTCCGAAGTTCAAACCCCAGCCAGGGCAGAAATTTGAAACAAATTTTCTGCTG
CATGCGCTTCGCCTCTGGGACTGTGCACAATGCCGGGTATCAGATCCAGTCCAAAGCCT
GGAAACTCACCGAAGAAATGAGTAAGTGAAGGGTCGACCGATGGGTGCAGATGCATCAG
ATGCATCGTAAAGAAGAAGCCCGAATGAAATGCAATGGCAGGAGAAGAAGAGTGAGTGGA
AAAACATCGAGATATGGAGGGCGAAGGTAAAGGCGATGGACACCAACACGGGCGAAAG
CCCAGAATGCCAGAGCCAGTCCTAATAGTCTGGAAACTCACGTAGTGAGGAAGTGAGGG

GACATTGGACACCTACGGATATTGGATATCCTATCCCGGATATCCTATTGGATCCTATTGGAT
ATCCTATCCCGGCTCTACTCCTGCACCACAACACGTCCGCCATCATCACCTACTCCAACC
GTAGCACCACGCAGTCTCCCCGGCTTGACCTTTCCGGATTTTGACCTGGCACCGAAACG
AAACGAGAAGTTTGGCTATTGCGGACTGGATTTGGTATAGCGCCGGTGAATAAGCTGGTCT
TCCTGACGTGGCTTTTGTAGGCGCTGAGAAATTTTCGGAGATTTACATTACATTACATTACGG
GCGCTGAGCAATGGATGGATGGATGTAGGCCCTACAATAAAGTAATGCAATATGCCTATATA
AATAATTGCTGTATTATTAAATATGTTATAATAAATGTTTCCAAAATCTACTTCTAATAATAAATGTT
TACAAAATCTATTTCTAATAATAAATGTTTACAAAATCTACTTCTAATGAAACCATTGACAATGCT
CTTAGAGTCGGAGTCCTACTTTCACAAATTTCAATTTTCATATTGCTTTCAATCATTTCATGTAA
AGTGTAGGGCTACTGTATAACGTTACAAAGTATTGGTGGCTTTGGCCTGAATTTCTTAAATTTG
ACCCCCCTGATCAAAAATCCTGGATCCGCCTCTGTTCCCATCCATTCCAGAGTTCTCTTAC
ATGCAGCTAGGTGGTCTAAGGAAGCTATAAATTCTCAACATTTGGATCCGGGATTTATATTTT
AGAGCCTGCTGTGACTGTATGATTGTGTGGTTTGCATAACATTAACATTCCAAATGTAGGCCT
AATGTATTCATGAGCCTGCATGGTTTTGCAGGCTTTTATAATTGAAAGCCTGTCTTCATAAATT
TGTATTCGTGAATTCGAGCCCAAACAACCGAGATAAATAATAATTGTTGAGCATGCATATCAA
TTAATAAAATAAGTTCTTTGCTGATAAACTTTTGAAACAAGTATGCGACATTAGAGAACTGCG
CCAACCAGGCCTCTCCGCCGCCGACGACAGACTTGACCAGCGGCGTCAATCTGGTTTG
AAATCTGGGGTGGTCGTGGACCCGGGTCAAAACAAATTTGATTTTCTAGGAAAATTTCCGA
AAACTTTAGATTCTTTCAGGCAATTTCACAAAAAAAAAATCAATTTTTCAGGGCAAATTTCCG
AAAAATTTCGGTTTTTTCAGGTAATTTCACAAAAAATTTCGATTTTTCAAGTTAAATTTCCGAAG
AATTTCGATTTTTTTAGGCAATTTCCCAAAATATTTCGATTTTCTAGGGAAATTTCCGAAAAATT
TCGATTTTTTAGGCAATTTCACAAAAAAAATCGATTTTTCAGGGCACATTTAAGAAGAATTTTG
ATTTTTAGGCAATTTCACAAAAAAAATCGATTTTCTAGGAAGATTTTCCAAAAATTTAGATTTCG
TTTCAGGCAATTTCACAAAAAATACGATTTTTCTGGGTAAATTTGCGAAAAATTTTGATTTTTTT
CAGGTAATTTTATACAAAATTCCGATTTTTCATGGCAAATTTCCGAAAAATATCGATTTTCCAG
GCAAAAATTGGCTATTTCAAGCTATTTCTGGGAAAATTATCCTATTACTCTTCAAAAGTCACC
ACTTTCGAACATACTTCCTGTACATGAAAAGATATAATAATATTTTACGACCCGTCCACGACC
CCCCTACGACCCCCCTGCGATCCCCCACGACCCCCCGCCCAAAATCTGGGGGGTCG
CGACCCCCCAACCCCCAGGATTGACGCCTATGGGACTGACACCACTAAGTCGGCGCAT
AATAGCTCTACCTGAAATCGACCTCTCACAGACTCCACACTCCACAGAGTCTCACCACAA
CTGAGTTGGGAAACATTAGGCCTCTGATCAGAATAAAGCGCTGCGCCGGTCACACCATC
GCCGATTTTTGACGGTCCGCGAGACGCCGCCGCTAACTCGTCGGTGAACCATAGATTTAA
TTATATCGCTGTTATGATTAGAATTCTATGCTTTCAGGTGCTTGACAGAGCGCATGCGCCATC
TCTATGGGAACCTGATGGCAACAGCGTAAGAGGTTGTCATTGAAGCAGTAAACACCCAGT
GGAATAATTATTAAAGTTCTCCAAAACATTCTTGCGAGTTTTAAACACATATTTTCGCTACAAA
TTTTGTTGAAAAACAAGCCGGCATGTATAAATTAGATTTACCGGTGGATTACAAGGAGGCGG
CCGCGAGAGAGAGACGCCGCTTGCAAGAGGAGCAACGAAAGAGTCGTATATTCGATTCC
AAGACTCGACTTATAGGAATCGACAAACAGGCGCTGGAGCAGCAGATTCGAGATCGAAAA
CAGATGGAAGCCATGGAAAAGAAACGAGAAGAAGCATTCGGTATGAAATTGGTATACGCC
ATAGTCGCCCGTCATCCTTATCACAGCGTTACAATTGAGTAGGCGTATTTTATGCTTGCTCTA
TACTTGGGGCCGTCCATAAATTTTGTCAAGCGATTTTTAACCCCCGTCACATTTCTGCCTCA
AATCTCGGACTCCCCTAAAGTACGTCACACTTTGGAACTAAAAATCCTAATGATTGCCCGG
CTTCAATATGCACTTCTGAACATTGGAAAGAGGGCTCTATCAAATTACAGTTGTATTGTATTGT
AAAAAAATTGCTAAATAATTCCCAACTAATTAAACTTTAATCTAAAGCACACTTTACTATTAAAT
TGTACTGAGTTCAGAATATTTTAACGTAAAAGAACTATCTCATTGAATTCAGAATACTTTAATGA
ACAAGAACTATCTCATTATTCAAATGATTTTAACAGTAGACTAGTTGAACAGTTGTCACAGTTA
TTCATTGAAGCAATAATTTATCTAGACTGAACTTGAAATTTTTTCTTTAAAATACATTAGAGAATT

ATAATATAATGCACAACATTTATCTGTTGTTTTTTCATTTACAATCATTGGATTAAATAATAAAAAG
GGAAACAAAACAGTTTGACTAAGAAAGACTAGAATGCCAATTAGGTGACACACTCTTGTGT
CTTATTGATTCTGTAGTCGTTATTTTAAATTATCATCATCATCATCATAATCCTTGGGTTCAACG
CCCGTAGGAGTCCAATGTCCCATCTCTTCCTCACTAGGTGAGTTTCCGAGCTATTGGGATT
GGCTCTGGTGTCCTGGGCTTTCGCCCGTATTGGTGTCCATCGCCCTTCCCTTCGCCTTCC
ATACTTGATGTTTTCCATTCGCTGTTATCTGCCATTGAATTTCAATCGTGTTTCTTCTTTACGGC
CGACGTATCGCCACCCATTCGGTCGACCCTTCACTTACTCACTTTTAGGTGAGTTTCCAGG
CTTGGGACTGGCTCTGGTACCCGATGTTGTGGTCTCAGAGGCGGAACAGCGGTGCACAT
GGAGCAGAAAACTCGATCCAAATTTCTGCCCTGGGCGGGATAGAACCTCGGACCGTGG
CATCTAGCGGCCACGGATGTTGCCACTAGACTACCGCGCACCCCTCCTTTTAGTCGCCT
CTTACGACATGCAGGAGGCTACAGCAGAACAATTCTAACCCCGAACCTGCAGGGGTTATT
TTAAATTAAGAACCGTAGAAATATTAAACCTCTGGCATGATGAAAGTTCATTGAAATTTAAAAT
TCCATGTAGCATTATAATTTCTGTTATACTAATTACATCTTCAAGTTAGAATACAGAGTAGGCCT
ACATCCGTCACAACAGAAAGTTAAACATCACTTTCAATGTCAGGTTTCCTATGTATGATAAAA
GCATGTGTGACGTCACACATGATCTTTACCCTTACCCCCATCACTAGCTGTCACCCCTTCTT
AGACCCCCTTTCCCTCCTGGAGCATGACATGCTTTATGGATGGCACATTGATTATCACCAG
TGATGGTCGACACTTCTTTTCAAGTGTATTTTGATAATATTTTTTAAAAGTTTCTTAAAAATACAA
AATGGAAGTGTATTTTCAAAGCATTGGTGAAATCGTATTTGTGATTGGCATACTCCTATTGCAC
CAGTGTCTAATGCACTAGACATCAACTGACGGCCACACCAAAGTCAAGCTGTATGATGCAT
TTTCAATTGCAAAGACATTGCAATACAAAGATGGGTGTATGCAGCAGAGTTTGTGTTGTTCTC
ATAATTTGTGTTTGCGATTCTTTTGGTTGCAGCGGCAGATGCAAAGCGATACGACATGATCG
CAGAGCTGTTGGAGAGGCGTCAGGAGCAAGACATGCGTGAATTGAACGCTGCTCTCAAC
GAGTTCCGGATGTTGCATCAGCAGCCGTCCAGCCGCCGTGAATGGGATCTCTACGATCC
GGATCGACTGAAGAAAGATCGACCGGCTAGAGTTAGCGACGATGATCCAAGGTGTGGTGT
TTCCGGTCTCCAGAAGTTCGAAGGGGAAGACTTAAACAACAAAGTAATTTCAAATTTTAATAT
CATCTGTCATATTCTGGACATGTGCAGTGTGACCTATTCTCCCACAAACGTCTGGGATACGG
ATTAGGTTGCATTTTCAAAGAAGTTAAGCTGTATGCATTTTCTAAGCCTAAGCAGAGTACCGG
TATTGGTAAGTGCTATGTGTGTACATTATGTTCATATGCACGTATATTTATGTATCGATCAATTAA
TCGATCAAATCAATCAAGAAGTCAATCAATCAATGTACTGGAGAGTGTTAGTATAAAGTACAA
TGACGTCATGTTTATGTTTTTGCTATTCATATATACTCATATACTGCAATGAAAATTGCATCCCA
ATACCGTAAGCCCTTAGTACCTGAACTGGATGCACTCTACACTAAATATTCAGTAAAAATACT
AATTTAGTTGTCTGACTTGGTCTATAGAAAGCTTAAATGATTGAAAAAATATTGTCAAGCAGATT
TGTAGAAAATATTTAAAAGGAAAAACATGGTTGTGATTTCTTTCTCTAGGCTCGGACTAAATTC
CAGGAAGAACAGCGACGGAGATGGGCAGAGCAGCAGATGAAGGAGAGGAGGCAGGC
GGAAGATAACCAAAAGAGAGCAGACTATTTGTATCAGCTGAAGATGAAGGAAATGGGACA
GAGGGCAGCTGAGCTGGAGCAGGCGGAAGAGGAGTGCCGCCGAGCAATACTCCAATC
GAACAAGGACTACAATGCAGCATTGGTTGGTTCAGTAATAATAATAATAATAATAATAATAATA
ATAATAATAATAATAATAATAATAATAATAATTTTCCGTGTTTAATATTCAAACTGTCACAGATTCA
GTATGATTACAGATATAGTATGAACGGATGATAGCAAGTTATTACAGAAGTCGAATTTAACTGC
AAGTCAGCCACACAGGTTATACATCTCATTTAACAAACGCCACCACCTAGAGAGTAAAAGA
CTAGTCAACCTTATGGGGATCATACAAGGCATGAACTGAGGTCGGCCAGCACCAAGCAC
CAAAGTGTTATCCACTATTCTATGAACCCCAGAATGGTTTAACTGACACAATATGTGTGTATTA
TGGTGTGCTGCATTGCAAAAGACCATAAAAAGTGGTTGATGGCAAGGCAGACAATAAGTGA
TGAAAATTATTAGAATGCTAATTTAATTTTTTTTTTACCCGAGGTATTAATGATCCCTCAGGAAG
ACAAAATTATGTTAGGAGTAAAAATGTTCTCTAGGAAATCAGGCCTCGTCCCAGGCTTCTCG
ACCATCCTGAAATGTACCCCTGAATGCGATGGCATTGTGGCACTGCACACAGATGGAAAG
GCGCTGCAGGAGGAAATTTGGTTCGCGTTGATCTCCGGAGACAGATGAAATGCGACGTC

CCAACTCCGAGAAAAACTCTGCACCCTCAGCATTTACAGGCCCAAGAGTTTCGAAGGCA
ATGGGGACAAACACATGGGTTGCAGGCAATAATGCATATTTGGCAGTCTTCGAACAGCAG
CCTACTCAGCTACAGAACCTGCCGAGATTGCTGTCTGCAACAAGTATGATGCAGCACAAG
TGTGGACCCTGTGGCATCCCAGGCCATAATTTAATTATGTTGATTTATGTTTTGGTAAATGTGT
ATTTTATATGCATATACTCAAATTTCAGCCATAGTGGTCCAGTGGTCCTAACCTGCTGAACAA
AGAAAACATCCCAGTCCAAAGAGCAGAGGTCGATGTGATGCTGCAGAGAGAGAGAGGGG
AACAGAATTTTTTGGAACAATACTCTGCTTGGACTAGAGCTCTCGGTGCAAGAACTCTGAGA
GTAGAAACAAGTATTAAGAACAGAGAAGGATGTAGGAAGATTACCAAGCAGAATGGCGAG
ACATGGAGAGGAAGGGGGAAGGGAACATTTAAGAAGAACAAGAACTTTGAACTGTATGCC
ATCAGGGAAAAGAATCCAGTATAAATGATACCAATGAATATGAGGAGAAGTGGGAGAATTTA
TGAAGGTGAGCAATCAATCTGGCAGTTGCATCAATATCTGACTTTAGTAGGCCTAGAATTGC
AACGGTCAATTCGAGAATAGACAAATGAAAAGCGGTTCTCACCACCATGGATCCGCAGCC
ACCTGCCTGTTGGTCCGCAAGCAAGCCCTTACATATATCTGACTTTAAAGAAAAAATATGCA
GAAAGAAAAATGTTTTAAATTTTTCGCATTTTTTCATTTAAGTCTTGGCTATCGACAGCCTTGG
TCTGTGAACCTTAGTCTGTGAACCAGTCTGTGGACCAAAACAAAATTTACCAAGTGGTCCGT
GAGTCTGAAAAGGTTGAGAACCACTGCTTTGATGTTTTGACTTTAGGCTCGTGAGCAGGAG
ACAAAGAGTGCTCTGAAGAGACAGCAGGACGAGGACGACAAGCGAACGGAAATTGCAA
ATCATCTATACGGAGACTTCTTGACAGAGAACCCGGCTGCGGCTCAGAGCGCCTTTGGCT
CCCACCGTGTTGTTCCTGACAGGTGGAAGGGCATGACCCCGGAACAGATCGCGGAGATT
CGGAGACAGCAGGACATCCAAAGAAAGGAGGCTGAGGTTTGTGGTGCTACCATTCCTGG
CTTAAACTATGTACTTTTTGCAAATGATGAAATGTAAGACTTTTTCGTAGTGCCTAAAATAAAC
CTTTGAACTTGAGACTGTAAACATGAATTTCCTTGTACAGTTAAATCTGGAGTCATATAGAATTT
ACTAAGTTATGGTGTGCTGACACTTTGATGATGATCACAATTTGGAATCCACTTCCTGATTGAT
AATCTCTTAGCAGCAATTGCTTGCAAAGAACCATGCTATGGGCCCATGAACTGGCCAGGC
TCTTTCACCATGCCACCTTGTCAGCAATTAATTACTAAACAGTCAACACCTGCTTCAGTATCA
TGGTGCTGCAGCAGTGCCTGTGTTGAGTTGTAGTTACCCTATCTTGCCTCATGTGTGGGCT
GTCACACATTATAGAAGAGAATAGTAACATTTTCCACACTCTATGCATGCATTTATTAATCAGT
AATACATGTACATAGAAAACTGTAACCAAAAATTATTACTGCATTATTGCATATATGGTAATATA
ATAAAAACACCCAGAACGTATGCCATAAAAATTTAGAGCAAACATATGCAGTAAAGATTAAAT
AATTTATATTACGTAAGTAAATAATATACATGCATATATAATACCTACCTACATACATACATACAT
ACACATACATACATACACATACTCAGATACATGCACTCATGCATATGCACTTACTCATATGCA
TACATACGCATATATATACACGCATACATACATACACAAAGTAAATAGAAATCAATAAGTTCCT
CAAATGCGTGTTTCTTTAGCGAGCAAAACAAGAGGCCAAACGACTCGAGGATGAGCATGA
CAGAATTCGCTCAGCACAGGCTCGTGCTGCCATGCTCCTTGAGCAGGAACATGAGCGTC
GACAAAAAGACATTGAGAAGCAGCTCTACGATGAAAACCATCGCTTGGCTCGTGAACAGA
AAGCACACCAGGACTTCCTCAACAATGACGTCTACACCAACAAACCAACCGCAGCATATT
TCATGCAGTGGAACACAACGACGCGATAAACGTTAGGAATAGATATGGTGCATTGATTTCTT
ATCCAATCAGTTCGATTCTTACCCATCTGCCATTTTGGTCAAAACTGTATAGCCACAAAATGT
GCATCTTGTCATTGCTTTTAGGAGCTTGATTGTTTTAAACATCATGTGTTTTATTTTAAGTTTTAA
GATACAGGACACATTGTTCTGATAAGAGAAATGAGAAATGTTTACCAACGATATTATAAACGA
AAAAATAAAACAAACTGTTTTTGATCTTTGCTGTGTACGATGAACAATTTAAGTAGGTTTATGTG
ACATATGTGTGTTACAAAGTTCTGTGATAAATTAAATGTTGAAACAGTTTTATCATTCTGAATCA
GATCCAAAGATTTAGAAATTATGCAAGCATTATGTATACCACCTACCATTGCGGCCAACCAC
ATAGGCTTTTAGGCAGTAGTATTAGTTGCTTGTCAAGATTTAAATAACATTATAATATTACTGAC
AAAAAGTTTTTTTTTATCATTTTAAGACTCTTGAAGACACTGTGTAGTATATAGCTGCATATGAA
AATGTTCTTGTATTTGTCTTCTCATTGAGCCATGATTCAGAACTATTCTGGCTCTCATGCATTC
GTTGTCACTGCCTCAAGGGAATGAAACCAACAACCAAAGTTTTTGGATGGTAGAAAGCCTC

AAAATCTACTTTTCACATTGGCTTTCAGTGGGTATCTTCACTGGTGGCCATAGGCGTAGACC
TGGGGGACAGCCCTGGCATGTGCCCCCGAATAATTGAGAAACGCCATGCTTTTAGCAGTT
ATTGCCACCTTTGTCCCAGTATATTGTGGTTTCCCCCAATATTTTTGACAGGTCTACGCCAGT
GCTGATGACCAACTAACAGTGCTTCAGATGAACATTTATGTGTGACCTTGCTTGCGTTTAAAT
GTTAATAATAATGACTGCTGTGATTCCAAATGAACATGGAACACTGAAGCGCTTCCAGTGGT
TGATCCAGACATTCGACTACGAGGGCCACATCATGTGGCGGATCCAGACATTCTGGGGG
GATTTAATATGTTTTTCAACATCTCATGTTTATTTCTTTGATGAAGGGGGCCAGAGTCTATAGC
AAACTGGATTGGGCCATGGCCGGATTTCCCCCTCTGGATCCGCAACTAAACGCTTCTGAC
TCATTATAGTTTCCAAGTTGCATTGCAGATTTTGTAATGTGTTTTATTAAACAAAAGTGTATGCTT
TATACATTAATACAATAAGTGGCTTTTGTAAAGTTCTTGTATGCGTATTGTGTCAACTTTGATACA
CTATAACACATCTGTGATCTTATAAAGTGTTGAGCTTCTGAGATAAAAAAAATGCTTCATGCTT
CACCAATGCAGGCAAGACAAACTTGTTCATCCGATCCCATGGTGACTACTGCTGTCCAGC
TCTAAGCTGAATTTTATTATTTAAATATTAACTGAGATATTTAGTTACACTCTGCTGCAGATTAATT
GCACCTGTTTGTTCAGTAGAGTATTTTGCTTAGTTTAAATTGTTTTGTGTCCACTGGATAATGC
CGTGTTTGAATTAGCAATACAAATGATTATGTTAATTTTATTGTAGGTTATTAATAAACACATAAA
CAAAAATAAACACATTGAAGTGCACTTAATTGCTACCCTAAAATAACTCAAGATGATATTTGTA
AGTGGTATCCAAAAATGATTTTGTATCTTCTGATTTTAGAAGGTGTATCTTCTGAACAGAATAG
GTCTTCTGAAAATGTCTGAAGAGGTCCATAACTCTTGTGACACTAATTCTATAAGGGCCATG
GGCAGAAGATCAGTTTTGGAATAAGACTTGTTAAGAGTGTTGAAAAGCTCCGCAGAAAAGA
CAAGTTTGTTAAAATTATATTTGTAAGTTGTGATAAAACCTATGGAGGACCTGGGTGAGGGTA
AATGCAAACTGGTGTGTGCCACCAACAAAGATTCCTTGTTTCTATTATTCTATTATACATAAAA
TTATATAGTTTATGGAATACTATTTTTTCAACGGATATTCTGAAGCACACAGATTCAAAATTCCT
ATGCATCATTGGGAGAGGAAGCGCTCTGTAACCTGCATAATGTTTTGTTTGCTTTTCATGGAA
CAATCGGAATGGAGGATATGTGAAATTCAATCAGTCGACAACGTAGGTCTGATGTTTTAATTT
TCTACTTGATCCATGAAATTTTGTTATCCTCACAGTGTTGCAATAACACATTCCTTGTTAGCAT
GTCTTGTATGCAGGATAGAGGAATGCTGTATAACAATAACCCCATATGGGCTAAAATTTAATT
GTAATCGAGGCCATATGGGAGTGCTGTCATATGGGCTTTGTGTGTCCTAAATACAAGTCATT
AGGCATGCAACTGGTATTATTGTAGGAAAAACTTAAAAATCTTTAAAATGTGATTTGTGCAGTC
CTCTGTCCAAGGGCCCTCAATGCAAAGATTCAACGAATCTAAATAACAATAGGCCTAGAGG
GCATACTTTTGTACTCTGCTAAAAAATTTGTCCACCTTAATTGTTAAACTGGCATTTCAGTATTA
TATGACACACACATTGAATATAATATGAGTATGAGTCATTTAATACTGAAATGCCAGTTTATATAT
ATATATATATATATATATATATATATATATATATATATATAGAGAGAGAGAGAGAGAGAGAGAGAGA
GAGAGAGAGAGAGAGAGAGAGAGAGAGAGGGAGGGAGGGAGTGAAAAGCTCTTTTATATAC
GTCCTAACATAATTATTAAGCACTATATTCATGCAAAAGGAGAAGTCCTAGGCCTACTTCTAA
TAAATCATAATGATGCCAAATCGAAAATACCTATGCTGACATTTCAAGGCTTATAACTCAACTA
AAAAAAGATAAACAAAGGTGTAGGCTATTCATTCAAATTTTCAACTTTCCAAAAACTGCATAC
AGAGGTGCCCTAGACGCTGTAACAGGTTATGTAAGCCTATTCTGTTTACATACGAATAATTTT
TTAAAAATCCATGGGTTGTAATTGATTAGGCGCAATATTGTTAGGCCTCGCCTCTATAATATC
GTAGGATACCGAGCTTAACTATTACGTAATCGTAATCGTAATCTCAAGATTTCTAAAGCGCTA
CTTGAAAGCTAAGCGCACCAGGGCACCAGCTTATTCACGAGCGCTGAGACGAATCAAAG
GGGGGATTTCAAAGGGGGTCAAGAGAAGCTCAGGTCCGGTTTCCAGAATACCATGAGGG
TACAGAGTAGCTGTTAAGGTGGGTGTCGTTCAGATGGAGAAGGTGAATGATCAGATGAGTC
AGGGTAGGAGTGTTTGAAGAGATGACATTTTAATTTTGATTTTACTTGGTTCAATTTAGTTGCA
GGGCTTAACTATATATAGTAGGCCTATACATGGTTAGGCCTAACGTTTAAGACATCGGAAAC
CTCATCCAAATTATGCCCTTTGACCCGTAGGCCTAGTGTTTTCTTCAAGGTTCAGTTCACAA
CTCTTCGGGAAAAGCCTTATAAATCAAAGAAGCGAGTAGGCCTAAGCGTAATCTATTTCAAA
CGTGAGCCCTATTTCAACTAGAATCAGTAGGCATAATCATTCCACGCACAATATTTAATTGGA

AAATTGAATATTGCAGTAGGCCTATACCCAGTCAGCACTGAAATTTTGCCCCATTCATTGCC
CGAATGGGCGTTTAGGGCAAAACCTGGGCTTTGGCGGATTTGCCTTTGCCCTAAAGTTGC
CCGAATTGCTGAACACAAATTTGGCAGCCGTTAGGGCAAAGTTAGGGCAAACATCTTGTGT
CCCTAATGGGCCCCAAATTTGGTGCCCAATAGGGCAATAAATTGGGTTCCCAGCTGGGG
CCAATGAGCATAACCAAGCTCTCCCCTAATTGGGCTTGCCTGGGTAGGGCCTATAGGGC
CTAATATATTCGCTGGTTTTACATTTAAAATCAGAGTCGGTAATCATTCGATTAATTTTTATTCTG
CGGCTGGATCTACGAAACACCACAACTTTTATTCTTTGCTTACGTAGGCCTATATCGTTCTG
ACTGACAAGAAGCCTCGCATTGATCCGCTTATCAAAACTAAGCACGTCCATCTTTCCCTTC
TAAATTTGAATGTTTTAGTTCTCGTGCTCTATTTTGACAGGGTTTCAAACATAATATAAAACGTT
GTGTGATGAGTTTATACAACTAGGCCTATATAGGCCTACCAGCGTGTATACAAAAGCTTTAC
CATTACAGCCCTTAAAGGAATATTTAGCCTAAATTGTTATAGTGATAAGAAACTGAACTGTTTA
TATTTATAGTCAAACCATGGATTAGGCTAGGCCTAGCAGCAGCAGCTTTTAACTTAAGCTGG
ATCCGTGCATAAATAGGCCTAAATATAAAAACAATGTGAAAAGGGTGAAATATTTCAAAGCC
ACCCGAAAAGTTCTTAAGACGTTATGTGGAACTGTGATGTAAACTGTAGAAGCGATTTAGTTC
GGTTTAGTAACTTTAGCAGCAAAATCCAAGAATCTAGATGCTGTATGTCGGCCATAGGCCTA
TAGGCCCCTAAGCTTATATTCCTTCCTGGCAGGCCTAGCCTATAGCCCTATATCTATAGTGG
TCAGCAAGCAAAAGGATAAAGATGGCGCCAGTTCGTAGCCTTAAAAGTTTAAGAACCAGTT
CTCCTAAAAGCTGACGAATACCCATTATTATTCGTCTCTTTCTCTTTATCTACTCAGAGACGCT
CTCTGTGCTAGCCTATACATAATAGCTATACAGTGCTATACATAATAGCCTATGCCTAATGGC
AGATGGAACACATGTATTCGAAAGCAGGGACATATGAATGTCGTCTTTGACGATGTAGTCGG
TCTAGAACTATAATAATTATCTGCAGAGGAATAATCTGATGAAAATTTTTCCGCTCAACGCCTT
ATTTAACAAACAATATCGGACTAGTAATAACAATTACGGTTAAAACTGACTAATATGGTTTACA
TCAACCTGTGAAACTTAGGAAAGCGACTGTAGGGCCTACTCTTACAACGAGTGACACCAA
AATTCATATAGGCCTAATGGTCGATATTTTTGTACAAAGAAACGTTGATTCGCAGTTACACTAT
GTAATTTCACTTTTTTCATAATCGCCGTTAACTTTCGAAAATTCCCTAACACATGCTTCTAAAT
GCCGACCTCATAAAAACCCAACCAAGAACTTATGGGGATGCCTCCTTGCTAAGCCCGCT
AAACCAGGGGTTTGTGGGGTTTACCGCCATGCCACACACCCAATTAATTTACAAACCAGA
AGCTTATGAGGTCTAAAACCACTCCCACACACCCAAATTAAAACCAGGAGCTTAGTCCCTA
CCATTCCAACACACCCAACTTAAAACCAGGAGATTATGAAGACTTATTCCATACCACACAC
CCAACTTAAAATCAGGAGCTTATGAGGTGTTATTCCATACCACACACCCAACTTAAAACCA
GAAGCTTACGAGGTCTTATTCCATACCACACACCCGCATTCTAAACCAGGAACTTATGTCC
CACGGGTTCACTTACTCACGATCAACTACTAACGCATGCACACACATTCACACCTATACAC
GCACACACACACACACACACACACACACACACACACACACACACACACACACACACACAC
ACACACACACACACACACACACACACACACACACACACACACACACACACATGTAGTCCTA
TTGCAATCCCTAACATACGCTTCTAAACGCTGTGAATCCATTTTATCTCCCTTCAGCCAGGA
AACTCGTCGTCAGCAGATCCACGGTCATCTATAGAAATACAATAAAACAACTAAGCATATTA
GGCCTACTGCATATTACGACAGATGAAAGGAAAGAATCCGATACAGTTCGTTTTTGTCAGAA
CCGCACGGATATAGACTACATCAGGAAGCTTTACGCAGGGGCGTCCAATGCATGCATTGT
GTAATGCATAATGCATCGTGCATGGTCAAACTAGGGAAAGGTAAAACACGTAAAATATGCG
AAAAACTCACGTAAATTTCACGAAATCAGAGAAAAATTTTAAAACGTAAGGGGGAAATAAAA
CATTTCCCGAAATAGGAGAATGTAGCGTTTTACCGAAAACAGGGGGAAATTCGAAATTTTC
GTGGATGAAACTTACATTTAGGGGGAAAGTTTGAAAAATTTTCCCAGAGTCTGACAAATTCT
GAAAAGAGGGCGAAATCTGAAATAGGGGAGGAAATGCATCATTGGCTTTAGGGGGAATGG
ACGCCCCTGGCTTTATGTCAAGTCGAAAGTCAGCTGCACGAGAGGCAGCTACGAATATGA
AGTGTTGGCGACTTCGCCTTCTCAACCTCGGTATAAAAGTACTCGGGGCTCATGGCCTCC
CGCTCTACACACCGTCACCAGAGCGAAAACCATAGCACTACTCCTATGCGCCTCGTCTG
CACATGGTGTGATTTGCCGAGGGGTTGGGGGTCGGACCCACCCTTTTGTACTGCTCGATC

CTCCCACTTTAGTGTGTAATAAATAAGTGGAGGAGTCAGCGTCCGACCCTCCCACTTCGAT
CTATAGGGCTTAGCTAACAAAAGTTGGATCTTTGTATCACAGCTCAATGCAATAAATAAATAAT
AAAAATCAATTTAATTAGCATTACATGCTCAAAGATTCACTGAGCATCTGAGATAGTAAGTCTA
TAGGCTCCCCAAGACGAATAATACTTATAATTATCACAAAAAACACGGAAACACGGAAATAA
ATCAGAATAAATTATGTCGAGGGGACTACAGAAGTACAGAAGCCTAGAGGGCCTGTCATG
GATTAACACGGCATTGGCACTGAGAACACTGTCACTATATATACTCTATCTCCAAAGTGTTGA
TGCTAATCTGATGAGCGTGGGAAGGCGAATAGAGCTGCATGCAAAAGGTTTTCTCTCTAAC
TAGGAAAACTCTAAAAGGAAAAGCCTTGTACAAATATAATGCATACTACACTGCAACTAAATC
TTTTACGGGTGTAATGTTGAGACAATTGAACCAATGCAGATCAGGCACATTTAAATCTACTAA
GAATCAATTTTCCAAGCTTTACAGTAGACCTATATGACAACTTAGTTGAACGTTTTCATAACAC
CGCAATTCTTACAGCTGCGTAAGACGCTGGATGTAGAGCTCTGCGCCGGCCGCGCCGC
CGCCGCCGATAATTTGTTGGCGCGCCGTCGATGCCATTGTTGCTGTGTACTTATATTGGTA
GGCCTACTATAATTTAAATTCGCCCCGAATTATTTTTTTTTATAGAAGTATTATAGTGATGTCAC
CTACGTAGGAATCCTCAAGAATACGTTATTAATCTAAACTAATGTTCCTAAAGTGTCAAAATGG
AGAAATGCGAACTATTAGTGCGCCGGTGACCAAAAAATGGTTCAATCCATGTAGCGCGCT
GACGCCGTTGTATAGGAATATGGTACAGGGTTTCATGGATAGGAATCCAGCGGACCTAATA
AGGAAATAATTCGGGTGAATAAAAATTATAGTAGCCTTATTTGTTGCAAAAAGGTCATCAGAA
AATTTTGAGGATAGAACGAATTTTTTCTTAAATCTTTGAAAAAACATTTTTTGGCCGCCGCCG
ATTTTTTTGGATCGGCTGCGCGCCGCCGCCGACCAAAATTTACCGGCGCGTCGCCGCC
GACACAATGACGTCGGCGCACGGCTCTAGCCGGATGTGAAATTATTTTGCGCAGGATTGT
GTGGCATATTTTCAGTACGTAAATCATGGGGGGACAGGGAGGGACTGTCTACAGTTTTGAC
TGGGTGTAGACTGTTCCCCCAGCTCTGTCCACACCCAATTTATGTTTATGGGCCCTCTGTTA
TTTCATGAATCTTGGTTCTAGTTAATAAAAACTTCGATCATCTACTATTTTTTGATTTGATTACTAC
AGCGTCTCCAATGGATACGCTGTGTTAAGCCTCCATCGACGTCAATCTTATTAAGGTTAACA
TGAAATTTAGCTACAAAGGGTCACGCAGCTTCGGCGCTCGAGGTTTACTGGTCCGCAACA
CAAGAAAGTTTGAGAAGCACTGCACTAGTATAACACTGTCTTCTGTGATCTTATAAAGTGTTG
AGCTTCTGAGGTAAAAAGAATGCTTCATGCTTCACCAGTACAGGCAAGACAACCTTGTTCA
TCATAATTATGTTCGGCATAATTTTGAAGGCCTGTACAGTAATCAACCGTATAATCTTACTGTA
GACAGTGTACGGTCAGAGTACTCATGATAGCTGAGTTGGAAGCTTAGGCCAGAGTAATTTAT
TTAAGCTTAAGAGTGGTTAGCACACAGAATAATGTAGATTACAGATGACACAAGAAGTATTGT
ATTGCCGTTATGTCAAGTAAATTAAATTTTAAGAAACGAATAAATTTCCTCCCAGGTACCTGTT
GGAACCGGCCTTTGATCAACAAACAGAAAGGCTTTCTTCCTTCAGCTCCAGCACAGTCGT
TGAAAAGCGAGCTGGATCTATCTGGAGACAATCATCTCGGGGCTGAAAAACTGTGAGAAC
GCGTGCAACAGCGTTCCACCCACGGAAACTATAGGATAGAAAACAGCCAACTTCGAGAA
ACCAGAAATCACCGAGAAAAAGTGACGCACATGTACATGTATATTTCTTCTAAGATTTGAAG
ACTTTGGATTTACTCTAGTACATGTAATTGTAATTAGTAATTTGAATCACAATAGGTGTCAGACA
CAAACCGAAATCATTCAATCCATAGGCCTAATGGGCTACATAGACCTATGTATACGCGAGA
CAAGATAGACACACATAAACACACACATGACTTAAATATTGTACTGTATTTACGGACAGGCC
TATGCCACTGCGAAAAGCCCCGAGAGAAAAGACAAGTCTTAAAGAACACTGGAAGAGAC
GAAGTAGGTTTACACCATTTTAGATAGGCCTAAAAGCATGAGTCGCAGATGGAAGACTATAC
ATACATGTATAGGTCTATTCATATAATTTATGTAAATACGCATGTCAGTGTATATAATTATGATATG
TGTACATAAAATTATATATTATATGGCCTTGGTCAGAAAGGCTCCTATGTGGCTACATTAAAGG
ATCGCTATGTAAATGACGGTGTCACGAAATAGAGTATCTTCCGGAAATAGAGTATCCAGGG
GACAAACTATTTCTATCCCCATCGTATAAAATCACTTCCAATCGACAAAACGTTACAGGCATT
AGGTAGATCTCTATTTAGAAACCTGTATAATTGATAGTTGGATGCCGTAATCAAAGTCCAGAC
TCAGAAAATACACAGGCAAAGCAATTGTTTTAAGTTATAAGCCATTTTTCAAACTGTGGTCCA
CGATCAATAGACATATATCATATGTGTGGTGCGTGGTGGCTCAGTGGTAAGTTAGGTGTCTTC

CTTACGGAGGGTCGCAGATTCGAATCCCACTCCAGCCCTCACGCAGGGACCTTGGACA
AGTCCAACACTCAATGTGAAATCTACGATTAAGATAAGGTTCAATCCAATTGTTTACGTTTTAG
AGATAAAGGTTGTCACCGTTTGATATGGAATCGAAGTATAATTCTCGGACACAGTCAGTGCA
ATCGAAATGTTACCACAGAACCAATGACTTATATTGTATAGGGAGACCTGGTAGACTAATAC
GTATCTGCTTTTAGGTTGACTGCGAAAGCAACTCTAAACAATTGTATTCTAGAAGCATATGCG
AATTCCTCAAATGTCGTGGCATGACTTTGAATTAATCGACGCACAGACAACAAACCCGCC
GTTGAAGTTTCCCTACCTCCTCTCTCGAGATCTAGTTTCTCGTACAACGGAAATAGTAAAACT
CGCCAGTATCAGGTAAATATCTGACACATCTTACAAATTCACTAAAATCCTCATCTCTTGGCG
AATTCCTCAAATGTCGTGGCATGACTTTGAATTAATCGATGCACAGACAATAAACCCGCCTT
AGAAGTTCCCCTACCTCCTCTCTCTAGTTTCTCGTGCAACGGAAATAGTAAACTCGCCTGTA
TCAGGTAAATATCTGACACATCTTACAAATTCACTAAAATCCTCATCTCTTGGCGAATTCCTC
AAATGTCGTGGCATGACTTTGAATTAATCGACGCACAGACAATAAACCCGCCTTAGAAGTT
CCCCTACCTCCTCTCTCTAGTTTCTCGTGCAACGGAAATAGTAAAACTCGCCAGTATCAGG
TAAATATCTGACACATCTTACAAATTCACTAAAATCCTCATCTCTTGGCGAATTCCTCAAATGT
CGTGGCATGACTTTGAATTAATCGACGCACAGACAATAAACCCGCCTTAGAAGTTCCCCTA
CCTCCTCTCTCTAGTTTCTCGTGCAACGGAAATAGTAAAACTCGCCAGTATCAGGTAAATAT
CTGACACATCTTACAAATTCACTAAAATCCTCATCTCTTGGCGAATTCCTCAAATGTCGTGG
CATGACTTTGAATTAATCGACGCACAGACAATAAACCCGCCTTAGAAGTTCCCCTACCTCC
TCTCTCTAGTTTCTCGTGCAACGGAAATAGTAAAACTCGCCAGTATCAGGTAAATATCTGAC
ACATCTTACAAATTCACTAAAATCCTCATCTCTTAGCGAATTCCTCAAATGTCGTGGCATGAC
TTTGAATTAATCGACGCACAGACAATAAACCCGCCTTAGAAGTTCCCCTACCTCCTCTCTC
TAGTTTCTCGTGCAACGGAAATAGTAAAACTCGCCAGTATCAGGTAAATATCTGACACATCTT
ACAAATTCACTAAAATCCTCATCTCTTGGCGAATTCCTCAAATGTCGTAGTATGACTTTGAATT
AATCGACGCACAGACAATAAACCCGCCTTAGAAGTTCCCCTACCTCCTCTCTCTAGTTTCT
CGTGCAACGGAAATAGTAAAACTCGCCAGTATCAGGTAAATATCTGACACATCTTACAAATT
CACTAAAACCCTCATCTCTTGGTATTCTATATGTATCCAAAGGTCTTTCACTCAAAACTGAAA
ACACTGCTTTTCACAAATCTTATCCGGCTTTCTCATCTGCTCATCTCCTACCTCGCCAGTTC
AGCTCTGAAAACTATCCACCTCAGGTTCTCAGCCGTCTGAATGCCTGACTCTGAGCTCTCG
ATTGCTTTTGACTAGAAGAATGATCTTGAACCAGTGTCTGGATTTTGTTCTGATTAAACGACTG
CGAATAAGCTGGTCCCGCGATTTCGCTTTTATGGGCGCTGTAAAAATTTCGATTTCGATTCC
GTGAACAAACTGACCATGCCTGATTTGATGCCGCACATGATAATATGCCAAGTGAGAGGTC
CGAAGGTCAAATCCCGGCCAAGGCAGAAATTTGGATCGAGAATTCTGCTCGTGCGTAGG
CCTACCCTGGTGGGGCGTAGAGTAGCGGATAACATTTGGCGCAAATGCTGGTAATGAGC
CAATGTGGTTGCACATTTAACTGGTTGAATTTAATTTAAATATTCTCACGTTTGATGTTGAAATC
TTGCTTCCAGGGGTATAGTCGGGCCTGGACGGAGGCGGACTATCGTAAGGCGTCCACCA
ACTTCTCCCCATTGCTCGCTTCCACTGAGCAGATTTTCAAAATTCAATTTCGCTTTTGCTGAT
CGCTATTAGTTTCTTGCTGCACCTCAGCCACCAAGAAGAACAGAGGCTAGCTGCACGAAG
ACATCCGTCATCTGCATTCTACAAGCAGGCAATCGTTACGTTACTATCGTTAATTGCCAGATT
ATAATTATACTGATAAGTCGACCAAAATATTCATTTTTTATATTCCACGTTTATATGCATGGGCG
ATATATTATTATTATAACGAGAACGGGGAACCTACTTTGACCATGGTGTATTTATTTAGCATGCT
TTACACGTACTGAACGCCTCTGACTTGGTAGACTAATAATATTATATGCAAATGTCCGTAATGA
GCGAATGCACATTAAACTGGTAGAATATAATTCAAATATTCTCACGTTTGATGTTAAATATCTCT
TCCAGGGGTATAGTCGGGCCTGGACGGAGGTGGACGTTGTCCACCCACTATCGTAAGGC
GTCCACCAACTTCTCCCCGTTGCTCGTTTCTATTGAGCAGATTTTCAAAATTCAATTTCGCTT
TTGCTGATCGCTATTAGTTTCTTGCTGCACCTCAGCCACCAAGAAGAACAGAGGCTAGCTG
CACGAAGACATCCGTCATCTGCATTCTACAAGCAGGCAATCGTTACGTTACTACCGTTAATT
ACCAGATTATAATTATTATAGAGATGATAAGTCGACTAAAATATTTATAATTCATAATCCACGTTT

ATATGCGTGGGCGATATATTATTATTGTATTGTATTGTATTGTATCTATACATTTATATAACGCTTC
TTGCAGTGCACACCAATCAGAAGCGATTCCAGTGCGAGAGACCCAGAAAGAAGAGATCA
TGAGAGAAAGAAAAGAGGCACTTGTTGGCTCACCAGTTAATAAAGTGGATCGCGTAGAAG
AAAGACGTTGGTTCCAAAGCGCAGGGACAATGATTGCAAAGGCTCGTGCATGTCTGAGC
CATAGAAGTCCTCGCTCGCGGGACAAATAGATCATGACGGTCAAGAGAGATGGCAGAGG
AAGGCAAGCGGATAAGGTCACGTAAATACCTGGGAGCTTGACCAATATGGGAGCGGTATA
CTCCTAAATCAATATGAGGACCTTGAGTTGTATCCGAGCAATGAGTGGTAGCCAATGAAGAT
GGTCAAACATGAATGCAGAGATGTGAAACGTACGAGGAAGTCGGTTAATCAGTCTAGCAG
CAGCGTTTAGAACTGACTGAACAGGAGAAAGACGAACCTTTGGAAGACCAACCAGAAGA
GAATATTGCAACGAACACGGGAACCTATTTTGACCATGGTGTATTTATCCATCATGCTTTACA
CGTACTGGACGCCTCTGGCTTGGTAAACTAATAATATTAGATGTATGTTTCTACTTCTATGTTG
ACTGCGAGAGCAACTCTACAACAAACTGTAGGCCATATGTAACATACATGTATAGGCCTACT
ATGTGAATTCCTCACATGTCGTCGCATGAAAAAGGCGCCACCACATCGTGGGACGTGATG
GCGCCTTGGTTGAGACGATGACTTTCAACCGGAGGGTCGTGGGTACGACTCCCGCTCTA
GCCGCCACGTAGGGACCTTGGACAAGTCCTTTACCTGCAGTTACCTGTGCGCTACGGCG
TGAAACTCCGATACAGTATCCGAGCTGTAGTCGGGAGCGCCTCTGAGTAGTAGAGGACTT
GAAGGGGCGCTATAGAAATGGCCGAAATTAATGAATGACTTTGAGCTACTAGACGCCCAG
ACAACAAACCCGCCGTAGAAGTTCCCCTACCTCCTCTCTCTAGTTTATCGTGCAACGGAA
ATCGTAAAACCCGCCAGTATCAGGCAAATATCTTACCCATCCTATGAATTCACTAAAACCTC
ATCTCTTGGTATCTATCGACAGGTCTTTCACTCAAAACTGAAAGCGCTGTTTTTCACAACTCC
TAACCTGATTCCTCCTCTCCTCTCTCGCCAGTTCAGCTCTAAACACTAGCCACCTCGGCC
GTCAAACTGTCTGCCTGCTTGACTCTCTGGACCTTGAACCACTGACTATCGATCTATCTTGT
TTTGATTAATAAGCTGGTTTCCGCGACTTCGCTTTTGTGGGCGCTGTAGAAATTTCGAGATTG
AGGTTGCGATTCCGTGAATATACCTGATTTGACGCCGCACTTGATAACGAGCTTAGCAAATT
TTGAGTTTACGATGATAGAACATGATTTTATCGGTAGCCGTTCAAAAGAGCGACAGATCGAA
AGAGCGACATACCGAAAGAGCGACATAAATACTGTATCAAAAGAGCGACATCGTCTTGAG
GGTAGGTTAGTGTCGGGTTAGGCGTCGTGAGGGTCATTGAGGGTCAGGTTAAGCATCGTA
AGGGTCGTTTAGGGTTAGTATTAGGGTTAGTTTAGTCATTAGAAACACAATTTTCAGCTCAAAT
TGGCAATATTTTTGTCGCTCTTTTGAAACTGTTTTCGATTTGTCGCTCTTACGTGATGTCGCTC
TTTCGTTCTGCCGCTCTTTCGATGCTAACTGATTCTATCGGATTAGGCCTACTTAACTCGGTG
CAGGGTTAAGTCAAATTGTCACTGTTTGACGTTGCAGGATAACAACATTCCAGGCTATATTG
ACAAGTGAAGGGTAGGCTAGTCATCTGTAGATGCAGTATAATAATAAATATAGTTTTTTGAAAC
ATTAAAATCAAATCTACAGAGTAGACATCGTTAACACTGTCCATATGAGCTTAATGGGTTTTAA
GCTCACCTTAACGAGTGCTCCCTCCAGGGTGTGGATGCATATCGGGATTAGAAGAGCAAT
TGCCGACTCTTCGTCCGCATCCATAACTTTTCACGTTTTGGTGAATTGGACATTGATGATTAT
CGGTCCATCCAATTACGTCCTTTTCGGTCTTTCTGCTTTCATATCGCTCGTTCGAATAGTTGA
ATCTGATGTAGGTTGCGTTGCGAAGACCGGTTCTGTTCTCATCATCAATATAATCTTTGAATTA
AGAAGTTGACGTTTCGTTTTCAGCGAAGTTGGACCTCTTCAGTGTTAACGAGGTAGTTGGAT
TTCTTTTCACTTCTCAGCGTTTTCTAAGCTCGCCTTAGCGAATGTTGTCTGCAAGCACTCGAT
ACATCTGAATTAGAAGAGTGTTGCTGGTCATCTCTGTGCATCGTCCACATCCGTAATTTTTCA
CGTTTTGTTAAATTCGACATTGATTTTACTTTAGCGTTTATCAGTTCATCCAATGACGTCCTTTC
GGTTGGTCTATCTTTATGTCGTTCGTTCGAAAGCATTGACTTTGAAGATGGTCGATTCCAATTA
CGTCCTTTCCGATCGGTCTGTTTTCATGTCGCTCGTTCGAAAGCTTAGAATAGTTGCATCTG
GTGTTGAAGTTGTGTTGTTAAGGCCGGTTCTGTCCTCATCTTATGTAGGATCTTCGAATTCAG
AAGTTTGAGTTTCGTTCTCGGCGGAAGTAGAATCTTTGAATACACAAGTTGAAGTTTCCTTTT
CGGTAGAAGTAGGATCTTTGAATTCAGAAGTTGAAGTTTCATTCTCAGCTGGAGTTAGACTTC
TTCGTTTTTAAGGAGGTAGTTAGATTTCTTTCTCTGGTGTAGTATAGTTCATTTCTCAGCGTTTTT

CGTTAACGTCGTGAATAGAATGTTTTGATTCAAGATAACGACCACTTTCATTCTCAGCATTCA
TTTATTCATTCATCCATTAATTCATTCACTCACTAATTCTCTCATTCATTCATTCCGTCCGTCCG
TCAGTCAGTCAGTCAGTCGTCAAGTGTCTCAGTCAGTCAGTCAGTCAGTGGATTGCGTTCC
CTGTCCACTCCCAAGTCAATGCCGTGGAAACCCAAAGACAGACCATGCATATTCCAAGTG
GATAGCGTTCCCTGTCCATTCCCAAGTCAATGCCGTGGATACCCAAAGACATACCATATTC
GACGTGGATGGCGTTACGTATCCATTCCCAAGGTAGTTCCGGGCGAACCCAAAATCATAC
CATATTCCATGTGGAATTGTTAAAAGGCGTTCCCTATCCATTCCCAGGCTAATCCAGTACAA
TACCAAAGGGTAGATTATGTTCCACCAAATTGGTGTACCTTTTCATTTCCTTAAGATTCGCAT
GGATAATCCCAACTTAAAACCCTTGTAGACCCAAAGGAGACAATTCCACGTGAATACCAAA
GGAACCACACTATCCTGTATTTTAGAGGCTGACCCAGAGAGCGTTGTTTTCGCCATGACTA
CTACTTCCACTTCTATAAGGGATAAGGGGCATCGCTCTCATACTGGGTGGGATTATTATATTT
TGTTTTCAGCCTGTGTAGGTGGGTGTTGAGGTGAAGGAGGTGGTGGAGGTGATGGAAGGA
GATGGGGAGGAGGTGGAGGTGGAGGGAAGGAGGTGGAGGTGGAGGTTGGGTGGGTGG
TGGAGAGGTGGAGATGGAAGTGGAGGACGGGTGGAAGTGGCGGTGGTAGTGGAGTAGA
CGGTGGAGTAGATTGGGAGGTGGAGTGGATGAGGATGAGGAGGTGACTCTTTTGAGTGCG
ATTTTCCGTCGATTCTCTGACTCAACTGTTGATTGACTTGAGTTCATAAGGTAATTCATTCGAT
GACAGCATCACCTCTTTGTCGTGTTTTGTTTTCTGAGGTAAGTCACGTTTAGTGCAATTCATT
CGAGGGTATTGCTTATTCTTAGTCGCCATATCTTTCGCAACTAAACTGGGTTCTGTGACAGAT
GTGGGGGAATGGAGGGGAGGAGGGCAGGTGGAAGTGGAGTAGACGGTGGAGTAGATTG
GGAGGTGGATGAGGACGAGGAGGTGGCTCGTTTTAGTTCGATTTTCCGTCGATTCTCTGAC
TCCAATATACCAGTTACTGTTGATTGAATCCAGTTCATCCGATGAGTCATTTGACGACAGCAT
CACTTCTTTTCGTGGTTCGGTGTTTGAAGTAAGTCACGTTTAGTTTGCTTGCATATAGTAGCAT
ACATTTACGCTGGAAGGGTCTCTTTAAAGTGTCCATTGTTTGGTTGGCGCGATTTTCTTATTT
GCCGATGCAGAGAAAATGAAGTTGTCAGCAGCGAGCCGTTCACGTCTCGATTGGTTGAAG
ACTGATCTCTCATCTTCATCTTCTTCGCTTTGTTGAATAACACTGCAAGGAAGTCAATAAGTTA
ATCGAATGACAAAATCCTCTTTGCTGCAAACTATCCTATATTTACGCGCAGAATATCTGCGCT
CTAGTTAGGCAAAAAATGTTACTTTTGGGATACCCTATGTTCCTGTTTCTCACTCTCCCCTAA
ATACTACTCCTCTACCGAACATTACCGAATGACCAAATGAAAGACAGTCTCGTACATTCTTA
CTAAACGGCCAAGTCTAGCAGCTCTGCACAAGGGCGGTACCAGTTACAAACTCAGGTTGT
CATCAAATCACTTATCAGACTATCAGTAAATACAGAGACCGATAAATTGGCAACTGCAGAGT
GATAGGCCCTTAGTTGGATCTATATTAACTTTAAATTCAATATTATGTGATGCGATTGAGCAATG
TAATCCAGTTTACAACCATCGGATGGAGAAAAATTATCTGCTGTTCAGCTAACTCCACAGAT
TTATATGCAATCATTAACCCGGAAATAACACAGGCAGACAGACAGGTGATCGACCATCGAC
TGATCTCTTGGCCATTAAAATAACGGCGCTATGGAAGTCATTCTATTACAACAGAAGTGCAC
GAATACCTTTAGCCTACATAGCTTGATATTCAAATTTGGATCATTATCTTAATCGTAATCGTAAA
TCGTAATCGTAAACTCCTGATTTCTAGAGCGCCCACAAAAGCGAAGTCGCAGGAACCAGC
TTATTCACAGGCGCTTAACCAAAACAAAAGCAGGGGTCAAGATCCAGAGAGTTAGGCAGG
GGTCAAGATCCAGAGAGTTAGGCAGGGGTCAAGATCCAGAGAGTTAGGCAGGCAGACA
GTTAGACGGCTATGGTGGATGGTGTTTGGAGTTGAGACAGGGAGGTAGGTATGGGGATGA
CGATGAATCAGGATAGGATTTGCTAAAGAGCAGTGTTTTCAGTTTGGAGTGAAAGAGTTGTG
GAGAGATGGCAAGGGGAGAGGTTTGAGTGAGCTCGTAGGATGGGTCAGACATTTGTCGC
AATGCTGGCGGGAGTTTGTTCCAGAGAGGCGGGACAGCTACGGCTATGGAGCGATTGGA
AAATTTGAGTGACGAGGTGACAGAAGGGCGAAGTAGTGTTAGAGTGGATGAGGATCGGGT
TGAACGAGGTGGTTGGATGGTGAACAACTGACGTAAGTATGAGGGTTGGGAGGATTGAAGT
GTGTTGTATGTGAGTGATATGACTTTGTATTCTATTCGTTGAGGTATTTTGAGCCAGTGGAGTTT
TTTGAGAACAGGAGTGATGTGGTGGTGTTTTGGAGTTTTAGTAACAGCACGGGCGAGAGCA
TTCTGAATGGCTTGGAGGCGGTTTATTTGGGTGACGTCTATGTTGAGGAAGAGAGAATTACA

GTAGTCTAGTTTAGCGTGGACTACAGATGTAGCGATGGTAGATGCAGTTTTTAGATCGAGCA
TAGGGCGAATTCGGCGAAGGTCCCGGATGTGCATCTTGGGCCTCTTGGCCAACGAGGA
CAAACGGAAGAAGCCAAATATTCAAACGAGTAATTATGCAAATGTTTATAGGCCTATAATATT
CTAGATACGGCTATTAACCCATTATAATTGAAGACAACAGCCACTTGTTAGATCAGCGGTTC
TCAGCAGGTGGGTCGCGACCCCCGCGGGGGTCGCTTGACGATTTGCCGGGGATCGGC
TGGGATCTTCAGAAATTTGTGAACAAAGTGTATATGAATCTATACAGCTTATTGGCGCGTCAT
CAACTCTGTGTCAAAAAGGTTTCCAGTTTGCTGGTTCTTCATCCAATTATTTGCTTCCTAGGC
AGGTTCATCTGGCCAAAGCCGCTACTCATCTCACTGCTGGGCCTCATCTTTAGGCTTTCCA
TATACAATAAAACAACAGCCACCATCCGATAGGCCTATCGCATTATTTTCTTTCGATAACTAT
TCTTAGTTGTGAGTCTCCTTCCAGGGTGTGGCCAATCCATTTCTTTTTTCTTTTTTGTTAAGTG
CACGTATTAAAGCTCTTTCTTCCCCAATCCTTTCCACCACTTCTTTATGTGCCTTATGTTCCG
TCTAACTGATTTTCTATATCTTCTCCATGGCCACATTTTAATGTCTTCTGACTCTTCTCCTTCAC
GCTTTTCCATCAGTCCATGTCTCTGCTCTATTTGACCATGCTCTATATCAGAGTCTATATCATT
CTTTTCTTCAGATTATTGCTAAACCTTCGACTCAGTAGTTAGCCCATCTGTAGAAAGTGATTTG
ATTTGTGTTCTTGCAAAGACTGCCCAGAGAATTTCCAATCCGATGAGATAGAAGTTTTGTTTT
GTGGTAGCACAGCCTTCTCATTGACGCTCTTCTTTGGATTGCGGAACATTCTGTTTCTTATAA
TCTATTCTATTTATAATTGAAATTATCTTGTTGGCTATCTGTAGCTTTCTTGTCTTATTAAATCTGA
AGTAATGTTAAACTTAAAAGTTAATAGCTGCCATTGACATTTTTTTAAATCTAAAATTAGTATTAA
GCTTCAGGTAAACATCTCATTTACTAAATTGTACCTTTAGAATTTTTAAATTATTGTAAAAATTGC
AAATTGTGTCTTTTTCTTATTAATGGCTGGTTACGCGGATGGATTAACAAAATGGTAATTAGTG
AAATGCATCATGAATCATGCTAGCAGAAATACGGTGAAGAGGAATAGCCCCATCAATATAG
ATTCCATTGAAGTGTCCCAATCAATAATTCTATTGCTGACGATCGCCACAACTTTTGAAAAGT
CATCAAGGGGTCGCAGCACTGTAACTGTTGAGAACCGCTGTGTTAGATAATGCCATCTTTT
CAACATGAAACCAGTAACATGAGAAGCTAATAAAAATTTGTGACGAAAGCTATTAGAAAATG
AACTGAAATGCACCTAAGACTTTCAAGGAGTAAACGACCTTTGTAACTAAGAAATGTAATTAA
ACATTGGAAAACATACATTTCATGAAGACCGATTTCGAGTCAACAGTTTCGTCTTTTTGGAGT
CTTGAGTTTGAAGTAGTACGAGACTCAGTCGGTAGTTCGGTGTTTGTCGAATGTTGGCGCC
ATAACATCCGAAGAAGATCAAACGGACAGGTCGTTCGGTTCTCGAATATGTCAAGATTGGT
CGTCATTTTATATATAGTTCTCTCTCTCTCTTTGCGTGATGTCGTTTATTTCGTCCTATCTCCGG
CGAATTCTCTCTCTCTCTTTGCTTGTTGTCGTTCATTTCGTCCTCTCTCCGGTTAATTATCTCT
CTCTTTGCTTGATGTCGTTTATTTCGTCCTCTCTCCGGTTAATTATCTCTCTTTTTGCTTAATGTC
GTTTATTTCGTCCTCTCTCCGGTTAATTATCTCTCTTTTTGCTTAATGTCGTTTATTTCGTCCTCT
CTCCGGTTAATTCTCTCTTTCTGCTTAATGTCGTTTATTTAGTCCTCTCTCCGGTTAACGTTGTA
TGCCTCGTCCTGCTGTTGCAGTCCTGTTGTTTTTGTCCTGTTGTTGTTTTTTTGTTTTCTATCTC
TCTCGCTGATTATGCTTCGTCAGGCAGTAAGCTTAGTTGTCTCTCTTCCGGCTCTTTATGGTT
GCTTTCTGCTTCGTCCGGTGGAAATTCTCTCTCTCTCAGGTTGATGTATATTTCGTCCTGCTG
TAGTTTCCCTCGTTTTCTCTATCTGCTTCGTCAGGTCATATACACAGTGCAGGAGAAATGTTC
TCATGATTCAGCCACAATGTGATAAGTCCGTTCAGGAGATATGGCTGTTCAGTCAGCTCGAA
ATATAGACCGCGCAGGTGGCTTGCATGTGAATCGGTCTACTCGGAATACAGACTAGTCGA
GGGACATAACCCGCTGATACAATCACTTCATAGTACAACCAGTTTGGGACATAGGCCTATG
ATGGGTGGATGACTCAGTTCCATTGTAATAGAGAATGTGCTGAAGATTATTATGGGCTCGAG
AGTCAGTCACATCGAAATACAGTAGGCCTACACGTTCGGGCGATCTGGGCTTCGTAAAAC
CGACCGTGCAAGAGATTTCCGTCAAGAAGCTGATGTTGAGTCGGTCACTTCGTTATAGACC
TACAGTCCGTTCTGAGGTGGGAGGCTGATAGTTCAGTCACTTTGTTAGAGAGGCCGTCCAG
GGTATAGTGTGACGTAGGGATTTAATCTAACCCTTAAGAATAATTTTGTAATGATATATAATTTTG
AACGGTACAGAGAAACTTCGTGGTTTTGTTCAGCATAATATAGGCCTAACCTTAGTAGTTATA
TGAGGTGAGAGATCGTAGAATTCTCCCACAAGAGTGTTGGCAATTGTTCTAAGTTCTCTAAA

CCAGTTTTCTCAAACTGTGGTCCGCGGACCAAGATAAATATATATATATATATATATATATATATA
TATATATATATATATATCAAAGATCTATGCCGAAAGTTCTCGGGAGCGTTCCATACATTGACAC
TGCACATGATTAACATTAACAGGCTGTGTTTTATGTCTAATTTTAATTTTAAGGGGGTACTCTAT
ACTGTATAATTCCATTCGGGGATAGACTTATTCAACCATCCGGATAGTCTTCTATCACGGGA
CACCGGCATCCATCTCGCGCCTAGAAAGAGCGACGAGGTTTCTTTCGTAAGATAGTTAGC
ATCTATGTTTGCTGAGAGATTATTTAAATATTTTTAGACCTTTAGCCTATGCCAGCTATGCATTT
ATTTATGCTAGAAAACTTCACAAGGCCTAATTCCTTATATCTCTAATACGTGTGGGTAAAGTAC
TTGTCTCCCGCAAAATAATTCTTACTGCCCTTTACTGATGCAGCCCAAGTCACTTCGTTCGC
GAGTCATAATCAGAGGTCCACACTGTACTCCCGTGTATTAAGTATAGGCCTATTGGTCAATG
AAGGTAGGGCCTATAATATAAGTTAAGTAGTTCCTTTGAGGGTAGCAGGTAGGCTATTTTTCA
TGATACCTAATTTTTTGCTATTTTATCTGATATTACTCTAATGTTATTTTCCCATGTTAGGCATTCA
TAAATTGTATATAGGCCAATTCCCAAGAATTTTGCGTTGTCAACGCGGTCAATGTTTTTCCCA
TCAACCAGTAAATCTATATTTGTCTCAGGAATTTTGTGTTTGAAAAATGGAAGAGAATGTAGCA
AGGTGCTTTCACATTTTAGCGATAGTCGGTTAGCCTTAAGGCATTCTGCCAATATAACCAATT
CCGTACATGCATTGTCCATTTTTTAAATTCAGTCAGGCAACTATAAGAGAAAAAATCATTTGTG
TCGTCAGCAAATAGAATTAATCTTAAAGGTTTTGAAACATTAGGCAGCAACATAATGCGGAT
GTCCGATAGTATGGAATGAAATCCCATTGACCACGCGTCAAATGCCAAATATTTTCACTTTA
AAGATAGCCTGGAAGACATATAATATTATGATGACTGGAGTGGCGATTATTTGTATTATCAAAT
ATCTAATTATGAAACATACGCATGGAGAGGTCTGACACTTGAATACGATAGTTTAAAACGGA
GAGCAAGGAGTAGCCTACTTACGCATTAACACAACTTGTGTCACTTATGGAATAGCCTTATT
CACGTATAGAGAAAGAATTCACATGAGAGCCGAACAACTGAACTCCGTAAATAACGATTGA
CATCCCTAACAAACAGGGATGTAGCCCATTCTGGGATGTACCTAATCCAAATAGTCATGTAG
ACTTTGGTATGCTAATAGCACTGAAAAAAATATCTTCGAGTTTCTTCCCAGAGGTATTAAATTG
CTTTAAATAACTGCTGAAGGCATATGAATGAAGACCACTTAAGAAGCCGACCCTATAAAAGA
GACATGCCGATGAATGAGGCAATAATATTGATTATATCTATATTGTAGATAATTATAGCCTAATA
GGCCTAATTATGGAAGACGTTTCCTTTATTTTATTAGTGTTTTAAATTTATATGTATAGGCCTGC
GTTCTGTGACCGGGTGTCACTATATATATAGTTTTCTGTTGTGGTATCTCTTCTTCTTGGTATTG
CTTACTTTAGACGGCCCTTTTAGTTCATTTATTTTGACCGACTCTATTTAATCGGATTTAAAGTA
GCTCCATTTAATTTCGCGCCATTATCTCTATTTAATTTTGCGCACTTAACTTCATTTAATCTGAT
TTATCTTAATCAAATCCGTTTAATCGGATTTGTTTATATTAGCTACACTTGATTTCGCACTTTATA
GTTCCCTTGAACCTCCGTCCGCGCCCCCACCATTTTGCACTGCCCCTTAAAGGCACGTA
CAATTTCACCCACAGAATGCTGTTTAGTTGTCTTCGTCCACGCCCCCACCCTCTTGCACTA
GCTGCCCTTAGAGACACGAACAATTTCATCTTTAGAATGCTGTTTAGTTTTCTTCCTCCGTGC
CCACCCCTTGCACTGCCCTTTAAAGATATGAACAATTTCATTCCTAAAATGCTGTTTAGTTGT
CTTCTCCCTCCAGGAGTCCCCTTCGCCCGCTCACATCCTTCTCAACTGGTGGTCTTGCAA
TATAGCTGGCCCTGAGTGATACGCATGTGCTCTATTCTATTTAAGGTCATGGACAGATGTCTT
TTTTCGTTTTATGTTTTATTGTTACTAATCACTGTTGTTCTATTAGTCTAATATTGTTGTGGCTGTGT
GATGGTCTATCGACCGGCCGTTGCCTTGCTTCATCAAATAAATGAATATGCACACAGAGAG
TTGAAGAATTATTTTGACTCGTTTAACGACTTCGGTGAGTACAGTAGGCCTACCGATCGTACT
GGATGTCCGCCACTCCCCTCTCACACTAAGAAGGAGCTCACTCTTGACACATAAACCAC
GTCCAGGCCACTGAAGCCTAGGTAGGCCTGAAGGTCTATTAATCCCCACTGTCTTAGAAA
AAGAGCCAATCTGGGATCCTAGAGAAAGACCGGAAGCGGGAGATGGAAACTCACGAAAA
CACGTTCTCTGCCTCAGACAAGACATCTCGGGGCAGCGAACGAAATGGCGGTTGGAAC
GAACAGACTCATAGATAGGATGGTCTTTTGAATTGCCTTCTTTTGCTTGTGATATTTGATTTCCT
TCGAGATTGAAGCCCCGCAGGGGTGCGCAAAACTGCGCATGGAGCAGAAATTTCGATCC
AAGCTTCTGCCCTAGCCGGGGATTGATAACTATTAATAATTCTGAAATCACGCTGATCTCCT
CTTGGAATCTAGCCGGCTTAAGTACTGAGACTGCAGACCCCGAGAGAGGTAATGCAATAA

TAATAATTGTCTATAATAATATTTCCGTTTTAGTATTCAATCTGACATGCCGCAGAATGATTACA
ACTATAGTATGAACGGATGATAGAAAGTTATTACAGAAGTCTAATTTAACAGGGCGCCCATC
ACGTTATAGTTTAATTTAGCCAACACCGCCGCCTAGCGAAAGAAGGACTAGTCAACCTTGC
GGGATACAGCACCACACTCCAGATGTTATCCACTACCCTACGGCCCCGAAGTGGTGCGC
ATGGACTATAAATTTCGATCCAAGTTTCTGCCCTAGCCGGGTTTTGAACCTCAGACCTCTCA
CTTGGCAGTGCAGCACGCAACCGCTAGACCACAGTGCACCCAACAGTAAAACACTAAGA
AAGCCTTCAGTCATGTCAAAAACATGATCGGTCCTATAGGAAGACATGATGGCCCCTGACG
ATTGAGTTCCTGTCCTCTCTCCTAATTCTCTCGGGGATAAAGCCTTTCTTTTCTTTACCTGTTT
TAACTCTGCCATTGACAGTAACTGTTGTTTGGTTTTGTCGGTCCAATATTATACAAGGCGGGA
TAGAGTTCTAAATTATGTCAATTCTTTGGGTCAACATACGTGGCGACTTTGTTTTACTGATCATA
TTGCTGAGGTGCACGCCTCTGCGCTCGCTCATTATAGTTCATTCTTAATAATTCCTCCACCTT
GTTGTCAGGACTATGCAGCCCCCGTCTGGTATTCATTCATTCATTCATTCATTCATTCAGGC
CATTTCTATTGCACCTCTTCGAGTTTGCTACTACTCATAACTAAGCGCTCCCGACACAGCAT
GGATACTGTGTGAGAGATTGACGCTGAAGCGCCGCGGGCAACTGCGAGTTACGTGGCG
GCTAGAGCGGGATTCGAACTCAAGACCATCCAGTCGAAAGGCTTTGACTCTACCAATGTAT
TGTATTGTATTGTATTGTATCTATAAATTTATATAGCGCTTCTTGCAGTGCACACCAATCAGAAG
CGCTTCCAGTGCGAGAGACCCAGAGAGAAGAAAGCAGTCTTGAGAGAACGAAAAGACG
CACTTGGCACACCAGTTAATAAAGTGGATCGAGTAGAAGGAAGGAGTTGGTCACCACGTC
CCACAAACGTCCCCTTCGGTATGGCTATACACAAGAATCACGCCCGTATTTAATGCCTCCT
TTCGGGGTTAATCCATCTGATCTATACATGTATCTGCCTTCAGACTCTCCTTCTTATCAAGAC
CTTGTGAGCAAAACTGATCTGACACCTTCTTGACTCTCATCTTTGACTCCCATCATGTTCTTC
ACCCAATATTTCCCCCTATTTTTATCCGGCGTCCTGGCTTGCGCAAGCGCTTCCACCCCTT
TGACCTACCAATCAAAGGCTCCATGAACTTCATACCTCGTGTCCTCTGTGGGTCCCTTATC
CCCCACAACAACATTCAACATCCAGCTATCCTTCCACCCAGGATCCATAGTGGTTAAGGA
CTTTAGCATCTCTTATATTGTATTCTGTTATTGTTGCAATAGGTCTCTAATTCTTCCAATGAATGA
AGTTTATTAGATATGGACTGCTGATCCAATCACTCTCACATGGGTTGGGAAAAGCAGAGGA
GGGAGGAGTATAGGATGACAGAGAGGCAAAGTGATGAGAGGATGCAAGAGGAGAGAGTG
AAAGATGGAGGGGTGAAGGGAGTCGGAAGATATGAGTGGGTGAAAGGAAGAGGAGTGAG
ATGTGATACAGGATGCATATGGCAGAGGAACCACCCGAGTTCTTTAAACTTTACCCACTTA
GGATGGGCTTCACTGCTGGTTTATGAATATTAGCTATTATAATTAATTACAATTTTGAGCTAATA
ACATTTCATTAATTTTATTAATTATTATAGTGTCCAAGTTATCATCACTTATGTTATTAATTATTTTAA
TCAATTACTAATCAATTATTGCAAATTAAAATGAGCGGTCATTATTATCATCATTATCAATTATTT
CTATTATTTATTGTTACTATTAATCATTGGTGTGCTTAATTATTGTTATTGGTTATTATAAAAAATTAA
AGCCTAATATATTTTTTATTCTTGAAAGTATTATTATTAGGTATTATTAGTTATTAATAATTAATTTATA
CAATCACTTAATGTCTTTGTTAATTATCATCAGAATTATTCATTATTGGTTTTTATTTGGCATTATTA
TTAGGCTAATTTTCATTGTTATTTATCATCATTGGGCCTATATCTTTTCATTATAGGTATTTATAAC
CATTATTAATATAATTTTTTATAGTATTATAAAGTTTAGTTATTACTATTTTCATTATTAATAATTATAA
ATCATCATTATTAATTAATTTTATTATTCATTATTAATTATAATAATTGTTTGTTATTATCATCATTATA
GCCTAAGTCAGTAGCCTATTAAGAACTATTATTATCTAGCGGTGAAGCCCGTCTAAGGTAGA
GCTGTAACGAACGGTTGAACGGCTACACTGACTCGTACCAGTTATTAGAATATTATATAATTA
TAATTATGTGTATATATATAGTAGGCCTATATATAACAGACTATCACGGGTTTATTACAATCCCC
TTGATATCCAATCCCGTATCATTATATATAATAGACATCTTAAATTAAACACTGAAGGACGCCA
GCAATGCTCTGGTCCACGAACTTGGGTGCATTTAGATGTTTCAATGTAACTTTTACAAAATGT
GGTCCGTTTTTTCCAGAATTTGTACACCCGCTCCTACAAACAAAATGAACAACAGTACTGTA
GCGAATTTGGTAGTGAGATGTTTAATATTTGCGATTTTATTACATACGTCACGTTGGGTCTAGT
AATTTTACATGTATATATTTGAGGCACTTGTTATTTCGCATAATAACAACTGCACTGTGAAAATG
TTGAAAAAGATAGGAAAAATCTGAAAATCATGTGCGATGACTAAAAAAAGGTCATCAGAAATT

TTGGGCGTGAAAATGTAAATTTTTCCCTTAAAAAACCTCATTCAGAAATCGGCATCATGACC
GGTATCATCAATTTCAATAACCGACATTCCGACAATTGGCTGTGTAGGCCTAATCGGCAAAA
AGTGATATCGGTACAGCTCTAGTCCCATAGGACAGTCAGGAGCTCGGGTGATTGTCTTCAA
ACCAATCCTTCCCCCATTTTCACACTCTCCAGCAGGGGAGTCCAGTGGGTCTAAGTGTAA
AGCATAATGCGTAAATGCATCATGGTCAAAATATGGGGTAAGCCAAAAGTGCAATCATGCA
GAAAAAACGTAATTTTATCAAAACTAGTGGATTTCGTAAATTTTGTGGAAATATGGGGATTTATA
AATTTTGTGGAAATAGGGATCAATATGAAATATGCATCATTGGCTTAGAAGCGATGGACGCC
CCTGCTATCCTACCATTTCCCTCCCATATGCCCTCCCAAACCTGTTCCCATACCACATGCA
TCCTATACCCCATCACACTACCCTTCCCCTCATCCTCTCATACCTCTCGACTCCCACCTTT
CCATCTTCCGCTCCCTCCTTTCGCATCACCCGCCGCTCCACCTCTCATGCCACCCTCTC
CCAACATCCTTCCTTCCTCCTCCCTCTCCATTCCTTTTCCCATCCCCTACTCGTCCCCACC
TGTCTCTCTTTCTTCCTCGTTCCAACCCGATGCTGCCCCTCCTCTGCTTTCCCCATGTGTGA
GTAGCGAAGCGATTGGATCATATCTAATAAACCACTTCCCTCCAATTCCCGATTTCCTGCC
CCTCTCTTTCCAAATCCACTCTAAATCCCATTAACCTCTACTTTAACCCTCTATTCAAGAAAT
GCCCCTCTCACACCTGTCCCTACTTCCTCCTCTTCCTCCACCCTGCACTTCAAGAAACAC
TCCTCTCACTCCTTCTCCATTCCTCATTCTGTTCTCTTCCTCCTCGCCCAGTTCTCCTCTCCT
TCGTTCTCTTCCTCCACCTGTTTCTTCATTGCCTCCTTATCTGCCACCTTCACCTGTTTGTTC
ATCGTCCTTCGAAGTCATCGCACTCAGATGTTTCACCTTCTCCACCTAATCTCTGACAAAGT
CATTCGGTAGAGGGTACCAGGATGTCTTTTCATCACCTAGCTCAAAGATGACGCTCGAAGA
CCCTGATCCCCATAACTTAGTGATCGATGAAGAGAGAGGGCGGAGTCTAGAATAAATCTTA
GATCACGATCATAGGAGGAGAATGCACATTAGGAAATCTAAGACGAAGGTAGTCCTAGTCA
ATCTTTGAAAGCTGACTGTCAAGCCAGATGTGTTGCGTTTTGTCTTGATTTAGACGAAGACTA
TTATAGGGCAGCCATGGTAATCGTAACCGTAATAAAATATTTACACTTAAATCAGAGGTGCTG
CCGACCCGGGTCGGTGTCTTCACTGACAAACAAGGAAGTTTCCAGGTGCTGAATTGATCA
GACGATAGAAAGAGGTCTCTAAGGGACTGAAGTAACTTGTCCAAGTGGATGGGCCCACAA
TAGCAACACACCTAGATCGAGCAATAGACGTTCGTGTCCGCGGGACAACCAAGTGGCCC
CGATCCGAAGACCGAGCTTCAGAAGCCTCTAAACTACGCTCAACCATAGAAGAAGCTTTG
GCAGCAGTACCATGAAGGTATGTCTGTGGCCCATCGGAATTAACAGATCTGTTCTACGACC
GGATGTCGCCCTAAGTTAACACCCACCTAAAAAATCGGACCGGTTCAATCGGCAACAACA
ATTGAGCATTAAGGTTTGACCTGGGTTCATTGGTTGATAGTTTTACCTACGAGTTTTAGGGTAT
CTGAGTGACTGACAGACGCGCTGCAGGGTGTGTTCAGGGAGTGACCTTTCTTCGAATTGTT
CGAGTTATTCCTTTCCTATAAATCCACTACTATAAGATGGGCTGTAATACTAGGATTCATTACC
CTGCTATTAAGTGCCCTCATTCTTATAGCTCCCCAGCGACCTGTTCTTATTATTTTTATTCCGT
GCCATGTGCTTGTTCTGTTACAGCGCCCTATACTTCCCTTCCTCCTCGGATTTGTCACTGAA
ATTCTTCGTACCCCCCTTTCCTTAAACTCTACTCTTCGATATCCTGTCATTCTCGCTTTGTTGCA
TCATTTCTTCGATGATTAAACCCTACAGTTTCTTGCTAATAACACCCTACTTTCTTCTCCTCTG
TCCGTTGAGTTGTCACCTGTGAAACATTTTAACCCATTCATTTGATGAGAAGTTAGCCACTTC
TCGCTCTATCGCAGACCTTCTAGACAAATGTGCTGACGAGAATATCTACCGCTCGATCGTA
GACATTCTTTGGTGGGTTTACGTGCGTTCAAAATTTCTCTTCATTCCTTCCGGATATTTGGCT
CTAACACAGGCTTCATCGGAACGAGTCTGTGTGCGGTCGGGCTGTTCTGCTGCAACCGG
AAACTATCTAAGCTTCTCTTCTCCTCGTCTTCGATGTCTCCCCAAAGCATAACTATTCGTAGA
AGTTCATTTTGCGTGATGTCGGCTTCAGCGTTCTTTGCCTTCTTATTTATGTTGCGCCTCGTTT
ATTTTTATTCATTCATTACGAATATTTCTAAAGCGCCTCTTCAAGTCCACTATACTACTCAGAG
GCGCTCCCGACTACAGCATTGATATTGTTTCGTAATTAACACGCCGAAACGCTACATGCAA
CTGTGAGTGAAGGATTTGCCCACGGTACTTACGTGGCGGCTATAGTGAGATTCGAACCTG
CGACCCTTCGGACGCAAGGCACCAAACTAACCACTAAGCCACCAAGCTCTTCATTTAGG
CCTTACTACTAGTACTTTCTCGTTACCGTGTAGCTTGTTTATTTTAAAGTCTTTCGGCTTTTATA

AGCACTATTTTTATCTTAATGGTTTATCATTCGTGATAGTGTTTCGCTCTTTCAGGCTGACTACC
CAAGTTGTCAAGCTGTTTCGTGTTTGTGCCGTTTAGGTTCGCTTTGCTTCTGCTGTAAAAGTA
GTTATTTCTAAACGAGAATTACCGACAGCGGCCCCTTTCAGTCTAGAAAAAGAACGTGTGA
AAGATTCAAGATAGATTTGCTTAACGAGTATATCGCGACCGAACTGCCATCGTTGGTTGCCA
AGCAAACCGTTGTGATAAAAGAGGCTTTTATATTATCGGTATACTTCCGATGACGTGGTTTCT
TAGCTAATGCCAAGTACTGCATGGTAGGCCTAAGCGACGGGACGAATGGAAAGTTGGACT
ATCACCATAAGACACATAAAAACACGGATAAGAAACAATTTAGGCTACAGTGGGAGAGACT
TTTTGCCTGAGCACTAAACGAGGCAACTCGGGACAAATGTAAAAAAATTAAAACCCAAGA
GGAACCTCTGGAAAAGCATTTTGAGTTCAAGAGTCGTAGATTTATGGGATGCTCTGGATGAT
ATAACTGTCTTAGTACACCCTTACCGAAGTGTTCAGAGAAGGCTAGTGAAACTGAACTATGT
TCCACCTTAATTTGCAGTAGTTTTAAGTTTTAACACAGACTACACCTATTCTGAACCACTTAAA
GCTCATGTTATGTTGTGCTATGTGTTTTCTGTGCACGTAATCTTCATAAATATAGACAAGCATTC
ATCTGACTTAGGTGGTTACACTTGTTTGGCTCCCGAAAGGTTTAAGAAATCGATTAGTGACC
CGTGGCTTAAAAGAAGTTTATAGTTTTACATTCGAGGGAGTGCTTAAGTGACTGATTGCCTGT
TCTGGAAGTGACCTTTCTTCGGATTGGTTCGCGCCATTCCTTTCCTAGAAAACTGTAAGATG
TGATGTAATACTAGGATGCATGTACGTTGGTTTGGTGGGAAGTCTTCGAAATTGCTTGTCGG
CGGGGTAATTATCTGTCGCGTTCTCGTACAGGTATGAATAGCGAGCACTTGACGTAAAGATT
GTTAAGTACTATTATTAAAATTATATTTAATGCAAGTTTACTACTATTTAACATTTTAATCGATGTG
AATATCATAAGACATTCGGTTATACTGTTCGTGATTGTTACTACCTATCCGACTATAGGCTAGT
TCAATGAAAATACTTATTATCATGCATGTCCTACGTCTCAGTGAGCTAGTCTTAGGCCTACTC
CCTGCAGTTAGCATACCACCCGTCAGAGGGGGTGCTGAAGTACTGTGCATCCTATAGTATA
ATACTACCATCCAGAGGACTGCGCTAAAGTGCTGTGAGCACAGTGCTCACATTACGACCG
TCAAAGCAAGCTAAATTGAGAAAATGCAGTCTTTGCGTATGTCATATTATGCTACCTGATATG
GAGTTGCGATCGTGTAGTTTGACATCCGTCGATATTCTAAAAACTTTTATGGCTTTTGTGAGAA
GAAGGGCACCGACCGTGTATTCAGCAAGGTGAAATAAGCAATTGGAAGGCGTTTGTTCAA
GCTGTTCATGAGCAGACCCAGAATTGGCCAATGATTTGGGCTATGCCAGCCAATGTAACTT
TTCAAGAAGTTCAGTTTAAGACCCTATAGCTGTGTAATTCAGCTTAGACTGTTAGATCCACTA
CCCCATTTGCATTTTGCAAGTGTTTTCTTTCACGCCTACTACAAAATTATTTTCTACCTTTGCG
TCATTAATTAAATTAAGTCACAGATCTATTCGACTGTTATTAAATTTAAAAGACCTGAAAGATTTT
GTGCACCTACCCTTATATATCAAACGTTGTTCATTTTAGCATATGGCGTAGGCCTACAAGTTG
GTTTAGCCTATTTCTTCAGTCAGCCTATAGTTAGGCTACTTTCTTGGTTTTAAGTGTGACAAGA
AAGTTTAGCAGGCTATAAGGCTTATTACCTTATAAATCTGCACTAGTCTCCTATGAAACAACG
TTTATCTGTAGGGCCTAATTTATTATTCAATATGCCTACTACAAAATTATATCCTCCCTTTGCTAT
ATTAAGAACTATTTAATTAAGTTAGGGTAGAACATTTTATGTGAGGCCTTTGACGTCCCATCTA
TCAACACCTCCTCTGCCCCCTCGTCACACATCGTCACAAAATGTCGGACCCACCTCTAC
GTGCGTGGCGTCATTTATGGACGGCCCCTCACATCGCAATTCGGGCTGTATACAGTTTGA
AGGAGGCTTGGGCTGGTGTGTCAGTCATTTCGCAATAGGCCTACAGGTAGTTATGGAGAC
ACGGACAGCTGATTCAGTCGCGTCGCTATACAGTCCGCTCTGAGGATAACTTTGTGGGCTT
ATGATTCAATCAAATCGTCATAGACATATGTAATGTATGAGACAGGGTGCTCCTGATTAAGTC
ACTTCGTAATAGAGGTAGTACGGGACATTATAAGCTGCTGGTTCAGTCACTTGGGAATATAG
ATGGGCACAGTGTCGAGAATGCCAAGGCAGCATCTATCCAAGCCACGGACTGGTGAGTC
ATTTCTTCGCTCGTATATCATCGTGTCCTACCTTCCTGATGGCGTTCCCTATGACCTGCATTT
TAAGAACGTTCTGGTAACTGATGTTTGTCGATGCCTTTTTATCGATGACTGATCGCCAGCTG
GCGATGATTTGTGCAGACTTATTGAAGTTAAGGCGACGGAACAGCTGAGGCTGAAGTCTCA
GTAGGCCTAAAATTAAAGTACATAATATTAAGGTAAGAAGCACTGAAAACGTAACGTCTCGA
GGAACAATTCTAATAATTCTAATAGAGCTGTGCGCCGGCCGCGCCGCGCCGCCGCCGC
CGACAATTTGTGAGCGCCGCCGCCGCCGACTTTTGTTGATCGGCGCGCCGCCGCCGC

CGCCGCCGATAATTTGTCGGCGCGCCGCCGATACCATTTTTGCTGCGTAGTTATTTTTTGG
AATTTTCGATGACTAAGTCTTCTTCTTTAAGTTCTCTGCCCTTTGGCAGGTCCAGACCTCCAA
TTGTTTCATGTTTTTCGATGTTGAGCTTCTTCTTTAAGCTTGCTATAATATTCCTCGCTCATCATC
CAGTCCAGTAATTTTTTTCTTGGTCTTCTCCTTCCTCATTTACAATCCATTCTTCCTTCTATTATT
TCTTTTTGAAGTGAGTCCCCTGTCATTGTATGTCGCATCCAGTTCCGTTGTCTGGTTCTTATTA
TCGTTAACAAGGATCTTTCTTCTTCTACCAGTTTTAACACCTCATTGGATATGTGGGATGTGCT
CTGTCCAACTGATCTTCTTCATCCTACGCCATATCTTCATTTCAAAGACTTCCAATCTTGTGAT
GTCCTCCTTTCTCATAGTCCTTATTTCTGCACAGTACAAGGTCACGCTTCAAATCAGTGTTTT
CACCATTCTTTTCTTGAGGCATCTTCATTCTTCTCAATCCTCCCCTTGGTAATTCTTTCCTCCT
GGAAAAGGCTTCTTTCCCATTGCTATCCAGGGGCGGTAATCCTGGGAGCTGAGGGTCGC
GACCCCCAGATTTTGGGCAGGGGGGTCGAAGGGAGGGTCGCAGGGGGTCGTTGGAGT
CGTGGACGGGTCGTGAAATATTATTATATCTTATCATGTACAGGGAAGTATGTTCGAAAGTGG
TGACTTTTGAAGAGAAATAGAATAATTTGCCCAGAGGTAGCTGTAAATGGACTTTTTTTGCTT
GGTAAATCAATTTTTTTAATTACCTGAAAAAATCAAAATTTTCGAAAATTTGCCTGCAAAACTC
GATATTCTTTTGTAAAATTGCCTGAAAAATTTGAAATTTGCCTGTAGAAATTGAAATATTTTTGAT
CCGGATCCACGACCCCCAGATTTCAACCAGATTGACGCCGCTGTTGCTATCCTTCTCTTG
ATTTCTCAGTGGCATTATCGGAGATCATGCTTCCTAAGTAGCAGAATTCCTTGACTTGTTCTAT
AATTTCCCCTGCAACTACTATCTTCATTATTTGATTCTGATCCTTTGCTTACTCTCAAGACTTTT
GTCTTTTTAGTATTTATTTTCATGTTGTAATTCACTGACACCATATTCAATCTATCCATCATTTTTT
GCAGGTCGGACTCACTGCCCGTTAGCATTGGTCATCTGCAAACCTCAAAGCTTTGATGAC
TAAAAAAGTTCCTAAGAAAAGTTTGAGGATAGAACGACATTTTTTCGTGAATCTTAGAAAAAA
CTGTTTTTGGCCGCCCACGCCGCGCCGCGCCGCCGCCGCCGACTTTTTGGGACCGG
CGGCGCGCCGCCGCCGCCGACCGAAATTGACCGGCGCGCCGCCGCCGACACAATG
ACGTCGGCGCACAGCTCTAAATTCTAATGGCGGTAACCGTGGCAAAACTATTACAACACA
GCAGCCTATAGGCATACTAACTGGGAATTATCGAAAAGGAATATGAAAACCTTGAGTTGAAA
ACAGTTTACGCTTAACGTTGAAAATCTCGGTTTCATTGAACAGGTTTCGAAACTTCTGTCAAC
TTCTTTCCTAATTGCCTTATTAGGGCCTACCTGCTACTCATTTCCACCAAGAATAGCCCTCTG
TTCATGTGCAAAAAGGCAACAGTCACCCATTGAGATGACCATCATAAATCACTGATCAGATT
AGCATAGAGCACAAAGACTGATAAGTCTGTTCTATATTAACTTTAAATCTAGTCATAGGAATCG
TAATCGTAATCTCCAAACTTCTAAAGCGACACTCGAAAGCCAAGAGCAGGGAACCCGCTT
ATTCACGAGCGCTGGAGCTTATTCACGAGCGCTGATAAGGAATAAGTTATTACCAAAGCGA
CAAAGTTGAGAACCATCGAGTGGCGTCAAACTCTAAAGCTCTCTGCTGCTCAGCTGACTTC
GCAGACTTATATGCAATCATCAATCGGGATATTATATAGACTGACAGACAGAGACAGACATG
TGGACAGACAGGGAGACAGAAAGAGGGGCAAGCATTGATGCACATACATCTAGGTGATC
GACCATAGAATGCTTTCTAGGATATCACGATAACATCGCCATAGACATGGGCGTATAGATTT
AGAGAAGTTTCGTGTTCAACCCCCCCCCAAATGAATCTTTTACTGTTAAAAAACCTAACTGT
AGGAAAATAGGACCAAATTCAAAACAGACCCCCGAATTCTTTTTCTAGCTACACTTCTGGC
TATGAAAGTCTTTTGATAAGAATAGACCTACATTCATTTAATTAAATTAGAGTAGGTTGTAAGAG
CACTCCACCTACAGTCAGCGAATTGATCTTTACGGTCAATTAAAAGAGAAAAATAAATGAAA
ATGAAGAAGAAAAACAGCTGTCAAAAACTCCGTTGAGGTCATTGCTGTCCAACCTCCAAG
CTTCCAACTGATCCTAAGTGGCCCGCCCAAGCTCGATTGGATTGTGCAATTTGTGTAAAAA
AAGAAGTTAAACGTGAAATCAGTGCTATTTGTTCAACTTGAGTTTGTCAGCAGGCCCTATAC
GAGCGCAAAATCCAGTTCAAGCGACAAATTAAACTTGTTGAAAGTTTTAAATCGTACGGATC
TCAGCCCGAAATACAGTTAATAATATTGCTCGGTGAATTTAGCTTCTGCTTTTGAGTTAATTATA
GCTATAGTGCTACCCGCGTCTTAAAGAATAGTTTCCTCCGTGGCCTTGTCAATAGAGTTGG
GCAGCATTGCGTTACGCCAGCGAGCACAAGGAAGAGAAGTCAAATGTCTAAATGAATTATT
ATGTATAATAATTATATACATAGTCTAGACTAGACAAAAATTAACCTACTAAGATACAAAGATCG

ACAGCAACCACTTCATTCATTAATTCAGCAATTCAGGCTATTTCTATAGCGCTTCTTCAAGTC
CACTGCTACTCAGCACTGTGTCGGAGTTTGACGCCGAAGCGCCGCAGGCAACTGCGAG
CGAATGACTTGCCAAAGGTCCCTACGTGGCGGTTAGAGTGGGTATCGAACTCACGACCCT
TCGAACGATAGGCGTTGACTCTACCAATGAGCCACCACGCCACGCACTTGTCGAATTTTG
ATGAGAAATATTAAGGAGACGCTATTTAAATCTTCCCGTAGGCCTAATGAATGAATGAATGAA
TGAACGAACGAACGAACGAATGAATGAAATAACGAACGAATGAATGAAAGAAAGAAAGAAT
TAATGAATGAATGAATGAGTTGCGGTCTCAACAACACGGAATCAATAACGAGAGAATCGAA
GAACTTGAAACGAAAGCTATTGGAAAGTGCTCTGGAATACACTTACCGCTTTAAAGAAACG
ATCTCAGTAAGGTGTTAGCTAACTATATACACATTGGAATATACATTATTAAATACATACACAAA
GTTCTGTCTGTCTGCCGAATGTTGACAATCTCGCTTCGTAGATCCCAGGGTCTAGCGAATA
CTGGACATCGGACAACAGTGTCGTTAACCGCGGAGAGAGGGAGAGAGACCTACAATAGA
ATTAAGAAGTAAAACAGATTAGCGCGCTTACCAAAAGCCGCATTCCTTAGATAAGAGCCTT
CTACAGTATCTAATAAAATATGCAGAAATAGATAATAGTTGATTGCGTTTCAAGAAGACAGCA
GTTCAGTCTATTTGTATAATTAATGATTAGTCATGAGGTCGCCTGCACATGTTAAGTTGAAGTTA
ATGCCGTAAAGCTTAATGTGCTAGAGAATTAATTTGTGAAGAAGATTAAGAGTTTTAAAAAGC
GAAAATAGCAACTAAAGCAACTAATTTCATAAAAGAAAAAGTGTCTTTTATGACCATCAAACG
ATCCTTTATGGCAAATTTATAAAGGGCCTACGCAAAATCAAGGAAAACAGATCAGAGCCAT
ATTGGAAAAACAGCCTCTATGTAGGCCAAACATATTTTATATCGAAACATGTAGATGTGGGG
AACATCATCAGGCAGAGAAGACTACTGAGGCTATGGCACGTGTGCTCATAGACAAGGTTA
GACGAGCCAAAAAGATATTGTTTTGGATTCATGAGGAAAGACAGAGAAGAGGAAGAGCGC
GCCGAAGAACTGGACAGACCTTTAAGAACGACTTGAGAGGCCAGAAATACCATGGGAGA
GGGCGGAGGAGCCAGCAATGGACAAAACCGAGTGGAGAAGATGCGTACCGATGTGCAG
ACAAGAACTGGATAAACTAAGGTAAGGCTAAATAGCTGAAGGAAAAAGCCAGGTGGTCAA
ATGAATAATAATGCATCCATATAAAATTTAAATATCTTAATAGATCGACACCAACCACCTTTCC
CCGTCTCCACAGCCTATTAGACGCTGAAAGAATAACGAGAGACAACTGATGACGAAAATT
CTCAGAAAATAAACTGAAATAGGCCTACATCTAAAACTTTAAGGAAATAAATAGAACTGTATC
TCTGATACATGGTGTTAAAAATGCAATAGATACATGTACATTATATAGGCTAGGCGCTTGACTC
CACAGCTCCGTCTTAATTTTTCTTGAGAAACTCGCTTCAGATATCTTGGTTCCTATCTCTAGTA
CGGTCTGCTTGACATTAAGCGATTGAATTAACAGCGCATACCTGAAAAGTTTATCGTATAAGT
TTGAAGATCTTGGTTTCACTGAACGGGTTTCGTAGCTGCATTCCACGTGGGTCTATTTCTAG
GTATTCTCATTTCCTGTTATATCCTCGCCTACATGAACGACACATACATAGTCACAGGACGC
GTCTAGACCATCAATCAGTCTGTTCATTGATTCGTTTATTCGTTCATTCATTCATCGATTCATTT
ATTTTTTGTCCAGGAACCCATTAATTTTATCCAGCAGGAAAAGAAGATGCAAACAAAAGATA
CAATGGGAGACCCAAAAGGGACTAGGGTTCAGGTCATTTAGGCAAGTCCATGTTCCATGG
GTCGAGGAAAATGGCAACTGCTTCACCAATGAACAGACGCAGAGAATATTTAACAGACAC
ATTGTCAAGCCAAATCAAATCAAGCAAGACACGCTTAACAAGCGTGAGGAACGAGAGACA
AAAACAACTGCAAACAAAGAATCACATGGTAAGAAAATTTAATGAATGCAGAATCACGTGAA
AAAGCTTTTCATAATGAAGATAGAGACAAATTGACTTTTAGACAAAGCGATACTATAGGCAGA
TTACTTTGAACCTCTCACTTCCTGATCATGTTCTCACTTCCGCTATTCTGACAGCGCATGAAA
TCCATATTTTGTTATAGGCCCTAGTTACAGTTTTATATGTCATTGACTTAGGTCTGGAGCGCTT
CTGAATCACCGAGTGGCGCTTTATAAATGTTTGGTTACAATTACAGTTGCATTCACAATCGGA
TACATTGGAAATGTAATCTAGAAAGGAGGAAGGAAGTAGGCCTAGACCATGGCATCGTAAA
TTCGCTAATTTGCAGTTCTTAATTTGCTCGACGACTAAAATTCCAAGGTCATGACATTCAATG
AATTATTGACATAGGAAGAATCACCCTCGTCCTCATCTTTGGCCCGTCTAAATGGCCCGTCT
AGTGCGGGTCTGTACGGGCCATAAGTGGCCCGCGTAGCCGCTAAACAGCAAACAGGCG
TCGCCCCGCTTGGCCGCTATATAAGCCGATTCAACATGTCGTGTGCGCAGGCGCACTGG
GTCGATCCATTGAGATGTGCAAATAAAACTTCATAAACCGAGAATAACCATTGGCCTTTCAC

TGCACATATGCATAAAACATGATGATTCGATCAATCCAGGTAGAAGTAACAAATGTATTTAGG
TCAAAACCGATCCGAAAGGTGAGATGTCAAATCGGTTTATTACCGCACATGGCCCCTCTAT
GGCCCGCCTACTCAATGCGTCATGGGCCATTTTTGGCTCGCCTGGTGTTTTTTCAAATGAG
AAGGCCTGCTCATCCCATTCTGCTAAGAACTTATCACAATTCAGACAGCCAGCTTCTGAAG
TACAGATAATTTCGTCAATCAAGAGGAGTATTATACTTAGCCCAGGCCGGGCCAGGCGAC
ACTGCAACAGGTCAAATACGGACAGACGAAGGGATTGAACAGGCGAGGGTGAACATTAC
AAGGCAGTAAGTAGGCCTAGGCGTTGCCTATGCTCAATGAATAGTGAAAACCACTTCTGAG
TCTCAACGTTCCGGTGAGATAAGAGAAGAACAAAATTCGCTTTTGTGACATTAAGACACAG
CGTGTTAGTCCTAGATCAATCGGGAACAGCTGTAGGTTCGGAGTCAACAACCACTGACAA
GGAAGGAGTCGAGCGTTTGTATCGTCAGCGAAGAGAAAAAGTGCAGAGAGACAATGGGC
TTCAGAGATCACTAATACAGAGTAGAAACATCACTTTTGAGCTTAATGAAGAAGAGCGATATC
AAAGGTCAGTTACATCTATATCCATCAGTCGATTTACCCATCCACCAATCGACCCACCCAA
CTGTTCGCTCACACATCCCTCCATCCATCCATTCCTCCGTTCATCCATTCATCGATCCTACC
ACCAATCGACCAAACTTTTTGTACGTCCATCCATTCATCTGTCCTTCTAGCCATCTGTTCGTC
CGTCCACCCATTCATTCGTTTGTTCGTTCGATTCATTCATTCGTTCATTCATTCATTCGTTCATT
CATTCATCAATGAAGAGGTCCAATTTTCAGTCACAGTACATTCGGAGATTATTCATAGCAAAT
GTCGATCTTCTATCCAGAGCACTGAACAAACATTTTACACGTATACATTGAATGAACGAAAG
CACAGGTAAGGTATGAGATAGGTCCTACAGTGACGACTCTTAATCCAGGTGAACGCTTAAT
CCAGGCAGCCTATACATGATGAGTGAACGCTTAATCCAGGTAGCCCATACATGATGAGTGA
ACGCTTAATCCAGGTAGCCTATACATGATGAGTGAACGCTTAATCCAGGTAGCCTATACATG
ATGAGTGAAAGCTTAATCCAGGTAGACTATACATGATGAGTGAACGCTTAATCCAGGTAGCC
TATACATGATGAGTGAAAGCTTAATCCAGGTAGACTATACATGATGAGTGAACGTTTAATCCA
GGTAGACTATACATGACGAGTTAAAGCTTAATCCAGGTAGCCTATACATGATGAGTGAACGT
TTAATCCAGGTAGACTATACATGATGAGTGAACGTTTAATCTAGGTAGCCTATACATGATGAGT
GAACGCTTAATCCAGGTAGCATATACATGATGAGTGAACGTTTAATCCAGGTAGCCTATACA
TGATGAGTGAACGCTTAATCCAGGTAGCCTATACATGATGAGTGAACGCTTAATTCAGGTAG
ACTATGAGTGAACGTTTAATCCAGGTAGCCTATAAATGATGAGCGAACGCAAAATATAGAAA
CTAAACTTAAGAAATTCCATCTAAGGTAGAGTGAAATGAAGGAAGGTGAGATGGTTTGAGGT
AAGGTATGCTAGGCCTATACAGTGAAAAGCGATAATTAAAGTAAGGGCCACGTAAGGCAG
GAATATTAGCAAATGCTTATTAGGCCTACCTCTTCTTTCACATTTTCAGTATTCGACTTTTCCTT
TCTGCAGATGACAGCTTCTTCGACCGGCACTTATGTCGGAATTCTCCACACCCTTCGCCAT
CACAGTAGACGACGTATGACGTATGCGCTCCTCGAATGCTTTCCATCAACCATCCGTATAG
ACAGACAGGCTCCCACCATCATCGGTGCGGATTTGACCCTCGACGTGTGCCCTCTCTCT
GGTGGAGAGCATCACCGCCTTGGGCCGCCTCCTTCAAAGCACCATAGGTGTGGATTCTA
TAACCCCCTCGCCGACACAGATGACCAGCCGTGTGTCCACCATCATCGGTGCGGATTTG
ACCCTCGACGTGTGCCCTCTCTCTCGTGGAGAACATCACCGCCTTGGGCCGCCTCCTTC
AAAGCACCATAGGTGTGGAAGTGATCTTCGACAAGGGTATAAACCGTTCCCGTCAAGTGG
GAACCACAATGTACCAAAGCACTCCTCTAGGCCCCACTAGAAAACATGTATAGTTAAAATG
GCCGACCAAAGAAATCGTGGAAAGAGACCAGGGACCAATATGCCAGAGTAAATCGCAGA
GTAAGTCTCGCTTACTCGCTTGTTTCTCTGATATCCAAGCGTGGTGTGCATCTGTGCGCCCA
AAACTCACTACTTCTAAGACATAACTTATCTGATGCAATCGCCATCTGACACCTTCCACTGA
AACCTAGAACACACTTTAATTATTGTAGAACACACTTTCATTATTCTTAGGCAAGAGGCGAAC
ATCTAAGGGGAGTCCATTCCAGGTATAGAGGGACCAAAAATGGAGACTGCTCAGCGCTAC
CGGACGCAGTAGTAGCACGAGGCATGCACCAAGAGCTGGCCAGAAACAGAAGAACGTC
GACCAGATACATTATAGATACCGAATTACAGAGGTGGCAGAGGTATGCGGGGCCACTAGC
GGATCCAGACCTTCAGACAGGGGAGGGGCTACATTTCTTAAAAAATTATGAACTTTCAGAA
AAGAAATTTAAGTTCACCCCAAAGATTCTATCCTCCGAAATTTCTGATGACCCTTTTTAGTC

ATTCACCGCTTTTGTGGACATTTTTCCATATACAACGGTAAAGGCGAGGCCAAACTCTCTTT
ACCAAGCACTTTTTCCAACCATTTTTTATTCTAAATTATCTTTTTTACTCGTTTTTACTCCCATTT
ACTCTAATAAATCATTTTTTCTTCGCCCAAGGGGGCCAAACTCTATTTCCTAAACGGATGATA
ATAATAATAATAGTAATAATAATAAAATAAAATGACGGTCCCTCCTCCTTGACAAGACTTGGGA
CTTGCACCTGAGCAATAGGGCTACAGGTGGTGGAGTTGCCCGTCTACGGGTCAATATAAA
AGTGGGATCGTCTAAACCTCTGTATACAGGAGTGCGCATGGAACAGAAATCTCGATCAAAA
TTTCTGCCCTTGTCGTGATTTGTACCTCGGATCTGTCGCTGGGCATCCCAGAACGCAACC
GGTCTTTCGTCACATGAGAACCTTCAAATACCACAATCACCATCAAAAGGGGAGGTGAGC
ACGAATCTATCTCTCCTATGACGAGAGATAGACCGGCAGAAAAAAACGGTTGGACATCGGC
CTCAGAGTGAGGCCGGATTATAATGGTGATTGCGATAGATGTCGGCTCTTTCTGTGACTGAA
GACCAGTTGCGTGCTGGACTGCCAAGCGATAGATCCGAAGTACAAATCACGACAAGGGC
AGAAATTTTGATCGGATTTTATGTTCCACGAGCACTCCTATATCCAGAAGTTTAGACGATACC
ACTACCTACTACTATTACTACTACTACTACTAACAATAATAATAATAATAATAATAATAATAATAAT
AATAATAATAATAATAATAATAATAATAATAATAATAATAATAATAATAATAATAATAATAATAATAAA
ATAAAAATAATAATAATAATAATAATAATAATAATTATTATAATAATAATAATAATAATAATACTACTA
CTACTACTACTACTACTACCACTAATATAATTATTATTTTTATTCATTATTTATATTATGCCTACTAT
AATAAAAATATAATAAAAAGGTAAAAAATTTTATTATCCCTAGTATTATTATTATAGCCACCGATG
TTTTGAAGCAGGTGTTAATACTTCTTGGCTAGACCTAAGGACATTTCAGTTAGATCATTGCTGT
GTTATAAACCTTCGCATAACTTCCCTGCACTGAAATCATTTCAAGTATACAGCATAGCCCTG
ATTATATAGTCGTGCAATCCACCCGGAGCAAACTGGAGCCAATAGATGGATAAATCACATT
CTTGGGACTGGCTCTGGCAAGTCGTTTTTTACCGAGTTGCTGGCTGGCAACCGGCTTCAA
TAAAAACGACTAAACACGCACACAGTCCCAATCCTATGATTCATCCATCTATTGGCTCCAGT
TGGCTCCGGGAGTTTTGCCCGGCTATTTAATAAGGACTATGCTCTACTTGAAATGTCTTCGG
TACAGGGAAGTTATGCGAAGGTTTCTAACACGTCAATGCGCTACCTGATCTACCTGAGGGG
AAACGAAGGTCTGGAAGTGGCCATTGATGGAAAAGACCTGAGAAGGAGGAAGACAACGA
CTGGTGAAGAGAATAGAAACGAGACGAGAACAGCGGAAGAAGAAAGATGTTTCAATGCCA
GAAATGCAGAAAGCTGGGAAAACAGGAGCAGCGGGATGAGGCAACCACCTCAACCTCT
TTTGGGGGTGGTTGCTGTTCCGTCTCAGGAGTCTCCTCTGCCCCACGGGGAGGAATTAGA
AACACCATGTCCGCCTTTCCCCATTTCAGCGGTGCCGATGTTCTTGGCACCCTGGAGTCA
GGCGACTCGGGTTCAGAGGTGGGGTCCCCTTCCGAACTGACTGTTTTTCCAGCTGTGGC
CTTCAACCAGTTCCTGGTGGTAACTGACCTCCTCTGAACGCCGATGTTCTTCAGCCCCCG
ATGAAAAGCGGTCGCCTTCCTTTCCTCCTCCTCCGCCACCTTGGCCGCCTCCTCGCTGA
ACCCGGCGTCCTTCTTAAACTCCTGTTTGAGACGTTCCTCCAGCTGACCCGAACTCAGCT
GGCTAAGTCTCTCGGAGTTTTGGTTCAGACTGTCCGAGTCCAGCTCAGAGGGGTCAAAGG
CGGTGGGTGGAAGTCGGTCGGGAAGGCGACGGGTGAGGTCTGTTCGGGCGGTCGTCA
CCTTCAGAGCGTGTCGAATGATCGACCCAACATACTCTGAAACATCGGGGTTGGTCTTAC
GCCTGCGTTGGAAAAATCCCCTTACTCTCTTCAGGGGAATCCCACAAGCCTCCCTCATTC
CTTCCCTCACCGAACCAACGGCCACTTGCAGCGTCTGCTCTGCAACCACTACATCTTCCT
CACGACCGGTGGCCCCCAACACTACCGTTGGCGCCACCCTTGCAGAGGGGCCTCCTC
TCACGACATCACGTAAACTACGACGAGTCATCGTGGCTGTGCTACAAATGAACACCCCAC
GCAACTCAATATACACCGAACTCACCAAAAATCAGAACCTCCAAGCGCAAACCGAGAATT
CTCTACAAGAATGCTAGCAGAAGTGCCAAACGCTTCAACGCGCGTCACCGTGGCAATGG
AAAATTGCGTCATAAACATATACTGGCCTGAAACATGTAAACATTGCTATAAATATTATAGAAT
AATTATTCTAAATCTTACTTGACTATAGTGTAGTCGCTGTAATACATTTAAATTGCGACGACGA
CGACAATGATGATGATGATGACGACAACCTTGAACGAAGTATTGGTTACGAGATATGCTCCA
ATTGTTGCGAACCTCTAAAACCTGCTGCATGCGAGATTTTGGGTTACGAATAACGTAACCTG
ATGGATGGTAGATCTAATATGCCCCAATCTAATACAAGAATTATTATTAGTGTTAATATAGTCA

TTATTATTTTTGTTATTAGTAATATCTATTATTTGTAGGTCTATGATTGTAGTGGTGTTAATAGAAGT
ATGGCCTTATCGTGGATATCTCCTTTATACAATTTCGCTCATTTCTACTGGAATGTCATCTCGC
AAAATGTTCTGTTAAAATACGCTGTAGGCCTACCACATCTCTGACAAAACTTACTGAAACGA
GTATATATGATAGCATAAAAGGAGACTTGGATTTGGGTTTGGGGAACCAAAAATCATCTTGC
CAGCGTTTCCTTCAATTTTTCGAGCCAAATTTCCGAATGATCTTTTCTACAAAAGTCTAATTTT
CCAGCAAAAATTGTTCCTGATGACTTTTTAGTCATTAGTCATTTCATCATTGGAACCGTACTTT
GTTTATTTATTGCATTGAGCAGAATAAACACAAGAACAGAACAACGACAAAACAATAGAAAA
AAACTAACGTAACACCTAAAATGTACAATGGTTCCTAGACAAAGTTCCGTCCGGTTTCTTAA
CAGAAGCGCCTAATATTGTGTACAGGTAAAAGAAAGACTTAAAAGCAGTACCAGTGCAACT
ATAACTTGACAATAGACTGCTATTGAACATAATCCATGATAACGCTTTAATTACTTTCAACGTAT
TCACACAAACCCATAACTCAAAACCAAACACTATATAAATTTTGTGGCATTAATTAAGGAACA
TAGTAAACATAACAGAGAATCCAACAGGCAAAGGTGTAAAGTAGCCGGTATTCTGTTGCAC
ATTACAGGCTAGTCCCAAAAGGAGCAGAAGCCCCGCTATTTAGGGTCAGCAGATTACAGA
TTGTACATATTGTGCTTATTTTCCATTAGGTAGAGAGATAAGTAGGTGGCTGAGATAAACATTTT
ACAATTTCGCTTACATGTGTATGTTTAAATTTATTTTTTAAAATAAAAGACATAAAAGAAAAAATA
AAGGAAATAGATGCGCAACATAGATGCTTAACAGAGTGAAGTCTAATATTTAGAGGGTACAA
AAACCTACGGAGGAGGATGGAAAAGGATAGAATTGATAAATATATGCATCTAAATATCCAAA
CCCTCATAGGTCATATGCACCTTCCATAAGCACTTGAACCTAGAGAAAGTCTGCATGTCAC
GTATTTTGTATGGTATGGCATTCCATACTAGTGGTCCTCTACAGTGTATTGAGAATTTTCTGTAG
TTGGTGCGAGCAAACTGCACAGATAGACCATCTGAAGATCTGGTGAAGTGATTATGCACTT
CCGTCCTAGGTTTAAAATAATCATTAAACTCTTTGGGGAGGAGGTGGTTAGAATATTTATACA
AGAAAGTTCCTATTAGTTAAGTGTTAATATTGCCAAATTTCATTATACCGAATTCCAAAAATCTC
AAAGAGGTGTGAGCAAGATATCCATCCCTGGCAATAATTCTTATAGCTCTTTTCTGTAAGAGG
AAAAGACAATTAATCCTGGAATAATAATTCGAAGCCCATACTCAGTGTTGGAAGTAACGCGT
TACAAAAGTAACGCGTTACTGTAATAAGTTACTTTTTTGAGTAACTAGTAATATAACTAGTTATTT
TCACAAAAAAGTAACTAGTAATAATAACGCGTTACTTTTTCGAGTAACTAGTAACGAAAATGTT
CGTTACTTTTTCGTTACTTTTTTGCGTATTAGCGTATACACGCGAGACGAGTCATAGCATACA
CCGCTCTCTTGGTGTTTAGATCTATAGTTCGTTAAACTCCCGTTTCCACATATTCTTAGCCCG
TTTTCTTGTCGTCTATTACAGATATTTGTGAATCATGCGTCGTACAAATGATACCATACACTGC
CCCCTTGGTATTTATAGGCTAAGTGTTTTATTTGCATTGTTTCCAATCCACATCGTGCGTTCG
GTTAGAGGAAATAATTATTCTTTTCTTATAATAATTACTAGTCTATAGTAGCTAGTAAACTTTGGA
AAATGGTTATTGGAGGGAAATGCTCACTTCAACAATCCGTTTACCCGGAATTCTTCGAATTA
ATCACGATGGACTCTAAAGTCATGTAGGCCTAGGCCTATTTCAAGCTCTGTCCGCCAACAA
AGAGACGCCTCGTTTCTTATTTAACTGTTGCTTAATTTTGTAGTTCATTTCGGCCAGTAAGGCT
ACACTAAAGATGCAGCTACGCAAATACTTAGAAAACCGGCTTCATTACTATAATACGCACAT
TTTGATAAATTGTAATGTGAGAAACTGTACCAGACATATGTAAAAGTAACTAAAAAGTAACTAG
TTACTTTTTAAAGTAACTAGTAATATAACTAGTTACTTTTATAAAAAAAGTAACTAGTAATAGTAAC
GCGTTACTTTTATGCCAAAGTAACTAGTAATAGTAACTAGTTACTTTTAAAAGTAACTAGCACA
ACACTGCCCATACTACATTACCATAAATCAGATAAGGATTAATCAGTGTGTAATATAAGTTAAC
CAATATCTTTGATGGGAGAAGATAAGCTATCCTTCTAAGTAGGCCTACACCTAGATTTTTAGC
AATTTTATTAGCAATTATGTTAATATGGACATCCCAAGTAAGGTGTTCATCTATGTACACACCTA
AAAACTTAGTAGATGAGACTAATTCAATGTCAGTGCCATCAATCGTAACCTTGTTAGTAAGGT
GAGAAATCTTTTTCTGGGGAGTACGAAAAGTCATGTAGCAAGATTTATTACGTTCAGTGACA
GACGGTTCGCCCTGAACCATTCACTAAGAAGTAATAGTTCACTATTAATTATATCAATTAATAC
TACTTGGGAATCATGCGAATAAAAAGCATTAGTCTCATCTGCAAATAAAATGATATTAAGTAAT
TTGGATGCATTGGGCAGATCATTAATAGCTAGTGCACATTTCCCTGCTGAAATGTGCACTAA
CTCGGAGGCTAATGATATAAAATGAATACTAATGATAATAAAAAAATTTAAACATTGGGGGGC

GTGAGCACGGGCCGTCTGTGAGGAATTTGCATCCATCTCTTATTTATTTATAATCGAAGCCTA
GTGAAGGGAAGGAAGAGCAGGAGCCGAGCGCGTGAGAAGACAGTTGTCGCGCTTTCTT
CCATAGCCTACCTGGATGGGAGAGGGAAAGAAGGACGCTGACATTGCGCGCCAAGCTG
CAATGCATATAGGCAGCCAACACGCGGTCTTCTTCGCCAGTCGTCAGAAGATCTCGAAGA
GATGAGACCTGTGCCGGAGTTATGTAGTGTCAGTCGTTTCGAGATTCGTACGGATACGATTC
AAGTCGTTACTTCGAGGGTTCCAAAGCCTTAAAATTGTGCTTCCCTGGAATCTAATAGAAGT
AGTTATAAGTTGCGATTTCGTCGAAACGAGCTGAAACAGAATTTAACCGCGAAGAATCAAC
GTTGGATCAATTACGCGTTGGATCATCTTCGATCTTCGCCGATCACAGGTATTGCCTTGAAT
TGTACATCGTCGATTGCATCGTTCGAACCAGGATCTATCGTTCTTCGATCTCCCTCGTTCTTC
GTCGATCCCTCGAGCTTCACGTCGTCGATCTACGTCTTATCGCTGGATTTCCCGCGCTTTT
CTGGATTTCCCGCGCTTTCTGTCGACCTACGTCGCGTCGCTGGATTTCCCGCGCTGTCGT
CGATCGTCGGGCCATCTTAGTTTTCCCGCGCTTTCGCCGATCTGCGAGATCAAGCCTCGA
TCTCCCGCGTCTTCGATCTCTCGCGTCTTTCACTCTGGAGCTACATTATTGTCGAGTTCTTCT
GCGTCACTCGATTGAAACTATCTGGATTAAACTGATATCGAGCCTGATGCTGAAGAAGTGAA
CAGTGACGCAAGTCAGGTCAAAACATTTCTTTTATATAATTGATCGTACTTTTAAGTCAAAGG
CACATTCCTTGTTACTTTAAGTTTAGTCATTGTGTAATTGTAATTGAAGTTTTGTTGAGACATTTA
ATAATAATTGCATTAAATCAACATCATGTCTGTTCATTCACAAGATAGCGACAATGAAGAGTTA
GGAGCTATAGGTGGCAATAAACCAATTTCCCGCCAAGTCAGCCCTTTCTCCAGGCCTTCC
TCAGTAGTTCTTATGGAGAGTCTACTTAAACAACAACGAGAATATATGCAACAGCAGCAAGA
ACTCATGCAACAACAACAACGAGAACTCATACAAAGCGTAAACACAATAAAGGATCAATCG
GTCACCACTAATTCTACTTTGGCTGAGCTTTCTAGATCACATACTGAATTACACAGATCTCAG
ACTGACTTAAGTGCTAGATTGGAAAGAATAGAAAGAGCTAGTCTGGATTTGAACCCAGTGGA
ATTAAGGACGAAACCTTGTGATGGTAACTTCGTGTTTCATGCTGTTCCAACCCAAGTAACCG
CGCCGTTAGCCACTCGACCCTCGATCACCTCAGAAATAAGTGAAGCAATATCCCCTTTTG
GACGACTCTCTCGAGCTATAGAAGGTCCCCAACTGCCTAGAACTGATTGCCTATTGTCTGT
TCCACGGAAAACACGGCTAACCTCGGAATCAGTAACTATCTCGCCAGTAGCGCGGCTGA
GAGAGCCGGGGTCGAAGCTCAAATTCCCCACACGTCCCCCACTTCAAACTTTGGGGAA
CCGTCATCCCCAGTCCCCCTAAGTCTCTGCCCACGTATGATAGTAACGGAACTGATTCAAT
GACTCACCAGATCAGGTGTAGGCCTACCACCATGCTTCTGTGTCGTTAAATAGTTTAGAGTA
GCATAAGTGCTATAGGCCCTAATCTCTGATAAAGAAATTATCAAATAGAACGTCTGTTGCACT
TACTTGTGTCGAGACTGTCACAGATAACGGCGCTGAGCGATAACACCATGGCTGGTAAAA
CCCTAAGTTTAGAAAAAAGTTTTTACGTTTTTAAGTTTTTTCAGGTGTTTTAGGTCTTTAAGTTTT
TTTAGGTATTAATGCGCGCACAATCGTACGCGGAACACTAAACCCAAGAATACGATCAATC
GTAATCGTAATTGTAAACTATAGATTTATAGAACGCCCACAAAAGCGAAGTCGCTGGAACCA
GCTTATTCACAGGCGCTTAACCAGAACAAAATTGATAGGCAGCGGTCAAGACCCAGAAAG
TCAGGCAGGCAGGCAGACAGTCAGACGGCTATATGGTGGATGGTGCTTGGAGTTGAGAC
GGGGAAGGAGGTATGGGGAAGAGGATGAATCAGGAAAGGATTTGCTAAAGAGGAGTGTTT
TCAGTTTGGAGTGAAAGAGCTGTGGAGAGATGGCAAGAGGAAAGATTTGACTTTGCTTGTA
CGATGGGTAAGATATTTGTCGCAATACTGGCGAGAGTTATTCCAAAAGGGGGACAGCCAG
GGGCATCCATCCCCAGAGGCGATGATGCATTTTTCCCTATTTCTCATTTCCTCTTCTTTTTAG
AACATTTGACAGCATGGGAAAATTTTCCAACTTTTTCCACAAAAAGGTATATTTCATCATCCA
AAATTTCTTTTACTCCTATTTTCAAAAATTCACCCTGATTTCGTGGAGTTTACTTGCTTTTGGCA
TACTTTACGTGTTTTTCGCTTCCCCCTAGTTTGGCAACGATGTATTTATGCATCATACAATGCA
CGTACTGGACGCCTCTGGGGACAGCTATGGATATGGAGCGATTGGAACATTTCAGTGACG
AGGTGACAAAAGGACGGAGTAGTGTTAGAGTGGAAGAGGAACGGGTTGAACTATATAGGT
GGTTGGATCGTGAACAGCTGACAGAAATAGAAGAAGGCCTTCACGAAGATTAACTCATGC
ACTGCTATAGTTGAAAAAGGTTATAAAATAATATCAACTCCGATGAATTCAAAACATTTACAGA

GTTTGACATGTAAAAGTAAAAAGTCTGACTTAGAAAATCTAAAACCTTACTTTTCAGGTTTTCA
AGGTTTTTAACGAAGCAGGACGTTTGGGCGCCGGCCGTTTGGGCGCCGGCAACGGAAA
CCTTCGCAGTTATCATTCCAGCTTCTATTTTATCAAGACAGTACTTTAATCAATCTCATTTTCAT
CAGTTGAACACATGTCAAGCACGAATTCGTTATTTCTTTCATAGCTCCAACTTCTTTTTAAGTC
ATTATAGGCATCATTTTCTTCAACAGAATATACACTTAATCAGAATGCTTTGCATGCATCCAGT
TCCTATAGTTCAGTTATACTATGATTTATGTCAATCATCCCGCTTCCACTTTCAGTATGTTTATTG
ATTTTAATCAACCTGTCGCACCAAACGGCAGGCGCCCAAAAGTCCCGTACCCACCTAAA
AACCTAGGTTTTAACCTTCGCAGGACGGAGCGGGGTAAATAATCACCCCAGCGGTTTTTG
AATGCTGATTGCGGAGCAACTGTAGGTAGGTACAATTTGACGTTCCATGTGTTTACAAGTTAA
CACCTGTGACATATTTCTGGACCATACCCATGTCAATGGAGAAAGTGCAAAGAAGAGCCA
CAAAAATGATTCAGGGCTTGAAGTATTTAAGTTACGAGGAAAGGTTGATAAGGTGTGGGCTA
ACAACACTGGAGAAAAGGAGGAGCAGAGGAGACTTAATCGATGCCTAGGCCTATAAGATT
ATTACTGGAAAGGAATCAATACAGTGAGAGAGATTCTTTGAGTTGGCACCATGCAAGGTAA
CTCGGGGGCACAGATACAAACTATTTAAGAAAAGGAAAGGATCATTAGGGCAGAAATTTTG
TAGTGCAAGAGTTGTAGACTTATGGAATGAGTTGGATGATAGTAGGCCTACTGTTTCAGTGGA
TAATGTCACAGCATTTAAAAGGAAGCTAGGGAAATTGGGCTATTAAGCATTCCATAGTAGTGT
ATAGTGGTTCTAACACAGACTACAAGTCTTCTGAACGACTTAAAACCCTTGTTATGTTAATGT
CACGTTCCGTTAAAAAGATTTTTGGGTCCAAAGTGACTGTAGGGGTAAACACTTACCCCGC
TTCGTCTTCGACGTCGTTATTTTTTTTTGTAAAGAGTGAAAATTGATCTAGGTTAATGGTTAGTTT
ACATTCATAAATTAAATGTTGAATCTACCATGGATCACAGTATCAATACTTGCAAACTTGTGGT
GGAAATTAACAGTTTCAAGCTATTGCCACTAGGAGGAATCGATTCCCTGACCTTGATATTATA
AAAATCGACTTCTCACTTATCTATGTGGCTGACCTATGAGCTGAAATGTATGTCTGTTTTGGC
GGCCATATTGGCCGCGATCTTGGACATTTGCATTTCCAGCCTATTGCCACTACGATAGATC
GATTCCCTGACCTAGGGATTATAGAAATTGACTCATCAATTATTTTTGTAGCTGACCTATGAG
CTGAGATATACAAATAATACAAATATATGTTTTGGCGCCATATTGGCCGCCATCTTGGAAATTT
ACGTTTTCAGGCTATTGCCATTACGAGGGACCGATTCCCTGACCTTGATATTATAGAAATAG
ACTCCTCATTTGTCTATGCATGTAGCTGACCTATGAGTTGAGATATTGCACAATTTGTTGGGG
TAAATAATTACCCCGCTTCGTCCTCCGTGTTCCGCAAATAGGCTTCGTCCTGCGAAGGTTA
AAATCTAAGAATAGGTATAGGAATGGGCCTGGACCATAGATTTAATAAACCTTATAATATCTAT
GGTCTGGACAGCCGTGCATTAGAGTAGAGCATCGGTCGATCTGTGTTTAGCTATAAGTTTTT
GCTAAGGGGTCATTGCAAAATGACGTCACGGTCGAGGGTGGGTGAGTGGGTCCCATGAA
AACATAACAAAATGTGGCGGGGTGGGTGGGTTCAGCTGAACGTGACGTCACGCCTAAATT
AGACTAAATTTGGGTATGAATTTTCCAACGGTTATTGCCAGACAAATCAATCTCGCATCTGTC
GTTTACAAAAAATGAATACTGCACTAGCTATTCTCTATTCTCATAGTAACACCCATCCACATTT
CATGAACAATAAATGACGTCACAGTGAGGGGGTGGGTAAGTGGGTCTGGAGAAACCTGAC
CAAGCCTAGCAGGGTGGGTGCGTGGGTCCAAAACGGCAATTTTCAGAGTTACATCGTTAT
GCAATGGGCCAATGGCCAATAATCAGTGTTTTCAAAGTGCAAAACTCTTTATTCAATATAATA
ATCTTCCTAATGACTGACTGATAGACGCAGCACGCTGTTATAGCCGTAAACAGCACGCTGT
TATAGCCGTAAATCCAATCAGGTCGAGCCATAGGCAAGACACAGCCCACTGCTATGAATC
CAATCAGGTCGAGCCGTAGGCAAGACACAGCCCACTGCTATGAATCCAATCAGGTCGAG
CCGTAGGCAAGACACGGCCCACTGTTATGATCCGCCGCCAGGGCTGCTGGCCTCGCA
CCGTCGCCCGCAGCCTTCTAGTCTGGGCGCGAGTCTGAACGTGGACGTCTGAACAAGA
GTAAAGAACCTCAAACTGGGTCTGGGTCCATTGATTGCTACACGCGTCGTTCGAATAAGG
GTTTACAACTATTTATTATAAAAGAGGCGTCCTTGCATGGCGCGGCTGGGCGCGGTCAAC
CAATAGAAGACGGTGTAAACTCGAGAAAGGGAGGAGGTTTGACTTGCCGTATGAACTAGA
CGCAGCTCGCTGCTATAAGCTGAGGAATAAGACCAACACGCCAGACTCCAAACGCTCTG
CTTGACACAGCCACTTACTGCCGTAGCCATAATGAACTAGACGCAAAGCGCGCTGCTATG

AATATATGGACTTCACATGCAATAAGCCGGACGCTCCCATTAGGCTCTCTCTCACACACAG
AGAGGCAGAGACAGAAACAGTAAAAACTGAACAGTGAGGCAGAAATAGACAGCATAGGC
GAAATTCGCCGTTAGAACATCTAGTTATATAATAATTATACAAACGGTAGTCGTTTAAAACTTA
ATTCAGATAAACTTGTATAGCATAGTCTGTTCCGACTCTAGGGCTGTCCAGACCCATTTAAAA
AAACTAGGTTTTTGAAAAAACCTAAAAACCTGAAAAGTTAGATTCTTAGGTTTTTGGATTTTCA
AGTCATAATTTTTACCTTTTCATGTCAAACTCGGTAAATTTATTTAAATTCATTGGCGTTTCTATT
ATTTTGTAACGTTCAACGTAGCAGTCAGGGGCTGACCACGATGGGTGTCATGGGTGACCG
GTCACCCACCAAATTTAGAATTCTTGAAGAGTCACCCACCATTTTTCCAATGGCCACCCAT
AGACACCCACATCCAAATTTGCATAATATAATTATAAGCCTACTATAAAATTGTAAAGGAATCA
TAATGTAGCCCATAGTTTGCAACACCTTAGTCTACACTACAGGCTACAACATGAGCCCTACA
ATCAGGTCAAGGTTTTTAAAAAAAGTTTTTAGGTTTTTAGGTTTATTGTTTTTTTTAGGTTTTTAG
GTTTTAATGTTTTTAGGTTTAAATGAGGACATTCGAAAGGAGATAGGAGTGGCATGCATAACA
GACAAAATACGGGAGGCCAGACTGAGGTGGTACGGACATGTGGAGCGATCAGGAGATAC
CAACATCAGAAGAATAAGGAAGTCAGAAGTACAGGGACTTCGCAGCCGAGGAAGGCAAA
AGAAGAGATGGATGGACATGATTCAAGAGGACCTGAAATTTCTCAACCTGAAACCAGATGA
CACCGGGAGAAGAGACATGTGGAGGCAAAGGATCCGAGTGGCTGACCCCTCGCCGGC
GGGGGATTAATTCAAGCCTGAAGGAAAACAAGAGGTTTTAATGTCTACGCAAAGTCGCAC
GCGGAACATTGGACATAAAAATACGATCAACTAAAAAGCTATAGGCCTACACGTAGACTAA
CTCGGGAGCAGGACATTACCCCCCTAGGACATTACCCCCCGGACATTACCCTCCTGGAT
ATTACCTCTGTCATTAAATCAACAATTATGTATGTATGCATGCATGTATGTATATATGTGTATGTAT
GCATGTATATATGTAAGTATGTACTTGTATATGTATGCATGTATGTATGTATGTATGTTTGTATGTAT
GTATGTATGTATGTATGTATGTATGTAAGTATGTATGTATGTATGTATGTATGTATGTATGTATGTAT
GTATGTATGTATGTATGTATGTATGTATGTATGTATGTATGTATGTATGTATGTATGTATGTATGTATG
TATGTATGTATGTATGTATGTATGTATGTATGTATGTATGTATGTATGTATGTATGTATGTATGTATGT
ATGTATGTATGTATGTATGTATGTATGTATGTATGTATGTATGTATGTATGTATGTATGTATGTATGTA
TGTATGTATGTATGTATGCATGCGTGCATGTTTGTATGTATGTACGCATGTATACCAGTGTGCT
GTCATGTTACTAGTAGAATTAATAATAATCTTATCTTCTACGTACATATATATACACGTACATACA
TACATAGGCTACATACATATATACATACATACATGCATACATATATACGTACATATTCACATACA
TGCATACATACGTACATATTCACATACATGCATACAGACCTCCGTCAGTTCTCACATCCTCC
CAACCGAGCTCTTAATTTCACCTATCCTCCGCTCGCACTCTCCTCTGCTACTTTCCATTCAC
GACTGAAAACAGAACTCTTTAAGATATCCTATCCCGGCTCTACTCCTGCACCACAACACGT
CCGCCATCATCACCTACTCCAACCGTAGCACCACGCAGTCTCCCCGGCTTGACCTTTCC
GGATTTTGACCTGGCACCGAAATGAAACGAGAAGTTTGGCTATTGCGGACTGGATTTGGTA
TAGCGCCGGTGAATAAGCTGGTCTTCCTGACGTGGCTTTTGTAGGCGCTGAGAAATTTTCG
GAGTTTTACATTTACATTTACATACGTACATGCATACACACATGCATATACAAGTACATACATA
CATATATATACGCATGCATGCATACATACATAGTTGTTTAATGGCAGGGGGTAATATGGGTTG
GGGGGTAATGTCCTAGGGGGTAATGTCCGGGGGTTAATGGCGGGGGGGGGTAATGTCC
GGGGGTAATGTCCTAGACCCGTCAACAGATGGGAAAGCACTGTCGAGTATGATACTGGAT
GTTCTGTTGAGGTGCAACTTGTCAGTAGCCAACCTTAGAGGTCAAACCTTCGATGGGGGC
AGTAAAATGGCGGTCCCAATCAATGGTGCACAAGCCCTCATTGCAGCCGCTTGCAATATTT
GATCATTGCCTGATACATTGCCTCTTGCATTGCGTGAACCTCATTGTTGCAGACTACCTGGA
AGACTCTACAATTGTACCTGATAAAATGGGTATCGCCAGTGATGCAGGAGTACTGTTTCAATT
TTCTACGCAATTAAACAACCTCATGGAGAATGTTAACTTTAGGGCACACCTCCACATAGGC
CTACGGCCGCTTTGTCATACACGAGTTTTGTGCAGAGCAAAAGGTATTAGGGCTCTACTGA
CGAACTTAGGAAGTGTTTGCTTTGCTGCATTAGGCCTACTCTGATCAGAGCAGAGGAGAAT
CTAGCTCCAAAGCTATATGACTAAGTCTTCATTGCATCCATGAGCGATGGAAGTAATTATGTT
TGCAATTGACCGTTCATGTGATATGCATGCCACTGGAGCTTTGAATTGCGGCTGTTCAGTTA

AAGAAGGCCTCTCTTTTAGACATCCCATTAGTAAAGCAAATGACTTGCAGTAATCTGCAGAA
CATGACAAGTGACGTTAACTTTAACAAACTCTTCGGTGAAACAGAGAAAGTTTGCTCAGAGT
TGAACGTATCCAAGCCACAGCTTCCATGCCAGCAAAGACCAGCGTCAAGATACACGGAT
TCTGGAGAAAGTTTTTGCCACAAGTGGAATTCAGCAGAAGAGTTCTTCAGAAATCACTACTT
TGCGCTATAGAGCCCTGCCGCCGACTTTTCGTCATCGGCGCGCCGCCGCCGATATTTTG
TCGGCGCGCCGCCGATATTTTGTCGGCGCGCCGCCGATGCGAGCCGCAGATGCGATTT
TGTGTGTGTTTATTTTCGATTGCTAAAAAACATAAGGTCATTAGAAAATTTTGGAGGATAGAAC
GAATTTTTTCTGGATTCCTTGAAAAAAAATTTTTTTCCCGTTCACGCCGCTCCGCGCCGCC
GCCCACGATTTGTTTTACCGGCGCGCTGCAGCCGACCCAATGACGCCGGCGCATGGAT
CTACTTTGCGCATATTGGTGCTGCAGTGAATGGGTTACATCATCGACTTATATTCAGTGGAC
CCAGCATGCGCCAGCTAGACCCCCAAGCCGGCTTAGAATAAATTTCCGGCCGGCTTAG
CAACAATTGCGGCTTAAATTTGGCTAAGCCGCAAATTTCCGGCCGGCGGCGGCTTAACA
AAAATCCGGCCGGCTGACGGCTTAAATTCGGCTAAGGATAGTTTTCAAAAAACACTTTTATT
TATAAAGAGAAGCAAATCTAAAAGCAAGAATCCAATTCACTTAATGTGCGAAGGCGTTCAAA
GTGCTGATCAATTTTGTAATAATATTACAAATATGCGTTCAACTTTTGGGCCTCTTGGCTCTGA
CCGACTGCCGGAGACTTCGGGTGATCAGATGCCAGCTCACAACTTTACCTGTCAACATAA
CGCTGCTTCACGAAGAAAGAATCAATGTGCTGCTCGTCAGTAGGGCCCACAGTTTTTCCG
CGGTACCGCGGAATTTCGCGCCGAACCGCGGAATTTGGGTTTTTGCCACGTGATGATGAT
TTTGCGTTTGATGGTGATGGTTGAATGATGAAGATGATGATTAATGAATGAATAATGAAGGATT
CATAAGTTCATAACATGTATTTGGCCTGTTGATTCATGATTTATTCAAGCCGCGGACAGTTAA
CTGTGTAGCCGCGGTGAGTCGCGGAATTTCATCTGCCTTTCCGCGGTGAACCGCGGAAT
GTTTAAAAAAATATCCGCGGAAAAACTGTGGGCCCTACTCGTCAGTCATTGACAAAACAGA
AATACTGACATTCTATTCGCGACATTTTATTAAATTCAATATAATTAGTTTCGCAAAGTCATTTTC
AGTTCTGCATCACAATTATTATTCATTCGCGTTTTTGTAAGACCGGATACGCGTCACTTTATG
GTGTAGGCCTACACAACATAGAGCTCAACATAGAAATCTATCACCCACAGAGTTCTAAATAA
AATTATCTTCTCGCTCTTGCCGATCTTGATGCAGCACTGTTACTTAATGACGTGATCGTATGA
ACTCTGAACGGTAATTGCTAATTTTTTTTAACATCGCGTGGTAACGCCACCTTTAACCGGAC
ATTTTGCGGCATTTCCCGGTCCTGCTGACGGCTAGCCGCATTTTATCCGGCGGCTAAGCC
GCATATTTTTTCCGGCCCGGCTGACGGCTAGCCGCAATTTTTCCGGCGGCTAAGCCGCA
TTTTTTTCCGGCCGGCGACCGGCGGCTAATTCGGCGGCTCGGGGGTCTAGCGCCAGCT
AAAGCCGTTCTGTAAAATGAAGCCTACGCATATTATGCTAATGAGCTCTGCCTGACGTCAC
GTATGGCAGAAAACTACGTGGCTGCCGTTAACGCGCATAATACTTTAATATCGTAGGCATCA
AGACCTAGGTTCTACTTTCAGAATTTGTTAATAAAGGCGTATAATTTGTAAGGAAACGAAGGA
TACAGTGAATCTGGATAATTTGCTATGTTTTTGGCAAATTTAATCTGGCTGTAGGCCTATCGAT
GTGCTGTTGTTGTTGATCCAAACACGTAGGCCTAGGCCTATACAAGTTGAGACATCCAGAC
AGAAAGAGTGATTCAGAGAATAGAGACACAAAGAGAGAGTCAGACAGACAAACCGACAC
ATAGAGACACAGAGAGACACAGAGAGACAGAGAGAAAGACAGGCAGACACAGAGAGAC
AGAGAGAAAGACAGAGAGAGACATAAAGGGAGATGGTGAGACACAGACAGGGAGATGGA
GACACACAGAGACAGAGAACCACAGACAGACGGAGATAAAGACACAGCCAGAAAGAAA
CACAGGGACAGAAACAGAAAAAGGGTCACAGACAGGGACACAGGCAAAGACAGTAGCA
GAGACACAGCCAGCGACAGAGAGGGACAGACAGATACGCTGCATACGCAGAGACAGA
CACAGACAGACAAACAGAGGCAGGCAGACGGAAACACAGGACAAAGACAAAGACAGA
ACCATAGACAGGAAGAGACAGAGGCAGGCAGACGTAAACAAAGAACAAAGACAAAGAC
AGAACCCTAGACAGGAAGATACAGAGACAGGCAGAGAGCCAAACAGACAAGAACACAG
ACACATAGATACAGAGACCAAGACATACAGAGATAAACAAACAGGGACATAGGCACATAG
AGACACAGACAGACAAACAGAAGCAGAGGCACAGACATACAGATACAATGACAGGGAGA
CAGATACAGACAGACAGGAAAACAGACAGACACAGACATGCAAAGACTCAGACAGAGAC

AGAGAGAGGCAGTGAGATAGAGGGACACAGACAAAGATAGAGAACCAAAGACAGACGG
AGACAGAGACAAACACAGACACAGACAAACATGCAGAGACAGAAACAGAAGCAGAGAC
ACAGACAGGGACAGTAGAAGATACACAGCCAAAGACACAGAGGGACACAGACAGAGGC
ACTCGAGAGACAGACGCAGATATACAGAGACATATGTATACGGACAGAGACAGACAAACA
GAGGGAAACTGAGAGACAAATACAGAAAAAAGACAGAACCGCGGACAGGCAGAGACAG
AGGCAGACAGACAGGGACACAGATACAGAGAGACAGAGACTAATTAAGACAGATAGAAA
CACAGACACAAACATGCAGAAACTCAGACAGAGGCAGGGAAATAGAGAGACATCGGGAT
AGAGAGAGAACCACGGACACACGGAGACACAAACACTGCAGGGAGTCAGACACAGGC
ACAAAGACAGACAGACAGACAAACAGAGACAGAAGCATATTTATGCGGGAAAATACTTGT
GTTATTCTTTAATTAAAAATTCTTCTTTCGCTTAAATAAAACGGATAGAATACTACTAAGACAAC
CATATTCCCATTATGCTCGTCTTATTCTTGCCATAAGTGACGTCACCCGCGCGCAAACGAC
CCGACTTCAGTTTGGACTAGTTCATGCTGGGTCCACTGAATATAAGTCGATGGGTTACATGC
ACGCTACACTCAATCTGGACTCCACAATATCGGTCTGGGAAAGATGGTGTTGCTGCTGAAC
TGTTACTATAATATTGCACCTGCTACAGCTGGCTATCCAGAGTTGTGTGCAGAGTCACTGTC
AACAGAACCCTGAACAGAAGGGATGGAGTAGATGTTCTTTATCTAAACGACCTTTACATTTG
ATGAAAAACGTTCTTTGATGAAATGGAAGTGGGATTGATGGAAATAGTTCGGTCCGTAAATA
GAAGTTGGATTTATGACACTTAGTTCAGCTTATATAGAGCTTTTCATCCGGGGGAAATGTCCG
AGGGAAAATGTCCGGATACCAGTCTGATAGCCATTGATAGAGGCTAGAGCCGCTAAGCGG
CTAGAGCCGAGTTTAGTCATTGAAACAATTAACGATGTGCAATCATGGATATTTACCAAGGA
CCTCCTTGATATTTACTCTCCTTAGTAATGAATCAACTGGACCGGAAGAAGACGTTGGAACA
AGCGCAGCTCAGTGACTTGTCATTTTGGAAGAGTCGTAAGATTTGCACAACGTAGAACGTA
TTTTGAAACACTTCGGCACTATTCAGCAAATTTAGACATTATCAGCACAAATGGCGGAAGAC
CAGAAGTAAGTCTGATTGCCACTGTGAAATCAGTTTTTAAACTGGAAATATTTTGACATCTGC
GTATATTATAAACCATTAGAGAGAAGGCGTCGGCATAATTTCTTCTATCCTAAGGATTACGTG
GACAGTTTTTGTGCCGTTTTACGGTCATCTGATTGAAAGTGACATTTAAAATACCAGGCTCAA
GAGTGCTTGCCAAATGTTTACATATTCAGTGACACGAGTGGTGTGTGACAACAAATTTATTAA
AATTGGACCAGCATTGATTGACTTCCTTTATGACAACACTAAACTGGAAGTAACAATAAGACT
ATTATGTCACACTGGCGTGTCATTGTTTTCATAAAACTCGATTTGTTGTAACATTGACTAGACT
GTGATTCAGTATGTGACATTTTTGTCGAGTGATAATGAATATAAAGGCTACATTATTGAGACTG
AAACGTTTGGTTTTTCCGCCTCAGAGATACTAAAACTGAAAGTTGTTTATTTAGGCTTTGGATA
TTTTCATCTTGTGTGATCAAATTTAGTGAAGGAATACTTTGTTGTTTTGACTGCTTTCCAATTGT
GTTTGACTTGTTCTTTAAATTTGCACCCCAAAATCTGCAGCAAGTAATGTACATTACATGATGA
GATGAAACATAGACACTAATGAAGCATAGATTATCACAATTGTCAATCTTAGAAGATCATAAG
AATCATGATTTCTGAAATCCCCAATCTTAGAAATTGAGACACAGCTGATTCCAACTATTTTTCT
AAGTGAAAGTAAAATTATTTGCACAAATCTCACTTATTTGTGATTTGTACTTATGCTCCAAGGG
TAAGTTTTCGGGATTTCAGGTAGACCCCAGAAGATTAGGCCACCCATCTCAGTTTAGTTGC
CAAGTTATTTAAGAGGAACTGGAACACAGAGAATAAATTTCAAGAAAATCCACATGATATAAA
GCAGATAAGCTCATGTTACGGTTGTTACCGCTATTTAATTTTGTCCTGTCATTGACATTTGATTT
GTTCATAGAAGGCCCAACTGCAACTATTCTAAGATAGACACTGTAAACTATCTTTGACACCTT
TTAAAATCTTTGTCAGCCACATTGCGACTGCATCCTTTGGAAAAGTTTCTATACTTGTTTCCAA
CTGAATAAAATCCAAACGGAATGAATCATTTAGCTGTCACTTGAACCTAGAACTTCATATTTT
GTTTCATTGTCCCAGATAGTAGAAGTAGGGAGGTCTGCATTTAGTGAAAGGCAATTGAAGAA
CTTGTCATTTTGTAAATTCTCTTCTTCACTTTATTTTGCTTTTGTTGCAACATTGATGATCATCAAT
CATATCAGCTGCTTGTTGTGCACACAGTCCATGTAGATCACTTGGACAAGTACATTCCCATA
CAATTGAAGTGAACTATAACTGAAGTTACAATGGATATTATGAGCATAACTCGGCTGTGGGTG
AACTTCCTGTAGGCTGGCAGGGGGTATCTTCATCAGACCCTTATTTGGCTATTTGGAAGTTG
AGTAGTTTATGAATAAATTGCTGTCATAAATTAATGTTGTAGTGGTGTGTGACAAGACATTGTTT

TGCTTTTTGCAGCAGTGATGATCTTGCCACCGCAATCCTGCGCAGGAAGGCCAAACCGA
ACCGTCTTCTGGTTGAGGAGGCCATCAATGAGGACAACTCTGTTGTCTGTGTCTCTCAGGT
AACTGCCAGTGCTCAGATGTGTTGTCATTATGGACGATACATCTGCTCTAGACGAAGACAA
CTCACTCCGAAGTAACAGCCCAAGTTCTGGTGATGAAAGTATAAATGTAGAATTCAACAGTT
GAAGATGTTTATTTAGAGCAGGGATGCACAACCTGCTGGCTGAATATGTTATATCCAGCCCT
CAGAGCAGGCTAAAAATACAAGAAACTTCTCCTGAATGATGGAGATTTAATAAATGAATTGAA
TTGTCAACAATTTTGCAACTTATTACAATCCAAAACAGCTCTGATGGCAATATTATCAAACCA
CAAGCAATAAAAAGGCTGGTTAAATCAGGTTTTTATACTCGGCTATCGAAAATTCAGCTCAA
AATTTTGTCTTTGGCCTTCGAGTGCACTAAAAATACGCTGCGGCCCTTGGACTAAAAAGGTT
GTGCACCTCTGATTTAGAGGAAAGCTTTGCCATATAGACCATTGTGCAAAAGAAAACTTGTG
CTGTGTGTCCATATCAACTTGGCAATTTCAACATCTCTTAATTAAGAGTAGTAATTTCCATTGT
CATTCATTCATATTGAACATTTATATAGCGCCTCTTCAAGAAAATTACTCAGAAGCGCTCCCA
ACACCAGCACGGTCAAACAAAGCAGTCTTAAGGTGAGAAAAAAACGCAGGTGAAGTCGT
TCTGTTAAAAATGCGAAGTTGTTAAAAATGCGAAGTCATTGGTTAGTTACTTTTGGTGAGTGTT
TAATTGTTGGTTAATTTCACAACTTATGGCATAGGCAGAGTTTCATGTGCCTTATATGTTGTGTG
AAATTAACAGAATATATTTACAACTGTATTTAGTTGCTCCTTTGTGTATGGCAGTGAGATGCAA
ATGTGGATAAATATTTGTCAAATCAATGCTTAGTTCACTTCAGCAGATGTATCTCACTCATACA
GCCAGCTATGCATAAGCACATGTGCTAATGCTGGCTGCAGACATATGGCTATTATTAGTAAA
TACTTGCCAAGCGGATTTTGTTGGTATTAAATCCTACCGGTAATTTGTTTTATTAATAAAATTTT
CAACATTTGGCAATGGCTGTCATTTAACGGGAGTTCTAGTCATTGACATTTTGAAATTAACAA
TTATAACCTGTGCTATTTGTTATGATTTTTCAGTGATAAGTTCTTGAATCTCTGTCTCACATAATG
AAAGGATCCAAAGATAATGGGAGATGGGCAGAGTGATTGATAAGAAACTGGTGTGAAGAG
CAGAGGTACCATGTTCGATTCCCACTACCCCCATACAGACTACCCCATACACCTGACTAG
TGCTCGACAGTGGCCGGTTCGGTTAGATGTAGGTTGTGATGGGCACCCTGCAGGGCTGC
CACTCAGTTAAATTCAACTCCCGTGATAACTTGTAATCGTTCGTTCATAATGACTTTGTAATCA
TTTTGCGGTCTGTCAGCATGAACATTCAATGAAGATATTAATAATAATGCTAATAATATCGTCTT
TATTAGTTCGGGCTGCCAGGAACAATATGATTACCAGCAATCCAGTATATAATATGAACAGAT
GATCACAAGTTATTACAGGAGTAGAGTCTTTCTGTATGCAGTCATGCAAAGCATCCATCGCT
TCCTACATATAATTTAACCAGTGCTGCTGCTTAGTGAATGGAAGGACTAGTCAACCTTAATGT
GTTGAGTGAAAGATGCTACACGGATGTCTAAGTTACTACTGCCCTTGACTCTTATGTATGTAG
ATGGCCACTACACCTAATCTACTATTTATAATTACTTGACATAGCTTATTTGAATTTCAGTGAAA
TAAGATTCAGATTCCTATTTCAGTGAAATAGGAATCTGAGTGACGATGCCTGAATTGGAGAAC
TGTGACCTGGGTTATGTGACAGATTTCTTTCTCTTTATTAGAACAAGATGGATGAGCTTCAGTT
GTTTCGTGGTGACTCGGTCCTGCTGAAGGGCAAGCGTCGGCGTGAGGCTGTGTGCATTG
TCTTATCAGAAGATACTCTCACAGACGAGAAGATTCGCATCAACAGAATCGTTCGTAACAAT
CTGCGCGTCAGACTTGGCGACATCGTCAGGTCAGTTCACTTAGTAGTTTTCTCTAATAGTTAT
GCGCATGGTGGTCTAGTGGTTGGTGTGCTGGAATGCCAATCGAGAGGTTTGGGGTTCAAG
TCCTGACCAGGGCAGAAATTTTGTTTGAGATTTCTGCACCCCTGATCAACTCAGCAATGAT
GAGTACACTGACCATACACTGTCAGTGGGAAGGTGAGGGAGAGGACTGGCCACCCGCC
CTCATATGCTGAGGCTAAGAAAATCGGGATGCTAACGCTTCATACACATGGCTGGCCTAG
GTTAAGATTACTCTTTTCCTCTTCATGTAAGTGCTTGTGTCTAAGTTAGGATGACTCAAGTAAC
CAGACCAGAGCTTCCTCCTATAATGTTAGCAAAACATTTCTTCAGGGTATATGGTTAACAATT
TTTTGTCAGTAGCACAATGGTTTAAAACCATTCTGTTTGATCTTGATCTTATGTAGTAAGTCAG
GAGGACAGACTATGAGTTAATGTGTTTCTTCAAACAGCTCAATATTAATTAACACAACTGCAA
TCTGGTATCCTGTCAGGATGAAATCTGGTTTAAACACACACTTGAATAACATTCGATAAGTTTA
CTATCAAGATTCTGACATTTTCTAGTTAATTGTGGCAGTCATTTTCAGGCCAGTGATTGATTAA
TAGTCTAAAAGAACACAGTGATTGCATCGGCATCATCTTGTGGGGCATGGTGGCTCAGTGG

TAAGTTCGGTGCCTTGCGTCTGGAGCGACACAGGTTCGAATGCTACTCTAGCCGCCACAT
TAGGACCTTGGACTAGTCCTTCACTCACAGTTGCGCTTCGGCGTGTTAACTCCGACACGG
TATCAATGCTGTAGTTGGGAGCGCCTCTGAGTAGTAGTGGACTTGAAGAGGCGCTATATAAA
TAATCCAGAATGAATTAATGAATTTTGTAGATAATTAGGCGTTTTGTTACTTATGTATGTCTGGAA
GTATTCTATCATATTCTTTGATGGCTTAATATGGTCAGGGCTAAGTGTTGAAGTGTCACCTGTT
TCAGCATCCAGCCATGCCCTGATGTAAAGTATGGAAAGAGGGTGCACATCCTACCAATTG
ATGACACAGTTGAAGGTCTTACTGGGTGAGGAAGTTTTGACATTCTTTTTCTGTTGCTATTGTT
GGTATTTCAGCAACAGTTCAAACCCAAGTTCATAGGTGTTTGGCTAACGGTCAGGATGTTGT
AGAATTTCATTATCCCGAAAGCCAATCTTTTTTGAACAGTTGAATTAAAGTTAATATGTTACCTT
TCATTACAGCCTATACTTTGCAAAAAATTCTGCATTATAACCATGAAAAGATTGGTTGTATAGC
TATTTAATCATTTCTATAGCTGGACTAATTTCTTCTTGTTTTAACAAGACTTTGATGCGGTGCTTT
CTAGTAACCTTTTTGAGGTGTACCTGAAGCCATACTTCCTGGAGGCATACCGTCCTGTTCAC
AAAGGCGACATCTTCCTTGTGCGAGGCGGCATGAGAGCAGTTGAGTTCAAGGTGGTTGAG
ACGGACCCGGCTCCTTACTGCATCGTGGCCCCTGATACGGTCATCCACTGTGAGGGAGA
ACCGGTGAAGCGTGAGGTATGCTAATAGGCTACTGACCATTGAGTAATAAACTGTATTACAA
TTTTCAAAGTGACTATTGCACCATCAATCGTGCAATAATAGTTACGTACAAAGTCATAAAATAC
TGGTGCACAAAAGCTTTCAAAAGAGTCGTGAGGGTCGGTTAGGGGCAGGGTAAGCGTTGT
GAGGGTCGGTTAGGGTTAGTAGGCTAGTGTAGTCATTAGACGGAAATTTCACCTGCTGAGT
CACATCATCAATTTGTAACCTTCCGCAATCACATGATGTGCTGGTCTAATAGCATTTCAGCTT
GCTGCAGCCATCGACATTGTTGTGGTTATGTCCTACAATTTTACATCAATTTATGGTCGTCAT
GTTGATGTTTGCTTCAAATTTGCCCTTGATTTAGTTTTATTCAGAAAGTTTTTTCAAAAGCAGTT
TGCTATATATAATTATAATGGTAATTTGCATCGGATGAAACGGTAATTTTTTTAGCACAGAATTT
CTTGAAACATTTTTTAAGTGCTAACAAAGCTTCAAATTAATGATTTAAATTAGAGGTGTGAAATC
TGAGAGTTTCAGTTATAAGTGAAATTTTTGATTAGTCATAGAGCTTGACCGATACATTGGCAAC
ACTCACCATCGTGGCCGATTGTAGGACAATAAGCAATCGATGACAATCGGTGACTAGTCC
GTGCCGATGTTTCACGATGATGTTACTCACCGAATTCACCTAGCCATAGGCCACCTGTGCA
GTACTATTCAAGGCTCCGCGCGGTATCTGTCTTTGAAGACTGTTCAAGCGCTGGGGGAGG
TATCTGTCTGAGCAAGCCGTTCTTCTATTGCCTTGTTGGACGAACAATCATTATCGCAGGCC
TGAATTGAATGTGGTAATAAAACCTTATAGACTGGCCCTCGTCTATAGACAAGGCATTTCTTG
TCATTGGTATGCAATTTTCAATAGGCAAAATGATAAAGAGACAAAATTTGATTACCCTGTACC
CAATTGATAGAACCGGTTATCATTTTCGTAACCGGTGGGCATAATTGCACAACTTTGATAGAA
ATTGTGTCCATAATCAAATCGCCAGATGAGGGTTATTCAGTAAACAATCGGAAACACAATCG
GCTGTTCTCACAATCGGGACCATCGGGTAAATGTGCCATCGGTCAAGCTCTAATTAGGCCT
ATTCTTATTTTGAAGGTCAGTCTATATCTTCTTCTGTCCTGTGATGCTTGTCTGATTCTGTCCAG
TTCATTATGTATTTGGAATGTGATTGTCATTAGACGCAGTCATTGTTTCACAGCTGTAATTGGC
ATTGTTTGACAGTATTCCGAAAGTGACTGGGAAACCAATGCAGCCCATTGTTAAAGTGTTAA
ACAATGCCACAGACGTTTCTAGATTACATTTTAGTGTTTACTTTATTATAGGAAGAGGAAGAG
GCACTGAATGAGGTCGGCTACGACGACATTGGAGGATGCCGTAAGCAGTTGGCTCAGAT
CAAGGAGATGGTGGAACTGCCTTTGCGCCATCCTCAGCTCTTCAAGGCCATAGGTGTCAA
GGTCAGTCATATCTTACAGCTTCTGGAGTGCAGTGATTAGCATGCACGGCTGTCAAGAAATA
GATCGAGGCTCAGAAATCTGTCACAGGAAACATTTAAATCAATAAATTTTCGTTTATTAGGTG
GAATGCACAAATGATCACAACTGGCACACATACGCAATTGTGATTTAACTTTGTATTGTATCT
ATACGTTTATATAGTGCTTCTTGCAGTGCACACCATTCAGAAGCACTTAACATGATACTGCTA
CACTTGTGCATTGTTCCAAATTCTGTCATACAATGACTTAGCTGACCATGCACAAGAGTCAG
AGCTGACAAACGTCTGTCAACAATTGTGTATTGTTATTGCACTGAAGCTTTGTCTTCATTTCCA
GATGACGGAAACAGTATGATCTTGCAATTCAGATCCATGTTCAAATGTCCATCAGCCTTTGTT
ATCTTATGTGAATATTTGTTTCTGTTTTGTCTACATCATTAGTCCTTCTGTGAGGTTATCATGCCA

CTGCTTGTTGTCATGGTTTCAGCCACCTCGCGGAATTCTCCTATATGGTCCGCCGGGTACT
GGCAAGACCCTGATTGCCCGTGCCGTTGCCAATGAGACCGGTGCGTTCTTCTTTCTGATC
AATGGTCCGGAGATCATGAGCAAGCTGGCTGGCGAATCTGAGAGCAACCTGAGGAAGGC
ATTTGAGGAGGCGGAAAAGAATGCTCCAGCAATCATCTTCATTGATGAACTGGATGCTATTG
CACCTAAAAGAGAGAAGGTAAGTAAGGCAGCATTTGTGTAATTGTTATAAGAGCTACATGTAT
CTGAGGAAGGCATTTCAGGAAATGCCTTGTGAAATGCCTGAAGTTTAAAATCAATTTTGGAG
GTGAACAGTTTAATGGTATTGTTCGGTTATGGTTTATTTCATGTTTTTTTAATGACTGGTATCTAT
CTGCCTTTTATGAATAATCACAACTAGTGATGCATTTAGCTCTGGGGTTCCCAATTTGTGGTG
CACGCACCTCACTGGGGTGTGACGTGTCATTTCAATGTGCGATAAAGAGGGAACATAAGA
ATACGACCATAGGATTAGAAGTTTATTTCCATCTCAGAAGTTGGAAGTTACATATGTTCTATTCT
GACTTAAGGACTGTTTTCTTTAGCTGGGCTTTGGTCAGGAAAACTCCTGTGTAGATAATCTTG
AAGGGTGCTACATAGATGAATGAAATTTCACGAGATTAGTTTGTTGTAATGAGGTAATACTTTT
TGTTGAGCGCATGAACACTGCCCAAACCTTTCTGATAGGTGTGGGACAAAGAAAAGATTG
GGATGTCGCTCCTTTGTATAAATCACAGTAATGCAGACATGGCCATTATTTTTTTAAAATCAGA
ACTTACAGCGCCAGATATGTGGCCCCCATATTAATATACAGATTGTCAAACACATAAGCGTG
TGTACTGTCAGTGATCGTATATATGGCCATTTTGTAAATACTTGCCCAAACACAATTTTTCTGTT
AGAAATTTGATTTTATCGCACTTTTGAAAATAAAATTTTTTGACATTTGGTGTTTATGTAATGCCT
AGACCCATGGAGAGGTGGAGAGGCGTATTGTTTCGCAGTTGCTTACTCTGATGGATGGACT
GAAGCAGAGAGCTCACGTGATTGTCATGGCGGCCACCAATAGACCCAACAGCATTGATC
CAGCTCTGAGAAGATTCGGTTAGTATAACCTCAGTTATGCTTAAAGGCCTAACTTAGGAAAT
CTCGTTCACCGGAGAAAGGGCGTGTCTAATAAAGACCTTCGGGCCAATCATTGTAAAGTTT
AGGTCTGCCTGGCGGATTCTGTCATTGAACTATGAATAGGTATTGTCTAGGGCCTGGGCTC
TCGGCAGCTGACTCTTCGCTTCGCCGTGATTCAGGTGCCTGTATTCAAGCTGTGAGCGTAT
GCAGAGACTCTAGCATGTAGAAAAGGCCCCGTAAAGTTAGGACCATTCATTTATGGAAGG
GAGGGGGTCAGGCTCAGGTGGACACATATGGACGGGTGAGTGGGGGTCAGCCCTTATCT
TGCGATGTATATGTGTCGTACAATGTCCAAGCCTGTAGCAATTCCAGCTTGGCAGTTTATGA
ACCAAAATCTCGCACATATAAAAACAATATGATGATGACGATGACGACGATGAGGATGATTC
TCTGCCCAAAAGCAGGTTCATTGATTTTCGCCACCTGGGAATGAGAAGTATGTATGTATGTA
TGTATGTATGTATGTATGTATGTATGTATGTATGTATGTATGTATGTATATGCATGCATGTATG
TATACGCATGCATGTATGTATGTATACGCATGCATGTATGTATGTATGTATGTATGTATGTATGCA
TGCATGTATGTATGTATGTATGTATGTATGTATGTATGTATGCACATAAGCTTGATACACTGTATA
CAGGGATTTTACACAGGTGTGCCAGCCAACTTTACCTGGCCCCCTATATGTGGAAATACTA
TTGTCTATAGCAGCCGGCTCACCTTTGTCCCTCGTGGTGATTCGCGGTTGTTTACATTTGGG
TCTCAATTGAACTGACCAGTTTAGGCTGTGCCTGATAAATTACGACATAACAATTCGAGTTCA
ATCACGGCTTAGGGGGGCGACCGCTTTAGCTGGTGTCTTTTCATCCGAACACACATTCAC
ATGTATACATGTATACATGTATGTTTGTATGTATGTATGTATGTATGTATGTATCTATGTATGTATGTA
TGTATGTATGTTTGTATGTATGTATGTATGTATGTATGTATGTATGTATGTATGTATGTATGTATGTAT
GTATGTATGTATGTATGTATGTATGTATGTATGGAATACATGTATACATGTATGCATGTATGCATAC
ATGTATGCATATAGGCAAACATACGTACAAACATACATACATACATACATACATATATGCATAC
ATGCATACATACATGTAAACATGTATTCCATACATACATACATACATACATACATACATACATAC
ATACATACATACATACATACATACATACATGTATACATGCATACATGTGAATGTGTGTTCGGATG
AAAAGACACCAGCAAGTTGAAAGATGGGATAGTTTCATGCTGCAAAGACCTGAGTAAATAA
TTATCTGCAAATGTGCCTTAGGTACGGGGTATTCTTCTACCAAAATGAAACACGAGTCTCTC
AATTCGACACTGCTGTGTGGAAGTGAGAGACTCAGGCATACATATGCAAATAAGCCTATGAT
ACGTAATATAGGACAGGTATGAGCGGAGGTCGGCTATACTCAAATTCAGAGGTGATTTCCAT
ACATACATACATGCATACATACATACATACATACATACATAGGTGTGTGTGTGTGTGTGTGT
GTGTGTGTGTGTGTGTGTGTGTGTGTGTGTGGTTGTGGTTGTGTGTGTGTGTGTGTTTTCGG

CTGGCCGCATCACACGCCGGGAAGGTAATTATTCCCTGGAGAGTTCGATCGGCCAGCAG
GGCGGTGAAGTGAAGCGAATGCTCTCAGTCTGAGTCTCACTCATTGACAACCAAATCTTGT
TTAGCTACGCCTATTTCTGTTCCGCCAGAAGAATTTACCCTTGTCGGGCTCGGGCTTTAAG
GATATTAGATGAGTTCAGGCCTTGTCAGTACTGCATTGTTACTCTTTGCAGTATTATACTGGTT
GTCTTAGGTATTAGTCTAGAAAGGCTGACTATGATTCAGGTAGATAGGGTTTTGCAATATAAAA
TATTAGTTAATCTAATTAGAGTGCAAACGGTTGACACATTGGCAGTGTGCCAAGAAACCGTC
TAAACTAATAGCACAATCGTTGCATAATTCTATCTTGAAATTAACTCTTTGTTGTATTAAGATGA
AGGTTGTAAATTTGTAGAACAAAACTGTTGTGGGGCTTTGAAGAAGAGCAGTCTTTTAAGATA
GCTACCTTGCCCTGAGACCACTCTGGGTATGATGTATCAGCAACTTCATTTTCTTAGCCTCT
GCGGATAGGGTGTGTGACCAGCCCTCTCCTTTGCCACCTCGTCTTCCCACCAACAGTGT
GTGGTCAGAGAACGCGTTACAGATTAGTTGGCTAGAGATTCTTTTGGAGCAGACGTTTCTG
CTGTTGGGGATTTGAACCAGTTGCCAGTCCAACCCACTAACCACTTGATCACCCCATTGC
ATAGTGTGAAATGTTTAAGGATCCTTTTAATGGGTTTTCTTTCCTTTAGGGCGTTTTGACCGAG
AGGTGGACATCGGAATTCCAGACACTAGTGGTCGCTTGGAAATTCTTCGCATTCATACGAA
GAACATGAAGCTTGCAAATGACGTTGACTTGGAACAGGCAAGTACACACACACACACACA
TACCACGTTCTCCGCCCGCTCTTCCCCCCTATCCTCACCAGACGGCCTGGCCTCTGTAA
GCGCGCTCATCCATTCACCCTACCCTTAAAAGATGACAAGCAATGTATTCCCCGTGTCCTA
TATAGAGCCTTGCTTCCTCCTGTCCAGTCTTAGGCTTGTTTTCCACATCGGTTTTTCTCTATAC
CTTACTCTCTTTTCACAGCAATTATCTCAATCTCGCTCTCTCTTCATCTAGTGCTCCTGGCTC
CCGTTAACTCGTGTGAATTGCATATTTATCATCTTGTATTAAGCTTTTTGTTCATGTATTCGAGTA
GCGGTTTGTCTACCGATGTTTTTTTCCTAATAAAACGAATATAAACACACATACACACTTAACT
TCACTCTTCATATAACACCATTTAGGGATTATTGGTTGAAATGTCGCTTCAGTTTTAGCTGGGT
CTTCCAGTTGATAATTGTTTGGATTTCACATGTAACTGATTGCTGCCTTTCTCATTGTATTGTAC
CATGTTGTGGTTTGTCACCATGATCGCATTAATTGGTTTTCATGGCTGGATTTTAATGTACTTG
ACATAAGTCATAACTTACTTGGGAGTTTGTTAGTGGAATTTTAGTGCAATATGGCTTTGGAATA
TGTATCTCAGATTACTTTAAATGATCTTTTGGGTGATCTCATTATGACATGCTACTGTGTTAGATA
TTATTACCTCAAGCACAGCATCATGTTGATGGTCTGGTGTAGTATTACCTGGGTGTCCATTGA
CTTGGCGCTTCTTTTTACTGACCATGAGTTTTTGTTGTTGCAGATTGCTTCAGAGACTCATGG
TCACGTTGGTTCTGATCTTGCGGCTCTGTGCTCTGAGGCTGCCCTTCAGCAGATTCGTGAA
AAGATGGACGTCATTGATCTTGAAGATGAAGCGATTGACGCCGAGGTCCTCAGCTCCTTG
GCTGTGTCTCAGGAGAATTTCAGAGTAAGTAATTCCAAATGTGAACCATGATCTCTGCACTC
CACCTGCACTCTCGTGAGCACAGGTGGAATGTAGTACTCGGTTGTAGGTTCGAATCCCACT
CTCGTTGTCACATAGTGACCTTGAGCAAGTCCTTCACACCGCAGTTGCCTGTAGCGCTTC
GACATGTTAACTCGGACACTATTAATGCTGTAGTCGGGAGCGTCTATGAGTAGTAGTACACT
TAAAGAAGCGCTATAGAAAAATCCGGATTGAATAAATGAGTCATGAACCTGTTTTTGATAGAT
GGGCCAGCATGCGACAGTGAAGCATTTTATTTGCAGTGTCTGCAGTACAGTAGTCAAAATG
ATTTACTTATAAAAATCTCTTTTAAAGTAGCAGTTTATATATTAAAAATGGAAAACAGAAGTGTTT
CTATTAGACTTTGTAATAGTATGAGGAAGTATTATTATTCCTGTTTCTTTGGTTTCTAGCATAACA
ACCTGTTTGTGGTATAACAGCAGTACTTTTAGACCCATCACATCTGGCCACAAGTTACCAGT
CATATGCATTACTGAATATGGGTTTTATAGCATGTTACAAAATACTATGAACTAGGGATGTTAAA
TTAAAACCGGTTTTCTGTTTTAAAAACGATAACCAGTTCTACACCTTCAGCAAACGATGTCG
AATTTATCTTTTTGTCATTTTGCTCAGCAATAATTGCGTAATGGTGAGACTTGCCTTGTCTCTAC
AACACAATTTATAGCTGTTAATTCCCATGCTCACGATTGTTTATTAATCAACAGTTGGCTAATG
ATTATTAGCCAGGTCTTTTCAAAGATATATAGTATATACTGTGCCAAACACTGGTCTAAACATAT
CGTCTGATACATTTTAATAGATTGTACTGTTTTCATTTAAATGCTAGAAATCTGTTTCGGCATTTC
TTCTATCACAAGATGGATGTTTTTGCAGTGGGCACTCAGTAAGAGCAATCCAAGTGCTTTGC
GAGAGACTGCTGTGGAAGTACCCACAGTGACCTGGGAAGATGTTGGCGGTCTGGAAAAT

GTCAAACGGGAACTGCAGGAACTTGTGCAATACCCGGTAGAGCATCCAGACAAGTTCCT
GAAGTTTGGAATGACTCCGTCAAAGGGGGTGTTGTTCTACGGACCCCCAGGTTGCGGTAA
GACGCTCCTCGCCAAGGCCATTGCAAACGAGTGCCAGGCCAACTTCATCTCGATCAAAG
GCCCAGAGCTGCTGACTATGTGGTTTGGCGAGTCGGAAGCCAATGTCAGAGATATCTTTGA
TAAGGTAAGTCACTCAGCTTAGATGACTAGGATTGGGTTAGATGGCTAGGATTGGATTAGAT
GACTAGGATGACCCTCATTCTTTAGCCAGGATCAAGTCTGGCACTATGCAAACAGTAGCGA
AGCTCAGATGGCTAGGGCCTACCTGACCTACATTGCTAGGACCTACTTGACCTACACTTAA
TCATATATATGATTATCATTTATTTATCTAACAAAGCTATTGGCCCCCATGACCTACAGTTATGT
CCCCCCATGACCATCTAAACGACCATCTCTTAATCTGTGTCTGTTATTGGCCAATCCTAACC
CCGACAGACAGAAAGACAGGCTGTCCTTCTTAAAGCCAGGTGCGTGTTGAGTGCTGTGC
GAAAGCTAGCCAGAGTCTACATAGTTCGGAACATTACACTTTCCACTTCTGTCTGCTCATTC
TCGTGTTTCATTTTGATCTGAACTTGTAATTCATCTTTTCAATTAGCAACTCTGTTTTAAAAGAG
CAGACTTTCTAGTTATTGACCTCACTACGATTTGATATTAGATTACCATCAGTCGATGTATTAGA
CACCGACACTGTATAATTAATCATCCGGACAAACTTATGCTCACCGACTGTTTACTTTGGTTA
GATTAGCTTGTTCCATTTGTTACGTTCTGTCAGCAATGTTTTCTTCCAATAAAACGAAAATACA
ATACAATACTGTAGGGCTGTTAATCACAATGACTACATCTAGCTTGAACTGCACATGGTTGTA
ACAATGGATGTGGTGAATGGACTGTGCGCTCATTCTGTTCATTGTCTTCTGTAGGCGCGTTC
TGCAGCTCCGTGCGTGCTGTTTTTTGATGAGCTGGACTCGATTGCTAAGTCTCGTGGTGGG
AATGTTGGGGATGGTGGTGGTGCTGCCGACCGTGTCATAAACCAGCTGCTGACTGAGATG
GATGGTATGAGCTCAAAGAAGAATGTGTTCATCATTGGAGCCACGAACAGGTACCAGTTCA
ATTTACATCCGGAGTTGTATGCTTGAATCATTCATCCAAATTATAATAATATTAGTGGTAGTAGC
AATAAGATGGGTTACTTACTGCCGTAGCACAATTGTAGCAAAACGTCAAAGTAAAATATTATT
TTCAGTCAGAGGATAGTTTTCTGGATTGACTTTGTTAAAGTCTTTGATCATGTACCAGCCATTT
CAACCACACTCTTGTTCGGCAATGTATCTTTCAGTTCTCTGCAGTTTGCAGTTGTACCACATT
ATTTAAAGCAGTTGGTATTTTCAGTTTTTGTGTGAAATAGTACCAGCATGTGTTGTCACAATCAT
CACAATGTGTTGTTTTGTATCTTCAAGACCTGACATCATTGATTCGGCCATTCTGCGTCCTGG
CCGTCTTGACCAATTGATCTACATACCTCTCCCAGATGACAAGTCTCGTATTCAGATTCTTAA
GGCCAATCTTCGCAAGTCTCCCGTTGCTAAGGTAGACTTTGATCAATTTTTGTCTATCGCTA
GATAATTAATTTGTTGATTATAGACCATTCATTTACTGATGATTTGCATTTACAGAGTACATCAC
CAGTTATTTATTTATTCATTCAGGCTATTTCTATAGTGCCTTTTCTAGTCCACAATTACTCAGAG
GCGCTCCCAACACAGCACGGATACTGTGTCGGAGTTTCACGCTGAAGCGCCACAGGTAA
CAGCGAGCGAAGGACTTGCTCAAGGCCTGTTTGGCGGCTAGAGCGGGATTCGACCTTTC
GGACAAAAGGCGATGAATCTACAAATGAGCCACCACGCCCCACCATTTAGGTTGTGTCTT
AATAAAAATTCTCATGGTCACAACTTTCCTTGCTAGTATTATTGCTCTGAAGATGCTCTTCAGT
GGGTGAAATACAAGATGGTACTGGAGGCCTGAAAGTCTGTGAGAAGCGCTGGAACAGTAT
CTTCGCTTGTTGCGTCTTTGCATGGGCATTCTATTGCACGGCTACTTGGGATTGTGTTCAATT
AGTAGTACCTCGTTGCTTCTAGGTGAAGCTTCTTATGTTTAACAATGATCTGTGCTTGCTTGAT
GCATGCTAATCGAAATGGTTTGATTGTGCAGGATGTTGACCTTGATTATTTGGCAAAAGTGAC
ACACGGATTTAGTGGTGCCGACTTGACAGAAATCTGCCAGAGAGCATGCAAGCTGGCCAT
CCGTGAATCGATCGAGCTAGAGATTCGCAGGGAGAGGACTCGCGATCAGAACCCGGATG
CAGCAGCAGAGATGGTAGGTTCATTAATGGTCATGACATCATTTGTGACTGTCATAAGAACC
ATAAGTCATGTCAAGGCCTTTGATTGAAGAACCAAGATACTTTGTGTGAATTGTAAAATAGTC
AATATCCTTGAATCATTGTTCTACTATTGATGGCCTAATATGCTTAGCTGTATGAGTTGGCAATT
TAGGCATAGTTTAGCAATGTTGCAATGTGCACTCTTTTTCGGCACTATCATGGTTAGAGTAAA
TAATAAAAGGTTTTGTTATGTTAAATGCTTGGGTTGGTTGAAGCAGGTCACTGAACTGGATAA
GTAGTTCAACTTATTAAATCTCAAAGGGGTGGGAGTTATAAGGTGCATTTATCTAATTGCTCTG
GTGGTTGATGTCTGTTAAAAAAATTGACTTGTGTAAATTCTGGCTGGCAGTGTTGGACTCTGT

AGATCAGGATCTTTGTTTATTTGATTACATTCAGAAATATCACCGCTGTCTAGACACAAAATTG
TGATTTACAATAAATTCAAGGACAGAGAACAAGTACATAACAGCTAAGATGATTAAATAATTAA
GCAATTGAGAGGAAATTAAGCATATAAATGGCACTAAAGCAAAACGGCATAAAGTACATGA
GATACACAATTTTGAGAGTCTTTGCAAGGAAGAGCGACGTTGTGAGCACGGGGACGCAGA
TTACGTTCAGTAGGTTTGTGATTGATATTTGCTGAGGTATGTGTTGTTGTTGTACCAGGAGGAC
GACTATGATCCCGTGCCAGAGATCAGAAGGGACCATTTCGAGGAAGCAATGAAGTTTGCA
CGACGATCTGTGACCGACAATGACATTCGCAAGTACGAGATGTTTGCCCAGACCCTTCAG
ACAAGCAGAGGAATTGGATCAAACTTCAGGTTAGCCTATGTTTTAACCCTCACCTTCTATTGT
AGCAGAACAAACCTACAGGATCTAAGCCTTCTCATTTACAAATACCAGGTGGGCCAAAAAT
GGCTCTTGCCGCATTAAGTAGACGGGCACTCGACCTCAGGTGGTTTTGTGTAGTGCATATA
GTTTTTGTCGTATAACGATATAGGTGCATTAATTATATGGTAATAATCAATTAACTCTGTGTGAAT
TAAGTTCCATTTATACTCGCTCGACTGCATCATATTTGAAATTAAACATTAAACAATCACATCA
ACTAATACAGTAGCTCACTGCTCACTAATGTAGTGGGGCGATGCCTGTTTCTGTTTGGCCAA
AAAATGGCCCGCCTCAAATGAGTAGGCCCAGGCTTGCAAAGTCTACAGGATGTAGATGAG
ATATATGCTTGATTGAGCTCTGTCTAAATATTCTGCAACTACTGACTGCCTCACGATGCAGTC
TAATGACACGAGCCCAAGAAATTATTTATTTACTGGCTGTGCATAGAGAAATTCCAGACTCT
CAATTAGAGCTTGACCAATGTTACATTTAGCCGATTGTTCCGATGGCCGATTGTCCCGATTG
TGAGAACAGCCGATGTTGAGGCCGATGTTGAGTGTTACCGATGTCTACCCAATAAGCCAAA
TGTGGCGATTTGATTATGAACACAACTATATCAAAGTTGCGCAATTACGCCCACCGGTTACG
AAAATGGTAATAGGTACTATCAATTAGGCACAGGGTAATCAAATTATGTCTTCCTTTTATCATTT
TCCCTATTGAAAATTGCATACCGATGACAAGAAATGAATTGTCTATAGAGGCGGTGGGAAG
GAGGGCCAATAATGTTTTATTACCACATTCAATCCAGGCCTGGGACAATGATTGTTGGTCCA
ACAAGGTAATAGAAGAACAGCTTGATCAAATGACGGATACCCCCCTCAAAACTTGAACAGT
ACTGCATGGGTTGTTGGCCTTCCACCATCAGACAGATCCCTCCCTCAGCGCTTGAACAGT
CATCAAAGACATGTACGCGGAACCTTGAATAGTTCTGCACGGGTGGCCTACAGAGTCTAT
GGCTAGGTGAATTCAATGAGTAACACCATCGTGAAACATCGGCACGGACTGGTCACCGAT
TGTCATCATTTGCCGATGGTTCCGATTGTCCCACAATCGGCCCCGATGGTGAGTGTTGCC
GATGTATCGGTCAAGCTCTACTCTCGATGTGGGTCATTACCGGTGGGAGATTTATGGTATAG
GATGGACAGAATAGACAATTTTTGAGCTAATCCTGTGAAGTGTTAGTGACTTAATTTCTCTGAC
ATATGAAGGCGAACGACTCTCCTTTCCAGCCCGTCTCCTAACCGTCAGTATGCAGTCAGTA
TGCAGAACTCCTTATGGCTGAGTTTAGCTAGAAGAATGTGCTGGATTAGATCATAATTCATCA
CCAAGCCAGTTTTCCACTGACATTCCTGTATCCAGGTCATTCTGGGGACTTGTACATAGTG
CCCCTTCAAGAGACGTTCACTCAGAGGCAACCTTAATCCATAGAGCCACTGTGATTTATTA
AGGTACCCATACGAAGAGAGTCATCCTATGAGGCTACTCTATAGATGCAGTCATCTTCCATG
TCAGGCTGTTTTATCCTTTAGCGCTTAATGATCGGGGTGTATTGGATGCCACTGGGAAACCA
GTGCAGCATGTGGATAAAGTGTCCAGTTAAGGATGTGATTTCTTTGCATTTACACACAATGGT
TGGTTGCTTCTCTCTTATGCCAGTTCAAAAACAGTTGGCCCATCTGTTGCAGCAGCCTGTG
CTGTTAATTAATCCATCAGTCACTGTGATGTATTAAGGTATCTGAGTAATTATATTAATATTACCT
CTTTAAGTTCATAACTACATATTATCAATCTGCCTTTATACAATAATGATGAAAGTAATATTAACA
TTCAGTATTAATTCAAACTGACTAGTATGCAGTAATAGTACATAATGTAGTATCACTGACAGTAT
CAAATTTTTCTGTCGTCGTAGATTTCCAGGTGGCCAGCCCCCACGTGGTGGACAGGGTTC
TGGTGCTGGTGGGCAAGGAGGCGGCAGCGGTGGAAACCCATATGAAGAGGGCGAAGA
AGACCTGTACAGTTAGGCATTGAGCTGTGTGCTGCAGAGACGTTGGTTGGGCCATGCCAA
TTTTAGTGTTTAAACCTTGTCAATCGAATTGGTATAAGAGTGACTTTTGACTTTAATAGGTGACT
GTTCTCTGTTAAATTGTTAAATGCAAAAATTGAACATCTGTGCTACTAACCTACAAAAATGGTT
TTCTGTCAATAAAAAATCTATCCGCGTTCCTCATTTTGCGGCAATGTTTGTGACTGAGGACAT
GTCATAACAGGCAGACTTCATTTTTTTTTAAATTATACAGATTAATCCAGTATAATCTGAATCCA

AAGTTAGTGATTTTTATGTATCTGTAAATTTATTCTGTTTGTGTTGGTAAATGAAATTGCTTCAGTC
TCTTTGGTATTGAATTGTTATTGAGTGCCTCATTGTACCGAAACTCCACTCGGTACTTGCACT
CGGCCAAGAATCTTAATACTTCGACTCTGGCAAATAATGAGTCCAAGTATTACTCCAGAGTA
AATAATAAACAATAAAGGTGTTTTTGCCGAGTAATAACATACAAACTCTTGTTATGCGTTCGTT
ATTTTCTGGTCTTTATTGTATATTCTTCATCATAATTTAGGCTGTTCTTCTCCGCCACTAGCGGA
TCCAGACCTTAGGAAGGGGGAGCAAAAATTCTGAAGAAAAGTTTTTGAATCCTTCTATCCTC
CAAAATTGCTGATGACCTTTTTTAGTCATTCACCACTTTTCGATGATTTATCTCCTTGGCACAA
GGGGGGCCCAAACTCTCAATACAGAACCACTTTTACACCCAAATTTTCTTCACGATGGGG
AGCCAAACTGTATTGCCAAAACGGATGGGGGCCATGGCCGGATAAGCCTCCCTGGATCC
GCCACTGTTCTCCACTGATCGTGATTTATGCTGAGCTTGTCATAAGATGTTTAGAGGGTAGAA
GATGGGCTACTGATAGACAATAGGCTTTCGGCATTAAGATTGTAAAACGTGGGATGCATGTT
GGCCTATGGACGAGTTCTGTTCAGAAATTTGCCTATTGCTGCCATGTTAAACTCGATCGGTT
AAACGTCGTTTAACTGTGGAAATGCATGGGCAAAATTTTTCTAGATTAATGTGCAACCAGTCT
TGTTCTAAGTTTAGACTTAATCTGATGTAAACTGTCCTCTCACTCTTGCCGACCTTGGTGCAG
CACAGTAGGCCTAGCTTTAACGGTGTGATTTGTATGAACTCTGAACTTTAATTGGCAATTTTG
TGGTTAACCTCGTGTGGCCAGCCGAACATTCGGCGGGGGACTTATGGGCCGTACATAAA
CTATGTCAGGCTGGTGAGGGGGGTATGAGAAGGTGTGACAGTTTGCAACGTGGGAGGGT
CAAGAGCATGTAATGACACCCTTTTACATTCTTTGTCATACATATGACACCTGAAATTTAGAG
GGATGTTTAACTTTCTGTTGTAGCAGATGTATTCTGACAGAAGGGGGAGCTGACCAAAACC
ACCCCGGACAAAACTTTCCAGACAAAAGACCCCTGACGAAACCCCTTGGACAAAAACC
CCCACAAACAATTGAGAGAGAATTTGTACAATGGGTTTTTGTCCGAAGTTTTTGTACTAGGC
CTACTAAAATGGGAGGTCCGAGATGTGTGACGTACTTTTCGAGGGTTCCGAGATGTTTCAA
AATTGGCCAAAAATAGCGTGACGTACTTTATGGAAGGCCCATAGCCGCAATTTTTCAATGG
CCTGGCTGACGGCTAAGCCGCAGATATTTTCCGGCTGGCGGCTAATTCGACGGCCGGG
GTCTACTTGTGTGGAAAGGTTAAACCCAAAGGGAGAAATTAAATTCCAGCACTTGGAAACC
TGATTGCGGACCATTCGACGAGTGCGTAAAATTTCATAAACGGTTAATTTTCAAAAGGCCTG
CATAAAACAACGTCGCTTTACTAAATGGTAAGTAGGCTAATTTACAAGTTATATTGCATGTGTG
TAAGCTACTTATTGAATCGCCTGCGACGGACGCTGAATCCCTTGTCCCGACACTTGTGAAT
CGTCTATTGCTGGTCTAAGAACAGAAAGTGATGATACTAAACCTCTCTCGATGCGTTCTATTT
CTGGACATGGAAGCCTTGCATCAGTGCTTCTCAAACTTTCTTGTGGCGCGGACCGGTAAC
CTGGAATGCCGAAGCAGCGCGGCCCTTTGTAATTAAATTTTATTGTAAATTGGTTGAAGTCG
TTTACATATAATGTCTCCATAATTAGATGTATTGGTTTTATTTTCAGCCAGCCTCAGCCAGGAT
AGGCAATTTATTCAGTTAAAAATCCTAAAATGTAATTTTAAAAACAAAACGATGTTCATTTATTTA
CAATAATCCATACTTTAGCCAGCAAAAATTAATGTGAGCAATGTGCTTGGTTTTGGTTAACAA
GATCATCAAACCTTGGCTCTGCCGTAGAAAGTGCAACTGGAAAGGAGAACAAGATGAAGG
AATATCAAGATTGCCCATGTACTTTGTTCTTCTGATTCTAAAATAGCTCTGGTCATGCATTTTTA
GGAACTGGGAATGTTTCTTGCTAAAAGTTAATCATTGGTCCTAGCTTTGTCACGGTGATTTAC
GTAGTTTTTAATTTATTATTAAAGCATTTAGGCCTACCTTTAGGATCCTTAGGTTTTTAAGCGAT
AAATAAATTTGATATTATTGATTTTGACGTCCATCTGTTTTACGGTTTGGCATTTACAATTTTTAC
ATGTGTATCATTATCGTTTGTGACGTTTGAAATGAATGAGAATACCAATAATACCTGAAAGAGA
CGAGCGTAATTTCATAAACAGTTTTGTTCGCCTGCTAACGCAAGTCATGAGTCAGTCAATCA
ATAATCGTTTAATAGGCTTAGAAGAAATTCGTAGCTGACAGGAGACGCAACTAAGGCCTAC
AAAATGAATCAATAACACAGATAGTTCAATGCAAGAAGAAACTGTGTCCACCAGACTGCCT
AGTTGCCTACATATCTTTGTAAAGAAGACGTGGAATAAAATTTCGTTCATCACGTGACAAAAA
ACGTCACGTGACTAAAACCCGGTCACGTGACTAAAAATCAATCATGTGCTCAAACAGCGG
TCACGTGATCAAAACCCGGTCACGTGACTGAAACTGAGTCACGTGATCAAAAAGGGTCAC
GTGATCAAAACCCGGTCACGTGACTAAAACTGAGTCACGTGATCAAAAAGCGGTCACGTG

ACCAATAGGTAGTCACGTGAGCAAAACCCGGTCACGTGATCAATAGGTGGTCACGTGACC
GGTAGGCAGTCACGTGATCAAAACCCGGTCACGTGACAAAAACTGAGTCACGTGATCAAA
CAGCGGTCTCGTGATCAATAGGTAGTCACGTGACCGGTTTCTGGTCACGTGATCGAAAGC
CGGTCACGTGACCAGTAGGTAGTCACGTGATCGAAACCCGGTCACGTGACTAAAATTCAG
TCACGTGATCAAAAAGCGGTCACGTGGTCAAAAACCGGTCACGTGACTAAAACTGAGTCA
CGTGATCAAAAAGCGGTCACGTGACCAGTAGGTAGTCACGTGAGCAAAACCCGGTCACG
TGATCAATAGGTGGTCACGTGACCGGTTTCTGGTCACGTGATCAAAAACCGGTCACGTGA
CCAGTAGGCAGTCACGTGATCAAAACCCGGTCACGTGACAAAAACTGAGTCACGTGATCA
AAAAGCGGTCACGTGACCAGAAACCCGGTCACGTGACTAAAACTGAGTCACGTGATCCA
AAAGCGGTCACGTGACTAGTAGGTAGTCACGTGATCAAAACCCGATCACGTTACTAAAATT
CAGTCACGTGATTAAAACTCAATCACGTGATCAAAAACGGTCATGTGATCAAAATCTGGTCA
CGTGATCAAAAACCGGTCACGTGACCAGTAGATAGTCACGTGATCAAAATCTAAAACTCAA
TCACGTGATCAAAAACGGTCAGGTGATTAAAAACCACTCACGTGATCAAAAAGCGGTCAC
GTGACAAAAAACGGTCACGTGACTAAAACCTGTTCACGTGACTAAAACCCGGTCACGTGA
TCAAAAACCGGTCACGTGATCAAAAATCAGTCACGTGACTAATATTCAGTCACGTGACTAAA
ACTCAATCACGTGATCAAAGAGCGGTCACGTGATCAAAACCCGGTCACGTGATCAAAAAA
CGGTCACGTGATCAAAAAACGGTCACGTGAGTAAAAACCAGTCACGTGATCAAAAAGCG
GTCACGTGATCAATAAATGGTCATGTGACCAGAAACCGTTCACGTGACTAATACTCAGTCAC
GTGATCAAAAACCGGTCACGTGACCAGTAGGTTGTCACGTGATCAAAATCTAAAACTCAAT
CACGTGATCAAAAACGGTCACGTGATTAAAAACCACTCACGTGATTAAAAAGCGGTCACGT
GACAAAAAACGATCACGTGACTAAAACCCGGTTACGTGATCAAAAACCGGTCACGTGATC
ATTATTAGGTAGTCACGTGATCAAAACCCGGTCACGTGACTAAAATTCAGTCAAGTGACTGA
AACTCAGTCACGTGATCAAAACCCGATCACGTGACTAAAATACCGTCACGCGACTAAAAC
TCAGTCACGTGACTGAGTTTTAGTCACGTGATCAACAACCGGTCACGTGATCAAAAACAGA
TAACGTGACTAAAAGTTGGTCAAGTGATCAAAACCGGTCACGTGACTAAAATTCAGTGGCC
GGTTTTTGATCACGTGACTAAAACTCAGTCACGTGATCATGCAAAAAACCGGTAACGTGAC
TAAAACCTGGGCTTTTTTAACGTAAAAGCGCTGCCGTTTGTCAGATGTTTCTACTCAATAAAG
AAGTATTGATTATCAGGATAGCTGTAATTCTTTGTCCATAAAATATGCCCTCAACAAATTACAA
AGGCCTACTCAGGATCTCTACATTGTCTGCATTACGGATCCCCCCGTGTTCATTCGAACTC
CAATGGAATAAGAACGTTATCCTGTTTAATCTTGCAGAGCTAACTATTAAATTACGCCAAAGG
GTTGGAGTATACTGTTATGATATTGTCGCGCCCTGGAAATGGATTATTTTTAAAATCATTGTTTA
AAGCGCATGCTGTGCCTACTAAAGTAGATAAGATAAAGGCGTTATCTGCATCAACAATGATT
GCTTGATTGAACTGTGTGCCAAATTCGCAAGTGGACTGGAATTCTAGTTATAAAATGTAAGAT
AGGCACAGACGGCTATGTGGGTAGGCCTGCTGTAGCTTAAACCCATCGCCTTATATATCGA
TTAGTGTTTTGATTAAGCTCCTGTGAATAAAATCTCTGTGTAACCCCCGACTGCGCCTTTTTG
GGAGCTGCAGATACTTGGATTAAAAGGTGGGCAATGCCACTAAAGAGAAAGTTAATGAGC
AAAAATTATGCTCAAAAATCTAGAAAGTGACCTATCAGTAGGGCTGTCAGTGCCCCTACTTA
GAAACAAAATTGTGGGATTCAACCAGCTTAGAGGTCTTATTCAGCAGGGGCGTCCATTCC
CCAATGTCACTTACCACAAAACAGGAATTCCCATGTTTTCGCGCAATCCCCTTATTTTCACA
AAAATGACGTTTTCCGTGTATTTCTCTTCTCCCTACTTTTACCATGATACATTTATGCATCACA
GTTTACACGTACTGGATGCCCTGTGCTTCAGTGGACGCTGTAGAAATCGCGAATTTACGATT
ACGATAATTTATGATTGAGATGTATTCTCGTCAAATACTGCATGCATTGTCAGCCGTTTTGCGT
TCAAGCTTCATCTGTTCACTGTTCAGTTAAACGCCGTAGTGCTAAATCACTGCATCTGACTTG
TACTGTCATTGTCAGCTACAAGTTATGACACAAAGCCATGTGATAAGTGCTAGTGGTTTGCC
GTGGGATATGGTAGGAAAATGACGATTACATTAAAATTTCTGAATTTCTAGATTTGAAAATCTA
CCGAAAAATACCCGAATAGAAGCCCCCGACAAACTGGGTCCCAAATACAAAGGCCGGG
TCTCCAATATACGCCTGGGACCCAACTTAATTGTGCTAATAGATGTCTTTGATCACAATTTGT

GCAAGTCCGCGTGAGATCTGCAAAATTCGTTTAACTCCCCGGGCGTAAGCCAGGTAAAAT
ACTAGTATAATATAGATGTCACCACCCTCGTTCCTGATGGTCAACTTTACAATTGTCCTTTCC
CTTGATAGGCTGCCACCAATGAGTTTTCCCCTAGACTCATAAACTATCTCAGCACTTGTACC
TTAATCTACACAAAATACTCTACTCTGTAGGTATTGTAGAAACTACTAGAGAATACGCAAAAC
GATTTATCAGTTTTCTACGTATTATATTATCAGAGAACACAGCAATTTCACAAATCAACTCTTAA
CATCGAGCATACAGTCACTTCAACAAAACGCGCGCTGCCAAACTAATCGATAATGCACCT
GTGCCAAGAGCGCGAAAACCGCTCGAAGGCCTTAACAATCTAATCAGCCGATCGCCTAA
ATATCCAAGCACGCCCGCTCAACGTGCACACACAACACCCTGAAGGTCTAGAGGCCTTC
AAATGTGTGTATAACTAAAACATACGCTCACAATAGATCCATGGTTTATTACTTTATTTCAAAG
CGAAAACTGAAATTCACTCTTCTACCGAAATAATGAAGTCAGATTACATAGCATTAAGAAAAT
TTCTGGTGGCCCAGTAAGTAAGTAACCAGAGCAAGTAATTGAAAATTTCAAAAGGACAGCC
TAGGGCCTATCCACAAAAATGTTCTTGATGAGAATTAATTTTATGCCAATTGACAAATCAACA
ACTGCACATTTTACAGCTGCACAAATTACCTTTTTCAACTGCACATTTCGATTTTACAACTGC
ACAAATTGTGATCAACTGCACGTTAAAATATGCCCTGTTGCTGTGCTGTTACCCACCCGGG
CCGTAAAATTGCGAACGGGTACCCGTGGAAAGGCGGGGGTAACGTCTTTACATCAGTTG
GCGCGCTGCGCAGCTGAGCACGCAGAGCTGTAGTTCAAGAAAAACTCGGAGGAAAAGC
ATGGAAAACGAAGCGAGAGATGAGAGACAACCCGGACGAGACATAAGCCATACTGCTCC
GCGGTCGATCACGGACGACTCGATTTATCGTAGGCCTATTTTCCCCTCCGATCGCACTCG
CATGATCGTATCCCATGTGAAATAGTAGATCGCATGGTTGACAGTTTCTCAGCCAGCTTGAC
ACATAATTATTGAGGCTTAATTTCGGTTTAGGCTCAATTCTTATGGCCAGCTGTCAGCTTCGG
CTTAGGTTATTTATTTATTTATTTATTTATTTGTATTTATAGAATATTTTATGACTCTAAATGTCATTAT
GAACGAATGTGTTCATTCGTTTAGCAAACCAATGAAAGATTCGCCAGTGGTGGGCACACTC
TCAGGCGTGGAAACTTCAAGATTCCAGGCGGCAGCTGAGAGATGACGTAACTGTTCAGAT
AGTAGTGCAGTTTCAGTTTGTAGGCCTATACACGTTATCTTAATCTAATTCTAATATTGAAACAT
CGAAAGGAATGTTTTGACAATGCAAACGTTGCTTAGACAAGCCTTCTTGTCAGCTTTGTCTA
ATAAACGAACGTTTGGGTAGATCAGTGGTAAGTTCGGTGCCTTGCGTCCTGAGGCTGACA
GGTCAAATCCCACTCTTGCCTGTGAGTGAAGGACCTTGGGCAAGTCCTTCACTCACAGTT
GCCTGTAGCGCTTCGGCGTGTTAATTAAGACACATTATCAATGATGTAGTGGACTTGAAGAG
GCGCTATAGAAACATCCGGAATAAATGAATGAATATAAATGATTGTGCCAGTGAGATCTTATT
TCTCAAAGTAATCAACTTCTCATTACGATAGCCTATAATCCCATATACCTGGTATCACACTAA
GTCATTATAATTATTCACGAGCACGTGACCTTACTCGTCGCAAAATTCGCAAATGCATGTAG
ACAGTAGGCCTATTAGGCTGTCCAAGTTATCACGGAGAAAGCTCATCTTGTTCAACTTTAAC
ATTTGATCCTCTGCTGCAACAAATAAAACAAGTATAGGCTACTGTATGCCTTATCGAAACAGT
GGATAAGCTTGACACTTTGCTGATATGAAACTGCCAATAGCACAGACTGCTGTAATATCTCT
GTAGCCTACGTCTTCTTTCTAAGTACCGGTATGCCAGTTCGAGTGTCATCATCAACAAAATG
CTTGGAAGGATCTCGAGTACGTTTTACACATACACGCAAAGAATACAAAACAGATCCTACG
TGGTACGCCCGTCGCGAATAGTTTGCAGTTGCCGAAAACTTTACAGTAATAGATGTGTCAGT
GTCTCAGTCGAACCTTTCTGGGTACTAATCGGTAGCTTAGCCGCAATTTTTCCTGCCAGCT
CGCGACCAAGCCGCAGTCTTTGCGACCGGTTATCCGGCTTACCACAACTTGCAGCTGAC
AGCCGCTTTAGCCTAAGTAGGCGGCTTATGGGACTAGTTAGGTAGCTTTTGCGTAGAGATC
GCCACGGAGAAGATATATGACAATTATCAAACGGATACCCGGGAACCCGTTAAAAATGTG
GAGTACCCGAGTAGCTCCTTAAACAAAATTGGCACCCCTACCTGTCAGCCAAATTTCAACT
GATCATTCTGCTGCTCCAAGAAATGGGTTCAAATACAGTAGTCCTATCGTCAATACTGCGTT
CATGTCAACGTGAATAAGCCAATTCGACATGTCGTGTGCGCAGGCGCACTGGGTCGAGTC
AAACCGATTCCTAGAGTTGTTCCTCTAAACGAATTAGGCCTACAAACACTCAGCTCGCTATT
GAATATTTAGGATGTTTCATCGGTGCATCAACAGCTGTGGGGAACACGGTCCTTCACTCGC
AGTTGCCTGTGGCGCTTCGGGGTGAAATTCCGGCACAGTATCCGTGCTGTGTTGGGAGC

GCCTCTGAGTAGCAGTGGACTTGAAGAGACACTATAGAAATAGTCTGAATGAACACTATGA
GCTAGCATTACATGTCTGAAGAAACTAAAAGTTCTGTTGCTTGTTTTTCTCCGATAACACTTTA
TAAACCCACAATAGCCAGTGACCCGACTCACTGCGCATGTATACACGACATGTCGATTCG
ATCAATCCAGGTCGAAATAACCTTGTATTTAGGTCAGACAGATCCCAGAAGCTGAGATGTC
GACTCGGCTTATAGGCCAGGCACACGTTTCCGCTAACAAACACCAGGTAGGTTAACATCA
CATACACTGAGCCCTTGATTTGGAATGCATTATTTCTGCAGGCTCGTGATTTTCATTAATTTTG
TTTAATAGAATTGTATGCTTTGACAAATCACGGCAACGATTTTATTTGTAGGCCTATGCAGGA
CATGTACACAAGCCTGAGATAAAATTGTAATTCTATTTGCACTCGAATAGGTTCGATTTAGTTC
CTTATCATAAAGTCTTTTTCTGTAGCTTAAAGTGCAAAATGTTTAAAAGAGCAAAATGGGTAAA
TGTTGGAAAAGGAATTGTAGAAGGCGTGCACTGTTGAAATGATTATGGAAGGGATTATGGAA
GGGATTATGGAAGAGATTACGGAACGGATTGTGGAAGGGATTATGGGAAAGATTGTGAATG
GGATTGTGGAAGGAATAATTCTGCAGTTGTGTAAGAGTATAGGCCCCAGGGCTGGTAAAAA
CCTAAGGTTTTTTAAAAAAGTTTTTAAGTTTTTAGGTTTTTTAGGTTTTTAGGTTTTAATGTCTACG
CACAGTCGCACGCCGAACATTGGACACAAGAATACGATCAACTAAAAAGCTATACACGTA
GACTAACTAATGCATTTCTATGTTGCACGTTATAAATCCAATGAATTCATAGAAATTTACAGAG
TTTGACATGAAAAAGTAAAAAATCTGACTTGAAAATCAAAAAACCTAAAAAACCTAAAAACCT
AACTTTTCAGGTTTTTTAGGTTTTTTAAAAAACCTAAAAAACCTAGGTTTTTTTAAATGGGTCTC
GACAGCCCTGATAGGCCCCTATAATTGGACGATATCATCATTCATCATGGCGTCTCCCTGG
GTCCAATTGGTTCCTTTGACCCCGAGTTCATTCAGTCCAGTCTCGTTGAACGACCCTCAAA
GTCCTTCTATACATGTTCTTTGGTATTCTCTCGATATCGCGTTGATTCTGATGATTAGTCGCCA
AGACGACTGGGTCGACCACGACTTTCGTTATCTAAGAAATTATACGAAGTTTTATATTTTCAA
ACTGTTATATTGTACTTTATGTAAAATAGGCTATTATTGTCTCTTACTTCTAAACTTCCAGGAAAG
ATGTTTTTCAGCCGACTATATAGCCTGGCCATTACCCGGTTTTAGCGTATACCTGGATATAAC
CTAACTGCACCTGCATTATAGCGTAGGCCTACTTACTGGCTTTAGCGCAGGTGAATAAGCA
ATTAGGCTCTCTAAGATTCCGCTTGTCTTTGAGAGCTGAGAATCTCTGAGATTCATATTTGAAT
CATTTAGGAAACATTTATTTTCATGCTATTCGACACGACCGTCCTTTGATAATGCCCTTTACAA
TATTCTTTTCCGTTATGGGCTCTTCCCTTTGGAATCGCCGTCCACTTTCAGCTCGTGTTTCTT
TCTTATCATCCAATCTGTCTACGTCATTATCGCTTCTTAAAACATGTTTCTTTTCTTGGAGCTAA
TCGAACCAAAAGCATTGTATTGTATTGTATCTTCGTTGGCCCACGGGTATTAAGGGGCGCTA
TATAAATACTCGAGTACAATACAATTCCGCCTTACGTTGTTCAGAAGGGCACATCCCAGCC
AGACAAATGTTGAAAACAATAAGTAGATTTATGTATATAGACCTATTATAAGTAGGATTCTGAAA
GCTTTTAATCACTAAAAGCGGTGATCATCGTTTAGCTTTCGTAGCAGAATGTTACAGATCAAT
CTGTTTAATTGAGGAGCCTTGCATTTGAATGTTATGCTATGTTATGTCAATGGGACTCTCAATT
GCAGCAGATTGCCTGAACATAATATTATCGTATCGTATAATACCTCAAGCGATGCTGCGTCC
TTCTGTTTTCACTCCGACGCCTACGGACGGCTTTCGGCATCGCTGAAGATGACCGTGTCG
TCCGTGCATTTCTCGGGTTCCAGGATCGGACCTTTTTCCCGCACTGCCACTCCTCCGTCT
CCACAGACTGACTGAGGCCTACACAGGACACCGTCGAACATTTCACACCTGCTCCTAAT
GGCGAGAAAAGACAATGAATCACAATCATAAATCTCCGAAATATCAGTCATACGTTAATGGA
AGGATGGTCGAGCAGAAAGGTTGCGAGTATGGATGACGTTGGATGTGTGGACTTGCACCT
AAATGGATGACTGGATCCTTTTTAGGGCCTACTCTTTAGATGATACGTCGTATCTTTTCTTTGG
CCGCCTCTCGTAGCGCACACGAATAAGCTAGTGCCTTGGGATTAGCTTTTGCGTATAGCG
CTTGAGTATACAGTGAACGAGTATCGCGATTATGACTAGGCCCATGATTAGGCCCATGACTA
GGCCCATGATTATGATTAGGGCCCACTATTACCACACGATTTTCCATTGCATTGAACTACAA
ACGAATATATCACGAATCTGTTGAAATAAGGACGACAAGCGAAGTCTTTGCATCTCAATGTC
TTTCGATTTTTCTACTTGTCTCAGGTTTGAAATAACTGGGCGATAAGCTAATATATTATAGGAAT
GCTCACGATATTTTTCACTTACATTTGTGATAGTCTTGACCACTGGTTTGTGACCAAGTCTAAA
TATCCGGTCACTAAGTCCACGCACGACTCTTTATCCCCGCCCCATTCCACGTTCCAGTCG

AGGGAACCGGTCTGCAGAGTACTCACAGAGTAGGCTACGCAGACCCTGAACAGATTCTC
TAAACGATGTTCACTAGCCTGGTATCACCATTGACCGCTTCCATGACAACAAGAACTTCGC
TTGAAAACGAAAAGACATGTTCTTCTGACCTGAAACTAAATCTGTTGACGTGTCCTGTGACG
AAGTCTACGCATGACCTGTTTAGCTATTTGACTCCACCCCAGTATAAATCGAGCGTCAGGA
ATCAGTTCTGTCACGAAGGCTGACCATCTTACTTACTTCACAGATCCGAGAAGTTGTTGGC
GGTGATCGAGAACTGTTCTATTAAAGTCTTATAATTTTCAGATGGTCTTCGTCGGAACTGATC
GCCGTTTTCCGCTGCCACGTTTCTGGAAGATCTGTAGACTTCTCCGTGCACGTGCTGAACT
TCTCATAAGGTATCCAGGGTAAGGGATCATGCAGCAACAGCATAGGTTTGCGATGTCTGCC
TCTTCTGGCTGTGAGCTACGCGGTTGGTCACGTGGTTGTTCACGCTCTGGGATGTAACAAA
CAGACTCTTCCACAGCCCAGGATATTTTTTGGCCTGTCCTCGAAACTGGTTCGTCCGAAG
CCTCTCCTTCTGAGCCAATCTCGGCACTTGTCTTGCTTGTCTCACCTACATGAGCTCTGTTA
GCAGCAGCCACTTCGTTGGGCAGAACTAGACCTTTCGGTATCGTCGTCTCGGAGGATTCA
TCGAAAGCATCGATCTTGTGAGGGTCGAAATGGATTAAGTCAGTGCGTGCAACTGACGGC
TTTAGCGAGGTTCTTAGAATAGAAGACAATTGTCGAGAGTCATTGGCTTCGTGTACTTCTGGA
ACATTGGCATCAATCGGGACATTAGTGGCAGACGACTGAGGCGGTAAACTGTCCGGAGC
TTTATCTTCCACCGCGGCTGGAACAACTCCTCGACCCCGTCTCCAAAACAGGCCCGTGC
ATCCGCTACCTCGTGCTTCATCCTTGTCGCCACCCTTATTGTTATCAACACCCTCCTCCCC
CACAGCTGTTGATGCAGCTGCATCGCCGGAGGGTCGCCTTAGCGCACGCCTCAGATTG
GGAAACATATTTGATACACATTGAAAGTCTGAGTCTAGCGGAATTATCGACTGGTAGAACGA
CAATCCAACAGACGAACGCTAAATCTCCTTTGCATATATATGTTGCAGAAAAGCTAAGCAAG
GCGGTTGAAGAACTATCAAAACACTATTGTCAATGCTTCCGAAAAATGAAAGCCAAAGATA
GAATGAGCGATAGTCCAATTACCATGGCAATATTACGTAATCAACAAATGGTCGTTGGTATTC
CTACCTGAACAGTGAACAAGTCTGTAAACGTTGTTATTAATCGAGAGAGAGTCTATTCGTTTT
ATTGAACTTACGTTCTGTTGACAAACCGCAACTATAGACTCACAAATGTTGAAAAATGTAGAA
CAATGGATAGGACTATGCACATACTGATTAAATAAGAGAAAGAGTCATGAGAACGAGAGTTA
ACTAATAGGTACAATCGGTTTTATGGGCTAAAGCTCTTTTCAGCACTCTAGGAATAAAGTTTTT
ATCGTTTTCTGGAAGAAGTTTAAAGGGGCGCAGCCGTTTTCGAAGCCCTGGGCAGCGATC
AAAAGACCTACATGATATTTCTAATTAGTTAGTGTAATCACTATAATATATCGTCATCTGAGAAG
TATCTTGCCGTTTCTCCTATTGTGTTTAGAAAACGTTTTTGATTAAGTTACCTTAATTTGTCAGA
AACATTATTGTCTGTCTTCAGTTTCAATGTCTACTCTTGCATAACCAAACGTTTTCCGTTTTTTT
TAAACTTCCAGTGCTGCATTATCTAGTCTGCCTTGGAGCTAACATAACTAATTTTATCTAGTGT
TGCAGAGGTTGTAGGCCTATTTAGTCTTGAAAATCAGAATCTTTGGTTTTGTACTATATAGCAG
TAACCGCCTATAGAAGCTTCATAGAAATGATGGGTTGTTAGTCAGAGTTGGATCAAGATTAAC
CGACTATAAATATGAGCCTTCTCCATCATGTAGTGGCTTTCGAAAGGCCACTTTTACGTTTAC
TTGACGAGTTAATATAGGCAGGCCCTATTTTGGGCCAGCCTTTGCTCATAATCGCTTTTCAG
CATCGTGTCGGAATTGAGCTAGTGGACAGCTTGAAGGGGTGGTCGTGGGTGACTTCCCAG
TTCCATGCGGCATTGGGCGACTACATGTAGATCACCTTGCGTTTGATTTCCGGATGTCTCTT
GACTATCTCCAAGGTCACTTTACGCCAGAGATTGCAATCGGATTCATATCCTGTGATTGGGC
GGCACGGTCAGCGTCCTGGTATTGTTCTGGCACTTCCAGTTGGTGACCAGTTTGGCACGA
TGACATGGCTCACTGTAATTCTTCATTTAGATATGCTAATAGCACCACGCACTTCAGCAGTAT
ACCGATGCATTTGGTCACATTGACAGCTCCATCGACAATGTCCAACTTCAGTATAAGCCAG
GCAGCACCAGATCATGATCTTCAGAAGATGTTTCACTGTCATATTCAAGCACTCCGGCTTG
AATTCTTCTAGAAGAAACCTCCTCGCATACATGTGGACCTAGTTGCAAATAGGCAGGAAGT
GCTTTCATCGCTGACGATCATCTTCTCCAACATCTTACAATTCCAGCTGGTGTACCGCCTCT
GGTTGGTCAAAGATAATTATACAGCGATGTGTACGGTTCTGACGATGTTCATATTGTTGTACC
GATTAATGTGAAATTAAAAGTCATCTCTCTTAAGGTAATGATTTTCAAGGCTAAAATTTCATAGA
TTGTTATACTATATTAGTGCAACGAAAACAATTATAGAAATGGACTATAGTCCATTTCTATAATTG

TTATAGATGCTGCACCAGATATTATGATAATAATTCATCTTTCCAGCATTAAGCCAAATATTTTA
AAAAAATGCGCTGAGAAGAGGCAATAGCACAGTTCACATTAACAAAACATCACATTGTTGG
CACCACGCAAGGTAACTTGGGTACACATGTAGGCCTATGTAGGATTACAAATTATTTAAGAA
ACCAAAAAAGCGATCAGAGAAGAACATCTTATGTTATAATACCCAATACTACATGTAGACCTA
TTTGTCTACACTTACGTAGATGGTTTTTTCGTGTGAATAAGTACGCGTTTGTAGAACATTAAGT
GCTTTTCAACGCGAGCATGTTCACTCTTCACTGTTATAGCATTGGAGTCTTTTCTATCGGAGT
AAAACTCCATTGCTAAAATAGTGCTTTTCTTGTTTTACCTGTAGGGTTCTTAGCATAATATGAA
CCCAAATATCTGTAGTATTGGACAGGCCACCAGAGGGTCCGCGAAAAATATGTAACAGCA
AAAGGGGTCCGCATCCCAAAAGGTTTTGGAACCGCTGGATTCTATGGGGGTTACACCTAC
AGAAAACTATGTTGGAGTTTGCTACGTGTAGGTTTTCTATTGGCAACGCTTGATCATCCTCCG
TAGCCGACAGGATCTGCAGACCTGCTTCGCCCCCTTAGCCTCACTAAATAAGAACTTCAC
TGATTCTGTTCGAGGAGAATCTCCTGCCCATGCATTTTGTTATTACGCTTATTCCAGAATATTC
CTCGGCTTGTATCCTGATTGAGGACTGGTTTTCCTCCTATGCCTGCAGGGTAATTTAGGATC
TGCAGCTCTTCTTTAGACAACTATTAGGTCATATCACAGCATGTAACGTAATGCATGTAATGTA
GATCTCCGAAACTTCTCAGCGCCCACAAAAGCCACGTCAAGAAGACCAGCTTTTCACCG
GCGCTATGCCAAATCAAGTCCGCAAAGCAAAACGTCTCGTTTCGTTTCGGAACCAGGTCA
AAATCTGGAAAAGGTCAAGCCGAGGAGACAGCGTGGGCTAAGGTTGCAATCGGTGGTGA
TGGCGGACGTGTCGTGGCGCAGGAGCAGAATCGGGGTGTGATAGCTTGAAGAGTTCAGT
CGTGCGTGGAATGGCGTGGCATATATTTGTCCTCATTGCAGAAATCAGAGTTGAGAAAACC
AATTAAAATTCTCAGTTGTCATTAAACATTCACTGATAAACATAGCGCTGTGACAACTGTGAC
AATGTGTCTGCCAACAGGGAAGCTAGACCGATGAAACTTAACTCGTAATTTTGTACTATTGG
GATGCCAACCGCTAGCGAAACATAAATCGTAAAATAGTTTTCAGCGCTCCACGTTCGTGAA
TGATGATCACGTGGTCCTTCACCTTATGATCTTCGCATCGACAGTCAAACTAGCCCTTTAAG
TAGGCCTAGGCCCACTAGTTTCACAGCGGATATTCGGTGACTAACACGAATCGTAAAATAT
CTGTGAAAAACAGCAGAGATTTGATCCTGATATGTTCTTCGTATAATACCAAAAGAGACGAC
ACAGTGTCACAAAATAGATCTAGTAGACTAAATGAAAAGCCTGTTTCATAGATTGTAAATCAA
AACACACCGATTTTAATCCCCATGAATACATGCATGACTTCATATTCTCTAGATGGAGAGTGG
AGACAATGTGTGACAGATTTTTAGATTTTCCCAGCCCACATAGGCCTACCTTCGCGAACTCT
TCACCAGCCAGCCAACCCGCTCTACTCGATCATCATCCTGTCTCACCCTTTCTCGACCCC
CCGGTCACTTCTCATCTCATGTTTTCCAACCGAGCCATATCAATCACTGCACCACGTCTTTG
GAATGACCTACCACCTGAACTCAGCACCATTTCTTCGCCTCCACCGCCGTCATTGCCAAT
CACAAGACATCATCTTCATCCAAAATTTCCGCTCAGGCCTAGGGCCTACTTCCAACGAAAT
TACTGGAAGCACTAAAAATTTCACGATCTGACGTAGGATATGCAACTTATAAGCTTATAGCCT
ACTTTAGTTCAAAAACATTTCGCCAACAATGCAGGCAGCTTAATTATGGTGCAACTCAAAAT
TAGCATGGGTTGATGTAGGCCTACATTTCAAAGATCAGGCAATAGGCCTAATCGTAATCGTA
AACTAGAGATTTCTACATCGCCCATAATGGACTGCAGGCTTAGCCTAGCGTCACCCTAGC
GTCATATATAGGTTAGTTTAGTCGCATTCACTGCTATCGCTCATCACCTTTAGCTTTGCAGCT
GACCAATGACCATGTTGGGCATTCAGCACTTCCGAAGAGTGCTTTTAACTGATCAGAACGA
ATGCCATTAAATAAAAAGTTTGAGACGTTGAGGCGGTTCAGAAGACTAATGACGGAATAAAT
GAATTTATTAATTTATTTAATTATAAAGTATTCACTCACTCAGCCTGCATGCTTAATGAGTTTCAG
TCGTGCTCACCTCACAAACAACAACAAGACTAAGTTTTCGTTTAAGGAGCAGCACAGGTAA
TGAACCTAAAAATATTGCCTAACTTTCACATTTACTGTTGTTATTAATGTGTATTATGTAATGTCA
TTGAAATTTGTTATAGCATCCCTTGAAGGTATACCTACTCAGAGGCGCTCTTTGCGCAAGCC
TTTAAATGATGACTTAGGTCTACGATTACTATAAGACGACTCTTAGAACGGCAGATGGAATAC
ATTCAAAAACAGGGACATTTTAACATTTATGATTGTCTTCTTTCAAGATGTAGTAGGTAGCCTA
CTAGGTAAGGCCTATACAGGCCTGCAAGGAATAATCTGATGATGTTGTCGCTCAACGCCTT
ATTTAACACGCAAATCGGACTGGCGACAACGATAACGGTTAAAACTGAGTAATAAGCTGTC

ACATAAAGGTTGACGTCAACCTCTGAAATTTAGGAAAGCGACTGCAATCTTACAGGAAATAA
CACGGCAATTCAAATAATGAATTTTATGTCCCAACGTTTATAGTTTATACAAAGAACGTTGATT
CCCAGTAACACAAGTACCGCACTGATTATTATTTTTTTATAATCGACGTTAATATCCGAAAATT
CCCTAACAGATGCTTCTACATGCTGAGCTCACTAAGACTCAACCAGGAGCTTAAGGGGAT
GCCAACCTGCTAAACCAGAGTTGGGGTTAACTCCATGCTACACACTACCAAATCAGGAGC
TTTTAGGGGTTTAATCCATACCACACACCCGACTACCAAACCGGGAGCTTTTAGCCTTAGG
GGCTTAATATATACCACACACCCGACTTACAAATTAAGGAGCTTGTGGGGTTCTACTTCACA
CTAAACGCCCACGTCTGTCAAGCCAGGAGTTCACGCAGATTTAGGCCACACCTCATGTTC
CATGGGTCAGCTGATTCGTAATCAATTACTGACGCAGGCATACACACACGCACGCACGCA
CACACACACTCACACCTACACAGACACCCACATACACTCAAATATACACACACGCACACA
TATTTCGGTCCGTTAAATGCATCGGAATCCAATCCATCTCCGTTTAGGTAGAAGACTCGCC
GCCAGCAGATCCACAGGAGGCAGATCCTATAGCATACAACAAAGCGACGAAGGTTATTAC
TGTATGTTACGACAGATAAAATAAAGAATCAGATACAGTTCTAGTCAAGGTCAGATAATGTTCT
ACTTAAGGTCAGATAAGTTCTAGTCAAGGTCAGATACAGTTCTAGTCAAGGCCAGATATAGTT
CTAGTAAAGGTAAGATAAAGTTCTAGTCAAGGTCAAAGAAAGTTCTAGTTAAGGTCAGATACA
GTTTTAGTCATTCATTCATTCATTTATTCCGGCTATTTCTATAGCGCCCCTTCAAGTCCTCTAC
TACTCGGAGGCGCTCCCGACTACGGCACGGATACTGTACATGTATCTGAGTTTCACGCCG
AAGCGCACAGGCAACTGCAGGTAAAGGACTTGCCCAAGGTCCCTAAGTGGAGGCTAGA
ACGGGAGTCGAACCCACGACCCTCCGGTTGAAAGTTATCGTCTCATCCAAGGCGCCTCA
ACGTCCCACAAAGGTCAAGGTCAGATACAGTTCTATTTAAGGTCAGACACAGTTGTGGTCA
AGGTGCGGGTCCTTTTCGTAAGCACCCTGTTGGTCAGCAGCGCACTGACATGGATCGTAC
ATGAGGCAGCTTTATGTCCAGTCGTAGGCTAAGTCAATTGTGAAAGAGACCGTTATGACTAT
GAAGTGTTAGCGACTTCATTTTCTTAACCTCAATTTAAAAGGGTATGTGGCCAGTGCTCTCC
ATTGCCATCGGTTCTAACCGCAGACAGCGAGTAGTCCTACTCATAAGAAACCCATTAAAAC
ACTAACGGCAAAAAAGCCGCGACTGTAACCGTGTCGTCACTCGTCACGCTTGGCTTTCTC
CTGTCGCTTTTAGAGCCGGAACCTTCGTTCAATTCGCTGATGATGTAGGCTACTTTACCTTAT
TTCTGTGTAATTCTAGTTGCGGCTCTTGATAGCTGGAAGTCTTCTCGCCATTTAACGATTATTG
ATGGTTGATTGATCCTGTGAGGGTAATTGATTTGCAGATGCCAAGGAGTAAGGTATCGGATT
ACAAACGAAGATGAGCTCATAGATTCTGAAGTCTAACCTACATAGCTTACCTCGAGGATCA
GCGTAGCAACACAGGCCGATGCTTTTCATCTCCGCTTATAATATTATTATCGCTGTAATTATTT
ATACTTGCCTAACGAATAAAGGAATCAATTAATCGTAGCAGAAACAAGGCGGGTGATATTCG
TACAATAAACTCTAGCAAGGTAAGTTAACAATAATAATTATTTGTAAGTCAACAATCAGTAGGC
CTAATAACACTATCTTACAAATTCTTACTTTTTTTGTTTCTGGGTACAAAGTGTGTTTCCTAACT
CGTTATGCCTAAAACAAATGGAAAATAGGCCTGGCTGTTATTCCACAAAAACTTTGCTTCTG
TGATCAGGACAATGATAATTTTAAATTTGTCTGTTTTCGAGAAAATTCTCGATTCGTATTCAATA
CTGAGTATTCTTCTAGAATATTCTTGAACTGCTAGAAATGTCCAGAAGTTTCTAGAATTTTTCAG
AATTAGTAGTCATACAAATTTAAAAGCACAGGTGACTGGAACCAACATTTCGGTTTATGTTATT
GCTGAGTAAGAGAATAACGAATCGTAGTTAAAGAGCAATTTAGCCTATTTGATCTAAAAGGTA
TCAAGAAGTTGTTTGCACCCAACAGATGCATTTTTGCTACGTGTGTGGACAATTATAAAGAC
AAGAGCGAGAAAGTATTCCATGAAAGCATGTCGTAAGATGTGTGAGGCCTAGAAGGCATAC
TTCGGCAATCGGCATGCCTGTCGGGGATCAAGACAAGTCTTGGGCACCTCATTTCACATG
CGAATATTGAAAAAACTCTTGAAGGTAAGGTGAACAATTGCTGCTCGCTTAGATGGCAGTG
CTTCATTTTGTAGAATTTCAATGATTTAGATACAGTACAGTTTAAGGAGTTGCAATTGACAAAAT
TTTCACATATGTCAATGTAGGCCTACTATAGGAAAATATGTTCTATGTTAATATCATACTTTGTGT
TAAGTAGTGTGTAAAATTTCCTGCTTTTAAATTTCTGTAATTAATTTATATGTGCTATGTTACAGGT
TGGTACATGGGAGAAAAGAGAGCCATAAAGTTTGCTATCCCGCGAAATTGGCCGCAACC
GACTGATCACTCAAGCAACTGCTACTTCTGCATGGTGGATCCTATCAAACGTCGCACTGGC

AAGAATGCGCCTCAAATCGTTTATCCAGACATTCCTTTTTCCATTGCCCCAGTACTACACTG
CCCCGAGCTGCCTGTTCCCACTCCCCGAAGAGGGATCAGCCATCTTCAGGAGACAGAA
GCAAGTCAGACAGCGAGAAAGATATTGGAGATCCAGATTATGTTTTCACAGATGCATTGAG
GAGAGAAGGCCATACTTCCCTAACCAGAAAGACGTCAACGATCTGATCAGAGACCTTGGT
CTTACCAAGTCCAATGCTGAGCTTCTGATATCCAGGCTCAAACAACGGAACAGTCTTGTTG
GATGAAAGCGTGCAAGTCACAGATCAGAGAAAGCGTCACGAAACATTTTCCAACTCCTTC
AGTCGGCAAGATGGGCTGTGCTTCTGCAACAATGTGGCCGGTCTATTCGAGGCTATAGGT
ATCACTTGTAACCCGAGTGAATGGCGCCTATTCATAGCCAGATTATCCCGGAGCCTCAAAG
CCGTGCTGCTTCACAACAGAAACAACTAGCCTACCCGTCTCTCCATAGGCTATTGCTAACT
CTGTGCATCTCAAATAGGACTACACCAGTGTCAAGATGTTGCTGAGTGCCTTGAAGTATGAC
GACTATGGATGGGATGTCATCGGAGACTTCAAAATGGTGTCATTCCTTATGGTCCTTCAAGG
AGGTTTCACGAAATTTCATTGTTTCCTTTGCCTCAAGGACAGTAAAATTATAACGAAAACATG
ATGGGGGACTAGTTCCGGACTACATCTGGGGGCTTCTTCGAGAAAGCGAAAGAATGATAC
AGTGAGCATGAAAGGATGATAGCCATCTCGTTCGAGGTCTTATTTAACACTGCTGCCGCCT
AGCGAGTGACAATTAGTCAACTTTTTTAACAAGAATGCCAAGTTAGCGTCCAATACGGGGC
GCGAACCCGGTGTAACAGGCCAGAGTATACCCCCGGCCGGACTATACCCGGGGTTAAA
GCGCCCTAGGCTAGATTATACCCGGGTGTATTATGGCCTAGGCCAATTTATACCCCGGGTT
AAATTATGGCCTAGGCCAATTCATACTCCCTCAGGCCAAATTATACACCCCTGAAATTAATC
ACGATATCAATAGTGTTGAGCGATATACTCAAGAATTATTTAATTTTTCAACAACTTACTGAGG
ATTCGATTTTGTCCGGTAAGTTAGGTGAGAGAGCTAAGCTAACCGAACTGAAATCTGAAAAC
TCTAAAGCTGACCATGGTTGAAAACTAATAAGTAAATATTAATTTAATATAAATTGGGGCATGG
CCGGATTTAGTCGTGGTCAAGGTGTGCCACTGCACAGGGGTCTCCACCAGCTAGGGGC
CTCCACCAACTCCAATAAAATGCTTTATAGATTAGGCCTAATTACAGTCGTGTACACAGGTC
TCCTAGCGCAGCCTAGTTGATTTGCTTTAGAACGTTGGCCGTGGCTTACATTCTTCTACAAT
TAACTAAAAGTATTAGCATAATCAGTCTACATTTAATCTGGACAGGTTACTCGCAGCCTTGATT
ACACCTGGGGTTAAAGCGGCCCAGGCTAGATTATACTCGGGTATATTATGGCCTGGGCCC
ATTTATACCCCGGGGTATATTATGGCCTGAGGGGGTATGAATTGGTTTAGGCCAAAATATAC
CCCGGGGTATAAATTGGCCTAGACCATAATATATCCGGGTATATTCCGGCCGGGGGTTTAT
TCTGGCCTGTTACACCGGGTTGTCAGCACTAACACACCAGATATTTCCCACTACAAATTGT
GTTAGGCTAATGCGTTCCCGAACCACAAATGAGTTCCCGAACCTTTTTCCTAGAAAACGG
CACTGCTGACCGACCCGTCACGATCTCTTTGTCCCGCTAGCGAGGATTTCTATGGCTCAG
AAACGAGCCTTTGCGGTCATTGGCCCTTCGCTTTGTAACCAACACCTTCCTTCTACGCGAT
CCTCTTTATTAGCTGGTGAGCCGAGTGCCTCTTTTCGTTCTCTCAAGTCTGTTCTCTAGTTCT
CTCGCACTGGAAGCGCTTCTGATTGGTGTGCACTGCAAGAAGCGCTATATAAATGTATAGAT
ACAATACAAAACGATACAATACAATACAATAAAAGATAGCCTACTTATTACGGTAAAAATCATT
TGGATAGTTTTACATGTATTTTCAAAAATAATACTTGCATAGCTTAGTTAAAATTCGAAAGGTGG
AAGTAGTGCAGTTTCAGCTCTTGTCTTAAGGTAGGGCAATGCCGAAGGCCCTGTCGCATTG
TGATGGATGAGATTCGAAGTGTTTGTCCTGTTTTGTGTGTGTCGTATGCTCATTCTATTTGCCT
CTCGTCTTTCTGTTCATATCAGCTTATAGATCGCTGGGATGACTTGCACAGACACAGGATCG
AACATTTACACTGCGTAAATTTAACGGAAATATTCTTGCACAGAACGGCTTGAATCGTTTAAC
TTGAAACGTGACTTCTCATACGTTTTACACTTTGTAAACCACGGCTGTCGGCATAAATTAATC
CAACATAGCAAATAGTTTAGGCAAAGTCATGATATTCAACCAAAACCAAAGACGAATATTCG
AGAAATATTAAGTGTGCATCAGTTGGTTCTTCGTAGATTTATGTCTGGAAAAGTTAATCGATATA
AGGATTCTTTTAAAAACAATCACCAATAAACATCATTTATGATCATTAATTCTTTTCATCTTCATC
ACACGGCACCTGCTATGTCATCTAGATCTCAGAATGGATCACATGCCGGATGCTGTCGCTG
TCTTCTCATCACCTGCAGATGGCGATGGCAGCAAAACTTACAGCAACCAGTATGTTTACGT
TGACAACCACCATGGTGGCTACATCTAAAATATATTTAGCAAACAATAATTAACTTAATAATAA

```
TGCATACACTTAAGCCATTAATAGATGAGGGTCAACGTTTTACTAAACTAAAATATGCAGAAA
AGAACTCATTAAAAAATTAGCTGGGCCGCAGTCAGACAAATTTTGGGCTCATTTTATGCAAT
TTTATGCTTTATAACAGGCGGATTCATTTGAGCGTTCGAACTCGGAAACCCCCTCCTAAATA
CGACAATGAGTATTATTTATTTATAGCATTCATTAGGCGCACTGAGTCTGCAAATCACGTGCG
CAATTAATGAGCCAGGGACTGTGCGCGTTTGTCATGTAGTTCGATCAATGGACTCGAACCG
GCTATCGTTGAATGGAGAGAAAATGGCGAGTATACAGAAGTCATAACAGGCGACGTGGCT
ACTGCATCGGTTTCTGTCTCTCTCTCTCTCTGTCTCTCTGTCTCTCTCTCTCTCTGCCTCTCT
GTCTCTGACCTTCTGTAGGTTACTAACCGTGCTGCTGCGTAGGTTCATCAATGCATCGATATT
TATTCTTGAAAGACGACAGGCAGACACTTCCCTCCGAACAAGGCGACGACAGACAAGGA
TTACATGAAGTGTCTGAAGAGAAATAAAACGAAAGTATTAAGGATGATCGCACCGGCGTGG
CAGGCACCACGTGCTCTAGGTAGAACGTACTCTAGAGTCCATCATGCCTACAGACATCTC
GGACTCTATAGAGTACAAAATTTCTAGCCCACACCCAGTCATGACGTAGTCTATCCAGGCA
AGGTGTACTCTAGGCAAATACTTACAATAGATTGCATTACGCCTAGGCACAACGTACTCTAG
CTTACGTCGCACCTAGGCACAGCGTACTCTAGAGTGCATCATACGTAGGCACAGTGTATTC
TACAGCCAACGCACTCTAACACACTGCGTACATTTGAGTACATAATGCCTAGGCACAGCGT
ACCATAGGGTACATTATAACTAGGCGCAGCGTATCAAGTGTATCATGTCTATAGGCCTAAAG
TCTATCCACCGTAAGACATGGTGTACTCTACGCAAAGAGaagctt
```
