# Supplementary material for: Insights into the architecture of earthworm metallothionein genes, powered by long-read genomics and transcriptomics
Source: NAR Genom Bioinform. 2026 Jan 8;8(1):lqaf195. doi: 10.1093/nargab/lqaf195 (PMC12783039; doi:10.1093/nargab/lqaf195)
Supplement: lqaf195_Supplemental_Files [file lqaf195_supplemental_files.zip › Supplementary_Tables.pdf]

%	Lr13H12_down	Ef472C1	Lr13H12_up	Ef318A10	Lr6F14
Lr13H12_down	100	90.48	83.63	34.41	4.38
Ef472C1	90.48	100	91.42	35.35	4.91
Lr13H12_up	83.63	91.42	100	27.18	5.17
Ef318A10	34.41	35.35	27.18	100	8.86
Lr6F14	4.38	4.91	5.17	8.863	100

Supplementary Table 1: Identities of pairwise comparisons between wMT regions of BACs.

	Nucleotide sequence	Protein sequence
Lr13H12<br>up/downstream	ATGGCAGACGCACTCGACACTCAGTGCTGTGGAAAATCAACCTGCCAAAGAGAGGGATCAACTT<br>GTTGCTGCACAAACTGCAGATGTTTGAAAAGTGAGTGCTTGCCAGGCTGCAAAAAGCTTTGCTG<br>TGCTGACGCCGAGAAGGGCAAATGTGGAAATGCCGGCTGCAAGTGCGGGGCAGCTTGCAAATG<br>CTCGGCGGGTTCGTGCGCCGCAGGATGCAAGAAGGGATGCTGTGGTGACTAG	MADALDTQCCGKSTCQREGSTCCCTNCRCL<br>KSECLPGCKKLCCADAEKGKCGNAGCKCGA<br>ACKCSAGSCAAGCKKGCCGD*
Lr6F14	ATGGCTGACGCACTCAACACTCAGTGCTGTGGAAAAGACCAATGCCCAA<br>GACGGGAGTCAGCGTGTGTCTGCAACAATTGCAGATGTCTGAAAAGTGAATGCTTGCCAAACTG<br>CAAGAAGCTGTGCTGTGCTGATGCCCAAGGACAATGTGGAA<br>ATACAGGCTGCAAGTGTGGGGCTGCCTGCAAGTGTTCAGCTGGTTCGTGTGCAACAGGCTGCA<br>AGAAGGGATGCTGTGGTGATTAA	MADALNTQCCGKDQCPRRESACVCNNCRCL<br>KSECLPNCKKLCCADAQGQCGNTGCKCGAA<br>CKCSAGSCATGCKKGCCGD*
Ef472C1	ATGGCAGACGCACTCGACACTCAGTGCTGTGGAAAATCAACCTGCCAAAGAGAGGGATCAACTT<br>GTTGCTGCACAAACTGCAGATGTTTGAAAAGTGAGTGCTTGCCAGGCTGCAAAAAGCTTTGCTG<br>TGCTGACGCCGAGAAGGGCAAATGTGGAAATGCCGGCTGCAAGTGCGGGGCAGCTTGCAAATG<br>CTCGGCGGGTTCGTGCGCCGCAGGATGCAAGAAGGGATGCTGTGGTGACTAG	MADALDTQCCGKSTCQREGSTCCCTNCRCL<br>KSECLPGCKKLCCADAEKGKCGNAGCKCGA<br>ACKCSAGSCAAGCKKGCCGD*
Ef318A10	ATGGCAGACGCACTCGACACTCAGTGCTGTGGAAAATCAACCTGCCAAAAGGGATCAACTTGTT<br>GCTGCACAAACTGCAGATGTTTGAAAAGTGATTGCTTGCCAGGCTGCAAAAAGCTTTGCTGTGC<br>TGACGCTGAGAAGGGCAAATGTGGAAATGCAGGCTGCAAGTGCGGGGCAGCTTGCAAATGCTC<br>GGCGGGTTCGTGCGCCGCAGGATGCAAGAAGGGATGCTGTGGTGACTAG	MADALDTQCCGKSTCQKGSTCCCTNCRCLK<br>SDCLPGCKKLCCADAEKGKCGNAGCKCGAA<br>CKCSAGSCAAGCKKGCCGD*

Supplementary Table 2: Proposed canonical nucleotide sequences of the wMT coding regions on BACs used in this study, and their translations acquired with NCBI's standard codon table.

BAC	Site type	Intron 1 seq	Intron 2 seq	Intron 3 seq
Lr13H12_down	5’	CTCGACACTCGTAAGTCGAC	ACCTGCCAAAGTAAGCCAAA	AAATGTGGAAGTGAGTTTGC
	3’	TGCTTTTAGAGTGCTGTGGA	TTTTTTTAGGAGAGGGATCA	ACATTTCAGATGCCGGCTGC
Lr13H12_up	5’	CTCGACACTCGTAAGTCGAC	ACCTGCCAAAGTAAGCCAAA	AAATGTGGAAGTGAGTTTGC
	3’	TGCTTTTAGAGTGCTGTGGA	TTTTTTTAGGAGAGGGATCA	ACATTTCAGATGCCGGCTGC
Lr6F14	5’	CTCAACACTCGTAAGTATAT	CAATGCCCAAGTAAGCCACA	CAATGTGGAAGTACGTTTTC
	3’	TGCTTTCAGAGTGCTGTGGA	TCGTTTTAGGACGGGAGTCA	ACATTTCAGATACAGGCTGC
Ef472C1_rc	5’	CTCGACACTCGTAAGTAGAC	ACCTGCCAAAGTAAGCCAAA	AAATGTGGAAGTGAGTTTGC
	3’	TGCTTTTAGAGTGCTGTGGA	TTTTTTTAGGAGAGGGATCA	ACATTTCAGATGCCGGCTGC
Ef318A10	5’	CTCGACACTCGTAAGTAGAC	ACCTGCCAAAGTAAGCCAAA	AAATGTGGAAGTGAGTTTGC
	3’	TGCTTTTAGAGTGCTGTGGA	TTTTTTTAGGAGAGGGATCA	ACATTTCAGATGCAGGCTGC

Supplementary Table 3: Proposed canonical splice-site sequences of wMT genes in BACs.

	eukaryota_odb10	bacteria_odb10	archaea_odb10
**Complete**	99.2%	38.0%	69.6%
**Complete single-copy**	4.3%	6.5%	2.6%
**Complete duplicated**	94.9%	31.5%	67.0%
**Fragmented**	0.0%	15.3%	4.6%
**Missing**	0.8%	46.7%	25.8%
**n**	255	124	194

Supplementary Table 4: BUSCO completeness of the long read RNAseq dataset.

Tissue type	wMT-1 (count)	wMT-1 (%)	wMT-2 (count)	wMT-2 (%)	wMT-3 (count)	wMT-3 (%)
Nephridia	4	6.9	27	26.7	27	8.0
Gut	33	56.9	39	38.6	28	8.3
Calciferous gland	7	12.1	19	18.8	64	19.0
Pharynx	1	1.7	3	3.0	60	17.8
Body wall	0	0	2	2.0	45	13.4
Crop	11	19.0	2	2.0	25	7.4
Gizzard	2	3.4	5	5.0	9	2.7
Nerve cord	0	0	4	4.0	51	15.1
Seminal vesicles	0	0	0	0	9	2.7
Clitellum	0	0	0	0	19	5.6
Total count	58	100	101	100	337	100

Supplementary Table 5: wMT homologue transcript counts by tissue.

wMT1 gut	GGAAAGAAGAAAAAGAAAATCACAACCTGAGGCTCTGAAATGTGGAAGTCTG<br>TCATCCGTTAATGACTTAATTCGTTTTAATTATTCACTTGAGGAAGAATATGAAC<br>AACATTTTGCAACTTTTCTTGAGTAACTTGTGTTAATCAATATTCCAATATGATAC<br>ATTTGATTTTTTATTGGATGCATTCTGTGATAGTTTAATTTTTCAAAGAAAGATCA<br>AAAGATGTGTGCTGTAACTAACTAGTCGTTGAGACGTATGATAAGCTACTGAAT<br>TGTTGTCGAAAGATTGTTGGTGAATAAAAAAATTGTCTGAGAACT
wMT2 gut	CTACATGTAGTGTGGAGTCACAGTCACTTTGAAATGTGGAATTCTGTCTGCTG<br>CGGCTGATTACTTAATTCATTTTAATTGCTTATCTGAGAAAGAATATGAAGAACA<br>TTATGTAACATTATTCCGATTTGATACATTTTTATTTATGGACGAAATTGTTTGATT<br>TGGCAGACGAAATCAAAAGATGTATGTGGTAAAGTTGAAATGAACTTGCAAGA<br>AACATTCAGAGCTAAATTCATATGATTTAACTCATCTTTGTGAAATATGATCAGTT<br>CTTGTTCAAATTGTTTTGTAGAAATTGTTTGCTAATAAAAATTGTTCAATAAAAAC<br>TACTTCATAAAAC
wMT3 gut	GAAGAGAAAGACGAATCCAGAGGCATTGGATGATGCGTCGAATTCTGTCGTC<br>GATGCATCCATGTAACCGAAAGACTGTGAAGAACATTCTCTAGCTTCTCTTAAC<br>CATTCGATGGCAAAAGGTGTTTAGTTTTGATGCATTTTAGTGTGCACTCGTTGT<br>CAAAGTATTGACCATACATTCTCATTTTGTGTTGGTTTAAGTTTATTACTTGAAAA<br>ACTGAGGCAGCATTCTGGCAGTTCGCATTGATTAAAAACATAAACTATGCGCAA<br>CAGAGTTGGAAAGAATTTACGCTTGAGTGAAGTTTTCAATATGATCGATATCTTT<br>TAAGATGAAATGTTGATTTATTGCTTTATTTGTTACACAGTTGAAAGTGACTTCA<br>AGATCAGTTTATTTTGTGTTAAAACTGATGGGTTGTTTTTCCATTCATGTAGCTC<br>TCACTAATGTTTTCAGTTCTCTTCCTCTGTGTTTTACACATGAGTATTCCTCTCC<br>ACTTTCTTTTCACACACTGCTGCTCATCATTTCGTCTGTAACACTTGCAGAAGA<br>CCCACGTACGCTTTTTACAGCAGTCGCCGTGGAAACCTGTTTTCTTTCTTTAG<br>ATTGCTAAACCATCGGAGCAATTCTGTATATCATTTAGTACTAATCAGTAGTAGT<br>CCCTAAACAATATCTTTCATTGAATTTCATGTGGTCGGCTTTACTTTTCTTATCAT<br>TCCTAACCTTTACTTTGCTTATCATTTCAAACCTTTACTTATCATTCATAGACTGT<br>CTCACACAACATTTGTACATGGAATGTCTTGTTATGATTTCATTATAATAGTTGAC<br>GTGTTTACAATTTTTAAAATATTCCATTACTCGTGGCT

Supplementary Table 6: 3'UTR sequences of randomly selected gut metallothionein transcripts of each wMT clade.

wMT1 neph	GGAAAGAAGAAAAAGAAAATCACAACCTGAGGCTCTGAAATGTGGAAGTCTG<br>TCATCCGTTAATGACTTAATTCGTTTTAATTATTCACTTGAGGAAGAATATGAAC<br>AACATTTTGCAACTTTTCTTGAGTAACTTGTGTTAATCAATATTCCAATATGATAC<br>ATTTGATTTTTTATTGGATGCATTCTGTGATAGTTTAATTTTTCAAAGAAAGATCA<br>AAAGATGTGTGCTGTAACTAACTAGTCGTTGAGACGTATGATAAGCTACTGAAT<br>TGTTGTCGAAAGATTGTTGGTGAATAAAAAAATTGTCTGAGAAC
wMT2 neph	CTACATGTAGTGTGGAGTCACAGTCACTTTGAAATGTGGAATTCAGTCTGCTG<br>CGGCTGATTACTTAATTCATTTTAATTGCTTATCTGAGAAAGAATATGAAGAACA<br>TTATGTAGCTTTCTCGAGTAGTTTTAGATAATCTTTATTCCGATTTGATACATTTT<br>TATATATGGACGAAATTGTTTGATTTGGCAAACGAAATCAAAAGATGTATGTGGT<br>AAAGTTGAAATGAACTTGCAAGAAACATTCAGAGCTAAATTCATATGATTTAACT<br>CATCTTTGCGAAATATGATCAGTTCTTGTTCAAATTGTTTTGTAGAAATTGTTTG<br>CTAATAAAAATTGTTTAAT
wMT3 neph	GAAGAGAAAGACGAATCCAGAGGCATTGGATGATGCGTCGAATTCTGTCGTC<br>GATGCATCCATGTAACCGAAAGACTGTGAAGAACATTCTCTAGCTTCTCTTAAC<br>CATTCGATGGCAAAAGGTGTTTAGTTTTGATGCATTTTAGTGTGCACTCGTTGT<br>CAAAGTATTGACCATACATTCTCATTTTGTGTTCGTTTAAGTTTATTACTTGAAAA<br>ACTGAGGCAGCATTCTGGCAGTTCGCATTGATTAAAAACATAAACTATGCGCAA<br>CAGAGTTGGAAAGAATTTACGCTTGAGTGAAGTTTTCAATATGATCGATATCTTT<br>TAAGATGAAATGTTGATTTATTGCTTTATTTGTTACACAGTTGAAAGTGACTTCA<br>AGATCAGTTTATTTTGTGTTAAAACTGATGGGTTGTTTTTCCATTCATGTAGCTC<br>TCACTAATGTTTTCAGTTCTCTTTCTCTGTGTTTTACACATGAGTATTCCTCTCC<br>ACTTTCTTTTCACACACTGCTGCTCATCATTTCGTCTGTAACACTTGCAGAAGA<br>CCCACGTACGCTTTTTACAGCAGTCGCCGTGGAAACCTGTTTCTTTCTTTAGA<br>TTGCTAAACCATCAGAGCAATTCTGTATATCATTTAGTACTAATCAGTAGTAGTC<br>CCTAAACAATATCTTTCATTGAATTTCATGTGGTCGGCTTTACTTTTCTTATCATT<br>CCTAACCTTTACTTTGCTTATCATTTCAAACCTTTACTTATCATTCATAGACTGTC<br>TCACACAACATTTGTACATGGAATGTCTTGTTATGATTTCATTATAATAGTTGAC<br>GTGTTTACAATTTTAAAATATTCCATTACTCGTGGCTTATTTTTGTGTTTGCTTCT<br>TTTCTCCGTTTCATTCATTCCTATGGGTGCAGCAAAAAGTTGGAAAGCATAATG<br>TACTGTATGCATGAAGTGTTTAAAACTGAGTTTTTTGCTTAACAAGTTTTTGTGA<br>AAATGTTCAATTTCATTGTGGTGAAATCGCAGTGCTAATAAAATTGTTTAATATC<br>A

Supplementary Table 7: 3’UTR sequences of randomly selected nephridia metallothionein transcripts of each wMT clade.

wMT1 cal_g	GGAAAGAAGAAAAAGAAAATCACAACCTGAGGCTCTGAAATGTGGAAGTCTG<br>TCATCCGTTAATGACTTAATTCGTTTTAATTATTCACTTGAGGAAGAATATGAAC<br>AACATTTTGCAACTTTTCTTGAGTAACTTGTGTTAATCAATATTCCAATATGATAC<br>ATTTGATTTTTTATTGGATGCATTCTGTGATAGTTTAATTTTTCAAAGAAAGATCA<br>AAAGATGTGTGCTGTAACTAACTAGTCGTTGAGACGTATGATAAGCTACTGAAT<br>TGTTGTCGAAAGATTGTTGGTGAATAAAAAAATTGTCTGAGAACTACTC
wMT2 cal_g	CTACATGTAGTGTGGAGTCACAGTCACTTTGAAATGTGGAATTCAGTCTGCTG<br>CGGCTGATTACTTAATTCATTTTAATTGCTTATCTGAGAAAGAATATGAAGAACA<br>TTATGTAGCTTTCTCGAGTAGTTTTAGATAATCTTTATTCCGATTTGATACATTTT<br>TATATATGGACGAAATTGTTTGATTTGGCAAACGAAATCAAAAGATGTATGTGGT<br>AAAGTTGAAATGAACTTGCAAGAAACATTCAGAGCTAAATTCATATGATTTAACT<br>CATCTTTGCGAAATATGATCAGTTCTTGTTCAAATTGTTTTGTAGAAATTGTTTG<br>CTAATAAAAATTGTTTAATAAAAAACTACTTCATC
wMT3 cal_g	GAAGAGAAAGACGAATCCAGAGGCATTGGATGATGCGTCGAATTCTGTCGTC<br>GATGCATCCATGTAACCGAAAGACTGTGAAGAACATTCTCTAGCTTCTCTTAAC<br>CATTCGATGGCAAAAGGTGTTTAGTTTTGATGCATTTTAGTGTGCACTCGTTGT<br>CAAAGTATTGACCATACATTCTCATTTTGTGTTGGTTTAAGTTTATTACTTGAAAA<br>ACTGAGGCAGCATTCTGGCAGTTCGCATTGATTAAAAACATAAACTATGCGCAA<br>CAGAGTTGGAAAGAATTTACGCTTGAGTGAAGTTTTTCAATATGATCGATATCTT<br>TTAAGATGAAATGTTGATTTATTGCTTTATTTGTTACACAGTTGAAAGTGACTTC<br>AAGATCAGTTTATTTTGTGTTAAAACTGATGGGTTGTTTTTCCATTCATGTAGCT<br>CTCACTAATGTTTTCAGTTCTCTTCCTCTGTGTTTTACACATGAGTATTCCTCTC<br>CACTTTCTTTTCACACACTGCTGCTCATCATTTCGTCTGTAACACTTGCAGAAG<br>ACCCACGTACGCTTTTTACAGCAGTCGCCGTGGAAACCTGTTTCTTTCTTTAG<br>ATTGCTAAACCATCGGAGCAATTCTGTATATCATTTAGTACTAATCAGTAGTAGT<br>CCCTAAACAATATCTTTCATTGAATTTCATGTGGTCGGCTTTACTTTTCTTATCAT<br>TCCTAACCTTTACTTTGCTTATCATTTCAAACCTTTACTTATCATTCATAGACTGT<br>CTCACACAACATTTGTACATGGAATGTCTTGTTATGATTTCATTATAATAGTTGAC<br>GTGTTTACAATTTTTAAAATATTCCATTACTCGTGGCTTATTTTTGTGTTTGCTTC<br>TTTTCTCCGTTTCATTCATTCCTATGGGTGCAGCAAAAAGTTGGAAAGCATAAT<br>GTACTGTATGCATGAAGTGTTTAAAACTGAGTTTTTTGCTTAACAAGTTTTTGTG<br>AAAATGTTCAATTTCATGTGGAGAAATCGCAACGCTAATAAAATTGTTTAATATC<br>AACA

Supplementary Table 8: 3'UTR sequences of randomly selected calciferous gland metallothionein transcripts of each wMT clade.

wMT1 phar	GGAAAGAAGAAAAAGAAAATCACAACCTGAGGCTCTGAAATGTGGAAGTCTG<br>TCATCCGTTAATGACTTAATTCGTTTTAATTATTCACTTGAGGAAGAATATGAAC<br>AACATTTTGCAACTTTTCTTGAGTAACTTGTGTTAATCAATATTCCAATATGATAC<br>ATTTGATTTTTTATTGGATGCATTCTGTGATAGTTTAATTTTTCAAAGAAAGATCA<br>AAAGATGTGTGCTGTAACTAACTAGTCGTTGAGACGTATGATAAGCTACTGAAT<br>TGTTGTCGAAAGATTGTTGGTGAATAAAAAAATTGTCTGAGAACTACTTAAGC
wMT2 phar	CTACATGTAGTGTGGAGTCACAGTCACTTTGAAATGTGGAATTCTGTCTGCTG<br>CGGCTGATTACTTAATTCATTTTAATTGCTTATCTGAGAAAGAATATGAAGAACA<br>TTATGTAACATTATTCCGATTTGATACATTTTTATTTATGGACGAAATTGTTTGATT<br>TGGCAGACGAAATCAAAAGATGTATGTGGTAAAGTTGAAATGAACTTGCAAGA<br>AACATTCAGAGCTAAATTCATATGATTTAACTCATCTTTGTGAAATATGATCAGTT<br>CTTGTTCAAATTGTTTTGTAGAAATTGTTTGCTAATAAAAATTGTTCAATAAAAAC<br>T
wMT3 phar	GAAGAGAAAGACGAATCCAGAGGCATTGGATGATGCGTCGAATTCTGTCGTC<br>GATGCATCCATGTAACCGAAAGACTGTGAAGAACATTCTCTAGCTTCTCTTAAC<br>CATTCGATGGCAAAAGGTGTTTAGTTTTGATGCATTTTAGTGTGCACTCGTTGT<br>CAAAGTATTGACCATACATTCTCATTTTGTGTTCGTTTAAGTTTATTACTTGAAAA<br>ACTGAGGCAGCATTCTGGCAGTTCGCATTGATTAAAAACATAAACTATGCGCAA<br>CAGAGTTGGAAAGAATTTACGCTTGAGTGAAGTTTTCAATATGATCGATATCTTT<br>TAAGATGAAATGTTGATTTATTGCTTTATTTGTTACACAGTTGAAAGTGACTTCA<br>AGATCAGTTTATTTTGTGTTAAAACTGATGGGTTGTTTTTCCATTCATGTAGCTC<br>TCACTAATGTTTTCAGTTCTCTTTCTCTGTGTTTTACACATGAGTATTCCTCTCC<br>ACTTTCTTTTCACACACTGCTGCTCATCATTTCGTCTGTAACACTTGCAGAAGA<br>CCCACGTACGCTTTTTACAGCAGTCGCCGTGGAAACCTGTTTCTTTCTTTAGA<br>TTGCTAAACCATCAGAGCAATTCTGTATATCATTTAGTACTAATCAGTAGTAGTC<br>CCTAAACAATATCTTTCATTGAATTTCATGTGGTCGGCTTTACTTTTCTTATCATT<br>CCTAACCTTTACTTTGCTTATCATTTCAAACCTTTACTTATCATTCATAGACTGTC<br>TCACACAACATTTGTACATGGAATGTCTTGTTATGATTTCATTATAATAGTTGAC<br>GTGTTTACAATTTTTAAAATATTCCATTACTCGTGGCTTATTTTTGTGTTTGCTTC<br>TTTTCTCCGTTTCATTCATTCCTATGGGTGCAGCAAAAAGTTGGAAAGCATAAT<br>GTACTGTATGCATGAAGTGTTTAAAACTGAGTTTTTTGCTTAACAAGTTTTTGTG<br>AAAATGTTCAATTTCATTGTGGTGAAATCGCAGTGCTAATAAAATTGTTTAATAT<br>CAAC

Supplementary Table 9: 3’UTR sequences of randomly selected pharynx metallothionein transcripts of each wMT clade.

wMT2<br>bod_w	CTACATGTAGTGTGGAGTCACAGTCACTTTGAAATGTGGAATTCTGTCTGCTG<br>CGGCTGATTACTTAATTCATTTTAATTGCTTATCTGAGAAAGAATATGAAGAACA<br>TTATGTAACATTATTCCGATTTGATACATTTTTATTTATGGACGAAATTGTTTGATT<br>TGGCAGACGAAATCAAAAGATGTATGTGGTAAAGTTGAAATGAACTTGCAAGA<br>AACATTCAGAGCTAAATTCATATGATTTAACTCATCTTTGTGAAATATGATCAGTT<br>CTTGTTCAAATTGTTTTGTAGAAATTGTTTGCTAATAAAAATTGTTCAATAAAAAC<br>TACTTCAT
wMT3<br>bod_w	GAAGAGAAAGACGAATCCAGAGGCATTGGATGATGCGTCGAATTCTGTCGTC<br>GATGCATCCATGTAACCGAAAGACTGTGAAGAACATTCTCTAGCTTCTCTTAAC<br>CATTCGATGGCAAAAGGTGTTTAGTTTTGATGCATTTTAGTGTGCACTCGTTGT<br>CAAAGTATTGACCATACATTCTCATTTTGTGTTGGTTTAAGTTTATTACTTGAAAA<br>ACTGAGGCAGCATTCTGGCAGTTCGCATTGATTAAAAACATAAACTATGCGCAA<br>CAGAGTTGGAAAGAATTTACGCTTGAGTGAAGTTTTCAATATGATCGATATCTTT<br>TAAGATGAAATGTTGATTTATTGCTTTATTTGTTACACAGTTGAAAGTGACTTCA<br>AGATCAGTTTATTTTGTGTTAAAACTGATGGGTTGTTTTTCCATTCATGTAGCTC<br>TCACTAATGTTTTCAGTTCTCTTCCTCTGTGTTTTACACATGAGTATTCCTCTCC<br>ACTTTCTTTTCACACACTGCTGCTCATCATTTCGTCTGTAACACTTGCAGAAGA<br>CCCACGTACGCTTTTTACAGCAGTCGCCGTGGAAACCTGTTTCTTTCTTTAGA<br>TTGCTAAACCATCGGAGCAATTCTGTATATCATTTAGTACTAATCAGTAGTAGTC<br>CCTAAACAATATCTTTCATTGAATTTCATGTGGTCGGCTTTACTTTTCTTATCATT<br>CCTAACCTTTACTTTGCTTATCATTTCAAACCTTTACTTATCATTCATAGACTGTC<br>TCACACAACATTTGTACATGGAATGTCTTGTTATGATTTCATTATAATAGTTGAC<br>GTGTTTACAATTTTTAAAATATTCCATTACTCGTGGCTTATTTTTGTGTTTGCTTC<br>TTTTCTCCGTTTCATTCATTCCTATGGGTGCAGCAAAAAGTTGGAAAGCATAAT<br>GTACTGTATGCATGAAGTGTTTAAAACTGAGTTTTTTGCTTAACAAGTTTTTGTG<br>AAAATGTTCAATTTCATGTGGAGAAATCGCAACGCTAATAAAATTGTTTAATATC<br>AACA

Supplementary Table 10: 3’UTR sequences of randomly selected body wall metallothionein transcripts of each wMT clade (no wMT-1 transcripts in body wall).

wMT1 crop	GGAAAGAAGAAAAAGAAAATCACAACCTGAGGCTCTGAAATGTGGAAGTCTG TCATCCGTTAATGACTTAATTCGTTTTAATTATTCACTTGAGGAAGAATATGAAC AACATTTTGCAACTTTTCTTGAGTAACTTGTGTTAATCAATATTCCAATATGATAC ATTTGATTTTTTATTGGATGCATTCTGTGATAGTTTAATTTTTCAAAGAAAGATCA AAAGATGTGTGCTGTAACTAACTAGTCGTTGAGACGTATGATAAGCTACTGAAT TGTTGTCGAAAGATTGTTGGTGAATAAAAAAATTGTCTGAGAACTACT
wMT2 crop	CTACATGTAGTGTGGAGTCACAGTCACTTTGAAATGTGGAATTCAGTCTGCTG CGGCTGATTACTTAATTCATTTTAATTGCTTATCTGAGAAAGAATATGAAGAACA TTATGTAGCTTTCTCGAGTAGTTTTAGATAATCTTTATTCCGATTTGATACATTTT TATATATGGACGAAATTGTTTGATTTGGCAAACGAAATCAAAAGATGTATGTGGT AAAGTTGAAATGAACTTGCAAGAAACATTCAGAGCTAAATTCATATGATTTAACT CATCTTTGCGAAATATGATCAGTTCTTGTTCAAATTGTTTTGTAGAAATTGTTTG CTAATAAAAATTGTTTAATAAAAAACTACTTCATC
wMT3 crop	GAAGAGAAAGACGAATCCAGAGGCATTGGATGATGCGTCGAATTCTGTCGTC GATGCATCCATGTAACCGAAAGACTGTGAAGAACATTCTCTAGCTTCTCTTAAC CATTCGATGGCAAAAGGTGTTTAGTTTTGATGCATTTTAGTGTGCACTCGTTGT CAAAGTATTGACCATACATTCTCATTTTGTGTTGGTTTAAGTTTATTACTTGAAAA ACTGAGGCAGCATTCTGGCAGTTCGCATTGATTAAAAACATAAACTATGCGCAA CAGAGTTGGAAAGAATTTACGCTTGAGTGAAGTTTTCAATATGATCGATATCTTT TAAGATGAAATGTTGATTTATTGCTTTATTTGTTACACAGTTGAAAGTGACTTCA AGATCAGTTTATTTTGTGTTAAAACTGATGGGTTGTTTTTCCATTCATGTAGCTC TCACTAATGTTTTCAGTTCTCTTCCTCTGTGTTTTACACATGAGTATTCCTCTCC ACTTTCTTTTCACACACTGCTGCTCATCATTTCGTCTGTAACACTTGCAGAAGA CCCACGTACGCTTTTTACAGCAGTCGCCGTGGAAACCTGTTTCTTTCTTTAGA TTGCTAAACCATCGGAGCAATTCTGTATATCATTTAGTACTAATCAGTAGTAGTC CCTAAACAATATCTTTCATTGAATTTCATGTGGTCGGCTTTACTTTTCTTATCATT CCTAACCTTTACTTTGCTTATCATTTCAAACCTTTACTTATCATTCATAGACTGTC TCACACAACATTTGTACATGGAATGTCTTGTTATGATTTCATTATAATAGTTGAC GTGTTTACAATTTTTAAAATATTCCATTACTCGTGGCTTATTTTTGTGTTTGCTTC TTTTCTCCGTTTCATTCATTCCTATGGGTGCAGCAAAAAGTTGGAAAGCATAAT GTACTGTATGCATGAAGTGTTTAAAACTGAGTTTTTTGCTTAACAAGTTTTTGTG AAAATGTTCAATTTCATGTGGAGAAATCGCAACGCTAATAAAATTGTTTAAT

Supplementary Table 11: 3'UTR sequences of randomly selected crop metallothionein transcripts of each wMT clade.

wMT1<br>gizz	GGAAAGAAGAAAAAGAAAATCACAACCTGAGGCTCTGAAATGTGGAAGTCTG<br>TCATCCGTTAATGACTTAATTCGTTTTAATTATTCACTTGAGGAAGAATATGAAC<br>AACATTTTGCAACTTTTCTTGAGTAACTTGTGTTAATCAATATTCCAATATGATAC<br>ATTTGATTTTTTATTGGATGCATTCTGTGATAGTTTAATTTTTCAAAGAAAGATCA<br>AAAGATGTGTGCTGTAACTAACTAGTCGTTGAGACGTATGATAAGCTACTGAAT<br>TGTTGTCGAAAGATTGTTGGTGAATAAAAAAATTGTCTGAGAACTA
wMT2<br>gizz	CTACATGTAGTGTGGAGTCACAGTCACTTTGAAATGTGGAATTCAGTCTGCTG<br>CGGCTGATTACTTAATTCATTTTAATTGCTTATCTGAGAAAGAATATGAAGAACA<br>TTATGTAGCTTTCTCGAGTAGTTTTAGATAATCTTTATTCCGATTTGATACATTTT<br>TATATATGGACGAAATTGTTTGATTTGGCAAACGAAATCAAAAGATGTATGTGGT<br>AAAGTTGAAATGAACTTGCAAGAAACATTCAGAGCTAAATTCATATGATTTAACT<br>CATCTTTGCGAAATATGATCAGTTCTTGTTCAAATTGTTTTGTAGAAATTGTTTG<br>CTAATAAAAATTGTTTAATAAAAAACTACTTCATCA
wMT3<br>gizz	GAAGAGAAAGACGAATCCAGAGGCATTGGATGATGCGTCGAATTCTGTCGTC<br>GATGCATCCATGTAACCGAAAGACTGTGAAGAACATTCTCTAGCTTCTCTTAAC<br>CATTCGATGGCAAAAGGTGTTTAGTTTTGATGCATTTTAGTGTGCACTCGTTGT<br>CAAAGTATTGACCATACATTCTCATTTTGTGTTGGTTTAAGTTTATTACTTGAAAA<br>ACTGAGGCAGCATTCTGGCAGTTCGCATTGATTAAAAACATAAACTATGCGCAA<br>CAGAGTTGGAAAGAATTTACGCTTGAGTGAAGTTTTCAATATGATCGATATCTTT<br>TAAGATGAAATGTTGATTTATTGCTTTATTTGTTACACAGTTGAAAGTGACTTCA<br>AGATCAGTTTATTTTGTGTTAAAACTGATGGGTTGTTTTTCCATTCATGTAGCTC<br>TCACTAATGTTTTCAGTTCTCTTCCTCTGTGTTTTACACATGAGTATTCCTCTCC<br>ACTTTCTTTTCACACACTGCTGCTCATCATTTCGTCTGTAACACTTGCAGAAGA<br>CCCACGTACGCTTTTTACAGCAGTCGCCGTGGAAACCTGTTTCTTTCTTTAGA<br>TTGCTAAACCATCGGAGCAATTCTGTATATCATTTAGTACTAATCAGTAGTAGTC<br>CCTAAACAATATCTTTCATTGAATTTCATGTGGTCGGCTTTACTTTTCTTATATCA<br>TTCCTAACCTTTACTTTGCTTATCATTTCAAACCTTTACTTATCATTCATAGACTG<br>TCTCACACAACATTTGTACATGGAATGTCTTGTTATGATTTCATTATAATAGTTGA<br>CGTGTTTACAATTTTTTAAAATATTCCATTACTCGTGGCTTATTTTTGTGTTTTGC<br>TTCTTTTCTCCGTTTCATTCATTCCTATGGGTGCAGCAAAAAAGTTGGAAAGCA<br>TAATGTACTGTATGCATGAAGTGTTTTTAAAACTGAGTTTTTTTGCTTAACAAGT<br>TTTTTGTGAAAATGTTCAATTTCATGTGGAGAAATCGCAACGCTAATAAAATTGT<br>TTAATATC

Supplementary Table 12: 3’UTR sequences of randomly selected gizzard metallothionein transcripts of each wMT clade.

wMT2 nerve	TGTGGAGTCACAGTCACTTTGAAATGTGGAATTCAGTCTGCTGCGGCTGATTA CTTAATTCATTTTAATTGCTTATCTGAGAAAGAATATGAAGAACATTATGTAGCTT TCTCGAGTAGTTTTAGATAATCTTTATTCCGATTTGATACATTTTTATATATATGGA CGAAATTGTTTGATTTGGCAAACGAAATCAAAAGATGTATGTGGTAAAGTTGAA ATGAACTTGCAAGAAACATTCAGAGCTAAATTCATATGATTTAACTCATCTTTGC GAAATATGATCAGTTCTTGTTCAAATTGTTTTGTAGAAATTGTTTGCTAATAAAAA TTGTTTAATAAAAAACTACTTCAT
wMT3 nerve	GAAGAGAAAGACGAATCCAGAGGCATTGGATGATGCGTCGAATTCTGTCGTC GATGCATCCATGTAACCGAAAGACTGTGAAGAACATTCTCTAGCTTCTCTTAAC CATTCGATGGCAAAAGGTGTTTAGTTTTGATGCATTTTAGTGTGCACTCGTTGT CAAAGTATTGACCATACATTCTCATTTTGTGTTGGTTTAAGTTTATTACTTGAAAA ACTGAGGCAGCATTCTGGCAGTTCGCATTGATTAAAAACATAAACTATGCGCAA CAGAGTTGGAAAGAATTTACGCTTGAGTGAAGTTTTCAATATGATCGATATCTTT TAAGATGAAATGTTGATTTATTGCTTTATTTGTTACACAGTTGAAAGTGACTTCA AGATCAGTTTATTTTGTGTTAAAACTGATGGGTTGTTTTTCCATTCATGTAGCTC TCACTAATGTTTTCAGTTCTCTTCCTCTGTGTTTTACACATGAGTATTCCTCTCC ACTTTCTTTTCACACACTGCTGCTCATCATTTCGTCTGTAACACTTGCAGAAGA CCCACGTACGCTTTTTACAGCAGTCGCCGTGGAAACCTGTTTCTTTCTTTAGA TTGCTAAACCATCGGAGCAATTCTGTATATCATTTAGTACTAATCAGTAGTAGTC CCTAAACAATATCTTTCATTGAATTTCATGTGGTCGGCTTTACTTTTCTTATCATT CCTAACCTTTACTTTGCTTATCATTTCAAACCTTTACTTATCATTCATAGACTGTC TCACACAACATTGTACATGGAATGTCTTGTTATGATTTCATTATAATAGTTGACG TGTTTACAATTTTTAAAATATTCCATTACTCGTGGCTTATTTTTGTGTTTGCTTCT TTTCTCCGTTTCATTCATTCCTATGGGTGCAGCAAAAAGTTGGAAAGCATAATG TACTGTATGCATGAAGTGTTTAAAACTGAGTTTTTTGCTTAACAAGTTTTTGTGA AAATGTTCAATTTCATGTGGAGAAATCGCAACGCTAATAAAATTGTTTAATA

Supplementary Table 13: 3’UTR sequences of randomly selected nerve metallothionein transcripts of each wMT clade (no neural wMT-1 transcripts found).

wMT3 sem_v	GAAGAGAAAGACGAATCCAGAGGCATTGGATGATGCGTCGAATTCTGTCGTC<br>GATGCATCCATGTAACCGAAAGACTGTGAAGAACATTCTCTAGCTTCTCTTAAC<br>CATTCGATGGCAAAAGGTGTTTAGTTTTGATGCATTTTAGTGTGCACTCGTTGT<br>CAAAGTATTGACCATACATTCTCATTTTGTGTTGGTTTAAGTTTATTACTTGAAAA<br>ACTGAGGCAGCATTCTGGCAGTTCGCATTGATTAAAAACATAAACTATGCGCAA<br>CAGAGTTGGAAAGAATTTACGCTTGAGTGAAGTTTTCAATATGATCGATATCTTT<br>TAAGATGAAATGTTGATTTATTGCTTTATTTGTTACACAGTTGAAAGTGACTTCA<br>AGATCAGTTTATTTTGTGTTAAAACTGATGGGTTGTTTTTCCATTCATGTAGCTC<br>TCACTAATGTTTTCAGTTCTCTTCCTCTGTGTTTTACACATGAGTATTCCTCTCC<br>ACTTTCTTTTCACACACTGCTGCTCATCATTTCGTCTGTAACACTTGCAGAAGA<br>CCCACGTACGCTTTTTTACAGCAGTCGCCGTGGGAAACCTGTTTCTTTCTTTA<br>GATTGCTAAACCATCGGAGCAATTCTGTATATCATTTAGTACTAATCAGTAGTAG<br>TCCCTAAACAATATCTTTCATTGAATTTCATGTGGTCGGCTTTACTTTTCTTATCA<br>TTCCTAACCTTTACTTTGCTTATCATTTCAAACCTTTACTTATCATTCATAGACTG<br>TCTCACACAACATTTGTACATGGAATGTCTTGTTATGATTTCATTATAATAGTTGA<br>CGTGTTTACAATTTTTAAAATATTCCATTACTCGTGGCTTATTTTTGTGTTTGCTT<br>CTTTTCTCCGTTTCATTCATTCCTATGGGTGCAGCAAAAAGTTGGAAAGCATAA<br>TGTACTGTATGCATGAAGTGTTTAAAACTGAGTTTTTTGCTTAACAAGTTTTTGT<br>GAAAATGTTCAATTTCATGTGGAGAAATCGCAACGCTAATAAAATTGTTTAAT

Supplementary Table 14: 3'UTR sequences of randomly selected seminal vesicle metallothionein transcript of each wMT clade (wMT-3 is exclusive to seminal vesicles).

wMT3 clitel	GAAGAGAAAGACGAATCCAGAGGCATTGGATGATGCGTCGAATTCTGTCGTC<br>GATGCATCCATGTAACCGAAAGACTGTGAAGAACATTCTCTAGCTTCTCTTAAC<br>CATTCGATGGCAAAAGGTGTTTAGTTTTTGATGCATTTTAGTGTGCACTCGTTG<br>TCAAAGTATTGACCATACATCTCATTTTGTGTTGGTTTAAGTTTATTACTTGAAAA<br>CACTGAGGCAGCATTCTGGCAGTTCGCATTGATTAAAAACATAAACTATGCGCA<br>ACAGAGTTGGAAAGAATTTACGCTTGAGTGAAGTTTTCAATATGATCGATATCT<br>TTTAAGATGAAATGTTGATTTATTGCTTTATTTTGTTACACAGTTGAAAGTGGAC<br>TTCAAGATCAGTTTATTTTTGTGTTAAAACTGATGGGTTGTTTTTCCATTCATGT<br>AGCTCTCACTAATGTTTTCAGTTCTCTTCCTCTGTGTTTTACACATGAGTATTCC<br>TCTCCACTTTCTTTTCACACACTTGCTGCTCATCATTTCGTCTGTTAAACACTT<br>GCAGAAGACCCACGTACGCTTTTTACAGCAGTCGCCGTGGAAACCTGTTTCTT<br>TCTTTAGATTGCTAAACCATCGGAGCAATTCTGTATATCATTTAGTACTAATCAGT<br>AGTAGTCCCTAAACATATCTTTCATTGAATTTCATGTGGTCGGCTTTACTTTTCT<br>TATCATTCCTAACCTTTACTTTGCTTATCATTTCAAACCTTTACTTATCATTCATAG<br>ACTGTCTCACACAACATTTGTACATGGAATGTCTTGTTATGATTTCATTATAATAG<br>TTGACGTGTTTACAATTTTTAAAATATTCCATTACTCGTGGCTTATTTTTGTGTTT<br>GCTTCTTTTCTCCGTTTCATTCATTCCTATGGGTGCAGCCAAAAAGTTGGAAAG<br>CAAATAATGTACTGTATGCATGAAGTGTTTAAACCTAGTTTTTTGCTTAACAAGT<br>TTTTGTGAAAATGTCAATTTCATGTGGAGAAATCGCAACGCTAATAAAATTGTTT<br>AATATCA

Supplementary Table 15: 3’UTR sequences of randomly selected clitellum metallothionein transcript of each wMT clade (wMT-3 is exclusive to clitellum).

qseqid	sseqid	qlen	qstart	qend	sstart	send	length	mismatch	gapopen	pident	evalue	bitscore
wMT1_gut	13H12	318	30	223	83383	83587	205	46	5	72.19	2e-23	101
wMT1_neph	13H12	317	30	223	83383	83587	205	46	5	72.19	2e-23	101
wMT1_cal_g	13H12	322	30	223	83383	83587	205	46	5	72.19	2e-23	101
wMT1_phar	13H12	326	30	223	83383	83587	205	46	5	72.19	2e-23	101
wMT1_crop	13H12	321	30	223	83383	83587	205	46	5	72.19	2e-23	101
wMT1_gizz	13H12	319	30	223	83383	83587	205	46	5	72.19	2e-23	101

Supplementary Table 16: Complete BLAST result of all randomly selected wMT 3'UTR sequences against Lr13H12. E-values were rounded. Subject length = 102281. No wMT-2 transcripts aligned. BLAST parameters – word_size=4, e-value=1e-2.

qseqid	sseqid	qlen	qstart	qend	sstart	send	length	mismatch	gapopen	pident	evalue	bitscore
wMT1_gut	472C1	318	30	223	9636	9432	205	46	5	72.19	2e-23	101
wMT1_neph	472C1	317	30	223	9636	9432	205	46	5	72.19	2e-23	101
wMT1_cal_g	472C1	322	30	223	9636	9432	205	46	5	72.19	2e-23	101
wMT1_phar	472C1	326	30	223	9636	9432	205	46	5	72.19	2e-23	101
wMT1_crop	472C1	321	30	223	9636	9432	205	46	5	72.19	2e-23	101
wMT1_gizz	472C1	319	30	223	9636	9432	205	46	5	72.19	2e-23	101

Supplementary Table 17: Complete BLAST result of all randomly selected wMT 3'UTR sequences against Ef472C1. E-values were rounded. Subject length = 97604. No wMT-2 transcripts aligned. BLAST parameters – word_size=4, e-value=1e-2.

qseqid	sseqid	qlen	qstart	qend	sstart	send	length	mismatch	gapopen	pident	evalue	bitscore
wMT1_gut	318A10	318	30	223	97115	97319	205	47	5	71.71	2e-22	96.9
wMT1_neph	318A10	317	30	223	97115	97319	205	47	5	71.71	2e-22	96.9
wMT1_cal_g	318A10	322	30	223	97115	97319	205	47	5	71.71	2e-22	96.9
wMT1_phar	318A10	326	30	223	97115	97319	205	47	5	71.71	2e-22	96.9
wMT1_crop	318A10	321	30	223	97115	97319	205	47	5	71.71	2e-22	96.9
wMT1_gizz	318A10	319	30	223	97115	97319	205	47	5	71.71	2e-22	96.9

Supplementary Table 18: Complete BLAST result of all randomly selected wMT 3'UTR sequences against Ef318A10. E-values were rounded. Subject length = 117984. No wMT-2 transcripts aligned. BLAST parameters – word_size=4, e-value=1e-2.

qseqid	sseqid	qstart	qend	sstart	send	length	mismatch	gapopen	pident	evalue	bitscore
wMT2_gut	6F14	222	335	54597	54703	114	16	4	79.82	1e-20	91.5
wMT2_gut	6F14	222	335	57081	56975	114	16	4	79.82	1e-20	91.5
wMT2_gut	6F14	222	335	59719	59825	114	16	4	79.82	1e-20	91.5
wMT2_neph	6F14	246	348	54597	54692	103	14	4	79.61	2e-17	80.6
wMT2_neph	6F14	246	348	57081	56986	103	14	4	79.61	2e-17	80.6
wMT2_neph	6F14	246	348	59719	59814	103	14	4	79.61	2e-17	80.6
wMT2_neph	6F14	156	190	110969	110934	36	5	1	83.33	8e-04	35.6
wMT2_cal_g	6F14	246	360	54597	54703	115	15	5	80.00	4e-20	89.7
wMT2_cal_g	6F14	246	360	57081	56975	115	15	5	80.00	4e-20	89.7
wMT2_cal_g	6F14	246	360	59719	59825	115	15	5	80.00	4e-20	89.7
wMT2_cal_g	6F14	156	190	110969	110934	36	5	1	83.33	8e-04	35.6
wMT2_phar	6F14	222	331	54597	54699	110	16	4	79.09	1e-18	84.2
wMT2_phar	6F14	222	331	57081	56979	110	16	4	79.09	1e-18	84.2
wMT2_phar	6F14	222	331	59719	59821	110	16	4	79.09	1e-18	84.2
wMT2_bod_w	6F14	222	335	54597	54703	114	16	4	79.82	1e-20	91.5
wMT2_bod_w	6F14	222	335	57081	56975	114	16	4	79.82	1e-20	91.5
wMT2_bod_w	6F14	222	335	59719	59825	114	16	4	79.82	1e-20	91.5
wMT2_crop	6F14	246	360	54597	54703	115	15	5	80.00	4e-20	89.7
wMT2_crop	6F14	246	360	57081	56975	115	15	5	80.00	4e-20	89.7
wMT2_crop	6F14	246	360	59719	59825	115	15	5	80.00	4e-20	89.7
wMT2_crop	6F14	156	190	110969	110934	36	5	1	83.33	8e-04	35.6
wMT2_gizz	6F14	246	360	54597	54703	115	15	5	80.00	4e-20	89.7
wMT2_gizz	6F14	246	360	57081	56975	115	15	5	80.00	4e-20	89.7
wMT2_gizz	6F14	246	360	59719	59825	115	15	5	80.00	4e-20	89.7
wMT2_gizz	6F14	156	190	110969	110934	36	5	1	83.33	8e-04	35.6
wMT2_nerve	6F14	238	352	54597	54703	115	15	5	80.00	4e-20	89.7
wMT2_nerve	6F14	238	352	57081	56975	115	15	5	80.00	4e-20	89.7
wMT2_nerve	6F14	238	352	59719	59825	115	15	5	80.00	4e-20	89.7
wMT2_nerve	6F14	146	182	110971	110934	38	5	1	84.21	6e-05	39.2

Supplementary Table 19: Complete BLAST result of all randomly selected wMT 3'UTR sequences against Lr6F14. E-values were rounded. Subject length = 122990; Query length = 343. No wMT-1 transcripts aligned. BLAST parameters – word_size=4, e-value=1e-2.
